# Evolution of disorder in Mediator complex and its functional relevance

**DOI:** 10.1093/nar/gkv1135

**Published:** 2015-11-20

**Authors:** Malini Nagulapalli, Sourobh Maji, Nidhi Dwivedi, Pradeep Dahiya, Jitendra K. Thakur

**Affiliations:** Plant Mediator Lab, National Institute of Plant Genome Research, Aruna Asaf Ali Marg, New Delhi 110067, India

## Abstract

Mediator, an important component of eukaryotic transcriptional machinery, is a huge multisubunit complex. Though the complex is known to be conserved across all the eukaryotic kingdoms, the evolutionary topology of its subunits has never been studied. In this study, we profiled disorder in the Mediator subunits of 146 eukaryotes belonging to three kingdoms viz., metazoans, plants and fungi, and attempted to find correlation between the evolution of Mediator complex and its disorder. Our analysis suggests that disorder in Mediator complex have played a crucial role in the evolutionary diversification of complexity of eukaryotic organisms. Conserved intrinsic disordered regions (IDRs) were identified in only six subunits in the three kingdoms whereas unique patterns of IDRs were identified in other Mediator subunits. Acquisition of novel molecular recognition features (MoRFs) through evolution of new subunits or through elongation of the existing subunits was evident in metazoans and plants. A new concept of ‘junction-MoRF’ has been introduced. Evolutionary link between CBP and Med15 has been provided which explain the evolution of extended-IDR in CBP from Med15 KIX-IDR junction-MoRF suggesting role of junction-MoRF in evolution and modulation of protein–protein interaction repertoire. This study can be informative and helpful in understanding the conserved and flexible nature of Mediator complex across eukaryotic kingdoms.

## INTRODUCTION

In last two decades, Mediator complex has emerged as a key regulatory component of class II gene expression. It acts as an interface between the DNA bound transcription factors and RNA polymerase II within the pre-initiation complex (1–3). At times, it can also help in recruitment of other cofactors in the complex. Mediator is a gigantic complex consisting of several subunits. It was first discovered in *Saccharomyces cerevisiae* as a necessary part of activator-dependent transcription (4–6). In yeast, the core part of the complex consists of about 21 subunits arranged in different modules called Head, Middle and Tail. Four other subunits form a Kinase module which can reversibly associate with the core complex as and when required (7). Following the lead from yeast research, Mediator complex could be isolated, purified and characterized from few metazoans like human (8–10), mouse (11), *Caenorhabditis elegans* (12) and *Drosophila melanogaster* (13), and a plant, *Arabidopsis thaliana* (14). Mediator subunits were further identified in many more eukaryotes through comparative genomics and bioinformatics analysis (15,16). In comparison to yeast, number of subunits constituting Mediator complex in animals and plants is more.

In animals and fungi, Mediator complex subunits have been found to play a crucial role in cell and organismal viability (17,18), multiple drug resistance (19–21), immunity (22,23), pathogenesis (24–26), embryonic viability (27–29) and fatty acid metabolism (30–32). On the other hand, plant Mediator subunits have been implicated in phenylpropanoid pathway (33), embryo development and patterning (34,35), flowering and correct floral organ development (36,37), plant development (38,39), regulation of non-coding RNA production (40), regulation of plant defence (41), biotic and abiotic stress responses (42–44), helicase activity (45,46), regulation of methylation and cleavage of rRNA (47–49) and hormone signaling (50–52). Thus, Mediator plays important role in almost all the cellular and physiological processes in eukaryotic organisms ranging from unicellular yeast to multicellular animals and plants.

Despite the discovery of Mediator complex in metazoans and fungi nearly two decades ago, due to its massive size and conformational flexibility, high-resolution structural information and its relation to functional mechanism of Mediator complex is not so clearly understood (53). Low-resolution cryo-EM images of the Mediator complex were reported earlier (54,55). Now, high-resolution structures of the Head module of *S. cerevisiae* and *S. pombe* Mediator complex and a few subunits or domains of different fungi and metazoans are also reported (31,53,56–58). The tentative architecture of yeast Middle module was predicted using mass spectrometry and homology modeling (59). Different models of the modular organization of the core Mediator complex have been also reported (60,61). The cryo-EM and X-ray crystallography studies revealed unique folds and domains in Mediator subunits (62). Duplicated folds were observed in Med18 and Med20 (63,64) and four-helix bundle folds were observed in Med11/Med22 (65) and Med7/Med21 (66). The four-helix bundle is found in multiple copies in different subunits of the Mediator complex. The conformational flexibility between the Head and Middle modules was revealed through the identification of flexible hinge in the Med7/Med21 sub complex (66). Furthermore, the structurally characterized sub-modules are also connected to the rest of the Mediator complex through flexible linkers (67). Many Mediator subunits interact with transcription activators. Interaction between the disordered transactivation domains (TADs) present in the transcriptional activators and the activator-binding domains (ABDs) in various Mediator subunits were captured through spectroscopy and EM studies. It has been found that in some subunits, ABDs are separated by flexible linkers. Structural studies indicated that the TAD-ABD interaction occurs through disorder-to-order transition of TADs upon binding to ABD. Structurally dynamic TADs can adopt various conformations on the same surface or on different surface forming a ‘fuzzy’ complex (68). Moreover, structural rearrangements induced in Mediator by activator binding are thought to aid in binding RNA polymerase II and other regulatory molecules (69–71). There is no structural analysis available for any of the Mediator subunits in plants.

The overall structure of the Mediator complex is flexible so that it can recognise different interacting proteins and, following these interactions, adopt different confirmations. The variable structural architecture and subunit composition of the Mediator complex enables it to function in mechanistically distinct ways at different genes in different cells (2). The structural and functional flexibility of Mediator is probably due to abundance of polar, charged and structure breaking residues and the presence of high number of intrinsically disordered regions (IDRs) as evident in human and yeast Mediator complex subunits (72). Intrinsic disorder and IDRs in plant Mediator complex have not been studied at all. IDRs are the regions that do not assume a globular structure in physiological conditions and have been reported to participate in crucial biological roles (73). IDRs contribute to the formation of interface that can interact with multiple partners and thus may act as hubs in the protein interaction networks (74). Several IDRs harbor short stretches of Motif Recognition Features (MoRFs) which undergo disorder-to-order transition upon binding to their cognate ligands (75,76). IDRs adjust to the structure of binding partners by folding into stable complexes (77). It is considered that the disordered regions or proteins evolve more rapidly than the ‘ordered’ proteins which contribute to evolutionary divergence (78). Several studies indicate that despite the sequence variation, the disordered regions or protein families are functionally conserved (79–81). In order to understand the significance of intrinsic disorder and IDRs in Mediator, in-depth comparative analysis of disorder in the metazoan, plant and fungal Mediator subunits within and across the kingdoms is unprecedented. In this study, we have tried to identify the conservation patterns of intrinsic disorder and understand its structural – functional relationship in Mediator complexes of different kingdoms.

We considered a large dataset of 146 eukaryotes from three kingdoms viz., metazoans, plants and fungi, and analysed the disorder in the Mediator subunits within and across kingdoms. The role of disorder in evolution was explored and a correlation with acquisition of novel functions was studied. We found that similarities and differences in the positioning of IDRs in specific Mediator subunits between different kingdoms are quite conspicuous. The functional relevance of intrinsic disorder and IDRs in Mediator complex subunits was revealed by the presence of several conserved MoRFs and post-translational modification (PTM) sites in it suggesting that the disorder of subunits probably serves to perform specific crucial and basic functions.

Thus, this study not only unravels the importance of intrinsic disorder and IDRs within the Mediator complex but also explains their role in networking of Mediator with diverse transcription factors and other proteins. A novel concept of junction-MoRFs has been introduced and its role in the extension of existing IDRs during evolution has been proposed. This is the first report to shed light on disorder and IDRs in plant Mediator subunits not only computationally, but also experimentally. We believe that this comprehensive study of disorder propensity and the placement of IDRs in Mediator complex will be very helpful in understanding the conserved and diverged structural and mechanistic details of its involvement in different cellular processes.

## MATERIALS AND METHODS

### Sequence retrieval and identification of Mediator subunits in metazoans and plants

Mediator subunit sequences of 19 metazoans, 3 plants and 25 fungi were obtained from published literature (15). The metazoan and plant sequences were segregated into respective subunits. The methodology described previously (16) to identify Mediator complex subunits in plants was adopted to expand the sample size of the current study. Already known subunit sequences of metazoans and plants were obtained from Bourbon, 2008 (15). HMM profiles were constructed for individual Mediator subunits using metazoan and plant sequences, separately. UniParc and nr databases from UniProt (82) and NCBI (83), respectively, were downloaded and searched in-house using the HMM profiles of individual subunits. Sequences of 22 fungal Mediator subunits were obtained from Bourbon, 2008 and used directly. Additional Mediator subunit sequences were identified in 78 metazoans and 21 plants through HMM profile search. However, eight metazoans that have partial sequences for most of the Mediator subunits were excluded from the quantitative analysis. As reported earlier, we could not find Med1 in plants and Med26 in fungi. Although orthologs of Med34, Med35, Med36 and Med37 could be found in metazoans and fungi, they have so far been biochemically purified only in plant Mediator complexes and hence considered as plant specific Mediator subunits (84). List of all the organisms considered for this study is given in Supplementary Table ST1. Only one of the isoforms per subunit was considered for all the organisms, wherever applicable.

### Calculation of disorder

Disorder of each amino acid was predicted for all the Mediator subunits using IUPred (85) and DISOPRED2 (86). Protein FASTA sequences were used as input files and the disorder propensity of each amino acid was obtained in a tabular form. Amino acids with a predicted disorder score greater than or equal to 0.5 were considered to be disordered. As consensus results obtained from both the tools, results obtained from the IUPred algorithm detailed in the current study (Supplementary Figures S1–S5). Further, average disorder of each subunit was calculated as a mean of the disorder score of each amino acid constituting the subunit. IDRs were characterized as continuous stretch of at least 30 amino acids with a predicted disorder score above or equal to 0.5 allowing a maximum of three residues long ordered gap (72). Gnuplot available at http://www.gnuplot.info was then used to construct bar graphs for qualitative visualization of IDRs in each kingdom.

### Statistical analysis and programming

The mean and standard deviation of average disorder for the three kingdoms were calculated using individual scores of each subunit of each organism. The significance of difference between the kingdoms was assessed by the non-parametric Mann–Whitney test. All the programs were written in perl.

### Clustering of metazoans, plants and fungi based on the average disorder of Mediator complex subunits

Euclidean distance was calculated between two organisms as a root of the sum of squared difference in average disorder of the corresponding Mediator complex subunits. The distance thus calculated was used to make 146 × 146 distance matrix.

\begin{equation*} d(X,Y) = \sqrt {\sum\limits_{i = 1}^n {(X_i - Y_i )^2 } } \end{equation*}

where *d*_(*X,Y*)_ is the distance between two organisms *X* and *Y*, *X_i_* is the average disorder of subunit *i* in organism *X* and *Y_ji_* is the average disorder of the corresponding subunit *i* in organism *Y*.

The distance matrices were then used as input to construct dendrograms with PHYLIP-3.695 using neighbor-joining method (87). The dendrogram was visualized in FigTreeV1.4.0 (available at http://tree.bio.ed.ac.uk/software/figtree/).

### Assignment and position of conserved IDRs

Protein sequences were divided into three regions of equal length. The first one-third of the protein length was considered the N-terminus, the next one-third as the middle and the remaining part as the C-terminus. An IDR was considered to belong to a region if >50% of its length lied in that region. In case of conflict in the position of IDR as observed for long IDRs, the N- and C-termini were given precedence over the middle region. The percentage of organisms with IDR in three zones was thus calculated for Mediator subunits of three kingdoms and the proteomes downloaded from UniProt. For the purpose of the current study, an IDR is called ‘conserved’ if at least 70% or more organisms of a kingdom have an IDR in the same region of the Mediator subunit.

### Mediator subunit interaction network in human and yeast

Direct interactions were identified and downloaded for each Mediator subunit from BioGRID (88) and iRefWeb (89). Cytoscape V3.1.0 (90) was then used to visualize the protein–protein interaction networks and to calculate the number of interactions of each human and yeast Mediator subunit. For the purpose of the current study, Mediator subunit with 10 or more direct interactions was considered as ‘Hub’ (91). For subunit-subunit interactions five or more direct interactions were considered as threshold.

### Prediction of post translational modification sites (PTMs) in Mediator subunits and proteomes of eight model organisms

Four major types of PTMs such as phosphorylation of Ser, Thr and Tyr, N-linked Asn and O-linked proline glycosylation, Lys/Arg methylation and Lys acetylation were analyzed in Mediator subunits of *S. cereviseae*, *A. thaliana*, *O. saitva* subsp. *japonica*, *C. elegans*, *D. melanogaster*, *D. rerio*, *G. gallus* and *H. sapiens*. PTMs were predicted with freely available web tools NetPhos 2.0 (92), NetNGlyc 1.0 (93), PMeS (94) and PAIL (95) with default parameters. A stretch of 30 residues in Mediator complex subunits was considered as PTM hotspot if the fraction of predicted PTM sites in this stretch was between 0.1 and 0.3.

### Prediction of molecular recognition features (MoRFs)

The protein–protein recognition and interaction sites were predicted in Mediator subunits of 30 organisms using MoRFpred (96). A stretch of at least five amino acids with a score greater than or equal to 0.5 was considered as a potential recognition and binding site. Such stretches were highlighted on multiple sequence alignments constructed using MAFFT (97) and ALSCRIPT (98). MoRF was called ‘conserved’ if it is aligned in the multiple sequence alignment for at least four organisms.

### Homology modeling

Homology models of AtMed7, AtMed21 and AtMed31 were constructed by submitting FASTA sequences to Phyre2 available at www.sbg.bio.ic.ac.uk/phyre2 (99). Quality of model was assessed using PROCHECK (100). Modeller was also used to build *Arabidopsis* Mediator subunit models (101). PyMOL was used to align the corresponding models thus generated (https://www.pymol.org).

### Yeast two hybrid assays

Full length *AtMed4, AtMed6, AtMed9* and *AtMed19a* were amplified using specific primers (Supplementary Table ST2) and cloned in yeast two-hybrid bait and prey vectors, i.e. pGBKT7 and pGADT7, respectively (Clontech, CA). Yeast two-hybrid assay was conducted using Matchmaker Gold Yeast two-hybrid System (Clontech, CA) according to manufacturer's protocol. All the cloned subunits were checked for their interaction with vector alone as control for the study. To check the interaction both BD and AD constructs were co-transformed in yeast strain AH109, a reporter strain. The transformed yeast colonies were selected on DDO (Double Dropout Synthetic Media, SD-Trp^−^/Leu^−^). Positive colony was inoculated and cultured till the OD_600_ reached ∼0.2 and spotted on QDO (SD-Trp^−^/Leu^−^/His^−^/Ade^−^) plate. To map the regions involved in interaction, different fragments of selected subunits were used for yeast two-hybrid assay.

### Bimolecular fluorescent complementation (BiFC)

*AtMed4* and *AtMed9* were cloned in pENTR vector and transferred to pSAT4-DEST-N (1–174) EYFP-C1 and pSAT5-DEST-C (175-END) EYFP-C1 vectors, respectively, using Gateway cloning technology (Invitrogen). These recombinant plasmids were bombarded on onion epidermal cells using PDS-1000 Helios Gene Gun (Bio-Rad) for simultaneous expression of both subunits. After 24 h of bombardment, fluorescence was checked under TCS SP2 (AOBS) laser confocal scanning microscope (Leica Microsystems).

## RESULTS

### Disorder in Mediator complex is related to the complexity of organism

**Figure 1.**
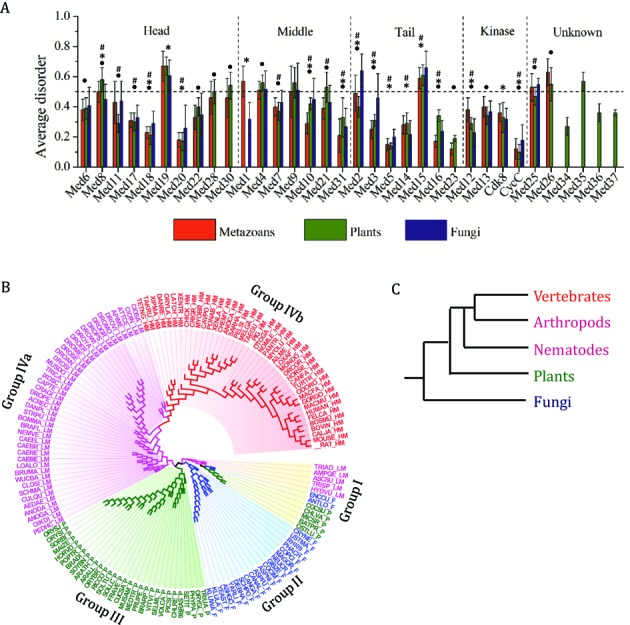
Disorder in Mediator complex subunits of metazoans, plants and fungi. (**A**) Average disorder of Mediator subunits in metazoans, plants and fungi. Error bars represent the standard deviation in the average disorder. The subunits belonging to different modules (Head, Middle, Tail and Kinase) have been separated by vertical dashed lines. 0.5 (dashed line) is the threshold for disorder of the subunit. Significant differences as determined by Mann–Whitney test at alpha level 0.05 are indicated by (.) for metazoan versus plants, (*) for metazoans versus fungi and (#) for plants versus fungi. (**B**) Clustering of metazoans, plants and fungi based on the average disorder of the Mediator subunits. Euclidean distance between any two organisms was calculated using average disorder of the corresponding subunits to make a 146 × 146 distance matrix. Unrooted tree was constructed from the distance matrix using PHYLIP program by the neighbor-joining method. Lower organisms of all the three kingdoms metazoans, plants and fungi cluster in Group I. Fungi (blue), plants (green), lower metazoans (magenta) and higher metazoans (red) segregate into Group II, Group III, Group IVa and Group IVb, respectively. (**C**) Summary of the clustering of the major phyla and kingdoms.

In addition to the known Mediator subunit sequences (15,16), subunits from 99 different organisms were also identified (Supplementary Table ST1, see Materials and Methods section). In total, 146 organisms representing major clades across metazoans, plants and fungi were used in the current study. Two different web servers were used to assess intrinsic disorder in these Mediator subunits; IUPred, which is based on the estimated energy of pair wise interactions in a window around a residue, and DISOPRED2 based on linear support vector machine algorithm (85,86). In addition we randomly picked 50 Mediator subunit sequences from three kingdoms and assessed intrinsic disorder using PONDR VLS1 which uses feed-forward neural network based on physiochemical properties of amino acid composition (102). Since all the three methods gave similar results, only IUPred results are presented here. Average disorder (disorder score were averaged over the entire protein sequence) was calculated for all the Mediator subunits in these organisms and analyses were done for individual subunits, individual modules and also for the whole complex. A preponderance of intrinsic disorder (average disorder above or equal to 0.5 threshold value) was found in Med19, Med4, Med9 and Med15 of all the kingdoms indicating that these subunits exist as flexible proteins throughout eukaryotes (Figure 1A). Med26, which is not found in fungi, also turned out to be significantly disordered in metazoans and plants (Figure 1A). Med1 in metazoans, and Med28, Med30, Med21 and Med35 in plants, and Med2 in fungi, are disordered suggesting their importance in providing the respective Mediator complexes kingdom-specific structural flexibility. There are few Mediator subunits which are significantly disordered in two kingdoms. For instance, Med8 and Med26 are disordered in metazoans and plants, and Med25 is disordered in metazoans and fungi (Figure 1A). Average disorder of Med8 appears to be significantly higher in plants compared to that of metazoans whereas in Med26 average disorder is significantly higher in metazoans compared to plants (Table 1). Next, all the organisms were clustered with respect to the Euclidean distance calculated as a function of the average disorder of the corresponding subunits. This approach clustered 146 eukaryotes into four major groups and a clear grouping of fungi, plants and metazoans was evident in Group II, Group III and Group IV, respectively (Figure 1B). Group I comprised of lower organisms of each kingdom including placozoan, poriferans, cnidarians, microsporidians and green algae. This is not surprising, as this group represents the early point of divergence and thus have similar extent of disorder. Group IV is further divided into subgroups; Group IVa comprising of lower metazoans and Group IVb with higher metazoans. Within animalia, clustering as a function of average disorder of Mediator complex subunits does not favor formation of ecdysozoa clade but strongly supports the coelomata topology in which arthropods cluster with vertebrates. Interestingly, contradicting to opisthokonta topology, plants and metazoans formed sister clades and appeared to be more closely related to each other than to fungi (Figure 1C). In comparison to fungi, plants and animals in groups III and IV, respectively, are more complex organisms, suggesting that disorder in Mediator complex has evolved as a function of the organismal complexity.

**Table 1.** Average disorder of each Mediator subunit in Metazoans (M), Plants (P) and Fungi (F) and related Mann–Whitney *U* test probabilities

Average disorder								
					Probability			
Module	Subunit	Metazoans (M)	Plants (P)	Fungi (F)	MP	MF	PF	Rank order
Head	Med6	0.38 ± 0.07	0.39 ± 0.07	0.41 ± 0.12	0.047*	0.066	0.325	(P>M) = F
	Med8	0.50 ± 0.07	0.58 ± 0.08	0.45 ± 0.10	7.211e-07*	0.016*	1.810e-05*	P>M>F
	Med11	0.43 ± 0.14	0.29 ± 0.06	0.44 ± 0.13	9.738e-08*	0.492	5.005e-05*	P<(M = F)
	Med17	0.31 ± 0.05	0.30 ± 0.06	0.33 ± 0.08	0.007*	0.052	0.022*	P<(M = F)
	Med18	0.23 ± 0.04	0.21 ± 0.06	0.29 ± 0.08	1.630e-07*	0.0005*	3.386e-05*	P<M<F
	Med19	0.67 ± 0.10	0.67 ± 0.06	0.61 ± 0.10	0.169	0.006*	0.031*	(P = M)>F
	Med20	0.18 ± 0.05	0.17 ± 0.06	0.26 ± 0.15	0.227	0.008*	0.011*	(P = M)<F
	Med22	0.33 ± 0.08	0.40 ± 0.06	0.35 ± 0.14	3.10e-06*	0.274	0.249	(P>M) = F
	Med28	0.46 ± 0.11	0.50 ± 0.08	_	0.044*	NA	NA	P>M
	Med30	0.46 ± 0.13	0.54 ± 0.09	_	0.006*	NA	NA	P>M
Middle	Med1	0.57 ± 0.10	_	0.32 ± 0.11	NA	2.196e-10*	NA	M>F
	Med4	0.51 ± 0.06	0.56 ± 0.05	0.52 ± 0.12	1.034e-05*	0.548	0.131	(P>M) = F
	Med7	0.40 ± 0.06	0.37 ± 0.06	0.43 ± 0.08	0.011*	0.075	0.003*	P<(M = F)
	Med9	0.50 ± 0.17	0.56 ± 0.10	0.51 ± 0.18	0.145	0.650	0.484	P = M = F
	Med10	0.29 ± 0.07	0.42 ± 0.04	0.45 ± 0.14	6.23e-13*	1.775e-07*	0.042*	(P>M)>F
	Med21	0.39 ± 0.07	0.53 ± 0.10	0.43 ± 0.11	1.786e-11*	0.140	0.002*	P>(M = F)
	Med31	0.21 ± 0.11	0.33 ± 0.13	0.27 ± 0.12	1.269e-05*	0.024*	0.01*	P>(M<F)
Tail	Med2	0.49 ± 0.12	0.40 ± 0.07	0.64 ± 0.11	1.555e-05*	7.387e-06*	2.553e-07*	(P<M)<F
	Med3	0.25 ± 0.06	0.31 ± 0.04	0.46 ± 0.16	7.303e-09*	1.217e-07*	9.412e-05*	(P>M)<F
	Med5	0.15 ± 0.04	0.14 ± 0.02	0.20 ± 0.05	0.192	0.0002*	2.739e-05*	(P = M)<F
	Med14	0.28 ± 0.06	0.29 ± 0.07	0.22 ± 0.09	0.167	0.002*	0.004*	(P = M)>F
	Med15	0.59 ± 0.07	0.61 ± 0.07	0.66 ± 0.11	0.144	0.0002*	0.009*	(P = M)<F
	Med16	0.17 ± 0.04	0.34 ± 0.04	0.24 ± 0.07	8.181e-14*	2.772e-05*	1.120e-05*	P>(M<F)
	Med23	0.12 ± 0.04	0.19 ± 0.02	_	5.335e-11*	NA	NA	P>M
Kinase	Med12	0.38 ± 0.08	0.29 ± 0.04	0.23 ± 0.09	1.193e-09*	7.579e-09*	0.006*	(P<F)<M
	Med13	0.40 ± 0.07	0.34 ± 0.06	0.37 ± 0.07	8.838e-08*	0.087	0.107	(P = F)<M
	Cdk8	0.36 ± 0.06	0.33 ± 0.10	0.32 ± 0.07	0.064	0.015*	0.714	P = (M>F)
	CycC	0.12 ± 0.07	0.10 ± 0.05	0.18 ± 0.10	0.005*	0.023*	0.001*	P<M<F
Unknown	Med25	0.53 ± 0.09	0.47 ± 0.06	0.55 ± 0.04	1.229e-05*	0.613	0.007*	P<(M = F)
	Med26	0.63 ± 0.09	0.55 ± 0.11	_	0.001*	NA	NA	P<M
	Med34		0.27 ± 0.06					
	Med35		0.57 ± 0.06					
	Med36		0.36 ± 0.06					
	Med37		0.36 ± 0.02					

*Indicates that the means are significantly different at alpha level 0.05.

### Analysis of IDRs revealed conserved and unique patterns of IDRs in the Mediator subunits of different kingdoms

**Figure 2.**
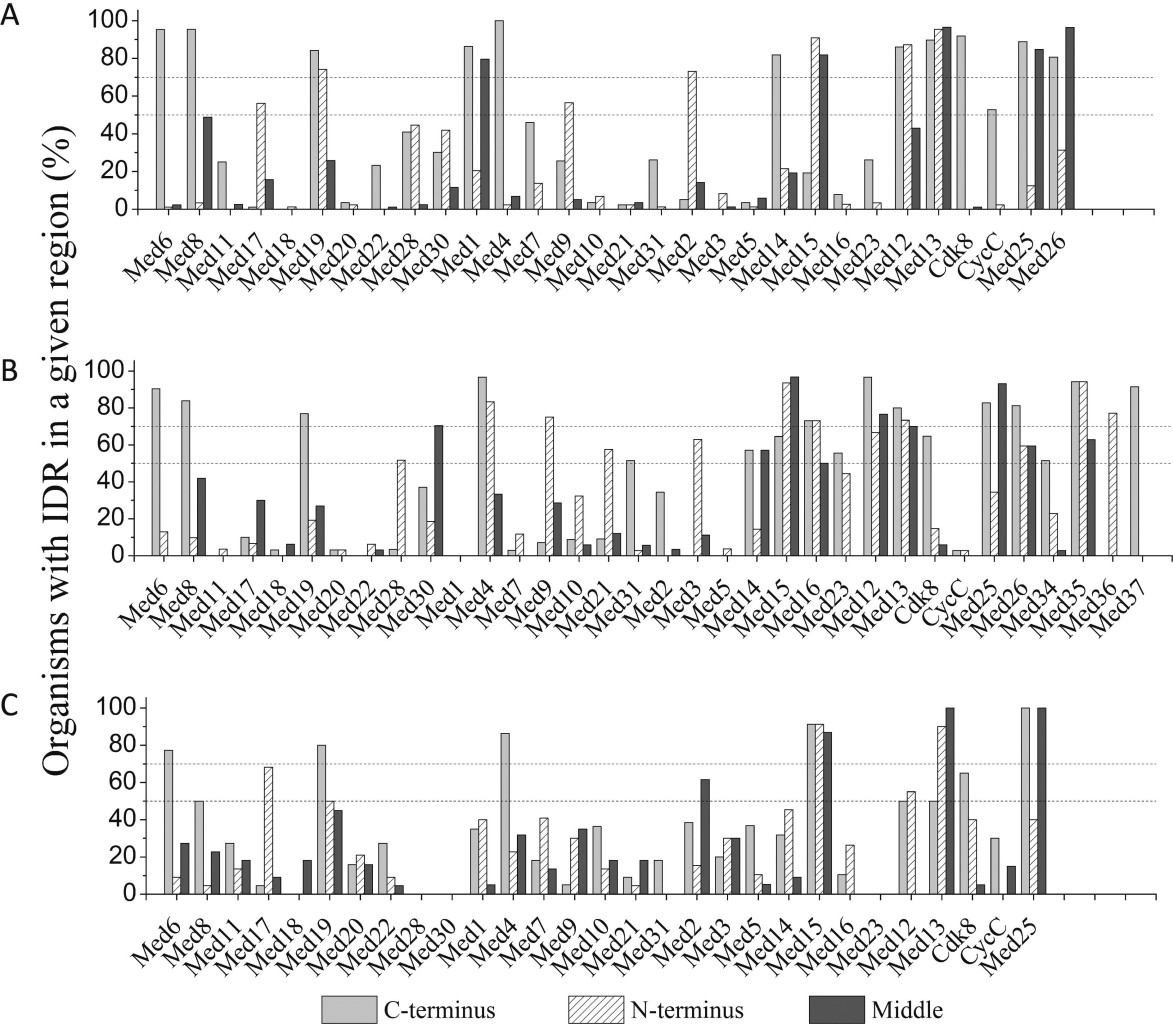
Conservation of IDRs in the Mediator subunits. Percentage of organisms with an IDR at the C-terminus (gray), N-terminus (cross-hatched) or Middle region (dark gray) of the subunit in (**A**) metazoans, (**B**) plants and (**C**) fungi is plotted. Dashed lines indicate the conservation of IDR in 70% and 50% organisms.

Disordered proteins usually harbor IDRs which have been found to be involved in several biological functions. Most of the cell signaling proteins and transcription factors contain IDRs. As it is clear from earlier section, that several Mediator subunits are enriched in disorder promoting residues, we looked for continuous stretch of such residues constituting IDRs. This is in accordance to the observations made for yeast and human Mediator subunits (72). By using diverse selection of taxa, we uncovered distinct patterns of IDRs in the Mediator subunits across different kingdoms (Supplementary Figures S1–S5). Each subunit was first divided into three equal C-, N- and middle regions and then every region was assessed for the presence of >50% of IDR (Figure 2). For the purpose of the current study, an IDR is called ‘highly conserved’ or ‘moderately conserved’ if more than half of the IDR is present in a particular region in at least 70% and 50% of the organisms in a kingdom, respectively. IDRs present in only a particular group of organisms are called ‘restricted’. Such an analysis revealed several general and unique patterns of IDR placement in Mediator subunits across species and kingdoms.

#### Highly conserved IDRs

Out of the 24 Mediator subunits generally found in all the eukaryotes, six subunits (Med6, Med19, Med4, Med15, Med13 and Med25) have highly conserved IDRs across the three kingdoms (Figure 2A–C). The IDRs in these six subunits are placed at the C-terminus of Med6, Med19, Med4 and Med25, and at the N-terminus of Med15 and Med13. Highly conserved IDRs are also present in the middle regions of Med15, Med13 and Med25. In metazoans and plants, Med13 has an IDR at the C-terminus. In metazoans, an additional IDR is present towards the N-terminus of Med19. In Fungi, unique conserved IDR is present at the C-terminus of Med15. In plants, an additional IDR is present at the N-terminus of Med4.

In metazoans, unique highly conserved IDRs are present at the C-terminus of Med14 and Cdk8, at the N-terminus of Med2 and Med12, and in the middle regions of Med26. Med1 of metazoans have conserved IDRs in the C-terminus and middle regions. In plants, IDRs uniquely present at the C-terminus of Med37, at the N-terminus of Med4, Med3, Med9 and Med36, and in the middle regions of Med12 and Med30, are highly conserved. In addition, the unassigned plant specific subunit, Med35 and the Tail module subunit Med16, have conserved IDRs at both the termini. IDRs at the C-terminus of Med8, Med12, Med13 and Med26 are conserved in both metazoans and plants. Med26, which is not assigned to any module at present, has an additional conserved IDR in the middle region in metazoans. The lengths of IDRs and their placement pattern seem to have evolved from lower organisms to higher organisms. For example, the length of the IDR in Med8 of lower metazoans appears to be shorter than that of higher metazoans in contrast to the subunit length which is more or less similar (Supplementary Figure S1). In plants, the length of Med8 and its IDR at the C-terminus are significantly longer than metazoans and fungi) (Supplementary Figure S6).

#### Moderately conserved IDRs

Moderately conserved IDRs were found in several Mediator subunits in all the three kingdoms. Metazoan specific moderately conserved IDRs are present at the C-terminus of Cyclin-C and N-terminus of Med9. Similarly, moderately conserved IDRs at the C-terminus of Med31, Med15, Med23 and Med34, at the N-terminus of Med28, Med21 and Med12, and middle regions of Med35 are unique to plants. IDRs specific to fungi are present at the C-terminus of Med8 and Med13, and at the N-terminal regions of Med19. Some subunits have moderately conserved IDRs in more than one region. For example, in plants, Med14 and Med26 have IDRs at the C- and N-termini, respectively. In addition, both of them have moderately conserved IDRs in their middle regions (Figure 2B).

The N-terminus of Med17 has an IDR in at least 50% of metazoans and fungi. The N-terminus of Med12 has an IDR in plants and fungi. The C-terminal regions of plants and fungi have moderately conserved IDR in Cdk8. The C-terminus of Med12 has an additional moderately conserved IDR in fungi. Interestingly, some of these subunits have highly conserved IDRs in other kingdoms, in the same regions. For example the Kinase module subunits, Med12, Med13 and Cdk8 were found to have highly conserved IDRs in one or more kingdoms. Also, the moderately conserved IDR at the N-terminus of Med9 in metazoans has a highly conserved counterpart in plants. The IDR in Med9 is specific to only the higher metazoans and the Drosophila group. The other moderately conserved N-terminal IDR of Med17 is specific to worms, fishes and mammals (Supplementary Figure S2). Also, Cyclin C appears to have an IDR at the C-terminal end in higher metazoans and in few worms (Supplementary Figure S3). These IDRs are probably present or absent in organisms due to selection process during evolution.

#### Restricted IDRs

Only few related organisms were found to have restricted IDRs in some of their Mediator subunits. In general, Med11, Med18, Med20, Med22 of Head module, Med7 and Med10, of Middle module, and Med5 of Tail module have a minimal or insignificant propensity to have an IDR in all the kingdoms (Supplementary Figures S1–S5). However, some of these subunits show genus- or group-specific IDRs. For instance, short IDRs were observed at the N-terminus and middle region of Med23 in many plants including *Arabidopsis*, all rice species and at the C-terminus of Caenorhabditis, Drosophila groups and fishes in metazoans (Supplementary Figures S3 and S4). Drosophila and Caenorhabditis share similar pattern of IDRs in Med11 (Supplementary Figure S2). Interestingly, Caenorhabditis group and few other worms seem to have unique IDRs in Med3 and Med10 (Supplementary Figure S1). Restricted IDRs are also present in Med7, and Med22 of higher metazoans (Supplementary Figures S1 and S3). Unique pattern of restricted IDRs was observed in Med28 and Med30 of Head module in metazoans (Supplementary Figure S3). Interestingly, in Med28, IDR position shifted from the C-terminus in lower metazoans to both the termini in cephalochordates, hemichordates, fishes and amphibians, to N-terminus in reptiles, aves and mammals. In contrast, a long IDR at the N-terminus of Med30 in lower metazoans shifted to a short IDR in the middle region in higher metazoans. In plants, monocots seem to have an IDR at the C-terminal end of Med2 (Supplementary Figure S4).

Overall, most of the IDRs are present at terminal regions of Mediator subunits. Mediator is a large complex which is very flexible and interacts with a plethora of other proteins. In this regard, positioning of more IDRs in the terminal regions will be more useful as IDRs might be involved not only in the assembly of the complex but also in establishing contacts with other regulatory proteins and complexes.

### Phosphorylation and acetylation are preferred at disordered sites of Mediator subunits

**Figure 3.**
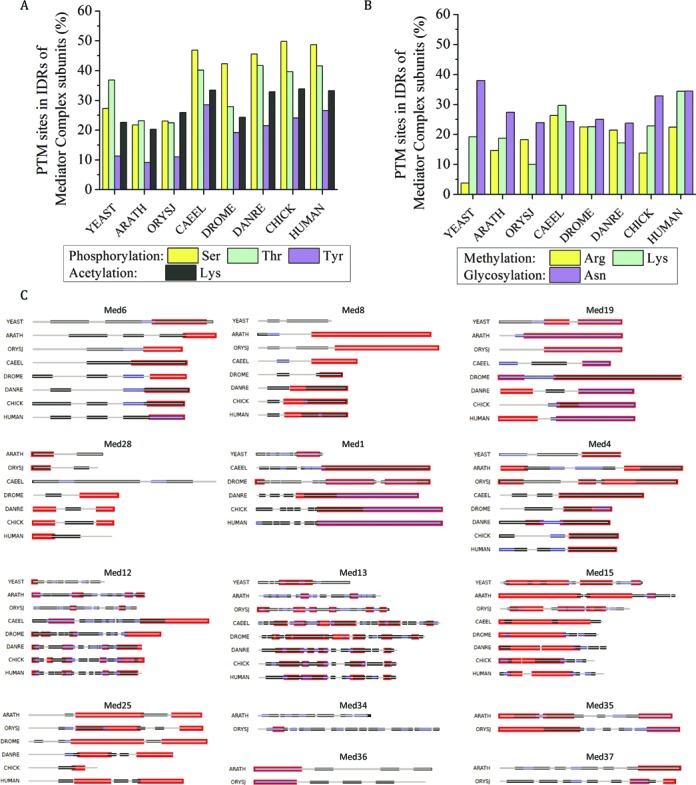
Post translational modifiation sites (PTMs) in IDRs of Mediator complex subunits of model organisms. (**A**) Phosphorylation and acetylation sites. (**B**) Methylation and glycosylation sites in the IDRs of Mediator complexes of *S.cerevisiae* (YEAST), *A. thaliana* (ARATH), *O. sativa* subsp. *Japonica* (ORYSJ), *C. elegans* (CAEEL), *D. melanogaster* (DROME), *D. rerio* (DANRE), *G. gallus* (CHICK) and *H. sapiens* (HUMAN). (**C**) PTM hotspots in the Mediator subunits. Mediator subunits (gray line) with highly conserved or moderately conserved IDRs (red bars) are shown. PTM hotspots with a density of 0.2–0.3 are shown in blue while, hotspots with a density of 0.1–0.2 are shown in black.

Post-translational modifications (PTMs) play important roles in protein–protein interactions and functions. In the dynamic disordered protein complexes, PTMs, especially phosphorylation and acetylation, could serve as means to fine-tune the electrostatic interactions of disordered regions of the proteins. We predicted the PTM sites (phosphorylation and acetylation) in the sequences of Mediator subunits of eight selected model organisms and compared the average disorder between the PTM and non-PTM sites. We found that more than 40% of the serine phosphorylation sites in metazoans are present in IDRs relative to 27% in *S. cerevisiae* and 21–23% in plants (Figure 3A). Even, methylation and glycosylation sites were found to be present in IDR regions of Mediator subunits of selected model organisms (Figure 3B). Next, the mean disorder score of all the PTM sites and non-PTM sites was computed separately and compared (Supplementary Table ST3). In all the selected model organisms representing the three eukaryotic kingdoms, phosphorylation and acetylation sites were found to have higher mean disorder scores relative to its corresponding non-PTM sites.

In general, the Tail and Kinase module subunits have higher number of PTM sites (Supplementary Figure S7). To assess if the propensity of PTM sites is concentrated in only specific regions as the numbers suggest, we calculated the density of PTM sites in the ordered and disordered regions of the Mediator subunits with highly conserved and moderately conserved IDRs. In fact, many of the subunits have PTM hotspots flanking or within the IDRs (Figure 3C). Among subunits with highly conserved IDRs, Med1, Med19, Med4, Med26, Med35 and Med36 have PTM hotspots within their IDRs in all the organisms. Med6 and Med8 have dense PTM sites in the IDRs only in metazoans. It is interesting to note that even though Med36 has lower number of PTM sites compared to other unassigned plant subunits, these sites are concentrated in the IDRs. In metazoans, the PTM sites are concentrated in the IDRs of Med8, Med14, Cdk8 and CycC. In plants, Med30 has PTM hotspots in IDRs. The presence of PTM hotspots in different Mediator subunits follows the trend of conservation of disordered regions in specific kingdoms. Med12 and Med13 on the other hand have very dense PTM sites throughout the length of the subunits. In subunits like Med25 and Med15 hotspots are found flanking the IDRs. Next, we analyzed the experimentally observed PTMs in the Mediator subunits of yeast and human. Phosphosite Plus^®^ is an open systems biology resource for studying experimentally observed PTMs in the regulation of biological processes (103). We found 75% of phosphorylation and 84% of acetylation events localized in the IDRs of human Mediator complex (Supplementary Table ST4). When we analysed previously reported experimentally observed phosphorylation sites in yeast (104), we found 74% phosphorylation sites in the disordered regions of Mediator subunits (Supplementary Table ST4).

Thus, post-translational modifications especially phosphorylation and acetylation can predominantly occur within IDRs, probably due to easier steric access of modifying enzymes like kinases and acetyl transferases. This also suggests that IDRs of Mediator subunits are most frequently involved in post-translational modifications for mediating pre-initiation complex formation and relaying signals from one end of the complex to the other. Many of these predicted sites have been observed experimentally to be modified by kinases and acetylases (Supplementary Table ST4). However, as the predictions were performed on individual sequences, we cannot exclude the possibility of some of these sites being buried within the protein structure and not available all the time for modification.

### IDRs in human and yeast Mediator subunits are associated with protein–protein interaction

Proteins with IDRs are involved in numerous biological processes by virtue of their ability to interact with other proteins (105). In order to understand the importance of disordered regions of Mediator subunits, we analysed their involvement in protein–protein interactions. For this, first the interactions for all the human and yeast Mediator subunits were downloaded from BioGRID^3.2^ (88) and iRefWeb (89). The interactions which were experimentally validated as direct interactions as defined in BioGRID^3.2^ were separated and analysed using Cytoscape^3.1.1^ (90). The interaction data for plant Mediator subunits is not yet available.

**Figure 4.**
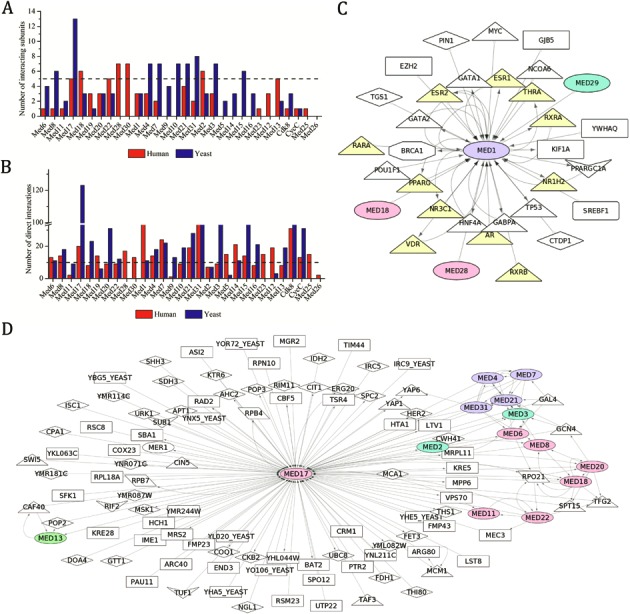
Direct interactions for human and yeast Mediator complex subunits. (**A**) Number of direct inter-subunit interactions reported for human and yeast Mediator complex. Dashed line represents the arbitrary threshold of five direct interactions which defines a ‘hub’. (**B**) Number of direct interactions of a given Mediator subunit reported in human and yeast. Dashed horizontal line corresponds to 10 interactions, a threshold to define ‘hub’ subunit. Disordered hubs with highest number of direct interactions in (**C**) human Med1 and (**D**) yeast Med17 are shown. The interaction maps contain nuclear hormone receptors (yellow triangles), general and specific transcription factors (triangles), enzymes (diamonds), polymerase subunits (parallelograms), histone proteins (round rectangles), transcription coactivators, repressors and cofactors (vee), ubinquitinylation complex proteins (octagon) and other proteins (rectangles). The networks contain both upstream and downstream interaction partners.

The number of interactions of each subunit with the other subunit(s) in human and yeast Mediator complexes were delineated and analysed (Figure 4A). Med17 in the Head module interacts with 14 other subunits and appears to be crucial for the yeast Mediator complex architecture (Figure 4A). Other Head module subunit, Med8, also interacts with many other subunits in yeast. However, in yeast, it is the Middle module subunits that interact, relatively, with more number of subunits compared to the subunits in other modules. Also, Med3 and Med15 of yeast Tail module appear to interact with several other subunits. In human Mediator complex also, Med17 is involved in several interactions. Other subunits in the Head module, Med18, Med22, Med28 and Med30, interact with several other Mediator subunits. In Tail module it is Med2 which engages itself in interaction with several other subunits and in Kinase module, Med13 does the same (Figure 4A).

The upstream and downstream interactions as defined elsewhere were computed for each subunit. A protein which has been found to interact with 10 or more different proteins in different contexts has been considered as a ‘hub’ (91). According to this definition, 19 of 30 Mediator subunits in human and 20 of 25 subunits in yeast act as hubs of protein–protein interactions (Figure 4B). We found that a number of Mediator subunits are conserved in yeast and human in terms of their ability to function as hub. This includes Med6, Med8, Med17, Med1, Med4, Med7, Med21, Med31, Med14, Med15, Cdk8 and CycC. However, there are also several subunits like Med19, Med28, Med30, Med5, Med23, Med12 and Med25 in human and Med18, Med20, Med22, Med9, Med10, Med3 and Med16 in yeast, that are probably the unique ‘hub’ proteins in the respective organisms. Interestingly, the complete Middle module of yeast Mediator complex is constituted by hub proteins.

In order to correlate the degree and stretch of disorder with the ability to interact with other proteins, the Mediator subunits were classified into two groups based on their average disorder and presence of highly conserved IDRs; ordered subunits with highly conserved IDRs and disordered subunits. While, the number of ordered hubs with an IDR is similar in both the organisms, the number of disordered hubs increases from yeast to human. In yeast, 12 hub proteins have IDRs whereas in human the number is 15. 9 of the 15 hubs in human Mediator complex have IDRs that are either highly or moderately conserved in metazoans. In yeast, only 4 out of 12 hub subunits harbor IDRs that are conserved in fungi. Among all the hub subunits, disordered hubs, Med1 and Med17, were found to have the highest number of interactions in human and yeast, respectively (Figure 4C and D). This is consistent with the functional role of these subunits in maintaining the physiology of the organism (8,106,107).

### Experimental validation of importance of IDRs in Mediator subunits of *Arabidopsis*

**Figure 5.**
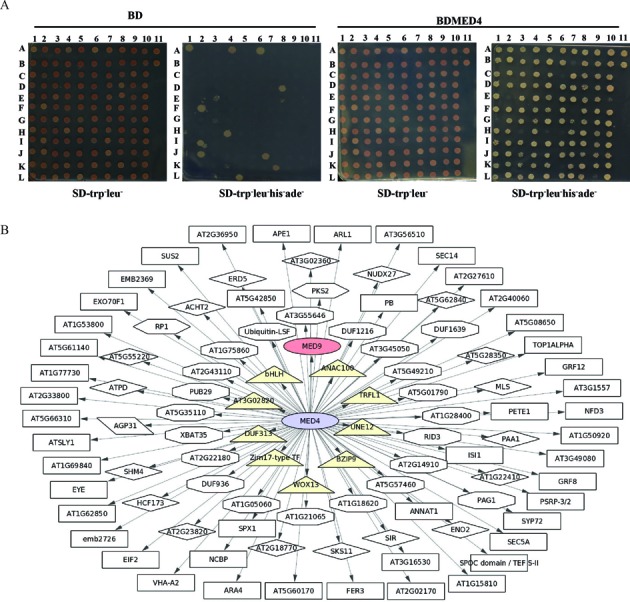
Characterization of AtMed4 as a hub subunit in *Arabidopsis* Mediator complex. (**A**) Clones obtained after Yeast two-hybrid screening using AtMed4 as bait. False positive clones were characterized by yeast two hybrid analysis using empty vector (BD) as a control. Cultures of co-transformed yeast clones adjusted to an OD of 0.2 were spotted on synthetic drop out medium SD-Trp^−^/Leu^−^/His^−^/Ade^−^ to score the interactions. Growth on synthetic double drop out (DDO) medium SD-Trp^−^/Leu^−^ media was used as control for proper growth. (**B**) The interactome of AtMed4 represents diverse proteins belonging to the categories of general transcription factors (yellow triangles), enzymes (diamonds), Mediator subunit (vee), ubinquitinylation proteins (octagon), kinase (hexagon) and other proteins (rectangles).

**Figure 6.**
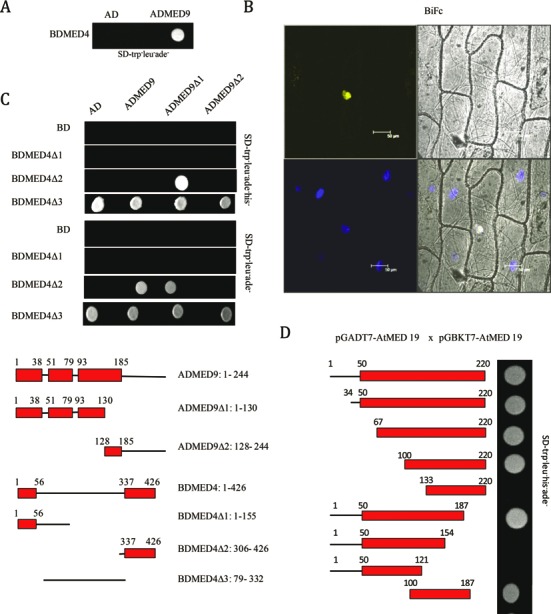
Interaction between Mediator complex subunits of *Arabidopsis*. (**A**) Yeast two-hybrid assay of the interaction between AtMed4 and AtMed9. Yeast cells expressing AtMed4 with AD vector alone and AtMed9 were spotted and grown on SD-Trp^−^/Leu^−^/Ade^−^ media. (**B**) *AtMed4* and *AtMed9* cDNAs were cloned in BiFc vectors and bombarded on onion peel. Fluorescence was observed under confocal microscope after overnight incubation of the peel at 22° C. DAPI (blue) staining was used to identify nuclei in the cells. (**C**) Interaction was fine mapped using deletion constructs of AtMed4 andAtMed9. Upper panel shows yeast two-hybrid assay whereas lower panel shows line diagram of IDRs in the deletion constructs. Red boxes represent IDR regions. (**D**) Schematic view of AtMed19 deletion constructs used in yeast two-hybrid experiments. Red regions represent IDRs. Growth on SD-Trp^−^/Leu^−^/His^−^/Ade^−^ media was used to score interactions.

As mentioned earlier there is no interaction data for plant Mediator subunits in BioGRID ^3.2^ (88) and iRefWeb (88,89). Also, there is no report on structure and IDR of any plant Mediator subunit. For this study, we chose two Mediator subunits, Med4 and Med19 of *Arabidopsis* as AtMed4 has IDR at both N and C termini whereas AtMed19 has a long C-terminus IDR covering more than 75% of the protein. We performed yeast two-hybrid screening with AtMed4 and found 101 proteins interacting with it, establishing it as a ‘hub’ in plant Mediator complex (Figure 5A and B). So many interactions suggest that AtMed4 could be a very important subunit in the Mediator complex. In accordance to this we could not find homozygous lines of T-DNA insertion at *AtMed4* locus in *Arabidopsis* (under preparation). In this screening, AtMed9 also came out as an interactor of AtMed4 (Figures 5B and 6A). We confirmed the interaction by BiFC which suggests that the interaction takes place inside the nucleus (Figure 6B). This is similar to the interaction of Med4 and Med9 already reported in yeast (108) and suggests conserved function of these subunits. However, mapping of the interacting regions in these two plant subunits revealed, previously unknown, involvement of IDRs in the interaction. C-terminal IDR of AtMed4 interacts with the IDR present towards the N-terminus of AtMed9 (Figure 6C) underlining the importance of IDRs in plant Mediator complex. In *Arabidopsis*, interaction of AtMed4 with AtMed9 is specific as it did not show interaction with other randomly selected subunits (AtMed7, AtMed14 and AtMed3) in yeast two-hybrid assay (Supplementary Figure S8). It is interesting to note that though Med4 interacts with Med7 in yeast (108), we did not find such interaction in *Arabidopsis* (Supplementary Figure S8). This suggests that a Mediator subunit can have different interaction repertoire in different kingdoms. In yeast, Med4 interacts with ≈20 proteins whereas in *Arabidopsis* it interacts with 100 proteins (Figure 5). In the case of AtMed19, we found its homodimerization in the yeast two-hybrid assay (Figure 6D). As per our knowledge, formation of dimer by Med19 is not reported in any organism. A big part of AtMed19 (from amino acid 50 to 220) is an IDR. Middle part (100–187aa) of this IDR is important for dimer formation by AtMed19 (Figure 6D). All these results highlight the importance of IDRs in protein–protein interaction within the Mediator complex of *Arabidopsis*.

### MoRFs are protein–protein interaction sites in Mediator complex

**Figure 7.**
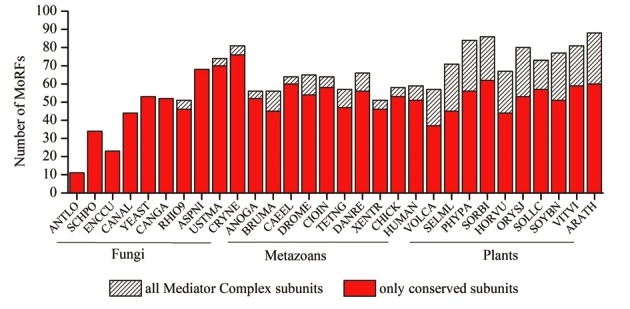
Molecular Recognition Features (MoRFs) in Mediator subunits. Total number of MoRFs in conserved subunits (red bars) and all the subunits (cross-hatched) of 30 model organisms listed on x-axis.

Within disordered regions, the interfaces participating in protein–protein interactions often contain small recognition sites called MoRFs. These small stretches of amino acids are known to undergo disorder-to-order transition upon binding to specific partners. MoRFpred (96) was used to predict these sites in the Mediator subunits of 30 selected model organisms; 10 organisms from each kingdom. We found MoRFs in both conserved and kingdom specific Mediator subunits. The number of predicted MoRFs in conserved subunits was more or less similar in metazoans and plants (Figure 7). Comparatively less number of MoRFs were found in most of the fungi excluding *A. nidulans*, *U. maydis* and *C. neoformans* (Figure 7). Conserved MoRFs were found to be present in the disordered regions of subunits with highly conserved IDRs. For instance, Med6, Med8, Med19, Med1, Med4, Med15, Med16 and Med12 which have highly conserved IDRs in at least one kingdom have conserved MoRFs in their IDRs. Also, the plant specific subunits, Med34, Med35, Med36 and Med37, have MoRFs conserved in almost all the selected plants (Supplementary Figure S9).

In order to understand the functional relevance of MoRFs, we explored already reported structures of Mediator subunits in yeast and human for the presence of MoRFs in them. We found some MoRFs located at the junction of IDR and well-defined helices/strands of a domain, and so, we named them as ‘junction-MoRFs’. In the following examples, we validated the relevance of junction-MoRFs in protein–protein interaction in all the three kingdoms.

**Figure 8.**
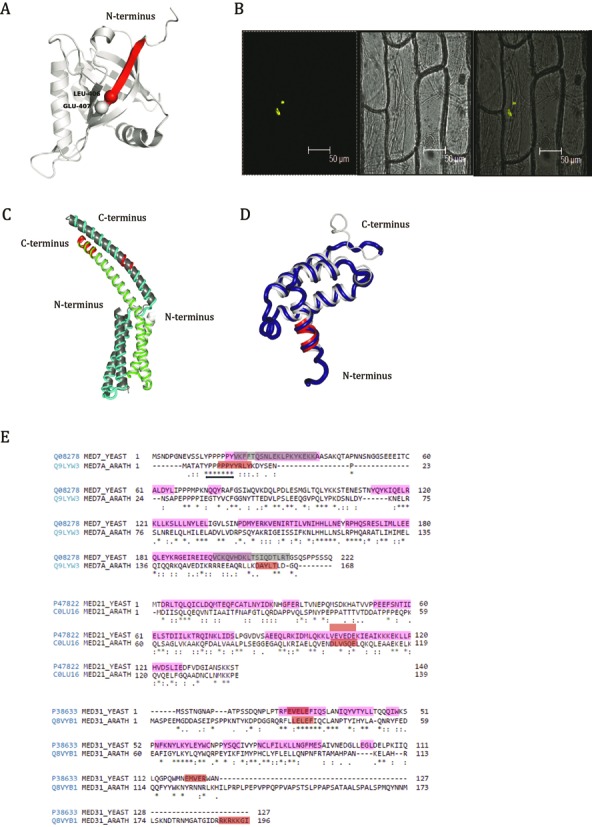
MoRFs in Mediator complex subunits of *Arabidopsis*, yeast and human. (**A**) Junction-MoRF in ACID domain of human Med25 is shown in red. Two Amino acids (L406 and Q407) involved in interaction with VP16 TADs are highlighted as spheres. Structure of ACID domain was generated using PDB id 2XNF. (**B**) Interaction between AtMed7 and AtMed21 was confirmed by BiFC assay. (**C**) Homology models of AtMed7 (green), AtMed21 (cyan) and (**D**) AtMed31 (blue) are aligned on known yeast counterparts (PDB id 1YKH and PDB id 3FBI in gray). MoRFs in all the structures are shown in red color. (**E**) Conserved MoRFs in Med7, Med21 and Med31 of yeast and *Arabidopsis* are shown as red and gray boxes in sequence alignment. Gray boxes represent MoRFs published earlier whereas red boxes represent MoRFs predicted in this study. Double headed arrow represents the highly conserved poly proline stretch flanking MoRF in Med7. Residues with helical propensity are highlighted in pink. Interestingly, some MoRFs are in these highlighted regions.

In human Med25, there is an IDR (233–390) preceding the ACID domain (393–541). The interaction of HsMed25 ACID domain with helix1 and helix2 of VP16 TADs was elucidated through NMR spectroscopy and reported elsewhere (109). The helices bind to the opposite surfaces of ACID domain in a cooperative manner. We found a junction-MoRF at 400–406 that is involved in the binding with helix 2 (Figure 8A). The residues L406 and Q407 towards IDR form parallel β-strand in a seven antiparallel β barrel and are important for interaction with full length TAD of VP16. It is interesting to note that V405 in the junction-MoRF is an exposed residue that is conserved in animals.

There is no report on the IDRs and MoRFs present in the plant Mediator subunits. We employed BiFC and found that AtMed7 and AtMed21 interact with each other (Figure 8B). This is a conserved interaction found to be crucial in yeast where these two proteins and Med31 participate in complex formation (66). In our analysis, we found MoRFs in AtMed7, AtMed21 and AtMed31. So, in order to understand the importance of these MoRFs in *Arabidopsis*, AtMed7, AtMed21 and AtMed31 were modeled on the available structures (1YKH and 3FBI) of yeast proteins using Phyre2 (99). All the structures were modeled with >90% confidence. Quality of the modeled structure was assessed by PROCHECK (Supplementary Figure S10) and the detailed statistics of the modeling is given in the Supplementary Table ST5 (100). The models thus obtained aligned well on the templates with backbone RMSD of 0.66, 3.99 and 0.6Å for Med7, Med21 and Med31, respectively (Figure 8C and D). The higher backbone RMSD of Med21 is due to the absence of amino acids from 34 to 48 in the crystal structure of 1YKH which along with the long linker region caused increase in the RMSD value (Figure 8C). The quaternary structures of the subunits appear to be conserved in yeast and *Arabidopsis*. Our analysis predicted a novel MoRF in AtMed21 (101–106) falling in the centre of the coiled coil region indicating that this region could probably change its conformation as and when required (Figure 8C). MoRFs predicted in AtMed7 align well with that reported in ScMed7 (Figure 8E). A stretch of proline amino acids which partially form the MoRF at the N-terminal end is highly conserved in yeast, *Arabidopsis* and human (Supplementary Figure S9). While a part of the C-terminal MoRF in yeast forms the terminal coiled-coil of Med7, the rest of the MoRF in yeast and the one of *Arabidopsis* flanks the terminal coiled-coil in the model. The missing part has preponderance for disorder and therefore has not been captured in the crystal structure and hence could not be modeled. In Med31, the N-terminal MoRF is highly conserved in both yeast and *Arabidopsis* and both show helical propensity (Figure 8E). Although the sequence length varies between the two organisms, both have a MoRF at the C-terminal end (Figure 8E) suggesting structural/functional conservation.

### Junction-MoRF in the extended IDR following the KIX domain of CBP has evolved from Med15

**Figure 9.**
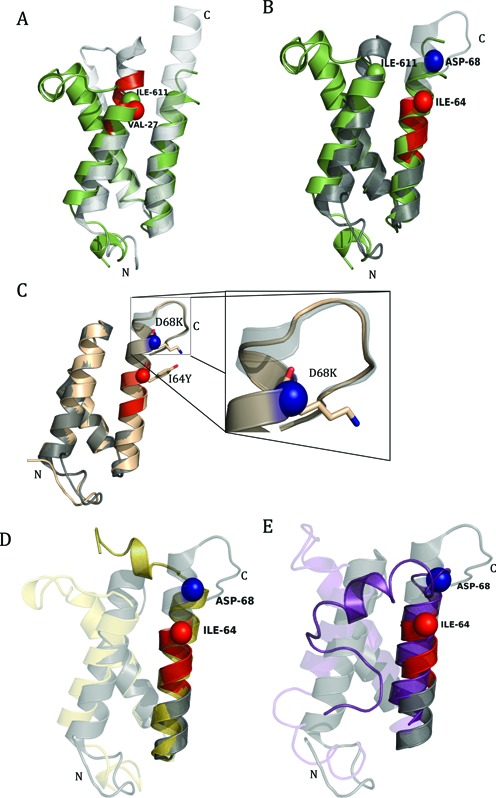
Junction-MoRF in human and yeast Med15 KIX domains. (**A**) Alignment of yeast Med15-KIX (gray) with human CBP-KIX (green). Yeast KIX structure was generated using PDB id 2K0N. Mutation at VAL-27 (red sphere) reduces the binding affinity of yeast Med15-KIX. VAL-27 aligns with ILE-611 (green sphere) which is the start of G2-loop in human CBP-KIX. (**B**) Alignment of human Med15-KIX (gray) with human CBP-KIX (green). KIX domain of human Med15 was generated using PDB id 2GUT. Residues ILE-64 (red sphere) and ASP-68 (blue sphere) play a role in maintaining the binding specificity of human Med15-KIX. (**C**) Alignment of mutated (I64Y and D68K) KIX (wheat) with human KIX (gray) domain. (**D**) Homology Model of *Capsaspora owczarzaki* CBP-KIX (yellow) aligned on human CBP-KIX (gray). (**E**) Homology model of *Salpingoeca rosetta* CBP-KIX (purple) aligned on Human CBP-KIX (gray). Homology models of *C. owczarzaki* and *S. rosetta* CBP-KIX were generated using protein sequences E9CAO3 and F2TVQ2 respectively. Protein sequences were retrieved from UniProt. Human CBP-KIX (gray) was generated using PDB id 2LXT. In all the structures, MoRFs are highlighted in red color, C and N represented C-terminus and N-terminus, respectively.

Med15, a subunit in the Tail module, physically interacts with many unrelated gene specific transcription factors both in metazoans and fungi. In yeast, Med15 interacts with Pdr1 and Oaf1 to regulate multidrug resistance and fatty acid homeostasis (19). At its amino terminus, Med15 has a KIX domain which is a three helix bundle containing two loop regions in between them (110). We looked at the NMR spectroscopy data explaining the interaction of KIX domain of ScMed15 with activation domain of transcription factors Oaf1 and Pdr1 (19). In this analysis, we found a junction-MoRF at the C-terminus of helix 1 of the KIX domain from residue 25 to 31 (Figure 9A). Significant chemical shift perturbations were observed in this region on titrating KIX with the activation domain of the transcription factors. A point mutation, V27D, in this region was reported to affect its binding affinity with Pdr1 and Oaf1 (19,31). When we aligned the yeast ScMed15-KIX with human CBP-KIX, we found that the start point of G2-loop region in CBP, I611, overlaps with V27 residue of ScMed15-KIX (Figure 9A). Importantly, I611 plays a key role in the allosteric modulation of CBP-KIX interactions with c-Myb and CREB (110). Thus, it seems that the extended IDR in CBP is originated from extension of an IDR of ScMed15 into helix 1 of the KIX domain. To the best of our knowledge yeast does not have any CBP orthologs. However, Ichthyosporea and choanoflagellates, which are evolutionarily placed between metazoans and fungi, have HAC proteins with the conserved CBP motif (LxxxxYxxxK) in the third helix. Thus, this interesting discovery provides a direct evidence for the evolution of functional junction-MoRF in the IDR next to helix 1 of the KIX domain of CBP from Med15-KIX.

In order to validate our hypothesis of evolution of junction-MoRF in an IDR following the KIX domain of CBP from KIX domain of Med15, we looked at these proteins in the same organism (Figure 9B). In human, KIX domain of Med15 (ARC105) has been shown to interact with transcription factor SREBP1 but not to c-MYB and CREB (111). We found one MoRF (from 58 to 64 residues) near the C-terminus of helix 3 that plays a crucial role in the interaction with SREBP1 (111). Two mutations, I64Y and D68K, in HsMed15-KIX make it bind to CREB and c-MYB, and so mimic CBP-KIX. It was observed that when helix 3 is truncated by six to eight residues, free CBP-KIX unfolds or aggregates depending on the pH of the solution. This suggests its probable tendency to be disordered (112). In fact, the average structure of CBP-KIX shows a completely unfolded helix 3 C-terminus beginning at residue 657 (113). Alignment of different stabilized structures of liganded CBP-KIX revealed importance of C-terminus of third helix (Supplementary Figure S11). Upon ligand binding, the C-terminal residues of helix 3 are stabilized and there is a significant increase in the helicity. Indeed, homology modeling of HsMed15-KIX with these double mutations (I64Y and D68K) revealed increased disorder in the C-terminal region of the third helix, making it similar to CBP (Figure 9C). Statistics of homology modeling is given in the Supplementary Table ST6. Double mutation with other amino acids in these sites also disrupts the helix at position 68 and further increases the extent of disorder (Supplementary Figure S12). Also, homology modeling of CBP-KIX of primitive organisms reveals the malleability of third helix (Figure 9D and E). The third helix of CBP-KIX in these two organisms appears to be disordered after the amino acid corresponding to D68 in human Med15-KIX. This provides a second line of evidence that the KIX domain and IDR following it in Med15 and CBP could have evolved from the same ancestor to serve specific cellular functions. This also suggests that during evolution, sequences in Med15 provided template for the formation of extended IDR with junction-MoRF in CBP.

## DISCUSSION

Mediator complex is a gigantic multiprotein complex found in all the eukaryotes. It plays a critical role in transcription by relaying signals from transcription regulators to RNA polymerase. In response to different signals, Mediator hosts many different types of transcription factors, cofactors and other proteins (106,111). Though overall structure of Mediator complex is similar in different organisms, they can also accommodate kingdom specific proteins including transcription factors (114). Not only in the initiation of transcription, but involvement of Mediator has also been established in elongation of transcripts, splicing of primary transcript, gene looping and termination of transcription (108–110). In some cases, Mediator also functions as a co-repressor (115,116). In all these different functions, Mediator interacts with diverse group of proteins and complexes. These interactions change the overall conformation of Mediator complex, highlighting its structural flexibility (67). Nonetheless, the subunit composition and hence the modular architecture of Mediator complex subunits varies even between closely related organisms (62). It is only logical then to presume that the working mechanism of this huge complex might vary from species to species. Primary amino acid sequences of many of the Mediator subunits are not so well conserved and contain disordered regions. We think that the disordered regions might have evolved to render flexibility to the complex and make it accommodate so many interactions, some of them specific to different kingdom.

In the present study, Mediator complexes of metazoans, plants and fungi were analysed for the evolution of specific disorder patterns and their functional or structural role in each kingdom. The analysis revealed that the extent of disorder and placement of IDRs in some subunits is evolutionarily conserved across the kingdoms (Figures 1 and 2). However, in many subunits, the kingdom- or group-specific positioning of IDRs is also observed (Supplementary Figures S1–S5). Plants and fungi have highest and lowest number of conserved IDRs, respectively, partly due to presence of conserved IDRs in kingdom-specific subunits. Thus, IDR could be acquired or lost specifically in a group or kingdom. In metazoans, disordered regions appear to have resulted due to domains gained during evolution (117). An extension of an existing exon into previously non-coding regions can result in enrichment of disordered regions (118). In *D. melanogaster* the extension of exon at the carboxyl terminus appears to be predominant which explains the appearance of restricted IDRs in this group in Med11 and Med23 (Supplementary Figure S2). In human, mouse and frog, both the termini gained novel exons which explains the conservation of IDRs at the N-terminus of Med9 and Med17, and at the C-terminus of Med7 and CycC in higher metazoans. The observed pattern of IDR in Med28 could be due to mix of domain gain through insertion of novel exons at N-terminus and through exon extension in the C-terminus of *D. rerio* (118). On the other hand, loss of IDRs between closely related organisms appears to have resulted due to selection pressures on IDRs after gene duplication events during evolution (119). For example, the short IDR found towards the C-terminus of Med14 of *C. elegans* and *C. brenneri* is absent in *C. remanei* and *C. briggsae* (Supplementary Figure S2). Similarly, IDR at the N-terminus of Med14 in higher metazoans selectively appears or disappears in closely related organisms. Overall, we found increase in disorder of Mediator complex from lower simpler organisms to higher complex organisms and in general, is conserved within a kingdom (Figure 1B).

Distribution of IDRs along the length of proteins revealed that Mediator subunits, in general, have higher propensity to possess IDR towards C- and N-termini (Figure 2). This is similar to other known functional proteins like cryptochromes and nuclear hormone receptors (116–118). Thus, distribution of IDRs in Mediator subunits is consistent with that found in other functional proteins involved in signaling and transcriptional regulation. Just like the trend prevalent across whole proteome, Mediator subunits have more number of short IDRs than long IDRs (data not shown). There are some subunits which have higher number of short IDRs in their middle regions probably to allow structural flexibility and allosteric cross talk between multiple domains (120,121). It is clear that disorder of the Mediator complex subunits plays a crucial role in maintaining the structural pliability of the complex in a kingdom specific manner and thus is able to interact with different number and types of protein partners in different kingdoms. In fact, overall disorder and the distribution and conservation of IDRs in different organisms of the same kingdom further suggests that the conformational pliability of Mediator complexes and its modules might have even diverged between different organisms of the same kingdom. We think that the differential conservation, gain or loss of disorder and disordered regions might have allowed Mediator subunits in modular assembly and to reconfigure and rewire the interaction network repertoire of the whole complex. The plasticity of the network could then facilitate emergence of novel functions and acquisition of additional subunits or domains in pre-existing subunits (122).

The Head and Middle modules of the Mediator complex are highly conserved throughout the eukaryotes and therefore constitute the core part of the complex (123). In Head module, Med6 is one of the most conserved subunits (17). Med6 has a conserved IDR at the C-terminal end in metazoans, plants and fungi and appears to be a hub of protein–protein interactions (Figure 4). Importance of Med6 is evident from the fact that it interacts with general transcription factor GTF2B and nuclear hormone receptor VDR (123). Med6 acts as a conserved flexible bridge between the Head and Middle modules by physically coupling to Med17 of Head and Med21 of Middle modules (66). The ‘unstructural’ integrity of Med6 is probably the key to maintain the architecture and function of the core Mediator part. This explains the high degree of conservation of IDR of Med6 across the three kingdoms. About 82% Metazoans, 76% plants and 80% of fungi have an IDR at the C-terminal end of Med19 (Figure 2). In addition, 74% Metazoans and 50% fungi have a second IDR in the middle region of Med19. Med19 is known to bridge transcription factors and RNA PolII and stabilize the architecture of Mediator complex (124,125). In human, Med19 interacts with Med17, Med31 and Med3 of Head, Middle and Tail modules (Supplementary Figure S13) and so has a stabilizing effect on Mediator architecture. Also, Med19 has a conserved lysine rich Homeodomain Interacting Motif (HIM) in its IDR which has a conserved MoRF in all the eukaryotes (Supplementary Figure S9) (125). In *Arabidopsis*, ability to homodimerize was mapped to IDR of Med19 from 100–187 residues (Figure 6D). At least 90% metazoans, plants and fungi have a conserved IDR at the N-terminal end of Med15, a subunit in the Tail module (Figure 2). A second IDR is predicted in the middle regions of Med15 in 97% plants and 87% fungi. Med15 of 64% plants and 91% of fungi have a third IDR in the C-terminal region. Like Med19, Med15 shows a high average disorder. In fungi and animals, Med15 has been shown to interact with TADs of various unrelated transcription factors. Med15 mutants in *Arabidopsis* are insensitive to salicylic acid and impaired in systemic acquired resistance (126). Though not known yet, this could be due to interaction of Med15 with other proteins involved in salicylic acid signaling. In rice, Med15 has been proposed to regulate seed development by interacting with transcription factors involved in the process (127). In our study, all the animals and more than 90% and 87% of plants and fungi, respectively, were found to have an IDR at the carboxyl end of Med4 (Figure 2). We also found another IDR at the N-terminal end in 77% of the plants. In *Arabidopsis*, the C-terminus IDR of Med4 interacts with Med9 (Figure 6C). In yeast, Med4 interacts with all the Middle module subunits except Med1. It also interacts with Med17 and Med3 of Head and Tail modules, respectively. C-terminal of Med4 has a highly conserved IDR (Figure 2) and is known to be necessary for the viability of yeast cells (59). Med25 is present mostly in metazoans and plants and at least 80% have highly conserved IDRs at the C-terminus and middle region (Figure 2). In both human and *Arabidopsis*, Med25 is reported to be the hub of several protein–protein interactions (43). Human Med25 interacts with many transcription factors and with the Middle module subunit Med4. It is also implicated in retinoic acid resistance in cancer therapy (128). Similarly, in *Arabidopsis*, Med25 interacts with several transcription factors and is known to be involved in abscisic acid and jasmonate signaling pathways (129). In addition, it provides resistance to necrotrophic pathogens and determines final size of determinate organs (130,131). The junction-MoRF located at the junction of ACID domain and preceding IDR is conserved across all the model organisms chosen for the study (Supplementary Figure S9). Thus, the disordered region and junction-MoRF of Med25 might be involved in common mechanism of gene regulation in signaling pathways of different kingdoms. Med13 belonging to the detachable Kinase module harbors IDRs at the N- and middle regions in at least 80% of all metazoans, plants and fungi (Figure 2). Deletion of Med13 causes anomalies in eye and wing development in *Drosophila* (132,133). Also, congenital heart and neuronal defects can result from mutations in Med13 (24). All these examples reveal that Mediator subunits with IDRs play important role in fundamental cellular and physiological processes.

IDRs are known to provide interaction surface to multiple partners owing to its conformational flexibility. IDRs perform several functions such as inhibitors, competitors, activators, benders and twisters, affinity tuners, signal carriers, interwinders, switchers, recruiters and assemblers (105). Consistent with the number of cited roles for IDRs, a strong correlation was observed between the presence of IDR in a subunit and its role as a hub. When all the reported interactions were analysed for each subunit, the number of hubs with IDRs was significantly greater than hubs without IDRs (Figure 4B). Particularly interesting is Med17, which appears to hold the Mediator complex together in both human and yeast by interacting with several other subunits (Figure 4A). Deletion of Med17 resulted in loss of conserved Head module and was reported to be lethal to yeast culture (134). In fact, it has been shown that Med17 plays a major structural role in the Head module architecture (56). In yeast, the Middle module subunits in yeast have higher number of inter-subunit contacts and almost all of them act as hubs. In human, Med28 and Med30 of the Head module have higher number of inter-subunit interactions and therefore probably keep the Head module intact. Med1 in human has relatively high number of interactions compared to other subunits (Figure 4B). Human Med1 has the longest IDR among all the subunits and interacts with most of the nuclear hormone receptors, a group of ligand-activated transcription factors (2). Med1 is present in metazoans and fungi, but the C-terminal IDR is specific only to metazoans (15). Unlike in metazoans, ligand-activated transcription factors of yeast and fungi do not interact with Med1 (19,31). The increase in the number of interactions from yeast to human Med1 is therefore in accord with the novel and crucial functional roles acquired by disordered region of human Med1. In contrast, Med3 and Med18 have an IDR in yeast but not in human which could be a contributing factor to the decrease in the number of interactors of these subunits in human.

IDRs are known to modulate the protein's functional profile through short stretches of preformed elements or MoRFs which impart low affinity but high specificity for the interacting partner (135). MoRFs were found to be conserved in conserved IDRs. The different degree of conservation of IDRs in Mediators could be to conserve these MoRFs which appear to be kingdom-specific in many cases (Supplementary Figure S9). Also, conserved MoRFs were quite prevalent, as expected, in the kingdom-specific subunits. This corroborates our hypothesis that additional subunits and their IDRs might have evolved to acquire novel functions as per the requirement. Further, these stretches were implicated in maintaining the structural integrity of the domain and binding affinity and specificity (19,68,111). Most interestingly, we found strong evidence to support their contribution to evolution of domains and thus protein diversity to modulate the interaction repertoire of the subunit. For example, our analysis of junction-MoRFs suggests the evolutionary link between metazoan CBP-KIX-IDR and yeast Med15-KIX-IDR. The junction-MoRF in ScMed15-KIX-IDR has got comparable properties as G2-loop in animal CBP-KIX-IDR (Figure 9A). The absence of CBP proteins in yeast but their presence in Icthyosporea and Choanoflagellida suggests an event of domain gain. Homology modeling of CBP-KIX sequences in these primitive organisms indicates that the third helix is malleable whose structure breaks following the junction-MoRF towards carboxyl end (Figure 9D, E). Purified CBP-KIX of mouse is not stable and forms aggregate, and upon interacting with other peptides like pKID forms a stable complex (106). Moreover, mutations within the junction-MoRF of Med15-KIX-IDR reduce its binding specificity and increase the disorder at the C-terminal end of the third helix mimicking CBP-KIX-IDR (Figure 9C). Furthermore, homology modeling of KIX like sequences in plants reveals a short third helix indicating the malleability of this region (data not shown). Our analysis therefore suggests a strong link between the extension or evolution of disordered regions through junction-MoRFs, may be in response to increased diversity of interacting proteins including transcription factors. We think that junction-MoRFs provide partial conformational heterogeneity to the neighboring structured domain, and thus, create an environment which makes disorder-to-order transition a fast and feasible process.

The importance of IDRs and MoRFs in *Arabidopsis* was further established by experimental validations. AtMed4, which has IDRs at both the termini, was found to interact with >100 proteins (Figure 5). Surprisingly, unlike in yeast, it did not show interaction with AtMed7 suggesting that Mediator subunits might have different interacting partners in different systems. However, like in yeast, AtMed4 was found to interact with AtMed9 (Figures 5B and 6A). Previously unknown role of the IDR regions in the interaction of AtMed4 (306–426) and AtMed9 (1–130) could be discovered in our analysis (Figure 6C). The interaction between these two subunits already reported in yeast appears to be crucial for the structural integrity of the Mediator complex as ScMed4 and ScMed9 interact with most other Middle module subunits. Deletion of ScMed9 affects the modular architecture of the complex (136). The interaction between Med7 and Med21, and their MoRFs are conserved between yeast and *Arabidopsis*. Homology modeling further revealed conserved quaternary structures which suggest that the mechanism of interaction between Med7 and Med21 is also conserved. It appears that these subunits maintained their functions throughout the course of evolution, which explains the conservation of MoRFs across the three kingdoms (Supplementary Figure S9).

Many Mediator subunits have regions rich in one or two amino acids within their IDRs (Supplementary Figure S9). The higher density of a particular amino acid in a region probably enhances the propensity of specific protein–protein interactions (137). Glutamine rich regions are reported to be involved in interaction with different transcription factors (138). Med15, Med25 and many other Mediator subunits contain glutamine rich regions. Proline repeats are found in proteins that interact with SH3 and SH2 domain proteins, EH domain proteins and 14–3–3 domain proteins. The C-terminus IDR of Med1 in *C. elegans* lacks the conserved LxxLL motif. This is compensated with the proline rich region to make it interact with SH3 domain of T04C9.1 (139). Putative LxxLL motifs, which are implicated in the interaction with nuclear hormone receptors, are present in several Mediator subunits in the model organism. LxxLL motif also forms the core pattern of ϕxxϕϕ found in several TADs (140). It is possible that these motifs act as MoRFs depending on their location (Supplementary Figure S9). Thus, Mediator subunits probably modulate the interaction repertoire of the complex by competitive or cooperative binding depending on the ambient conditions. This explains the variable subunit composition in different conditions and the interaction of one protein with several Mediator subunits.

## CONCLUSION

Mediator has been found to be involved in almost all the aspects of transcription of class II genes. In this study, it was found that many Mediator subunits are disordered and contain short or long disordered regions that might be rendering the requisite flexibility to the complex. Conservation of extent of disorder and positioning of IDRs in some Mediator subunits indicate that a basal level of flexibility is conserved in all the eukaryotes. However, there are kingdom, and within a kingdom, group specific IDRs in some selected Mediator subunits to cater to the requirement of interaction with kingdom and group specific transcription factors and proteins. Thus, this study addresses not only the conserved function of Mediator but also correlates the gain or loss of IDRs in some Mediator subunits with kingdom specific processes. This is the first report which gives details of IDRs in plant Mediator subunits and provides structural insight for some of them. Experimental data have been provided to demonstrate the involvement of IDRs of *Arabidopsis* Mediator subunits in protein–protein interaction. Presence of PTM sites within IDRs was analysed, and several PTM hotspots were characterized. Usually, protein–protein interaction by an IDR happens through MoRF(s). This study has introduced a novel concept of junction-MoRFs physically localized at the junction of well-structured domain and disordered region, and raised an important concept of extension of disordered regions in the existing neighboring structured domain to incorporate more flexibility and broaden the diversity of protein–protein interaction.

There are so many reports that implicate Mediator subunits in homeostasis, multidrug resistance, peroxisome proliferation and function, signaling pathways and other growth and development related phenomenon. Mutation(s) in IDRs of Mediator subunits have been related to many deformities and diseases. We think that this study will trigger a burst of research activity in search for small molecules targeting IDRs in Mediator subunits. We also hope that this study will serve as a platform to popularize the disordered regions and MoRFs/junction-MoRFs of Mediator subunits among structural biologists.

## Supplementary Material

SUPPLEMENTARY DATA
