# Supplementary material for: Evolution of disorder in Mediator complex and its functional relevance
Source: Nucleic Acids Res. 2015 Nov 20;44(4):1591–612. doi: 10.1093/nar/gkv1135 (PMC4770211; doi:10.1093/nar/gkv1135)
Supplement: SUPPLEMENTARY DATA [file supp_44_4_1591__index.html]

Evolution of disorder in Mediator complex and its functional relevance — Evolution of disorder in Mediator complex and its functional relevance — SUPPLEMENTARY DATA 

# Evolution of disorder in Mediator complex and its functional relevance

## SUPPLEMENTARY DATA

- SUPPLEMENTARY DATA
