# Supplementary material for: Evolution of disorder in Mediator complex and its functional relevance
Source: Nucleic Acids Res. 2015 Nov 20;44(4):1591–612. doi: 10.1093/nar/gkv1135 (PMC4770211; doi:10.1093/nar/gkv1135)
Supplement: SUPPLEMENTARY DATA [file supp_gkv1135_nar-01763-n-2015-File011.zip › SF_1.pdf]

This file contains a schematic of the Intrinsically disordered regions (IDRs) in the Mediator complex subunits (Med1, Med2/Med29, Med3/Med27, Med4, Med5/Med24, Med6, Med7, Med8, Med9 and Med10) of Metazoans. List of organisms used in the current study are present in supplementary table ST1

MED1

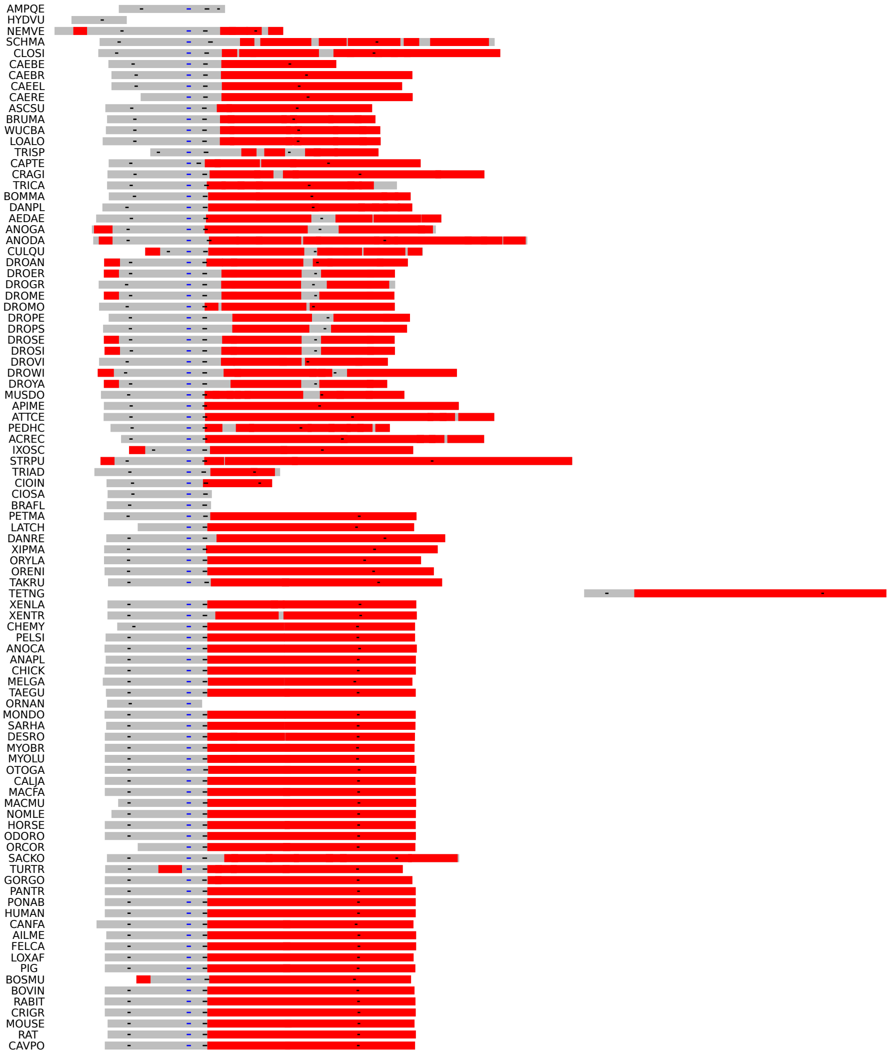

AMPQE
HYDVU
NEMVE
SCHMA
CLOSI
CAEBE
CAEBR
CAEEL
CAERE
ASCSU
BRUMA
WUCBA
LOALO
TRISP
CAPTE
CRAGI
TRICA
BOMMA
DANPL
AEDAE
ANOGA
ANODA
CULQU
DROAN
DROER
DROGR
DROME
DROMO
DROPE
DROPS
DROSE
DROSI
DROVI
DROWI
DROYA
MUSDO
APIME
ATTCE
PEDHC
ACREC
IXOSC
STRPU
TRIAD
CIOIN
CIOSA
BRAFL
PETMA
LATCH
DANRE
XIPMA
ORYLA
ORENI
TAKRU
TETNG
XENLA
XENTR
CHEMY
PELSI
ANOCA
ANAPL
CHICK
MELGA
TAEGU
ORNAN
MONDO
SARHA
DESRO
MYOBR
MYOLU
OTOGA
CALJA
MACFA
MACMU
NOMLE
HORSE
ODORO
ORCOR
SACKO
TURTR
GORGO
PANTR
PONAB
HUMAN
CANFA
AILME
FELCA
LOXAF
PIG
BOSMU
BOVIN
RABIT
CRIGR
MOUSE
RAT
CAVPO

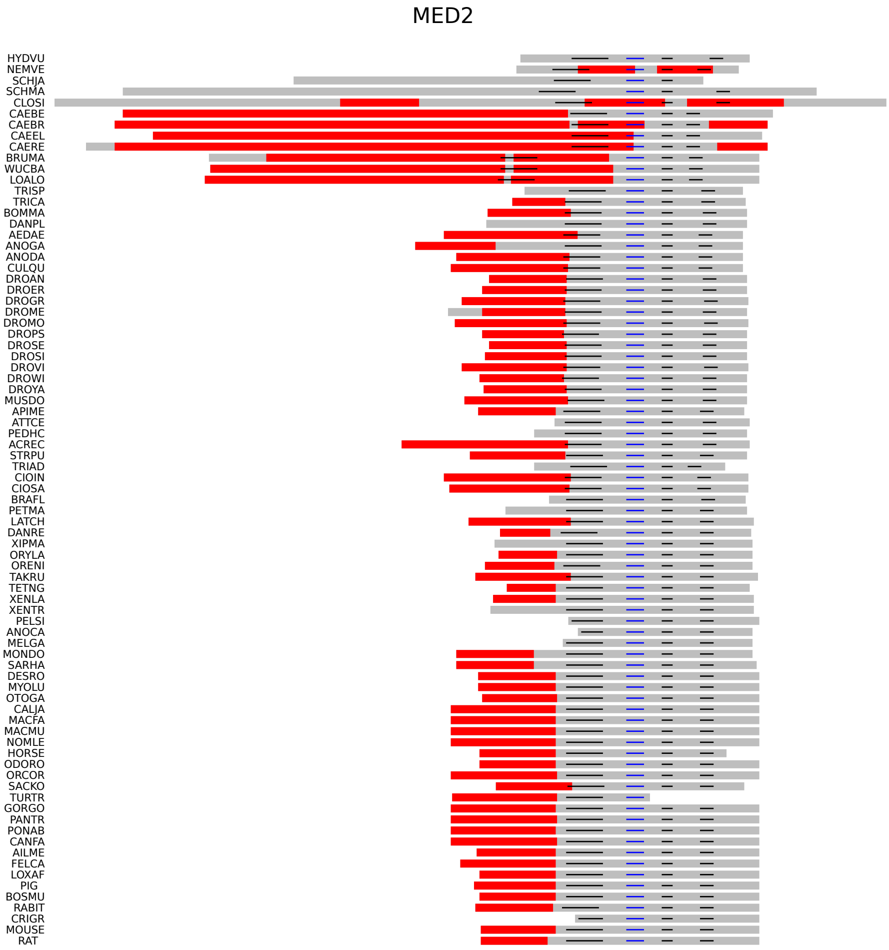
MED2
HYDVU
NEMVE
SCHJA
SCHMA
CLOSI
CAEBE
CAEBR
CAEEL
CAERE
BRUMA
WUCBA
LOALO
TRISP
TRICA
BOMMA
DANPL
AEDAE
ANOGA
ANODA
CULQU
DROAN
DROER
DROGR
DROME
DROMO
DROPS
DROSE
DROSI
DROVI
DROWI
DROYA
MUSDO
APIME
ATTCE
PEDHC
ACREC
STRPU
TRIAD
CIOIN
CIOSA
BRAFL
PETMA
LATCH
DANRE
XIPMA
ORYLA
ORENI
TAKRU
TETNG
XENLA
XENTR
PELSI
ANOCA
MELGA
MONDO
SARHA
DESRO
MYOLU
OTOGA
CALJA
MACFA
MACMU
NOMLE
HORSE
ODORO
ORCOR
SACKO
TURTR
GORGO
PANTR
PONAB
CANFA
AILME
FELCA
LOXAF
PIG
BOSMU
RABIT
CRIGR
MOUSE
RAT

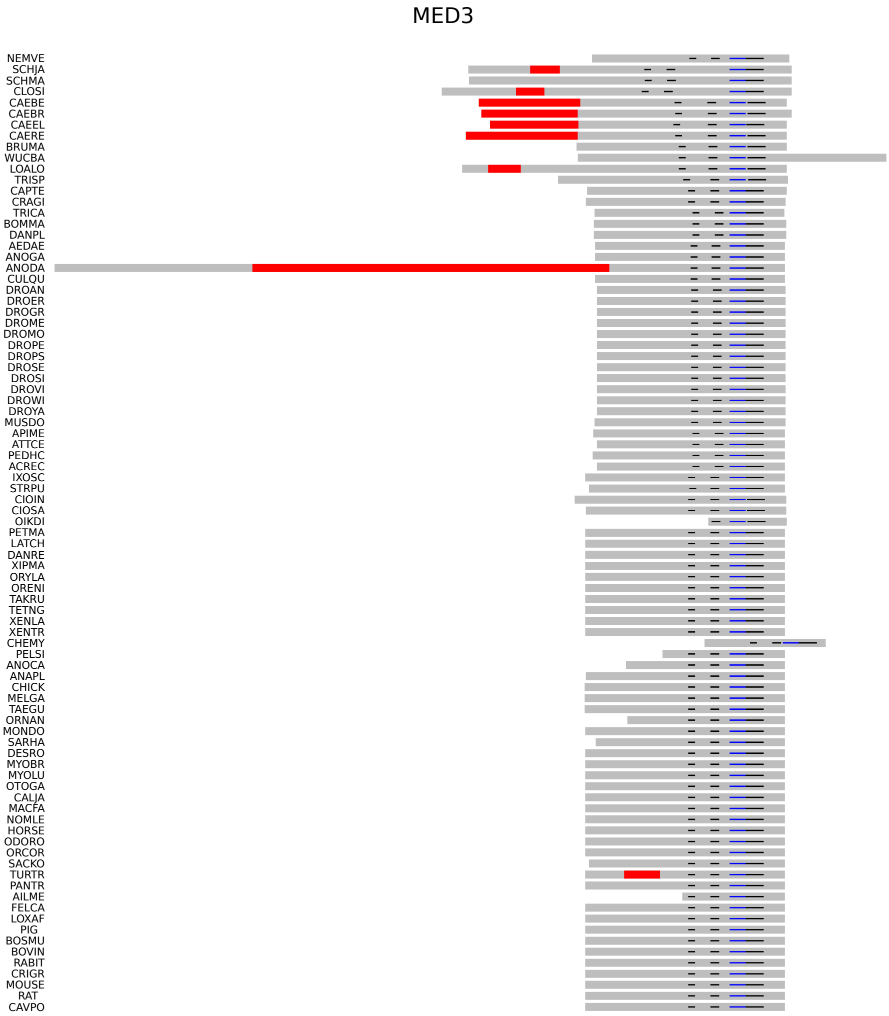
MED3
NEMVE
SCHJA
SCHMA
CLOSI
CAEBE
CAEBR
CAEEL
CAERE
BRUMA
WUCBA
LOALO
TRISP
CAPTE
CRAGI
TRICA
BOMMA
DANPL
AEDAE
ANOGA
ANODA
CULQU
DROAN
DROER
DROGR
DROME
DROMO
DROPE
DROPS
DROSE
DROSI
DROVI
DROWI
DROYA
MUSDO
APIME
ATTCE
PEDHC
ACREC
IXOSC
STRPU
CIOIN
CIOSA
OIKDI
PETMA
LATCH
DANRE
XIPMA
ORYLA
ORENI
TAKRU
TETNG
XENLA
XENTR
CHEMY
PELSI
ANOCA
ANAPL
CHICK
MELGA
TAEGU
ORNAN
MONDO
SARHA
DESRO
MYOBR
MYOLU
OTOGA
CALJA
MACFA
NOMLE
HORSE
ODORO
ORCOR
SACKO
TURTR
PANTR
AILME
FELCA
LOXAF
PIG
BOSMU
BOVIN
RABIT
CRIGR
MOUSE
RAT
CAVPO

MED4

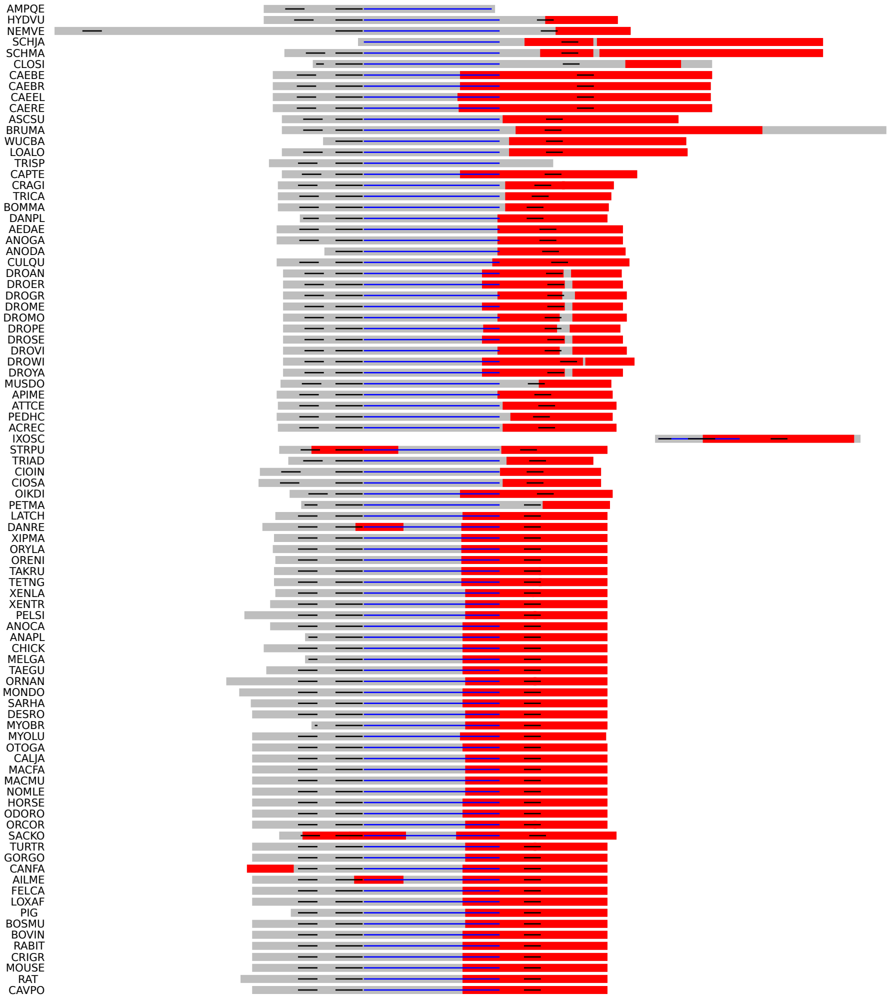
AMPQE
HYDVU
NEMVE
SCHJA
SCHMA
CLOSI
CAEBE
CAEBR
CAEEL
CAERE
ASCSU
BRUMA
WUCBA
LOALO
TRISP
CAPTE
CRAGI
TRICA
BOMMA
DANPL
AEDAE
ANOGA
ANODA
CULQU
DROAN
DROER
DROGR
DROME
DROMO
DROPE
DROSE
DROVI
DROWI
DROYA
MUSDO
APIME
ATTCE
PEDHC
ACREC
IXOSC
STRPU
TRIAD
CIOIN
CIOSA
OIKDI
PETMA
LATCH
DANRE
XIPMA
ORYLA
ORENI
TAKRU
TETNG
XENLA
XENTR
PELSI
ANOCA
ANAPL
CHICK
MELGA
TAEGU
ORNAN
MONDO
SARHA
DESRO
MYOBR
MYOLU
OTOGA
CALJA
MACFA
MACMU
NOMLE
HORSE
ODORO
ORCOR
SACKO
TURTR
GORGO
CANFA
AILME
FELCA
LOXAF
PIG
BOSMU
BOVIN
RABIT
CRIGR
MOUSE
RAT
CAVPO

MED5

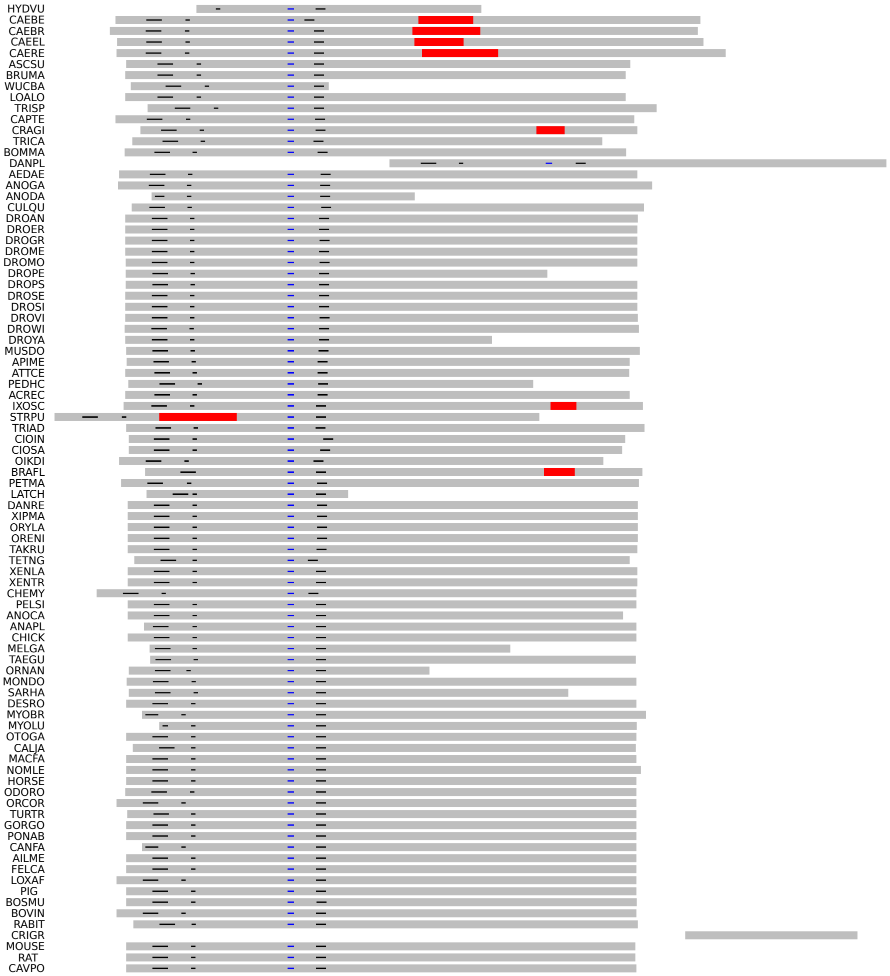
HYDVU
CAEBE
CAEBR
CAEEL
CAERE
ASCSU
BRUMA
WUCBA
LOALO
TRISP
CAPTE
CRAGI
TRICA
BOMMA
DANPL
AEDAE
ANOGA
ANODA
CULQU
DROAN
DROER
DROGR
DROME
DROMO
DROPE
DROPS
DROSE
DROSI
DROVI
DROWI
DROYA
MUSDO
APIME
ATTCE
PEDHC
ACREC
IXOSC
STRPU
TRIAD
CIOIN
CIOSA
OIKDI
BRAFL
PETMA
LATCH
DANRE
XIPMA
ORYLA
ORENI
TAKRU
TETNG
XENLA
XENTR
CHEMY
PELSI
ANOCA
ANAPL
CHICK
MELGA
TAEGU
ORNAN
MONDO
SARHA
DESRO
MYOBR
MYOLU
OTOGA
CALJA
MACFA
NOMLE
HORSE
ODORO
ORCOR
TURTR
GORGO
PONAB
CANFA
AILME
FELCA
LOXAF
PIG
BOSMU
BOVIN
RABIT
CRIGR
MOUSE
RAT
CAVPO

MED6

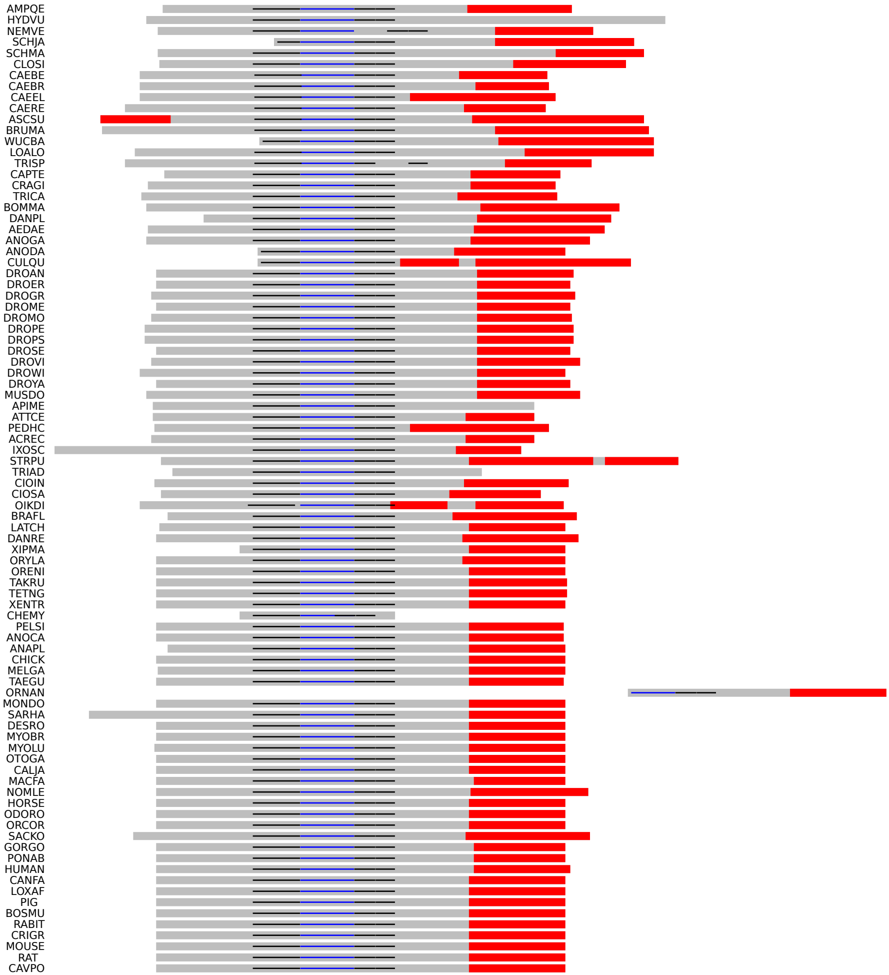
AMPQE
HYDVU
NEMVE
SCHJA
SCHMA
CLOSI
CAEBE
CAEBR
CAEEL
CAERE
ASCSU
BRUMA
WUCBA
LOALO
TRISP
CAPTE
CRAGI
TRICA
BOMMA
DANPL
AEDAE
ANOGA
ANODA
CULQU
DROAN
DROER
DROGR
DROME
DROMO
DROPE
DROPS
DROSE
DROVI
DROWI
DROYA
MUSDO
APIME
ATTCE
PEDHC
ACREC
IXOSC
STRPU
TRIAD
CIOIN
CIOSA
OIKDI
BRAFL
LATCH
DANRE
XIPMA
ORYLA
ORENI
TAKRU
TETNG
XENTR
CHEMY
PELSI
ANOCA
ANAPL
CHICK
MELGA
TAEGU
ORNAN
MONDO
SARHA
DESRO
MYOBR
MYOLU
OTOGA
CALJA
MACFA
NOMLE
HORSE
ODORO
ORCOR
SACKO
GORGO
PONAB
HUMAN
CANFA
LOXAF
PIG
BOSMU
RABIT
CRIGR
MOUSE
RAT
CAVPO

MED7

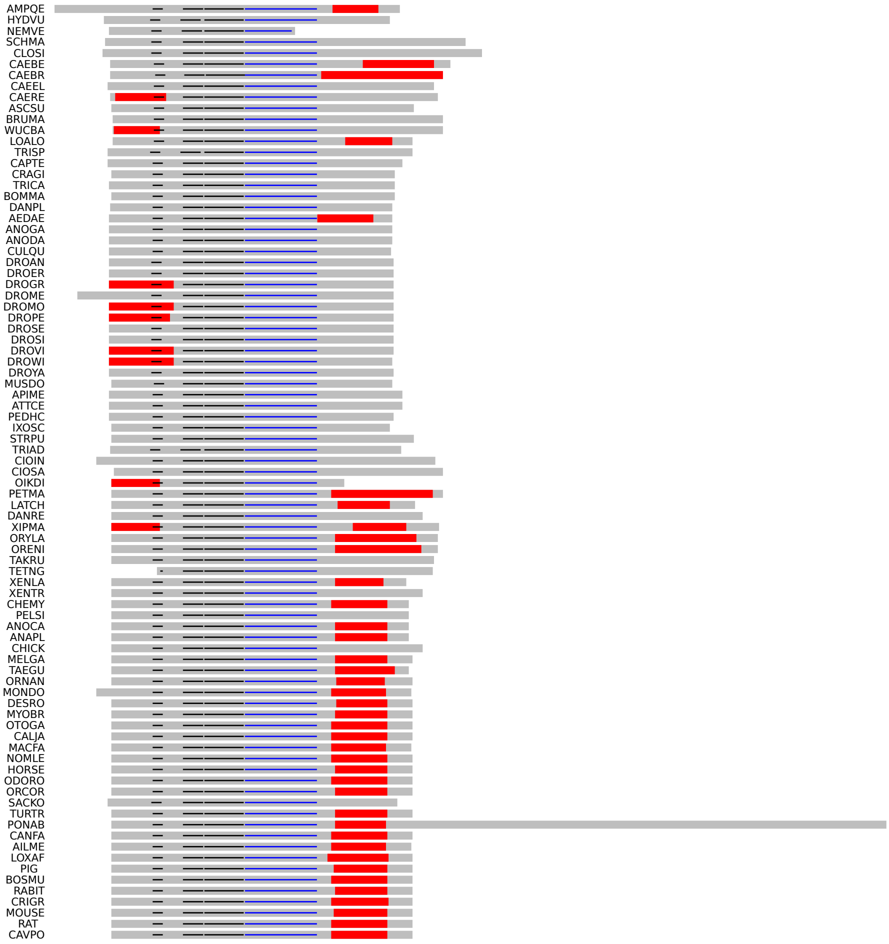
AMPQE
HYDVU
NEMVE
SCHMA
CLOSI
CAEBE
CAEBR
CAEEL
CAERE
ASCSU
BRUMA
WUCBA
LOALO
TRISP
CAPTE
CRAGI
TRICA
BOMMA
DANPL
AEDAE
ANOGA
ANODA
CULQU
DROAN
DROER
DROGR
DROME
DROMO
DROPE
DROSE
DROSI
DROVI
DROWI
DROYA
MUSDO
APIME
ATTCE
PEDHC
IXOSC
STRPU
TRIAD
CIOIN
CIOSA
OIKDI
PETMA
LATCH
DANRE
XIPMA
ORYLA
ORENI
TAKRU
TETNG
XENLA
XENTR
CHEMY
PELSI
ANOCA
ANAPL
CHICK
MELGA
TAEGU
ORNAN
MONDO
DESRO
MYOBR
OTOGA
CALJA
MACFA
NOMLE
HORSE
ODORO
ORCOR
SACKO
TURTR
PONAB
CANFA
AILME
LOXAF
PIG
BOSMU
RABIT
CRIGR
MOUSE
RAT
CAVPO

MED8

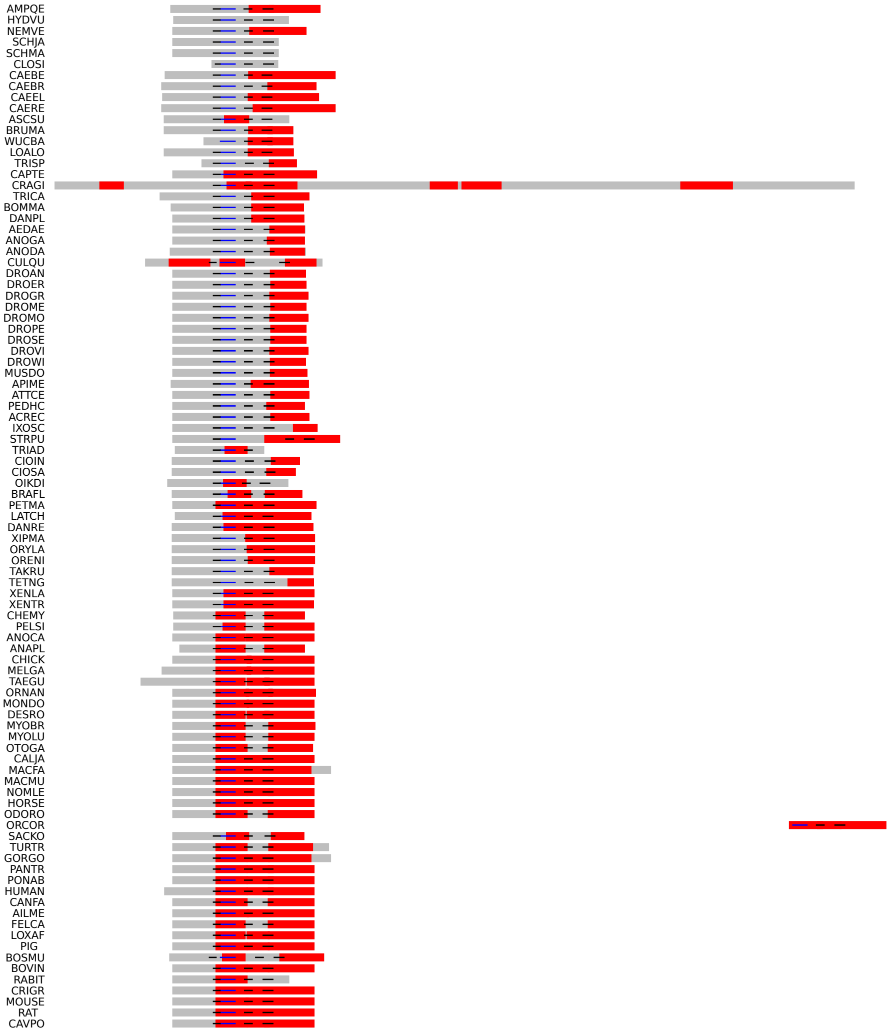
AMPQE
HYDVU
NEMVE
SCHJA
SCHMA
CLOSI
CAEBE
CAEBR
CAEEL
CAERE
ASCSU
BRUMA
WUCBA
LOALO
TRISP
CAPTE
CRAGI
TRICA
BOMMA
DANPL
AEDAE
ANOGA
ANODA
CULQU
DROAN
DROER
DROGR
DROME
DROMO
DROPE
DROSE
DROVI
DROWI
MUSDO
APIME
ATTCE
PEDHC
ACREC
IXOSC
STRPU
TRIAD
CIOIN
CIOSA
OIKDI
BRAFL
PETMA
LATCH
DANRE
XIPMA
ORYLA
ORENI
TAKRU
TETNG
XENLA
XENTR
CHEMY
PELSI
ANOCA
ANAPL
CHICK
MELGA
TAEGU
ORNAN
MONDO
DESRO
MYOBR
MYOLU
OTOGA
CALJA
MACFA
MACMU
NOMLE
HORSE
ODORO
ORCOR
SACKO
TURTR
GORGO
PANTR
PONAB
HUMAN
CANFA
AILME
FELCA
LOXAF
PIG
BOSMU
BOVIN
RABIT
CRIGR
MOUSE
RAT
CAVPO

MED9

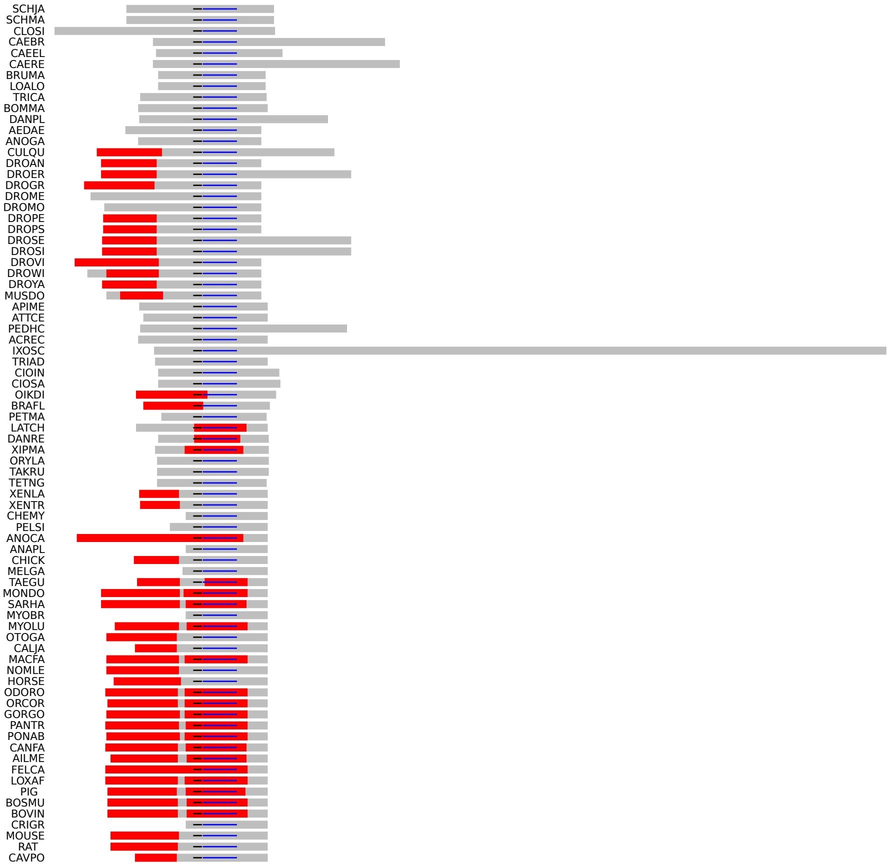
SCHJA
SCHMA
CLOSI
CAEBR
CAEEL
CAERE
BRUMA
LOALO
TRICA
BOMMA
DANPL
AEDAE
ANOGA
CULQU
DROAN
DROER
DROGR
DROME
DROMO
DROPE
DROPS
DROSE
DROSI
DROVI
DROWI
DROYA
MUSDO
APIME
ATTCE
PEDHC
ACREC
IXOSC
TRIAD
CIOIN
CIOSA
OIKDI
BRAFL
PETMA
LATCH
DANRE
XIPMA
ORYLA
TAKRU
TETNG
XENLA
XENTR
CHEMY
PELSI
ANOCA
ANAPL
CHICK
MELGA
TAEGU
MONDO
SARHA
MYOBR
MYOLU
OTOGA
CALJA
MACFA
NOMLE
HORSE
ODORO
ORCOR
GORGO
PANTR
PONAB
CANFA
AILME
FELCA
LOXAF
PIG
BOSMU
BOVIN
CRIGR
MOUSE
RAT
CAVPO

MED10

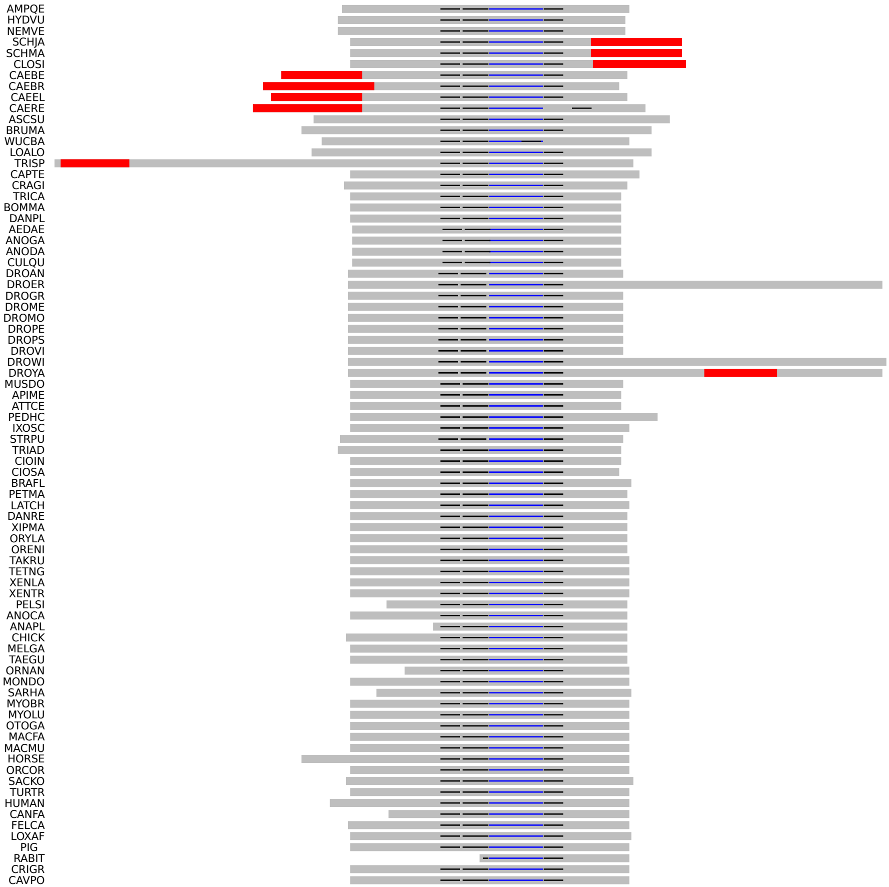
AMPQE
HYDVU
NEMVE
SCHJA
SCHMA
CLOSI
CAEBE
CAEBR
CAEEL
CAERE
ASCSU
BRUMA
WUCBA
LOALO
TRISP
CAPTE
CRAGI
TRICA
BOMMA
DANPL
AEDAE
ANOGA
ANODA
CULQU
DROAN
DROER
DROGR
DROME
DROMO
DROPE
DROPS
DROVI
DROWI
DROYA
MUSDO
APIME
ATTCE
PEDHC
IXOSC
STRPU
TRIAD
CIOIN
CIOSA
BRAFL
PETMA
LATCH
DANRE
XIPMA
ORYLA
ORENI
TAKRU
TETNG
XENLA
XENTR
PELSI
ANOCA
ANAPL
CHICK
MELGA
TAEGU
ORNAN
MONDO
SARHA
MYOBR
MYOLU
OTOGA
MACFA
MACMU
HORSE
ORCOR
SACKO
TURTR
HUMAN
CANFA
FELCA
LOXAF
PIG
RABIT
CRIGR
CAVPO
