# Supplementary material for: Evolution of disorder in Mediator complex and its functional relevance
Source: Nucleic Acids Res. 2015 Nov 20;44(4):1591–612. doi: 10.1093/nar/gkv1135 (PMC4770211; doi:10.1093/nar/gkv1135)
Supplement: SUPPLEMENTARY DATA [file supp_gkv1135_nar-01763-n-2015-File011.zip › SF_5.pdf]

This file contains a schematic of the Intrinsically disordered regions (IDRs) in the Mediator complex subunits (Med1, Med2, Med3, Med4, Med5, Med6, Med7, Med8, Med9, Med10, Med11, Med12, Med13, Med14, Med15, Med16, Med17, Med18, Med19, Med20, Med21, Med22, Med25, Med31, Cdk8 and CycC) of Fungi. List of organisms used in the current study are present in supplementary table ST1.

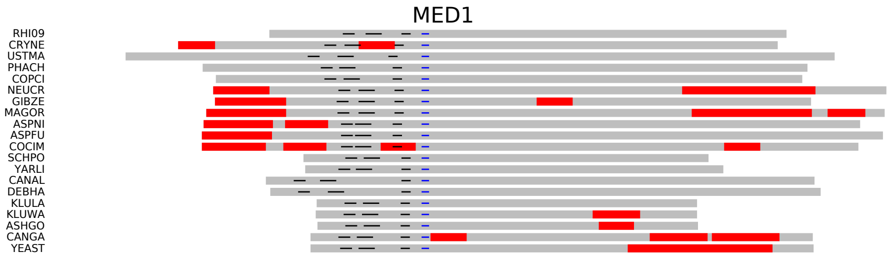
MED1
RHI09
CRYNE
USTMA
PHACH
COPCI
NEUCR
GIBZE
MAGOR
ASPNI
ASPFU
COCIM
SCHPO
YARLI
CANAL
DEBHA
KLULA
KLUWA
ASHGO
CANGA
YEAST

MED2

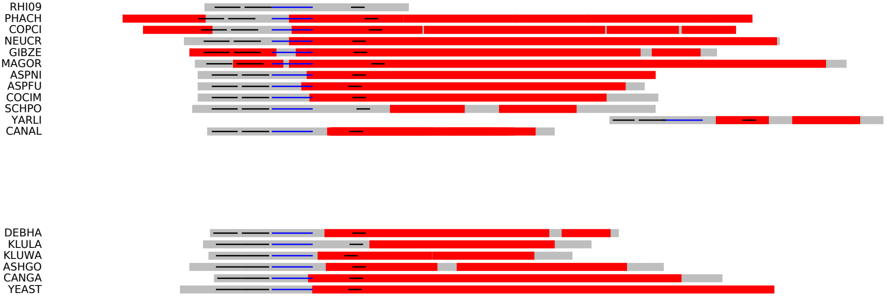
RHI09
PHACH
COPCI
NEUCR
GIBZE
MAGOR
ASPNI
ASPFU
COCIM
SCHPO
YARLI
CANAL
DEBHA
KLULA
KLUWA
ASHGO
CANGA
YEAST

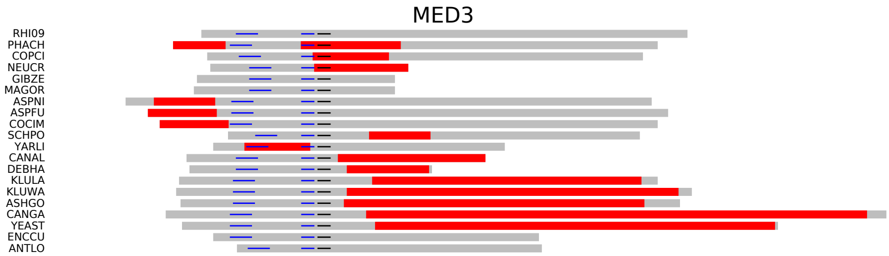
MED3
RHI09
PHACH
COPCI
NEUCR
GIBZE
MAGOR
ASPNI
ASPFU
COCIM
SCHPO
YARLI
CANAL
DEBHA
KLULA
KLUWA
ASHGO
CANGA
YEAST
ENCCU
ANTLO

MED4

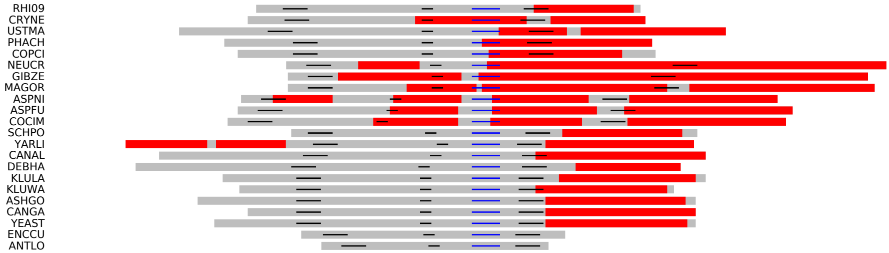
RHI09
CRYNE
USTMA
PHACH
COPCI
NEUCR
GIBZE
MAGOR
ASPNI
ASPFU
COCIM
SCHPO
YARLI
CANAL
DEBHA
KLULA
KLUWA
ASHGO
CANGA
YEAST
ENCCU
ANTLO

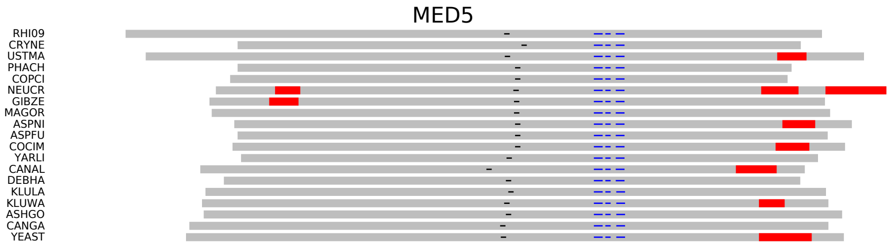

MED5
RHI09
CRYNE
USTMA
PHACH
COPCI
NEUCR
GIBZE
MAGOR
ASPNI
ASPFU
COCIM
YARLI
CANAL
DEBHA
KLULA
KLUWA
ASHGO
CANGA
YEAST

MED6

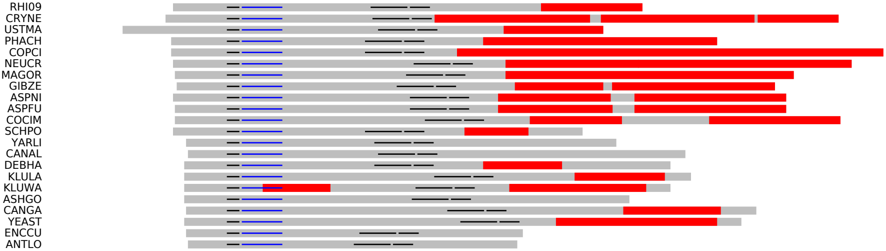
RHI09
CRYNE
USTMA
PHACH
COPCI
NEUCR
MAGOR
GIBZE
ASPNI
ASPFU
COCIM
SCHPO
YARLI
CANAL
DEBHA
KLULA
KLUWA
ASHGO
CANGA
YEAST
ENCCU
ANTLO

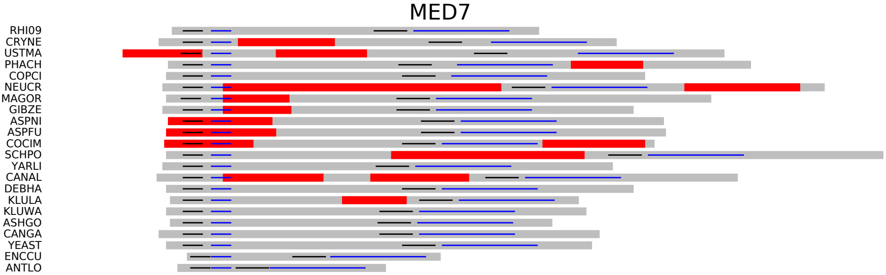
MED7
RHI09
CRYNE
USTMA
PHACH
COPCI
NEUCR
MAGOR
GIBZE
ASPNI
ASPFU
COCIM
SCHPO
YARLI
CANAL
DEBHA
KLULA
KLUWA
ASHGO
CANGA
YEAST
ENCCU
ANTLO

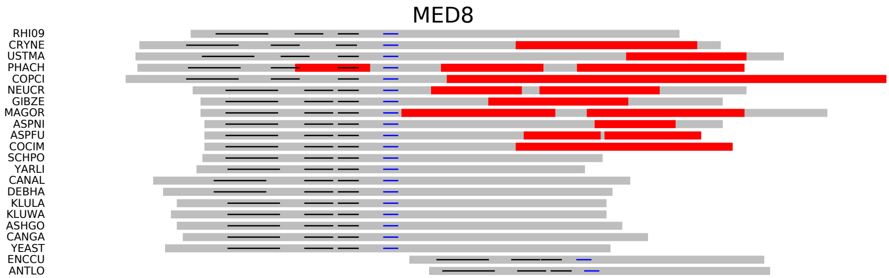
MED8
RHI09
CRYNE
USTMA
PHACH
COPCI
NEUCR
GIBZE
MAGOR
ASPNI
ASPFU
COCIM
SCHPO
YARLI
CANAL
DEBHA
KLULA
KLUWA
ASHGO
CANGA
YEAST
ENCCU
ANTLO

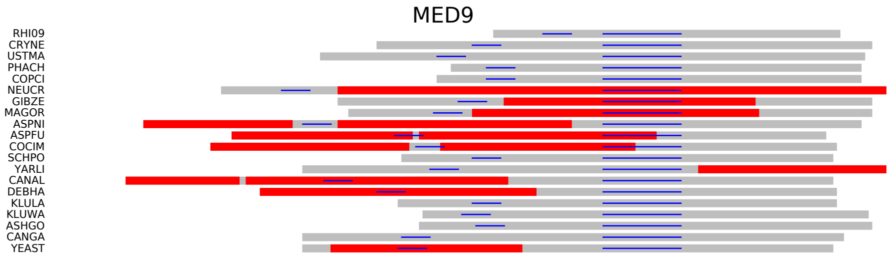
MED9
RHI09
CRYNE
USTMA
PHACH
COPCI
NEUCR
GIBZE
MAGOR
ASPNI
ASPFU
COCIM
SCHPO
YARLI
CANAL
DEBHA
KLULA
KLUWA
ASHGO
CANGA
YEAST

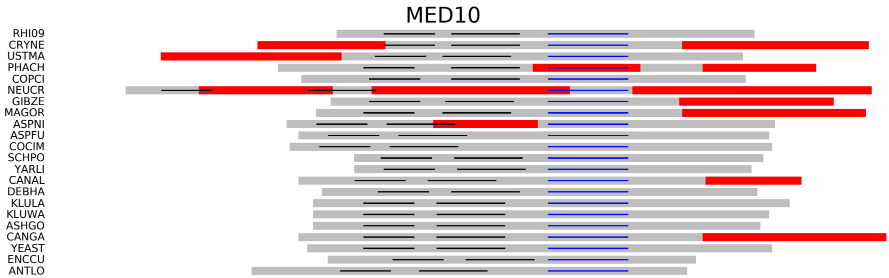
MED10
RHI09
CRYNE
USTMA
PHACH
COPCI
NEUCR
GIBZE
MAGOR
ASPNI
ASPFU
COCIM
SCHPO
YARLI
CANAL
DEBHA
KLULA
KLUWA
ASHGO
CANGA
YEAST
ENCCU
ANTLO

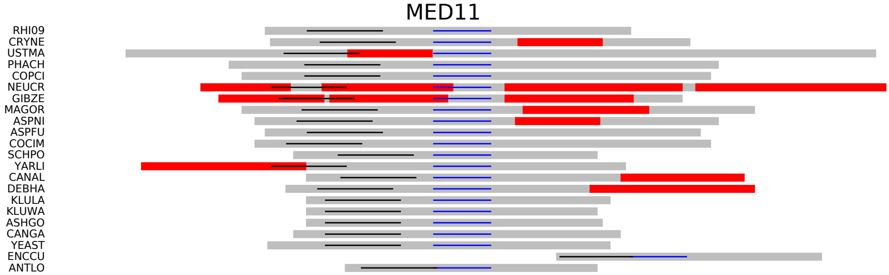
MED11
RHI09
CRYNE
USTMA
PHACH
COPCI
NEUCR
GIBZE
MAGOR
ASPNI
ASPFU
COCIM
SCHPO
YARLI
CANAL
DEBHA
KLULA
KLUWA
ASHGO
CANGA
YEAST
ENCCU
ANTLO

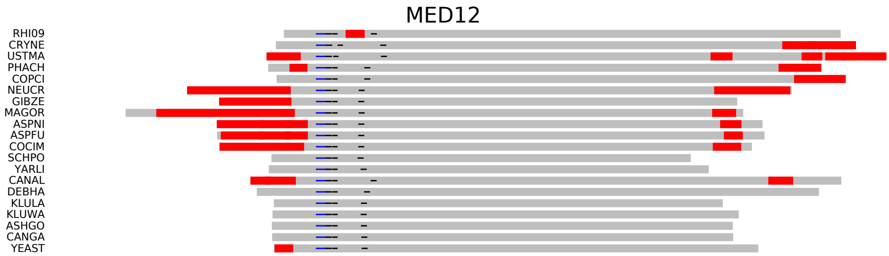
MED12
RHI09
CRYNE
USTMA
PHACH
COPCI
NEUCR
GIBZE
MAGOR
ASPNI
ASPFU
COCIM
SCHPO
YARLI
CANAL
DEBHA
KLULA
KLUWA
ASHGO
CANGA
YEAST

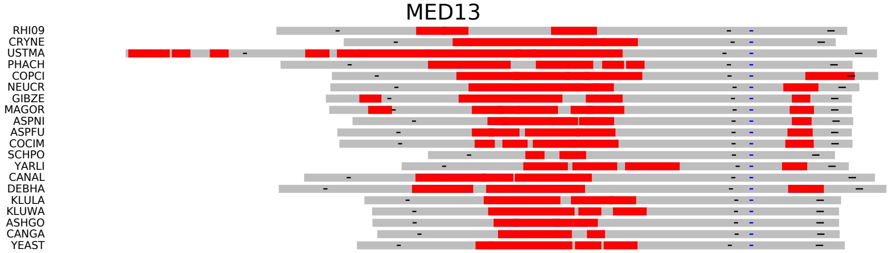
MED13
RHI09
CRYNE
USTMA
PHACH
COPCI
NEUCR
GIBZE
MAGOR
ASPNI
ASPFU
COCIM
SCHPO
YARLI
CANAL
DEBHA
KLULA
KLUWA
ASHGO
CANGA
YEAST

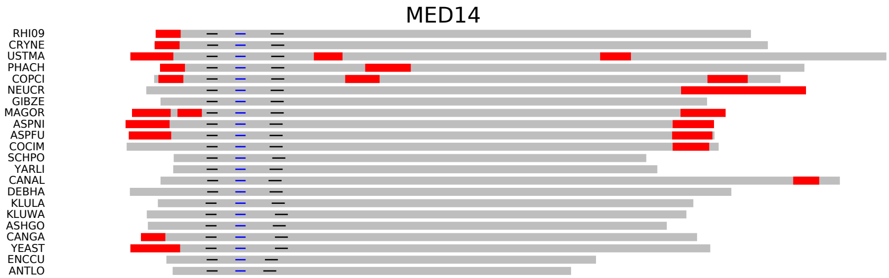
MED14
RHI09
CRYNE
USTMA
PHACH
COPCI
NEUCR
GIBZE
MAGOR
ASPNI
ASPFU
COCIM
SCHPO
YARLI
CANAL
DEBHA
KLULA
KLUWA
ASHGO
CANGA
YEAST
ENCCU
ANTLO

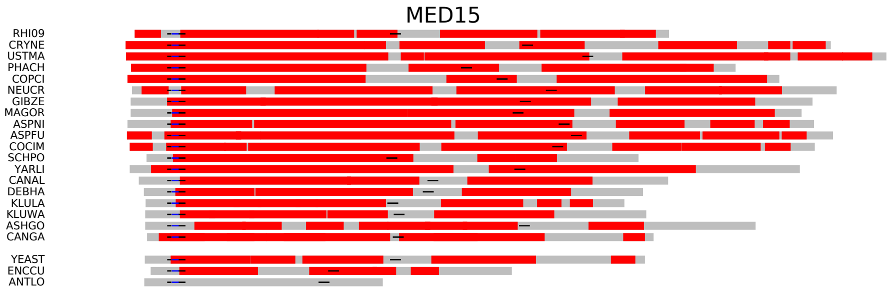
MED15
RHI09
CRYNE
USTMA
PHACH
COPCI
NEUCR
GIBZE
MAGOR
ASPNI
ASPFU
COCIM
SCHPO
YARLI
CANAL
DEBHA
KLULA
KLUWA
ASHGO
CANGA
YEAST
ENCCU
ANTLO

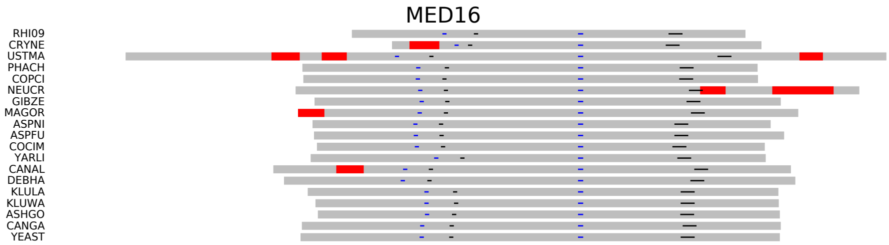
MED16
RHI09
CRYNE
USTMA
PHACH
COPCI
NEUCR
GIBZE
MAGOR
ASPNI
ASPFU
COCIM
YARLI
CANAL
DEBHA
KLULA
KLUWA
ASHGO
CANGA
YEAST

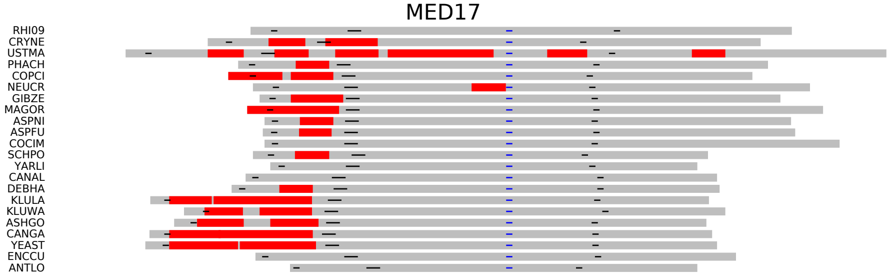
MED17
RHI09
CRYNE
USTMA
PHACH
COPCI
NEUCR
GIBZE
MAGOR
ASPNI
ASPFU
COCIM
SCHPO
YARLI
CANAL
DEBHA
KLULA
KLUWA
ASHGO
CANGA
YEAST
ENCCU
ANTLO

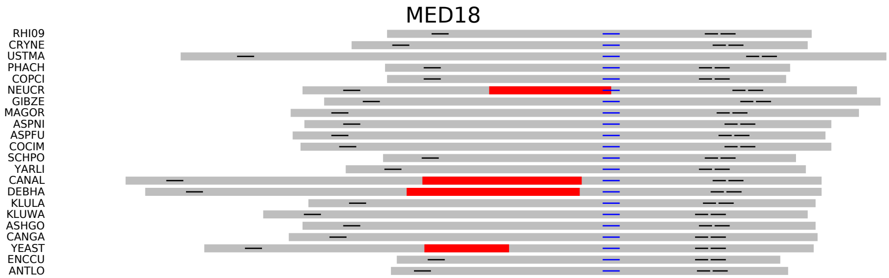
MED18
RHI09
CRYNE
USTMA
PHACH
COPCI
NEUCR
GIBZE
MAGOR
ASPNI
ASPFU
COCIM
SCHPO
YARLI
CANAL
DEBHA
KLULA
KLUWA
ASHGO
CANGA
YEAST
ENCCU
ANTLO

MED19

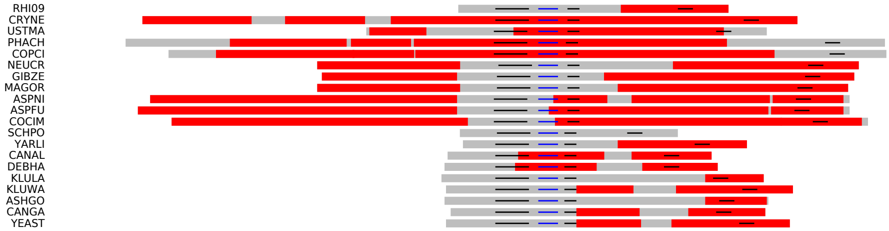
RHI09
CRYNE
USTMA
PHACH
COPCI
NEUCR
GIBZE
MAGOR
ASPNI
ASPFU
COCIM
SCHPO
YARLI
CANAL
DEBHA
KLULA
KLUWA
ASHGO
CANGA
YEAST

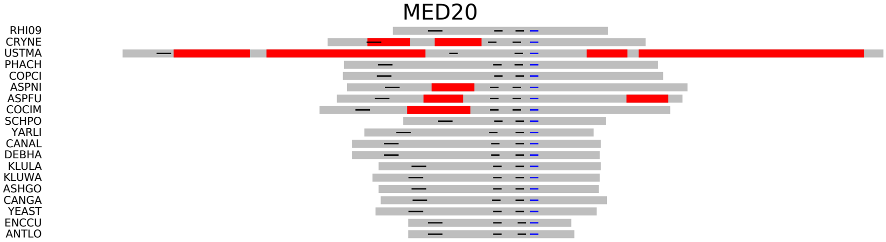
MED20
RHI09
CRYNE
USTMA
PHACH
COPCI
ASPNI
ASPFU
COCIM
SCHPO
YARLI
CANAL
DEBHA
KLULA
KLUWA
ASHGO
CANGA
YEAST
ENCCU
ANTLO

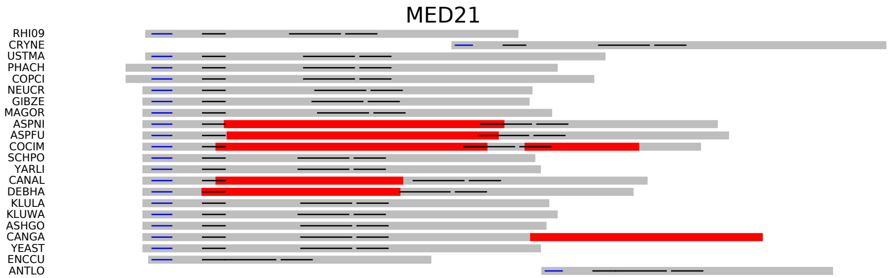

MED21
RHI09
CRYNE
USTMA
PHACH
COPCI
NEUCR
GIBZE
MAGOR
ASPNI
ASPFU
COCIM
SCHPO
YARLI
CANAL
DEBHA
KLULA
KLUWA
ASHGO
CANGA
YEAST
ENCCU
ANTLO

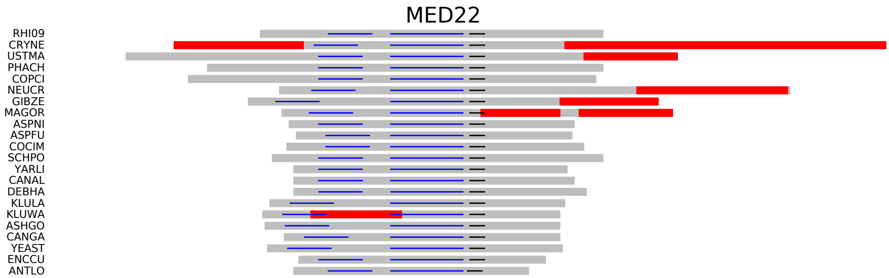
MED22
RHI09
CRYNE
USTMA
PHACH
COPCI
NEUCR
GIBZE
MAGOR
ASPNI
ASPFU
COCIM
SCHPO
YARLI
CANAL
DEBHA
KLULA
KLUWA
ASHGO
CANGA
YEAST
ENCCU
ANTLO

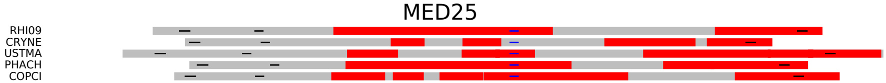

MED25
RHI09
CRYNE
USTMA
PHACH
COPCI

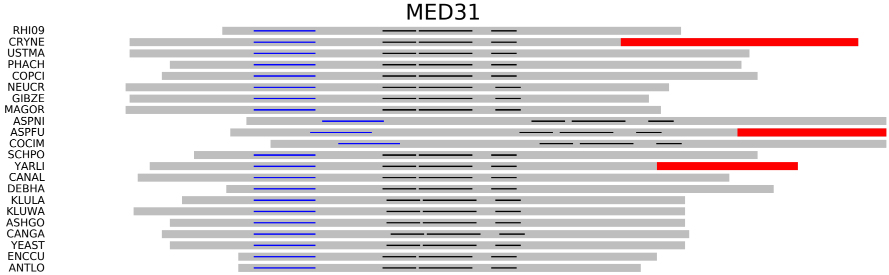
MED31
RHI09
CRYNE
USTMA
PHACH
COPCI
NEUCR
GIBZE
MAGOR
ASPNI
ASPFU
COCIM
SCHPO
YARLI
CANAL
DEBHA
KLULA
KLUWA
ASHGO
CANGA
YEAST
ENCCU
ANTLO

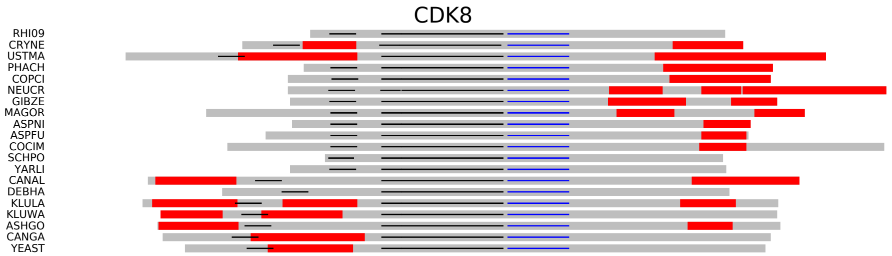
CDK8
RHI09
CRYNE
USTMA
PHACH
COPCI
NEUCR
GIBZE
MAGOR
ASPNI
ASPFU
COCIM
SCHPO
YARLI
CANAL
DEBHA
KLULA
KLUWA
ASHGO
CANGA
YEAST

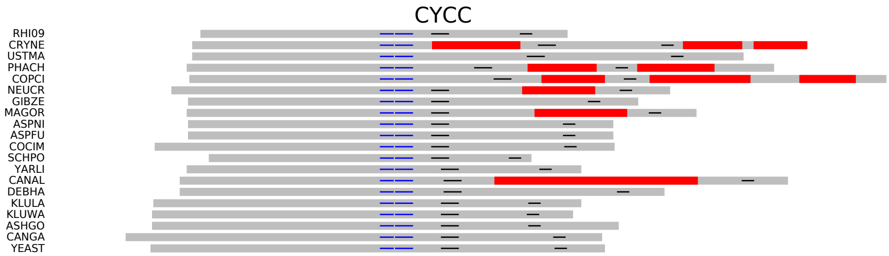
CYCC
RHI09
CRYNE
USTMA
PHACH
COPCI
NEUCR
GIBZE
MAGOR
ASPNI
ASPFU
COCIM
SCHPO
YARLI
CANAL
DEBHA
KLULA
KLUWA
ASHGO
CANGA
YEAST
