# Supplementary material for: Evolution of disorder in Mediator complex and its functional relevance
Source: Nucleic Acids Res. 2015 Nov 20;44(4):1591–612. doi: 10.1093/nar/gkv1135 (PMC4770211; doi:10.1093/nar/gkv1135)
Supplement: SUPPLEMENTARY DATA [file supp_gkv1135_nar-01763-n-2015-File011.zip › SF_2.pdf]

This file contains a schematic of the Intrinsically disordered regions (IDRs) in the Mediator complex subunits (Med11, Med12, Med13, Med14, Med15, Med16, Med17) of Metazoans. List of organisms used in the current study are present in supplementary table ST1

MED11

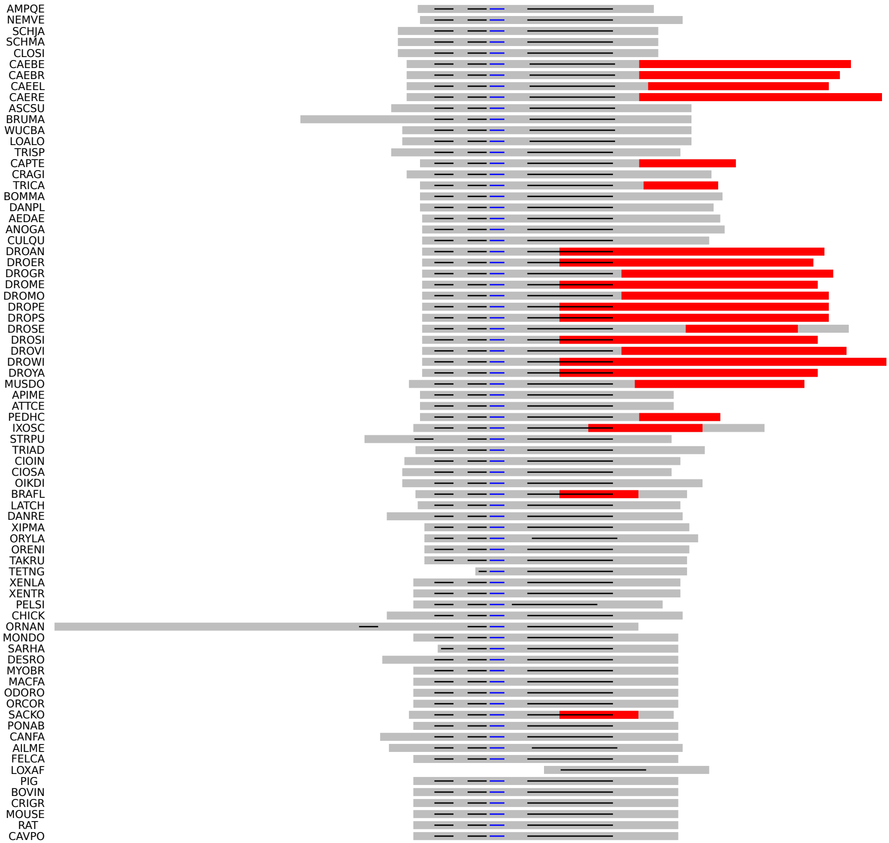
AMPQE
NEMVE
SCHJA
SCHMA
CLOSI
CAEBE
CAEBR
CAEEL
CAERE
ASCSU
BRUMA
WUCBA
LOALO
TRISP
CAPTE
CRAGI
TRICA
BOMMA
DANPL
AEDAE
ANOGA
CULQU
DROAN
DROER
DROGR
DROME
DROMO
DROPE
DROPS
DROSE
DROSI
DROVI
DROWI
DROYA
MUSDO
APIME
ATTCE
PEDHC
IXOSC
STRPU
TRIAD
CIOIN
CIOSA
OIKDI
BRAFL
LATCH
DANRE
XIPMA
ORYLA
ORENI
TAKRU
TETNG
XENLA
XENTR
PELSI
CHICK
ORNAN
MONDO
SARHA
DESRO
MYOBR
MACFA
ODORO
ORCOR
SACKO
PONAB
CANFA
AILME
FELCA
LOXAF
PIG
BOVIN
CRIGR
MOUSE
RAT
CAVPO

MED12

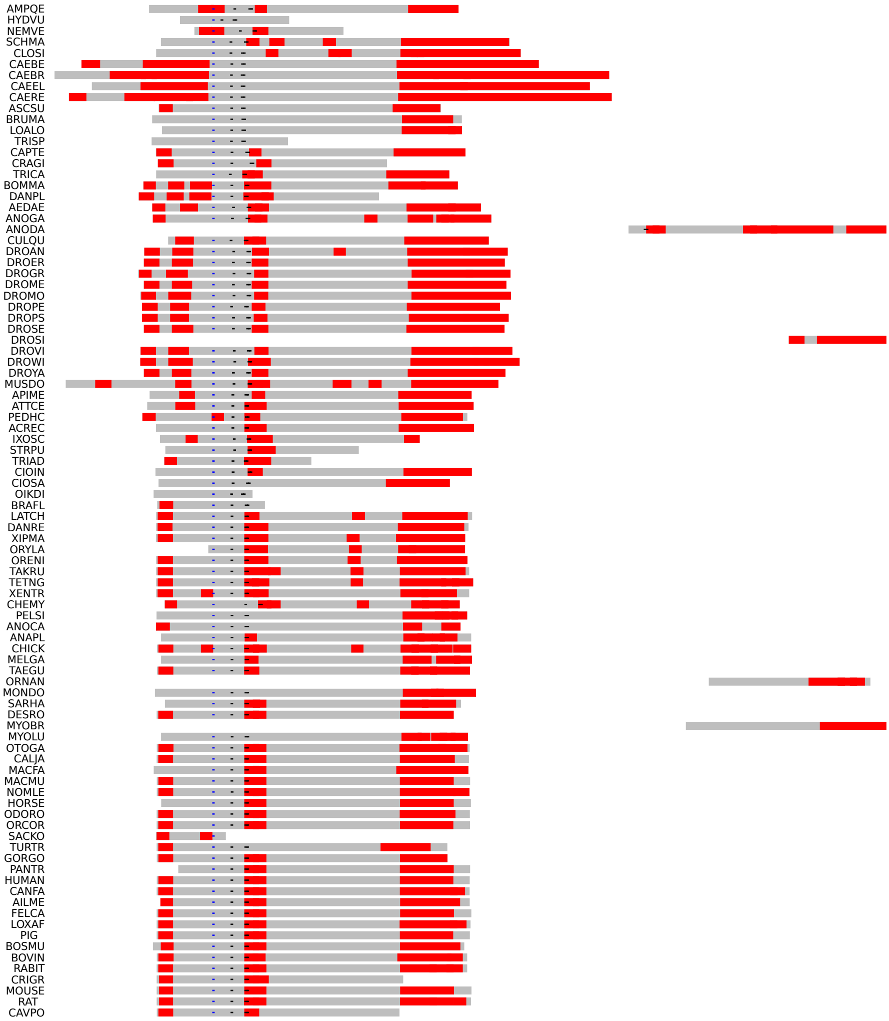
AMPQE
HYDVU
NEMVE
SCHMA
CLOSI
CAEBE
CAEBR
CAEEL
CAERE
ASCSU
BRUMA
LOALO
TRISP
CAPTE
CRAGI
TRICA
BOMMA
DANPL
AEDAE
ANOGA
ANODA
CULQU
DROAN
DROER
DROGR
DROME
DROMO
DROPE
DROPS
DROSE
DROSI
DROVI
DROWI
DROYA
MUSDO
APIME
ATTCE
PEDHC
ACREC
IXOSC
STRPU
TRIAD
CIOIN
CIOSA
OIKDI
BRAFL
LATCH
DANRE
XIPMA
ORYLA
ORENI
TAKRU
TETNG
XENTR
CHEMY
PELSI
ANOCA
ANAPL
CHICK
MELGA
TAEGU
ORNAN
MONDO
SARHA
DESRO
MYOBR
MYOLU
OTOGA
CALJA
MACFA
MACMU
NOMLE
HORSE
ODORO
ORCOR
SACKO
TURTR
GORGO
PANTR
HUMAN
CANFA
AILME
FELCA
LOXAF
PIG
BOSMU
BOVIN
RABIT
CRIGR
MOUSE
RAT
CAVPO

MED13

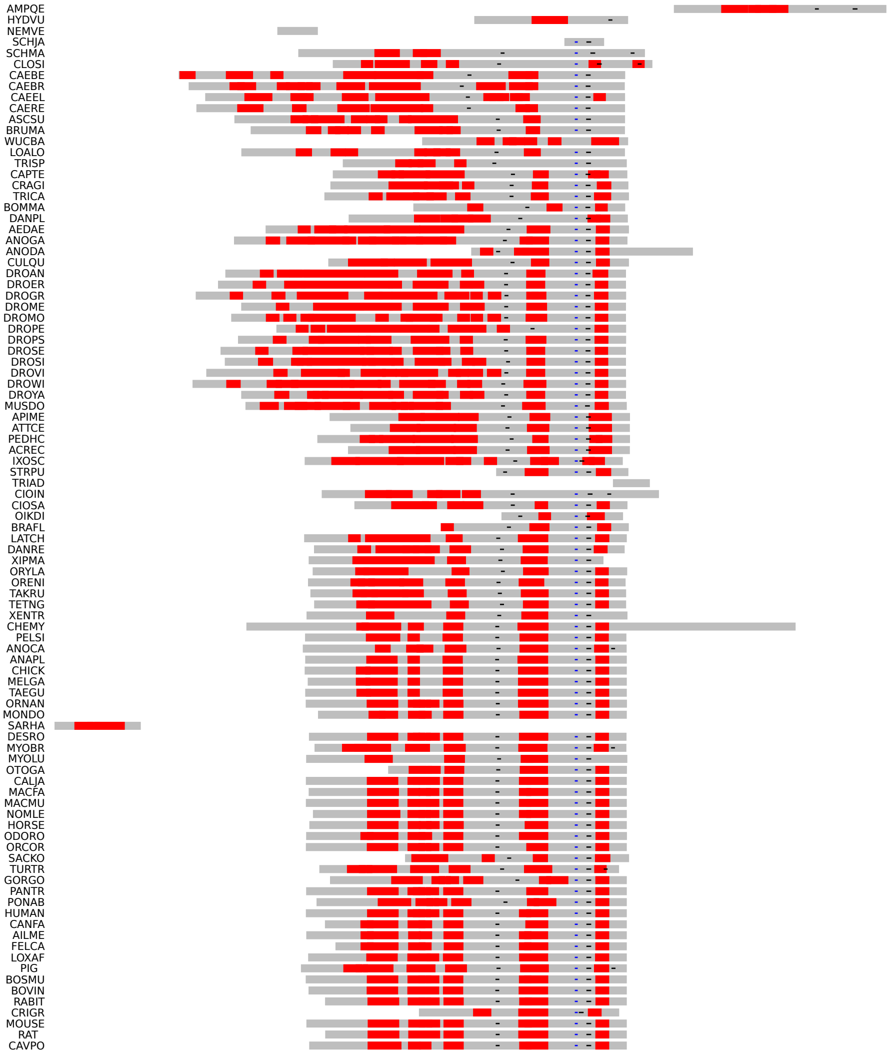
AMPQE
HYDVU
NEMVE
SCHJA
SCHMA
CLOSI
CAEBE
CAEBR
CAEEL
CAERE
ASCSU
BRUMA
WUCBA
LOALO
TRISP
CAPTE
CRAGI
TRICA
BOMMA
DANPL
AEDAE
ANOGA
ANODA
CULQU
DROAN
DROER
DROGR
DROME
DROMO
DROPE
DROPS
DROSE
DROSI
DROVI
DROWI
DROYA
MUSDO
APIME
ATTCE
PEDHC
ACREC
IXOSC
STRPU
TRIAD
CIOIN
CIOSA
OIKDI
BRAFL
LATCH
DANRE
XIPMA
ORYLA
ORENI
TAKRU
TETNG
XENTR
CHEMY
PELSI
ANOCA
ANAPL
CHICK
MELGA
TAEGU
ORNAN
MONDO
SARHA
DESRO
MYOBR
MYOLU
OTOGA
CALJA
MACFA
MACMU
NOMLE
HORSE
ODORO
ORCOR
SACKO
TURTR
GORGO
PANTR
PONAB
HUMAN
CANFA
AILME
FELCA
LOXAF
PIG
BOSMU
BOVIN
RABIT
CRIGR
MOUSE
RAT
CAVPO

MED14

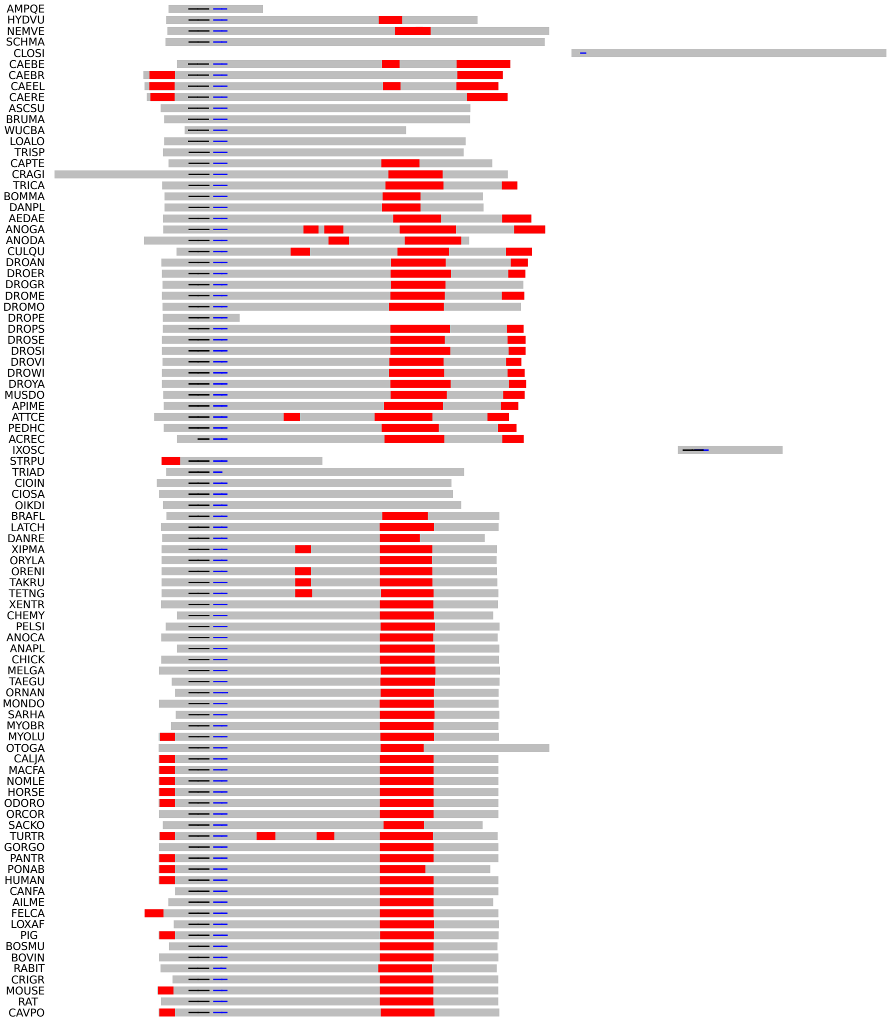
AMPQE
HYDVU
NEMVE
SCHMA
CLOSI
CAEBE
CAEBR
CAEEL
CAERE
ASCSU
BRUMA
WUCBA
LOALO
TRISP
CAPTE
CRAGI
TRICA
BOMMA
DANPL
AEDAE
ANOGA
ANODA
CULQU
DROAN
DROER
DROGR
DROME
DROMO
DROPE
DROPS
DROSE
DROSI
DROVI
DROWI
DROYA
MUSDO
APIME
ATTCE
PEDHC
ACREC
IXOSC
STRPU
TRIAD
CIOIN
CIOSA
OIKDI
BRAFL
LATCH
DANRE
XIPMA
ORYLA
ORENI
TAKRU
TETNG
XENTR
CHEMY
PELSI
ANOCA
ANAPL
CHICK
MELGA
TAEGU
ORNAN
MONDO
SARHA
MYOBR
MYOLU
OTOGA
CALJA
MACFA
NOMLE
HORSE
ODORO
ORCOR
SACKO
TURTR
GORGO
PANTR
PONAB
HUMAN
CANFA
AILME
FELCA
LOXAF
PIG
BOSMU
BOVIN
RABIT
CRIGR
MOUSE
RAT
CAVPO

MED15

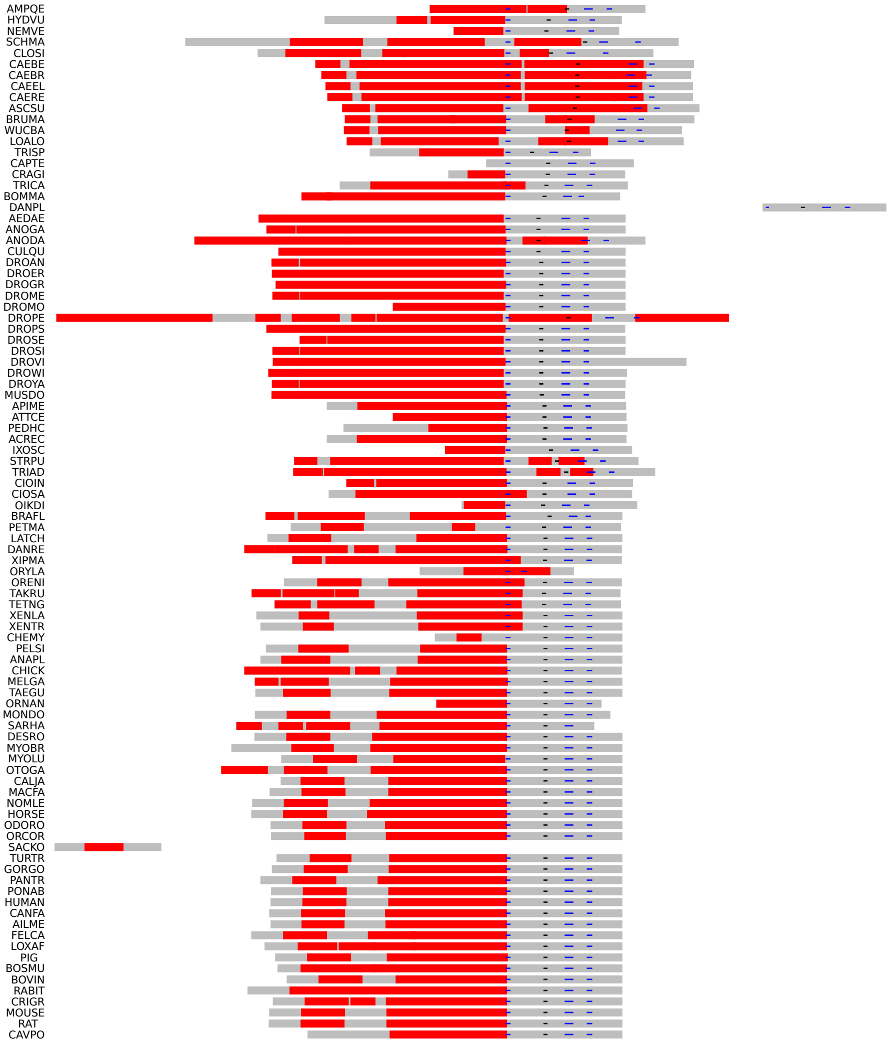
AMPQE
HYDVU
NEMVE
SCHMA
CLOSI
CAEBE
CAEBR
CAEEL
CAERE
ASCSU
BRUMA
WUCBA
LOALO
TRISP
CAPTE
CRAGI
TRICA
BOMMA
DANPL
AEDAE
ANOGA
ANODA
CULQU
DROAN
DROER
DROGR
DROME
DROMO
DROPE
DROPS
DROSE
DROSI
DROVI
DROWI
DROYA
MUSDO
APIME
ATTCE
PEDHC
ACREC
IXOSC
STRPU
TRIAD
CIOIN
CIOSA
OIKDI
BRAFL
PETMA
LATCH
DANRE
XIPMA
ORYLA
ORENI
TAKRU
TETNG
XENLA
XENTR
CHEMY
PELSI
ANAPL
CHICK
MELGA
TAEGU
ORNAN
MONDO
SARHA
DESRO
MYOBR
MYOLU
OTOGA
CALJA
MACFA
NOMLE
HORSE
ODORO
ORCOR
SACKO
TURTR
GORGO
PANTR
PONAB
HUMAN
CANFA
AILME
FELCA
LOXAF
PIG
BOSMU
BOVIN
RABIT
CRIGR
MOUSE
RAT
CAVPO

MED16

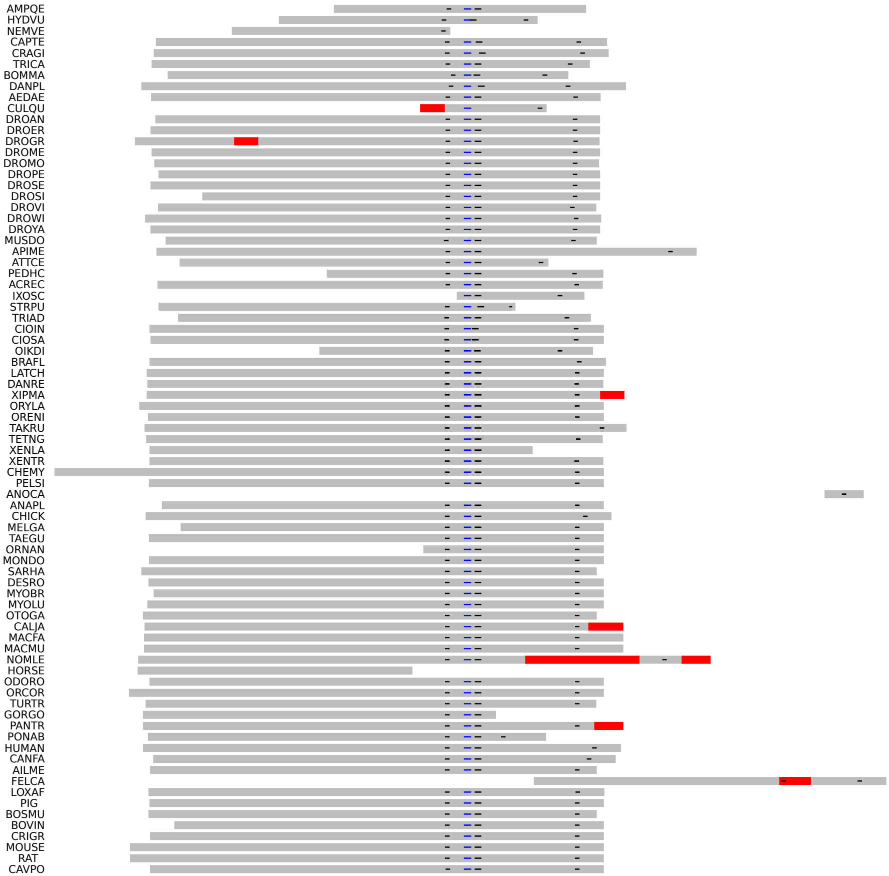
AMPQE
HYDVU
NEMVE
CAPTE
CRAGI
TRICA
BOMMA
DANPL
AEDAE
CULQU
DROAN
DROER
DROGR
DROME
DROMO
DROPE
DROSE
DROSI
DROVI
DROWI
DROYA
MUSDO
APIME
ATTCE
PEDHC
ACREC
IXOSC
STRPU
TRIAD
CIOIN
CIOSA
OIKDI
BRAFL
LATCH
DANRE
XIPMA
ORYLA
ORENI
TAKRU
TETNG
XENLA
XENTR
CHEMY
PELSI
ANOCA
ANAPL
CHICK
MELGA
TAEGU
ORNAN
MONDO
SARHA
DESRO
MYOBR
MYOLU
OTOGA
CALJA
MACFA
MACMU
NOMLE
HORSE
ODORO
ORCOR
TURTR
GORGO
PANTR
PONAB
HUMAN
CANFA
AILME
FELCA
LOXAF
PIG
BOSMU
BOVIN
CRIGR
MOUSE
RAT
CAVPO

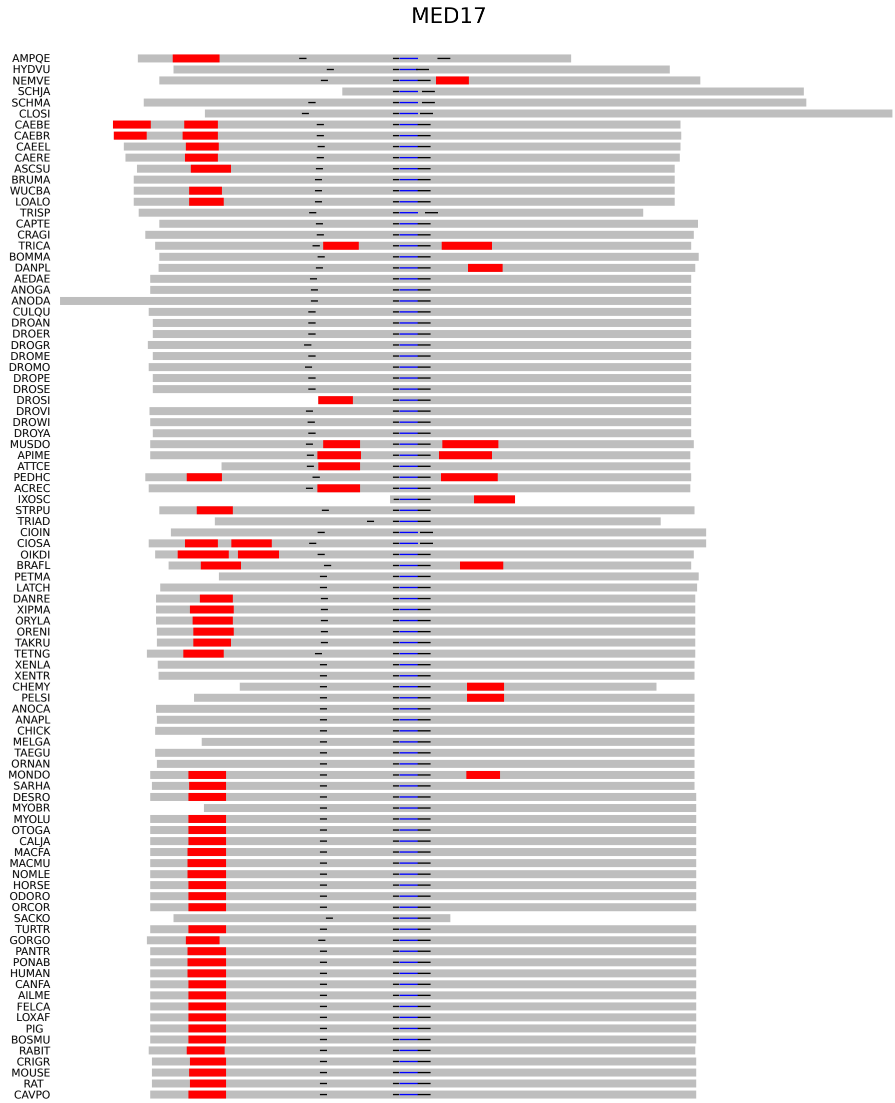
MED17
AMPQE
HYDVU
NEMVE
SCHJA
SCHMA
CLOSI
CAEBE
CAEBR
CAEEL
CAERE
ASCSU
BRUMA
WUCBA
LOALO
TRISP
CAPTE
CRAGI
TRICA
BOMMA
DANPL
AEDAE
ANOGA
ANODA
CULQU
DROAN
DROER
DROGR
DROME
DROMO
DROPE
DROSE
DROSI
DROVI
DROWI
DROYA
MUSDO
APIME
ATTCE
PEDHC
ACREC
IXOSC
STRPU
TRIAD
CIOIN
CIOSA
OIKDI
BRAFL
PETMA
LATCH
DANRE
XIPMA
ORYLA
ORENI
TAKRU
TETNG
XENLA
XENTR
CHEMY
PELSI
ANOCA
ANAPL
CHICK
MELGA
TAEGU
ORNAN
MONDO
SARHA
DESRO
MYOBR
MYOLU
OTOGA
CALJA
MACFA
MACMU
NOMLE
HORSE
ODORO
ORCOR
SACKO
TURTR
GORGO
PANTR
PONAB
HUMAN
CANFA
AILME
FELCA
LOXAF
PIG
BOSMU
RABIT
CRIGR
MOUSE
RAT
CAVPO
