# Supplementary material for: Evolution of disorder in Mediator complex and its functional relevance
Source: Nucleic Acids Res. 2015 Nov 20;44(4):1591–612. doi: 10.1093/nar/gkv1135 (PMC4770211; doi:10.1093/nar/gkv1135)
Supplement: SUPPLEMENTARY DATA [file supp_gkv1135_nar-01763-n-2015-File011.zip › SF_10.pdf]

Structural quality of the homology models was assessed using PROCHECK. Ramachandran plots and other related statistics were generated for

A. *Arabidopsis* mediator subunits

1. Med7
2. Med21
3. Med31

B. Double mutations in human Med15 KIX domain

1. I64A, D68A
2. I64A, D68S
3. I64G, D68G
4. I64M, D68K
5. I64P, D68P
6. I64Y, D68K

PROCHECK

Ramachandran Plot
AtMed21

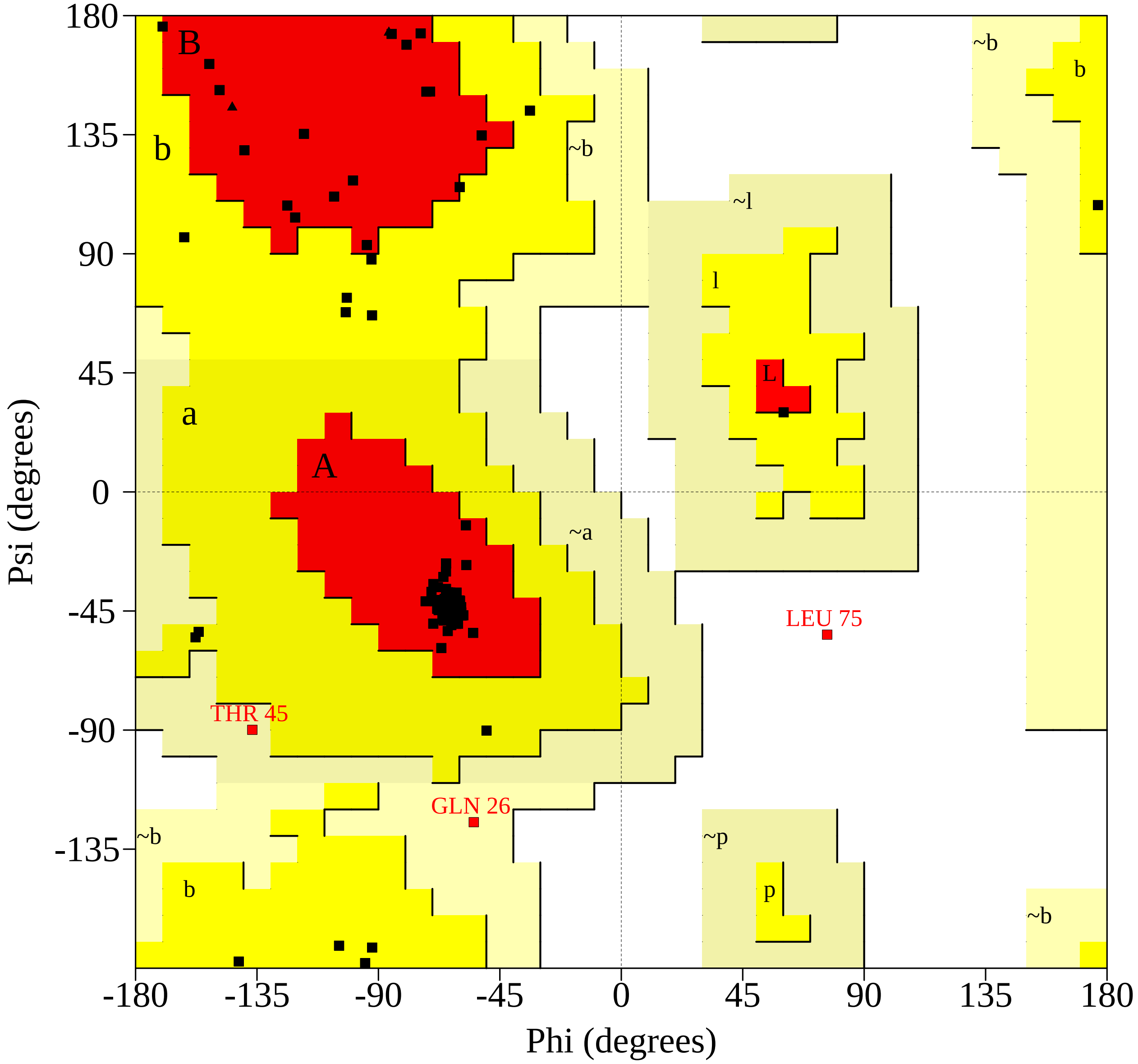

Plot statistics

Residues in most favoured regions [A,B,L]	104	87.4%
Residues in additional allowed regions [a,b,l,p]	12	10.1%
Residues in generously allowed regions [~a,~b,~l,~p]	2	1.7%
Residues in disallowed regions	1	0.8%
	----	------
Number of non-glycine and non-proline residues	119	100.0%
Number of end-residues (excl. Gly and Pro)	2	
Number of glycine residues (shown as triangles)	7	
Number of proline residues	11	

Total number of residues	139	

Based on an analysis of 118 structures of resolution of at least 2.0 Angstroms and R-factor no greater than 20%, a good quality model would be expected to have over 90% in the most favoured regions.

AtMed21_01.ps

PROCHECK

Ramachandran Plot

AtMed31

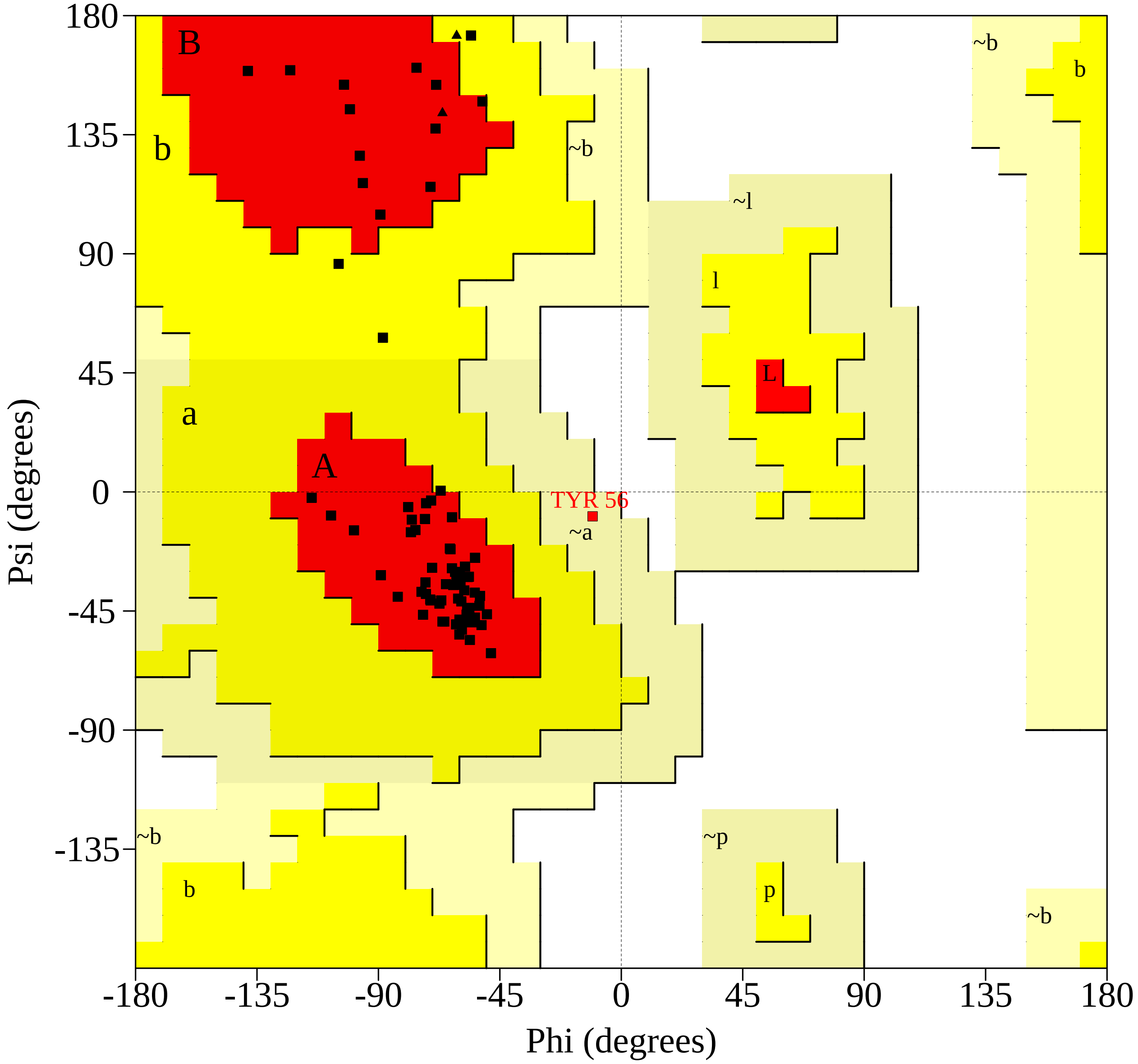

Plot statistics

Residues in most favoured regions [A,B,L]	67	93.1%
Residues in additional allowed regions [a,b,l,p]	4	5.6%
Residues in generously allowed regions [~a,~b,~l,~p]	1	1.4%
Residues in disallowed regions	0	0.0%
	----	------
Number of non-glycine and non-proline residues	72	100.0%
Number of end-residues (excl. Gly and Pro)	0	
Number of glycine residues (shown as triangles)	3	
Number of proline residues	6	

Total number of residues	81	

Based on an analysis of 118 structures of resolution of at least 2.0 Angstroms and R-factor no greater than 20%, a good quality model would be expected to have over 90% in the most favoured regions.

AtMed31_01.ps

PROCHECK

Ramachandran Plot

AtMed7

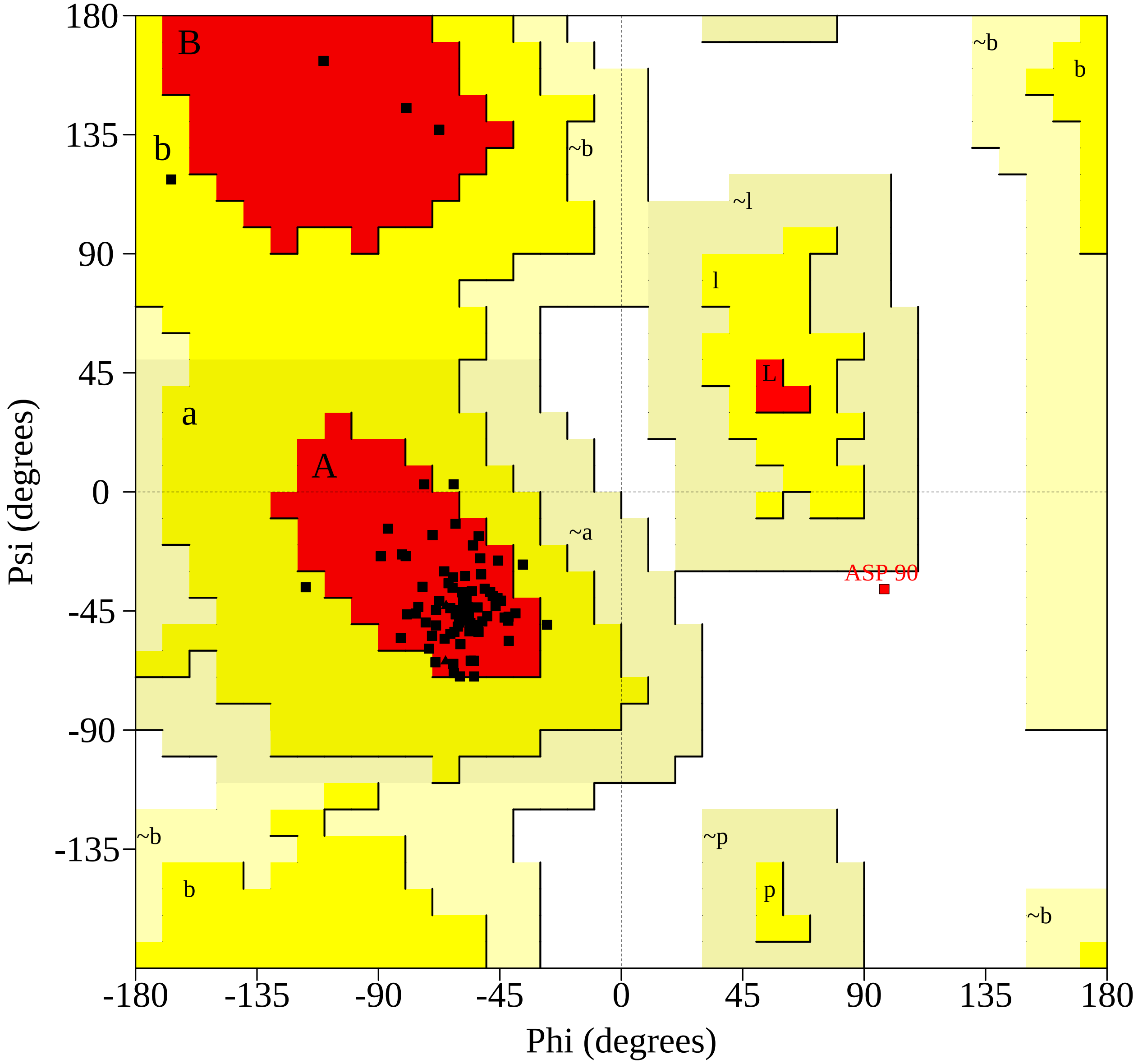

Plot statistics

Residues in most favoured regions [A,B,L]	79	94.0%
Residues in additional allowed regions [a,b,l,p]	4	4.8%
Residues in generously allowed regions [~a,~b,~l,~p]	0	0.0%
Residues in disallowed regions	1	1.2%
	----	------
Number of non-glycine and non-proline residues	84	100.0%
Number of end-residues (excl. Gly and Pro)	2	
Number of glycine residues (shown as triangles)	2	
Number of proline residues	2	

Total number of residues	90	

Based on an analysis of 118 structures of resolution of at least 2.0 Angstroms
and R-factor no greater than 20%, a good quality model would be expected
to have over 90% in the most favoured regions.

PROCHECK

Ramachandran Plot

HUMAN Med15 kix model with double mutations I64A, D68A

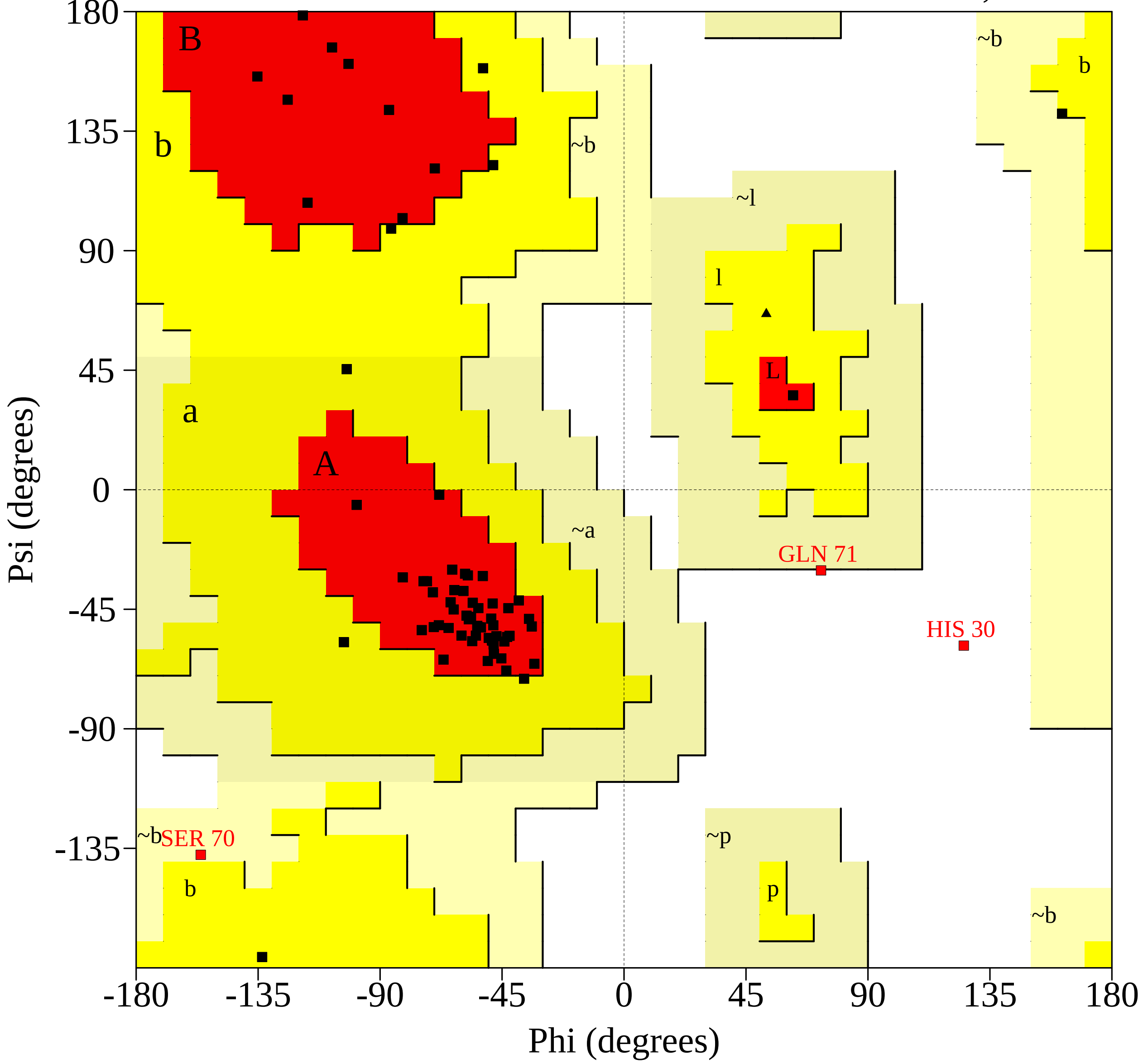

Plot statistics

Residues in most favoured regions [A,B,L]	60	84.5%
Residues in additional allowed regions [a,b,l,p]	8	11.3%
Residues in generously allowed regions [~a,~b,~l,~p]	1	1.4%
Residues in disallowed regions	2	2.8%
	----	------
Number of non-glycine and non-proline residues	71	100.0%
Number of end-residues (excl. Gly and Pro)	1	
Number of glycine residues (shown as triangles)	2	
Number of proline residues	0	

Total number of residues	74	

Based on an analysis of 118 structures of resolution of at least 2.0 Angstroms and R-factor no greater than 20%, a good quality model would be expected to have over 90% in the most favoured regions.

HUMAN_kix_I64A_D68A_cor_01.ps

PROCHECK

Ramachandran Plot

HUMAN Med15 kix with double mutations I64A, D68S

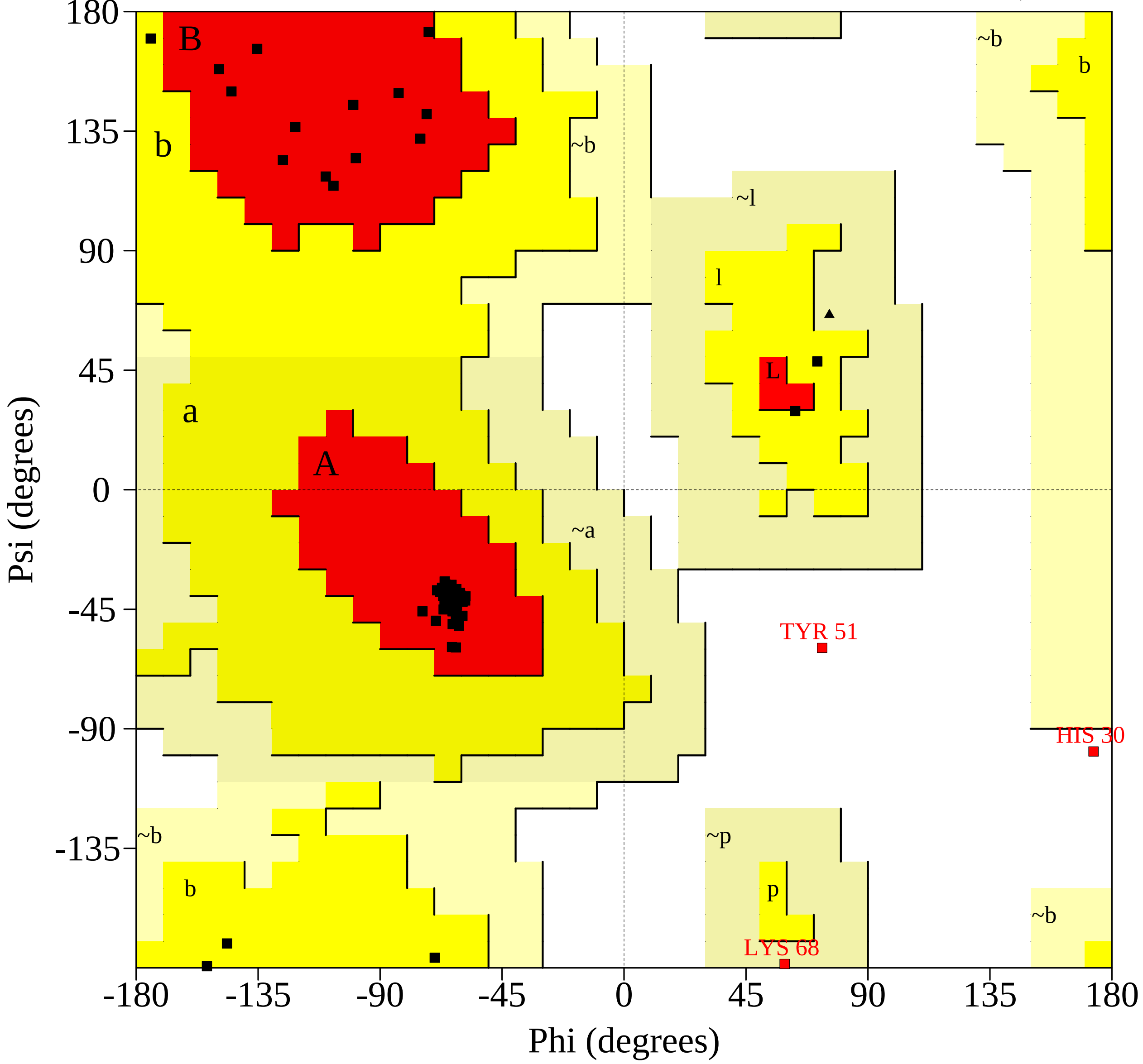

Plot statistics

Residues in most favoured regions [A,B,L]	62	87.3%
Residues in additional allowed regions [a,b,l,p]	6	8.5%
Residues in generously allowed regions [~a,~b,~l,~p]	1	1.4%
Residues in disallowed regions	2	2.8%
	----	------
Number of non-glycine and non-proline residues	71	100.0%
Number of end-residues (excl. Gly and Pro)	1	
Number of glycine residues (shown as triangles)	2	
Number of proline residues	0	

Total number of residues	74	

Based on an analysis of 118 structures of resolution of at least 2.0 Angstroms and R-factor no greater than 20%, a good quality model would be expected to have over 90% in the most favoured regions.

HUMAN_kix_I64A_K68S_01.ps

PROCHECK

Ramachandran Plot

HUMAN Med15 kix with double mutations I64G, D68G

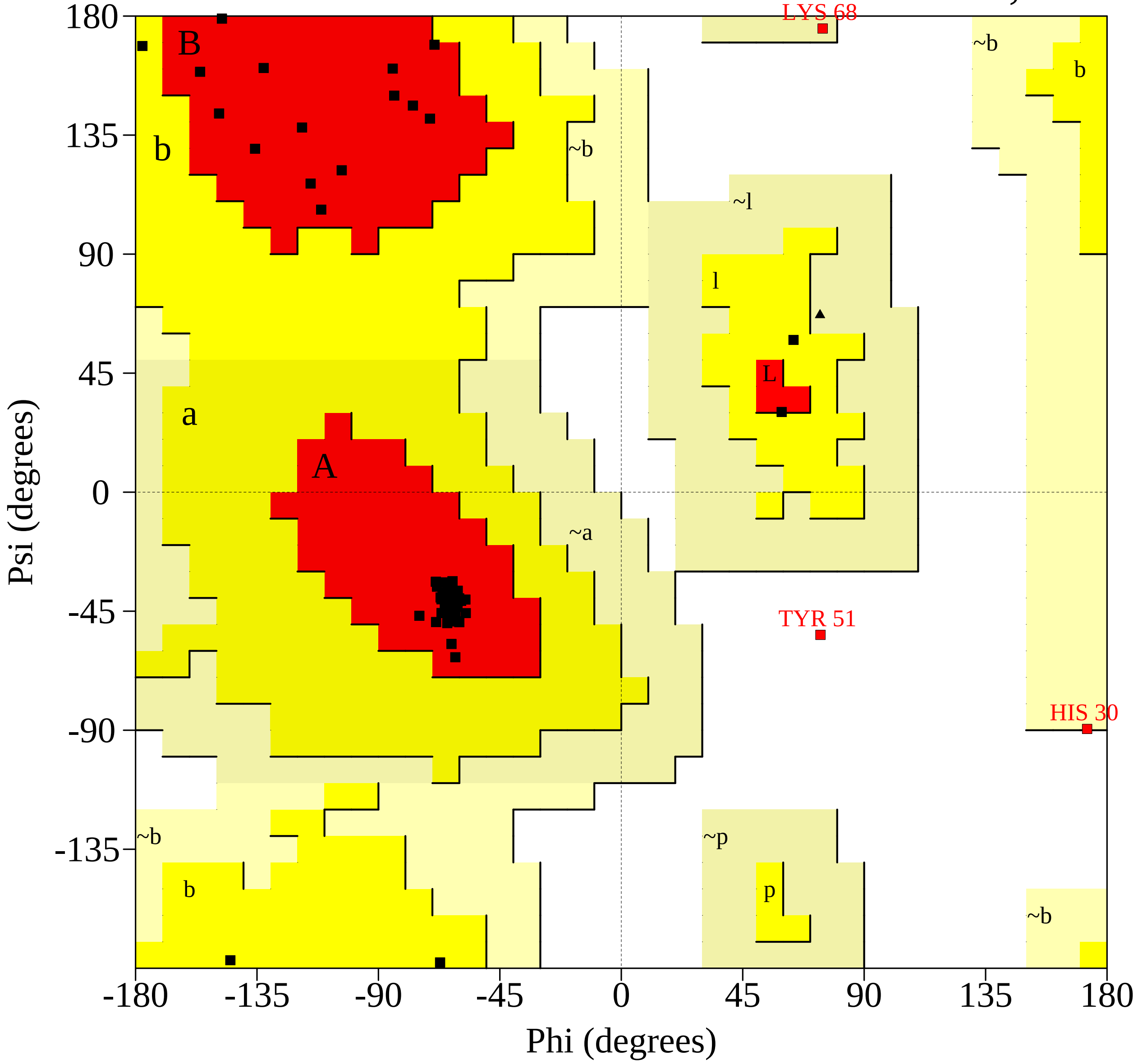

Plot statistics

Residues in most favoured regions [A,B,L]	62	89.9%
Residues in additional allowed regions [a,b,l,p]	4	5.8%
Residues in generously allowed regions [~a,~b,~l,~p]	2	2.9%
Residues in disallowed regions	1	1.4%
	----	------
Number of non-glycine and non-proline residues	69	100.0%
Number of end-residues (excl. Gly and Pro)	1	
Number of glycine residues (shown as triangles)	4	
Number of proline residues	0	

Total number of residues	74	

Based on an analysis of 118 structures of resolution of at least 2.0 Angstroms and R-factor no greater than 20%, a good quality model would be expected to have over 90% in the most favoured regions.

HUMAN_kix_I64G_D68G_cor_01.ps

PROCHECK

Ramachandran Plot

HUMAN Med15 kix with double mutations I64M, D68K

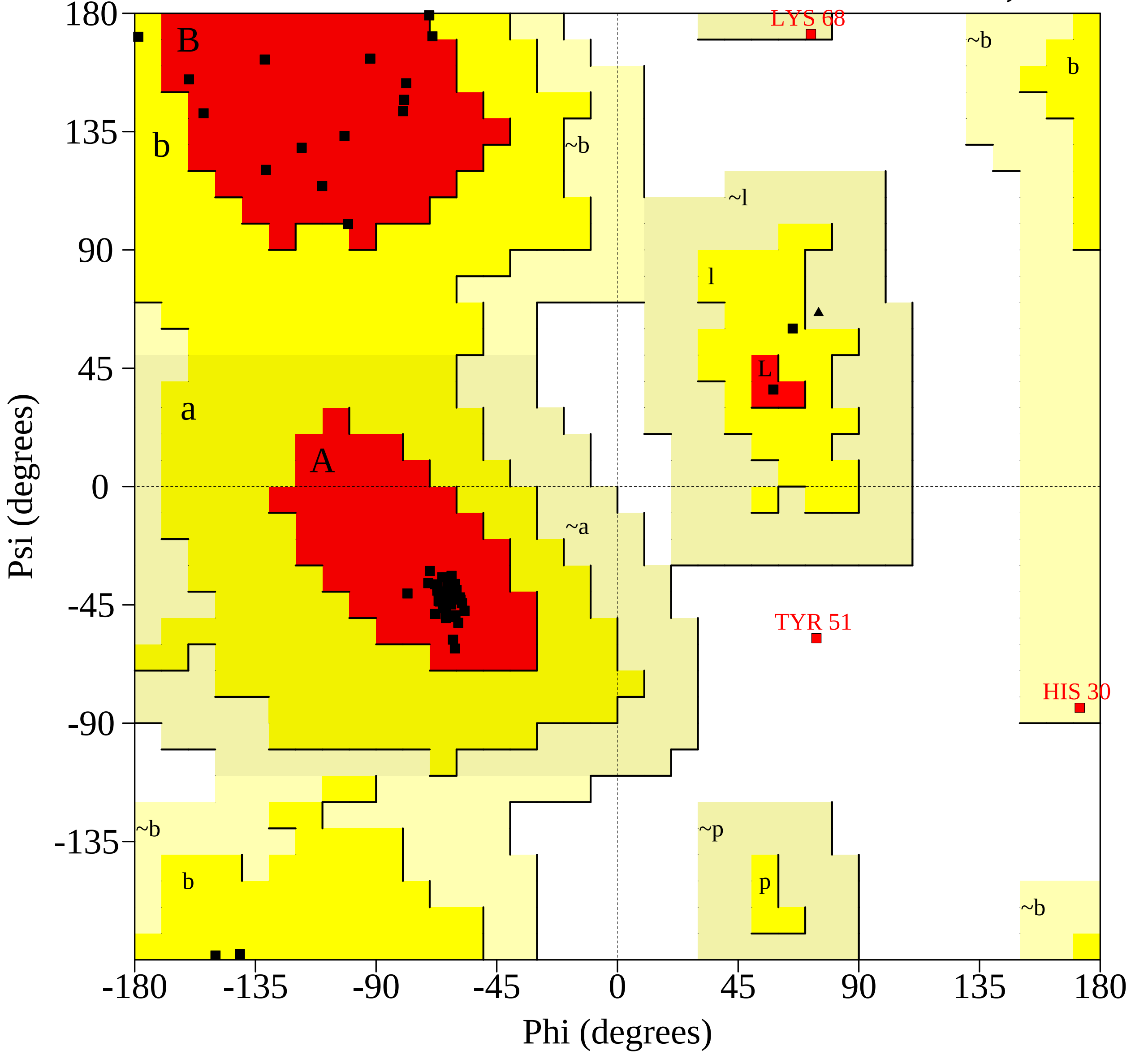

Plot statistics

Residues in most favoured regions [A,B,L]	62	87.3%
Residues in additional allowed regions [a,b,l,p]	6	8.5%
Residues in generously allowed regions [~a,~b,~l,~p]	2	2.8%
Residues in disallowed regions	1	1.4%
	----	------
Number of non-glycine and non-proline residues	71	100.0%
Number of end-residues (excl. Gly and Pro)	1	
Number of glycine residues (shown as triangles)	2	
Number of proline residues	0	

Total number of residues	74	

Based on an analysis of 118 structures of resolution of at least 2.0 Angstroms and R-factor no greater than 20%, a good quality model would be expected to have over 90% in the most favoured regions.

PROCHECK

Ramachandran Plot

HUMAN Med15 kix with double mutations I64P, D68P

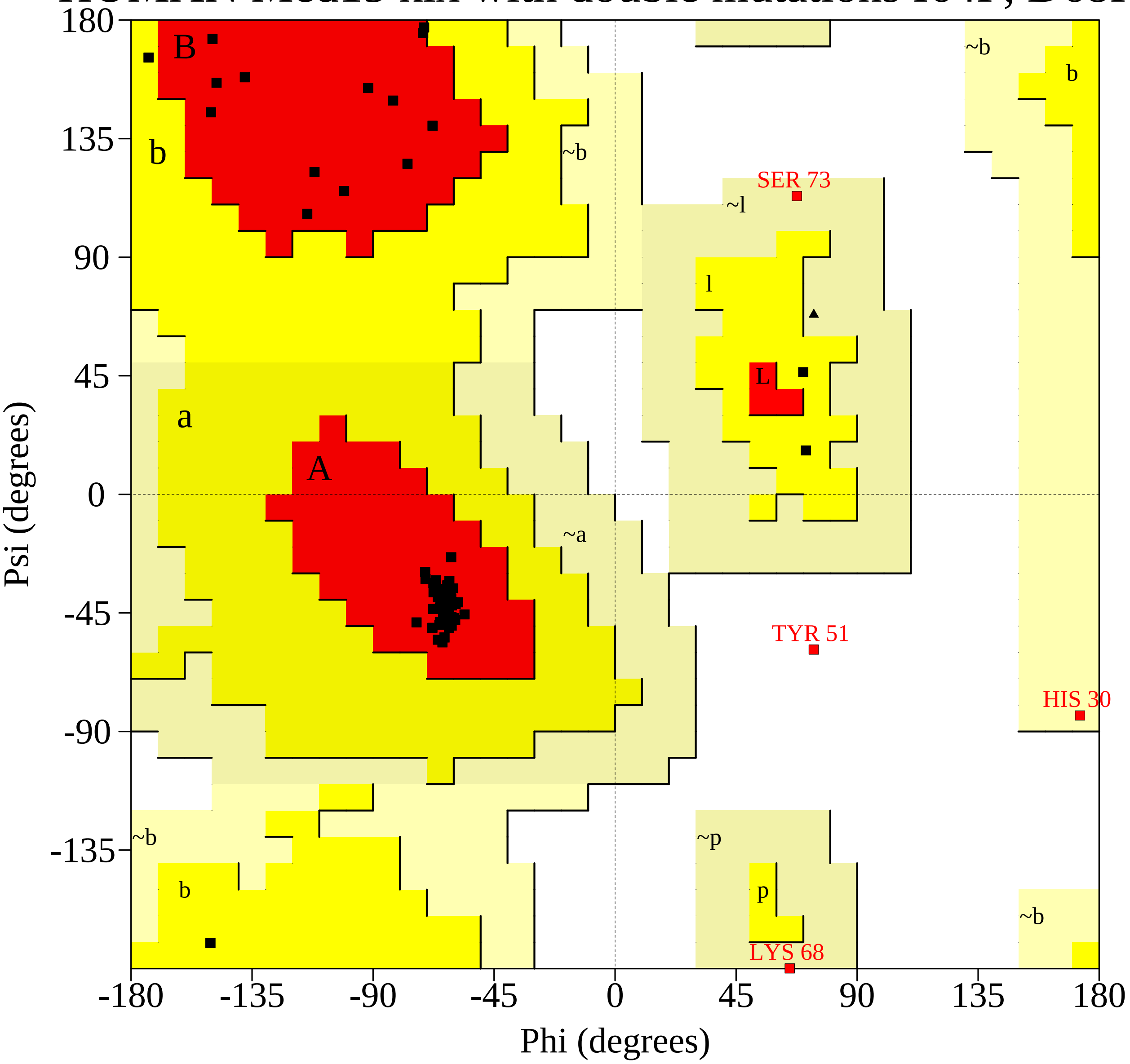

Plot statistics

Residues in most favoured regions [A,B,L]	61	88.4%
Residues in additional allowed regions [a,b,l,p]	4	5.8%
Residues in generously allowed regions [~a,~b,~l,~p]	3	4.3%
Residues in disallowed regions	1	1.4%
	----	------
Number of non-glycine and non-proline residues	69	100.0%
Number of end-residues (excl. Gly and Pro)	1	
Number of glycine residues (shown as triangles)	2	
Number of proline residues	2	

Total number of residues	74	

Based on an analysis of 118 structures of resolution of at least 2.0 Angstroms
and R-factor no greater than 20%, a good quality model would be expected
to have over 90% in the most favoured regions.

HUMAN_kix_I64P_K68P_01.ps

PROCHECK

Ramachandran Plot

HUMAN Med15 kix with double mutations I64Y, D68K

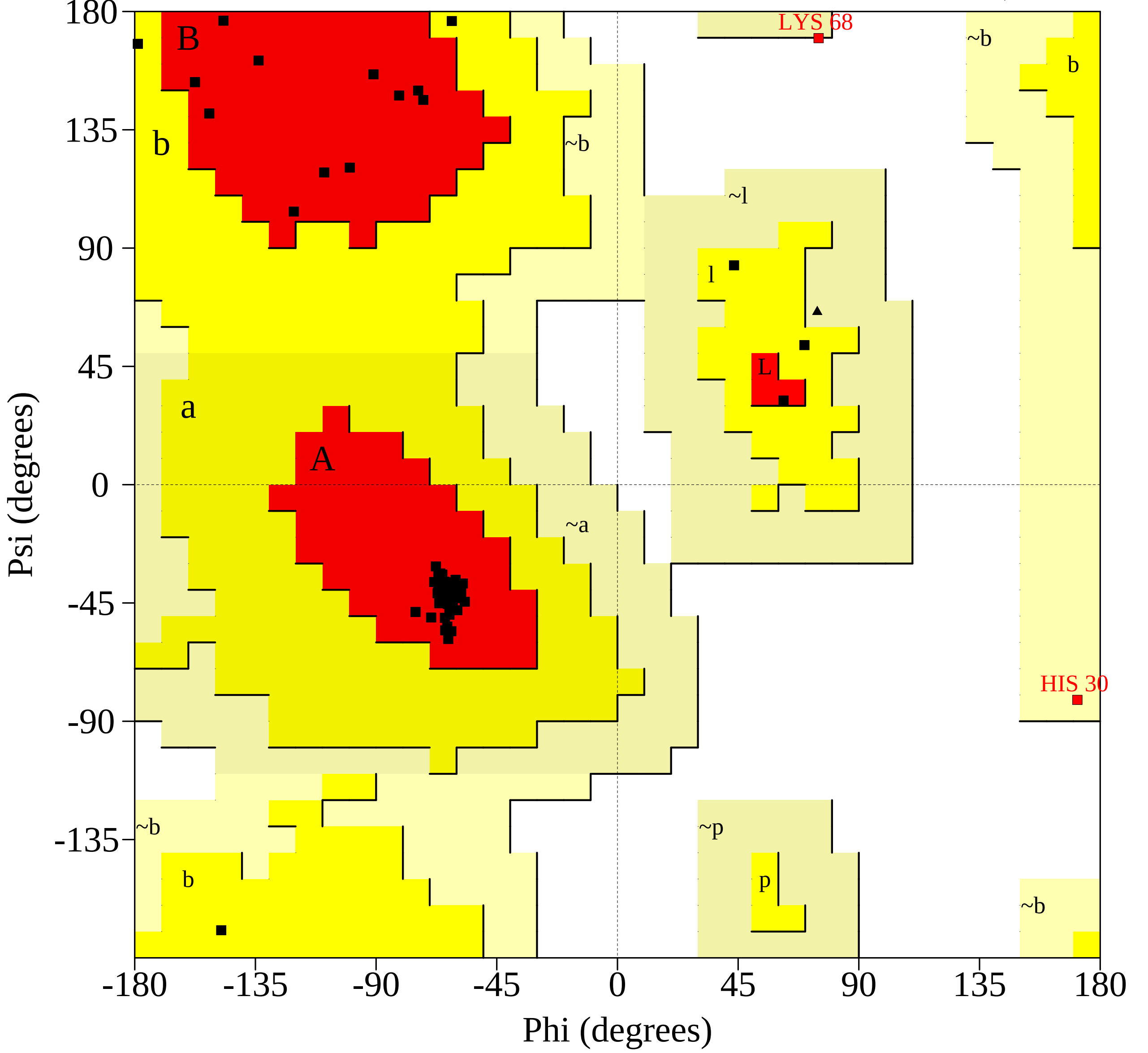

Plot statistics

Residues in most favoured regions [A,B,L]	64	90.1%
Residues in additional allowed regions [a,b,l,p]	5	7.0%
Residues in generously allowed regions [~a,~b,~l,~p]	1	1.4%
Residues in disallowed regions	1	1.4%
	----	------
Number of non-glycine and non-proline residues	71	100.0%
Number of end-residues (excl. Gly and Pro)	1	
Number of glycine residues (shown as triangles)	2	
Number of proline residues	0	

Total number of residues	74	

Based on an analysis of 118 structures of resolution of at least 2.0 Angstroms and R-factor no greater than 20%, a good quality model would be expected to have over 90% in the most favoured regions.

HUMAN_kix_I64Y_D68K_cor_01.ps

Some more homology modeling statistics

```
+----------<<<  P  R  O  C  H  E  C  K     S  U  M  M  A  R  Y  >>>----------+

 AtMed21                                   139 residues

* Ramachandran plot:   87.4% core   10.1% allow    1.7% gener    0.8% disall

* All Ramachandrans:   13 labelled residues (out of 137)
  Chi1-chi2 plots:      0 labelled residues (out of  82)
  Main-chain params:    6 better     0 inside      0 worse
  Side-chain params:    5 better     0 inside      0 worse

* Residue properties: Max.deviation:    18.8              Bad contacts:    8
*                  Bond len/angle:    8.7    Morris et al class:  1  1  2
*     5 cis-peptides
+ G-factors           Dihedrals:   0.01  Covalent:  -0.55    Overall:  -0.19

  M/c bond lengths: 98.1% within limits   1.9% highlighted
* M/c bond angles:  87.9% within limits  12.1% highlighted       3 off graph
  Planar groups:   100.0% within limits   0.0% highlighted

 +----------------------------------------------------------------------------+
   + May be worth investigating further.  * Worth investigating further.


 +----------<<<  P  R  O  C  H  E  C  K     S  U  M  M  A  R  Y  >>>----------+

 AtMed31   1.5                                  81 residues

+ Ramachandran plot:   93.1% core    5.6% allow    1.4% gener    0.0% disall

+ All Ramachandrans:    2 labelled residues (out of  79)
  Chi1-chi2 plots:      0 labelled residues (out of  63)
  Main-chain params:    6 better     0 inside      0 worse
  Side-chain params:    5 better     0 inside      0 worse

* Residue properties: Max.deviation:     4.2              Bad contacts:    3
*                  Bond len/angle:   41.4    Morris et al class:  1  1  3
+     1 cis-peptides
  G-factors           Dihedrals:   0.15  Covalent:  -0.39    Overall:  -0.01

  M/c bond lengths: 99.8% within limits   0.2% highlighted
* M/c bond angles:  94.6% within limits   5.4% highlighted       2 off graph
  Planar groups:   100.0% within limits   0.0% highlighted

 +----------------------------------------------------------------------------+
   + May be worth investigating further.  * Worth investigating further.
```

```
+----------<<<  P R O C H E C K    S U M M A R Y  >>>----------+

 AtMed7   1.5                                    90 residues

* Ramachandran plot:   94.0% core    4.8% allow    0.0% gener    1.2% disall

* All Ramachandrans:    3 labelled residues (out of  88)
+ Chi1-chi2 plots:      1 labelled residues (out of  71)
  Main-chain params:    6 better     0 inside      0 worse
  Side-chain params:    5 better     0 inside      0 worse

+ Residue properties: Max.deviation:     4.3              Bad contacts:     8
+                     Bond len/angle:    3.5    Morris et al class:  1  1  2

  G-factors           Dihedrals:   0.41  Covalent:   0.54    Overall:   0.46

  M/c bond lengths:100.0% within limits   0.0% highlighted
  M/c bond angles:  99.4% within limits   0.6% highlighted
  Planar groups:   100.0% within limits   0.0% highlighted

 +----------------------------------------------------------------------------+
   + May be worth investigating further.  * Worth investigating further.
```

```
 +----------<<<  P R O C H E C K    S U M M A R Y  >>>----------+

 HUMAN Med15 kix with double mutations I64A, D68A   2.0                          74 residues

* Ramachandran plot:   84.5% core   11.3% allow    1.4% gener    2.8% disall

* All Ramachandrans:    5 labelled residues (out of  72)
+ Chi1-chi2 plots:      1 labelled residues (out of  46)
+ Main-chain params:    3 better     0 inside      3 worse
  Side-chain params:    5 better     0 inside      0 worse

* Residue properties: Max.deviation:    10.9              Bad contacts:    18
*                     Bond len/angle:   17.9    Morris et al class:  1  1  2

* G-factors           Dihedrals:  -0.12  Covalent:  -3.27    Overall:  -1.23

* M/c bond lengths: 85.6% within limits  14.4% highlighted       5 off graph
* M/c bond angles:  72.4% within limits  27.6% highlighted      20 off graph
  Planar groups:   100.0% within limits   0.0% highlighted

 +----------------------------------------------------------------------------+
   + May be worth investigating further.  * Worth investigating further.
```

```
+----------<<<  P R O C H E C K    S U M M A R Y  >>>----------+

 HUMAN Med15 kix with double mutations I64A, D68S    2.0                    74 residues

* Ramachandran plot:   87.3% core    8.5% allow    1.4% gener    2.8% disall

* All Ramachandrans:    4 labelled residues (out of  72)
  Chi1-chi2 plots:      0 labelled residues (out of  46)
  Main-chain params:    6 better     0 inside      0 worse
  Side-chain params:    5 better     0 inside      0 worse

+ Residue properties: Max.deviation:    18.7             Bad contacts:    2
+                   Bond len/angle:    4.3    Morris et al class:  1  1  2

  G-factors          Dihedrals:   0.09  Covalent:  -0.18    Overall:  -0.01

  M/c bond lengths: 99.7% within limits   0.3% highlighted
  M/c bond angles:  93.2% within limits   6.8% highlighted
  Planar groups:   100.0% within limits   0.0% highlighted

+----------------------------------------------------------------------------+
  + May be worth investigating further.  * Worth investigating further.


+----------<<<  P R O C H E C K    S U M M A R Y  >>>----------+

 HUMAN Med15 kix with double mutations I64G, D68G   2.0                    74 residues

* Ramachandran plot:   89.9% core    5.8% allow    2.9% gener    1.4% disall

* All Ramachandrans:    3 labelled residues (out of  72)
  Chi1-chi2 plots:      0 labelled residues (out of  46)
  Main-chain params:    6 better     0 inside      0 worse
  Side-chain params:    5 better     0 inside      0 worse

+ Residue properties: Max.deviation:    18.5             Bad contacts:    2
+                   Bond len/angle:    5.0    Morris et al class:  1  1  2

  G-factors          Dihedrals:   0.11  Covalent:  -0.14    Overall:   0.02

  M/c bond lengths: 99.7% within limits   0.3% highlighted
  M/c bond angles:  94.1% within limits   5.9% highlighted
  Planar groups:   100.0% within limits   0.0% highlighted

+----------------------------------------------------------------------------+
  + May be worth investigating further.  * Worth investigating further.
```

```
+----------<<<  P R O C H E C K    S U M M A R Y  >>>----------+
```

HUMAN Med15 kix with double mutations I64M, D68K   1.5   74 residues

```
* Ramachandran plot:   87.3% core    8.5% allow    2.8% gener    1.4% disall

* All Ramachandrans:    3 labelled residues (out of  72)
  Chi1-chi2 plots:      0 labelled residues (out of  48)
  Main-chain params:    6 better     0 inside      0 worse
  Side-chain params:    5 better     0 inside      0 worse

+ Residue properties: Max.deviation:    18.6            Bad contacts:    3
+                     Bond len/angle:    4.8    Morris et al class:  1  1  2

  G-factors           Dihedrals:   0.09  Covalent:  -0.17    Overall:   0.00

  M/c bond lengths: 99.5% within limits   0.5% highlighted
  M/c bond angles:  92.6% within limits   7.4% highlighted
  Planar groups:   100.0% within limits   0.0% highlighted

+----------------------------------------------------------------------------+
   + May be worth investigating further.  * Worth investigating further.
```

```
+----------<<<  P R O C H E C K    S U M M A R Y  >>>----------+
```

HUMAN Med15 kix with double mutations I64P, D68P   2.0   74 residues

```
* Ramachandran plot:   88.4% core    5.8% allow    4.3% gener    1.4% disall

* All Ramachandrans:    5 labelled residues (out of  72)
  Chi1-chi2 plots:      0 labelled residues (out of  46)
  Main-chain params:    6 better     0 inside      0 worse
  Side-chain params:    5 better     0 inside      0 worse

+ Residue properties: Max.deviation:    19.0            Bad contacts:    3
+                     Bond len/angle:    4.7    Morris et al class:  1  1  2

  G-factors           Dihedrals:  -0.01  Covalent:  -0.31    Overall:  -0.12

  M/c bond lengths: 98.9% within limits   1.1% highlighted
  M/c bond angles:  89.8% within limits  10.2% highlighted
  Planar groups:   100.0% within limits   0.0% highlighted

+----------------------------------------------------------------------------+
   + May be worth investigating further.  * Worth investigating further.
```

```
+----------<<< P R O C H E C K   S U M M A R Y >>>----------+

 HUMAN Med15 kix with double mutations I64Y, D68K   2.0          74 residues

* Ramachandran plot:   90.1% core    7.0% allow    1.4% gener    1.4% disall

* All Ramachandrans:    3 labelled residues (out of  72)
  Chi1-chi2 plots:      0 labelled residues (out of  48)
  Main-chain params:    6 better     0 inside      0 worse
  Side-chain params:    5 better     0 inside      0 worse

+ Residue properties: Max.deviation:     4.0            Bad contacts:    1
+                     Bond len/angle:    4.3    Morris et al class:  1  1  2

  G-factors          Dihedrals:   0.09  Covalent:  -0.12    Overall:   0.01

  M/c bond lengths: 99.5% within limits   0.5% highlighted
  M/c bond angles:  93.0% within limits   7.0% highlighted
  Planar groups:   100.0% within limits   0.0% highlighted

+----------------------------------------------------------------------------+
   + May be worth investigating further.  * Worth investigating further.
```
