# Supplementary material for: Evolution of disorder in Mediator complex and its functional relevance
Source: Nucleic Acids Res. 2015 Nov 20;44(4):1591–612. doi: 10.1093/nar/gkv1135 (PMC4770211; doi:10.1093/nar/gkv1135)
Supplement: SUPPLEMENTARY DATA [file supp_gkv1135_nar-01763-n-2015-File011.zip › SF_4.pdf]

This file contains a schematic of the Intrinsically disordered regions (IDRs) in the Mediator complex subunits (Med1, Med2/Med32, Med3, Med4, Med5/Med33, Med6, Med7, Med8, Med9, Med10, Med11, Med12, Med13, Med14, Med15, Med16, Med17, Med18, Med19, Med20, Med21, Med22, Med23, Med25, Med26, Med28, Med30, Med31, Med34, Med35, Med36, Med37, Cdk8 and CycC) of Plants. List of organisms used in the current study are present in supplementary table ST1.

MED2

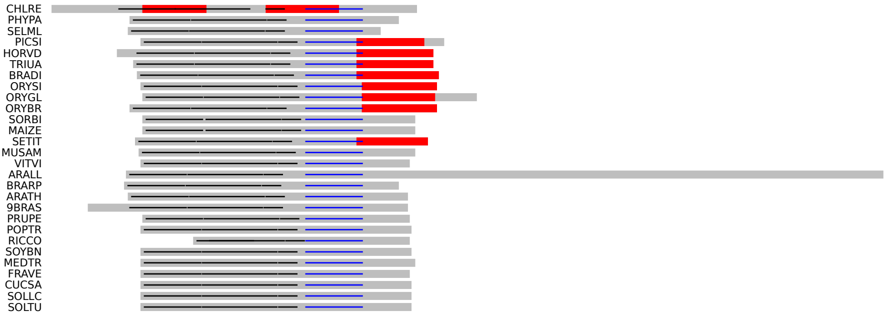

CHLRE
PHYPA
SELML
PICSI
HORVD
TRIUA
BRADI
ORYSI
ORYGL
ORYBR
SORBI
MAIZE
SETIT
MUSAM
VITVI
ARALL
BRARP
ARATH
9BRAS
PRUPE
POPTR
RICCO
SOYBN
MEDTR
FRAVE
CUCSA
SOLLC
SOLTU

MED3

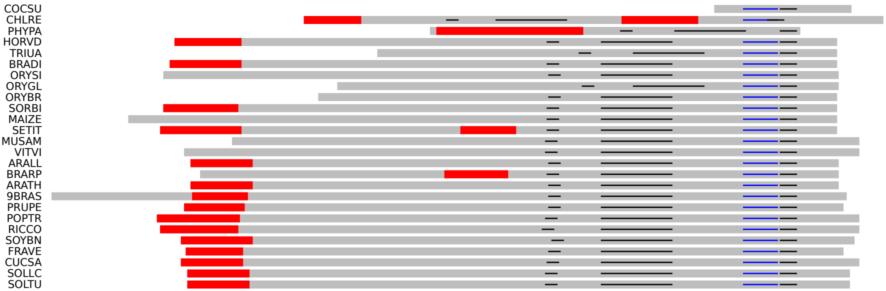
COCSU
CHLRE
PHYPA
HORVD
TRIUA
BRADI
ORYSI
ORYGL
ORYBR
SORBI
MAIZE
SETIT
MUSAM
VITVI
ARALL
BRARP
ARATH
9BRAS
PRUPE
POPTR
RICCO
SOYBN
FRAVE
CUCSA
SOLLC
SOLTU

MED4

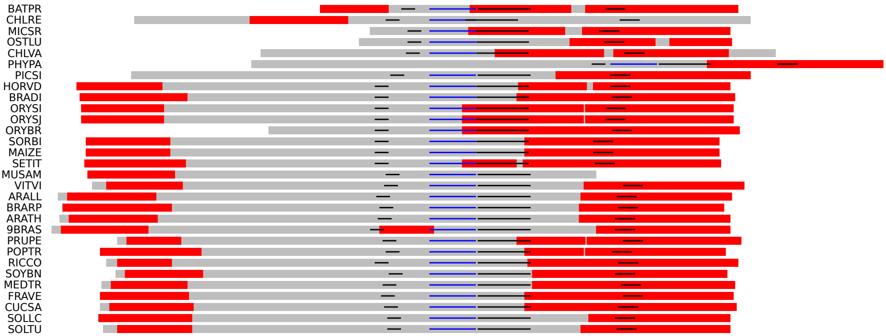

BATPR
CHLRE
MICSR
OSTLU
CHLVA
PHYPA
PICSI
HORVD
BRADI
ORYSI
ORYSJ
ORYBR
SORBI
MAIZE
SETIT
MUSAM
VITVI
ARALL
BRARP
ARATH
9BRAS
PRUPE
POPTR
RICCO
SOYBN
MEDTR
FRAVE
CUCSA
SOLLC
SOLTU

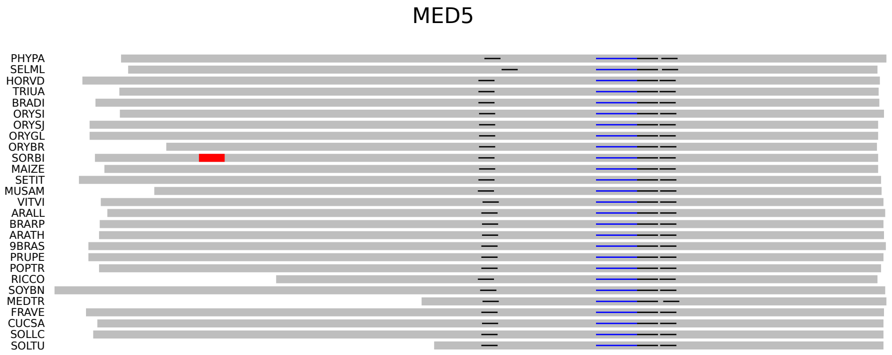
MED5
PHYPA
SELML
HORVD
TRIUA
BRADI
ORYSI
ORYSJ
ORYGL
ORYBR
SORBI
MAIZE
SETIT
MUSAM
VITVI
ARALL
BRARP
ARATH
9BRAS
PRUPE
POPTR
RICCO
SOYBN
MEDTR
FRAVE
CUCSA
SOLLC
SOLTU

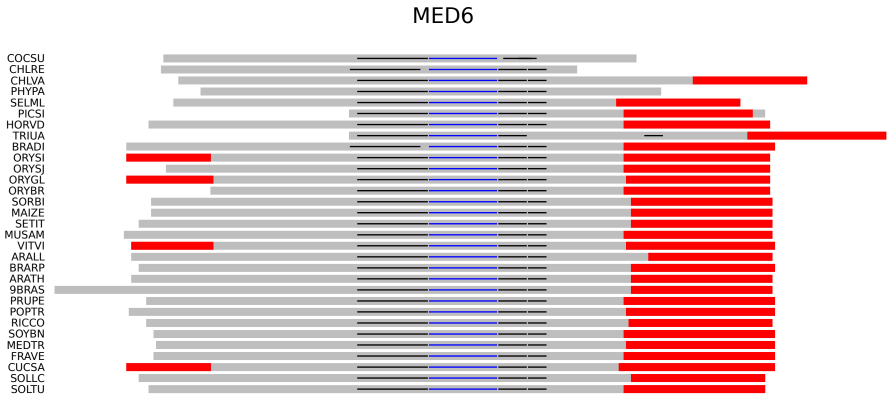
MED6
COCSU
CHLRE
CHLVA
PHYPA
SELML
PICSI
HORVD
TRIUA
BRADI
ORYSI
ORYSJ
ORYGL
ORYBR
SORBI
MAIZE
SETIT
MUSAM
VITVI
ARALL
BRARP
ARATH
9BRAS
PRUPE
POPTR
RICCO
SOYBN
MEDTR
FRAVE
CUCSA
SOLLC
SOLTU

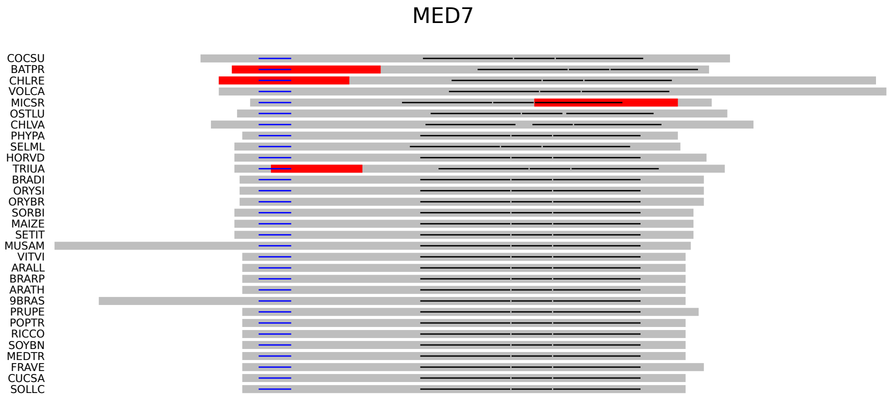
MED7
COCSU
BATPR
CHLRE
VOLCA
MICSR
OSTLU
CHLVA
PHYPA
SELML
HORVD
TRIUA
BRADI
ORYSI
ORYBR
SORBI
MAIZE
SETIT
MUSAM
VITVI
ARALL
BRARP
ARATH
9BRAS
PRUPE
POPTR
RICCO
SOYBN
MEDTR
FRAVE
CUCSA
SOLLC

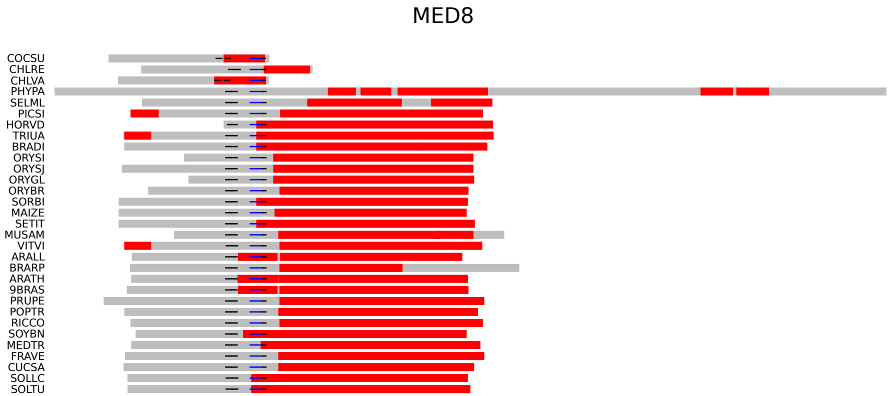
MED8
COCSU
CHLRE
CHLVA
PHYPA
SELML
PICSI
HORVD
TRIUA
BRADI
ORYSI
ORYSJ
ORYGL
ORYBR
SORBI
MAIZE
SETIT
MUSAM
VITVI
ARALL
BRARP
ARATH
9BRAS
PRUPE
POPTR
RICCO
SOYBN
MEDTR
FRAVE
CUCSA
SOLLC
SOLTU

MED9

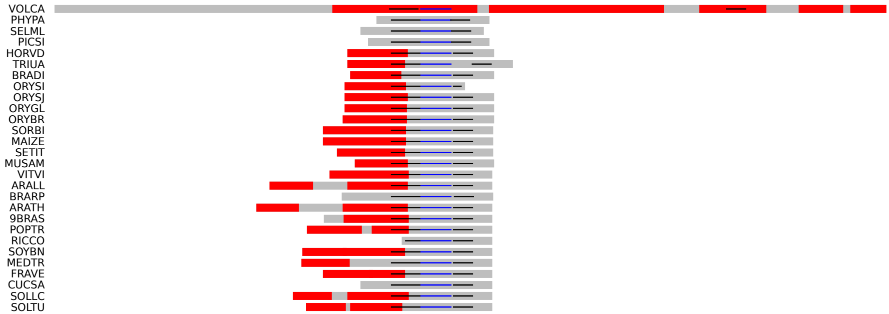
VOLCA
PHYPA
SELML
PICSI
HORVD
TRIUA
BRADI
ORYSI
ORYSJ
ORYGL
ORYBR
SORBI
MAIZE
SETIT
MUSAM
VITVI
ARALL
BRARP
ARATH
9BRAS
POPTR
RICCO
SOYBN
MEDTR
FRAVE
CUCSA
SOLLC
SOLTU

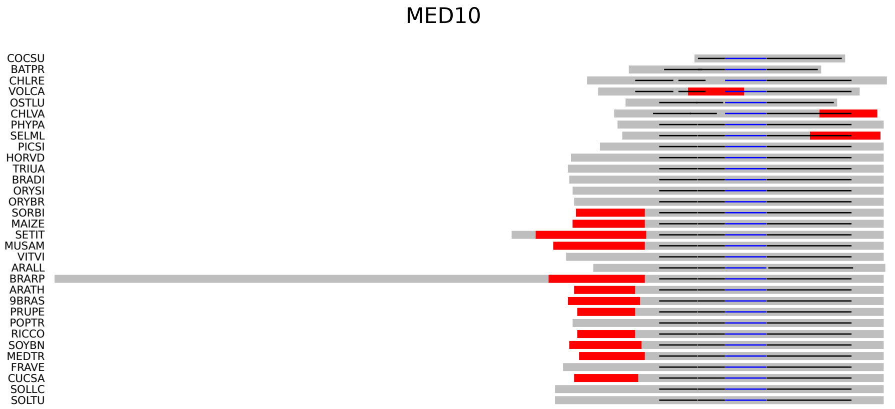
MED10
COCSU
BATPR
CHLRE
VOLCA
OSTLU
CHLVA
PHYPA
SELML
PICSI
HORVD
TRIUA
BRADI
ORYSI
ORYBR
SORBI
MAIZE
SETIT
MUSAM
VITVI
ARALL
BRARP
ARATH
9BRAS
PRUPE
POPTR
RICCO
SOYBN
MEDTR
FRAVE
CUCSA
SOLLC
SOLTU

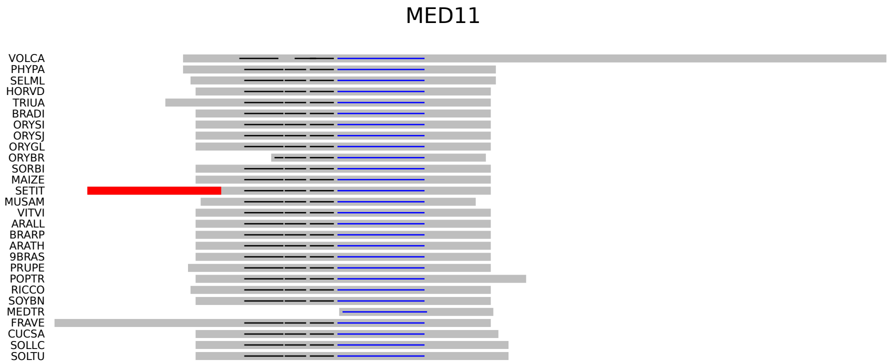
MED11
VOLCA
PHYPA
SELML
HORVD
TRIUA
BRADI
ORYSI
ORYSJ
ORYGL
ORYBR
SORBI
MAIZE
SETIT
MUSAM
VITVI
ARALL
BRARP
ARATH
9BRAS
PRUPE
POPTR
RICCO
SOYBN
MEDTR
FRAVE
CUCSA
SOLLC
SOLTU

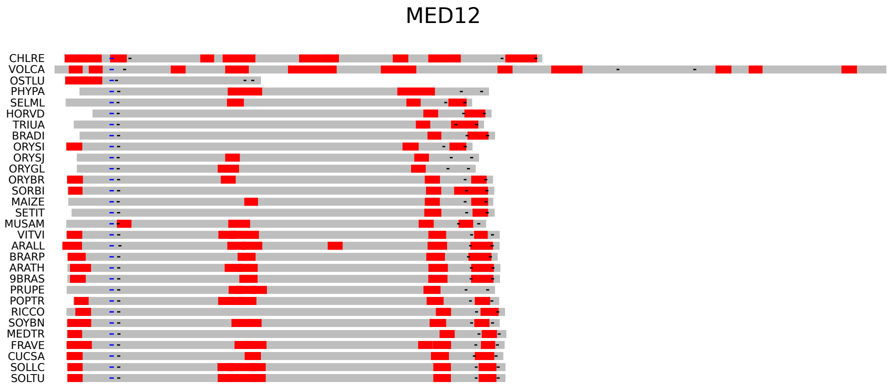
MED12
CHLRE
VOLCA
OSTLU
PHYPA
SELML
HORVD
TRIUA
BRADI
ORYSI
ORYSJ
ORYGL
ORYBR
SORBI
MAIZE
SETIT
MUSAM
VITVI
ARALL
BRARP
ARATH
9BRAS
PRUPE
POPTR
RICCO
SOYBN
MEDTR
FRAVE
CUCSA
SOLLC
SOLTU

MED13

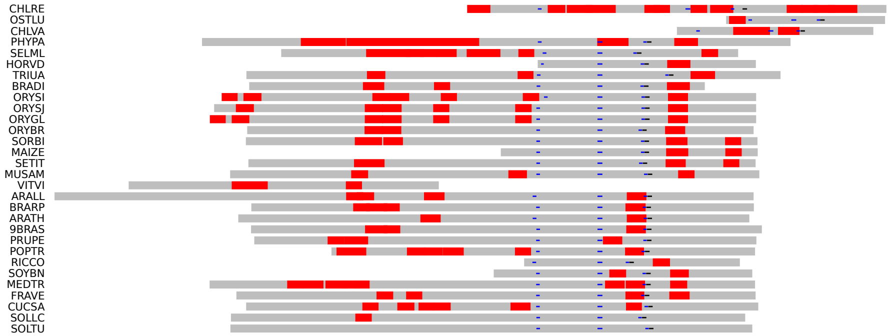
CHLRE
OSTLU
CHLVA
PHYPA
SELML
HORVD
TRIUA
BRADI
ORYSI
ORYSJ
ORYGL
ORYBR
SORBI
MAIZE
SETIT
MUSAM
VITVI
ARALL
BRARP
ARATH
9BRAS
PRUPE
POPTR
RICCO
SOYBN
MEDTR
FRAVE
CUCSA
SOLLC
SOLTU

MED14

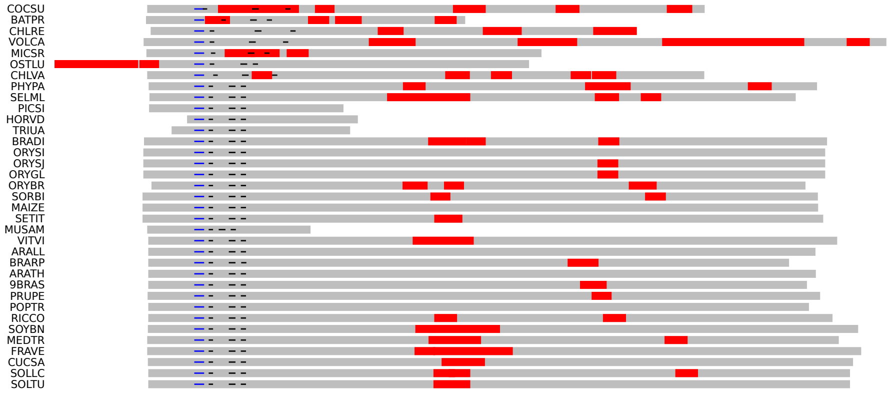

COCSU
BATPR
CHLRE
VOLCA
MICSR
OSTLU
CHLVA
PHYPA
SELML
PICSI
HORVD
TRIUA
BRADI
ORYSI
ORYSJ
ORYGL
ORYBR
SORBI
MAIZE
SETIT
MUSAM
VITVI
ARALL
BRARP
ARATH
9BRAS
PRUPE
POPTR
RICCO
SOYBN
MEDTR
FRAVE
CUCSA
SOLLC
SOLTU

MED15

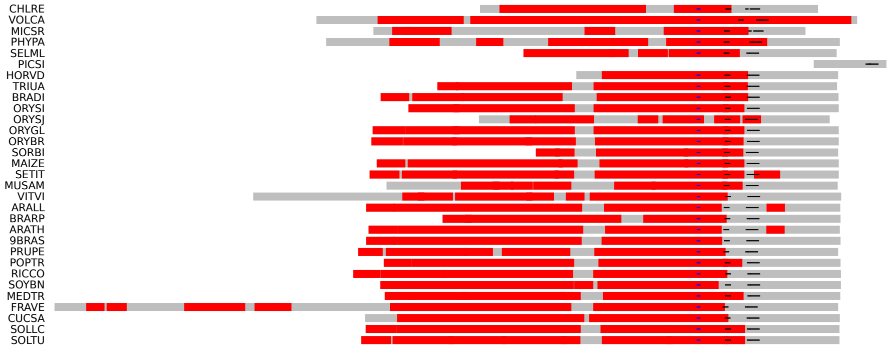
CHLRE
VOLCA
MICSR
PHYPA
SELML
PICSI
HORVD
TRIUA
BRADI
ORYSI
ORYSJ
ORYGL
ORYBR
SORBI
MAIZE
SETIT
MUSAM
VITVI
ARALL
BRARP
ARATH
9BRAS
PRUPE
POPTR
RICCO
SOYBN
MEDTR
FRAVE
CUCSA
SOLLC
SOLTU

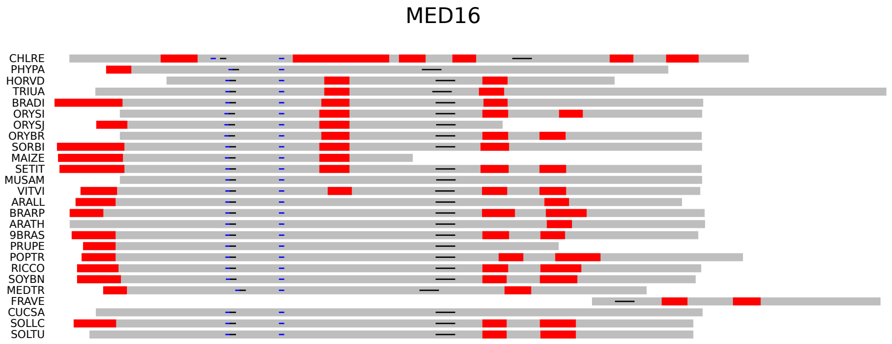
MED16
CHLRE
PHYPA
HORVD
TRIUA
BRADI
ORYSI
ORYSJ
ORYBR
SORBI
MAIZE
SETIT
MUSAM
VITVI
ARALL
BRARP
ARATH
9BRAS
PRUPE
POPTR
RICCO
SOYBN
MEDTR
FRAVE
CUCSA
SOLLC
SOLTU

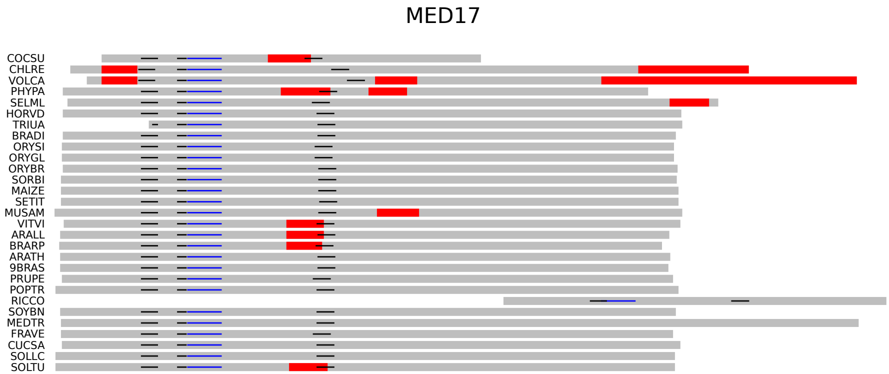
MED17
COCSU
CHLRE
VOLCA
PHYPA
SELML
HORVD
TRIUA
BRADI
ORYSI
ORYGL
ORYBR
SORBI
MAIZE
SETIT
MUSAM
VITVI
ARALL
BRARP
ARATH
9BRAS
PRUPE
POPTR
RICCO
SOYBN
MEDTR
FRAVE
CUCSA
SOLLC
SOLTU

MED18

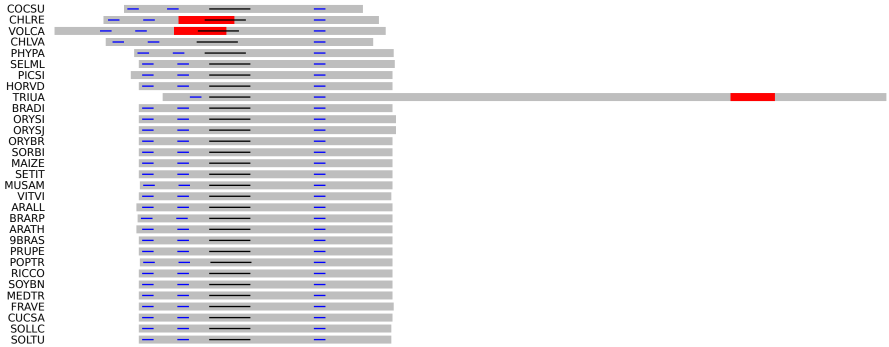
COCSU
CHLRE
VOLCA
CHLVA
PHYPA
SELML
PICSI
HORVD
TRIUA
BRADI
ORYSI
ORYSJ
ORYBR
SORBI
MAIZE
SETIT
MUSAM
VITVI
ARALL
BRARP
ARATH
9BRAS
PRUPE
POPTR
RICCO
SOYBN
MEDTR
FRAVE
CUCSA
SOLLC
SOLTU

MED19

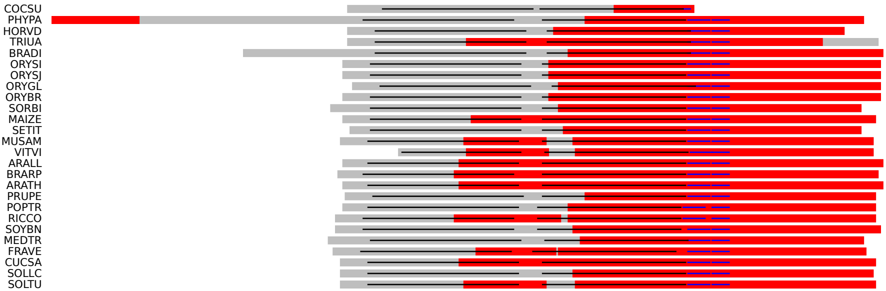
COCSU
PHYPA
HORVD
TRIUA
BRADI
ORYSI
ORYSJ
ORYGL
ORYBR
SORBI
MAIZE
SETIT
MUSAM
VITVI
ARALL
BRARP
ARATH
PRUPE
POPTR
RICCO
SOYBN
MEDTR
FRAVE
CUCSA
SOLLC
SOLTU

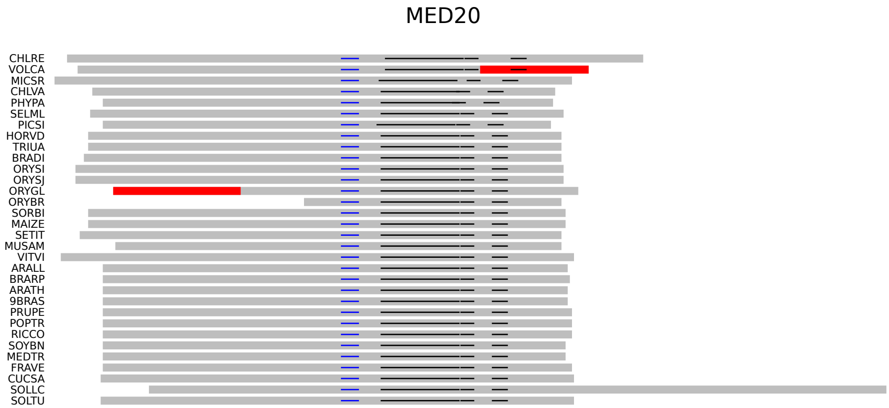
MED20
CHLRE
VOLCA
MICSR
CHLVA
PHYPA
SELML
PICSI
HORVD
TRIUA
BRADI
ORYSI
ORYSJ
ORYGL
ORYBR
SORBI
MAIZE
SETIT
MUSAM
VITVI
ARALL
BRARP
ARATH
9BRAS
PRUPE
POPTR
RICCO
SOYBN
MEDTR
FRAVE
CUCSA
SOLLC
SOLTU

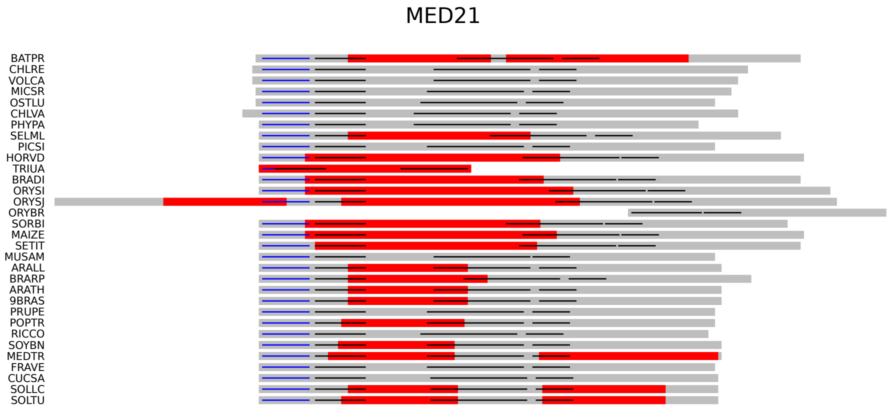
MED21
BATPR
CHLRE
VOLCA
MICSR
OSTLU
CHLVA
PHYPA
SELML
PICSI
HORVD
TRIUA
BRADI
ORYSI
ORYSJ
ORYBR
SORBI
MAIZE
SETIT
MUSAM
ARALL
BRARP
ARATH
9BRAS
PRUPE
POPTR
RICCO
SOYBN
MEDTR
FRAVE
CUCSA
SOLLC
SOLTU

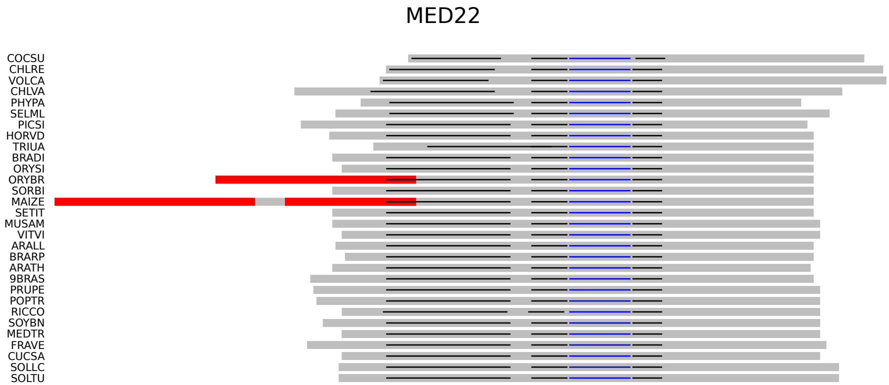
MED22
COCSU
CHLRE
VOLCA
CHLVA
PHYPA
SELML
PICSI
HORVD
TRIUA
BRADI
ORYSI
ORYBR
SORBI
MAIZE
SETIT
MUSAM
VITVI
ARALL
BRARP
ARATH
9BRAS
PRUPE
POPTR
RICCO
SOYBN
MEDTR
FRAVE
CUCSA
SOLLC
SOLTU

MED23

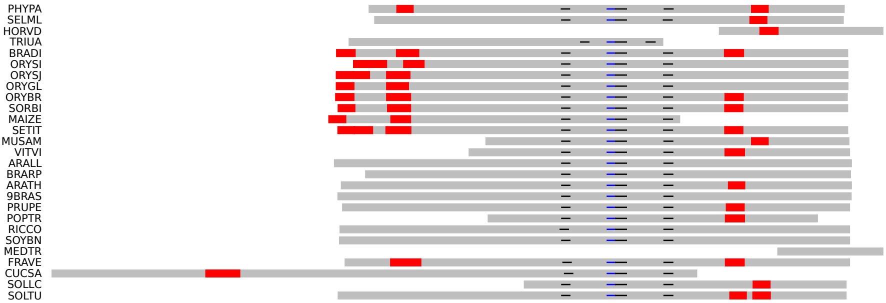
PHYPA
SELML
HORVD
TRIUA
BRADI
ORYSI
ORYSJ
ORYGL
ORYBR
SORBI
MAIZE
SETIT
MUSAM
VITVI
ARALL
BRARP
ARATH
9BRAS
PRUPE
POPTR
RICCO
SOYBN
MEDTR
FRAVE
CUCSA
SOLLC
SOLTU

MED25

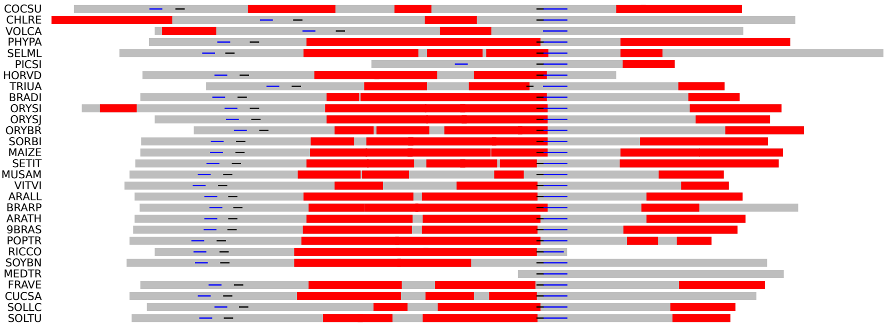
COCSU
CHLRE
VOLCA
PHYPA
SELML
PICSI
HORVD
TRIUA
BRADI
ORYSI
ORYSJ
ORYBR
SORBI
MAIZE
SETIT
MUSAM
VITVI
ARALL
BRARP
ARATH
9BRAS
POPTR
RICCO
SOYBN
MEDTR
FRAVE
CUCSA
SOLLC
SOLTU

MED26

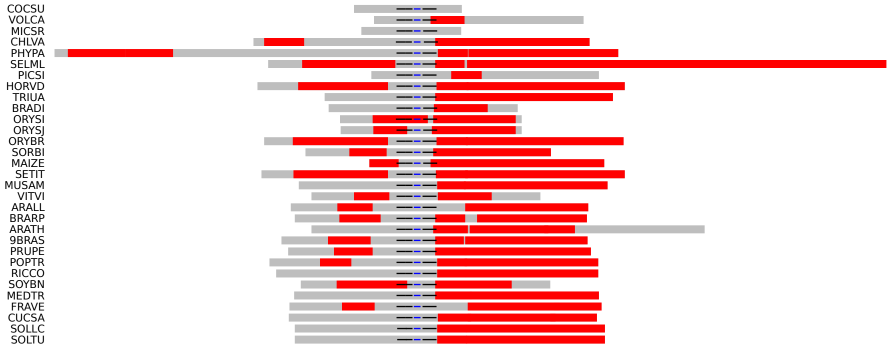
COCSU
VOLCA
MICSR
CHLVA
PHYPA
SELML
PICSI
HORVD
TRIUA
BRADI
ORYSI
ORYSJ
ORYBR
SORBI
MAIZE
SETIT
MUSAM
VITVI
ARALL
BRARP
ARATH
9BRAS
PRUPE
POPTR
RICCO
SOYBN
MEDTR
FRAVE
CUCSA
SOLLC
SOLTU

MED28

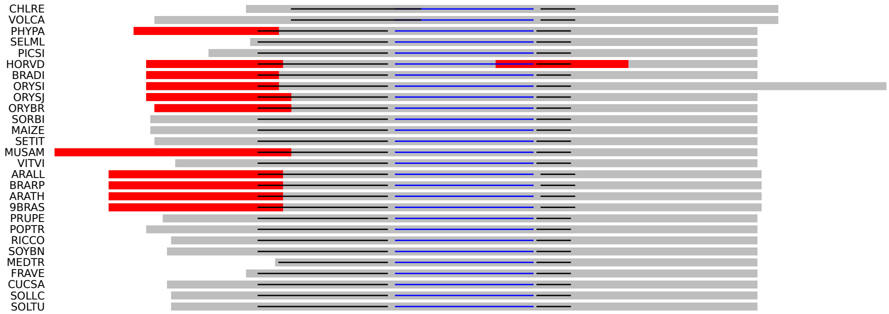
CHLRE
VOLCA
PHYPA
SELML
PICSI
HORVD
BRADI
ORYSI
ORYSJ
ORYBR
SORBI
MAIZE
SETIT
MUSAM
VITVI
ARALL
BRARP
ARATH
9BRAS
PRUPE
POPTR
RICCO
SOYBN
MEDTR
FRAVE
CUCSA
SOLLC
SOLTU

MED30

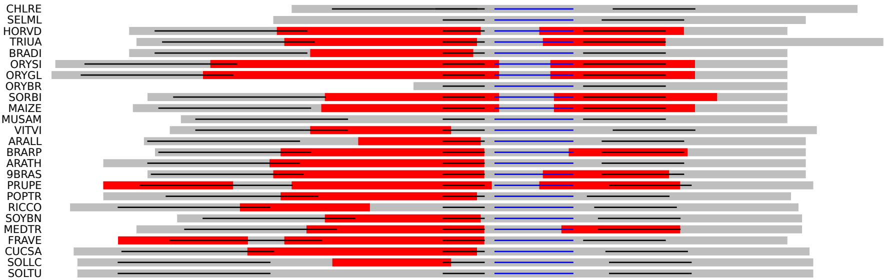
CHLRE
SELML
HORVD
TRIUA
BRADI
ORYSI
ORYGL
ORYBR
SORBI
MAIZE
MUSAM
VITVI
ARALL
BRARP
ARATH
9BRAS
PRUPE
POPTR
RICCO
SOYBN
MEDTR
FRAVE
CUCSA
SOLLC
SOLTU

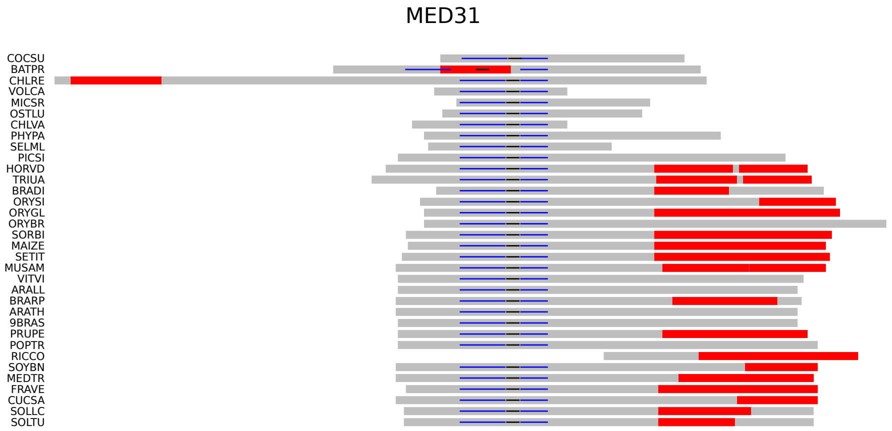
MED31
COCSU
BATPR
CHLRE
VOLCA
MICSR
OSTLU
CHLVA
PHYPA
SELML
PICSI
HORVD
TRIUA
BRADI
ORYSI
ORYGL
ORYBR
SORBI
MAIZE
SETIT
MUSAM
VITVI
ARALL
BRARP
ARATH
9BRAS
PRUPE
POPTR
RICCO
SOYBN
MEDTR
FRAVE
CUCSA
SOLLC
SOLTU

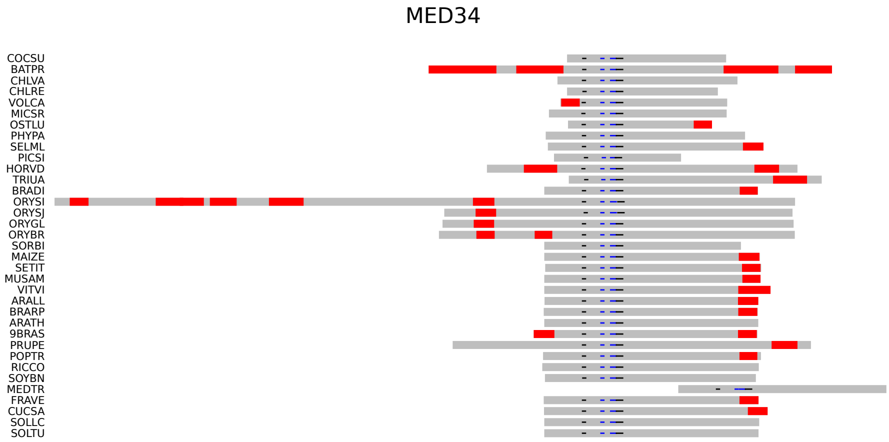
MED34
COCSU
BATPR
CHLVA
CHLRE
VOLCA
MICSR
OSTLU
PHYPA
SELML
PICSI
HORVD
TRIUA
BRADI
ORYSI
ORYSJ
ORYGL
ORYBR
SORBI
MAIZE
SETIT
MUSAM
VITVI
ARALL
BRARP
ARATH
9BRAS
PRUPE
POPTR
RICCO
SOYBN
MEDTR
FRAVE
CUCSA
SOLLC
SOLTU

MED35

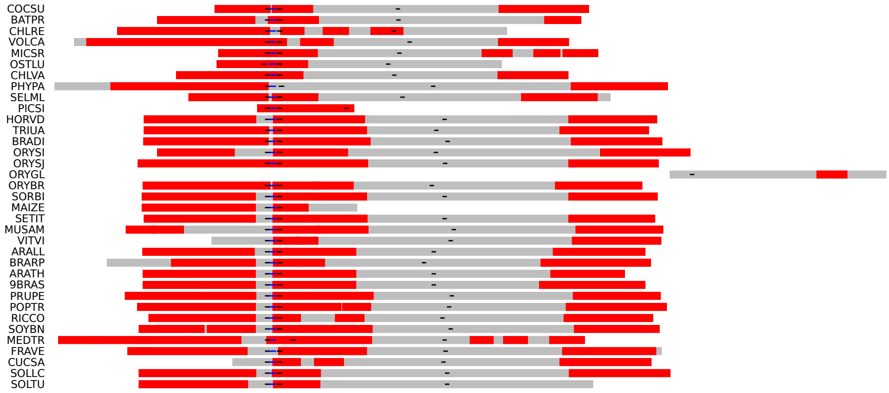

COCSU
BATPR
CHLRE
VOLCA
MICSR
OSTLU
CHLVA
PHYPA
SELML
PICSI
HORVD
TRIUA
BRADI
ORYSI
ORYSJ
ORYGL
ORYBR
SORBI
MAIZE
SETIT
MUSAM
VITVI
ARALL
BRARP
ARATH
9BRAS
PRUPE
POPTR
RICCO
SOYBN
MEDTR
FRAVE
CUCSA
SOLLC
SOLTU

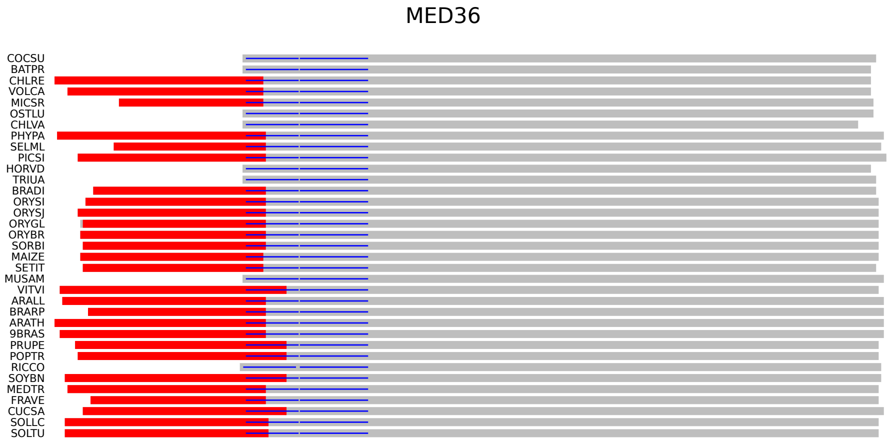
MED36
COCSU
BATPR
CHLRE
VOLCA
MICSR
OSTLU
CHLVA
PHYPA
SELML
PICSI
HORVD
TRIUA
BRADI
ORYSI
ORYSJ
ORYGL
ORYBR
SORBI
MAIZE
SETIT
MUSAM
VITVI
ARALL
BRARP
ARATH
9BRAS
PRUPE
POPTR
RICCO
SOYBN
MEDTR
FRAVE
CUCSA
SOLLC
SOLTU

MED37

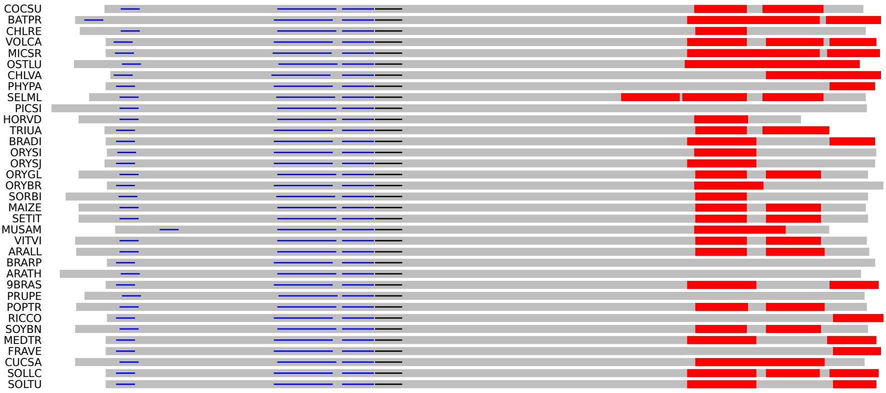
COCSU
BATPR
CHLRE
VOLCA
MICSR
OSTLU
CHLVA
PHYPA
SELML
PICSI
HORVD
TRIUA
BRADI
ORYSI
ORYSJ
ORYGL
ORYBR
SORBI
MAIZE
SETIT
MUSAM
VITVI
ARALL
BRARP
ARATH
9BRAS
PRUPE
POPTR
RICCO
SOYBN
MEDTR
FRAVE
CUCSA
SOLLC
SOLTU

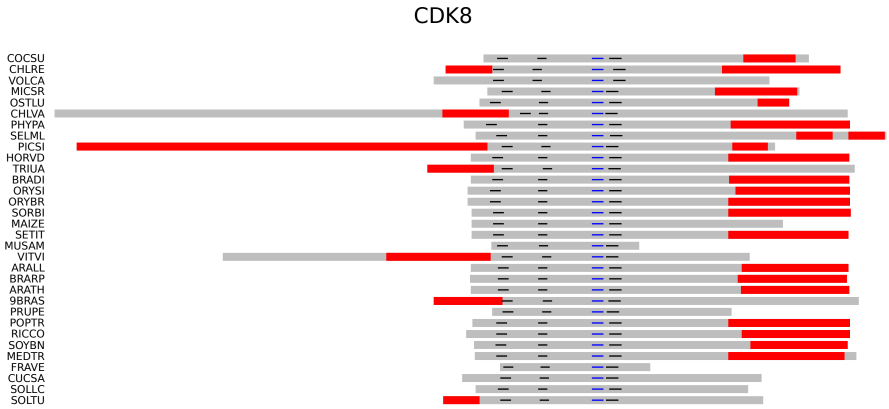
CDK8
COCSU
CHLRE
VOLCA
MICSR
OSTLU
CHLVA
PHYPA
SELML
PICSI
HORVD
TRIUA
BRADI
ORYSI
ORYBR
SORBI
MAIZE
SETIT
MUSAM
VITVI
ARALL
BRARP
ARATH
9BRAS
PRUPE
POPTR
RICCO
SOYBN
MEDTR
FRAVE
CUCSA
SOLLC
SOLTU

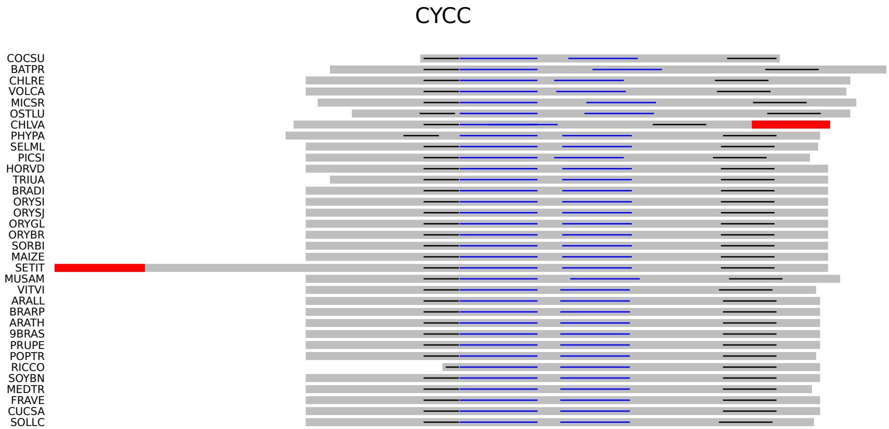
CYCC
COCSU
BATPR
CHLRE
VOLCA
MICSR
OSTLU
CHLVA
PHYPA
SELML
PICSI
HORVD
TRIUA
BRADI
ORYSI
ORYSJ
ORYGL
ORYBR
SORBI
MAIZE
SETIT
MUSAM
VITVI
ARALL
BRARP
ARATH
9BRAS
PRUPE
POPTR
RICCO
SOYBN
MEDTR
FRAVE
CUCSA
SOLLC
