# Supplementary material for: Evolution of disorder in Mediator complex and its functional relevance
Source: Nucleic Acids Res. 2015 Nov 20;44(4):1591–612. doi: 10.1093/nar/gkv1135 (PMC4770211; doi:10.1093/nar/gkv1135)
Supplement: SUPPLEMENTARY DATA [file supp_gkv1135_nar-01763-n-2015-File011.zip › Supplementary_Table_ST4-6.pdf]

Supplementary Table ST4. Number of experimentally observed PTM sites in disordered regions of Mediator subunits of yeast and human.

**Position of experimentally observed phosphorylation sites in Mediator subunits of human**			
Mediator Subunit	Number of Phosphorylation sites	Number of Phosphorylation sites in IDR	Number of Phosphorylation sites outside IDR
Med6	4	1	3
Med8	3	2	1
Med17	7	3	4
Med19	14	9	5
Med7	2	2	0
Med9	7	5	2
Med14	18	13	5
Med15	6	1	5
Med16	12	12	0
Med23	4	4	0
Med12	16	8	8
Med13	47	28	19
Med25	1	0	1
Med28	1	0	1
Med1	81	81	0
**Position of experimentally observed phosphorylation sites in Mediator subunits of Yeast**			
Mediator Subunit	Number of Phosphorylation sites	Number of Phosphorylation sites in IDR	Number of Phosphorylation sites outside IDR
Med6	8	8	0
Med17	2	2	0
Med18	1	1	0
Med19	8	8	0
Med4	10	6	4
Med9	2	0	2
Med31	1	0	1
Med3	5	5	0
Med14	6	6	0
Med15	40	26	14
Med1	15	11	4

**Position of experimentally observed acetylation sites in Mediator subunits of human**			
Mediator Subunit	Number of acetylation sites	Number of acetylation sites in IDR	Number of acetylation sites outside IDR
Med6	2	2	0
Med8	2	2	0
Med20	2	0	2
Med15	1	1	0
Med12	3	2	1
CDK8	1	1	0
Med25	1	1	0
Med28	1	0	1
Med1	19	18	1

Supplementary Table ST5: Statistics of the homology modelling of AtMed7, AtMed21 and AtMed31

**Tool used**	Statistics	AtMed7	AtMed21	AtMed31
	Template PDB ID	1YKH	1YKH	3FBI
**CE-MC[1]**	Sequence similarity (%)	60.00	32.73	55.56
	Sequence identity (%)	35.56	11.82	39.51
	Score	270	235.62	222.42
	Z-score	6.11	5.86	5.73
**ProSA-web[2]**	Z-score	-2.87	-2.73	-3.87
**SWISS-MODEL[3,4]**	D-fire energy	-86.31	-110.24	-112.06
	C_beta interaction energy	-14.7	-12.65	-52.72
	All-atom pairwise energy	-1609.09	-1717.14	-2755.91
	Solvation energy	-0.27	-2.63	1.72
	Torsion angle energy	-2.14	-9.4	-6.87
	Secondary structure agreement (%)	90.00	95.00	80.20
	Solvent accessibility agreement (%)	53.30	65.50	64.20
	QMEAN6 score	0.359	0.547	0.513
	Z-score	-3.21	-2.17	-1.71

Supplementary Table ST6: Statistics of the homology modelling of KIX variants with double mutation.

**Tool used**		I64Y, D68K	I64A, D68A	I64G, D68G	I64M, D68K	I64A, D68S	I64P,D 68P
	Template Protein PDB ID	2GUT	2GUT	2GUT	2GUT	2GUT	2GUT
**CE-MC[1]**	Sequence similarity (%)	97.10	97.10	97.10	98.55	97.10	97.10
	Sequence identity (%)	97.10	97.10	97.10	97.10	97.10	97.10
	Score	153.98	149.92	155.01	148.12	150.39	140.48
	Z-score	5.46	5.46	5.46	5.33	5.46	5.33
**ProSA-web[2]**	Z-score	-4.59	-4.77	-4.7	-4.6	-4.97	-4.38
**SWISS-MODEL[3,4]**	D-fire energy	-76.78	-40.96	-73.61	-74.89	-75.69	-75.34
	C_beta interaction energy	-36.32	-39.28	-35.25	-40.24	-40.58	-41.36
	All-atom pairwise energy	-2353.13	-1961.16	-2350.21	-2485.69	-2390.61	-2414.87
	Solvation energy	-8.73	-6.43	-7.69	-7.35	-8.5	-6.87
	Torsion angle energy	-7.22	-3.69	-5.19	-8.13	-7.88	-8.39
	Secondary structure agreement (%)	97.30	97.30	95.90	95.90	95.90	95.90
	Solvent accessibility agreement (%)	74.30	79.70	81.10	75.70	81.10	78.40
	QMEAN6 score	0.697	0.753	0.787	0.73	0.802	0.771
	Z-score	-0.21	0.18	0.41	0.02	0.51	0.3

References:

1. Guda,C., Lu,S., Scheeff,E.D., Bourne,P.E. and Shindyalov,I.N. (2004) CE-MC: a multiple protein structure alignment server. Nucleic Acids Res., 32, W100–W103. 9
2. Wiederstein,M. and Sippl,M.J. (2007) ProSA-web: interactive web service for the recognition of errors in three-dimensional structures of proteins. Nucleic Acids Res., 35, W407–W410.
3. http://swissmodel.expasy.org
4. Benkert P, Kunzli M, Schwede T. QMEAN server for protein model quality estimation. Nucleic Acids Res. 2009;37:W510-W514.
