# Supplementary material for: Evolution of disorder in Mediator complex and its functional relevance
Source: Nucleic Acids Res. 2015 Nov 20;44(4):1591–612. doi: 10.1093/nar/gkv1135 (PMC4770211; doi:10.1093/nar/gkv1135)
Supplement: SUPPLEMENTARY DATA [file supp_gkv1135_nar-01763-n-2015-File011.zip › SF_6.pdf]

This file contains a schematic of the Intrinsically disordered regions (IDRs) in Med8 of chosen metazoans, plants and fungi. The length of IDR varies between metazoans, plants and fungi. List of organisms used in the current study are present in supplementary table ST1.

MED8

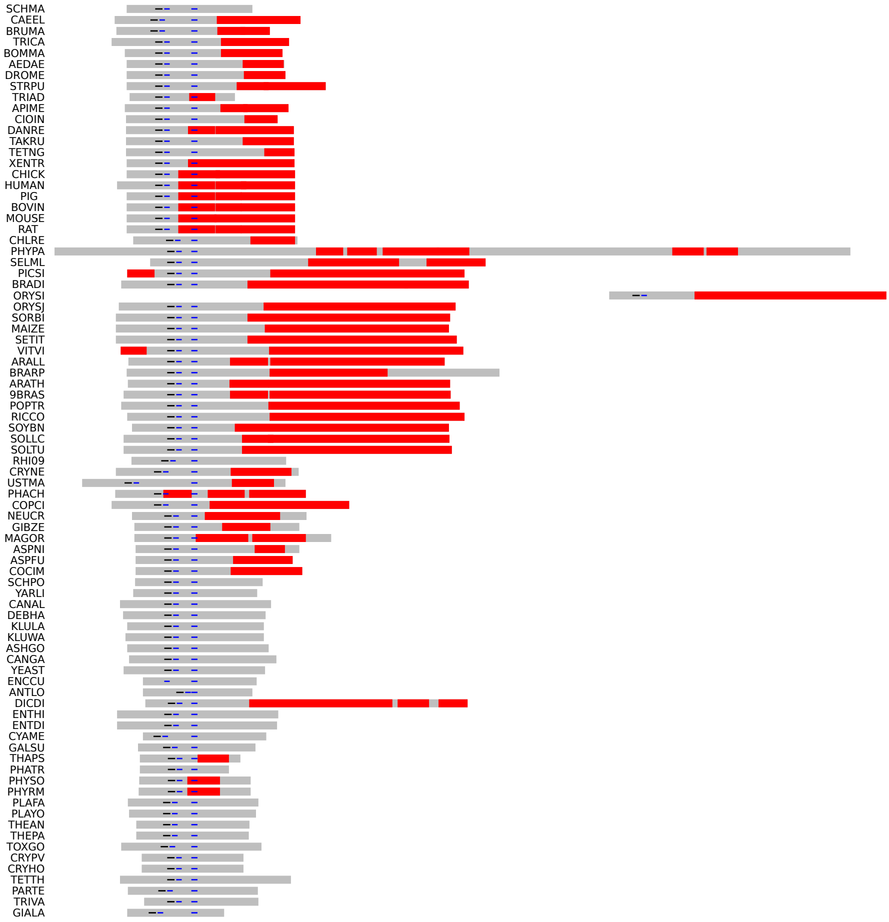
SCHMA
CAEEL
BRUMA
TRICA
BOMMA
AEDAE
DROME
STRPU
TRIAD
APIME
CIOIN
DANRE
TAKRU
TETNG
XENTR
CHICK
HUMAN
PIG
BOVIN
MOUSE
RAT
CHLRE
PHYPA
SELML
PICSI
BRADI
ORYSI
ORYSJ
SORBI
MAIZE
SETIT
VITVI
ARALL
BRARP
ARATH
9BRAS
POPTR
RICCO
SOYBN
SOLLC
SOLTU
RHI09
CRYNE
USTMA
PHACH
COPCI
NEUCR
GIBZE
MAGOR
ASPNI
ASPFU
COCIM
SCHPO
YARLI
CANAL
DEBHA
KLULA
KLUWA
ASHGO
CANGA
YEAST
ENCCU
ANTLO
DICDI
ENTHI
ENTDI
CYAME
GALSU
THAPS
PHATR
PHYSO
PHYRM
PLAFA
PLAYO
THEAN
THEPA
TOXGO
CRYPV
CRYHO
TETTH
PARTE
TRIVA
GIALA
