# Supplementary material for: Evolution of disorder in Mediator complex and its functional relevance
Source: Nucleic Acids Res. 2015 Nov 20;44(4):1591–612. doi: 10.1093/nar/gkv1135 (PMC4770211; doi:10.1093/nar/gkv1135)
Supplement: SUPPLEMENTARY DATA [file supp_gkv1135_nar-01763-n-2015-File011.zip › SF_7.pdf]

Number of phosphorylation and acetylation sites in the Mediator complex subunits of eight model organisms *S.cerevisiae* (YEAST), *A.thaliana* (ARATH), *O.sativa subsp. Japonica* (ORYSJ), *C.elegans* (CAEEL), *D.melanogaster* (DROME), *D.rerio* (DANRE), *G.gallus* (CHICK) and *H.sapiens* (HUMAN). Here 'conserved subunits' implies subunits present in the three major kingdoms.

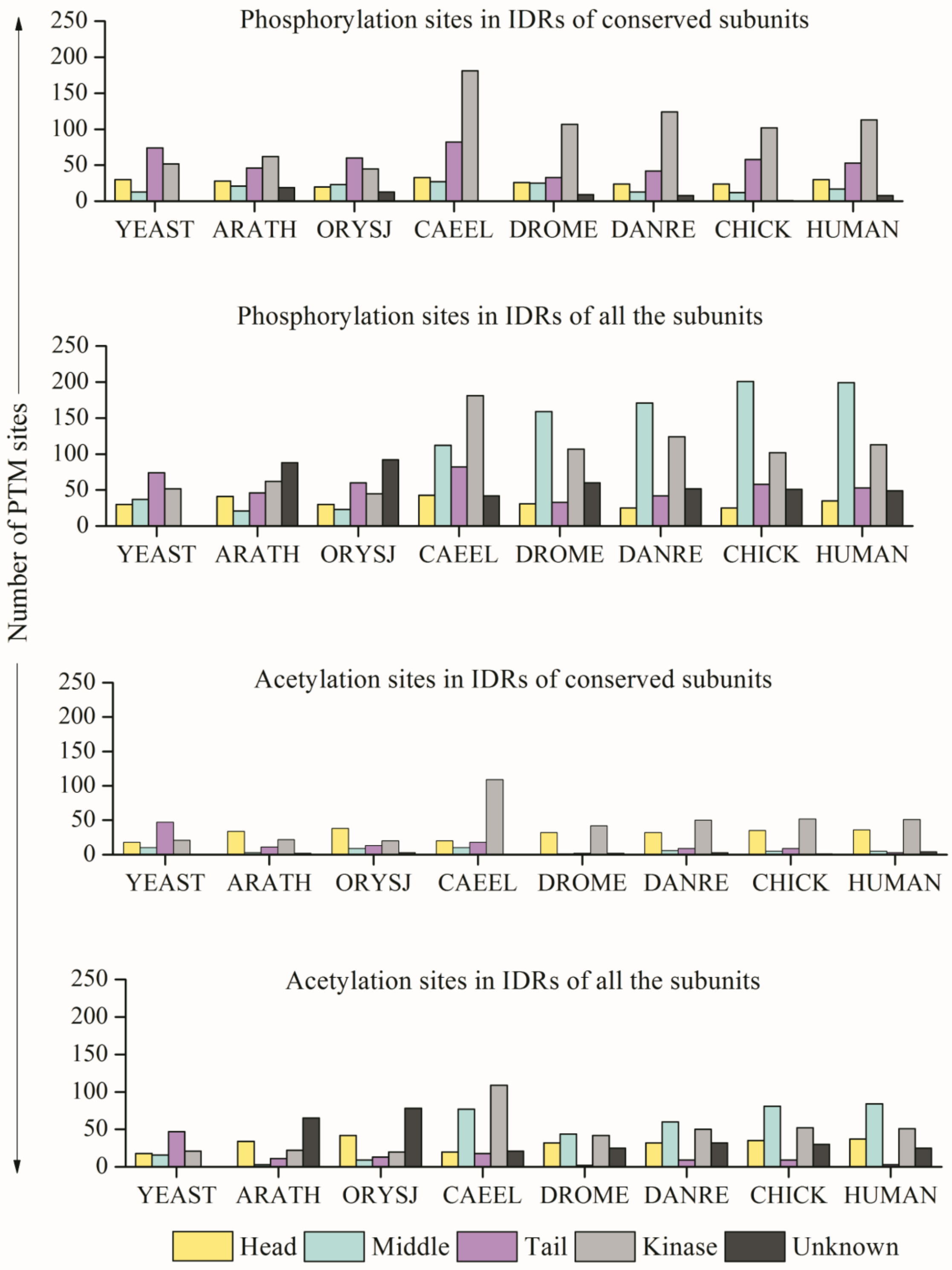

Phosphorylation sites in IDRs of conserved subunits
Phosphorylation sites in IDRs of all the subunits
Acetylation sites in IDRs of conserved subunits
Acetylation sites in IDRs of all the subunits
Number of PTM sites
0
50
100
150
200
250
YEAST
ARATH
ORYSJ
CAEEL
DROME
DANRE
CHICK
HUMAN
Head
Middle
Tail
Kinase
Unknown
