# Supplementary material for: Evolution of disorder in Mediator complex and its functional relevance
Source: Nucleic Acids Res. 2015 Nov 20;44(4):1591–612. doi: 10.1093/nar/gkv1135 (PMC4770211; doi:10.1093/nar/gkv1135)
Supplement: SUPPLEMENTARY DATA [file supp_gkv1135_nar-01763-n-2015-File011.zip › SF_8,11-13.pdf]

Interaction of AtMed4 and AtMed9 with other randomly selected Med subunits

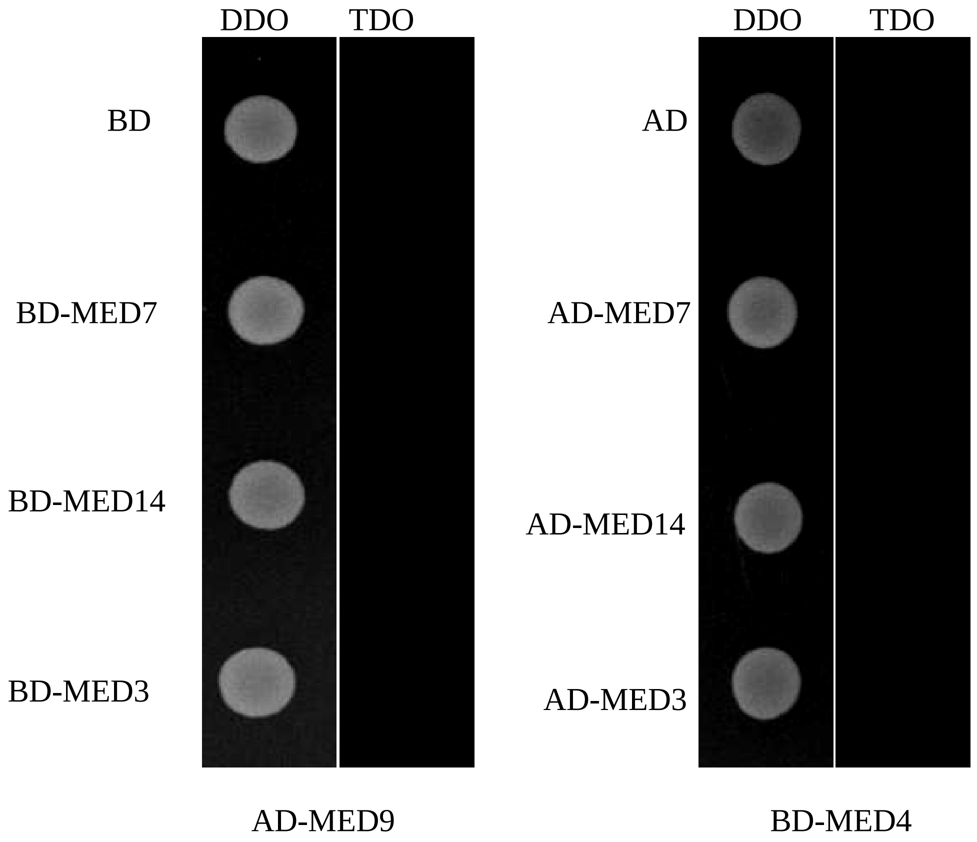


**Supplementary Figure S8.** Yeast two-hybrid assay of AtMed4 and AtMed9 with randomly selected subunits.

# CBP-KIX structures generated from PDB

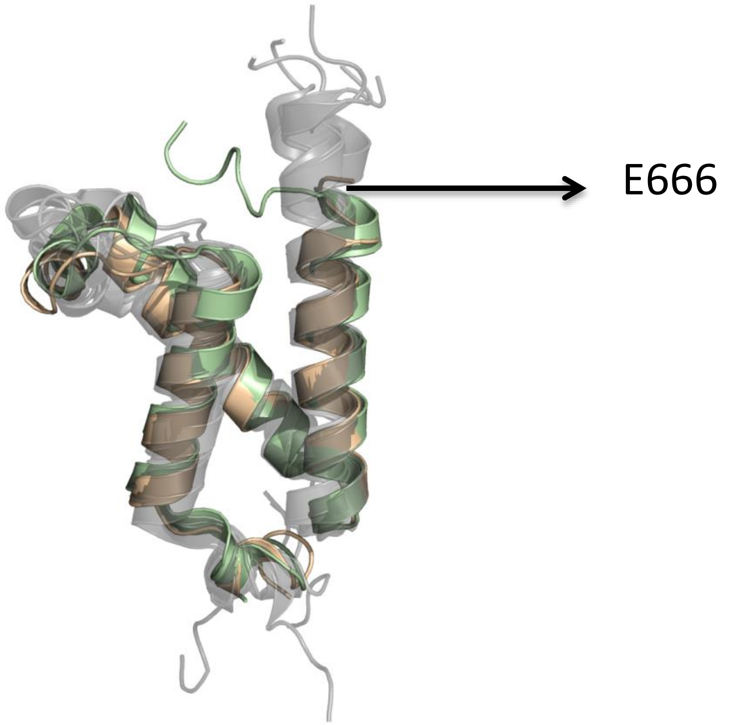


**Wheat-** 1KDX, short variant of CBP-KIX (587-666)
**Green**- 1SB0, long variant (587-673) interacting with one TAD at MYB site only. Note the disordered region starting at residue 666 in this case.
**Gray helices –** 2LQH, 2LQI, 2LXS, 2LXT, 2KWF, long variants interacting with two TADs, one each in MYB and MLL sites.

**Supplementary Figure S11.** Structure of CBP KIX domain stabilized by diverse ligands (TADs). Different structures were obtained from PDB database and aligned to see the differences. Please note that the unstable carboxyl end of third helix is stabilized in ligand-bound complex. Partially stabilized carboxyl end of third helix after binding to one TAD at Myb site (green structure) is further stabilized by binding to second TAD at MLL site (grey structure) highlighting the importance of carboxyl end in protein-protein interaction.

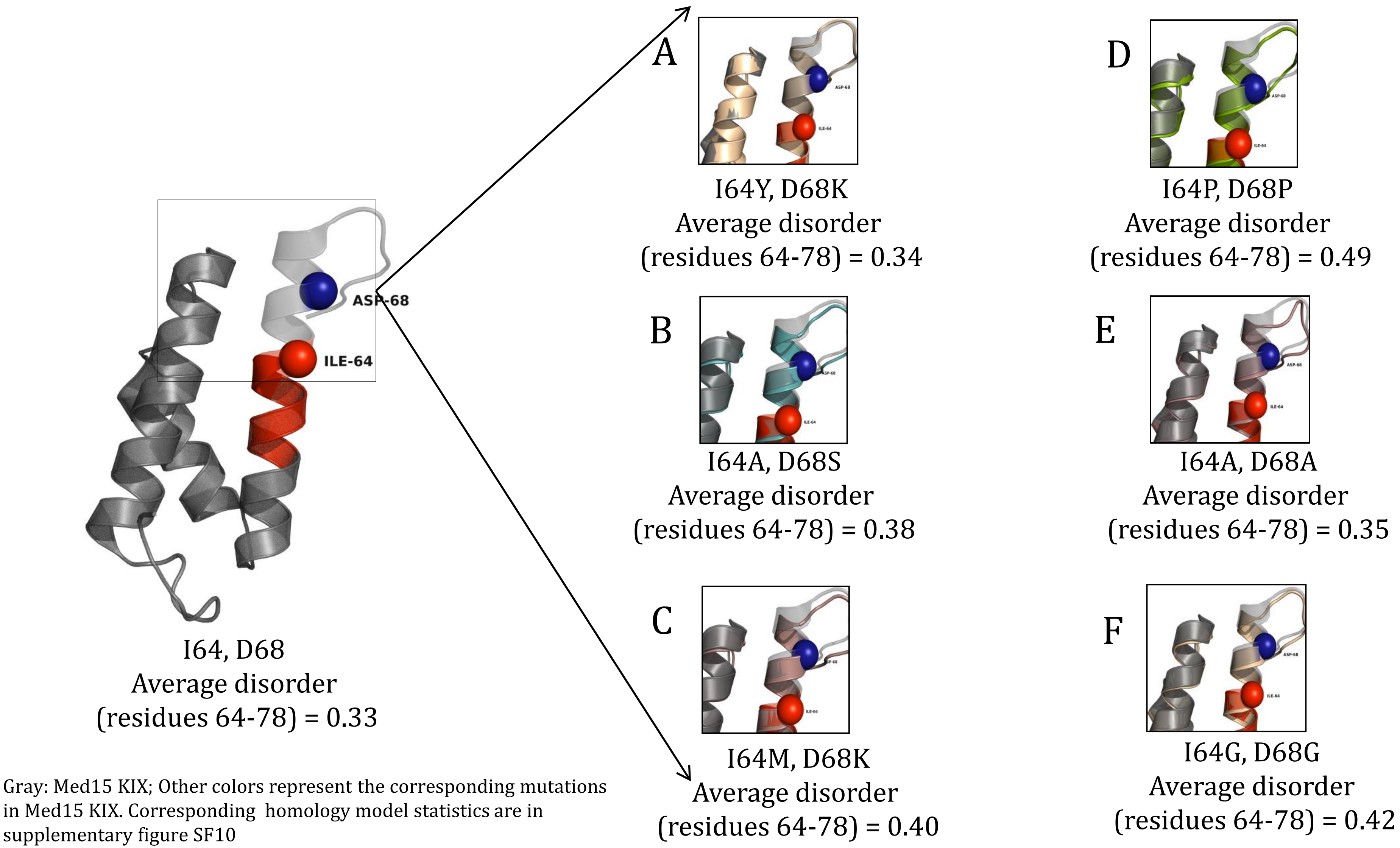


**Supplementary Figure S12.** Homology models of double mutated human Med15 KIX domain

# Interacting partners of 'hubs' in Human Mediator complex subunits

## Med4

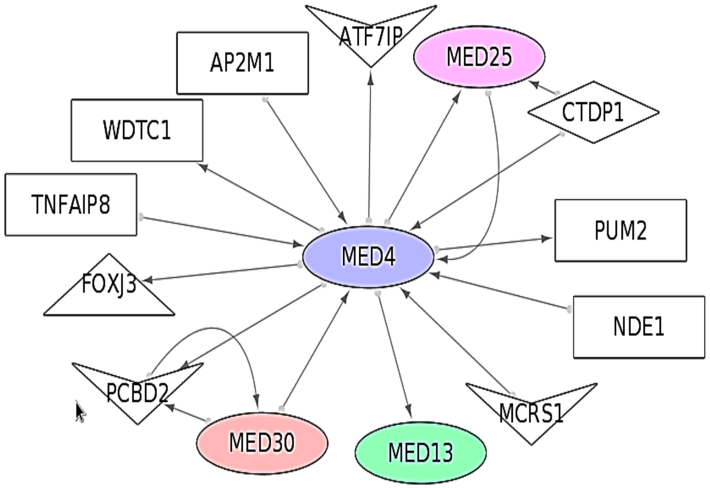


## Med5/Med24

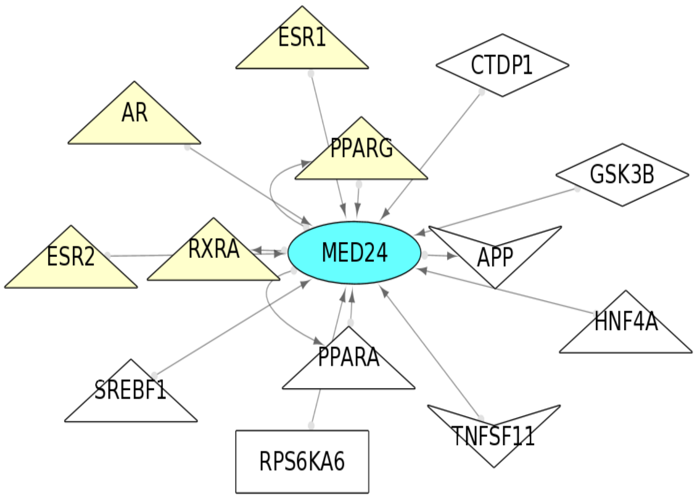


## Med6

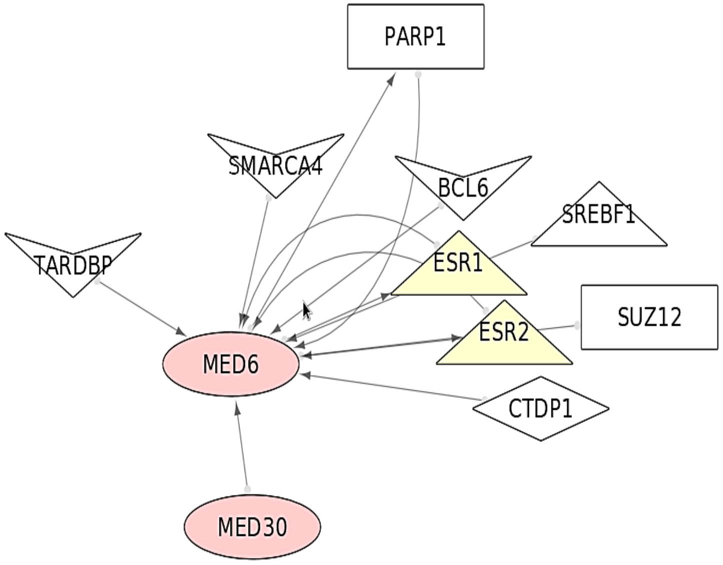


## Med7

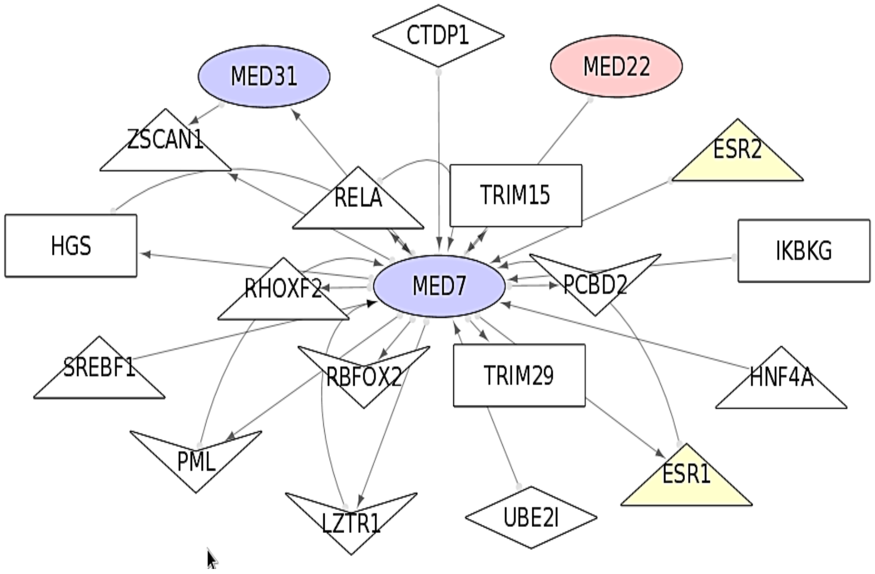

# Interacting partners of 'hubs' in Human Mediator complex subunits

## Med8

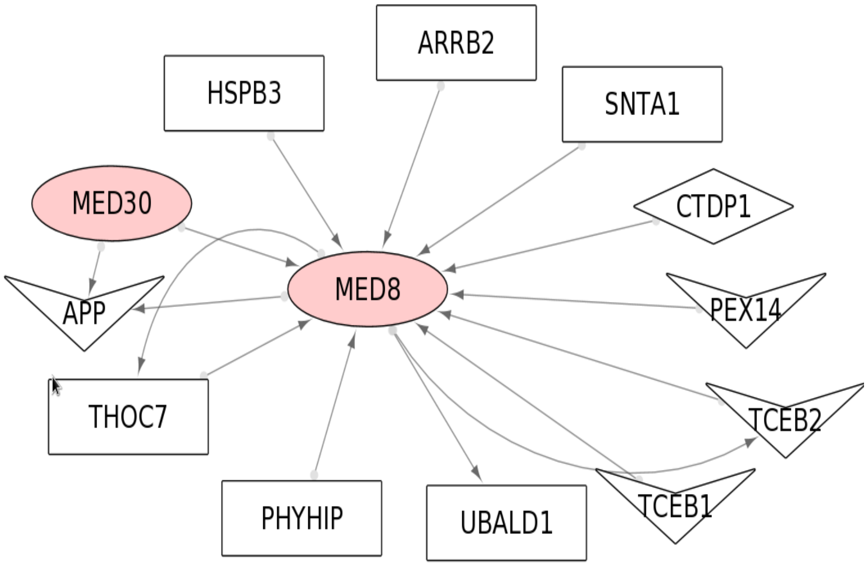


## Med14

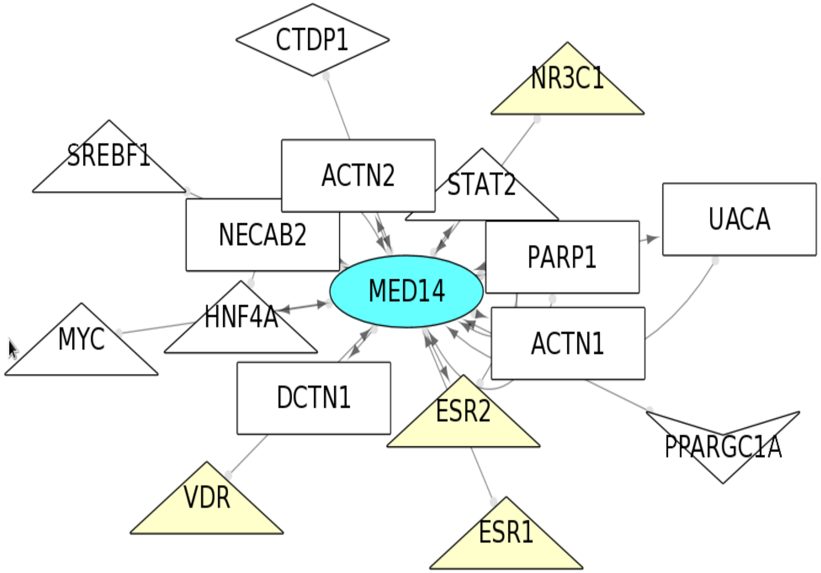


## Med15

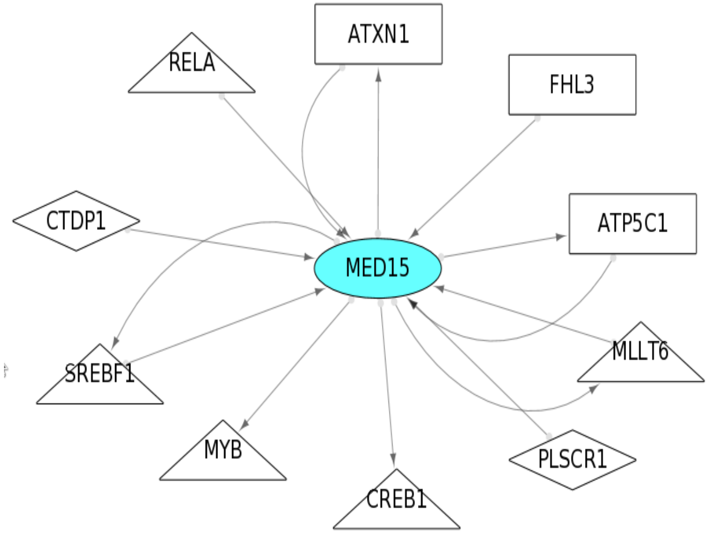


## Med17

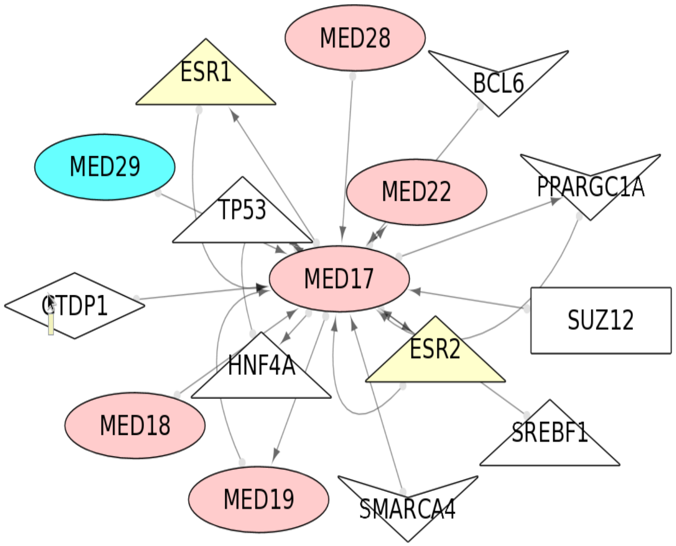

Interacting partners of ‘hubs’ in Human Mediator complex subunits

Med19

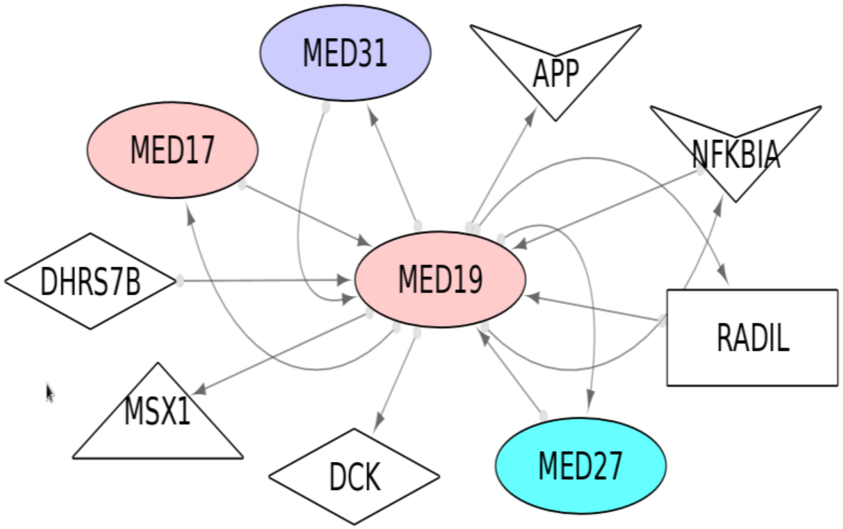


Med21

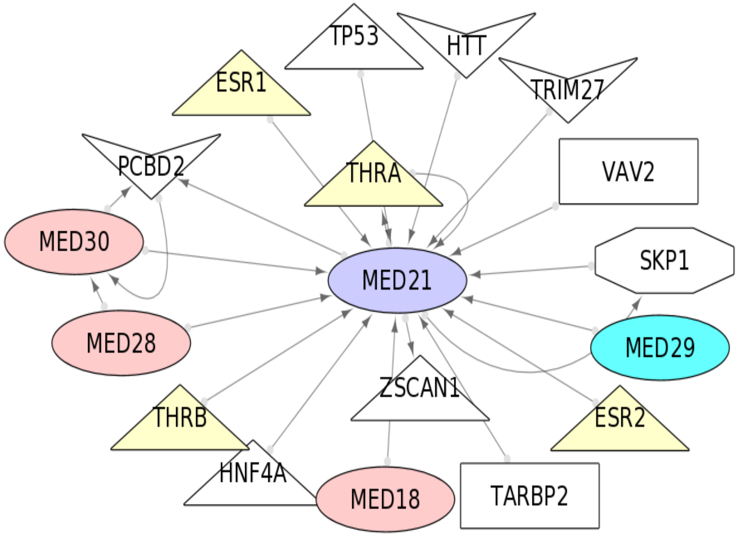


Med23

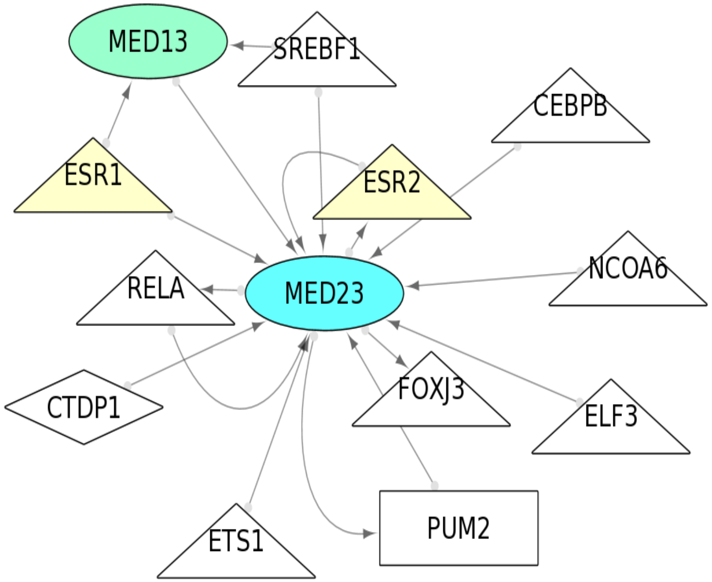


Med25

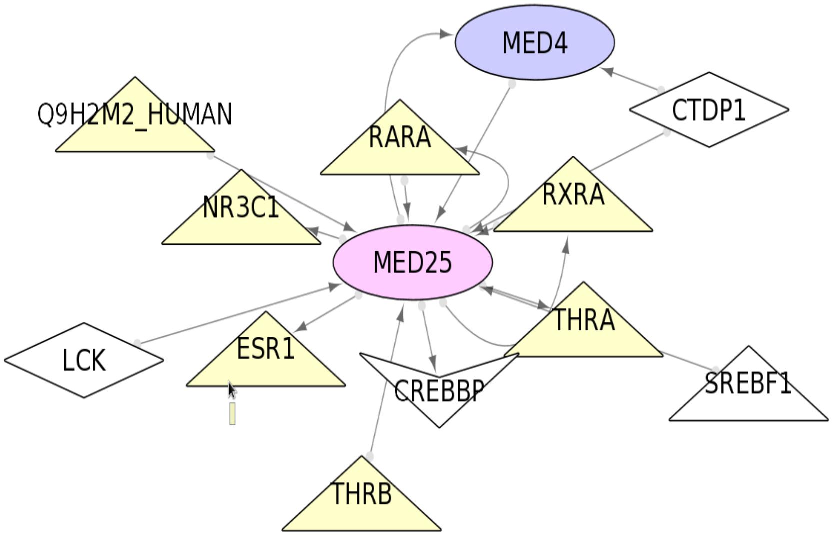

# Interacting partners of 'hubs' in Human Mediator complex subunits

## Med28

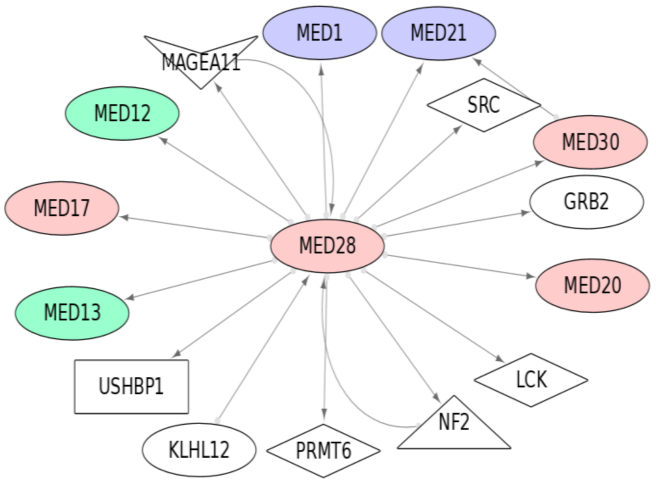


## Med30

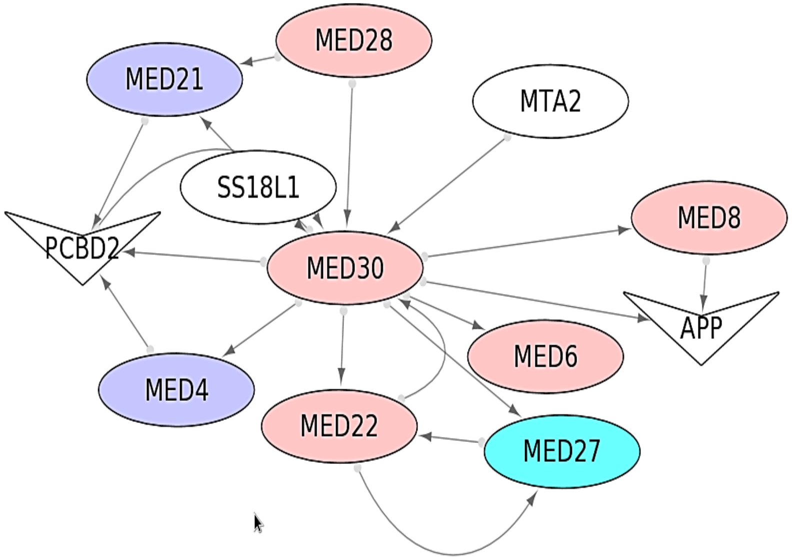


## Cdk8

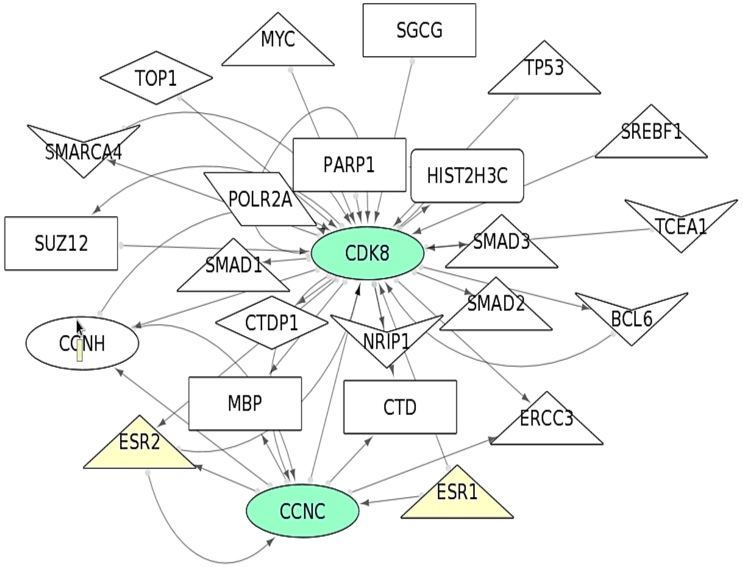


## CycC

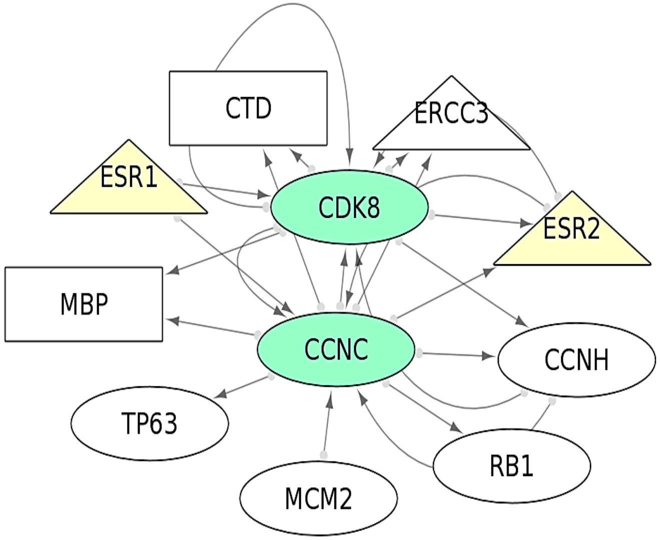

# Interacting partners of 'hubs' Yeast Mediator complex subunits

## Med1

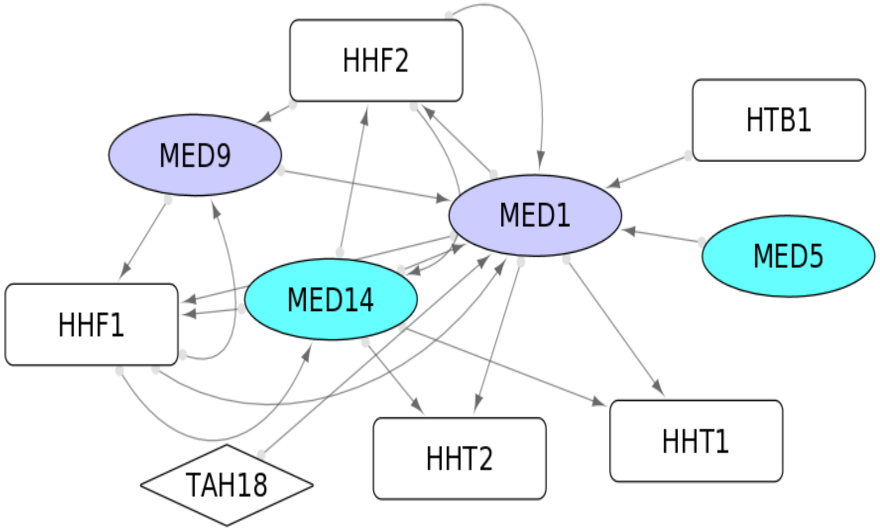


## Med3

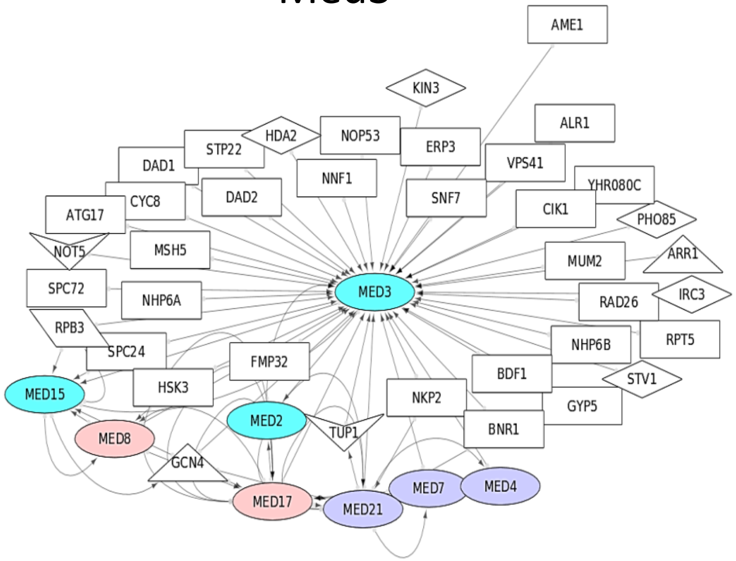


## Med4

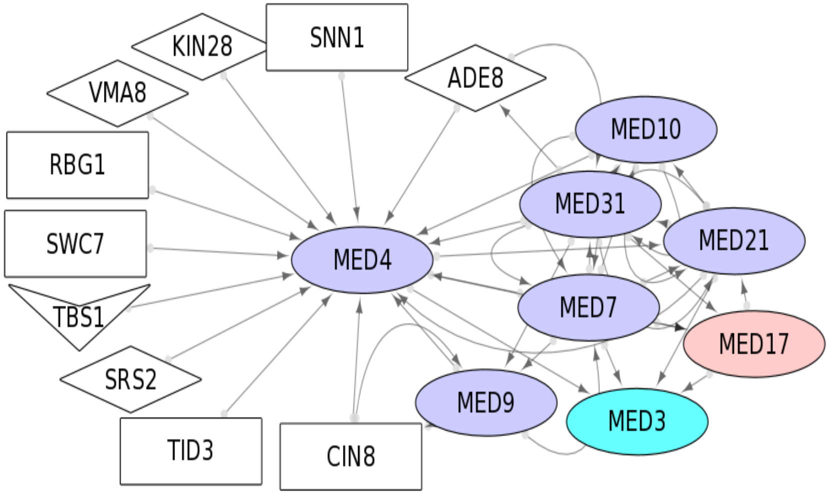


## Med6

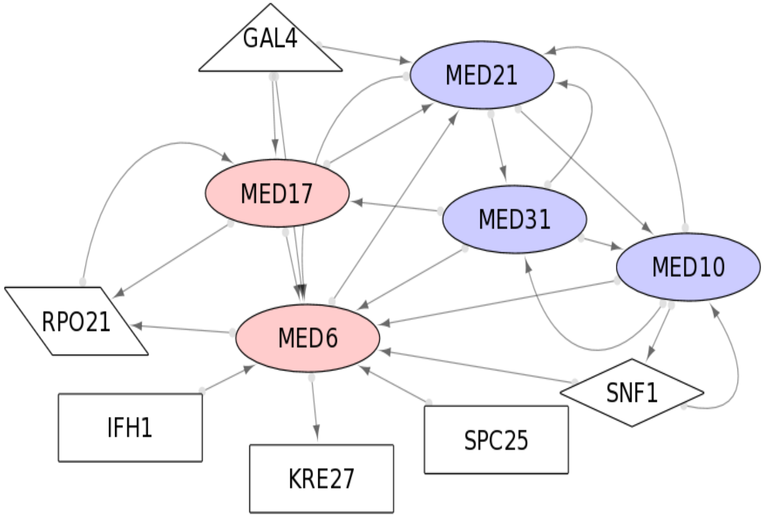

# Interacting partners of ‘hubs’ Yeast Mediator complex subunits

## Med7

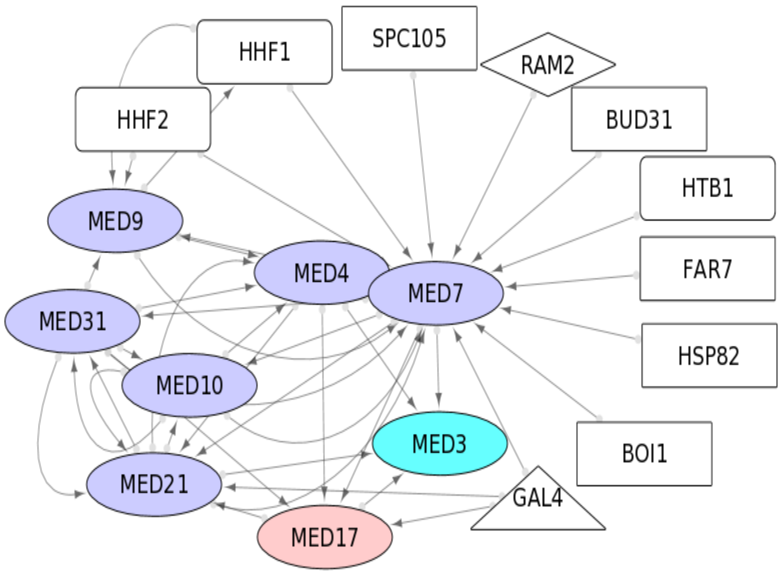


## Med8

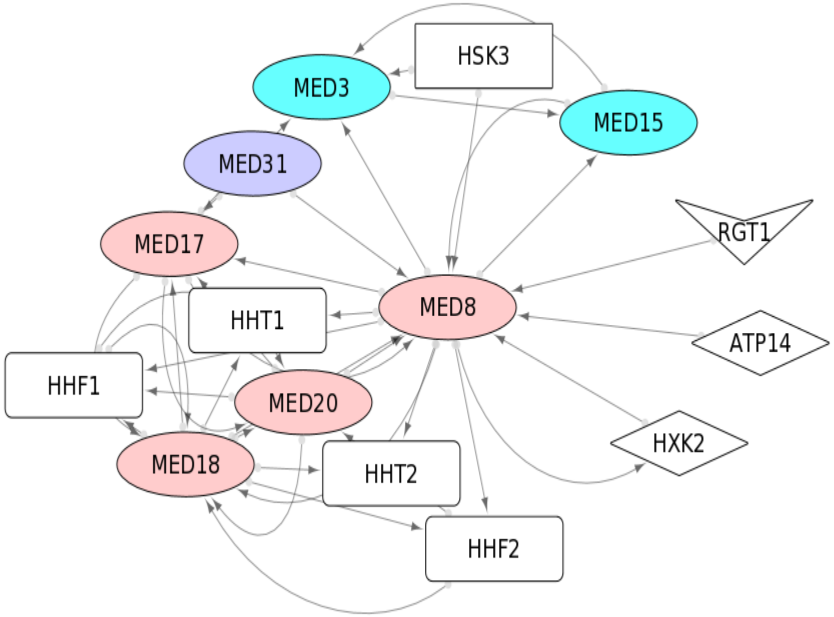


## Med9

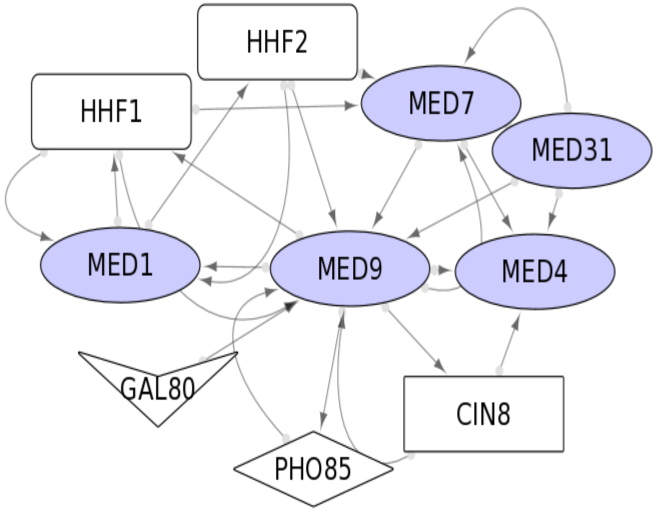


## Med10

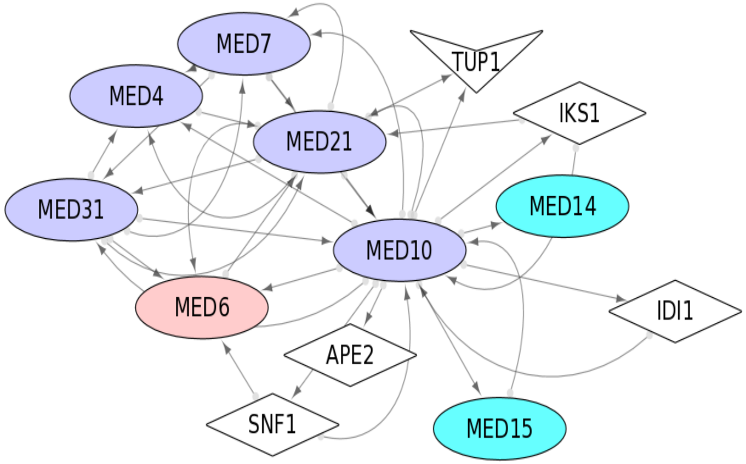

# Interacting partners of 'hubs' Yeast Mediator complex subunits

## Med13

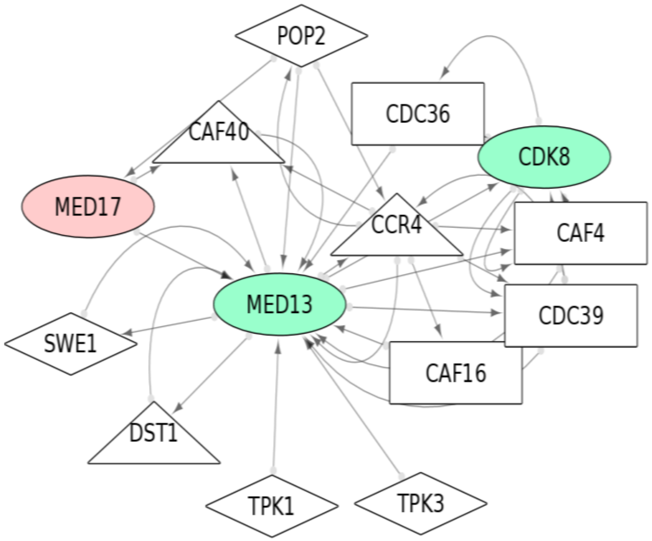


## Med14

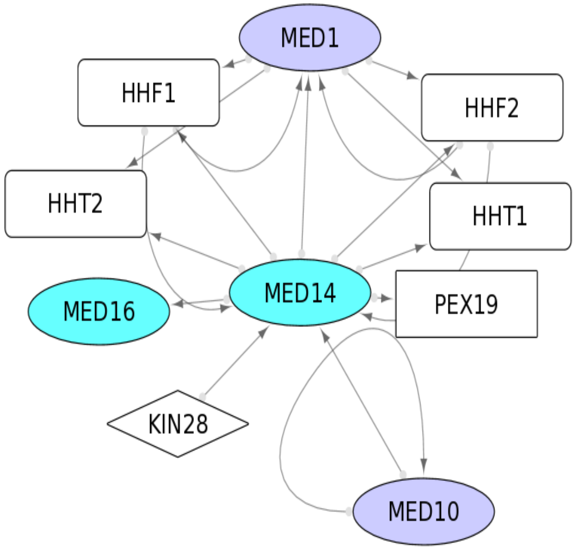


## Med15

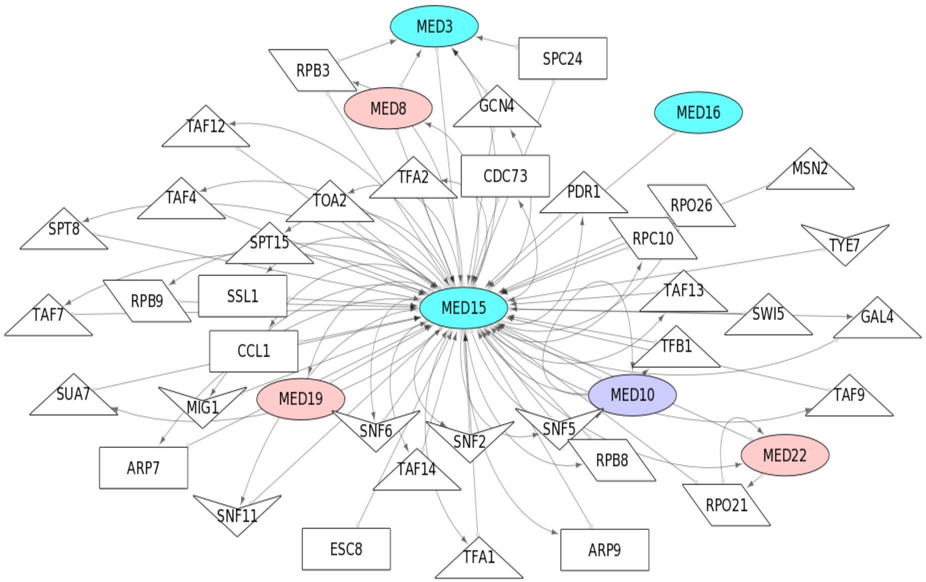


## Med16

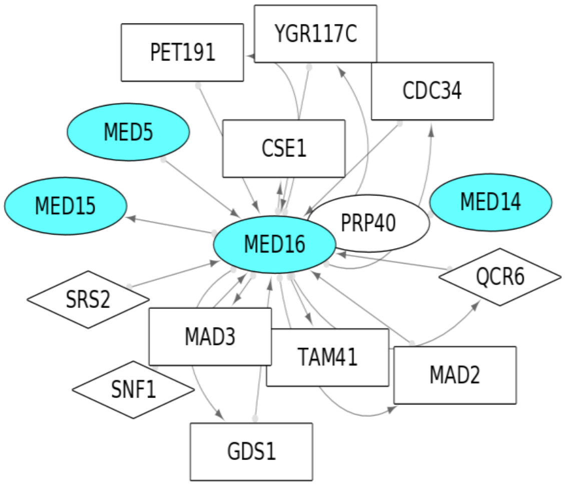

# Interacting partners of ‘hubs’ Yeast Mediator complex subunits

## Med18

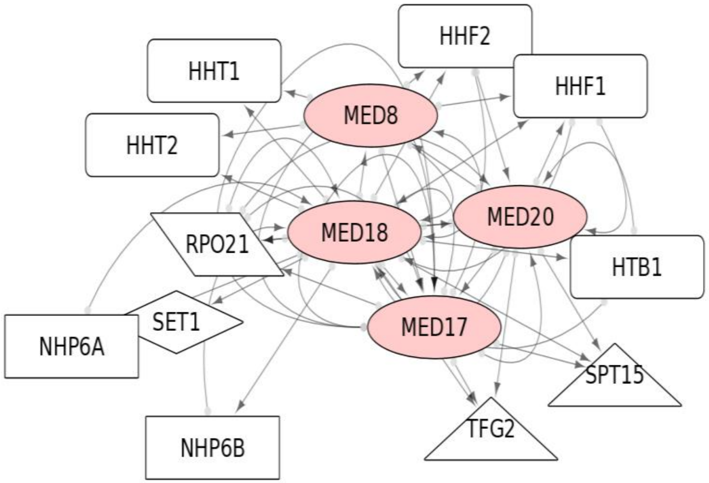


## Med20

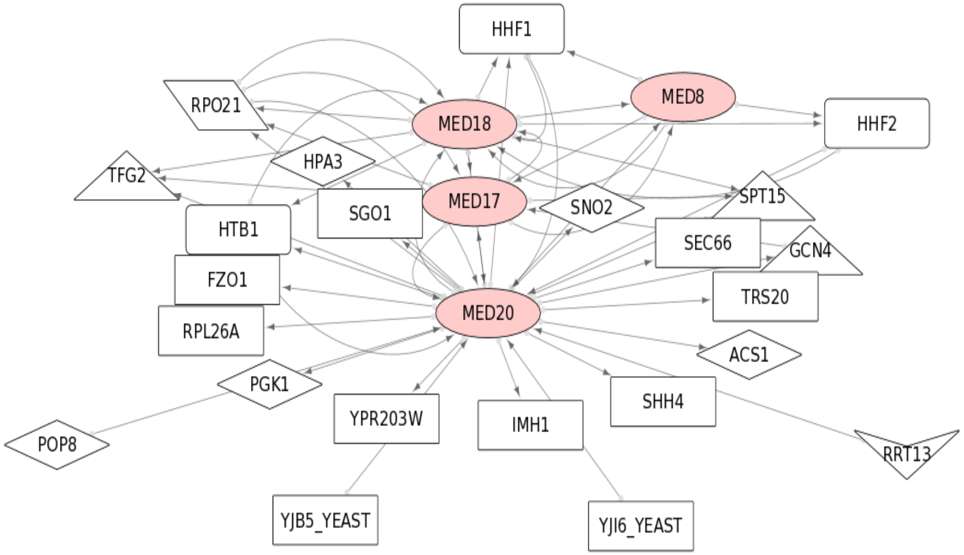


## Med21

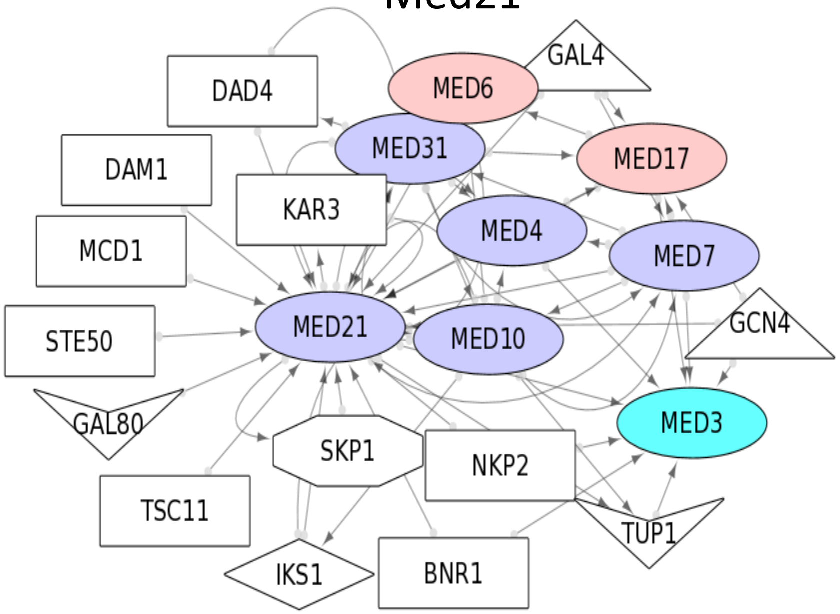


## Med22

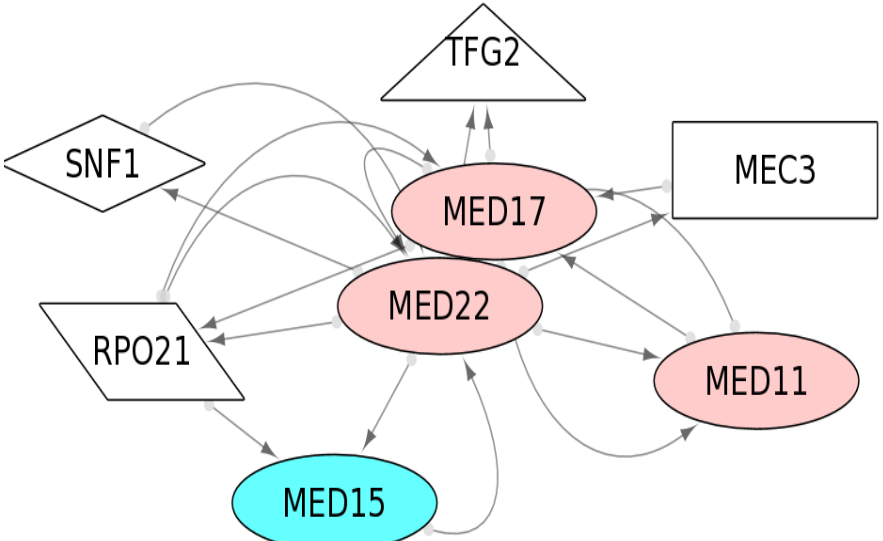

Interacting partners of ‘hubs’ Yeast Mediator complex subunits

CycC

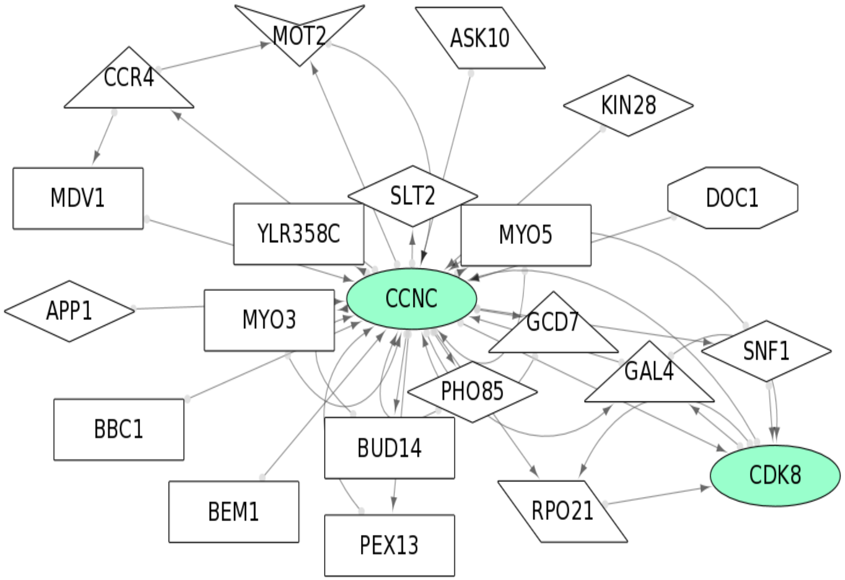


Cdk8

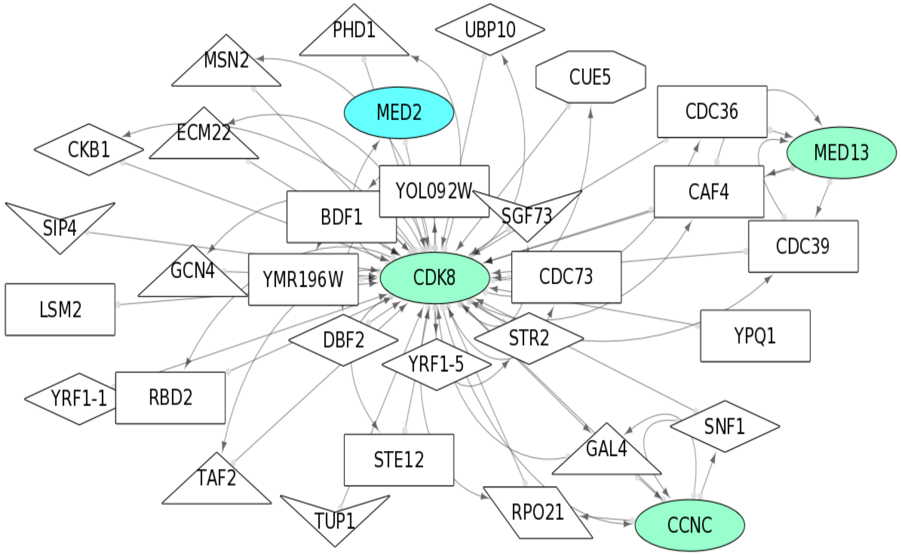


**Supplementary Figure S13.** Interacting partners of ‘hubs’ in human and yeast Mediator complex subunits.
