# Supplementary material for: Evolution of disorder in Mediator complex and its functional relevance
Source: Nucleic Acids Res. 2015 Nov 20;44(4):1591–612. doi: 10.1093/nar/gkv1135 (PMC4770211; doi:10.1093/nar/gkv1135)
Supplement: SUPPLEMENTARY DATA [file SF_9.pdf › SF_9.pdf]

This file contains Molecular Recognition Features (MoRFs) predicted for all the subunits in 30 selected model organisms. Amino acids belonging to the Intrinsically Disordered Regions (IDRs) are colored in red. MoRFs are highlighted in green and amino acid repeats are highlighted in yellow.

List of model organisms used are :

HUMAN	Homo sapiens
CHICK	Gallus gallus
XENTR	Xenopus tropicalis
DANRE	Danio rerio
CIOIN	Ciona intestinalis
ANOGA	Anopheles gambiae
TETNG	Tetraodon nigroviridis
DROME	Drosophila melanogaster
BRUMA	Brugia malayi
CAEEL	Caenorhabditis elegans
YEAST	Saccharomyces cerevisiae
SCHPO	Schizosaccharomyces pombe
CANAL	Candida albicans
CANGA	Candida glabrata
ASPNI	Aspergillus nidulans
RHIO9	Rhizopus oryzae
CRYNE	Cryptococcus neoformans
USTMA	Ustilago maydis
ENCCU	Encephalitozoon cuniculi
ANTLO	Antonospora locustae
ARATH	Arabidopsis thaliana
VITVI	Vitis vinifera
SOYBN	Glycine max
SOLLC	Solanum lycopersicum
ORYSJ	Oryza sativa subsp. japonica
HORVU	Hordeum vulgare var. distichum
SORBI	Sorghum bicolor
SELML	Selaginella moellendorffii
PHYPA	Physcomitrella patens subsp. patens
VOLCA	Volvox carteri

MED1

```
               1                                                50
HUMAN*  --------------------------------------------------
CHICK   --------------------------------------------------
XENTR   --------------------------------------------------
DANRE   --------------------------------------------------
CIOIN   --------------------------------------------------
ANOGA   MSASGTAAVSGGSLPALLMANGGGGGGSGGPGGGPGGGKGIGKPDIKPPG
TETNG   --------------------------------------------------
DROME   MS------------------------------------------------
BRUMA   -----------------------------------------MEKEDIMER
CAEEL   --------------------------------------------MIIEER
YEAST*  MVEG----------------------------------------------
SCHPO   --------------------------------------------------
CANAL   MP------------------------------------------------
CANGA   MA------------------------------------------------
ASPNI   MA---------------------TPSSKPNPGATPTHLTSSPRPSGGPMH
RHIO9   MS------------------------------------------------
CRYNE   --------------------------------------------MAQPSP
USTMA   ----------------------------------------MATDTTIDSL
                 10        20        30        40        50
```

```
HUMAN*  ----------MKA-QGETEESEKLSKMSSLLERLHAKFN--QN-------
CHICK   ----------MKAVPGGAEEAEKLNKMSTLLERLHAKYN--QN-------
XENTR   --------------------------MSSLLERLHSKYS--QN-------
DANRE   -------------------ESEKQSRIMTLLERLYCKHS--SS-------
CIOIN   ---MGLDWETKSSRMLDEESSREGESSEELMEMLRKKNET----------
ANOGA   GGGPSAGGTGTAGKVLLGGDKHETWQMELLMERLRTKAKSAQY-------
TETNG   -------------------EEEKESRSAVLLERLHAKYI--AS-------
DROME   GSNAKSSGTGFGSHIPSIEEKNKQIQQETMMEKLRAKYRN-KP-------
BRUMA   QEQKPVMNSAQCDKYSAVDLSLEEVEEEIRLEKIRQTS---LK-------
CAEEL   KNH---------------ELNLESIDRDLRMENVRQSA---EK-------
YEAST*  --------------------------------------------------
SCHPO   ------------------------MDRPHLFSEVHFRRNASNP-------
CANAL   --------------------------------------------------
CANGA   --------------------------------------------------
ASPNI   RAMSLKSPSTRTPSASGHGQLNQPASTQQYATPLAVTAENDNA-------
RHIO9   --------KSTDPSKNSISSAIFGLQDTLQFHALGPVNLDKAR-------
CRYNE   SAILGQQPFPIDPAPAPAQPNPIPTSTPTLLTYLHALLDVRQSQNLTLPY
USTMA   PVTGDGAAEALTATVLVASTPGAEAQTSALFDPLHQAKGSLQL-------
                 60        70        80        90       100
```

```
HUMAN*  -RPWS------------------------------ETIKLVRQ-VMEKRV
CHICK   -RPWT------------------------------ETMKLVRQ-VMEKRV
XENTR   -RPWP------------------------------ETIKLVRQ-IMEKRT
DANRE   -RSWQ------------------------------ETSKVIRQ-AMEKRG
CIOIN   -KSFD------------------------------DIIKLLHVVSSEQKA
ANOGA   -KSFQ------------------------------EMSKSVRMSLLEKRY
TETNG   -RPWQ------------------------------ETFKVVRQ-TMDKRG
DROME   -KSYE------------------------------EIKKSVRMYFIEKHY
BRUMA   -FSWD------------------------------YLSQLLRRNLMEKRF
CAEEL   -LGWE------------------------------HFARSVRRNLLEKRQ
YEAST*  -DSYV---------------------------------------------
SCHPO   -----------------------------------NQINWTR--------
CANAL   --------------------------------------------------
CANGA   -DQYV---------------------------------------------
ASPNI   -ISFSSPSALLLGAYPGISPSPAVHDALVGTGMNDSDIQALGMPGLKLGA
RHIO9   -DEFS------------------------------QQINSIRNICSQFET
CRYNE   LQPFAPLSSRPLPRMNNNGQRGDGGKGKEKVKSDLDVIRELRESVASWRG
USTMA   -QTEASAELHPLNSSLQSLYSVPSADGRLSRHQYRQLIAQIRHSVSQFAA
                110       120       130       140       150
```

```
HUMAN*  VMSSGGHQ-----------------HLVSCL-------ETLQKALK----
CHICK   VMNSGGHQ-----------------HLVSCL-------ETLQKALK----
XENTR   GMMSGSHQ-----------------HLVSCL-------ETLQKALK----
DANRE   AVNTGGHQ-----------------LLLNCL-------ETLQRALK----
CIOIN   RI-----------------------MLRSCM-------DKVLMNMG----
ANOGA   ALDAVEKS-----------------NLQKTL-------DSMQYCIK----
TETNG   PTNAPSHQ-----------------LMHSCL-------ENLQRAMK----
DROME   PLDPLCKA-----------------TLQSAL-------DKLQHYIK----
BRUMA   VLSADDKE-----------------DLMQAA-------ADMRKYMP----
CAEEL   TLDARTRL-----------------DVLGSL-------VFMKEHLP----
YEAST*  -------------------------ETLDSM-------IELFKDYK----
SCHPO   -------------------------YWIDCL-----------SSVP----
CANAL   -------------------------GTKSAL------DKSLQLLYN----
CANGA   -------------------------EVLGDL-------IGLLKDYK----
ASPNI   ARDNDEERRR---------------HIIEVV-------QKLRERVA----
RHIO9   -------------------------EVLGDL---------ELPLKEK---
CRYNE   VLTKENQEGG---------------RLTTALKEVITHQSALLSSAA----
USTMA   TTPALQLPPSSPQQSNHANTANQIRRYISLLTETSTHTAALAHSIAPIKS
                160       170       180       190       200
```

MED1

```
HUMAN*  -----------------------------------------VT-SLPAMT
CHICK   -----------------------------------------VS-SLPAMT
XENTR   -----------------------------------------VS-SLSAMT
DANRE   -----------------------------------------VS-TLPSMT
CIOIN   -----------------------------------------GSYNLRDVI
ANOGA   -----------------------------------------VT-TRQGLV
TETNG   -----------------------------------------VS-SLSAMT
DROME   -----------------------------------------VT-SRHGLV
BRUMA   -----------------------------------------SD-SDRDLS
CAEEL   -----------------------------------------ID-NDATIA
YEAST*  --------------------------------------------PGSITL
SCHPO   --------------------------------------------WNDISV
CANAL   -----------------------------------------YSTNFQVSI
CANGA   --------------------------------------------PGTITI
ASPNI   -----------------------------------------GRGVSRKGI
RHIO9   -----------------------------------------EESKSKIEI
CRYNE   --------------------------ALLPQASSSTKPLFLSSHLLSQPT
USTMA   ADVDRGNTAIHPMPLVSFPQQLQVGAFRRGSSDHPTSSWSLALETLDALC
                 210       220       230       240       250

HUMAN*  DRLESIARQNGLGSH--LSASGT-----------------ECYITSDMFY
CHICK   DRLESIARQNGLGSH--LSANGT-----------------ECYITSDMFY
XENTR   DRLESIARQNGLTSH--LSPNGT-----------------ECYITTDMFY
DANRE   DRLESIARQNMLGSH--LSPSHT-----------------ECYITSDMFY
CIOIN   DRLRSVASVNGLRFNVVLDQDTS-------------SPGTTCQILADMFT
ANOGA   ERLDCLTRQ--LGLK--LSEDTS-----------------GLFISSDMFY
TETNG   DRLESIARQNMLGSH--LSPSGT-----------------ECYITSDMFY
DROME   ERLESLSRQ--LGLK--FMEDQQ-----------------LLFISTDMFY
BRUMA   THLRHLAES--LSSD--FQAHPG-----------------YYKIRNADFS
CAEEL   RKVQQLSEG--LGEHCVFSTQNS-----------------GFSIRNPDVT
YEAST*  ENITRLCQT--LGLE-SFTEELS-------------NELSRLSTASKIIV
SCHPO   TGFEYLAKK--YGLE-VFQDSSK-------------PNEVVLSLAGKIIL
CANAL   ELIQKLAQQ--LKLE-TFIDSLG----FNLDDSKPGQKTQKLSIAGSSIL
CANGA   ENITKLCQS--MGLE-SFIDELD-------------NDISRLSTASKIIV
ASPNI   ERLSHLERF-----ESIWQDD-------------------NLNIAGNFVD
RHIO9   QKLEKLAQS--MGLI-TFVDSSK--------MFESDSPTSTITLGGTLIV
CRYNE   KLLQSIASA--VSLQ-TFLEDSQ-----------FGLQQSSLAIAGERLV
USTMA   ETLETTAKA--LGLE-TFSEPSSEATEVPSDTASGSVLTHTLTLGAKILV
                 260       270       280       290       300

HUMAN*  VEVQLD------------------------------------PA---GQL
CHICK   VEVQLD------------------------------------PT---GLL
XENTR   LEVLLD------------------------------------TE---GQL
DANRE   VEVHMD------------------------------------KS---GKL
CIOIN   MEVTLD-----------------------------------ESQFGGLSV
ANOGA   LEIILD------------------------------------PAG--GTV
TETNG   VEVQLD------------------------------------NS---GQL
DROME   VEILLD------------------------------------AA---GSL
BRUMA   LEITVS-----------------------------------LDG---QQI
CAEEL   IDIGVA-----------------------------------ED-----QI
YEAST*  IDVDYN-----------------------------------KKQ---DRI
SCHPO   IDITVPIN---------------------------------------ASP
CANAL   LDIDFE------------------------------------TD---NKI
CANGA   IDIDYN-----------------------------------KTQ---STV
ASPNI   LEIDFY-----------------------------------RGQ---NVV
RHIO9   IDIDID------------------------------------DK---GKV
CRYNE   VDVDLGVDLGGEEDESRMGTPMGTPRPYTPVPGAMPTSALTQNSDERGKV
USTMA   IDIELHIVKTGT----------------------------VEGF---RPK
                 310       320       330       340       350

HUMAN*  CDVKV--A-------HHGE-NPVSCPEL----------------------
CHICK   CDVKV--A-------HHGE-NPVSCPEL----------------------
XENTR   CDVKV--A-------HHRE-NPVSCPEL----------------------
DANRE   MDVKV--A-------HHGE-NPVSCPEL----------------------
CIOIN   LDVKIVYS-----------DHVKSCPNM----------------------
ANOGA   QDVKV--H-------HECKMKQQSCSEL----------------------
TETNG   VDVKV--A-------HQGE-NPTSCPEL----------------------
DROME   SDVKV--H-------HECKIEQQS-SEL----------------------
BRUMA   TSCSV--S-------WFGE-RAVEAQAL----------------------
CAEEL   STCKI--G-------YFGQ-PLTDAPGA----------------------
YEAST*  QDVKLVLASNFDNFDYFNQ--RDGEHEK----------------------
SCHPO   KDINVVLA-------FANASGEQYNNPV----------------------
CANAL   VGLSLSVN--------AGDSLEQQNNHREIGPPNHTTNGQSSSHISIVTD
CANGA   QDVKLVLASNFDNFDYSNEQLEEAGDKQ----------------------
ASPNI   RDVSLKYA-------TPDTADGERREEA----------------------
RHIO9   QKTKVT---------YVSESMQSDQDDR----------------------
CRYNE   KLLKLVAS-------HVTPSGGTGQCEW----------------------
USTMA   TKLKLSYA-------TDSVDSQQTRDPR----------------------
                 360       370       380       390       400
```

MED1

HUMAN*	-----------------------------VQQLRE--------------K
CHICK	-----------------------------VQHLRE--------------K
XENTR	-----------------------------VEQLRE--------------K
DANRE	-----------------------------IQHLRD--------------K
CIOIN	-----------------------------VQIIRE--------------G
ANOGA	-----------------------------VACLQR--------------G
TETNG	-----------------------------IQHLRE--------------K
DROME	-----------------------------VACLKS--------------G
BRUMA	-----------------------------RLYVSR--------------N
CAEEL	-----------------------------LALMKN--------------G
YEAST*	-----------------------------SNILLNSLTKY---------P
SCHPO	-----------------------------AEKLLKDAIYN---------C
CANAL	ELGIHHVKLNCNNNPISFLNKPNDLNKTQVEQILLRNLQA---------S
CANGA	-----------------------------SNILLNSLTNY---------D
ASPNI	-----------------------------TAILKRDLVQTPEDGARGDWK
RHIO9	-----------------------------VDKILTENLQL---------R
CRYNE	-----------------------------VRKVLNELVNDY-------LA
USTMA	-----------------------------LGAVLERDVEL---------I
	410 420 430 440 450

HUMAN*	NFDEF----------------------SKHLKGLVNLYNLPGDN------
CHICK	NFDEF----------------------SKHLRGLVNLYKLPGDN------
XENTR	NFEDF----------------------SQHLKGLVNLYKVPGDN------
DANRE	NFDEF----------------------SKHLKGLVNLYNLPGDN------
CIOIN	KFLEL----------------------SKQLKGLTDIYPPSMDQ------
ANOGA	DFADF----------------------TTQLEGLASIYQLNAEK------
TETNG	NFDEF----------------------SKHLKGLVELYKLPGDN------
DROME	DFADF----------------------TVQLEGLSSIYQLNAEP------
BRUMA	RWDLI----------------------HTALTAMFHLVPSDLSR------
CAEEL	EFSKL----------------------RDSITAILSSLPKEITL------
YEAST*	DLKAF----------------------HNNLKFLYLLDAYSHIESDS---
SCHPO	NTPLF----------------------EKNVKWLATFDHSSP--------
CANAL	KLGNF----------------------PVNLQRLATIDKFT---------
CANGA	SLKQF----------------------HHNLEFLYLLDTYSSVESDNQIS
ASPNI	SLDNF----------------------HKNLQWLARHDKL----------
RHIO9	KFDLF----------------------KRNLWSLALLDQLNIK-------
CRYNE	LWNAPVLEEGYSLEKGLWEREETARRLEQEFADLKWFDDVAG--------
USTMA	AEKLFGPVSSSSLQQDRTVVAQTLAKWTTNLNELLVLDDLEARASQA---
	460 470 480 490 500

HUMAN*	------------------------------------KLKTKMYLALQSLE
CHICK	------------------------------------KLKTKMYLALQSLE
XENTR	------------------------------------KLETKMYLALQSLE
DANRE	------------------------------------KLKTKMYLALQSLE
CIOIN	------------------------------------TTRTRMYMALQSLD
ANOGA	------------------------------------KIKVNAFVALQALE
TETNG	------------------------------------KLKTKMYIALQSLE
DROME	------------------------------------KVKKKAFVALQAME
BRUMA	------------------------------------DEKIYCLRSLEYAE
CAEEL	------------------------------------SEKNACKEA--FRA
YEAST*	-------------TSHNNGSSDKSLDSSNA--SFNNQGKLDLFKYFTELS
SCHPO	------------------------------------SVQQSCFQYLDSLS
CANAL	------------------------------------NYNPDLFIYVESIA
CANGA	SGNNLGSGNGTNGSSLTNKTDKKSVSSGNGLDSKLNEGKLNLFKYFRELK
ASPNI	------------------------------------SEEVNCFEAIEGLY
RHIO9	------------------------------------YKPLDFFSITKNLL
CRYNE	------------------------------------DESVDWFEELEKIT
USTMA	--------------------------------ATEGSRTSDLFAAMQGLC
	510 520 530 540 550

HUMAN*	QDLSKMAIMYWKA-TNAGPLDKILHGSVGYL-TPRSG----GHLMNLKYY
CHICK	LDLSKMAGMYWQA-TNANPLDKILHGSVGYL-TPRSG----GLLMNLKYY
XENTR	LDLTKMAAMYWQA-TNASVLEKILHGTVGYL-TPRSG----GQVMSLKYY
DANRE	LDLTKMMHMFRIA-TNANTVETILHGSVGLL-TSRSG----GHLVSLQCY
CIOIN	MDLAKMSQVYRDSHGDSDTLHIIMNGYVGLL-FPREV----GTCSQLKYF
ANOGA	TDLHTLYTLSLQ--HYADVHAQLHKAPLGVV-QKRRG----GHPMRLTYF
TETNG	LDLTKMMHMYRLA-TSANTLETLLHGSVGLL-SARSG----GHLVSLHCY
DROME	TDIQSLYQLHLQG-HSGDSYSLMTSSSVGLV-LPRRG----GHPMRLTYF
BRUMA	KDLLNMSG------SAPSIFEQINGGILGLC-KPRTE----STPFTIYPF
CAEEL	LDQLLIRD------AADSSFTAINTGKYGFY-APRNE----LRSGRIYYV
YEAST*	HYIRQCFQDNC----CDFKVRTNLNDKFGIY-ILTQGINGKEVPLAKIYL
SCHPO	SSLNAIYEAEL---SLLAQEENVIMHGNGKPLSNYEG----QLGLRIVYW
CANAL	LLFNTIFEIEQQSNSSNWEIENGLSSSIGKVNINNED----RLGVTLDFW
CANGA	KYINTFFKESC---DNRFKVVANVDNRFGLYIYETHGYSMRSRPLAKVYF
ASPNI	ESLKRLWNEESSQRKFGGDYEHICSGGIGRPSLHRGS----RVGLWLDYW
RHIO9	QDLKAVCLIES---EIEFDFSSILTEGHGIP-CLHLS----YPGISIAYW
CRYNE	GTVERLVA------ETRYVSLNIFPKGSKMY-NDNEK----KNSEQRLYP
USTMA	SAVARISEAEA---SSSSSAAALLDRGHGLH-KLHET----KPFLHSIYA
	560 570 580 590 600

MED1

```
HUMAN*  VSPS-----DLLDDKTASP--I-ILHENN--------V------------
CHICK   VSPY-----DLFEDGTGAP--V-ILHESN--------V------------
XENTR   VSPY-----DLFDDTTGAS--I-SLSEGHA-------V------------
DANRE   VSPN-----DIFEEEKAAH--L-N-TDVN--------I------------
CIOIN   LSPY-----DLVDVKTKQL--K-TLTNQ---------V------------
ANOGA   VAPY-----ELLDLTTRTM--N-VLSAELI-------A------------
TETNG   LSPY-----DLLEEKTCSP--L-SLTDNN--------I------------
DROME   CPPL-----HLPEGDPKLA--SGDFTIDQV-------M------------
BRUMA   VEPV-----IFLDAASGGI--I-SPTDEQ--------V------------
CAEEL   AEPV-----LIRLAEKEG---K-TKADAS--------Y------------
YEAST*  EENKSDSQYRFYEYIYSQE--TKSWINESAEN-----F------------
SCHPO   ---------KLLDKTYSTQIFMDNLSHESLPHLLFGYN------------
CANAL   QDY------RFINHEYEIVKNQSGLL-----------V------------
CANGA   ERAKVPQQ-RFYEYIYSSE--TGSWINENSEN-----Y------------
ASPNI   VPRA-----RVMDAKQRKS--ADAMDIDQSENSANGELSGGNGEWRIAVE
RHIO9   LDKP-----TIASTDWNQV-------------------------------
CRYNE   TDKH----------------------------------------------
USTMA   HDPT---------------------------------L------------
        610 620 630 640 650
```

```
HUMAN*  ----------------SRSLGMNASVTIEGTSAVYKLPI--------APL
CHICK   ----------------PRSLGMNVSVTIEGTMAMYKLPI--------APL
XENTR   ----------------PRSLGMNVSVTIEATSAMYKLPI--------APL
DANRE   ----------------PHTLGVSVSVTVEGTTSVYKLPI--------APL
CIOIN   ----------------LENIGMQAEIGLEVYETPCKLPI--------TPL
ANOGA   ----------------QERIGCSVAVVLEASSA-NKLQI--------QPL
TETNG   ----------------PRSLGI----------------------------
DROME   ----------------RSSYGLSATINLEGSSA-NKLQT--------LPT
BRUMA   ----------------LYDVEPYLQLCVVSSSTPNFFXD--------TTL
CAEEL   ----------------L-DLLPYIEISFFKHDKPSKLPV--------FT-
YEAST*  ----------------SNGISLVMEIVANAKESNYTDLI--------W--
SCHPO   -----------------------------------LLNS--------PPI
CANAL   ----------------GDYYTIQVDIVEASKQTDYLLDN--------QVQ
CANGA   ----------------SVGVNLILEVNFDDTDEDVFW-------------
ASPNI   CEEGYPSLRVSKDWLGSEVFTTAHDDAEASASDSATLEVRVINWAEPPPA
RHIO9   -----------------------KQLLQQNENHDVLLRA--------SKI
CRYNE   ----------------------------------SVFPT--------FRL
USTMA   ----------------KQNYTLSIGV----------------------QA
        660 670 680 690 700
```

```
HUMAN*  IM---GSHPVDNKWTPSFSSITSAN-------------------------
CHICK   IM---GSHPVDSKGTPSFSSITSAN-------------------------
XENTR   IM---GSHPIDNKGTPSFTSITNAN-------------------------
DANRE   IT---GSHPVDNKGTPSFSSITNSN-------------------------
CIOIN   IA---NNHPTDETSKPRFAEINQHN-------------------------
ANOGA   LVVNAGSGSAASVLQPVYTPIEKHN-------------------------
TETNG   --------------------------------------------------
DROME   VTLVRDAQTGLEV--PTYAQLNQNN-------------------------
BRUMA   FA------------HGEWRNAVSGR-------------------------
CAEEL   -Q------------TGEWSTFNDAN-------------------------
YEAST*  --------------------------------------------------
SCHPO   LQSSKINWALENTLTPIPTTMELVFDDINLI-------------------
CANAL   EWDISMSKYKFEFDSNSLKPLVLDGDGF----------------------
CANGA   --------------------------------------------------
ASPNI   LNGNQSSSGNMDLDSNMLGSSSPNR-------------------------
RHIO9   LISFEDSTQLMHYLPLSRPNYLLAFDETEDSIKEGVDGEHFKVVYENSYP
CRYNE   LPSSSIGTNPIWRIRPAKSSNESVPTISSIHSTKSDVGGAGDLDMEEQWL
USTMA   LDLPVADQTGISTVTPSFPLSLSAQDLLASSSTMDQAQALGSIPSETDR-
        710 720 730 740 750
```

```
HUMAN*  -----------------SVD------------------------------
CHICK   -----------------SVD------------------------------
XENTR   -----------------SVD------------------------------
DANRE   -----------------CVD------------------------------
CIOIN   -----------------SVP------------------------------
ANOGA   -----------------STS------------------------------
TETNG   --------------------------------------------------
DROME   -----------------SLL------------------------------
BRUMA   -------------------Q------------------------------
CAEEL   --------------------------------------------------
YEAST*  --------------------------------------------------
SCHPO   --------------------------------------------------
CANAL   --------------------------------------------------
CANGA   --------------------------------------------------
ASPNI   --------------------------------------------------
RHIO9   KFMSPMRYVKPLPTIPDSVP------------------------------
CRYNE   ETDWVIECIEENGIAGVVVSRNWLIDSGHEEKQQVSGTIRLENLLYHPFQ
USTMA   -----------------EKL------------------------------
        760 770 780 790 800
```

MED1

```
HUMAN*  LPACFFLKFPQPIPVSRAFVQK------------LQNCTG----------
CHICK   LPACFFLKFPRPIPVSRAFIQK------------LQGCTG----------
XENTR   LPACFFLKFPQPIPVSRAFIQK------------IQHCTG----------
DANRE   LPACFFLKMNCLMPFSLSYIKK------------MGSTTG----------
CIOIN   LPACYSLKLHPPVPILDHIVKQ------------IEQETK----------
ANOGA   LPATFVLRLSKPMPLNSAMMRA------------IRAIVG----------
TETNG   --------------------------------------------------
DROME   MPATFVLRLNKPMPVCLESLKA------------L----G----------
BRUMA   LPAAFCLRFSRPIIFSSAAIKK------------LSKIAS----------
CAEEL   --VCICVRFNQGFLLSQQTIRK------------LSHIVP----------
YEAST*  FPEDFI---SPELIIDKVTCSS----------------------------
SCHPO   IPEQGVKPLLDLLHVHDITIPW----------------------------
CANAL   LAWSLQFKLNHLVYLPVYILEY------------FSLALG----------
CANGA   FPKEFV---PTDLFIDEK--------------------------------
ASPNI   ---RFVARLEPALDIPFLVATE------------VYRHLGIQMPQDFRLS
RHIO9   VPVRFVATVEPPIAMADELCQT------------LMNVIG----------
CRYNE   QPTFLAIPQQQPFPYTAPFIHSSKDGNGLDQHWSIAQPGP----------
USTMA   VPLHFVVQLSPPVVVTRPTAAKLACICNLQQLTPHNNNPGAPVPASGGAT
                 810       820       830       840       850
```

```
HUMAN*  ----------IPLF-------ETQPTY----APLYELITQFELSKDPDPI
CHICK   ----------IPLF-------DTAPTF----VPLYELITQFELSKEADPI
XENTR   ----------IPLI-------DGSHTF----LPHYELVTQFELAKEKEPG
DANRE   ----------IPVF-------ETAPTL----SPLYDLIIKSQLREEGGAS
CIOIN   ----------------------LPVLYKDKSTSYLSLIQKDDECCKVTFG
ANOGA   ----------GSGAGEQQQAGEGSSGGGGLPGSLLGLIAHHASAGSVTD-
TETNG   --------------------------------------------------
DROME   ----------LPGL-------DSVATPPGPPTTVLNLIVQTASKQAIKN-
BRUMA   ----------VQLNVEGS-------------YNLYQYLLNE--NE-----
CAEEL   ----------KSPLVRNY-------------VNFYRYMTGRVGVK-----
YEAST*  --------------------------------------------------
SCHPO   --------------------------------------------------
CANAL   -------------------NNYTLDTGDHNCCNVFAKINEGESFEQVLN-
CANGA   --------------------------------------------------
ASPNI   TYDGLLAPGWSLGSEDSHI-------------------------------
RHIO9   -------------------------------------LLNTEAMEQYAIK
CRYNE   --------------------------------------------------
USTMA   WFEDVLATSW----------------------------------------
                 860       870       880       890       900
```

```
HUMAN*  PLN-HNMRFYAALP--GQQHCYFLNKDAPLP-DGRSLQGTLVSKITFQ-H
CHICK   PLN-HNMRFYAALP--GQQHCYFLNKDAPLP-DGRSLQGTLISKIAFQ-H
XENTR   TLN-HNMRFYASLP--GQQHCYFVNKDAPLP-DGRSLQGTLLSKIPFQ-H
DANRE   PPSAQSMRFYASLP--GQQHCYFLNGDAPVQ-DGRSLQGALVSKIPFR-H
CIOIN   ----------------SVTHRHHL--------DFSHVEGLLVHQVRFT-S
ANOGA   ----LAKGLLVSLP--DQYHCYFLT-------DNPSLRGMMVSSIPFT-E
TETNG   ----QSMNHWFV--------------------------------------
DROME   ----TQRGLYVNLP--KETHCYFFT-------DNRKLQGTLVSSLPFT-E
BRUMA   ----ECHDLFVRLP--GCSTQHYIFDDS----MKSSQEDGILRQIPFA-H
CAEEL   ----SNLKLLTQFPDEGDIQQQYQVDSK----MLTKEDDVVAMEMYLS-D
YEAST*  --NSSSSPPIIDLFSNNNY------NSRIQLMNDFTTKLINIKKFDIS-N
SCHPO   --PVQNYSHMLGLPSKSTVNYKFINKNQAIQLTGFDVSARSLRHVPFH-H
CANAL   -----------------------IDSNSVSFQLTNDIPGKFVPLKSFHIN
CANGA   --DQLTSRKVSDVLCHAFYELGSSKKQTIELMNDFTTDLIQIRRFNIN-N
ASPNI   ----DRKRSKISVQSFDEEGKPCIKRHSYSFQTFEPTSGKTLRNLPFS-H
RHIO9   NTSQCPSLEEILVPNIMLTKQYSCDKQVYKWMSSSSTSAKMVSRIPFQ-H
CRYNE   -------------------------------------LGWVVGRIGMGRN
USTMA   ----SRRPRQASEDARKSSKCTFTLAQTVESVRSTELQGLVIDCLPLLSN
                 910       920       930       940       950
```

```
HUMAN*  P--------------------GRVPLILNLIRHQVAYNTLIGSC--V---
CHICK   P--------------------GRVPIILNLIRHQVAYNTLIGSC--V---
XENTR   P--------------------GRVPIILSLIRHQVACNTLIGSC--V---
DANRE   P--------------------AQVPALLDIIRHQAAYNTLIGSC--V---
CIOIN   P--------------------EHVVAVINLLRKQCYLNTVLTSCMRK---
ANOGA   P--------------------QHVPKILTYLRQQAVFNQLLSSC--IRT-
TETNG   --------------------------------------------------
DROME   P--------------------AQVPRIVAFLKKQALFYTLLASC--VRE-
BRUMA   L--------------------QDVVFLVSIIRTQLAHNAFFESL--VRA-
CAEEL   F--------------------RDLSKLIETLRSEWKHASLWESILAICE-
YEAST*  ---------------------DNLDLISEILKWVQWSRIVLQNVFKLVS-
SCHPO   P--------------------KQIRGILAIVRQYLLLQLILENI------
CANAL   K-------------------LVDISKLIPILRNFLVLINLCRTVIKSKLT
CANGA   ---------------------DNLDLIADILNWTIWYRTVLRPIYLKLV-
ASPNI   P--------------------RQLADVLPTLRQYALLANMIQGTFPSHD-
RHIO9   P--------------------VQIYNILQCLRQQEMFNTLFKSIFNEAH-
CRYNE   L--------------------KGLYGTIKALRKQLVLNQMFASVFKSEAL
USTMA   PAPPADAMDESSSPQVGASTLARLLAAIEVLRDEVKVTEIMHSAISSSE-
                 960       970       980       990      1000
```

MED1

```
HUMAN*  -----KRTILKEDSPGLLQFEVCPLSE-SRFSVSFQHPV-NDSLVCVVMD
CHICK   -----KRTVLKEDSPGILQFEVCPLSD-SCFSVSFQHPV-NDSLVCVVMD
XENTR   -----KRTMLKEDCPGLLQFEVAPLSD-SCFSISFQHPV-NDSLVCVVMD
DANRE   -----KKACIKEDTPGLLQFEVCPLTD-SSFSVSFQHPV-NESLVCVVME
CIOIN   ----SKKAQVSDPADMEWCFDVSYNDQRSKLNVTFEHPTCSCSTAFAEIS
ANOGA   ----NAPGGERNDR-RLLVFEVSALSC-QNITVSLIHPY-EESLATVEFD
TETNG   --------------------------------------------------
DROME   ----QQKQYNDMD--STVILEVTAVSF-NQITVELQHPY-EESLATVDFL
BRUMA   ----HSRSACVDE--NVVDIHIASSYI-DCFDLQFYLKK---NFFIARV-
CAEEL   ----SSKGEQKEV--NAVDMQLVLRRT-EFF-LQFDTDY---GSMTMQIF
YEAST*  ----TPSSNSNSSELEPDYQAPFSTSTKDKNSSTSNTEP-----------
SCHPO   -----KSADLAETAASSSLHLSLYFKEHPIVHAQYQEINKLNNSEQIIIV
CANAL   QLPVFNSKRRRSSRFLGIPTTERELTEEFKHKLKESLKLPDDVTDEEILG
CANGA   ----SSISDDDDQHIRADI-----ANTKDEFTVENTNPE-----------
ASPNI   ----RVKPEREKPKSIVQPQDEKYKTTRNGDITILTNENPNEKKLNMLLG
RHIO9   ----SEDRSAANVSDNYLILEVATMDAPYSIHMTLAPPQ-GSSFVMVPLS
CRYNE   TTYENGNGEEGHDEDDDMDDDWFSGPP----KAVPCSATLHQNSISITIP
USTMA   --SEAGTVDGGQGQADDLSLDDVLASASSE----------DSGKIAVTIA
                 1010      1020      1030      1040      1050
```

```
HUMAN*  VQDSTHVSCKLYKGL-SD-----A-LICTDDFIAK---------------
CHICK   VQDSTHVNCKLYKGL-SD-----A-LICTDDFIAK---------------
XENTR   VQDSSHVSCKLYKGL-SD-----A-LICTDDFITK---------------
DANRE   VIDSMQVACKLYKGL-SD-----A-LICTDDFITK---------------
CIOIN   FSSFGITSCHMFVGRGDQ-------PLCSDKLATT---------------
ANOGA   LQNIANIAVRIYCSE-GE-----TSDEHTVERISA---------------
TETNG   --------------------------------------------------
DROME   LED-GQPTCSVYCLT-NE-----Y--ELLSQKLTR---------------
BRUMA   AVSPSNINVSLECVING-------AVLPESQHATA---------------
CAEEL   EKSPSNYVVKTTSQITGE-----SPSLDFDKMLTE---------------
YEAST*  -------------------------IPRSNRHGSV---------------
SCHPO   LSVLSDGRLNVDYMSSGGKELSKQQSHVFQKLIQQ---------------
CANAL   LSAISETANYSTIPPANKDDIDLESFLGNEDTTNSGDVDTNGKRGTDHLP
CANGA   -----------------------IMKGPTNKNASF---------------
ASPNI   LAD----------------------DLKLEDEVQE---------------
RHIO9   IDIPTDDPTKPIVRLHSSPIHKWNMNVFNQDLMTK---------------
CRYNE   LLDHEGKCEHLEIIAKASDEGPGYTNVEYKGGKID---------------
USTMA   FRTPLHGAVD----------------------------------------
                 1060      1070      1080      1090      1100
```

```
HUMAN*  ------VVQRCMSIPVTMRAIRR-KAETIQADTPALSLIAETVEDMVKKN
CHICK   ------VVQRCMSIPVTMRAIRR-KAETIQADTPALSLIAETVEDMVKKN
XENTR   ------VVQRCMSIPVTMRAIRR-KAETIQADTPALSLIAETVEDMVKKN
DANRE   ------VVQRCMSIPVTMRAIRR-KAETIQADTPALSLIAETVEIMVKKN
CIOIN   ------VMQKSLSLPVTMRTVMF-HTKKNRHKEPEVHRQSRHPTPHPPMP
ANOGA   ------VLQRCMSIPVTLRALMK-WEEEHEQKFKPNVLGSSTVSSGGAGG
TETNG   ----------------------------------------TVEIMVKKNN
DROME   ------TARKVVSIPMVIYKLLKCWDEEHEFKLHGAI---------GPGS
BRUMA   ------ILAKSWSLAVMMRSVLR---------------------------
CAEEL   ------KLNSTWSIPATLTFAIT---------------------------
YEAST*  ------VEASR---------------------------------------
SCHPO   ------TCNIGLAIEVFIKKVVN---------------------------
CANAL   VNGNNYFLLKLESIDLSSRGDLQ---------------------------
CANGA   ------MQRNSMSLQ-----------------------------------
ASPNI   ------SGSDELKVDVTLRTQLG---------------------------
RHIO9   ------ELQSHYNVPLFIRWLFD---------------------------
CRYNE   ------ESEADRDLVSLVRKVLE---------------------------
USTMA   ------EAQRSLSLQLVFRALAD---------------------------
                 1110      1120      1130      1140      1150
```

```
HUMAN*  LPPASSPGYGMTT----GNNPM-----SGTTTPTNTFP------GGPITT
CHICK   LPPASSPGYGMTT----GSNPM-----SGTTTPTNTFP------GGPITT
XENTR   LPPASSPGYGMAS----GSTAL-----SGITTPTSSYT------SGQISS
DANRE   LPPTGSPGYMGMGTGPDGSNPMGLPLNSGNNSVVCVG--GNGSFQGPITS
CIOIN   PVKPQPPIMAPQQQQPQLLNPLQPTLPTPATTPPVSINQSMSHFTEPSNA
ANOGA   GPGGGGGGGGGAGGATPGLNN-----GGDGTGPAGGGLTGSG--DDGTMN
TETNG   LPPSGSPTYNMASC--DGPNPMGLPGLTGGNTPTAGGPPGGPNFAGPITS
DROME   GSGAIGGGGMSGGGPVSGVGNNFSQFSMDTPTPSDGSLPGG-----GFAN
BRUMA   ----------------------------------RGGAISLAALEKPFVM
CAEEL   ----------------------------------DLKCRINSVLN--CVI
YEAST*  --------------------------------------------------
SCHPO   --------------------------------------------------
CANAL   --------------------------------------------------
CANGA   --------------------------------------------------
ASPNI   --------------------------------------------------
RHIO9   --------------------------------------------------
CRYNE   --------------------------------------------------
USTMA   --------------------------------------------------
                 1160      1170      1180      1190      1200
```

MED1

HUMAN*	LFNMSMSI------------------------KDRHESVGHGEDFSKVSQ
CHICK	LFNMSISM------------------------KERHDSVGHGEDFSKVSQ
XENTR	LFNM--GI------------------------KERHDSAGHGDDFSKVTQ
DANRE	MFNMGRTG---QGSSCIDTMGQVGGQQQAMQQQHQQSQSHGSDDFNKVTQ
CIOIN	GYFTQQRS----------------TYYHQQQQQQQQQQMQQQQLHYQGDM
ANOGA	GFNLSSLA-----------------------------KKRRMEDFCKS--
TETNG	LFGMSRTERQVQGGEC-PSQGGLTGQQQLQQHQQQQPGQGHSEDYNKVTQ
DROME	INNLKMDA-----------------------------KSRSLAD------
BRUMA	SENVDNNC----------------DVKISQSWLFVPNITDNSINYNKTEH
CAEEL	SFDSSSSS----------------DKQHQNYW---------SISNNNTQL
YEAST*	--
SCHPO	--
CANAL	--
CANGA	--
ASPNI	--
RHIO9	--
CRYNE	--
USTMA	--
	1210 1220 1230 1240 1250

HUMAN*	NPILTSLLQITGNGGSTIGSSPTP-PHHTPPPVSSMAGNTKNHPMLM---
CHICK	NPILTSLLQITGNVGSAIGSSPTP-PHHTPPPLSSPAGNTKNHPMLM---
XENTR	NPILTSLLQITSNSGGTLGSSPTP-PQHTPPPASSPASNTKNHPMLM---
DANRE	NPILTSLLQITGSVGSSPTPQQ-PQPHQTPPTTTSPASNTKNHPMLM---
CIOIN	LPQYNMTTPMQTNQLPDNYFTHPPPPRQMPPPPSYPNS------------
ANOGA	-PKSDSNLSVGGGGGGGA---------------SDAVGTEKANTGAA---
TETNG	NPILTSLLQITGSVGSSPNSQNAPTPHQTPPPTSSPASNTKNHPMLM---
DROME	-------------------------------------AFAASTSAAA---
BRUMA	EYVVDERISLNCSCDSNALGRQSPL------------------SLLALFK
CAEEL	MQSAAQKSAAKCTKKKVTVLEVGPAIEPTQLVYTRENDEEFQGSLLSSFG
YEAST*	--
SCHPO	--
CANAL	--
CANGA	--
ASPNI	--
RHIO9	--
CRYNE	--
USTMA	--
	1260 1270 1280 1290 1300

HUMAN*	----------NLLKDNPAQDFSTLYGSSPLERQNSSSGSPRME----ICS
CHICK	----------NLLKENPPQDFSTLYGSSPLERQNSSSGSPRME----MGP
XENTR	----------NLLKDNPAQDFSTLYGGSPLERQN-SSGSPRTE----LGS
DANRE	----------NLLKDNPSQDFAALYSSSPLERQN-SSGSPRTDNQGQPCS
CIOIN	----------MQVKGMNQQQYQMPYQSQP---------------------
ANOGA	----------GVAGATGATSSSSTTTTTTITSQDVGAAA--------SGA
TETNG	----------NLLKDNPAQDFAALYSSSPLERQNSSSGSPRTEGMGGTCP
DROME	----------AIAGLINLKRE---------TDPQSGSSA--------SGT
BRUMA	ELE-------KIEELIPPQ--------KPMNGAQQCTAPPRNEML-----
CAEEL	EVDDYYVKTSTIGRTRPQP--------RPKREPNQLTS---MDMM-----
YEAST*	--
SCHPO	--
CANAL	--
CANGA	--
ASPNI	--
RHIO9	--
CRYNE	--
USTMA	--
	1310 1320 1330 1340 1350

HUMAN*	GSNKTKKKKSSRLPPEK--------------------PKHQT--------
CHICK	GGSKQKKKK-SRMPADK--------------------PKHQT--------
XENTR	SASGKPKKKRPRTGTEK--------------------MKNQT--------
DANRE	G--TKGKKKRLR-GSDKGVM----------GAAGGMSMKQSQQQVVDMTQ
CIOIN	--
ANOGA	TGAGNSSEQRLQTGGATE--------------------------------
TETNG	GGNPKGKKKRPR-GMEKGGILPGAAGGGGGGGGGGLGMKSQQSSSMSQQH
DROME	TVSGSS--
BRUMA	--
CAEEL	--
YEAST*	--
SCHPO	--
CANAL	--
CANGA	--
ASPNI	--
RHIO9	--
CRYNE	--
USTMA	--
	1360 1370 1380 1390 1400

MED1

Positions 1401–1450

```
HUMAN*  --------EDDFQRELFSMDVD-SQNPIFDVNMTADTLDTPH--------
CHICK   --------EDDFQRELFSMDVD-SQNPIFDVNMTADTLDTPH--------
XENTR   --------EDDFQRELFSMDVD-SQNTIFDVGMAGDALDTPH--------
DANRE   HHHLTSHSEDDFHRELFSMDVDASQNPIFDVSLPGDGLDTPH--------
CIOIN   --------------------------------------------------
ANOGA   --------EDTGGLPLFGETVPAAAGTDAKGSSLGGTTTTNAMISAK---
TETNG   QHHPVTH-EDDFHRELLSMDVDASQNSIFDVNLTGDGLDTPH--------
DROME   -------------------------------SSSGSAKTSDHDIADKYKN
BRUMA   -------------------------------APRTSRIHSENTMTINAFS
CAEEL   -------------------------------SAELDK--SRHTM------
YEAST*  --------------------------------------------------
SCHPO   --------------------------------------------------
CANAL   --------------------------------------------------
CANGA   --------------------------------------------------
ASPNI   --------------------------------------------------
RHIO9   --------------------------------------------------
CRYNE   --------------------------------------------------
USTMA   --------------------------------------------------
```

Positions 1451–1500

```
HUMAN*  --------------ITPAPSQCSTPPTTY---PQPVPH-PQPSIQ-----
CHICK   --------------ITPAPSQCSTPPTTY---PQPIPH-SQPSIQ-----
XENTR   --------------ITPAPSQCGTPPNVY---QQAIPH-TQANMQ-----
DANRE   -------------SITPAPSQCGTPPPG-----PGMQYHPQCHVQPQIQV
CIOIN   ------SPLNLDDLTSPISTSMTSPSFGCHTSVTPPLVGIHGLASPV---
ANOGA   ----NEPPSPALGAVSPIASKVVSPSGGAGLEKTPLPTISGSGLEIIPL-
TETNG   -------------SITPAPSQCGTPPSG-----SSIPY-SQSHVQSQ---
DROME   IWKDKTPNLKHCVSITPIPGDGKSGSAGG---VSGVEVQRTGGIEIIPLN
BRUMA   VLNQRLRPQIPPQSPSPASERYRASQAGNFSQLSPLDIARQRLAQQAPMS
CAEEL   ------------------EGSMRDAQTSSF-----LDAARVRMKQSIGAA
YEAST*  --------------------------------------------------
SCHPO   --------------------------------------------------
CANAL   --------------------------------------------------
CANGA   --------------------------------------------------
ASPNI   --------------------------------------------------
RHIO9   --------------------------------------------------
CRYNE   --------------------------------------------------
USTMA   --------------------------------------------------
```

Positions 1501–1550

```
HUMAN*  ----------RMVRLSSSDSIGPDVTDILSDIAEEASKLPS---------
CHICK   ----------RMVRLSSSDSIGADVTDILSDIAEEASKLPA---------
XENTR   ----------RMVRIPSTDAIIPDVTDILSDIAEEASKLPG---------
DANRE   QSQSQGSI-PRMVRLSSSDSIGPDINDIL-DIPDQANKGSNIGHGQHLM-
CIOIN   --------------------------------------------------
ANOGA   ---ATSAG-----TVAASGFPSPSVTSAPIAVPQSNATGSTSSTTATSS-
TETNG   QQQPQAAAPPHMARLPSSDSIGPDITEILSDLPEQTGKGNSGSHGQNPIG
DROME   AQAAIAGG-----GVTASSSATPTTITITPITGKDPSKDSTKKSTAASAG
BRUMA   NPTDIFEF------------------------------------------
CAEEL   HAVGLASH------------------------------------------
YEAST*  --------------------------------------------------
SCHPO   --------------------------------------------------
CANAL   --------------------------------------------------
CANGA   --------------------------------------------------
ASPNI   --------------------------------------------------
RHIO9   --------------------------------------------------
CRYNE   --------------------------------------------------
USTMA   --------------------------------------------------
```

Positions 1551–1600

```
HUMAN*  -TSDDCPAIGTPLRDSSSSGHSQSTLFD-SDVFQTN-----NNENPYTDP
CHICK   -TNEDCPPIGTPVRDSSSSGHSQSALFD-PDVFQTN-----NSENPYTDP
XENTR   -PGEDCPNLGTPVRDSSSSGHSQSTLFD-TDVFQVDGGGGSGGENPYPDP
DANRE   -TSEDGGSLGTPVRDSSSSGQG-STVFE-ADLFNTN-----SNENPFTDA
CIOIN   --------------------------------------------------
ANOGA   ----------STVKKSSSSSGGGSG-------------------------
TETNG   GGGEEGGTLGTPIRDSSSSGQG-SAVFDSADIFNTN-----SNENPFTDA
DROME   VGVAAKRPHESSTSSSSTSGCSGSG-------------------------
BRUMA   ------DDP--PRMNSAGSMGTPAQQFH----------------------
CAEEL   ------QPPASPVAAAAAAPGGPMRHNS----------------------
YEAST*  --------------------------------------------------
SCHPO   --------------------------------------------------
CANAL   --------------------------------------------------
CANGA   --------------------------------------------------
ASPNI   --------------------------------------------------
RHIO9   --------------------------------------------------
CRYNE   --------------------------------------------------
USTMA   --------------------------------------------------
```

MED1

```
HUMAN*   ADLIAD---AAGSPSSDSPTNHFFHDGVDFNPDLLNSQSQSGFGEEYFDE
CHICK    ADLIAD---AAVSPNSDS-SNHFFPDGVDFNPDLLNSQSQSGFAEEYFDE
XENTR    VDLIVD---SHGSPNSDS-PNTFF-NSVDFNPDLLNSQSQSGFTDDLNDD
DANRE    ADLIAE-AATATTPNSDTSSTNFFPD-TDFNPELLS--GQGSFSQTYFED
CIOIN    --------------------------------------------------
ANOGA    --------SHRKTSDSSERTSSS---------------KSEKKKKRKTDD
TETNG    VDLIAEAAATAATPTSDSSSTNFFPDAADFNPDLLT--SGHGFSQNYFDD
DROME    --------SSMSSSASS-GSSDT---------------QKEKKRKKKRDD
BRUMA    --------FPEAGQ-----SPYPF---------------AA-------VQ
CAEEL    --------YPSGFEPPPSVAPYDF--------------PADPSKKQRKQR
YEAST*   --------------------------------------------------
SCHPO    --------------------------------------------------
CANAL    --------------------------------------------------
CANGA    --------------------------------------------------
ASPNI    --------------------------------------------------
RHIO9    --------------------------------------------------
CRYNE    --------------------------------------------------
USTMA    --------------------------------------------------
                  1610      1620      1630      1640      1650
```

```
HUMAN*   SSQSGDND-----DFKGFASQALNTLG-VPMLGGDNGETKFKGNNQADTV
CHICK    SSQSGDTD-----DFKGYASQALSTLG-VQVLGGDGGDNKFKGSNPSDTV
XENTR    SSQSGDN------DFKDFAGPGLASLGIVSGLPVDGGDGKYKMALGADTV
DANRE    SSPSPDPDLELVKSFSGGSQQNTPT-G-TPQNPASHGQNTPETSL-KDPF
CIOIN    --------------------------------------------------
ANOGA    SAMGPPNKLPYK--------------------------------------
TETNG    SSPSADGDMDIVKSFGGNSQQNTPS-G-TPQNPASQGQSTPEPSL-KDPF
DROME    SPMGPPEKIYSRQNSPAGGADASATGGVVRK-------------------
BRUMA    STRGPLSAQPTLKRRG--RGRKAGNVGDRM----------------LDP-
CAEEL    AKKQPGEEPPAGRGKGGKKGRGAGAVGAAAAGGRKSAGGAGENPFGMDP-
YEAST*   --------------------------------------------------
SCHPO    --------------------------------------------------
CANAL    --------------------------------------------------
CANGA    --------------------------------------------------
ASPNI    --------------------------------------------------
RHIO9    --------------------------------------------------
CRYNE    --------------------------------------------------
USTMA    --------------------------------------------------
                  1660      1670      1680      1690      1700
```

```
HUMAN*   DFSIIS----VAGKALAP--ADLMEH---HS-GSQGPLL----TTGDL--
CHICK    DFSIIA----AASKALGS--SDIMEH---HS-GGQSPLL----NTGDL--
XENTR    DFSIIAG---GGSKNLGG--PDIQET---QS-RSQSPLL----GS-DL--
DANRE    EIGI-FGGNGGGGKPLMAPASDIADSHHAHTGGGQSPLIGIVVAGNDFKN
CIOIN    --------------------------------------------------
ANOGA    ------------------------------SSGESSP-------------
TETNG    DMGIVFGGNSGGAKTLLGQASDMSDT---HSGGSQSPLI-MGAGCGDFKS
DROME    -----------------------------FSSPSSSP-------------
BRUMA    ----------------------------------ARPILDQ---------
CAEEL    ----------------------------------MRPPMLQ---------
YEAST*   --------------------------------------------------
SCHPO    --------------------------------------------------
CANAL    --------------------------------------------------
CANGA    --------------------------------------------------
ASPNI    --------------------------------------------------
RHIO9    --------------------------------------------------
CRYNE    --------------------------------------------------
USTMA    --------------------------------------------------
                  1710      1720      1730      1740      1750
```

```
HUMAN*   --GKEKTQK---RVKEGN------GTSNSTLSGPGLDSKPGKRSRTPSND
CHICK    --GKEKSQK---RVKEGN------G-SGSNMAGSGIDGKPGKRSRTPSSD
XENTR    --GKDRPQK--QKVKESG-NGGGAGGGLSGMQSAGMEGKSMKRSRTPSSD
DANRE    VDAKAKQQQ--MRTKEDH-VGGSSGQGM--VSACSSEMKQVKRSRTPSSE
CIOIN    ----------------------------------------TPNAPSPMYT
ANOGA    ----------------------HRSGGGVG-GSSLSTSKSSPKHSSPVHH
TETNG    AEPKLKPQQSHMRPKEENGSSGSSNAGI-GMGSSSTDCKQVKRSRTPSSE
DROME    ----------------------KAGGGGQGLMAGVPTARPSPKH-SPV-Y
BRUMA    ----------------------NTGLMFRGMNIPTRKPRGTSTARRPRRP
CAEEL    --------------------------------------RSSSEQHNPNPH
YEAST*   --------------------------------------RRRSSTNKSKRP
SCHPO    --------------------------------------------------
CANAL    --------------------------------------------------
CANGA    --------------------------------------RRRRSSNKAKRP
ASPNI    --------------------------------------------------
RHIO9    --------------------------------------------------
CRYNE    --------------------------------------------------
USTMA    --------------------------------------------------
                  1760      1770      1780      1790      1800
```

MED1

HUMAN*	GKSKDKPPKRKKADTEG-KSPSHSSS----------------NRP-----
CHICK	GKSKEKLPKRKKQETDG-KSPSHSSS----------------NRP-----
XENTR	GKSKDKPPKRKKTESDG-KSPSH-IT----------------NRP-----
DANRE	GKSKDKPAKRKKLEPDA-KSPSHSAG----------------GRP-----
CIOIN	SLPSPMNPPMNTPQPLPITSPMH---------------------------
ANOGA	SSPKHSGSG----GPLGFHSPKHGSSVSPKHPLHSGGSSTGGGKPSMSAL
TETNG	GKCKEKPPKRKKVDSDG-KSPSHSSG----------------GRP-----
DROME	SSPKHNTASNSPKSPFGTHSPKHGSS----------------GKPSMSTL
BRUMA	RKAAHMPASPAHSHQFALNRPMLQRSFSEMPV----------YPS-----
CAEEL	QMSQYQMQQYQQNQQFRMHQ-MQQQQQQQFQM----------QSP-----
YEAST*	SITEAMMLKEEGLQQFNLHEILSEPAIEEENG------------------
SCHPO	--
CANAL	--
CANGA	SMSEAVVFKDEGLQQFSLHEIMAD--------------------------
ASPNI	--
RHIO9	--
CRYNE	--
USTMA	--
	1810 1820 1830 1840 1850

HUMAN*	----------FTPPT---------STGGSKSPGSAG-----RSQTPPGVA
CHICK	----------FTPPA---------STGGSKSPGSSG-----RSQTPPGVA
XENTR	----------FTPPT---------STGGSKSPGTSG-----RSQTPPGMA
DANRE	----------YTPPCGSATSVGLVSTGGSKSPGSVG-----RSQTPPGGA
CIOIN	--
ANOGA	KSAAASG---SSPGSKSSDVGG-------GGGGTSGA-------------
TETNG	----------YTPPSGGSGSGGSMSGGGSKSPGSSG-----RSQTPPGGA
DROME	KSAATAATILSPKGDKSSSAVGNTSSGPSASSGSSGATGLVRSFASVGAP
BRUMA	----------QMPPVSLSQENRSEASEFDGTEESSD-----------DET
CAEEL	----------KNPLVPAY------------TDEDSD-----------EEC
YEAST*	--
SCHPO	--
CANAL	--
CANGA	--
ASPNI	--
RHIO9	--
CRYNE	--
USTMA	--
	1860 1870 1880 1890 1900

HUMAN*	TPPIPKITIQIPKGTVMVGKPSSHSQYTSSGSVS--------SSGSKSHH
CHICK	TPPIPKITIQIPKGTVTVGKPSSHGQYTSSGSVT--------SSSSKSHH
XENTR	TPPIPKITIQIPKGTMSVGKPSSHSQYSSSGSSS--------SSSSKSHH
DANRE	TPPIPKITIQIPR-ALTGGKTSSHGGYTSSSSTSGGTGNTIGTSNSKSHH
CIOIN	--
ANOGA	--------------SISTAGSSSKERDKERSEKKALHAAGSSSSSSSG--
TETNG	TPPIPKITIQITKGTITGGKTSSLSGYTSSSSATGSTGGT-GGSSSKSHH
DROME	PPPPPIPPLASSSGSISSSQSLKKEKTSSASGSSSTSSSATAGVASGG--
BRUMA	DPPPPVCTTAIPQP----S-----SQLTVSSSATVPQTTVVGNLSSSS--
CAEEL	DPPPP------PKPQVSTS-----SRMSSVPSHPPPPPQQLGNPMVGY--
YEAST*	--
SCHPO	--
CANAL	--
CANGA	--
ASPNI	--
RHIO9	--
CRYNE	--
USTMA	--
	1910 1920 1930 1940 1950

HUMAN*	SH--SSSSSSSASTSGKMKSSKSEGSSS----SKLSSS---MYSSQGSSG
CHICK	SHSSSSSSSSSSTSGKIKSSKSEGSSG----SKMSSS---LYSSQGGSS
XENTR	GH--SSLSSSA---SGKIKSNKSDGSSG----MKIGSSGGGMYSGQSGQS
DANRE	S---SS--------SGKIKSSKLESSVGQGNIAKQSSA-----AGTGSGI
CIOIN	--
ANOGA	----GRSKNS------SLKLKQLDLAAG-SSMS-ISMAAGIGD------A
TETNG	SH--SS--------SGKIKSK--ESSATPGSTSKPGS------AGLAGGV
DROME	----GISPASVAAAVAALKSSQQQMKSV-ASLSHLAAGGGLGSYAAPSGA
BRUMA	--------------------------------ATLPMVNKCSSLTTASCL
CAEEL	--------------------------------PGMP-------LQSPNHL
YEAST*	--
SCHPO	--
CANAL	--
CANGA	--
ASPNI	--
RHIO9	--
CRYNE	--
USTMA	--
	1960 1970 1980 1990 2000

MED1

```
HUMAN*  SSQSKNSSQSG--GKPGSSPITKHGLSS--------GSSSTKMKPQG--K
CHICK   SGPSKSSAQSV--GKPGSSPITKHGLSS--------GSGSTKMKPQG--K
XENTR   NSQSKNSSQSM--GKAGSSPITKHGLSSN-----VNNSSGSKTKPQG--K
DANRE   PSQSKVSSMGMGTGKPSSSPITKHGLSSSGGGGSSGSGSGIKNKPQGGKP
CIOIN   -----------------------------------------------PAM
ANOGA   GSSSPVAPMIDLTGPVPGAAAALGGL-----------------GLPGPGM
TETNG   PSQSKSASQGIGVGKPGSSPITKHGLSGP-GSSSSGMVGGNKTKPPGGKP
DROME   GASGAAAVVV---GAGAGAGAGASGLEL----------SALRKGMAGGAV
BRUMA   PSIPSSAPAAELTPPTSSSPTSTSNSSMQSAALLAHKARKGSLEAVVGKL
CAEEL   PLTPSPLSA----PPKPFSPEQQHHFGM--------KMRETSYWKELERL
YEAST*  ---------------------------------------------DSIKE
SCHPO   --------------------------------------------------
CANAL   ------------------------------------LAFESQFKSEEVYI
CANGA   --------------------------------------------------
ASPNI   -----QAPLIMLLFTVNRASSSIHGPEHSVSKVSIAFEIGLNARVSVVEV
RHIO9   --------------------------------------------------
CRYNE   --------------------------------------------------
USTMA   ----NGATVNFDASISPSKPTSEVGWLL----------------------
                 2010      2020      2030      2040      2050
```

```
HUMAN*  PSSLMNPSLSKPNISPSHSRPPGGSDKLASP--MK--PVPGT---PPSSK
CHICK   PSSLMNPSMSKPNISPSHSRPSGGSDKLASP--MK--PVPGT---PPSSK
XENTR   PSVLMNPSLSKPNISPSHSRPSGGSDKLSSP--MK--PMPGT---PPSSK
DANRE   PGSLMNPNI-KPNISPSHSRSGGS-DKLSSP--MKQGQVPGT---PPSSK
CIOIN   TPPLTSPAPRKGSVSRRASRQKSNSIEATTSIDM----------------
ANOGA   TTASSMLQALQAAKNRKTPTAAGGSTGLGGSLSAVIDKLKSA---QSADD
TETNG   PGSLMNPNM-KPNISPSHSRSSTSGEKMSSPLKMQQSQVPGT---PPSFK
DROME   SLMTSTAALAPTIPAPTTTVAAGSAASLVSPVSAVVG--QGQ---ETAGA
BRUMA   HCK----------TAPASSSSSTTSNPYLASDLYDDESEPVSKPPEPVPA
CAEEL   ETKPDVEKLKQQLSSSSSSSQAEASDPPSTSTEAQDPPKPSSSSAPPPPK
YEAST*  HSTTMDGANDLGFTASVSNQENAGTDIVMEDHGVLQGTSQNY--------
SCHPO   --------------------------------------------------
CANAL   PFRISNGEIVKLQNSDDMETDDTPSSKFIKALNLTEDIISSYKAVYSN--
CANGA   -TTTEPDQKPDNLNISNNSESFIGQQELMEGSNIINDDKMDI--------
ASPNI   TGLLDDENSSTSNASPDTQAAEDPTRELQAKLAYVLETSQDI--------
RHIO9   ---------------RMSQESFIKRERMDIDDDLFKKSIKTE--------
CRYNE   ---------------QRTKQS-----------------------------
USTMA   --SAVAESDASGKTASKQARLESSSSEAQSMVAELRDLDSLE--------
                 2060      2070      2080      2090      2100
```

```
HUMAN*  AKS------------------PISSGSGGSHMSGTSSSSGMKSSSGLGSS
CHICK   AKS------------------PISSGSGGSHMSGTGSSSSMKSSSGMGSS
XENTR   AKS------------------PI--GSGGQHLSGSGSNSTTKSSSGLVSS
DANRE   AKS------------------PIGS-GSGSGGNKCSSGGG------VG-S
CIOIN   --------------------------------------------------
ANOGA   EAVLILPEMTITQQTTSLAAGTGGIGTGVNIGGFGGLKDSAKSGSGLLPS
TETNG   AKS------------------PIGSGGSSSAGSKLSSGPGMCSQKPMG-G
DROME   AAAATLATATILQQQPQ----PGAAPTSSCLTTSGGSSDSAGS---INPA
BRUMA   KR---------------------------DSPSLSTSSPGARMSPNLAKM
CAEEL   KKLGLEAAISKIRGQQEQALQKQLQQQESVESELATPHPGSSGAPPLAPH
YEAST*  --------------------------------------------------
SCHPO   --------------------------------------------------
CANAL   --------------------------------------------------
CANGA   --------------------------------------------------
ASPNI   --------------------------------------------------
RHIO9   --------------------------------------------------
CRYNE   --------------------------------------------------
USTMA   --------------------------------------------------
                 2110      2120      2130      2140      2150
```

```
HUMAN*  GSL---------SQKTPPSSNSCT------ASSSSFSSSGSSMSS-----
CHICK   GSM---------SQKPPPSSNSST------ASSSSFSSSGSSMSS-----
XENTR   GSL---------TQKPNSSSSSSS------SSSSSSSSSSSSSSSFSSGV
DANRE   KSL---------SSSSSNSASTSC------ASSTGSSSSGSM--PFSSN-
CIOIN   --------------------------------------------------
ANOGA   GGT---------SSLANCVSSTAG----IAGAGKNKSVLSSSNSSFNPCF
TETNG   ISS---------SGTTLSSSSSSS------TSSSSSSSSGSM--TFLGG-
DROME   GAS---------TEYMVKPSSQEGLKLTINKTGSSKSSGTGSGSS-----
BRUMA   NA----------EQPGYGSELKIMIKLKQQASTRPSTGPSQSSSGGVPPS
CAEEL   QVNRARNLNDIFDDEADDSSPTADIKPSLQALQKPTDTSSQSTSSEPPPK
YEAST*  --------------------------------------------------
SCHPO   --------------------------------------------------
CANAL   --------------------------------------------------
CANGA   --------------------------------------------------
ASPNI   --------------------------------------------------
RHIO9   --------------------------------------------------
CRYNE   --------------------------------------------------
USTMA   --------------------------------------------------
                 2160      2170      2180      2190      2200
```

MED1

```
HUMAN*  SQNQHGS-------------------------------------------
CHICK   SQNQHGS-------------------------------------------
XENTR   SQNLHSS-------------------------------------------
DANRE   GQSQYGSSGGNCSGGG-----GGGGCG-----------------------
CIOIN   --------------------------------------------------
ANOGA   slthls--------------------------------------------
TETNG   SQSQYGGGGSGGGGGGGSTTTGGGGCGGSGSGGGGGGGGGGGGGGGGGTV
DROME   SSSGLQ--------------------------------------------
BRUMA   MR------------------------------------------------
CAEEL   KE------------------------------------------------
YEAST*  --------------------------------------------------
SCHPO   --------------------------------------------------
CANAL   --------------------------------------------------
CANGA   --------------------------------------------------
ASPNI   --------------------------------------------------
RHIO9   --------------------------------------------------
CRYNE   --------------------------------------------------
USTMA   --------------------------------------------------
                            2210      2220      2230      2240      2250
```

```
HUMAN*  ----------SKGKSPSRNKKPSLTAVIDKLKHGVVTSGPGGEDPLDGQM
CHICK   ----------SKGKSPSRNKKPSLTAVIDKLKHGVVTSGPGGDDPMDGQM
XENTR   ----------SKGKSPSRNKKPSLTAVIDKLKHGVGTGGPGSEDPMDGGV
DANRE   ------NNPNAKGKSPSRNKKPSLTAVIDKLKS-VGSGGNGGEEIESCGG
CIOIN   --------------------------------------------------
ANOGA   ----------altqsssatsAPSLTAALTSGKASDGAFPSSTKSP-----
TETNG   AGQNNANNPNAKGKSPSRNKKPSLTAVIDKLKS-VGGGGVGEDGCEVGPP
DROME   ----------AKAKSSSSGATSFAGSTGSTKKQHTGLKPGVNSGPASKKA
BRUMA   ----------LVGQPPSSS-------------------------------
CAEEL   ----------PADEQPEREKEKLILKIPKMLKPVVDDRREDRKERDRDRD
YEAST*  --------------------------------------------------
SCHPO   --------------------------------------------------
CANAL   --------------------------------------------------
CANGA   --------------------------------------------------
ASPNI   --------------------------------------------------
RHIO9   --------------------------------------------------
CRYNE   --------------------------------------------------
USTMA   --------------------------------------------------
                            2260      2270      2280      2290      2300
```

```
HUMAN*  -GVSTNSSSHPMS----------------S-----------KHNMSG--G
CHICK   -GPSSNSSSHTMS----------------S-----------KHNMSG--G
XENTR   GGGSTGAPSHGMS----------------S-----------KHGLGV--G
DANRE   SSGVGQQNTVPTS----------------S-----------KHSMSSQGG
CIOIN   --------------------------------------------------
ANOGA   --HYTSSTSGSSKAPFQKSNSFGSL-------GGSGGTGL-KASKVSSPK
TETNG   VSGPG-SGTVPTGGGPGNVPSSGPLNICPS-----------KHISTSQSG
DROME   TAAVSSATASSSKHFFQKANSSGNLSSKLSGSGSGGGIPLTKSNSTNSFQ
BRUMA   ------------RSKEEKAL---------------------LRQKQLKEK
CAEEL   RDRDRDEDREKVRDKEDKA----------------------QREKDKKEK
YEAST*  --------------------------------------------------
SCHPO   --------------------------------------------------
CANAL   --------------------------------------------------
CANGA   --------------------------------------------------
ASPNI   --------------------------------------------------
RHIO9   --------------------------------------------------
CRYNE   --------------------------------------------------
USTMA   --------------------------------------------------
                            2310      2320      2330      2340      2350
```

```
HUMAN*  EFQGKREKSDKD-KSKVSTSGSSVDSSKKTSESK---------NVGSTGV
CHICK   EFQGKREKSDKE-KSKVSVSGGSVDSSKKTSDSK---------NVGSTGV
XENTR   EFPTKREKGEKD-KSKGSSGGPSDPSKKGGGDSK---------GSVSTGV
DANRE   EFLSKRDKIDKDGKSKMSGSGGN-SSDKKMIDAKS-------NTMTGTGV
CIOIN   --------------------------------------------------
ANOGA   STSNPASSSSSSAYSSKEKGGST-SGRSKSSSASSLTGAMLTGATGGTGS
TETNG   EY--KREKSEKEAKAKVSVSGGN-SGDKKLMDPKA-------GGVGTSGL
DROME   EHNAPRRRPSMGALASGSSGGG--SGQRKLGSASG-------GGSGSSGS
BRUMA   NRSAPDERARKAGKRERKGELSSSSSKKLKL--------DRPLSEQKTAT
CAEEL   ERERRRQRDRDR-TEQKKSDREKEPSKKRKLEKKDEKEKDRREPERKKGK
YEAST*  ---------GTATADDADIEMKDVSSKpskpessvlqlivsedhiildti
SCHPO   --------------------------------------------------
CANAL   --------------------------------------------------
CANGA   ---------DEEAINDDD------SDSQSGEEGNIPvllvnedhvsfkgl
ASPNI   --------------------------------------------------
RHIO9   --------------------------------------------------
CRYNE   --------------------------------------------------
USTMA   --------------------------------------------------
                            2360      2370      2380      2390      2400
```

MED1

```
HUMAN*  A - - - - - - - - - - - - - - - - - - K I I I S K H D G G S P S I K A K V T L Q K P G E S S G E - G
CHICK   A - - - - - - - - - - - - - - - - - - K I I I S K H D G G S P S I K A K V T L Q K P G E G G G D - S
XENTR   A - - - - - - - - - - - - - - - - - - K I I I S K H D G G S P S I K A K V T L Q K P - E G G G E - G
DANRE   A - - - - - - - - - - - - - - - - - - K I I I S K P E G G S P S I K S K V T L Q K P G E G S G D G G
CIOIN   - - - - - - - - - - - - - - - - - - - - - - - - - - - - - - - - - - - - - - - - - - - Q D L T D I G
ANOGA   G T G S S S S A L V N P - - - - - - - I S I M K M L G Y P T G S M - G N M - - - - - - E G F A K - S
TETNG   A - - - - - - - - - - - - - - - - - - K I I I S K P D G G S P S I K A K G P L Q K A G E G S G D - S
DROME   V S P A L S G S M S Q P P P R F D H H T D M M T I L Q Y A S P T M A A S M - - - - - - E G F I K - G
BRUMA   I - - - - - - - - - - - - - - - - - - S P K V E F P Q A - - - - - L A F A S L K N - - - - - - - - -
CAEEL   S - - - - - - - - - - - - - - - - - - D G K - E Y S K A S T T S L I P T L S L K N - - - - - - - - -
YEAST*  S E C N L Y D D V K C W S K F I E K F Q D I V S - - - - - - - - - - - - - - - - - - - - - - - - - -
SCHPO   - - - - - - - - - - - - - - - - - - - - - - - - - - - - - - - - - - - - - - - - - - - - - - - - - -
CANAL   - - - - - - - - - - - - - - - - - - - - - - - - - - - - - - - - - - - - - - - - - - - - - - - - - -
CANGA   G S C S F Y D D K D K W A K F V E D L F N V F S - - - - - - - - - - - - - - - - - - - - - - - - - -
ASPNI   - - - - - - - - - - - - - - - - - - - - - - - - - - - - - - - - - - - - - - - G V L V E W V L R W L
RHIO9   - - - - - - - - - - - - - - - - - - - - - - - - - - - - - - - - - - - - - - - - - - - - - - - - - -
CRYNE   - - - - - - - - - - - - - - - - - - - - - - - - - - - - - - - - - - - - - - - - - - - - - - - - - -
USTMA   - - - - - - - - - - - - - - - - - - - D V V V G L L G W A E Q Q L E L R L K K P A A S D A A F D A L
                            2410                2420                2430                2440                2450
```

```
HUMAN*  L R P Q M A S S K N Y G S P L I S G S T P K H E R G S P S H S K S P A Y T P Q N L - - - - - - - - -
CHICK   L R P Q M A S S K S Y G S P L I S G S T P K H E R C S P S H S K S P A Y T P Q N I - - - - - - - - -
XENTR   L R S Q M - - Q K N Y G S P L I S G S T P K H E R C S P S H N K S P A Y T P Q A L - - - - - - - - -
DANRE   I R S Q H S G H K - - A S P L F S G S T P K H D R S S P S H S R S P G C T P M N P - - - - - - - - -
CIOIN   F N Q Q F D V M N - - - - - - - - - - - - - - - - - T E D M N N P M A F T P Q N P G I N F D - - - -
ANOGA   L D T K F Q I P K - - L S A R - - - S N S S A T G T A S T G S S E S G E A G S K S A K D P A A R K Q
TETNG   V R P Q I S S V K - - A S P L F S G S T P K H D R S S P S H S R S P G Y T P L N H - - - - - - - - -
DROME   L H N K F Q I P K - - L S Q R G S G G N T T S G R S T P S G S S E P A L A G T S S - - - - - - - - - -
BRUMA   - - - - F K I P K - - - V M E N E - - - - K C E S P T P T T T T T T P A V A T P S S - - - - - - - -
CAEEL   - - - - F R I P K - K D T V E E E K K D V K E E A P G P S S S T E S K K E P P S A - - - - - - - - - - -
YEAST*  - - - - - - - - - - - - - - - - - - - - - - - - - - - - - - - - - - - - - - - - - - - - - - - - - -
SCHPO   - - - - - - - - - - - - - - - - - - - - - - - - - - - - - - - - - - - - - - - - - - - - - - - - - -
CANAL   - - - - - - - - - - - - - - - - - - - - - - - - - - - - - - - - - - - - - - - - - - - - - - - - - -
CANGA   - - - - - - - - - - - - - - - - - - - - - - - - - - - - - - - - - - - - - - - - - - - - - - - - - -
ASPNI   S A N N I P I E K - - - - - - - - - - - - - - - - - - - - - - - - - - - - - - - - - - - - - - - - -
RHIO9   - - - - - - - - - - - - - - - - - - - - - - - - - - - - - - - - - - - - - - - - - - - - - - - - - -
CRYNE   - - - - - - - - - - - - - - - - - - - - - - - - - - - - - - - - - - - - - - - - - - - - - - - - - -
USTMA   S Y E Q Q N V H A - - - - - - - - - - - - - - - - - - - - - - - - - - - - - - - - - - - - - - - - -
                            2460                2470                2480                2490                2500
```

```
HUMAN*  - - - - - - - - - - - - - - - - - - - - - - - D S E S E S G - S S I A E K S Y Q N S P S S D D G - -
CHICK   - - - - - - - - - - - - - - - - - - - - - - - D S E S E S G - S S I A E K S Y Q N S P S S D D G - -
XENTR   - - - - - - - - - - - - - - - - - - - - - - - D S E S E S G S S S I A E K S Y Q N S P S S D D G G -
DANRE   - - - - - - - - - - - - - - - - - - - - - - - D S E S E S G S S S V A E K S H Q N S P S S D D D Q -
CIOIN   - - - - - - - - - - - - - - - - - - - - - - - - - - - - - - - - - - - - - - - - - - - - - - - - - -
ANOGA   K Q Q T P V T T A A P S V G S S P V R Q L S S T S P S G E A S A R L L D G S L Q T K S N S S D G S M
TETNG   - - - - - - - - - - - - - - - - - - - - - - - D S E S E S G S S S V A E K S H Q N S P S S D D D Q -
DROME   - - - S I L G P I A S S T G L T E P E A K P P V P P S Q S G N E G L L N L S S T A G T P S A D G I -
BRUMA   - - - - - - - - - - - - - - - S G A H L N V G Q S L S S S T M Q V V S S T S L S A A T T A T T V T A
CAEEL   - - - - - - - - - - - - - - - A P I - - - - - - T R K E S T T A P V A P A V Q Q Q Q Q R K E S F T A
YEAST*  - - - - - - - - - - - - - - - - - - - - - - - - - - - - - - - - - - - - - - - - - - - - - - - - - -
SCHPO   - - - - - - - - - - - - - - - - - - - - - - - - - - - - - - - - - - - - - - - - - - - - - - - - - -
CANAL   - - - - - - - - - - - - - - - - - - - - - - - - - - - - - - - - - - - - - - - - - - - - - - - - - -
CANGA   - - - - - - - - - - - - - - - - - - - - - - - - - - - - - - - - - - - - - - - - - - - - - - - - - -
ASPNI   - - - - - - - - - - - - - - - - - - - - - - - - - - - - - - - - - - - - - - - - - - - - - - - - - -
RHIO9   - - - - - - - - - - - - - - - - - - - - - - - - - - - - - - - - - - - - - - - - - - - - - - - - - -
CRYNE   - - - - - - - - - - - - - - - - - - - - - - - - - - - - - - - - - - - - - - - - - - - - - - - - - -
USTMA   - - - - - - - - - - - - - - - - - - - - - - - - - - - - - - - - - - - - - - - - - - - - - - - - - -
                            2510                2520                2530                2540                2550
```

```
HUMAN*  - - - - - - - - - - - - - - - - - - - - - - - - - - - - - - - - - - - - - - I R P L P E Y - - - - -
CHICK   - - - - - - - - - - - - - - - - - - - - - - - - - - - - - - - - - - - - - - I R P L P E Y - - - - -
XENTR   - - - - - - - - - - - - - - - - - - - - - - - - - - - - - - - - - - G S G - S R A Q T E Y - - - - -
DANRE   - - - - - - - - - - - - - - - - - - - - - - - - - - - - - - - - - - T M R L H Q P I Q D Y M S S I S
CIOIN   - - - - - - - - - - - - - - - - - - - - - - - - - - - - - - - - - - - - - - - - - - - - - - - - - -
ANOGA   F P T A G R A S P G M L S G S A S R D G F R S G G G S G S A P G T P T T T S S M V L P P F P S P I S
TETNG   - - - - - - - - - - - - - - - - - - - - - - - - - - - - - - - - - - T M R - P L P P Q D Y L S S I P
DROME   - - - - - - - - - - - - - - - - - - - - - - - - - - - - - - - - - - - - - - - - - - - - - - - - - -
BRUMA   T S S - - - - - - - - - - - - - - - - - - - - - - - - - S S S A G T S S - - I F P - - - - - - - - -
CAEEL   L P S L P E Q Q Q H H R E P L L K K K P P L Q P P P P P Q M T G P P I G - - I Y P G P P A P G N L P
YEAST*  - - - - - - - - - - - - - - - - - - - - - - - - - - - - - - - - - - - - - - - - - - - - - - - - - -
SCHPO   - - - - - - - - - - - - - - - - - - - - - - - - - - - - - - - - - - - - - - - - - - - - - - - - - -
CANAL   - - - - - - - - - - - - - - - - - - - - - - - - - - - - - - - - - - - - - - - - - - - - - - - - - -
CANGA   - - - - - - - - - - - - - - - - - - - - - - - - - - - - - - - - - - - - - - - - - - - - - - - - - -
ASPNI   - - - - - - - - - - - - - - - - - - - - - - - - - - - - - - - - - - - - - - - - - - - - - - - - - -
RHIO9   - - - - - - - - - - - - - - - - - - - - - - - - - - - - - - - - - - - - - - - - - - - - - - - - - -
CRYNE   - - - - - - - - - - - - - - - - - - - - - - - - - - - - - - - - - - - - - - - - - - - - - - - - - -
USTMA   - - - - - - - - - - - - - - - - - - - - - - - - - - - - - - - - - - - - - - - - - - - - - - - - - -
                            2560                2570                2580                2590                2600
```

MED1

```
HUMAN*  --STEKHKKHKKEKKK---VKDK--DRDRDRDKDRDKKK--------SHS
CHICK   --SSEKHKKHKKEKKK---VKDKDRDRDRDRDKDRDKKK--------SHS
XENTR   --SAEKHKKHKKEKKR---LKDK--------DRDREKKK--------SYT
DANRE   ISQSEKHKKHKKEKKK---QKDR--ERDKERDKEKEKKK-SMSCGP-SHT
CIOIN   --------------------------------------------------
ANOGA   GGASNDGDSGTLMRPPSRPSSTISNHSHSSQDGIGGGSGNSGDAVAGSTP
TETNG   LSSGEKHKKHKKEKKKKERERER--DRDRERDRDKEKKKSSMSMVPSSHP
DROME   --------------------------------------------------
BRUMA   --PKPKSILKSPAEPPPSLFRG------------------MGGMPP----
CAEEL   GSSRPSGNRKQPLPPPPPMIRGPPPDQMYRDRSGGGGGSMRGGFPPASHY
YEAST*  --------------------------------------------------
SCHPO   --------------------------------------------------
CANAL   --------------------------------------------------
CANGA   --------------------------------------------------
ASPNI   --------------------------------------------------
RHIO9   --------------------------------------------------
CRYNE   --------------------------------------------------
USTMA   --------------------------------------------------
                 2610      2620      2630      2640      2650

HUMAN*  IKPESWSKSPIS-SDQSLSMTSNTILSADRPSR-----------------
CHICK   MKPESWSKSPIS-ADQSLSMASSAILSAERPSR-----------------
XENTR   MKPESWSKSPIS-ADPTMAMGGGAMMSSDRASR-----------------
DANRE   VKTDNWSRSPIS-SETSVSL-----LSSERSSR-----------------
CIOIN   --------------------------------------------------
ANOGA   AVASMASMMMMNAMSAAAGGGHTQQLIAALAAKSGVLPPP-PPNNLLNNH
TETNG   IKADSWSRSPISASDSSLSM-----LGPERQTR-----------------
DROME   ----------------------DEELLASLAGE-----------------
BRUMA   -----------------MLPGRRPLLPDPRNVMHH------PP--RNQFR
CAEEL   HGGTSGVNKQVASYAQGLPPGMGPPVVKPHGNNYQASQWVRPPTHRDSSH
YEAST*  --------------------------------------------------
SCHPO   --------------------------------------------------
CANAL   --------------------------------------------------
CANGA   --------------------------------------------------
ASPNI   --------------------------------------------------
RHIO9   --------------------------------------------------
CRYNE   --------------------------------------------------
USTMA   --------------------------------------------------
                 2660      2670      2680      2690      2700

HUMAN*  ------------------------------------------------L-
CHICK   ------------------------------------------------P-
XENTR   ------------------------------------------------P-
DANRE   ------------------------------------------------P-
CIOIN   --------------------------------------------------
ANOGA   HSNHLNNHVVGGAAALNNLPSPASVSVHLVKSPAPSPLPFASPHSNASS-
TETNG   ------------------------------------------------P-
DROME   --------------------------------------------------
BRUMA   PLVALPRMIPSVLSGANFMPPPSSSGRWMALAPQRQPSPRSSPSEQSAPS
CAEEL   SYHGMPSLGPPQLQREPPAPPP----------PQMIPLPKDPPVLREHPR
YEAST*  --------------------------------------------------
SCHPO   --------------------------------------------------
CANAL   --------------------------------------------------
CANGA   --------------------------------------------------
ASPNI   --------------------------------------------------
RHIO9   --------------------------------------------------
CRYNE   --------------------------------------------------
USTMA   --------------------------------------------------
                 2710      2720      2730      2740      2750

HUMAN*  SPDFMIGEEDDDLMDVALIGN-------
CHICK   SPEFLIGEEDDDLMDVALIGN-------
XENTR   TPSFLMG-DDDDLIDVPLTL--------
DANRE   SPVYMHT-EDDDLMDSALTGN-------
CIOIN   ----------------------------
ANOGA   THAAGAAGLDDDLMDEALIGIGSK----
TETNG   SPLYMRN-EDDDLMDSALTGNI------
DROME   ----------------------------
BRUMA   SGLSETATAMSKMQDSPTEALKIVADEE
CAEEL   EHRAHRGGADDDGPDSPEEGTLRIDDE-
YEAST*  ----------------------------
SCHPO   ----------------------------
CANAL   ----------------------------
CANGA   ----------------------------
ASPNI   ----------------------------
RHIO9   ----------------------------
CRYNE   ----------------------------
USTMA   ----------------------------
                 2760      2770
```

MED2

```
HUMAN*  --------------------------------------------------
CHICK   --------------------------------------------------
XENTR   --------------------------------------------------
DANRE   --------------------------------------------------
CIOIN   --------------------------------------------------
ANOGA   --------------------------------------------------
TETNG   --------------------------------------------------
DROME   --------------------------------------------------
BRUMA   MYGH----------------------------------------------
CAEEL   MSGQGPPPSMTPQQQHMLMQQQQQQQMMRQQQMQQQQMQQQRQQQIQQQA
YEAST*  --------------------------------------------------
SCHPO   MDAEGEQKSNGVKEPN----------------------------------
CANAL   --------------------------------------------------
CANGA   --------------------------------------------------
ASPNI   --------------------------------------------------
RHIO9   --------------------------------------------------
ARATH*  --------------------------------------------------
VITVI   --------------------------------------------------
SOYBN   --------------------------------------------------
SOLLC   --------------------------------------------------
ORYSJ*  --------------------------------------------------
HORVU   --------------------------------------------------
SORBI   --------------------------------------------------
SELML   --------------------------------------------------
PHYPA   --------------------------------------------------
                 10        20        30        40        50
```

```
HUMAN*  --------------------------------------------------
CHICK   --------------------------------------------------
XENTR   --------------------------------------------------
DANRE   --------------------------------------------------
CIOIN   --------------------------------------------------
ANOGA   --------------------------------------------------
TETNG   --------------------------------------------------
DROME   --------------------------------------------------
BRUMA   ----------QSIPRSVSAGQLNAMMSHPGMVITSQQMINTPQGVMVSQA
CAEEL   QQPYQRARTPQMVPQGGSPGGAHLQM-HPHLQPQGQMQPRSP--LVGAQL
YEAST*  --------------------------------------------------
SCHPO   --------------------------------------------------
CANAL   --------------------------------------------------
CANGA   --------------------------------------------------
ASPNI   --------------------------------------------------
RHIO9   --------------------------------------------------
ARATH*  --------------------------------------------------
VITVI   --------------------------------------------------
SOYBN   --------------------------------------------------
SOLLC   --------------------------------------------------
ORYSJ*  --------------------------------------------------
HORVU   --------------------------------------------------
SORBI   --------------------------------------------------
SELML   --------------------------------------------------
PHYPA   --------------------------------------------------
                 60        70        80        90       100
```

```
HUMAN*  --------------------------------------------------
CHICK   --------------------------------------------------
XENTR   --------------------------------------------------
DANRE   --------------------------------------------------
CIOIN   --------------------------------------------------
ANOGA   --------------------------------------------------
TETNG   --------------------------------------------------
DROME   --------------------------------------------------
BRUMA   GYPQGMINTAQP--PQMMPSQTHISQQM--------------------IN
CAEEL   QAPSSVPTAANPTTPQMMQQQMGMNQPMSLPPTHVSRPGSVAPPSSVPVN
YEAST*  --------------------------------------------------
SCHPO   --------------------------------------------------
CANAL   --------------------------------------------------
CANGA   --------------------------------------------------
ASPNI   --------------------------------------------------
RHIO9   --------------------------------------------------
ARATH*  --------------------------------------------------
VITVI   --------------------------------------------------
SOYBN   --------------------------------------------------
SOLLC   --------------------------------------------------
ORYSJ*  --------------------------------------------------
HORVU   --------------------------------------------------
SORBI   --------------------------------------------------
SELML   --------------------------------------------------
PHYPA   --------------------------------------------------
                110       120       130       140       150
```

MED2

```
HUMAN*   ----------------MLKSNGERRSRNALPAVYARKMAASQQQA-----
CHICK    -------------------------------------MAAPPQPQ-----
XENTR    -------------------------------------MAMLLNQS-----
DANRE    -------------------------------------MAS----------
CIOIN    --------------------------------------------------
ANOGA    --------------------------------MMNQMGMMMQQQG-----
TETNG    -------------------------------------MAS----------
DROME    --------------------------------------------------
BRUMA    -THMGVVQGL--NQQGHMNVQHQQMQQMQQVQMAPQQA-YIQQQG-MSHP
CAEEL    LQHTSGAPGLPGSQMEHQYPMHLQPQQQTLSRPGSQQSQHIQQPGSIQRP
YEAST*   --------------------------------------------------
SCHPO    --------------------------------------------------
CANAL    --------------------------------------------------
CANGA    --------------------------------------------------
ASPNI    --------------------------------------------------
RHIO9    --------------------------------------------------
ARATH*   --------------------------------------------------
VITVI    --------------------------------------------------
SOYBN    --------------------------------------------------
SOLLC    --------------------------------------------------
ORYSJ*   --------------------------------------------------
HORVU    --------------------------------------------------
SORBI    --------------------------------------------------
SELML    --------------------------------------------------
PHYPA    --------------------------------------------------
                             160                 170                 180                 190                 200
```

```
HUMAN*   -------------SAASSAAGVSGPSSAGGPG------------------
CHICK    -------------PGPPAAPAPPPLSAAPGPG------------------
XENTR    ----------------------QPPQGREGGG------------------
DANRE    --------------------------------------------------
CIOIN    --------------------MNFRPTSNPGGNPQNIMPVRHPQPQQIVST
ANOGA    -------------VGVPGGPGGVGGVGMPGPGGVGVAPGMMQSPQMQQAQ
TETNG    --------------------------------------------------
DROME    --------------------FFFTKRKQFAAVQYFSPPISIDFECQMNPN
BRUMA    NFVNGQARSPAVGP----GPQGISQQGTPGR---PSSTSGPPSNNPQTPL
CAEEL    GSVLAPGSIPPGGPASQTGPQSIQVFG-PGSVQPPGSTQAPSSVQPASTF
YEAST*   --------------------------------------------------
SCHPO    --------------------------------------------------
CANAL    --------------------------------------------------
CANGA    --------------------------------------------------
ASPNI    --------------------------------------------------
RHIO9    --------------------------------------------------
ARATH*   --------------------------------------------------
VITVI    --------------------------------------------------
SOYBN    --------------------------------------------------
SOLLC    --------------------------------------------------
ORYSJ*   --------------------------------------------------
HORVU    --------------------------------------------------
SORBI    --------------------------------------------------
SELML    --------------------------------------------------
PHYPA    --------------------------------------------------
                             210                 220                 230                 240                 250
```

```
HUMAN*   --------------PQQQPQPPAQLVGP-----AQ----SGLLQQ--QQQ
CHICK    -----------------QGAGPGPPLPAQ------------VAAA--QAQ
XENTR    --------------TQVGSLGPGIPVGQQ----QQ----QLGLQQ--QQQ
DANRE    ---------------QQMSTNNAQMSAQQAAVLQQ-----TQAQQLSQQQ
CIOIN    MNRMPTPAAVPMGTNVQNVRLRGAGTFPQ-RLPNQSSNTQQASAG--NAQ
ANOGA    QQQVQQQQVQQQQVQQQQVQQQQQQVQQQQQQQQQHSQSAQQQAQ--QTE
TETNG    ---------------QQQQAQPGGPMAQPG--LQQ----PSALQQLSQQQ
DROME    MNMMPMSGPQMMQVMQSSPSGPPGPVQHQQQQPPQ----PLQQQQ--QAE
BRUMA    NVMSTLNPSSQQVSTPATPQQAPSSVQSTTTQDNISTPTIVPP-------
CAEEL    NPGSIQAPASQQ--PPASVQPPPSAASGSTVAGAQSSKEPLKPNEEQIRM
YEAST*   ----------------------------------MVVQNSPVSSVHTANF
SCHPO    --------------------------------------------------
CANAL    --------------------------------------------------
CANGA    --------------------------------------------------
ASPNI    --------------------------------------------------
RHIO9    --------------------------------------------------
ARATH*   --------------------------------------------------
VITVI    --------------------------------------------------
SOYBN    --------------------------------------------------
SOLLC    --------------------------------------------------
ORYSJ*   --------------------------------------------------
HORVU    -------------------------------------------------M
SORBI    --------------------------------------------------
SELML    --------------------------------------------------
PHYPA    --------------------------------------------------
                             260                 270                 280                 290                 300
```

MED2

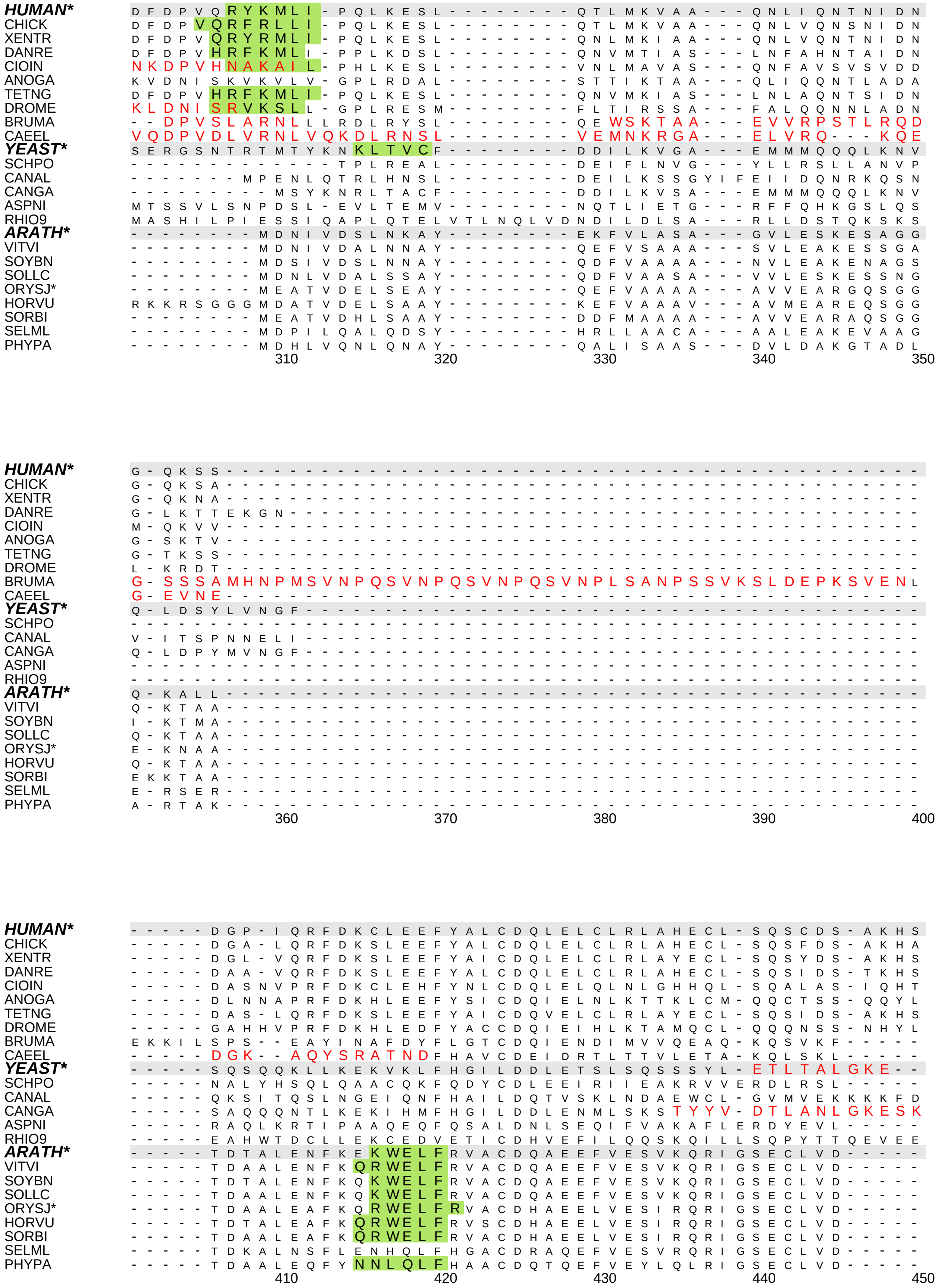
HUMAN*
CHICK
XENTR
DANRE
CIOIN
ANOGA
TETNG
DROME
BRUMA
CAEEL
YEAST*
SCHPO
CANAL
CANGA
ASPNI
RHIO9
ARATH*
VITVI
SOYBN
SOLLC
ORYSJ*
HORVU
SORBI
SELML
PHYPA
310
320
330
340
350
360
370
380
390
400
410
420
430
440
450

MED2

```
HUMAN*  PTLVP-TATKPDAVQ-------------PDSLPYPQYLAVIKAQISCAKD
CHICK   PALVP-AAPKGEGAA-------------GESLPYTQYLPLIKAQIAGAKD
XENTR   PTLVP-TATKPDAVQ-------------TESLPYTQYLSMIKSQISCAKD
DANRE   PNLVP-TATKPDTVQ-------------TESLSYSQYLSMIKSQISCAKD
CIOIN   PLMRN-VNLKDDA---------------NGPQIYGNYINTVESQLQCAEE
ANOGA   PIPV--ATSQPPLPE-------------TNALTYNQYLEVVKLQIGYAKD
TETNG   PNLVP-TATKPDTVQ-------------TESMSYGQYLGMIKSQITCAKD
DROME   PGPVTPMRMETFMPDN------------AGPISYPTYLNTVRVHIQSAKD
BRUMA   ----DKMF--AGELKI------------VGEMANVNYSQYAQGYVESTAR
CAEEL   ----DKVFFDRSSRDL------------DGEV----MVNSVQNFVDNTEI
YEAST*  ------------------------------KEKEREEAEKKRAEQENMRK
SCHPO   ----------------------------------------------EAKE
CANAL   ELKVKEEEARKKREEE---AKKKEEEAKKKEEEAKKKAEEAKKKAEEAKK
CANGA   RQKELELEKQREQEEEEKKQKLLELERKKKEQEEEEEKKKKQKEEEEKRK
ASPNI   KARKAALRSKRPADDVVMGEAKATVTSQPVSVSEQTAAGEIVPNPSESKP
RHIO9   PMEV------------------------DGGEEEGDMEELLQIQKDRLDR
ARATH*  --------------EATGLTTTAA-GGQAPAAVTGAATSLPPISAVRLEQ
VITVI   --------------EATG-SVAGK-PGQA------VTTGLPPISAVRLEQ
SOYBN   --------------EATR-PVAGK-PGQA------TMTGLPPISAVRLEQ
SOLLC   --------------EATG-TVSGK-PGQP------AASGLQPISAVRLEQ
ORYSJ*  --------------EATG-ASSSS-SA------ALAAPGIKPISAVRLEQ
HORVU   --------------EATG-SSSSS-SAPA--SVALAAPGIKPISAVRLEQ
SORBI   --------------EATG-SASAG-SA--------AAPGIKPISAVRLEQ
SELML   --------------EATG-PVSGRVVAGDAAKAGGGGAAVAPLSAVRLEQ
PHYPA   --------------EATG-SVTAK-VGKPGQDAADNRVAIPSLSALRLEQ
                   460       470       480       490       500

HUMAN*  IHTALL-------------DCANKVTGKTPAPPAGPGGTL----------
CHICK   IHNALL-------------EGANKITGKLAPTGGP---------------
XENTR   IHNALL-------------ECSKKIMGKTPNTQGGL--------------
DANRE   IHNALL-------------ECSKKIAGKGQGACN----------------
CIOIN   IQNLLL-------------NCCKKLQEHQTTHPV----------------
ANOGA   IHDTLI-------------CAAQNISPSE---------------------
TETNG   IHNALL-------------ECSKKIAGKIGLDY-----------------
DROME   IHDTLI-------------SAAQNISQAD---------------------
BRUMA   VRNAIT-------------RTLKNLNATMERMNAAKIVQKDIE-----EK
CAEEL   VQRMFD-------------DTIGSVTSSMESMRRRQKKWEDQHKNDDVEM
YEAST*  VREQ---------------EELKKRQELEEASQQQQLQQNSKEKNGL---
SCHPO   VEEKSQ-------------FASFVSATPPGANSLQGNVSLPSGNNFFTSS
CANAL   VEEAAK-------------KAEEAKKAEEEARKKAETVPQKFDNFDDFIG
CANGA   KELEEQ-------------ERKKKEQEEEEKRRRQQEQDGDKQQSMF---
ASPNI   VSDTVKIEQQPDLNGDEPGTANVPAKEEESHGAGAGTAPDFSGQNAGESE
RHIO9   LRNVIL-------------LGMDADKAANNHLDNKEDLLF----------
ARATH*  MSRAVR-------------WLVLELQRGSGVAPGSVH-------------
VITVI   MSKAVR-------------WLVIELQHGSGTAGGPSH-------------
SOYBN   MSKAVR-------------WLVIELQHGSGAGSANSA-------------
SOLLC   MSKAVR-------------WLVIELQHGSGANSASAV-------------
ORYSJ*  MSKAVR-------------WLVIELQHGAGGASAAGPGGGGGAAAAASGV
HORVU   MSKAVR-------------WLVIELQHGAGGPSAAGP--GGGVSTPAAGA
SORBI   MSKAVR-------------WLVIELQHGTSAQGAGGS---GGAATPNAGA
SELML   LSKAVR-------------WQVIDLQQGGGGGALSNAA------------
PHYPA   MSKAVR-------------WLVIELQQGATAGDNSAA-------------
                   510       520       530       540       550

HUMAN*  --------------------------------------------------
CHICK   --------------------------------------------------
XENTR   --------------------------------------------------
DANRE   --------------------------------------------------
CIOIN   --------------------------------------------------
ANOGA   --------------------------------------------------
TETNG   --------------------------------------------------
DROME   --------------------------------------------------
BRUMA   MAM-----------------------------------------------
CAEEL   ME------------------------------------------------
YEAST*  ---------------------GLNFSTTAPANTTDANGSKENYQELGSLQ
SCHPO   FDSENISKPGEVSLSESQLLGNLNQQSSIQLPDRMPKTTNGTL-------
CANAL   FDINDNTN-------DEDMLSNMDY-EDLKLDDKVPATTDNNL-------
CANGA   --------------------DGLDF-TNADLDTSQPGTSGQNDIKSPTMG
ASPNI   QMLYN---------------------------------------------
RHIO9   --------------------------------------------------
ARATH*  ---SSSTGFDSRFSEDSTQ-------------------------------
VITVI   --SHVSAPFDARFSEDAAQ-------------------------------
SOYBN   -LTHPSAPFDARFSEDAAQ-------------------------------
SOLLC   -HPNPSAPFDARFSEDAAQ-------------------------------
ORYSJ*  AGQHGHGGVDTRFPEDGAQ-------------------------------
HORVU   GGHHVHGGVESRFPEDGTQ-------------------------------
SORBI   GGQH---------PEEGGQ-------------------------------
SELML   --------------------------------------------------
PHYPA   --------MDIRTEDDANQ-------------------------------
                   560       570       580       590       600
```

MED2

```
HUMAN*  --------------------------------------------------
CHICK   --------------------------------------------------
XENTR   --------------------------------------------------
DANRE   --------------------------------------------------
CIOIN   --------------------------------------------------
ANOGA   --------------------------------------------------
TETNG   --------------------------------------------------
DROME   --------------------------------------------------
BRUMA   --------------------------------------------------
CAEEL   --------------------------------------------------
YEAST*  SSSQTQLENANAANNG--AAFSPLTTTRIQSQQAQPSDVMFNDLNSMDIS
SCHPO   ---------------------------------ADPNMPPKTTVNDQDVT
CANAL   --------------------------------------------------
CANGA   AGPQTAGTDKPNTADGPDKTNPPIAAFGLGDSQ---SGGLYNDLNTMDLS
ASPNI   -------------------------------------------------S
RHIO9   --------------------------------------------------
ARATH*  --------------------------------------------------
VITVI   --------------------------------------------------
SOYBN   --------------------------------------------------
SOLLC   --------------------------------------------------
ORYSJ*  --------------------------------------------------
HORVU   --------------------------------------------------
SORBI   --------------------------------------------------
SELML   --------------------------------------------------
PHYPA   --------------------------------------------------
                         610       620       630       640       650
```

```
HUMAN*  --------------------------------------------------
CHICK   --------------------------------------------------
XENTR   --------------------------------------------------
DANRE   --------------------------------------------------
CIOIN   --------------------------------------------------
ANOGA   --------------------------------------------------
TETNG   --------------------------------------------------
DROME   --------------------------------------------------
BRUMA   --------------------------------------------------
CAEEL   --------------------------------------------------
YEAST*  MFSGLDSTGFDSTAFNATVDETKGFDDNDSGNN-------YNDINISSIE
SCHPO   KDASQQFGGSNFSNYLRDNDSFQFGMNTDASGDAHLQASEFLLPNLFGNS
CANAL   ----------DMNNILENDESILDGLNMTLLDNGDHVNEEFDVDSFLNQF
CANGA   MFSELDGGGFDASGFDTAN-------------------------------
ASPNI   MLNNPEHNEFDLNLDFGGNNNNNNNNNNDNAANESFFNTTFGDTNPNSGL
RHIO9   --------------------------------------------------
ARATH*  --------------------------------------------------
VITVI   --------------------------------------------------
SOYBN   --------------------------------------------------
SOLLC   --------------------------------------------------
ORYSJ*  --------------------------------------------------
HORVU   --------------------------------------------------
SORBI   --------------------------------------------------
SELML   --------------------------------------------------
PHYPA   --------------------------------------------------
                         660       670       680       690       700
```

```
HUMAN*  --------------------------------------------------
CHICK   --------------------------------------------------
XENTR   --------------------------------------------------
DANRE   --------------------------------------------------
CIOIN   --------------------------------------------------
ANOGA   --------------------------------------------------
TETNG   --------------------------------------------------
DROME   --------------------------------------------------
BRUMA   --------------------------------------------------
CAEEL   --------------------------------------------------
YEAST*  NNINNNINSTKNGKDNNNESNKNNNGDEK-NKNNNEDNENNNNSSEKNNN
SCHPO   STSPQDNFGTSNSALSRNLDLFGSPSSENKSTAGSASLFPNQQMGIDLQA
CANAL   GN------------------------------------------------
CANGA   ---TSNANATTNSVPNNN------------NPATNDSNMNNDPTAAINAF
ASPNI   ESVNTQMPNTEPGQDNNALPTGGDAFDLELQKFSTQSGDANEQFGGNTED
RHIO9   --------------------------------------------------
ARATH*  --------------------------------------------------
VITVI   --------------------------------------------------
SOYBN   --------------------------------------------------
SOLLC   --------------------------------------------------
ORYSJ*  --------------------------------------------------
HORVU   --------------------------------------------------
SORBI   --------------------------------------------------
SELML   --------------------------------------------------
PHYPA   --------------------------------------------------
                         710       720       730       740       750
```

MED2

HUMAN*	--
CHICK	--
XENTR	--
DANRE	--
CIOIN	--
ANOGA	--
TETNG	--
DROME	--
BRUMA	--
CAEEL	--
YEAST*	NNNNNNNNNDDNGNNNNNNSGNDNNNTTNNDSNNKNNSITTGNDNENIVN
SCHPO	QENYSAAFGNDGSFASHLTNTFDNALNLPTDIPTTESINDLFGENFDFTM
CANAL	--
CANGA	DGTA-------AGNNETLGQGEKLEFDQSNPSAMLGNDINMG--------
ASPNI	IMGPGESSFDDLFMESENMGGNDNNDQDLLGGDGLMQLNELDDNWFT---
RHIO9	--
ARATH*	--
VITVI	--
SOYBN	--
SOLLC	--
ORYSJ*	--
HORVU	--
SORBI	--
SELML	--
PHYPA	--
	760 770 780 790 800

HUMAN*	--
CHICK	--
XENTR	--
DANRE	--
CIOIN	--
ANOGA	--
TETNG	--
DROME	--
BRUMA	--
CAEEL	--
YEAST*	NDLPTTVVSNPGDNPPPADNGEEYLTLNDFNDLNIDWSTTGDNGELDLSG
SCHPO	T----------------K--------------------------------
CANAL	--
CANGA	------------------DNGEDYLTLNDFNDLNIDWSAAGEGGDLDLNG
ASPNI	--
RHIO9	--
ARATH*	--
VITVI	--
SOYBN	--
SOLLC	--
ORYSJ*	--
HORVU	--
SORBI	--
SELML	--
PHYPA	--
	810 820 830 840 850

HUMAN*	---
CHICK	---
XENTR	---
DANRE	---
CIOIN	---
ANOGA	---
TETNG	---
DROME	---
BRUMA	---
CAEEL	---
YEAST*	FNI
SCHPO	---
CANAL	---
CANGA	FNI
ASPNI	---
RHIO9	---
ARATH*	---
VITVI	---
SOYBN	---
SOLLC	---
ORYSJ*	---
HORVU	---
SORBI	---
SELML	---
PHYPA	---

MED3

```
HUMAN*  MAD-VIN-------------------------------------------
CHICK   MAEGVLS-------------------------------------------
DANRE   MAD-VMN-------------------------------------------
CIOIN   MADTINQ-------------------------------------------
ANOGA   --------------------------------------------------
TETNG   MAD-GIN-------------------------------------------
DROME   --------------------------------------------------
BRUMA   --------------------------------------------------
CAEEL   MSGAGHSLNSDPVTTIATSSSEPVTSPSSASHVPVQTAPASHGAPSSVAS
YEAST*  --------------------------------------------------
SCHPO   MSLEEQRTRDE---------------------------------------
CANAL   --------------------------------------------------
CANGA   --------------------------------------------------
ASPNI   MAASSRLPIVVPKMEHSPSVKV----------------------------
RHIO9   --------------------------------------------------
ENCCU   --------------------------------------------------
ANTLO   --------------------------------------------------
ARATH*  -------------MQT---LHQ----------------------------
VITVI   -------------MQQ---QQQ----------------------------
SOYBN   -----------MQMQQ---TQQ----------------------------
SOLLC   -------------MQQSTLSSH----------------------------
ORYSJ*  -------------MQQ---AQA----------------------------
HORVU   ------------MMQQ---SQA----------------------------
SORBI   ------------MMQQQPQAHA----------------------------
                 10        20        30        40        50

HUMAN*  --------------------------------------------------
CHICK   --------------------------------------------------
DANRE   --------------------------------------------------
CIOIN   --------------------------------------------------
ANOGA   --------------------------------------------------
TETNG   --------------------------------------------------
DROME   --------------------------------------------------
BRUMA   --------------------------------------------------
CAEEL   QPPASVDGKAGAPDEARTASSASNPQGSEHPGSVDPAATTTAATTSASTT
YEAST*  --------------------------------------------------
SCHPO   --------------------------------------------------
CANAL   --------------------------------------------------
CANGA   --------------------------------------------------
ASPNI   ---------------------------------------------NELEI
RHIO9   --------------------------------------------------
ENCCU   --------------------------------------------------
ANTLO   --------------------------------------------------
ARATH*  ----------------------------------------SQLLQ-----
VITVI   ----------------------------------------TQAAR-----
SOYBN   ----------------------------------------QATATPFSTC
SOLLC   ----------------------------------------TSAVQ-----
ORYSJ*  ----------------------------------------SAVVA-----
HORVU   ----------------------------------------TAAVP-----
SORBI   ----------------------------------------SALAV-----
                 60        70        80        90       100

HUMAN*  --------------------------------------------------
CHICK   --------------------------------------------------
DANRE   --------------------------------------------------
CIOIN   --------------------------------------------------
ANOGA   --------------------------------------------------
TETNG   --------------------------------------------------
DROME   --------------------------------------------------
BRUMA   ----------------------MQSNLSSQQ-------------------
CAEEL   SITSTAATTTTTTTTGTTATGRVSPTIPPETL------------------
YEAST*  ----------------------MDSIIPAGVKLDDLQVILAKNENET---
SCHPO   --------------------------------------------------
CANAL   --MSDKPKNENKLPDILKIDEITSGKIDPQL-------------------
CANGA   ------------MATKQEEQANLSDVLTPSMSLTELEMKFADETDGKA--
ASPNI   DKDTTMAEPNPTAAPSAAPEDDVDPVIDPELLETELQ-------------
RHIO9   ----------------------MTTTRDPAT-------------------
ENCCU   ------------------MSKCFLLGINPSQDVFAFLQFHGHLFGIKFWG
ANTLO   -------------------------MSAPSSSGTRFLLQKKHICTFYRES
ARATH*  --NPAEA-----------ANNQSESDAPPKQ-------------------
VITVI   --NPTSA--------------ESTAEAPPKQ-------------------
SOYBN   TPPPSSG--------------GSASEAPPKQ-------------------
SOLLC   --GASAA-----------ESNEMSGDAPPKQ-------------------
ORYSJ*  --APVAAAAAAAAGATGHQHEGARGDAPPKQ-------------------
HORVU   --APATAA---------RAHEPAGGDAPPKQ-------------------
SORBI   --APSAAAATAA-----HPQDPAGGDAPPKQ-------------------
                110       120       130       140       150
```

MED3

```
HUMAN*   -----------------VSVNLEAFSQAISAIQALRSSV-SRVFDCLKDG
CHICK    -----------------PGVNLEAFSQAIAAIQALRSSV-TRVFDCLKDG
DANRE    -----------------VGVNLEAFSQAINAIQALRSSV-TRVFDFLKDG
CIOIN    -----------------AKLTISCIAQAITSTQQLRSTV-TNVFNLLCEG
ANOGA    -------------------MNLEPINNALSQLRVLRSSV-GQVFETLGTG
TETNG    -----------------VGVNLDAFAHAINGIQALRSSV-SRVFDFLKDG
DROME    ---------------------MDKLNSTLTAVKNLRSNV-RLCFEHLADG
BRUMA    -----------------FQSMTTHLDHCLNALRLLRNEV-ISVHRRVMEN
CAEEL    -----------------FHDAIKRCNNCLFLIRKLRNKQ-DHIARLFLDT
YEAST*   -----------------RDKVCKQINEARDEILPLRLQF-NEFIQIMANI
SCHPO    -----------------LKHKLLDLNQLHEQLAELRTIC-PSLLKLL---
CANAL    --------------------IYDEIERLKAEINTLRNDM-SLFLKALATI
CANGA    -----------------KDVVQARIKKAEDGILPLRLQF-NDFTQIMSSL
ASPNI    -----------------LVSSLAKLQKLEEMIHQLRTLLPERLLEPLAPI
RHIO9    -----------------IERDISQIDQALQQVSNLRAAL-KQFVQLV---
ENCCU    FLSGSMHLHHPWPRMRLAALIRKRANDLLASLRRLRTEI-----------
ANTLO    FKTP-----------------------MASQKNRLRDAC-LEMLDCL---
ARATH*   -----------------VAQAMERLNQAARVIADIRLGA-DRILEAMFVA
VITVI    -----------------VALAMDRLAQAARLIADVRLGA-DRLLEALFVS
SOYBN    -----------------VAQAMDKLGQAERIIADIRIGA-DRLLEALFVA
SOLLC    -----------------VAMALERLGQASRLIADIRIGA-DRLLEALFVS
ORYSJ*   -----------------VAQAMERLGRAGRLIADIRLGA-DRLLEALFVA
HORVU    -----------------VAQAMERLGRAGRIIADIRLGA-DRLLEALFVA
SORBI    -----------------VAQAMERLGRAGRLIADIRLGA-DRLLEALFVA
                  160       170       180       190       200

HUMAN*   ------MRNKETLEGR---------------EKAF------IAHFQDNLH
CHICK    ------MRNKETLEGR---------------EKGF------VAAFQESLH
DANRE    ------MKNKETLESR---------------EKVF------ITEFQENLQ
CIOIN    ------VKPDEDHDGRAEETETSAGSLLGRDRKKF------LQKFQEHLQ
ANOGA    ------VRADHGEEGK---------------EQKF------LQELQELLN
TETNG    ------MKNRENLEGR---------------EKLF------LTEFQDNLQ
DROME    ------TDGESGEESR----------------NKF------VNDFQERFA
BRUMA    ------SWESDPTDGEKTLEE----------RLDF------INQIYDNLE
CAEEL    ------SGNDQAEFNS-AFQN----------YKDFGLMVHEVMMFYDKLD
YEAST*   ------DQEGSKQADR---------------MAKYLHIRDKILQLNDRFQ
SCHPO    ----------HPETGT---------------SRKF------EKSAQEAIE
CANAL    ----------PPNQSQ---------------TEYYKLVVSRLKKVQDSID
CANGA    ------DEERYANVSK---------------QEKFQMIRSKVLGLTERLQ
ASPNI    VHPKAAAAIGIPNSPQAL-------------HQRL------SQSACDGVR
RHIO9    ------EQEQKGPNHVQAFSE----------RIKR------LKQELISLG
ENCCU    --------------------------------------------------
ANTLO    ------GSLRQPFHDENMLVH----------RLSL------LNRCYLNLI
ARATH*   ------SQPRHTDMPLQLFLR----------EDAS------MRQHLQDLR
VITVI    ------AQPHQTTKPLHLFLK----------EDSS------MRQHLQDLR
SOYBN    A-----AQPHQGNKPLQVFLK----------EDAC------MRQYLQDLR
SOLLC    ------AQRQHSSKPLHLIHK----------EEAS------MRQHLQELR
ORYSJ*   GDAPPHSAPQHVEKTALAVAQ----------EEAA------MHRHFDDLR
HORVU    ASAPPHSTQQHIERNEDVVAK----------EEVS------MHRHFDDLR
SORBI    GGAPPYSVHQHVDRMERVIVK----------EEAA------MRLHFQDLR
                  210       220       230       240       250

HUMAN*   SVNRDLNELERLSNLVGKPSENHPLHNSGLLSL-----------------
CHICK    SVSRDLGELERLSNLVGKPSENHPLHNSGLLSL-----------------
DANRE    SVNRDLNELERLSTLVGRPSESHPLHNSGLLSL-----------------
CIOIN    AVNKDYNEVEKYCNSMIPTPELQVLGPLSHLSL-----------------
ANOGA    SVNANLKDFESCINDLTPPQTPLTLANSAYLSL-----------------
TETNG    AVNRDLNELERLSGLVGRPSESHPLHNSGLLSL-----------------
DROME    AINSQIREVEQLINGLPVPPTPYSLGNTAYLAQ-----------------
BRUMA    SHAKQLSVSTPMTVQMERLSRFLHDG------------------------
CAEEL    HIARHLPKALPPIDNINLMRTLYTQEHT----------------------
YEAST*   TLSSHLEALQPLFSTVPEYLKTADNRDRSFQLLEPLSTYNKNGNAVCSTA
SCHPO    KVNSFYTHLKSSQNVFD---------------------------------
CANAL    DYCEKYNKLLPIINLAQIKLGHDVEGPPPSKIKSETSTNNNTPNMGNKTT
CANGA    ELSNDFEELQPLFATVGEYSKTYKNKNFQVLEN--LASYNHRGKAG---A
ASPNI    EVE-----------------------------------------------
RHIO9    TESEKIKDGRID--------------------------------------
ENCCU    --------------------------------------------------
ANTLO    SKAEYVLYSVSL--------------------------------------
ARATH*   LIGKKLEESGVLTESLRSRSNSWGLHMPLVCPD-----------------
VITVI    TIGKQLEDSGVLNESLRSRSNSWGLHMPLVCPD-----------------
SOYBN    SLGKELEESGVLSESVRSRKDFWGLHMPLVCPD-----------------
SOLLC    SVGRQLEDSGVLNDSLRSRSNSWGLHMPLVCPD-----------------
ORYSJ*   ALGRQLEESGVLNGALKARGNSWGLHMPLVCPD-----------------
HORVU    ALGRQLEESGVLNGALKARGNSWGLHMPLVCPD-----------------
SORBI    ALGRQLEESGVLNGALKARGNSWGLHMPLVCPD-----------------
                  260       270       280       290       300
```

MED3

**HUMAN***	------------DPVQDKTPLYSQLLQAYKWSN----------KLQYHAG
CHICK	------------DPVQDKTPLYSQLLQAYKWSN----------KLQYHAG
DANRE	------------DPVQDKTPLYSQLLQAYKWSN----------KLQYHAG
CIOIN	------------DSNTDKKNTFRDAVQTYEWNQ----------RMHEYAQ
ANOGA	------------ETNLERQALYPHLVQSYKWHD----------KLHEYST
TETNG	------------DPVQDKTPLYSQLLQAYKWSN----------KLQYHAG
DROME	------------ETSQDRQALYPQLVNSYKWMD----------KVHDHSF
BRUMA	------------QIDPHTNELYDKALEASTWME-----------------
CAEEL	------------MCHININNLLEKMIDCGNWNE-----------------
**YEAST***	TVVSTNHSAAASTPTTTATPHANPITHAHSLSNPNSTATMQHNPLAGKRG
SCHPO	-----------YAEKSLQADSSNLLPTYLYNS------------------
CANAL	PVMGNKTTPVMGNSSTPSTVTANTNTGAYSTSS-----------------
CANGA	SISNSTPTPAAATPTTAPTPGAGTKKAAKTAPTPTATATI----------
ASPNI	----------------DFKALWRSEEMKAVWERIDTLINENAGRLLQSNG
RHIO9	-------------------------VLEYVQSK----------TVEDEFD
ENCCU	--
ANTLO	---HVIDKNG
**ARATH***	------------GA-----------VVAYAWKR----------QLAGQAG
VITVI	------------GA-----------VVAYAWKR----------QLAGQAG
SOYBN	------------GA-----------VVAYAWKR----------QLAGQAG
SOLLC	------------GA-----------VVAYAWKR----------QLAGQAG
ORYSJ*	------------GA-----------VVAYAWKR----------QLAGQAG
HORVU	------------GA-----------VVAYAWKR----------QLAGQAG
SORBI	------------GA-----------VVAYAWKR----------QLAGQAG
	310 320 330 340 350

**HUMAN***	-LASGLLNQ------QSLKRSANQMG------------------VSAKRR
CHICK	-LASGLLNQ------QSLKRSANQMG------------------VSAKRR
DANRE	-LASSLLNQ------QSLKRSANQMG------------------TSAKRR
CIOIN	-IASSILLS------HQLKRSQSSGGA-----------------ILAKRR
ANOGA	-FASTLLQQ------NSLKRSYY---------------------TNTKRR
TETNG	-LASSLLNQ------QSLKRSANQMG------------------ASAKRR
DROME	-LAFNNLNQ------NTLRRSYN---------------------YCSQKR
BRUMA	-----TNNQLLQMYAEFLRTVVGNRR------------------RSIMTD
CAEEL	-----GNFQTFFYLSELLRCGSTRKY------------------SNLPEQ
**YEAST***	PKSGSTMGTPTVHNSTAAAPIAAPKKPRKPRQTKK---------AKAQAQ
SCHPO	---------------EDLSNDTENNET-----------------KSINGK
CANAL	--
CANGA	---GTPSNNAPTPATTATTPGTQAKKPRKPRQTKKQQQAAAAAAAVAQAQ
ASPNI	-MWQCDYNQILQDIAQHDNHRKEQQR------------------KAKEEQ
RHIO9	-WNAMKSEK------EGNQKMTTRKE------------------IGSSVK
ENCCU	--
ANTLO	DLERLQCSE---
**ARATH***	-ASAVDRTR------LALKAFTDQKRRFFPHIDDG-LKM-----EPSSKK
VITVI	-ASAVDRTR------LALKAFTDQKRRFFPHLDDG-LNGQSSASESVPKK
SOYBN	-ASAVDRTR------LALKAFTDQKRRFFPHLDDG-----LETSESASKK
SOLLC	-ASAVDRTR------LALKAFNGQKRRFFPHLDTN-YGG-----EGVTKK
ORYSJ*	-ASAVDRTR------LALKAFTDQKRRFFPHLDDEGFNHLHDGEPGLAKK
HORVU	-ASAVDRTR------LALKAFTDQKRRFFPHLEDEVLNHLHDGESGIAKR
SORBI	-ASAVDRTR------LALKAFTDQKRRFFPHLEDEVLSHLHDGEPGVTKK
	360 370 380 390 400

**HUMAN***	PKAQPT--------------------------------TLVLPPQYVDDV
CHICK	PKAQPT--------------------------------TLVLPPQYVDDV
DANRE	PKVQPS--------------------------------TLALPTQYVDDV
CIOIN	RALPPK--------------------------------FQNMSSQHVENT
ANOGA	RSLPSS---------------------------------HLATPQMVENL
TETNG	PKVQPS--------------------------------TLVLPPQYVDDV
DROME	QRLPFS--------------------------------SFNNDPDHIDKL
BRUMA	RPISYS-----------------------------NYGLSSSPQNIFEQT
CAEEL	RLVPPA----------------------------MFHSQATNSHAAFEHA
**YEAST***	AQAQAQAQVYAQ--------------------------QSTVQTPITASM
SCHPO	SALDLK----------------------------------EPHHSELHDN
CANAL	--
CANGA	AQAQAQAQNQNQNNMQNKNISNPGMNSNMGTPVMGNPNMKQMQSPIPANA
ASPNI	ERAQLQ----------------------------------SAEGGWKAIV
RHIO9	DKADYA-------------------------------------YKQLSSI
ENCCU	---HSPDVFGLV
ANTLO	--
**ARATH***	HRASHLLLENGR--------------------------EEPVDYKTLPDI
VITVI	HCGSQISTVTQQ--------------------------EELSDYRTLSDV
SOYBN	CCGSEEIAVDPK--------------------------EGISFLRTLPDV
SOLLC	LRGPPS---SDQ--------------------------EETCELKSVSEV
ORYSJ*	PRLPAS--------------------------------NGELEERTLSEI
HORVU	QRMPAG--------------------------------NGELEEKTLSEI
SORBI	PKLISS--------------------------------NGDLEEKSLSEI
	410 420 430 440 450

MED3

```
HUMAN*  ISRIDRMFPEMSIHLSR---------------------------------
CHICK   INRIDRMFPEMSIQLSR---------------------------------
DANRE   ISRIGRMFPDMSIELFR---------------------------------
CIOIN   LQLLARQPADLTFKLIK---------------------------------
ANOGA   IGSIH--YNNMNLKIAR---------------------------------
TETNG   ISRIGRMFPDMTIELFR---------------------------------
DROME   LSEIN-TPPHTSYRIFR---------------------------------
BRUMA   LTVVLKDPNIKKIGLVGRYLE-----------------------------
CAEEL   FNGLRKEIANKSLGIYPKVLQ-----------------------------
YEAST*  AAALPNPTPSMINSVSPTNVMGTPLT------------------------
SCHPO   DNFQNSDINIESFK------------------------------------
CANAL   --------------------------------------------------
CANGA   MNNMNVPHNGAMRPSVPNGNMGNPSMGNLNMNAPNMGNPNMNNPNMNNPN
ASPNI   DNYAQAGIPGMRVIPTKSD-------------------------------
RHIO9   VFD-NNPISIESFFTTYTNEWITQNK---------------------DQA
ENCCU   LGDIQK--------------------------------------------
ANTLO   --------------------------------------------------
ARATH*  QSRLEKLVPSVKVSTYGRLNWLKRAN---------------------SLP
VITVI   LMHVEKDMPNMKIFTYQRLDWLKRAS---------------------TLP
SOYBN   LKSLEKDVPNLKILTFERLDWLKRAS---------------------TLT
SOLLC   LTHLKKEVSNVITFSYRRLDWLLRAT---------------------SLP
ORYSJ*  LKNLENEVPNMKIFTYRRLDWSKRAA---------------------SLA
HORVU   LKNLENEVPNMKVSMYRRLDWSKRAS---------------------SLA
SORBI   LKNLENEVPNMKIFTYWHLDWSKRAS---------------------SLA
               460       470       480       490       500
```

```
HUMAN*  ------------------------------PNGTSAMLLVTLG-------
CHICK   ------------------------------PNGTSAMLLVTLG-------
DANRE   ------------------------------PNGTSAVLLVTLG-------
CIOIN   ------------------------------TLGDTRILQVTVG-------
ANOGA   ------------------------------PFMTNAILHITIA-------
TETNG   ------------------------------PNGNSAVLLVTLG-------
DROME   ------------------------------PFGTNAVTIVTIS-------
BRUMA   ------------------------------KSTLSALVEFKFG-------
CAEEL   ------------------------------RTTCSLIIEFLFGCGGAKPG
YEAST*  ----------------NMMSPMGNAYSMGAQNQGGQVSMSQFNGSGNGSN
SCHPO   ----------------------------GDIEASGSILTTHEN-------
CANAL   --------------------------------------------------
CANGA   AMMSPMAGQGQLNQMFPMQNHNQNGQFMGQQSPGPNIGQMQFPPNNGNMN
ASPNI   --------------------------------------------------
RHIO9   SKVTISFER---------------------EEQSTFSGTICVH-------
ENCCU   --------------------------------------------------
ANTLO   --------------------------------------------------
ARATH*  GSGSDDPTEASKP-IFQSSSKLRSGLQ-TEVVDKIAVIELSFP-------
VITVI   CSAEEFLMDSSKEHDFHISNKPRLGLLGAVAADKVAVIELLFP-------
SOYBN   SSTNEN----SLEHNYHGSNKLRLGSVGTVPAEKVAVIELLFP-------
SOLLC   SSTSENSIELSKESTFQSTRTLRQGSSDDDVSDKVAIIELLIP-------
ORYSJ*  TLMNDDFVDPSKELNLQNMSKSGSGD--TTPIDQVAIIELLAP-------
HORVU   SLMDDDFVDPSKELNLQNMGKSRPGSV-TTPIDQVAVIELLVP-------
SORBI   SLMDDDFVDPSKELNLQNMGKSRSGAL-TTPIDQVAVIELLVP-------
               510       520       530       540       550
```

```
HUMAN*  ---------KVLKVIVVM------RSLFIDRTIVKGYNENVYTE----DG
CHICK   ---------KVLKVIVVM------RSLFIDRTIVKGYNENVYTD----DG
DANRE   ---------KVLKAIVVM------RSLFIDRTIVRGFHENIYTE----DR
CIOIN   ---------RVFTVILML------RNLLMEKVLVKGLDEELYYE----NG
ANOGA   ---------RVLRAAVIL------KGLLIEWVTVKGYEESLL------DG
TETNG   ---------KVLKAIVVM------RSLFIDRTVVRGFSENVYNE----DG
DROME   ---------NVMKAAIVF------KGVLIEWVTVKGFDEPLEHD------
BRUMA   -QVIDKQYVCLLKMLMVV------NSGLPEFVQMIAPHEEWSYL-DIGSA
CAEEL   EKNTDKEIVYTIKFLIIE------RNGSIEYINIMAPTEDWHMVNDYAIP
YEAST*  PNTNTNSNNTPLQSQLNL------NNLTPANILNMSMNNDFQQQ------
SCHPO   ---------KSFTLKLAN---------ELEFIFFHD--------------
CANAL   --------------------------------------------------
CANGA   GMPGTSDMNLGMNPSMNMNMGMNLNQITPANILSMNTKGKDDQMQNIGMD
ASPNI   ---------SSFGVLLPK------VGLAFKVNAVSSGQEG----------
RHIO9   ---------KGFIAELELEYVRASNRLVVHQHDIRGLKEE----------
ENCCU   ---------SYLRLAVLL---NKPGIQHQEPVEVDSVNGIVRYK----AG
ANTLO   ---------SGFSAVISK--------------------------------
ARATH*  ---------SLFRAIVSL---SPAGSVDPDAVAFFSPDEG--------GS
VITVI   ---------SIFRAIISL---HPAGSIDPDAVAFFSPDEG--------GS
SOYBN   ---------CVFRAVISL---HPAGSMDPDAVAFFAPDES--------GS
SOLLC   ---------SVFRAIASV---SPAGSLDPDAVAFFSPNEG--------GS
ORYSJ*  ---------SIFRAIVSL---HPAGSTDPDAVAFFSPTEG--------GS
HORVU   ---------SIFRVVVSL---HPAGSVDPDAVAFFSPTEG--------GS
SORBI   ---------SIFRAVVSL---HPAGSTDPDAVAFFSPTEG--------GS
               560       570       580       590       600
```

MED3

```
HUMAN*   KLDIWSKSNYQVFQKVTDHATTALLHYQL---PQMPDVVVRSFMTWLRSY
CHICK    KLDIWSKSNYQVFQKVTDHATTALLHYQL---PQMPDVVVRSFMTWLRSY
DANRE    KLDIWSKSNYQAFQKVTDHATTALLHYQL---PQMPDVVVRSFMTWLRSY
CIOIN    TIDLFSESQFECMRRITDHAMTAVLQYSTTH-ATQPEIPLKYFLLWLQSY
ANOGA    VDEQWTESRHQVFRKVQDHAHSAMLHFFS---PTLPELAIRSFITWFRSY
TETNG    KLDIWTKSQYQVFQKVTDHATTALLHYQL---PQMPDVVVRSFMTWLRSY
DROME    --DLWAESRYEVFRKVQDHAHSAMLHFFS---PTLPDLAVKSYITWLNSH
BRUMA    QVDIHKESRYLVYRKMSVQANIHLMQTIMPCIDIRNAHTLSYVLNLFAKF
CAEEL    TINPFAPSRYEVYRRLTKQANIHLFNCFAQ--NRWTAGTLFHFISLYGKF
YEAST*   ----QQQQQQQQQPQPQYNMNMGMNNMNNG--------------------
SCHPO    -----TRGKFSVYCSSSKDDAITFSINR----NNNFLGNLWSLMPKILDY
CANAL    -------NTAAKPTKKATKRASVS--------------------------
CANGA    QNQNQNQSQNQSQNQNQSMNMNMNNDSNNP--------------------
ASPNI    -VPEFNVSSKSPSGEPSSRVETAVLACLN---ARPRKWDLKFLFGMISSY
RHIO9    -KHIWQDSQYLIFQKLNLLTSVAFEDMT----MFFARESFYYILDWFSSY
ENCCU    ELEFLYHADHGVVSVDAGDIGVSSHILCSV-RSEPVVKHLETIGNMLAMY
ANTLO    --------KYGVLSVSTGNDRINLKAFRF---ARTSESSVEALCEFLSPY
ARATH*   YLHARGFSVYHVYKHITEHAATALQYFL----GFGTGTALYSLLLWICSF
VITVI    YVHARGFSVYHVFKHVTEHAAMALQHFL----GTNTETALYSLLHWIFSY
SOYBN    YVHARGFSVHHVFRHITEYAATALQYFL----GNQSETCLYFLLHWICSY
SOLLC    YIHARGFSVHHVFRHITEHAAMALQHFT----GVGTESPLLSLLLWVCSY
ORYSJ*   YLHARGLSVHHVFKHVSEHADKALQYFV----SVEPTKSLSLLLRWIASY
HORVU    YLHARGLSVHHVFKHVTEHADKALQYFI----SVEPSKALSLLLRWIADY
SORBI    YLHARGTSVHHVFKHVKEHADKALQYFI----SVEPNKALSLLLRWIASY
                  610       620       630       640       650
```

```
HUMAN*   IKLFQAPCQRCGKFLQDG------LPPTWRDF-RTLEA------------
CHICK    IKLFQAPCQRCGKFLQDG------LPPTWRDF-RTLEA------------
DANRE    IKLFQTPCQRCGKYLQEG------LPPTWRDF-RTLEA------------
CIOIN    KNLFIDPCSICGKHLDGG------LPPVWRDF-RNPVA------------
ANOGA    VTLFADPCKKCGKHLHNT------LPPTWRDL-RTLEP------------
TETNG    IKLFQSSCQRCGHFLQDG------LPPTWRDF-RTLEA------------
DROME    VKLFLEPCKRCGKFVVNG------LPPTWRDL-RTLEP------------
BRUMA    SGVFDIKCRVCKRIMK------DYLPPLMFDL-RCPKN------------
CAEEL    RELFTTKCRSCKQVLSRK----NFLPPLIFDI-RNPNN------------
YEAST*   -----------GKELDSLDLNNLELGGLNMDFL-----------------
SCHPO    QHLYSKPCDFCKSLIS---PVYLELPSVRRNANSTVKP------------
CANAL    ---------------NKDMGSSANQPIVI---------------------
CANGA    -----------KSAYDLVDFNSLDLSSLNMDFL-----------------
ASPNI    SNIFQTTCTRCGKMQD----QAANLPTLRRPKTKEPQT------------
RHIO9    HDLFTAPCYRCHKVLQFDSPVSKYLPPIVRTW-NSKHA------------
ENCCU    VGYERAPCDVCGSYAT--------VPGLLTPTGRSIED------------
ANTLO    SKYRERRCDVCLCVKN---------PELKYATERVAED------------
ARATH*   ESVFSKPCTKCGRLLAMDKKSALILPPLHRAY-QELPLA-----------
VITVI    QTLFTEPCSKCKRLLSMDRQSDLLLPPVYHPY-RSFCAIKSSSPQSISST
SOYBN    QTLFSRPCSKCSRLLAMDKQSTLLLPPVHRPY-WQFSFSKILLNI---SS
SOLLC    QTLFSKACSKCSRLLSMDKESAMLLPPVNRPY-RNFSAGKFL------SK
ORYSJ*   QTLFTKVCSKCGRLLLMDKSLALLLPPVQRPYHHQTSS------------
HORVU    QTLFTKLCSKCRRLLLMDKSLALLLPPVHRPY-HQISS------------
SORBI    QTLFTKVCSKCGRLLMMDKSLALLLPPVHRPY-HQTSS------------
                  660       670       680       690       700
```

```
HUMAN*   ----------FHDTCRQ---------------
CHICK    ----------FHDTCRQ---------------
DANRE    ----------FHDTCRQ---------------
CIOIN    ----------CHDTCRP---------------
ANOGA    ----------FHEECKQ---------------
TETNG    ----------FHDTCRM---------------
DROME    ----------FHEDCRNC--------------
BRUMA    ---------ALHESCR----------------
CAEEL    ---------AAHEVCR----------------
YEAST*   --------------------------------
SCHPO    ---TSKDILALHAECVPAQSDL----------
CANAL    --------------------------------
CANGA    --------------------------------
ASPNI    --DQPPTFEAYHATCI----------------
RHIO9    ---AQVTPTAYHKTCFHEHKNSQAMSHKSTCQ
ENCCU    -----DFVLVHHAECRMESLE-----------
ANTLO    -----DYVAAYHAECLDKKETLLCF-------
ARATH*   --LNLDVCEAYHSSCSQDDT------------
VITVI    KNQTLDASHAYHVGCFSEEA------------
SOYBN    KDQNSDTTQAYHIDCLSEET------------
SOLLC    EEPSVDSSQCFHIDCFSEEA------------
ORYSJ*   --VGSDPQDAYHIGCSSYDA------------
HORVU    --IGSDHQEAYHIGCSSYDA------------
SORBI    --IGPDLQEAYHIGCSSYDG------------
                  710       720       730
```

MED4

```
HUMAN*  ------------------------------------------------MA
CHICK   --------------------------------------------------
XENTR   --------------------------------------------------
DANRE   --------------------------------------------------
CIOIN   --------------------------------------------------
ANOGA   --------------------------------------------------
TETNG   --------------------------------------------------
DROME   --------------------------------------------------
BRUMA   --------------------------------------------------
CAEEL   --------------------------------------------------
YEAST*  ----------------------------------------MSVQDTKAVE
SCHPO   --------------------------------------------------
CANAL   MLPFKKADSPFKSNPVSRVGSSTRLNQLGNIKSNPTTPNAALYVTSSLNP
CANGA   --------------------------------------------------
ASPNI   --------------------------------------------------
RHIO9   --------------------------------------------------
CRYNE   --------------------------------------------------
USTMA   ------------------------------------MHGSLALQLYQVVG
ENCCU   --------------------------------------------------
ANTLO   --------------------------------------------------
ARATH*  -----MLQ---HQI-----------------------------VQS----
VITVI   -----MLQSVQHQI-----------------------------VQS----
SOYBN   -----MLQ---HQI-----------------------------VQS----
SOLLC   -----MLQNVPHQP-----------------------------LQS----
ORYSJ*  -----MMQSH---------------------------------HPS----
HORVU   -----MMQH----------------------------------MPS----
SORBI   -----MMQSH---------------------------------LPS----
PHYPA   MSLLGRLPSNCDQVLGYAMVMRIRALAIGGLVESGRRVKGFGEVVT----
                 10        20        30        40        50

HUMAN*  ASSSGEKEKERLGGGLGVAG----GNSTR---------------------
CHICK   -----------MAAAAAAAGGGGERSSTR---------------------
XENTR   ----------------PSADMAVPEKSTK---------------------
DANRE   ------MRTERETIMAAAAA----DKSTK---------------------
CIOIN   ---------------------MESEVSTK---------------------
ANOGA   --------------------MSSYHLSTR---------------------
TETNG   ---------------MATTT----EKCTK---------------------
DROME   ---------------------MSFHLSTK---------------------
BRUMA   ---------------------MADNCSLK---------------------
CAEEL   -------------------MAETDERSLR---------------------
YEAST*  FSMGHIRSSSVSLVAEATSNTNSEDKLSK---------------------
SCHPO   --------------------------------------------------
CANAL   LKNLPTNAANVKLRLQTQKDLDAFEQLPM---------------------
CANGA   ----------MSLVAESNSGTISEQELDK---------------------
ASPNI   ---------------------------MN---------------------
RHIO9   -----------------------MQHSTR---------------------
CRYNE   --------------------------------------------------
USTMA   PGCHSPSPCRLILAPPVQAGAACNEPSTS---------------------
ENCCU   --------------------------------------------------
ANTLO   --------------------------------------------------
ARATH*  -------PARLGLTGPGSPSVQN-PTPTRHGHPTSSSSSQSQHQQIQQQP
VITVI   -------PARLGLPNPNSPSLQN-STAPK------LSSQIPQSQQPHQQS
SOYBN   -------PARLGLANPNSPSIPN-PTPPK--------LPPTQTHHPQDR-
SOLLC   -------PARLGLPTPSSPSLQNPNTAPK------FSSQVSQPHQPHQQA
ORYSJ*  -------PARLGL-TASSPSLPPNPAAGN------PTSSPPQGNPSAAAA
HORVU   -------PARLGV-TASSPSLPPNQSPAN------PTSSPPQANPPSASA
SORBI   -------PARLGL-TASSPSLPPNPVPLN------PTSSPPPGSLPSSAA
PHYPA   -------MSMLSMGSAGHGELQSMWGPAR--------------------S
                 60        70        80        90       100

HUMAN*  --------------------------------ERLLSALEDLEVLSRELI
CHICK   --------------------------------ERLLAALEDLELLARELI
XENTR   --------------------------------ERLMSFLDDLEVLSRELI
DANRE   --------------------------------ERLLSTLDDLEVLSRELI
CIOIN   --------------------------------KQLLSLVDDVDLVIRQLF
ANOGA   --------------------------------ERLLAIINDIEIVAKELI
TETNG   --------------------------------ERLLSALADLEVLSRELI
DROME   --------------------------------ERLLLLIDDIEMIAKELI
BRUMA   --------------------------------ELLQESTEDLEATIKMIV
CAEEL   --------------------------------DLLLESADDLEHIVKMIV
YEAST*  --------------------------------VQLYEDLCRYEDTLSKLV
SCHPO   --------------------------------MEYQRAIDSIEECLNKQL
CANAL   -----------------------------------VQKVNEYERLLNELS
CANGA   --------------------------------VQLYSDLCLYEEALTKLV
ASPNI   --------------------------------AEFQTALSTFESRLNSLV
RHIO9   --------------------------------DQVNTYLTEYADLVKKYF
CRYNE   --------------------MAHTTPRSHPPPHSLLQNLTTQSLLLSHLF
USTMA   --------------------------------CLVLSTLGAYSELTKHLF
ENCCU   -------------------------------------MVLIFYPLMDRLY
ANTLO   ---------------------------------------------MDVIF
ARATH*  NLLPSSTVAAASSASASAAVSSSALLSLLPPLPRAQALLQQMAVLTSKLF
VITVI   NL--------------STTTTSSTLLSLLPPLSRAQSLLLQMASLASRLF
SOYBN   ----------------NSSTPSSALLSLLPPLPRAQALLAQMASLASKLF
SOLLC   NIL-------------TTTTTSSTLLPLLPPLSRAQSLLIQMASLASRLF
ORYSJ*  VGA--------AAAAAPTLTTSPSLLPLLPPLPRAQALLQLISALASNLF
HORVU   AAA-----AAGAGAAVPTLTTSPALLPLLPPLPRAQSLLHLISSLASNLF
SORBI   AGA----------SAAPTLTTSPSLLPLLPPLPRAQSLLQLISSLASNLF
PHYPA   QQQQHDQAVPMDTAATARPAVVRSALMNQPPQRRAHMLLQEMAGLANRLV
                110       120       130       140       150
```

MED4

```
HUMAN*  EMLAISRNQKLL-----------------------QAGEENQVLELLIHR
CHICK   EILAISRNQKLP-----------------------QPGEESQILELLIQR
XENTR   EMLALSRNQKLS-----------------------QPGEENQILELLIQK
DANRE   EMLALSRSQKLP-----------------------PPGEDTLILELLIQR
CIOIN   DTLSQQSSQNTVVNQVGSGESETQS----------HVMDVGQLTDLLLQK
ANOGA   ENTIAPKAQKM------------------------SSTDHAQLVELLVLK
TETNG   EMLALSRTQKLP-----------------------QPGEDTQVLELLVQR
DROME   EQA----HQKI------------------------SSTELVDLLDLLVAK
BRUMA   AFVLDRDR---------------------------SCGSLTSLCESFRKK
CAEEL   DTLINRDKSVMLK----------------------NGETVTNIIKLFDSK
YEAST*  ESVD-------------------------------RFKPNLDIAKDLIRT
SCHPO   RLSSEKVDQYVLI----------------------------ENWTSLVGH
CANAL   EAVSQF-----------------------------KNDELQEKIGQIITC
CANGA   DSVD-------------------------------NFKPQLEIGKQLIEA
ASPNI   TSLTTSP----------------------------TASGAPKAAQELLNA
RHIO9   ASLSTVADHSISE----------------------EANSPEQFIKQIITI
CRYNE   TLIASPPNPNTS-----------------------TQTQLNQVYSALQLS
USTMA   SIIENPSLSSKTIVDASAGKMW-------------NVNDITGLLSALQEV
ENCCU   REVS----------------------------------------------
ANTLO   DRES----------------------------------------------
ARATH*  DVSPNRAIWLSAFRGSLPSFLSSHSLPPPPPLENPSPSSTKEILSQFNSL
VITVI   EVSPNRSLWLTSFRGTLPTFLSSQSIPPTPTPLEHSPSSTKEILSLFTSL
SOYBN   DVSPNRSLWVTAFRGSLPTFLSSHS----STPLDTSPSNAKEIISLFTVL
SOLLC   EVSPNRSHWLSAFRGSLPSFLPSVA----PVPQDSCPSSSKEILSVFTSL
ORYSJ*  ELSPSRAAWISAYRGSLPTFLPSPS-SAPPPPLPAPISSTKEALSLLNTL
HORVU   ELSPNRAAWISSYRGSLPNFLASSSASAAPTPLPAPVSTTKEAMSMLTSL
SORBI   ELSPNRAAWIQAYRGSFPTFLPSASSS--APPLPTPISSTKDALSLLTTL
PHYPA   ELAATKSWQPGHMKVSIPPFLAGAI----LPPEGQAAVTSKEVQALLENH
                 160       170       180       190       200
```

```
HUMAN*  DGEFQELMKLALNQGKIHHEMQVLEK---------EVEKRDSDIQQ----
CHICK   DGEFQELMKLAVDQGKIHHEMQLLEK---------VVEKRDNDIQQ----
XENTR   DGEFQELMKVALSQGKIHQEMQVLEK---------EVEKRDSDIQQ----
DANRE   DKEFQELMQTALEQGRVHQEMQSLEK---------EVEKRDSDIQQ----
CIOIN   NKKLADLTEVACQQQKLEVKVKATRA---------EIAMKDTEIKC----
ANOGA   DKELKATLQLAAEQAGIEKNMDALRE---------QVRKQDEEINQ----
TETNG   DAEFQELMEVAQQQGKVHEEMQLLEK---------EVEKRDSDIQQ----
DROME   DEEFRKMLELAEEQAKVEEAMDQLRA---------KVEVHDREIQK----
BRUMA   HAELAKLLAKVPAHQERERREIRRLEA--------EVEARDRILGD----
CAEEL   QDSIKTLLKRVPEFQEREQLIRTLEA---------HVEKRDEVIQQ----
YEAST*  DEALFENVKLLAEYDNIYRNLQKIDK---------DSEELDSKTRK----
SCHPO   LKTLHSLISNYTNGRELQNEISSLLK---------QDKELDLQIQD----
CANAL   NDVLKQQIEDLNKHRNYSYEVDKLSD---------RNKILEENSKF----
CANGA   DKKLYSTLDLLPQYDSVFTRLRTLDD---------EISKVDQQTKN----
ASPNI   DDSLTSTIDTLKQHQSNYARILQLRA---------EAQSLEDKVKD----
RHIO9   DDNLQKAVEHIEEHQKRQQKIIQVQD---------EIQQYNIKLLN----
CRYNE   TLDLSGLVKEVGHHQEAYRRLVEKKN---------EVAGLEMRVRG----
USTMA   DQLFNSRLEIAHQHAINQRRIEALQE---------QAKRRDRETRQ----
ENCCU   ----EEFLAGLRKYINDEMSYAELERLSTREALAFNSHRWGAVIEE----
ANTLO   ----ETFLALLEKYLDDNASIHEVVV---------QAKKVDLVQQVCDEG
ARATH*  QTQLFEAVTELQEILDLQDAKQKVAR---------EIKSKDSSLLA----
VITVI   QTQLFEAVAELQEILDLQDAKQKITR---------EIRSKDSAILA----
SOYBN   QTQIFEAVAELQEILDLQDAKQKIDR---------EIRSQDSTLLA----
SOLLC   QTQLFEAVAELQEILDLQDEKQKLTR---------EVRSKDSSILA----
ORYSJ*  QTQLFEAVAELQETLDLQDARARLAR---------EARAKDASILA----
HORVU   QTQLFETVAELQETLDLQDARAKLAR---------EARAKDASLLA----
SORBI   QTQLFEAVAELQETLDLQDARARLAR---------EARAKDAALVA----
PHYPA   QNLLYEAVSELQELQDIEQEKQQIAE---------EINTKDVVVRA----
                 210       220       230       240       250
```

```
HUMAN*  ----LQKQLKEAEQILATAVYQAKEKL-K---------------------
CHICK   ----LQKQLKEAEHILATAVYQAKEKL-K---------------------
XENTR   ----LQKQLKEAEHILATAVYQAKEKL-K---------------------
DANRE   ----LQKQLKEAEHILATAVYQAKEKL-K---------------------
CIOIN   ----LQKHLKAAEQLLGTALHQAKEKL-K---------------------
ANOGA   ----LQRQLKEAEQILATSIFQARQKL-A---------------------
TETNG   ----LQKQLKEAEHILATAVYQAKEKL-R---------------------
DROME   ----LQKSLKDAELILSTAIFQARQKL-A---------------------
BRUMA   ----LQNQLMAAEKLVTKMVFQADKKV-K---------------------
CAEEL   ----LETNLKSCEVALTRSCFHANQKL-K---------------------
YEAST*  ----ILEILNECHDELKALPMLEQVEFEK---------------------
SCHPO   ----CMREMTSI----------YDTHLPK---------------------
CANAL   ----ILKELVSYRNELKKLPKLP---------------------------
CANGA   ----ILSILNECHDDLNALPLLEEVEFEK---------------------
ASPNI   ----IVRLVVRYEKDIREACGDSDSDSDTDYDSEESDHDMNAPHTTQ---
RHIO9   ----LVQKLNSSKEELEMSLTNARMEL-K---------------------
CRYNE   ----LVKRLEEGRKELEGMIDQGERSL-E---------------------
USTMA   ----AILELSRIQSELAEMARLSEEEV-A---------------------
ENCCU   ----KSCEALRMKRRVYDELLGIEEKV-RTME------------------
ANTLO   ILGLLKKRIFTFANEEYRRLIDIDRQI-K---------------------
ARATH*  ----FANKLKDAERVLDMLVDDYSDYR-K----PKRSKIEEDDEDNDNES
VITVI   ----FANKLKEAEAVLDILVDDYSDYR-R----PKRSNTADDSED----S
SOYBN   ----FANKLKEAESCLDILVDDYSDYR-R----SKRSKSGDDDS------
SOLLC   ----FANKLKEAERVLDMLVDDYSDYR-R----PKQAKLENDTEE-----
ORYSJ*  ----FAKKLREAHHVLDRLVDDYADYR-RDPKRPRGAAAADDPEP----V
HORVU   ----FAKKLREAHHVLDRLVDDYADYR-RDPKRPRGAAAVDEPEP----V
SORBI   ----FAKKLHEAHQVLDRLVDDYADYR-RDPKRPRGAAAADDPEP----A
PHYPA   ----FAKRVRDAHQVLEDALEEYADLK-K---------------------
                 260       270       280       290       300
```

MED4

```
HUMAN*  ---SIEKARKGAISSEEIIKYAHRISASNAVCAP-LTWVP----------
CHICK   ---SIEKARKGAISSEEIIKYAHRISASNAVCAP-LTWVP----------
XENTR   ---SIDKARKGAISSEELIKYAHRISASNAVCAP-LTWVP----------
DANRE   ---SIDKARKGSISSEEIIKYAHRISASNAVCAP-LNWVP----------
CIOIN   ---SFDQANKGSVMSEDIIKYAHKISASCSTAAP-LNWTP----------
ANOGA   ---SIAKANKRPVSSEELIKFAHRISASHAICAP-LTWQQ----------
TETNG   ---SIDKARKGNISSEEIIKYAHRISASNAVCAP-LNWVP----------
DROME   ---SINQANKRPVSSEELIKYAHRISSANAVSAP-LTWCI----------
BRUMA   ---EMRQSEANRVNSETIIRFANQISRTYAVAAP-LGWQL----------
CAEEL   ---QMKEAELRPVNSEMLIKLAHQISKHNSVSAP-LTWQM----------
YEAST*  ---NTILQQRSKINSTELLDYATKLSKFTK--IP-PTFDK----------
SCHPO   ---TVSGRKRQKVNAETLLDYGRKLSKFSS--AP-PGYNP----------
CANAL   ---KSDKMVNRNVDVDDILKYAFKLAKFTK--AP-ATVAN----------
CANGA   ---KMILKQREKIKSNVLLEYATKLAKFTK--IP-PTFDK----------
ASPNI   ---TRTSRKRNEVDYRLLLDFARRISKYNHEAAADAAAGMAKGNHVLAGR
RHIO9   ---ATEYAKKSNINFTDILSYASKLSKYTS--AP-PNFDL----------
CRYNE   ---DIQKSEREPVPAKTLMAHAQSLSKHSS--AP-VSSLL----------
USTMA   ---SIEKAEKQPIGHSTLLGYAQRLARYTS--AP-PGYKLPRVASAAATQ
ENCCU   ---KMENGREFDVDLAGLVSHSEIVGRNRS--HP-PGYEN----------
ANTLO   ---ASNDRTVVDVDPDVFFAQAARISCVRE--PP-YGYER----------
ARATH*  S--SSSTTVSSQLKLKDILAYAHKISYTTF--AP-PEFGA----------
VITVI   S--SCTTTVASQLNLSDILSYAHRISYTTF--AP-PEFGA----------
SOYBN   ---MTSSTVSSQLKLSDILSYAHRISYTTF--AP-PEFGA----------
SOLLC   ---SSVTTVATQLKLSDILSYAHRISYTTF--AP-PEFGA----------
ORYSJ*  SDGDFGASLHSKLNLDDVLTYAHRISYTTF--AP-PEHGA----------
HORVU   SQGDFGASLHSKLKLDDILTYAHRISYTTF--AP-PEHGA----------
SORBI   SEGDFGTSLHSSLKLDDILAYAHRISYTTF--AP-PEHGA----------
PHYPA   ------GKHRKSVPVAELVSYAHRISYTTF--AP-PDYGA----------
                          310       320       330       340       350

HUMAN*  -----------------------------------G---------DPRRP
CHICK   -----------------------------------G---------DPRRP
XENTR   -----------------------------------G---------DPRRP
DANRE   -----------------------------------G---------DPRRP
CIOIN   -----------------------------------G---------DQRRP
ANOGA   -----------------------------------G---------DLRRP
TETNG   -----------------------------------G---------DPRRP
DROME   -----------------------------------G---------DLRRP
BRUMA   -----------------------------------G---------DTSRP
CAEEL   -----------------------------------G---------DPSRP
YEAST*  -----------------------------------G------AVGPNNFI
SCHPO   ---------------------------------ETG----QDAKAPVHYP
CANAL   ---------------------------------------MPFQIHPNNYV
CANGA   -----------------------------------G------TIGPNNFI
ASPNI   DTEMTGVNTNGNQEGSPAEPVAAVTKEATSWLDESA----NMTRQVYMLP
RHIO9   -----------------------------------L---NRDAKIDFEKP
CRYNE   ----------------------------------------APVDKAQYAP
USTMA   KDDVAGVKSEQRADGEPVEQISLGADYNQYAKRAAAYYDPAIPSMPQEMP
ENCCU   -------------------------------------------TDLYFPP
ANTLO   -------------------------------------------SRAFKPP
ARATH*  -----------------------------------G----QAPLRGALPP
VITVI   -----------------------------------G----QAPLRGALPP
SOYBN   -----------------------------------G----QAPLRGALPP
SOLLC   -----------------------------------G----QAPLRGALPP
ORYSJ*  -----------------------------------G-----LPLRGALPP
HORVU   -----------------------------------G-----LPLRGALPP
SORBI   -----------------------------------G-----LPLRVSLPP
PHYPA   -----------------------------------G----ETILRGALPP
                          360       370       380       390       400

HUMAN*  YPTDLEMRSGLLGQMNNPSTNGVNGHLP----------------------
CHICK   YPTDLEMRSGLLGQMNNPSTNGVNGHLP----------------------
XENTR   YPTDLEMRSGLLGQMSNLPTNGVNGHLP----------------------
DANRE   YPTDLEMRSGMLGNMSNMSTNGVNGHLP----------------------
CIOIN   YPQDIEMRCGWLGQINNSDMNVKDPMLKPP--------------------
ANOGA   YPTDIEMRLGFLGKSDLNINGH------------------------NLQH
TETNG   YPTDLEMRSGLLGHMANLPSNGMNGHLP----------------------
DROME   YPTDIEMRNGLLGKSEQNINGG------------------------TVTH
BRUMA   YPTEAELRLSALAAPRVIVQAA----------------------------
CAEEL   FPQEHEFRAGHLLNSKVQSTGP----------------------------
YEAST*  WPAEDALRRGMLAMASLHSKEL----------------------------
SCHPO   WPSEDQMRKTLLFQFSTSMVPNLSATASQLFSEQPPKTNEPTETETEIDA
CANAL   WPAEDSLRRGMLAQASLQAEEIIRHELGETDKENPNEVKTESKVDHDDDD
CANGA   WPAEDALRKGMLAMASLHGKEL----------------------------
ASPNI   YPMEDRIRMGLMGQIQLAAAEGRPGFDPDKEVERLIREAEGVGIADAVAP
RHIO9   YPDEERMRRGLLYWQNSSQQPT----------------------------
CRYNE   WPTEMSMRMGLLFQLEGSMSGM----------------------------
USTMA   FPSDAMMRQGILNSQQMLDGAA----------------------------
ENCCU   FPSLGMVR------------------------------------------
ANTLO   YPTQEMINR-----------------------------------------
ARATH*  APQDEQMRASQLYTFADLDIGL-----PKTV-ENMEKKVE-----ALIEP
VITVI   APQEEQMRASQLYNFSDLDVGL-----PKAV-GIKEKTIE-----TFIEP
SOYBN   APQEEQMRASQLYNFADLDVGL-----PKEV-ETKEKIVE-----AIVEP
SOLLC   APQDEQLRASQLYIFADLDVGL-----PKTD-EGK-KIVE-----PLIEP
ORYSJ*  APQENEMRMSQLYQFADLDVGV-----PKSQ-EAKERTAAEGDATPLFQP
HORVU   APQDNELRMSQLYQFADLDVGV-----PKKPLETKEGLTAEMESMPLFEL
SORBI   VPQENQMRASQLYLFADLDVGV-----PKKPLDAKEGIAAEVEAAPLYEP
PHYPA   APQEENMRASQLYHVQVSDLGF----------------------TSAFAP
                          410       420       430       440       450
```

MED4

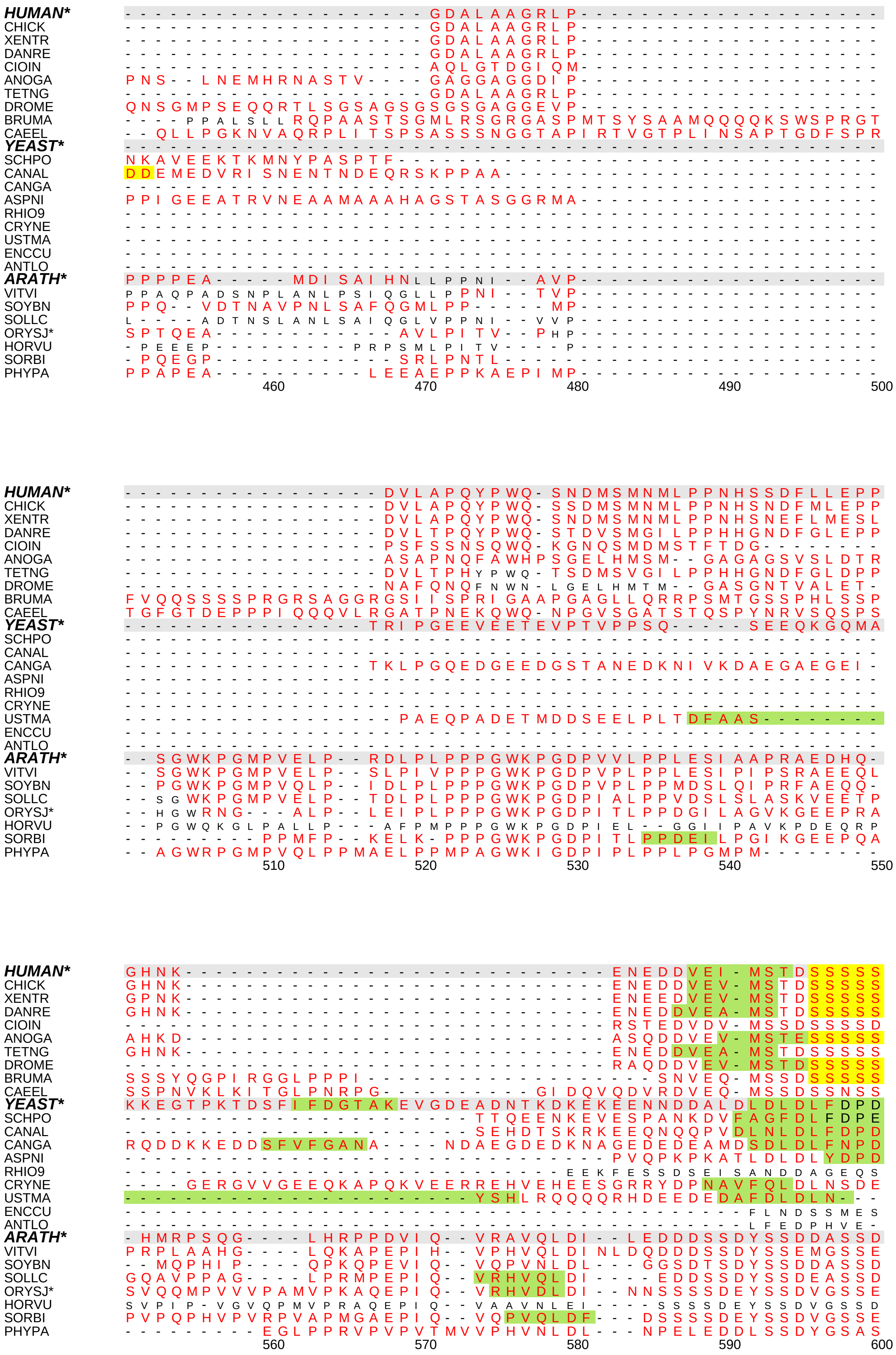

HUMAN*
CHICK
XENTR
DANRE
CIOIN
ANOGA
TETNG
DROME
BRUMA
CAEEL
YEAST*
SCHPO
CANAL
CANGA
ASPNI
RHIO9
CRYNE
USTMA
ENCCU
ANTLO
ARATH*
VITVI
SOYBN
SOLLC
ORYSJ*
HORVU
SORBI
PHYPA
460
470
480
490
500
510
520
530
540
550
560
570
580
590
600

MED4

```
HUMAN*  SESD----------------------------------------------
CHICK   SDSD----------------------------------------------
XENTR   SDSD----------------------------------------------
DANRE   SDSD----------------------------------------------
CIOIN   SSDSSGVND-----------------------------------------
ANOGA   SSDSQ---------------------------------------------
TETNG   SDSD----------------------------------------------
DROME   SSDSQ---------------------------------------------
BRUMA   SSDEVHMGEPKRTVDKTVSSSDSNSGACGTETLPSSKESSNTNKELSDFP
CAEEL   DSSDDEGASKKTGSSNK---------------------------------
YEAST*  DF------------------------------------------------
SCHPO   MEEDF---------------------------------------------
CANAL   DEYSD---------------------------------------------
CANGA   EF------------------------------------------------
ASPNI   EDED----------------------------------------------
RHIO9   THKH----------------------------------------------
CRYNE   SDED----------------------------------------------
USTMA   --------------------------------------------------
ENCCU   SDDDQESAAD----------------------------------------
ANTLO   --------------------------------------------------
ARATH*  DED-----------------------------------------------
VITVI   DED-----------------------------------------------
SOYBN   DED-----------------------------------------------
SOLLC   SED-----------------------------------------------
ORYSJ*  EDDE----------------------------------------------
HORVU   EDDE----------------------------------------------
SORBI   EDDE----------------------------------------------
PHYPA   ESDEDED-------------------------------------------
                 610       620       630       640       650
```

```
HUMAN*  --------------------------------------------------
CHICK   --------------------------------------------------
XENTR   --------------------------------------------------
DANRE   --------------------------------------------------
CIOIN   --------------------------------------------------
ANOGA   --------------------------------------------------
TETNG   --------------------------------------------------
DROME   --------------------------------------------------
BRUMA   DLTDFVNQRTENDLDELFSSRYTMANKLYVEVANGFPDPVIVCPWSKRPK
CAEEL   --------------------------------------------------
YEAST*  --------------------------------------------------
SCHPO   --------------------------------------------------
CANAL   --------------------------------------------------
CANGA   --------------------------------------------------
ASPNI   --------------------------------------------------
RHIO9   --------------------------------------------------
CRYNE   --------------------------------------------------
USTMA   --------------------------------------------------
ENCCU   --------------------------------------------------
ANTLO   --------------------------------------------------
ARATH*  --------------------------------------------------
VITVI   --------------------------------------------------
SOYBN   --------------------------------------------------
SOLLC   --------------------------------------------------
ORYSJ*  --------------------------------------------------
HORVU   --------------------------------------------------
SORBI   --------------------------------------------------
PHYPA   --------------------------------------------------
                 660       670       680       690       700
```

```
HUMAN*  --------------------------------------------------
CHICK   --------------------------------------------------
XENTR   --------------------------------------------------
DANRE   --------------------------------------------------
CIOIN   --------------------------------------------------
ANOGA   --------------------------------------------------
TETNG   --------------------------------------------------
DROME   --------------------------------------------------
BRUMA   RNFDYTGGQRRSWREDRGRNVNSEWRSGWRNPNYDPYVAHGSWKRRNADA
CAEEL   --------------------------------------------------
YEAST*  --------------------------------------------------
SCHPO   --------------------------------------------------
CANAL   --------------------------------------------------
CANGA   --------------------------------------------------
ASPNI   --------------------------------------------------
RHIO9   -------------------------------------------NEDTGGD
CRYNE   --------------------------------------------------
USTMA   --------------------------------------------------
ENCCU   --------------------------------------------------
ANTLO   --------------------------------------------------
ARATH*  --------------------------------------------------
VITVI   --------------------------------------------------
SOYBN   --------------------------------------------------
SOLLC   --------------------------------------------------
ORYSJ*  -------------------------------------------D------
HORVU   -------------------------------------------D------
SORBI   -------------------------------------------D------
PHYPA
                 710       720       730       740       750
```

HUMAN*	-	-	-	-	-	-	-	-	-	-	-	-	-
CHICK	-	-	-	-	-	-	-	-	-	-	-	-	-
XENTR	-	-	-	-	-	-	-	-	-	-	-	-	-
DANRE	-	-	-	-	-	-	-	-	-	-	-	-	-
CIOIN	-	-	-	-	-	-	-	-	-	-	-	-	-
ANOGA	-	-	-	-	-	-	-	-	-	-	-	-	-
TETNG	-	-	-	-	-	-	-	-	-	-	-	-	-
DROME	-	-	-	-	-	-	-	-	-	-	-	-	-
BRUMA	D	H	V	R	V	S	Y	P	G	R	T	N	S
CAEEL	-	-	-	-	-	-	-	-	-	-	-	-	-
YEAST*	-	-	-	-	-	-	-	-	-	-	-	-	-
SCHPO	-	-	-	-	-	-	-	-	-	-	-	-	-
CANAL	-	-	-	-	-	-	-	-	-	-	-	-	-
CANGA	-	-	-	-	-	-	-	-	-	-	-	-	-
ASPNI	-	-	-	-	-	-	-	-	-	-	-	-	-
RHIO9	P	F	W	I	L	D	L	N	P	D	M	Q	P
CRYNE	-	-	-	-	-	-	-	-	-	-	-	-	-
USTMA	-	-	-	-	-	-	-	-	-	-	-	-	-
ENCCU	-	-	-	-	-	-	-	-	-	-	-	-	-
ANTLO	-	-	-	-	-	-	-	-	-	-	-	-	-
ARATH*	-	-	-	-	-	-	-	-	-	-	-	-	-
VITVI	-	-	-	-	-	-	-	-	-	-	-	-	-
SOYBN	-	-	-	-	-	-	-	-	-	-	-	-	-
SOLLC	-	-	-	-	-	-	-	-	-	-	-	-	-
ORYSJ*	-	-	-	-	-	-	-	-	-	-	-	-	-
HORVU	-	-	-	-	-	-	-	-	-	-	-	-	-
SORBI	-	-	-	-	-	-	-	-	-	-	-	-	-
PHYPA	-	-	-	-	-	-	-	-	-	-	-	-	-

760

MED5

HUMAN*	M-------------------KVVNLKQAILQAWKERWSDYQWAINMKKFF
CHICK	M-------------------KVVNLKQAILQAWKERWSDYQWAINMKRFF
XENTR	M-------------------KLVNLKQAILQAWKERWSDYQWAINMKKFF
DANRE	M-------------------KVVNLKQAILQAWKERWSDYQWAINIKKNC
CIOIN	Q-------------------NSADLKHLVITSWKERWSEKKWSIHVKKLM
ANOGA	MIMDNSKTTS----------KTTLLKLMLLRAWKERWTDCQWGINVKTIL
TETNG	M-------------------KVINLKQAILQAWKERWSDYQWAINIKKNF
DROME	MK------------------ENNKILQLIYVAWRERWTDSQWGINIKKVL
BRUMA	MGVINEDKQQT---------DVSWVTSLVTRAHAEGLSSTEFGAVLKKRI
CAEEL	---MNSIKIDS---------STKIVIEYIDKLRNCSNEKRQKVSSVIRPV
YEAST*	--------ME-----------KESVYNLALKCAERQLTSMEFSNLYKEFF
CANAL	--------MSM---------DTISISKLIKKSINQKLPPKLFISLFNKLI
CANGA	--------ME-----------SKSLNTLALRCAARKIPASEFLNLYKEFY
ASPNI	----------------------------MQSRNTQSMASEQWRAFVQRCL
RHIO9	MT------------------SAKQLEVVLRYAYLHGLSCEQWSIPTKECL
CRYNE	---------ML---------SSPSIPQLIATAYAQALPPSRT--------
USTMA	------MSTGE---------AQQLIQKLLLEALARRLAQPKWLSLLHQLV
ARATH*	MVVPGRRT------------VWDCVIELTKMAQENCVDPRLWASQLSSNL
VITVI	MAVSVHTS------------IWDNILEHTNVAQEKGSDPLLWAVQLSSSL
SOYBN	MALPAQTSEVD---------AWEAVLRQTKVAVDTNADPNAWALGVTSTL
SOLLC	MEVSEEGN------------LWDSVLEITKVEQEKGGDPLVWAVQVSSCL
ORYSJ*	--------MVPAGGGGGE--LERRVMAALKASEARGDQPLVWAVEVARVV
HORVU	MGAAEAPALAPASGAGGELLLERRVMAAVKASEARGDPPLLRAVEVARLV
SORBI	-------------------------MAAVKASEARGDPPLLRAVELSRVV
SELML	--------------------MAERAAELARTAAERLESPLPWAVSACSVM
PHYPA	--------MQP---------SWKAVMEVTKTAMAQNDFPLVWGTDLVTCI

10 20 30 40 50

HUMAN*	PK-------------------------GATWDILN---------------
CHICK	PR-------------------------GATWDILN---------------
XENTR	PK-------------------------GVTWDILN---------------
DANRE	PK-------------------------GATWDYLN---------------
CIOIN	---------------------------TSPSDMLH---------------
ANOGA	TR-------------------------GVSGDVYN---------------
TETNG	PK-------------------------GATWDYPN---------------
DROME	PR-------------------------GVSGDVYN---------------
BRUMA	I--------------------------TSGCNEQQCRA------------
CAEEL	D--------------------------FPGISTQQ---------------
YEAST*	NEK-FPSLIQEEE--------------EDTTTTANINE-----------V
CANAL	KKQ-SINNEEFINELLILDYDQHTCSSFSSLKQHKWNE-----------Y
CANGA	NET-FPA--NSID--------------NDDAKTQEGSG-----------S
ASPNI	LHR------------------------IDAAEFRDLSMLLFQRCPIGDLL
RHIO9	VDKQFENE--
CRYNE	---------------------------LQEVHLHNLNPS-----------
USTMA	RRI-EAAQDDDGTFDV-----------IPSLEGSH---------------
ARATH*	KFF-AVE--------------------LPSTE------------------
VITVI	SSA-GVS--------------------LPSVE------------------
SOYBN	RSS-AVT--------------------LPSVE------------------
SOLLC	SSS-GVS--------------------LPSFE------------------
ORYSJ*	AGE-GAG--------------------LPSAD------------------
HORVU	AGEPGAG--------------------LPSAD------------------
SORBI	AGE-GAGAGP-----------------LPSAD------------------
SELML	HGA-GVS--------------------MPSLD------------------
PHYPA	HKH-GIG--------------------LPSVE------------------

60 70 80 90 100

HUMAN*	LADALLEQA-MIG--PSPNPLILSYLKYAISSQ------------MVSYS
CHICK	LAEALLEQA-MIG--PSPNPLILSYLKYAISSQ------------MVSYS
XENTR	LAEALLDQA-MIG--PAPNPLILSYLKYAINSQ------------MVSYS
DANRE	LAEALLEQA-MIG--PSPNPLILSYLKYAISSQ------------MVSYS
CIOIN	VSDVLVSQS-FIG--SSPNALLLRYISYALECG------------LLSYA
ANOGA	LADAIIQQA-VVG--SGANMLFLSYLKHSLCAH------------LISHA
TETNG	LAEALMEQA-MIG--LSPNPLILYYLKYAVSSQ-----------------
DROME	LADCLMQQA-LIG--STANPLVLNYLKHSLCAH------------LVSHA
BRUMA	IASMLLRFA-TAS--VTPDQRMLSCLEALVKAE------------VLRWA
CAEEL	LCDILIRFA-YNG--TYIDDVFRDHLLKLVVDK------------SITWN
YEAST*	KKASDLVDT-PSNNTAATADTTHLHEALDIVCSDFVKILNLEKPLILADY
CANAL	KQTLIIELS-FSN--VENNHLFWNNLNQIPNNL------------QCKYL
CANGA	QDKTDVEES-ISKPVGSKND------PVAILCAEFMKLLENGKYVILADY
ASPNI	LTETLLQARLATG--IKWDPLLPVYIDCLCRMG------------RVQIP
RHIO9	LCELLLSSIFSVT--KVSDPLLESYITFATTGANTLLDSTNTNNSIISLE
CRYNE	VQYDLIRAF-LDHLLPVPPSIMLAYLAHALSTS------------VITTQ
USTMA	AAECLVKYL-LSQ--SFIDPLLIEYLQALIYGSANRSGVSPDQGPITDVM
ARATH*	LAEVIVSYI----CWDNNVPIVWKFLERAMALK------------LVSPL
VITVI	VANLLVSHI----CWGNNVPIAWKFLEKALMIK------------IVPPM
SOYBN	LAYRLVSFF----FWDNHCATAWKLLHTAMSLN------------LLPSS
SOLLC	LANFLVSHI----CWENNLPIAWKFLEKALVLK------------IVSPI
ORYSJ*	LAGILVSNL----CFAHNSPSLWKLVGHAVASR------------LLCPL
HORVU	LAGILVSNL----CFAHNSPSLWKLLDQAMSSR------------LLCPL
SORBI	LAGILVSNL----CFAHNSPSLWKLLGQAVACR------------LLCPL
SELML	LAKAMVADV--------PSPQLMPFVDQSIASG------------LVFPM
PHYPA	LSSVLVHCLVTNASYASSTATIWTYIQHAMSCQ------------MVSVL

110 120 130 140 150

MED5

```
HUMAN*   SVLTAISKFDDFS--------------------RDLCV-------QALLD
CHICK    TVLTAISKFDDFS--------------------RDLCV-------QSLLE
XENTR    TVLTAISKFDDFS--------------------RDLCI-------QALLE
DANRE    SVLTAISKFDDFS--------------------RELCV-------KSLLE
CIOIN    SVITAVSKHEDLS--------------------KIEST-------KAVYG
ANOGA    AVLNRIAKYDQYD--------------------KHHCL-------IALLD
TETNG    --------FDDFS--------------------RDLCV-------KSLFE
DROME    AVIRCIAKYDKLE--------------------RVYCI-------TALLE
BRUMA    VFIEVIAEFRQFS--------------------REYCI-------EALAT
CAEEL    QVIHSVINTKTD---------------------RIYSK-------CLQCE
YEAST*   IVEVLLVNYNS--------------------------------------D
CANAL    SKITKLLSHQSQHYDK-----------------------------EVLKN
CANGA    VVEVLFVNYHS--------------------------------------E
ASPNI    TVLTALLKHSSIHDKPSPGADVASVKKNAKPRAKCYTL-------MTDIR
RHIO9    TFVPQLLKFGPSTVNRNPYQW------------------------SFLLR
CRYNE    DFTIGLLFYMSGHEIPPI---------------------------ESLVS
USTMA    TVTLHLLANVQAAASNVPAIEIISSVIGQGLLMTFQAPVPFRVSSPSFLA
ARATH*   VVLALLADRVVPTRSTQQAAY------------------------RIYLE
VITVI    PVLALLSTRVIPSRHSRPTAY------------------------RLYLE
SOYBN    LLMALLSATVVPSRQLYPTAY------------------------RLYME
SOLLC    IVFPLLSSRVIQNRRLRPAAF------------------------RLYME
ORYSJ*   HVLALLTPRVLPQRRAQPEAY------------------------RLYLE
HORVU    HVLALLTARVLPQRRAQPEAY------------------------RLYLE
SORBI    HVLALLTPRVLPQRRAQPEAY------------------------RLYLE
SELML    HMLSLLAAKVIPVRQQQPETY------------------------MMFLN
PHYPA    HMLALLTSRILPSRHQQPENY------------------------RMYLE
                  160       170       180       190       200
```

```
HUMAN*   IMDMFCDRLS----------------------------------------
CHICK    IMDMFCDRLS----------------------------------------
XENTR    IMDMFCDNLS----------------------------------------
DANRE    IMDMFSNRLS----------------------------------------
CIOIN    LLKDCLQVVC----------------------------------------
ANOGA    FLKSIIDGVT----------------------------------------
TETNG    IMDMFCHHLS----------------------------------------
DROME    FLASIVDGVT----------------------------------------
BRUMA    ILRNSVPLLK----------------------------------------
CAEEL    LIREMVNYVQ----------------------------------------
YEAST*   MIKCFLPKLN-----------------------------SVRNSLLLAHF
CANAL    FIKNEIIDYV----------------------------------------
CANGA    LVREFLPKLK-----------------------------DLMNKGILIHF
ASPNI    VIQDAMLSIN----------------------------------------
RHIO9    LLPVLLSTAV----------------------------------------
CRYNE    IVNVLMENPT------------------------------CFEDGTILPS
USTMA    LLQRLFADPT---------------YEQDQTPSFPVDHVDTGASDIKVAP
ARATH*   LLKRNMFTIK----------------------------------------
VITVI    LIKRHAFTLK----------------------------------------
SOYBN    LLKQLDDMLARWNVNCIIALVQFELNKSRMIQPKPNLASDVALSLCLCLS
SOLLC    LMRRHIFTLK----------------------------------------
ORYSJ*   LLRSNVTSSF----------------------------------------
HORVU    LLKGNITSPS----------------------------------------
SORBI    LLKCHVTSSL----------------------------------------
SELML    LLDTYAFTLS----------------------------------------
PHYPA    LTSTFVFSLS----------------------------------------
                  210       220       230       240       250
```

```
HUMAN*   ------------CHGKAEECIGLCRALLSALHW-----------------
CHICK    ------------CHGRAEECIGLCRALMSALNW-----------------
XENTR    ------------CHGKAEECIGLCRALMSALNW-----------------
DANRE    ------------CHGKAEECIGLCRAMLCTVVW-----------------
CIOIN    ------------KTRLMEDAMELCFSLRMVLKW-----------------
ANOGA    ------------CRGKQEEAI-LTKATVSLVHW-----------------
TETNG    ------------CHGKAEECVGLCRALLTVVTW-----------------
DROME    ------------CRIKSEEAV-LPSSVVHLVYW-----------------
BRUMA    -----------CNYTSEKECVELSGSLMTVLNW-----------------
CAEEL    -------IKSFSDKLHADELISIMKPTVFFLAQ-----------------
YEAST*   FSKSCSFFAKLSDTLIIDQVRKDLGNVIVPNILSLDMNSMNKELIAIVSK
CANAL    --------------MKYTSITSITTTTTTTTTTTTTTSKVSGNVLENIVL
CANGA    FSKSCAFFVNLTDNLVISQLIKDLRATIVPCILETNFCDISNELVVAIAK
ASPNI    ------------SGTTVKTLLEVIHTFCAIADW-----------------
RHIO9    ---------------STDTIVSNLQQGNPNLAWVDILSDLFCLLSHIVAV
CRYNE    MLSDPALVQSDTNGELSKYRDNNQTSTLSLLP------------------
USTMA    ASLGFVVANLRLLALAADPAIASPQNLLAPRVSITVLINVGQGLLAYVTQ
ARATH*   ------------DHISGPHYQKVMISVSNILRLSELFDLDTSKPGVLLVE
VITVI    ------------SLIDGPNYQKDMKFIDSVLHLSLAFGLQASEPGILVVE
SOYBN    MGKFPCVFLNTNDYISYQWVLRIMKSIDEVLQLSQVYSQKVWEPGVVLVD
SOLLC    ------------NHVNMLSYKKVVNFLDSILHLTEIFGVHADEPGVLVVE
ORYSJ*   -----------LSMEAGPNRDKITKSIDDALQLSKIYGFSGIDNGHVIIF
HORVU    -----------LSPLPVPNRDKITKSIDAALQLSKSYGVSGMDFGHVVIL
SORBI    -----------LSMEAGPNRDKIGKSIAEALQLSKVYGFSGTEFGHVVIM
SELML    ------------LRTTVSSTERSLKSILEILELPFN-GDAPKELGTVAVQ
PHYPA    -------------VLIGIIRFRVVKAVDEALQLSNSPDTPITEVGIVIVH
                  260       270       280       290       300
```

MED5

```
HUMAN*  LL----------------------RCTAASAERL----------------
CHICK   LL----------------------RCAAFYAEKV----------------
XENTR   LL----------------------RCSTFYTDKL----------------
DANRE   LL----------------------QGCAWYCERL----------------
CIOIN   LL----------------------QNIEQCLKRV----------------
ANOGA   LL----------------------QIYECVLQSY----------------
TETNG   LL----------------------QGCDWYCEKL----------------
DROME   LL----------------------QIFARTVQHY----------------
BRUMA   CL----------------------LAL-----------------------
CAEEL   LI----------------------QDV-----------------------
YEAST*  LL----------------------QTTLKLSPSPILLTSAGCKNGSF---
CANAL   LL----------------------SVLIDKFEKYLQEDTTNSFQNFI---
CANGA   FL----------------------QAVLRFTPQPIQINSETYRDNTF---
ASPNI   --------------------------------------------------
RHIO9   GLYPDNTQSPENSPVTLTSALSFHTNTQGGFDSQFSLASQPSISGMDLDA
CRYNE   LL----------------------RVSATPFPPPTLTAFIARLLNCLSPF
USTMA   IL----------------------SAAQ----------------------
ARATH*  FV----------------------FKMVSQLLDAALSDEGLLELSQDSSS
VITVI   FI----------------------FSMVLMLLDASLDDEGLIELTPEKKS
SOYBN   FV----------------------FSIVWQLLAASLDDEGLLDHTAENKP
SOLLC   II----------------------FSLVWQLLDASLDDEGLLQLTPEKKS
ORYSJ*  FM----------------------MFVITKLIDCTLEDCGFPSGLTEEQG
HORVU   FV----------------------LILVTKLIDSVLEDCGISSGMAQEQE
SORBI   FV----------------------LTVVNKLIDSILEDCGLPSAMAEGQE
SELML   FI----------------------LALCCRLVDATAEDWSMALSSPSKPA
PHYPA   FL----------------------FALMARLAESVYEDWKSNEGSIHGRT
                 310       320       330       340       350
```

```
HUMAN*  ---------------------------REGLEAGTPAAGEKQLAMCLQRL
CHICK   ---------------------------KEMLEQ---VAAEGQMKMCLERL
XENTR   ---------------------------KDMLDP---VAGENHLSMCLQRL
DANRE   ---------------------------RDPNDF---SALESSLRACLERM
CIOIN   -------------------------------WDMYGSTAEEIIKLNCDIL
ANOGA   -----------------------------AEDSTLQPEEEEVVQRVAVVL
TETNG   ---------------------------REMGPS---VSTETSLSACLDRL
DROME   -----------------------------ELYGEISAEQSYMLDQTCVVI
BRUMA   -------------------------------EYSLKNENKRSTQALIHCA
CAEEL   ----------------------LSDEDELEMIQIEYGDVRKAYYKPLEAL
YEAST*  --------------------------------------------------
CANAL   ----------------------VKLLSSSNLLSSLVNDDGYLTNYLLNKS
CANGA   --------------------------------------------------
ASPNI   --------------IQAIIAWHNSQLDADQPAGMMGSPDAMLLFEAVGIL
RHIO9   TLDMDNTQRMDEDVKEEISIPHRGFVTDEERRKSKRWIEAENAVAAAQIM
CRYNE   PVPPL-----------------DVGLEAGQLLPSLTDEISIPLRNCLGGL
USTMA   ----------------------TAKNDKLKPDGAILQQLRSMASTSLTEI
ARATH*  QWLVK--SQDMEID--------APERYNEK-TGSLEKLQSLNTIMAIELI
VITVI   KW-----ANDMEID--------SHDDYDEKRTDLHERLQKINTVMAIDLI
SOYBN   RWLSR--SHDMNID--------GPDSFTEKKTEQAEGFQKKNTAIAIEII
SOLLC   RWPIK--PEDVEID--------GC-IADMERNEQRERLKNLNTLLAIELI
ORYSJ*  SIYAIEGPQDMDLD--------VKGVSTEKQNEHRAQLRRKNTVMALDVL
HORVU   GVYPTEGAQPMDLD--------VKGVSALKQNEHREQLRRNNTVMALEVL
SORBI   SVYATDGPQPMDLD--------VKRGSTENQNEHREQLRRKNTLMALDVL
SELML   GSYLT--VNDFDDD--------FG-----RRRQEESEQARGNSLRALELV
PHYPA   MESGTAG-------------------HEEKHEMGLEQLKKTNSLAAVHLM
                 360       370       380       390       400
```

```
HUMAN*  EKTLSSTKNRAL-LHIAKLEEASS---------WTAIEHSLLKLGEILAN
CHICK   EKMLSSTKNRAL-IHIAQLEETSS---------WNAVEQSLLKLEESLNG
XENTR   ESMLSSTKNRAL-IHIAKLEEPAS---------WTSVEQSLLRLAESISL
DANRE   ANLLHSTKNRAL-VHIARLEEQAS---------WTNVEQAVLKLTENLSA
CIOIN   KLIATKPRTSGL-LIIARFAEGGV---------WSHIETDLAVIKNSSGQ
ANOGA   ERIVENPFLLGV-LSIGKCDDPEL---------FIALQKRFNGISNLTLT
TETNG   HTLMSSNKNRAL-VHIAWLEDQGA---------WNNVEQAVMKLTEGLDG
DROME   DRLSQQQFLLSM-LYVGCHEELEI---------CGRIRDKYATIKGSLTN
BRUMA   CAYANSKFVRAL-LYIHEHIGAES---------VNYVVEAAVSFAQSYPD
CAEEL   SAFIHDDLCSSL-LAFSEAS---------------EITHQLELCRDSFSK
YEAST*  -----------------------------------TLLNQLSQTNKLLFK
CANAL   KIILSKNQYDQI-VNVNNMVRTER---------ERERQASIVALNN----
CANGA   -----------------------------------NLTKRLSLINKILSK
ASPNI   LSVLAGTGKGIEVLSAGPHEATKI-----------RIGQALSAYLPLCAD
RHIO9   IHLIEKKGAKRI-FEVRNNLRRQAGLATTNHEPWYSCQHKLTPSDPFIKN
CRYNE   MADATTNDPQQVEQVVFQQDSQLA---------QGHSPSQVEMPTNS---
USTMA   EAVLRS-------MNPAWKDEIAL---------LRSLTSQIGVIDQISES
ARATH*  AEFLRNTVIARL-LYLVSSNRASK---------WHEFVQKVQLLGENSSA
VITVI   GQFLQNKATSKI-LYLARRNMPTH---------WVVFLQRIQLLGANSSA
SOYBN   AEFLQQKMTSRI-LSLVHWNMSSH---------WGSFIHQMQLLVSNSSV
SOLLC   GQFLQNKVTAKI-LYLARQNMPVH---------WGAFVQRIQLLAGNSSA
ORYSJ*  IMMVADRKIQSF-LRLIFLNMPEK---------FSVLSQRLSLVEAHKME
HORVU   HMMAADKKIQAF-LRLICLNMPDK---------FSVLSQRLTLIEAHKMA
SORBI   HMMAADRKIQSF-LRLIFLNMPEK---------FSSLRQRLSSIEAHKVS
SELML   TNFLHHKKTSVL-LRLARRNLSEQ---------WGLFVQKLKFL---EVI
PHYPA   AKIIQNKRTADL-LRIARRNLSVL---------ISAYLLGVV--------
                 410       420       430       440       450
```

MED5

```
HUMAN*  LS---------------------------NPQLRSQAEQCGTLI------
CHICK   LS---------------------------NSTLRSQADDCISLI------
XENTR   LP---------------------------SVQLRNQAEECVSLI------
DANRE   VT---------------------------NPTLKESLEECVSLV------
CIOIN   HPL--------------------------HSEISASCHHVSQLS------
ANOGA   TGYVANVGAGNAN----------------SLTVTDCMRKLVLLE------
TETNG   VA---------------------------NQALRTKLEECLSLV------
DROME   SNFTLN-----------------------APQVEQQLQQLAFIE------
BRUMA   -----------------------------CEELKTMIEALQQFG------
CAEEL   LRSPDKS-------------------------ATTLVEMLIERH------
YEAST*  RVSQTFEAKLHFKDTKPFLNKDSTN----EFVGSPSLTSPQYIP------
CANAL   ------------------------N----NTIDIRSGNTMQQQQ------
CANGA   KFAGIIDKKLQFKEVLGPFTKDSTL----DFDNSPSITSPQFIP------
ASPNI   MYPPLHNRLDGLQKQFNLYGQPPSK-LDVTMMDNVNVNALQFEA------
RHIO9   NASTHVSQNAHVQKLLTLVQRLADRDSERRMAVHMKYHELEDEG------
CRYNE   -----------------------------SLPLKATVAFLLEQA------
USTMA   ------------------FAEANTS----KLSTRPTKRKLETLQDAQQRA
ARATH*  LKHSKVLNSGDLLQLISNRRFGYSY------DSKVTSARKSNA-------
VITVI   LRNSKVITPEALLHLTSDTRIVLSR------KCKSSSLQKFHA-------
SOYBN   FRNLKHITAESLLRWTQNIHEVVSH------ELKKKSKMETNL-------
SOLLC   LQSSSIISPKALLQLASDAHNL-----------SKANSLQEHYV------
ORYSJ*  LE-TLLTANHKIDDLLMNMWRVSNT-------AYQPNNKRLLGV------
HORVU   LE-RLLPTSHKIDDLVMYIWRVSNL-------DYQPNNKRLVGI------
SORBI   LE-TLLPSGHKISDLLINIWRVCKT-------DYQPNNKRILGI------
SELML   IRDTTLGAPRETGELFARLAAAIQQ----GLVQEQSNRRVVYKV------
PHYPA   --------------------------------------------------
                  460       470       480       490       500
```

```
HUMAN*  -----RSIPTMLSVHAEQMHKTGFPTVHAVILLEG---------------
CHICK   -----KSIPTMLSVRSEQLNKTGFPTVHAVVLLEG---------------
XENTR   -----RSIPSMLSVHSDQLQRNGFPTVHAVVMLEG---------------
DANRE   -----KSIPQMLCLQCDPPVHTTFPSVHAFIMLEG---------------
CIOIN   -----KCSISPKPVYKLPVSSTLCQSIMSLILLEV---------------
ANOGA   -----VDALEMRPFD-GGKVEPITYSLQPLIAIEV---------------
TETNG   -----KGIPTMLAVQCDQPVHALFPSVHAFIMLEG---------------
DROME   -----AKHLEMQPLNPPSTLEKISCCVQPLLAVEV---------------
BRUMA   -----ASPAERIEDNLGGLVKQSHPAILTLVSVFE---------------
CAEEL   -IEKCKPVKFQYIPEGIALFDLKNPSIRILIPIFA---------------
YEAST*  -----SPLSSTKPPG--SVNSAAKYKDMKLLRYYK-----------NIWL
CANAL   -----------------QNVNVASLKMVNLIDEYY-------TKMRQIWI
CANGA   -----SPLPSMKERSVTSSQSAIKYKDLKLLRYYK-----------NIWL
ASPNI   -------------------SVMDGPTIHSRAGLYI-------------YI
RHIO9   -----------------TARAMPSAGIMGFLYHMV---------------
CRYNE   -----------------HRASQWDPSELALEMEHY-------QAPAPSYV
USTMA   TWDHFLDVSTIAASEKPAVEPETALLMHLLVDHHT---------------
ARATH*  --------------------IVDFGSLSSYAGLCHGASLSSLWLPLDLVF
VITVI   --------------------VMAFGSLASSAGLCHGASRSALWLPLDLVL
SOYBN   --------------------VTPAGSLMPLAGQSYGDSWSSLWLPIDLIL
SOLLC   --------------------RSTSRSLATCAGLCFGSSRSSLWLPLDLFL
ORYSJ*  --------------------LGNMKYGGSMLGQFTGAGRAACWVIFDIYV
HORVU   --------------------LGNLRSSNSMLGQLTGAGRAACWIIFDIYL
SORBI   --------------------LGNMGSSGSLLGQLTGAGRPACWIIFDIYV
SELML   --------------------LLDTNTSMSVFGNYWGTGRTAPWLPFDIFM
PHYPA   -------------------------SLTFLTFMTGGLGYSSPWLPFDIYM
                  510       520       530       540       550
```

```
HUMAN*  -------TMNLT---GETQSLVEQLTMVKRMQHIPTPLFVLEIWKACFVG
CHICK   -------TMNLT---GEIQPLVEQLMMVKRMQRIPPPLFMLEIWKACFVG
XENTR   -------TMNLT---GDAQPLVEQMNTVKRMQRIPSHLFLLEVWKACIVG
DANRE   -------TMNLT---GETQPLVEQLMMIKRMQRIPAPLFVLEIWKACFTG
CIOIN   -------EERKL---TNIEAIVQQLILLCNIHGLSTSNLIYHLIQTCFMG
ANOGA   -------LLNPM---CDTHQYVAQLINLKRLKGFTYARLYCELMRGCLIA
TETNG   -------TMNLT---GETQPLVEQLMMIKRMQKIPTALFVLKIWKACFTG
DROME   -------LLNPC---KDTSYYVAELQMLQRLKKYSNTRLFYEIIRAGFLT
BRUMA   -------GFRMT---KKVDQMADFFFELAEILEFPFPKMFEDMIRASLLI
CAEEL   -------CFKNH---ESSKLMAETIQTFAEIMRFYGPDVIFDILHGAILL
YEAST*  NNKIINWEISNPDFLSKYSAITSSIFQESFNSVQNLDQLLTDLIETSFTC
CANAL   RKIFHNFEYDNN---NNILTIFMGNFIPSSIQRNPY-LIAYEFIKTIFGC
CANGA   NSKLMNWQPFDSEFISNYSAIKSSLYPDQVQNIQNVDLLFTDLIETAFTC
ASPNI   NAMLVGRPLVDD---------SILLNYLYNRYGEHYDVLIEEIITASFDV
RHIO9   -------QIRPS---LEDDFIVDHLLKLQTVKGSFDESFYLEIWFTALTG
CRYNE   RMLKAGQHICSN---PEEFIVVLFEVSIERLISEDGRSLI-----AKAEG
USTMA   -SWDFKLDAIKTLFLARRSAAAPSLTLEKSLTA-----FYFELLVAAIDA
ARATH*  EDAMDGYQVNPT---SAIEIITGLAKTLKEINGSTWHDTFLGLWIAALRL
VITVI   EDAMDGSLVSAT---SAIETITGLIKVLQAINGTTWHDTFLGLWIAALRL
SOYBN   EDALDGAQVAAF---SAIEIITGMVKTLHVVNGTMWHNTFLGLWVAALRL
SOLLC   EDAMDGSQVNAT---SAIEIITDLVKSLQAINATTWHETFLGLWMAALRL
ORYSJ*  ENAIDGKHLSAI---SAIEVLKEMTKTLQAINEASWQETFKALWISALRL
HORVU   ENAMDGKHLGGI---SAIEIIKEMSKTAQAINEASWQETFKALWISALRL
SORBI   ENAIDGRHLNLI---SAIGIIKEMTKTMQVLNEASWQETFKALWVSALRL
SELML   EDALEGRRVPAS---STAEALADLIKSLRAVQGASWHDVFLGLWIAGLRH
PHYPA   EAAMEGRRLSQR---SNAEILADVMKAMQSVHAANWVDLFLGLWTAGLRL
                  560       570       580       590       600
```

MED5

```
HUMAN*   LIE-------------------------SPEGTEELKWTA-----FTFLK
CHICK    LIE-------------------------SPEGTEELKWTA-----FTFLK
XENTR    LIE-------------------------SPEGTEELKWTA-----FTFLK
DANRE    LIE-------------------------SPEGNEELKWTA-----FTFLK
CIOIN    YLD-------------------------SRGNDLEQEWCA-----MVFLR
ANOGA    LNN-------------------------VEGTAKESLLCA-----FAFIK
TETNG    LIE-------------------------SPEGTEELKWTA-----FTFLK
DROME    LSN-------------------------VADTSPDTMWGA-----FMFFK
BRUMA    MLD-------------------------AKESLGEELVASQA---FYYVK
CAEEL    KCE-----------------ESMDLLHFPKQHRADFRWQSTT---FFYKK
YEAST*   FAQ--------------FVSNKQYHQANSNLTLLERKWVI-----FITKH
CANAL    LNM--------------------------------KGYCLFNVKNYILTR
CANGA    FAQ--------------FVSNKLYHQSNSTYNLLERKWIL-----FLSKI
ASPNI    LSN--------------------------AMYRNESARTMLLFRSFLVNK
RHIO9    LREAALNTSCHGPHLEKDSNENQDKGCNIVVATNRLLWKS-----LVLVK
CRYNE    IRDWGWVTESLPNLLRWWRSQREEDG-------------------FVGWQ
USTMA    CAA----------------------VVEMPPAFKGAEVYATIWRNALCGM
ARATH*   VQR-------------------ERDPIEGPIPRLDTRLC------MSLCI
VITVI    VQR-------------------ERDPIEGPMPRLDTRLC------LLLSI
SOYBN    VQR-------------------ERDLKEGPIPRLDTCMC------MLLSI
SOLLC    VQR-------------------ERDPIEGPVPRLDTRLC------MLFSI
ORYSJ*   VQR-------------------AREPLEGPIPHLDSRLC------MLLAL
HORVU    VQR-------------------AREPLEGPIPHLDTRLC------MLLAL
SORBI    VQR-------------------AREPLEGPIPHLDSRLC------MLLSL
SELML    VNR-------------------EREHVEGPRPHVESRLC------MLLSI
PHYPA    VKRIVSFYSVVFCWFMHSTATLDRESMEGPNTHVESRLC------MLLSI
                  610       620       630       640       650
```

```
HUMAN*   IPQVLVKLKKYSHGD-------------------KDFTEDVNCAFE-FLL
CHICK    IPQVLVELKNYPHGD-------------------KDFTEDVNCAFE-FLL
XENTR    IPQALAKLKKLSPME-------------------QDFNEDMKCAFE-YLL
DANRE    IPQVLLRLKKYPQGEKT-----------------QDFTEDVNVAFE-YLL
CIOIN    LPKIMVQLKAQLTGSDFESPSKLSSKKI------AQFGVDLQEALK-MLL
ANOGA    VPHILHQLHAKTNGAEQQDEATG-----------ATYSVEVLEAFE-LLL
TETNG    -----------------------------------DFMEDVNVAFQ-HLL
DROME    MPHIIKQLHALQRIPGEQPPP-------------ADYIPELVEALE-LLI
BRUMA    LPQIVKHLLLL-----------------------GAPLDDLITALD-TIC
CAEEL    LPQIIEYLIRSE----------------------KLTVAEVQFGLEKALT
YEAST*   LPLLILENSSRSPRVVTNAL--------------DNIDEKVVKAIR-IYF
CANAL    LPILFNNFRFNNE---------------------DNLEKAVIDALK-SIE
CANGA    LPLLVYKNSSRTAHVIGNAL--------------DGIDDKVIKAIS-AYY
ASPNI    LPPFFAAMLAVPMVTFPMEECISHAL--------SRIDPNIFPSFS-QMF
RHIO9    LPAIISKLQERKTHQGVSTSSS------------VEEMNAVESSLR-ELK
CRYNE    LPSVVAEPLTKAVQVFLPAM--------------QSYSEKVLETYE-VMV
USTMA    IPELVLQLEQWLDGNQELPLRGQRL---------EAPHVRLEAALRAALL
ARATH*   VPLVVANLIEEGKYESVM----------------EKLRDDLVTSLQ-VLG
VITVI    TTLVVADLIEEEEKKHVP----------------GKCRKDLVSSLQ-MLG
SOYBN    TTLVVTNIIEEEEGQLIEEPEHSPTNQGKNKLALGKCHGELITSLQ-LLG
SOLLC    ITLVIADLIEEEESEACDEIESSIGRHMKKQVE-GTRREDVISCLQ-NLG
ORYSJ*   IPLSIGAILKEETDVHGAQGSKS-----------LPKTSGLVSSLQ-DLI
HORVU    IPLAIAAILMEETDACGAEGNKS-----------LPRRLGLVSSLQ-DLV
SORBI    IPLAVAEILQEESDMLGAEGNKI-----------LPQRQGLISSLQ-DLI
SELML    VPLASAAVIEEEENSQQYNISRVDDSE-------RGRRAAFVSSLQ-VLG
PHYPA    LPLAAGIVIEEEERGQ-PHPENISGDDKDRKIP-GGRRAALETCLQ-VLG
                  660       670       680       690       700
```

```
HUMAN*   KLTPLLDKADQ---------------------------------------
CHICK    KLTPLLDKADQ---------------------------------------
XENTR    KLTPLLDKADQ---------------------------------------
DANRE    NLTPLLDKADQ---------------------------------------
CIOIN    KLDSLLDTIDQ---------------------------------------
ANOGA    QDTPVLDYMDM---------------------------------------
TETNG    KLTPLLDKADQ---------------------------------------
DROME    EDNLLLDFMDT---------------------------------------
BRUMA    DNKTLLNQVDM---------------------------------------
CAEEL    DLSMMFDAADV---------------------------------------
YEAST*   TEKDDNKTNNE---------------------------------------
CANAL    FDDN----------------------------------------------
CANGA    QENDDGRSRND---------------------------------------
ASPNI    SMQG----------------------------------------------
RHIO9    GFTGLLNACSQPACCPEFYAPDSMYSTLVDKIAFGEEDDEDEDDIMNMIN
CRYNE    QSAEIEESSG----------------------------------------
USTMA    VMSDRLNVCES---ATNQSHAHGNVAATNGEEAAGAHANMMSDV------
ARATH*   DFPGLLAPPKCVVSAANKAATKAILFLSGGNVGKSCFDVINMKD------
VITVI    DYEGLLTPPQSVISAANQAAAKAMMLVSGINVGSAYFEFISMKD------
SOYBN    DYESLLTPPQLVLVEANQAAAKAIVFLSGNPVGSGCFKYMSTND------
SOLLC    DYQSLLTPPQAVTNAANQAAAKAMMFRSGAN--TSYFECINMKD------
ORYSJ*   QYSGLLVPPSSVVNAANAAASKAAAFKANYKSGGGNPGMMGQND------
HORVU    QYSGLLVPPSSLVNVANVAASKAAIFRANYKVGGGNPSMIGQSD------
SORBI    QYSGLLVPPSSVVNAANAAASKAAIFKANYKAGVGNSSMMDQTD------
SELML    QFEGLLCPPPIAVPAANQAAMKASAFVAGIKTTRDGY---VPAD------
PHYPA    QFESLLVPPTAAVTAANQVAAKVAAFVST----GGQKMNVEISN------
                  710       720       730       740       750
```

MED5

HUMAN*	-------------------RCNCDCTNFLLQE--CGKQGLLSEASVNNLM
CHICK	-------------------RCNCNCMSLLLQE--CSKQGLLSEANMTNLT
XENTR	-------------------RCNCDCVSLLLQE--CNKLGLLSDQNLENLV
DANRE	-------------------RCNCDCLSLLLQE--CNKLGLLSEANTATLT
CIOIN	------------------ICDGINCLKLLTEQ--FVKRELITDAYHQDLQ
ANOGA	-------------------KCACNVVECLLKE--MVKLQLLTDTHVKQLV
TETNG	-------------------RCNCDCLGMLLQE--CHKLGLLSEVNTSCLT
DROME	-------------------KCSCNMIEFLLND--WTKQQLVNDVHVKKFA
BRUMA	-------------------KTKTNTFQHLLEQ--MAAAGVIDESVLKNLL
CAEEL	------------------AWQNASFLTLLNELEPVLGKTITDP---LRLR
YEAST*	-----------DLFDDYPSTS-LDIRHDFIKG--LIMLNLQPASVINNYL
CANAL	----------------------DEFKQLFLKGFSITTTTTNEESVVGFNL
CANGA	-----------DLFDDYPSTS-LDIRHDFIKS--LTMLGVVPPVFITNYL
ASPNI	------------------NAALSDVRQEFLFS--CASHKLIPESSIERLL
RHIO9	DINYSTDLNGPGVVKSIRSLSSNDIYTNLVYV--CERQGFVRPHMVAELL
CRYNE	-------------FTPPESWQLVSLQEYLIGK--LIEMELITRTE-----
USTMA	-------LGLG-------TPPSQPIRAWLLRA--CIEHSLARPEAIADEF
ARATH*	-------MP---------VNCSGNMRHLIVEA--CIARNILDMSAYSW--
VITVI	-------MP---------INCSGNMRHLIVEA--CIARNLLDTSAYFW--
SOYBN	-------MP---------MKCSGNLRHLIVEA--CIAKKLLDTSAYLW--
SOLLC	-------MP---------TNCSGNLHHLIVEA--CIARNLLDTSAYFW--
ORYSJ*	-------SS---------TKTVGNMLHLIVEA--CISRNLIDTSSYLW--
HORVU	-------SS---------TKAVGNMLHLIVEA--CISRNLIDTNAYLW--
SORBI	-------SS---------MKAAGNMLHLIIEA--CISRKLIDTSAYLW--
SELML	-------GS---------TKAVGNMRHLIVEI--CISRGLLDASAYLW--
PHYPA	-------SG---------KSAVGTMRHLIVDS--CVSRGLLDNSAYFWLV
	760 770 780 790 800

Highlighting is not shown above: USTMA "LLRA--CIE" is highlighted in green.

HUMAN*	AKRKA--DREHAPQQKSGENANIQPNIQ-----------LILRAEPTVTN
CHICK	DKRKA--DREDAPQLQSAENANIQPNPR-----------LILRAEPTVTN
XENTR	AKRTA--DREQAPRMKSAENSTIQPNPG-----------LILRAEPTVTN
DANRE	AKRSE--DREHAPRLKTAENANIQPNPG-----------LILRAEPTVTN
CIOIN	NIRCS--MIENMPVNNVKERCLNPV--------------IILKAENNIDQ
ANOGA	ALREP--VTAGLHKLD-ANSAPLTMMLK-----------FVFRVESPLAG
TETNG	AKRYKFRLREFAPRLKTAENANIQPNPG-----------LILRAEPTVTN
DROME	SQREA--ASQLLKKCD--NGQQTPSNIN-----------FIKRAEVPLSG
BRUMA	EKRSQYWPELFQMLAQTSTTPVHQP--------------LILRANSAKMA
CAEEL	RREHMKTNDQLLPFADTDNQLIENTDID-----------------KLLNA
YEAST*	REDQMI-DTSILPTRDDLFVRNLQGIQE----V------VHNTNSFIISS
CANAL	REKF---NGKLLDINSEFTSLEESGLIE----F------INSLASSSSSS
CANGA	RGDQTV-DSKALATTDDLTFTNQQGIIE----I------VNDIPNFIRSS
ASPNI	GEN----PMQTLPVGGSYMKDELKAQIS----------ANHERAEQLISD
RHIO9	GNK----NQSLMDIDQDNEERSSPAVVV--------DQNLDQRMESLRAN
CRYNE	-------ACKMIPTISVPQTVQGQSLLE----------RMESESRAHVKP
USTMA	ANGHK--------LASEVQSLTQSLRMD----AQLEGLALNTLFETRIPT
ARATH*	-PGYVNGRINQIPQSLPNEVPCWSSFVKGAPLNAAMVNTLVSVPASSLAE
VITVI	-PGYVNGRINQIPHSIPPQVLGWSSFMKGAPLSPVMINALVSTPASSLAE
SOYBN	-PGYVNTCSNQIPCSISNHVSGWSSLMEGSQLTPALVNVLVATPASSLAE
SOLLC	-PGYVNGRLNQLAHSMPTQVPGWSSFMKGAPLTPAIINALVSAPASSLAE
ORYSJ*	-PGYVVSSGHLKD-ATLPQESPWLNFMQGAPLSGPLIDALIATPASSTTE
HORVU	-PGYVVLTGHSKD-TALPQESPWVNFMQGAPLSDPLKNALIATPASSVAE
SORBI	-PGYVVPSGTLKD-TALPQESPWLNFMKGARLSGPLIDALVATPASSVAE
SELML	-PGYA----VAIPLSGSSQSSPWAAFMEGSSLAGPLKGALINTPASSVAE
PHYPA	TGGQL----ANIP-SSPSQPSPWSVFMDGAPFSGSLRVALMSCPAGSVAE
	810 820 830 840 850

Highlighting is not shown above: CANAL "SSSSSS" is highlighted in yellow.

HUMAN*	ILKTMDADHSKS-----------------------PEGLLGVLGHMLSGK
CHICK	ILKTMDADHSKS-----------------------PEGLLGVLGHMLSGK
XENTR	ILKTMDADHSKS-----------------------PEGLLGVLGHMLSGK
DANRE	ILKTVDADHSKS-----------------------PEGLLGVLGHMLSGK
CIOIN	LVKLLSSTDFSE-----------------------PERLLNTFAGMMSIR
ANOGA	ILKALSTDCSKV-----------------------QDALLAMLCQVLTGN
TETNG	ILKTVDADHSKS-----------------------PEGLLGVLGHMLSGK
DROME	VLKTLCTNKVQD--------------------------MVNVLCQVPVGN
BRUMA	VDKILRQPNNAV---------------------------INVLLKLASGS
CAEEL	VKEVMNLQFGQT------------------------EEFSKLFVDKVKTG
YEAST*	LDTLELESITESITHDSSNGLFQVLHNFESVAPTKQREIVKAFLSIFEDA
CANAL	SSPSSSSSPTST--------------TIMFRLAKLQQEFTQLVLNIIDEL
CANGA	LEGMEMENVSEP-TLVSSNGLLQVLSNFDTVSPTKQFELANAIVDMLSES
ASPNI	IESTE----------------------------GNAGAIVGAITEVMHDL
RHIO9	LSIVELTELLHI----------------GLVSSIHLRRVLDFILEILQEK
CRYNE	LVFIITYAFGAA------------------------KSFAYEIVNIIKSS
USTMA	DHPVELLQRVAS-------------------DPGTHFIFARQLVLQVQGW
ARATH*	LEKLFEVAVKGS-DDEKISAATVLCGASLTRGWNIQEHTVEYLTRLLSPP
VITVI	LEKVFEIAVRGS-DDEKISAATILCGASLIRGWNIQEHIVHFITRLLSPP
SOYBN	IEKIYEIAINGS-DEEKISAATILCGASLVRGWNVQEHTVLFITKLLSPI
SOLLC	LEKIFEMAVKGE-DDEKIAAATILCGASLIRGWNIQEHTVNFITRLLSPP
ORYSJ*	LDRLYHIALNGS-EEEKSAAAKILCGASFVCGWNIQEYVVRMVVKLLSPP
HORVU	LDKLYHIALNGS-EQEKSAAAKIVCGASLVRGWNIQEHVVRMVVKLLSPP
SORBI	LDKLYSIATNGS-EEEKTAAAKILCGASLVRGWNIQEHVVGMVVKLLSAS
SELML	LEKVYQIAINGA-ENERVAAASVLCGASLVRSWSIQEHAVRLAVRLVSPP
PHYPA	LEKVYKTAIVGP-EEERAAAASILCGASLIRSWNVQEYAVHFAVQLLSPP
	860 870 880 890 900

Highlighting is not shown above: CANAL "SS" at the start of the row is highlighted in yellow.

MED5

```
HUMAN*   S-------------------------------------LDLLLAAAAATG
CHICK    S-------------------------------------LDLLLAAAAATG
XENTR    S-------------------------------------LDLLLAAAAATG
DANRE    S-------------------------------------LDLLLAAAAATG
CIOIN    S-------------------------------------FSALIAAATTTG
ANOGA    S-------------------------------------LDLVLSVASVEG
TETNG    S-------------------------------------LDLLLAAAAATG
DROME    S-------------------------------------FELILSVATVEG
BRUMA    G---------------------------------GLFTFDSVCASFCADG
CAEEL    Q----------------------------------TDNFDAITSILITEG
YEAST*   I---------KELNYNRIAK------ICALLFFNFSHSLTTILSFSSPAA
CANAL    K---------YSRDFEKLNR-------LILAIMNNIEIVN-IILFNSNLS
CANGA    S---------TTFELNTFVK------LTAVLTFNYSHSLTSILMYVTPEV
ASPNI    C----------------MQKETMTLKNICNSLSRRPQSLDVMLLFRSPRQ
RHIO9    T----------------LENNFHSVSKICDALIECPCSVDLILQLYTPSH
CRYNE    P-------------------QVPPPENLFLHVSYQPSIFSALVTFISPLS
USTMA    L---------EQHDLESIAR---WCKALTENACEGGAMLDTIMIYIDPAQ
ARATH*   VPADYS----RAENHLIGYACMLNVVIVGIGSVDSIQIFSLHGMVPQLAC
VITVI    VPADYS----GSDSHLIAYAPMLNILLVGIASVDCVQIFSLHGLVPHLAG
SOYBN    DPPNYS----GAESHLTSQAPFLNVLLIGISSMDCVHIFSLHGLVPLLAP
SOLLC    VPTDYS----GKDSHLIGYAPMLNVLLVGIAPVDCVQIFSLHGMVPQLAA
ORYSJ*   LPSNSSTQ--GSMSHYLAQMSTLNALLLGISYGDAIHIISLYGMVPDVAA
HORVU    LPSDSSLQ--GSMSHYLSQKSTLNAILLGVSYVDAVHIFSLYGMVPDVVA
SORBI    LPSDSSTSTPGSMSHYLAHMSTLNEILLGVSYGDAIHILSLYGMVPDVAV
SELML    VPAES-----RSGHPLMNYSSMLLAALGALTEVDAVHVLSLYGMFPELAA
PHYPA    VAENWG----GNSNPLIGHAPMLYAALQGMNTADAMNVLSLFGMFPELAA
                910       920       930       940       950
```

```
HUMAN*   KLKSFARKFINLN------------EFTT-YGSEESTKPA----------
CHICK    KLKSFAWKFIKLN------------EFTK-HISTENSKSA----------
XENTR    KLKSFARKFIQLN------------EFTR-HIGVESSKAA----------
DANRE    KLKSFARKFIKLN------------EFPK-HISGEGSKSA----------
CIOIN    QLPAFANALIDCN------------DAMK-SCPAETSKNA----------
ANOGA    KFKAFISGLLKCN------------DYAKQATTAEIGKAP----------
TETNG    KLKSFARKFIKLN------------EFPK-HISGEGSKSA----------
DROME    RLKTFVSRLIQCN------------ENSK-PVPGDLGKLC----------
BRUMA    NLTYFSSKLAMIN------------GQSERPAEGLDMDDE----------
CAEEL    RLMEVGKAFVLKN------------------VEAQRSSAV----------
YEAST*   LMKTLIKFVDLSR--------NGRN-GSN-GNDESSEYET----------
CANAL    LLYKLIDIIDSSNFTTDDNGNGNDKNNGN-DNDNNNNNDNDNDDDDNVGV
CANGA    LTKLYLEFVDKHW--------NSQVIGKQ-ETDGESQFEN----------
ASPNI    ILQPLCALLDSWH-------------------WDEDQGES----------
RHIO9    LLTPLENFCNHWT--------------PESTSIDFDMDEGEQKTTPEQGL
CRYNE    LYTLMSDRLLEAY--------------SE-LDEFARSDDP----------
USTMA    MLDPLASILDHQD-------------------LGQTSDEP----------
ARATH*   SLMPICEEFGSYT--------------PS-VSWTLPSGEA----------
VITVI    SLMPICEVFGSCV--------------PN-VSWTLTTGEE----------
SOYBN    GLMLICEVFGSCV--------------PD-SSWTLASGEK----------
SOLLC    SSMTICEVFGSCA--------------PN-ISWTLTTGED----------
ORYSJ*   ALMPICEVFGSIP--------------PP-SNHKPAIVGE----------
HORVU    ALMPLCEAFGSMP--------------PP-SNHRSTIFDE----------
SORBI    ALMPLCEAFGSIA--------------PP-PNHKSTILGE----------
SELML    ALLPICEVFGSAT--------------PA-PQ---STGEE----------
PHYPA    SLLPICEVFGSLS--------------NS-KPVATVAGED----------
                960       970       980       990      1000
```

```
HUMAN*   ---SVRALLFDISFLMLCHVA------------------------QTYGS
CHICK    ---PVRALLFDISFLMLCHVA------------------------QTYGS
XENTR    ---NVRALLFDISFLMLCHVA------------------------QTYGS
DANRE    ---SVRALLFDISFLMLCHVV------------------------QTYGS
CIOIN    ---QIRAVLFDITFLMVCYMI------------------------KLYGK
ANOGA    ---TTRSALFDVSFMILASIA------------------------QSYGS
TETNG    ---SVRALLFDISFLMLCHMV------------------------QTYGS
DROME    ---VIRSTLFDVSFLMLTSIV------------------------QTYGS
BRUMA    ---RQRALAFNLSFILLTRIR------------------------FIYD-
CAEEL    -GVNERIQIFDDTFILLSRILIKNPSVSINMFVNGGPGKSDAEQTMFYKW
YEAST*   ---INISLSFSWAILLIINLT------------------------QTYGI
CANAL    ENFQDYYSYCGNLIISVITIV------------------------EIFQI
CANGA    ---VNISMSFSWAILMLTILY------------------------KQYHI
ASPNI    ---QPVYDEFGSILLLVLTFK------------------------YRYDL
RHIO9    DGIQLLYNKFGKVWHLIFSVM------------------------NRFKL
CRYNE    ---QGYLTRFGEGVILLESFA------------------------REFNL
USTMA    -------STLSDILLFVQLLC------------------------YRYST
ARATH*   ---ISAYSVFSNAFTLLLKLWRFNHPPIEHGV--GDVPTVGSQLTPEHLL
VITVI    ---INAHAIFSNAFTLLLKLWRFNHPPLEHGV--GDVPPVGSQLTPEYLL
SOYBN    ---LTHWEVFCNAFTLLLRFWRFDHLPIEQVRSDATTPPFGSLPSPECLL
SOLLC    ---ISVHAVFSNAFALLLKLWRFNHPPIEYRV--GDVPPVGCQLTPEYLL
ORYSJ*   ---ISVYSVFSCAFLCLLRLWKFYKPPQEYCLA-GRGGSVRLELTLDYLL
HORVU    ---TSVYSVFSCAFLCLLRLWKFYKPPQEYCLA-GRGGSVRLELTLDYLV
SORBI    ---TSVYSVFSCAFLCLLRLWKFYRPPQEYCLA-GRGGSVKLELTLDYLL
SELML    ---VSPHMVFSVAFLLLLRLWKFHRPPLEHRLL-GFESPLGGDLSLDYIL
PHYPA    ---VTAHMLFSVAFLQLVKLWKFHRPPLEHCLL-GSGASLGADLSLEYLL
               1010      1020      1030      1040      1050
```

MED5

```
HUMAN*  EVI-----------------------------------------------
CHICK   EVI-----------------------------------------------
XENTR   EMI-----------------------------------------------
DANRE   EVI-----------------------------------------------
CIOIN   ETT-----------------------------------------------
ANOGA   DVI-----------------------------------------------
TETNG   EVI-----------------------------------------------
DROME   DVV-----------------------------------------------
BRUMA   DLRPSEL-------------------------------------------
CAEEL   SMRYVKRVAKHRDPEPKDETEYIALRKEVEMLMRLANAEVGIEDDLENDV
YEAST*  SVVDV----------------------------ALKYPELSIKNSFIINF
CANAL   DLSKLEIKLLSGSSESFIIDYL-------------------------NNF
CANGA   DFVSM----------------------------RADYTTDNIKNSFAITF
ASPNI   RPWDL-------------------------------------------GI
RHIO9   ------------------------------------------YKDMDSVF
CRYNE   DLPEL---------------------------------------------
USTMA   PITRI-------------------------SRYTVSNHDMDTVDSSAPLH
ARATH*  SVRNSYLVSSEILDRDRNRKR-----------------------------
VITVI   LVRNSHLVSSGTI-HNRNKTR-----------------------------
SOYBN   LVRNCKLASFGRTEKDQQRLK-----------------------------
SOLLC   LVRNSHLVSSENMLKDPNRRR-----------------------------
ORYSJ*  LMRNNHIDFANSSASSRNSSN-----------------------------
HORVU   LMHNSRIEFPNSSATSTNSGS-----------------------------
SORBI   LMRNKGIEFSNSSAPNRASYN-----------------------------
SELML   QLRNLALSSQGTQ-------------------------------------
PHYPA   QLRNMQLASPSDRFGKQRMQV-----------------------------
                 1060      1070      1080      1090      1100

HUMAN*  ---------------------------LSE--SRTGAEVPFFET------
CHICK   ---------------------------LSE--SRPADEVPFFET------
XENTR   ---------------------------LSE--GCPSGEMPFFES------
DANRE   ---------------------------LSD--PSPSSETPFFET------
CIOIN   ---------------------------VGSYIVRSKEETPILLQ------
ANOGA   ---------------------------LAE------GGNSFFEK------
TETNG   ---------------------------LSD--PSPSGETPFFET------
DROME   ---------------------------LSE------RGDSFFEK------
BRUMA   ---------------------------VNGTVRGMDNTQSCFYKFASQYG
CAEEL   EEEMPEVQEPNIETQNVDEISIPDPLHLLI------ENDPTVTAESKETE
YEAST*  ISNLPNVSDKYYLEESNVNDSD-----MLT------KSHNTVQS------
CANAL   YYRMCDNLVNTEPTNCDNDDRI-----IID------NYNNLMID------
CANGA   VENLPDISDLFFIDEKNSDDPE-----VQV------KSHKLVRD------
ASPNI   ASNDSFIIKLIEQGSSSQKLDE-----LTE------KQNKNLGE------
RHIO9   KEPKGFTFQFFKRGTIIYGEDIDDI-----------NMDQLVNS------
CRYNE   ----------LANGRRATGYGL-----LDA------EQKDCLNG------
USTMA   RSMPPFLATHLATSSVSYPLSV-----LSE------EDRGLVSR------
ARATH*  ---------------------------LSEVARAASCQPVFVDSFPKLKV
VITVI   ---------------------------FSGVASSSSEQPIFLDSFPKLKV
SOYBN   ---------------------------RWPKILCFSVEPVFMDSFPKLNF
SOLLC   ---------------------------LATVARSSFPNPIFVDSFPKLRV
ORYSJ*  ---------------------------NIGPLNEVPAQPLYIDSFPKLRA
HORVU   ---------------------------SMGSFGEVPTQPIYIDSFPKLRA
SORBI   ---------------------------NMGSVNEVPAQPIYIDSFPKLRA
SELML   -----------------------------------PVHHVKLDSFPKLKA
PHYPA   ---------------------------LGSTYTPSSGSVVSLDSFPRLQI
                 1110      1120      1130      1140      1150

HUMAN*  W-------MQTCMPEEGKILNPDHPCFR-PDSTKVESLVALL--------
CHICK   W-------MLTCMPEEGKILNPDHPCFR-PDSTKVESLVALL--------
XENTR   W-------MQTCMPGEGRVLNPDHPCFRHPDIAKVEALVALL--------
DANRE   W-------LQTCMPEEGKTLNPDHPCFR-PESGKVESLVALL--------
CIOIN   W-------INIWWTDKI---TQISTLKSEPEPNRVENLIPVL--------
ANOGA   W-------VRDYMVERKKTKSPMTMVKQ-ADPMVLEELIMAM--------
TETNG   W-------LQTGMPEEGKTLNPDHPCFR-PEPGKVESLVTLL--------
DROME   W-------VRECMVERNKLKNPRQILAL-CDDSIVDELLLSF--------
BRUMA   WTAADSCSLSNAYTNEQKAT-------------FVDRVNM----------
CAEEL   EVKFNPEGVEQMDTNEKPAE-------------ISDSLIEPTLLEEPKDI
YEAST*  W-------LCDLFVNGS----------------ITDQLIQNI--------
CANAL   W-------VNALFDDRNEG--------------LSDELIKSL--------
CANGA   W-------LNDLFVNGS----------------LSDSLLQNI--------
ASPNI   W-------IAALFIAEG----------------ISEETMSAC--------
RHIO9   W-------LSTLAGRGR----------------DINNLLEST--------
CRYNE   W-------VKAIFGSDG----------------IEDQILLAT--------
USTMA   W-------IHALFGNEG----------------ISDDLISAS--------
ARATH*  WYRQHQRCIAATLSGLT-HGSPVHQ--------TVEALLNMT--------
VITVI   WYRQHQACIASTLSGLV-HGTPVHQ--------IVDGLLNMM--------
SOYBN   WYRKHQECIASFRSGLV-PGRPVNQ--------IVDALLSMM--------
SOLLC   WYRQHLACIASTLCGLV-NGTLVCQ--------TVDVLLSMM--------
ORYSJ*  WYFQNQACIASTLSGLC-NKNPVHQ--------VANKILSMI--------
HORVU   WYVQNQACIASTLSGLG-NTNPVHQ--------VANKILSMI--------
SORBI   WYFQNQACIASTLSGLC-NKNPVHQ--------VANKILNMI--------
SELML   WYTQNQACVASTLSGLSGSGNPVHQ--------NADRLLNMM--------
PHYPA   WYMQHQACISATVSGLL-RNNPMHQ--------VGDRLLAMM--------
                 1160      1170      1180      1190      1200
```

MED5

```
HUMAN*  ---------- ---------- ---------- ---------- ------NNSS
CHICK   ---------- ---------- ---------- ---------- ------NNSS
XENTR   ---------- ---------- ---------- ---------- ------NTST
DANRE   ---------- ---------- ---------- ---------- ------NSSS
CIOIN   ---------- ---------- ---------- ---------- ------RSSQ
ANOGA   ---------- ---------- ---------- ---------- ----NNSEGM
TETNG   ---------- ---------- ---------- ---------- ------NNSS
DROME   ---------- ---------- ---------- ---------- ----SKPEAS
BRUMA   ---------- ---------- ---------- ---------- ----------
CAEEL   LENIIVPIIK VEAPKPDSPI KKKKDPDVTW HEVHCPLPRI SRRTARAYLA
YEAST*  ---------- -------ETR ---------- ---------- ----------
CANAL   ---------- -------NIK ---------- ---------- ----------
CANGA   ---------- -------EPK ---------- ---------- ----------
ASPNI   ---------- -------SPQ ---------- ---------- ----------
RHIO9   ---------- -------KPQ ---------- ---------- ----------
CRYNE   ---------- -------PPQ ---------- ---------- ----------
USTMA   ---------- -------PPT ---------- ---------- ----------
ARATH*  ---------- -------FGK V-RGSQTLNP VNSGTS---- SSSGAASEDS
VITVI   ---------- -------FRK INRGSQSLSS VTSGSS---- SSSGPGSDD-
SOYBN   ---------- -------FKK VSNGVKPSTP TTSGSS---- NSSGNALDDA
SOLLC   ---------- -------FKK INGGSQSLIS ITSGSS---- SSSGTGSEDT
ORYSJ*  ---------- -------CRK MNKPVVSSGN LSSTSSSSVS GSSVSTPDD-
HORVU   ---------- -------CRK MTKSGVVSGN LSSASSSSVS GSSLSTSDDS
SORBI   ---------- -------CRK MNKSGVSSSN LSSTSSSSVS GSSVSASDDS
SELML   ---------- -------FKR IKA------- ---------- ----AAPDET
PHYPA   ---------- -------FKK VNKSSPGPTP ---------- GISGSPSEDV
              1210       1220       1230       1240       1250

HUMAN*  E--MKLVQMK WH-------- -EACLSISAA ILEILNAWEN --GVL-AFES
CHICK   E--MKLVQIN WH-------- -EVCLSISAA ILEILNAWEN --SVL-TFES
XENTR   E--MKLAQTK WH-------- -EVCLSIPAA ILEIHNAWEN --GVL-SFEA
DANRE   E--MKLVQMK WH-------- -EICLSTPAA ILEVLNAWEN --GVL-SVEA
CIOIN   E--FKLSMTK WN-------- -DICMNLPQS MIELLTARQK --GII-SITE
ANOGA   YVGLKASSLR WE-------- -DICYTIPAL VHQVLMAWES --ESI-APLE
TETNG   E--MKLVQVK WH-------- -EICHSIPAA ILEVLNAWEN --GVL-SVEA
DROME   Q--LKPNNLS WQ-------- -ETCLNLPGV LHHVLIAWEQ --ETL-SSAD
BRUMA   ---LKRAQPF WDPRTVNYA- -ELVDFVPVI GEILLDDFRM --TKFENYEH
CAEEL   Q--MKEGIPF WKTDDPNLNI GSILAAIPRL GQLLVDEHEE KRNRI-DRKS
YEAST*  ---------- ---------- -QLANLIPFI VKQVLLSVEI --GVLTDISS
CANAL   ---------- ---------- -QIYKIIPII YRQGIIDTNN --KKI-NWEI
CANGA   ---------- ---------- -QLAILVPYI FKQVTLAMEI --GAVGDLQN
ASPNI   ---------- ---------- -EFYMLVSTL FSQSLGACEA --GKL-EFDT
RHIO9   ---------- ---------- -HLLMIAPTI VHRSMLLYSQ --NQI-QQET
CRYNE   ---------- ---------- -TLCCLVPTL IQQAIAAVAC --NQI-DLDT
USTMA   ---------- ---------- -TLLRLSPLL FSQSISACQH --GII-DLET
ARATH*  N--IRPEFPA W--------- -DILKAVPYV VDAALTACTH --GRL-SPRQ
VITVI   P--LRPKLPA W--------- -DILEVVPFV VDAALTACAH --GRL-SPRE
SOYBN   L--MKLKVPA W--------- -DILEAIPFV LDSALTSCAY --GKT-STRE
SOLLC   S--MRPKLPA W--------- -DILEAVPFV VDAALTACAH --GRL-SPRE
ORYSJ*  Y--QRPTVPA W--------- -EFLEAVPFV LEAVLTACAH --GRF-SSRD
HORVU   Y--QRPTLPA W--------- -EILEAVPYV LEAVLTACSH --GRI-SSRD
SORBI   C--QRPAVPA W--------- -EFLEAVPFV LEAVLTACAH --GRL-SSRD
SELML   S--ARPMLPA W--------- -EIMTSVPFV LDAVLTACGH --GRL-SSKD
PHYPA   S--GRPILCA W--------- -DIIAAVPNV LEYSLTACAH --GSL-SPRD
              1260       1270       1280       1290       1300

HUMAN*  IQKITDNIKG KV----CSLA VCAVAWLVAH VRMLGLDERE KSLQMIRQLA
CHICK   IQKITDNIKG KV----CSMA VCAVAWLVAH VRMLGLDERE KSLQMIRQLA
XENTR   IQKITDNIKG KV----CSLA VCAVAWLVAH VRMLGLDERD KSLQMIRQLG
DANRE   VQKITDNIKG KV----CSMA ICAVAWLVAH VRMLGLDERE KPQTMIRQLM
CIOIN   VVRACTLIKD GC-PLSVTLS VMAAVARCAR LVVSRNDHI- -------QVL
ANOGA   IKKMLDGLKA YF----STFA VCAASWLCAY MQMVRQDEQL KPMNMVQQLC
TETNG   VQKITDNIKG KV----CSMA ICAVAWLVSH VRMLGRDERE KPQTMIRQLV
DROME   VKSILDNIKR RL----FSFS VCATSFLCAY MYSVKETELL KPLNMIQQFL
BRUMA   LHENIKNVLH AFRDDANFMI VCLVQWMCSQ PVTNPRQSLV KSFIRALEYP
CAEEL   AEENMTNILH AIES-MPSLF LCLLEWLDCE PESGARTSLA FTINLSLERY
YEAST*  LIGGFEYFLQ PL---LLVGL IKTFYWLEQF LSCVKNDTIS EDILQGIF--
CANAL   LNNGLEYLSQ PF---LTPII PIIIKSLLRD FTIDNDLKF- ----------
CANGA   LIGGFEYFLQ PF---MLVGL IKVMYWLEQY LSCLKSDETD EKLIQKVL--
ASPNI   LKGGFEYLLE PF---LLPSL VVALTWLGNH IWESEADPTI PL--------
RHIO9   FTSMMSYFQT AY---LDFTQ ASLFPLLCKE LVHDESTIAL SCLRLFIMSS
CRYNE   LHSGLSYFSQ PL---LSWCL GGVIEWLCDE ILRQGALSAL HL--------
USTMA   LRGGLSYFLQ DL---LSFAL PGALVWLLSE ISRTPLQPIL DFLSESGLQA
ARATH*  LATGLKDLAD FL----PASL ATIVSYFSAE VSRG------ ----------
VITVI   LATGLKDLAD FL----PASL ATIISYFSAE VTRG------ ----------
SOYBN   LATGLKDLAD FL----PASL VTIASYFSAE VTRG------ ----------
SOLLC   LCTGLKDLAD FL----PASL ATIVSYFSAE VTRG------ ----------
ORYSJ*  LTTSLRDLVD FL----PASI AAIVSYFLAE ITRG------ ----------
HORVU   MTTSLRDLVD FL----PASL AAIVSYFSAE ITRG------ ----------
SORBI   LTTSLRDLVD FL----PASL AAIVSYFSAE ITRG------ ----------
SELML   LTTGLRDLVD FL----PASI ATIVSYFTAE VTRG------ ----------
PHYPA   LTTGLRELVD YL----PGAI ATIVSYCSAE TTRG------ ----------
              1310       1320       1330       1340       1350
```

MED5

HUMAN*	GPLF-------SENTLQFYNERVVIMNSIL----ERMCADVLQQ------
CHICK	TPLY-------GDNTLQFYNERVVIMSSIL----EHMCADVLQQ------
XENTR	SPMF-------GDNTLQFYNERVIIMSSIL----DNMCAEVLQQ------
DANRE	TPMS-------GENTLQFYHERVVIMSSIL----EHMCADVFQQ------
CIOIN	SALTEKSPTQVQSLSNMMYNERFVFFQSVS----SSIMSDLL--------
ANOGA	SALA--GPLADEWQQQDYCKERFGLMGQIV----RRMQQEFLRT------
TETNG	TPLY-------GEHTLQFYNERVIIMSSIM----ERMCADIFQQ------
DROME	APLT-----SEELSSQENAKERLALSYQII----RKMQHDVHPA------
BRUMA	HQLN------------HTQQERMVLTSATC----RRTLDDLSICDRLRDP
CAEEL	LAVS----------THGINYMKWIFIKSTV----QQMIDELVDKSPAFPE
YEAST*	------NLLNTLFNPVTLNEDSKAFHTAVL----RLNAIPL---------
CANAL	------KIIRELIKDNNNNSNNIII--KMV----INICGNEILTFNPPAD
CANGA	------SLLNTIICPSTLNEDSKAFHFAVL----RLNAIPL---------
ASPNI	------RTLQSLVSPSSISGEAREIHLTVLNITSRSLEEQLKDV-RARHP
RHIO9	SSDRQAYHYGYLALGSLESLLDYKRQEAAF----VKKEGNETIINDISEC
CRYNE	------VVLQALAMGHACPEQLLKVNDRAL----RKLFNPLSGLDEVIGT
USTMA	SVVDVPSSDGTLANGLPRNATS----KSVC----LEVLALLVDTDACPSS
ARATH*	-----------VWKPVFMNGVDWPSPATNL----STVEEYITKI------
VITVI	-----------VWNPVFMNGTDWPSPAANL----SNVEEQIRKI------
SOYBN	-----------IWKPSFMNGTDWPSPAANL----AHIEQQIKKI------
SOLLC	-----------VWKPVFMNGTDWPSPAANL----SNVEEQIKKI------
ORYSJ*	-----------IWKMVPMNGTEWPSPGASL----HSIEAEIKEI------
HORVU	-----------IWKAVPMNGTEWPSPGAAL----QSIEDEVKEI------
SORBI	-----------IWKPVPMNGIEWPSPGASL----HSIEAEVKEI------
SELML	-----------LWKYASMNGNDWPSPAANL----LSVEAEIKEI------
PHYPA	-----------LWKYASMNGQDWPSPAANL----LTIQGEVKDI------
	1360 1370 1380 1390 1400

HUMAN*	-------TATQIKFPSTGVDTMPYWN------------------------
CHICK	-------TATQIKFPSTGMDTIPYWN------------------------
XENTR	-------TATQIKFSAPGSEPVPYIH------------------------
DANRE	-------TGLSLRPAVEGQEPIPYRN------------------------
CIOIN	---------PEFEPKTLNLDKTLHCT------------------------
ANOGA	---------PQVNPKLRALFPSQQQQ------------------------
TETNG	-------TGATLRPPMDGQESIPYRN------------------------
DROME	---------------------TSTKS------------------------
BRUMA	RFTWIINCAKRKLPTVPFTLP-----------------------------
CAEEL	VTCTAFSTARRFCPFVG---------------------------------
YEAST*	-----LKVLRKFRV--QSQSNYGIYS------------------------
CANAL	IRESIIKTMV--
CANGA	-----LGILYQFRSNKHAESNYGIYS------------------------
ASPNI	TRTDIKPILDALDPCLSFQRTASCHKSELEPWTTHNPGGLLGSIRATFQS
RHIO9	IDFILKKKKNKMMEELDNDGVFHTET------------------------
CRYNE	SNFDMFA----FKAKLESLGITTSHH------------------------
USTMA	VRQLIAKAFDAFVSTIEADVTAAPQL------------------------
ARATH*	---------------LATTGVDIPSL------------------------
VITVI	---------------LAATGVDVPSL------------------------
SOYBN	---------------LAATGVNVPSL------------------------
SOLLC	---------------LAATGVDVPSL------------------------
ORYSJ*	---------------LASAGIQIPSC------------------------
HORVU	---------------LASAGVQIHSC------------------------
SORBI	---------------LASAGVQINSC------------------------
SELML	---------------LAATGVQVPNLVTG---------------------
PHYPA	---------------LAAVGVHIPNP-TG---------------------
	1410 1420 1430 1440 1450

HUMAN*	-LLPPKRPIKEVLTDIFAKVLEK---------------------------
CHICK	-LLPPKKPIKEVLTSVFTKVLEK---------------------------
XENTR	-RLPPKKPIKEVLRGAFSSTLEK---------------------------
DANRE	-LLPARLPIRQALQSQFRKVLEK---------------------------
CIOIN	-----------LLKQTFIEANQV---------------------------
ANOGA	-HVVSHLPLEEQFEEAWKAIAER---------------------------
TETNG	-LLPAKDPIHTSLSKQFQAVLSK---------------------------
DROME	-RLISHSPLVEQFREVWRTVVDA---------------------------
BRUMA	-ATNAQSSDAEMLKQAFTYARQQ---------------------------
CAEEL	---RNENPDQLKLKHAWYYMRQQ---------------------------
YEAST*	-SDAQGDPNLEPLIAKLVAVLNVSPVYDVDPRIINSENDYSRKQLGYGKF
CANAL	-YLDKSDPIARDI-------------------------------------
CANGA	-SDNEGNPTLELMISRLISSLSISPVYDIDSTILVTDNNFVQKPPKFQSF
ASPNI	LILWSTSPDVSMAPPSYTHRQVITA-------------------------
RHIO9	-VTTGVTPGTLFEKTQMMFRYIV--------------------KSGRSMF
CRYNE	-YGPLNQPSVKSSIQSISSLPFA---------------------------
USTMA	-AVGCETFNLASLKARM---------------------------------
ARATH*	-APGGSSPATLPLPLAAFVSLTITYKIDKASERFLNLAGPALE-------
VITVI	-AAGGNSPATLPLPLAAFASLTITYKIDRASQRFLNLAGPALE-------
SOYBN	-DIDGDSPATLPFPLAAFVSLTLTYKLDKATEPFLALIAPAMN-------
SOLLC	-VAGGSSPAILPLPLAAFVSLTITYKLDKASQRFLNLAGPALE-------
ORYSJ*	-YPRG-VPPMLPLPMAALVSLTITFKLDKSSEYIHAISGQALE-------
HORVU	-YPRG-VPPMLPLPMAALVGLTITFKLDRSLDYIHGIIGQALE-------
SORBI	-YPRG-VPPMLPLPMAALVSLTITFKLDRSLEYIQGVTGQALE-------
SELML	-SLGGNAPVSLPLPLAAFLSLTITFRQDKSSELVLGVAGPALE-------
PHYPA	-SGGGNAPVSLPLPLAALIGLTITFKLDRAGDTLLSVAGPGLE-------
	1460 1470 1480 1490 1500

MED5

```
HUMAN*   ----------------------GWVDSRSIHIFDTL-----------LHM
CHICK    ----------------------GWVDSRSIHIFDSL-----------LHM
XENTR    ----------------------GWVDSRTLHVFDTL-----------LHM
DANRE    ----------------------GWVDSHAFHLFENL-----------LHM
CIOIN    ----------------------GWINHAIITKFLDC-----------YNM
ANOGA    ----------------------GWLPIETTFLLDTL-----------LQS
TETNG    ----------------------GWVDSQALHLFDSL-----------LNM
DROME    ----------------------GHLPVRAAQSLESL-----------LLA
BRUMA    ----------------------AWGSPNVLQLVDRC-----------NKA
CAEEL    ----------------------AWASPHALRLLEHA-----------NTA
YEAST*   LILNENPINKIMTNQINSFWSLHSSTYYNLDYLFEL-----------IEL
CANAL    ----------------------NFKEFIKHQLLGDIHIQIEENFLNKYIL
CANGA    FVTNEISMNKMLTNQMNSFWSLHSSTYYNLDFLKTL-----------IDT
ASPNI    -----------------------------------------------IRT
RHIO9    MSDVDANTGSLWDSNQNMSDQQVVSHYLDMVLFETA-----------LEI
CRYNE    ------------------------TAFWANPLLDSL-----------WAS
USTMA    -----------------------------------------------EGA
ARATH*   -----------------------------------------------CLA
VITVI    -----------------------------------------------ALA
SOYBN    -----------------------------------------------AVA
SOLLC    -----------------------------------------------SLA
ORYSJ*   -----------------------------------------------NCA
HORVU    -----------------------------------------------NCA
SORBI    -----------------------------------------------NCA
SELML    -----------------------------------------------STA
PHYPA    -----------------------------------------------SCS
                  1510      1520      1530      1540      1550
```

```
HUMAN*   GGVYWFCNNLIKELLKETRKEHTLR--AVELLYSIFCL------------
CHICK    GGVYWFCNNLVKELLKETRKEHTLR--AVELLYAIFCL------------
XENTR    GGVYWFCNNLVKELLRETRLEFALR--AVELLYAIFCL------------
DANRE    GGVFWFTNNLVKELLKETRKEWASR--VVELLYSIFCL------------
CIOIN    LGVCGFMRQTLNQILKETHLCGITN--AVDIVFAVMRF------------
ANOGA    CGPAWLVEKLIGKLFVCKYVRDLNK--TMDIIFAIMHL------------
TETNG    GGVFWFTNNLIKELLKETRQEWANR--VVELLYSIFCL------------
DROME    GGAAWLSTQLVEQLLACKYTRDMSR--TMDVVFAVMHL------------
BRUMA    GVVENWCMVWLNAMLKLSTNDEMLC--ASELCLAAGIM------------
CAEEL    REYDVWSHIYISKTIKSGCGDIMTS--SVDMIFSFLML------------
YEAST*   VTPKSFLFDVLKTLEYKLAT----------------------------YG
CANAL    NNRLNFISNLIEEIYNFQKF------------------------------
CANGA    LTPKQFLLDVLRTLEYKVET----------------------------LG
ASPNI    LGASRVLAALLDELKFQTESNPGTAGIALDIAASIVSAPLSESFAFDQFQ
RHIO9    GGGHWFVSMIVDEVLEAGKSGGAVR--AAELGSCLISTPLLY--------
CRYNE    AGTSNWMTVVLKNIFSQLDT----------------------------FP
USTMA    GVTARLLSETGASWLQSVAAGHQDS--RSKLFSMLTTLQPSVHAMDRLIG
ARATH*   AGCPWPCMPIVASLWTQKAK------------------------------
VITVI    ADCPWPCMPIVASLWTQKAK------------------------------
SOYBN    SGCPWPSLPIVTSLWIQKVK------------------------------
SOLLC    AGCPWPCMPIVASLWTQKAK------------------------------
ORYSJ*   GGSSWPSMPIIAALWTQKVR------------------------------
HORVU    GGSSWPSMPIIGALWTQKVR------------------------------
SORBI    GGSSWPSMPIIGALWTQKVR------------------------------
SELML    GGSPWPSMPVVAALWAQKVK------------------------------
PHYPA    GAGPWFSMQVVAALWAQKVR------------------------------
                  1560      1570      1580      1590      1600
```

```
HUMAN*   -----------DMQQVTLVLL---------------------GHILPGLL
CHICK    -----------DMQQLTLTLL---------------------GHILPNLL
XENTR    -----------DMQQLTLTLL---------------------GHVLPSLL
DANRE    -----------DTQQITLTLL---------------------GHILPRLL
CIOIN    -----------DIVSTTMYLL---------------------KYGLPTFF
ANOGA    -----------DIERCTIALL---------------------SQLVPMML
TETNG    -----------DTQQITLTLL---------------------GTIVPNLL
DROME    -----------DIEKTTEALL---------------------QYVVAPLI
BRUMA    -----------SPVPCMLSFA---------------------RSITEYIF
CAEEL    -----------DDLNSIIRIH---------------------ESLMAFWL
YEAST*   VPGSENKRGSLDSEHVFDYFF---------------------YFLVLYDV
CANAL    ------NHEDSKIYINLMIFI---------------------MLLDSIET
CANGA    VKDVRNKSSSNESDQVIDYLF---------------------YFLVLYDI
ASPNI    THHDHSTSKESEQAPHYPIFNLRDALNTQHELVPKLSEKDLLRAEIIVRL
RHIO9    -----SKNRHNNSTHLLRSLL---------------------QDVLPTSL
CRYNE    VSPVGSACQRSEVYANFVPFLLMVDIKGNEPLLSGLAAWLRNSTEDLIHY
USTMA    ALKSGQVGRNGRDQKGLAAWL---------------------CFIAPASE
ARATH*   ------------RWFDFLVFS---------------------ASRTVFLH
VITVI    ------------RWSDFLVFS---------------------ASRTVFLH
SOYBN    ------------RWSNYFVLS---------------------ASSTVFHH
SOLLC    ------------RWSDFLVFS---------------------ASRTVFLN
ORYSJ*   ------------RWHDFIILS---------------------CLRSPFGR
HORVU    ------------RWHDFIVLS---------------------CIRSPFGR
SORBI    ------------RWHDFIVLS---------------------CMRSPFGT
SELML    ------------RWHSFIVFG---------------------ASRTVFKQ
PHYPA    ------------RWHDYIVFI---------------------SSCSVFKH
                  1610      1620      1630      1640      1650
```

MED5

```
HUMAN*   TDSS----KWHSLMDPPGTALAKLAVWCALSSYS-------SH-KGQAST
CHICK    TDSS----KWHTLMDPPGKALAKLSVWCALSSYS-------SHNKGQASA
XENTR    TDSA----KWHMLMDPPGRALAKLSVWCAMTSYS-------TQNKGQPSP
DANRE    TDPA----HWHSLADPPGRALAKLSVWCALSSYS-------SHHKGQASA
CIOIN    KTNS------RPLAAPIGSAIAKLTVASLDIALN-------EITLQQKS-
ANOGA    LNKA----QTPDIVDPQSRVLAKLCVYCIIVTME-------TSLTATKK-
TETNG    TDSA----HWHSLTDPPGKALAKLCVWCALSSYS-------SHPKGSFSA
DROME    LRRQ-----GEDINEPQSLVLARLCVYCIISCLESRKGNGTSALTAMKK-
BRUMA    EQNV-----DFGCVEPKALVSARFLVHCLQLAMH-------AYWRQQKE-
CAEEL    SEDAGQCQADGRFDPLSIRVVIRLMTQVLLIAEW-------TLDRLLNE-
YEAST*   KTAE------------EASQLIEYMENDAKKSKG-------DVDIKGEDL
CANAL    KSDKDYWKKNFNLPYPMNLETNDLVVVVAVDSRF-------ELSMDNHYS
CANGA    ENSE------------DAKAMAQFMEDTV-------------------DI
ASPNI    YRRV------NTLLAPPAQVAPNLDMNMGMN-------------------
RHIO9    EKCA-----QQNMSFFQGQTLGVFTSDCLVLM------------------
CRYNE    TRGS--------------RVLAGIFTQCILLADL-------IWGRVMAK-
USTMA    PTSALRFVQRLELGDIEESAIEGVV-------------------------
ARATH*   NQDA----------------VIQLLRNCFSATLG-------LN---AAPM
VITVI    NSDA----------------VVQLLKSCFTATLG-------LK---TTPI
SOYBN    NKDA----------------IAQLLKSCFTSTLG-------LG---YGSI
SOLLC    NHHA----------------VIQLLKSCFNATLG-------LN---SSSI
ORYSJ*   DKDA----------------VAQLIQSCFSSFLR-------SSCS-GSDF
HORVU    DKDA----------------VAQLIQSCFSSFLR-------SSPSNGSDI
SORBI    DKDA----------------VAQLIQSCFSSFLQ-------SSPS-GSDI
SELML    DKNA----------------VKQLLRSCFAVTTG-------TTGTLMSKL
PHYPA    NKPA---------------MLQLLKSCFAVTLS-------SSPSLGSKL
                  1660      1670      1680      1690      1700
```

```
HUMAN*   ----------------------------------------------RQKK
CHICK    ----------------------------------------------RQKK
XENTR    ----------------------------------------------RQRK
DANRE    ----------------------------------------------RQRK
CIOIN    --------------------------------------------------
ANOGA    ----------------------------------------------RTRG
TETNG    ----------------------------------------------RQRK
DROME    ----------------------------------------------RSRS
BRUMA    ----------------------------------------------RQTR
CAEEL    ----------------------------------------------DPAF
YEAST*   HEKNDSAEVRQ----------------------------------ETQPK
CANAL    SIFNDDSR--------------------------------------SSSS
CANGA    SINGDSGIVKQ----------------------------------ETQPK
ASPNI    ----------------------------------------------VNVN
RHIO9    --------------------------------------------------
CRYNE    ----------------------------------------------RVIK
USTMA    ----------------------------------------------RIVG
ARATH*   SNDGGVGALLGHGFGSHFYGGISPVAPGILYLRMYRALRDTVSVSEEILS
VITVI    SSNGGVGALLGHGFGSHFCGGISPVAPGILYLRAYRSIRDVVFMAEEIVS
SOYBN    YNNGGVSALLGDGSVSRISNGISPVAPGILYIRVYRSIGDITLLIKEIVP
SOLLC    SSNGGIGALLGHGFGSHFYGGISPVAPGILYLRVYRSIRDIMFLREEIVS
ORYSJ*   TANRGVGALMGDAI-T-GQGLQLPMAPGFIYLRTCRTFHDTYFVSEVILK
HORVU    TASRGVGALMGESI-TGQQGLHFPMAPGFIYLRTCRTFHDTYFVSEMILR
SORBI    IANRGVGALLGDSI-T-NQGLRLPMAPGFIYLRTCRTFHDTYFVSEVILK
SELML    QVHGGVGALLGHG---GMQGGQYPLAPGILYLGIYPALHEIMFVTDEILF
PHYPA    QMNGGVGALLG----SWSAYGPEPVAPGIVYLRSYVFFHDIMFLSDEILI
                  1710      1720      1730      1740      1750
```

```
HUMAN*   RHREDIEDYISLFPLDDVQPSKLMRLLSSNEDDANILSSPTDRSMSSSLS
CHICK    RHREDIEDYISLFPLDDTRPSKLMRLLSSNEEDANILSSP-NRSMSSSLS
XENTR    RHREDIEDYSSLFPLDDTQQSKLMRLLSSNEEEHSVQANPVDRPMNSSLS
DANRE    RHREDIEDYTSLFPLDDTQPSKLMRLLSSNEDENSMLSSPGDRSISSSLS
CIOIN    -----------LLPKSRKQNPMLQNIVFDKQQKVRRLFSQTEHDLPPPGS
ANOGA    TASSELEDLDALCTSAKLRKIELDGMGVGCTEAATGGASGTEFSLDSALE
TETNG    RHREDIEDYNSLFPLDDTQPSKLMRLLSSNEDEPAALSSPADRSMSSSLS
DROME    HDDEELA-----ANAAKVRKVIGDGSDNSSDFTDTTTGAGLAALLGSTST
BRUMA    IVRGDCAERKRSDDDDDAETDQ----------------------------
CAEEL    VERLNLKEVKAPEPEDPDKREKWI--------------------------
YEAST*   AEATQDDDFDMLFGENDTSTQAYEEEEEENEDNDGNNRTNNVPMIKAEETP
CANAL    SSSSNSKDVDNFLKNGDEDEDEFMKDDNNTNNNNNNNNNNGERITTSQQL
CANGA    SEYNPDDDIDMLFGENDTSMQA--NEEDTLDNKELKSDRNCALGK-----
ASPNI    MNMNNIIGDMHLDSSGDGASNQNMNMNMETDTRTQAQEQGHNNEQANLN-
RHIO9    ---------------QDKKSESVTKLGTS---------------------
CRYNE    ELVVELHSVTTLGVRSGGQSEETTQLHSRKMKHRDGRRLTSEQRSM----
USTMA    LVVGLIMAAEAQRATTDAVADDLLALARAADTNARGTKATVADTTAAARP
ARATH*   LLIHSVEDIAQNRLSKE-KLEKLKTVKNG--SRYGQSSLATAMTQVKLAA
VITVI    LLMHFVREIASSQLSGE-RSEKLKKAKNE--MKYGQISLGAALARVKLIA
SOYBN    ILMLSVTDIASSDLMPKGVVRKPKKTKFG--VKYGQVSLARSMARVKHAA
SOLLC    LLMQSISDIARSELPRQ-RLNKLKILKNG--KKFGNVSLAATMTRVKLAA
ORYSJ*   QVIEWADKLANGFSSSG-----------PPQLKSGRTPLSSAACMAHQVA
HORVU    QVINCSHKLANGWSSNG-----------PPHLKSGRPPLSGAASMASQVA
SORBI    QVIEWSHKLANGWSFNG-----------PPQLKSGRTPLSCAASMAHQVA
SELML    LVVKAARDLTAAKGTTS------------------KISCASAMSRVFQAS
PHYPA    LVAEAARELG-----TQGDFNKESLVGLGSRLRCVQASLPNSMARAVQAS
                  1760      1770      1780      1790      1800
```

MED5

```
HUMAN*   AS-------QLHTVNMRDPLNRVLANLFLLI------------SSILGSR
CHICK    AS-------QLHAVSMRDPLNRVLANLFLLI------------SSILGSK
XENTR    AS-------QIHNISSKEPLNRVLANLFLLI------------SSVLGAK
DANRE    AS-------QLHTVNMRDPLNRVLANLFLLI------------SSQLGSK
CIOIN    TPG----------LPAEDPLIQVLSDFMDLL------------WNSMESN
ANOGA    PTASAWTTEAASIATLKEPLQSCLQTLFRTF------------AQHIATD
TETNG    AS-------QLHTVNMREPLNRVLANLFLLI------------SSIVGSK
DROME    SE------LRTTPLTLREPLQTSVQHIFAVF------------LQFVSGD
BRUMA    ----------------GNPVERSVLVIFEKF-------------VRETRAG
CAEEL    -------------FLLRSMLERTINRFFKIV----------------RKG
YEAST*   SKTN----------------------------------------------
CANAL    EK-----------LNEKIHRHECLLNEFRKI-----------RTNTTETN
CANGA    --------------------------------------------------
ASPNI    -------------QIMDNAAAAVGMDMDMNMGGGGDGMGVGVGVGELGLG
RHIO9    -------------------FFETLVIEGHDFPRQQQEQEDGTQFTEWSHK
CRYNE    --------------------------------------------------
USTMA    AEAD--------------------------LFDDEPCSPQPPHAAANATS
ARATH*   SLSASLVWLTGGLGVVHVLIKETIPSWFLSTDKSDREQGPSDLV----AE
VITVI    SLAASLVWLSGGLGLVQSLIKETLPSWFISVHRSEQEEGSGGMV----AM
SOYBN    LLGASLVWISGGQKLIQYLMRETLPSWFLSATMFEQDGGESGVMV---AK
SOLLC    LLGASLLWLSGGSGLVQSLIKETLPSWFLSVNSSNQEGDKGDLV----PM
ORYSJ*   MLGGGLLCVAGGPLLVQVLYEETLPTLLLSA-REESMKDPGPVS----ST
HORVU    MLGAGLLCVAGGPLLVQVLYEETLPTLLLSA-QEQMLEDPGPVA----ST
SORBI    LLGGGLLCIAGGPLVVQVLYEETLPTLLLSA-REQSLKGPGPVS----ST
SELML    TLGASLLHISGGSTLVQTLYSESLPAWFLAGGNPEESSSSTSSGSGDGSL
PHYPA    SLGASLLYVSGGAILVAKLFTDSIPTWFLSGKGSKGIHSTGG------LI
                  1810      1820      1830      1840      1850
```

```
HUMAN*   TAGPHTQFVQWFMEECVDCLEQGG--------------------------
CHICK    TAGTHTQFVQWFMEECVDCLEQGS--------------------------
XENTR    TAGAQTQFVQWFMEECVESLELGG--------------------------
DANRE    TAGPHTQFVQSFVEECVDCSEQGS--------------------------
CIOIN    EGGPQADFIFKFIKLSLESSSLL---------------------------
ANOGA    ELSPKVYFVFQFLSLLVESGRER---------------------------
TETNG    MAGPHTQFVQSFIEECVECLEQGS--------------------------
DROME    ELSPKAVFVYQFISLLVECGGER---------------------------
BRUMA    HLKSTITFIYHFVVSLATAPQNAA--------------------------
CAEEL    LLSNVVNTIIQLLKSIAGSADCKA--------------------------
YEAST*   --------------------------------------------------
CANAL    LFAKSIRLL-----------------------------------------
CANGA    --------------------------------------------------
ASPNI    EVDQSIDDVLNAAEM-----------------------------------
RHIO9    VTESAV--------------------------------------------
CRYNE    --------------------------------------------------
USTMA    HTSNAAPTVTSAASLLASGELRTT--------------------------
ARATH*   LRGHALAYFVVLCGALTWGVDSRS-----------SASKRRRQAILGSHL
VITVI    LGGYALAYFTVLCGAFVWGVDSSS-----------SASKRRPK-ILGSHM
SOYBN    LKGYALAFFVFLSAAFAWGIDNSY------------SPKQRAE-VVGLHL
SOLLC    LKGYALAYFAVLCGAFASGVDSLS-----------MASKRRPK-IIGRHV
ORYSJ*   LQGYAMANMLFFCGSLLWGAERTS------PVMKLSFLSRRPR-VVGNHM
HORVU    LQGYAMANMLFFCGSLLWGSEKTS------PVMKLSFLSRRPR-VVGTHM
SORBI    LQGYAMANMLFYSGSLLWGADRTD------PVMKLSFLWRRPR-VVRKHM
SELML    VEGYAVAHFALLSGALVWGISSTS--------TKTSHRTRRRR-VLGSHM
PHYPA    LEGYAIAHFVLLSGALAWGVSGSSAVHLQAENTGIPSQIQRHY-VLGAHM
                  1860      1870      1880      1890      1900
```

```
HUMAN*   ------------------------------------------RGSVLQFM
CHICK    ------------------------------------------HGSILQFM
XENTR    ------------------------------------------KGCILQFM
DANRE    ------------------------------------------RSSILQFM
CIOIN    ------------------------------------------SRTVKLHI
ANOGA    ------------------------------------------IMCVLKLL
TETNG    ------------------------------------------RGSILQFM
DROME    ------------------------------------------VAPVLRLL
BRUMA    ------------------------------------------TRRIIELL
CAEEL    ------------------------------------------KRLLVKRI
YEAST*   ------------------------------------------KISILKRH
CANAL    ------------------------------------------NDKLIEKI
CANGA    -----------------------------------------------NRH
ASPNI    ------------------------------------------NPEFLDLD
RHIO9    ------------------------------------------WRGFIKGL
CRYNE    ------------------------------------------CEDIVKML
USTMA    ------------------------------------------SKQVLDMM
ARATH*   EFIASALDGKISVGCETATWRTYISGLVSLMVSCLPLWVTEIDTEVLKSL
VITVI    EFLASALDGNISLGCDCATWRAYVSGFVSLMVGCTPTWVLEVDVNVLKRL
SOYBN    KFLASTLNRNGAMFSRCTTWKAYVSGLVSLMVGQAPSWVREVDADLLKRL
SOLLC    EFIASVLDGKISLGCDPSTWHAYVSGFVSLMVGCTPTWVYEVDAELLKRL
ORYSJ*   DFMAGVLDGHILLGCDHGTWKAYVSRFVFLVVKFVPSWLRDIKLDTLKKI
HORVU    DFIAGVLDGHILLGCDPGTWKAYVSCFVFLVVKFVPTWLRDIKLDTLKKI
SORBI    DFIAGVLDGHILLGCDPGTWKAYVSQFMFLVVKFVPSWLRDIKLETLKKI
SELML    EFLASALDGKIALGCGRATWKAYLTGFIALLVSSTPNWILDVKLDVLKRL
PHYPA    DFLASGLGGEIFISCEQTLWRSYVVGFLALMVTCTPMWILELKLDTLQKL
                  1910      1920      1930      1940      1950
```

MED5

HUMAN*	PFTTVSELVKVSAMSSPKVVLAIT--------------------------
CHICK	PFTMVSELVKVSTMSSPKIVLAIT--------------------------
XENTR	PFSMVSELVKVSTLSSPKIVLAIT--------------------------
DANRE	PFTMISELVKLQSLAKPKTVLSIT--------------------------
CIOIN	PVPMLSKLRYPLAKDSHVLLFAAS--------------------------
ANOGA	PNGLVQSLLKINATDEMTVGLILRLY------------------------
TETNG	PFTMVSELVKLPALAKPRVVLAIT--------------------------
DROME	PNGLVQQLLKVLVTDDIKVGLISRLY------------------------
BRUMA	PHDLMLNLARL--------------------------------------D
CAEEL	PPDLIFQLAYI-------------EPSSVDYSLMNIYC------------
YEAST*	SFAVLLHERKLLNDLALENGEIT---------------------------
CANAL	TNWTI---
CANGA	TFGFIIHEIK-LSYGTLESDSMS---------------------------
ASPNI	**MEGM**FQ--
RHIO9	MSNPMIEEI**WPNAFM-**----------------------------------
CRYNE	TYDDGLRARWG**NHWVDL**AV-------------------------------
USTMA	ALKLVRFRSHMLGEKNAKSTTISAKW**ATLQSAI**ADHLAGGEAWHGAQQCR
ARATH*	SNGLRKWGKDELAIVLLSLGGLKTMDYAA**DFIIH**LRS-------------
VITVI	SKGLRQWNEEELALALLGIGGVGTMAAAAE**LIIETEI-**------------
SOYBN	SWGLSQMDEHKLALRLLEIGGIGVMGAAAEMIIGFEHRS-----------
SOLLC	SKGLRQWHEQDLALALLSIGGVGTMGSAAELIVEAST-------------
ORYSJ*	ASGLRSWNEHDLALALLERGGPQAISTVV**DTLL-**----------------
HORVU	AVGLRSWHEHNLALSLLERGGPKAISVV**VETLLH-**---------------
SORBI	AAGLRSWHEHDLALSLLERGGPQAISLV**VEAFM-**----------------
SELML	ARGLRLWHEQELAVALLERGGPAAMGPAAELA------------------
PHYPA	ATGLRFWHEHDLAVALLERGGPSAMGAAA**ELILC-**---------------

1960 1970 1980 1990 2000

HUMAN*	-------------DLSLPLGRQVAAKAIAAL------------
CHICK	-------------DLGLPLGRRVAAKAIAAL------------
XENTR	-------------DLTLPLGRRVAAKALTAL------------
DANRE	-------------DLSLPLGRRMAAKAIAAL------------
CIOIN	-------------DMGNEEIRKL**AAHAICV**L------------
ANOGA	-------------DLNTPSGRQFAMSDICLLRNIQMRKESIKL
TETNG	-------------DLTLPLGRRVAAKAISAL------------
DROME	-------------DLRIQAGRLSAVSDLCLWRNMQMARHSIHL
BRUMA	PQTFNLDLYLPLINLCDVTSTKRALQFTCLLRKLGGF------
CAEEL	-------------DPDNKEHTQTKIMFLCALRRSQSFSF----
YEAST*	-------------KTENEKFISYHDKYLCMLKTCVF-------
CANAL	---
CANGA	-------------YEDYKKICEYHSRYLKMLKTCIF-------
ASPNI	---
RHIO9	---
CRYNE	---
USTMA	ERTQNDAPVNGGKDADSGSG**LSAWLSAMS-**-------------
ARATH*	---
VITVI	---
SOYBN	---
SOLLC	---
ORYSJ*	---
HORVU	---
SORBI	---
SELML	---
PHYPA	---

2010 2020 2030 2040

MED6

HUMAN*	------------------------------MAAVDIRDNLLGISWVDSSW
CHICK	------------------------------MAAVDVRDNLLGISWVDSAW
XENTR	------------------------------MAAVDIRDNLLGISWVDSSW
DANRE	------------------------------MATVDLRDNLLGISWVDSGW
CIOIN	------------------------------MSSVVNEKNHLDISWNDPAL
ANOGA	------------------------MNPGRLGLAPLLQENPLWISWHDSNW
TETNG	------------------------------MAAGDFRDNLLGISWVDSSL
DROME	------------------------------MASRQMTNDHLRLSWHDTQM
BRUMA	MMQAGGPSRSVMMGQTQGSYMGVPNVVSQSGSSYQQIVSLLHQSFKNPNW
CAEEL	------------------------MMPRMGPPAAARQDNPLHVSFRNPQW
YEAST*	----------------------------------MNVTPLDELQWKSPEW
SCHPO	-----------------------------MASGAPPSVDLTSIQWRMPEW
CANAL	------------------------------------MESLDEIQWKSPEF
CANGA	-----------------------------------MDTPLDELQWKSPEW
ASPNI	------------------------------MAGTQDPSSLEEILWRSPSH
RHIO9	------------------------------MQRLAATEDLTGVEWRDTNW
CRYNE	----------------------------MAQQVEEIEQDLSHIHWSWPEA
USTMA	-------------------------MSTQPAPPPEAPSNLLHVQFKNPEF
ENCCU	-----------------------------------MEEREESISFVDQRF
ANTLO	------------------------------------MTKMEEICFVDPYW
ARATH*	MDSSLLSAATADTFNGN-------AADQIPPPLQPPGTDMTGISFRDQLW
VITVI	MASTPMAPPNPAAGNPN-------FDGNPPAVPTPPGTDMTGICFRDQLW
SOYBN	MATAP---------GNQ-------MLEGGGGAPQPPGTDMTGICFRDQLW
SOLLC	MTMTPVMAP---MGNAN-------FDGGAPALPPQPGTDMTGICFRDQLW
ORYSJ*	MSGTPLPPP---------------------ALPPPPGTDMTGICFRDQLW
HORVU	MAATPLPPPPP--------------ADGPAALPPPPGTDMTGICFRDQLW
SORBI	MSATPLPPPGP---------------DGAGALPPPPGTDMTGICFRDQLW
SELML	------------------------MAEGLGGAAPPLPTDQTGISFHDQLW
PHYPA	-----------------------------------PGTEMTSISFRDQLW
	10 20 30 40 50

HUMAN*	IP----------------------------ILNSGSVLDYFSERSNPFYD
CHICK	IP----------------------------ILNNGSVLDYFSERSNPFYD
XENTR	IP----------------------------ILNNGTVLDYFSERSNPFYD
DANRE	VP----------------------------ILNPSNVLEYFSERSNPFYD
CIOIN	LN---------------------------PLPNKEQILDYFSGRSNPFYD
ANOGA	IP----------------------------VLNPGNVMDYFSEKSNPFYD
TETNG	VP----------------------------ILNPGNVLDYFSERSNPFYD
DROME	MA----------------------------TLSPQTVMDYFCRKSNPFYD
BRUMA	PP------------------------NF---ITPDNVLDYFCDPGNVFYD
CAEEL	PP------------------------NF---INKDNVLDYFCNQANAFYE
YEAST*	IQ------------------------VFG--LRTENVLDYFA--ESPFFD
SCHPO	VQ------------------------SMGG-LRTENVLEYFS--QSPFYS
CANAL	IQ------------------------ERG--LNTNNVLEYFS--LSPFYD
CANGA	IQ------------------------AFG--LRTDNVLDYFA--ESPFFD
ASPNI	VQ------------------------MMGGYLHSNNVLFYFA--ESPFFD
RHIO9	LE------------------------RVGGFQNQQTVLDYFA--LSPFWD
CRYNE	IA----------------------ANPARSLATPDLAMDYFA--YSPFWD
USTMA	LSYLSHLQSTGQVSLGRDSSCAKFDPAHP--LTEHNVMAYFA--TSPFFD
ENCCU	LG------------------------SKP--LDDTNVLEYFS--GSPFYD
ANTLO	LA------------------------QNV--LSRSNALEYFS--SSPFYE
ARATH*	IN------------------------SYP--LDRNYIFDYFA--LSPFYD
VITVI	LN------------------------SYP--LDRNLVFDYFA--LSPFYD
SOYBN	LN------------------------SFP--LDRNLVFDYFA--LSPFYD
SOLLC	LN------------------------TYP--LDRNLVFDYFA--LSPFYD
ORYSJ*	LN------------------------TYP--LDRNLVFDYFA--LSPFYD
HORVU	LN------------------------TYP--LDRNLVFDYFA--LSPFYD
SORBI	LD------------------------TYP--LDRNLVFDYFA--LSPFYD
SELML	LS------------------------TYP--LDRNLVFDYFV--LSPFYD
PHYPA	LS------------------------AYP--LDRNLVFDYFA--LSPFYD
	60 70 80 90 100

HUMAN*	RTCNNEVVKMQRL-------------------------------------
CHICK	RTCNNEVVKMQRM-------------------------------------
XENTR	RTCNNEVVKMQRL-------------------------------------
DANRE	RTCNNEVVKMQRS-------------------------------------
CIOIN	RTCNNETIKMQRL-------------------------------------
ANOGA	RTCNNEIVRMQRQ-------------------------------------
TETNG	RTCNNEVVKMQRL-------------------------------------
DROME	HMCNNETVRMQRL-------------------------------------
BRUMA	VSSCNQHIRMQNL-------------------------------------
CAEEL	MNSCNQQIRMQNIV------------------------------------
YEAST*	KTSNNQVIKMQRQFSQL-NDPNAAVNMTQNIMTLPDGKNGNLEEEFAYVD
SCHPO	HKSNNEMLKMQSQFNAL---------------------------------
CANAL	RTSNNQVLMMQFQYQQI---------------------------------
CANGA	KTANNHVIKMQRQFSQLPNNGAAAGSMNPETMRDAHSEDMGQEQEFSYVD
ASPNI	ATSNNASLAIQANYNEA---------------------------------
RHIO9	RQCNNQVLAMQTQYNDL---------------------------------
CRYNE	AKSNNNVLRTQRRIENP---------------------------------
USTMA	RRSNNEQIRMQNIANGI---------------------------------
ENCCU	KSCNNEILKMQTQFRGL---------------------------------
ANTLO	KTCNNEVLKMQSKFCEV---------------------------------
ARATH*	TTCNNEILRRRSI-------------------------------------
VITVI	WTCNNEQLRLRSI-------------------------------------
SOYBN	WTCNNEQLRMRSV-------------------------------------
SOLLC	WTCNNEQLRSRSI-------------------------------------
ORYSJ*	LTCNNESLRSRQI-------------------------------------
HORVU	ITCNNESLRSRQI-------------------------------------
SORBI	ITCNNESLRSRQI-------------------------------------
SELML	RSCSNEQLRMRSV-------------------------------------
PHYPA	RTCNNEQLRMEAI-------------------------------------
	110 120 130 140 150

MED6

```
HUMAN*  ---------------TL-EHLNQMVGIEYILLH-------------AQEP
CHICK   ---------------TL-DHLNQMVGVEYILLH-------------AQEP
XENTR   ---------------TL-DHLNQMVGIEYILLH-------------AQEP
DANRE   ---------------TL-DHLTQLVGVEYILLH-------------TQEP
CIOIN   ---------------SL-AHLENMVGVEYILLH-------------EQEP
ANOGA   ---------------SL-ELLNNMTGVEYIPLH-------------VQDP
TETNG   ---------------TL-EHLNQMVGVEYILLH-------------AQEP
DROME   ---------------GP-EHLHNMIGLEYILLH-------------VAEP
BRUMA   ------------NRPLH-ECLQSMQGIQYTVVG-------------TFPP
CAEEL   ------------NRTVE-ECLRTMPGIQYVLWY-------------SQPP
YEAST*  PARRQILFKYPMYMQLE-EELMKLDGTEYVLSS-------------VREP
SCHPO   -----------DLGDLN-SQLKRLTGIQFVIIH-------------ERPP
CANAL   ----QIPPGVSFHQYFQ-SRLSEMTGIEFVIAY-------------TKEP
CANGA   PIRRAILEKYVVHAFLE-RELMKVRGIEYVLAN-------------VREP
ASPNI   -----FRHFVETREAFE-ARLKTMQGLEFMVAYDPLQAAAGANAQFVHEP
RHIO9   ---------RQPYEATI-QALRKMTGVEFAIVH-------------EQPP
CRYNE   ---------AYGHAEEKVELNAFMSGFEYVVAH-------------SQPP
USTMA   ----QNLSGGMGAKQEE-QELKRFTGLEFVLVH-------------SREP
ENCCU   --------------DQK-SKLFSMVGIFYEVESS------------NHEK
ANTLO   ---------------TQ-DQLQNMVGIEYAL---------------GKPT
ARATH*  ------------HPLDL-SHLSKMTGLEYMLTD-------------ATEP
VITVI   ------------HPLDT-SHLSKMTGTEYMLNE-------------VMEP
SOYBN   ------------HPLDI-SQLSKMTGTEYMLSE-------------VMEP
SOLLC   ------------HPLDT-SHLSKMTGSEYMLSE-------------VMEP
ORYSJ*  ------------HPLDM-SHLTKMTGMEYVLSD-------------VMEP
HORVU   ------------HPLDM-SQLTKMTGLEYVLSE-------------VMEP
SORBI   ------------HPLDM-SHLTKMTGMEYLLSE-------------VMEP
SELML   ------------HPLDM-TQLSKMTGVEYVLLE-------------AQEP
PHYPA   ------------HPLDM-THLSKKTGMEYILHE-------------AQEP
               160       170       180       190       200
```

```
HUMAN*  -ILFIIRKQQRQSPAQ--------VIPLADYYIIAGVIYQAPDLGSVINS
CHICK   -ILFIIRKQQRQSPTQ--------VIPLADYYIIAGVIYQAPDLGSVINS
XENTR   -ILFIIRKQQRQSPTQ--------VIPLADYYIIAGVIYQAPDLGSVINS
DANRE   -ILYIIRKQQRQSPTQ--------VIPLADYYIIAGVVYQAPDLGSVISS
CIOIN   -ILYVIRKQRRHSPNQ--------VTIIADYYVIAGTVYQAPDLASVCNS
ANOGA   -ILYVIRKQHRHSPTE--------ATPMADYYIIAGTVYQAPDLASVFNS
TETNG   -ILYIIRKQQRQSPAQ--------VIPLSDYYIIAGVVYQAPDLGTVISS
DROME   -ILYVIRKQHRHNPSE--------ATPIADYYIIGGTVYKAPDLANVINS
BRUMA   --LYVIMKQRRNSPTN--------VTPLAYYYIVNGTVYQCPDIYTYIQS
CAEEL   --LFIICKQRRNNVTN--------VSPIAYYYVINGSVHQAPDMYSLVQS
YEAST*  -DFWVIRKQRRTNNSGVGSAKGPEIIPLQDYYIIGANIYQSPTIFKIVQS
SCHPO   -FLWVIQKQNRLNENE--------VKPLTVYFVCNENIYMAPNAYTLLAT
CANAL   -DFWIIRKQKRQDPQN--------TVTLQDYYIIGANVYQAPRIYDVLSS
CANGA   -DFWVIKKQRRTSPTA--------IEPLQTYYIVGANVYQSPTVFKIVQS
ASPNI   SNVWVIRKQTRRKRSGFED----EVVVLATFFVVGDCIYMAPSAASVIGN
RHIO9   --VWVIQKRYRRGPAE--D----DVNPIATYYITGANVYQSPTIYSVIAN
CRYNE   -DLFVIHRREVESSGK-------RDRVTGAWFILHEKIYQCPTLFDVMST
USTMA   -TCFVVQKRWRTSPTE--------TTPLAAYYVINDSIYQAPDLYSILAT
ENCCU   -TLFVIRKAYNHGDT---------AETLGMYYIIHGHVYAAPTNYSIYRC
ANTLO   -PVIAIEKLYRHSSKN--------VDVLAVFYIVNGYIYQAPQVQKIYST
ARATH*  -NLFVFRKQKRDGPEK--------VTPMLTYYILDGSIYQAPQLCSVFAA
VITVI   -HLFVIRKQKRDGPEK--------VTPMLTYYILDGSIYQAPQLCNVFTA
SOYBN   -HLFVIRKQKRDSPDK--------VTPMLAYYVLDGSIYQAPQLCNVFAA
SOLLC   -NLFVIRKQKRDGPEK--------VTPMLTYYILDGSIYQAPQLCNVFAG
ORYSJ*  -HLFVIRKQRRESPEK--------SNAMLAYYILDGSIYQAPQLCSVFAS
HORVU   -HLFVMRKQKRSNSEK--------ADPLLAYYILDGSIYQAPLLGSVFAS
SORBI   -HLFVIRKQKRESPEK--------SNPMLAYYILDGSIYQAPQLCNVFAS
SELML   -NLFVLRKQKRESPDK--------VSHLSAYYILDGHIYQAPLLYSVVCS
PHYPA   -HLFVIRKQKRDGPEK--------VSAQAAYYVLDGSIYQAPHLYNVIGS
               210       220       230       240       250
```

```
HUMAN*  RVLTAVHGIQSAFD-EAMSYCRYHPSKGYWWHFKD-------HEEQDKVR
CHICK   RVLTAVHGIQSAFE-EAMSYCRYHPSKGYWWHFKD-------QEEREKAK
XENTR   RVLTAVHGIQSAFD-EAMSYCRYHPSKGYWWHFKD-------QEERDKTK
DANRE   RALSAVHGIQSAFD-EAMSFCRYHPSKGYWWHFKD-------QEEKERVK
CIOIN   RLMTTFHHLDSAFS-QALSFSRYHPAKGYTWQFNN-------QDEQKSS-
ANOGA   RILSTVHHLQTAFD-EASSYSRYHPSKGYSWDFSSNKAIA--EKTKTQTK
TETNG   RVLSAVHGIQSAFD-EAMSYCRYHPSKGYWWHFKD-------QEEREKNK
DROME   RILNTVVNLQSAFE-EASSYARYHPNKGYTWDFSSNKVFSD-RSKSDKKD
BRUMA   KLISIVDPLRQALE-QARNFNRFNIAKGYYWEFKDSXGETVKKATEEE--
CAEEL   RLLGALEPLRNAFG-EVTNYSRYNTAKGYYWEFKNKP--NVKKREEEKKE
YEAST*  RLMSTSYHLNSTLE-SLYDLIEFQPSQGVHYKVPTD------TSTTATAA
SCHPO   RMLNATYCFQKALT-KIEKFPQYNPQEGYTYPKLSNDNLEVDHSNTNEPA
CANAL   RLLASVLSIKNSTD-LLNDMTSYHISDGGHSYINS-------IHGSSSKP
CANGA   RLLACSSHLSATLA-ELNNLVEFKPSQGVQYKNILDMPVT--TRKTGNTA
ASPNI   RILSAVTSLTSLLK-TASTLPKFTPSHGHTYLPPAPKSTDVSHPSVQSQT
RHIO9   RLLTSLFHVNSAFK-ETQKMMSFHPSKGYSWKQAN-------VDQQKKQP
CRYNE   RLKNATSLISKTLSTLSENRPPANPRTTTLWRSIPSVTSNQPDGTQPSSL
USTMA   RLQSTVYALRMSLSTQRRARPSFDPRRGHHGRFIV-------ADAPTSID
ENCCU   RMGDSMWQLNSFID-RMMEKRRFNPFSPPKGRRLA-------KSLEDSKD
ANTLO   RMANALFYIYNALD-LYRERRTFDIRDGFRRKSHE-------EDLYCEND
ARATH*  RVSRTIYNISKAFT-DAASKLE---TIRQV------------DTENQNEP
VITVI   RIGRALYHISKAFT-TAASKLE---KIGYV------------DAENENPA
SOYBN   RIGRALYYIQKAFT-TAASKLE---KIGYA------------DSENETAL
SOLLC   RLGRALYHISKAFS-FASSKLE---KTGYV------------ASENESAA
ORYSJ*  RISRAMHHISKAFT-TACSKLE---KIGHV------------ETEPDTAA
HORVU   RISRAMHHISKAFS-TACSKLE---KIGNA------------ETEADAAA
SORBI   RISRTMHHISKAFT-IACSKLE---KIGNV------------ETESDAVA
SELML   RVARAVHHISAAFS-QVSAKLE---KIGY-------------DDENEH--
PHYPA   RVVRSLYHITNAFA-QASAKLE---KIGTG------------EADPADVQ
               260       270       280       290       300
```

MED6

```
HUMAN*  PK------AKRKEEPSSI----------------------------FQRQ
CHICK   PK------AKKKEEPSSI----------------------------FQRH
XENTR   PK------AKKKEEASSH----------------------------FQRQ
DANRE   PK------SKRKEEPSSL----------------------------FQRQ
CIOIN   --------KKKKPETGSV----------------------------FQIS
ANOGA   KE------APVKEEPSSI----------------------------FQRQ
TETNG   PK------TKKKEEPSSL----------------------------FQRH
DROME   AN------SAKDENSGTL----------------------------FQKQ
BRUMA   --------EKPQLARSTF----------------------------YQRT
CAEEL   DE------EEKLEDRSTN----------------------------FQKT
YEAST*  TN------GNNAGGGSNK--------------------------------
SCHPO   DE------NKNQSIENAD-----------------------YSFSPEDFS
CANAL   SQ------SSAVSKPLST----------------------------NTGT
CANGA   NV------GSVPGTISAS--------------------------------
ASPNI   SKENTPMPDADATNKSQS-------------FTGSQNSSGPAVYDMRSLA
RHIO9   NT-------------------------------------------ILRAQ
CRYNE   DQ------AEKPDEGKKDDSLNGSSPLDGSDGKASPAGPDWHLFHALQAT
USTMA   AD------SNRTCTTQST-------------------------HTHHADD
ENCCU   LDFMMEIFNDFKKEQAES--------------------------------
ANTLO   SELQYAIKLMNEYDINNQ--------------------------------
ARATH*  AE------SKPASETVDL----------------------------KEMK
VITVI   LE------SKVGKETIDF----------------------------KEVM
SOYBN   LE------SKTAKETIDI----------------------------KEVK
SOLLC   SE------PKAAKETIDF----------------------------KELK
ORYSJ*  SE------SKTQKEAIDL----------------------------KELK
HORVU   SE------SKAQKETIDL----------------------------KELK
SORBI   SE------SKTQKEAIDL----------------------------KELK
SELML   -E------SDTSG-RVDL----------------------------KEVL
PHYPA   VK------QSTTK-FVDM----------------------------KEVM
                  310       320       330       340       350
```

```
HUMAN*  RVDALLLDLRQKFPPKFVQLKP---------------------GEKPVPV
CHICK   RVDALLLDLRQKFPPRFVQQKP---------------------GEKPIPV
XENTR   RVDALLMELRQKFPPKFIQQKT---------------------GEKPVPV
DANRE   RVDTLLLDLRNKFPPTFYQTKP---------------------GEKPVPV
CIOIN   RVDTLLASLTAKFPHKYVQHQN---------------------GEKPLPI
ANOGA   RVDMLLGDLLRKFPLPLPQMTNNPT------------------GGNP---
TETNG   RVDTLLVDLRSKFPPTFYQPKP---------------------GEKPIPA
DROME   RVDMLLAELLRKFPPPIPPMLQN--------------------LQQPPPA
BRUMA   RTDQILRELFAKFPPPDDVQFNFGGNEIS--------------VENDTSG
CAEEL   RTMMLLNQLFSEMPAEDA-------------------------LEREEKE
YEAST*  ------------------SSVRPTGGANM--------------ATVPSTT
SCHPO   VVRAFMQSLHSS--------------------------------------
CANAL   NATTTPITLTTPLGATVP--------------------------------
CANGA   ----------LNMPMVNTGRLNSAGAATI--------------GTSGVPN
ASPNI   ESFSLVARYGDEFMDESPLMGEPGSFILS---------------RPGDAD
RHIO9   ETLALRHWMDRTIEMSAVKIVQSRQAMDQ---------------------
CRYNE   RASLVSLETLAKTPTQTTDPREELNNIEIAMDNQFGGQNQSLSGKGPNVK
USTMA   HTPQLEHQQDHIHPPPPAAF------------------------------
ENCCU   --------------------------------------------------
ANTLO   --------------------------------------------------
ARATH*  RVDVILTSLYRKLAPPPPPPPFPEGYVSQ--EALGEKEELGTQGGESQPP
VITVI   RVDQILASLQRKLPPAPPPPQFPEGYAPPP-TAEGEKGPETQQAAEPQLP
SOYBN   RVDHILASLQRKLPPAPPPPPFPEGYVPPP-TAETEKGTETQEAAETQAP
SOLLC   RVDHIFASLQRTLPPVPPPPPFPEGYVPPS-TAEASE----NEQEETQLP
ORYSJ*  RVDHILMSLQRKLQPAPPPPPFPEGYVP----SEQEKASDDLLASEALPP
HORVU   RVDHILMSLQRKLPPAPPPPPFPDGYVP----TEQEKGPDDLLASEALPP
SORBI   RIDHILSSLKRKIGAAPPPPPYPEGYVPP--SAEQEKAPDDVAASEA-PP
SELML   RIDQILGNVLRKLPPAPPPPPMP---TPPSGVPEQPQDSQPEQSTEQGPP
PHYPA   RVDSILAGIMRKLPPAPPPP------------------------------
                  360       370       380       390       400
```

```
HUMAN*  D-QTKKEAEPI----PETVK-PEEKE---TTKNVQQTV-----SA-----
CHICK   D-QIKKEPEPA----PEAVK-TEEKE---TVKNAQQSA-----AA-----
XENTR   E-QIKKEAEPP----AETIK-QEEKE---PVKVAQQGA-----TP-----
DANRE   --EVKKEPEVP----VETVKPQEERETKPPAPPAPPRPPPQTTPN-----
CIOIN   ETSIKKEPKQEKNNFAKVAK-PNANSKSVTTQNKQAL-------------
ANOGA   -----SDTANASNNHGGAAG-DSDHVGADATLIKQEPTEGGVGRAGNN--
TETNG   V-EVKKEPEAP----TEAVK-QEERE--PGAKSSAPAP-----PS-----
DROME   GDDLNTARNASEMNNATG-----------PLDIKTEGVDM----------
BRUMA   VVEKSVQSSSSVNNQNLTATEDPTSGISQQTAFTSPSSV-----------
CAEEL   EVEEEEEETLKTEEPTTSTDEPK---------------------------
YEAST*  NVNMTV---NTMGTGGQTIDNGTGRTGNGNMGITTEMLDKLMVTSI----
SCHPO   --------------------------------------------------
CANAL   ---STVSNGISTSTEIASGVFDT------------------LLNDVVMND
CANGA   SAGMTGANTMATGQTGATSRFENGSSRSSTDAISTDTLDKLLITSM----
ASPNI   RGAAPKQSQPSSTNAGGRVGTPLAKVDTPGKLSDKNSA------------
RHIO9   ---ADQEDASKTNTDISSALRED---------------------------
CRYNE   GGAAAVKNISVPFVPKHSAGHATSTTGLTPSIWGGSASLGVSTAAVPARE
USTMA   --------------------------------------------------
ENCCU   --------------------------------------------------
ANTLO   --------------------------------------------------
ARATH*  QVDPII--------------------------------------------
VITVI   ALDPII--------------------------------------------
SOYBN   TADPII--------------------------------------------
SOLLC   PVDPII--------------------------------------------
ORYSJ*  QVDPII--------------------------------------------
HORVU   AIDPII--------------------------------------------
SORBI   QLDPII--------------------------------------------
SELML   TKKAKV--------------------------------------------
PHYPA   --------------------------------------------------
                  410       420       430       440       450
```

MED6

```
HUMAN*  ------------KGPPEKRMRLQKAV------------------------
CHICK   ------------KGPPEKRMRLQ---------------------------
XENTR   ------------KGPPEKRMRLQ---------------------------
DANRE   ------------KPPPEKRARVQ---------------------------
CIOIN   ------------KAPPEKRLKFM---------------------------
ANOGA   ------------DVPPEKKMKL----------------------------
TETNG   ------------KQPPEKRARLQ---------------------------
DROME   ------------KPPPEKKSK-----------------------------
BRUMA   ------------PLIPAHRSTAESSSGPLSFPGTIPVLGVSSTDSQHETD
CAEEL   ------------FAEPTARTTSKQ--------------------------
YEAST*  ------------RSTPNYI-------------------------------
SCHPO   ------------KEAPDVK-------------------------------
CANAL   DHLYIDEIPLYGEGSTLERLGLKGNKD-----------------------
CANGA   ------------KSTPEYI-------------------------------
ASPNI   ------------AEEPKLRKKKSKPAS-----------------------
RHIO9   ------------GKRPRKRNDDGGSSN-----------------------
CRYNE   AGGSPLKWPGGDKSAPIAGATLAGQTG-----------------------
USTMA   ------------TQPQLKKARFD---------------------------
ENCCU   --------------------------------------------------
ANTLO   --------------------------------------------------
ARATH*  ------------DQGPAKRMKF----------------------------
VITVI   ------------DQGPSKRMKF----------------------------
SOYBN   ------------DQGPAKRMKF----------------------------
SOLLC   ------------DQGPSKRMKV----------------------------
ORYSJ*  ------------DQGPAKRPRFQ---------------------------
HORVU   ------------DQGPAKRPRFQ---------------------------
SORBI   ------------DQGPSKRPRFQ---------------------------
SELML   ------------EKR-----------------------------------
PHYPA   --------------------------------------------------
                 460       470       480       490       500
```

```
HUMAN*  -------
CHICK   -------
XENTR   -------
DANRE   -------
CIOIN   -------
ANOGA   -------
TETNG   -------
DROME   -------
BRUMA   TKRFKTN
CAEEL   -------
YEAST*  -------
SCHPO   -------
CANAL   --AGLSL
CANGA   -------
ASPNI   -------
RHIO9   --RKKKK
CRYNE   --R----
USTMA   -------
ENCCU   -------
ANTLO   -------
ARATH*  -------
VITVI   -------
SOYBN   -------
SOLLC   -------
ORYSJ*  -------
HORVU   -------
SORBI   -------
SELML   -------
PHYPA   -------
```

MED7

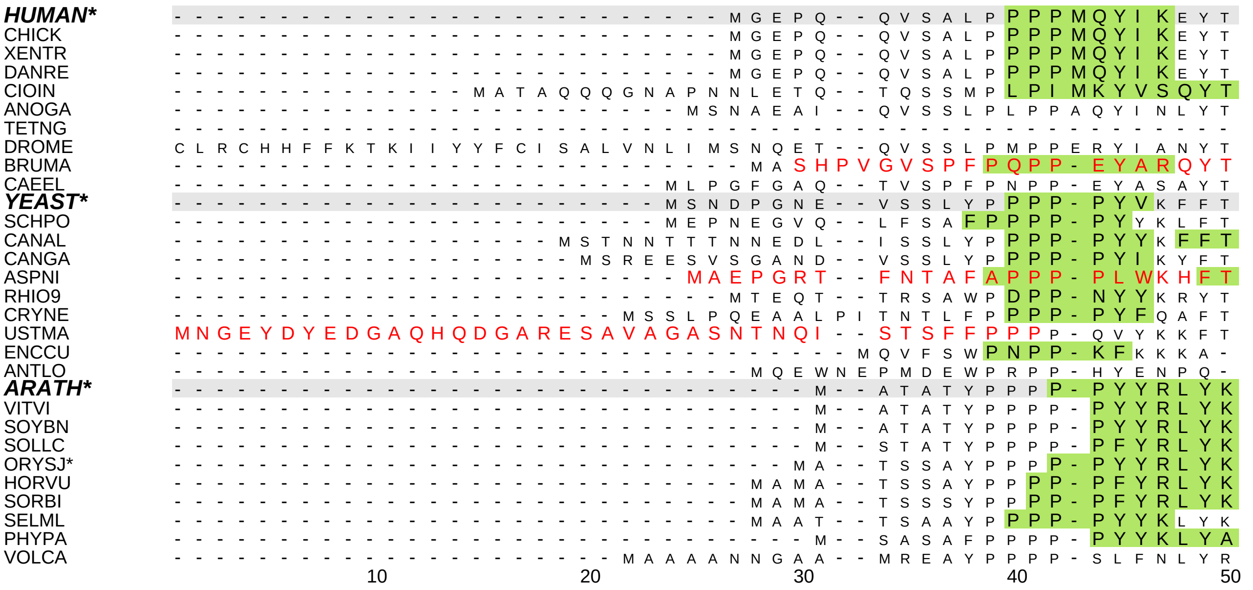
HUMAN*
CHICK
XENTR
DANRE
CIOIN
ANOGA
TETNG
DROME
BRUMA
CAEEL
YEAST*
SCHPO
CANAL
CANGA
ASPNI
RHIO9
CRYNE
USTMA
ENCCU
ANTLO
ARATH*
VITVI
SOYBN
SOLLC
ORYSJ*
HORVU
SORBI
SELML
PHYPA
VOLCA
10
20
30
40
50

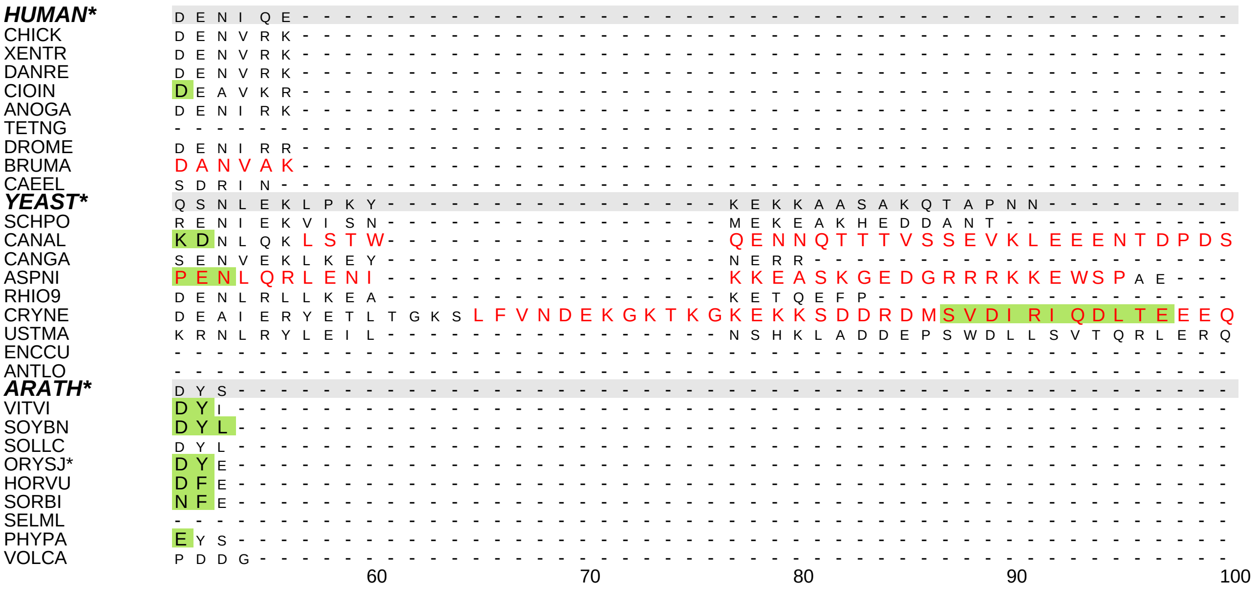
HUMAN*
CHICK
XENTR
DANRE
CIOIN
ANOGA
TETNG
DROME
BRUMA
CAEEL
YEAST*
SCHPO
CANAL
CANGA
ASPNI
RHIO9
CRYNE
USTMA
ENCCU
ANTLO
ARATH*
VITVI
SOYBN
SOLLC
ORYSJ*
HORVU
SORBI
SELML
PHYPA
VOLCA
60
70
80
90
100

MED7

Species	101–150
HUMAN*	-------GLAP---------------------------------------
CHICK	-------GLAP---------------------------------------
XENTR	-------GLAP---------------------------------------
DANRE	-------GLAP---------------------------------------
CIOIN	-------GTAP---------------------------------------
ANOGA	-------NRAP---------------------------------------
TETNG	--
DROME	-------NRAP---------------------------------------
BRUMA	------KNVLP---------------------------------------
CAEEL	----NDSGSAP---------------------------------------
YEAST*	----SNGGSEEEITCA------LDY-----------------------LI
SCHPO	----LQPKTEEEIESL------AKL-----------------------FK
CANAL	NN--LEENSIP--------PGELRF-----------------------LI
CANGA	----EEGESAEN---E------LDF-----------------------LI
ASPNI	----LRSLKIP---------PELRF-----------------------LV
RHIO9	----MDPITSP-----SIPEFYIQS-----------------------LE
CRYNE	NEKLELEGKLE---------------------------------------
USTMA	NAILRQPGSSSKIDAEQLQDEEADMAAIQDQDCDKPMTDLPDFDLKAELE
ENCCU	--
ANTLO	--
ARATH*	----ENPNSAP---------------------------------------
VITVI	----QNPKSAP---------------------------------------
SOYBN	----QNPKSAP---------------------------------------
SOLLC	----QDPKSAP---------------------------------------
ORYSJ*	----KDPSSAP---------------------------------------
HORVU	----QDPSSAP---------------------------------------
SORBI	----QDASSAP---------------------------------------
SELML	-----DQTSAP---------------------------------------
PHYPA	----DDPSSAP---------------------------------------
VOLCA	----VSPLPPP---------------------------------------

110 120 130 140 150

Species	151–200
HUMAN*	KPPPP--I--------KDSYMMFGNQFQCDDLIIRPLESQGIERLHPMQ-
CHICK	KPPPP--I--------RDSYMMFGNQFQCDDLIIRPLESQGIERLHPMQ-
XENTR	KPPPP--I--------RDSYMMFGNQFQCDDLIIRPLESQGIERLHPIQ-
DANRE	KPPPP--I--------RDSYMMFGNQFQCDDLIIRPLESQGIERLHPMQ-
CIOIN	KPPAP--V--------RTSYEMFGQECHQDDAIIRPLETQGLSRLHPEV-
ANOGA	KPPAP--I--------QDAYTMFGSPFSNDDNIIRPLESQGFKRLYPQH-
TETNG	----P--I--------RDCYMMFGNQFQCDDLIIRPLESQGIERLHPQQ-
DROME	RPPPPPAQ--------HEVYSMFGIQYNNDE-MIRSLESQNIKRLIPIH-
BRUMA	PPPVP------------SEFTVFGEEYNFEEEMIRSLQSQKMQQLFSSN-
CAEEL	PPPHP-----------LTEFKVYGEEYRLEDDVIAPLKNAGVAELYKNK-
YEAST*	PPPMP--K--------NQQYRAFGSIWQVKD-QLPDLESMGLTQLYKKST
SCHPO	KPSCL--T--------SGTYQMFGDTWRLDE-AIPSLKEFGIPELYKDIK
CANAL	PPKQP--E--------GPQYRGYGNIWLFED-KLPNLKDSQWEQLYNTST
CANGA	PPPMP--S--------SGSYRAFGSVWQIKD-HLPDLETMGITQLYKKTS
ASPNI	PPEIP-----------SEQYSIFGEVQNLST-ALPSLEEQGITQLYPSSP
RHIO9	PPAAP-----------TSEYTVFDQKWQIED-QLPTLNELGVKQLFKDGE
CRYNE	KPRAD-------WVNEDGRWMCFGTMYTTEP-IIPTAQSIGLPPFIDPA-
USTMA	PPNVD-------WIEEDGGYTVFGQLWPIPD-VTPTLQQLGIPVLYPLEG
ENCCU	PPKIP------------SSYTSFGTRYEVVS-GTPVNTSFSSTE------
ANTLO	IPEVP------------AEVFCFGER------IVERETSTGESP------
ARATH*	EPPPP--I--------EGTYVCFGGNYTTED-VLPSLEEQGVPQLYPKDS
VITVI	EPPPP--I--------EGTYALFGATYTTDD-VLPSLEEQGVRQLYPKGS
SOYBN	DPPPP--I--------EGTYLCFGANYTTSD-VLPTLEEQGVRQLYSKGP
SOLLC	EPPPP--I--------EGTYVLFGSNYTTDD-VLPNLEEQGVRQLYPKGP
ORYSJ*	EPPPP--V--------DGPYQLFGATYTTDV-VLPSLEDQGVRQLYPKSP
HORVU	EPPPP--I--------EGSYQLFGATYTTDV-VLPSLEDQGVRQLYPKGP
SORBI	EPPPP--I--------DGPFKALGVDYTGDV-VIPSLESHNVRQLYPKGP
SELML	SPPSP--L--------QGTYSVFGNTYTTDD-ALPSLEEVGLRQLYPKGP
PHYPA	PPPPP--V--------VGTYVQYGANYTTTD-ILPGLEEEGLRQLYPTGS
VOLCA	PPPIPTLADVAALRERKIELKVLGNPLKLHEDLVPNLTTQALFRQQPDGT

160 170 180 190 200

MED7

Species	Alignment (positions 201–250)
HUMAN*	---FDHK-----
CHICK	---FDHK-----
XENTR	---FDHK-----
DANRE	---FDHK-----
CIOIN	---YDKR-----
ANOGA	---FDRR-----
TETNG	---FDHK-----
DROME	---FDRR-----
BRUMA	---GDWR-----
CAEEL	---NNWK-----
YEAST*	E-----------------------------------NES--TNYQY----
SCHPO	DGEDEIEVVEYDPKSNAIVGTSFTRVHDYDKNSPIENEKILEDDQHTAMK
CANAL	STFTSTSTSTANTNIITNTDTDINGNQEVDAGDSTGDENLTSETK-----
CANGA	E-----------------------------------GEAEVTDYQY----
ASPNI	K--PDTK-----
RHIO9	---IDRV-----
CRYNE	---VEPQ-----
USTMA	---TDRK-----
ENCCU	---FDK------
ANTLO	---EDIV-----
ARATH*	N--LDYK-----
VITVI	N--VDFK-----
SOYBN	N--VDFK-----
SOLLC	N--VDFK-----
ORYSJ*	D--IDFK-----
HORVU	D--IDFK-----
SORBI	N--IDFK-----
SELML	N--IDYK-----
PHYPA	D--VDYK-----
VOLCA	---VDFK-----

210 220 230 240 250

Species	Alignment (positions 251–300)
HUMAN*	--
CHICK	--
XENTR	--
DANRE	--
CIOIN	--
ANOGA	--
TETNG	--
DROME	--
BRUMA	--
CAEEL	--
YEAST*	--
SCHPO	EKDNESDKTMKDVEEKTEPSLKKEEEDIQMKEPLDSQDTGAVSASSVNEG
CANAL	--
CANGA	--
ASPNI	--
RHIO9	--
CRYNE	--
USTMA	--
ENCCU	--
ANTLO	--
ARATH*	--
VITVI	--
SOYBN	--
SOLLC	--
ORYSJ*	--
HORVU	--
SORBI	--
SELML	--
PHYPA	--
VOLCA	--

260 270 280 290 300

MED7

```
HUMAN*  ----------------------KELRKLNMSILINFLDLLDILIR-----
CHICK   ----------------------RELKKLNMSILVNFLDLLDILIK-----
XENTR   ----------------------RELKKLNMSILVNFLDLLDILIK-----
DANRE   ----------------------RELKKLNMSILVNFLDLLDILIK-----
CIOIN   ----------------------NELKKISVSILCSFLDLLDVLVK-----
ANOGA   ----------------------KELKKLNHSLLVNFLDLIDLLVH-----
TETNG   ----------------------RELKKINMSILVNFLDLLDILIK-----
DROME   ----------------------KELKKLNHSLLVNFLDLIDFLIL-----
BRUMA   ----------------------SELKKLNRSTVAAFLDLLDILIR-----
CAEEL   ----------------------TEMKKLNRSAIVAFFDLVEILIR-----
YEAST*  --------------------KIQELRKLLKSLLLNYLELIGVLSI-----
SCHPO   FRADQKSKDGETSDLIKIPRRAYELRFLSRSLMLNFLELLGIMAK-----
CANAL   ---------------------IKELHKLMDSLLLNFLELIGILSI-----
CANGA   --------------------KIKELRRLSYSLLLNFVELVGVLSV-----
ASPNI   --------SGDSSQPAQPLNHAYYLLKISKSLLLNFLEFVGILSV-----
RHIO9   ----------------------QELKRLNRILITQFLQLLDTLVK-----
CRYNE   ----------------------ESLPPLLHSFLHTLLLLLDTLTMTAR--
USTMA   ----------------------ELLLTLLRTLLQTYREITGDLLKPAQPY
ENCCU   ----------------------SKLRELVNLSFSTFVELLS---------
ANTLO   ----------------------ADIRALVRESELGFRKALETM-------
ARATH*  ----------------------NELRSLNRELQLHILELADVLVD-----
VITVI   ----------------------KELRSLNRELQLHLLELADVLVE-----
SOYBN   ----------------------KELRSLNGELQLHVLELADILIE-----
SOLLC   ----------------------KELRALNRELQLHILELADVLVE-----
ORYSJ*  ----------------------KELRTLNRELQLHILELADILVE-----
HORVU   ----------------------KELRTLNRELQLHILELADILVE-----
SORBI   ----------------------KELRTLNRELQLHILELADILVE-----
SELML   ----------------------KELKALNRELVLQMMELADVLVE-----
PHYPA   ----------------------KELRALNRELILQFLELIDALIE-----
VOLCA   ----------------------GDLKRLSGELLFMFLELLKAVVE-----
                 310       320       330       340       350
```

```
HUMAN*  ------------SPGSIKR---------------EEKLEDLKLLFVHVHH
CHICK   ------------SPGSIKR---------------EEKLEDLKLLFVHMHH
XENTR   ------------SPGSIKR---------------EEKLEDLKLLFVHMHH
DANRE   ------------SPGSIKR---------------EEKLEDLKLLFVHMHH
CIOIN   ------------SGMSTAR---------------DEKIEDLNLLFIHMHH
ANOGA   ------------YPDSPRR---------------AEKIEDLSLLFVHIHH
TETNG   ------------SPGSMKR---------------EEKLEDLKLLFVHMHH
DROME   ------------NPDSPRR---------------TEKIDDISLLFVNMHH
BRUMA   ------------CPNHPER---------------LEKINNLRLLFINMHH
CAEEL   ------------APDHPMR---------------EEKMVDLHTIFINMHH
YEAST*  ------------NPDMYER-----------------KVENIRTILVNIHH
SCHPO   ------------APEQFPS-----------------KVENIRVLLLNLHH
CANAL   ------------DPIKYDK-----------------KIHDINLILININH
CANGA   ------------NPELYES-----------------KVENIRTILVNIHH
ASPNI   ------------APEQFEP-----------------KVEDIRNLFINAHH
RHIO9   ------------DPEEFGK-----------------YIENISTTFINMHH
CRYNE   ------------TPNELAAA--------GWASEGDQYIQHLTNLSANMMV
USTMA   DVWVPAVPDPNLTPEQQQQQMATNPGFWTQTTEAKDRLKHMQNVVVNMQF
ENCCU   ------------FPPGHEE-----------------LIETISSIHLEINQ
ANTLO   ------------------------------------ETNVLERTHRKIHK
ARATH*  ------------RPSQYAK-----------------RIGEISSIFKNLHH
VITVI   ------------RPSQYAR-----------------RVEDISLIFKNLHH
SOYBN   ------------RPSQYAR-----------------RVEEISTVFKNLHH
SOLLC   ------------RPSQYAR-----------------RVEEISLIFKNLHH
ORYSJ*  ------------RPSQYAR-----------------RVEDISLIFKNLHH
HORVU   ------------RPSQYAR-----------------RVEDISLIFKNLHH
SORBI   ------------RPSQYAR-----------------RVEDITLIFKNLHH
SELML   ------------RPSQYAR-----------------RVEDMGLILRNMHH
PHYPA   ------------RPSQSAR-----------------CVEDITLILRNVHH
VOLCA   ------------KPSGYAA-----------------QLTPVNVLLSNLVQ
                 360       370       380       390       400
```

MED7

```
HUMAN*   LINEY-RPHQARETLRVMMEVQKRQRLETAERFQKHLERVIEMIQNCLA-
CHICK    LINEY-RPHQARETLRVMMEVQKRQRLETAERFQKHLERVVEMIQGCLS-
XENTR    LINEY-RPHQARETLRVMMEVQKRQRLETAERFQKHLERVVEMIQGCLS-
DANRE    LINEY-RPHQARETLRVMMEVQKRQRLETAERFQKHLERVVEMIQGCLS-
CIOIN    LINEY-RPHQARETLKVMMALQKKNMIDVSETLCKNMDKASEILHQCSQ-
ANOGA    LLNEF-RPHQARETLRVMMELQKRQRIETAQRFQNHLEKVREMVKNAFA-
TETNG    LINEY-RPHQARETLRVMMEVQKRQRLETAERFQKHLERVVEMIQGCLA-
DROME    LLNEF-RPHQARETLRVMMEMQKRQRVETAARFQKHLERVREIVNTAFS-
BRUMA    LINEY-RPVQARDALQSMMRLLIKTIEDVTSRFRTYLAMGHEALNQVID-
CAEEL    LINEF-RPVQARDSVRILQERQIEELSDICKDFKKYLRDGREVVDDQFQ-
YEAST*   LLNEY-RPHQSRESLIMLLEEQLEYKRGEIREIEQVCKQVHDKLTSIQD-
SCHPO    LINDY-RPHQSRESLIMLLEKQLKHEESQVELLRTHNRQMTETLEKYKSL
CANAL    LLNTY-RPHQSRESLIMLLKNQIDYKFLEIDQINKKCLDIKSKIKNLIN-
CANGA    LLNEY-RPHQSRESLIMLLEEQLEHKKQEVANIELVCQQVTEKLKQVQT-
ASPNI    LLNLY-RPHQARESLIMMMEAQLARTKDEIQQMDKLKEEVNAVLDQLAA-
RHIO9    ILNAY-RPHQARETLRLLMEDQIAKKRQQTQEIREKIKETLNTLQAIEHE
CRYNE    ASNQL-RSAQSEATLVLLMEKELEERRKQTEKLRSKCKEIASGIRALKGL
USTMA    LINEL-RPVQAKETLKLIMQMQLERRKQEMQLIQERCATMRARVEELKK-
ENCCU    ILNGG-KGMEAASEIRRIRNDHTRNKNRVAEEVRKKILNFKI--------
ANTLO    RISELERAVQEHNLWRRAVPE-----------------------------
ARATH*   LLNSL-RPHQARATLIHIMELQIQQRKQAVEDIKRRREEAQRLLKDAYL-
VITVI    LLNSL-RPHQARATLIHILELQIQRRKQAVEDIKRRREEAQRLLKEAVG-
SOYBN    LLNSL-RPHQARATLIHILELQIQRRKQAVEDIKRRREEARRLLNESLA-
SOLLC    LLNSL-RPHQARATLIHILELQIQRRKQAIEDIKRRREEAQKLLKEALG-
ORYSJ*   LLNSL-RPHQARATLIHMLENQIQRRKQAIEDIKQRREEAQKLLGESLL-
HORVU    LLNSL-RPHQARATLIHLLESQIQRRKQAIEDIKQRREEAQRLLGESLI-
SORBI    LLNSL-RPHQARATLIHMLESQIQHRKQAIEDIKQRREEAQKLLGESLH-
SELML    LLNSL-RPHQARATLIRILETQVQRRKQAVEEVRKRRNEARLLLQESTA-
PHYPA    LLNSL-RPHQARATVIHMLEAQLIRRKEALAKIRRKRAEARAMLQEFSV-
VOLCA    LTNLM-RPYQARATLEATLALQVGTMRDSIRRVRQQVTAADEVLKGMAR-
                  410       420       430       440       450
```

```
HUMAN*   ------------SLPDDLP------HSEAG-------------------M
CHICK    ------------SLPDDLPLPNSSSMGEAGSAAGS--------------S
XENTR    ------------SLPDDLPLPNSSSMGEAGCAAGS--------------S
DANRE    ------------SLPDDLPLPNSSSMGEAGSAAGS--------------S
CIOIN    ------------ELAAMKVKTEASTILPNNSNHSEGIDSMDIGSVLGF--
ANOGA    ------------SLPD----------LTDADRLAGGL-------------
TETNG    ------------SLPDDLP------QIDCVDGVGDGLNSVSVGCPTGLLP
DROME    ------------ALP----------VLDDDDDSGGAK-------------
BRUMA    ------------EVPPVLSASPMLNVEDVNVGVS----------------
CAEEL    ------------MIRGKLPAPPQPSELTRVKLQDGVLHMLQETEASDDVE
YEAST*   ------------TLRTGSQSPPSSSQ------------------------
SCHPO    DFNMEKEGDVIQQLKSSIKKPLSGAEDEQKS-------------------
CANAL    --------ERITVVSDGTTTNTTTTT------------------------
CANGA    ------------LLKESQSQSQSQSQSQSQSQS-----------------
ASPNI    ------------EGADVGSTIQSTTK------------------------
RHIO9    --------------------------------------------------
CRYNE    --------------------------------------------------
USTMA    ------------MLGKQSDCYGSS--------------------------
ENCCU    --------------------------------------------------
ANTLO    --------------------------------------------------
ARATH*   ------------TLDGQ---------------------------------
VITVI    ------------TLEGQ---------------------------------
SOYBN    ------------TLDGH---------------------------------
SOLLC    ------------TLEGQ---------------------------------
ORYSJ*   ------------ILDGNQPSLPAM--------------------------
HORVU    ------------IIEGSQQQVMTPM-------------------------
SORBI    ------------ILDGSQTN------------------------------
SELML    ------------TLENQLS-------------------------------
PHYPA    ------------TL------------------------------------
VOLCA    ------------AL--VLCGNCHRRIIPEAFKEGGLCACITAVGLCASGC
                  460       470       480       490       500
```

MED7

```
HUMAN*   RVKTEPMDADDS-NNCTGQNEHQRENSGHRRDQIIEKDAALCVLIDEMNE
CHICK    RLKTEPMDVDDV-AGASCMVLQTDKNVPVKKDGVCDNDVVMCRIIDEMT-
XENTR    RLKTEPMDVDDV-AGASCMVLQTDKNVPVKKDKVCDNDAAMCRIIDEMT-
DANRE    RLKTEPMDVDDV-AGASCMVLQTDKNVPVKKDGVCDNDVVMCRIIDEMT-
CIOIN    ----DSSSVIDQSNSMMEVDSGYRENSHNEEDTQDEKDIIMCSFVDGMKE
ANOGA    ----EPMDVGES-----------GDLAGGRGEGCHPLDRLMCELVDRM--
TETNG    KLKTEPMDVEEVTASCMAAGEQDKSVVTSKKEKIWDKDGAVCSIIDEI--
DROME    ----IKTEVDPL-----------EANAAAKNDPSYQHDRMLCKLVDAIE-
BRUMA    --IEQQVGAVETEISKRRSSYDEARKTTVSRREAWRRDVMLCELLDNMGD
CAEEL    MKEEEGSEYSKKKA---------RLELLSREDGPPSVVHLLARQFHDISL
YEAST*   --------------------------------------------------
SCHPO    -----------------------RSMFSKNDEKLKKSLELMEDVIKRDLS
CANAL    -----------------------KTTGLGDGDSIDEKEQLKQDIINRLLL
CANGA    -----------------------QSQLQSDSQ------------------
ASPNI    -----------------------DKKGVKPEDQIPEDSKLLWDILDGKLD
RHIO9    --------------------------------------------------
CRYNE    --------------------------------------------------
USTMA    --------------------------------------------------
ENCCU    --------------------------------------------------
ANTLO    --------------------------------------------------
ARATH*   --------------------------------------------------
VITVI    --------------------------------------------------
SOYBN    --------------------------------------------------
SOLLC    --------------------------------------------------
ORYSJ*   --------------------------------------------------
HORVU    --------------------------------------------------
SORBI    --------------------------------------------------
SELML    --------------------------------------------------
PHYPA    --------------------------------------------------
VOLCA    WLCACLPFCVNVTKDTVYRCPACNAEIHRRRAPFE---------------
                  510       520       530       540       550
```

```
HUMAN*   ----------------RP
CHICK    ------------------
XENTR    ------------------
DANRE    ------------------
CIOIN    ------------------
ANOGA    ------------------
TETNG    ------------------
DROME    ------------------
BRUMA    ADLERRLSPSTSRVQMEY
CAEEL    ----------------KK
YEAST*   ------------------
SCHPO    ------------------
CANAL    ----------------SI
CANGA    ------------------
ASPNI    ----------------D-
RHIO9    ------------------
CRYNE    ------------------
USTMA    ------------------
ENCCU    ------------------
ANTLO    ------------------
ARATH*   ------------------
VITVI    ------------------
SOYBN    ------------------
SOLLC    ------------------
ORYSJ*   ------------------
HORVU    ------------------
SORBI    ------------------
SELML    ------------------
PHYPA    ------------------
VOLCA    ------------------
                  560
```

MED8

HUMAN*	--
CHICK	--
XENTR	--
DANRE	--
CIOIN	--
ANOGA	--
TETNG	--
DROME	--
BRUMA	--
CAEEL	--
YEAST*	--
SCHPO	--
CANAL	--
CANGA	--
ASPNI	--
RHIO9	--
CRYNE	--
USTMA	--
ENCCU	--
ANTLO	--
ARATH*	--
VITVI	--
SOYBN	--
SOLLC	--
ORYSJ*	--
HORVU	--
SORBI	--
SELML	--
PHYPA	MVVSPTPILHGTVLHACWRRAFKARCCRTGIRASKAVRVVALTPSSLPSP
	10 20 30 40 50

HUMAN*	--
CHICK	--
XENTR	--
DANRE	--
CIOIN	--
ANOGA	--
TETNG	--
DROME	--
BRUMA	--
CAEEL	--
YEAST*	--
SCHPO	--
CANAL	--
CANGA	--
ASPNI	--
RHIO9	--
CRYNE	--
USTMA	--
ENCCU	--
ANTLO	--
ARATH*	--
VITVI	--
SOYBN	--
SOLLC	--
ORYSJ*	--
HORVU	--
SORBI	--
SELML	--
PHYPA	SRPPRTFAPLHTRSFPFPCSWNWRVSGHAEGEQLLAESRNWKANVSVQPN
	60 70 80 90 100

HUMAN*	----------------------------------DWKTGSEIVAAASAAA
CHICK	--
XENTR	--
DANRE	---M
CIOIN	---M
ANOGA	--
TETNG	---L
DROME	--
BRUMA	--MNAQPGGLFG
CAEEL	-------------------------------------MEYPNPPPPVTSN
YEAST*	-----------------------------MSQSTASLVPEGNQGSLQEDV
SCHPO	---M
CANAL	------------------------------MSQTPFNPNSPSVPTTTSLP
CANGA	--------------------------------------MSGINGALVEDI
ASPNI	--
RHIO9	--MQ
CRYNE	-------------------------------------MQHQHRPPQEIYH
USTMA	---------------------------MSSKRAGEATATAAVAATTAAPP
ENCCU	--
ANTLO	--
ARATH*	-------------------------METQPQQPPP---------PPVAEK
VITVI	-------------MAAMEGIQDQSQQQQQQQAAPP---------KPAVER
SOYBN	---MEGEGGGEG
SOLLC	------------------MEGVVAAQDQQQQQPQP---------PRVVEG
ORYSJ*	----------------MD--AAALGA--AAGAPNPAAPVGGDQ-QPRTER
HORVU	--
SORBI	----------------MDPAAAALGAAPAAGAPPPGAAAGEQQAAPRVER
SELML	--
PHYPA	VNGESGIAKHGPDSLSEEVMAAPGGQVQAAVAAAP---------GQAAQR
	110 120 130 140 150

MED8

```
HUMAN*  MQREEKQLEASLDALLSQVADLKNSLGSFICKLE------NEYGRLTWPS
CHICK   MQREEKQLELTLEALISQVADLKNSLVSFIYKLE------NEYDRLTWPS
XENTR   MQREEKQLEACLDALISQVSDIKNSLVGFIHKLE------NEYDRLTWPS
DANRE   QQREEKQLEAFLESLVARVAHLKGSLQSFIYKLE------NEYDRLTWPS
CIOIN   QANEAKVLEMSVDALIVRAKAIRKALEDFLFKLT------NEHQTLTWPT
ANOGA   MQREEKQLDMLLEAVLNRLNDLKHSIGVMIHRLE------TEYETINWPT
TETNG   QQREEKQLETSVESLISRVAHVKAALHSFIYKLE------NEYERLMWPS
DROME   MQRDEKLFELTLDTVLQRLNDLKLAVLSMIQKLE------LEYETINWPT
BRUMA   HQHEPERLAGAISHIENKALDVKTNIEQLLFMLD-------LQEEVEWPD
CAEEL   YQGEPEKIAQATDMMIKRVTDAKKIIEELLQMLD-------LQEKCPWPL
YEAST*  SFDFNGVPGQALDAVRMRLAQLTHSLRRIRDEMS-------KAELPQWYT
SCHPO   EDISTEKTVESLEAIRHRIAQIVQSLTHFLAILH------QSESLSPWPT
CANAL   STDFSQIPYDVLESIRNRLNQIRQSLTKLADQINNHNRHPQKIKLPSYLY
CANGA   KPDYSGVPGPALDSVRMRLAQLTHSLRRIRDDLS-------RADLTQWYS
ASPNI   MTTLSPDHLKTLEQSRQRLIQLTHSLGSLITSLN------QSDPLPTWSS
RHIO9   AIQLDPNTYQELETLRNKLWSLQETFATHITYLK------EPKYPFTWPD
CRYNE   PPQPPSIPLPTINTLIPRLTTTINDIDSLKTLVA-NGA--HNDTIPSWDV
USTMA   TLPRAQIPVEHLQILLQRFESLLSSIVGLKEEIA------VVPGMDEWPT
ENCCU   -----------MGDLNGRMDSLTKEIRHLSKELQ---------------N
ANTLO   -----------MHAESGRYDMLILELR----------------------T
ARATH*  LN-PKLEKELNLESLKTRAVSLAKAIARILEDFD-AYGRTN--TTPKWQD
VITVI   LN-PAVQQQLNLESVKTRAISLYKAISRILEDID-AVARAN--AVPKWQD
SOYBN   LN-QAVQQQLNLKQVKTRAISLFKAISRILEDFE-AYGRTN--STPKWQD
SOLLC   LN-PAVQQQLNLESVKTRAVSLFKAISRILEDFD-AIARTN--AVPKWQD
ORYSJ*  LS-AGVQQQLNLEGMRARAVGLYKAISRILEDFD-AIARANPNASPKWQD
HORVU   --------------------------------------------------
SORBI   LS-AGVQQQLNLEGMRARAVGLYKAISRILEDFD-VIARTNPSASPKWQD
SELML   ----------------------MHDLQAILTRIT-HSIHTN--PSSKWGD
PHYPA   TDMAPVLQQLNLASLRTRTQDLHNAISRILHSFH-TL------PALKWSE
                  160       170       180       190       200
```

```
HUMAN*  VLDSFALLSGQLNTLNKVLKH-------EKTPL--------FRNQVII--
CHICK   VLDSFALLSGQLNTLNKVLKH-------EKTPL--------LRNQVII--
XENTR   VLDSFALLSGQLNTLNKVLKN-------EKTPL--------LRNQVII--
DANRE   VLDNFALISGQLNTINKLLRN-------EKTPS--------YKSQVII--
CIOIN   VLDNFALLSGQISSLLAAMKS-------EKMPA--------LRNYPIV--
ANOGA   FLDNFALISSHLTGLMKILST-------EIGTP--------LRNLTVL--
TETNG   VLDNFALLSGQLNTINKLLRN-------EKTPS--------FRNQVII--
DROME   FLDNFAIISSHLTGLTKILAK-------EQCPP--------LRNRTVL--
BRUMA   MLDKFSSLASAMTQLQFILKK-------SALPSGFEDFGFFLRTHVLV--
CAEEL   MLEKFSTLASFMSSLQSSVRK-------SGMPHGHEDYGQFLRSHVLV--
YEAST*  LQSQLNVTLSQLVSVTSTLQH-------FQET---------LDSTVVY--
SCHPO   IHKNFNILLSQIHSLSNNLAA-------HSHT---------LQTTSIY--
CANAL   FQNQFQVLITQLMSITNQLYN-------NEDL---------LRNTNVY--
CANGA   LQSQINVTLSQLMSVTSTLQH-------FEDT---------LDSTIVY--
ASPNI   LQTQASIISNNLQSISTHLSE-------NHEL---------LRSISAL--
RHIO9   LLNKFNMLTAKFASLSEDFYR-----------YTEKASTATLPKLMLH--
CRYNE   LLQRYSLLLGRINALSATITAPPARPNVTGYSQKPPPQTPILSRYLVH--
USTMA   LLHRYTNLLSHAYSLSSSLLSASPHFLPSQLASFNRVLEAEANNANLYAD
ENCCU   GNREIKRLEAKVREQREEI-------------------------------
ANTLO   ANDKYRRLEERHTSLSKQLFD-----------------------------
ARATH*  ILGQYSMVNLELFNIVEEVKR-------V------------SNAFVVL--
VITVI   ILGQFSMVNLELFNIMEDIKK-------V------------SKAFVVH--
SOYBN   ILGQYSMVNLELFNIVDDIKK-------V------------SKAFVVH--
SOLLC   ILGQFSMVNLELYNIVEDIKK-------V------------SKAFVVH--
ORYSJ*  VLGQFSMVSMELFNIVEDIKK-------V------------SKVFVVY--
HORVU   --------------------------------------------------
SORBI   VLGQFSMVSMELFNIVEDIKN-------V------------SKVFVVY--
SELML   VLGQFSVVNLELISLVEEIKP-------I------------LRAFVVY--
PHYPA   VLGQFAMVNVELLNLVEDIKP-------I------------LKAFVVY--
                  210       220       230       240       250
```

```
HUMAN*  -------------------------------PLVLSPDRDEDLMRQTEGR
CHICK   -------------------------------PLVLSPDRDEEIMRQTEGR
XENTR   -------------------------------PLLLSPDRDEEIMRLTEGR
DANRE   -------------------------------PLLLSPDRDEELAKLTEHR
CIOIN   -------------------------------PVRLSPDPDPHLLALTEGR
ANOGA   -------------------------------PLMLTPERDEALLQLTEGR
TETNG   -------------------------------PLLLSPDRDEDLAKLTEQR
DROME   -------------------------------PLLVSMDRDDTLINITEGR
BRUMA   -------------------------------PHCLSNDIDPNLQQATSNR
CAEEL   -------------------------------TQRLQYEPDETLQRATQGR
YEAST*  -------------------------------PL---------------PK
SCHPO   -------------------------------PS---------------LE
CANAL   -------------------------------PT---------------PI
CANGA   -------------------------------PL---------------PK
ASPNI   -------------------------------PA---------------PE
RHIO9   -------------------------------PY-----------------
CRYNE   -------------------------------PL---------------TP
USTMA   TSGSSSLFGGILGGGSDSSADDGVKATADGLPELSFYDRSNPLPLLLAHP
ENCCU   --------------------------------------------------
ANTLO   --------------------------------------------------
ARATH*  -------------------------------PKNVNAM------------
VITVI   -------------------------------PKNVNVE------------
SOYBN   -------------------------------PKNVNAE------------
SOLLC   -------------------------------PKNVNAE------------
ORYSJ*  -------------------------------PRNVNAE------------
HORVU   --------------------------------------------------
SORBI   -------------------------------PRNVNAE------------
SELML   -------------------------------PKNVSAG------------
PHYPA   -------------------------------PKNVNAE------------
                  260       270       280       290       300
```

MED8

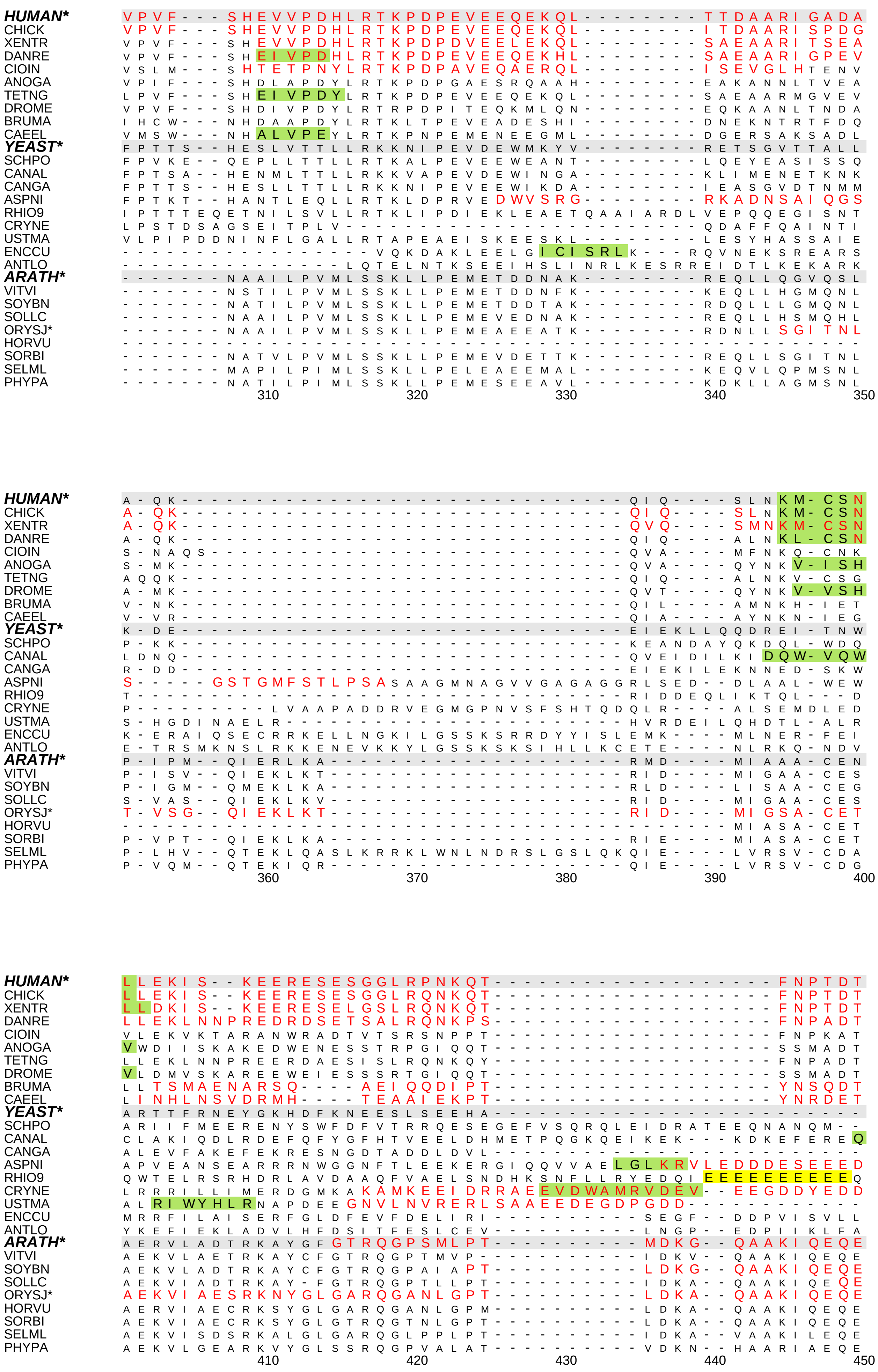
HUMAN*
CHICK
XENTR
DANRE
CIOIN
ANOGA
TETNG
DROME
BRUMA
CAEEL
YEAST*
SCHPO
CANAL
CANGA
ASPNI
RHIO9
CRYNE
USTMA
ENCCU
ANTLO
ARATH*
VITVI
SOYBN
SOLLC
ORYSJ*
HORVU
SORBI
SELML
PHYPA
310
320
330
340
350
360
370
380
390
400
410
420
430
440
450

MED8

```
HUMAN*  NALVAAVAFGKGLSNWRPSGSSGPGQAGQPGAGTIL--------------
CHICK   NALVAAVAFGKGLSNRRPPGSGGSVQSGQPGAGAII--------------
XENTR   NALVAAVAFGKGLSNRRPPGQGGPMAPGQTGASSML--------------
DANRE   NALVAAVGFGKGLSKCRPP---GPVAPGHPGQ-PMM--------------
CIOIN   EELVAAAYFGRGLKPPQ-----SLASQPQINTSTVT--------------
ANOGA   QALVAAVGLGNGLTAPV-----G----PPTGAGVMI--------------
TETNG   NTLVGAVAFGRGLSKCRPA---GPVAPGHPGPGTMM--------------
DROME   QLLVAAVGMGKGLKLTN----------YGPGPGMMV--------------
BRUMA   QKLVRAIVNGEGLRPSK-----TLVSAEGSLTGGSV--------------
CAEEL   AKIVKSILTGEGIRSQR-----TMAPAPPSSAPMTT--------------
YEAST*  ---------SLLVRDSK---------------------------------
SCHPO   --------------------------------------------------
CANAL   IELKITAGGQKSLHPNQ---------------------------------
CANGA   ---------GNALKPVK---------------------------------
ASPNI   EEMDVGFGGRIDGAPDN-----AGAAAGEHALPLVP--------------
RHIO9   DWEKMGFPNEEIWKKWK---------------------------------
CRYNE   EIAVAPTKGNDETRDDD-----DLFGSDDESMEAVP--------------
USTMA   ---------EEGNESDQ-----DKDDDSEDGVEAVT--------------
ENCCU   DSISPSKQMLQGQEEQD-----GIHLK-----------------------
ANTLO   EKVGNIHNAAKQANG-----------------------------------
ARATH*  KMLRDAVNDGKGTQLPP-----D----QRQITTALPP-HLADVLIIN---
VITVI   NLLRAAVNFGEVLRIPL-----D----QRKITSTLPM-HLVDVLTVG---
SOYBN   NLLRSAVNAGDGLRIPG-----D----QRHITPAQPPLHLADALPVIV--
SOLLC   NLLRTAVNHGEGLRVPG-----D----QRHITSALPG-HLVDVLTVS---
ORYSJ*  GLLRAAVNYGEGLRVPG-----D----QRQMYSSLPS-HLVDVLPFG---
HORVU   SLLRAAVNYGEGLRVSG-----D----QRQMHSSLPS-HLMEVLATG---
SORBI   GLLRAAVNYGEGLRVPG-----D----QRQ-PQSLPS-HLIEVLPLG---
SELML   KLLHAAANFGEGLRILP-----E----QRNAPVVLPP-HIASALASA---
PHYPA   NLLRAASNSGDGLRISP-----D----QQQAPAMLPP-HLSSALGTSAID
                 460       470       480       490       500
```

```
HUMAN*  --------------------------------------------------
CHICK   --------------------------------------------------
XENTR   --------------------------------------------------
DANRE   --------------------------------------------------
CIOIN   --------------------------------------------------
ANOGA   --------------------------------------------------
TETNG   --------------------------------------------------
DROME   --------------------------------------------------
BRUMA   --------------------------------------------------
CAEEL   --------------------------------------------------
YEAST*  --------------------------------------------------
SCHPO   --------------------------------------------------
CANAL   --------------------------------------------------
CANGA   --------------------------------------------------
ASPNI   --------------------------------------------------
RHIO9   --------------------------------------------------
CRYNE   --------------------------------------------------
USTMA   --------------------------------------------------
ENCCU   --------------------------------------------------
ANTLO   --------------------------------------------------
ARATH*  -------------------------DAGKI--------------ALPGQS
VITVI   -------------------------DGVQTFSDASGMYMKN---TP--PQ
SOYBN   -------------------------NDPTA--------------QPQLSS
SOLLC   -------------------------DGPQSFADSSGTYLKN---TPPFPS
ORYSJ*  -------------------------DGAHNFGDNSGVYPKN---TSTFVP
HORVU   -------------------------DGAHNFGDNSGAYPKN---TPAFSP
SORBI   -------------------------DGAQNFGDSSGSYPKN---TSTFAP
SELML   -------------------------DDGKGAPIIGGM-------RPTTPQ
PHYPA   TGCFRSPYYFSSKHILAMILLLHNYEGSSFPRGSSSALPPSGLLRPAAPQ
                 510       520       530       540       550
```

```
HUMAN*  --------------------------------------------------
CHICK   --------------------------------------------------
XENTR   --------------------------------------------------
DANRE   --------------------------------------------------
CIOIN   --------------------------------------------------
ANOGA   --------------------------------------------------
TETNG   --------------------------------------------------
DROME   --------------------------------------------------
BRUMA   --------------------------------------------------
CAEEL   --------------------------------------------------
YEAST*  --------------------------------------------------
SCHPO   --------------------------------------------------
CANAL   --------------------------------------------------
CANGA   --------------------------------------------------
ASPNI   --------------------------------------------------
RHIO9   --------------------------------------------------
CRYNE   --------------------------------------------------
USTMA   --------------------------------------------------
ENCCU   --------------------------------------------------
ANTLO   --------------------------------------------------
ARATH*  NNINNQG-MMQVS---GTQFVGRSAASPSGP----NFDN-TTSPLPYSNS
VITVI   SSINATQGLLQAS---GAQLIGRSAASPSGPTGATSFDNTTTSPLPYANS
SOYBN   NTMPAQNSLLQTSS--ASQLLGRSAASPSGATSTTSFDNTTASPIPYANS
SOLLC   SNVNSQGALLQAS---GAL---RAAVSPSGP---TSFDTTTASPLQHVNS
ORYSJ*  NVVNAQGNPMQVS---GGQLLGRPAPSP-GATGTPNFENVSTPPMPYANS
HORVU   NNVNAQGNPMQAS---GGQLLGRSAPSP-GTAGTPNFENVSTPPMPYANS
SORBI   NNVNNQGN--QAS---GGQLLGRPAPSP-GTTGTPNFENVSTPPMPYANS
SELML   QVRHFISVFKCDLTMFSPLKIIANATMNGAPA-------AAAALAGFVNS
PHYPA   QQINSSQVLPRSNIGVSMQALGGAVSLPGG---------TAATTIPYVNS
                 560       570       580       590       600
```

MED8

```
HUMAN*  --------------------------------------------------
CHICK   --------------------------------------------------
XENTR   --------------------------------------------------
DANRE   --------------------------------------------------
CIOIN   --------------------------------------------------
ANOGA   --------------------------------------------------
TETNG   --------------------------------------------------
DROME   --------------------------------------------------
BRUMA   --------------------------------------------------
CAEEL   --------------------------------------------------
YEAST*  --------------------------------------------------
SCHPO   --------------------------------------------------
CANAL   --------------------------------------------------
CANGA   --------------------------------------------------
ASPNI   --------------------------------------------------
RHIO9   --------------------------------------------------
CRYNE   --------------------------------------------------
USTMA   --------------------------------------------------
ENCCU   --------------------------------------------------
ANTLO   --------------------------------------------------
ARATH*  PRA-TGMVNVPSPQHQIQQQQFQQQ----------QQRSKLMQLPQHQQQ
VITVI   PRSGTNMMNTPSPQQQTQHQQQQQQQQQQ----QQQQRQKLMQHMSPHQQ
SOYBN   PRSSTNMMNTPSPQPQTTQQQPQVQQ---------QQRQKLMQLPKQQQQ
SOLLC   PRSSANMMNTPSPQQQTHQQQQQQQQQQQLQQHQLQQRQKMLQLPQHQQ-
ORYSJ*  PRSGTNMMNTPSPQQHLTAQ---------------QHRQKLMQTSQQQQL
HORVU   PRSGTNMMNTPSPQQHLTPQ---------------QQRQKLMQASQQQQ-
SORBI   PRSGTNMMNTPSPQQHLTPQ---------------QQRQKLMQASQQQQL
SELML   PRPG-------DLQAQQQQQQQQQQQ---------QQQQLLMQQRNTKVQ
PHYPA   PRSSTANFNVPSPQQLQNQQQQQAI----------IRQQRIQQLQKQQQQ
                         610       620       630       640       650
```

```
HUMAN*  --------------------------------------------------
CHICK   --------------------------------------------------
XENTR   --------------------------------------------------
DANRE   --------------------------------------------------
CIOIN   --------------------------------------------------
ANOGA   --------------------------------------------------
TETNG   --------------------------------------------------
DROME   --------------------------------------------------
BRUMA   --------------------------------------------------
CAEEL   --------------------------------------------------
YEAST*  --------------------------------------------------
SCHPO   --------------------------------------------------
CANAL   --------------------------------------------------
CANGA   --------------------------------------------------
ASPNI   --------------------------------------------------
RHIO9   --------------------------------------------------
CRYNE   --------------------------------------------------
USTMA   --------------------------------------------------
ENCCU   --------------------------------------------------
ANTLO   --------------------------------------------------
ARATH*  --QLLAQQQQQLRQSSMQGLGQSQIP---------ALHDM----HGQAQQ
VITVI   --QLLA---QQIRQSSMPGLGQNQLP---------QLHDL----QGQAQQ
SOYBN   HHQILA-QQQQFRQSAMQGLGQ----------------------------
SOLLC   --QILA--QQQLRQASMPGLGQ----------------------------
ORYSJ*  HAQ------QQLRPSAAGMLAQSAIP---------QLQDL----QGQSQQ
HORVU   ---------QQLRPSAAGMLAQSSVP---------QLQDL----QGQAQQ
SORBI   HA-------QQLRPSAAGMLAQSALP---------QLQDL----QGQAQQ
SELML   --QLQKHF-HQLRQPA-PALQQMQVA--------NQAQSAKMQQFSQSHL
PHYPA   --ALQAQQGQQLRRPGTPPIPQTQTTLQPQGQHSLQLQQAKLQQQNQLQQ
                         660       670       680       690       700
```

```
HUMAN*  --------------------------------------------------
CHICK   --------------------------------------------------
XENTR   --------------------------------------------------
DANRE   --------------------------------------------------
CIOIN   --------------------------------------------------
ANOGA   --------------------------------------------------
TETNG   --------------------------------------------------
DROME   --------------------------------------------------
BRUMA   --------------------------------------------------
CAEEL   --------------------------------------------------
YEAST*  --------------------------------------------------
SCHPO   --------------------------------------------------
CANAL   --------------------------------------------------
CANGA   --------------------------------------------------
ASPNI   --------------------------------------------------
RHIO9   --------------------------------------------------
CRYNE   --------------------------------------------------
USTMA   --------------------------------------------------
ENCCU   --------------------------------------------------
ANTLO   --------------------------------------------------
ARATH*  KFQTSHGQHQMPYSQPMGAHQQFQAR---QLSGGHIQHSM----------
VITVI   KFQPLPGQQQMQFSQTMG-HQQFQSR---QLPSGHVQHGM----------
SOYBN   ----LHGQHQMQFSQPLG-HQQFQGR---QLPSGHVQHGI----------
SOLLC   ----LHGQPQMQFSQPLG-AQQFQGR---QLPSGALHHGM----------
ORYSJ*  KLQ-VPGQQQMQYNQALS--QQYQNR---QMQAGRMQPGM----------
HORVU   KVQ-VSGQQQMQYSQAQA-LSQFQNR---QMQ-ARMQPGM----------
SORBI   KLQ-VPGQQQMQYNQALS--QQFHNR---QMQPGRMQPGM----------
SELML   QMN----QQQQHAAQ---LHQHMHRDGVPQLQQMHIQQHN--------AQ
PHYPA   QLQ-QQQQQQQQHYQAQQLQAQQQQQ---QQQSLQQQHALQHQLQTTQLH
                         710       720       730       740       750
```

MED8

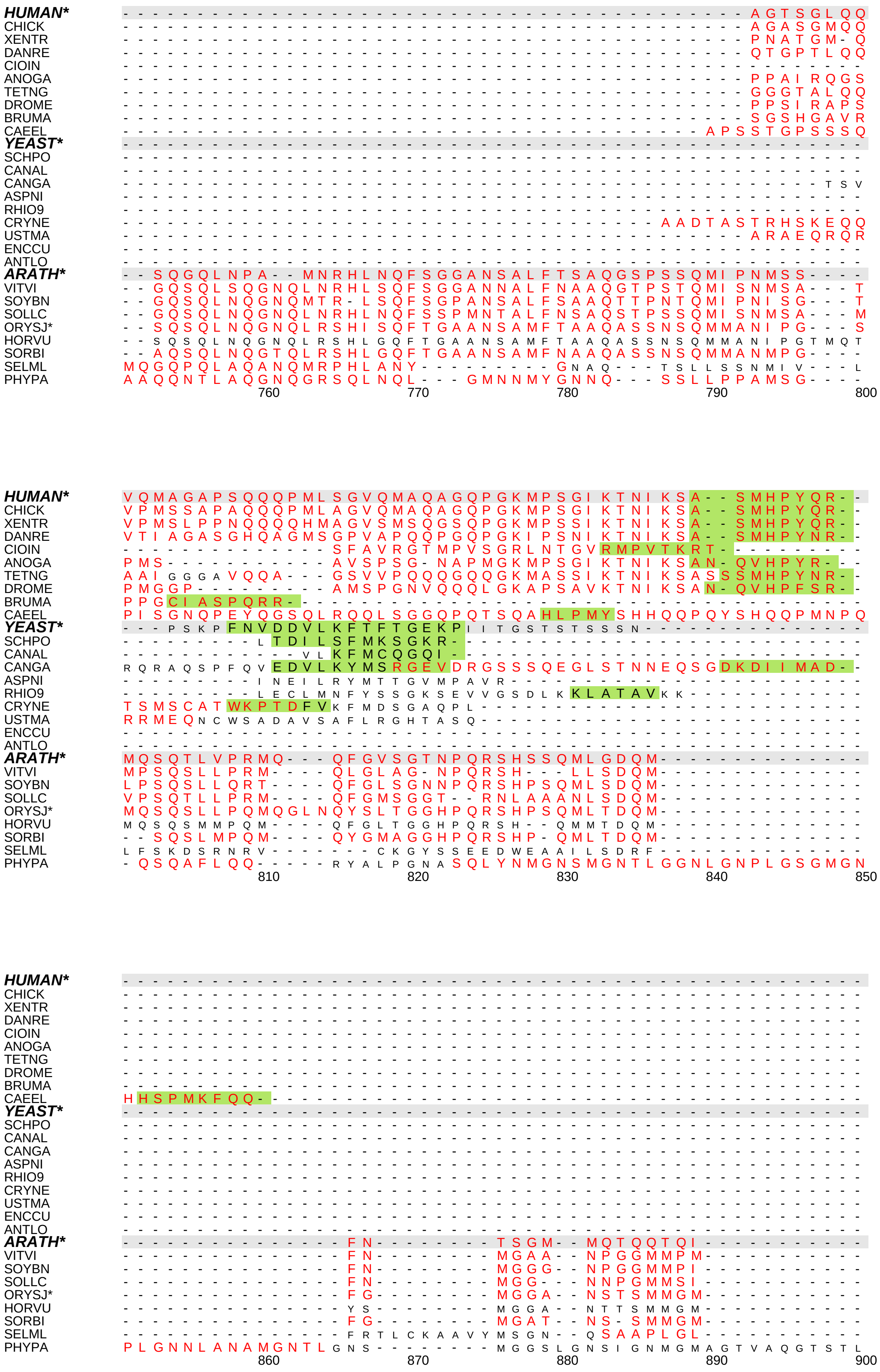

MED8

```
HUMAN*  - - - - - - - - - - - - - - - - - - - - - - - - - - - - - - - - - - - - - - - - - - - - - - - - - -
CHICK   - - - - - - - - - - - - - - - - - - - - - - - - - - - - - - - - - - - - - - - - - - - - - - - - - -
XENTR   - - - - - - - - - - - - - - - - - - - - - - - - - - - - - - - - - - - - - - - - - - - - - - - - - -
DANRE   - - - - - - - - - - - - - - - - - - - - - - - - - - - - - - - - - - - - - - - - - - - - - - - - - -
CIOIN   - - - - - - - - - - - - - - - - - - - - - - - - - - - - - - - - - - - - - - - - - - - - - - - - - -
ANOGA   - - - - - - - - - - - - - - - - - - - - - - - - - - - - - - - - - - - - - - - - - - - - - - - - - -
TETNG   - - - - - - - - - - - - - - - - - - - - - - - - - - - - - - - - - - - - - - - - - - - - - - - - - -
DROME   - - - - - - - - - - - - - - - - - - - - - - - - - - - - - - - - - - - - - - - - - - - - - - - - - -
BRUMA   - - - - - - - - - - - - - - - - - - - - - - - - - - - - - - - - - - - - - - - - - - - - - - - - - -
CAEEL   - - - - - - - - - - - - - - - - - - - - - - - - - - - - - - - - - - - - - - - - - - - - - - - - - -
YEAST*  - - - - - - - - - - - - - - - - - - - - - - - - - - - - - - - - - - - - - - - - - - - - - - - - - -
SCHPO   - - - - - - - - - - - - - - - - - - - - - - - - - - - - - - - - - - - - - - - - - - - - - - - - - -
CANAL   - - - - - - - - - - - - - - - - - - - - - - - - - - - - - - - - - - - - - - - - - - - - - - - - - -
CANGA   - - - - - - - - - - - - - - - - - - - - - - - - - - - - - - - - - - - - - - - - - - - - - - - - - -
ASPNI   - - - - - - - - - - - - - - - - - - - - - - - - - - - - - - - - - - - - - - - - - - - - - - - - - -
RHIO9   - - - - - - - - - - - - - - - - - - - - - - - - - - - - - - - - - - - - - - - - - - - - - - - - - -
CRYNE   - - - - - - - - - - - - - - - - - - - - - - - - - - - - - - - - - - - - - - - - - - - - - - - - - -
USTMA   - - - - - - - - - - - - - - - - - - - - - - - - - - - - - - - - - - - - - - - - - - - - - - - - - -
ENCCU   - - - - - - - - - - - - - - - - - - - - - - - - - - - - - - - - - - - - - - - - - - - - - - - - - -
ANTLO   - - - - - - - - - - - - - - - - - - - - - - - - - - - - - - - - - - - - - - - - - - - - - - - - - -
ARATH*  - - - - - Q Q S Q Q Q Q Q Q Q Q Q G G - - - - - - - - - - - - - - - - - - - - - - - - - - - - - - -
VITVI   - - - - - - - - - - Q Q Q Q Q Q H G S Q G A - - - - - - - - - - - - - - - - - - - - - - - - - - - -
SOYBN   - - - - - - - - - - - Q Q Q Q Q H G S Q A - - - - - - - - - - - - - - - - - - - - - - - - - - - - -
SOLLC   - - - - - - - - - - - - Q Q Q Q H G T - - - - - - - - - - - - - - - - - - - - - - - - - - - - - - -
ORYSJ*  - - - - - - - - - - - Q Q Q Q Q - - - - - - - - - - - - - - - - - - - - - - - - - - - - - - - - - -
HORVU   - - - - - Q M Q Q Q Q Q Q Q Q Q Q G L - - - - - - - - - - - - - - - - - - - - - - - - - - - - - - -
SORBI   - - - - - - - - - - - Q Q Q Q Q Q G V - - - - - - - - - - - - - - - - - - - - - - - - - - - - - - -
SELML   - - P L Q Q Q M P G Q T N - - - - - - - - - - - - - - - - - - - - - - - - - - - - - - - - - - - - -
PHYPA   A L P L Q Q Q L A G Q A N Y S A Q G A R A P C A Q S D Y D L H D S E L N F F H I S V P C A V P V Q F
                            910                 920                 930                 940                 950
```

```
HUMAN*  - - - - - - - - - - - - - - - - - - - - - - - - - - - - - - - - - - - - - - - - - - - - - - - - - -
CHICK   - - - - - - - - - - - - - - - - - - - - - - - - - - - - - - - - - - - - - - - - - - - - - - - - - -
XENTR   - - - - - - - - - - - - - - - - - - - - - - - - - - - - - - - - - - - - - - - - - - - - - - - - - -
DANRE   - - - - - - - - - - - - - - - - - - - - - - - - - - - - - - - - - - - - - - - - - - - - - - - - - -
CIOIN   - - - - - - - - - - - - - - - - - - - - - - - - - - - - - - - - - - - - - - - - - - - - - - - - - -
ANOGA   - - - - - - - - - - - - - - - - - - - - - - - - - - - - - - - - - - - - - - - - - - - - - - - - - -
TETNG   - - - - - - - - - - - - - - - - - - - - - - - - - - - - - - - - - - - - - - - - - - - - - - - - - -
DROME   - - - - - - - - - - - - - - - - - - - - - - - - - - - - - - - - - - - - - - - - - - - - - - - - - -
BRUMA   - - - - - - - - - - - - - - - - - - - - - - - - - - - - - - - - - - - - - - - - - - - - - - - - - -
CAEEL   - - - - - - - - - - - - - - - - - - - - - - - - - - - - - - - - - - - - - - - - - - - - - - - - - -
YEAST*  - - - - - - - - - - - - - - - - - - - - - - - - - - - - - - - - - - - - - - - - - - - - - - - - - -
SCHPO   - - - - - - - - - - - - - - - - - - - - - - - - - - - - - - - - - - - - - - - - - - - - - - - - - -
CANAL   - - - - - - - - - - - - - - - - - - - - - - - - - - - - - - - - - - - - - - - - - - - - - - - - - -
CANGA   - - - - - - - - - - - - - - - - - - - - - - - - - - - - - - - - - - - - - - - - - - - - - - - - - -
ASPNI   - - - - - - - - - - - - - - - - - - - - - - - - - - - - - - - - - - - - - - - - - - - - - - - - - -
RHIO9   - - - - - - - - - - - - - - - - - - - - - - - - - - - - - - - - - - - - - - - - - - - - - - - - - -
CRYNE   - - - - - - - - - - - - - - - - - - - - - - - - - - - - - - - - - - - - - - - - - - - - - - - - - -
USTMA   - - - - - - - - - - - - - - - - - - - - - - - - - - - - - - - - - - - - - - - - - - - - - - - - - -
ENCCU   - - - - - - - - - - - - - - - - - - - - - - - - - - - - - - - - - - - - - - - - - - - - - - - - - -
ANTLO   - - - - - - - - - - - - - - - - - - - - - - - - - - - - - - - - - - - - - - - - - - - - - - - - - -
ARATH*  - - - - - - - - - - - - - - - - - - - - - - - - - - - - - - - - - - - - - - - - - - - - - - - - - -
VITVI   - - - - - - - - - - - - - - - - - - - - - - - - - - - - - - - - - - - - - - - - - - - - - - - - - -
SOYBN   - - - - - - - - - - - - - - - - - - - - - - - - - - - - - - - - - - - - - - - - - - - - - - - - - -
SOLLC   - - - - - - - - - - - - - - - - - - - - - - - - - - - - - - - - - - - - - - - - - - - - - - - - - -
ORYSJ*  - - - - - - - - - - - - - - - - - - - - - - - - - - - - - - - - - - - - - - - - - - - - - - - - - -
HORVU   - - - - - - - - - - - - - - - - - - - - - - - - - - - - - - - - - - - - - - - - - - - - - - - - - -
SORBI   - - - - - - - - - - - - - - - - - - - - - - - - - - - - - - - - - - - - - - - - - - - - - - - - - -
SELML   - - - - - - - - - - - - - - - - - - - - - - - - - - - - - - - - - - - - - - - - - - - - - - - - - -
PHYPA   N N E S R W H L L Q D S N V P L A F N L Y R S F T V V D G R R D F V E E C E D G C D D H G E H G E D
                            960                 970                 980                 990                 1000
```

```
HUMAN*  - - - - - - - - - - - - - - - - - - - - - - - - - - - - - - - - - - - - - - - - - - - - - - - - - -
CHICK   - - - - - - - - - - - - - - - - - - - - - - - - - - - - - - - - - - - - - - - - - - - - - - - - - -
XENTR   - - - - - - - - - - - - - - - - - - - - - - - - - - - - - - - - - - - - - - - - - - - - - - - - - -
DANRE   - - - - - - - - - - - - - - - - - - - - - - - - - - - - - - - - - - - - - - - - - - - - - - - - - -
CIOIN   - - - - - - - - - - - - - - - - - - - - - - - - - - - - - - - - - - - - - - - - - - - - - - - - - -
ANOGA   - - - - - - - - - - - - - - - - - - - - - - - - - - - - - - - - - - - - - - - - - - - - - - - - - -
TETNG   - - - - - - - - - - - - - - - - - - - - - - - - - - - - - - - - - - - - - - - - - - - - - - - - - -
DROME   - - - - - - - - - - - - - - - - - - - - - - - - - - - - - - - - - - - - - - - - - - - - - - - - - -
BRUMA   - - - - - - - - - - - - - - - - - - - - - - - - - - - - - - - - - - - - - - - - - - - - - - - - - -
CAEEL   - - - - - - - - - - - - - - - - - - - - - - - - - - - - - - - - - - - - - - - - - - - - - - - - - -
YEAST*  - - - - - - - - - - - - - - - - - - - - - - - - - - - - - - - - - - - - - - - - - - - - - - - - - -
SCHPO   - - - - - - - - - - - - - - - - - - - - - - - - - - - - - - - - - - - - - - - - - - - - - - - - - -
CANAL   - - - - - - - - - - - - - - - - - - - - - - - - - - - - - - - - - - - - - - - - - - - - - - - - - -
CANGA   - - - - - - - - - - - - - - - - - - - - - - - - - - - - - - - - - - - - - - - - - - - - - - - - - -
ASPNI   - - - - - - - - - - - - - - - - - - - - - - - - - - - - - - - - - - - - - - - - - - - - - - - - - -
RHIO9   - - - - - - - - - - - - - - - - - - - - - - - - - - - - - - - - - - - - - - - - - - - - - - - - - -
CRYNE   - - - - - - - - - - - - - - - - - - - - - - - - - - - - - - - - - - - - - - - - - - - - - - - - - -
USTMA   - - - - - - - - - - - - - - - - - - - - - - - - - - - - - - - - - - - - - - - - - - - - - - - - - -
ENCCU   - - - - - - - - - - - - - - - - - - - - - - - - - - - - - - - - - - - - - - - - - - - - - - - - - -
ANTLO   - - - - - - - - - - - - - - - - - - - - - - - - - - - - - - - - - - - - - - - - - - - - - - - - - -
ARATH*  - - - - - - - - - - - - - - - - - - - - - - - - - - - - - - - - - - - - - - - - - - - - - - - - - -
VITVI   - - - - - - - - - - - - - - - - - - - - - - - - - - - - - - - - - - - - - - - - - - - - - - - - - -
SOYBN   - - - - - - - - - - - - - - - - - - - - - - - - - - - - - - - - - - - - - - - - - - - - - - - - - -
SOLLC   - - - - - - - - - - - - - - - - - - - - - - - - - - - - - - - - - - - - - - - - - - - - - - - - - -
ORYSJ*  - - - - - - - - - - - - - - - - - - - - - - - - - - - - - - - - - - - - - - - - - - - - - - - - - -
HORVU   - - - - - - - - - - - - - - - - - - - - - - - - - - - - - - - - - - - - - - - - - - - - - - - - - -
SORBI   - - - - - - - - - - - - - - - - - - - - - - - - - - - - - - - - - - - - - - - - - - - - - - - - - -
SELML   - - - - - - - - - - - - - - - - - - - - - - - - - - - - - - - - - - - - - - - - - - - - - - - - - -
PHYPA   V D G I L E P S V S V W Q G L V N G L F I K T S K R G C R L L A R F W E C T C A W F G A V C A L L N
                            1010                1020                1030                1040                1050
```

MED8

Species	1051–1100
HUMAN*	--
CHICK	--
XENTR	--
DANRE	--
CIOIN	--
ANOGA	--
TETNG	--
DROME	--
BRUMA	--
CAEEL	--
YEAST*	--
SCHPO	--
CANAL	--
CANGA	--
ASPNI	--
RHIO9	--
CRYNE	--
USTMA	--
ENCCU	--
ANTLO	--
ARATH*	--
VITVI	--
SOYBN	--
SOLLC	--
ORYSJ*	--
HORVU	--
SORBI	--
SELML	--
PHYPA	LLPYSNSSSLAHSRCLLICHVRVNLCLSGCGRYVEPRMRNHCKAQCNTPE
	1060 1070 1080 1090 1100

Species	1101–1150
HUMAN*	--
CHICK	--
XENTR	--
DANRE	--
CIOIN	--
ANOGA	--
TETNG	--
DROME	--
BRUMA	--
CAEEL	--
YEAST*	--
SCHPO	--
CANAL	--
CANGA	--
ASPNI	--
RHIO9	--
CRYNE	--
USTMA	--
ENCCU	--
ANTLO	--
ARATH*	--
VITVI	--
SOYBN	--
SOLLC	--
ORYSJ*	--
HORVU	--
SORBI	--
SELML	--
PHYPA	IDFLSRQGISSMDRITHRHLSSCSQMPKFMPIPAAKRIHSFILLSSIKCC
	1110 1120 1130 1140 1150

Species	1151–1200
HUMAN*	--
CHICK	--
XENTR	--
DANRE	--
CIOIN	--
ANOGA	--
TETNG	--
DROME	--
BRUMA	--
CAEEL	--
YEAST*	--
SCHPO	--
CANAL	--
CANGA	--
ASPNI	--
RHIO9	--
CRYNE	--
USTMA	--
ENCCU	--
ANTLO	--
ARATH*	--------------------------YGNMQTN-------------QQSL
VITVI	--------------------------FGNMQPN-------------AQNL
SOYBN	--------------------------FGSMASN-------------AQNL
SOLLC	--------------------------FGNMSQN-------------TQNL
ORYSJ*	---------------------------FNMQAN-------------AQNL
HORVU	--------------------------YGNMQGG-------------GQSL
SORBI	--------------------------YGNMQAG-------------AQNM
SELML	--------------------------YTLMNPNQNPLLSPSPNTI-HNSL
PHYPA	LRTEKASHLKKVRLSSSSASWSYFEVYGDVQGHLSPLDETVCRTPYEQTL
	1160 1170 1180 1190 1200

MED8

Species	1201–1250
HUMAN*	--
CHICK	--
XENTR	--
DANRE	--
CIOIN	--
ANOGA	--
TETNG	--
DROME	--
BRUMA	--
CAEEL	--
YEAST*	--
SCHPO	--
CANAL	--
CANGA	--
ASPNI	--
RHIO9	--
CRYNE	--
USTMA	--
ENCCU	--
ANTLO	--
ARATH*	QPNNMM--
VITVI	-QPGM---
SOYBN	-QSGL---
SOLLC	-QQGM---
ORYSJ*	-QQGM---
HORVU	QQQGM---
SORBI	-QQGM---
SELML	LNQGLMNPNA--
PHYPA	HREGTSRVLAAWKKIQSWSPTKSRIEEKIRDRPIPSQKCAARSDRAREQS

1210 1220 1230 1240 1250

Species	1251–1300
HUMAN*	--
CHICK	--
XENTR	--
DANRE	--
CIOIN	--
ANOGA	--
TETNG	--
DROME	--
BRUMA	--
CAEEL	--
YEAST*	--
SCHPO	--
CANAL	--
CANGA	--
ASPNI	--
RHIO9	--
CRYNE	--
USTMA	--
ENCCU	--
ANTLO	--
ARATH*	--
VITVI	----------------VALQ-------------------------NATQN
SOYBN	----------------VTLQ-------------------------NTQQN
SOLLC	----------------MPLQ-------------------------NAPQT
ORYSJ*	----------------TGLQ-------------------------NQTQ-
HORVU	----------------VGLQ-------------------------NQQQQ
SORBI	----------------VGLQ-------------------------NQTQ-
SELML	----------------LAFR-------------------------QQQQQ
PHYPA	ATMYNRKGSGPRSSWYTPLQAEAYIGQSRNFHSSRAISQPPTKKMNTKRQ

1260 1270 1280 1290 1300

Species	1301–1350
HUMAN*	--
CHICK	--
XENTR	--
DANRE	--
CIOIN	--
ANOGA	--
TETNG	--
DROME	--
BRUMA	--
CAEEL	--
YEAST*	--
SCHPO	--
CANAL	--
CANGA	--
ASPNI	--
RHIO9	--
CRYNE	--
USTMA	--
ENCCU	--
ANTLO	--
ARATH*	---QNAQQ
VITVI	--------------------------HP-----------------NFQQQ
SOYBN	--------------------------HP-----------------NFSQQ
SOLLC	--------------------------HP-----------------SFQQQ
ORYSJ*	--------------------------NP-----------------NFPQQ
HORVU	GMVGLQNQQQNQMQNQMQNQLQNQMPNP-----------------NFSQQ
SORBI	--------------------------NP-----------------NFTQQ
SELML	---QQQQQ
PHYPA	LAVAAITQALTPSL------------HPQKRGQGELGLCMANFLKSFTSP

1310 1320 1330 1340 1350

MED8

```
HUMAN*  --------------------------------------------------
CHICK   --------------------------------------------------
XENTR   --------------------------------------------------
DANRE   --------------------------------------------------
CIOIN   --------------------------------------------------
ANOGA   --------------------------------------------------
TETNG   --------------------------------------------------
DROME   --------------------------------------------------
BRUMA   --------------------------------------------------
CAEEL   --------------------------------------------------
YEAST*  --------------------------------------------------
SCHPO   --------------------------------------------------
CANAL   --------------------------------------------------
CANGA   --------------------------------------------------
ASPNI   --------------------------------------------------
RHIO9   --------------------------------------------------
CRYNE   --------------------------------------------------
USTMA   --------------------------------------------------
ENCCU   --------------------------------------------------
ANTLO   --------------------------------------------------
ARATH*  RHQNPQ--------------------------------------------
VITVI   RQQNQQ--------------------------------------------
SOYBN   RQQNLQ--------------------------------------------
SOLLC   RPQGQQ--------------------------------------------
ORYSJ*  RQQNQQ--------------------------------------------
HORVU   RQQNQQ--------------------------------------------
SORBI   RQQNQQ--------------------------------------------
SELML   QQQQQQQHQHQQQQQHPPPNQ-----------------------------
PHYPA   AQTNETRFCEDRVRQSLEIMLKITDAAEREVKAIQSWINDAENHLHSLPS
                 1360      1370      1380      1390      1400
```

```
HUMAN*  --------------------------------------------------
CHICK   --------------------------------------------------
XENTR   --------------------------------------------------
DANRE   --------------------------------------------------
CIOIN   --------------------------------------------------
ANOGA   --------------------------------------------------
TETNG   --------------------------------------------------
DROME   --------------------------------------------------
BRUMA   --------------------------------------------------
CAEEL   --------------------------------------------------
YEAST*  --------------------------------------------------
SCHPO   --------------------------------------------------
CANAL   --------------------------------------------------
CANGA   --------------------------------------------------
ASPNI   --------------------------------------------------
RHIO9   --------------------------------------------------
CRYNE   --------------------------------------------------
USTMA   --------------------------------------------------
ENCCU   --------------------------------------------------
ANTLO   --------------------------------------------------
ARATH*  --------------------------------------------------
VITVI   --------------------------------------------------
SOYBN   --------------------------------------------------
SOLLC   --------------------------------------------------
ORYSJ*  --------------------------------------------------
HORVU   --------------------------------------------------
SORBI   --------------------------------------------------
SELML   --------------------------------------------------
PHYPA   ERDIERLEALLDNLLCHSEEGLARDKRDQFLRVASNMKYILANMHTVAEI
                 1410      1420      1430      1440      1450
```

```
HUMAN*  --------------------------------------------------
CHICK   --------------------------------------------------
XENTR   --------------------------------------------------
DANRE   --------------------------------------------------
CIOIN   --------------------------------------------------
ANOGA   --------------------------------------------------
TETNG   --------------------------------------------------
DROME   --------------------------------------------------
BRUMA   --------------------------------------------------
CAEEL   --------------------------------------------------
YEAST*  --------------------------------------------------
SCHPO   --------------------------------------------------
CANAL   --------------------------------------------------
CANGA   --------------------------------------------------
ASPNI   --------------------------------------------------
RHIO9   --------------------------------------------------
CRYNE   --------------------------------------------------
USTMA   --------------------------------------------------
ENCCU   --------------------------------------------------
ANTLO   --------------------------------------------------
ARATH*  --------------------------------------------------
VITVI   --------------------------------------------------
SOYBN   --------------------------------------------------
SOLLC   --------------------------------------------------
ORYSJ*  --------------------------------------------------
HORVU   --------------------------------------------------
SORBI   --------------------------------------------------
SELML   --------------------------------------------------
PHYPA   QELQKELEAIDKEISRCNNSSKEAVSLLRSAIARGEVAWLRNLESVAACL
                 1460      1470      1480      1490      1500
```

HUMAN*	-	-	-	-	-	-	-	-	-	-	-	-	-	-	-	-	-	-
CHICK	-	-	-	-	-	-	-	-	-	-	-	-	-	-	-	-	-	-
XENTR	-	-	-	-	-	-	-	-	-	-	-	-	-	-	-	-	-	-
DANRE	-	-	-	-	-	-	-	-	-	-	-	-	-	-	-	-	-	-
CIOIN	-	-	-	-	-	-	-	-	-	-	-	-	-	-	-	-	-	-
ANOGA	-	-	-	-	-	-	-	-	-	-	-	-	-	-	-	-	-	-
TETNG	-	-	-	-	-	-	-	-	-	-	-	-	-	-	-	-	-	-
DROME	-	-	-	-	-	-	-	-	-	-	-	-	-	-	-	-	-	-
BRUMA	-	-	-	-	-	-	-	-	-	-	-	-	-	-	-	-	-	-
CAEEL	-	-	-	-	-	-	-	-	-	-	-	-	-	-	-	-	-	-
YEAST*	-	-	-	-	-	-	-	-	-	-	-	-	-	-	-	-	-	-
SCHPO	-	-	-	-	-	-	-	-	-	-	-	-	-	-	-	-	-	-
CANAL	-	-	-	-	-	-	-	-	-	-	-	-	-	-	-	-	-	-
CANGA	-	-	-	-	-	-	-	-	-	-	-	-	-	-	-	-	-	-
ASPNI	-	-	-	-	-	-	-	-	-	-	-	-	-	-	-	-	-	-
RHIO9	-	-	-	-	-	-	-	-	-	-	-	-	-	-	-	-	-	-
CRYNE	-	-	-	-	-	-	-	-	-	-	-	-	-	-	-	-	-	-
USTMA	-	-	-	-	-	-	-	-	-	-	-	-	-	-	-	-	-	-
ENCCU	-	-	-	-	-	-	-	-	-	-	-	-	-	-	-	-	-	-
ANTLO	-	-	-	-	-	-	-	-	-	-	-	-	-	-	-	-	-	-
ARATH*	-	-	-	-	-	-	-	-	-	-	-	-	-	-	-	-	-	-
VITVI	-	-	-	-	-	-	-	-	-	-	-	-	-	-	-	-	-	-
SOYBN	-	-	-	-	-	-	-	-	-	-	-	-	-	-	-	-	-	-
SOLLC	-	-	-	-	-	-	-	-	-	-	-	-	-	-	-	-	-	-
ORYSJ*	-	-	-	-	-	-	-	-	-	-	-	-	-	-	-	-	-	-
HORVU	-	-	-	-	-	-	-	-	-	-	-	-	-	-	-	-	-	-
SORBI	-	-	-	-	-	-	-	-	-	-	-	-	-	-	-	-	-	-
SELML	-	-	-	-	-	-	-	-	-	-	-	-	-	-	-	-	-	-
PHYPA	E	A	M	A	R	T	V	L	P	L	E	G	L	Y	K	T	N	Q
											1510							

MED9

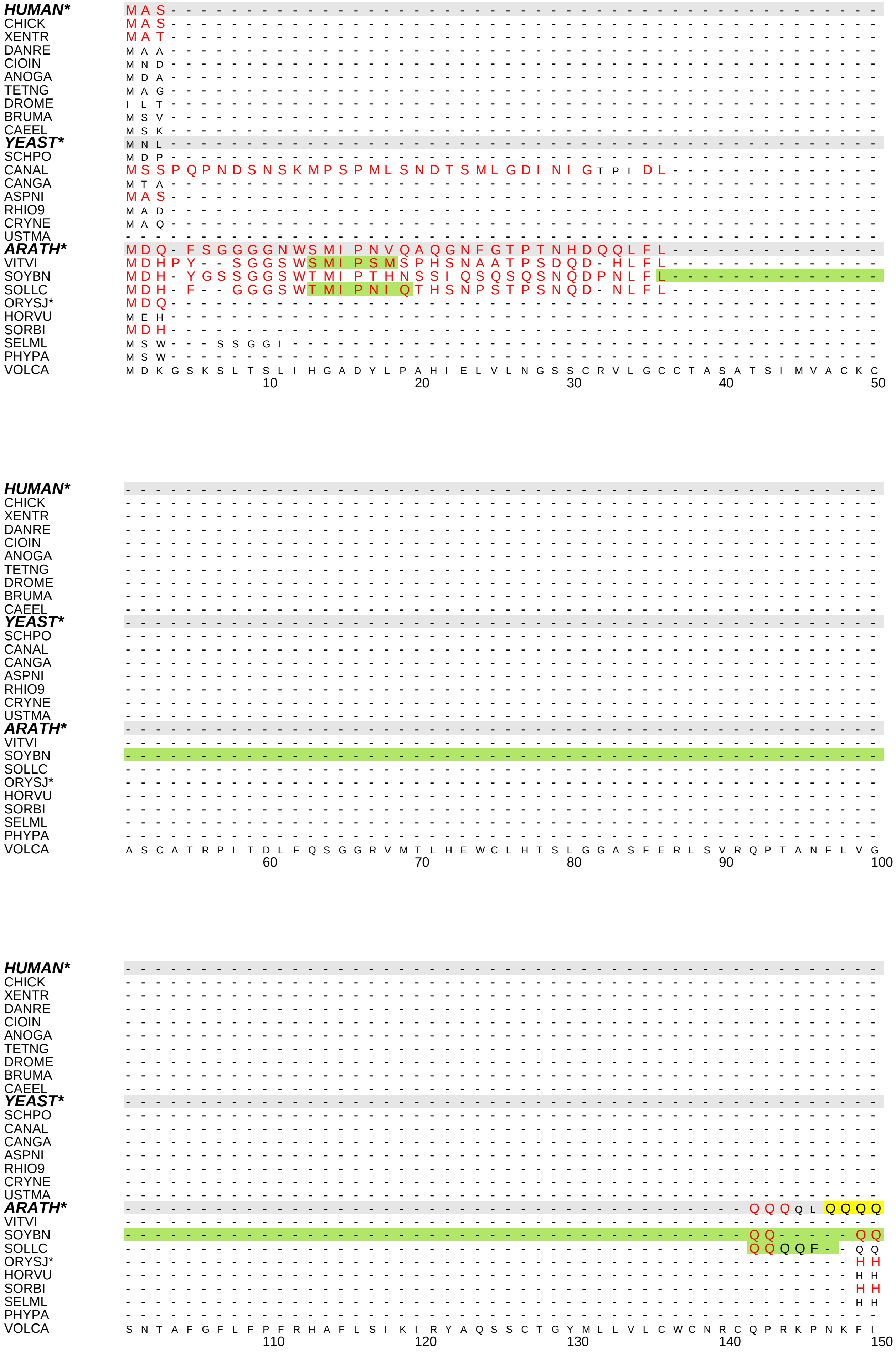

HUMAN* MAS
CHICK MAS
XENTR MAT
DANRE MAA
CIOIN MND
ANOGA MDA
TETNG MAG
DROME ILT
BRUMA MSV
CAEEL MSK
YEAST* MNL
SCHPO MDP
CANAL MSSPQPNDSNSKMPSPMLSNDTSMLGDINIGTPIDL
CANGA MTA
ASPNI MAS
RHIO9 MAD
CRYNE MAQ
USTMA
ARATH* MDQ-FSGGGGNWSMIPNVQAQGNFGTPTNHDQQLFL
VITVI MDHPY--SGGSWSMIPSMSPHSNAATPSDQD-HLFL
SOYBN MDH-YGSSGGSWTMIPTHNSSIQSQSQSNQDPNLFL
SOLLC MDH-F--GGGSWTMIPNIQTHSNPSTPSNQD-NLFL
ORYSJ* MDQ
HORVU MEH
SORBI MDH
SELML MSW---SSGGI
PHYPA MSW
VOLCA MDKGSKSLTSLIHGADYLPAHIELVLNGSSCRVLGCCTASATSIMVACKC
10 20 30 40 50
VOLCA ASCATRPITDLFQSGGRVMTLHEWCLHTSLGGASFERLSVRQPTANFLVG
60 70 80 90 100
ARATH* QQQQLQQQQ
SOYBN QQ--QQ
SOLLC QQQQF--QQ
ORYSJ* HH
HORVU HH
SORBI HH
SELML HH
VOLCA SNTAFGFLFPFRHAFLSIKIRYAQSSCTGYMLLVLCWCNRCQPRKPNKFI
110 120 130 140 150

MED9

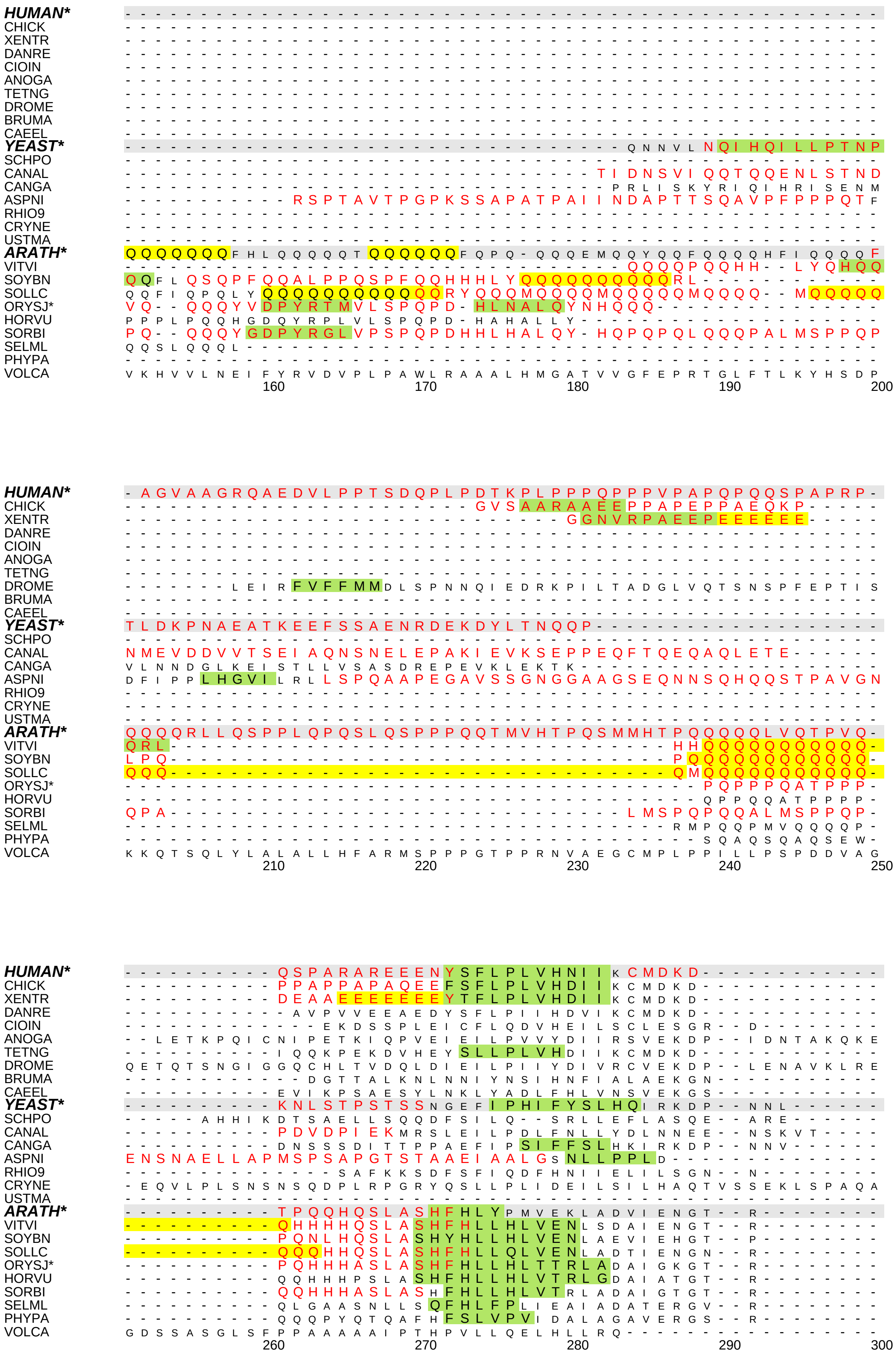
HUMAN*
CHICK
XENTR
DANRE
CIOIN
ANOGA
TETNG
DROME
BRUMA
CAEEL
YEAST*
SCHPO
CANAL
CANGA
ASPNI
RHIO9
CRYNE
USTMA
ARATH*
VITVI
SOYBN
SOLLC
ORYSJ*
HORVU
SORBI
SELML
PHYPA
VOLCA
160
170
180
190
200
210
220
230
240
250
260
270
280
290
300

MED9

```
HUMAN*   SPEVHQDLNALKSKFQEMRKLISTMPG----IHLSPEQQQQQLQ----SL
CHICK    SQDVHQVLNELKNKFQEMRKLISAMPG----IGVSPEQQQQQLQ----NL
XENTR    SQDVYQELNELKSKFQAMRKLVGNMPG----IDLSPEEQQRHLQ----SL
DANRE    SPDVHQELNKLKTKIQEAREQISSMPG----IDMSPAVQESKLQ----TL
CIOIN    VENLSQKVTNLKEKMEQARQEINQAKG----IDNTLPQQTVLLK----SL
ANOGA    SAECSHKVTELQKTLEAARSTIRKLHG----IEYSKEEQLRRLE----SL
TETNG    SQDVHQELAKLKVKIQEAREQISNMPG----IESSPLAQQQQLA----TL
DROME    SQDCNHKIFELQKRFESAREQIRQLPG----IDFNKEEQQQRLE----LL
BRUMA    SSDIALKLRHLEASLEQLKEAIDSTSD----IIGNENYQRARIA----DL
CAEEL    GSELTVRLRNVEADIASFKDALKSIPD----IGVEEYKERRKIA----AL
YEAST*   SNQLETLTGSIRHRLKLCKSLISENEDTKDLLSKSPSEWQDIIH----QR
SCHPO    TVTASKELTLLRQGIRQLKEKVSKMEP----EEMTVKEKKSIIE----IL
CANAL    PKDFDKYLGSLRLKLSNLKNLMLEVEG----INETLSYTLDKIE----SL
CANGA    SSQLETSTGFIRHRIKRCKALLQENEEVRNLLANSIEEWENIIA----DK
ASPNI    VKNLPTEVSSIKIRIQKAQAVVEGLPD----VDRSVAEQEKEIR----DL
RHIO9    QDSIGKAVAHLEERFVHARQVLEELPG----LHYAKEEQERLYQ----QE
CRYNE    SEQVAPKAKQLAQVLENMKLSAMSLPG----AHLSIEEINNLTD----IL
USTMA    -------MIALRIRFGSIRKVLCLHDRLVPRLLHLVELGWQNVE----RV
ARATH*   DQNSDALVNELNSHFDKCQQLLNSISGSLGSKTMTVDGQKRNVE----ES
VITVI    DQHSDALVRELNSHFEKCQQLLNSISVSINTKAMTVEGQKRKLE----ES
SOYBN    DQQSDALINELSNHFEKCQQLLNSISDSISTKAMTVEGQKKKLE----ES
SOLLC    DQHSDALVTELKNQFEKCQQLLTSISGSISSRSMTVEGQKRKKA----EC
ORYSJ*   DQNSDALVEDLTSQFARCQQLLNSISGTLSSKSITVEGQRKSLE----ET
HORVU    DQAFDALVEELTSQFARSQQLLNSISGTLSSKSVTVEGQMQSLE----ET
SORBI    DQNSDALVEELTSQFARCQQLLNSISGTISSKSTTVEGQRQSLD----ET
SELML    DQRVDDLVNELGNRFERCQQLLAPHAA--SSKPLTLRNQKMKLQ----EY
PHYPA    DQH-DELIRELVARFQKAEQILHSLES--NSIEKDVATQKRTLD----DH
VOLCA    QQLLQQQLQKQQLQLQELQQRLQQAPHANMLTSLQQDMQQQRLEPQPQQL
                310       320       330       340       350
```

```
HUMAN*   REQVRTKNELLQKYKSLCMFEIPKE-------------------------
CHICK    REQVRTKNELLQKYKSLCMFEIPKE-------------------------
XENTR    REQVQTKNELLQKYKSLCMFEIPKE-------------------------
DANRE    REQVQTKNQLLQKYKSLCMFDIPKPS------------------------
CIOIN    QDQLKIKCDIVEKYRNFSTKPNSDNLMQLQYPSYEG--------------
ANOGA    RKQLALKQQLIKKYKNVQF-------------------------------
TETNG    REQVRTKNQLLQKYKSLCMFDVPK--------------------------
DROME    RNQLKLKQQLIRKYKDTEF-------------------------------
BRUMA    NRRITLKDGLINSFRNGQCSFGT---------------------------
CAEEL    YKQIEKKDELLDELAAFSLDAALKKPDGEQKQRSESGDK-----------
YEAST*   EQELQIKRDVLDDLYRKLQR------------------------------
SCHPO    KARIALKKAFLKMALSESNDTVVKKEDVRESPVDTFMENAT---------
CANAL    KQNNIKKEAFLSNFKNSVNMNEEI--------------------------
CANGA    EQQLRVKAKVLRDLDARIEKITN---------------------------
ASPNI    EDKIARLKSVIFDLGRKADKARSERGANTSGP------------------
RHIO9    LDLLEHKKKQLETYLSLPPFKKQQEQ------------------------
CRYNE    EKEGEKRRQILRTFELLDLPIVDDFSLPGAETRAMGTHAFPSPNK-----
USTMA    FNRQQYRVLLWSNWDFRMAVIRPRQTDVLTPGADLFLLLEHPVQLLLDFF
ARATH*   EQLLQQRRDLIVEYRKSIEEIVTMEH------------------------
VITVI    EQLLNQRRDLIAKYRNSVEDLIKSEP------------------------
SOYBN    EQLLNQRRDLIANYRKSVEDLVRSEP------------------------
SOLLC    EQMLNQRRDLISRYKGSVEEIINSEL------------------------
ORYSJ*   QQLLDQRKDLITKYRSSVEDLLKGDTRR----------------------
HORVU    RQLLDQRKDLMAKYKTSVEDLLKGDPTR----------------------
SORBI    RQLLDQRKELITKYRSSVEDLLKGDKR-----------------------
SELML    ENQLNLRREALSKYREMVE-------------------------------
PHYPA    RNKLEERRAVLAKYKSMVERIYPADN------------------------
VOLCA    RQQLQLRQQLQQQYLKQQQQQQQQQPPQEVSGGPTLTHDLARKRKPAGGG
                360       370       380       390       400
```

```
HUMAN*   --------------------------------------------------
CHICK    --------------------------------------------------
XENTR    --------------------------------------------------
DANRE    --------------------------------------------------
CIOIN    --------------------------------------------------
ANOGA    --------------------------------------------------
TETNG    --------------------------------------------------
DROME    --------------------------------------------------
BRUMA    --------------------------------------------------
CAEEL    --------------------------------------------------
YEAST*   --------------------------------------------------
SCHPO    --------------------------------------------------
CANAL    --------------------------------------------------
CANGA    --------------------------------------------------
ASPNI    --------------------------------------------------
RHIO9    --------------------------------------------------
CRYNE    --------------------------------------------------
USTMA    RAFFKAFDFVFRYRSCILTELRLGRRYARWIGPGLGAWGIVLGTVLLGLG
ARATH*   --------------------------------------------------
VITVI    --------------------------------------------------
SOYBN    --------------------------------------------------
SOLLC    --------------------------------------------------
ORYSJ*   --------------------------------------------------
HORVU    --------------------------------------------------
SORBI    --------------------------------------------------
SELML    --------------------------------------------------
PHYPA    --------------------------------------------------
VOLCA    VGGYDGGEDGGASSPVHPFSRVSSPISGVFGSGAASPVGLPSAAAVAAPS
                410       420       430       440       450
```

MED9

```
HUMAN*   --------------------------------------------------
CHICK    --------------------------------------------------
XENTR    --------------------------------------------------
DANRE    --------------------------------------------------
CIOIN    --------------------------------------------------
ANOGA    --------------------------------------------------
TETNG    --------------------------------------------------
DROME    --------------------------------------------------
BRUMA    --------------------------------------------------
CAEEL    --------------------------------------------------
YEAST*   --------------------------------------------------
SCHPO    --------------------------------------------------
CANAL    --------------------------------------------------
CANGA    --------------------------------------------------
ASPNI    --------------------------------------------------
RHIO9    --------------------------------------------------
CRYNE    --------------------------------------------------
USTMA    RFAGSLIVSVQLGR------------------------------------
ARATH*   --------------------------------------------------
VITVI    --------------------------------------------------
SOYBN    --------------------------------------------------
SOLLC    --------------------------------------------------
ORYSJ*   --------------------------------------------------
HORVU    --------------------------------------------------
SORBI    --------------------------------------------------
SELML    --------------------------------------------------
PHYPA    --------------------------------------------------
VOLCA    DAAPAIGGGNGNVCNHLGAIGCAINPTTAPASRQLLSAFPHRHHHLQHEA
                  460       470       480       490       500
```

```
HUMAN*   --------------------------------------------------
CHICK    --------------------------------------------------
XENTR    --------------------------------------------------
DANRE    --------------------------------------------------
CIOIN    --------------------------------------------------
ANOGA    --------------------------------------------------
TETNG    --------------------------------------------------
DROME    --------------------------------------------------
BRUMA    --------------------------------------------------
CAEEL    --------------------------------------------------
YEAST*   --------------------------------------------------
SCHPO    --------------------------------------------------
CANAL    --------------------------------------------------
CANGA    --------------------------------------------------
ASPNI    --------------------------------------------------
RHIO9    --------------------------------------------------
CRYNE    --------------------------------------------------
USTMA    --------------------------------------------------
ARATH*   --------------------------------------------------
VITVI    --------------------------------------------------
SOYBN    --------------------------------------------------
SOLLC    --------------------------------------------------
ORYSJ*   --------------------------------------------------
HORVU    --------------------------------------------------
SORBI    --------------------------------------------------
SELML    --------------------------------------------------
PHYPA    --------------------------------------------------
VOLCA    IPEAPAAYGGSGGGSSSSSTSTGSSGSGSGSGGMQRGFVPAVSRFGSAAD
                  510       520       530       540       550
```

```
HUMAN*   --------------------------------------------------
CHICK    --------------------------------------------------
XENTR    --------------------------------------------------
DANRE    --------------------------------------------------
CIOIN    --------------------------------------------------
ANOGA    --------------------------------------------------
TETNG    --------------------------------------------------
DROME    --------------------------------------------------
BRUMA    --------------------------------------------------
CAEEL    --------------------------------------------------
YEAST*   --------------------------------------------------
SCHPO    --------------------------------------------------
CANAL    --------------------------------------------------
CANGA    --------------------------------------------------
ASPNI    --------------------------------------------------
RHIO9    --------------------------------------------------
CRYNE    --------------------------------------------------
USTMA    --------------------------------------------------
ARATH*   --------------------------------------------------
VITVI    --------------------------------------------------
SOYBN    --------------------------------------------------
SOLLC    --------------------------------------------------
ORYSJ*   --------------------------------------------------
HORVU    --------------------------------------------------
SORBI    --------------------------------------------------
SELML    --------------------------------------------------
PHYPA    --------------------------------------------------
VOLCA    LPGSASPWPPLSLQPPDAKPTASESAAAAAGPAPAAAAASHKGKAPAPAP
                  560       570       580       590       600
```

MED9

```
HUMAN*  --------------------------------------------------
CHICK   --------------------------------------------------
XENTR   --------------------------------------------------
DANRE   --------------------------------------------------
CIOIN   --------------------------------------------------
ANOGA   --------------------------------------------------
TETNG   --------------------------------------------------
DROME   --------------------------------------------------
BRUMA   --------------------------------------------------
CAEEL   --------------------------------------------------
YEAST*  --------------------------------------------------
SCHPO   --------------------------------------------------
CANAL   --------------------------------------------------
CANGA   --------------------------------------------------
ASPNI   --------------------------------------------------
RHIO9   --------------------------------------------------
CRYNE   --------------------------------------------------
USTMA   --------------------------------------------------
ARATH*  --------------------------------------------------
VITVI   --------------------------------------------------
SOYBN   --------------------------------------------------
SOLLC   --------------------------------------------------
ORYSJ*  --------------------------------------------------
HORVU   --------------------------------------------------
SORBI   --------------------------------------------------
SELML   --------------------------------------------------
PHYPA   --------------------------------------------------
VOLCA   APVQQQQQQPPPQRAQSTSVLSGHAAAVDTSWTLGGVGDVNGGGWHGAGG
                 610       620       630       640       650
```

```
HUMAN*  --------------------------------------------------
CHICK   --------------------------------------------------
XENTR   --------------------------------------------------
DANRE   --------------------------------------------------
CIOIN   --------------------------------------------------
ANOGA   --------------------------------------------------
TETNG   --------------------------------------------------
DROME   --------------------------------------------------
BRUMA   --------------------------------------------------
CAEEL   --------------------------------------------------
YEAST*  --------------------------------------------------
SCHPO   --------------------------------------------------
CANAL   --------------------------------------------------
CANGA   --------------------------------------------------
ASPNI   --------------------------------------------------
RHIO9   --------------------------------------------------
CRYNE   --------------------------------------------------
USTMA   --------------------------------------------------
ARATH*  --------------------------------------------------
VITVI   --------------------------------------------------
SOYBN   --------------------------------------------------
SOLLC   --------------------------------------------------
ORYSJ*  --------------------------------------------------
HORVU   --------------------------------------------------
SORBI   --------------------------------------------------
SELML   --------------------------------------------------
PHYPA   --------------------------------------------------
VOLCA   GPSILDADFEAMFQALEAAAWSSPTLSACGGGGASTAAATATAAGGAAIG
                 660       670       680       690       700
```

```
HUMAN*  --------------------------------------------------
CHICK   --------------------------------------------------
XENTR   --------------------------------------------------
DANRE   --------------------------------------------------
CIOIN   --------------------------------------------------
ANOGA   --------------------------------------------------
TETNG   --------------------------------------------------
DROME   --------------------------------------------------
BRUMA   --------------------------------------------------
CAEEL   --------------------------------------------------
YEAST*  --------------------------------------------------
SCHPO   --------------------------------------------------
CANAL   --------------------------------------------------
CANGA   --------------------------------------------------
ASPNI   --------------------------------------------------
RHIO9   --------------------------------------------------
CRYNE   --------------------------------------------------
USTMA   --------------------------------------------------
ARATH*  --------------------------------------------------
VITVI   --------------------------------------------------
SOYBN   --------------------------------------------------
SOLLC   --------------------------------------------------
ORYSJ*  --------------------------------------------------
HORVU   --------------------------------------------------
SORBI   --------------------------------------------------
SELML   --------------------------------------------------
PHYPA   --------------------------------------------------
VOLCA   SVQPLTTTLFASSSSDAGGPLPHAVQMPLQSQSQRQQPGSMKSLLEQQQQ
                 710       720       730       740       750
```

MED9

```
HUMAN*  - - - - - - - - - - - - - - - - - - - - - - - - - - - - - - - - - - - - - - - - - - - - - - - - - -
CHICK   - - - - - - - - - - - - - - - - - - - - - - - - - - - - - - - - - - - - - - - - - - - - - - - - - -
XENTR   - - - - - - - - - - - - - - - - - - - - - - - - - - - - - - - - - - - - - - - - - - - - - - - - - -
DANRE   - - - - - - - - - - - - - - - - - - - - - - - - - - - - - - - - - - - - - - - - - - - - - - - - - -
CIOIN   - - - - - - - - - - - - - - - - - - - - - - - - - - - - - - - - - - - - - - - - - - - - - - - - - -
ANOGA   - - - - - - - - - - - - - - - - - - - - - - - - - - - - - - - - - - - - - - - - - - - - - - - - - -
TETNG   - - - - - - - - - - - - - - - - - - - - - - - - - - - - - - - - - - - - - - - - - - - - - - - - - -
DROME   - - - - - - - - - - - - - - - - - - - - - - - - - - - - - - - - - - - - - - - - - - - - - - - - - -
BRUMA   - - - - - - - - - - - - - - - - - - - - - - - - - - - - - - - - - - - - - - - - - - - - - - - - - -
CAEEL   - - - - - - - - - - - - - - - - - - - - - - - - - - - - - - - - - - - - - - - - - - - - - - - - - -
YEAST*  - - - - - - - - - - - - - - - - - - - - - - - - - - - - - - - - - - - - - - - - - - - - - - - - - -
SCHPO   - - - - - - - - - - - - - - - - - - - - - - - - - - - - - - - - - - - - - - - - - - - - - - - - - -
CANAL   - - - - - - - - - - - - - - - - - - - - - - - - - - - - - - - - - - - - - - - - - - - - - - - - - -
CANGA   - - - - - - - - - - - - - - - - - - - - - - - - - - - - - - - - - - - - - - - - - - - - - - - - - -
ASPNI   - - - - - - - - - - - - - - - - - - - - - - - - - - - - - - - - - - - - - - - - - - - - - - - - - -
RHIO9   - - - - - - - - - - - - - - - - - - - - - - - - - - - - - - - - - - - - - - - - - - - - - - - - - -
CRYNE   - - - - - - - - - - - - - - - - - - - - - - - - - - - - - - - - - - - - - - - - - - - - - - - - - -
USTMA   - - - - - - - - - - - - - - - - - - - - - - - - - - - - - - - - - - - - - - - - - - - - - - - - - -
ARATH*  - - - - - - - - - - - - - - - - - - - - - - - - - - - - - - - - - - - - - - - - - - - - - - - - - -
VITVI   - - - - - - - - - - - - - - - - - - - - - - - - - - - - - - - - - - - - - - - - - - - - - - - - - -
SOYBN   - - - - - - - - - - - - - - - - - - - - - - - - - - - - - - - - - - - - - - - - - - - - - - - - - -
SOLLC   - - - - - - - - - - - - - - - - - - - - - - - - - - - - - - - - - - - - - - - - - - - - - - - - - -
ORYSJ*  - - - - - - - - - - - - - - - - - - - - - - - - - - - - - - - - - - - - - - - - - - - - - - - - - -
HORVU   - - - - - - - - - - - - - - - - - - - - - - - - - - - - - - - - - - - - - - - - - - - - - - - - - -
SORBI   - - - - - - - - - - - - - - - - - - - - - - - - - - - - - - - - - - - - - - - - - - - - - - - - - -
SELML   - - - - - - - - - - - - - - - - - - - - - - - - - - - - - - - - - - - - - - - - - - - - - - - - - -
PHYPA   - - - - - - - - - - - - - - - - - - - - - - - - - - - - - - - - - - - - - - - - - - - - - - - - - -
VOLCA   P P Q P Q A T Q Q Q Q Q Q Q P F L P A V A G S W W Y G N Q A L P L P P L P P G V A S S P S K R A R L
                          760                 770                 780                 790                 800
```

```
HUMAN*  - - - - - - - - - - - - - - - - - - - - - - - - - - - - - - - - - - - - - - - - - - - - - - - - - -
CHICK   - - - - - - - - - - - - - - - - - - - - - - - - - - - - - - - - - - - - - - - - - - - - - - - - - -
XENTR   - - - - - - - - - - - - - - - - - - - - - - - - - - - - - - - - - - - - - - - - - - - - - - - - - -
DANRE   - - - - - - - - - - - - - - - - - - - - - - - - - - - - - - - - - - - - - - - - - - - - - - - - - -
CIOIN   - - - - - - - - - - - - - - - - - - - - - - - - - - - - - - - - - - - - - - - - - - - - - - - - - -
ANOGA   - - - - - - - - - - - - - - - - - - - - - - - - - - - - - - - - - - - - - - - - - - - - - - - - - -
TETNG   - - - - - - - - - - - - - - - - - - - - - - - - - - - - - - - - - - - - - - - - - - - - - - - - - -
DROME   - - - - - - - - - - - - - - - - - - - - - - - - - - - - - - - - - - - - - - - - - - - - - - - - - -
BRUMA   - - - - - - - - - - - - - - - - - - - - - - - - - - - - - - - - - - - - - - - - - - - - - - - - - -
CAEEL   - - - - - - - - - - - - - - - - - - - - - - - - - - - - - - - - - - - - - - - - - - - - - - - - - -
YEAST*  - - - - - - - - - - - - - - - - - - - - - - - - - - - - - - - - - - - - - - - - - - - - - - - - - -
SCHPO   - - - - - - - - - - - - - - - - - - - - - - - - - - - - - - - - - - - - - - - - - - - - - - - - - -
CANAL   - - - - - - - - - - - - - - - - - - - - - - - - - - - - - - - - - - - - - - - - - - - - - - - - - -
CANGA   - - - - - - - - - - - - - - - - - - - - - - - - - - - - - - - - - - - - - - - - - - - - - - - - - -
ASPNI   - - - - - - - - - - - - - - - - - - - - - - - - - - - - - - - - - - - - - - - - - - - - - - - - - -
RHIO9   - - - - - - - - - - - - - - - - - - - - - - - - - - - - - - - - - - - - - - - - - - - - - - - - - -
CRYNE   - - - - - - - - - - - - - - - - - - - - - - - - - - - - - - - - - - - - - - - - - - - - - - - - - -
USTMA   - - - - - - - - - - - - - - - - - - - - - - - - - - - - - - - - - - - - - - - - - - - - - - - - - -
ARATH*  - - - - - - - - - - - - - - - - - - - - - - - - - - - - - - - - - - - - - - - - - - - - - - - - - -
VITVI   - - - - - - - - - - - - - - - - - - - - - - - - - - - - - - - - - - - - - - - - - - - - - - - - - -
SOYBN   - - - - - - - - - - - - - - - - - - - - - - - - - - - - - - - - - - - - - - - - - - - - - - - - - -
SOLLC   - - - - - - - - - - - - - - - - - - - - - - - - - - - - - - - - - - - - - - - - - - - - - - - - - -
ORYSJ*  - - - - - - - - - - - - - - - - - - - - - - - - - - - - - - - - - - - - - - - - - - - - - - - - - -
HORVU   - - - - - - - - - - - - - - - - - - - - - - - - - - - - - - - - - - - - - - - - - - - - - - - - - -
SORBI   - - - - - - - - - - - - - - - - - - - - - - - - - - - - - - - - - - - - - - - - - - - - - - - - - -
SELML   - - - - - - - - - - - - - - - - - - - - - - - - - - - - - - - - - - - - - - - - - - - - - - - - - -
PHYPA   - - - - - - - - - - - - - - - - - - - - - - - - - - - - - - - - - - - - - - - - - - - - - - - - - -
VOLCA   D G A V G S S V A S L F P G G G N S F M S A T N N S L F T Q R G G G S A V S T G S G S S G I A S R E
                          810                 820                 830                 840                 850
```

```
HUMAN*  - - - - - - - - - - - - - - - - - - - - - - - - - - - - - - - - - - - - - - - - - - - - - -
CHICK   - - - - - - - - - - - - - - - - - - - - - - - - - - - - - - - - - - - - - - - - - - - - - -
XENTR   - - - - - - - - - - - - - - - - - - - - - - - - - - - - - - - - - - - - - - - - - - - - - -
DANRE   - - - - - - - - - - - - - - - - - - - - - - - - - - - - - - - - - - - - - - - - - - - - - -
CIOIN   - - - - - - - - - - - - - - - - - - - - - - - - - - - - - - - - - - - - - - - - - - - - - -
ANOGA   - - - - - - - - - - - - - - - - - - - - - - - - - - - - - - - - - - - - - - - - - - - - - -
TETNG   - - - - - - - - - - - - - - - - - - - - - - - - - - - - - - - - - - - - - - - - - - - - - -
DROME   - - - - - - - - - - - - - - - - - - - - - - - - - - - - - - - - - - - - - - - - - - - - - -
BRUMA   - - - - - - - - - - - - - - - - - - - - - - - - - - - - - - - - - - - - - - - - - - - - - -
CAEEL   - - - - - - - - - - - - - - - - - - - - - - - - - - - - - - - - - - - - - - - - - - - - - -
YEAST*  - - - - - - - - - - - - - - - - - - - - - - - - - - - - - - - - - - - - - - - - - - - - - -
SCHPO   - - - - - - - - - - - - - - - - - - - - - - - - - - - - - - - - - - - - - - - - - - - - - -
CANAL   - - - - - - - - - - - - - - - - - - - - - - - - - - - - - - - - - - - - - - - - - - - - - -
CANGA   - - - - - - - - - - - - - - - - - - - - - - - - - - - - - - - - - - - - - - - - - - - - - -
ASPNI   - - - - - - - - - - - - - - - - - - - - - - - - - - - - - - - - - - - - - - - - - - - - - -
RHIO9   - - - - - - - - - - - - - - - - - - - - - - - - - - - - - - - - - - - - - - - - - - - - - -
CRYNE   - - - - - - - - - - - - - - - - - - - - - - - - - - - - - - - - - - - - - - - - - - - - - -
USTMA   - - - - - - - - - - - - - - - - - - - - - - - - - - - - - - - - - - - - - - - - - - - - - -
ARATH*  - - - - - - - - - - - - - - - - - - - - - - - - - - - - - - - - - - - - - - - - - - - - - -
VITVI   - - - - - - - - - - - - - - - - - - - - - - - - - - - - - - - - - - - - - - - - - - - - - -
SOYBN   - - - - - - - - - - - - - - - - - - - - - - - - - - - - - - - - - - - - - - - - - - - - - -
SOLLC   - - - - - - - - - - - - - - - - - - - - - - - - - - - - - - - - - - - - - - - - - - - - - -
ORYSJ*  - - - - - - - - - - - - - - - - - - - - - - - - - - - - - - - - - - - - - - - - - - - - - -
HORVU   - - - - - - - - - - - - - - - - - - - - - - - - - - - - - - - - - - - - - - - - - - - - - -
SORBI   - - - - - - - - - - - - - - - - - - - - - - - - - - - - - - - - - - - - - - - - - - - - - -
SELML   - - - - - - - - - - - - - - - - - - - - - - - - - - - - - - - - - - - - - - - - - - - - - -
PHYPA   - - - - - - - - - - - - - - - - - - - - - - - - - - - - - - - - - - - - - - - - - - - - - -
VOLCA   W D T T A L L A E V S W I L E S P T P A A P L P Q P P L P V A A L P P P A T A G A A H M T T
                          860                 870                 880                 890
```

MED10

```
HUMAN*  --------------------------------------------------
CHICK   --------------------------------------------------
XENTR   --------------------------------------------------
DANRE   --------------------------------------------------
CIOIN   --------------------------------------------------
ANOGA   --------------------------------------------------
TETNG   --------------------------------------------------
DROME   --------------------------------------------------
BRUMA   ----------------------------------------LKGKLMDPSL
CAEEL   -------------------------MVQQQQQSQQRMMELHERNDREKLA
YEAST*  --------------------------------------------------
SCHPO   --------------------------------------------------
CANAL   --------------------------------------------------
CANGA   --------------------------------------------------
ASPNI   --------------------------------------------------
RHIO9   --------------------------------------------------
CRYNE   ------------------------------MSLDHQHLPSPAPTPSAAPP
USTMA   MSSTAGTRRPRQITPTSPSPSPEPQPGATNGSSSTINVAVPSASAPAQVA
ENCCU   --------------------------------------------------
ANTLO   ------------------------MCLQAFSLMECYLPHIQHPFMKVKVV
ARATH*  ---------MD-------PTQN---TSAGIGGSNGTIRYQTND-------
VITVI   ---------ME-------SSQNAA-SGTGVSGGNGITIPQINA-------
SOYBN   ---------MD-------SSQSAALGGNGGSGGNGTLISQNND-------
SOLLC   ---------MD-------SSQHN--SAAGSIGGNGIINPRSNDASPADLA
ORYSJ*  ---------MDGGA----ATNASGVAAAAAAAGNGV--------------
HORVU   ---------MDSAAPNSSSSSSAAVVAAAAASGNGV--------------
SORBI   ---------MENAAANPS-------AAAAAAAGNGV--------------
SELML   --------------------------------------------------
PHYPA   --------------------------------------------------
VOLCA   --------------------------------------------------
                 10        20        30        40        50
```

```
HUMAN*  ----GCVGGGRAEAMAEKFDHLEEHLEKFVENIRQLGIIVSDFQ--PSSQ
CHICK   ------------MAMAEKFDSLEEHLEKFVENIRQLGIIVSDFQ--PSSQ
XENTR   --------------MAEKFDNLEEHLEKFVENIRQLGIIVSDFQ--PSSQ
DANRE   --------------MAEKFDNLEEHLEKFVENIRQLGIIVSDFQ--PSSQ
CIOIN   --------------MAEKLEVLEACLEQFIENVRQLGVIVTDFQ--PQGQ
ANOGA   --------------MTSPLENLENHLEMFIENVRQIRIIVSDFQ--PQGQ
TETNG   --------------MAEKFDSLEEHLEKFIENIRQLGIIVSDFQ--PSSQ
DROME   --------------MSAPLENLETQLEMFIENVRQIRIIVSDFQ--PQGQ
BRUMA   MGSMSNAPILQETATDTRYNHLEQTLENFQENARQMGVIASDFS--TRSQ
CAEEL   RKTEKEREEERRKQEDDKILQLEKKLEEFQENARFIGDLASNFQ--TKYQ
YEAST*  ----MNGNSTNNEQLQQELATTQDQVASIIESFVELGVSIYDFPGTPEAT
SCHPO   --------------MTDEMKSLASRLEDTTQAFYDLALIVYNLED-TTPS
CANAL   ----MTTGALPSTPANEPLIKSADNVANLIESFIELGVLVHDNQGTPQSN
CANGA   -MGSVKSSNPALEGLHEELGVMEEQIRSIIESFIELGVSVYDFPGTPEAS
ASPNI   -------------MAPITLSTIDAELKDIIQHLFEIQSAVHGYLG-PETQ
RHIO9   -------MQPNTISSDEARHLLKEQLDELLQNLFELSVIVYDFQ--PDGN
CRYNE   NPAVGPAMTGAPPDQAAIRTAVENQLLQLSQDLYEMEISAGHVK--ENRE
USTMA   PSQVAAAAATPVDAAEAVRINLETRVRSVVDLLYQLAVCSADVQ--EGSQ
ENCCU   -MKSYIKNSLRIGFAKEVSTGRIESVSLEVARRFELLSSICDNRALPEFP
ANTLO   DEDPSKLVVYTENIEDIHLDKAANVARDVADGFSSLASSALD----TNTK
ARATH*  ----GTSTVTVADDSKENLSQVINSIEKTLGVLHQLHLTVTSFT--PASQ
VITVI   -AAAAATAGAGSEDPKQNLNQVINSIQKTLGLLHQLYLTVSSFN--VASQ
SOYBN   ----MAASATGADDSMQKLNQVSNSIQKTLGLIHQLYLTVSTFN--AAFQ
SOLLC   ANAVASAAGSPAEESKQNLNQVINSIDKTLGILHQLNLTVSSYN--VSSQ
ORYSJ*  -QA-GGGGERAEDASKQNLALMMASIQRTLGLLHQLNLNVSSFS--SASQ
HORVU   -----QGGDRPEDPSKQNLAQVTASIQRTLGLLHQLNLNVSSFS--SASQ
SORBI   -QASGAGGERPEDASKQNLAQVTSSIQKTLGLLHQLNLTVSSFN--SASQ
SELML   ----------MESNSKMALAGMEESLHSALATLHQLHCSVSSFT--LPSQ
PHYPA   -------MADPTPSSQLPTEDTLASVHRALALLHQLQCSVSGFQ--LPSQ
VOLCA   ----------MAGNGPPSAAGIASQLSRVLWKIHELEATLASYD--ASHA
                 60        70        80        90       100
```

MED10

```
HUMAN*   AGLNQKL---NFIVTGLQDIDKCRQQ---------------------LHD
CHICK    AGLNQKL---NFMVTGLQDIDKCRQQ---------------------LHD
XENTR    AGLNQKL---NFLVTGLQDIDKCRQQ---------------------LHD
DANRE    TGLNQKL---NFMITGLQDIEKCRQQ---------------------LHD
CIOIN    EVLNQKI---NSTITCLKDIENCKAS---------------------VSD
ANOGA    NVLNQKI---QSLVTGLQEIDKLKNQ---------------------I-D
TETNG    AVLNQKL---NFMISGLQDIEKCRQQ---------------------LHE
DROME    NVLNQKI---NSLVTGLQEIDKLRSQ---------------------VQD
BRUMA    EPLNQKI---HTLISGLHELDHLKNQ---------------------FMD
CAEEL    DALNGRI---YTLVRGLQDLDRMKGT---------------------FSD
YEAST*   KGMITNL---QRNVDRLYKLNVRSNDPQSS-----------------LSK
SCHPO    DAIPESL---DTLIRDLKSLPDISRK---------------------V-N
CANAL    QALMNKL---NQLILQLSQTSTTANDPNDANTN-------------LKQ
CANGA    QGMVTNL---KRNVDRIFKLNQTSNDPQSA-----------------LKD
ASPNI    QELVRKI---KNLTTALQTLSLHTRDDPTASTTAPNQYQSTNPNDPALHS
RHIO9    KLVWKKI---NSIIEHYKEIDELKGG---------------------I-T
CRYNE    TAVADYM---ENINKAFINLSNLAGQ---------------------M-T
USTMA    HLVANKV---NECIQALAALDATKDELHRA-------------------H
ENCCU    VGVVREI---VSLCSDLEDL---------------------------APH
ANTLO    VFPVDKL---TDVVRKMGMLKGMNLR------------------------
ARATH*   LHLLQRL---NSLVMELDNMTKLSEK---------------------C-N
VITVI    LPLLQRL---NALVSELDNMTKLSEK---------------------C-N
SOYBN    MPLLQRI---NGLVAELDNMVKLAEK---------------------C-N
SOLLC    LPLLQRM---NNLVLELDNMAKLGEK---------------------C-N
ORYSJ*   LPLLQRL---NSLVAELDTMQKHAEG---------------------C-N
HORVU    LPLLQRL---NALVAELDTMQKLADG---------------------C-N
SORBI    LPLLQRL---NALVAELDTMQKLAEG---------------------C-N
SELML    LLVLERL---NELVKDLNLMQSSSNQ---------------------C-N
PHYPA    LVLLERL---NGLVSEFGNMHSASQD---------------------C-H
VOLCA    ELLEQRLVQYEELLSETRDMLKRRGDAAPGEETQRNT----------LEG
                  110       120       130       140       150
```

```
HUMAN*   ITVPLEV-FEYIDQGRNPQLYTKECLERALAKNEQVKGKIDTMKKFKSLL
CHICK    ISVPLEV-FEYIDQGRNPQLYTKECLERALAKNEQVKGKIDTMKKFKSLL
XENTR    ITVPLEV-FDYIDQGRNPQLYTKECLERALAKNEQVKGKIDTLKKFKSLL
DANRE    INVPLEV-FEYIDQGRNPQLYTKECLERALAKNEQVKGKIDTLTKFKSLL
CIOIN    IHVPLEV-FDYIDSGRNPQHYTKDCIEKSLMKNEALKGKIDVYGKFRQEL
ANOGA    VNVPLEV-FDYIDQGRNPQLYTKDCIDKALTKNEEVKGKIDSYRKFKSNL
TETNG    INVPLEV-FEYIDQGRNPQLYTKECLERALARNEQVKGKIDTMTKFKSLL
DROME    VYVPFEVFFDYIDQDKNPQLYTKDCVEKALAKNEEVKGKIEGLKKFKTNL
BRUMA    VKIPLEL-LEYLDQGKNPQLYTKECLERTLNKNKEMNGKIEMYKKFRAML
CAEEL    KKVPLDL-LPYLDDGKNPCLYSKHCMEKTLEKNKAVNGKIEIYKKFRAHL
YEAST*   VDIPLEV-VQYIEDGRNPDIYTREFVEAIRRSNQYQRGKMHGLKQLRDSL
SCHPO    NLIPQDV-LEYIEQGRNPDVYARQFSELVQKDNQYVNGKLYAIEGFQKAF
CANAL    FLIPIDV-ISYIEDGRNPDIYTREFIEVNAKSNARLKGKMLGFKKLRDVF
CANGA    VNVPLEV-VQYIEDGRNPDIYTREFVEAIRRSNQYQRAKMHGMGMLRDSL
ASPNI    IALPPEI-IDYVDAARNPDIYTREFVELVQRGNQDLKGKKEAFADFRDVL
RHIO9    EHIPEEV-INYVEHGRNPDIFTQSFVERTATENQYTNGKVKAVDEFKQLL
CRYNE    DSVPHQI-IENVDRYKNPHVYTRQTITRATGENQYALGRVLGLESFRRQL
USTMA    MMIPQDV-LEMLDTGKNPDIHTRNFVNRLASENQYSYGQHRAVERYKATL
ENCCU    YEMPAEY-VDYVKENRDVLGYFERSEDSVKSEEVRIINKRKTFDIFRGMV
ANTLO    LKVPQEL-LGILSSATPPSEFFNRLCSEINNEEYALSAKKKLFSTFLQRL
ARATH*   IQIPMEV-LNLIDDGKNPDEFTKDVLNSCIARNQVTKGKTDAFKDLRKHI
VITVI    IQVPMEV-LNLIDEGKNPDEFTRDVLNSCIAKNQITKGKTDSFKGLRRHL
SOYBN    IQVPMEV-VNLIDDGKNPDEFTKDVINSCIVKNQITKGKTDALKSLRKHL
SOLLC    IQVPMEV-LNLIDDGKNPDEFTRDVLNSCIAKNQITKGKTDAFKGLRGHL
ORYSJ*   IQVPMEV-VNLIDDGKNPDEFTRDVLNSCIAKNQVTKGKTDAFKSLRKHL
HORVU    IQVPMEV-VNLIDDGKNPDEFTRDVLNSCIAKNQITKGKTDAFKSLRKHL
SORBI    IQVPMEV-VNLIDDGKNPDEFTRDVLNSCIAKNQITKGKTDAFKSLRKHL
SELML    LEIPVEV-IRFIDEGKNPDEFTKDLLNHCIQRNQATKGK-----ALRKHI
PHYPA    IQIPLEV-LNFIDEGRNPDEYTKHLLNGCIQQNQASKGKVDSFKALRKHL
VOLCA    RKVPEEL-VRALDGGVNPDEYLRETFKLCRRDNQISKGKCEALQLLFANL
                  160       170       180       190       200
```

MED10

HUMAN*	IQELSKVFPEDMAKYRSIRGEDHPPS------------------------
CHICK	IQELTKVFPEDMAKYKAIRGEDPPP-------------------------
XENTR	IQELSKVFPEDMAKYKAIRGEDHPPS------------------------
DANRE	ISELGKVFPEEMAKYKAIHGDDPPS-------------------------
CIOIN	MTQIGKVYPQQIIEYKQARGEL----------------------------
ANOGA	MKELSETFPVEISKYKAIRGDE----------------------------
TETNG	ISELSKVFPEDMSKYKAIHGEDASSL------------------------
DROME	LLELYKTFPNEMNNYRAYRKDSM---------------------------
BRUMA	LKELGEEMPNDMVLYRNLRDRKDASPQHENCNEDASD-------------
CAEEL	MKEFSEEMPDLVMYYRSIREDLDLS-------------------------
YEAST*	ADKIVDEFPELKEPVEDIIKRTSPIDNVSNTH------------------
SCHPO	AEEIKQAYPEVSSVVDKILNEGKVESTVS---------------------
CANAL	GDKLKQEFPQLEKGVDDIINRTNDNSSHTASVITDTANNNNS--------
CANGA	AEKIKEQFPELKDDVENIVQRTNMKPTGQARVNSGTTNMTTTAEQSNVVN
ASPNI	AREMRSAMPELRGEVDKVIQATGGKKQSERERG-----------------
RHIO9	TEEFTKSFPDLYNYENSIDADINSNN------------------------
CRYNE	HDAISSDFPEIPLPERRHQPIKSSQEEDAPQEGGSTMPNGYSVKIEDGAG
USTMA	DAALDQAFPELKAAQAESTETK----------------------------
ENCCU	EKGEEM--
ANTLO	DNT---
ARATH*	LEELEQNFPDEVDMYREIRASSAAEAKRLAQSQSV-LPNGDAKVKNEL--
VITVI	LEELEQAFPDEVESYREIRAASAAESKRLAQAQSV-LPNGDLKVKTEL--
SOYBN	LEELEHNFPDEVETFRESRAAAAAELKRQAQAQSA-LPNGDVRVKSEH--
SOLLC	LEDLEQAFPEEVEAYREIRAASAAEAKRLAQAQSL-LPNGDVKVKPEV--
ORYSJ*	LEELEQAFPEDVEAYREIRATAAAESKQLAQSQSA-LPNGDVKVKPEH--
HORVU	LEELEEAFPEDIEAYRQIRATSAAESKRLAQSQSV-LPNGDSKVKAEH--
SORBI	LEELEQAFPEDVEAYREIRATSAAEAKRLAQSQGT-LPNGDVKVKAEH--
SELML	LEEVEEAFPEETETYRMLRTASALESRRSAAQPIPGLSNGDARVKPEHTG
PHYPA	LEQVEEVFPAETEAYRDIRNQSSLESRRSLHTGNP-MSNGDMKLKIEH--
VOLCA	LTDSAEVFPQEAAEYRRGRGGDVPLHLRLYLA------------------
	210 220 230 240 250

HUMAN*	---------------------
CHICK	---------------------
XENTR	---------------------
DANRE	---------------------
CIOIN	---------------------
ANOGA	---------------------
TETNG	---------------------
DROME	---------------------
BRUMA	---------------------
CAEEL	---------------------
YEAST*	---------------------
SCHPO	---------------------
CANAL	---------------------
CANGA	ATAGDVGTNANTTESVEQNGI
ASPNI	---------------------
RHIO9	---------------------
CRYNE	PDGQNGQSIVNGMSS------
USTMA	---------------------
ENCCU	---------------------
ANTLO	---------------------
ARATH*	---------------------
VITVI	---------------------
SOYBN	---------------------
SOLLC	---------------------
ORYSJ*	---------------------
HORVU	---------------------
SORBI	---------------------
SELML	---------------------
PHYPA	---------------------
VOLCA	---------------------
	260 270

MED11

```
HUMAN*  --------------------------------------------------
XENTR   --------------------------------------------------
DANRE   ---------------------------------------SSSNLSISVLL
CIOIN   -----------------------------------------------MNQ
ANOGA   --------------------------------------------------
TETNG   --------------------------------------------------
DROME   --------------------------------------------------
BRUMA   MNLFIILSFNGYIRKPMFLEVHKQRQSGTSCRFCGSKTTLFSTVFDMSNL
CAEEL   ------------------------------------------------ME
YEAST*  ------------------------------------MQVLNTKSETKQEN
SCHPO   -----------------------------------------MTNSDDDLF
CANAL   ---------------------------------------------MSSLD
CANGA   ----------------------------------------------MSKS
ASPNI   ------------------------------------------MTESNDSG
RHIO9   ------------------------------------------MSKDQYVD
CRYNE   ---------------------------------------MSSPATSSKDP
USTMA   MSSAVATGSLAEHRAQASLDVLPAELQNTIMEEKFAQIKQQRQDARTVLH
ENCCU   --------------------------------------------------
ANTLO   --------------------------------------------------
ARATH*  --------------------------------------------------
VITVI   --------------------------------------------------
SOYBN   --------------------------------------------------
SOLLC   --------------------------------------------------
ORYSJ*  --------------------------------------------------
HORVU   --------------------------------------------------
SORBI   --------------------------------------------------
SELML   --------------------------------------------------
PHYPA   -----------------------------------------------MSA
VOLCA   -------------------------------------------------M
                 10        20        30        40        50
```

```
HUMAN*  -MAT---YSLANERLRALEDIEREIGAILQNAGTVILEL-----SKEKTN
XENTR   -MAT---YGMANERLRALEEIEREIAAILLNAGNVILEL-----SKEKPN
DANRE   YIFV---KGMANDRLRALEDVEKEIALVLQSAGTIVLEL-----SKEKAN
CIOIN   SNAN---LENVSNRIKKLEDIEKKITTAIQSAGLTFQEL-----SKEKPT
ANOGA   --------MTAIDKIQALDLIEKELIMCLQSAGAALMEL-----SKEKTS
TETNG   --------------------------------GNIVLEL-----SKDKHN
DROME   --------MNPLDKIHALDEIEKEIILCMQSAGQALQEL-----GKEKSS
BRUMA   STSS---GSELAWRLKAIGDVEQKIAELIRHAQTCINEL-----SKEKQI
CAEEL   PNPS---DPVLTDRIQAIVTTEKSIDEMMKCAREIIQDL-----GKEKQI
YEAST*  ETMQ---PPYIQERLKSLNDIETQLCSMLQEASQVTFIF-----GELKRG
SCHPO   SEKS---TSSDTQQVQNILELEAKIPDILSSAGKCIEAI-----QLNNSL
CANAL   NDKT---ENFIQERLDSLHEIDCKVVTLLDQFSSIFQSF-----YTKSKE
CANGA   TLEQ---PEYVKERLESLAEVDRQLCSMLQEASQVAFTY-----GEVLRG
ASPNI   EPFQ---VFTSADRIRQLNEIDKDVAKLIHSAGLAIQAL-----TNARSN
RHIO9   PTKR---MAESTMRIKDLYQVERKLVLLIETAGEALAIL-----SDEGTK
CRYNE   LELE---SLDGDSLFAALTGVEKAIPELLMQLKPILAQL-----SGPTED
USTMA   EAKQRTRNQQTDQQILLLMQADERLAALLSQAADSMRALLPPVAHTDPSV
ENCCU   -----------MERIELVKGIEANAERILEDFTEILQYL-----RSGGSD
ANTLO   -------MQSAYIEFDMKIMGQKTSFRIFETRTHLFLYI-----SQDAPD
ARATH*  --MD---PQTQNTSLQRLQNVENRVVKVLELAGGVMEEL-----ASPSGP
VITVI   --MD---SQTPNTSLQRLQNVEKRIIKVLELAGGVMDEL-----CNPSGP
SOYBN   --MD---SQGQTTSLQRLQNVEKRIVKVLELAGGVMDEL-----ASPVGP
SOLLC   --MD---SQSQNTSLQRLQNVEKRIVRVLELAGGVMDEM-----ANPSGP
ORYSJ*  --ML---PQSQSSSLQRLNHVEQMIVRAVNLAGTVMEEL-----GNATGP
HORVU   --MN---PQGQSSSLERLHNVEQRIVRVVELSGAVMEEL-----GNSQGP
SORBI   --MI---PQSQSSSLQRLQHVEKRIVRVLELAGAVMEEL-----GNSQGP
SELML   MTTS---TQPQSPALLRLAHVERKIVQAVEFAGTVMEEL-----ANAGGP
PHYPA   LTPR---PGDPSTSLQRLHHVEKKIVHTVELAGRVMEEL-----AKTGGP
VOLCA   SSIS---PPQPTEDYKRLQLIEQKVISCVQIAAQVTEHF-----SRVGRT
                 60        70        80        90       100
```

```
HUMAN*  ERL---------------LDRQAAAFTASVQHVEAELSA-----QIRYLT
XENTR   ERV---------------LDKQATQFTASVQRVESELSG-----QIRYLT
DANRE   ASL---------------LDRQLNQFQTSINRVESELSS-----QIRYLT
CIOIN   DRL---------------IEKHSSTFTQSLDEVETEMAS-----QISYLS
ANOGA   QKA---------------TETNVNQFLKSLNNVETKLSE-----QINYLT
TETNG   TSH---------------LDRQLVQFQSSVNRVESELSG-----QIRYLT
DROME   QKN---------------AETQSQQFLKSLSSVESKLSE-----QINYLT
BRUMA   SKSK--------------MEEASSAFKKCLNSIESDLSA-----QMQYLS
CAEEL   GKNK--------------MEDNANNFKKLITQVENELSA-----QMQYLS
YEAST*  NESV-----------KPQFENHVKQFYERLDKSTTQLRK-----EIQLLD
SCHPO   ED----------------FRKYSKEFLETVEFISTGLRR-----QALELE
CANAL   D-----------------FSQQTSDIYSTLSKVAIDLRK-----EIKIMD
CANGA   NAMM-----------KPQFQEHVSAFYSTLDNASGKLKK-----EIELLD
ASPNI   DSTTLASADNSLDSHKARFKEATSQYFALLSSIDVRLRR-----QVYALE
RHIO9   DENT----DKFVREKALAFRDLSSRYFSLVNDIQLALRS-----HTYYLA
CRYNE   GDEE---------RRGMEAREGVEQYMSLLDKIQFVLRQ-----TVYYLR
USTMA   TTPASPAADSTPAHGAKAFEDKAQQWFSILNEVQYSLRS-----VVRYLR
ENCCU   KCDE--------------VQNLLFASRRTVDLIDSALEMYHYFVRLKYLT
ANTLO   ------------------AKYFNKMLEKSVSKKSIASRK-----------
ARATH*  KKEF--------------VNSHCREFMQSMKDIQVTLRE-----EIKSAC
VITVI   RKEL--------------LSIHCGEFMQLIKDIQMTLRD-----EIKSAC
SOYBN   RKDV--------------VQNHCLEFMQLIKDIQVALRD-----EIKSAC
SOLLC   RKEL--------------INNHCSEFMQLIKDIQVTLRE-----EIKSAC
ORYSJ*  RTEG--------------VAGHCREFMLAMKEIQTTLRE-----EIKSAC
HORVU   RGEA--------------VASHCRDFMLFMKEIQTTLRE-----EIKSAC
SORBI   RTDA--------------VGAHCREFMIAMKEIQTTLRE-----EIKSAC
SELML   RMEF--------------VTTQCHEFMKSMKDIQTVLHE-----EIKSMC
PHYPA   RQEV--------------VNVQCQEFMQAVKEIQTTLRD-----EIKSMC
VOLCA   AELA------------GPVQQLCTTFMNTIAEALQLVKE-----GAESSS
                110       120       130       140       150
```

MED11

```
HUMAN*  Q-------------VATGQPHEGS--------------------------
XENTR   Q-------------VATGQPHEGS--------------------------
DANRE   Q-------------VATGQPHEGS--------------------------
CIOIN   Q-------------VATNQQHEGS--------------------------
ANOGA   Q-------------VSTGQPHEGS--------------------------
TETNG   Q-------------VATGQPHEGS--------------------------
DROME   Q-------------VSTGQPHEGS--------------------------
BRUMA   H-------------VCVGTAHQGS--------------------------
CAEEL   H-------------VCVGSSHQGS--------------------------
YEAST*  E-------------------------------------------------
SCHPO   K-------------------------------------------------
CANAL   D-------------------------------------------------
CANGA   E-------------------------------------------------
ASPNI   E-------------ASILAPDSSSRTG-----------------------
RHIO9   K-------------TASFTPSANKTIPFKTS-------------------
CRYNE   ETRSSPNTLRAPPVNLIPTPFASTIPSSPGEGLD----------------
USTMA   QAQLSPITPPVGPDARLSGRAGSAGRGHNLSLLEAFTDIAGLPGGTTFKL
ENCCU   ADVEKPE-------------------------------------------
ANTLO   --------------------------------------------------
ARATH*  E----------------YRPFEKC--------------------------
VITVI   E----------------YRPFEKC--------------------------
SOYBN   E----------------YRPFEKC--------------------------
SOLLC   E----------------YRPFEKC--------------------------
ORYSJ*  E----------------YRPFEKC--------------------------
HORVU   E----------------YRPFEKC--------------------------
SORBI   E----------------YRPFEKC--------------------------
SELML   E----------------YRPYENC--------------------------
PHYPA   E----------------YRPYENS--------------------------
VOLCA   G----------------DHRLELH--------------------------
                 160       170       180       190       200
```

```
HUMAN*  --SYSS--------------RKDCQMALKRVDYARLKLSD-VARTCEQML
XENTR   --SYSA--------------RKDGTMALNRIDYARVKLAE-LSRTCDQML
DANRE   --TYSA--------------RKDCQMALNRAEYARVKLGE-LGRTCEMML
CIOIN   --SYAA--------------RNKAQQSKEVISHLSTQVGD-MLQTCTQAS
ANOGA   --GYAS--------------AKVLQMAWHRIQHVKSRIKD-LEECKMRYI
TETNG   --TYSA--------------RKDCQMALNRAEYARVKLGE-LGRTCELVL
DROME   --GYAS--------------AKVLQMAWHRIQHARSRVRE-LEETKAKHS
BRUMA   --TFAS--------------QQNIALAEQTLVSLKDRLSA-IQHIYLPVI
CAEEL   --TFGV--------------LQNSLLAQSGLSSLHSELFQ-IVRYLDPTS
YEAST*  --NVGT--------RLLPI-NVNKKALGQDTEKMEEQLDL-LSAILDPSK
SCHPO   -----------------------AEVPVVSLQPKKRYASTPLSNLIFDQS
CANAL   --NIGAYDKNDDNVMILPISNVDQKNTKLGRKRLDLELAE-LKRLISDEK
CANGA   --NVGN--------RLLPI-NVNKKALGQDDDKLKEQMET-LKEFLKTDN
ASPNI   --DSGSGAAAGGASNPLDVSWLNSRKDTVGKEKEAELWAA-ARQFVQQIH
RHIO9   --IVGQ--------------HKQLEIWTEAIKAIQQRIHE-IKQIGQE--
CRYNE   --GYAT--------GPAELGLYASRIEAKVLKDMEEALGS-LRSELGQQ-
USTMA   QPLHTTTPSAPSASSSLSTILPESNLSLQALRERQSNWKT-LSGSLHQIH
ENCCU   --AMDK--------------RDGGKMLANMFGELNANLGV----------
ANTLO   --------------KHLVFCRLKGQEYLKTVSHIILDVLQ--GKAVDKIT
ARATH*  --DYNA--------------RIANEICFQKLEYVLTQLED-LKQTADRYP
VITVI   --DYIS--------------RISNEICCKKLEYVISQLDG-MKETIDEYH
SOYBN   --DYGS--------------RIANEICHKKVEFIMSQLDA-MKQTIDEYH
SOLLC   --DYVP--------------RISNEICCKKLDYVISQFDN-MKQTIEGYH
ORYSJ*  --DYSA--------------RIANEICCKKVEYVLEKLDA-MQKNIEECS
HORVU   --DYSA--------------RIANEICCKKLEYVIEKMDA-MQLNMEQSS
SORBI   --DYSA--------------RIANEICCKKLEYVIEKLDT-MQQNLEQST
SELML   --DYIP--------------RMGGEISLKKIDCVLDELQK-LEDTINNYQ
PHYPA   --DYPA--------------RINSEISVKKLECVIGQIEA-MREAVAQYE
VOLCA   --AYHS--------------LARAHINTEKLQVMRMHLDA-MQAELEAAA
                 210       220       230       240       250
```

```
HUMAN*  EN------------------------------------------------
XENTR   EQP-----------------------------------------------
DANRE   DPQT----------------------------------------------
CIOIN   FPR-----------------------------------------------
ANOGA   QANRMQSNRVGQNTSSSTAGVML---------------------------
TETNG   EPQQQT--------------------------------------------
DROME   HAARQQLKRQQEHAAAQQQQQQQQQQQQQQQQ------------------
BRUMA   TDVSTTR-------------------------------------------
CAEEL   DEPQTTEEDEEDGSDDLNEDGADGAPSSTVTS------------------
YEAST*  SK------------------------------------------------
SCHPO   SKLIPKYC------------------------------------------
CANAL   QVETLENESNNEIQPKTESDTNQVETNENGNDINNKESEDIEMKE-----
CANGA   EETSTS--------------------------------------------
ASPNI   QANSEDKATVKVEGEQETMEVD----------------------------
RHIO9   --------------------------------------------------
CRYNE   --------------------------------------------------
USTMA   QSMIDSTADGSFKTSPELSLLENIKNGCSCDRILIDALVNTNNVA-----
ENCCU   --------------------------------------------------
ANTLO   GGR-----------------------------------------------
ARATH*  SSD-----------------------------------------------
VITVI   DAV-----------------------------------------------
SOYBN   AAV-----------------------------------------------
SOLLC   AAASDHMALD----------------------------------------
ORYSJ*  TTS-----------------------------------------------
HORVU   DGV-----------------------------------------------
SORBI   DDV-----------------------------------------------
SELML   SSGPS---------------------------------------------
PHYPA   AVKPM---------------------------------------------
VOLCA   AGRECGRAGGGCCSACDVGSGSLGAGVEAGNGGMAVVMVVMVVAVAVMLV
                 260       270       280       290       300
```

MED11

```
HUMAN*  --------------------------------------------------
XENTR   --------------------------------------------------
DANRE   --------------------------------------------------
CIOIN   --------------------------------------------------
ANOGA   --------------------------------------------------
TETNG   --------------------------------------------------
DROME   --------------------------------------------------
BRUMA   --------------------------------------------------
CAEEL   --------------------------------------------------
YEAST*  --------------------------------------------------
SCHPO   --------------------------------------------------
CANAL   --------------------------------------------------
CANGA   --------------------------------------------------
ASPNI   --------------------------------------------------
RHIO9   --------------------------------------------------
CRYNE   --------------------------------------------------
USTMA   --------------------------------------------------
ENCCU   --------------------------------------------------
ANTLO   --------------------------------------------------
ARATH*  --------------------------------------------------
VITVI   --------------------------------------------------
SOYBN   --------------------------------------------------
SOLLC   --------------------------------------------------
ORYSJ*  --------------------------------------------------
HORVU   --------------------------------------------------
SORBI   --------------------------------------------------
SELML   --------------------------------------------------
PHYPA   --------------------------------------------------
VOLCA   PLGMKPCISRFTALVIRFTGGTSSSRVRQFGTPTHPCLRMRVRMCSRSCT
                 310       320       330       340       350
```

```
HUMAN*  --------------------------------------------------
XENTR   --------------------------------------------------
DANRE   --------------------------------------------------
CIOIN   --------------------------------------------------
ANOGA   --------------------------------------------------
TETNG   --------------------------------------------------
DROME   -----------------------MQQAAQQQQQQTGGGNAGSGDHPMGGD
BRUMA   --------------------------------------------------
CAEEL   -----------------------STTDGSGGGDDAASSSAPRSQEESGRQ
YEAST*  --------------------------------------------------
SCHPO   --------------------------------------------------
CANAL   --------------------------------------------------
CANGA   --------------------------------------------------
ASPNI   --------------------------------------------------
RHIO9   --------------------------------------------------
CRYNE   --------------------------------------------------
USTMA   --------------------------------------------------
ENCCU   --------------------------------------------------
ANTLO   --------------------------------------------------
ARATH*  --------------------------------------------------
VITVI   --------------------------------------------------
SOYBN   --------------------------------------------------
SOLLC   --------------------------------------------------
ORYSJ*  --------------------------------------------------
HORVU   --------------------------------------------------
SORBI   --------------------------------------------------
SELML   --------------------------------------------------
PHYPA   --------------------------------------------------
VOLCA   ASIFPRPDCMNLDYPCAPGLALFALFAWGVSGRRCRSVLPVFDTCQRPPS
                 360       370       380       390       400
```

```
HUMAN*  ----------
XENTR   ----------
DANRE   ----------
CIOIN   ----------
ANOGA   ----------
TETNG   ----------
DROME   SSMSTN----
BRUMA   ----------
CAEEL   MTDDDDDMEQ
YEAST*  ----------
SCHPO   ----------
CANAL   ----------
CANGA   ----------
ASPNI   ----------
RHIO9   ----------
CRYNE   ----------
USTMA   ----------
ENCCU   ----------
ANTLO   ----------
ARATH*  ----------
VITVI   ----------
SOYBN   ----------
SOLLC   ----------
ORYSJ*  ----------
HORVU   ----------
SORBI   ----------
SELML   ----------
PHYPA   ----------
VOLCA   SYLTWLLPRW
                 410
```

MED12

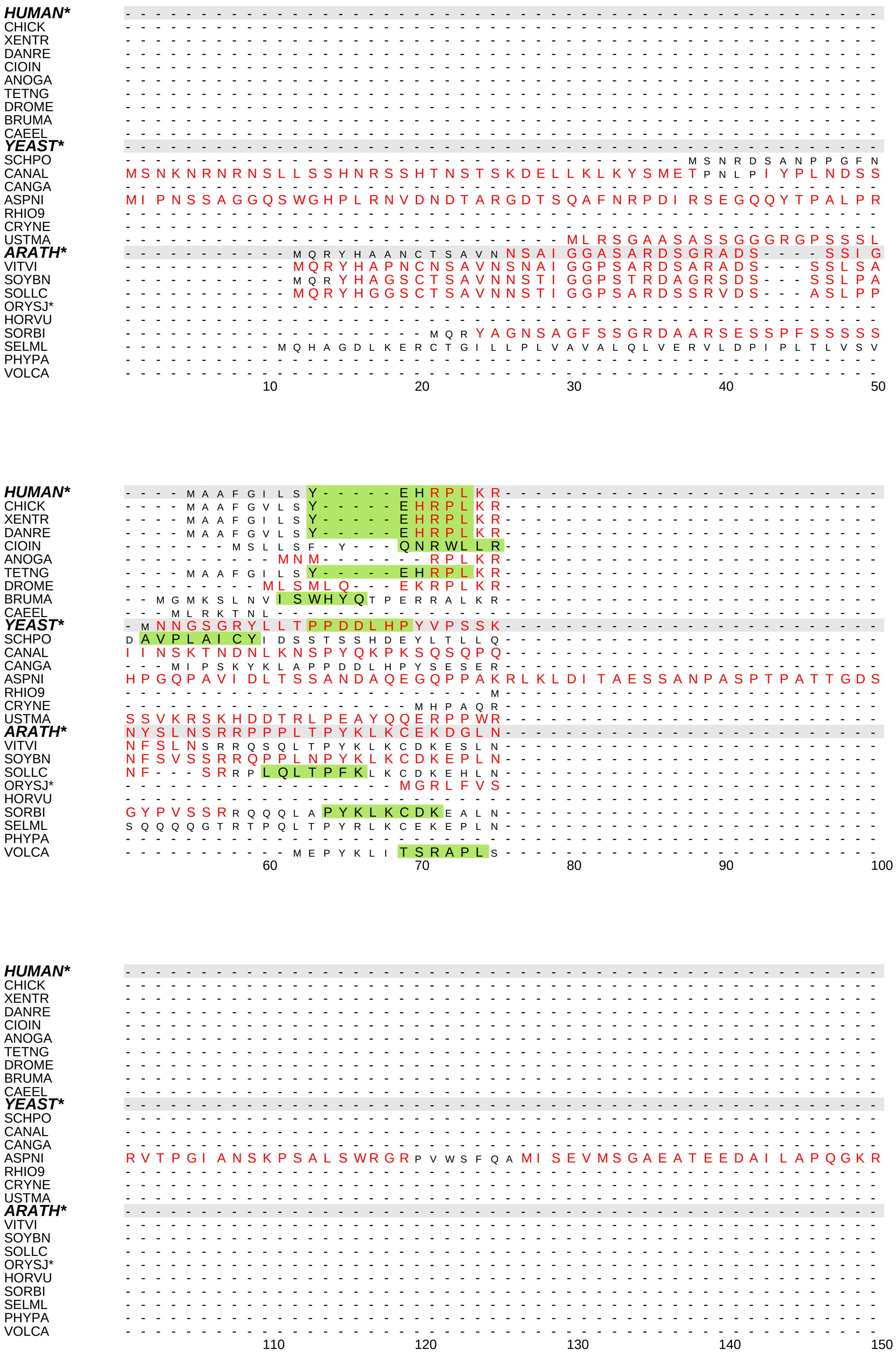
HUMAN*
CHICK
XENTR
DANRE
CIOIN
ANOGA
TETNG
DROME
BRUMA
CAEEL
YEAST*
SCHPO MSNRDSANPPGFN
CANAL MSNKNRNRNSLLSSHNRSSHTNSTSKDELLKLKYSMETPNLPIYPLNDSS
CANGA
ASPNI MIPNSSAGGQSWGHPLRNVDNDTARGDTSQAFNRPDIRSEGQQYTPALPR
RHIO9
CRYNE
USTMA MLRSGAASASSGGGRGPSSSL
ARATH* MQRYHAANCTSAVNNSAIGGASARDSGRADSSSIG
VITVI MQRYHAPNCNSAVNSNAIGGPSARDSARADSSSLSA
SOYBN MQRYHAGSCTSAVNNSTIGGPSTRDAGRSDSSSLPA
SOLLC MQRYHGGSCTSAVNNSTIGGPSARDSSRVDSASLPP
ORYSJ*
HORVU
SORBI MQRYAGNSAGFSSGRDAARSESSPFSSSSS
SELML MQHAGDLKERCTGILLPLVAVALQLVERVLDPIPLTLVSV
PHYPA
VOLCA
10 20 30 40 50
HUMAN* MAAFGILSYEHRPLKR
CHICK MAAFGVLSYEHRPLKR
XENTR MAAFGILSYEHRPLKR
DANRE MAAFGVLSYEHRPLKR
CIOIN MSLLSFYQNRWLLR
ANOGA MNMRPLKR
TETNG MAAFGILSYEHRPLKR
DROME MLSMLQEKRPLKR
BRUMA MGMKSLNVISWHYQTPERRALKR
CAEEL MLRKTNL
YEAST* MNNGSGRYLLTPPDDLHPYVPSSK
SCHPO DAVPLAICYIDSSTSSHDEYLTLLQ
CANAL IINSKTNDNLKNSPYQKPKSQSQPQ
CANGA MIPSKYKLAPPDDLHPYSESER
ASPNI HPGQPAVIDLTSSANDAQEGQPPAKRLKLDITAESSANPASPTPATTGDS
RHIO9 M
CRYNE MHPAQR
USTMA SSVKRSKHDDTRLPEAYQQERPPWR
ARATH* NYSLNSRRPPPLTPYKLKCEKDGLN
VITVI NFSLNSRRQSQLTPYKLKCDKESLN
SOYBN NFSVSSRRQPPLNPYKLKCDKEPLN
SOLLC NFSRRPLQLTPFKLKCDKEHLN
ORYSJ* MGRLFVS
HORVU
SORBI GYPVSSRRQQQLAPYKLKCDKEALN
SELML SQQQQGTRTPQLTPYRLKCEKEPLN
PHYPA
VOLCA MEPYKLITSRAPLS
60 70 80 90 100
HUMAN*
CHICK
XENTR
DANRE
CIOIN
ANOGA
TETNG
DROME
BRUMA
CAEEL
YEAST*
SCHPO
CANAL
CANGA
ASPNI RVTPGIANSKPSALSWRGRPVWSFQAMISEVMSGAEATEEDAILAPQGKR
RHIO9
CRYNE
USTMA
ARATH*
VITVI
SOYBN
SOLLC
ORYSJ*
HORVU
SORBI
SELML
PHYPA
VOLCA
110 120 130 140 150

MED12

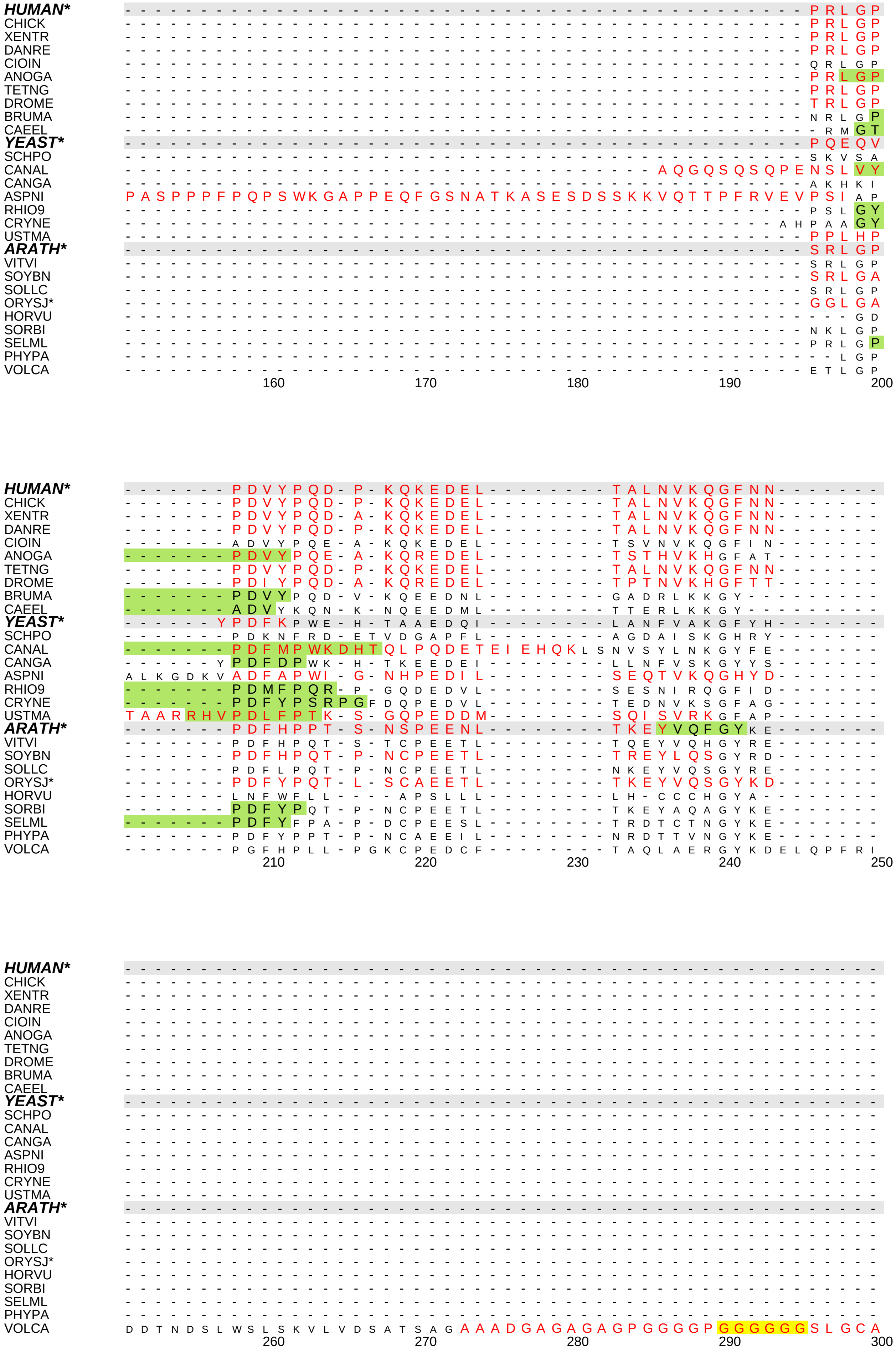
HUMAN*
CHICK
XENTR
DANRE
CIOIN
ANOGA
TETNG
DROME
BRUMA
CAEEL
YEAST*
SCHPO
CANAL
CANGA
ASPNI
RHIO9
CRYNE
USTMA
ARATH*
VITVI
SOYBN
SOLLC
ORYSJ*
HORVU
SORBI
SELML
PHYPA
VOLCA
160
170
180
190
200
210
220
230
240
250
260
270
280
290
300

MED12

```
HUMAN*  --------------------------------------------------
CHICK   --------------------------------------------------
XENTR   --------------------------------------------------
DANRE   --------------------------------------------------
CIOIN   --------------------------------------------------
ANOGA   --------------------------------------------------
TETNG   --------------------------------------------------
DROME   --------------------------------------------------
BRUMA   --------------------------------------------------
CAEEL   --------------------------------------------------
YEAST*  --------------------------------------------------
SCHPO   --------------------------------------------------
CANAL   --------------------------------------------------
CANGA   --------------------------------------------------
ASPNI   --------------------------------------------------
RHIO9   --------------------------------------------------
CRYNE   --------------------------------------------------
USTMA   --------------------------------------------------
ARATH*  --------------------------------------------------
VITVI   --------------------------------------------------
SOYBN   --------------------------------------------------
SOLLC   --------------------------------------------------
ORYSJ*  --------------------------------------------------
HORVU   --------------------------------------------------
SORBI   --------------------------------------------------
SELML   --------------------------------------------------
PHYPA   --------------------------------------------------
VOLCA   GPHPLAAAASALQRAALSMLTTAQRQRLEAEQGGPLSALGWGYGMGIPRE
                 310       320       330       340       350
```

```
HUMAN*  --------QPAVSGDEHGS---------AKNVSF---------------N
CHICK   --------QPAVSGDEHGS---------AKNVNF---------------N
XENTR   --------QPAVSADEHGS---------AKNVNF---------------N
DANRE   --------QPAVSGDEHGS---------AKNVNF---------------N
CIOIN   --------QPPFN-EEYSS---------AHLYTI---------------D
ANOGA   --------EHKLS-EEFGT---------ARNCNV---------------S
TETNG   --------QPAVSGDEHGS---------ARNVNF---------------N
DROME   --------TPPLS-DEFGT---------AHNSNV---------------N
BRUMA   --------QVAVTSYEHESIVYNVKMPRLDRLDD---------------T
CAEEL   --------QMPLTQYELQSLVFNASENKLIRLEK---------------A
YEAST*  --------TPMVN-FESIS---------ARSSVHESLVT----------Q
SCHPO   --------SPPVR-SDRAS---------AQGLLQQHLEKSS--------T
CANAL   --------PPQVA-NEYYS---------ARNLIQATVFLSTDNCTN---V
CANGA   --------TSKVN-FESIS---------ARSSLQDSLPK----------L
ASPNI   --------RTQVSQNESNT---------ARPSLYAQLKHRS--------G
RHIO9   --------RPIVS-NENSS---------AHDIVF-------GKLQEDQRA
CRYNE   --------KPFVSEAETFS---------MHGPIHQHLSGG---------C
USTMA   --------RTYVH-NEYFC---------AHDAIHTRLLSPD--------A
ARATH*  --------T--VDGLKESEEI---------ILSQVHTFSKP-VVHKCKEA
VITVI   --------T--VVGLEDAREI---------ALTQIQAFSKP-TVLKCKEA
SOYBN   --------T--VEGLEEAREI---------SLTQVPHFNKK-VVLSCKEA
SOLLC   --------T--VEGLEEVKEI---------SLTQLPAFTKP-VIFKCKEA
ORYSJ*  --------T--VEGIEEAREI---------VLSQIPYLSKPDIATKCKEA
HORVU   --------------------------------------------------
SORBI   --------T--VEGIEEAREI---------VLSQIPYFCKPDAIIKCKEA
SELML   --------A--IEGIEEARETT------WTLATNNALWAKE-NISRYKES
PHYPA   --------L--IAGVEVSY-------------FDKHLSNNPISVVCLFQS
VOLCA   RRDKSRRARAVVTHISTSSRVIWVRANITSPPVSSASPPPPPQPRAPHKR
                 360       370       380       390       400
```

```
HUMAN*  PAKISSNFSSIIAEKLRCNTLPDTG---RRK-------------------
CHICK   PAKISSNFSSIIAEKLRCNTLPDTG---RRK-------------------
XENTR   PAKISSNFSSIMSEKLRCNTLPDTG---RRK-------------------
DANRE   SSKISSNFSSIIAEKLRCNTFPDTG---KRK-------------------
CIOIN   ANKINQSFNQILTQKQVVTTFNDAGAGKRLK-------------------
ANOGA   ASKVGAYFNAILAKKEELMTLPDSG---RKK-------------------
TETNG   PSKISSNFSSIIAEKLRYNTFPDTG---KRK-------------------
DROME   ASKVSAFFSGVLAKKEELMTLPDTG---RKK-------------------
BRUMA   MLRGAQLVMQIMAKKMELNANLDKE---RRRAGSVITKDSTGSGGNGVG-
CAEEL   QKRSQSMIASVIHKKGEFHMNYDRE---RKI-------------------
YEAST*  SNILSQQFDKIIKIREDH-----------------INKIPSNST------
SCHPO   LENLKRYSSTLLGRQNDA--------------------------------
CANAL   INELSIHLSNAYKSRNEI--------------------------------
CANGA   SSTLSEKFSEVVRIRERE-----------------INTIKGNSK------
ASPNI   LQMLSSVFMAALEKRQNH--------------------------------
RHIO9   LSDLSDFIVDVLHRQNRA--------------------------------
CRYNE   LAMLMQLGKELVEKQEQS-----------------------MPQFQYAAC
USTMA   LDTLSDWMDEVMQRRREL--------------------------------
ARATH*  VRKCLRAINESRALKRKA-------------------------------G
VITVI   IRKRLRAINESRAQKRKA-------------------------------G
SOYBN   IRKRLRAINESRAQKRKA-------------------------------G
SOLLC   IRKCHRAINESRAQKRKA-------------------------------G
ORYSJ*  LKKRFRAINESRAQKRKA-------------------------------G
HORVU   --------------------------------------------------
SORBI   LKKRLRAINESRAQKRKA-------------------------------G
SELML   LRKRLRAVTNALVRKRKA-------------------------------G
PHYPA   IRKRLRELSIALIRKRKA-------------------------------G
VOLCA   ATAMPLYGNPAVAMSQGT--------------------------------
                 410       420       430       440       450
```

MED12

```
HUMAN*  -------PQVNQKDNFWLV---TARSQSAINTWFTDLAG-TKPLTQLAK-
CHICK   -------PQVNQKDNFWLV---TARSQSAINNWFTDLAG-TKPLTHLAK-
XENTR   -------PQVNQKDNFMLV---TVRTQSSINTWFSDLAG-TKPLTHLAK-
DANRE   -------PQVNQKDNFWLV---TARSQSSINNWFTDLAG-TKPLTQLAK-
CIOIN   -------PQV-YKEQFWLVPVINAKCRVHLQTWFKDLIR-GTSLLNLCR-
ANOGA   -------QQINPKDNFWPV---TARNKTTLDTWFKDLAG-TKPLSSLAK-
TETNG   -------PQVNQKDNFWLV---TARSQSSINNWFTDLAG-TKPLTQLAK-
DROME   -------QQINCKDNFWPV---SPRRKCTVDAWFKDLAG-NKPLLSLAK-
BRUMA   -----GGPQI-YFQQFSHN-AEKAKKSKERNDWFTDLAH-GKPLAVLAR-
CAEEL   -------PKE-QLPCFAPH-NWKRILS-DRVKFFSALAR-GTPLQQLSQ-
YEAST*  --------TTLHGPGFQLP-NRITLTDHRKETWLHELSSSHTSLVKIGK-
SCHPO   -------VIDEPFAPFRPP-PRVTLSESRRDRWLQGLSDPMVPLSSLIK-
CANAL   -----INKIKFDSNKFKLP-TRVTLTSSKKEIWLNELSNPNVSMTKLGE-
CANGA   DGMAASKFTDLCGPDFKLP-SRLTLTDHRKELWLQELSSPYTPLHKITS-
ASPNI   -------NTINVPSNFKPP-PRVTLTDNKREAWLRDLANPSVPLRRLSR-
RHIO9   -------SYITGPSSFRPP-VRTTLIDAKREQWMHELSSGVVPLKQLAK-
CRYNE   PSPACRLLTAYSERSFRIP-GRVIYTDNKRLGFLADLGNRDIPLHWLMRN
USTMA   ------DRASIATNQYKPP-SRVTLNDAKLANYVKDLADPTVPLMRLSR-
ARATH*  QVYGVPLSGSLLCKPG-FP-EQRSCGEETKKRWIESLSQQHKRLRSLAD-
VITVI   QVYGVPLSGSLLTKPCVFP-EQRPCGEDFRKKWIEGLSQHHKRLRSLAD-
SOYBN   QVYGVALSGSQLGRSGIFP-ELRPYGEDFQKKWIEGLSQQHKRLRSLAD-
SOLLC   QVYGVPLEGLQLTKPGIFP-DQRSSGEEFRKKWIEGLSQQHKRLKSLAD-
ORYSJ*  QVYGVPLSGSLLTKPGMYP-EQMHSNEDTRRKWIEALVQPNRRLWSLAE-
HORVU   --------------------QFNLCSLFLCVITLQALSQPNKRLRLLSE-
SORBI   QVYGVPLSGSLLIKPGVYP-EQRPCNEDARRKWAEALAQPNKRLRSLSE-
SELML   QVYDVPLVGALLAKAGQFP-ERHTYGEDYRKKWIEDLSQ-RKRLSLLAE-
PHYPA   QVYGVPLSGNLLAKSGVFP-EHRTCGEDFRKKWIEDLSQ-RKRLRALAE-
VOLCA   ---------------VQLP-ELRTASDVGRRLWLSALAG-SASLGYLAQ-
                 460       470       480       490       500
```

```
HUMAN*  KVP----IFSKKEEVFGYLAK---------------YTVPVMRAAWLIK-
CHICK   KVP----IFSKKEEVFGYLAK---------------YTVPVMRAAWLIK-
XENTR   KVSHMFNRFVHIEENLGNIIP---------------SVLPLVQPLCILKN
DANRE   KVP----IFSKKEEVFGYLAK---------------YSVPVMRSAWMIK-
CIOIN   RVP----VFSKKDDLFRTLSE---------------YEVPMVRATWVIK-
ANOGA   KAP----SFNKKEEIFAMLCE---------------NQVTMQRAAWFIK-
TETNG   KVP----IFSKKEEVFGYLAK---------------YSVPVMRSAWMIK-
DROME   RAP----SFNKKEEIFITLCE---------------NQVNMQRATWFIK-
BRUMA   KPP----FFRKKEDALEYLCD---------------YKVPVNRALWFLK-
CAEEL   RIP----GFRRKELLFLEFSD---------------YNISYDRAIWCVK-
YEAST*  FIP----HGLKRRQVIEQCYL---------------KFIPLKRAIWLIK-
SCHPO   TIP----HGLWGEDILRMLVK---------------FRVPFTRAIWFIR-
CANAL   KIP----HGIRNKVLIDCICT---------------KKIPINRSIWFTK-
CANGA   FIP----HGLKRRQVLEQCYI---------------KQIPLKRAVWLIK-
ASPNI   TIP----HGIRGKNLLDQCLS---------------KWIPVNRAVWLVK-
RHIO9   NVP----HGFKGEKLLETLAT---------------KQVPFLRATWYIK-
CRYNE   QIP----HGFKGVELLDVMFSPLTTVRQPPPNLAPMDPIPIDRAIWLIR-
USTMA   SVP----HGFRGEKLFEMLWV------------APRKSVDIPRAVWFIR-
ARATH*  NIP-----GYRRKTLFEVLIR---------------NNVPLLRATWFIK-
VITVI   HVP----HGFRKKNLFEVLIR---------------NNVPLLRATWFIK-
SOYBN   HVP----HGYKRTSLLEVLIR---------------NNVPLLRATWFIK-
SOLLC   HVP----HGYRRKSLFEVLVR---------------NNVPLLRATWFVK-
ORYSJ*  QVP----RGFRRKLLFNYLIR---------------YNVPLLRASWLVK-
HORVU   HVP----HGYRRKSLFDVLTR---------------CNVPLLRATWFVK-
SORBI   HVP----HGYRRKSLFEGLIR---------------YNVPLLRATWFIK-
SELML   HVP----HGFHRRTLFEALVK---------------QNVPFLRATWFIK-
PHYPA   HVP----HGYQRRTIFEALIK---------------HDVPLLRATWFIK-
VOLCA   TAP----TMPDRRLFLGLMWQ---------------TGVPIPRATWFVR-
                 510       520       530       540       550
```

```
HUMAN*  ---MTCA--YYA--AIS-ETK-----------------------------
CHICK   ---MTCA--YYA--AIT-ETK-----------------------------
XENTR   FQNFPCS--HFP--LIF-LIW-----------------------------
DANRE   ---MTCA--YHA--AIT-ENK-----------------------------
CIOIN   ---ITSA--YHL--TQT-DAK-----------------------------
ANOGA   ---LSSA--YTV--AVS-EAK-----------------------------
TETNG   ---MTCA--YHA--AIT-ETK-----------------------------
DROME   ---LSAA--YTL--SFT-ESK-----------------------------
BRUMA   ---LIAVSGQGC--NSN-VNK-----------------------------
CAEEL   ---LLCI--LGF--NSS-SKT-----------------------------
YEAST*  ---CCYF--IEW--KSNHKKKRSNAAG-----------------------
SCHPO   ---CAGV--NEA--RSFLRK------------------------------
CANAL   ---CVLY--GEL--LAL-RRKHQNRLSTIPNNNSNTGGGGGGGGSGNSLT
CANGA   ---CCYS--IEW--KTG-IAKYANSPQ-----------------------
ASPNI   ---CVGA--NEI--RACKRKGTGGT-------------------------
RHIO9   ---IVGL--SEM--SQRNAS------------------------------
CRYNE   ---VIGS--NDI--AAHRARQHQTAASVPAPSPAAATPSSTTTVTATPA-
USTMA   ---ALGA--AEL--SS----------------------------------
ARATH*  ---VTYL--NQV--RP-SPAAISSG-------------------------
VITVI   ---VTYL--NQV--RP-ASASISSG-------------------------
SOYBN   ---VTYL--NQV--RP-GSVGISSG-------------------------
SOLLC   ---VTYL--NQV--RP-GSSSISSG-------------------------
ORYSJ*  ---VTYL--NQV--QT-SSNNVSSA-------------------------
HORVU   ---VTYL--NQV--RP-TSSSISTG-------------------------
SORBI   ---VTYL--NQLQTRQ-TPNSISVA-------------------------
SELML   ---ISYL--NQV--RPASSA------------------------------
PHYPA   ---IIYL--NQV--KP-MSIGMSAG-------------------------
VOLCA   ---LLYT---HL--ARQQLRPLPPPPPLPAGSAAAAAAAAGGGGASAAAA
                 560       570       580       590       600
```

MED12

```
HUMAN*  ------VKKRHV-DPFMEWTQIITKYLWEQLQKMAEYYRPGPAGSG----
CHICK   ------VKKRHVIDPFIEWTQIITKYLSEQLQKIAEFYRQLPGQ------
XENTR   ------VIKGHV-DLCAEWTQIITRYLWEQLQKIADYYRPALG-------
DANRE   ------MKKRHVIDPCIEWTPIITKYLWEQLQKVAEFYRQSPSQ------
CIOIN   ------IKKRAANDPNLEWTQAICRFMREHLVKLNNDNNNQPNSNSSMNL
ANOGA   ------IKKRQMPDPATEWTGTMIKFMKDLIPKLHEHYHQGPLQEKPSSG
TETNG   ------VKKRHVIDPCIEWTQIITKYLWEQLQKVAEFYRQFPNQ------
DROME   ------NKKRSIYDPAAEWTGNMIKFMKELLPKLQEYYQQNHDKSSSNGT
BRUMA   ------QKKSTADQLASEFASLFTKYIKLMLNQMSDSAKL----------
CAEEL   ------QKKTLLDVATLELSQACNKTTIWLVTQLYKSFRN----------
YEAST*  ------ADDAISMHLLKDWTDTFVYILEKLIFDMTNHYN-----------
SCHPO   ------VQTTDITEWVKNWTDVAAGFLISFISSFLNA-------------
CANAL   GMMDYNTAEKFEIHWLQEWTQQVSDYIYKFSREMNNIANL----------
CANGA   ------EKDKFNAHLLKEWTDNFVHILEKLIFEMTQHYN-----------
ASPNI   ------LVVGSEVKWVRDWTSGVQQFLEGVIGACGSA-------------
RHIO9   ------NAAASMGPQSIQWTTIVTNHLKKQLNELVPSPHTSNSNNAAIP-
CRYNE   ------MPVSSNDWYSQEFTNTVISWLRIQLGQLALPNTVKVAGKLPAT-
USTMA   ------LRNKSAATIIPEITSNLCAWMAKQVAELNLVHTAESNSTATAS-
ARATH*  ------TPDKTQASRCEQWTKDVIEYLQYLLDELLSRNSSFPAQQTRDR-
VITVI   ------SPDKIQLSRTELWTKDVIDYLQGLLEEFFSRNNSHSTQHSRDK-
SOYBN   ------AADKIQLSRSDVWTKDVINYLQTLVDEFLSKNALHSASHGRER-
SOLLC   ------VPDKTHISRSEQWTKDVIDYLQYLLDEFISRNSVHSALQIRDR-
ORYSJ*  ------APDS---LRSQHWTKDVIEYLQLLLDELCSKNGFFGLPSSQEQ-
HORVU   ------ASDN---QRSNQWTKDVVEYLQQLLDEFSLKEAHL---SFKEQ-
SORBI   ------ASDN---QRSNQWTKDVVEYLQQILDEFCSKEGAVVPPSFREQ-
SELML   ------GADKSHGKRLEQWTRDVLDYAQSVLDEICSNASRAALAQAYDS-
PHYPA   ------GPDKSQSKRVELWTKNVLEYTQSLLDDLCQTGGASGSHSQILS-
VOLCA   GAGAQAASSALASTRASLWTSDLLTYLDSQIAAVEAAAAAAAAAAAAAA-
                 610       620       630       640       650
```

```
HUMAN*  ----------------------------------------------GCGS
CHICK   ----------------------------------------------SCGS
XENTR   ----------------------------------------------GCSS
DANRE   ----------------------------------------------GCGS
CIOIN   SSGIETVC-------------------------------------SVMNS
ANOGA   GAGSSGGLGSGASGLG---------LSSGAGGTNAVTIP------PPLSS
TETNG   ----------------------------------------------GCSS
DROME   TSGSLTAAGNGPASNGSTGTSSINSVTGSSASTNVIPVPSMASPLPPIHS
BRUMA   --------------------------------------------------
CAEEL   --------------------------------------------------
YEAST*  --------------------------------------------------
SCHPO   --------------------------------------------------
CANAL   --------------------------------------------------
CANGA   --------------------------------------------------
ASPNI   --------------------------------------------------
RHIO9   --------------------------------------------------
CRYNE   --------------------------------------------------
USTMA   --------------------------------------------------
ARATH*  --------------------------------------------------
VITVI   --------------------------------------------------
SOYBN   --------------------------------------------------
SOLLC   --------------------------------------------------
ORYSJ*  --------------------------------------------------
HORVU   --------------------------------------------------
SORBI   --------------------------------------------------
SELML   --------------------------------------------------
PHYPA   --------------------------------------------------
VOLCA   --------------------------------------------------
                 660       670       680       690       700
```

```
HUMAN*  TI------------------------------------------------
CHICK   PA------------------------------------------------
XENTR   PS------------------------------------------------
DANRE   PL------------------------------------------------
CIOIN   QT------------------------------------------------
ANOGA   PA------------------------------------------------
TETNG   PP------------------------------------------------
DROME   PANGQQAAPGGGVNAGSVMPTTGSLGGVVGGPGSSVVGGAAGAGAAVPPG
BRUMA   --------------------------------------------------
CAEEL   --------------------------------------------------
YEAST*  --------------------------------------------------
SCHPO   --------------------------------------------------
CANAL   --------------------------------------------------
CANGA   --------------------------------------------------
ASPNI   --------------------------------------------------
RHIO9   ---------------------------------HAAGTPNRGYRNYSNHH
CRYNE   --------------------------------------------------
USTMA   --PAPVSPSTHLPRTPSAAPSSLSRSVAAAHFARTPSNLNPASSPSLAPA
ARATH*  ------------------------------------SPQMLYTGS--MQ-
VITVI   ------------------------------------SQQILYAGS--IQH
SOYBN   ------------------------------------SPQMSYTGS--LQN
SOLLC   ------------------------------------SQQMVYAGS--IPL
ORYSJ*  ------------------------------------SLPCLVAGDSPIKL
HORVU   ------------------------------------PSAGLISGTTQVKL
SORBI   ------------------------------------SSPGLTAGTNQIKI
SELML   --------------------------------------------------
PHYPA   --------------------------------------------------
VOLCA   ---------------------------------AATAASSAGGGAGTAGP
                 710       720       730       740       750
```

MED12

```
HUMAN*    ------GPLPHDVEVAIRQWD-----------------------------
CHICK     ------GPMPQEVEQALKQWD-----------------------------
XENTR     ------GPMPPEVEVALKQWE-----------------------------
DANRE     ------PAPPAEVETAMKQWE-----------------------------
CIOIN     ------MLTREDIEVMLK--------------------------------
ANOGA     ------GSMHKEQRIAQKQWN-----------------------------
TETNG     ------PAMPSDVDTAMKQWE-----------------------------
DROME     STISGIGSQFEDSRNALKYWK-----------------------------
BRUMA     ---------E-SNIVYTDRWP-----------------------------
CAEEL     ---------PIKRREATKTWN-----------------------------
YEAST*    --------DSQQLRTWKRQIS-----------------------------
SCHPO     -----------DIYSFADDYT-----------------------------
CANAL     ----------DGKNQYMGKLS-----------------------------
CANGA     --------DSVKLEAWKTEVS-----------------------------
ASPNI     --------------DWKSKMT-----------------------------
RHIO9     ENSAKPWLTPESRARFEQRWS-----------------------------
CRYNE     KAGSGILQDEKARAKWLAKWD-----------------------------
USTMA     KEVCYVLQNDADEERWIAKWS-----------------------------
ARATH*    KNSPASTSLYGEETSLHFKWW-----------------------------
VITVI     KSDP-VSGLDSEEPSLHFKWW-----------------------------
SOYBN     KNDPLLSVSDGEGPSLHFRWW-----------------------------
SOLLC     KSDPTLGSVDCEEPSLHFKWW-----------------------------
ORYSJ*    KTGASPASADVEEPSLHFKWS-----------------------------
HORVU     KNEAPSAGGDTEEPLVHFKWW-----------------------------
SORBI     KTEASPAAGDSEEPLVHFKWR-----------------------------
SELML     ------VQSVETDLDGHAKWH-----------------------------
PHYPA     ----VRGDSVENEPDLLAKWR-----------------------------
VOLCA     LPSPPVDYTAASPNVASPDWASLSADDAFPAAGSGGGGGGTAACGADPRA
                 760       770       780       790       800
```

```
HUMAN*    ------------YTEKLAMFMFQDGMLDRHEFLTWVLECF----------
CHICK     ------------YNEKLAMFMFQDGMLDRHEFLTWVLECF----------
XENTR     ------------YNEKLALFMFQDGMLDRHEFLTWVLECF----------
DANRE     ------------YNEKLAMFMFQDGMLDRHEFLTWVLECF----------
CIOIN     ------------YISCLAHHMYSEGLFDRNEFLSWVVDEF----------
ANOGA     ------------YSTQLCKYMYEEGLLDKQEFLNWIIDLL----------
TETNG     ------------YNEKLAMFMFQDGMLDRHEFLTWVLECF----------
DROME     ------------YCHQLSKYMYEESLLDRQEFLNWILDLL----------
BRUMA     ------------HFVYICKHAYEDGMVEKQEFLMDLLDIF----------
CAEEL     ------------YLTGFMKYMFDDGVLDKQEFLNDLTEVF----------
YEAST*    ------------YFLKLLGNCYSLRLINKEIFHHWLVEFI----------
SCHPO     ------------YLLKLFGRLLAEELVSPKHFLLRIVSFS----------
CANAL     ------------YLLNYIQSLYVESLLDKSLFLSLIMKFLKDGLCLQSKY
CANGA     ------------YFLKLVGNCYTLELIDKNTFHFWILQFV----------
ASPNI     ------------YATSLTARLFFERLLDHDQYFAWFLTSL----------
RHIO9     ------------YSVQLARWQYYEGLIDQKTFLRWSLDTL----------
CRYNE     ------------YSTRLLRALHTKHLISTRLFTGWLADHL----------
USTMA     ------------YSLSLARHLHAQNLLDRSVLVRWIVDAF----------
ARATH*    ------------YMVRLLQWHHAEGLLFPNLIVDWVLKLL----------
VITVI     ------------YVVRILQWHHAEGLILPSLIIDWALRQL----------
SOYBN     ------------YIVRLLQWNHAEGLLHPSLVIDWVFNQLQASCFDFSHF
SOLLC     ------------YVVRILQWHHREGLLIPSLVIDWVLNQL----------
ORYSJ*    ------------YMIRIVQCHLMEQLLVPSLLIEWVFNQL----------
HORVU     ------------YMVRLVQWHLTEELLVPSVLIEWSCHQL----------
SORBI     ------------YMVRLIQWHLTEELLVPSVLIEWLSNQL----------
SELML     ------------YIVRLVQWQYAEGLLQQSHVVEWALKQI----------
PHYPA     ------------YIVGLAQWHYTEGLLNRTQVVDWALKQL----------
VOLCA     RLLDYQPGAPVLYLVRLAGSTFLDGLLDTAKLVDWGCGQL----------
                 810       820       830       840       850
```

```
HUMAN*    ----EKIRP---GED-----------------------------------
CHICK     ----EKIRS---GED-----------------------------------
XENTR     ----EKIRQ---GED-----------------------------------
DANRE     ----EKIRP---GED-----------------------------------
CIOIN     ----ECISL---YDE-----------------------------------
ANOGA     ----EKMKSSPNADD-----------------------------------
TETNG     ----EKVRP---GED-----------------------------------
DROME     ----DKMRTQASFDE-----------------------------------
BRUMA     ----NDRFVQPIENRKND-------------------------------K
CAEEL     ----NQLFLQ-KNFKNP---------------------------------
YEAST*    ---NKMENF-----------------------------------------
SCHPO     ----GDSSL-----------------------------------------
CANAL     INGLIYSSMEENSDDYNDDDDDDDDEEDANNAENPESTSKSKKRNLAWMD
CANGA     ---SKVENF-----------------------------------------
ASPNI     ----EGASL-----------------------------------------
RHIO9     ---SDCTSF-----------------------------------------
CRYNE     ----THVNL-----------------------------------------
USTMA     ----SASNL-----------------------------------------
ARATH*    ---QEKEIF-----------------------------------------
VITVI     ---QDKELL-----------------------------------------
SOYBN     CNFQEKDLL-----------------------------------------
SOLLC     ---QEKELL-----------------------------------------
ORYSJ*    ---QERDST-----------------------------------------
HORVU     ---QERDSV-----------------------------------------
SORBI     ---QERDSV-----------------------------------------
SELML     ---QEKESL-----------------------------------------
PHYPA     ---QEKESV-----------------------------------------
VOLCA     ----AAVSAALGGPPAAPVPAGAVAPPGNGMAASAGGAGGVPPPLPVAAA
                 860       870       880       890       900
```

MED12

```
HUMAN*  -------------------------ELL-KLLLPLLLRYSGEFVQSA---
CHICK   -------------------------EFL-KMLLPLLLRYSGEFVQSA---
XENTR   -------------------------ELL-KLLLPLLLRYSNSLVQSA---
DANRE   -------------------------ELL-RLLLPLLLQYSGEFVQSA---
CIOIN   -------------------------IAL-SYFVPILQNYIEEVTMNQ---
ANOGA   -------------------------GLL-RIYLPLAMQYLYDLVQSE---
TETNG   -------------------------ELL-RFLLPLLLQYSGEFVQSA---
DROME   -------------------------PLK-KLVLSFALQYMHDFVQSE---
BRUMA   TLIHPFLHHGSGFASASSTNHSHYVTLL-RLFLLFICQYTDQITQNI---
CAEEL   -------------------------SVL-STFMKYYVYFVEDLSSSL---
YEAST*  -------------------------EFL-PLSLHILMIFWNDICQIDTNA
SCHPO   -------------------------KSF-SLHFFALQFFSTSLIQYT---
CANAL   EIDMNYG------------------QRL--VSLTLIKMFWNDILKFD---
CANGA   -------------------------EYL-PLPLHILELFWSGICNTN---
ASPNI   -------------------------NMV-PVWLLMLGIYWDSMLRYR---
RHIO9   -------------------------EVIWVMLTGLIQDYVDEYKRNR---
CRYNE   -------------------------AQL-GFLAQLIGEYLDDLVHHL---
USTMA   -------------------------VQL-PFLLELVQEVLLLVLRRR---
ARATH*  -------------------------EIL-QLLLPIVYGVLESIVLSQ---
VITVI   -------------------------EIL-QLLLPIIYGVIETVVLSQ---
SOYBN   -------------------------EVW-QLLLPIIYGFLETIVLSQ---
SOLLC   -------------------------GVL-QLLLPVVYGFIDTVVLSQ---
ORYSJ*  -------------------------EVL-ELLLPIVLSLVDTITLSQ---
HORVU   -------------------------EAL-ELLLPIVLGLVETITLSQ---
SORBI   -------------------------DVF-ELLLPIMLGLVDTITLSQ---
SELML   -------------------------DGL-ELLLPVLLEFMDSLSLSQ---
PHYPA   -------------------------EAL-ELLLPILLELVDGISMSQ---
VOLCA   PAAGSAAGAGMAAAGPGGRALTAAEQLRAQVALQLLLACLPDITQSL---
                  910       920       930       940       950
```

```
HUMAN*  --------------YLSRRLAYFCTRRLALQLD------GVS--------
CHICK   --------------YLSRRLAYFCTRRLAMQLD------GAG--------
XENTR   --------------YLSRRLAYFCTRRLAQLLE------GSG--------
DANRE   --------------YLSRRLAYFCTRRLNLLLSDGSIGPGPG--------
CIOIN   --------------LLARRMSFAAARHLSWLFI------EGA--------
ANOGA   --------------RFCRRLAYAVSKKLAQLIN-----------------
TETNG   --------------YLSRRLAYFCTRRLNLLLSDGSMGPGAG--------
DROME   --------------RLCRKMAYIVSKKLAQLLN-----------------
BRUMA   --------------VLSKRCAFLVCHRL----------------------
CAEEL   --------------VLARRSSTLLCQALGFLFEMAQDKRKIDAYYDSANK
YEAST*  PVAATITSSQKEPFFLVTKITDMLLHKYYIVSSSKSMINDENYIINDIK-
SCHPO   --------------HICRKCVITILQSYQQLIVDQPANLL----------
CANAL   --------------YLSKELSELLLLNYFFINKLLLFSVNNDNGNSTAVI
CANGA   --STKVNESESNPLYMVTKLTDILLNQYYIILHSKSMINDDKYIINDIK-
ASPNI   --------------KRGRRLAEVLLGKLHQTTELQRPTLL----------
RHIO9   --------------TLTKILIETLIRFYSALQQYINHNTLEET-------
CRYNE   --------------GSARHCLRAACDKLVEVRNSPGKE------------
USTMA   --------------CFVKPFLTALLVQIPNVDARLDPS------------
ARATH*  --------------TYVQSLVAIAVRFIQEPAPGG--SDLVDNS------
VITVI   --------------TYVRTLVGVAVRFIKEPSPGG--SDLVDNS------
SOYBN   --------------TYVRTLAGLALHVIRDPAPGG--SDLVDNS------
SOLLC   --------------SCVRTLVGIALRFIQEPSPGG--SDLVDNS------
ORYSJ*  --------------TYIHMLVEILIQRLSDASPGS--LSVKNNP------
HORVU   --------------TYVRMFVEILVKRLSDA-------SAVDNP------
SORBI   --------------TYVRMFVELLVRRLNDA-------SVADSP------
SELML   --------------VHVRMLVDICQHWLRQLGVSNVLPSPEEQP------
PHYPA   --------------THSRMLVELCLQWLRKLYPLGHVPSIGDNS------
VOLCA   --------------LHVRVIVDAVVELLMAARCTAPGSGAVAAA------
                  960       970       980       990      1000
```

```
HUMAN*  --------------------------------------------------
CHICK   --------------------------------------------------
XENTR   --------------------------------------------------
DANRE   --------------------------------------------------
CIOIN   --------------------------------------------------
ANOGA   --------------------------------------------------
TETNG   --------------------------------------------------
DROME   --------------------------------------------------
BRUMA   ------------ELYRDE--------------------------------
CAEEL   TFIKKEFENEDGEIYEFEDYDDFIDMLDEIPDVPYFDETDKTPEAQAEEL
YEAST*  --------------------------------------------------
SCHPO   --------------------------------------------------
CANAL   PDG-----------------------------------------------
CANGA   --------------------------------------------------
ASPNI   --------------------------------------------------
RHIO9   --------------------------------------------------
CRYNE   --------------------------------------------------
USTMA   --------------------------------------------------
ARATH*  --------------------------------------------------
VITVI   --------------------------------------------------
SOYBN   --------------------------------------------------
SOLLC   --------------------------------------------------
ORYSJ*  --------------------------------------------------
HORVU   --------------------------------------------------
SORBI   --------------------------------------------------
SELML   --------------------------------------------------
PHYPA   --------------------------------------------------
VOLCA   --------------------------------------------------
                 1010      1020      1030      1040      1050
```

MED12

```
HUMAN*   --------------------------------------------------
CHICK    --------------------------------------------------
XENTR    --------------------------------------------------
DANRE    --------------------------------------------------
CIOIN    --------------------------------------------------
ANOGA    --------------------------------------------------
TETNG    --------------------------------------------------
DROME    --------------------------------------------------
BRUMA    --------------------------------------------------
CAEEL    IKQHEKRKAGSLKKSERRRRRGLSKNRPKKMPQNPSNEASLDHDKVQIKQ
YEAST*   --------------------------------------------------
SCHPO    --------------------------------------------------
CANAL    --------------------------------------------------
CANGA    --------------------------------------------------
ASPNI    --------------------------------------------------
RHIO9    --------------------------------------------------
CRYNE    --------------------------------------------------
USTMA    --------------------------------------------------
ARATH*   --------------------------------------------------
VITVI    --------------------------------------------------
SOYBN    --------------------------------------------------
SOLLC    --------------------------------------------------
ORYSJ*   --------------------------------------------------
HORVU    --------------------------------------------------
SORBI    --------------------------------------------------
SELML    --------------------------------------------------
PHYPA    --------------------------------------------------
VOLCA    --------------------------------------------------
                  1060      1070      1080      1090      1100
```

```
HUMAN*   --------------------------------------------------
CHICK    --------------------------------------------------
XENTR    --------------------------------------------------
DANRE    --------------------------------------------------
CIOIN    --------------------------------------------------
ANOGA    --------------------------------------------------
TETNG    --------------------------------------------------
DROME    --------------------------------------------------
BRUMA    --------------------------------------------------
CAEEL    EPMDYDEYGGQQSDNSMDYDSFSHQYDPSYEPLPFKPLHTDIPKKLTDDE
YEAST*   --------------------------------------------------
SCHPO    --------------------------------------------------
CANAL    --------------------------------------------------
CANGA    --------------------------------------------------
ASPNI    --------------------------------------------------
RHIO9    --------------------------------------------------
CRYNE    --------------------------------------------------
USTMA    --------------------------------------------------
ARATH*   --------------------------------------------------
VITVI    --------------------------------------------------
SOYBN    --------------------------------------------------
SOLLC    --------------------------------------------------
ORYSJ*   --------------------------------------------------
HORVU    --------------------------------------------------
SORBI    --------------------------------------------------
SELML    --------------------------------------------------
PHYPA    --------------------------------------------------
VOLCA    --------------------------------------------------
                  1110      1120      1130      1140      1150
```

```
HUMAN*   --------------------------------------------------
CHICK    --------------------------------------------------
XENTR    --------------------------------------------------
DANRE    --------------------------------------------------
CIOIN    --------------------------------------------------
ANOGA    --------------------------------------------------
TETNG    --------------------------------------------------
DROME    --------------------------------------------------
BRUMA    --------------------------------------------------
CAEEL    EFKYMKIGKRSVTPERISGRYGSMTYPFWIQQGGVGIDPPKDEVLDFSNP
YEAST*   --------------------------------------------------
SCHPO    --------------------------------------------------
CANAL    --------------------------------------------------
CANGA    --------------------------------------------------
ASPNI    --------------------------------------------------
RHIO9    --------------------------------------------------
CRYNE    --------------------------------------------------
USTMA    --------------------------------------------------
ARATH*   --------------------------------------------------
VITVI    --------------------------------------------------
SOYBN    --------------------------------------------------
SOLLC    --------------------------------------------------
ORYSJ*   --------------------------------------------------
HORVU    --------------------------------------------------
SORBI    --------------------------------------------------
SELML    --------------------------------------------------
PHYPA    --------------------------------------------------
VOLCA    --------------------------------------------------
                  1160      1170      1180      1190      1200
```

MED12

```
HUMAN*   --------------------------------------------------
CHICK    --------------------------------------------------
XENTR    --------------------------------------------------
DANRE    --------------------------------------------------
CIOIN    --------------------------------------------------
ANOGA    --------------------------------------------------
TETNG    --------------------------------------------------
DROME    --------------------------------------------------
BRUMA    --------------------------------------------------
CAEEL    TPTETVNTQPQIDFESSPSASPALSVDKENECEKKEDESKTKEKNKDKEK
YEAST*   --------------------------------------------------
SCHPO    --------------------------------------------------
CANAL    --------------------------------------------------
CANGA    --------------------------------------------------
ASPNI    --------------------------------------------------
RHIO9    --------------------------------------------------
CRYNE    --------------------------------------------------
USTMA    --------------------------------------------------
ARATH*   --------------------------------------------------
VITVI    --------------------------------------------------
SOYBN    --------------------------------------------------
SOLLC    --------------------------------------------------
ORYSJ*   --------------------------------------------------
HORVU    --------------------------------------------------
SORBI    --------------------------------------------------
SELML    --------------------------------------------------
PHYPA    --------------------------------------------------
VOLCA    --------------------------------------------------
                  1210      1220      1230      1240      1250
```

```
HUMAN*   --------------------------------------------------
CHICK    --------------------------------------------------
XENTR    --------------------------------------------------
DANRE    --------------------------------------------------
CIOIN    --------------------------------------------------
ANOGA    --------------------------------------------------
TETNG    --------------------------------------------------
DROME    --------------------------------------------------
BRUMA    --------------------------------------------------
CAEEL    DKEKEKSVDEHTNDLDVPINPDDAEMADANDKTDASEKQKLVEEEPTGKE
YEAST*   --------------------------------------------------
SCHPO    --------------------------------------------------
CANAL    --------------------------------------------------
CANGA    --------------------------------------------------
ASPNI    --------------------------------------------------
RHIO9    --------------------------------------------------
CRYNE    --------------------------------------------------
USTMA    --------------------------------------------------
ARATH*   --------------------------------------------------
VITVI    --------------------------------------------------
SOYBN    --------------------------------------------------
SOLLC    --------------------------------------------------
ORYSJ*   --------------------------------------------------
HORVU    --------------------------------------------------
SORBI    --------------------------------------------------
SELML    --------------------------------------------------
PHYPA    --------------------------------------------------
VOLCA    --------------------------------------------------
                  1260      1270      1280      1290      1300
```

```
HUMAN*   ---------------SHSSHVISAQSTST-LPTTPAPQPPTSSTPSTP--
CHICK    ---------------GHPPHILSTQTGNA-LPSTPTPQPATGNPPPSP--
XENTR    ---------------SHPGHLMTAQTGTA-LPPAPAPPPPSGSAPSSP--
DANRE    ---------------GHQAHGISAQQGNA-LPPTPTSQPAGGNQPQTP--
CIOIN    ---------------NSRSQSPALQSGNN-TPGTSTPQLTRPTSVQLK--
ANOGA    ---------------------QMAESHNI-NLSSPEPQDASGKQPKPPKV
TETNG    ---------------GHPGHSILAQQGNA-LPPTPTSQPSGGSQPQTP--
DROME    ---------------------TVVEQQTIKELDEPKLQQ-----------
BRUMA    ---------------AEEREGRSV--------------------------
CAEEL    NEDDTSSKTAKTSTSAEKSEAPSIVDSNDKIDKEPNASSTSNDETSKDDT
YEAST*   --------------------------------------------------
SCHPO    --------------------------------------------------
CANAL    --------------------------------------------------
CANGA    --------------------------------------------------
ASPNI    --------------------------------------------------
RHIO9    --------------------------------------------------
CRYNE    --------------------------------------------------
USTMA    --------------------------------------------------
ARATH*   --------------------------------------------------
VITVI    --------------------------------------------------
SOYBN    --------------------------------------------------
SOLLC    --------------------------------------------------
ORYSJ*   --------------------------------------------------
HORVU    --------------------------------------------------
SORBI    --------------------------------------------------
SELML    --------------------------------------------------
PHYPA    --------------------------------------------------
VOLCA    --DAAAANGLGAGNPTNTSGAAGAGASNSASPPGPSSPSGAVSPPLGYGA
                  1310      1320      1330      1340      1350
```

MED12

```
HUMAN*  --------------------------------------------------
CHICK   --------------------------------------------------
XENTR   --------------------------------------------------
DANRE   --------------------------------------------------
CIOIN   --------------------------------------------------
ANOGA   NPYE----------------------------------------------
TETNG   --------------------------------------------------
DROME   DPYE----------------------------------------------
BRUMA   --------------------------------------------------
CAEEL   VPMESDPPAATEKPKESTEITTEEPLEVDKAPEVDKSEKEHEDDIMIIES
YEAST*  --------------------------------------------------
SCHPO   --------------------------------------------------
CANAL   --------------------------------------------------
CANGA   --------------------------------------------------
ASPNI   --------------------------------------------------
RHIO9   --------------------------------------------------
CRYNE   --------------------------------------------------
USTMA   --------------------------------------------------
ARATH*  --------------------------------------------------
VITVI   --------------------------------------------------
SOYBN   --------------------------------------------------
SOLLC   --------------------------------------------------
ORYSJ*  --------------------------------------------------
HORVU   --------------------------------------------------
SORBI   --------------------------------------------------
SELML   --------------------------------------------------
PHYPA   --------------------------------------------------
VOLCA   TPAGNPVFGLTAVSAAAALGGNDTGAGG----------------------
                  1360      1370      1380      1390      1400
```

```
HUMAN*  --------------------------------------------------
CHICK   --------------------------------------------------
XENTR   --------------------------------------------------
DANRE   --------------------------------------------------
CIOIN   --------------------------------------------------
ANOGA   -------------------------------------------------M
TETNG   --------------------------------------------------
DROME   -------------------------------------------------L
BRUMA   -------DCV---------------------------------------E
CAEEL   NKKADEDDCVVIAVVDPDQEQTPESEKKKDGEEERDKNKDTDVADNEPEK
YEAST*  --------------------------------------------------
SCHPO   --------------------------------------------------
CANAL   --------------------------------------------------
CANGA   --------------------------------------------------
ASPNI   --------------------------------------------------
RHIO9   --------------------------------------------------
CRYNE   --------------------------------------------------
USTMA   --------------------------------------------------
ARATH*  --------------------------------------------------
VITVI   --------------------------------------------------
SOYBN   --------------------------------------------------
SOLLC   --------------------------------------------------
ORYSJ*  --------------------------------------------------
HORVU   --------------------------------------------------
SORBI   --------------------------------------------------
SELML   --------------------------------------------------
PHYPA   --------------------------------------------------
VOLCA   --------------------------------------------------
                  1410      1420      1430      1440      1450
```

```
HUMAN*  -FSD--------------------------------------------LL
CHICK   -FSD--------------------------------------------LL
XENTR   -FSD--------------------------------------------LL
DANRE   -FTD--------------------------------------------FY
CIOIN   -------------------------------------------------Q
ANOGA   IFTE--------------------------------------------YL
TETNG   -FTD--------------------------------------------FY
DROME   ALQE--------------------------------------------QM
BRUMA   LFDD--------------------------------------------MQ
CAEEL   IYTDEELAEIKIRKEKEMKIATLASHIKEKMVSDKKWKEKTSHAGWRTLD
YEAST*  --------------------------------------------------
SCHPO   --------------------------------------------------
CANAL   --------------------------------------------------
CANGA   --------------------------------------------------
ASPNI   --------------------------------------------------
RHIO9   --------------------------------------------------
CRYNE   --------------------------------------------------
USTMA   --------------------------------------------------
ARATH*  --------------------------------------------------
VITVI   --------------------------------------------------
SOYBN   --------------------------------------------------
SOLLC   --------------------------------------------------
ORYSJ*  --------------------------------------------------
HORVU   --------------------------------------------------
SORBI   --------------------------------------------------
SELML   --------------------------------------------------
PHYPA   --------------------------------------------------
VOLCA   --------------------------------------------------
                  1460      1470      1480      1490      1500
```

MED12

HUMAN*	M C P Q H - R - - P L V F G L S C I L Q T I L - - - - - L C C P S A L V - - - - - W H - Y S L - T D
CHICK	L C P Q H - R - - P V V Y G L S C I L Q S I I - - - - - L C C P S A L V - - - - - W H - Y S L - T D
XENTR	Q C P Q H - R - - P L V F G L S C M L Q S I V - - - - - L C C P S A L V - - - - - W H - Y S L - T D
DANRE	I C P Q H - R - - P V V F G L S C M L Q S I V - - - - - L C C P S A L V - - - - - W H - Y S L - T D
CIOIN	Q C P E H L L - - S I A H T L A C V I Q C T V - - - - - I T C P G A L V - - - - - W Y - - Q P - F D
ANOGA	Q C S H H - R - - D V I L Q L S S I L Q V I T - - - - - L E C P T A L V - - - - - W C - - G V - G E
TETNG	I C P Q H - R - - P I V Y G L S C M L Q S I V - - - - - L C C P S A L V - - - - - W H - Y S L - T D
DROME	S C P H H - R - - D I V L Y L S T I L Q I I T - - - - - I E C P T A L V - - - - - W S - - G I - A A
BRUMA	Q C I H Q - R - - A I I L T L C G M L Y A I L - - - - - I D C P A A L I - - - - - W N K Y E V - S P
CAEEL	Q C S Q F - S - - E A L H L L S S M V Q Y M A - - - - - C V T P E S F V - - - - - W N D L S V Q Q E
YEAST*	- - - - - - K N N K I K L N I L K I L S S L I L K I F Q E Q S L E V F I F P T S N W E - - - - - - -
SCHPO	- - - - - - K F S L L S K K V S H F L F T L A - - - - - Q K N I E S F F F P - T E W D - - - - - - -
CANAL	- - - - - - L Q S K I L K L I S D C V R Y L F - - - - - E Y N C N I F I I P - N H W T - - - - - - -
CANGA	- - - - - - K N N S I K E S L L A S I Q K L I C I L F K Q Q S L E V F L F P P T S W E - - - - - - -
ASPNI	- - - - - - Q - - P L A E R L S C C I R K L V - - - - - L E H T S S L I L P - N S W E - - - - - - -
RHIO9	- - - - - - - D Q R V H Q G L Q K D I E T V L - - - - - Q V S K E K K I - - - - - - - - - - - - - -
CRYNE	- - - - - - L L Q K V E D M L I T V I K A L Y - - - - - E A N P D V L L S P - S T W R - - - - - - -
USTMA	- - - - - - S S G G L R T K L V L L F R I V C - - - - - E N S P E S L I S P - R L W F - - - - - - -
ARATH*	- - - - - - R R A Y T L S A L I E M V R Y L V - - - - - L A A P D T F V - - - - - - - - - - - - - -
VITVI	- - - - - - R R A Y T S S A L V E M L R F L I - - - - - L A V P D T F V - - - - - - - - - - - - - -
SOYBN	- - - - - - R R A Y T A Y A V I E M L R Y L I - - - - - L V V P D T F V - - - - - - - - - - - - - -
SOLLC	- - - - - - R R A Y T M A A L V E M L R Y L M - - - - - L A V P D T F V - - - - - - - - - - - - - -
ORYSJ*	- - - - - - K R S S I T S A L V E L L Q Y L I - - - - - L A V P D T F V - - - - - - - - - - - - - -
HORVU	- - - - - - K R S S I S S V I A E L L R Y M V - - - - - L A V P D T F V - - - - - - - - - - - - - -
SORBI	- - - - - - K R P S V S S V I A E L L R Y M V - - - - - L A V P D T F V - - - - - - - - - - - - - -
SELML	- - - - - - Q Q Y R I V V T F V E I M R Y L L - - - - - V A V P D T F V - - - - - - - - - - - - - -
PHYPA	- - - - - - R N N R V A D T L V K L L Q Y L V - - - - - L V I P D T F V - - - - - - - - - - - - - -
VOLCA	- - - - - - L D S S V T E L A L E C L E G C V - - - - - R N A P A A V L - - - - - - - - - - - - - -

1510 1520 1530 1540 1550

HUMAN*	S R I K T - - - - - - G S P L D H L P I A - - - - P S N L P M P - - - - - - E G N S A F T Q Q V R A
CHICK	S R I K T - - - - - - G S P L D H L P I A - - - - P S N L P M P - - - - - - G G N S A F T Q Q V R A
XENTR	N R I K T - - - - - - G S P L D H L P I A - - - - P S S L P M P - - - - - - G G S S A F T Q Q V R R
DANRE	S R N K T - - - - - - G S P L D L L P I A - - - - P S N L P M P - - - - - - G G N S T F N Q Q V R A
CIOIN	A K A S - - - - - - - G S P L D L L P C A - - - - P S D L P L P D I P K W D T E A L E T D R L T R L
ANOGA	T R S S S V - - - L S G S P L D H L P V A - - - - P S A L P I P - - - - - - T R Y E K S N E D I R R
TETNG	C R N K T - - - - - - G S P L D L L P I A - - - - P S S L P M P - - - - - - G G N T A L T Q Q V R T
DROME	H R A P S S - - - L L G S P L D H L P L A - - - - P S V L P M P - - - - - - T R C P R T N H E I R R
BRUMA	G R P P T M L H Q L C G S P L D H L P C P - - - - F E S L P L P - - - - - - P G H G T E R L Q E F I
CAEEL	E R R H R I L P Q L C G S P L D Y L P C E - - - - L H K L P V - - - - - - - - M E G V E E V V D C L
YEAST*	- - - - - - - - - - - - - - - I Y K P L L - - - - F E I V S N - - - - - - - - A D T N Q N S D M K K
SCHPO	- - - - - - - - - - - - - - - K L K P T I -
CANAL	- - - - - - - - - - - - - - - L I E P S L - - - - M R I L L L - - - - D S K A K S E K E Q E E I Q K
CANGA	- - - - - - - - - - - - - - - K Y K P C L - - - - I Q I T S A L - - - - - - Y R K S D E V A E I K R
ASPNI	- - - - - - - - - - - - - - - T H R D L I - - - - L S C L N M K E T A D K - - - - - - - - - - - - -
RHIO9	- - - - - - - - - - - - - - - - K K P L F S T F Y H H L L N V I I G E E A T S R S T K A S S D M R R
CRYNE	- - - - - - - - - - - - - - - L H S P L L - - - - A T I L S T S S - - - - - - - - - - - - - - - - -
USTMA	- - - - - - - - - - - - - - - E H S A S L - - - - T Q L L N E L N A E T P L P L S H Q A F E - - - -
ARATH*	- - - - - - - - - - - - - A S D F F P L - - - - - P P S V - A A C G P N D V S Y T S K A Y E N L E K
VITVI	- - - - - - - - - - - - - A L D C F P L - - - - - P P C V - V S H V A N D G S F L T K V S E D T I K
SOYBN	- - - - - - - - - - - - - A L D C F P L - - - - - P S S V - I S H T M N D G N F V L K S T E A A G K
SOLLC	- - - - - - - - - - - - - A L D C F P M - - - - - P P C V - M T N V V T D G S L Y S K V T E D T R K
ORYSJ*	- - - - - - - - - - - - - S L D C F P L - - - - - P S V V - A P D V Y G K G A L L - - - - - - - - K
HORVU	- - - - - - - - - - - - - S L D C F P L - - - - - P S F V - A P D V Y G R G A L L - - - - - - - - K
SORBI	- - - - - - - - - - - - - S L D C F P L - - - - - P S F V - V P D V Y G R G A L L - - - - - - - - K
SELML	- - - - - - - - - - - - - A F D C F P L - - - - - L P C V T T L E G M E I V K D G - D P K A D T T K
PHYPA	- - - - - - - - - - - - - A L D C F P L - - - - - P Q C V - S L G G S E S S A I E - S S S N D L G R
VOLCA	- - - - - - - - - - - - - S L D S L P L L - - - - V R A L K A P W P P A S G A A A A A A A G P I G V

1560 1570 1580 1590 1600

HUMAN*	K L R - E I E Q Q I K E R G Q A V E V R W S F D K C Q
CHICK	K L R - E I E Q Q I K E R G Q A V E V R W S F D K C Q
XENTR	K L R - D M E Q Q I K E R G R S V E V R W S F D K C Q
DANRE	K V R - E I E E Q I K E R G Q A V E F R W S F D K C Q
CIOIN	L L Q - E Q E A D I V R R S R A S E M K W S T D K C Q
ANOGA	Q L F - E A E E S I K V R S R H A E S R W C I D K W Q
TETNG	K L R - E I E E Q V K E R G Q A V E F R W S F D K C Q
DROME	Q L R - A A E S D I V L R T Q H A E Q R W F A A K W L
BRUMA	Q L R - L A E - - V R R R S R A C E N K W S L N Y A Q
CAEEL	R L R - H C E - - I V R R S Q A A E D R W L P N A A F
YEAST*	K L E - L I S Y R N E S L K N N S S I R N V I
SCHPO	- - - - - - - - - - - - - - - - I L L W K D F P N Y S T L L S I M Q E R N S K A M Y M Y K P V T S S
CANAL	Q L E - L V K Y R N E S L I L N L K S E L N N
CANGA	K L E - L I S Y R N E T L K Y T P P I - - - -
ASPNI	- T I F D S L A E R N S R I Q L P S S R S D S T
RHIO9	V M N - E Y W A M I K A R N E T F C G K V D K S P V G
CRYNE	- P Q W E N L K R R N E A L A F K P T V T A G S
USTMA	- F V E Q T V R P R V D R L L L R P H S G N T T
ARATH*	L R S N S A E I S A Q F Q G R G V L S R F E F L S F D Y T I S T I Q R S A D D L A K I A S A G Y P Q
VITVI	I K N R P A E V P T V L R D K V L D G Q Y P S L S F D H I V S S I Q K R A D N L A K A A S P G Y P C
SOYBN	I K N - - - - - - - - - - - - - - - - - S S D D F G H I I S C I Q K H T E D L V K A A S P G Y P G
SOLLC	V K N G P F E V A Y F L R D K G P E I R S D S Y S I S R A V S S I Q K R A Q H L A T A A R P G H P G
ORYSJ*	I A G G G K I A S S R R Q N - - - - - A S R H L S C G Y A I C S V Q R R A S D L S L V A N P N L Q V
HORVU	I T G G G E M S S S K R R D - - - - - V Y R Y L S C G Y A V C S I Q R R A S D L A T L A S P N V H A
SORBI	I T S G G G I S S S K R R D - - - - - A Y R Y L S C G Y A V C S I Q K R A Y D L A T V A N P N L Q A
SELML	G N G R P G N L C - - - - - - - - - - P Y R E V Y I M E V V D S I Q K R A S S L T K A V N P G V L R
PHYPA	V N S G K R K - - - - - - - - - - - - T Y S E V G V V E S V D L I Q K R T A S L A S A V S P T L L R
VOLCA	S G G - - - - - - - - - - - - - - - - - E D A A A L V A A A A I V C P R R R A M A D K L A A A H A S

1610 1620 1630 1640 1650

MED12

```
HUMAN*  EATAGFTIGRVLH---------TLEVLDSHSFER---SDFSNSLDSLCNR
CHICK   ETTAGFTIGRVLH---------TLEVLDSHSFER---SDFSNSLDSLYNR
XENTR   EATAGFPIGRVLH---------TLEVLDSHSFEK---SDFSNNLDSLYNR
DANRE   ETTAGFTISRVLH---------TLEVLDNHSFEK---SDFSNSLDSLYNR
CIOIN   ESTTGHTINRVLS---------ALEALDKQHFDRRPADSWGDPLDLLYHK
ANOGA   -TAAGNASLKILA---------TLDALDGHCFDR---MDSNNSLDSLYSK
TETNG   ETTAGFTIGRVLH---------TLEVLDSHSFEK---SDFNNSLDLLYNR
DROME   -SAGKNQYTSVLA---------TLDHLDTHCFDR---MEHNNSIDTLYAQ
BRUMA   KKGFAALVLQCLE---------IVGVLDAARLDQ------PNCVEKIYAV
CAEEL   LQSFGRIIDTCVD---------VIGIMDNIDVEK------PNAITNAGLR
YEAST*  MSASN-----------------ANDFQLTIVTCK--------QFPKLSCI
SCHPO   IR--------------------FLQIISCLSFPV--------GWRTLAKD
CANAL   SNNTN----------------------NSSNLGT--------SKRSVRSG
CANGA   -SENK-----------------VKDDLILTTFTE--------NSGSITVV
ASPNI   RQSAHQR---------------VIQLFDSIQSSQ--------DIASTSVA
RHIO9   QKTSQSTHESGGGDEE-----RIVTLLDSIGK----------NIDSVSGL
CRYNE   SNPRRQRME-------------EIQKLDSICKDT--------DMRQLTFL
USTMA   QNASLETQETLSSPLQHHLDIQALDSFDMTTIDR----------------
ARATH*  HNVAK-----------------AVQALDKALSDG--------DIRAAYSY
VITVI   HSEAK-----------------AVQALDKALVDG--------DVRGAYKF
SOYBN   HCLAK-----------------VAKALDKALVLG--------DLRVAYKF
SOLLC   QSVVK-----------------ALHALDKALAHG--------DLREAYKF
ORYSJ*  RGAAN-----------------VVQALDKALVTG--------NLTAAYTS
HORVU   RGAAK-----------------VVQALDRALVTG--------NLTMAYSS
SORBI   RGAAK-----------------VVQALDKALVTG--------NLSVAYSS
SELML   NNDGK-----------------VIQALDKSLAAG--------DLLSAFQA
PHYPA   NNEGK-----------------IVQALDKALEKG--------DIAGAYRS
VOLCA   LATSVNTRLLAFNPVA------ALRDLDRAAAAG--------DVDAGMAT
                  1660      1670      1680      1690      1700

HUMAN*  IFGL------------GPSKDGHE--IS----------------------
CHICK   IFGL------------GPTKDSHE--IS----------------------
XENTR   IFGL------------GLGKDGHE--IS----------------------
DANRE   IFGS------------GQSKDGHE--MS----------------------
CIOIN   IFDQ------------GHGKDGDNQLTK----------------------
ANOGA   IFPP---------FQVQPLKPAES--AS----------------------
TETNG   IFGS------------GQSKDSHE--MS----------------------
DROME   IFPSPTVSRRREEDQVEPRPPYEP--KQ----------------------
BRUMA   IFN-------------GPSKESFE--------------------------
CAEEL   LF---------------AFREKFE--------------------------
YEAST*  QLNCIDTQFTKLLDDNPTEFDWPTYVDQ----------------------
SCHPO   LFKLLPV-------------------------------------------
CANAL   SFSNYTTTPIKPISNTTIEQDYSFINRNSDDVLNIVEKLDNLKLNKEFAS
CANGA   ELSQVDTGLTGALDDNATDFDWTSYSDQ----------------------
ASPNI   CLN-----------------------------------------------
RHIO9   QINGETWT-----DAHGHSAKW-------------------ASKTIFGTA
CRYNE   FFDGAASPTCSPVDLVKF--------------------------------
USTMA   VFLQNTSATHRYAKHDRDDTVW----------------------------
ARATH*  LFED------------------LCNG--AVDEAWITDVSPCLRSSLRWIG
VITVI   LFDD------------------HCDG--AVNEGWIAEVSPCLRSSLKWIG
SOYBN   LFED------------------LCGG--TVSEGWISKVSPCLRLSLKWFG
SOLLC   LFEN------------------VHES--SIDDCWFAEVSSCLRSSLKYIR
ORYSJ*  VFNY------------------LSDT--LMEETWIKEVSPCLRSSLMWMG
HORVU   LFND------------------LSDA--LMEERWIKEVSPCLQSSLMWIG
SORBI   LFND------------------LSDA--LMEERWIKEVSPCLQSSLMWIG
SELML   VFDS------------------DFSGYDQLPGEWKTSSSN-LPFLSGMLN
PHYPA   VYDD------------------EFCSSDNLPADWRADVTS--RTAFNLSG
VOLCA   LAQACWGDVSRPEPAVQLLGNWTVGLPIVLPPVLVPPVSPSVSLQAAATA
                  1710      1720      1730      1740      1750

HUMAN*  -SDDDA-------------VVSL--LC-EWAVSCKRSGR-----------
CHICK   -PDDDA-------------VVAL--LC-EWAVSYKRSGR-----------
XENTR   -PDDDA-------------VVTL--LC-EWAVSCKRYGH-----------
DANRE   -PDDDA-------------VVTL--LC-EWAVSCKRSGP-----------
CIOIN   -SSEDA-------------VIGL--LC-EWAISPKRLGE-----------
ANOGA   -AADSS-------------IVKI--LC-EWAVSWQRWGE-----------
TETNG   -PDDDA-------------VVTL--LC-EWAVCCKRSGR-----------
DROME   ---DKD-------------TVRI--LC-EWAVSGQRWGE-----------
BRUMA   -HEHAI-------------RVKM--LL-QWAVTVEREGT-----------
CAEEL   -KQEAL-------------LKTM--LMFKWCITEEREGS-----------
YEAST*  --NPLT-------------MHKIIQLI-LWSIHPSRQFDHY---------
SCHPO   -------------------YTGVPLLL-HWCINCRSIFSG----------
CANAL   FLKPTKKCNANW-------KLNLKVLL-FWCVSPWRNSTNSN--------
CANGA   --EIST-------------KIQIIQII-LWAIHPSRH-SHY---------
ASPNI   -ALEDR-------------QTLVIKLL-EWSATSFRSGL-----------
RHIO9   RLEPDR-------------MIKLIHLVCRWAVSESRYGD-----------
CRYNE   -------------------EEKVFTLL-NWSMGLFQLGA-----------
USTMA   -------------------ATRIDTIL-TWACTDRRSGV-----------
ARATH*  AISTSF-------------VCSVFFLI-EWATCDFRDFRAGVPKDI-KFS
VITVI   TVSSSL-------------VCSVFFLC-EWATCDFRDFRTAPPHDM-KFT
SOYBN   TVNTPL-------------IYSVFFLC-EWATCDFRDFCSTPPRDI-KFT
SOLLC   GVTLSS-------------ICSVFYIC-EWATCDFRDFRFAPPRGM-KFT
ORYSJ*  AVELSL-------------VCSVFFIC-EWATCTFRDCRTSQCQNV-KFS
HORVU   TVELSL-------------ICSIFFLC-EWATCDYRDCRTSPSQNV-KLT
SORBI   TVELSL-------------ICSIFFLC-EWATCDYRDCRASPSQNV-KFT
SELML   NVNLSD-------------LFAVRYLC-EWAVCDFRDTRQASSIKV--ST
PHYPA   PVNPSE-------------LYPVRFLC-EWAVCDFRDCRGIVSKTIRKQS
VOLCA   ATDNPAARADQVDMGLACRTVYACRLMVRLAAECRR--------------
                  1760      1770      1780      1790      1800
```

MED12

```
HUMAN*  -----HRAMVVAKLLEKRQAEIEAERCGESEA-----ADEKGSIASGSLS
CHICK   -----HRAMVVAKLLEKRQAEIEAERCGDSEV-----VDEKGSISSGSLS
XENTR   -----HRALVVAKLLEKRQAEIEAERCGDSEA-----VDEKGSVSSGSLS
DANRE   -----HRAMVVAKLLEKRQTEIEAERCGESEV-----VDEKGSVSSGSLS
CIOIN   -----HRAAVVAKLLELRQSHHEQVEYPDTTI----ETDTSLNTSVGGVI
ANOGA   -----HRAMVVAWLLDKRQNEVLTALENDSYTNNLNNSDDKDSVLSGSGL
TETNG   -----HRAMVVAKLLEKRQAEIEAERCGDSEV-----VDEKGSVSSGSLS
DROME   -----HRAMVVAILLDKRQIDVTSTPADQQSS----DKDDKDSLASGAGL
BRUMA   -----HRAILVAQLLAFR-------------------INHRKTFKFGSLT
CAEEL   -----FRATYIAKLLRFG-------------------MDQNPENTIGGWQ
YEAST*  -----ESNQLVAKLLLLRINSTDEDLHEFQ--------------------
SCHPO   -----DRNFIVSSIFDNANFDRNL--------------------------
CANAL   -----EDILFICNFLKNTVVKSLPKSLRTE--------------------
CANGA   -----EAGQLAAKLLLLKINTMD-GFPEYE--------------------
ASPNI   -----HRVYTAARLLRKWKISGVD--------------------------
RHIO9   -----WKAYLVASILSSWKEENGSPEYKTL--------------------
CRYNE   -----HRPYAVYTLLKHWHEQQEEHQASQAKLGKRQHID-----------
USTMA   -----VRQYLAATLIEKIRFGVPWEESSEETDQVSLPAFDLEHNTTKTRK
ARATH*  GRKDCSQVYLVIQLLKQKILGGEF--------------------------
VITVI   GRKDFSQVYIAIRLLKLKLRDVQN--------------PGCCKNNSTAGI
SOYBN   GRKDLSQVHIAVRLLKMKIRDVKI---------------SQKQTNENHRA
SOLLC   GRKDFSAIYVAVRLLKLKMRETGI--------------SSRPRDPKIVKN
ORYSJ*  GSKDFSQVYMAVSLLKDKMNEINN--------------LSSSKSSSQLAM
HORVU   GRRDLSQIHLAVSILKNKMTEMNK--------------LSRSKSSSRIAV
SORBI   GRRDLSQIHIAVSILKNKMDEMNN--------------LSRSKSSTRVTL
SELML   GIKDLSRIYTAASVLLLKK---DFPPGSHAKKARVKGASDSK--------
PHYPA   ACRDMSRVYVAVSVLRMRLAEMDSKIGPHSPDTRLPEHFDSCDPLSRGGL
VOLCA   -----SRVEAAAAASQGLSPPVPPPLASASNIGAQAGTASAAAGPATAGG
                 1810      1820      1830      1840      1850
```

```
HUMAN*  ---------------------------------APSA-PI----------
CHICK   ---------------------------------AASA-PV----------
XENTR   ---------------------------------ALSAPPV----------
DANRE   ---------------------------------AATL-PV----------
CIOIN   ---------------------------------NTSA-PINNYQPNNHTN
ANOGA   ---------------------------------NGGQ-PV----------
TETNG   ---------------------------------AATV-PV----------
DROME   ---------------------------------IDGL-PV----------
BRUMA   --------------------------------------------------
CAEEL   --------------------------------------------------
YEAST*  --------------------------------------------------
SCHPO   --------------------------------------------------
CANAL   --------------------------------------------------
CANGA   --------------------------------------------------
ASPNI   --------------------------------------------------
RHIO9   --------------------------------------------------
CRYNE   --------------------------------------------------
USTMA   IN------------------------------------------------
ARATH*  -TARKGKNCRNNFLGVSKPSG-----------------SMDAFESPGP--
VITVI   NTLAKGSSQPNNNSGRISVVNAYENKNNLKNMDRASIDSLDIFQSPGP--
SOYBN   SHLAKHSSQRHNW---NYVGNVSRLRSSSKST------GSSVFESPGP--
SOLLC   NHLRKDPGQLTNYAGRTLASGASESLSNSRRAREKCNDFLGMFDSPSP--
ORYSJ*  KDHLKSATLNHSSIKVTAMETASGFRDSTGSIDE--NNKKDIFSSPGP--
HORVU   NNIGKGSSLNDASVAATKVGDSSGLRSNGKNEEEK-KEKKDIFESPGP--
SORBI   NTITKGSSLTDACLTAATADDSSGLRSKAKNVDDK-KDTNDIFESPGP--
SELML   -------------------------------------------LKRSP--
PHYPA   PVHVRNRKSKKGQAVHAPMDHSVEFSSGNFSALHNHSHNSIA--------
VOLCA   AQGTLRTGPGPIPPGVQPLAAAPGSAGPPGTTRGSRAGSAGGISSVDFGA
                 1860      1870      1880      1890      1900
```

```
HUMAN*  ----FQDVLLQFLDTQA-PMLTD-PRSESER---------------VEFF
CHICK   ----FQDVLMQFLDTQA-PMLTD-PGKENEK---------------VEFF
XENTR   ----FQEVLLQFLDTQA-PMLTD-PANESER---------------VEFY
DANRE   ----FQDVLLQFLDTQA-PVLTE-PGNESER---------------VEFS
CIOIN   NVPYFQNLLFTFLDQQA-PELKN-PKDGEET---------------QSFK
ANOGA   ----FQHILMNFLDHDA-PVLDE-AGSPQNK---------------SQFT
TETNG   ----FQDVLLQFLDTQA-PTLTE-PGNESER---------------VEFS
DROME   ----FQHVLMHFLDHDA-PVLDEHVSSPQQR---------------TEFT
BRUMA   ----LQEILLEFLVTEG-PDPEN-TDKNRFS---------------QEFA
CAEEL   ----VMDLFFKFMSTEG-P---K-HGSKMYQ---------------AHFD
YEAST*  ----IEDAIWSLVFQLA-KNFSA-----------------QKRVVSYMMP
SCHPO   ----IVDLTLSFVLKLH-PLEYN------------------------ECV
CANAL   ----FDNELLEIIYHIAEDNNSEEGDNLQ-----------------FTSS
CANGA   ----IEDEIWSLVFKLA-K-LSD-----------------KSKALPAVLE
ASPNI   ----IDTYIISFLSTVQ-DLAQ------------------------LDMD
RHIO9   ----LQEALIQFLDGEV-DPESMSCDEDEAFRIEGEEDHTK-----RPNV
CRYNE   ----LFQVVYKWLDTAP-AARNE-----------------------DNVR
USTMA   ----VEPMLIKWLGDVE-TSLNQKLDESSTQTRVSLLAT-------VDVG
ARATH*  ----LHDIIVCWIDQHE-VHKG------------------------G-AK
VITVI   ----LHDIIVCWIDQHE-AHKGE-----------------------G-FK
SOYBN   ----LHDIVVCWIDQHV-VQKGE-----------------------G-PK
SOLLC   ----LHDTIVCWIDQHE-VQNTE-----------------------G-FK
ORYSJ*  ----LHDIIVCWLDQHE-ISDAS-----------------------G-FK
HORVU   ----LHDIIVCWLDQHE-VSSAA-----------------------G-FK
SORBI   ----LHDIIVCWLDQHE-VSSVA-----------------------G-FT
SELML   ----MHEIIVAWIDQHD-SRKGE-----------------------S-ME
PHYPA   ----IHDLIYAWLDQHE-LWQGD-----------------------S-SD
VOLCA   SAPVLQDAIVSWLVSRPGPWRRSSGLAGDGAAAAAAGGAAVGGWQELHLR
                 1910      1920      1930      1940      1950
```

MED12

	1951–2000
HUMAN*	NLVLLFCELIRHDVFSHNMYTCTLISRGDLA--------FGAPGPRPPSP
CHICK	NLVLLFCELIRHDVFSHNIYMCTLISRGDLA--------MDSHGPRPPSP
XENTR	NLVLLFCELIRHDVFSHNMYMCTLISRGDLG--------IDPHAPRPRSP
DANRE	NLVLLFYELIRHDVFSHNIYMCTLISRGDLA--------SNSHLPRPRSP
CIOIN	NLVLLFSELIEHDVFSHDIYMNTLISRGEIS--------PGDIGPLPSFN
ANOGA	NLVHLFSELIRHDVFSHDAYMCCLISRGDLLTGAGGMLSLDSQGICNAGL
TETNG	NLVLLFCELIRHDVFSHNIYMCTLISRGDLA--------SDSHLPRPRSP
DROME	NLVQLFSALIRHDVFSHNAYMHTLISRGDLL--------LESVLVIKSG-
BRUMA	NLMLLFIELQRAGVFSHDLYMKELIRTGEML----------EYIPLMQKI
CAEEL	STVAIMIEMMREKIFSITDILRELEKDSDLD----------YNAPLMERQ
YEAST*	SLYRLLNILITYGIIKVPTYIRKLISSGLLY-------------------
SCHPO	AAAQLLDHLAACGYFFFSKYIARLASLGYLR-------------------
CANAL	RLYVLINELYQLKLITIASYLRKLIASGIFY-------------------
CANGA	ALYSLLNTLIVYGLIKVSTYIRKLISSGILY-------------------
ASPNI	NVYHIISELVRSQTFSVSRYLQWLMAKGVAR-------------------
RHIO9	HIVYLYDVLIRSSLFSYQKYLLRLIARGDLE-------------------
CRYNE	AIGITIGELTRRGMFSYGRYLQTLIANGHTA-------------------
USTMA	AIVVLLGELARRGVFSYTKYLQRLIARGMTA-------------------
ARATH*	RLQLLVFELIRSGIFNPIAYVRQLIVSGMID-------------------
VITVI	RLQLLIMELARSGIFYPQVYVRQLIVSGIMD-------------------
SOYBN	RLNLFMVELIRAGIFYPLAYVRQLIVSGIMD-------------------
SOLLC	RVQLLIIELIRAGIFYPQAYVRQLIVSGIMD-------------------
ORYSJ*	SVDVFMTELIRSGIFYPQTYVRQLIVSGITI-------------------
HORVU	RVDVLIIELIRSGVFYPQAYVRQLIISGVTD-------------------
SORBI	RVDLLIVELIRNGIFFPQAYVRQLIISGITD-------------------
SELML	KMQLLLYELVRRGIFHPDAYVRQLIVEGLLE-------------------
PHYPA	RLPLLLSELVREGLFSPEAYVRQLLCNGVLD-------------------
VOLCA	RTVQFLVCMTEAGVFSPTAFVQRLLVDGVFD-------------------

1960 1970 1980 1990 2000

	2001–2050
HUMAN*	FDDPADDPEHKEAEGSSSSKLEDPGLSESMDIDPSSSVLFEDMEKPD---
CHICK	FDDPAEEHDRKETEGNSGIKLEDTGLSEPMDIDHNSSTIFDDMEKTD---
XENTR	YEDPSDDTDRKEGEANSSIKLEDTTLSETMDIDHGSGALFEDMEKSE---
DANRE	SDEPSDESERKDQDAGSSVKMEDTGMSESMEIDHNSSANFDE--------
CIOIN	TGSVDGQMDSNQHLSN-LPNFNNKQMEVKME--EEDPRCPDVGSMDFLTS
ANOGA	GTGPISNKPATSSSPNGGGGVEDDVLQTDFKAKLED---LDDSNVDDDL-
TETNG	SDEPSDESERKDQEAGSNCKNE----AKSMEIDHNSSANFDEVQRDDNFS
DROME	TTATKTSPPPPAPPPTTTHGFDDDGFGGGLDFKHNE---FDDSNVDDDL-
BRUMA	RKRKEDKQKQHQQLSN-TVAFPSTFVSQ-----------PNETTAALLTG
CAEEL	RKQKIPKISKRHRKPE-T---------------------PDDTK------
YEAST*	--
SCHPO	--
CANAL	--
CANGA	--
ASPNI	--
RHIO9	--
CRYNE	--
USTMA	--
ARATH*	--
VITVI	--
SOYBN	--
SOLLC	--
ORYSJ*	--
HORVU	--
SORBI	--
SELML	--
PHYPA	--
VOLCA	---------------------------------------PVPAAMDYWPD

2010 2020 2030 2040 2050

	2051–2100
HUMAN*	-FSLFSPTMPCEGKGSPSPEKPDVEKEVKPPPKEK-IEGTLGVL--YDQP
CHICK	-FSMFSPPMHCESKASPSPEKPDPEKEAKPLLKDKSAEGMLASL--YDQP
XENTR	-FPMFSPAMRCEGR-SPGPE--DGDRDIRRTVKGLNGEGPLSAL--YDQP
DANRE	---MFSPPMHCESKGSPSPEKQAQEQESKSTAKDKGMDPAFPLV--YEQP
CIOIN	NESVFSPGECRSDRHFLDAVRSSMENPNKSSANFDASDGSYSNIWGHGQP
ANOGA	-DKLLQHIKEDQQNSMDAPDSPKDPEHAAP------SVTGLGKA--ESSS
TETNG	FADMFSPPMHCESKGSPSPEKPPPEQDSKAGSKDKGMDPAFPQL--YDQP
DROME	-DKLVQNIKEKGQQH-EAPDSPK---IGPP------GDGETNPG--GSIS
BRUMA	AVTTPPPLVTMPSELPDE-----------------------HPFLFKDLS
CAEEL	-------LVHFTTEYTPK-----------------------RLFMGKKMD
YEAST*	---------------------LQDSNDKFV--------------------
SCHPO	---------------------ESMLNSSFMDD------------------
CANAL	---------------------LEPGSTVDQSLNN----------LPRIME
CANGA	---------------------LTESNDKYI--------------------
ASPNI	---------------------KSSGTSGEVLAA-----------------
RHIO9	---------------------PRRRNEHTIQQ------------------
CRYNE	---------------------RNLRSASDDRSH-----------------
USTMA	---------------------PSASSATEQHQNPDGSVQTPSSVSASRDS
ARATH*	---------------------VIQPAVDPERRMR----------------
VITVI	---------------------RYGPIVDLDRRKR----------------
SOYBN	---------------------VNVNVVDLERQRR----------------
SOLLC	---------------------GDEPLSDPVKQKR----------------
ORYSJ*	---------------------WNDSLFDLEKKTR----------------
HORVU	---------------------KNGTLLDMERKRR----------------
SORBI	---------------------KNDTMLDVERKRR----------------
SELML	---------------------RRESPSDISRAAR----------------
PHYPA	---------------------RQSTATEVARASR----------------
VOLCA	MRTAVAAGPAIAPAVANSLPAEVPPACGEGGGSGSYSRDDAAALALYLQH

2060 2070 2080 2090 2100

MED12

```
HUMAN*  RHVQYATHFP------------IP-QEESC----SHECNQR---LVVLFG
CHICK   RHIQYATHFP------------IP-QEESC----SHECNQR---LVVLFG
XENTR   RHIQYATHFP------------IP-QEESC----SHECNQR---LVVLYG
DANRE   RHIQYATHFP------------IP-QEESA----SHECNQR---LVVLYG
CIOIN   RHVQYLMHFP------------ISFQEDGM----TQEWNQR---LMVLYG
ANOGA   RHFLYTEHFP------------LC-QDDPIS---QHDCNQR---YILLYG
TETNG   RHIQYATHFP------------IP-QEESS----SHECNQR---LVVLYG
DROME   RHYVYTKHFP------------IP-QDDPSMSSYSSESNQR---YILLFG
BRUMA   VHERLLIHLP------------IP-QSAEY----RSECNQR---SLLLYG
CAEEL   LLERMIIILP------------QL-DVDED----TDEYRLR---RLLLFG
YEAST*  -HVQLLINLK---------ISPLM----------KSQYNMV---LRNVME
SCHPO   -QRKILVQLP---------ILRMS----------QQLKNKI---YYILSK
CANAL   VHLKILENLP---------VLNNR----------QCDSILK---RWTPMG
CANGA   -HCRLLINLK---------MSPLM----------KSQYNMV---LKNVMD
ASPNI   -DARLLTQLP---------MSRSP----------EHVRNLR---LTLLAR
RHIO9   -RLHYLASFP--------LLTPAP----------AYLINQR---RVALYG
CRYNE   -HLALLKSMP---------VFVMA----------KDLLQQR---RIALSG
USTMA   LHLRLLRSLP---------LYDQP----------ASVYQQR---RQAIYG
ARATH*  -HHRILKQLPGCFVHETLEEAQLFGGDKLSEAV-RTYSNER---RLLLRE
VITVI   -HYRILKQLPGSYMRDALEEGAQVTEVGLLSDAI-LIYSNER---RLVLQG
SOYBN   -HYRILKQLPGCFIHDVLEESGIVEGSQLKEAL-QIYLNER---RLILRG
SOLLC   -HCKILKHLPGPYVHDALEEARIADTPVLSEVV-NVYCNER---KLVLHG
ORYSJ*  -HYKILKHLPGFCLFNILEEAKIAEDQVLYEIV-STYSSER---RLVLSE
HORVU   -HHRILKQLPGSSLFDILEEDIVAEQQQLHEVL-STYSSER---RLVLSE
SORBI   -HHRTLKQLPGSSLFDILEETRTAEEQQLYEMM-STYSSER---RLVLSE
SELML   -HRRVLRYLPGLP---TLDLDDTKDTDDIPENA-RLYRNER---RLALHG
PHYPA   -HRHVLQQLP---FPGGLIGDAVSNIDSRQSVAFRMYRIER---RLALDG
VOLCA   LHPHLVAALD----------------EQAAATAAAMASNVRPGFRRTLGG
                 2110      2120      2130      2140      2150

HUMAN*  VGKQRDDARHAIKKIT----------------------------------
CHICK   VGKQRDDARHTIKKIT----------------------------------
XENTR   VGRQRDDARHIIKRIT----------------------------------
DANRE   VGKQRDEARHAIKKIT----------------------------------
CIOIN   AGRLKTDVKRAVRKVT----------------------------------
ANOGA   VGKERDEKKHAVKKMS----------------------------------
TETNG   VGKLRDEARHTIKKIT----------------------------------
DROME   VGKERDEKKHAVKKMS----------------------------------
BRUMA   IGAERDAVKLELRRLA----------------------------------
CAEEL   LKPAANVYFRRARAIY----------------------------------
YEAST*  YDVKFYEIFNFDQLVE----------------------------------
SCHPO   GNYFVD--------------------------------------------
CANAL   FDFKGKFSQGQSILQD----------------------------------
CANGA   QDPEYYNNYNFDQIVS----------------------------------
ASPNI   AGVSVDEESSTIKCLK----------------------------------
RHIO9   TRNDSNNKETETEALN----------------------------------
CRYNE   DDLEVRRRDELEEKAV----------------------------------
USTMA   DRTKETYEDAAQRRAL----------------------------------
ARATH*  L---LVEKGKYWNNLVLSDQKSKKISTSLSSVIFPR-ACNAKSNSKGP--
VITVI   L---LWDQYKSKNIASISSRRPKHLPVSARDGASPA--------------
SOYBN   H---LSVSCGS----NLSALKKKKYPASTKDEVFAVPIDQRNVISTTI--
SOLLC   M----IDSYNSGCGSSHHKHKPR--PNSGENLSAPS-IDQLSSSESGPFM
ORYSJ*  L---SSGL-------ATDANVEGRVPLSS-----CLQKQPDLLMDSTD--
HORVU   L---SSGQ-------SFDASNRGGYTSSS-----YPR------IPSGG--
SORBI   L---SCGH-------SFYASGRGGYAASS-----CIRKQSDLPVASGG--
SELML   F---HSDDKIAKDRRLKDRTYSSEVKVPMSGSFVTNTLDR----------
PHYPA   LGIRRHEQHIQGTGATGSIVRREGSKGSESRPSVHEFRDR----------
VOLCA   SVAAFGQQQQQQRVGG------------SSGGASVLAEAAELAGSSGPVS
                 2160      2170      2180      2190      2200

HUMAN*  --------------------------------------------------
CHICK   --------------------------------------------------
XENTR   --------------------------------------------------
DANRE   --------------------------------------------------
CIOIN   --------------------------------------------------
ANOGA   --------------------------------------------------
TETNG   --------------------------------------------------
DROME   --------------------------------------------------
BRUMA   --------------------------------------------------
CAEEL   --------------------------------------------------
YEAST*  --------------------------------------------------
SCHPO   --------------------------------------------------
CANAL   --------------------------------------------------
CANGA   --------------------------------------------------
ASPNI   --------------------------------------------------
RHIO9   --------------------------------------------------
CRYNE   --------------------------------------------------
USTMA   --------------------------------------------------
ARATH*  -RKHTKSSVDIRELKERISALLQFPG-MSCGVETPVRDEFQNSVKRSSGS
VITVI   ----------------------SLPN-SSTTSADTGLDESQGSVKKSVGS
SOYBN   -SSKNAKDTNIEELRTAISVLLQLPN-CSSNLSTTG-DESEGSDRRAIGS
SOLLC   SSKNVGRDVELEELKRSITALLQFPS-SSS--TDTGVEDSQVSLRKAVVY
ORYSJ*  -DNHGRVAVQVEEVKLMISGLLNLGY--STLLAESGREETKKTKKGQTDL
HORVU   -TNHGGVPEQVEDVKVLVSSLLCFIY-PHS--AESEQNETKINFQGSSTS
SORBI   -DKHGRAPEQVEDVKALLSSLLGFTY-PPL--VESEPGQIKTSYQESATS
SELML   -KQVSRKPMKLLEVKQAVCAYLQIPD-TLAGQQKFCAGPVGI-LKRVAGS
PHYPA   -KKRQKRNFMIAELKQLIAINLQLPEYSFRMVAKQLEMKVGVGSRGSKRF
VOLCA   GHAYGRTRKSLLAVARCQGLLLRTAAVAAPGQADSGDGLRKRQRRRRLGP
                 2210      2220      2230      2240      2250
```

MED12

```
HUMAN*  --------------------------------------------------
CHICK   --------------------------------------------------
XENTR   --------------------------------------------------
DANRE   --------------------------------------------------
CIOIN   --------------------------------------------------
ANOGA   --------------------------------------------------
TETNG   --------------------------------------------------
DROME   --------------------------------------------------
BRUMA   --------------------------------------------------
CAEEL   --------------------------------------------------
YEAST*  --------------------------------------------------
SCHPO   --------------------------------------------------
CANAL   --------------------------------------------------
CANGA   --------------------------------------------------
ASPNI   --------------------------------------------------
RHIO9   --------------------------------------------------
CRYNE   --------------------------------------------------
USTMA   --------------------------------------------------
ARATH*  VYSKMDQPEATPGCEDCRRAKRPKMNDEKSSCYQGNSPIASDEEDNWWIK
VITVI   NCNKMDLVEGTPGCEECRRAKRQKLSEDRSSY-QGHSPNPSDDEDTWWVR
SOYBN   PYGKIDPVEGTPGCEECSRAKRQRLSEERSTFVQGHSPVQSDDDDTWWVK
SOLLC   GSNGMDNSEGTPGCEECRRAKKQKLSEEKSSYSQIYQQNPSDDEETWWMR
ORYSJ*  VDSEEDVGHAKTGCKDSSRTKRQKLDKNVFPF-QGSPLVQSDEEDFWWVR
HORVU   TLTQVDTGEAKIGCEDCMRINGQKLDERASPF-QAFPLIQSDEEDVWWVR
SORBI   TLSQVETGEAKSGCEDCMRFKGQKLDDSATPF-QGFPLIQSDEEDIWWVR
SELML   SIGD------THGCEECNGSKRLKTGESKGTG------FIADPEDSWWIK
PHYPA   SYNNMGTRDATPGCEECSRAKRQKTAEGKEWIATGSMTPLEDEDENWWFK
VOLCA   ASYGGGNEAKRRRTTAGVLAARGPPPPPRAASVAEPPSTPLPVCSPAASP
                         2260      2270      2280      2290      2300
```

```
HUMAN*  --------------------------------KDILKVLNRKGTAETDQL
CHICK   --------------------------------KDILKVLNRKSTAETEQL
XENTR   --------------------------------RDILKVLNRKSTSETGKI
DANRE   --------------------------------KDILKVLNRKSTAET---
CIOIN   --------------------------------RHLTKLVQCKRTMDDGVD
ANOGA   --------------------------------KEICKLFSKKFSIDV---
TETNG   --------------------------------KDILKVLNRKSTAETGKG
DROME   --------------------------------KEIGKLFTKKFSIDV---
BRUMA   --------------------------------RELAKLWQKRIVVEFSFN
CAEEL   --------------------------------KSITKEFTTRLYIEFDRS
YEAST*  ------------------------------ITEQIKMRILSNDITNL---
SCHPO   ---------------------------WSICDEYYVKRFKEDHFSFMFKKE
CANAL   --------------------------------SIITKICNNQLTDDIDSQ
CANGA   ------------------------------IVEDLKVKLPNGD--AI---
ASPNI   --------------------------------SSISQRLPNIFEVEASNS
RHIO9   ---------------------------------DLKHLARLAVMGFDHHN
CRYNE   ---------------------------LAAFEEQVKEYVPEVYGLESYGQ
USTMA   --------------------------------RQLQSFLPFAFAQEVQAE
ARATH*  KGSK-TVESSLKVDPQIE-ITKQVPRGRQKMARKTQSLAQLQAARIEGSQ
VITVI   KGPK--SSESFKIDPPLK-AAKQTSRGRQKIVRKTQSLAQLAAARIEGSQ
SOYBN   KGMK--SPEPLKVDQPQK-STKQVTKSRLKNVRKTQSLAQLAASRIEGSQ
SOLLC   KGQK--SIESFRAEPPPK-PAKTASRGRQKIVRKTQSLAHLAAARIEGSQ
ORYSJ*  KEQK---QELFTVETIHQ-SIEQTSGGKATVVQKTQNIAQLAAARIDGSQ
HORVU   KGTE--LQESFKAEPALK-SIKQTSRGRAKVVRKTQNLAQLTTAKIEGSQ
SORBI   KGTK--LQESFNIEPVQK-SIKQTSRGRAKVVRKTQSLAQLAAARIEGSQ
SELML   KVPK--VSDPVKLEASIK-PPKQGVRGRPKTVRKTQSLAQLHSARVDGGQ
PHYPA   KGPQKVVENPIKLEPPVKVAPKQTTRGRPKPVRKTQSLAQMATNRLDSNL
VOLCA   APAHAPSGTPSHTATPAASVPDTFERVLAAAFRSLDSLTYLSLTAAVRGR
                         2310      2320      2330      2340      2350
```

```
HUMAN*  A-PIVPLNPGDL---TFLGGE-----------------------------
CHICK   ------LSPGGQVLSTFVGGE-----------------------------
XENTR   E-KQVNVPKLTL---SPCGTD-----------------------------
DANRE   ------------------GGE-----------------------------
CIOIN   T-NPTL-------------TP-----------------------------
ANOGA   -------------------AE-----------------------------
TETNG   S-SHLST--------VMFGGE-----------------------------
DROME   -------------------AA-----------------------------
BRUMA   K------------------PL-----------------------------
CAEEL   S------------------KV-----------------------------
YEAST*  --------------------------------------------------
SCHPO   E-------------------------------------------------
CANAL   C-------------------------------------------------
CANGA   --------------------------------------------------
ASPNI   K-HINF--------------------------------------------
RHIO9   T-DCLFGINAQDMTQPVPADT-----------------------------
CRYNE   S-EALKTMVNYEIPASSQ--------------------------------
USTMA   A-SRIAVSPSPSTPDPVH--------------------------------
ARATH*  G-ASTSHVCDNKVSCPHHGPG-----------------------------
VITVI   G-ASTSHVCDNRISCPHHRTG-----------------------------
SOYBN   G-ASTSHVCGNRVSCPHHKTA-----------------------------
SOLLC   G-ASTSHVCDSKVSCPHHRPG-----------------------------
ORYSJ*  G-ASTSHVCDNNLSCPHHKPG-----------------------------
HORVU   GEASTSHLCESKLSCPHHKPS-----------------------------
SORBI   G-ASTSHVCESKVSCPHHKPN-----------------------------
SELML   G-ASSSHSFDSKVHCPYHRPA-----------------------------
PHYPA   G-ASTTHVLEPKISCPIHHSS-----------------------------
VOLCA   S-AIPSGRPPQRAPSPGVPPSPGLAASPGPFVSSPSPSSSPLRPEIMEVE
                         2360      2370      2380      2390      2400
```

MED12

```
HUMAN*  --------------DGQKRRR------NRPEAFPTAEDIFA---------
CHICK   --------------EGQKRKK------SKPEAFPTAEDIFA---------
XENTR   --------------EGQRRRR------AKPEAFPTAEEIFC---------
DANRE   --------------EGQKRKR------SKPEAFPTAEDIFS---------
CIOIN   --------------SGKKIKK------SPKS---SHIELLS---------
ANOGA   --------------GGKVKKH------SRSEF--NFESTSN---------
TETNG   --------------EGQKRKK------SKPEAFPTAEDIFS---------
DROME   --------------AGHVKKH------SRNEF--NFEATTS---------
BRUMA   --------------EIRFKKR------TTRE---QVQESMA---------
CAEEL   --------------TTAHKK-------INQN---RLDDLLR---------
YEAST*  --------------------------------------------------
SCHPO   ---------------------------NYAIITLSLVKIASTPMSKLYED
CANAL   --------------------------------------------------
CANGA   --------------------------------------------------
ASPNI   --------------------------------------------------
RHIO9   --------------FESYNLA------FSQELKHKLMSTMD---------
CRYNE   --------------------------------------------------
USTMA   --------------------------------------------------
ARATH*  --------------VEGENQKV--VDVFRTSTPVDMVSVGN---------
VITVI   --------------MEGEAPKS--IDEVKATHCSDIVSIGK---------
SOYBN   --------------MDGDGQRS--VDSIRTSHFGDIVSIGK---------
SOLLC   --------------IEGSVPKS--GDGIRMPN-GDVVSIGK---------
ORYSJ*  --------------TNSDILKD--ADHMSMLT---LAEVGK---------
HORVU   --------------TDGDNAKD--ADHTRMTN---LAEVGK---------
SORBI   --------------IDGDNVKD--FDHTRAAN---LTEIGK---------
SELML   --------------------ASTKLSLNDLRPKNDFRSIGS---------
PHYPA   --------------LEGGTAAQLKTSHYSKMT-GDLRAIGE---------
VOLCA   AVVDGGAAGNVGGSTSPAGVRGRRPEDSATPSAGRVRVVGRIAGRGGDPD
                 2410      2420      2430      2440      2450
```

```
HUMAN*  ---KFQHLSHYDQHQVTAQVSRNVLEQITSFAL-----------------
CHICK   ---KFQHLSHFDQHQVTSQVSRNVLEQITSFAL-----------------
XENTR   ---RFQQLSHFDQHQVTAQVSRNVLEQITSFAL-----------------
DANRE   ---KFQHLSHFDQHQVTSQVSRNVLEQITSFAL-----------------
CIOIN   ---RYRVLSYFDQHMITSTVAKSLLTAINNYIA-----------------
ANOGA   ---KCQAMSYFDQHVVTWQCAVQVQEMLNAFAI-----------------
TETNG   ---KFQHLSHFDQHQVTSQVSRNVLEQITSFAL-----------------
DROME   ---KCQQMAYFDQHVVTAQCAANVLEQLNGFAL-----------------
BRUMA   ---KFRSCTYYDQLVLCGWCCESFIQMVQDFVD-----------------
CAEEL   ---QFRAQTYHDQHLILERIVYNFIDGIGGFLK-----------------
YEAST*  ---QLSKTPLSIKIMVAEWYLSHLCSGIL---------------------
SCHPO   YLVMLFAFHYSMFQVMTKLIADNLVHFSFQSCA-----------------
CANAL   -IGYIDSLSVGLRFLLINWLTNELKSKIKSTNKLIHFTPT----------
CANGA   ---EYSNYPMSVKISLGEWYLTYLCNGKL---------------------
ASPNI   ---SKHDLTWAVKSEVSMWIRQGVVKHLKDTTRKIIGRPLSLDSRISA--
RHIO9   -----STTRYSVLEFTSHWLLNEVKKFVVKSIQ-----------------
CRYNE   ---LTRYLYVFARFSIAANAGATLRS------------------------
USTMA   ---ALSRLWSASRFVRCRLFRSELLPAVTARVE-----------------
ARATH*  ---SLKQLQFVDKRSIAVWLTTAVRQLVEEPQK---------------SS
VITVI   ---ALKQLRFMEKRTITMWLATVVRQFVEENEK---------------TV
SOYBN   ---ALKQLRFVEKRAIAAWLLTVVRQVIEDVEK---------------NI
SOLLC   ---ILKRLRFVDKRTMTLWLIGIVKELVEESEK---------------TV
ORYSJ*  ---SLKRLRLLERRSISIWLLKSIKQLIEGDEV---------------KH
HORVU   ---SLKKLRLLERRSISVWLLKSVRQLVEGNET---------------TA
SORBI   ---SLKRLRLLERRSVSSWLLKSVRQLVEGNEM---------------TA
SELML   ---ALKQLTVSEKFNFSSWLDRTIRSLLGEGTA-----------------
PHYPA   ---AMKRLRVVENQMIVSWLDSQLRSILTNQGI---------------TS
VOLCA   LLAEVRGLRPWQQRLVASAVCRHVNAVLSAALM-----------------
                 2460      2470      2480      2490      2500
```

```
HUMAN*  ---------------------------GMSYHLPLVQHVQFI--------
CHICK   ---------------------------GMSYHLPLVQHVQFI--------
XENTR   ---------------------------GLSYHLPLVQHIQFI--------
DANRE   ---------------------------GMSYHLPLVQHIQFI--------
CIOIN   ---------------------------GRSSCLPRLEVVLLL--------
ANOGA   ---------------------------GNSNYLPVQEHVAFL--------
TETNG   ---------------------------GMSYHLPLVQHIQFI--------
DROME   ---------------------------GNNNYLPVQEHVAFL--------
BRUMA   ---------------------------GHSCLLPTAEGLDII--------
CAEEL   ---------------------------KNCDDVPAPEVANII--------
YEAST*  ---------------------------SSVNRTVLLKIFKIF--------
SCHPO   --------------CQTLFFICSVVPKTESQKLLLNEMGKLFQVELNFSY
CANAL   ----------------------------------------VI--------
CANGA   ---------------------------VNETRESLTEKYVLF--------
ASPNI   ---------------------------LTPGEFY------CV--------
RHIO9   -----------IGEDNWRVMTSPGSCLLNTRQLV------TI--------
CRYNE   ---------------------------DGERRAMDASTFARI--------
USTMA   --------------------------RLNGDQLS------QI--------
ARATH*  VRVGQFN----RGAPV--EEKNTIRWKLGADELY------SI--------
VITVI   AKGGQFS----RPFSV--DDRSSLRWKFGEEELS------ST--------
SOYBN   GKVGQFS----KPFPVV-DDRGSIQWKLGEDELS------VI--------
SOLLC   TKVGQYG----RPFSAA-DERGCVRWKIGEDELS------VV--------
ORYSJ*  SKANNSI----SASTVQHSGKIASGWRFGEDELL------SV--------
HORVU   CKASNSI----SSFSSQPEDKTASKWRLGDEELM------SI--------
SORBI   AKATNSI----SILSLQPDDKTASKWRLGDEELL------SV--------
SELML   -----------------NKTGPTARCQLSDEQFG------AV--------
PHYPA   PSLQGDRRLANGGVSNT-DSADPGTWRLGEEQFS------II--------
VOLCA   PPVPFGITAAPPPFPSAYVRACAAAALTKPAELPGGPLTEAVAAARAGGG
                 2510      2520      2530      2540      2550
```

MED12

```
HUMAN*  --------------FDLMEYSLS----------ISGLIDFAI--QLLNEL
CHICK   --------------FDLMEYSLN----------ISGLIDFAI--QLLNEL
XENTR   --------------FDLMEYSLN----------ISGLIDFAI--QLLNEL
DANRE   --------------FDLMEYSLN----------ISGLIDFAI--QLLNEL
CIOIN   --------------FDLMEEALN----------VGGLVRTAV--AIATRL
ANOGA   --------------FDLMESAFN----------IYGLIDTCI--QILREL
TETNG   --------------FDLMEYSLN----------ISGLIDFAI--QLLNEL
DROME   --------------FDLMELALN----------IYSLLELCD--SLLKEL
BRUMA   --------------LEMCELAQN----------IAGIMELAE--ELLPLL
CAEEL   --------------CEMYEFSMD----------ITSIFDFFE--MVNPYL
YEAST*  --------------CIDLEVFHH----------FFKWIEFIVYHQLLSDI
SCHPO   DSPDVNLLIEQFYEITSYESNYDDAFVEYKDATVANRKDFIE--FLFHNI
CANAL   --------------TKLYQFYAK----------NSGLTVFFK--VVIQTI
CANGA   --------------AHQLNTNHL----------YFKWIEFIVYHQLIEDI
ASPNI   --------------REILERFGD----------CSILADVLK--QAIE--
RHIO9   --------------IQIMECAKD----------YLSIIEMAL--WVLQK-
CRYNE   --------------TQVFRQSHG----------YATIADFMV--RALQE-
USTMA   --------------SAVLVMADD----------FEGLAQLLA--TLLLR-
ARATH*  --------------LFLLDISLD----------LVSAVKFLL--WLLPKA
VITVI   --------------LYLMDVCND----------LVSAAKFLL--WLLPKV
SOYBN   --------------LYLMDISDD----------LVSVVKFLL--WLLPKV
SOLLC   --------------LYLIDACDE----------LVLAARFLL--WLLPKV
ORYSJ*  --------------LYIMDTCCD----------LLSSVRLLI--WLLSKI
HORVU   --------------LYILDTCCD----------LASATRFLV--WLLAKI
SORBI   --------------LYVLDTCCD----------LVSGARFLV--WLLAKI
SELML   --------------VYMLDMVFD----------YNVLVRLLL--WLLPMA
PHYPA   --------------VYIMDVGND----------LHTLMQLLL--WLLPIS
VOLCA   FSAVKHGDTWLLRCLALLEACQC----------WHCGPQLLLHLTALHVE
                  2560      2570      2580      2590      2600
```

```
HUMAN*  SVVEAELLLKSS-D---LVG------SYTTSLCLCI-VAVL---------
CHICK   SVVEAELLLKSS-D---LVG------SYTTSLCLCI-VAVL---------
XENTR   SVVEAELLLKSS-S---LVG------SYTMGLCLCI-VAVL---------
DANRE   SLVEAELLLKSS-N---LAG------SYTTGLCLCI-VAVL---------
CIOIN   ADLANEIQRRKA-E---TNSLNVEYVWYTTELSLAV-VSVL---------
ANOGA   PEVELQLIGKSSMA---LVR------SYTTSLSLYV-VGVL---------
TETNG   SLVEAELLLKSS-N---LVG------SYTTSLCLCI-VAVL---------
DROME   PEVEHQLQLKKS-N---LVR------SYTTSLALYI-VSIL---------
BRUMA   VLIERVLLERRV-D---CVP-----SNLSAQLGYVL-TAYI---------
CAEEL   KAVDDKIAHFRM-D---VLP-----DMYYTETAFIF-VSFF---------
YEAST*  ------------------------------ESLEAL-MDIL---------
SCHPO   TVSSKHTAVIFTSDLLMVLKIALNHPPYFDDLATTTFSSLL---------
CANAL   LKNDGGMIIFYLDS-------------------LYLISKLV---------
CANGA   ------------------------------ETLEYL-MEVL---------
ASPNI   ---------------------------CDDNIILASVSDTV---------
RHIO9   --------------------------SNDTSLHPYI-IDTF---------
CRYNE   --------------------------AEDDEILDVI-LDII---------
USTMA   --------------------------PLSDNLARAA-FNMV---------
ARATH*  NSTP-SFAVQGGRN---LVTVPRNVENNMCEIGEAILVSSL---------
VITVI   LSNP-SSTIHGGRS---IMMLPRNVESHACEVGEAYLLSSI---------
SOYBN   LNSP-NSTIHSGRN---VVMLPRNVENQVCDVGEAFLLSSL---------
SOLLC   LGSC-SATVHGSRN---ILTIPKNTENNICEVGEAYLLSSM---------
ORYSJ*  YIGR-TTSGQVGRG---VMH-PKHKENQVFQVAEAFLFSSL---------
HORVU   RG------GQVGRS---VAH-MKNRDNHVCQVGEAFVFSSL---------
SORBI   RGGM-GSSGQPGRS---SMH-TRNRDHQVCQVSEALVFSSL---------
SELML   AGTSG------------MSARAPVNNKETCGIGEAAILSCL---------
PHYPA   GTISSPSVIHVNHG---IPAYVGSRDGSSCAVGETALLSCL---------
VOLCA   ATGQWATSSAATSP---VGT-------AAVSPAAAAAAAALENGGLLWHR
                  2610      2620      2630      2640      2650
```

```
HUMAN*  --RHYH----ACLILNQDQMAQVFEG-----LCGVVKHG--MNRSDGSS-
CHICK   --RHYH----SCLILNQDQMAQVFEG-----LCGVVKHG--MNRSDGSS-
XENTR   --RHYH----TCLILNQEQTAQVFEG-----LCGVVKHG--MNRSDGSS-
DANRE   --RRYH----SCLILNPDQTAQVFDG-----LRIVVKSG--VNPADCSS-
CIOIN   --RHYI----SFLTLNMDLTFNAFEG-----LLDATKQSCISNPTQCAS-
ANOGA   --RRYH----CCLLLSQEQTTAIFEG-----LCRIVKHV--SNPSDCSS-
TETNG   --RRYH----SCLILNPEQTAQVFDG-----LRIVVKSG--VNPADCSS-
DROME   --RRYH----SCLLLSPEQTLSVFEG-----VCRTIRHV--SNPSECTS-
BRUMA   --SHNY----YYFLYSQ-NAPNIVQG-----LYRVIESQLKSSDFCLTG-
CAEEL   --MKHW----QRFLLHP-RACAIVNQ-----CFVLIQDMIRADDHMITC-
YEAST*  --LCYQ----KLFSQF--INDHILFTKTFIFIYKKVLKEKDVPAYNVTSF
SCHPO   --KRDECTILSFFKILQFYGCKLLSVDQIWAVVSDVYEAQDNNTT-----
CANAL   --IRHY----KLIKSIA-GASELSAAYDLFKKLIIQVYKDLNTREYDCFKF
CANGA   --LRYK----KLFSQY--INDHVLFIKTFIFIIRRILKERDSIAYSLTSF
ASPNI   --NYHF----DALSMIG-ATSDLFRG-----LVGSYARLKRSGNLSLDFV
RHIO9   --RKYS----CYWKLTN-NSARVIQA-----VWTKHVNLCSRGERD----
CRYNE   --KRDA----DVWTAMD-LWPRLGDK-----LLDRHHALERKGKEHPRLV
USTMA   --IEYS----LVWRSMD-IMATLNQ------LVRQQLNHSSSIRNDRQAV
ARATH*  --RRYE----NILLSAD-LVPEAMTA-----LMNRAASLMSS--NGKISG
VITVI   --RRYE----NILVATD-LIPETLSA-----TVLRAAAVMAS--NGRVSG
SOYBN   --RRYE----NILVAAD-LIPEALSS-----AMHRVATVIAS--NGRVSG
SOLLC   --RRYE----GIIVAAD-LIPETLSV-----VMHRAQTILTS--NGRVSG
ORYSJ*  --LRYE----NILIAMD-LLPEVLSV-----SMNR--TVHKS--GERQST
HORVU   --LRYE----NILLATD-LLPEVLNA-----STNR--NFVLA--TARHPA
SORBI   --LRYE----NILLATD-ILPDVLSA-----LVNR--NSVSA--TVRHPG
SELML   --RRYE----LVITSLN-LLPEVLTV-----GIQCASSVIGTIPSGR---
PHYPA   --QRYE----GVLASMD-MLPQALSA-----VVQRAANVMAAVPGGRAGC
VOLCA   LRLQSS----LVLAALESLTPRLVGCG----LLLPSLDVLTSWAIRYGAL
                  2660      2670      2680      2690      2700
```

MED12

```
HUMAN*  ----------------------------------AERC------------
CHICK   ----------------------------------AERC------------
XENTR   ----------------------------------AERC------------
DANRE   ----------------------------------AERC------------
CIOIN   ----------------------------------ADRS------------
ANOGA   ----------------------------------AERC------------
TETNG   ----------------------------------AERC------------
DROME   ----------------------------------AERC------------
BRUMA   ----------------------------------WGRT------------
CAEEL   ----------------------------------WGRT------------
YEAST*  MPFWKFFMKNFPFVLKVDNDLRIELQSVYNDEKLKTEK------------
SCHPO   --------------------------------------------------
CANAL   SS------------------------------------------------
CANGA   MDFWKFIMKSYSLEIRADTDLRSEMSAVYEEEKAKKEI------------
ASPNI   FSLIELGLRLPDESGTV-------------------YL------------
RHIO9   ------------------------------------RN------------
CRYNE   QLLK----------------------------------------------
USTMA   MASTTLLRLRT---------------------------------------
ARATH*  ------------------------------------SA------------
VITVI   ------------------------------------SL------------
SOYBN   ------------------------------------SG------------
SOLLC   ------------------------------------SP------------
ORYSJ*  ------------------------------------SV------------
HORVU   ------------------------------------PA------------
SORBI   ------------------------------------ST------------
SELML   ------------------------------------SQ------------
PHYPA   ------------------------------------SA------------
VOLCA   PTVGRRYLGRATQCAGVLAAAGAAVEAPGVRAWWERFQKLDSAHGQHWLI
                 2710      2720      2730      2740      2750
```

```
HUMAN*  --------------------------------------------------
CHICK   --------------------------------------------------
XENTR   --------------------------------------------------
DANRE   --------------------------------------------------
CIOIN   --------------------------------------------------
ANOGA   --------------------------------------------------
TETNG   --------------------------------------------------
DROME   --------------------------------------------------
BRUMA   --------------------------------------------------
CAEEL   --------------------------------------------------
YEAST*  --------------------------------------------------
SCHPO   --------------------------------------------------
CANAL   --------------------------------------------------
CANGA   --------------------------------------------------
ASPNI   --------------------------------------------------
RHIO9   --------------------------------------------------
CRYNE   --------------------------------------------------
USTMA   --------------------------------------------------
ARATH*  --------------------------------------------------
VITVI   --------------------------------------------------
SOYBN   --------------------------------------------------
SOLLC   --------------------------------------------------
ORYSJ*  --------------------------------------------------
HORVU   --------------------------------------------------
SORBI   --------------------------------------------------
SELML   --------------------------------------------------
PHYPA   --------------------------------------------------
VOLCA   GDLTPYTQGLASSAGSPGQPGGGGGGGGAVNSVSPNAVGAASGGSMTPPG
                 2760      2770      2780      2790      2800
```

```
HUMAN*  -------ILAYLYDLYT--------SCSHLKNK----------FGELFS-
CHICK   -------ILAYLYDLYT--------SCSHLKSK----------FGELFS-
XENTR   -------ILAYLYDLYT--------SCSHLKSK----------FGELFS-
DANRE   -------ILAYLYDLYT--------SCSHLKSK----------FGEIFS-
CIOIN   -------VLVFLYDLYT--------SFGYIRSQ----------RSEAFG-
ANOGA   -------ILAYLYDLYS--------ACSSLKARP---------QQEPFH-
TETNG   -------ILAYLYDLYN--------SCSHLKNK----------FGEIFS-
DROME   -------IIAYLSDLHE--------SCVLLQGKE---------QSTEYYQ
BRUMA   -------IAVFVLDAKK--------QL--IETGL---------CSRKIE-
CAEEL   -------VAIFVFHARK--------AI--ANAGL---------QNEEFL-
YEAST*  -------LKNDKSEVLK--------VYSMINNSNQAVGQTWN-FPEVFQV
SCHPO   -------LKQFFNYLLD---------------------------------
CANAL   -------IWNFINAVIE-------------KSSPTSSATTTATTSVSISS
CANGA   -------TEGEKDSWMG--------LYQNIHPDSTSLDQNHTGFSELFLT
ASPNI   -------LRQDLARIES--------KSALAAPSP---------LSDHIPT
RHIO9   -------IMMYMVQLVQEGYQMDDLSRSQLQQDLQIKSKTRSHYTNNLSV
CRYNE   -------VLAQKKRLMP--DDEDEVKQLQTSVDKSTSPPAGVKLVDVFEV
USTMA   -------LIENVPSRISASALDPGADHDALQQHVQALRPNVDALLDRFAK
ARATH*  -------ALVYTRYILK--------RYGSLPSVVEWHNNFKATSEKKLLS
VITVI   -------ALVYARYLLK--------KYGNVSSVIEWERHFKSTGDKRLIS
SOYBN   -------ALAFARYLLR--------KYSNVASVIEWEKTFKTTSDARLSS
SOLLC   -------AVIYVRYLLK--------KYGSVGSVAEWEKNVKSTFDKRLAS
ORYSJ*  -------AFAYARYFLR--------KYRDVTSVARWERNFRSTSDKRLLA
HORVU   -------AFPYTRYFLR--------KYRDVASVVRWEKNFRTTCDQRLLA
SORBI   -------AFAYVRYFLK--------KYRDVSGVAKWEKSFRTTCDQRLLA
SELML   -------ILIYVSELLN--------KYKGLMTVQSWQKTWRGSCDQKVAM
PHYPA   -------LLCYVRDLLR--------KYGSLANVQAWEKSWKASCDQRLRA
VOLCA   LPQPRPPLVRLSEQVVQAMEQVRGGNASQLAARWKACTDACAPLAAALAA
                 2810      2820      2830      2840      2850
```

MED12

```
HUMAN*  --DFCSKVKNTIY----------------------------CNVEP----
CHICK   --DFCSKVKNTIY----------------------------CNVEP----
XENTR   --DFCSKVKNTIY----------------------------CNVEP----
DANRE   --EFCSKVKNSIY----------------------------YNIDP----
CIOIN   --GYVASVRQALC----------------------------STKKP----
ANOGA   --NAYPKIKQALY----------------------------TPLQP----
TETNG   --EFCSKVKNSIY----------------------------CNIDP----
DROME   QLQCIKRFKDIFN----------------------------TPEQL----
BRUMA   -------AKRELL----------------------------KRVIPPWKP
CAEEL   -------AEDSHF----------------------------WRVFP----
YEAST*  NIRFLLHNSEIID----------------------------TNTSK----
SCHPO   -----------------------------------------ESTWP----
CANAL   LYLTMGKPKNSKL----------------------------KSVQG----
CANGA   NLKTFLSSKQ--D----------------------------KQAKK----
ASPNI   TFNEVDASFQERL----------------------------EQLLS----
RHIO9   KSELMQLTKDFHV----------------------------SSIQM----
CRYNE   LPHILTTGKENTA----------------------------ISVAPALFR
USTMA   LTRSSVESDGSIA----------------------------SSSTPVEGS
ARATH*  ELDHTRSGNGEYGNPLGVPAGVDNPDDYLRKKISIGGARP-SRVGLSMRD
VITVI   ELESGRSLEGEFGFPLGVPAGVEDLDEFFHQKIS--HTRV-SRVGLSMKD
SOYBN   ELESGRSVDGELGLPLGVPAGVEDHDDFFRQKIS--GGRLPSRVGAGMRD
SOLLC   EVESGRLVDGEFGFPLGVPVGVQDPDDYFRQKIT--GVRV-SRVGLSMRD
ORYSJ*  ELDSGKSITGDSIIS-GISS------------------------------
HORVU   DLDNGRSIDGDFVSSSGVLGG-EEIDDQVRQKLN---GRG-SRIIPNMKE
SORBI   ELDNGRSIDGDLISSSGVSAG-EEIDEQVRQKLN---GKS-SRLVQNMKE
SELML   ELEALKVGDSESALGFINTVG-EDRDDPIPQRLN---SRL-ARVGASMKD
PHYPA   ELEALK-GDTEGGFGL----------------------------------
VOLCA   LLTGGSSAGGGGGGTSAATAAG-------------------ARGSPAVTT
                  2860      2870      2880      2890      2900
```

```
HUMAN*  ----------SESNMRWAPEFMIDTLENPA--------AHTFTYTGLGKS
CHICK   ----------SDSNMLWEPEFMIDTIENPS--------AHNFTYTNLGKS
XENTR   ----------SDSNMLWEQEFMLDTIINPS--------GHSVNCGMLGKL
DANRE   ----------SDSNMLWDQMFMIDAITNPT--------AHNLNHSMLGKI
CIOIN   ----------ATTNCRYDQTFLQSFIDDPM--------AQSPLSVAEKYS
ANOGA   ----------TPSAHTYNPQFMLDIITNPR--------RGGKIESGWARQ
TETNG   ----------SDSNMLWDPVFMVEAIANPS--------AHNFNHSMVGKI
DROME   ----------DLPPQGYNPLLLQELFMAPR--------RGGKLDPHWLGT
BRUMA   AHNIGDRNPCGGQRYAYNASTFKELLEEPK--------RIFSFNEYRRLH
CAEEL   ----------NAQHVDLDVGYFNEDFAGVKLQLRGGTLRYDSYNDFKWLV
YEAST*  --------------------------------------------------
SCHPO   ----------------------EGYLEERHWRSILCKEARKHDSGLKLFK
CANAL   -------------------LHVKQTMDSPM--------------------
CANGA   --------------------------------------------------
ASPNI   --------------------------------------------------
RHIO9   --VAESLSLPSQNAAGWIGIILENAGDVLR--------------------
CRYNE   --------------------------------------QCGRFSSWSLKW
USTMA   DMLTPFRSLFVQPDAMSDEMIERT--------------------------
ARATH*  VLQRHVEEATHYL----KKLIGTGTMKASLAEKNDDGYQVAQQIVVGLMD
VITVI   IVQRNVDDALHYLFGKERKLFAPATPKAPAIEKWDDGYQIAQQIVIQLME
SOYBN   IVQRNVEEAFHYLFGKDRKLFAAGTPKGPTLEKWDNGYQIAQQIVMGLID
SOLLC   IVQKKVDEAVNYFYGKDRKLFGPNSGKLPGFQKWEDVYQIGQQIVMGLMD
ORYSJ*  -------------------------------------------------D
HORVU   IVQRQAEEIQRNL--KEKKI--PAAPKSP-SFEKEDSYQIAHDTVLGLVE
SORBI   IVQRQADEVQRSL--KEKKV--LAAPRNPPTFEKEDSYQIAHDIVSGLVD
SELML   SVQKALGEAVSQIVSKERELQTANFAIKDAVAEKVE--EYSHRVVTGLLE
PHYPA   -------------------------------------------VSMGVMN
VOLCA   CPGSPVDATASTDGGDFTERLRTAARQAGRAVAGLCTACTAGESDGMLLP
                  2910      2920      2930      2940      2950
```

```
HUMAN*  ---LSEN---------PANRYSFVC-----NALMHVCVGHHDP-------
CHICK   ---LNEN---------PANRYSFVC-----NALMHVCVGHHDP-------
XENTR   ---LSDS---------PTNRYSFVC-----NALVHVCAGDHDT-------
DANRE   TNELNDS---------PANRYSFVC-----NVLMDVCVDHRDP-------
CIOIN   G-LLQD----------VSRRYSFVC-----SAVITVCNGHQDF-------
ANOGA   ---LNES---------ASNRYSFVC-----NAVVAVT-RDIDN-------
TETNG   ---LNDS---------KANRYSFVC-----NVLMDVCVDHRDP-------
DROME   ---LHES---------PANVYSFVS-----NALIAVC-RETDN-------
BRUMA   AV-IDGT---------DDARYSFVV-----SALLAAVHGIGNAGGNDNTG
CAEEL   SN-MKPNLKKKPHLKRPNTRYSFVV-----RAFMEARQHGRNF-------
YEAST*  ------------------QFQKARN-----NVMLLIATNLKEYNK-----
SCHPO   LGIKLCTRNEQIMKTIWSFIHVHDC-----NVISEVIPDQRFLRTLTEHF
CANAL   ---CINTVDESHTHDTRSGAKRYTSTDFRNDLYFLVTLQPRALTSE----
CANGA   ---------------------KARY-----NLLLLMQAKNRDYSK-----
ASPNI   ---CGNGLDESTMGAIISSLTKILT-----DGGGAAKVSAKDACR-----
RHIO9   ---MIGKEKGIDKEIKSYRHGRFLS-----QALFEFHRMLRTFA------
CRYNE   WTLIVDVVQKAGNTEQKPFLYHVIS-----THLSAVLGQHGDSLDDA---
USTMA   ---IVDSVFARSDTSLVPSAVRLID-----QLYLEASLEIDER-------
ARATH*  CIRQTGGAAQEGDPSLVSSAVSAII-----NSVGLSVARITDFSLGNIYQ
VITVI   CIRQTGGAAQEGDPSLVSSAVSAIV-----CNVGPSMAKLPDFSAGNNYL
SOYBN   CIRQTGGAAQEGDPSLVSSAVSAIV-----GSVGPTLAKMPDFSSGNSHS
SOLLC   CMRQTGGAAQEGDPTLVSSAISAIV-----INVGQVIAKIPELTASNNHP
ORYSJ*  CIRQNGGANPDGDHSLVASAVSAIV-----DNAGHAIAKHLDISGGN-NP
HORVU   CIRQNGGASPDGDPSAVASAVSAIV-----VNAGHVIAKHLDFAGGN-YH
SORBI   CIRQNGGANPDGDLSTVASAVSAIV-----VNAGHVIAKHLDFAGGN-YQ
SELML   AIKQNGTA--QTDSGLVNAAVSALV-----SSASSSAANAHEVLTANNAG
PHYPA   IIKQNSGGVSQNDVALLANAVSTIV-----NHAGQSVANVLENLGYN-AP
VOLCA   VSSILHAWIRCCCATVGPAAGRAAA-----AAVTRGSPSVSQPGSPTMGV
                  2960      2970      2980      2990      3000
```

MED12

```
HUMAN*   -------------D---------------------------------RVN
CHICK    -------------D---------------------------------RVN
XENTR    -------------D---------------------------------RVN
DANRE    -------------E---------------------------------RVN
CIOIN    -------------E---------------------------------KVN
ANOGA    -------------D---------------------------------CLN
TETNG    -------------ERWVTLLLCDVCLTVCCSSLLSARPQLHVMCRPIRVS
DROME    -------------E---------------------------------RLN
BRUMA    IGSGSAAVERLTGD---------------------------------RLV
CAEEL    -------------D---------------------------------RIN
YEAST*   --------------------------------------------------
SCHPO    MIDL-------------------------------------------RQL
CANAL    -----------------------------------------------EKQ
CANGA    --------------------------------------------------
ASPNI    --------------------------------------------------
RHIO9    -----------------------------------------------LII
CRYNE    --------------------------------------------------
USTMA    --------------------------------------------------
ARATH*   NHPS--GVDSSNIAR--------------------------------YIL
VITVI    NFPS--TTSSLNFAR--------------------------------RIL
SOYBN    NTMP--ATNALNYAR--------------------------------CIL
SOLLC    SSS---TSASLQFAR--------------------------------CIL
ORYSJ*   GVTS---INSLNLIQ--------------------------------HIL
HORVU    GVAS--IGNSLSFVR--------------------------------HTL
SORBI    GVNS--VSNSLNFVR--------------------------------HTL
SELML    ------PDISLRCAR--------------------------------RIL
PHYPA    GETTATALSALQAGR--------------------------------RTL
VOLCA    GAGGSRAAALGPRATASAWGVAGSGMMLCIEALRLELCNSLGGMVSEQRL
                     3010      3020      3030      3040      3050
```

```
HUMAN*   DIAILCAELTGYCKSLSA--E--WLGVLKALC----------CSS-NNGT
CHICK    DIAILCAELTGYCKSLSA--E--WLGVLKALC----------CSS-NNGT
XENTR    DIAILCAELTGSCKSLSA--E--WLGVLKALC----------CSS-NNGT
DANRE    DIGILCAELTAYCRSLSA--E--WLGVLKALC----------CSS-NNGN
CIOIN    ALAVLCAEMNARCNELSS--E--WLGVLRALC----------FSS-C--T
ANOGA    DIAAMCAELTACCNSLST--E--WLGVLIALC-----------GS-NRDA
TETNG    DIGILCAELTAYCRYLSA--E--WLGILKALC----------CSS-NNGN
DROME    DVALACAELTASCNVLSE--E--WIYALQSLC-----------SG-SK-S
BRUMA    ELANICGHVSAQAAQVEK--YLDWCGALQALC----------CSSLQGGT
CAEEL    ELANYCANITANDPPLSE--Y--WIGAIKGLC----------FLSLDAPY
YEAST*   -----------------------FMSIFLKRKDFTNKNLIQLISL-----
SCHPO    DIVTCLKKALVTLDEFSAPLYATWLTTL----------------------
CANAL    EVFDLIEIELKDLDSALE----FWYNNFELLGEEKEINLMKIIKSLAVTT
CANGA    -----------------------FISIYLKRKDFKTSDLVNLLSS-----
ASPNI    -----------------------YLAYLRPFN------------------
RHIO9    KQVVNATILTGQADNAVI--YWLWNN------------------------
CRYNE    --------------------VTSWLENLSAAN------------------
USTMA    --------------------HARWLSNLVL--------------------
ARATH*   RIHITCLCLLKEALGERQ--SRVFEIALATESSTALTGVFA--PVKGSRG
VITVI    RIHITCLCLLKEALGERQ--SRVFEIALAAEASSALATAFA--PVKAPRS
SOYBN    QMHIACLCLLKEALGERQ--SRVFDIALATEASNALAGVFS--PSKASRS
SOLLC    RIHVTCLCILKEALGERQ--SRVFEVALATETSSALAQLFA--PGKAPRS
ORYSJ*   DIHINSLALLRETLGDRF--SRIFEISLAVEASSAVAASFA--PPKAHRS
HORVU    HIHISSLCLLKEALGDRF--SRVFEVALAVEASSAVTAALA--PPKVQRN
SORBI    RVHINSLCLLKETLGDRF--SRAFEIALAVETSAAVTAAFA--PPKMHRN
SELML    QLHVRCLRLLKDGLDDRQ--SRVLDVSLASEASTIVAGHFVHIPGRAPRS
PHYPA    QFYVYCLHLLKGAVDDRT--FRMLEVAIANEANTIVQAGVGHFPGRAPRS
VOLCA    QRTLLATCCAALCDPAAA--AADAAAAFPDVC-----------AARGAGT
                     3060      3070      3080      3090      3100
```

```
HUMAN*   -----CGFNDLLCNVDVSDLSFHDSL----------A---TFVAILIARQ
CHICK    -----CGFNDLLCNVDVSDLSFHDSL----------A---TFVAILIARQ
XENTR    -----CGFNDLLCNVDVSDLSFHDSL----------A---TFVAILVARQ
DANRE    -----CGFNDLLCNVDVSDLSFHDSL----------A---TFVAILIARQ
CIOIN    -----VNFNDVLLIVEAGDAQIRDPL----------S---LFTAVLIARG
ANOGA    -----GYYVDVLTQVDVQNTNIHNAL----------S---VFTSILVARH
TETNG    -----CGFNDLLCNVDVSDLSFHDSL----------A---TFVAILIARQ
DROME    -----PRYPHLGGQVDIGQLKTHNAL----------A---VFVCILVARH
BRUMA    -----STFPDLLMEINVEDSTCHYNI----------A---TFYMLLAGRS
CAEEL    -----P-FKEMSQQIDISDCSTHYSL----------T---TFITCLAGKS
YEAST*   ----------------------------------------KLLTFEVTQN
SCHPO    DDDELSELTDDVVQKKVLESLDNYK---------------SGIWKLVLSG
CANAL    YNDHLSQFNEKLKQFVISKVGESIDH----------SKLSNFLKKLAIFD
CANGA    ----------------------------------------KLLTLDQIKN
ASPNI    -----PKYLDGMLVRYVYGLLKSSSR----------PTMSQVLSPLIGVG
RHIO9    ---------------EVVQEDMYQEN----------SWVPLFVITLVANN
CRYNE    ------------------------VVETFGGRTRPAGVLVRLLLFLVSHR
USTMA    ------------------------SIEQGTHSDASMKALLSLLTRLIAHG
ARATH*   QHQLSPESYDSNANNSTIDMSNGTGKMALSRATKITAAVSALVIGSITHG
VITVI    QFQLSPEAHDSNASMSN-EILNNSAK--LGRATKILAAVSALVIGAVIHG
SOYBN    QFPMSPEAHDSSNTISN-DMGSNSSKV-VAKTTKIAAAVSALLVGAIIYG
SOLLC    QFQLSPESNDSN--LSS-DILNNSSRVVIGRAAKISAAVSALVIGAILQG
ORYSJ*   --QQSSETHDESGNHAN-EVPSNPSKCFNVKAVKVSAAVSALVVGAIIYG
HORVU    QFQPSSETHDAYGNHTN-EPLSTSGKGFVGRAAKLAAAISALVVGAVVHG
SORBI    QFQPSPEVHDAYGNHTS-D-LSNSGKGFVGRAAKVSAAVSALLVGAVVHG
SELML    QFHLSPETPELG------TLGNDFAAGQNVITSGRAASATALVVMAVVHG
PHYPA    QFHLSPETPDTN------STLASDPGGTLGRPTMAASAVLALVTGLVVNG
VOLCA    SSPPRPRQHGPTPGSAAAAGTAAVSVAAAAGAAAAQEAVLRLVLCAVSYG
                     3110      3120      3130      3140      3150
```

MED12

```
HUMAN*  CLL----LEDLIRCA----------------------AIPSLLNAACS--
CHICK   CLL----LEDLIRCA----------------------AIPSLLNAACS--
XENTR   CLL----LEDLIRCA----------------------AIPSLLNAACS--
DANRE   CLL----LEDLVRCV----------------------AIPSLLNAACS--
CIOIN   CLS----LEDVIQHV----------------------AIPSLLAACKSRT
ANOGA   CFS----LENFVAHV----------------------ALPSLVQACK-GR
TETNG   CLL----LEDLVRCV----------------------AIPSLLNAACS--
DROME   CFS----LADFVSKF----------------------ALPTLARSVSAGG
BRUMA   CFS----INALVHQL-------------------LHTALKSLLNCGIA--
CAEEL   AFY----IPRLLAELTKHVFPLMLRHDGRLTSQKTHDVKRKVASKTTE--
YEAST*  VLG----LEYIIRLL-----------------------------------
SCHPO   LPN----CKTVFEHL-------------------LLFSLEERLDLPA---
CANAL   LLR----INDLVKLI-----------------------------------
CANGA   TYG----LKYVLELF-----------------------------------
ASPNI   CVT----IHSFVLLVNKLSASAQTTGAIANPDSLRLDILELLL-------
RHIO9   VIA----LDTLIKSF----------------------AIPWFERIGQEGQ
CRYNE   FLSTWVVLEKAVFPIWKHAAPFALP-------------------------
USTMA   TLN----LQLAMDTF----------------------LLPYLLSSVRRLT
ARATH*  VIT----LERIVGLL----------------------RLKDYLDFVQFVR
VITVI   VIS----LERMVTVF----------------------RLKEGLDVIQFIR
SOYBN   VTS----LERMVTVL----------------------RLKEGLDVVQFVR
SOLLC   VSS----LERMVSLF----------------------RLKDGLDVVHFVR
ORYSJ*  VVS----LERMMVVL----------------------RLKEGLDILQFLR
HORVU   AVS----LERMIAVL----------------------KVKDGLEVQQVLR
SORBI   AVS----LERMVAAL----------------------KIKDGLDILQLLR
SELML   VLS----LERLVTVL----------------------KIRDGLEALHLPK
PHYPA   VTT----LERMVSVL----------------------RVKDGLENMYLQK
VOLCA   QLL----LADAVMLL-----------------------------------
                 3160      3170      3180      3190      3200
```

```
HUMAN*  ---EQDSEPGARL-----------------TC------------------
CHICK   ---EQDSEPGARL-----------------TC------------------
XENTR   ---EQDSEAGARL-----------------TC------------------
DANRE   ---EQDSEPGARL-----------------TC------------------
CIOIN   SPDLPNPDTSARL-----------------TC------------------
ANOGA   GETTPEIEAGARL-----------------SC------------------
TETNG   ---EQDSEPGARL-----------------TC------------------
DROME   AELSVDAEAGARL-----------------TC------------------
BRUMA   KVLDADAEAGVCL-----------------AL------------------
CAEEL   SRTLSEAEPGVCL-----------------CL------------------
YEAST*  -PINLENNDGSYGL-FLKYHKEQFIKSNFEKILLTCYELEKKYHGNECEI
SCHPO   --------------------------------------------------
CANAL   -----------------QSIQRDEINHIDDTI------------------
CANGA   -DIESESN----CV-FYEYQKRCYIELNYKSV-LSEYNVVSHHEMN----
ASPNI   -PQEESSADMVTYR-FRLAQQEFLVKYPEETL------------------
RHIO9   -QQFCSEDTKLNVF-CQNL-------------------------------
CRYNE   ---------------PRKRLSSKQIQAITSTV------------------
USTMA   DPATRNTEVPMHIGSIV---------------------------------
ARATH*  RTKSSSNGSARSMG-ASKVESPIEVYVHWFRLLVGNCKTVSE--------
VITVI   STRSNSNGNPRSLG-AFKVDNSVEVCVHWFRLLIGNCKTVCD--------
SOYBN   STRSNSNGNARSLM-AFKVDNSIEVHVHWFRLLVGNCRTICE--------
SOLLC   SMRSNSNGNARSVG-ILKADSLAEVSVHWFRVLVGNCRTVSD--------
ORYSJ*  ISKASTNGVTHSIG-NFKIDSSTEVLVHWFKILIGNCKTVYN--------
HORVU   GLRPSTNGASRSSV-TFKMDNSIEVLVHWFRILLGNCRTIYD--------
SORBI   GLKSSTNGVSRPTG-TFRIENSTEVSVHWFRILLGNCRTVYD--------
SELML   -LFGSSNGLSRGVA-MGSTLKSDEVHIHWFRILIGDCKAIAG--------
PHYPA   SGSLNSNGKVSSMGGNAKTESVPEAHIHWFRVLIGDCRTVLE--------
VOLCA   -CYDSSNSNGGGGGGNRASRGSGTTTLAWDVM------------------
                 3210      3220      3230      3240      3250
```

```
HUMAN*  ---RILLHLFKTPQL------------NPCQSDGNK---PTVGIRSS---
CHICK   ---RILLHLFKTPQL------------NPCQQDGNK---PTVGIRSS---
XENTR   ---RILLHLFKTPQL------------NPCEPDGSK---PTVGIRSS---
DANRE   ---RILLHLFRTPQR------------NPCPQDGTKSDKSIVGIRSS---
CIOIN   ---HLLLQLFKAPSS-------------------NK---SSFRLPFS---
ANOGA   ---HLLLRLFKTIECPQPGLYSVSTSPNPI-TVGNA-----HNIKLS---
TETNG   ---RILLHLFKTPQR------------NPVPQDGVK----SVGIRSS---
DROME   ---HLVLKLFKTLEIPQPGMYSVSTSPNPLHAVGND-----FSIRLS---
BRUMA   ---LVLANIVCQSDQPII---------LSPQYTG-------ERISSNICK
CAEEL   ---LIICGLCCVGDEPFG---------LSVHYRGIE--KKKKRFNNT---
YEAST*  NYYEILLKILIT--------------------------------------
SCHPO   ---AFLQDLIGA--------------------------------------
CANAL   ---SFIYELIWG------------------------------ELDKAHFS
CANGA   ---RFIFKI-VN--------------------------------------
ASPNI   ---NIISDAIPL---------------FDADFHDANLGSRRPDLPACTVV
RHIO9   ---IVLIRLLVI--------------------QNDNEPKSEEASLSWPLR
CRYNE   ---NIIAQLLIS---------------------------PPLIPDLAPVS
USTMA   ---SSLGEIFAS--------------------------SLGETVPATSYE
ARATH*  ---GLVLELVGE--------------------------------------
VITVI   ---GLVVDLMGE--------------------------------------
SOYBN   ---GLVVELLGE--------------------------------------
SOLLC   ---GFIVDLLGE--------------------------------------
ORYSJ*  ---GVIAEILGD--------------------------------------
HORVU   ---GLIADILGE--------------------------------------
SORBI   ---GLIADILGD--------------------------------------
SELML   ---GLVADILGD--------------------------------------
PHYPA   ---GLVADLLGE--------------------------------------
VOLCA   ---ACRRALLGP----------------------------PPGLPGCYCR
                 3260      3270      3280      3290      3300
```

MED12

Species	3301–3350
HUMAN*	CDRHLLAASQNRIVDGAVFAVLKAVFVLGDAE------------------
CHICK	CDRHLLAASQNRIVDGAVFAVLKAVFVLGDAE------------------
XENTR	CDRHLLAASQNRIVDGAVFAVLKAVFVLGDAE------------------
DANRE	CDRHLLAASQNSIVVGAVFAVLKAVFMLGDAE------------------
CIOIN	SDRHLLAAAQHSIAVGAVLAVLKGVLKLGDARLSS-------------GK
ANOGA	CDRHLLAAAHKNIGVAPVLAVLKGILVVGDATAHK-------------VS
TETNG	CDRHLLAASQNSIVVGAVFAVLKAVFMLGDAE------------------
DROME	CDRHLLVGAHKTIPIAAVLAVLKAILIVVDNAALKTPLASGSGTSSGGLG
BRUMA	ADRWILTEGHLHDIGDEVCALLVAICMIADYTKHK---------------
CAEEL	ADERIMHLFHWFEMDHAMFRTLGHISQLLE----A---------------
YEAST*	--
SCHPO	--
CANAL	CSQLVLLQISRRMYQE----------------------------------
CANGA	--
ASPNI	LLRTLLAQNSSLVLKYCM--------------------------------
RHIO9	IEEFFQLEVQRQMNLASSLDRIDPLFGL----------------------
CRYNE	LKESLIVQASRQPVL-----------------------------------
USTMA	DMRAFGAQTKLLFA------------------------------------
ARATH*	SSVVAISRMQRMLPLKLVFPPAYSIIAFVLWR------------------
VITVI	PSIVALSRMQRTLPLNLVFPPAYSIFSFVVWR------------------
SOYBN	PSIMALSRMQHMLPLNLVFPPAYSIFAFVRWR------------------
SOLLC	ASILPLFRMQRILPLNLVFPPAYSMFAFVLWR------------------
ORYSJ*	SYVLAFSRLQRTLPLGIVLPPAYSIFAMVLWR------------------
HORVU	SYVLALSRLQQKLPLSVVFPPAYSIFAMVRWR------------------
SORBI	SYILALSRLQQMLPLSVIFPPAYSIFAMVLWR------------------
SELML	ATLVALARTQRLLPLNLVFPMAYAMFCGLLRR------------------
PHYPA	PAVLGLARLQRSLALVTVFPPAYAIFSVALRR------------------
VOLCA	AEAAVRSRIQASLSLDLVWGCLADILPRA---------------------

3310 3320 3330 3340 3350

Species	3351–3400
HUMAN*	------------------------LKGSG-FTVTGGTEELP------EEE
CHICK	------------------------LKGSG-FSHPGGVDDLM------DDE
XENTR	------------------------LRGSG-FSHPTGTDNAS------EED
DANRE	------------------------LKGSG-FSHPAGLDDIG------EDD
CIOIN	NAFSHESELCVTSFLKPIGN----LDDFDDL-HMTGGQDIK---------
ANOGA	SIFGSGKRSGLNTPVHPGSTPKS-MAGSGDLSHILGTSDLSILGNPDESM
TETNG	------------------------LRGSG-LSHTSGLDDIS------E--
DROME	GAFGSGKRSGFNTPVHPGSTPKSNEQRPADLSQILGTSDLQ-LGSSLTSE
BRUMA	------------------------LRDRC--------SD-----------
CAEEL	------------------------LQSRC--------RDANLVLPKNFPI
YEAST*	--
SCHPO	--
CANAL	--
CANGA	--
ASPNI	--
RHIO9	--
CRYNE	--
USTMA	--
ARATH*	--
VITVI	--
SOYBN	--
SOLLC	--
ORYSJ*	--
HORVU	--
SORBI	--
SELML	--
PHYPA	--
VOLCA	--

3360 3370 3380 3390 3400

Species	3401–3450
HUMAN*	GGGGSGGRRQGGRNISVETASLDVYAKYVLRSICQQ---EWVGERCLKS-
CHICK	----LGTRKAGGRVVTVETASLDIYAKYVLRSICQQ---EWVGERCLKS-
XENTR	----IATRRAGGRPVSIETASLDEYAKYVLRSICQQ---EWVGERCVRS-
DANRE	----MGSKKSGGRNVSIETASLVVYAKYVLKSICHQ---EWVGERCLKS-
CIOIN	------NRKSGP-SSSMDGASLSEFAKHAVRVICSQ---QWVREKCLKD-
ANOGA	----LDVSQQNSHLNQDNATSLSDFAQHVLRQICSQ---EWVLERCLQH-
TETNG	-----------GGNVSIETASLDVYAKYVLKTICQQ---EWVGERCLKS-
DROME	----PEALQQPS-VGGMEQISLLEFAQAVLKQICAQ---EHVLERCLKN-
BRUMA	-DKPIDQRVFRRE-------YIAELSKGVLMAMCEQ---EWVTQRIYRR-
CAEEL	KNPPKHQQELHREKAPYRPQYLFNIAKTVQFVICEQ---DWVTLRMFRF-
YEAST*	---------------YGSSPKLLATSTKIIMLLLND---S----------
SCHPO	-------------------------SAYVMEQVPDSWFLEKLPCPLTQS-
CANAL	--
CANGA	---------------HSTSAKLKETASALLTKNLEA---N----------
ASPNI	------------QKLDGHSSFTTVLGKAVDILLGLDPEDGLFPRGINCN-
RHIO9	------------------------MERMILIAT---------------N-
CRYNE	---------------QGSSVGILIQHLPLLVVFEQS---KLLPDQVSHR-
USTMA	---------------QVNLPSLLRAATCLISAFDET---STVP-------
ARATH*	---------------------------------------PFVSNSNSNSS
VITVI	---------------------------------------PFILNANITN-
SOYBN	---------------------------------------PFILNATV---
SOLLC	---------------------------------------PLILNASSGT-
ORYSJ*	---------------------------------------PYLYDTSTSN-
HORVU	---------------------------------------QYIL-----S-
SORBI	---------------------------------------RYIF-----N-
SELML	---------------------------------------QVLSRMSNAS-
PHYPA	---------------------------------------QQTIFVNNMS-
VOLCA	---------------------------------------TVLPSPGGSA-

3410 3420 3430 3440 3450

MED12

```
HUMAN*  -LCEDSN--DLQDPVLSSAQAQRLM--QLICY------------------
CHICK   -LCEDSN--DLQDPVLSSTQAQRLM--QLICY------------------
XENTR   -LCEDSN--DLQDPVLSSAQAQRLM--QLICY------------------
DANRE   -LSEDSS--ALQDPVLVNIQAQRLL--QLICY------------------
CIOIN   -PVMD----LLMDQVLTREQRQNLI--QLICY------------------
ANOGA   -AEELCQHGMLIDNLLTAKQAQRLL--HMICY------------------
TETNG   -LSEDSS--ALQDPVLVNIQAQRLL--QLICY------------------
DROME   -AEQLCD--MIIDEMLTAKQAQRVL--HMICY------------------
BRUMA   -CEDGALDCFSSHQLRKNCLGQQLL--RMALR------------------
CAEEL   -FQTRKMEAFNQDKLKQNCLGQQIL--RMALR------------------
YEAST*  -----------------VENSSNIL--EDILYYSTCPSETDLNDIPLGSG
SCHPO   -LQSFSHLSNHIEVLDSTRQSRLTFLCHLILH------------------
CANAL   -KSSYQCIILLQQRLLKNKESQLQTNPFFIKY------------------
CANGA   -----------------KDKAIQLF--YQLVYYQ--------NNLEFLSS
ASPNI   -VQS-------TDGHIEMEPSSQAE--RVILM------------------
RHIO9   -LPLSSSLLQDLVMLRADLLQISWF--RQACL-------RDLNGVYQRFA
CRYNE   -----------IDEILRNLAMTAEF--KTAAF-------RNLNLLKDAFL
USTMA   -ASEKAQIESFCNALLQSQPMQIAF--RQEPRLCILAIKDTCKSMWGCNP
ARATH*  VHEDTHRLYQSLTMAFHDVIKHLPF--RDVCF-------RDTQGLYELIV
VITVI   -REDIHQLYQSLTLAISDAIKHLPF--RDVCM-------RDTHGFYDLVA
SOYBN   -REDMNQIYQSLTMAITDAIKHLPF--RDVCF-------RDCQGLYDFMA
SOLLC   -RDEVQHLHHSLMLAFADVIKHLPF--REVCL-------RDTHSLYDLIA
ORYSJ*  -HEDI-QLYQSLLGAISDITRHQPF--RDVCF-------RNMHLFYDLLA
HORVU   -REDM-QVYQSIANAINDITRHQPF--RDICF-------RNTHQLYDLLA
SORBI   -REDP-QLYQSLSNAISDITRHQPF--REICF-------RNTHRLYNLLA
SELML   -RDD--SILQSLSVAVADIVKHEPF--REVCL-------QDTRALYVLLS
PHYPA   -REE--AIIQAVTVAGNDVVAHEPF--RDVCL-------RDTTSLYRCLS
VOLCA   -AAGAAAPGSDPHVIAAVLVSYPPL--RHCLL-------SDPRALHERLC
                  3460      3470      3480      3490      3500
```

```
HUMAN*  ---------------------------PHRLLDN-----EDG--ENP---
CHICK   ---------------------------PHRLLDN-----EEG--ENP---
XENTR   ---------------------------PHRLNDT-----EQG--DNP---
DANRE   ---------------------------PHRQLDS-----EEG--DNP---
CIOIN   ---------------------------PENLLQV-----KLSGLSNE---
ANOGA   ---------------------------PEHES-N-----LIA--ELE---
TETNG   ---------------------------PHRQLDS-----DDG--DNP---
DROME   ---------------------------PEPEF-N-----IIS--ELD---
BRUMA   ---------------------------RRSEREIVRELTICN--GNS---
CAEEL   ---------------------------RRTERENVHKLFEAH--KIS---
YEAST*  QPDNDTVVTNDDKSDDDDHTVD----------------------------
SCHPO   --------------------------------------------------
CANAL   ---------------------------KADLLSFFHKAIITNTKAIQSQF
CANGA   EELQEFAVNNSPQ-------------------------------------
ASPNI   --------------------------------------------------
RHIO9   SREAEAVTEKRL---------------KKKMLSIVEELIGGDLVASQSII
CRYNE   AREWSGP--------------GMDQGLEGKMIDVLKRIMSEKPTQPS---
USTMA   SRVIDLAMACLDPTSLLLR-------------------------------
ARATH*  ADSTDAEFASVFESHGLDMHLKSVAFAPLRARLFLNSLIDCKVPSSGYSH
VITVI   ADASDSEFAAMLELNGPDLHLRAMAFVPLRARLFLNAIIDCKMPNTSLTQ
SOYBN   ADASDSEFATLLEFNGSDMHLRSTAFVPLRSRLFLNAMIDCKMPQSIYTK
SOLLC   ADTVDSDFASLLEASGVDLRLKTSSFVPLRARLFLNALIDCRIPQTIAKL
ORYSJ*  ADVGDSEFAAIVELRSPDECLKALS--PLRARVFLNALLDCEIPVTM--R
HORVU   ADVGDSEFAAMLETHCSDKNVKQL-FVPLRGRLFLNSLVDCKTPAAI--Q
SORBI   SDVGDSEFAAMLESHSPDRNSKILPFIPLRARLFLDALIDCNTPTI---Q
SELML   SDGGSSDYAALLDEQGFDARLKVEAMLPFSGRLFLHSILDGVLPSSMQWQ
PHYPA   GDSGDSKYAAMLDVQGFELYKKVSAMVPLRARLFLHALLDRKMPEDDTRS
VOLCA   SPAAVQAAATLLASVTEVPAGEPPLRLPQ--RLVVVSLASGRLVQLPAVA
                  3510      3520      3530      3540      3550
```

```
HUMAN*  ------------------------QRQRI--KRILQNLDQWTMRQSSLE-
CHICK   ------------------------QRQRI--KRILQNLDQWTMRQSSLE-
XENTR   ------------------------QRQRI--KHILQNLDQWTMRQSALE-
DANRE   ------------------------QRQRI--KRILQNMDQWTMRQSSLE-
CIOIN   ------------------------PRKYV--VSVLSGMTRWTFRQCLLE-
ANOGA   ------------------------QKAII--VRILENLEQWSLRISWLD-
TETNG   ------------------------QRQRI--KRILQ--------------
DROME   ------------------------QRSMI--VRILENLGQWTLRISWLD-
BRUMA   ------------------------KKSLI--DKLLNVLNIWNTRATYFD-
CAEEL   ------------------------KKATV--DKVLSFMNLWNFRATLFD-
YEAST*  ------------------------EIDHVEYYVMMDFANLWVFQAF----
SCHPO   ------------------------MHGFVELTDQLATLESLTIRKCIYRN
CANAL   FDKLSHSDSLQLLNVLLQRQENEYINTIQDFSLFIKHINEFNLPICQVL-
CANGA   -----------------------------KLYSYLDFTNLWLFQSF----
ASPNI   -------------------------------------NNDFSLPYCQLK-
RHIO9   QDT---------------APN---FIDKL--HKVFLNVSQWNEAQCRVQ-
CRYNE   ------------------------PKQGLPNFDTSARFSAWRWTRVVLE-
USTMA   ------------------------DVGTIDAASVKNRLDGWDTAVVATE-
ARATH*  EGV-------SEAKNRHQGNGTK-LVDKL--VSVLDCLQPAKFHWQWVE-
VITVI   DDV-SWVSGHAESKVPYAENETK-LLDKL--VHILDTLQPAKFHWQWVE-
SOYBN   DDG-SRMSGPGESKIKFTDSESK-LQDML--VHVLDTLQPAKFHWQWVV-
SOLLC   NDG-NQLALQGESKFHSAENETK-LLDKL--VYILDTLQPAKFHWQWVE-
ORYSJ*  DDG-TYALEPGCAE-ASTKNDVK-FPERL--IEILNVLQPAKFHWQWVE-
HORVU   VDG----SEPGEAK----ENELKLLSERL--VQSLDTLQPAKFHWQWVE-
SORBI   GDG---ASEPCDPK----DNELK-LSERL--MQLLDTLQPAKFHWQWVE-
SELML   DDE-------AWVKVRHVHDPKH-AQQQM--VLILDELQPATFHWQWLE-
PHYPA   QNR------------SHTSNGPG-QIEQV--VQVLDELQAATFHWQWIE-
VOLCA   SLEGAAGLDSDALL-----------------VAALASLESSCFPLMWLL-
                  3560      3570      3580      3590      3600
```

MED12

```
HUMAN*  ---LQLMIKQT--PN-------------------NEMNS-----------
CHICK   ---LQLMIKQT--AN-------------------NEMNS-----------
XENTR   ---LQLMIKQT--PS-------------------NEMNS-----------
DANRE   ---LQLMIKQS--SN-------------------NELYS-----------
CIOIN   ---LQVMLRQS-----------------------PQEKS-----------
ANOGA   ---LQLMFKQTNCSS-------------------PDLSN-----------
TETNG   -----------------------------------ELYS-----------
DROME   ---LQLMYRQSLSNN-------------------AELNV-----------
BRUMA   ---FKLMIREISPEGCTSKHA---------QQGAIAADA-----------
CAEEL   ---LMLMIKEISPDG-NSRHA---------QQGAIAADA-----------
YEAST*  ---------TCFCIKKIMEN----------NEPAMAMED-LKNFIFQIIE
SCHPO   QELLDLLLFSIHLVKPNVETN-----------------------------
CANAL   ---LNVLFNNLVVDNDVGIKV-----------------------------
CANGA   ---------TKFYIHETFQNT---------NEDKVKVGD-V---IFSIIE
ASPNI   ---LKLLFNAK---------------------------------------
RHIO9   ---MNLLLDNISLYDVNSQNP---------NNPHILGDDLSMDITSDICV
CRYNE   ---MRVEFKALTMRIAEGQNV---------AEAKQTLNQLVHATLDRETT
USTMA   ---LVEIFERLQLVESSFQTR-----------------------------
ARATH*  ---LRLLLNEQALAEKLENHD---------MPLTDAIRS-----------
VITVI   ---LRLLLNEQALVEKVDNHD---------VSLAEAIHS-----------
SOYBN   ---LRLLLNEQALIEKLENRD---------VSLADAIKL-----------
SOLLC   ---LRLLLNEQAVMEKLEAHD---------LSLVEALRS-----------
ORYSJ*  ---LRLLLDEQSLIEKPKN-----------VPYVKALRS-----------
HORVU   ---LRLLLDEQALAEKLDKAEKSKIPMPILMTLADGLRK-----------
SORBI   ---MRLLLDEQALMEKVAAGK----------TALESLRS-----------
SELML   ---LRLHLNELVFVEKIEAHN----------SAVKAMQA-----------
PHYPA   ---LRLLLNEQVILEKLKIHP---------QNAREAVLA-----------
VOLCA   ---LRLLVDEQALLLAVGSRTAPAG-----SEARRGLREAMEAAVAPPVT
                            3610      3620      3630      3640      3650
```

```
HUMAN*  -----------------------------------LLENIAKATIEVFQQ
CHICK   -----------------------------------LLENIAKATIEVFQQ
XENTR   -----------------------------------LLENIAKATIEVFQQ
DANRE   -----------------------------------LLENIAKATIEVFQK
CIOIN   -----------------------------------LPEHIAKATIEVFQQ
ANOGA   -----------------------------------WLDMVARAAIDVFRV
TETNG   -----------------------------------LLENIAKATIEVFQK
DROME   -----------------------------------WLDTVARAAIDVFHM
BRUMA   -----------------------------------LLGEIGKCCRDLFTQ
CAEEL   -----------------------------------LMSEIGKCCRDMFLS
YEAST*  IT-----NSN------------------------DLCSQIFDQLKDMQTI
SCHPO   ----------------------------------DEVCNTLKAWENIESR
CANAL   ---------------------------------------QLEAFVNTLLQ
CANGA   TT-----KYN------------------------VLCAKIFEDIRDHRVT
ASPNI   -----------------------------------AGNEVKNHIVDVMFK
RHIO9   S------PSN------------------------KDLQSFVSFFYDVVLA
CRYNE   ADDT------------------------------DLLCEVFKGVDSMVTQ
USTMA   ------------------------------------ADSKTKALASGIFD
ARATH*  --SCPTSEK-------------------------PDASENEKNFIQILLT
VITVI   --MSPNPEK-------------------------AVASENENNFILIILT
SOYBN   --SSPSTEKA------------------------AAASENEKNFIQILLT
SOLLC   --LSPNTDK-------------------------ASVSENESNIIEMILT
ORYSJ*  --LSPNAEN-------------------------FTLSEREKGLTEVILS
HORVU   --LSPNSES-------------------------FTLSESEKGFTEIILS
SORBI   --LSPNAEG-------------------------FALSDSEKGFTEVILS
SELML   --SVGAS---------------------------DQLYECEKVFTENVLT
PHYPA   --AMQGPER-------------------------QQLDECEKTFTEILLT
VOLCA   VTMDGKDGSAPGSGARGGGGGGGGGGGPTGPGGGAELRAADKALCDAALV
                            3660      3670      3680      3690      3700
```

```
HUMAN*  SAETGSSS----------GSTASNMPSSSKTKPVLSSLERSGVWLVAP--
CHICK   SAETSSASSAGNGVNNI-SSSASATPASNKSKPILSSLERSGVWLVAP--
XENTR   SAENACAAPANSAALS--NATSSNAPPNSKTKPVLSSLERSGAWLVAP--
DANRE   SAEMNSSNPSWNGSAVS-GSSVSNSNSASKLKPVLSSSERSGVWLVAP--
CIOIN   QQQQQNLD----------------STKERKSSLTGPELDRCNVWLVAP--
ANOGA   NEFILNSP----------------DVKQEKVKP--------STWLVAP--
TETNG   SAEMNSSNPSGNGAVGSGGSTSNNNSTASKMKPILSSSERSGVWLVAP--
DROME   EEVVLPGA----------------VKATHKPKP--------STWLVAP--
BRUMA   SHKSDKKLPP------------------AAVGKAFRLKDITAYWLISP--
CAEEL   AYKTKIKMPI------------------AKTLTDFRLSDINKFWLIAP--
YEAST*  EMITQIVE---------------KDFCTSCLQNNNQKIDDNYIVVVIE--
SCHPO   PYTIDFPE---------------------------------ALQQYSPRI
CANAL   EIKFEFSN---------------------------------ENSFFGE--
CANGA   DLVIELFE---------------QDFFKKIISGEEFKAE--FLQMLIE--
ASPNI   AAVTDSRS---------------------------------KRSHWVG--
RHIO9   DGHFERAA------------------------------TQRRFQFFKN--
CRYNE   EILAAGMER-----------------------------------------
USTMA   D-------------------------------------------------
ARATH*  RLLVRPDA----------------------------------VPLFSE--
VITVI   RLLARPYA----------------------------------AALFSE--
SOYBN   RLLVRPDA----------------------------------APLFSE--
SOLLC   RLLVRPDA----------------------------------APLFSE--
ORYSJ*  RLLVRPDA----------------------------------APLYSE--
HORVU   RLVARPDA----------------------------------APLYSE--
SORBI   RLLARPDA----------------------------------APLYSE--
SELML   NVLFRPDA----------------------------------GALYSE--
PHYPA   RLLVRPDA----------------------------------AALYSE--
VOLCA   ALLGAPGR----------------------------------AELFAE--
                            3710      3720      3730      3740      3750
```

MED12

Species	3751–3800
HUMAN*	LIAKLPT--------------SVQGHVLK--AAGEELEK-----------
CHICK	LIAKLPT--------------SVQGHVLK--AAGEELEK-----------
XENTR	LIAKLPT--------------SVQGHVLK--AAGEELEK-----------
DANRE	LIGKLPT--------------SVQGHVLK--AAGEELEK-----------
CIOIN	LVSKLPS--------------QVQGRVLQ--EASAILEA-----------
ANOGA	LVSKLPS--------------AVQGRILK--VSGQVLEST----------
TETNG	LIAKLST--------------AVQGHVLK--AAGEELEK-----------
DROME	LIAKLTP--------------AVQGRILR--VAGQVLESM----------
BRUMA	LVHACPRPGNLPASFPAI---TVQGKFLK--EAAAMLDT-----------
CAEEL	LVRMCPKPINIPPQYANTTVGTVAAKFLR--EAALLMDT-----------
YEAST*	IITSLSM---
SCHPO	VLHLFSFFYWKCQHDDYLQENTSSKNNLL--QKEWILITK----------
CANAL	LFNFMPW--------------NQKNYILDILETKFLLETNLDNNQIKLII
CANGA	IINHLSM---
ASPNI	LVSLMDQ--------------EAARQIRERAEGCFFSVAMFD--------
RHIO9	LIHELRK--------------PVLSELLA--HAVRLLEGD----------
CRYNE	LATLLGHAIAAESQQQLETHIKSIDQLLRILDSMNNLPSQ----------
USTMA	LFVQLPD---------------IGAQLVRDCQSSGLVSRF----------
ARATH*	VVHLFGR--------------SVEDSMLK--QAEWFLAGQ----------
VITVI	VVHLFGR--------------SLEDSTLL--QAKWFLVGQ----------
SOYBN	LIHLFGR--------------SLEDSMLS--QAKWFLAGQ----------
SOLLC	VVHLLGR--------------SLEDSMLL--QAKWFLGGN----------
ORYSJ*	LIHLLGK---------------LQESFVM--GIKWFLQGQ----------
HORVU	VVHLLGK---------------LQESLVV--DVKWILQGQ----------
SORBI	VVHLLGK---------------LQESLVM--DVKWILQGQ----------
SELML	VLHSLGK--------------PLEEYIIK--LVKWSLQGV----------
PHYPA	VVHLLGK--------------ALEDYLIL--HVKWVLENN----------
VOLCA	MAWQLGR--------------GVGEYLLK--STDWLLQAVLGTSAEAGPS

3760 3770 3780 3790 3800

Species	3801–3850
HUMAN*	--
CHICK	--
XENTR	--
DANRE	--
CIOIN	--
ANOGA	-------------SMFSKHKDGNGGNNS----------------------
TETNG	--
DROME	-------------NYFSKVSK----SDC----------------------
BRUMA	--
CAEEL	--
YEAST*	--
SCHPO	--
CANAL	NDRNLLPPLNDFF-------------------------------------
CANGA	--
ASPNI	--
RHIO9	--
CRYNE	--
USTMA	--
ARATH*	------------------------DVLFGRKTIRQKLIIVGESKGLPTKP
VITVI	------------------------DVLFGRKSIRQRLINIAESKGLSTKV
SOYBN	------------------------DVLFGRKTIRQRLHNIAVKKNLSVKT
SOLLC	------------------------DVLLGRKSVRQRLHNIAVSRGLSTRA
ORYSJ*	------------------------DVLLGNNSVRQQLVNLTQRKGFPMKT
HORVU	------------------------DAVLGRKSTRQQLLTIATRRGVPIKA
SORBI	------------------------DAILGRRSTRQQLVHIAQRKGLSTKA
SELML	------------------------DVLYGKKSLRQRL---EQTKGYIRPT
PHYPA	------------------------DMLLGRKSLRQLLDNIAA-KSVPVRA
VOLCA	NGLVLAFLYYLLTDVQFYVRQDRKGEQLLGHTSLAQLVARLGRTAKPGMA

3810 3820 3830 3840 3850

Species	3851–3900
HUMAN*	--GQHLGSSS--
CHICK	--GQHLGSSS--
XENTR	--GQHLGSSS--
DANRE	--GQHLGPSS--
CIOIN	--PQNLSSTSTR
ANOGA	--------------------------------SSNNSHNHGANSNSSV--
TETNG	--GQHLGSSS--
DROME	--------------------------------NSSGSGDEREKSNSCH--
BRUMA	--SSDN--
CAEEL	--PPTT--
YEAST*	-----------------------RFQRETSGMIVISMENYHLLIKIIRQL
SCHPO	--
CANAL	--KKFSSS
CANGA	-----------------------HKGKTNDRSGEQSHTSYELCVQVMAHF
ASPNI	--ESMDDTSLPS
RHIO9	--PSQA
CRYNE	--
USTMA	-------------------TDIGIKILSDKVKAASTDADKTPRRMDNSHK
ARATH*	QFWKPWGWCNSSSSDHITANKAGKKRKF----EITSIEEGEVIEEGSGSR
VITVI	QFWKPWGWS-YSSLDPVATK--GDKKKF----EVTSLEEGEVVEEGTDSK
SOYBN	QFWEPWGWC-SLSTDPLTVK--GDNKKF----DSTSLEEGEVVEEGMDLK
SOLLC	QYWKPWGWC-TTNSDPTTSK--REKLKS----EVSSIEEGEVVDEGTTLK
ORYSJ*	QFWKPWGWS-KLVGYANANK--SSKRKL----EVTSIEEREV-DGLIDSR
HORVU	QVWKPWGWS-SLLSDVMANR--SAKRKL----EAAPIEEGEVVDDSADAK
SORBI	QVWKPWGWS-SLLSDVIANK--AAKRKL----EVTSIEEGEVVDDTVDAK
SELML	SALKSWGWQPPWSVV-----------------EAASPEEGEVSQDNASAA
PHYPA	RQVRALNWQ-PPRPLHRDSEEAAEKKQWSLQGGAASPEEGELEDERMDWQ
VOLCA	PLAAAAGGLPYIPAATAPGGAGGMLPG-----NGAADMDAGLAIIRAATP

3860 3870 3880 3890 3900

MED12

```
HUMAN*  -----RKERDRQKQ----KSMSLLSQQPFLSLVLTCL-KGQ--DEQREGL
CHICK   -----RKERDRQKQ----KSMSLLSQQPFLSLVLTCL-KGQ--DEQREGL
XENTR   -----RKERDRQKQ----KSMSLLSQQPFLSLVLTCL-KGQ--DEQREGL
DANRE   -----RKERDRQKQ----KSMSLLSQQPFLSLVLTCL-KGQ--DEQREGL
CIOIN   SGAGARSERD-NRQ----GGSSLLNHQPFLTLILTCL-KGQ--DEQRDSL
ANOGA   -----SSNGSTFGS----KQSAQLNHQPFLGLVLTCL-KGQ--DEQKEGL
TETNG   -----RKERDRQKQ----KSMSLLSQQPFLSLVLTCL-KGQ--DEQREGL
DROME   -----SSNSYGLGGVPARNKKMPLDYQPFLGLILTCL-KGQ--DEYKENL
BRUMA   -----NKERIQ-------QSTWLLSQEPFLNLVLTCL-NGE--EQQRDGL
CAEEL   -----PKERLL-------QCSWAMSKVPFINMILTCL-QCEKMQQSKDVF
YEAST*  SELNEGNLSKREIQIDAVLKIFSFHQDSIFQRIIADL----SADKPTSPF
SCHPO   ------------------KKVILEQCEYVILDILNFLKFSGIPTQSKKRL
CANAL   SSSSTSSSSSSLVQTIETDSSFFLNLFNFIGNLVNVA------------N
CANGA   QQLSDSELSAKEIELDVFMKIFTVHQNSVFQEILRDT----ES---SSAM
ASPNI   GASSLSSIESAKLYLNIIEKLAYSIPQAGVQSIPPLL------VERLDLL
RHIO9   FPDSVLLMPSSEQHAPFDSNTFAYRSQAFLNMTQHMLAENVWPNEKKVDL
CRYNE   ---------------SVTEPSVLNARHKLLDLLALAL-------------
USTMA   GMTNFGPDDGADAPAQAKTAADAAKHGPCVG------------EQQVEST
ARATH*  KVL--LPRVLD--ENSPSVGYGITTERAFVQLVLPCI--DQSSDESRSTF
VITVI   RYAKGSTQMSD--FDGFNVSQQHATERALVELVLPCI--DQSSDDSRNAF
SOYBN   R------------------CQLQVTERALIEMLLPCI--DQSSDESRNSF
SOLLC   RPVKGSGRTVD-------VEKLHVTERALVDLILPCL--DQASDDSRSTF
ORYSJ*  KSSNRKSLNVGTSPEGHGSTQKYFTQEALAELVLPCI--DRSSSEFRFVF
HORVU   RPSKSTLH-----------INKYVTEKAFAELMLPCI--DRSSPEFRSIF
SORBI   RPSKTPPHNVDRSFDAIRSINKYLTEKALAELVLPCI--DRSSADIRGIL
SELML   GGSSRDDA----------LSLHFAIEKALADLVLPCL--ARSLNDTCSGF
PHYPA   KSDEKPSRQSSGTLSGL-TCKPFATEKALADLVLPCL--ARSSMDNRNGF
VOLCA   AADVGGGAAAGAVVAVAQPPPPFAVESAVADLALAGL--YKAGTVRQREF
                 3910      3920      3930      3940      3950

HUMAN*  LTSLY-SQVHQIVNNWR---------------------------------
CHICK   LTSLY-SQVQQIVTNWR---------------------------------
XENTR   LTSLY-GQVQQIVSNWR---------------------------------
DANRE   LTSLY-SQVQQIVTNWR---------------------------------
CIOIN   LESLQ-SQIVMLHSEWQ---------------------------------
ANOGA   LQSLY-SQLSQFLQNRD---------------------------------
TETNG   LTSLY-SQLQQIVTNWR---------------------------------
DROME   LVSLY-AQLSQCLQSFA---------------------------------
BRUMA   VGSML-KQLQELALKAK---------------------------------
CAEEL   LQSLY-TQLQRETL--R---------------------------------
YEAST*  IDSIC-KLFDKISFNLR---------------------------------
SCHPO   EVHML---------------------------------------------
CANAL   SQELILEQANSINDTIS---------------------------------
CANGA   IESLF-ALFERVNFSLR---------------------------------
ASPNI   LQKLIIMQINSNSVAAS---------------------------------
RHIO9   VKTVF-RQILRFRDSLS---------------------------------
CRYNE   ------QTVERNLSTTR---------------------------------
USTMA   LRSVV-RMCEQQHSFIF---------------------------------
ARATH*  VNELV-RQFSNIEQQLS---------------------------------
VITVI   ASDLI-KQMHIIEQQIN---------------------------------
SOYBN   ASDMV-KQLSYIEQQIT---------------------------------
SOLLC   ASDMI-KQMNLIEQQIN---------------------------------
ORYSJ*  AGDLI-KHMGVISEHIK---------------------------------
HORVU   AGELI-KQMGTVSEHIK---------------------------------
SORBI   SVDLI-KQMGAISEHIK---------------------------------
SELML   AFDLV-SQMAELEQQVT---------------------------------
PHYPA   AHELV-KEMNNLEQHIS---------------------------------
VOLCA   AGELI-RQLVRMADAVRRAALAAPLGATAAGGGGGSGLGAASAIGMQGMG
                 3960      3970      3980      3990      4000

HUMAN*  --------------------------------------------------
CHICK   --------------------------------------------------
XENTR   --------------------------------------------------
DANRE   --------------------------------------------------
CIOIN   --------------------------------------------------
ANOGA   --------------------------------------------------
TETNG   --------------------------------------------------
DROME   --------------------------------------------------
BRUMA   --------------------------------------------------
CAEEL   --------------------------------------------------
YEAST*  --------------------------------------------------
SCHPO   --------------------------------------------------
CANAL   --------------------------------------------------
CANGA   --------------------------------------------------
ASPNI   --------------------------------------------------
RHIO9   --------------------------------------------------
CRYNE   --------------------------------------------------
USTMA   --------------------------------------------------
ARATH*  --------------------------------------------------
VITVI   --------------------------------------------------
SOYBN   --------------------------------------------------
SOLLC   --------------------------------------------------
ORYSJ*  --------------------------------------------------
HORVU   --------------------------------------------------
SORBI   --------------------------------------------------
SELML   --------------------------------------------------
PHYPA   --------------------------------------------------
VOLCA   SGTLPPPPPLPPPPTMMPGRSRSIGTGGGVSAAAVGGVSARAPSEDQDAL
                 4010      4020      4030      4040      4050
```

MED12

```
HUMAN*  -----D--------------------------------------------
CHICK   -----E--------------------------------------------
XENTR   -----E--------------------------------------------
DANRE   -----E--------------------------------------------
CIOIN   -----D--------------------------------------------
ANOGA   -----Q--------------------------------------------
TETNG   -----E--------------------------------------------
DROME   -----E--------------------------------------------
BRUMA   -----D--------------------------------------------
CAEEL   -----D--------------------------------------------
YEAST*  --------------------------------------------------
SCHPO   --------------------------------------------------
CANAL   --------------------------------------------------
CANGA   --------------------------------------------------
ASPNI   --------------------------------------------------
RHIO9   -------------------VYKVMQSVNICYAEASAALQATKNNTDMAIT
CRYNE   --------------------------------------------------
USTMA   --------------------------------------------------
ARATH*  ------SVTNRSTTSNKQMGTASS------GSEISSNKGSTRKGLRGGSP
VITVI   ------TVTRGTT---KQAGTVLS------GVEGPANKGNNRKGMRGGSP
SOYBN   ------AVTGGS----KSVGSAPP------GVEGQPNKVNNRKNMRGGSP
SOLLC   ------AVTREAS---KPAGTVAS------GIESPTTK-SSRKGTRGGSP
ORYSJ*  ------AAVWNGI--NKLNSSNPS------GNEGLS-KPNGRKGIFSGSP
HORVU   ------AISRNGA---KQVGLVPS------GNDVSSNKSSGRKGIRGGSP
SORBI   ------AIARNGA---KQAGSVPL------GNEVPSNKSSGRKGIRGGSP
SELML   ------LLTRSSA----KFSVAPSTAD---GPAAKNQRRGARSGLETASP
PHYPA   ------NLTRNSG---RTIASTSPGGDGILGSKGSSVRRGGRSGMDVGSP
VOLCA   LSGLGDGDTAGGGGAGDGGDGTAGGGRKRGGSGMGFGSGRTRRMGGGGGG
                        4060      4070      4080      4090      4100

HUMAN*  ----D-----QYLDDCKPKQLMHEALKLR---------------------
CHICK   ----D-----QYQDDCKAKQLMHEALKLR---------------------
XENTR   ----D-----QYQDDCKTPQLLHDVLKLR---------------------
DANRE   ----D-----QYQDDCKAKQMMHEALKLR---------------------
CIOIN   ----ARSHRGSCKDLNNSQNCVHDALQLR---------------------
ANOGA   ----SLETIGGIEDPCGFEKML-DALQLR---------------------
TETNG   ----D-----QYQDDCKAKQMMHEALKLR---------------------
DROME   -----LDTIGGIDEPQAREEIL-DALQLR---------------------
BRUMA   ----N-----PLLPYLRIFSLEREGVLLR---------------------
CAEEL   ----H-----HRRSNWTNRREHRDCTIFR---------------------
YEAST*  ---------------------------LK---------------------
SCHPO   -------------------NFISSTLYLK---------------------
CANAL   -------------------------IFLRILIIHKSYLCSMISRNTNEEN
CANGA   ---------------------------LK---------------------
ASPNI   ---SSGSTIVSKINFERALAFWFSALLRL---------------------
RHIO9   MLLTEGSLESTLVEMDHERKLSLQDIRTR---------------------
CRYNE   -DGSDLLLPPGISPPQPSHLLKAVMGLLK---------------------
USTMA   -----------------NATDADSCAQLL---------------------
ARATH*  SLARRSSANTTDTSPPPSPAALRASMSLR---------------------
VITVI   GLARRPTGVA--DSAPPSPAALRASMALR---------------------
SOYBN   ALTRRQTVAT--DSSPPSPAALRASMSLR---------------------
SOLLC   GLARRATGPA--ETVPPSPGALRASLSLR---------------------
ORYSJ*  NIRKHSPVPN--DSTTPSASALRSSIWLR---------------------
HORVU   NIGRRGTVGN--DPTPPSASALQAIVWLR---------------------
SORBI   NIGRRAPVGN--DPSPPSASALRAALWLR---------------------
SELML   SVRRRPP-----EASAVSAAALQSSMWLR---------------------
PHYPA   GLGRRFTGPVP-EAVPVSPAALQTSVWLR---------------------
VOLCA   LVRSDSRGDLTEALRIGSPAALQVALWVR---------------------
                        4110      4120      4130      4140      4150

HUMAN*  -LNLVGGMFDTVQRS------TQQTTEWAML-----------------LL
CHICK   -LNLVGGMFDTVQRS------TQQTTEWAVL-----------------LL
XENTR   -LNLVGGMFDTVQRS------TQQTTEWAVL-----------------LL
DANRE   -LNLVGGMFDTVQRS------TQQTTEWAVL-----------------LL
CIOIN   -LSLVGGMFDTVMSN------SQWTSDVVVL-----------------LM
ANOGA   -YSLVGGLFDAIMKN------ATSTTDWAIL-----------------FA
TETNG   -LNLVGGMFDTVQRS------TQQTTEWAVL-----------------LL
DROME   -FSLVGGMFEAIQKN------STPTTDWAIL-----------------LA
BRUMA   -ISLVGGMFDSVCHT------SICDT-WALT-----------------LF
CAEEL   -ITLIGYIYKEILKA------THVET-WGLL-----------------IF
YEAST*  -LFLY---------------------------------------------
SCHPO   -VSFCSSNF-----------------------------------------
CANAL   VILFVTKLIDLLNSDYLTNHNDKLRILLYDL-----------------LL
CANGA   -LMFY---------------------------------------------
ASPNI   -IVLHRAAFNVPPASGSKVDSLREQTRLLVS-----------------IL
RHIO9   -LRLIVPYMSLIWEHPKED--ECDILDWIRT-----------------LV
CRYNE   -FTLGSVVSESVSLTAPKPNFPHLAVCFLKA-----------------IF
USTMA   -LHVVTSLEDALGSMAAASHQDKVARLVTVLHGSMTFQLMHLVLRFGCLW
ARATH*  -LQFLLRLLPVICGE---PSFKNTRHALAST-----------------IV
VITVI   -LQFLLRLLPIICAEGE--QSRNMRQSLASV-----------------IL
SOYBN   -LQLLLRFLPILCTDRE-PSVRSMRQFLATV-----------------IF
SOLLC   -LQFILRLFSIIYADRE-PSGRNMRHVLASV-----------------IL
ORYSJ*  -LQFIIRLLPVIIAD------SNMRQTLASS-----------------LL
HORVU   -LQFIIRLLQVILAD------RGMRHTLAPA-----------------IL
SORBI   -LQFIIRLLPVIMAD------RSMRQTLASA-----------------IL
SELML   -LQFLLPLLRIIYCDRE-----NMRLTLAPV-----------------LL
PHYPA   -LQFLLPLLPIILAEREQQSVPNKRQTLAPV-----------------LL
VOLCA   -VRVLMPLLPLIYMDRDPDPRKNLRSQLVPA-----------------LL
                        4160      4170      4180      4190      4200
```

MED12

```
HUMAN*  ---------EIII-------------------------------------
CHICK   ---------DIIS-------------------------------------
XENTR   ---------DIIS-------------------------------------
DANRE   ---------DIIS-------------------------------------
CIOIN   ---------QLII-------------------------------------
ANOGA   ---------QLIS-------------------------------------
TETNG   ---------DIIS-------------------------------------
DROME   ---------QLVC-------------------------------------
BRUMA   ---------QLML-------------------------------------
CAEEL   ---------QLMF-------------------------------------
YEAST*  ---------EILSSL-----------------------------------
SCHPO   --------------------------------------------------
CANAL   ----------LMKSSVSEEVNSTRELNENSQGNRNGTG------------
CANGA   ---------EVLSSL-----------------------------------
ASPNI   CISLARLPENILR-------------------------------------
RHIO9   ---------QLLGSPLVHGNG-----------------------------
CRYNE   VSSRPVLAKRLLT-------------------------------------
USTMA   NGTMRALALRLVKALL----------------------------------
ARATH*  ---------RLLGSRVVYEDYAVCSP--RSELSK----------------
VITVI   ---------RLLGSRVVHEDADLSLYSTQSPPSK----------------
SOYBN   ---------RLLGSRVVHEDVDISVNAVPS-LSI----------------
SOLLC   ---------RILGSRVVHEDASYSFN--QACNSK----------------
ORYSJ*  ---------SLVGTRVVYEDADS---------------------------
HORVU   ---------SLLASRIIYEDADFPPPPASPIASR----------------
SORBI   ---------GLLATRMIYEDADLPLPPTNANALR----------------
SELML   ---------RLLGTRVVYESAESFGIHQPESSML----------------
PHYPA   ---------RLLGTRIVQEETDPLCCWDPSSSS-----------------
VOLCA   ---------QLLASPLIWAAAPPPPLQLGATGTAAVAASTAAAAAAAAPG
                 4210      4220      4230      4240      4250
```

```
HUMAN*  -------------------------------------------------S
CHICK   -------------------------------------------------S
XENTR   -------------------------------------------------S
DANRE   -------------------------------------------------S
CIOIN   -------------------------------------------------S
ANOGA   -------------------------------------------------Q
TETNG   -------------------------------------------------S
DROME   -------------------------------------------------Q
BRUMA   -------------------------------------------------Y
CAEEL   -------------------------------------------------H
YEAST*  -------------------KSFAIY-------------------SSTIDA
SCHPO   --------------------------------------------------
CANAL   --------------------------------------------TSGGGS
CANGA   -------------------KSYCTY-------------------EAGIS-
ASPNI   -----------------LFPAADYF-------------------PHTIQS
RHIO9   --------------------------------------------------
CRYNE   --------------------------------------------KPQAGD
USTMA   --------------------------------------------ELTRQA
ARATH*  -------------------AETEST-------------------IDP---
VITVI   -------------------REAESL-------------------MEAST-
SOYBN   -------------------REAESS-------------------SEVAS-
SOLLC   -------------------REVDSL-------------------VEASA-
ORYSJ*  --------------------------------------------LEPYMD
HORVU   -------------------REVDSL-------------------LEPPM-
SORBI   -------------------REMDSL-------------------LEPPL-
SELML   -------------------NKGQWN-------------------AELAS-
PHYPA   -------------------NDFERN-------------------SQATS-
VOLCA   IGGCVGSVFGTATAAAAAAAVAAAAATVAAGAAGDLEMGSGGEGAAAAAA
                 4260      4270      4280      4290      4300
```

```
HUMAN*  GTVDMQSNNELFTT----------------------VLDMLSVLIN----
CHICK   GTVDMQSNNELFTT----------------------VLDMLSVLIN----
XENTR   GTVDMQSNNELFTT----------------------VLDMLSVLIN----
DANRE   GTVDMQSNNELFTT----------------------VLDMLSVLIN----
CIOIN   GMVDVQTNSELFDI----------------------VYDMIAVLLH----
ANOGA   GVIDLSNNSELFTT----------------------TLDMLATLIH----
TETNG   GTVDMQSNNELFTT----------------------VLDMLSVLIN----
DROME   GVVDLSCNRELFTT----------------------VVDMLATLVH----
BRUMA   GVISKERDGYLFDS----------------------CYDMLSTLLV----
CAEEL   GIINPVREKLIYET----------------------CFDMLHHMVL----
YEAST*  PAFHTSGKVELPKK----------------------LLNLPPFQVSSFVK
SCHPO   ------------------------------------LFKTISVLVY----
CANAL   NAISPNQDSILDDQNTNSTGIGKGQKSQIGVTNNNDMLDVAPTVSS----
CANGA   ---DEQSRTKLMHK----------------------LMCLPPFQVSSF--
ASPNI   HNFRPCPGILLQTH----------------------ALDVAASLID----
RHIO9   ------SQERFFEF----------------------ILDFVSILID----
CRYNE   GILDSNSARIMADM----------------------LVYIIDCKYC----
USTMA   GEKTECGGS--FAL----------------------LLDSLSFVLD----
ARATH*  SSMADLSSEVLFDR----------------------LLFVLHGLLS----
VITVI   AASLDLSGESLFDR----------------------LLLVLHGLLS----
SOYBN   AAFVDSSSGSLFDR----------------------LLLVLHGLLS----
SOLLC   TASVVVSLESLFDR----------------------LLLLLHGLLS----
ORYSJ*  DVLLDCPSESLFDR----------------------LLCVLQALLG----
HORVU   DVLLDRPSESLFER----------------------LLCVFHALLG----
SORBI   DVLLDRPGESLFER----------------------LLCVLHALLG----
SELML   MAAAASAGEELFDR----------------------LLSVLHALLS----
PHYPA   AAAAASAGEPLFDR----------------------LLSILHALLS----
VOLCA   AAAAAAAGEALHER----------------------LLHILCALDV----
                 4310      4320      4330      4340      4350
```

MED12

```
HUMAN*  ---------GTLAADMSSISQGSME--ENKR----AYMNLAKKLQKELG-
CHICK   ---------GTLAADMSSISQGSME--ENKR----AYMNLVKKLRKELG-
XENTR   ---------GTLAADMSSISQGSME--ENKR----AYMNLVKKLRKELG-
DANRE   ---------GTLAADMSSISQGSME--ENKR----AYMNLVKKLRKELG-
CIOIN   ---------HTLGGDCANKGEGS------KR----EYINIVKKMKKEMG-
ANOGA   ---------STLVSD----SQTERD--ENKK----LYTNLMKKLRKELG-
TETNG   ---------GTLAADMSSISQGSME--ENKR----AYMNLVKKLRKELG-
DROME   ---------STLVSD-------------DER----HYMNLMKKLKKEIG-
BRUMA   ---------WSITDPMNAAAMNTQD--PDTKFRFPTYSLIVKKLRKELN-
CAEEL   ---------WTLVD---GDSMNQHDRYGSIRVRWPQYAGLMKKIRKEMQ-
YEAST*  ETKLHSGDYG----------------------------------------
SCHPO   --------------------------------------------------
CANAL   -----------------IQVLFDLPEPNASNPFKEFIDDKLVKCSIMLEE
CANGA   --------FP----------------------------------------
ASPNI   ----------------------------------SFPDEARHQCVRFLRE
RHIO9   -----------------------GVFSELPRELKRASLQLLNSMQHELS-
CRYNE   -----------------------------------FFTSLDQEFKAESQA
USTMA   ----------------------------------ELPPTLLSTCLPELET
ARATH*  ---------NHQPKWLKPRPSS---NES-SKDFTLFDRDAAESLQNELSR
VITVI   ---------SCQPSWLKSKSASKSTTES-IKEFSGFDREAAENLQNDLDC
SOYBN   ---------SYPPSWLRAKPVSKTISEP-TREISGIDRELLETLQNDLDR
SOLLC   ---------SHQPRWLKWKSNSKAPSES-SKDYSAFEREGAESLQNDLDR
ORYSJ*  ---------NSQPSWLKTKPSSKPAVKF-LRDLSAIDKEVTKSLQCALDR
HORVU   ---------NCKPSWMKSKPVSKPTVRAPSRDFPAFDNEVAVALQSALDH
SORBI   ---------SYKPSWLKSRSASRSTNRT-QRDFSAFDNEAAEGLQSALDH
SELML   ---------NTWAVWLKPRGRAAKP----PREVPPFEREAAEKLQTELDH
PHYPA   ---------NTWAVWLKPRKGSVKP----LRDIPPFDKEAAERMQVDLEH
VOLCA   -----------------------------------PPYDNVLQLLTDLDQ
                 4360      4370      4380      4390      4400
```

```
HUMAN*  ---------------ERQ-SDSLEKVRQLLPL------------------
CHICK   ---------------DRQ-SDSLEKVRQLLPL------------------
XENTR   ---------------DRQ-SESLEKVRQLLPL------------------
DANRE   ---------------DRQ-SESLEKVRQLLPL------------------
CIOIN   ---------------DKQ-TESIRRLRQLLPI------------------
ANOGA   ---------------DKN-GPSIKYVRQLLPL------------------
TETNG   ---------------DRQ-SESLEKVRQLLPL------------------
DROME   ---------------EKN-NASIRVIRQLLPL------------------
BRUMA   ---------------ERALIPELRSLIQFLPI------------------
CAEEL   ---------------ERFTDQTRNSLHRFLPI------------------
YEAST*  ---------------EEEDADQEESFSLNLGI------------------
SCHPO   --------------------------------------------------
CANAL   EELKYGGDYHIFNNTGLYLKSTKNESVSLPPF------------------
CANGA   ---------------EEDDTDCTRDPALSLGL------------------
ASPNI   RCPPFL-----------KFQNDRRFLYLLGPM------------------
RHIO9   ----------------SVPEMFQGRVKRIMPF------------------
CRYNE   GTSPQARLVCQSALLAETVSPHAQSILSSFPELSAAMP------------
USTMA   QLQT-----------IELATQERSDKLQQLSF------------------
ARATH*  ---------------MQLPDTIRWRIQAAMPILLPSLRCSLSCQPHSVPP
VITVI   ---------------MQLPDTIRWRIQAAMPILVPSGRCSISCQPPSVSS
SOYBN   ---------------MQLPDTIRWHIQAAMPILIPSMRCSLSCQPPSISN
SOLLC   ---------------MQLPETVRWRIQCAMPILFPSARLSISCQPPSVLP
ORYSJ*  ---------------MELPATIRRRIQVAMPILPTSRLSSITCGPPLLSS
HORVU   ---------------VELPGAIRRRIQAAMPILPPARHPSIHCQPPQLSL
SORBI   ---------------MELPETIRRRIQAAMPLLPPSRHPSMQCQPPQLSL
SELML   ---------------MQLPMSLRMRLQAAMPVFPPGPTFSLSAAPPQVPQ
PHYPA   ---------------MQLPCGIRARLQAAFPLLPPTPVTTVSAAPPQVPV
VOLCA   ALAAAAAASGSSSGAAASLAWMRERIISVLPLQGVVPRQLL---------
                 4410      4420      4430      4440      4450
```

```
HUMAN*  -PKQTR-------------------------DVITCEPQGSLIDTKGNKI
CHICK   -PKQTR-------------------------DVITCEPQGSLIDTKGNKI
XENTR   -PKQTR-------------------------DVITCEPQGSLIDTKGNKI
DANRE   -PKQTR-------------------------DVITCEPQGSLIDTKGNKI
CIOIN   -TRPEV-------------------------YVVCCEPSGAHMDIKGNKI
ANOGA   -AKQTC-------------------------EVLTCDSAGSSTDAKGNKI
TETNG   -PKQTR-------------------------DVITCEPQGSLIDTKGNKI
DROME   -YKQPT-------------------------EVIACEHSG--MDTKGNKI
BRUMA   -PKPQF-------------------------ELITCEPYGTL-PTSPQKL
CAEEL   -GKMQM-------------------------STISYKKYQKR-PKVNQKM
YEAST*  -------------------------------GIVEIAHE-----------
SCHPO   --------------------------------------------------
CANAL   --------------------------------VTGLNKTAGDYDSSSARM
CANGA   -------------------------------DLGTASANSTSSTTPSEGT
ASPNI   ------TDTTIPSSQLSASISSPAAGGSTPTPIPSGTLSGGHSSQATQQM
RHIO9   -TAHNI-------------------------YLNNTHLASSILGLQSSDD
CRYNE   -------------------------------YLSPVQRHMSLVTPDTADH
USTMA   -------------------------------RLDNATTANAWLVASSAGA
ARATH*  T------------------------------ALTLVQPSGSTAA------
VITVI   A------------------------------AVASLQPSLSFPAFHPGN-
SOYBN   S------------------------------ALVCLQPSITNPGSNSSS-
SOLLC   A------------------------------ALSSLLPSNPVSVLHSSNG
ORYSJ*  A------------------------------ALSPFQCSTSAAG------
HORVU   A------------------------------ALSPLQSIPSTSG------
SORBI   A------------------------------ALTPLQSSTSGAG------
SELML   S------------------------------AFTALQANLSPSL----HL
PHYPA   S------------------------------VLSQLQVKGFNSTLG----
VOLCA   -------------------------------PLQQRAPAAATASVGTADS
                 4460      4470      4480      4490      4500
```

MED12

```
HUMAN*   AGF-----------DSIFKKEGLQVSTKQKI-------------------
CHICK    AGF-----------DSIFKKEGLQVSTKQKI-------------------
XENTR    AGF-----------DSIFKKEGLQVSTKQKI-------------------
DANRE    AGF-----------E----KEGLQVSTKQKI-------------------
CIOIN    P-------------VPVDGNQGLQVTSKQKL-------------------
ANOGA    A-I-----------DSIEKKNGLRLGDKQRV-------------------
TETNG    AGF-----------E----KEGLQVSTKQKI-------------------
DROME    C--------------DIDKKQ-LRISDKQRI-------------------
BRUMA    GKSQSQGQI--IQTTVKANRHGLQFAEKVKL-------------------
CAEEL    SKKFLAGE------GLKNGKYSFLPEEKAKT-------------------
YEAST*   NEQKWLIYDKKDHKYV----------CTFSM-------------------
SCHPO    --------------------------------------------------
CANAL    IKQ-------------------------------------------PFKM
CANGA    HKKRCAIWDKRLHKYT----------GELQR-------------------
ASPNI    AALTGPFSGLS---------------ENTKLVADRLRIQNGGRINGPYPV
RHIO9    AQQQQQHLEACLEQS-----------------------------------
CRYNE    VSDSALPLDD----------------------------------------
USTMA    ALKKGWFTSGGL--------------------------------------
ARATH*   AGTNQRNSPAISKSGTAA--------AQGKLKPTMLAPHQQQEADNTDVV
VITVI    TNQSQRNSSSLVRP--------------GKLK------NMPLQQDHDIEI
SOYBN    STIPQRNP-VLSRVASNA--------S-GKSK----------QQDNDLEI
SOLLC    SNQTQRNPGSLLRTATSV--------A-GKAK------HVSSQQENDHEV
ORYSJ*   ---PHQQF-PLNWIPTNL--------S-RRCK------AALPSQDPNMEI
HORVU    ---PQQKSTSHSWVPTNI--------S-SRSK------AVLPPQDPEMEV
SORBI    ---PQQKS-SVSWVPTNV--------S-SRSK------AALPSHDPEMEV
SELML    SHSQKLGADLKS--------------------------KPSPSLDTETGM
PHYPA    VSTTYPGSAQKSLYPLEN--------NAGKL-------KAVTSADSEMEV
VOLCA    GSLLAAIPFLEMDPAVLLPLESLSQGGEVSA-------------------
                  4510      4520      4530      4540      4550
```

```
HUMAN*   SPW--DLFE-----------------GL------------KPSA------
CHICK    SPW--DLFE-----------------GL------------KHSA------
XENTR    SPW--DLFE-----------------GI------------KQSA------
DANRE    SPW--DVFE-----------------GL------------KHSA------
CIOIN    NMW--DVIE-----------------GL------------KHSS------
ANOGA    SVW--DLLE-----------------GH------------KNPA------
TETNG    SPW--DVFE-----------------GL------------KHSA------
DROME    SVW--DILE-----------------GH------------KNPA------
BRUMA    SAY--DIIQ-----------------YL------------NIDSTLL---
CAEEL    NAF--EHTD-----------------HL------------G--DLIV---
YEAST*   EPY--HFIS-----------------NYNTKYTDDMATGSNDTTAFN---
SCHPO    -----DLIT-----------------------------------------
CANAL    KSF--EILEDVNNNKNSVNDG-----------------------------
CANGA    KPY--YCIK-----------------NY------------QDTEDIN---
ASPNI    RPW--ELLE-----------------------------DAAPILGVN---
RHIO9    KPW--EWLE--------------------------DYVNETPHDN---
CRYNE    RRW--ELFE----------YIGPSKRKIGPQDLFLASAPLKDAS------
USTMA    NPW--ECAEYLEAPPAANPAAAGAAGAAGAAAGGNARAGVVETGRSGVNS
ARATH*   DPW--TLLEDGTS--SGLSSSNAS--NSSDMANLRATCWLK---------
VITVI    DPW--TLLEDGAG--AGPSSGNTAVIGSGDHANLRASSWLR---------
SOYBN    DPW--TLLEDGTG--SYSSAGNTASIGSGDHANIRATSWLK---------
SOLLC    DPW--ILLEDGAG--SSHSSSNSPLVGGGDHANLKASNWLK---------
ORYSJ*   DPW--TLLEDGTSCPNTNSGSNSANGVTGDHANLKACSFLK---------
HORVU    DPW--TLLEDGTSCPSTSSGSNSASGITPDHSNLKACSWLK---------
SORBI    DPW--NLLEDGTSCPSTTSGSNGASGVTGDHANLKACSWLK---------
SELML    DPW--LLLEDGTGLPATVGSANSS--TTADGSTLKACPWLK---------
PHYPA    DPW--TLLEDGVG-------ASSGPIGSGEVGNLKACAWLK---------
VOLCA    QPYGHTLLPPALRPWARPPASGDAASGGDAVSVLTSEPLLADSSLADLLQ
                  4560      4570      4580      4590      4600
```

```
HUMAN*   --------------------------------------------------
CHICK    --------------------------------------------------
XENTR    --------------------------------------------------
DANRE    --------------------------------------------------
CIOIN    --------------------------------------------------
ANOGA    --------------------------------------------------
TETNG    --------------------------------------------------
DROME    --------------------------------------------------
BRUMA    --------------------------------------------------
CAEEL    --------------------------------------------------
YEAST*   --------------------------------------------------
SCHPO    --------------------------------------------------
CANAL    --------------------------------------------------
CANGA    --------------------------------------------------
ASPNI    --------------------------------------------------
RHIO9    --------------------------------------------------
CRYNE    --------------------------------------------------
USTMA    RAAVESPGAVTVNDAETCNSLPGARTGWTGKLWLNT--------------
ARATH*   --------------------------------------------------
VITVI    --------------------------------------------------
SOYBN    --------------------------------------------------
SOLLC    --------------------------------------------------
ORYSJ*   --------------------------------------------------
HORVU    --------------------------------------------------
SORBI    --------------------------------------------------
SELML    --------------------------------------------------
PHYPA    --------------------------------------------------
VOLCA    HVSQNVKGFRCISTAVLVAALEGVRNLKDLYGLRECRDFMVQHGLTQYAT
                  4610      4620      4630      4640      4650
```

MED12

```
HUMAN*  --------------------------------------------------
CHICK   --------------------------------------------------
XENTR   --------------------------------------------------
DANRE   --------------------------------------------------
CIOIN   --------------------------------------------------
ANOGA   --------------------------------------------------
TETNG   --------------------------------------------------
DROME   --------------------------------------------------
BRUMA   --------------------------------------KR----------
CAEEL   --------------------------------------KG----------
YEAST*  --------------------------------------DS----------
SCHPO   --------------------------------------------------
CANAL   --------------------------------------------------
CANGA   --------------------------------------NC----------
ASPNI   --------------------------------------DT----------
RHIO9   --------------------------------------DA----------
CRYNE   --------------------------------------------------
USTMA   --------------------------------------------------
ARATH*  --------------------------------------------------
VITVI   --------------------------------------------------
SOYBN   --------------------------------------------------
SOLLC   --------------------------------------------------
ORYSJ*  --------------------------------------------------
HORVU   --------------------------------------------------
SORBI   --------------------------------------------------
SELML   --------------------------------------------------
PHYPA   --------------------------------------------------
VOLCA   FWAKSLLPSYAWTIQSLWTDNNREAGVQSDTRTLVSDIKSNMEDLLIRFL
                  4660      4670      4680      4690      4700
```

```
HUMAN*  -------------------------PLSWGWFGTVRVDRRVARGEEQQRL
CHICK   -------------------------PLSWGWFGTVRVDRRVSRFEEQQRL
XENTR   -------------------------PLSWGWFGTVRVDRRVSRMEEQQRL
DANRE   -------------------------PLSWGWFGTIRVDRKVTKFEEQQRL
CIOIN   -------------------------PLCLAWFGARTIERKPMFYEEQHNL
ANOGA   -------------------------PLSWAWFGAVKIERKPLAYEETHRL
TETNG   -------------------------PLSWGWFGTIRVDRKVNKFEEQQRF
DROME   -------------------------PLSWVWFGAVKLERKPLTYEEAHRN
BRUMA   -------------------------PWNWSMLQAVKLDRLPVPVQRYIQR
CAEEL   -------------------------GWKFRMFQTTRLDKVAKNVQNVLRS
YEAST*  -------------------------CVNLSLFDARFERKNPH--------
SCHPO   --------------------------------------------------
CANAL   -------------------------CINLRLFDAYTTKENPR--------
CANGA   -------------------------SLNLSLFDARYERNNPR--------
ASPNI   -------------------------AVSLKLFDARRVRA-----------
RHIO9   -------------------------PISLTIFNALKPKSVDSTYIKWFKY
CRYNE   -------------------------SIPITLFDPKITRDAPPN-------
USTMA   -------------------------AIPLSMLGVRVTRDLVPNYASPASP
ARATH*  --------------------------------GAVRVRRTDLTYVGSVDD
VITVI   --------------------------------GTVRVRRTDLTYIGAVDD
SOYBN   --------------------------------GAVRVRRTDLTYVGAVDD
SOLLC   --------------------------------GTVRVRRTDLTYIGAVDD
ORYSJ*  --------------------------------DSVRVRRTDLTYIGSLDE
HORVU   --------------------------------GAVRVRRTELTYIGSLDD
SORBI   --------------------------------GAVRVRRTELTYIGSLDD
SELML   --------------------------------GAVRVRRTELTHLGSVDS
PHYPA   --------------------------------GAVRVRRTDLSYIGAVDE
VOLCA   PSKPYYHQLDDLKTDSWGRLVAAVVRTQVPIYLARELESFRRYLLETVNF
                  4710      4720      4730      4740      4750
```

```
HUMAN*  LLYHTHLRPRPR--------------------------------------
CHICK   LLYHTHLKPKPR--------------------------------------
XENTR   LLYHTHLKPKPR--------------------------------------
DANRE   LLYHTHIKPKQR--------------------------------------
CIOIN   MLHHQHSRKRDL--------------------------------------
ANOGA   LKYHTHSLFKPS--------------------------------------
TETNG   LLYHTHLKPKPR--------------------------------------
DROME   LKYHTHSLVKPS--------------------------------------
BRUMA   LVHHSHYNEFVRPTICGM--------------------------------
CAEEL   NMHHTHVLEFNRPQLLMS--------------------------------
YEAST*  --------------------------------------------------
SCHPO   --------------------------------------------------
CANAL   --------------------------------------------------
CANGA   --------------------------------------------------
ASPNI   --------------------------------------------------
RHIO9   GFDTGNTSQPTQ--------------------------------------
CRYNE   --------------------------------------------------
USTMA   GGVSVGSNDASS--------------------------------------
ARATH*  DS------------------------------------------------
VITVI   DS------------------------------------------------
SOYBN   DG------------------------------------------------
SOLLC   DS------------------------------------------------
ORYSJ*  DS------------------------------------------------
HORVU   DS------------------------------------------------
SORBI   DS------------------------------------------------
SELML   DT------------------------------------------------
PHYPA   EP------------------------------------------------
VOLCA   PQPAVHVGEATRCLLLSGRMAHLGLSQLDTCGAIADSHMLEHACALRLTL
                  4760      4770      4780      4790      4800
```

MED12

```
HUMAN*  --------------------------------------------------
CHICK   --------------------------------------------------
XENTR   --------------------------------------------------
DANRE   --------------------------------------------------
CIOIN   --------------------------------------------------
ANOGA   --------------------------------------------------
TETNG   --------------------------------------------------
DROME   --------------------------------------------------
BRUMA   --------------------------------------------------
CAEEL   --------------------------------------------------
YEAST*  --------------------------------------------------
SCHPO   --------------------------------------------------
CANAL   --------------------------------------------------
CANGA   --------------------------------------------------
ASPNI   --------------------------------------------------
RHIO9   --------------------------------------------------
CRYNE   --------------------------------------------------
USTMA   --------------------------------------------------
ARATH*  --------------------------------------------------
VITVI   --------------------------------------------------
SOYBN   --------------------------------------------------
SOLLC   --------------------------------------------------
ORYSJ*  --------------------------------------------------
HORVU   --------------------------------------------------
SORBI   --------------------------------------------------
SELML   --------------------------------------------------
PHYPA   --------------------------------------------------
VOLCA   AERPWNWDSTETDSCLFGLLKRAAHGLPWRWLQGVPSCSATIVLFRAPHI
                 4810      4820      4830      4840      4850
```

```
HUMAN*  --------------------------------------------------
CHICK   --------------------------------------------------
XENTR   --------------------------------------------------
DANRE   --------------------------------------------------
CIOIN   --------------------------------------------------
ANOGA   --------------------------------------------------
TETNG   --------------------------------------------------
DROME   --------------------------------------------------
BRUMA   --------------------------------------------------
CAEEL   --------------------------------------------------
YEAST*  --------------------------------------------------
SCHPO   --------------------------------------------------
CANAL   --------------------------------------------------
CANGA   --------------------------------------------------
ASPNI   --------------------------------------------------
RHIO9   --------------------------------------------------
CRYNE   --------------------------------------------------
USTMA   --------------------------------------------------
ARATH*  --------------------------------------------------
VITVI   --------------------------------------------------
SOYBN   --------------------------------------------------
SOLLC   --------------------------------------------------
ORYSJ*  --------------------------------------------------
HORVU   --------------------------------------------------
SORBI   --------------------------------------------------
SELML   --------------------------------------------------
PHYPA   --------------------------------------------------
VOLCA   FSGQCMPPDMSPAERRVCGQIWRLLSHATVQCYLGWWLAASTMVLRPGGV
                 4860      4870      4880      4890      4900
```

```
HUMAN*  --------------------------------------------------
CHICK   --------------------------------------------------
XENTR   --------------------------------------------------
DANRE   --------------------------------------------------
CIOIN   --------------------------------------------------
ANOGA   --------------------------------------------------
TETNG   --------------------------------------------------
DROME   --------------------------------------------------
BRUMA   --------------------------------------------------
CAEEL   --------------------------------------------------
YEAST*  --------------------------------------------------
SCHPO   --------------------------------------------------
CANAL   --------------------------------------------------
CANGA   --------------------------------------------------
ASPNI   --------------------------------------------------
RHIO9   --------------------------------------------------
CRYNE   -------------------------------------------------A
USTMA   --------QPWTTGGGASAIGVGTCSAMDMDAKEALAALPMFVESERTYG
ARATH*  --------------------------------------------------
VITVI   --------------------------------------------------
SOYBN   --------------------------------------------------
SOLLC   --------------------------------------------------
ORYSJ*  --------------------------------------------------
HORVU   --------------------------------------------------
SORBI   --------------------------------------------------
SELML   --------------------------------------------------
PHYPA   --------------------------------------------------
VOLCA   ISGFSLPDNLLVTCRACWQRRGEREEGELRGLQAPLYAAAVSWMYGMLTR
                 4910      4920      4930      4940      4950
```

MED12

```
HUMAN*  ---------------AYYLEPLPLPPEDEE-----P-PAPTLLEPEKKAP
CHICK   ---------------SYYLEPLPLPPEEEE-----P-PTPVALEPEKKAV
XENTR   ---------------SYYLENLPLPPEDEE-----P-PTPTSTEPDKKLS
DANRE   ---------------SYYLAPLNLPQEEEE-----P-LTPVPPEPDKKL-
CIOIN   ---------------IDFLSPPQLPPEEEEEAELIPVPTK--IPDHKGSV
ANOGA   ---------------SYYYEPLPLPPEEVD-----PLPDK--LKDDMKAD
TETNG   ---------------SYYLEPLPLPPEEEE-----P-LTPMSQEPEKKMM
DROME   ---------------SYYYEPLPLPPEDIE-----PVPEKICIKDEMKAD
BRUMA   ---------DRPPNLDIYLSP---PMMDVSE-----TPATLHHVSSSAAT
CAEEL   ---------DNMFD-DIFLAP---PDIEITKIIEQPVPVIDEEEAKKRAE
YEAST*  --------------------------------------------------
SCHPO   --------------------------------------------------
CANAL   --------------------------------------------------
CANGA   --------------------------------------------------
ASPNI   --------------------------------------------------
RHIO9   --------------------------------------------------
CRYNE   GTMDAYGKPAEDGELSPTTDPNPASPVEEEPRRSWETFASERNLGDGLAG
USTMA   ERSAGEPAYSRDLRRGLLVEPNLPCRTRDERGETSQEPARATMDPAGAHT
ARATH*  --------------------------------------------------
VITVI   --------------------------------------------------
SOYBN   --------------------------------------------------
SOLLC   --------------------------------------------------
ORYSJ*  --------------------------------------------------
HORVU   --------------------------------------------------
SORBI   --------------------------------------------------
SELML   --------------------------------------------------
PHYPA   --------------------------------------------------
VOLCA   QCRGSSAAVAGIAASGRTPPPPSLPPPAGSACRPTNTAAAAAAESATSVV
                 4960      4970      4980      4990      5000
```

```
HUMAN*  EPPKT--------------DKPGAAP-PSTE----ERKKKST--KGKKRS
CHICK   EPSKA--------------DKTSSNPATSTE----ERKKKQS--KTKKRN
XENTR   EALKV--------------DKSGVGGTTNTD----DRKKPSKPKREKKRN
DANRE   DTAKV--------------EKS-VSNVPTKK----KKKKPASTVKQEDYK
CIOIN   LKNPF--------------GAS----SASTE----GMKQPPPKKKKKSRV
ANOGA   TPSS---------------DQS---PAPSTG----SKKSKTTTRKRKTKA
TETNG   EAVKP--------------EKS-VPGVPSDS----NKKKPSKKKKNSSNK
DROME   TPSSV--------------DQS---PSAVVGGTGRGRGKGTTTRKRKPKN
BRUMA   STTTSA-------------SSTNVPVSSGGAAMDVGATLGPVVQG-RAMS
CAEEL   EEKEAAEKKEESKNAEDEKNKNNAENKKDTKEGEKGKSKDKEKDGEKEKC
YEAST*  --------------------------------------------------
SCHPO   --------------------------------------------------
CANAL   --------------------------------------------------
CANGA   --------------------------------------------------
ASPNI   --------------------------------------------------
RHIO9   --------------------------------------------------
CRYNE   EPAYAKQAATLLFSVRDNDVLEVAPVKSAPELVITSSTSPQKPKVKRSGS
USTMA   SVTSSATHDPSHVIDLTWDADEEMPLSTHPHHPAVATTSRKRRLSKRD--
ARATH*  --------------------------------------------------
VITVI   --------------------------------------------------
SOYBN   --------------------------------------------------
SOLLC   --------------------------------------------------
ORYSJ*  --------------------------------------------------
HORVU   --------------------------------------------------
SORBI   --------------------------------------------------
SELML   --------------------------------------------------
PHYPA   --------------------------------------------------
VOLCA   TPTPTAGAAAVAYVSPAAVHETAAAAPSTPPLPVAPVPTTAAAAPTAARE
                 5010      5020      5030      5040      5050
```

```
HUMAN*  QPATKT--------------------------------------------
CHICK   QSASKS--------------------------------------------
XENTR   MAPSKV--------------------------------------------
DANRE   PHNTVM--------------------------------------------
CIOIN   RTSSEQ---NLPVPYRQTFSVAPNPVPAG---------------------
ANOGA   TAAAQV---QQSQPQQPIMPAIVTMTPQQ---------------------
TETNG   TEDGVN--------------------------------------------
DROME   PKTPPVVNTQQQQPQLAQQPQQPQNVQQQ---------------------
BRUMA   YQSSSTPTCTSIPD-----GSTPSSTPGAPSTAATAMIFPGQSGPMPALP
CAEEL   KDASKKDDVTSEKNELEKRASDAAAATNAPETNKDMDTSTPKPAPVTRSP
YEAST*  --------------------------------------------------
SCHPO   --------------------------------------------------
CANAL   --------------------------------------------------
CANGA   --------------------------------------------------
ASPNI   --------------------------------------------------
RHIO9   --------------------------------------------------
CRYNE   GKDKATGSNKDAPAAAEEDENDEDSGAEVPLSKK----------------
USTMA   --------------------------------------------------
ARATH*  --------------------------------------------------
VITVI   --------------------------------------------------
SOYBN   --------------------------------------------------
SOLLC   --------------------------------------------------
ORYSJ*  --------------------------------------------------
HORVU   --------------------------------------------------
SORBI   --------------------------------------------------
SELML   --------------------------------------------------
PHYPA   --------------------------------------------------
VOLCA   PASTGGEDGSQLPYSAQNMHDIILPVSEH---------------------
                 5060      5070      5080      5090      5100
```

MED12

```
HUMAN*  --------------------------------------------------
CHICK   --------------------------------------------------
XENTR   --------------------------------------------------
DANRE   --------------------------------------------------
CIOIN   --------------------------------------------------
ANOGA   --------------------------------------------------
TETNG   --------------------------------------------------
DROME   --------------------------------------------------
BRUMA   DLTGRGQNNASPRGARGGRRKAAGLSTR----------------------
CAEEL   ATRGRGGGRKRNSGARGGGPRAKRANSRADTAQAAAATTQWNAPIANTSN
YEAST*  --------------------------------------------------
SCHPO   --------------------------------------------------
CANAL   --------------------------------------------------
CANGA   --------------------------------------------------
ASPNI   --------------------------------------------------
RHIO9   --------------------------------------------------
CRYNE   --------------------------------------------------
USTMA   --------------------------------------------------
ARATH*  --------------------------------------------------
VITVI   --------------------------------------------------
SOYBN   --------------------------------------------------
SOLLC   --------------------------------------------------
ORYSJ*  --------------------------------------------------
HORVU   --------------------------------------------------
SORBI   --------------------------------------------------
SELML   --------------------------------------------------
PHYPA   --------------------------------------------------
VOLCA   -------------------------CVDFVAAVGSLGSLFADTLAAAALS
                 5110      5120      5130      5140      5150
```

```
HUMAN*  -----------------------------------EDYGMG-PGRSGPYG
CHICK   -----------------------------------EDFVLG-PSRGVSYG
XENTR   -----------------------------------EEFPLG-PGRPGPYG
DANRE   -----------------------------------------------QYA
CIOIN   ------------------SLLNQ------MGTTSTQVNSFQHPNNFPNNP
ANOGA   ------------------TL-----------------QQMQ-NNSMGGMQ
TETNG   -----------------------------------------RAPGGVSYG
DROME   ------------------QLQQQQQQQQHMQQQHMQQQQMQ-PNQMGQMP
BRUMA   ------------------SQGTTRKQR--------QQRQQT-VDQLAVAA
CAEEL   PAAGGNFHAAMRGNQPPMSNGSSDETKVHIRNLLNRKKEEK-RNSLADAS
YEAST*  --------------------------------------------------
SCHPO   --------------------------------------------------
CANAL   --------------------------------------------------
CANGA   --------------------------------------------------
ASPNI   --------------------------------------------------
RHIO9   --------------------------------------------------
CRYNE   --------------------------------------------------
USTMA   --------------------------------------------------
ARATH*  --------------------------------------------------
VITVI   --------------------------------------------------
SOYBN   --------------------------------------------------
SOLLC   --------------------------------------------------
ORYSJ*  --------------------------------------------------
HORVU   --------------------------------------------------
SORBI   --------------------------------------------------
SELML   --------------------------------------------------
PHYPA   --------------------------------------------------
VOLCA   LSLSLQFMAPDAAEARLRSCWQLLGRILAMDLGSARSRSVRDLAQAAGRV
                 5160      5170      5180      5190      5200
```

```
HUMAN*  VTVPPDLLH----------------------HPNPGSIT-HLNY--RQGS
CHICK   VGMPTDLLH----------------------HQAGSTMS-RLAY--GQSP
XENTR   M-VPTDLLH----------------------TQAAG-MS-RLPY----AT
DANRE   PGMTPDLPL----------------------SMGQTNMSYRMGY---QAP
CIOIN   PNYP-NYQQTNQQITSTYGANNFPPSAQQAQAQSTHFKD-RTRQALQLKL
ANOGA   MGGPM----------------------------SSMNQQ-HMQQ--QYGQ
TETNG   TNMPPELMQ----------------------NHPYG----RLPY--GQQA
DROME   MNMPMNMQQ-------------FAPNPNNMMQQNAMLQQ-QQQQ--QMQQ
BRUMA   AAAAAAAQQQQQQMGSAVGCGGGPPQQPTYPSTWATGPQ-QTGVPQQFAS
CAEEL   AAAAAANSNAMGNTSSM------PPSGPPMP--MGSSMQ-SAGATQQLQG
YEAST*  --------------------------------------------------
SCHPO   --------------------------------------------------
CANAL   --------------------------------------------------
CANGA   --------------------------------------------------
ASPNI   --------------------------------------------------
RHIO9   --------------------------------------------------
CRYNE   --------------------------------------------------
USTMA   --------------------------------------------------
ARATH*  --------------------------------------------------
VITVI   --------------------------------------------------
SOYBN   --------------------------------------------------
SOLLC   --------------------------------------------------
ORYSJ*  --------------------------------------------------
HORVU   --------------------------------------------------
SORBI   --------------------------------------------------
SELML   --------------------------------------------------
PHYPA   --------------------------------------------------
VOLCA   LRLQLPNHLGRPESATMAAGATALPAAPLRCKTWSSVGGTGCGSRGAAPS
                 5210      5220      5230      5240      5250
```

MED12

```
HUMAN*   IGLYTQNQPLPAGGPRVD-PYRPVRLP-MQKL-PTRPTYPGVLP----TT
CHICK    VGLYAQNQPLPAGGPRLDTSYRPVRMP-LGKLVQSRPPYSGVLP----SG
XENTR    QNMYAQNQPLPPGGPRLDNSYRPTRMA-MVKV-PARPQYVSGVP---QAG
DANRE    MNMYAQNQPLPPGGPGLEPPFRP-RVP-MNKM-PPRPNYT--MPNMQTAG
CIOIN    LSRHPAQGKTNTGHMGT-------------------------------QG
ANOGA    ANPAQLVGQGNMGG------------------MQTGMGGLGAGM----GG
TETNG    MNMYTQNQPLPPGGPGLEPPYRPARNPQMNKMMPVRPNYTSMMSGMQ-GN
DROME    MGNNPMQQQLNVGGGNGQPNP-------QMNFMQQGPGGGGAGP----QG
BRUMA    GPSAPPSGTGQAGTAMGTQTTEDSKMKIHSMILQKRQAQAASSA----TA
CAEEL    MQKH------QMGGSMSGMNQNMGGMN------QSMSHQAPPPY----SS
YEAST*   --------------------------------------------------
SCHPO    --------------------------------------------------
CANAL    --------------------------------------------------
CANGA    --------------------------------------------------
ASPNI    --------------------------------------------------
RHIO9    --------------------------------------------------
CRYNE    --------------------------------------------------
USTMA    --------------------------------------------------
ARATH*   --------------------------------------------------
VITVI    --------------------------------------------------
SOYBN    --------------------------------------------------
SOLLC    --------------------------------------------------
ORYSJ*   --------------------------------------------------
HORVU    --------------------------------------------------
SORBI    --------------------------------------------------
SELML    --------------------------------------------------
PHYPA    --------------------------------------------------
VOLCA    SRGTTGDGGGSCGDLHLEGAGSSGSSDAAGPSIRLNAAYDLASACIAVDL
                      5260      5270      5280      5290      5300
```

```
HUMAN*   MTGV--------MGLEPSSYKTSVYRQQQPAVPQ---GQRLRQQLQQ---
CHICK    MGSM--------MGIDP-SYKPAVYR-QQPPVSQ---GQILRQQLQAKLQ
XENTR    MMD---------------SYKPVMYR-PQPPIPQ---GQLLRQQLQAKL-
DANRE    MGNL--------MGME--KQYQMNYK-PMPNMAQ---GQMLRHQLQGH--
CIOIN    YSDI-----------------------------S---KSVIRQRLEGHVR
ANOGA    V--------FCPLYVNIFDLFHASF------IAV---NRFDRPQLNTN--
TETNG    MPGI--------MGLD--KQYPMGFK-PQPNMPQ---GQ-IRQQLQVQ--
DROME    MPGQQQQWHNAPQQQQPPQPYHNQYAPHQQNMQS---NRIERPPLNAN--
BRUMA    TQQ------GVTGGPQPSMQDVNAYQISAGP-PQYGGNMIMKV---ETGA
CAEEL    TNEMNRPLMNQYGGPHFAAPNPGPLNRSSGPVSSETRQQIMEQQMREKLA
YEAST*   --------------------------------------------------
SCHPO    --------------------------------------------------
CANAL    --------------------------------------------------
CANGA    --------------------------------------------------
ASPNI    --------------------------------------------------
RHIO9    --------------------------------------------------
CRYNE    --------------------------------------------------
USTMA    --------------------------------------------------
ARATH*   --------------------------------------------------
VITVI    --------------------------------------------------
SOYBN    --------------------------------------------------
SOLLC    --------------------------------------------------
ORYSJ*   --------------------------------------------------
HORVU    --------------------------------------------------
SORBI    --------------------------------------------------
SELML    --------------------------------------------------
PHYPA    --------------------------------------------------
VOLCA    ALRRPGCWPALLAHGPPLQMASLILTMRKVAVAADEMELLLPQAGATAAA
                      5310      5320      5330      5340      5350
```

```
HUMAN*   SQGMLGQSSVH-----------------QMTPSSSYG-LQTS----QGYT
CHICK    GQGIMGQQTVR-----------------QMAPTPSYGALQPS----QGYT
XENTR    AQGMMPQ-PVR-----------------QMSQTPSYGSMQP-----QGYT
DANRE    -NHLIGQ-QIR-----------------QVTPNPQYSSMQPAQNIPQGYT
CIOIN    TSSHVPSHDMY-----------------SNQPQSRQVGLQQHMIN-QGSV
ANOGA    SKQALSN-MLR-----------------SKLPI--NAG--------IGTA
TETNG    NQSLIGQ-QIR-----------------QMTPNQPYTPMQAS----QGYT
DROME    SKQALSQ-MLR-----------------QRQPFQQQAQ--------QGPG
BRUMA    NSMQMVDHTQQ---------------QQQQRQQQQYAQQQQLIQMQQDVQ
CAEEL    AHHQLVEQQKQRDAREREAREREAREHQERMQREAYMKEQQLLERKRAIE
YEAST*   --------------------------------------------------
SCHPO    --------------------------------------------------
CANAL    --------------------------------------------------
CANGA    --------------------------------------------------
ASPNI    --------------------------------------------------
RHIO9    --------------------------------------------------
CRYNE    ----------------------------QKTAKNNNAAAKAPARKTTGGK
USTMA    --------------------------------------------------
ARATH*   --------------------------------------------------
VITVI    --------------------------------------------------
SOYBN    --------------------------------------------------
SOLLC    --------------------------------------------------
ORYSJ*   --------------------------------------------------
HORVU    --------------------------------------------------
SORBI    --------------------------------------------------
SELML    --------------------------------------------------
PHYPA    --------------------------------------------------
VOLCA    GTGVVTAGDPRVIWRLQLLALLEQCLPFTLTPGVAAAAVSGAAAAATAAA
                      5360      5370      5380      5390      5400
```

MED12

```
HUMAN*  PYVSHVGLQQHTG-------------------------------------
CHICK   PYVSHIGLQQHPS-------------------------------------
XENTR   SYGSHMTLQQHPPPS-----------------------------------
DANRE   SYGSHMGMQPHPS-------------------------------------
CIOIN   GYNSYNTPTPAMQQN-----------------------------------
ANOGA   GGGAVMGAQGMGAGG-----------------------------------
TETNG   TFGSHMGMQQHLS-------------------------------------
DROME   GGFNPMQQQPQASQQ-----------------------------------
BRUMA   GGSQYVVHQQQRGGI--AYGQSVQQATYDSRFNQQQL-------------
CAEEL   ENNRIMEEQQREREMEAARKEAARRAAEEAYAAEQQRLELLRRQEEERLR
YEAST*  --------------------------------------------------
SCHPO   --------------------------------------------------
CANAL   --------------------------------------------------
CANGA   --------------------------------------------------
ASPNI   --------------------------------------------------
RHIO9   --------------------------------------------------
CRYNE   GVSKKATGKSAKEGATGKAKGGRRKSQAE---------------------
USTMA   --------------------------------------------------
ARATH*  --------------------------------------------------
VITVI   --------------------------------------------------
SOYBN   --------------------------------------------------
SOLLC   --------------------------------------------------
ORYSJ*  --------------------------------------------------
HORVU   --------------------------------------------------
SORBI   --------------------------------------------------
SELML   --------------------------------------------------
PHYPA   --------------------------------------------------
VOLCA   ASAAAAVRGERSPRG-----------------------------------
                 5410      5420      5430      5440      5450
```

```
HUMAN*  --------------------------------------------------
CHICK   --------------------------------------------------
XENTR   --------------------------------------------------
DANRE   --------------------------------------------------
CIOIN   --------------------------------------------------
ANOGA   --------------------------------------------------
TETNG   --------------------------------------------------
DROME   --------------------------------------------------
BRUMA   --------------------------------------------------
CAEEL   KEAEERMRIQRENEERVRQEQMRLEAEERERIRRAEEERIQKELEDKVRR
YEAST*  --------------------------------------------------
SCHPO   --------------------------------------------------
CANAL   --------------------------------------------------
CANGA   --------------------------------------------------
ASPNI   --------------------------------------------------
RHIO9   --------------------------------------------------
CRYNE   --------------------------------------------------
USTMA   --------------------------------------------------
ARATH*  --------------------------------------------------
VITVI   --------------------------------------------------
SOYBN   --------------------------------------------------
SOLLC   --------------------------------------------------
ORYSJ*  --------------------------------------------------
HORVU   --------------------------------------------------
SORBI   --------------------------------------------------
SELML   --------------------------------------------------
PHYPA   --------------------------------------------------
VOLCA   --------------------------------------------------
                 5460      5470      5480      5490      5500
```

```
HUMAN*  ------------------------------------------------PA
CHICK   ------------------------------------------------QS
XENTR   ------------------------------------------------QA
DANRE   ------------------------------------------------QT
CIOIN   ------------------------------------------------QH
ANOGA   ------------------------------------------------MM
TETNG   ------------------------------------------------QG
DROME   ------------------------------------------------QP
BRUMA   ------------------------------------------------QY
CAEEL   EKEEAARQEKERQEQEARMREAREAELSRQRMEQQRRSQQNPYMNQQGQY
YEAST*  --------------------------------------------------
SCHPO   --------------------------------------------------
CANAL   --------------------------------------------------
CANGA   --------------------------------------------------
ASPNI   --------------------------------------------------
RHIO9   --------------------------------------------------
CRYNE   --------------------------------------------------
USTMA   --------------------------------------------------
ARATH*  --------------------------------------------------
VITVI   --------------------------------------------------
SOYBN   --------------------------------------------------
SOLLC   --------------------------------------------------
ORYSJ*  --------------------------------------------------
HORVU   --------------------------------------------------
SORBI   --------------------------------------------------
SELML   --------------------------------------------------
PHYPA   --------------------------------------------------
VOLCA   ------------RTSPLAFSVSQPSSLGAVHMAPVPDPPGPHLHVNHGLC
                 5510      5520      5530      5540      5550
```

MED12

```
HUMAN*  GTMVPPSYSSQPYQSTHPSTN-----PTLVDPTRH--LQQRPSGYVHQQ-
CHICK   GTMVPPTYSGQPYQNSHPSSN-----PALVDPVRQ--MQQRPSGYVHQQ-
XENTR   GALVPPPYSTQQYPGSLPPSG-----GALLDPVRQ--MQQRPSGYVHQQ-
DANRE   PGMVPNSYGNQGFQTGHPATN-----PTMVDSIRQ--MQQRP-GYVHQQ-
CIOIN   GIMQ--GYNNSGYRNTNPPNN-----QIGYQQSTNTSAQSNQQFMNQQQN
ANOGA   GAQQTAQGQQGAGGIMN-QST-----PAMMGTTGGSIMSQSGGSMIQTGM
TETNG   GGMAPSSYGSQNFQGTHPANN-----PAVVDPHRQ--LQQRPSGYVHQQ-
DROME   GPQQQMNPNQMRQQQMNPQQN-----PQSVAAFNA--MQQQPQQNAQQQQ
BRUMA   GQQP----------------------------------------------
CAEEL   SQQPPPSYQQSSYPNNYQPGQQGNQPPNYQQPSHQSMQQGHQAGYQQTSN
YEAST*  --------------------------------------------------
SCHPO   --------------------------------------------------
CANAL   --------------------------------------------------
CANGA   --------------------------------------------------
ASPNI   --------------------------------------------------
RHIO9   --------------------------------------------------
CRYNE   --------------------------------------------------
USTMA   --------------------------------------------------
ARATH*  --------------------------------------------------
VITVI   --------------------------------------------------
SOYBN   --------------------------------------------------
SOLLC   --------------------------------------------------
ORYSJ*  --------------------------------------------------
HORVU   --------------------------------------------------
SORBI   --------------------------------------------------
SELML   --------------------------------------------------
PHYPA   --------------------------------------------------
VOLCA   GVRDPWGSCGGGGGGAAEADDDGDDLHAGPGWLEVLGFGACPAIGSPACS
                  5560      5570      5580      5590      5600

HUMAN*  ----------------------AP-TYGHGLTST----------------
CHICK   ----------------------AP-GYGHTLGNT----------------
XENTR   ----------------------AS-GFSHSASAG----------------
DANRE   ----------------------APAAYGHTIQST----------------
CIOIN   ANTAYG----------------VS-GIHPNSQQT----------------
ANOGA   MNPQQNPALVGQGMGGSGGLGGAS-GMAGNSGMG----------------
TETNG   ----------------------AP-GYPHNMQNT----------------
DROME   MNPNQQ-----QQQQFMRGGNMRP-GMAPNQMNQ----------------
BRUMA   --------------------------------------------------
CAEEL   QMQMNMQQQQNRQQGGPQQSFSGPGGINQPSQPGYSGYNQQGGQGQQGQM
YEAST*  --------------------------------------------------
SCHPO   --------------------------------------------------
CANAL   --------------------------------------------------
CANGA   --------------------------------------------------
ASPNI   --------------------------------------------------
RHIO9   --------------------------------------------------
CRYNE   --------------------------------------------------
USTMA   --------------------------------------------------
ARATH*  --------------------------------------------------
VITVI   --------------------------------------------------
SOYBN   --------------------------------------------------
SOLLC   --------------------------------------------------
ORYSJ*  --------------------------------------------------
HORVU   --------------------------------------------------
SORBI   --------------------------------------------------
SELML   --------------------------------------------------
PHYPA   --------------------------------------------------
VOLCA   QQERMLVLAASSWLPLLLTALEEDGGAQGASEIHRARCKDLRLVPVHIAV
                  5610      5620      5630      5640      5650

HUMAN*  --------------------------------------------------
CHICK   --------------------------------------------------
XENTR   --------------------------------------------------
DANRE   --------------------------------------------------
CIOIN   --------------------------------------------------
ANOGA   --------------------------------------------------
TETNG   --------------------------------------------------
DROME   --------------------------------------------------
BRUMA   --------------------------------------------------
CAEEL   QQQRNPFGNQQDMQQPGAAKLMHAKPNEAHAQQYQHTQNQLSLAQKEKEK
YEAST*  --------------------------------------------------
SCHPO   --------------------------------------------------
CANAL   --------------------------------------------------
CANGA   --------------------------------------------------
ASPNI   --------------------------------------------------
RHIO9   --------------------------------------------------
CRYNE   --------------------------------------------------
USTMA   --------------------------------------------------
ARATH*  --------------------------------------------------
VITVI   --------------------------------------------------
SOYBN   --------------------------------------------------
SOLLC   --------------------------------------------------
ORYSJ*  --------------------------------------------------
HORVU   --------------------------------------------------
SORBI   --------------------------------------------------
SELML   --------------------------------------------------
PHYPA   --------------------------------------------------
VOLCA   LAVHGGHRVAWQWLLLLARQCLAAVGTAAHAGWMRLLRRVLVVDQITAVL
                  5660      5670      5680      5690      5700
```

MED12

```
HUMAN*  ---QRFSHQTLQQTPMI---------------------------------
CHICK   ---QRFPHQSIQQAPMM---------------------------------
XENTR   ---QRFPHQQITQTGMM---------------------------------
DANRE   ---QRFPHQSMQQTPMMHG-------------------------------
CIOIN   ---AYSNPQQVSSGMGM----------------------------IGMGG
ANOGA   ---ASAGGMGGTSGGGMVTSGGMVTGGMGSAGIGNTAPMGAGGQGGGMGG
TETNG   ---QRFVFHKEGCRFVCRC-------------------------------
DROME   ---MNMGGQGMSQNPMMQQQIPQNMVGMVNPNANQMMQSGGAQGGNGVGV
BRUMA   --------------------------------------------------
CAEEL   QYFQAKNLQASQANAQQQQQRFGDVVAGNVAGYGRPYGQQQLGASDQMGT
YEAST*  --------------------------------------------------
SCHPO   --------------------------------------------------
CANAL   --------------------------------------------------
CANGA   --------------------------------------------------
ASPNI   --------------------------------------------------
RHIO9   --------------------------------------------------
CRYNE   --------------------------------------------------
USTMA   --------------------------------------------------
ARATH*  --------------------------------------------------
VITVI   --------------------------------------------------
SOYBN   --------------------------------------------------
SOLLC   --------------------------------------------------
ORYSJ*  --------------------------------------------------
HORVU   --------------------------------------------------
SORBI   --------------------------------------------------
SELML   --------------------------------------------------
PHYPA   --------------------------------------------------
VOLCA   RLYCAWVRRCQEVMTTDAAVILSASGGAAGVAVATSAPAAASAASGDQHT
                 5710      5720      5730      5740      5750
```

```
HUMAN*  --------------------STMTPMSAQGVQAGVRSTAILPEQQQQQQQ
CHICK   --------------------TGMNHLGPQGVPSGIRPSQILPELQRCIEL
XENTR   --------------------PSMGHLTTQPVTGAIRP-QLLPERQQQILR
DANRE   --------------------LAQSHLGVQGIHPNMRPNQMMEQQQQQQQQ
CIOIN   QAGQPNQYGMTSMQRGTSQPTHMARMNQQQVHMG------GSNVHQMNTG
ANOGA   NAGMPNAAGMGNPAMGNPG-MGMQNMQQSGNMQGGGMFQ-GQNVPYQNVN
TETNG   --------------------LSMKDI-IHQVSPSAHPTE-----------
DROME   GVGV-GVGGAGN----NPN-MGMGGMPQQGMIQQQPQQQPQQQVQFQNFQ
BRUMA   --------------------------------------------------
CAEEL   SQLPGASTSRMNQGSSNPQGGMQSYQQQQPVLGQPGPIQTGQSTQQQIPA
YEAST*  --------------------------------------------------
SCHPO   --------------------------------------------------
CANAL   --------------------------------------------------
CANGA   --------------------------------------------------
ASPNI   --------------------------------------------------
RHIO9   --------------------------------------------------
CRYNE   --------------------------------------------------
USTMA   --------------------------------------------------
ARATH*  --------------------------------------------------
VITVI   --------------------------------------------------
SOYBN   --------------------------------------------------
SOLLC   --------------------------------------------------
ORYSJ*  --------------------------------------------------
HORVU   --------------------------------------------------
SORBI   --------------------------------------------------
SELML   --------------------------------------------------
PHYPA   --------------------------------------------------
VOLCA   PISSTTGDGSENNAGIDPAFYDLHQAALRVVEVLPTVLDVLETLVAATPD
                 5760      5770      5780      5790      5800
```

```
HUMAN*  QQQQQQQQQQQQQQ------------------------------------
CHICK   KEKHKNRKLEGIWL------------------------------------
XENTR   VRAPPQPPPQQVPT--------------------------VPQPQ-----
DANRE   QQQQQQQQQQVHQQQ------------------QHIRQGPVFRPQ-----
CIOIN   QNMQGTMNQR----------------------------------------
ANOGA   QNYPNYGNQGMGQQSG-----------------QGGGMMGNFNPM-----
TETNG   ---PHHARSGSHGR--------------------------TGRPS-----
DROME   NQYQQQQQQGMQQQGGGAGVGVGVGMAPNQQQQQQANMMGNFNPQ-----
BRUMA   --------------------------------------------------
CAEEL   QSQQQYNSGRPQMHTTPTKNDMSARAPSGAMGQIANRMGHGTNPQGYGST
YEAST*  --------------------------------------------------
SCHPO   --------------------------------------------------
CANAL   --------------------------------------------------
CANGA   --------------------------------------------------
ASPNI   --------------------------------------------------
RHIO9   --------------------------------------------------
CRYNE   --------------------------------------------------
USTMA   --------------------------------------------------
ARATH*  --------------------------------------------------
VITVI   --------------------------------------------------
SOYBN   --------------------------------------------------
SOLLC   --------------------------------------------------
ORYSJ*  --------------------------------------------------
HORVU   --------------------------------------------------
SORBI   --------------------------------------------------
SELML   --------------------------------------------------
PHYPA   --------------------------------------------------
VOLCA   AVALALADNRKARELISVAEDLRSGAAADGAGELPYPGAGSPAALAAQQR
                 5810      5820      5830      5840      5850
```

MED12

```
HUMAN*  --------------------QQQQQYHIRQQQQQQILRQQQQQQQQQQ--
CHICK   --------------------QHCAQFHLHPFTKVQPRREWQRRAVRWV--
XENTR   --------------------TQGQPPGLGMQTLQVSVGVGSVLSVSTE--
DANRE   --------------------QQQQVQQQVQQQVQQQQVQQQQVQQQVQ--
CIOIN   --------------------------------------------------
ANOGA   --------------------AQQQRNTQAEFLA-------------QQRA
TETNG   --------------------EHEAQPDAGRATAATTATATSSTATAAV--
DROME   --------------------MQQGNRNNPDFMAAAAVAQQQQQQQQQQRV
BRUMA   --------------------------------------------------
CAEEL   GQNVPGGYQQGQQQSGQGSYPQAQQQQPNQYSGSNQQVGQQAQQQQQQPL
YEAST*  --------------------------------------------------
SCHPO   --------------------------------------------------
CANAL   --------------------------------------------------
CANGA   --------------------------------------------------
ASPNI   --------------------------------------------------
RHIO9   --------------------------------------------------
CRYNE   --------------------------------------------------
USTMA   --------------------------------------------------
ARATH*  --------------------------------------------------
VITVI   --------------------------------------------------
SOYBN   --------------------------------------------------
SOLLC   --------------------------------------------------
ORYSJ*  --------------------------------------------------
HORVU   --------------------------------------------------
SORBI   --------------------------------------------------
SELML   --------------------------------------------------
PHYPA   --------------------------------------------------
VOLCA   LRQRQQRREELRQAALPPAWGPGLPLDASVVSVLSMYGRHGLMRCLAELS
                  5860      5870      5880      5890      5900
```

```
HUMAN*  --------------------------------------------------
CHICK   --------------------------------------------------
XENTR   --------------------------------------------------
DANRE   --------------------------------------------------
CIOIN   --------------------------------------------------
ANOGA   A----MAAGRGQYGQHAPNVTMGNM-----GVNQGAVPPYPRQ----GGK
TETNG   --------------------------------------------------
DROME   VPGGMMAGNRNQYMNQAPNVTMSTMMGPGPGGVVGQVPPYARQQSAGGGK
BRUMA   --------------------------------------------------
CAEEL   NQNVSQSQSAAQFGRPSQDSAYQQSGYNQTGNQSYQRPDQQQQGAQQNQW
YEAST*  --------------------------------------------------
SCHPO   --------------------------------------------------
CANAL   --------------------------------------------------
CANGA   --------------------------------------------------
ASPNI   --------------------------------------------------
RHIO9   --------------------------------------------------
CRYNE   --------------------------------------------------
USTMA   --------------------------------------------------
ARATH*  --------------------------------------------------
VITVI   --------------------------------------------------
SOYBN   --------------------------------------------------
SOLLC   --------------------------------------------------
ORYSJ*  --------------------------------------------------
HORVU   --------------------------------------------------
SORBI   --------------------------------------------------
SELML   --------------------------------------------------
PHYPA   --------------------------------------------------
VOLCA   TARGDRFSFRPQGLAAPTAPMPFSGVDGGNGWRTLPDPLVVRQQLGFSWC
                  5910      5920      5930      5940      5950
```

```
HUMAN*  -------QQQQQQQQQQQQQQQQHQQQQQQ--------QAAPPQ----PQ
CHICK   -------REHVRQRLSEQVSTVPQPQAQGQPP--GLGMQALPPQ------
XENTR   -------------------WRYLHELLNFTGDFIPIGFLSFLTD------
DANRE   -------PQQVQQQQVQQQQQ--QQVSAAQPPAQALGMQALPPQ------
CIOIN   --------------------------------------------------
ANOGA   PGVGGNMPQNQQQQFQQQRMRLIMQQHSGMG-----------------QG
TETNG   -------PQTTSTKTAAAAAAAGPAGSAAAAAAAAAAAATGPTS------
DROME   PGVLNTQQQFQQQQQQQQQLRHQMMQLQGMGGGAGGGMGAGPQQGGGAVG
BRUMA   --------------------------------------------------
CAEEL   SGSNQAQNQLRSQQQAQQPLQQPQQSQQFQQPAQQAKNPMAQSAQYGGFG
YEAST*  --------------------------------------------------
SCHPO   --------------------------------------------------
CANAL   --------------------------------------------------
CANGA   --------------------------------------------------
ASPNI   --------------------------------------------------
RHIO9   --------------------------------------------------
CRYNE   --------------------------------------------------
USTMA   --------------------------------------------------
ARATH*  --------------------------------------------------
VITVI   --------------------------------------------------
SOYBN   --------------------------------------------------
SOLLC   --------------------------------------------------
ORYSJ*  --------------------------------------------------
HORVU   --------------------------------------------------
SORBI   --------------------------------------------------
SELML   --------------------------------------------------
PHYPA   --------------------------------------------------
VOLCA   AYEGCTNLEGSSEATLLTRAYGSGRAHPAACYCRVSCQQADRQALAPASL
                  5960      5970      5980      5990      6000
```

MED12

Species	6001–6050
HUMAN*	PQSQPQFQRQGLQ-------------------------------------
CHICK	---QPIFQRQGLQ-------------------------------------
XENTR	-KSLSQFQRQGLQ-------------------------------------
DANRE	---QPMFQRQGMQ-------------------------------------
CIOIN	-------QVMGHQ-------------------------------------
ANOGA	GNAQGMMQNQG---------------------------------------
TETNG	---AGSSSAAGRT-------------------------------------
DROME	GGAGGGMVPQQ---------------------------------------
BRUMA	--
CAEEL	GQQQGYDQQQQGQIAPQQAQNPQASQSYGQQQTQQNRYGMGSSGYTANSG
YEAST*	--
SCHPO	--
CANAL	--
CANGA	--
ASPNI	--
RHIO9	--
CRYNE	--
USTMA	--
ARATH*	--
VITVI	--
SOYBN	--
SOLLC	--
ORYSJ*	--
HORVU	--
SORBI	--
SELML	--
PHYPA	--
VOLCA	EARGGSEMRLASRGTVNGLRS-----------------------------
	6010 6020 6030 6040 6050

Species	6051–6100
HUMAN*	--
CHICK	--
XENTR	--
DANRE	--
CIOIN	--
ANOGA	--
TETNG	--
DROME	--
BRUMA	--
CAEEL	GSSNILNQSMEESGLNQGFSGASSNASSQQGGSSQMQQSGYGMPGNQMQM
YEAST*	--
SCHPO	--
CANAL	--
CANGA	--
ASPNI	--
RHIO9	--
CRYNE	--
USTMA	--
ARATH*	--
VITVI	--
SOYBN	--
SOLLC	--
ORYSJ*	--
HORVU	--
SORBI	--
SELML	--
PHYPA	--
VOLCA	--
	6060 6070 6080 6090 6100

Species	6101–6150
HUMAN*	--
CHICK	--
XENTR	--
DANRE	--
CIOIN	--
ANOGA	--
TETNG	--
DROME	--
BRUMA	--
CAEEL	QQNQKQQVQRGMPTGMGQTNMGQSGMGQSGMGQTGMSRSGLGGGIGQQGQ
YEAST*	--
SCHPO	--
CANAL	--
CANGA	--
ASPNI	--
RHIO9	--
CRYNE	--
USTMA	--
ARATH*	--
VITVI	--
SOYBN	--
SOLLC	--
ORYSJ*	--
HORVU	--
SORBI	--
SELML	--
PHYPA	--
VOLCA	--
	6110 6120 6130 6140 6150

MED12

```
HUMAN*  --------------------------------------------QTQQQQ
CHICK   --------------------------------------------QTQQQQ
XENTR   --------------------------------------------QTQQQQ
DANRE   --------------------------------------------QTQQQQ
CIOIN   --------------------------------------------SVMNPQ
ANOGA   --------------------------------------------QGMSTQ
TETNG   --------------------------------------------TTTTTT
DROME   --------------------------------------------QSMNQQ
BRUMA   --------------------------------------------QQQQQQ
CAEEL   QSQQPQQPQVSQQQNQRGMNPGAQLPPYSTGQQQHQPQQSQISQQQQQQD
YEAST*  --------------------------------------------------
SCHPO   --------------------------------------------------
CANAL   --------------------------------------------------
CANGA   --------------------------------------------------
ASPNI   --------------------------------------------------
RHIO9   --------------------------------------------------
CRYNE   --------------------------------------------------
USTMA   --------------------------------------------------
ARATH*  --------------------------------------------------
VITVI   --------------------------------------------------
SOYBN   --------------------------------------------------
SOLLC   --------------------------------------------------
ORYSJ*  --------------------------------------------------
HORVU   --------------------------------------------------
SORBI   --------------------------------------------------
SELML   --------------------------------------------------
PHYPA   --------------------------------------------------
VOLCA   --------------------------------------------------
                            6160      6170      6180      6190      6200
```

```
HUMAN*  QTAALVRQLQQQLSNTQPQPS---TNI-----------------------
CHICK   QTAALVRQLQQQLSNTQPQQN---NNP-----------------------
XENTR   QTAALVRQLQQQLSSTQPQQN---SNP-----------------------
DANRE   QTAALVRQLQQQLSSTQPAQN---NSS-----------------------
CIOIN   Q------QYSTRMLNTQPQSNQLIDMRRNIHQ------------------
ANOGA   QTPNLVAQLQRQM----PNQN---NMMGQQYP------------------
TETNG   TAAAAAAAAGVGSTATRPGTEPGSGNAAATPTATYVSPTGNAADSAAAAD
DROME   QTPNLVAQLQRQ------------NMMGQQ-Q------------------
BRUMA   QY------------------------------------------------
CAEEL   QYRRMQAAQMQQQPTAQGQQNRMGMPSQQQSGAAYSNQMQFQGVRQGQQG
YEAST*  --------------------------------------------------
SCHPO   --------------------------------------------------
CANAL   --------------------------------------------------
CANGA   --------------------------------------------------
ASPNI   --------------------------------------------------
RHIO9   --------------------------------------------------
CRYNE   --------------------------------------------------
USTMA   --------------------------------------------------
ARATH*  --------------------------------------------------
VITVI   --------------------------------------------------
SOYBN   --------------------------------------------------
SOLLC   --------------------------------------------------
ORYSJ*  --------------------------------------------------
HORVU   --------------------------------------------------
SORBI   --------------------------------------------------
SELML   --------------------------------------------------
PHYPA   --------------------------------------------------
VOLCA   --------------------------------------------------
                            6210      6220      6230      6240      6250
```

```
HUMAN*  ------------------FGRY----------------------------
CHICK   ------------------FGRY----------------------------
XENTR   ------------------FGRY----------------------------
DANRE   --------------------YY----------------------------
CIOIN   ----------------QGPPPY----------------------------
ANOGA   ----------------HQPPQY----------------------------
TETNG   GSTGQTAATATLK-------------------------------------
DROME   ----------------YQPPPY----------------------------
BRUMA   ----------------HPTQQY----------------------------
CAEEL   MGGMGGSGQQQPQTQPHGSNQYYQQQQDQRMQQQPQQPGQQQQHGYGMGQ
YEAST*  --------------------------------------------------
SCHPO   --------------------------------------------------
CANAL   --------------------------------------------------
CANGA   --------------------------------------------------
ASPNI   --------------------------------------------------
RHIO9   --------------------------------------------------
CRYNE   --------------------------------------------------
USTMA   --------------------------------------------------
ARATH*  --------------------------------------------------
VITVI   --------------------------------------------------
SOYBN   --------------------------------------------------
SOLLC   --------------------------------------------------
ORYSJ*  --------------------------------------------------
HORVU   --------------------------------------------------
SORBI   --------------------------------------------------
SELML   --------------------------------------------------
PHYPA   --------------------------------------------------
VOLCA   --------------------------------------------------
                            6260      6270      6280      6290      6300
```

HUMAN*	-	-	-	-	-	-	-	-	-	-
CHICK	-	-	-	-	-	-	-	-	-	-
XENTR	-	-	-	-	-	-	-	-	-	-
DANRE	-	-	-	-	-	-	-	-	-	-
CIOIN	-	-	-	-	-	-	-	-	-	-
ANOGA	-	-	-	-	-	-	-	-	-	-
TETNG	-	-	-	-	-	-	-	-	-	-
DROME	-	-	-	-	-	-	-	-	-	-
BRUMA	-	-	-	-	-	-	-	-	-	-
CAEEL	Y	P	N	Q	Q	P	P	N	Q	Y
YEAST*	-	-	-	-	-	-	-	-	-	-
SCHPO	-	-	-	-	-	-	-	-	-	-
CANAL	-	-	-	-	-	-	-	-	-	-
CANGA	-	-	-	-	-	-	-	-	-	-
ASPNI	-	-	-	-	-	-	-	-	-	-
RHIO9	-	-	-	-	-	-	-	-	-	-
CRYNE	-	-	-	-	-	-	-	-	-	-
USTMA	-	-	-	-	-	-	-	-	-	-
ARATH*	-	-	-	-	-	-	-	-	-	-
VITVI	-	-	-	-	-	-	-	-	-	-
SOYBN	-	-	-	-	-	-	-	-	-	-
SOLLC	-	-	-	-	-	-	-	-	-	-
ORYSJ*	-	-	-	-	-	-	-	-	-	-
HORVU	-	-	-	-	-	-	-	-	-	-
SORBI	-	-	-	-	-	-	-	-	-	-
SELML	-	-	-	-	-	-	-	-	-	-
PHYPA	-	-	-	-	-	-	-	-	-	-
VOLCA	-	-	-	-	-	-	-	-	-	-
										6310

MED13

```
HUMAN*  --------------------------------------------------
CHICK   --------------------------------------------------
XENTR   --------------------------------------------------
DANRE   --------------------------------------------------
CIOIN   --------------------------------------------------
ANOGA   --------------------------------------------------
TETNG   --------------------------------------------------
DROME   --------------------------------------------------
BRUMA   --------------------------------------------------
CAEEL   --------------------------------------------------
YEAST*  --------------------------------------------------
SCHPO   --------------------------------------------------
CANAL   --------------------------------------------------
CANGA   --------------------------------------------------
ASPNI   --------------------------------------------------
RHIO9   --------------------------------------------------
CRYNE   --------------------------------------------------
USTMA   --------------------------------------------------
ARATH*  --------------------------------------------------
VITVI   --------------------------------------------------
SOYBN   --------------------------------------------------
SOLLC   --------------------------------------------------
ORYSJ*  MPEFPSPSSPAGGGRRRMLNNADNKPDLHGTMNDPYYYDCRILVDATPEK
HORVU   --------------------------------------------------
SORBI   --------------------------------------------------
SELML   --------------------------------------------------
PHYPA   --------------------------------------------------
                 10        20        30        40        50
```

```
HUMAN*  --------------------------------------------------
CHICK   --------------------------------------------------
XENTR   --------------------------------------------------
DANRE   --------------------------------------------------
CIOIN   --------------------------------------------------
ANOGA   --------------------------------------------------
TETNG   --------------------------------------------------
DROME   --------------------------------------------------
BRUMA   --------------------------------------------------
CAEEL   --------------------------------------------------
YEAST*  --------------------------------------------------
SCHPO   --------------------------------------------------
CANAL   --------------------------------------------------
CANGA   --------------------------------------------------
ASPNI   --------------------------------------------------
RHIO9   --------------------------------------------------
CRYNE   --------------------------------------------------
USTMA   --------------------------------------------------
ARATH*  --------------------------------------------------
VITVI   --------------------------------------------------
SOYBN   --------------------------------------------------
SOLLC   --------------------------------------------------
ORYSJ*  SSRKKSTFVRHFSCTSSHALVKIAAAIVDPRAVVLHGREPHGEQGGGGAT
HORVU   --------------------------------------------------
SORBI   -----------------------------------MRRAYDGSTGSQEAA
SELML   --------------------------------------------------
PHYPA   --------------------------------------------------
                 60        70        80        90       100
```

```
HUMAN*  ------------------------------------------MSASFVPN
CHICK   ------------------------------------------MSSCFVPN
XENTR   ----------------------------------------VRMSSCFVPN
DANRE   -----------------------------------------MMSSCFVPN
CIOIN   -----------------------------------------MANSSTVAN
ANOGA   --------------------------------------------------
TETNG   ------------------------------------------MNPCFVPN
DROME   -----------------------------------------MTHQNHQTN
BRUMA   ----------------------------------------------MSTN
CAEEL   -------------------------MSSAKDSDKDASRKMKLQKRPEWNN
YEAST*  --------------------------------------------MSSDAS
SCHPO   --------------------------------------------------
CANAL   ------------------------------------------------MV
CANGA   --------------------------------------------MKEATD
ASPNI   --------------------------------------------------
RHIO9   -------------------------------------MDEAEDLDEHLIY
CRYNE   ----------------------------------------MPDILNQAPA
USTMA   ----------------MWNQSFTSAPAVSIGGGSGNATSGVAHPSGSFAS
ARATH*  --------------------------------------------------
VITVI   --------------------------------------------------
SOYBN   --------------------------------------------------
SOLLC   --------------------------------------------------
ORYSJ*  GERGHSRSRAERTDPTTTAPFFSGSNRRQVADLFRRPPSRFSDVFGMLIR
HORVU   --------------------------------------------------
SORBI   AQ------------------------------------------------
SELML   --------------------------------------------------
PHYPA   ---------------------------------------------MTTEK
                110       120       130       140       150
```

MED13

HUMAN*	GASLEDCHCNLFCLADLTGIKWKKYVWQGPTSAPI---------------
CHICK	GASLEDCHSNLFCLADLTGIKWKRYVWQGPTSAPI---------------
XENTR	GASLEDCHSNLFCLADLTGIKWKRYIWQGPTSAPI---------------
DANRE	GASLEDCHSNLFCLADLTGIKWKCFVWQGPTSSPI---------------
CIOIN	GARLDDCHSSVFSLADLSGIKWHRYSS-----------------------
ANOGA	-------MDSSSSATDLCGIRWRQLVLGERPN------------------
TETNG	GASLEDCHSNLFCLADLTGIKWRRFVWQGPTSSPI---------------
DROME	GASLEDCHTNFYALTDLCGIKWRKFVNGERPN------------------
BRUMA	GGSLEDCHTNVFALTELNGLKWKCLTTPLSQR------------------
CAEEL	GGSLEDCISNVYALLELPGIKWKCYRTKPNAP------------------
YEAST*	TYRLEDVLSSFYRVEKIKKINYHQYISKAQND------------------
SCHPO	MVHDGVHLISIKTIPCPNTIYYAKYQVQRKDNED----------------
CANAL	GANASSISTQYYKLAHLGSISYTIYTSSDNND------------------
CANGA	DYDLSKLVSNFYRLEKITKINYAQYLPKKQND------------------
ASPNI	MDFPGGCITNVRVIDGFSNVYWRIYTEDTSGTNPPGDGPNNGHTLLKHIS
RHIO9	LMLTDSSLTNILIVSGVSQIRYRVYHQYCSKT------------------
CRYNE	TPSSALYATHILHLPK-DRIYIHRYALSSAPG------------------
USTMA	HARSNSLAASPSAHRRVSGIAQGQLASSPSANPPFSRPFANTAFPGTVVA
ARATH*	------MWTNVFRIGGLHNVSWFQFLPSETEL-----------------N
VITVI	------MPLSLAG-GGLHHISWFQFLPHESDL-----------------N
SOYBN	--
SOLLC	------MWTNVFKIGGLHQISWFQFLPHESDV-----------------N
ORYSJ*	DPRCYVYSCIGYLKGELQNVSWFQFLPVEPDP-----------------S
HORVU	--
SORBI	------------LRGELQTVSWFQFLPFEPDA-----------------S
SELML	-----MMFTNVFRI------------------------------------
PHYPA	GPVAKRVEKKVARGKGMTVLGGTVIGSRAGSGGARARLTPWLGTEVGSWA

160 170 180 190 200

HUMAN*	------------------------------------LFPVTEEDPILSSF
CHICK	------------------------------------LFPVTEEDPILSSF
XENTR	------------------------------------LFPVTEEDPILSSF
DANRE	------------------------------------LFPVTEEDPILCSF
CIOIN	------------------------------------ISPGLADDPVLAAF
ANOGA	------------------------------------AAGDPLDDPVVRSY
TETNG	------------------------------------LFPVTEEDPILCSF
DROME	------------------------------------ASSDPLADPILRSY
BRUMA	------------------------------------TWTSLEQDPVLSAY
CAEEL	------------------------------------RGVALTADFVLKAY
YEAST*	--
SCHPO	--
CANAL	--QA
CANGA	--
ASPNI	RLKELELRLRSQGSLVLIYPRRLGLLIFSATPDFEHLAPVLLSESGDQTN
RHIO9	---------LLTESLKKT------------------PEYDRSKNILLKAY
CRYNE	---AST
USTMA	PRSTAASNTASAPTRSLQ------------------RLPLLTSQTPTSIA
ARATH*	PGFDRSSRA---------------------------EQNEVATYLVLSSH
VITVI	PPNDKSVKV---------------------------EQKDPATLVVLSXH
SOYBN	--
SOLLC	SLVDKRVKP---------------------------DKKDVATWLVLSSH
ORYSJ*	TASERSSKA---------------------------EQKDALNNIVLSAH
HORVU	--
SORBI	AASEKSSKA---------------------------EQKDALNSIVLSAY
SELML	------------------------------------VSAAVAVGAVVADE
PHYPA	SSPELQPCLPMSSALEER------------------SQQDVAAMQVIRAH

210 220 230 240 250

HUMAN*	SRCLKADVLGVWRRDQRP--------------------------------
CHICK	SRCLKADVLSVWRRDQRP--------------------------------
XENTR	SRCLKADVLSVWRRDERP--------------------------------
DANRE	SRCLKADVLSVWRRHQTP--------------------------------
CIOIN	TKCLALDILAVWRKSSSKKSPMQHG-------------------------
ANOGA	SKCLAVDILCVWRRVVAPKPKPKPDPDPSSMFDMSIPGTGNSGSVVHP--
TETNG	SRCMAADVLSVWRRHHTQ--------------------------------
DROME	SRCIQADMLCVWRRVQSTKTDHADPNALTFEMTTSTKVHP----------
BRUMA	ARCLQAGILCAWRRQPPQPTGTLAAMQLP---------------------
CAEEL	SKCLLDGILCTWRRKPLPPTDNKETPKLT---------------------
YEAST*	----------QWSIQMEFMLRKQDPK------------------------
SCHPO	--
CANAL	LLELELTIRHKWPQ------------------------------------
CANGA	----------QWGIQMEIQMRKKDRK------------------------
ASPNI	RLLVGSTILKVWTSGSVSAADLAKS-------------------------
RHIO9	FELLAISIPVTWRIAHENEE------------------------------
CRYNE	KDVVEDKMAKFWRDIADRAGNGKRLLEGPVA-------------------
USTMA	KLAFPAHTTVFWHRLHKNNARLAPQLDPDYQRVDASTSTAASSANTSQGS
ARATH*	LRLQKEGFLTTWTNSFVG------------------------PWDPSQG-
VITVI	LQLQREGFLSTWTNSFVG------------------------PWDPSQ--
SOYBN	--------MFMWIEEV----------------------------------
SOLLC	LQLQKEGFLSTWTNSFVG------------------------PWDPSQG-
ORYSJ*	LHLQSEGFLSTWTNSFVG------------------------PWDPSQG-
HORVU	--
SORBI	LHLQSEGFLSTWTNSFVG------------------------PWDPSQG-
SELML	CRFYRAGCKKS-------------------------------RGFSSLR-
PHYPA	MWLQEAGTLSSWTLS-----------------------------------

260 270 280 290 300

MED13

```
HUMAN*  --------------------------------------------------
CHICK   --------------------------------------------------
XENTR   --------------------------------------------------
DANRE   --------------------------------------------------
CIOIN   --------------------------------------------------
ANOGA   --------------------------------------------------
TETNG   --------------------------------------------------
DROME   --------------------------------------------------
BRUMA   --------------------------------------------------
CAEEL   --------------------------------------------------
YEAST*  --------------------------------------------------
SCHPO   --------------------------------------------------
CANAL   --------------------------------------------------
CANGA   --------------------------------------------------
ASPNI   --------------------------------------------------
RHIO9   --------------------------------------------------
CRYNE   --------------------------------------------------
USTMA   QNAWNTAVQHIITTCKQSRIQPPVTEEPQHHLTPFQLLLKDVLFYTSSRH
ARATH*  --------------------------------------------------
VITVI   --------------------------------------------------
SOYBN   --------------------------------------------------
SOLLC   --------------------------------------------------
ORYSJ*  --------------------------------------------------
HORVU   --------------------------------------------------
SORBI   --------------------------------------------------
SELML   --------------------------------------------------
PHYPA   --------------------------------------------------
                         310       320       330       340       350
```

```
HUMAN*  ---------------GRRELWIFW------WG--------EDPSFAD-LI
CHICK   ---------------GRRELWIFW------WG--------DDPNFAD-LI
XENTR   ---------------GRRELWIFW------WG--------EDPNFAD-LI
DANRE   ---------------GRRELWIFW------WG--------DDPNFAE-LV
CIOIN   ----------LPQEGIERELWIFW------WG--------EEPNID----
ANOGA   ----------PLSLTAAKELWIFW------YG--------EEPDLTD-LV
TETNG   ---------------GRRELWLFW------WG--------DDPSFAE-LI
DROME   ----------PLSLAAAKELWIFW------YG--------EEPDLSE-LV
BRUMA   ----------DYRIDVVKELWVFW------YSH-------EEPDCLT---
CAEEL   ----------NFSNDAPKELWVFW------YD--------DEPALLQKNC
YEAST*  ----------TLVALLSRDLWCFS------IN--------DDPVPTPPAI
SCHPO   ----------AASLLEDKEAYLRN---------------------QDCIV
CANAL   ----------ILVTYYNKSLYYFV------FGH-------NKVDSETIDL
CANGA   ----------LLVALLSKELWCFS------IN--------DQVLPSPNDI
ASPNI   --------------------------------------------------
RHIO9   ----------NLDNQILLELWVFW------FDDGHTGKVDSNESLRELDE
CRYNE   ----------TIVGQIERELWCFA--------------CKEDGISPPDGI
USTMA   SSPTGDTHTHLIAPVSHADLWLFV------VV----------TDQEEIKA
ARATH*  ----------LYNPDEKIKLWLFL------PG--------RHSSISD-KA
VITVI   --------------DEKIKLWLFL------PG--------RHSSVAE-AA
SOYBN   ---------------EVSRLWVWLLRASRCPG------------------
SOLLC   ----------LYNPDEKIKLWLFF------PG--------QHSSVVE-KA
ORYSJ*  ----------EHNP------------------------------------
HORVU   --------------------------------------------------
SORBI   ----------EHNPDEKIKLWLFL------PG--------CHSSVSE-KA
SELML   ----------TIPRGQKLKLWLFL------PG--------RHIDSRS---
PHYPA   --------------DSKVKLWLFL------PG--------RHDGLNP-RV
                         360       370       380       390       400
```

```
HUMAN*  HH--------------DLSE--------EEDG-VWE-NGLSYEC------
CHICK   HH--------------DLSE--------EEDG-VWE-NGLSYEC------
XENTR   HH--------------DLAD--------EEDG-IWD-NGLSYEC------
DANRE   HH--------------DLSC--------NEDG-SWE-SGLTYEC------
CIOIN   ----------------EISQQRGLND--IGRG-SWD-EGLTYEC------
ANOGA   AP--------------ELLNSSEA----GDKG-SWE-NGLSYEC------
TETNG   HN--------------ELSS--------EEDG-EWE-SGLSYEC------
DROME   DA--------------ELLRVAANQA--LWNG-TWK-GALTYEC------
BRUMA   ----------------EYTSHLACSE--DGQG-NWSVPGIQYET------
CAEEL   EGLDNDD---------ELSSANQMNI-------------VSYEV------
YEAST*  EHK-PVSPDKIGTFTADYSKPNLPPH------------------------
SCHPO   HAKNDLLFVYDFQAIPSIPEESSSFMLLNSGAFSRLALFQKDELALLLDL
CANAL   TK--------------EFPQLSTKYT--DTVN-VDQFPNPHKSTKNDDNL
CANGA   DPELNVSADKTGSFTADYSKPNLPPH------------------------
ASPNI   ----------------HLSDLQTTNA--NSPAPSQRLQNNSVQPRRADNS
RHIO9   AKVGSFTWEN------AYSKLQSPTASPLSSG-VQADTAAPVEVSTE---
CRYNE   NEIQPPLPPLVPLELLSCPDHSSSYNCLSESS-CDVTLNLEGETRKAWEL
USTMA   ST--------------SASSAGASDT--NATG----AAAANQEGTSPLHP
ARATH*  QA--------------AVSKL---RV--VASG-IWVAPGDSEEI------
VITVI   QV--------------AVSRL---RV--VASG-FWLAPGDSEEV------
SOYBN   ----------------ALLFLVSVSM--YETGFVWRALGNV---------
SOLLC   QS--------------AISKL---RV--LASG-LWVAPGDSEEV------
ORYSJ*  -------------------------V--VSNG-LWVAPGNLEEV------
HORVU   --------------------------------------------------
SORBI   QP--------------AVNKL---RV--ASNG-LWVAPGNSEEV------
SELML   -----------------SLDL---RV--LKSG-VWTCPGTSTEV------
PHYPA   HA--------------AVAGL---KV--VGSG-LWRAPGECTEV------
                         410       420       430       440       450
```

MED13

```
HUMAN*  ------RTLLFKAVHNLLERCLMNR---NF--------------------
CHICK   ------RTLLFKAVHNLLERCLMNR---NF--------------------
XENTR   ------RTLLFKAIHNLLERCLMNR---NF--------------------
DANRE   ------RTLLFKAIHNLLERCLMNR---SF--------------------
CIOIN   ------RTLLFKAIHNLVERCLLNE---NF--------------------
ANOGA   ------RSLLFKALHNVIERCLLSR---GF--------------------
TETNG   ------RTLLFKAIHNLLERCLMNR---GF--------------------
DROME   ------RSLLFKALHNLMERFVLTK---DI--------------------
BRUMA   ------RTLFFKALHNLIERNMLKN---GY--------------------
CAEEL   ------RTIIFKALHVVLERDLTKD---GF--------------------
YEAST*  ------YALFLKALRRKIYINLALGSHNKL--------------------
SCHPO   ------YINFLQGLKKTVLYWLCKE--YNF--------------------
CANAL   AWA---SLSFLKATKKMILYNLSLSGAIKL--------------------
CANGA   ------YALFLKSLKRMIYLNLVEQSKNTL--------------------
ASPNI   GGSAAIYASFISAVTAAFSLQIVRQ--NNA--------------------
RHIO9   ------YKMFIRSLRNLVDAQMKNQ---GA--------------------
CRYNE   LANALIETFAWKGGRRIILRDS------GV--------------------
USTMA   TLVRRLPANLVKSLQRLSECAITSSGSYPLPVAAPNCIDLKGKRSSLTQD
ARATH*  ------SVAFSQSLRNCIERALSGI---SY--------------------
VITVI   ------AAALSQALRNCIERALIGL---NY--------------------
SOYBN   --------------------------------------------------
SOLLC   ------AAALSQALRNCMERTLRGL---SY--------------------
ORYSJ*  ------AAALCQALRNSLERTLRGL---SY--------------------
HORVU   --------------------------------------------------
SORBI   ------AAALSQALRNSLERSLKGL---SY--------------------
SELML   ------TAALCQALRNRFERALGAF---AY--------------------
PHYPA   ------GFALDQALRNRIEKGFQTV---SY--------------------
                 460       470       480       490       500
```

```
HUMAN*  -----------------------------------------VRIGKWFVK
CHICK   -----------------------------------------VRIGKWFVK
XENTR   -----------------------------------------VRIGKWFVK
DANRE   -----------------------------------------VRIGKWFVK
CIOIN   -----------------------------------------VRIGRWFVK
ANOGA   -----------------------------------------VRIEQWFVH
TETNG   -----------------------------------------VRIGKWFVK
DROME   -----------------------------------------VRFGKWFVQ
BRUMA   -----------------------------------------IRIGKYFTR
CAEEL   -----------------------------------------VRFGRWFTV
YEAST*  -----------------------------------------IQFGNACIS
SCHPO   -----------------------------------------VPIYGVLLP
CANAL   -------------------------------------------FGNYCVA
CANGA   -----------------------------------------VQFGNSCIN
ASPNI   -----------------------------------------IPLGPRTLF
RHIO9   -----------------------------------------VPLGEFYIF
CRYNE   -----------------------------------------VPFTPPFKP
USTMA   QRQAHRRFMASVKAKLVDALAASQSTSTNPTQSHAMPRRARLRLGRGVVF
ARATH*  -----------------------------------------MRFGDVFSK
VITVI   -----------------------------------------MRFGDVFSK
SOYBN   --------------------------------------------------
SOLLC   -----------------------------------------VRYGDVFTK
ORYSJ*  -----------------------------------------ARFGDVFTK
HORVU   --------------------------------------------------
SORBI   -----------------------------------------ARFGDVFTK
SELML   -----------------------------------------VRFGDMFVK
PHYPA   -----------------------------------------VRFGDVFVK
                 510       520       530       540       550
```

```
HUMAN*  PYEKDEKPIN----------------------KSEHLSCSFTFFLHGDSN
CHICK   PYEKDEKPIN----------------------KSEHLSCSFTFFLHGDSN
XENTR   PYEKDEKPVN----------------------KSEHLSCSFAFFLHGDSN
DANRE   PYEKDEKPIN----------------------KSEHLSCSFTFFVHGDSN
CIOIN   PLNKDNEED-----------------------ISDRFSFSFSFFLHGESY
ANOGA   PTSNDERVFGK---------------------SSHHASFSFTFFVHGDST
TETNG   PYQKEEKIIN----------------------KSEHLSCAFTFFVHGDSN
DROME   PCTSSDRLFGR---------------------SSQHLSFSFTFFVHGDT-
BRUMA   PYEVPSSDRIHCS-------------------PSYVNGISFNFFVHGENI
CAEEL   PYDARENYLHFTY-------------------PSHSPAIRFNFFVHGSST
YEAST*  LS------------------------------GVPNYLVQLEPHLFVNGD
SCHPO   LKLHPSFDTQLWN-------------------IYGYPVVDLQLSVLSKGH
CANAL   PTG-----------------------------DSVYSILCIDPILFQNGD
CANGA   ITKN----------------------------NESNNLLQIEPHLFSNSE
ASPNI   NTVNGNLDDGQGPAGNGS--------------TSVSALTSLHVQLTSAGK
RHIO9   PSNDEFNDVMRDE-------------SIDSHMLTSSLCCSYNVYLASTNL
CRYNE   -----------------------------------------SLYPFTSGS
USTMA   LPPRPSDSSSIALSDAAWFAAPKQSISFDGRVESDCLMTRFTTLLTTTSL
ARATH*  FSP--QSEEYL---------------------RRGQPTVEFIFAATEEAV
VITVI   YHPFSQSEELFRRINLRLEKIQRDFLWGSRAGRRGQPTIEFIFAATEEAI
SOYBN   --------------------------------------------------
SOLLC   YRPFSQNEELF---------------------RKGQPVVEFVFAATEEAI
ORYSJ*  YYPPTRNQNSF---------------------RRAQPTIEFVFAATEEAI
HORVU   --------------------------------------------------
SORBI   YNPPTRNQNSF---------------------RRAQPTVEFVFAATEEAI
SELML   CKRYTADDSDF---------------------RRTLPSCEATFFASEEGV
PHYPA   CLRQSSSELNL---------------------RKSLFSCELVFTASNDAI
                 560       570       580       590       600
```

MED13

```
HUMAN*  VCTSVEINQHQ------------PVYLLSEEHITLAQQSNSP--------
CHICK   VCTSVEISQHQ------------PVYLLSEEHLTLAQQSNSP--------
XENTR   VCTSVEINQHQ------------PVYLLSEEHLTLAQQSNSP--------
DANRE   VCTSVEINQHQ------------PVYLLSEEHLTLAQQSSSS--------
CIOIN   VCTTIEIAKHQ------------PLERLTYQHFQHVTSSTN---------
ANOGA   VITAMDIREHP------------PVRLLTMKHLEEAQRAAAPGAAAAAAS
TETNG   VCTSVEIAQHQ------------PLQRLSEEHLSLAQQSSSP--------
DROME   VCASIDLREHP------------AVRPLTKEHLTEAAAAFAAASSPPGSN
BRUMA   VCATVSVQRQP------------TLFRLSRRHLDLGKK------------
CAEEL   ICASVQAQRQP------------TLITLARRHLECKTKR-----------
YEAST*  LTVSLCAKNMG-------------LVPMKEENLEESFLSK----------
SCHPO   IEFYLKPTRQT-------------VYRLSEVDNLVKKLD-----------
CANAL   LVVSCVEKPFT------------QLFSSSVAYPRNLAIDTN---------
CANGA   LAISICVKDLG-------------LLPLKVNSIDPAFLSS----------
ASPNI   LTIALQTIAQT------------GLLRLSQPEESASAILPG---------
RHIO9   IFQPNTRRMR--------------LKPIALYQNRMRG-------------
CRYNE   LLLSLLPSRSPTSLLPPYPIPTHTLIPLSLPSILITTVLTPTPAQIARLN
USTMA   FVYAHSRRFG--------------ALPLTDSRYATFLPSLPSSSAS----
ARATH*  FVHVIISAKN--------------VRTLSSGDAERMLRSSLKNSS---YR
VITVI   FVHVIISAKH--------------VRALASGDMEMVLKHSSNKYS---ES
SOYBN   --------------------------------------------------
SOLLC   FVHVIISAKH--------------IRALSSGDIDKISENTSNVSV---EG
ORYSJ*  FVHVIISARY--------------MRNLCSDDIEKVLTHSPRSVG---EG
HORVU   --------------------------------------------------
SORBI   FVHVLISARY--------------VRNLCSDDIEKVLTHSPPSIG---EG
SELML   FVHLRISRRR--------------MRPLAASDVRAVLTQHLTSSTPPIEN
PHYPA   FVHVFVRRKR--------------VRQLAISDLQAALAQQRSNLGMCDNS
                 610       620       630       640       650
```

```
HUMAN*  ----------------------------------------FQVILCPFGL
CHICK   ----------------------------------------FQVILSPFGL
XENTR   ----------------------------------------FQVILSPFGL
DANRE   ----------------------------------------VQVILSPYGL
CIOIN   ----------------------------------------NSVVLAPYGL
ANOGA   TTGGPASPDGGLNAAPGCAPASPGGSASGTVVPADDAIEPREVLLAPFAI
TETNG   ----------------------------------------LQVILSPYGL
DROME   GSAASAGGAVPNPGQDPNGASMDGLDGGEGAAKAAPPPHARKVMLAPFGI
BRUMA   ----------------------------------------QPVILGPWSM
CAEEL   ----------------------------------------VVVVVGPWSM
YEAST*  ----------------------------------------HALYLAPSGI
SCHPO   ----------------------------------------TVIRLAPTGC
CANAL   ----------------------------------------FVIYLIPSGI
CANGA   ----------------------------------------HALYMAPSGV
ASPNI   ----------------------------------------ADLWLAPTGS
RHIO9   ----------------------------------------RKAIISPSGE
CRYNE   QT--------------------------------------FDTLLGPGWK
USTMA   ----------------------------------------PTMLIAPLGY
ARATH*  LPAFRKCLGLAKS----------EDNRLCYINTSHRPMLFPPVIVSPHGM
VITVI   L----------------------------------------PVIVSPHGM
SOYBN   --------------------------------------------------
SOLLC   L----------------------------------------PVIVSPHGM
ORYSJ*  L----------------------------------------PVIVAPSGM
HORVU   --------------------------------------------------
SORBI   L----------------------------------------PVVVAPSGM
SELML   ----------------------------------------PAVVVAPYGL
PHYPA   ----------------------------------------LAVIVAPNGL
                 660       670       680       690       700
```

```
HUMAN*  NGTL---TGQAFKM------------------------------------
CHICK   NGTL---TGQSFKL------------------------------------
XENTR   NGTL---TGQSFKM------------------------------------
DANRE   SGTL---TGQSFKL------------------------------------
CIOIN   AATL---TGTAYRN------------------------------------
ANOGA   TGTL---TGQSYRA------------------------------------
TETNG   NGTL---TGQAFKM------------------------------------
DROME   AGIL---TGNSYKA------------------------------------
BRUMA   RAVL---LPEQPRIVDSTTPLLQQQSSASQYGNFTSLSSVAHQINPVPSS
CAEEL   RGQL---VQDQIAL------------------------------------
YEAST*  RMHL---APASKQG------------------------------------
SCHPO   LATLTSVHANASA-------------------------------------
CANAL   RCHLFDPTSLRNNL------------------------------------
CANGA   RVYL---VENKNDN------------------------------------
ASPNI   IARLITSHSEKNSH------------------------------------
RHIO9   YVTV---VSNTYNL------------------------------------
CRYNE   TGREQ---------------------------------------------
USTMA   HVELLGIVPTSSVA------------------------------------
ARATH*  RGSL---TGFCP--------------------------------------
VITVI   LGRF---TGCCP--------------------------------------
SOYBN   --------------------------------------------------
SOLLC   RGRL---TGCCP--------------------------------------
ORYSJ*  LGRL---VGCCP--------------------------------------
HORVU   --------------------------------------------------
SORBI   LGRL---VGCCP--------------------------------------
SELML   TGWL---TGCCP--------------------------------------
PHYPA   SGLL---TGCSP--------------------------------------
                 710       720       730       740       750
```

MED13

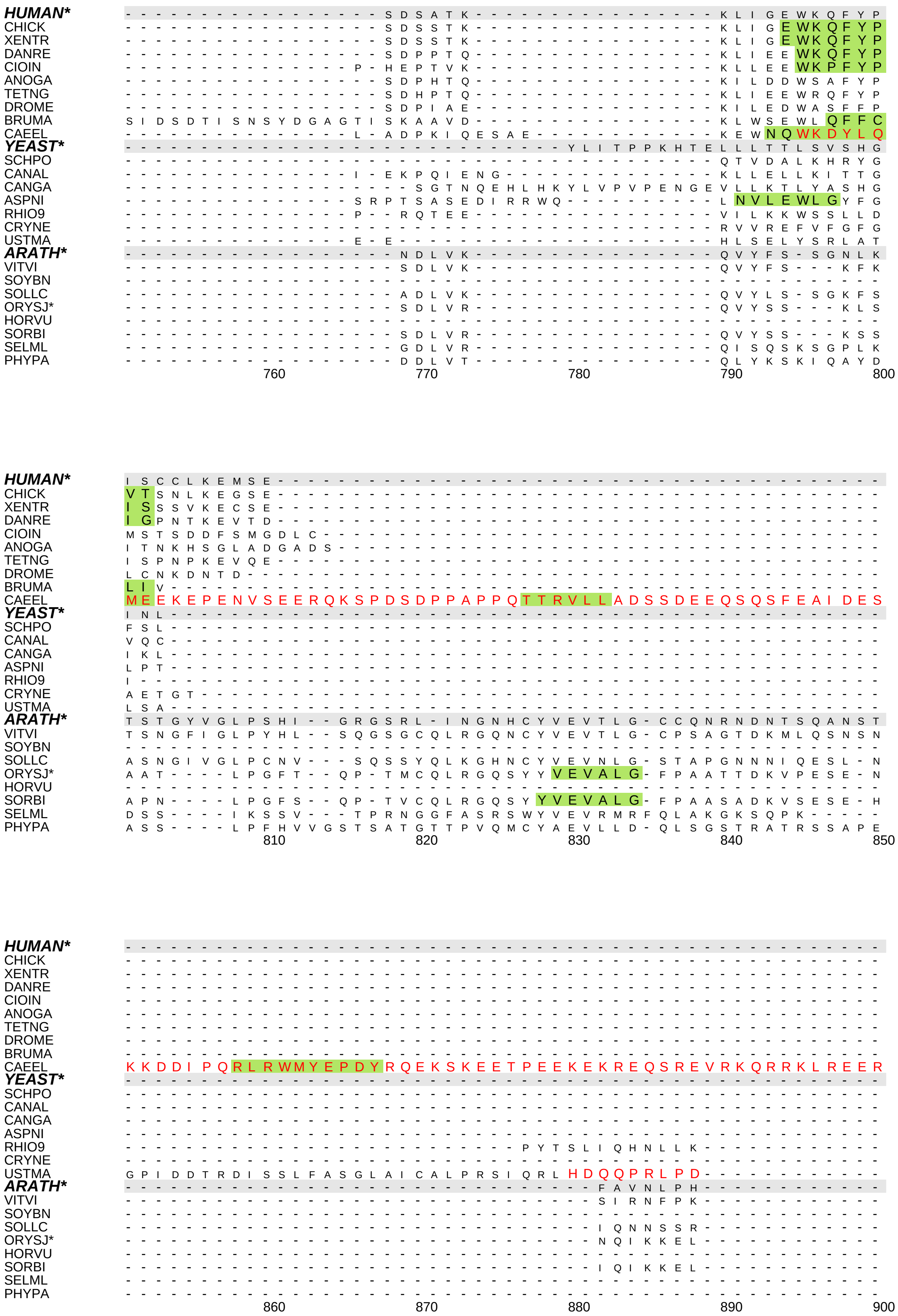

HUMAN*
CHICK
XENTR
DANRE
CIOIN
ANOGA
TETNG
DROME
BRUMA
CAEEL
YEAST*
SCHPO
CANAL
CANGA
ASPNI
RHIO9
CRYNE
USTMA
ARATH*
VITVI
SOYBN
SOLLC
ORYSJ*
HORVU
SORBI
SELML
PHYPA
760
770
780
790
800
810
820
830
840
850
860
870
880
890
900

MED13

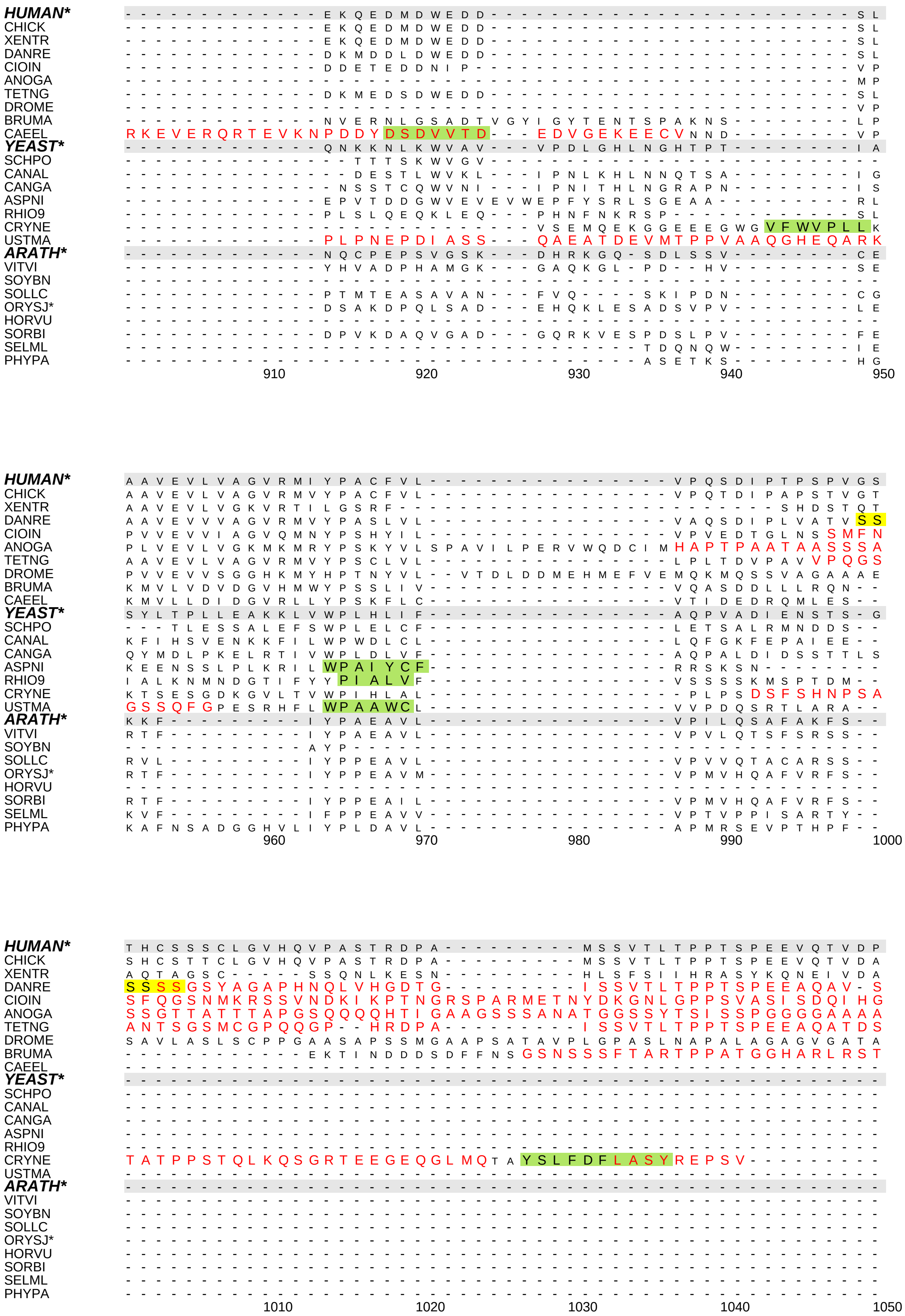
HUMAN*
CHICK
XENTR
DANRE
CIOIN
ANOGA
TETNG
DROME
BRUMA
CAEEL
YEAST*
SCHPO
CANAL
CANGA
ASPNI
RHIO9
CRYNE
USTMA
ARATH*
VITVI
SOYBN
SOLLC
ORYSJ*
HORVU
SORBI
SELML
PHYPA
910
920
930
940
950
960
970
980
990
1000
1010
1020
1030
1040
1050

MED13

```
HUMAN*  QSVQKWVKFSSVSDGF----------------------NSDSTSHHGGKI
CHICK   QSAQKWVKFSSVSDGF----------------------ISDSTSHHGGKI
XENTR   QSAQKWVKFPAVSDCF----------------------SSDTTSHHGGKI
DANRE   QPAQKWVKLSAMSGVF----------------------SVDSSSHHGGKI
CIOIN   PQSVGSSLFSPASA------------------------KPSPNKNTVVPV
ANOGA   GGGGSLHPHPKSSYSITYPGSSSGSSRSSHYHHQSTKDHHHHHHHHHHHR
TETNG   QLAQRWLKLSSASDGY----------------------SSHNT-LHGGKI
DROME   SSAAKEISRKAAPQAV-----SALERLAFQPYYDQRPTSGFTFNTNNTHI
BRUMA   PSFRKRKKRLSECDTG-------------------------SNAERHNAM
CAEEL   ------------------------------------------IGLKTNQQ
YEAST*  --------------------------------------------------
SCHPO   --------------------------------------------------
CANAL   --------------------------------------------------
CANGA   --------------------------------------------------
ASPNI   --------------------------------------------------
RHIO9   --------------------------------------------------
CRYNE   --------------------------------------------------
USTMA   --------------------------------------------------
ARATH*  --------------------------------------------------
VITVI   --------------------------------------------------
SOYBN   --------------------------------------------------
SOLLC   --------------------------------------------------
ORYSJ*  --------------------------------------------------
HORVU   --------------------------------------------------
SORBI   --------------------------------------------------
SELML   --------------------------------------------------
PHYPA   --------------------------------------------------
                  1060      1070      1080      1090      1100
```

```
HUMAN*  PRKLANHV--VDRVWQECNMNRAQNKRK----------------------
CHICK   PRKLANQV--VDRVWQECNMNRTQNKRK----------------------
XENTR   PRKLANQV--VDRVWQECNMNRAQNKRK----------------------
DANRE   PRRLASQM--VERVWQECNINRAQNKRK----------------------
CIOIN   PGYIQQQQRPPNQPLPSNIIKRICLSSNKTQSNQE---------------
ANOGA   PQRVAFHKRPPQEALFRPGTEPLSLSAAAALAAAAAAAAAANAASSPGAV
TETNG   PCRMAGQM--VETVWQEYNLNRTGHKRK----------------------
DROME   PASAAVEM--PERTWQDCVMNTLHVDAA----AAAAAVASSTPASGTGSL
BRUMA   YGTMVNGARAAQRFFEESFIAPASSRRKESTESP----------------
CAEEL   PPELQAQNRRPRNKLVVNVANPILSTMAVYQYCENERLISKEN-------
YEAST*  --------------------------------------------------
SCHPO   --------------------------------------------------
CANAL   --------------------------------------------------
CANGA   --------------------------------------------------
ASPNI   --------------------------------------------------
RHIO9   ---------------------------AGTNGLFKFNQGISENLGDKWNR
CRYNE   --------------------------------------------------
USTMA   --------------------------------------------------
ARATH*  ----------LKR-------------------------------------
VITVI   ----------LKRFWLQNWIGPS---------------LSGSSFFT----
SOYBN   --------------------------------------------------
SOLLC   ----------LKRFWLQNWIGPS---------------LSFTSSFMHCFD
ORYSJ*  ----------SKRMCSQACVGNSSWEG------WPFWNFSPSSYF-----
HORVU   --------------------------------------------------
SORBI   ----------SKRMCLQGSLGSSSWEG------WPFWNFSPSSYF-----
SELML   ----------LKRCWLRKQAGTH--------------------WLEDAFS
PHYPA   ----------IRRCWFQDWAGATWLED---------------------GM
                  1110      1120      1130      1140      1150
```

```
HUMAN*  ---------------YSASSGGLCEEATAAKVASWDFVEATQRTNCSCLR
CHICK   ---------------YSATSNGLCDEETADKVASWDFVEATQRTNCNCSR
XENTR   ---------------YSS-NGVVCEDESPEKTASWDFVESTQRTTCVCSR
DANRE   ---------------FSTMSNGVCEEET-DKASVWDFVESSQRSQCSCSR
CIOIN   ---------------TNNFNSNFNSSED-INETKWEFTDSFKDIRCRQ--
ANOGA   SSGD-------------------------SAADSLELADT-----CLSGS
TETNG   ---------------FPTLPNGTCEEEP-DKTGIWDFVESTHGPHCNCSR
DROME   SADGDENEQNKPPQDSKQLVQQQIQQQQ-QRQKLWNFVDPMQKAPCICTK
BRUMA   ---------------SSSGLGLLMSAAANDDDCRWNFTDGMRRKDREEGC
CAEEL   ---------------VPQKSATSGQLLPPLKEKFLNWKFTAKKYGCRGDV
YEAST*  ---------------DPSEFHCLQDALD-AIDDFIQLKQTAAYRTPGSSG
SCHPO   -----------------------------LSSNLTDLNN-----------
CANAL   -----E---------GLLESSELNNPLH-LISDFLDFRIAHNTQLQQQRD
CANGA   ---------------QINGLDTFEDTLS-LIDNFLQLKQSSSYRTPGSSG
ASPNI   ---------------QSETLDRTGESFP-FLCDPLDFAEGWCAK------
RHIO9   SSWNEK---------VANYWEFVCPKDS-VVSSVLDMLSQESTTSGNQAG
CRYNE   ---------------PPEGDGGQLDDQDPDYDNELSLGDTDADMNETDGE
USTMA   AAPDDL---------QNLQSQPMTPLKE-LVSFTLKVLNDANESSPVQNA
ARATH*  ---AGD---------FDCLGA-----SE-NKSDGFYEKNGYNSSGSSRNS
VITVI   -HWAGK---------TDSLDE-----SW-IDSNGTRTQHSYNSSSNSNDS
SOYBN   --------------------------------------------------
SOLLC   FHCDAK---------VNSVDG-----SW-LDANVIRSNRRYNSSSNSNSS
ORYSJ*  -RNSSC---------LGSSRG-----LG-VNSNFLRLRRQKNSNYSSMAS
HORVU   --------------------------------------------------
SORBI   -QNSSF---------LGSSCG-----LG-VNSNYLRLRRKKN-KCNSMAS
SELML   VRKFLP---------LTNLAGHRLQAMG-KANTIWKLNSNGSKSSTSSGS
PHYPA   NEQVPP---------LNSKNGLDGGESR-EKSTITEFGANGSVPGHSSGP
                  1160      1170      1180      1190      1200
```

MED13

```
HUMAN*  HKNLKSRN------------------------------------------
CHICK   HKNLKPRN------------------------------------------
XENTR   HKNLKQRN------------------------------------------
DANRE   LKNQKQRA------------------------------------------
CIOIN   --------------------------------------------------
ANOGA   GGGNSGAS------------------------------------------
TETNG   HKNQKQRS------------------------------------------
DROME   HLGNTPHG------------------------------------------
BRUMA   GCHHCRLQ------------------------------------------
CAEEL   SCRHCAEAEYQGESDGQVILDVDRHNRFLKYGPYTENSIRWHKIRSRIQK
YEAST*  VLSSNIAG------------------------------------------
SCHPO   --------------------------------------------------
CANAL   SLLDQQQG--------------------------ETVN------------
CANGA   GLTGTNIG------------------------------------------
ASPNI   --------------------------------------------------
RHIO9   FLQKPIHE--------------------------STTN------------
CRYNE   GLNLQETG------------------------------------------
USTMA   SAYTPGEP--------------------------DDASLAQHRVDLIPST
ARATH*  -----------------------------------SIS------------
VITVI   -----------------------------------SIS------------
SOYBN   --------------------------------------------------
SOLLC   -----------------------------------SVS------------
ORYSJ*  -----------------------------------SIS------------
HORVU   --------------------------------------------------
SORBI   -----------------------------------SIS------------
SELML   GGSS-------------------------------IKS------------
PHYPA   GTSSNSSG--------------------------GSTS------------
                       1210      1220      1230      1240      1250
```

```
HUMAN*  ----------------AGQQGQAPSLGQQQQILPKHKTNEKQEKSEKPQK
CHICK   ----------------SGQQGQAPPVGQQQQAAPKHKTNEKQDKGDKPQK
XENTR   ----------------LNQPGQPVTASTQQQVTQKHKTNDKQEKGDKPQK
DANRE   ----------------CSTPGHPPSAGQPPQPSTKHKMAEKLEKGDKQQK
CIOIN   ---------------------------LPSRPYLPLDGRHSAKLDRQQRS
ANOGA   -----------------NTTGAAPGSTTPYGGPPSYCRNPMSIGDSHPLP
TETNG   ----------------SSTSGHPPSSGQPSQPIPKHKLGEKPEKSEKQQK
DROME   ---------------------------TPHGGASTYSRNSLGGDSSMPVA
BRUMA   --------QSSSATALPNLPSQCITLQQGMGPAIFHRRSSSCTRSPSPPR
CAEEL   KFDFHAYHKKHPIGPITPRKAGDSVKQTKQLSAVKRRRRARRERAQKKRR
YEAST*  ---------------------------------------------TNPLS
SCHPO   --------------------------------------------------
CANAL   --------------------------------------------------
CANGA   ---------------------------------------------TNVFS
ASPNI   --------------------------------------------------
RHIO9   --------------------------------------------------
CRYNE   --------------------------------------------------
USTMA   RPDITPASISFP-----DFDFAMPTVASATSSQALPPAGHSVAATSPLKR
ARATH*  ----STSSASSG-----SG-------------------------------
VITVI   ----SITSSSSD-----SD-------------------------------
SOYBN   --------------------------------------------------
SOLLC   ----SISTSSSD-----SD-------------------------------
ORYSJ*  ----SVSSTSNG-----SE-------------------------------
HORVU   --------------------------------------------------
SORBI   ----SVSSTSDG-----SD-------------------------------
SELML   ----SDSRGSSG-----S--------------------------------
PHYPA   ----SSSQGSSG-----T--------------------------------
                       1260      1270      1280      1290      1300
```

```
HUMAN*  RPLTPF---HHRVSVSDDVGMDAD-SASQRLVISAPDSQVRFSN------
CHICK   RPLTPF---HHRVSISDDVAMEAD-SASQRLVMTAPDSQVRFSN------
XENTR   RPLTPF---HHRASISDDVNIEAD-SASQRLGIRSQDTRGRFSN------
DANRE   RPLTPF---HHRSSLCEEQPSLEQGESVHRLCLQGHE-DSRYPS------
CIOIN   RPNTPF---HHRTHENEAQNIETQ----SNIILPPHSRPSVLIN------
ANOGA   SEGSPASAAPSPMQPSSVSQPTSVPSGDQLITMSPHPPASNNPR------
TETNG   RPQTPF---HHRSSVSEEQPLEPQ---TQRLCLRPQE-EGSYPS------
DROME   SVESPATPAPSPHPNSAHSQPTSVPPAEQLLNMSPHAPTSVSNL------
BRUMA   SPPWEKKI-LATSSETGDSEHNLDWIRESYFRNSRSSSRSGRADL-----
CAEEL   DKKRPT---GSTRADSEDDSFRMDKLSQDDWANFEGEHPKGIPEIEVDKD
YEAST*  SDGAYTEQFQHYKNNSISSQPASYHSVQETNKISPKDFSPNFTGIDKLML
SCHPO   --------------------------------------------------
CANAL   ------VPLSAGSVHATGANSFGNVPDISKDVDTVGLSTT----------
CANGA   SEGAYTDQFQVYPKSQTNQQQTSSNS-----KVSPSDAASPYPGIDKII-
ASPNI   ----EIPKAVETKSELPSKNQERQVNSQDTSSPGPVGLPEGIESLSRASL
RHIO9   ----ASPLMVTTPMATPASTTGNQQRTDSSISSTPITHQEEGEDKSKLRN
CRYNE   ------------MGAGGDIEMEPTPKGGSDIDDLFSSSSP----------
USTMA   EPTNTEPMPGASTTEAFAQDLNWMQFLPSQINEAPASASASALADQSNLT
ARATH*  ---------WRMTSRTGD----LD--------------------------
VITVI   ---------VKMTTGAGD----PE--------------------------
SOYBN   --------------------------------------------------
SOLLC   ---------YK-TSGTGD----LE--------------------------
ORYSJ*  ---------HAVAAEGGD----LS--------------------------
HORVU   --------------------------------------------------
SORBI   ---------RAVATE-GD----LV--------------------------
SELML   ---------ESDVNDDGDYR------------------------------
PHYPA   ---------SSSSDADGDGLGEIE--------------------------
                       1310      1320      1330      1340      1350
```

MED13

```
HUMAN*  --IRTNDV--AKTPQ----------------MHGT----EMANSPQPPPL
CHICK   --IRTNDV--AKTPQ-----------MHNAEMHNA----EMANSPQPPPL
XENTR   --IRTNDV--AKTPQ----------------MHNN----EMASSPPPPPL
DANRE   --LHHADVTSSKTPM----------------LHSSAD--EMAGSPQPPPL
CIOIN   --------------------------GPTEFMGTPQTFCPVKLEPTPSPS
ANOGA   -------SVQPGTPC---------------------LDHLDKNTPAPTPT
TETNG   --LHHVDAVPSKAPT----------------LHTHGPPADLAGSPPPPPL
DROME   --------QQPPTPI---------------------DHLLDKNTPAPTPT
BRUMA   --LHEDRHICPKTPDASTIME-----------------------------
CAEEL   FELHNDIKI-PKVPN--DDNYKPKSFLPITYLTNNVDNNDFESMPGDDDM
YEAST*  SPSDQFAPAFLNTPNNNINENELFNDRKQTTVSNDLEN------------
SCHPO   --------------------------------------------------
CANAL   --------------------------------------------------
CANGA   -EQKQFTPDFMASPSVSGNSNELFNDRKNG--HTDLLI------------
ASPNI   HPEVQSANLVYPTPPDGTAGIGLNPVVPTTTKNA----------------
RHIO9   QQNLSLAEFAMANFALSDSNNGDMPEYPQQYAND----------------
CRYNE   --------------------------------------------------
USTMA   S-------------------------------------------------
ARATH*  --------------------------------------------------
VITVI   --------------------------------------------------
SOYBN   --------------------------------------------------
SOLLC   --------------------------------------------------
ORYSJ*  --------------------------------------------------
HORVU   --------------------------------------------------
SORBI   --------------------------------------------------
SELML   --------------------------------------------------
PHYPA   --------------------------------------------------
                 1360      1370      1380      1390      1400
```

```
HUMAN*  SPHPCDVVDE--G--VTKTPSTPQSQHFYQMPTPDPLVPSKPMEDRID--
CHICK   SPHPCDVVDE--G--VNKAPSTPQSQHFYQMPTPDPLVPTKTMEDRLD--
XENTR   SPHPCEVMDD--GGETMKNPSTPHSQHFYQMPTPDPLIPVKSIDERID--
DANRE   SPHPCERMEE--PADGMKSSSSPLHQHFYPPSSEPCLEPQKPPDESTLD-
CIOIN   NLGDKFYQPKANYELRNQ--------------------------------
ANOGA   DQHDSKSINA-----------SPYHTPSHPGGGGGGGGSSGGTTEGGQQQ
TETNG   SPHPCDHTDNEGTPGGMKNSSTPIHQTFYPPSVEPCILPQKGSPEEPQHG
DROME   DQHDSKSITA-SPYVHQTPSVEPPSYTDHAAGGGPAGGQGLGTGPGSVPA
BRUMA   --------------------------------------------------
CAEEL   SEERFNQIFK-DKYNRWKPGMSRAEKIEHAHYQFDPSNQMTGSPREYHLK
YEAST*  --------------------------------------------------
SCHPO   --------------------------------------------------
CANAL   --------------------------------------------------
CANGA   --------------------------------------------------
ASPNI   --------------------------------------------------
RHIO9   --------------------------------------------------
CRYNE   --------------------------------------------------
USTMA   --------------------------------------------------
ARATH*  --------------------------------------------------
VITVI   --------------------------------------------------
SOYBN   --------------------------------------------------
SOLLC   --------------------------------------------------
ORYSJ*  --------------------------------------------------
HORVU   --------------------------------------------------
SORBI   --------------------------------------------------
SELML   --------------------------------------------------
PHYPA   --------------------------------------------------
                 1410      1420      1430      1440      1450
```

```
HUMAN*  --SLSQSFPPQYQEAVEPTVYVG-------------------TAVNLEED
CHICK   --GLSQPFPAQFPEVIEPTMYIG-------------------TAVNLEED
XENTR   --GLPQTFASAFPEVLEPTMYVG-------------------NAVRLEAG
DANRE   --PLPLPCPPPYPETLEATIYVG-------------------SAINPNED
CIOIN   --------------TPQPFIPPPQEQWTNLDQNNLISTNQTYEVDDVEIK
ANOGA   QASANL--------MGNSSSSSSSNSSSNGSNSSSNSSNSL-SALKASPR
TETNG   NAPLPLPFPPSYSETLEPTLFVG-------------------SAVNPNED
DROME   QQ------------PATPTAATSAGGAGSGGPSNAIGANAV-GTISVKKL
BRUMA   --------------SPERRRVQRNSVQMFGKIGSCKPIYQNLSPTSRPRS
CAEEL   DLPKKIQKYESILPTPKGNKFFKGSICRKGLRG-----YDE-ICADQDEK
YEAST*  --------------SPLKTELEANGRSLEKVNNSVSKTGSV-DTLHNKEG
SCHPO   --------------LVLPSNVVNNKKELTEFANEEAEASDK-RKEGFTEK
CANAL   --------------TPIETDLFNLQNTEEFFKNDSIHMNLE-DQIHKKET
CANGA   --------------SPSKMDVDT-------------------DFPGSNLA
ASPNI   --------------LDPDTTMDFGPAAGLGVGSGLYDTNED-DDLFGDMN
RHIO9   --------------YSNGSIVMDNGSSIQQPQQQQMHLSPQ-VESIKLEN
CRYNE   --------------TPTPNMNVS-------------------SQPHSNKS
USTMA   --------------SSAPGAVDASTQGPGGTQNPQLDQSGT-NWLLSTGS
ARATH*  --------------ADADSLTCRQSGLTC-----------------ND--
VITVI   --------------ADADSLTCRQSGLSS-----------------NDQL
SOYBN   --------------------------------------------------
SOLLC   --------------ADADSLMCRQSGLSS-----------------LDRS
ORYSJ*  --------------ADADSMACHQSDLPS-----------------N---
HORVU   --------------------------------------------------
SORBI   --------------ADADSMPGRQSDMPS-----------------NN--
SELML   --------------EDADS-----AATLAGPNGTMISSSGR-TPD-GDNK
PHYPA   --------------ADGDSLGSKAAGVLASKHGPSTSLQGL-DMDTSDMK
                 1460      1470      1480      1490      1500
```

MED13

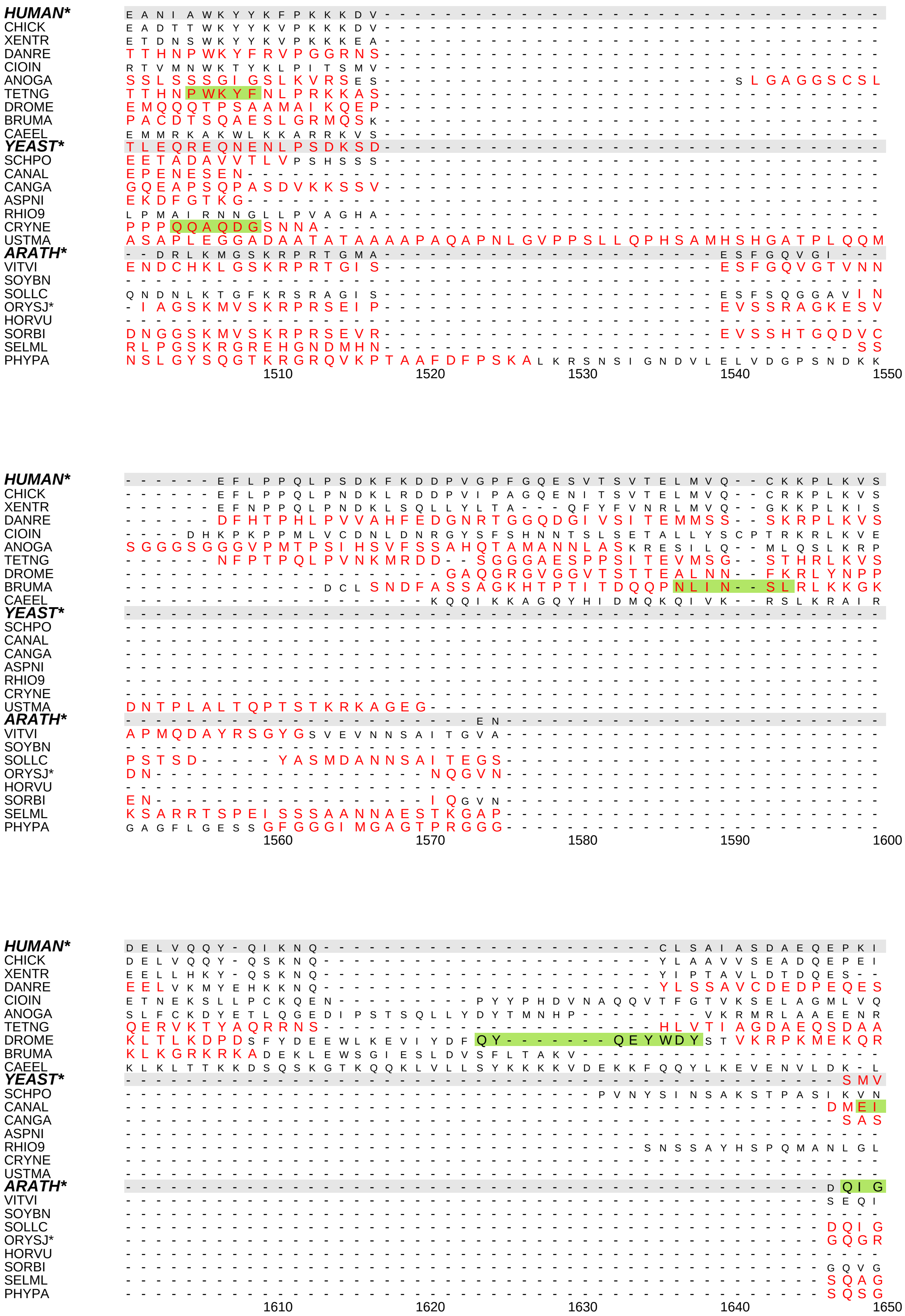

HUMAN*
CHICK
XENTR
DANRE
CIOIN
ANOGA
TETNG
DROME
BRUMA
CAEEL
YEAST*
SCHPO
CANAL
CANGA
ASPNI
RHIO9
CRYNE
USTMA
ARATH*
VITVI
SOYBN
SOLLC
ORYSJ*
HORVU
SORBI
SELML
PHYPA
1510
1520
1530
1540
1550
1560
1570
1580
1590
1600
1610
1620
1630
1640
1650

MED13

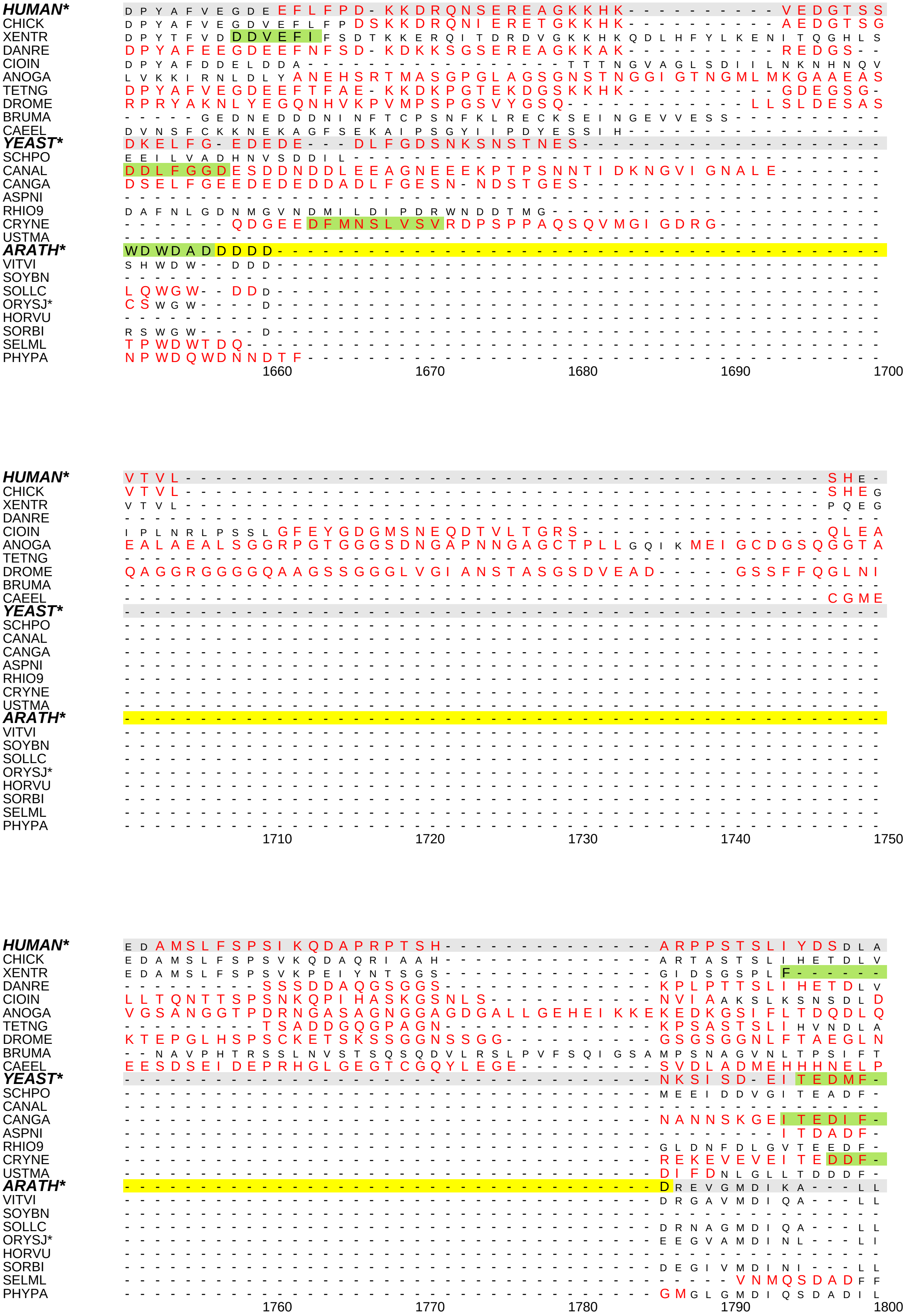

HUMAN*
CHICK
XENTR
DANRE
CIOIN
ANOGA
TETNG
DROME
BRUMA
CAEEL
YEAST*
SCHPO
CANAL
CANGA
ASPNI
RHIO9
CRYNE
USTMA
ARATH*
VITVI
SOYBN
SOLLC
ORYSJ*
HORVU
SORBI
SELML
PHYPA
1660
1670
1680
1690
1700
1710
1720
1730
1740
1750
1760
1770
1780
1790
1800

MED13

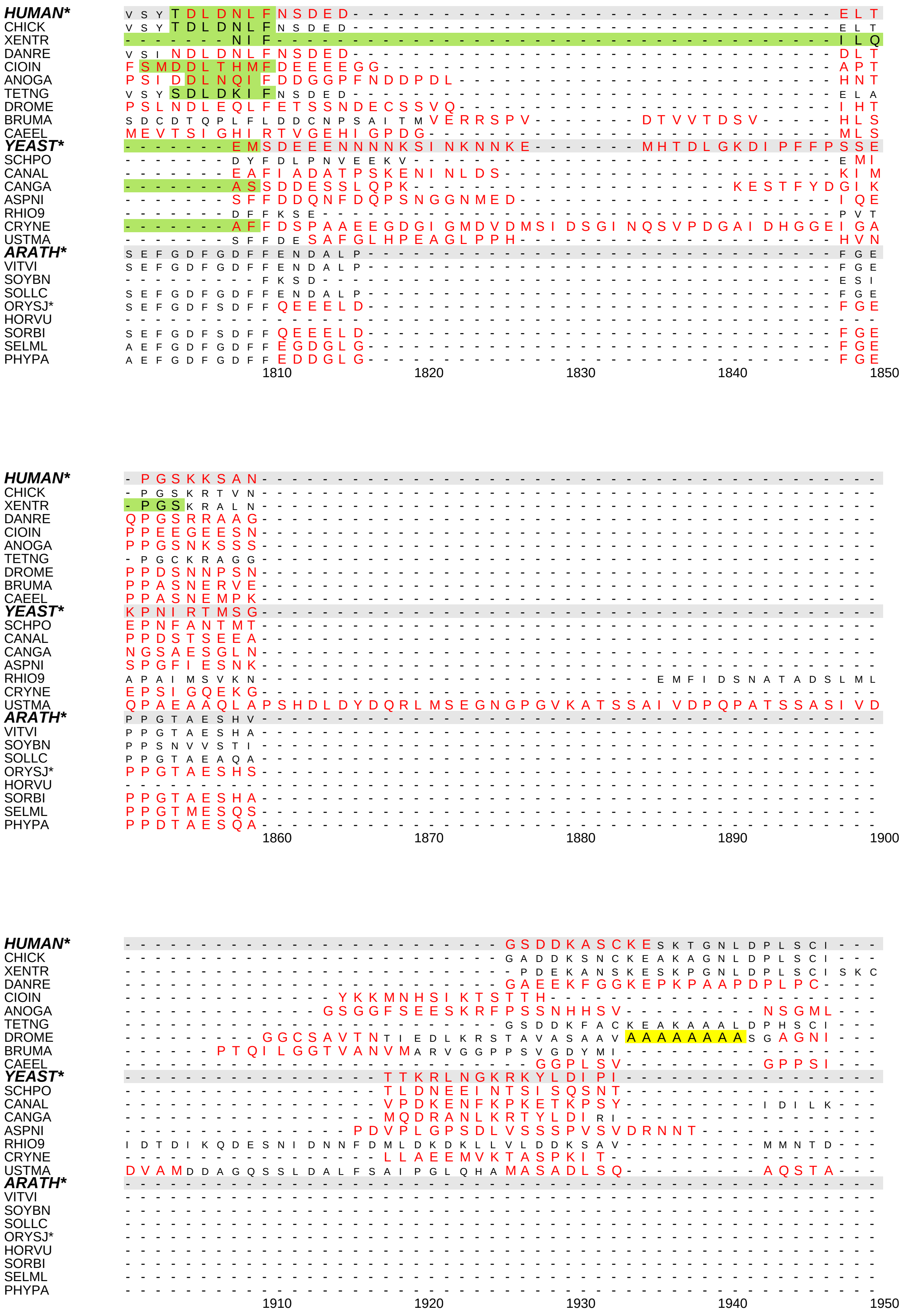

MED13

```
HUMAN*  ----------------------STADLHKM-----------------YPT
CHICK   ----------------------STADLHKM-----------------YPT
XENTR   CATHTRREPLVQPAMLGGTHAPSTADLHKM-----------------YPT
DANRE   -----------------------CADLHQM-----------------FPT
CIOIN   ----------------------AATDLQRM-----------------FPT
ANOGA   ----------------------SPKELSQM-----------------FPT
TETNG   ----------------------SSADLHQM-----------------FPT
DROME   ----------------------QAEDLTKM-----------------FPT
BRUMA   -----------------------------------------------YPT
CAEEL   ----------------------ESQGLNQI-----------------YPT
YEAST*  ----------------------DEMTLPTS--------PLYMDPGAPLPV
SCHPO   -----------------------------------------------SPN
CANAL   ----------------------DKMTIEKIVNSPDYKDPGAPLPVVPTPL
CANGA   ----------------------EETPLSS---------PLYTDPGAPLPV
ASPNI   ----------------------PKGSLEQI-----------------HPA
RHIO9   ----------------------IESTTARI--------------------
CRYNE   ----------------------EEAGQDELTLVPLVGNGMEVDGIPPEPL
USTMA   ----------------------PQATALSA-----------------APM
ARATH*  ----------------------------LV-----------------FP-
VITVI   ----------------------------LI-----------------FPA
SOYBN   -------------------------------------------GYEDFPD
SOLLC   ----------------------------LM-----------------FPA
ORYSJ*  ----------------------------LV-----------------IPG
HORVU   --------------------------------------------------
SORBI   ----------------------------LV-----------------IPA
SELML   ----------------------------YM-----------------FPF
PHYPA   ----------------------------LM-----------------FTL
                  1960      1970      1980      1990      2000
```

```
HUMAN*  -------------PPSLE----QHIMGFSPMNMNNKEYGSM---------
CHICK   -------------PPSLE----QHIMGFSPMNMNNKEYGSM---------
XENTR   -------------PPSLE----QHFMGFSPMNMNSKDYGNM---------
DANRE   -------------PPSLD----Q---GYSPINMGNT--------------
CIOIN   -------------PPSME-----QCQAFSP--------------------
ANOGA   -------------PPSLD-----PHPNSSPGGGGCLSDGGSIALDTLVDL
TETNG   -------------PPSLD----QP--GYSPMNSGTKD--GL---------
DROME   -------------PPSHE----QQHPNSSPCQT------DVVMTDLSVDT
BRUMA   -------------PPSVD---ATQSQQFSPQNTLIQANPTSILYPPTVSY
CAEEL   -------------PPSVQMLLGDAAQAHSPVMHTKSRYAMNTLEDDVERI
YEAST*  --------------------------------------------------
SCHPO   --------------------------------------------------
CANAL   VLSTVSQSAVTTNPPSVG--------------------------------
CANGA   --------------------------------------------------
ASPNI   --------------------------------------------------
RHIO9   --------------------------------------------------
CRYNE   PLPSVETAAKELQPPQLP--------------------------------
USTMA   -------------QPAQLRTGASTAPETAAATAGSMHAFHPAMNSFTPRD
ARATH*  -------------PDSAD--------------------------------
VITVI   -------------PDC----------------------------------
SOYBN   --------------------------------------------------
SOLLC   -------------ADSVD--------------------------------
ORYSJ*  -------------PDCGD--------------------------------
HORVU   --------------------------------------------------
SORBI   -------------SDGGD--------------------------------
SELML   --------------------------------------------------
PHYPA   -------------PDYGE--------------------------------
                  2010      2020      2030      2040      2050
```

```
HUMAN*  ------------------------------------DTTPGGTVLEGNSS
CHICK   ------------------------------------DTTLGGTVLEGNSS
XENTR   ------------------------------------DTTPGGSLTDGSNF
DANRE   ------------------------------------ESAAGLSLLDGA--
CIOIN   ------------------ESRIPIPNSVVAMDDTTPIIPTNEHQVIYSSE
ANOGA   QMSLSSGTGSGGGNGGGASSVPPIPN------------------------
TETNG   ------------------------------------EAGAGLTLLDGS--
DROME   TTTIITSSITTTCNTTITSSINTTTTACSNPSNSIMLAAAQAPVTVVAIQ
BRUMA   MAAAAAAAVNLCHNIPPVQLP---PTVVSETDATTETTPTIKRELENDDE
CAEEL   AVPGGV------------------GSLVEIEADDIETKNMQKWSKLSNQS
YEAST*  ------------------------------------ETP-----------
SCHPO   -------------------------------LNTHENIPKQMEIQSDDRM
CANAL   ----------------------------SAPGPAPGPAPGTETLYEANNA
CANGA   ------------------------------------ETP-----------
ASPNI   ------------------------------------PVPHGQPLSVNDAL
RHIO9   --------------------------------HDLSVTPLGVGMAQQDNI
CRYNE   --------------------IPPQPSSSPPILETSPKTPEQL--------
USTMA   ASVATPFGDPASLPGFTPSSL---TESSPTFGNPQYKTPRTPYSPIEEYR
ARATH*  --------------------------------V--GSSPV-DMMDVSDQI
VITVI   --------------------------------E--GSSPCTGMMDVSDQM
SOYBN   --------------------------------------------------
SOLLC   --------------------------------V--SSSPCPSMMDVQDQI
ORYSJ*  --------------------------------ATFTDSPS-TAMDIPEQR
HORVU   --------------------------------------------------
SORBI   --------------------------------VTFTDSPS-TAMDIPEQR
SELML   -------------------------------DGDGSNTPGTQFIETSDPM
PHYPA   ----------------------------------VSQTPGTAPLDASDPL
                  2060      2070      2080      2090      2100
```

MED13

```
HUMAN*  SIGAQFKIEVDEGFCSPKPSE-----------------------------
CHICK   SMGAQFRIEVDEGFCSPKPAE-----------------------------
XENTR   CIGAQFKIEVDEGFCSPKPSE-----------------------------
DANRE   -MSGNFKMEVEEGFCSPKPSE-----------------------------
CIOIN   KLETDKLLQDELPLLPTSYEMQ----------------------------
ANOGA   -------------LGSPQEEP-----------------------------
TETNG   LLNNHFKMEVEEGFCSPKPSD-----------------------------
DROME   TVSKMVKQEYNLELGSPMEEP-----------------------------
BRUMA   RLLGKLHYSIGEKPVYGKFMQLEMALSRKFAGNILAKEFLSLEAKECRML
CAEEL   KLSKFIALSCKDPFLKSKKKKYDTPV------------------------
YEAST*  --------------------------------------------------
SCHPO   VTEDLNPYNVEVDIPEI---------------------------------
CANAL   YAQSSQILQ-----------------------------------------
CANGA   --------------------------------------------------
ASPNI   VLSPHQDHAQTASPPLS---------------------------------
RHIO9   EFQQDQRVDPNLLHKQLLFHQQQQVDQDNLSFQRD---------------
CRYNE   --------------------------------------------------
USTMA   DGATIVDLQNDRRMRESAQASRERNATSAPHLYASSQHPVLKRGAEDQLR
ARATH*  --------------------------------------------------
VITVI   --------------------------------------------------
SOYBN   --------------------------------------------------
SOLLC   --------------------------------------------------
ORYSJ*  --------------------------------------------------
HORVU   --------------------------------------------------
SORBI   --------------------------------------------------
SELML   --------------------------------------------------
PHYPA   --------------------------------------------------
                 2110      2120      2130      2140      2150
```

```
HUMAN*  -----------------IKDFSYVYKPEN-------CQILVGCSMFAPL-
CHICK   -----------------IKDYSYVYKPEN-------CQALVGCSMFAPL-
XENTR   -----------------IKDFSYVYKPET-------CQVLVGCSMFAPL-
DANRE   -----------------IKDFSYVFKPES-------CQVLIGCSLYAPL-
CIOIN   -----------------TSACNKIKQHPT-------------PTLYQPI-
ANOGA   -----------------IDDWSFVFLPPP-------ICPYVGSSKYAPL-
TETNG   -----------------VKDFSFVYKPET-------SQLFVGCSMYAPL-
DROME   -----------------INDWDYVYRPPQ-------QEKFVGSTRYAPL-
BRUMA   EGVYADQLKLIKAAVAAREKRKKVGRAPDYLFMRERLRNLAPHSSFTTVR
CAEEL   -----------------TSKFKIENEPPS----------MSAELVYAPMV
YEAST*  -----------------RDRRKSVFAPLN----------------FNPI-
SCHPO   -----------------SLNISDSKIPTS----------AYMPSYYSAV-
CANAL   -----------------LPQQKSAFSPIL----------------FNPI-
CANGA   -----------------RDRRKSVFAPLN----------------FNPI-
ASPNI   -----------------PVEVKKILFPESSTSPKSLAKTSNALSHYSPV-
RHIO9   -----------------KPKQRELFIPPT----------------FAPVK
CRYNE   -----------------ASQSPHLTSPSIPLVPAAAPSNPLIPISFQPL-
USTMA   LADVSTAAAAAAAHAAMDMDLSNRKRPAI------------VPNAFLPLP
ARATH*  ---------------------------------------------VLPV-
VITVI   ---------------------------------------------LLSV-
SOYBN   ---------------------------------------------ITPL-
SOLLC   ---------------------------------------------LLPV-
ORYSJ*  ---------------------------------------------FSPV-
HORVU   --------------------------------------------------
SORBI   ---------------------------------------------LSPV-
SELML   ---------------------------------------------RLPI-
PHYPA   ---------------------------------------------VLPI-
                 2160      2170      2180      2190      2200
```

```
HUMAN*  --------------------KTLPSQYLPP----IKLPEECIYRQSWTVG
CHICK   --------------------KTLPSQCLPP----IKLPEECIYRQSWTVG
XENTR   --------------------RTLPSQCLPG----IKLPEDCIYRPSWTVG
DANRE   --------------------KTLPSQCLLP----VKLPEECVYRPSWTVG
CIOIN   --------------------KCLTTQLPLP---------TLSYTPAWQLP
ANOGA   --------------------PSLPSQQVMP-PIPTSTVAGLVYKSTRQKQ
TETNG   --------------------KTLPSQCLLP----IKLPEECVYTPSWSLG
DROME   --------------------TNLPSQTQPP----LTLPTGCFYQPTWSSH
BRUMA   HYRIPYNNGHMFSSHCLPTLQMLPHGMSMNGVPNFVTNGPPFGAASYNGN
CAEEL   TRNFYSAPHRTFGTLAQKVVDKRPSQFAIP--------------------
YEAST*  ------------------------IENNVD--------------------
SCHPO   --------------------IFPSSISSIF--------------------
CANAL   ------------------------IKSDID--------------------
CANGA   ------------------------IAKDVD--------------------
ASPNI   --------------------AFKSNISSWD--------------------
RHIO9   INFLVND-------------------------------------------
CRYNE   -------------------PLLPPSHSSSS--------------------
USTMA   QTETRKP----------LQRLAIGARANLG--------------------
ARATH*  ---------------------GFSSFESFN-PVPP-IIDECLIKS-----
VITVI   ---------------------GFQSFDNFN-PSPPVAMEECLTKN-----
SOYBN   --------------------------------------------------
SOLLC   ---------------------GFSSFDSFNQPPPPAILDDSLSKH-----
ORYSJ*  ---------------------GLSSLEVFN-HQTVAPIHDVVSKV-----
HORVU   --------------------------------------------------
SORBI   ---------------------GFTSLDAFD-HQIMVPAQDVVSKV-----
SELML   --------------------LDFPMLEVLG------QAEDLEKEQ-----
PHYPA   --------------------LDFPMLGKDQ------STGDLLSEM-----
                 2210      2220      2230      2240      2250
```

MED13

```
HUMAN*   KL------------------------------------------------
CHICK    KL------------------------------------------------
XENTR    KL------------------------------------------------
DANRE    KL------------------------------------------------
CIOIN    VQLPPDPSFILP--------------------------------------
ANOGA    KE------QAAAEREKQKQREQAERERERERQKQEQQQDQPDGQPESGGQG
TETNG    KM------------------------------------------------
DROME    KSRAATLAKAAAAQQQQHQKHQALQQRIQLHQQKLQQLQLQNQQQQQAAA
BRUMA    NSVMSMMMSSGCLPPSGTTIMQPVPSSSRMGVVHMNQQMSMAHLRPLARI
CAEEL    -------STSTAAHPGLTTPMASIFQSPTMPPPHTPSPFSAGPMSHHPMG
YEAST*   --------------------------------------------------
SCHPO    --------------------------------------------------
CANAL    --------------------------------------------------
CANGA    --------------------------------------------------
ASPNI    --------------------------------------------------
RHIO9    --------------------------------------------------
CRYNE    --------------------------------------------------
USTMA    --------------------------------------------------
ARATH*   --------------------------------------------------
VITVI    --------------------------------------------------
SOYBN    --------------------------------------------------
SOLLC    --------------------------------------------------
ORYSJ*   --------------------------------------------------
HORVU    --------------------------------------------------
SORBI    --------------------------------------------------
SELML    --------------------------------------------------
PHYPA    --------------------------------------------------
                  2260      2270      2280      2290      2300
```

```
HUMAN*   --------------------------------------------------
CHICK    --------------------------------------------------
XENTR    --------------------------------------------------
DANRE    --------------------------------------------------
CIOIN    --------------------------------------------------
ANOGA    GAGAGPGRPVGVAGPTTTALGVGLGLLPIVKQEVGGLTVPQPLSTPNSLL
TETNG    --------------------------------------------------
DROME    AAAAAASGGVGHQKHQHQHLHDLLSAAP--RTPLTPSTVPQPLSSGGS--
BRUMA    PSMPPYA-------------------------------------------
CAEEL    PPAYPGT-------------------------------------------
YEAST*   --------------------------------------------------
SCHPO    --------------------------------------------------
CANAL    --------------------------------------------------
CANGA    --------------------------------------------------
ASPNI    --------------------------------------------------
RHIO9    --------------------------------------------------
CRYNE    --------------------------------------------------
USTMA    --------------------------------------------------
ARATH*   -------------QEVL-------------HSSITSVPSNQM--SISS-T
VITVI    -------------QEVT-------------NNTLSSGPLNYT--PASS-I
SOYBN    --------------------------------------------------
SOLLC    -------------QEVI-------------KSAAVTNQVNSA--SASI-A
ORYSJ*   -------------QEPQ-------------KDIASPTSSQSV--VLS--S
HORVU    --------------------------------------------------
SORBI    -------------QEPH-------------KDSATPAQSHSL--VLS--S
SELML    -------------DTGT-------------VVDEKCEPESSP--PVLV-P
PHYPA    -------------KPIT-------------SMEVSQAPIGVPPSPTQQ-P
                  2310      2320      2330      2340      2350
```

```
HUMAN*   --------------------------------------------------
CHICK    --------------------------------------------------
XENTR    --------------------------------------------------
DANRE    --------------------------------------------------
CIOIN    --------------------------------------------------
ANOGA    GGLSGLNGGSLAHPYSNPPSQGAQSQPHSRPPSVNSLPGGIMGGGAGMLS
TETNG    --------------------------------------------------
DROME    ----------------------------------------QYLLNQLNCP
BRUMA    --------SSQTGPLQHPPFMGQSAQFCSPPGMMNAPNPISSFPGGPMCS
CAEEL    --------------------------PGSFPPTTPTYPGMTPRPGPNFGP
YEAST*   --------------------------------------------------
SCHPO    --------------------------------------------------
CANAL    --------------------------------------------------
CANGA    --------------------------------------------------
ASPNI    --------------------------------------------------
RHIO9    --------------------------------------------------
CRYNE    --------------------------------------------------
USTMA    --------------------------------------------------
ARATH*   GEFDHLLK------------------------------------------
VITVI    GEFDHLIK------------------------------------------
SOYBN    --------------------------------------------------
SOLLC    DEFNHLIK------------------------------------------
ORYSJ*   GRSSHLTR------------------------------------------
HORVU    --------------------------------------------------
SORBI    GRFDYITK------------------------------------------
SELML    AGLSLVAK------------------------------------------
PHYPA    LELNLRLK------------------------------------------
                  2360      2370      2380      2390      2400
```

MED13

```
HUMAN*   --------------------------------------------------
CHICK    --------------------------------------------------
XENTR    --------------------------------------------------
DANRE    --------------------------------------------------
CIOIN    -----------------------------------------------TMP
ANOGA    PSPAGTSGGGLFHPGG-----------------------------MGGIP
TETNG    --------------------------------------------------
DROME    QAPPGASMQQLMH-------------------------------------
BRUMA    MSTGPVQSSHMLSGMHGTTPSQPLGANNMHAAGPPNGQFSMQQPQQPFQQ
CAEEL    PGPVYPNSQQMMGPGGYPPQMGMINSQRHFQNQQMYQQQMVRMQQMRQMG
YEAST*   --------------------------------------------------
SCHPO    --------------------------------------------------
CANAL    --------------------------------------------------
CANGA    --------------------------------------------------
ASPNI    --------------------------------------------------
RHIO9    --------------------------------------------------
CRYNE    --------------------------------------------------
USTMA    --------------------------------------------------
ARATH*   -AEAMM--------------------------------------------
VITVI    -AEALL--------------------------------------------
SOYBN    --------------------------------------------------
SOLLC    -AEALM--------------------------------------------
ORYSJ*   -AEALL--------------------------------------------
HORVU    --------------------------------------------------
SORBI    -AEAKL--------------------------------------------
SELML    -SEAAL--------------------------------------------
PHYPA    -SEAML--------------------------------------------
                  2410      2420      2430      2440      2450
```

```
HUMAN*   ----------ELLSSGPSMPFIKEGD------GSNMDQEYGTAYTPQTHT
CHICK    ----------DLLPPGPAMPFIKDGD------GSTMDQEYGPAYTPQTHT
XENTR    ----------ELIPPVPSMPFIKDGKMNIPSVGSAMDQDYVQTYTPQTHT
DANRE    ----------EMLQPMAAMTFLNKDS-NIPSVGSAVEQE--QSCTPQTHN
CIOIN    INEYINNAPVTPATPGVSFTP-----------------------------
ANOGA    VPLRQGMSPISPATPGGSMRV-----------PTPQGSCPLPYPSPLGGA
TETNG    ----------ELIPPVTNVSLLAKDN-NIPSV----EPDYSQTYTPQTHT
DROME    ---RAGMSPISPGPGMGPYAA-----------------------------
BRUMA    LSHFVNNANFNGQVGMTAVNP----SQQSIYQQQIMFRCNMNGNLPAQHS
CAEEL    GPPGYGAPGVSPYQQVNAITS------------------PAGFGMPPNGP
YEAST*   ----------NKYKSGGKFSF-----------------------------
SCHPO    ----------QKYNYGGKYWC-----------------------------
CANAL    ----------TKYGKGGKFYV---------------------AKDSSTNG
CANGA    ----------NKYKNGGKYSF-----------------------------
ASPNI    ----------QKYGADGKFSF------MGVRTAFNHDPHTSSDGIPTIGL
RHIO9    ----------SKYFNGGKFTY------------------------PSIDQ
CRYNE    ----------YPYCSTYGFAP------------------PSPASTPESSL
USTMA    ----------RKYDLLGKFASRPKPAT----------ITNSASNANNVPL
ARATH*   ----------TFAPEYGAVEA-P-MSE----------ISSTFFKSPYLPK
VITVI    ----------TFAPEYGAVET-P-TSE----------SSSSIFRSPYLPK
SOYBN    ------------------LEV-----------------------------
SOLLC    ----------SFAPEYGAVET-P-TGE----------SSHSIFRNPYVPK
ORYSJ*   ----------TYAPEYAAIEI-S-TGE----------TPTSLFTNPYQPR
HORVU    --------------------------------------------------
SORBI    ----------TFAPEYAAVEI-S-IAE----------APTPLFTNPYFPR
SELML    ----------IFASEYAPIDI-----T----------GLDKLDRKPAYKP
PHYPA    ----------CFASGFEPVDM-TGVPD----------VGVSTLRDPYVPA
                  2460      2470      2480      2490      2500
```

```
HUMAN*   SFGMPPSSAPPSNSGAGILPSPSTPRF--PTPRTPRTPRTPR--------
CHICK    PFGMPPGSAPPSNGGAGILPSPSTPRF--PTPRTPRTPRTPR--------
XENTR    PFGM-SNSAPPSNGGAGILPSPATPRFSAPTPRTPRTPRTPR--------
DANRE    TF--MSNSAPPSNSGAGILPSPATPRISAPTPRTPRTPRTPR--------
CIOIN    ----GTPSRSSVRTPASVQPTPSPVTFSVPTPRTPGRSGIRS--------
ANOGA    GSPNPMGSLSFRRTTPLLPPPPYDVAIASPANSVATPSSYHS--------
TETNG    PF--MSSSAPPSNSGTGILPSPATPRFSVPTPRTPRTPRTPR--------
DROME    ------RSSPMSRATPTHPPPPYPYDLAVASPATSTSSYLNR--------
BRUMA    IHSSDSYGIDSQGQAPVEPPPPFSQSINQQYA------------------
CAEEL    SSVQRLNNFVNQSQFGPNNGHPTPPYGYMQQMIPQSPNFNQQYNGMGNQL
YEAST*   ------------------SPLQKEEALNFDISMADLSSSEEE--------
SCHPO    -----------------PSPSLSTEDLFESFSVAESVTSTDEDICSTNFI
CANAL    NLDMKNRSLRATSVSGMEIPFSSEDKKRLQQNLEVLDSSTTSDNEGGEDI
CANGA    ------------------SPLQKEEALNFDVSRTELSSSEGE--------
ASPNI    PRRKSHMKALAYSKKADAEGSPVDEVDSASSP----DSESESDDGCDNTG
RHIO9    DSNVESRLQKSNYYRPDYIPISRSKRLRKRKALKPAKALKED-----KDV
CRYNE    LARLTSYQTQYHSRPGSDAPSAS------------------QGQGATRRL
USTMA    PERRLSGDASRAGGRADKEGAAESAQFDKQ------SLTSHSRPRATRRL
ARATH*   SHKVESSNSRTSNYVYGATPPTT---DSDGAG----DMIL----------
VITVI    SRKVESSNSSAKDYVYGATPPSSPCFDGSDEK----PGMP----------
SOYBN    --------------------------------------------------
SOLLC    SREVETANSSSNSYFYSATPPLSPCFDACEEK----SSVT----------
ORYSJ*   SIKPGSSSFNSR--VYSYDAAQSSQMESGEDK----PEKS----------
HORVU    --------------------------------------------------
SORBI    SKKPGSSSF--R--VYSYDIAQSSQIESTGDK----PDKP----------
SELML    DARKETSASQSASYVYGATPPPTPKLDSYERK----AHSRWR------TE
PHYPA    SKRKAVESVQKETYQYSATPVSYPSIERKDVG----KDEQ----------
                  2510      2520      2530      2540      2550
```

MED13

```
HUMAN*  --------------------------------------------GAGGPA
CHICK   --------------------------------------------GAGGPA
XENTR   --------------------------------------------GGGGPA
DANRE   -----------------------------------------------GPA
CIOIN   --------------VDPLESPASSFATPNSHLTSKGMYTDQHGNA-----
ANOGA   ------------------------------------KQYPLDGVDSGGPG
TETNG   -----------------------------------------------GPS
DROME   ------------------------------------PLHSQEHPHMHGLG
BRUMA   ----AVHNVQGGSMIYPQSAHMMRPNTLMSQAQNQNMMC--------GPR
CAEEL   MSPIHQHQFHQQQMVQQQQQQQMQMHRQQMQMQNQNMSQPFQPPTAQMLQ
YEAST*  -------------------EDEEENGSSDEDLKSLNVRDDMKPSDNISTN
SCHPO   QQDFTMEYNHDFFSSSKTPTNISEQSNPDSNYDTLSLAHQVLMNESK---
CANAL   EDDDNDYEDEGDDDEEEEGEEEEEEESDEDEISDIGKSTPLKLNTQNESV
CANGA   -----------------------DSESSDDELEDLANKNDGVVYDK----
ASPNI   PENTMPYMTLTTLKRKRAVSSPINSPKPSSVMSSIGAEQEVNMPRSEDS-
RHIO9   TESNNAAEGQFVQKVESTTKLDSSHSSKHGSVVNS---------------
CRYNE   SSKPKSTYADAWFSLSGGDDDGDGEDGDEDDMDSQTYTCP----------
USTMA   PSRTPSGRGLALLQLHRDRQTKASPGSVLTALRHPSSSRTLEAPATPRS-
ARATH*  ---FGSKSCIGNNAGRTLYHSREHYTQVEG--RKGRHDKLPTVISDNSST
VITVI   ---VNSKTCPVRHEASSILHSKKYYTHVEG--GKEQHEKRSFTCDNSIAS
SOYBN   --------------------------------------------------
SOLLC   ---VNLKTGTGRHDTSSIVQSKKYYTHIES--GKEKNDDKVSVYVRSCAT
ORYSJ*  ---VRLASGNLSRD----IGSSNLYTVVQV--GKKESDKGLKNTDIQSGK
HORVU   --------------------------------------------------
SORBI   ---SKLTSGIHLHD----VGSSNLYTLVQG--GKKESDKSLKSTDIQPSK
SELML   FEERKNVAVNNFGNLKYGETSPTDNTLVAKTGNSHRIQGPL---------
PHYPA   ---GSTKSPNDFGDSKKKKELTADYIVVTGSHGSKHVNGKLPTTDDKGPD
                 2560      2570      2580      2590      2600
```

```
HUMAN*  SAQGSVKYENSDLYSPASTPSTCRPLN---------SVEPATVPSI----
CHICK   SAQGSVKYENSDLYSPASTPSTCRPLN---------SVEPATVPSI----
XENTR   SVQGSVKYDNSDLYSPASTPSTCRPLN---------SVEPATVPSI----
DANRE   SVQGSLKYENSDLNSPASTPSTCRPLS---------SVEPATVPSI----
CIOIN   --------------------------------------------------
ANOGA   SNRGPMTPSSTGGSGPLGAGTVHPPLS--SSGVGCGTGTPAGSRSI----
TETNG   SVQGSLKYDNSDLYSPASTPSTCRPLS---------SVEPATVPSI----
DROME   GGATGVVGHGTGGGGHMGMVAYTGDAGIVSGGTAMAAGSSSLLQEL----
BRUMA   TSQNPMMTVSSSIGSPNNGGLFGNRLMNMPHVMDTTTVPPNNIRTH----
CAEEL   MQQNEQMRIAFSTLSERDKAKFQKRQGQLRSGIMPKSLQPAVLKNLKNPP
YEAST*  TNIHE---------------------------------------------
SCHPO   --------------------------------------------------
CANAL   P-------------------------------------------------
CANGA   --------------------------------------------------
ASPNI   --------------------------------------------------
RHIO9   --------------------------------------------------
CRYNE   --------------------------------------------------
USTMA   --------------------------------------------------
ARATH*  KEG-VS--------------------QSIHSKHSAANAVKVVQGKK----
VITVI   GEG-LT--------------------PSSFSGFNSTNATKPVQRKT----
SOYBN   --------------------------------------------------
SOLLC   RETQVA--------------------ESPFSGFNSTNSVKYIHNKT----
ORYSJ*  EEA-----------------------SRPISGETSLDSSVVSQRK-----
HORVU   --------------------------------------------------
SORBI   GET-----------------------SPPISGVTSFSSSLVSQKK-----
SELML   --------------------------------------------------
PHYPA   RAAGMTLLSNKQPADISGGGGNGTQIPGNSLAAHSPSSLKHKDGKK----
                 2610      2620      2630      2640      2650
```

```
HUMAN*  --------------------PEAHSLYVNLILSESVMNLFKDCNFDSCCI
CHICK   --------------------PEAHSLYVNLILSESVMNLFKDCNFDSCCI
XENTR   --------------------PEAHSLYVNLILSESVMNLFKDCNFDSCCI
DANRE   --------------------PEAHSLYVTLILSESVMNLFKDCNFDSCCI
CIOIN   ------------------------AIFMNLLLSDSVLNLFRDRSFDQCAL
ANOGA   ---------------------ETNALLVNVLLYDTALNIFRDHNFKSCTL
TETNG   --------------------PEAHSLYVTLILSESVMNLFKDCNFDSCCV
DROME   --------------------PEVNSVLVNILLYDTALNVFRDHNFDSSSV
BRUMA   --------------------PEGKSLVLAVLLQDTVLDLHYDSVFDACPI
CAEEL   DANIPYMKQDALFRPPESNSLQGESLTIAIVLSDTLLDLHFDSVFDACPI
YEAST*  --------------------------------------------------
SCHPO   --------------------------------------------------
CANAL   --------------------------------------------------
CANGA   --------------------------------------------------
ASPNI   ------------------------------IFIGNFLSIFSDLSIAGFFT
RHIO9   --------------------------------------------------
CRYNE   --------------------------------------------------
USTMA   --------------------------------------------------
ARATH*  -------------------TDGISAVVSTLLSSKTLLATDVGSVMFQAFM
VITVI   -------------------TEGTIGMEHLVLPMKTVLATEVECIMFQASM
SOYBN   --------------------------------------------MPEFIL
SOLLC   ----------------DKASEGLLKAGSSGQSIKPVLATEVECLMCQAFM
ORYSJ*  -------------------SDSMFNAGYFLLSMKTALATEMECIKFQAAM
HORVU   --------------------------------------------------
SORBI   -------------------SDSTFNAGYFLLSMKTALATEIECIMFQAAM
SELML   ---------------------------------AATLATELECAMFQLQL
PHYPA   ---------------------------SPTRSSVAVLATELECALLQVMI
                 2660      2670      2680      2690      2700
```

MED13

```
HUMAN*  CVCNM------NIKGADVGVYIPDP-------------------------
CHICK   CVCNM------NIKGADVGVYIPDP-------------------------
XENTR   CVCNM------NIKGADVGVYIPDP-------------------------
DANRE   CVCNM------NITGADVGVYISDP-------------------------
CIOIN   CVCNS------NINGADIEMGCLPALA-----------------------
ANOGA   CVCNAGAKCVGNIRGADSGLYLALPGTNWMDSVTAAGAEPTPSTTPGPAG
TETNG   CVCNM------NIRGADVGVYLKD--------------------------
DROME   CVCNADTQKIGNIRGADSGVYVPLPGVSF---------NPFPSGAGGALA
BRUMA   CSCNG------SIRAKELGVYITPPEVLR---------------------
CAEEL   CSCSV------SIRSRDIGMYITPHTVLY---------------------
YEAST*  --------------------------------------------------
SCHPO   --------------------------------------------------
CANAL   --------------------------------------------------
CANGA   --------------------------------------------------
ASPNI   L-------------------------------------------------
RHIO9   --------------------------------------------------
CRYNE   --------------------------------------------------
USTMA   --------------------------------------------------
ARATH*  CRMRH------IITSSKH--------------------------------
VITVI   CKIRH------TLLSSSS--------------------------------
SOYBN   CNVFT---------------------------------------------
SOLLC   CKIRH------TLLSSSG--------------------------------
ORYSJ*  CRIRH------TLLSLSS--------------------------------
HORVU   --------------------------------------------------
SORBI   CRIRH------TLLSLRS--------------------------------
SELML   HSVKG------FAYCAHP--------------------------------
PHYPA   CRAQD------SSSSNSG--------------------------------
                  2710      2720      2730      2740      2750
```

```
HUMAN*  --------------------------------------------------
CHICK   --------------------------------------------------
XENTR   --------------------------------------------------
DANRE   --------------------------------------------------
CIOIN   --------------------------------------------------
ANOGA   GRSKASALAHLGLGGSS------------SAFGAGMLGSPAVGRQEDKSA
TETNG   --------------------------------------------------
DROME   GQRMLNGPSSAGFGGMRMISAFGGSPASASMPGAGSGHGHGPNGGSNSSS
BRUMA   ----------------------------------------------QPPA
CAEEL   -------------------------------------------STERGHT
YEAST*  --------------------------------------------------
SCHPO   --------------------------------------------------
CANAL   --------------------------------------------------
CANGA   --------------------------------------------------
ASPNI   --------------------------------------------------
RHIO9   --------------------------------------------------
CRYNE   --------------------------------------------------
USTMA   --------------------------------------------------
ARATH*  --------------------------------------------------
VITVI   --------------------------------------------------
SOYBN   --------------------------------------------------
SOLLC   --------------------------------------------------
ORYSJ*  --------------------------------------------------
HORVU   --------------------------------------------------
SORBI   --------------------------------------------------
SELML   --------------------------------------------------
PHYPA   --------------------------------------------------
                  2760      2770      2780      2790      2800
```

```
HUMAN*  -------------TQEAQY----RCTCGFSAVMNRKFGNNSGLFLEDELD
CHICK   -------------TQEAQY----RCTCGFSAVMNRKFGNSSGLFLEDELD
XENTR   -------------TQEVQY----RCTCGFSAVMNRKFGNNSGLFLEDELD
DANRE   -------------NMDSQYSSMDPCSCGFSAVMNRRYGNGAGLFLEDELD
CIOIN   -------------DQEAQF----RCNCGFSAVRNRAASVGNGLFLEDELE
ANOGA   AVTINTDSLQNGYLDEDPI----DCQCGFSAVVNRRMAHRAGLFYEDEME
TETNG   -------------NGEAQY----PCTCGFSAVANRRFGQSAGLFLEDELD
DROME   CTPPSSNPHITGYVDDDPV----ECTCGFSAVVNRRLSHRAGLFYEDEVE
BRUMA   QQQLIVSKPTSGFYNSTSN----SCNCGFSAVRHRYLSMKAGLFAEDAKE
CAEEL   NMREHTTGPWSGFNVNSAT----NCTCGFSAIRHRYLSCCSGLFEEDADE
YEAST*  -----------------------PQYINYSSIPSLQDSI----IKQENFN
SCHPO   -----------------------SANFDFSFLKSLDLQPTITLGKNDLLN
CANAL   -----------------------PQQSNYNPVNITDLGSNTTNNITDKQG
CANGA   -----------------------AENIGYKTFANLQDSVPPGLIKQDFLG
ASPNI   -----------------------SENHIFPSLASREIQVQVAQLLVDQV-
RHIO9   -----------------------NMTGNSSSVTNCSSSSSSGSSSDYDSS
CRYNE   -----------------------PTPMSQMEEMTTEIGSDAGSSDVDGVT
USTMA   -----------------------SDDLARSGAVSASESDASSSDGNDDSD
ARATH*  -------------------------------SSPVSLTRLSGNFFLNQLS
VITVI   -------------------------------PPSIGLSRLTGSTVLNSLP
SOYBN   --------------------------------------------------
SOLLC   -------------------------------CLPVGMSRMSGSTNRNQSQ
ORYSJ*  -------------------------------KASAELKSALSSLVHTDVS
HORVU   --------------------------------------------------
SORBI   -------------------------------KASAEFNSATSSFMQTNFC
SELML   -------------------------------NEHAKLTESSHKECTSEIT
PHYPA   -----------------------NSGAASPGNEAARYSPLPSKDSGSEIT
                  2810      2820      2830      2840      2850
```

MED13

```
HUMAN*   IIGRNTDCG--------------------KEAEKRFEALRATSAEHVNGG
CHICK    ILGRNTECG--------------------KEAEKRFEALRATSVEHGSAG
XENTR    IIGRHTDSG--------------------KEVEKRFEALRAAGGDHGV--
DANRE    IMGRGSDAA--------------------REIEKHFEAVRAASLKRGTVL
CIOIN    VVGAKFENE-----------------IIHARCRTSNYLPSDGETKRNSLG
ANOGA    ITGMAEDPAVHKNGSLFEFLNGTAKAIKLEDADRGALATSAGGSSSSAAG
TETNG    VVGRGSDSG--------------------RDTERCFEELRASSVQKAGSL
DROME    ITGIADDPGRNKQPTLLSIIQSLSRK---NQNKQGPGETSSALDKIGAGG
BRUMA    ATDINETQSQPAIPHTIWFDSMSGRDMNFI--------------------
CAEEL    ATASEHAVA------------------------PVIPPLDYPKKSTARDM
YEAST*   SVNDANITS-----------NKEGFNSIWKIPQNDIPQTESPLKTVDSSI
SCHPO    AILSQN--------------------------------------------
CANAL    YENTEGFGT------------------------PFPNQISKYSIKPESPF
CANGA    NPYLASLEG-----------SKEGQNTIWKMPQTDIAQTESPLKAIDTSI
ASPNI    --------------------------------------------------
RHIO9    SCYESDYSD--------------------------------DDDMTNKPG
CRYNE    GTDIDSGAT-----------------------------------------
USTMA    DARSDTEAT-------ALTLSPADQATLKASSLEVISTYLCGATSLSKPP
ARATH*   ----NEPST-------------------------LTDNISARNEIYKKD-
VITVI    ----GEPST-------------------------MTENISGKYEVKKKES
SOYBN    --------------------------------------------------
SOLLC    ----GEAVV-------------------------TVDNMSSKSEM-KKEI
ORYSJ*   ----NK------------------------------LDLVPKYDIKRKEN
HORVU    --------------------------------------------------
SORBI    ----NK------------------------------SDLTPKYDMRKKEI
SELML    ---------------------------------DSSDACDSLRRKPDRKK
PHYPA    QVQSDTLAQ-------------------------STSGLGKPHQE-RQKK
                  2860      2870      2880      2890      2900

HUMAN*   LKESEKLSDDLIL--------------------LLQDQCTNLFSPFGAAD
CHICK    LKEPEKLPDELIL--------------------LLQDQCTNLFSPFGAAD
XENTR    LRDPEKMPDELIL--------------------LLHDQCTNLFSPFGTLD
DANRE    K---EQVPDDLML--------------------LLQDLCTNPFSPIIRPD
CIOIN    KPIDKAENVEE----------------------LLQLAMEYSASPFTTLE
ANOGA    ---GDSLPLTLKVMD------------------LLREQCSVVQSSSNSIH
TETNG    V---EKPDNLIL---------------------LLQDQCTNPFAPMAGVE
DROME    LPNGQLEQLGHAVFD------------------LLLDQCSIIQTSSSSVH
BRUMA    ---------------------------------ALLREQTLVRDLGGLLD
CAEEL    SWFDSKSVHDLALLD-------------------QIRQMAFSNSLGKAVS
YEAST*   QP--IESNIKMTLED------------------NNVTSNPSEFTPNMVNS
SCHPO    --------------------------------------------------
CANAL    ASNELQSSVSPMYFD------------------TSQSHQSPQLQPSTGTL
CANGA    GPDGTLPNTTSSEQS------------------KSVYVHDYGISPTKMSN
ASPNI    --------------------------------------------------
RHIO9    RVLDHVSSAQDSYID------------------KLIFASPFQPSPKQSVD
CRYNE    GLMGVKDDDGEIKED----------------------DGEIEWKGTKCIG
USTMA    WSPAVSGPIDEASDD------------------VSLPRQSVQPPGLHLCT
ARATH*   IPTRIAGDFDGGMLD-SHMSAPVGVWRTVSVPKTAKPASSPNIEAGSSLP
VITVI    IPVRIAGDFDGGMLD-GPLNATVGVWRTVGVAKGAKPTNSPGVEVSSSLP
SOYBN    --------------------------------------------------
SOLLC    IPVRIAGDIDGGLLD-GTLNAPVGVWRTVGVSKGTKQPTT-GLESCHSVQ
ORYSJ*   IPARLSIDVDHEVYD-RSL-ENVGVWKPVGTPKGPTHLES--------FS
HORVU    --------------------------------------------------
SORBI    MTVRLSSDVDHEMFD-RSLMDNVGVWRPVVTPKGPKSLES--------LS
SELML    VPERIAGDTDDLSHD-GPRPVEVGVWRPVGLPKPQQRPPDGGRGVVSNIA
PHYPA    VPERIAGFADDDMQDGGSRVAQVGVWRPVGVPNPLRSSNTISSKYANAVD
                  2910      2920      2930      2940      2950

HUMAN*   ----------------------------------------------QDPF
CHICK    ----------------------------------------------QDPI
XENTR    ----------------------------------------------QEPI
DANRE    --------------------------------------------------
CIOIN    LCKDLDSNNFTAFDEDNMVVK-----------------------------
ANOGA    RAVNRYRGPLFGRGAENGAVH-----------------------------
TETNG    -----------------------------------------------H-S
DROME    RALQSHRRRMSRQR----------------------------RIFGNNGA
BRUMA    --------QVTLLSLQCERASQTERIG------------------SDSSK
CAEEL    --------HMATIKEHNRNLAIAVDLG------------------NDVTV
YEAST*   --------EISNLPKDKSGIPEFTPADPNLSFESSSSLPFLLRHMPLASI
SCHPO    --------------------------------------------------
CANAL    -----------------EVLKASSLESPSKISESSNYLPLILRSINVSTI
CANGA    --------DPAFDTAKIEGKYEFMNNFP-------SSFPFLLRHMPLSLI
ASPNI    --------------------------------------------------
RHIO9    KTMFEYDSPFADAVADTYSKSNRQITIKNMEDY-----------------
CRYNE    SEWVILRDHPRLVQPKGEAENEIGWKVESVWDEAWGTLPSAVSGKKDSTK
USTMA    VPRDSYTDSSAIQRRPTMRRSTLSRTAEWLIQN-----------------
ARATH*   --------H--SSFSEDSLLSYGQRQP-----------------------
VITVI    --------H--NSFNEEGMLSYGQRQP-----------------------
SOYBN    --------------------------------------------------
SOLLC    --------H--NSFIEDSMLAYGLRQP-----------------------
ORYSJ*   --------A--KTYTGTSQGLPVKRQP-----------------------
HORVU    --------------------------------------------------
SORBI    --------A--NTLAGASPSLSIQRQP-----------------------
SELML    --------S--PEVGTPSEISDAA--------------------------
PHYPA    LPVGVSNTSPIDPTMDDVMGSAERQCQ-----------------------
                  2960      2970      2980      2990      3000
```

MED13

```
HUMAN*   P K S G V I S N W V R V E E R D C C N D C Y L A L E H G R Q F M D N M S G G K - - - - - - - - - - -
CHICK    P K V G A V S N L V R V E E R D C C N D C Y L A L E H G R Q F M D N M S G G K - - - - - - - - - - -
XENTR    T K T S T V S Y S A R V E E Q D C C N D C Y L A L E H G R Q F M D N M S G G K - - - - - - - - - - -
DANRE    L L G T V I K S P I R L E E R D Y Y S D C Y M A L E H G R Q F M D N M S G G K - - - - - - - - - - -
CIOIN    - - - - - - - - - - - - - - H D I C E A C A L A L C G S L L A M E H P T K L D - - - - - - - - - - -
ANOGA    - - - - - - - - - - L L E Y V D A N D V I S L A L E Q G R L Q A I N E S K M D - - - - - - - - - - -
TETNG    K T N A S P T S F L R V E E R D C Y N D C Y M A L E H G R Q F M D N M S G G K - - - - - - - - - - -
DROME    P T A S L A S I A N V L E F M D A H D V I S L A L E Q S R L A F E N - Q R M D N M M D F H G N G S S
BRUMA    H C E Y I I S E V D Q R E L P L V F Q A A C E V A C M E M N C R R P P H D V R - - - - - - - - - - -
CAEEL    P S E Y I L S H V D A L E L L M L G N S V L G P M K N T S I A N Q V A S H N K - - - - - - - - - - -
YEAST*   P D I F I T P T P V V T - - - - - - - - - - - - - - - - - - - - - - - - - - - - - - - - - - - - - -
SCHPO    - - - - - - - - - - - - - - - - - - - - - - - - - - - - - - - - - - - - - - - - - - - - - - - - - -
CANAL    P S L Y L M N N L I S S K L L P S F T I S D D D L E N D L - - - - - - - - - - - - - - - - - - - - -
CANGA    P D T F K H L N P T I T - - - - - - - - - - - - - - - - - - - - - - - - - - - - - - - - - - - - - -
ASPNI    - - - - - - - - - - - - - - - - - - - - - - - - - - - - - - - - - - - - - - - - - - - - - - - - - -
RHIO9    - - - - - - - - - - - - - - - - - - - - - - - - - - - - - - - - - - - - - - - - - - - - - - - - - -
CRYNE    A K E N L K E K K V K - - - - - - - - - - - - - - - - - - - - - - - - - - - - - - - - - - - - - - -
USTMA    - - - - - - - - - - - - - - - - - - - - - - - - - - - - - - - - - - - - - - - - - - - - - - - - - -
ARATH*   - - - - - - - - - - - - - L Q D L L D G I A L L V Q Q A T - - - - - - S F V D L A L D S D C G D G P
VITVI    - - - - - - - - - - - - - L Q E L L D G M A I I V Q Q A T - - - - - - S F V D X A L D A D C G D G P
SOYBN    - - - - - - - - - - - - - - - - - - - - - - - - - - - - - - - - - - - - - - - - - - - - - - - - - -
SOLLC    - - - - - - - - - - - - - L Q E L L D G M A L L V Q Q A T - - - - - - S F V D V A L D A D N N D G S
ORYSJ*   - - - - - - - - - - - - - I V D L L S A M A L I V Q Q S T - - - - - - S F V D I A L D M D D G D G S
HORVU    - - - - - - - - - - - - - - - - - - - - - - - - - - - - - - - - - - - - - - - - - - - - - - - - - -
SORBI    - - - - - - - - - - - - - V V D L L C A M A L L V Q Q S T - - - - - - S F V D M S L D M D D G D G S
SELML    - - - - - - - - - - - - - M Q D I V D L I P L L V Q Q A A - - - - - - C C N D I S L N G E H G D G A
PHYPA    - - - - - - - - - - - - - W Q E I L E A V P L L A Q Q A S - - - - - - T A V D V S L D G Q Y E D G P
                             3010                3020                3030                3040                3050
```

```
HUMAN*   - - - - - - - - - - - - - - - - - - - - - V D E A L V K S S C L H P W S K R N - - D V S M Q C S Q D
CHICK    - - - - - - - - - - - - - - - - - - - - - V D E A L V K T T C L H H W S K R N V V D V S M Q C S Q D
XENTR    - - - - - - - - - - - - - - - - - - - - - V D E A L V K S T C L H R W S K R N A V D A S M Q C S Q D
DANRE    - - - - - - - - - - - - - - - - - - - - - V D E T L V K S S S L H H W A K R N A F D M S M L F S Q D
CIOIN    - - - - - - - - - - - - - - - - - - - - - - - - A D S I I N P I H Q W K D T R S E V Y E F A G G F D
ANOGA    V D Q Q Q Q Q Q S S S G A L V K - - - - - - G G T L R G Q M N V H K W P F L R A - - G G P K S N Q D
TETNG    - - - - - - - - - - - - - - - - - - - - - V D E T L V K S T C L H R W P K C K Y - - M S K L F S Q D
DROME    S S H Q Q Q Q L T A F H A P P P A L R H K L A G I G A G R L T V H K W P Y L P V - - G F T R S N K E
BRUMA    - - - - - - - - - - - - - - - - - - - - - - - - - - - - S V L L H D W G V Q I S N E M R E P R E S E
CAEEL    - - - - - - - - - - - - - - - - - - - - - - - - - - - F L G F F H P W G I Q I A N G M T E L E A S E
YEAST*   - - - - - - - - - - - - - - - - - - - - - - - - - - - - - - - - - - - - - - - - - - - - - - - - - -
SCHPO    - - - - - - - - - - - - - - - - - - - - - - - - - - - - - L W F R S L P F W K S M T T S F M M S Q D
CANAL    - - - - - - - - - - - - - - - - - - - - - - - - - - - - - - - - - - - - - - - - - - - D I T K S N E
CANGA    - - - - - - - - - - - - - - - - - - - - - - - - - - - - - - - - - - - - - - - - - - - - - - - - - -
ASPNI    - - - - - - - - - - - - - - - - - - - - - - - - - - - - - - - - - - - - - - - - - - - - - - - - - -
RHIO9    - - - - - - - - - - - K T L E Y L C Q Q V V L G G Y P F S G G F E S V S S N G I E A N E G E S T K A
CRYNE    - - - - - - - - - - - - - - - - - - - - - - - - - - - - - - - - - - - - - - - - D V N W T K V V Q E
USTMA    - - - - - - - - - - - - - - - - - - - - - - - - - - P Q F R S M Y G F G M A S I T T G T D V A V G D
ARATH*   Y G W L A L E E L W R R E L S C G P S A G H A G C G G T L A S C H S L D I A G V K L V D P L S A E V
VITVI    Y G W L A L Q E Q W R R G F S C G P S M V H A G C G G I L A S C H S L D I A G M E L V D P L S A D V
SOYBN    - - - - - - - - - - - - - - - - - - - - - - - - - - - - - - - - - - - - - - - - - - - - - - - - - -
SOLLC    Y G W L A L Q E Q W R R G F S C R P S M V H A G C G G V L A S C H S L D I A G V E L I D P L S A D V
ORYSJ*   F F W L S L D E Q K R R G F S C D P S M V H A G C G G I L G T C H S K D C A G V D L V D P L S A E V
HORVU    - - - - - - - - - - - - - - - - - - - - - - - - - - - - - - - - - - - - - - - - - - - - - - - - - -
SORBI    F F W L S L D E Q K R R G F S C D P S M V H A G C G G L L G T C H S K D C A G V D L V D P L S A E V
SELML    L A W L T L Q Q K L S Q Q D L S W T S - - - - - - - - - - - - - - - - - - - - - D V C D P S I T E I
PHYPA    F G W L A A Q E V Q R Q Q R K R R S D C S Q A G G G G M L A S T R C L D Q S G M E I L D P L S A E I
                             3060                3070                3080                3090                3100
```

```
HUMAN*   I L R - - - - - - - - - - - - - - - - - - - - - - - - - M L L S L Q P V L Q - - - - D A I Q K K - -
CHICK    I L R - - - - - - - - - - - - - - - - - - - - - - - - - M L L S L Q P V L Q - - - - D A I Q K K - -
XENTR    I L R - - - - - - - - - - - - - - - - - - - - - - - - - M L L S L Q P V L Q - - - - D A V Q K K - -
DANRE    V L R - - - - - - - - - - - - - - - - - - - - - - - - - M L L S L Q P V L Q - - - - D T I Q K K - -
CIOIN    V Y H - - - - - - - - - - - - - - - - - - - - - - - - - L L V S L K P V L Q - - - - D A V Q R K - -
ANOGA    I V R - - - - - - - - - - - - - - - - - - - - - - - - - I M K S M Q P L L Q - - - - D A F H K R - -
TETNG    V L R - - - - - - - - - - - - - - - - - - - - - - - - - V L L S L Q P V L Q - - - - D T I Q K K - -
DROME    I V R - - - - - - - - - - - - - - - - - - - - - - - - - T M N A I Q P M L Q - - - - N A F H C K S R
BRUMA    C M A - - - - - - - - - - - - - - - - - - - - - - - - - L L Q E I G P I L E - - - - E S L R I A - -
CAEEL    W V D - - - - - - - - - - - - - - - - - - - - - - - - - L L G V V T P T L E - - - - G S M K Q A - -
YEAST*   - - - - - - - - - - - - - - - - - - - - - - - - - - - - I S E K E Q D I L D L I A E Q V V T D Y - -
SCHPO    V L N F S S Y M R K P - - - - - - - - - - - - - - - - - I R D Y L E K I L L G E S S A V F L S K S P
CANAL    M I V - - - - - - - - - - - - - - - - - - - - - - - - - K L G F L K E F L D F M S P N I I F D L - -
CANGA    - - - - - - - - - - - - - - - - - - - - - - - - - - - - V N E R N N Q I I D L L A E Q I V Y D Y - -
ASPNI    - - - - - - - - - - - - - - - - - - - - - - - - - - - - T Q S S L D H K I D G R F S V S C L D N - Q
RHIO9    V I A R R R N L L Q T F Y G D T V H I C S T L H D V D Y M N Q S F K N I L N N I F S K K A P D V N H
CRYNE    I I G - - - - - - - - - - - - - - - - - - - - - - - - - N R A F R K S V L D - - P E D E Y E Q S S A
USTMA    K I E - - - - - - - - - - - - - - - - - - - - - - - - - V L E A M A S V L S I A P A S E G S K Q P S
ARATH*   F P S - - - - - - - - - - - - - - - - - - - - - - - - - S V I T L - - - L Q - - - - S D I K T A - -
VITVI    N A S - - - - - - - - - - - - - - - - - - - - - - - - - S V F T L - - - I Q - - - - S D I K N A - -
SOYBN    - - - - - - - - - - - - - - - - - - - - - - - - - - - - - - - - - - - - - - - - - - - - - - - - - -
SOLLC    Q A S - - - - - - - - - - - - - - - - - - - - - - - - - F T L T L - - - L Q - - - - N D I K A A - -
ORYSJ*   S D S - - - - - - - - - - - - - - - - - - - - - - - - - S V I S L - - - L Q - - - - S D I K A A - -
HORVU    - - - - - - - - - - - - - - - - - - - - - - - - - - - - - - - - - - - - - - - - - - - - - - - - - -
SORBI    S E S - - - - - - - - - - - - - - - - - - - - - - - - - S M I S L - - - L Q - - - - S D I R Q A - -
SELML    S P A - - - - - - - - - - - - - - - - - - - - - - - - - T V A S I - - - L Q - - - - S D M R Q A - -
PHYPA    S A A - - - - - - - - - - - - - - - - - - - - - - - - - T I A S L - - - L Q - - - - S D V R M A - -
                             3110                3120                3130                3140                3150
```

MED13

```
HUMAN*  --RTVRPWG----VQG--PLTWQQFHKMAGRG------------------
CHICK   --RTVRSWG----VQG--PLTWQQFHKMAGRG------------------
XENTR   --RTVRSWG----VQG--PLTWQQFHKMAGRG------------------
DANRE   --RSVRSWG----VQG--PLTWQQFHKMAGRG------------------
CIOIN   --RTSRTFGTTQHVKG--PLTWREFCKEAGKN------------------
ANOGA   --CTTRLWDAPYTIQG--PLTWRQFHRLAGRG------------------
TETNG   --RSVRSWG----VQG--PLTWQQFHKMAGRG------------------
DROME   GGSGSKDASSYNTVSG--PLTWRQFHRLAGRA------------------
BRUMA   --RSSPLFGSNNIVEG--PLTWRFFDRKALKA------------------
CAEEL   ---RHIPAETPFELDG--PMTWKQIVTKSNRG------------------
YEAST*  ------------NILG--NLGIPKIAYRGVKDCQEG--------------
SCHPO   ENYLSSINNGHHALND-NPPSQVNFSETLVNF------------------
CANAL   --GLMKNDESDYYINGVNDLLL---------NLDPGISSDWILNSLSKVF
CANGA   ------------NILG--NLTIPDVPYSGIRNYSNG--------------
ASPNI   PSWSRELLEGSEYLGGIEKLDLNSFVSLQEQN------------------
RHIO9   MCIDQPLLPASVTVKG--PLNVQQYYDLSETNQAHS--------------
CRYNE   WMGSLLEGDALCEVFGSDVLPVN---------------------------
USTMA   DTDMDSAAAGNEAQSA--LPTLKYLVKVTGRA------------------
ARATH*  ----LKSAFGQ--SDG--PLSVTDWCKGRNQSGDGGSISEGFTAESALSE
VITVI   ----LKSAFGI--LDG--PLSATDWCKGRSQSGDVGTTGDGFSAEH--GV
SOYBN   ---------KTSEVKGCDILVV----------------------------
SOLLC   ----LKSAFST--MEG--PLSVVDWCKGRSQSNDGGISGDGFSAESTASA
ORYSJ*  ----LKTAFAN--MDG--PLLVTDWCRGRSNAAEYASMGDAYSFQH--PT
HORVU   --------------------------------------------------
SORBI   ----LKAAFAN--MDG--PLSVIDWCRGRGNAAE-SVTADAYSFQY--ST
SELML   ----VGVAFSDINAAG--PLSLAEWCRGKTPTDNHS--------------
PHYPA   ----ISNAFGDTAADG--PLALIDWCKGCVQGSDGGLCAESTNTEV----
                  3160      3170      3180      3190      3200
```

```
HUMAN*  --------------------------------------------------
CHICK   --------------------------------------------------
XENTR   --------------------------------------------------
DANRE   --------------------------------------------------
CIOIN   --------------------------------------------------
ANOGA   --------------------------------------------------
TETNG   --------------------------------------------------
DROME   --------------------------------------------------
BRUMA   --------------------------------------------------
CAEEL   --------------------------------------------------
YEAST*  --------------------------------------------------
SCHPO   --------------------------------------------------
CANAL   PCTYPMKLIELLYDFKSLELEDQLDSQLNFLNEIADEEDFVGPKALYRKL
CANGA   --------------------------------------------------
ASPNI   --------------------------------------------------
RHIO9   --------------------------------------------------
CRYNE   --------------------------------------------------
USTMA   --------------------------------------------------
ARATH*  VNGVNISDDF-I-IDKYFGKQ----------AVSNAIDGGKGD-------
VITVI   NEGRDSSSTV-T-IGEPISPSQSSAGGSSCIKVSSVTDGARVD-------
SOYBN   --------------------------------------------------
SOLLC   SECRDSSSTISLSVGEPISPSQSSAGGS-----SSLRDGIRVD-------
ORYSJ*  GDIRESSSSISI-GGDSMSPPQSSHVI-----------------------
HORVU   --------------------------------------------------
SORBI   GDTLEPSSSLPI-GGDSLSPPQPA--------------------------
SELML   ------------------GPSDSKDLDLDNHAPDFRRSGREAE-------
PHYPA   -------NSTVTVVGESMTPSQSGGVLKGADIDDIRRNSSSSRAVDSSSL
                  3210      3220      3230      3240      3250
```

```
HUMAN*  --------------------------SYGTDESPEP---LPIPTF--LLG
CHICK   --------------------------SYGTDESPEP---LPIPTF--LLG
XENTR   --------------------------SYGTDESPEP---LPIPTF--LVG
DANRE   --------------------------SYGTDESPEP---LPIPTL--LVG
CIOIN   ---------------------------------AEP---QSIPSF--VVG
ANOGA   ------------------------------TGQCEP---QPIPSL--IVG
TETNG   --------------------------SYGTDESPEP---LPIPTF--LVG
DROME   ------------------------------SGQCEP---QPIPSV--VVG
BRUMA   --------------------------AGGTEDDSGP---EAVPNI--VVA
CAEEL   --------------------------KPPADDEEDFSLPEPVPAI--MRA
YEAST*  ------------LITTTMLQLFSTFDRLNGNDTISKFYNMKQPYV--FVK
SCHPO   ---------------------------------------SQPPRV--LLK
CANAL   KALEWDSFSLNELNKTSFEKYKNVMEKLSAENTVSDDDYFKLPMVKTRIL
CANGA   ------------VVKNTIDNLFSEFTRLDGTTLISRLYPMETPFV--SVR
ASPNI   -------AQNFLSPDAGPAPRQNLQHKDTGRGSITK---LSPPHL--RVR
RHIO9   -----------------KYGKYQVKRRRPAEPNLDT---LRPPNI--VVN
CRYNE   ---------------------------------------LPQPQVNATSP
USTMA   -----------------TGEVAAAACAADADSGLEIAEVLEPSRI--AVG
ARATH*  ----ETAQ----SQDIYSSE--------LLRPTLFV---LPSPAI--LVG
VITVI   ----DTCRRR-PNQEFSSSESEQQLG-SRLRPTLFV---LPLPAI--LVG
SOYBN   ----HPSRRG--------ASLMLALSLLATSPLNLS---LPHPV------
SOLLC   ----EASERR-LSQDTCLSESEQLPG-SRLRATLAA---VPYPAI--LVG
ORYSJ*  ------------SNDRGTSELEHHRGYHRVRPTVAV---LPLPSL--LVG
HORVU   --------------------------------------------------
SORBI   ------------SSNRGISELEYQKGYHRIRPTIAV---LPSPSM--LVG
SELML   ----DAAQRRLCVDD--------------AESQALSYAMLPVPTL--LVG
PHYPA   SDQTDPSQRRGLSDDSGFSHADAL---GYNESSILA---IPTPSL--LVG
                  3260      3270      3280      3290      3300
```

MED13

```
HUMAN*   YDYDYLVLSPFALPYWERLMLEPYGSQRDIAY--VVLCPENEAL-LNGAK
CHICK    YDYDFLVLSPFALPYWERLMLEPYGSQRDVAY--VVVCPENEAL-LNGAK
XENTR    YDYDFLVLSPFALPYWERLMLEPFGSQRDIAY--VVVCPENEAL-LNGAK
DANRE    YEYDYVVLSPFALPYWEKLLLDPYSSQRDVGY--MVVCPDNEAL-LNGAK
CIOIN    YNHDWVCISPESLRYWEKLYFEPYSSPRDVGY--VVVCPDGDNL-VSSAK
ANOGA    YEKDWLSLAPYALHYWDKLLLEPYSYPRDVAY--VAIVPDNDYV-VAKVR
TETNG    YEFDFLVLSPFVLPYWEKLLLEPFGSQRDVGY--VVICPENEAL-LSRAK
DROME    YEKDWISVAPHSIHYWDKFLLEPYSYARDVVY--VVVCPDNEHV-VNCTR
BRUMA    SEKDAVITSPQIIRLWEKMSLEPYDQPKDVLY--IGVVPDNLIC-IEKTK
CAEEL    LTKEAIRAAPNIEQYYEQAGLGPVDQPKDVMY--ITVIPDDDDI-YSRTV
YEAST*   KHHELIKVKHDSQPFIKFLNFRPPNGIKNFKS--LLLSSSF----KEDCL
SCHPO    YNEKKLSLDSSAPENWISLCLQPYGESKDFEV--FLLSSKSPDV-SSKAI
CANAL    KNGNIVNLNNIGLKFWKYLNFSPVKKQKDFQI--LLIAETHRNT-ASYAT
CANGA    KQHDQIKLRSDVQQFTKYANLKPVRGIKNFKF--LVLTDSF----KEDCI
ASPNI    RGKDFLETLPPAISFWETFDLEPAHGPKDITA--YCIHPQFS---TRAAN
RHIO9    RQEELIEGTTKFIMFWEKLRLEPYSCKKHINY--FIIYPENDST-ERSIS
CRYNE    STAHIIRLSIAALSYWSQLGLEPQGGKKDVKM--FIICKDSDEA-LGKML
USTMA    CQGSVVEALPSALTLWDKSKLSAVSGQKHVVA--KVLLTDASPAWHEEIV
ARATH*   YQDDWLKISTNSLTHWEKAPFEPYALPKSINY--AVVCPDIDPL-TCAAT
VITVI    YAS-----------------------------------------------
SOYBN    YQDDWLKTSANSLQHWEKAPLEPYALQKPITY--HVVCPDIDPL-TSAAA
SOLLC    YQDDWLKTSPSSLQFWEKAPFEPYAMPKHMTY--HVVCPDINAL-TTAAT
ORYSJ*   YQDDWLKTSANCLSLWEKAPLEPYASPKPVTY--YALCPDIEML-TSAAT
HORVU    ---------------------SPYNYTNSLCFKVYSLLLSIFIL------
SORBI    YQDDWLKASVNSLKTWEKAPFEPYALPKPVTY--YALCPDIDML-TSAAA
SELML    YQEDWLRTAPRSLRYWEKAPFEPYGYPKSVTY--YVICAGLDPL-LCSAL
PHYPA    YQEDWLKTSSSALHLWEKAPLEPYAPSKQVNY--HVICLASEPL-VTATA
                  3310      3320      3330      3340      3350
```

```
HUMAN*   SFFRDLTAIYESC--------------RLGQHRPVSRLLTD---------
CHICK    SFFRDLTAIYESC--------------RLGQHRPICKLLPD---------
XENTR    SFFRDLTAMYESC--------------RLGQHRAISKQLTD---------
DANRE    TFFRDLTAVYESC--------------RLGQHRPIAKSHAD---------
CIOIN    VFFKELSSLYESS--------------RLGKFKAINKVLKDG--------
ANOGA    TYFKELSTTYEMC--------------KLGRHTPIKGWD-----------
TETNG    TFFKDLSTTYEAC--------------QLGQHRPICRSHPE---------
DROME    SYFRELSSTYEMC--------------KLGKHTPIRGWD-----------
BRUMA    KYLMDLSRMYEQC--------------RFGRHIPFSREVLRD--------
CAEEL    EFMDTLTRTYEKM--------------RLGRHIPFPVSTGTATRFREAFK
YEAST*   SFAPTLSQTYINQ--------------ELGFCELLKLTNEDPP-------
SCHPO    SFFYDVQLAYENC--------------KLGKLNLSETSINERVMGFST--
CANAL    EFLDQIIQNYKEC--------------NFGTISKVNLSTVETRSDLEPIS
CANGA    QFISSLSQTYINH--------------ELGFCEHLQLTNEDSK-------
ASPNI    VFLDRLGLLYPSC--------------NLGSHSRGENIDACRN-------
RHIO9    QFFKNLGTLYETC--------------QLGSHQPGNIGSYRK--------
CRYNE    G--EGVKLGWEEE--------------RYGKAEFS---------------
USTMA    AWLDRVRIAFETH--------------GLGTHIGNAQ-------------
ARATH*   DFFQQLGTGESTSLSFVKEVMLKHDRNWLGTHLPQSLGNQME---KDVGR
VITVI    --------------------------------------------------
SOYBN    DFFQQLGTVYETC--------------KLGTHSPQGLGNQIE---IESAK
SOLLC    DFFQQLGTVYETC--------------KLGTHSPQFMGNEME---IDSGK
ORYSJ*   DFFLQLGTIYEVC--------------KLGTHSPQNSGGQME---LSPGK
HORVU    -------IVYEVC--------------KLGTHSPQHSGGQID---QSPGK
SORBI    DFFMQLGTVYEVC--------------KLGTHSPQNSGGQME---LSSGK
SELML    EFFQQLSSVYEAC--------------KLGAHIPATVTAQ------TVSK
PHYPA    DFFQQLSSVYEVC--------------RLGSHMPVGTAAGQSGSIASSSK
                  3360      3370      3380      3390      3400
```

```
HUMAN*   --------------------------------------------------
CHICK    --------------------------------------------------
XENTR    --------------------------------------------------
DANRE    --------------------------------------------------
CIOIN    --------------------------------------------------
ANOGA    --------------------------------------------------
TETNG    --------------------------------------------------
DROME    --------------------------------------------------
BRUMA    --------------------------------------------------
CAEEL    SCQSQFPFQEQQHLAYTNQTLRNMPQTLADTPDPVNSDPKVWSRRAKRKL
YEAST*   --------------------------------------------------
SCHPO    --------------------------------------------------
CANAL    D-------------------------------------------------
CANGA    --------------------------------------------------
ASPNI    --------------------------------------------------
RHIO9    --------------------------------------------------
CRYNE    --------------------------------------------------
USTMA    --------------------------------------------------
ARATH*   LSSS----------------------------------------------
VITVI    --------------------------------------------------
SOYBN    LSSC----------------------------------------------
SOLLC    NASS----------------------------------------------
ORYSJ*   YLSS----------------------------------------------
HORVU    YLPS----------------------------------------------
SORBI    YLPS----------------------------------------------
SELML    RLLP----------------------------------------------
PHYPA    QALS----------------------------------------------
                  3410      3420      3430      3440      3450
```

MED13

```
HUMAN*  --------------------------------------------------
CHICK   --------------------------------------------------
XENTR   --------------------------------------------------
DANRE   --------------------------------------------------
CIOIN   --------------------------------------------------
ANOGA   --------------------------------------------------
TETNG   --------------------------------------------------
DROME   --------------------------------------------------
BRUMA   --------------------------------------------------
CAEEL   DFEEELKLPEAGSETIDESDDDEENEEVSKEENNELIDSEDVMDEVPKGF
YEAST*  --------------------------------------------------
SCHPO   --------------------------------------------------
CANAL   --------------------------------------------------
CANGA   --------------------------------------------------
ASPNI   --------------------------------------------------
RHIO9   --------------------------------------------------
CRYNE   --------------------------------------------------
USTMA   --------------------------------------------------
ARATH*  --------------------------------------------------
VITVI   --------------------------------------------------
SOYBN   --------------------------------------------------
SOLLC   --------------------------------------------------
ORYSJ*  --------------------------------------------------
HORVU   --------------------------------------------------
SORBI   --------------------------------------------------
SELML   --------------------------------------------------
PHYPA   --------------------------------------------------
                  3460      3470      3480      3490      3500
```

```
HUMAN*  ----GIMRVGSTASKK---LSEKLVAEWFSQA------------------
CHICK   ----GIMRVGPTASKK---LSEKLVTEWFSQT------------------
XENTR   ----GIMKVGSTAAKK---LSEKPVAEWFSQA------------------
DANRE   ----GIVTVSDAGSKA---LTDQTLIDWLPKT-----------------I
CIOIN   ----GILKIGKNSSKV----SHLPLDDWFEQL----------QQTLNSTS
ANOGA   ----GILRVGSQRVLK--ETQNSNLDEWFTNMGVDQQRKNGTTTTTAAGS
TETNG   ----GILKFGATEGRS---ITEQPLSDWFLKM-----------------A
DROME   ----GFLQVGAARNNVPADRETTPLDDWLRTL------------------
BRUMA   ----GLLRVPTRYPAT----NPGECDNFLNQV----------------ER
CAEEL   YEREGILRVGAPMEPSRSPHTVTNTPEFDNMT-----------------K
YEAST*  ----GLMYLKAFDKNK----------------------------------
SCHPO   ----NINETDNYDDNETTQSDTATSYE-----------------------
CANAL   ----GLVLVHKEHDQLYNDFYIQTNKKLI---------------------
CANGA   ----GLIYLKDFEDSK----------------------------------
ASPNI   ----GLVEWDVKSVKR----------------------------------
RHIO9   ----GLVPVRLLSQ------------------------------------
CRYNE   ----GVVKGKMEDFG-----------------------------------
USTMA   ----SILAIADGSETL----------------------------------
ARATH*  ----GFVLLDCPQSMK----------------------------------
VITVI   --------------------------------------------------
SOYBN   ----GFVLLDCPQSMK----------------------------------
SOLLC   ----GFVLIDCPQSMK----------------------------------
ORYSJ*  ----GLVLTECPDQVK----------------------------------
HORVU   ----GLVLVDCPDQLK----------------------------------
SORBI   ----GLVLVECPEQLKK---------------------------------
SELML   ----GFVPVDFPSKLK----------------------------------
PHYPA   ----GFSLVDISGVSHSTA------------------------------R
                  3510      3520      3530      3540      3550
```

```
HUMAN*  -ADGNNEAFSKLKLYAQVCRY--DLGPYL-ASLPLDSSLLSQPNLVAPTS
CHICK   -ANANNEAFSKLKLYAQVCRY--ELGPYL-ASQPLDNSLLAQTNLVPPSS
XENTR   -INGNSEAYSKLRLYAQVCRY--ELGPYL-ASQPLDSSLLCQPNLTSSAI
DANRE   NSSSSSEALNKLKLYAHVCRH--DLASCL-ASQSLDGSLLTQRNPASSS-
CIOIN   DNLVGNENVDKLLLYARECLH--SLGPLLEAEQSLEINSLHVREGRNRMQ
ANOGA   TGSSSSGLNELLRLYAQTCQQ--QLAPYL-SKVPSDKSLLDPPESHHHTS
TETNG   ARDRYNEAFNKLKLFAQVCRY--DLAPYL-SEQSLDSSLLSQRSPA----
DROME   ---EHAALAEQIRRYAVAFIH--QLAPYL-SRVPNDKTLLNPPDGSGNSH
BRUMA   HIGDNKSLITRLKVYMQYFEN--EMARIL-INNDDIFSRDKYRAALAESQ
CAEEL   YLSDENGFVSRLRLYLQQME---DLVHFALSENPEAFDRRGYRYQLAVEG
YEAST*  -----------LLLLAA------QIVSYCSNNKNSIKN------------
SCHPO   -----------------------QLASVCVNELSGKN-------------
CANAL   ------SLVELIKLDLINKTNNFEFGKPL---------------------
CANGA   -----------LLLLAA------QIVSYLSTNRTSGK-------------
ASPNI   --PNYQTAVQQLKLICE------ELGTALLDGMPSKD-------------
RHIO9   ---DESLEDQQMRSYLTECQ---NLGSTLGSVPGENV-------------
CRYNE   -----------------------ELAIGITEQSTGSV-------------
USTMA   -----------------------ALSSYLEQLWQDGEAWLDTLRSISNRV
ARATH*  IESNNTSLLGSLSDYFLSLSNGWNVNSYL-KSLSKALKGLKLGSGLYTN-
VITVI   --------------------------------------------------
SOYBN   IESSNASLVGSVSDYFLSLSNGWDLTSYL-KSLSKALRGLKIGSCFSTN-
SOLLC   IDSSSASMLGSISDYFLSLSNGWDLESYL-KSLSKVLRNLKLSSCMTMN-
ORYSJ*  IGNGHSSSISSTSEYLQALSRNWTVKSFV-TSLTRVIKDIKLNSSILAN-
HORVU   TSGSHSVSISSVTEYFQALSKSWSVKSFI-SSLARIIKDIKLTLNISTN-
SORBI   VGCGHLSPITSTSDYLQVFSKHWSVKSFV-TSISRILKDIKLTSNISTN-
SELML   ---GSSSSINLANDYITAMDNGWDVSEFR-KSLRKACKSLNLGNSVSL--
PHYPA   REYFTASNYSLAKAFVADMTANWNMAEYR-KALKKACKALPISSGSSVS-
                  3560      3570      3580      3590      3600
```

MED13

```
HUMAN*   Q-SLITPP--------QMTNTGNANTPSATLASAASSTMTVTSGVAISTS
CHICK    QPASALPP--------LTASTGNPNTPSAP-AASTSSTMTATSNSAMSSA
XENTR    QPAAASTP-------------GNPSTPSSLNSSAAN-----------SSS
DANRE    QTSSSSSP------------------------VTTAQNVTPTTN---SNS
CIOIN    QSLNRNAP------------------------------------------
ANOGA    SSASASAS------------------------------------------
TETNG    -LASSSSP------------------------SQTSSSSSITSG---PQS
DROME    SK------------------------------------------------
BRUMA    IHSVQHIS-----------------------------IAAYQCGGYTVSS
CAEEL    RLKRQKHKMDIIEERLRFEASKDFDEQSDRKEPLEENDPEITPGIVISNN
YEAST*   --------------------------------------------------
SCHPO    --------------------------------------------------
CANAL    --------------------------------------------------
CANGA    --------------------------------------------------
ASPNI    --------------------------------------------------
RHIO9    --------------------------------------------------
CRYNE    --------------------------------------------------
USTMA    QLDLLQGK------------------------------------------
ARATH*   QKEGSGSP------------------------------------------
VITVI    --------------------------------------------------
SOYBN    PSEGSNSS------------------------------------------
SOLLC    PKEGSTGP------------------------------------------
ORYSJ*   QKESTSGP------------------------------------------
HORVU    QKESSNMP------------------------------------------
SORBI    QKESSSGP------------------------------------------
SELML    EADHENSP------------------------------------------
PHYPA    QREPEQGP------------------------------------------
                            3610      3620      3630      3640      3650
```

```
HUMAN*   VATANSTLTTAST--SSSSSSNLNSGVSSNKLPSFPPFGSMNSNAAGSMS
CHICK    ATTANSTLTTTAT---SSSSANIGSGIPTNKPSSFPPFSSMNNTTSASLP
XENTR    TVTPNTLPQAAAT--GSAATSNVAGGLTANKPTSFPPYGNINSTPN-SIP
DANRE    NTNTNTTPTSTSTSSSSSSCPQGVGNMPSSKPNTIPPFGAQGLQSS----
CIOIN    -------------ISNSINNIGRSPAQMQSNPTAVALKD-----------
ANOGA    -------------LGGGAAGASSLGGGSMGQPGGNAMGQNRPDGPGQS--
TETNG    TTACGQPAQISSTPPSSSSVSQAIGGMASSKPSSYSPFGNAGLQSG----
DROME    ----------------GGSSCSSNSSSVSGLPGGDLPTDNIKLEPGTE--
BRUMA    SDHMPPPSAPGSIIISGQSSDASHGPNSTSGPSSSGITIGESLTP-----
CAEEL    PVESWIDDELEEKRQQRQKENEQYPPEAQQAPSPVPAGMIHIPETLSKEE
YEAST*   --------------------------------------------------
SCHPO    --------------------------------------------------
CANAL    --------------------------------------------------
CANGA    --------------------------------------------------
ASPNI    --------------------------------------------------
RHIO9    --------------------------------------------------
CRYNE    --------------------------------------------------
USTMA    --------------------------------------------------
ARATH*   --------------------------------------------------
VITVI    --------------------------------------------------
SOYBN    --------------------------------------------------
SOLLC    --------------------------------------------------
ORYSJ*   --------------------------------------------------
HORVU    --------------------------------------------------
SORBI    --------------------------------------------------
SELML    --------------------------------------------------
PHYPA    --------------------------------------------------
                            3660      3670      3680      3690      3700
```

```
HUMAN*   TQANTVQSGQLGG--QQTSALQTAGISGESSSLPTQPHPDVSE-STMDRD
CHICK    SQAATVQNGQTGGQ-QQQPVLQTAGMSGDATTAPAQPHPEVSE-STMDRD
XENTR    AQVNSVQSNQGGPQTQQQTLQQNSGMSSEQSAAAAAAAAEVTERSTMDRD
DANRE    -------QQSGG---QSAGTLGDA----TSATSQPQVPSEPAE-STMERE
CIOIN    --------------------------------------------------
ANOGA    ---------------PMPPPPPSTPDSSQPGDKAPNTPNPADG-------
TETNG    -------SSQNG---PQATPQGAAGPAENGATNQPQGSAEVPE-STVERD
DROME    ---------------PQVQPMETNEIKQEPGVGKGGTAAGETKPTLILGD
BRUMA    ----------------------------DGSIGDGSAQNMTNFSSFMTEQ
CAEEL    RAVQPTLEMILADPATVAPINAQNIVWKQRDTRVPNPFPSSNQPPVAFE-
YEAST*   --------------------------------------------------
SCHPO    --------------------------------------------------
CANAL    --------------------------------------------------
CANGA    --------------------------------------------------
ASPNI    --------------------------------------------------
RHIO9    --------------------------------------------------
CRYNE    --------------------------------------------------
USTMA    --------------------------------------------------
ARATH*   --------------------------------------------------
VITVI    --------------------------------------------------
SOYBN    --------------------------------------------------
SOLLC    --------------------------------------------------
ORYSJ*   --------------------------------------------------
HORVU    --------------------------------------------------
SORBI    --------------------------------------------------
SELML    --------------------------------------------------
PHYPA    --------------------------------------------------
                            3710      3720      3730      3740      3750
```

MED13

```
HUMAN*  KVGIPTDGDSHAVTYPPAIVVYIIDPF-----------------------
CHICK   KVGVPTDGDSHAITYPPAIVVYIIDPF-----------------------
XENTR   KVGVPTDGESHAVTYPPAIVVYIVDPF-----------------------
DANRE   KVGVPTDGDSHAVTYPPAIVIYIVNPF-----------------------
CIOIN   --TRPVYHPSYNASTACSIVVYLLDPF-----------------------
ANOGA   ------MRIDDDGKDPPHIVLYIVEPF-----------------------
TETNG   KVGKPTDGESHAVSYPPAIMVYIVDPF-----------------------
DROME   PLG---MGETLEDINPSAIVLYVVNPF-----------------------
BRUMA   LVADGIIAEDEPGTLPHVIVIYLVNPF-----------------------
CAEEL   --AAGSPDTDAYSTLPHVIVVYVVNPF-----------------------
YEAST*  -----------------VPPILIILPL-----------------------
SCHPO   -----------------VLFFYFLE-------------------------
CANAL   -------------------LLFFIDFN-----------------------
CANGA   -----------------EVAFMMIIPV-----------------------
ASPNI   -----------------NIVVYIINPF-----------------------
RHIO9   -----------------YNVIYMLNPS-----------------------
CRYNE   -----------------NTIVYLLTPP-----------------------
USTMA   -----------------HVVVYTLQPP-----------------------
ARATH*  -----------------CVVVYIVCPFPDPSAVLRTIVESSIALGSVI-Q
VITVI   --------------------------------------------------
SOYBN   -----------------CLVIYVVCPFPDPTAILQTVIESSVAIGSVVQQ
SOLLC   -----------------CTVVYVVCPFPEPLAVLQTVIESSIAVGSGVLS
ORYSJ*  -----------------CTVIYVVCPFPEPSAVLETLVECSVALGSVMLS
HORVU   -----------------CTVVYVVCPFPEPSAVLQTLVESSVALGSIL-S
SORBI   -----------------CTVIYVVCPFPEPCAILQTLVECSVALGYVISS
SELML   -----------------STVLYVVSPSSEPSMVLQTLMETCQSIGPNLNI
PHYPA   -----------------CTVVYVVCPVSDPAGILQIMVDACTSLGSGITG
                  3760      3770      3780      3790      3800
```

```
HUMAN*  -----------------TYENTDESTNSSSVWTLGLL-RC--FLEMVQTL
CHICK   -----------------TYEKKDENSSSSSLWTLGLL-RC--FLEMVQVL
XENTR   -----------------TYESKDEDSNSSSVWMLGLL-RC--FLEMLHIL
DANRE   -----------------SYEENCQGS-SSSVWTIALL-RC--YLEMLQLL
CIOIN   ----------------------------KNSASRGLW-HC--FRILQKSL
ANOGA   -------------------TCGNDSPDVERLACLSLL-RC--YSNILNAV
TETNG   -----------------SYEAADREA-HSSTYTLGLL-CC--YMEMLKLL
DROME   -------------------TFASDSCELERLALIALL-RC--YAELLKAV
BRUMA   ------------------LLGAEENPLVARVVTVALM-RA--FNALLYRL
CAEEL   -----------------SYGPEGHSALHMRIAILAFI-RA--FNSIMCKI
YEAST*  ----------------------DNATLTELVDKANIF-QV--IKNEVCA-
SCHPO   ------------------------DDSEKLLKACQHF-IC--VKDSIKRL
CANAL   ------------------------DSSNSNVQVCKIF-RN--FK-----V
CANGA   ----------------------QRCDISELVEKTAKF-QI--IQNEVKA-
ASPNI   ------------------------SHAASLVDICAAFWTL--LQNYFASA
RHIO9   ------------------------SDLQSNVHLTRCFSKL--VEAYDTAV
CRYNE   --------------------------------------SQIPLNIYSSLI
USTMA   ---------------------------NSTICASAGFHGLLRLEADLRAM
ARATH*  SDRDRRSILNSQVARAFSSSTAVDEASISHIPVLSGF-SV----------
VITVI   --------------------------------------------------
SOYBN   SDRERRSSLHSQVVKALSGLATVDEASASNILVLSGF-SI----------
SOLLC   SDKERRSTLHNQVGKALSYSAAVDEA-LSNVLTLSGF-CI----------
ORYSJ*  PERERKSFLYSQVAKALNCNASVDESSASNVVMLSGF-SI----------
HORVU   SERERKSFLYTQVAKALNSSASADEASASNVVMLSGF-SV----------
SORBI   PERERKS-LYSQVAKALNSSASADEASASNVVMLSGF-SI----------
SELML   KKASGK------------------NSMEDGKQPVVGF-SI----------
PHYPA   ADSQRH-----------GPSTPGEDAMSSNIL---GF-SI----------
                  3810      3820      3830      3840      3850
```

```
HUMAN*  --PPHIKSTV--SVQIIPCQYLLQPVKH----------------------
CHICK   --PPNIKNII--SVQIVPCQYLLQPVKH----------------------
XENTR   --PPHLRNTV--SVQIVPCQYFLQPVRQ----------------------
DANRE   --PPHIRNAV--YVQIIPSQYLLQPVWS----------------------
CIOIN   --PKPIRDHI--IFQIVPIEHVLRVSSP----------------------
ANOGA   --PDSIRSNI--SVQIISLESILELGRN----------------------
TETNG   --PARIRNAV--SVQIVPCQYLLQPVHS----------------------
DROME   --PDSVRSQM--NIQIISLESVMELGPC----------------------
BRUMA   --NNKRRPQL--QLEMIALQNIFDYCGASADFLKDERGRLNGYDQGRLEK
CAEEL   --PYEKRPQL--QLEIVGMEGMDNVAKPIPDYFNDAKIPFDLLNDRPIRV
YEAST*  -----KMPNIELYLKVIPMDFIRNVLVT----------------------
SCHPO   GDNKFEDKSL--RICTIPNSIFDSPNSH----------------------
CANAL   ALTTHQLPLVDIFTKIVPSSLLVKKVHH----------------------
CANGA   -----KIPSMELYLKIVPMDFIKSPLTS----------------------
ASPNI   EKPPKQVDEV--LLQIVPLDFVMSAESL----------------------
RHIO9   LNSGTRTSNAKPIMQLIPVEHVLRSSSF----------------------
CRYNE   PNSP--------NTLLIPVPTPSNPSTS----------------------
USTMA   LSEQVGVLAEQLLVRAVSPSMMTESGSL----------------------
ARATH*  -------PKL--VLQVVSVDSIFRITSP----------------------
VITVI   --------------------------------------------------
SOYBN   -------PKL--VLQIVTVDAIFRVTSP----------------------
SOLLC   -------PKL--VLQIVTVDAIFRVTSP----------------------
ORYSJ*  -------PKL--VLQIVTVETLLRLHKP----------------------
HORVU   -------PKF--VLQIVTVETLLRLHKP----------------------
SORBI   -------PKL--VLQIVTIETVLRIDKP----------------------
SELML   -------GRL--ALQVVTPETVIRLSSS----------------------
PHYPA   -------PRF--ALQLVTAETIFKNSGP----------------------
                  3860      3870      3880      3890      3900
```

MED13

```
HUMAN*  --EDREIYPQHLKSLAFSAFTQCRRPLP----TSTNVKTLTGFGP-----
CHICK   --EDRQIYTQHLKSLAFSAFTQCRRPLP----SSTNVKTLTGFGP-----
XENTR   --EDRQIYTQHLKSLAFSVFTQCRRPLP----ASTNVKTLTGFGP-----
DANRE   --EERHIYAQHLKSLAFSVYTQCRRRLP----TSTNVKTLTGFGP-----
CIOIN   --NTRQSFVHVVRSLSFSTFAQCRRNLL----HEVKVRSMTGFGP-----
ANOGA   --RNRLRLSDHMRCLALSVFSQSRKYLA----HMNTVKSLTGFGT-----
TETNG   --GERHVYSQHLKSLAFSVYTQCRRPLP----NSTNFKALTGFGP-----
DROME   --GNRKRFSDEIRCLALNIFSQCRRHLV----HAQSVKSLTGFGT-----
BRUMA   SQNERLSSSDSLKTIAFSVYTHSRVVLPDIV-RGILPKSMTRFGP-----
CAEEL   ERPGESVQGELARSLSIAVYTHPRVFFPDVY-KSASARCMTAFGP-----
YEAST*  ---------VDQYVNVAISIYNMLPPKSVKFTH-----------------
SCHPO   ----TTNSNSFFTKVSLDIYNNDPLLMDGSLKRRE---------------
CANAL   ETALKVFSNYKLTKISMNLYNECPNDLA----NKSIVKNLFTT-------
CANGA   ---------VDDYTNLCISIYNILPNKMIKFTH-----------------
ASPNI   ----VVPPQAEYLNLALEVYSRCSTRQM----QPSLVNC-----------
RHIO9   ----GGCLKFGLKEIAFSVYSKCRAVVT----RRRNNGNLESSVS-----
CRYNE   ------FVHEQYLTLVRTAYDRLPTFLRPIYVRGEPLRV-----------
USTMA   ---GFGQHSQALRTLAFSVYDQLPRLV-----RRQPAKVLHGKEP-----
ARATH*  ----SFNELVILKDTAFSVYNKARRISRGM-P-NDAFFSSSLPSR-----
VITVI   --------------------------------------------------
SOYBN   ----SVSELVILKETSFTVYSKARRISRGI---SSDFAQSAFSSR-----
SOLLC   ----ALSELVILKEIAFTVYNKARRISRGS--PSDMVQSSSMPGR-----
ORYSJ*  ----N-NELAVLKDMAFTVYNKARRIPKAI-STSDMFQSPAYMGR-----
HORVU   ------NELAAFKDIAFTIYNKARRIPRFV-STSDMFQSPTYMSR-----
SORBI   ----N-KELAVLKDIAFTVYNKARRIPRAV-STSDMFQSPTYLGR-----
SELML   ----PAAGLDILKELSFAVYNKLRRIPYKA---AEDTQQYGFASK-----
PHYPA   ----QASSVDVLKEVALGVYNKVRRIPRRS-QRTEGLQNAGAAGSRGQGG
                       3910      3920      3930      3940      3950
```

```
HUMAN*  --GLAMETALRSP-------------------------------------
CHICK   --GLAMETALKSP-------------------------------------
XENTR   --GLAIENALKSP-------------------------------------
DANRE   --GLAIDTALQSK-------------------------------------
CIOIN   --AASDKRYLQEN-------------------------------------
ANOGA   --AANAEQFIKRK-------------------------------------
TETNG   --GLAIDTALKNP-------------------------------------
DROME   --AANMEAFLKTK-------------------------------------
BRUMA   --ASAMVDLLNEL-------------------------------------
CAEEL   --GSQLMNTINKI--------------------EALNKDSFARMAKRSKT
YEAST*  --------------------------------------------------
SCHPO   --------------------------------------------------
CANAL   --------------------------------------------------
CANGA   --------------------------------------------------
ASPNI   --------------------------------------------------
RHIO9   --------------------------------------------------
CRYNE   --------------------------------------------------
USTMA   --------------------------------------------------
ARATH*  --SSSALTPMNS-ISGIWKDCGGSRMTGSTHPRDGEIDVSLR-TSGWDTS
VITVI   --------------------------------------------------
SOYBN   --SHSGLTQMPSPISGMWKDCVGPRMAGHSLPREGDIDASLR-PGTWD--
SOLLC   --SHPVLMQMNSPVPGMWKDCVGPRGIGTSLQREADLDANLR-PGSWD--
ORYSJ*  --SQSTMMHATSPGPTLWKECLVPRMSGSTLSRETEFDASMRSSVTWD--
HORVU   --PQSTMMHTASPGPTLWKECLVPRMSGQTLSRETEFDASMR-SVSWD--
SORBI   --SQSTMMHVTSPAPTLWKECLVPRMSGPTLSRETDFDASMR-SATWD--
SELML   -----SRMGLQSNTAMTWKDCRNVSLLDTG-------------NLRWD--
PHYPA   NIGNPSGIQSNASMPGLWKDCSGQRSSGN--PTMATVNSSNN-HNMWE--
                       3960      3970      3980      3990      4000
```

```
HUMAN*  ----------------------DRPECIRLYAPPFILAPV----------
CHICK   ----------------------DRPECIRLYTPPFILAPV----------
XENTR   ----------------------ERPDCIRLYTPPFILAPV----------
DANRE   ----------------------ERPQCLHLYAPPFVLAPV----------
CIOIN   --------------------NHNVLNETRLYTPPYVLSVP----------
ANOGA   --------------------DDKNRVPYRLYTPPYVLARS----------
TETNG   ----------------------ERPECLRLYTPPFILAPV----------
DROME   --------------------DEPNRRAYKMYTAPFVLAPM----------
BRUMA   --------------------ERKEPIYYKVPSKPFILAPP----------
CAEEL   TLDTMDMYRHPGMIQAQQSTEKKNYIAYRVPSNIAVLAPP----------
YEAST*  ------------------------------------IAHT----------
SCHPO   --------------------------------PAFLLKKP----------
CANAL   -----------------------------------IVKDP----------
CANGA   ------------------------------------IRKQ----------
ASPNI   -------------------------------APPVMLAET----------
RHIO9   --------------------------TAEIYAPLFVLPKS----------
CRYNE   -----------------------SEEDAWIQCPAWVLARK----------
USTMA   -----------------------GPISALVQFPAFSLSSAFGRASSGTRL
ARATH*  TSWQIPRSGGLSCDPNRNGDFYLNDEIFYLFEPLFILSEP----------
VITVI   --------------------------------------------------
SOYBN   NSWQPTRTGGLSCDPSRTGDNFLHDEIRYMFEPLFILAEP----------
SOLLC   -NWQASRGGGLGCEPNRIGDFSFQDEIRYLFEPLYILAEP----------
ORYSJ*  NSW-PGRAGGF-MDPNKIPDVCVQDDRKYAFEPLFILAEP----------
HORVU   NSWQPGRAAGL-PDPSKIPELCAQDDRKYAFEPLFILAEP----------
SORBI   NSWQPARSGGL-LDPSKMPDLCAQDDRKYAFEPLFILADP----------
SELML   KSWSSSRL-------LETSASGPPAPFRLLYEPLFILADG----------
PHYPA   NSWKQSIQ----TDLGTASNEITEEKIRYMYEPAYILARS----------
                       4010      4020      4030      4040      4050
```

MED13

```
HUMAN*   --KDKQTELGET--------------------------------------
CHICK    --KDKQTELGET--------------------------------------
XENTR    --KDKQTELGET--------------------------------------
DANRE    --KDKQTELGET--------------------------------------
CIOIN    ---REGNQI-----------------------------------------
ANOGA    --CEKSENTESFG-------------------------------------
TETNG    --KDKQTELGET--------------------------------------
DROME    --HERNDKTDFSRSAGSM--------------------------------
BRUMA    --SPVMQRPNCD--------------------------------------
CAEEL    ---PMVYQMDEKGK------------------------------------
YEAST*   --LPEKVNFRTMQQQQMQQQQQQQQQQQNNSTG-----------------
SCHPO    --LLSTLNYQLKDINP----------------------------------
CANAL    ---PSKIQFKFMNSSY----------------------------------
CANGA    --IPEKLTFKTSQ-------------------------------------
ASPNI    --LPPSIGFRLASE------------------------------------
RHIO9    --VPNSVQYSLKKALCP---------------------------------
CRYNE    --EAVKPEFVMTWPVD----------------------------------
USTMA    AGAAGRTQFSLSWPRE----------------------------------
ARATH*   --GSVERGVSPTFTSLGSESSKPIPEDGGRGSGPGMNSMEGITSGSSSQG
VITVI    --------------------------------------------------
SOYBN    --GSLENGIS-VIGSPTSESSKALADDSSGNYVQSTSTAGSVESASSTDA
SOLLC    --GSLDRGLS-------------FPMSGGGDTGLNTQSETSVPDGF----
ORYSJ*   --GSVDY-------SSGMESSKSGV-DASGSGIYSSISGGGSDSGASASA
HORVU    --GAVDY-------NDTMESSRFGV-DASSSRAYSSIS-GGSDSG--ASP
SORBI    --GSADL-------NALMEPSKSGA-DAGGSRVYGSMSGSNSDSG--VSP
SELML    ----------------CLLDHSMGQLTKARTEEVSTQSFGIPDTGSVADG
PHYPA    --GAQAPARTLS--CDATRDAVAVSSHRTSAAASSGPDVDSTATTERLDG
                  4060      4070      4080      4090      4100
```

```
HUMAN*   --FGEAGQKYNVLFVGYCLSH-----------------------------
CHICK    --FGEAGQKYNVLFVGYCLSH-----------------------------
XENTR    --FGEAGQKYNVLFVGYCLSH-----------------------------
DANRE    --FGEAAQKYNVLFVAYCLSH-----------------------------
CIOIN    --NCKHENPPNILFVSYCVSH-----------------------------
ANOGA    --KTSMKQQCSIMYCSYCLSE-----------------------------
TETNG    --FGEASQKYNVLFVGYCLSH-----------------------------
DROME    --HGQNEHRYSVMYCNYCLSE-----------------------------
BRUMA    --LMQINTDEAVLFVSYCLI------------------------------
CAEEL    --AIMNQLDEQTLFISYCLV------------------------------
YEAST*   --SSSIIYYDSYIHLAYSRSV-----------------------------
SCHPO    --RSSALGEYA-LHVTYTTV------------------------------
CANAL    --RDNSSNDDIFLHLAYERSI-----------------------------
CANGA    --QASAFKYDAYIHLAYSRSV-----------------------------
ASPNI    --RFSPLQEGRCLHVACSRSQ-----------------------------
RHIO9    --FPSILESHAVLHMGYCFSF-----------------------------
CRYNE    --EYDVLNRWRMWHVCYDVVSSTSL-------------------------
USTMA    --PAGVTDEHVLLHVGYRICEAVRGGKAAEARRSDRLSSRNGSAASLAGA
ARATH*   DVSQLEGKAIPSLHCCYGWTE-----------------------------
VITVI    --------------------------------------------------
SOYBN    --SGSDPKTPPSLHCCYGWTE-----------------------------
SOLLC    --GSAHQKSLPSLHCCYGWTE-----------------------------
ORYSJ*   LLEGSDNDNAASLHCCYGWTE-----------------------------
HORVU    LLEGSENDSGTSLHCCYGWTE-----------------------------
SORBI    LLDVSESDSAASLHCCYGWTE-----------------------------
SELML    --VSRTSSQPLSFHCCYSWTS-----------------------------
PHYPA    D-ASSAAQQAADLYCCYTWSE-----------------------------
                  4110      4120      4130      4140      4150
```

```
HUMAN*   ------------------DQRWILASCTDLYGELLETCIINIDVPNR---
CHICK    ------------------DQRWLLASCTDLYGEQLETCIINIDVPNR---
XENTR    ------------------DQRWLLASCTDLYGELLETCIINIDVPNR---
DANRE    ------------------DQRWLLASCTDQYGELLETCVISIDVPNR---
CIOIN    ------------------DQKFVLASATDQCGELLETCCINIDVPPRRLS
ANOGA    ------------------DQSWLLAVATDDRGEFLETVTINIDIPNR---
TETNG    ------------------DQRWLLASCTDQHGELLETCIISIDVPSR---
DROME    ------------------DQAWLLATATDERGEMLEKICINIDVPNR---
BRUMA    ------------------GNDWLAATVTDHQGHSLDNCLINLRLKPDHKR
CAEEL    ------------------GTDFLVATATDAQGKLIDNCISNIK-PRRQSN
YEAST*   ------------------DKEWVFAALSDSYGQGSMTKTWYVGNSRG---
SCHPO    ------------------EEHLLICNWNDSYGEFETERRYFLQDL-----
CANAL    ------------------DNSWFVASWSDPLGRVVHVKSWYCSDSVSKSE
CANGA    ------------------DKQWMFAALSDSAGKENMMKTWYLGSSKN---
ASPNI    ------------------DQRWMTVAWSDNSGALQRTMSYCLRFRNSTAS
RHIO9    ------------------DKRWMIIVWTDNNGELIEFATLDSQSQYR---
CRYNE    ------------------GPGALVVWVGDDRGETGHVRVF----------
USTMA    SISELGHLADDDAPGPAMREKVVMVSAIDERGGSSTVDALAANDGDG---
ARATH*   ------------------DWRWLVSIWTDAR-------------------
VITVI    --------------------------------------------------
SOYBN    ------------------DWRWLVCIWTDSRGELLDCNIFPFGGISSRQD
SOLLC    ------------------DWRWLVCIWTDSRGELLDNHIYPFGGISSRQD
ORYSJ*   ------------------DWRWLVCIWTDSKGELLDSLIFPFGGISSRQD
HORVU    ------------------DWRWLVCIWTDSRGELLDSLVFPFGGISSRQD
SORBI    ------------------DWRWLVCIWTDARGELLDSLIFPFGGISSRQD
SELML    ------------------NWLWLIAVWIDSQGKMLDVN------------
PHYPA    ------------------DWNWLVSVWTDARGELLDTLLLPLTGIDTTRE
                  4160      4170      4180      4190      4200
```

MED13

```
HUMAN*   --ARRKKSSARKFGLQKLWEWCLGL-------VQM-SSLPWRVVIGRL-G
CHICK    --ARRKKGSARRLGLQKLWEWCLGL-------VQM-SSLPWRVVIGRL-G
XENTR    --ARRKKGSARRCGLQKLWEWCQGL-------VQM-SSVPWRVVIGRL-G
DANRE    --ARRKKGSARRQGLQKLWEWCLDL-------VQM-TSLPWRIVIGRL-G
CIOIN    V-SRKHRVSVRSEAIEKLWKFCVTT-------VSRYSTTTWRIVIGES--
ANOGA    --GRRKRASARRYGLQKLMDFILGL-------ISQ-TVQPWRLVVGRI-G
TETNG    --ARRKNGNVRRVGLQKLWDWCLGL-------VQV-TSLPWRVVIGRL-G
DROME    --ARRRKAPARYVALKKLMDFIMGI-------ISQ-TSQMWRLVIGRI-G
BRUMA    VNMRYKQSTQIRDSLYRLWSYILGT-------LAN-GTRNWRLVIGRV-G
CAEEL    QVYRYRNKTQILDGMGKLWSFILGI-------MAS-ETKNWRLVVGRL-G
YEAST*   ---------KFDDACNQIWNIALNL-------ASK-KFGKICLILTRLNG
SCHPO    ---------EIEDALQQILEVTFTF-------LNS-MHMDWIVIVMKI-G
CANAL    KSTYRGDIMDIMSITDDIWSISTDLFKVLNDELGS-FGGKKFLVLTRINS
CANGA    ---------KFDEACNHIWEMALSL-------AGK-NYGKVCLILTRLNG
ASPNI    R--------TLSAVRSEIWAATRDI-------MDK-SQGRWRVIIVNT-D
RHIO9    ---------NMSQLFEEAWRRTKII-------SKR-TGFIWTFVIAKI-G
CRYNE    -----EDVGGWKEGMEKTCAYGREI-------AGR-WNLEWRLSVCRA-G
USTMA    ---------SIEACVERVWRFALAE-------ASR-AHLHWRLVISST-G
ARATH*   ----------VLQQGCQILQACSSP-------DNG-SFKPRDFVITRI-G
VITVI    --------------------------------------------------
SOYBN    TKGLQCLFVQILQQGCLILQSC----------DPG-LAKPRDFVIARI-G
SOLLC    TKGLQSLFVQILQQGCQILQSC-PP-------EAA-IAKPRDFVIARI-G
ORYSJ*   TKVLQSLFIQILQHGCQIMSS--AP-------ESS-NTRPRDVIITRI-G
HORVU    TKVLQSLFIQILQQGCQIMSS--SP-------EAS-NTRSRDVIITRI-G
SORBI    TKVLQSLFIQILQQGCQIMSS--SP-------EAS-NMRPRDVIITRI-G
SELML    --------------GQQLLATMSRT---------A-GNRGHTVTVTRM-G
PHYPA    N-CLRLLFDQILQQGLQLLNLAVEA---------G-SCRSRGLNITRL-G
                  4210      4220      4230      4240      4250
```

```
HUMAN*   ---------------RIGHGELKDWSCLLSRRNLQSLSKR----------
CHICK    ---------------RIGHGELKDWSCLLSRRNLQSLSKR----------
XENTR    ---------------RIGHGELKDWSCLLSRRNLQSLSKR----------
DANRE    ---------------RIGHGELRDWSILLSRRNLQSLGRR----------
CIOIN    -----------------------TFTVVVYYHALEGFHANNIPKPMGVRK
ANOGA    ---------------RIGHGELKGWSWLLSKPNLQRASKH----------
TETNG    ---------------RMGHGELRDWSILLSRRNLQSLSKR----------
DROME    ---------------RIGHSELKSWSFLLSKQQLQKASKQ----------
BRUMA    ---------------RIGHGEFKFWTQILSKSYLKQVGTR----------
CAEEL    ---------------RIGHGEFRAWTHLLNKTSLLRYSGS----------
YEAST*   ---------------ILPDDELMNWRRLSGRN------------------
SCHPO    ---------------EMSDAEYLFWDQAIIPENLQGNVSL----------
CANAL    ---------------IIPDVELVHWKRLSLKH------------------
CANGA    ---------------ILPDDELMNWRRLSGRN------------------
ASPNI    ---------------NVDQDEYDTWLNLADQYNKSRSMAI----------
RHIO9    ---------------LLFREELKIWINCIGQDE-----------------
CRYNE    ---------------IMAKEELQAWKDILK--------------------
USTMA    ---------------LMKQCELRAWQRLIDAYLSSSGAQE----------
ARATH*   NFFELEYQEWQKAIYSAGGPEIKKWPIQLRRSAPSGIATN----------
VITVI    --------------------------------------------------
SOYBN    GFYELEYLEWQKAIYSVGVSEMKRWPLQLRKSMSDGMSAT----------
SOLLC    CFLELECQEWQKALYSIGGSEVKKWSLQLRRSVPDGMTAS----------
ORYSJ*   GFLELEIQEWQKAIYSFGGNEVKKWPVQLRRSIPEGIPSN----------
HORVU    GFLELEIQEWQKAIYSFGGNEVKKWPVQLRRSIPDGIPSN----------
SORBI    GFLELEIQEWQKAIYSFGGNEVKKWPVQLRRSIPEGIPPS----------
SELML    VLYETEYQEWQRVFMNIAGDESRAWPVVIRPDSG----------------
PHYPA    GFYVRECQEWHRVIILAGADEMRKWPVQIRH--PDCYSSN----------
                  4260      4270      4280      4290      4300
```

```
HUMAN*   --LKDMCRMCG------------ISAADSPSILSACLVAMEPQGSF----
CHICK    --LKDMCRMCG------------ISAADSPSILSACLVAMEPQGSF----
XENTR    --LKDMCRMCG------------ISAADSPSILSACLVAMEPQGSF----
DANRE    --LKEMCRMCG------------ISAADSPSILSTCLVAMEPQASF----
CIOIN    GWIKQLAVVCDFKLFLYDMSDGGRGVTSQPSVIASQVIDMRDEQFA----
ANOGA    --LKDICEQCS-----------LMHPIAVPSILSACLVTLEPDSDL----
TETNG    --LKETCRMCG------------ISAADTPSILSACLVAMEPQGSF----
DROME    --FKDMCKQCT--------------LMYPPTILSACLVTLEPDAKL----
BRUMA    --MKHVCRACH----------VMPGTSEVPGVLSACLVSLEPEAHL----
CAEEL    --LKDICGACR----------SMPSVIGTPAILSACLITLEPEPSI----
YEAST*   --------------------------------IHLAVVCVDDNSKISFID
SCHPO    -------------------TVGYCSAEHGPGSTSKVFSRIPYSASS----
CANAL    ---KEISLIVVSVRQTPRIVGSNESEDLGPKSNDYAPTPMIQDKDIFFGF
CANGA    --------------------------------IHLAVVCVDDNTKISFFD
ASPNI    -----------ELAILSVNTAFDISLSPPPSPLSMSL--LNPQASS----
RHIO9    ---------------------------------KAAIVCLDLESPLNILP
CRYNE    -----------------------------GKTWPITLLTSEAPSPL----
USTMA    -----------------------------RVIASVVLLSVRPDESG----
ARATH*   ---------------------------SNGSSLQQQDLSLIQERA-----
VITVI    --------------------------------------------------
SOYBN    ---------------------------SNGSSLQQSDMSLIPERTL----
SOLLC    ---------------------------SNGTSLQQQEI------------
ORYSJ*   ---------------------------SNGPALQQQDMGLMQDRNM----
HORVU    ---------------------------SNGPTLQQQDMGLIQDRNM----
SORBI    ---------------------------SNGPTLQQQDMALIQDRNM----
SELML    ---------------------------GDGSSLSQ-------QRNV----
PHYPA    ---------------------------SVGGTGSSSDIGLMTDRGLGLAG
                  4310      4320      4330      4340      4350
```

MED13

```
HUMAN*  - - V I M P D S V S T - - - - - - - - - - - - - - - - - - - - G S V F G R S T T L N M Q T S Q - - L
CHICK   - - I I M P D S V S T - - - - - - - - - - - - - - - - - - - - G S V F G R S T T L N M Q T S Q - - L
XENTR   - - V I M P D S V S T - - - - - - - - - - - - - - - - - - - - G S V F G R S T T L T I Q T S Q - - L
DANRE   - - V I M P D S V S T - - - - - - - - - - - - - - - - - - - - G S V F G R S T T L N M Q T S Q - - L
CIOIN   - - V V P V T A V D V - - - - - - - - - - - - - - - - - - - - - - - - - - - - - I H A T K K E L P C
ANOGA   - - R V M S D Q F T P - - - - - - - - - - - - - - - - - - - - - - - - - - D E R F S Q R S M Q S P L
TETNG   - - V I M P D S V S T - - - - - - - - - - - - - - - - - - - - G S V F G R S T T L N M Q T S Q - - L
DROME   - - R V M P D Q F T P - - - - - - - - - - - - - - - - - - - - - - - - - - D E R F S Q I S M Q N P L
BRUMA   - - R V F P S S F Q H - - - - - - - - - - - - - - - - - - - - - - - - - - D D R F L K N S R H R A L
CAEEL   - - R I M P E - - - - - - - - - - - - - - - - - - - - - - - - - - - F H D Q E L S T K K S F L - - F
YEAST*  E D K L Y P S F K P I - - - - - - - - - - - - - - Y K D - T R F G G R M D M T R L Y D Y E I R - - D
SCHPO   - - T V I R N N S S H - - - - - - - - - - - - - - - - - - - - - - - - - - - - - - - - - - - - - - -
CANAL   K Q T F S T S N T S S - - - - - - - - - - - - - - - P S A S G G A L V T S P N S L S L H S P Q Q F L
CANGA   R N E S Y P S Y K R L - - - - - - - - - - - - - - Y Q N A N S I E T L V D P E R I D D Y L I R - - D
ASPNI   - - T P I A T P V P S - - - - - - - - - - - - - - - - - - - - - - - - - - G S I L S P D G P G N A P
RHIO9   I P S A P M T E S M A S S S M M D S I G T N V P S I V N S T S N V G G I G A G F N S K K S S F - - L
CRYNE   - - - - N P S Y Q K T - - - - - - - - - - - - - - - P P K V R A I A N I G P S I L S D S S T R - - I
USTMA   - - A V L A E R G V G - - - - - - - - - - - - - - - - - I K A R Q E W T A A S T A D K G S L M - - L
ARATH*  - - - S S T S T L Y S - - - - - - - S H S K Q S T F V K G S M G Q S A G R K Q I M G G Q T I S - - G
VITVI   - - - - - - - - - - - - - - - - - - - - - - - - - - - - - - - - - - - - - - - - - - - - - - - - - -
SOYBN   - - P S S P S P L Y S - - - - - - - P H T K S P G F M K G S L G Q P T A R K Q L I G G H S M V - - D
SOLLC   - - - - - - - - - - - - - - - - - - - - - - - - - - - - G G L G Q P S T R K Q L I G G Q G V V - - D
ORYSJ*  - - P S S P S P L Y S - - - - - - - P H A K S - S F M K G A L G Q S G N K K Q I L V E Q A G M - - D
HORVU   - - P S S P S T L Y S - - - - - - - P H S K S - S F T K - - - G Q P G N K K Q I L A E Q T G M - - D
SORBI   - - P S S P N P L Y S - - - - - - - P H S K S - S F M K G A L G Q S G N K K Q I L V E Q T G M - - D
SELML   - - S A V V G F P S S - - - - - - - P G S - S S S F V S R G L - - - - N P K P E I N F H A G K - - -
PHYPA   S G P T S P S P S S S S F G G R G K P S H Y S K S G A E M S S G M N R L Q N Q A T N Q M A A T - - E
                            4360                4370                4380                4390                4400
```

```
HUMAN*  N T P Q D T S C T H I L V F P T S A S V Q V A S A T Y T T E - - - N L D L A F N P N N - D G A D G M
CHICK   N T P Q D T S C T H I L V F P T S A S V Q V A S S T Y T T E - - - N L D L A F N T N N - D G A D G M
XENTR   N T P Q D T S C T H I L V F P T S A S V Q V A S A T Y T T E - - - N I D L A F N T N N - - A K N V M
DANRE   N T P Q D T S C T H I L V F P T S A G L Q A F S D V T - - - - - - - - - - - - - - - - - D G M G G M
CIOIN   I F R V V A S C M H R P V L R S Q V L L L A E S E T A K S K - - - - - - - - - - - - - - - W V G A L
ANOGA   S T P Q D A T C T H I L V F P T S A K A Q A - S F S V I G E L D L G E D L N M V I M D G D D D D D G
TETNG   S T P Q D T S C T H I L V F P T S A M V Q V - - N T N T S E - - - P I D I N F N P I N P D G S D G M
DROME   A T P Q D V T C T H I L V F P T S A V C A P F T R Q F Q N E P Q V D D D F L T F E E E G N E D F S D
BRUMA   T T P D D T S C T H I L V F P T S P E L N L D H Q G G N G V A E L E E D - - - - - - - - - - - - - -
CAEEL   Q T P G D L S C T H I L T F P V G T E I N L E V Q D Q T A D T K A D E N W E F - - - - - - - - - - -
YEAST*  I D Q D I H G I V F Q H P F P L - - - - - - - - - - - - - - - - - - - - - - - - - - - - - - - - - -
SCHPO   - E L S L V A F I R E M A M P V P N D - - - - - - - - - - - - - - - - - - - - - - - - - - - - - - -
CANAL   N A P A N F L S P Q D L I A P T S L G I S G - - - - - - - - - - - - - - - - - - - - - - - - - - - -
CANGA   L D Q D I H G V I F E N P F P L - - - - - - - - - - - - - - - - - - - - - - - - - - - - - - - - - -
ASPNI   T P P S G A N A A N N A L T P T D S L L E S E S E S L L V D - - - - - - - - - - - - - - - - - - - -
RHIO9   A D E N G V G Q T K A L L L N H R V A Y S S K R - - - - - - - - - - - - - - - - - - - - - - - - - -
CRYNE   I D T A L S A Q L T V L P L P L P L E L S S - - - - - - - - - - - - - - - - - - - - - - - - - - - -
USTMA   L D A A D L S Q M V C F A E P M P M E W T Q - - - - - - - - - - - - - - - - - - - - - - - - - - - -
ARATH*  T P R G L F Q W V H S I S F A S I S L D H S L H F V L P A E - - - - - - - - - - - - - - - - - - - -
VITVI   - - - - - - - - - - - - - - - - - - - - - - - - - - - - - - - - - - - - - - - - - - - - - - - - - -
SOYBN   N S R G L L H W A Q S I S F V A V S M D H T L Q L V L P A D - - - - - - - - - - - - - - - - - - - -
SOLLC   N S R G L L Q L V Q S I S F V S L S I D H S L Q L M I Q A D - - - - - - - - - - - - - - - - - - - -
ORYSJ*  S S K G S L H L V R S I S L V A I S Q D H S L H L T C Q A D - - - - - - - - - - - - - - - - - - - -
HORVU   S S R G S L H L V R S I S L V A V S Q D S S L H L A C Q A D - - - - - - - - - - - - - - - - - - - -
SORBI   T S R G P L H L V R S I S L V A V S Q D H S L H L T C Q T D - - - - - - - - - - - - - - - - - - - -
SELML   - - - - - - - - L A G V T L A T V S G D E S L Q V L S L A E - - - - - - - - - - - - - - - - - - - -
PHYPA   P N S G G F K W V H S I S L V S V R V D Q S L Q I V S T T D - - - - - - - - - - - - - - - - - - - -
                            4410                4420                4430                4440                4450
```

```
HUMAN*  - G I F D L L D T G D D L D P D I I N I L P A S P T G S P V H S P G S H Y P H G G D A G K - - - - -
CHICK   - G I F D L L D T G D D L D P D I I N I L P A S P T G S P V H S P G S H Y P H G G D M G K - - - - -
XENTR   - F T A V C F D T G D D L D P D I I N N L P T S P T G S Q G H S P G S H Y P H G G D A G K V F T F L
DANRE   D G I L D L F A E N D L V D P D L I N I I P N S P T T S P V H S P G S H Y H H G G D G S K - - - - -
CIOIN   S E V L R I L R K H Q L R D K S I Y R P L E T Y D P T L P M I - - - - - - - - - - - - - - - - - - -
ANOGA   I N M M D V F K C W D D L P M Q Q I N - M P H S R P G S P S Q F E G N Q Q S P G E S G S K - - - - -
TETNG   - G I F D L F D N - D M V D P D L I N I L P N S P T T S P V H S P G S H Y H H G G D G S K - - - - -
DROME   A D I G D L F - - - W D T H M D R V - - - - - S N H G S P G R M D D N R S W Q S A - - - - - - - - -
BRUMA   - - - - - - - - - - - - - D F S N L L T E E I G E E F S E L I G N G E D L M T N G T G P P P Q S A S
CAEEL   - G D L D I M E G L D D G D T E I M K D L G L E T P S S A A I R - - - - - - - - - - - - - - - - - -
YEAST*  - - - - - - - - - - - - - - - - - - - - - - - - - - - - - - - - - - - - - - - - - - - - - - - - - -
SCHPO   - - - - - - - - - - - - - - - - - - - - - - - - - - - - - - - - - - - - - - - - - - - - - - - - - -
CANAL   - - - - - - - - - - - - - - - - - - - - - - - - - - - - - - - - - - - - - - - - - - - - - - - - - -
CANGA   - - - - - - - - - - - - - - - - - - - - - - - - - - - - - - - - - - - - - - - - - - - - - - - - - -
ASPNI   - - - - - - - - - - - - - - - - - - - - - - - - - - - - - - - - - - - - - - - - - - - - - - - - - -
RHIO9   - - - - - - - - - - - - - - - - - - - - - - - - - - - - - - - - - - - - - - - - - - - - - - - - - -
CRYNE   - - - - - - - - - - - - - - - - - - - - - - - - - - - - - - - - - - - - - - - - - - - - - - - - - -
USTMA   - - - - - - - - - - - - - - - - - - - - - - - - - - - - - - - - - - - - - - - - - - - - - - - - - -
ARATH*  - - - - - - - - - - - - L V - - - - - S A G G G Q - S S T G M S S V N Y I E - G F T P V K S L G S T
VITVI   - - - - - - - - - - - - - - - - - - - - - - - - - - - - - - - - - - - - - - - - - - - - - - - - - -
SOYBN   - - - - - - - - - - - - - - - - - - - - - - - - - - - - - - S S T P G Y I E - G F T P V K S L G S T
SOLLC   - - - - - - - - - - - - - - - - - - S T S H G T S Q S S G I M S Q P G Y L E - G Y T P V K S L G S T
ORYSJ*  - - - - - - - - - - - - L L - T R P T P G E G N Q - T S S G P S S - - Y L E - G F T P V K S I G S M
HORVU   - - - - - - - - - - - - L L A T R P T S G E G N Q - S S T G S S S - - Y L D - G F A P V K S I G S M
SORBI   - - - - - - - - - - - - L L - T R P A S G E G N Q - G S S G L S S - - Y L E - G F T P V K S I G S M
SELML   - - - - - - - - - - - - - - - - - - - - - S A A A S T W S V A S S S G L A S - G F G M A K T I A S L
PHYPA   - - - - - - - - - - - - G P P E G N S S S G P S W P A G S G S A S S G V V S M G V N A V K T L A L T
                            4460                4470                4480                4490                4500
```

MED13

```
HUMAN*  -GQSTDRLLSTEPHEEVPNILQQ---------------------------
CHICK   -GQGTDRLLSTESHDEVTNILQQ---------------------------
XENTR   CGQGTDRLLSTESHDEVTNILQQ---------------------------
DANRE   -GQGTDRM---ESHDESPNILQQ---------------------------
CIOIN   -----------KSSMSASVIDQD---------------------------
ANOGA   -GAGSRDGYGSQDSEEVGLVLQQ---------------------------
TETNG   -GQSADRM---ESHEEALNILQQ---------------------------
DROME   -GGNNFKCTPPQEVEEVGSLNQQ---------------------------
BRUMA   FRLGGNTGIPSEYAD--VSIENQ---------------------------
CAEEL   -QTGGPSMFFSEDSSSI-EIQNQ---------------------------
YEAST*  --------------------------------------------AHSQHR
SCHPO   --------------------------------------------EFKKIS
CANAL   -AKPGSSGIDPEVVLENQDNEVY------------GVIPKVPLPSFNSPTR
CANGA   --------------------------------------------VNSLHR
ASPNI   --------------------------ICDESWCVLLSHRLNGNLHLTEYR
RHIO9   -ERASLAILSMDPSS-----------------------------NTENWM
CRYNE   -DKGSPQIIYPRRTFLLTTPALD---------------------------
USTMA   -ASGGWAAIGEEDEDAEQDGDEK-----------------------EATA
ARATH*  AF--SYMMIPSPNMRFLHPSPLQ-LPTCLTAESPPLAHLL----HSKGYA
VITVI   --------------------------------------------------
SOYBN   SS--AYILIPSPSMRFLPTTVLQ-LPTCLTAESPPLAHLL----HSKGSA
SOLLC   ST--SYILIPSPSMRFLPPVSLQ-LPTCLTAESPPLAHLL----HSKGCA
ORYSJ*  SA--SYLLVPSPSMRYLSPATLQ-LPTCLTSESPPLAHLL----HSKGTA
HORVU   SA--SYLLVPSPSMRYLSPATLQ-LPTCLTSESPPLAHLL----HSKGTA
SORBI   PASHSYLLVPSPSMRYLSPATLQ-LPTCLTSESPPLAHLL----HSKGTA
SELML   NG--AYLFIPAHGLRSLSSSDSV---ASNKVNAPAFGQWI----QSRGSV
PHYPA   GA--SYLVVPSHVNRPLSSSSLELLLETPYSEQSPLAQLL----QSSGVA
                 4510      4520      4530      4540      4550
```

```
HUMAN*  -PLALG-----YFV-----STAKAGPLPDWFWSACPQAQYQCPL------
CHICK   -PLALG-----YFV-----STAKAGPLPDWFWSACPQAQNQCPL------
XENTR   -PLALG-----YFV-----STAKAGPLPDWFWSACPQAQNQCPL------
DANRE   -PMALG-----YFV-----STAKAGPLPDWFWSACPQAKNQCPL------
CIOIN   -RVALGTEEGLYVLEFRTDEIVKVGDAKKIYAIEVIPQEDMIAV------
ANOGA   -PLAIG-----YLV-----STAPTGRMPEWFWSSCPHMENVCPV------
TETNG   -PMALG-----YFV-----STAKTGPLPDWFWSACPQAQNQCPV------
DROME   -PISVG-----YMV-----STAPTGRMPAWFWSACPHLEDVCPV------
BRUMA   -PLATG-----YYI-----STAPASDLPEWFWSACPSAKRRNPV------
CAEEL   -PLASG-----YYI-----STAPAPELPAWFWATCPSAKRHSPV------
YEAST*  CAIRSG-----ALIKFKKCDGDT---VWD---------------------
SCHPO   TILARG-----YLALDEDESYLP---------------------------
CANAL   FCMKTG-----YLM-----------------MQVNQPSEEEYKDDIKKTY
CANGA   CAIKSG-----ALVKFNVSTTATEPHSLD---------------------
ASPNI   PALASG-----YLVRRRGTTDADGAY------------------------
RHIO9   IPLASG-----YTIYTPLPTENSRHGLFNI--------------------
CRYNE   --VSQH-----YTTKYHILSHQTPDGRKD---------------------
USTMA   MPLASA-----ILVHRTRPDLVGGGGRTS---TSSETKHKHCPDGFTPYS
ARATH*  IPLSTG-----FVV-----SKAVPSMRKD---SRIN-VKEEWPS------
VITVI   --------------------------------------------------
SOYBN   LPLSTG-----FVV-----SKAVPSMRKD---YRAN-QKEEWPS------
SOLLC   IPLSTG-----FVV-----SKAVPTMRRD---VRSI-SKEEWPS------
ORYSJ*  IPLAMG-----YVV-----SKAVPPVRKD---SARL-ANEDRPS------
HORVU   TPLAMG-----YVV-----SKAVPPVRMN---AAQL-TKEDRHS------
SORBI   IPLAMG-----YVV-----SKAVPPVRRD---SAQL-TKDEQPS------
SELML   SPVASG-----YVI-----SGPTTALRAE---VAQVSMEEEWPS------
PHYPA   FPVSTA-----YAV-----SAGTSSTIND---FAQKGMKEDWPS------
                 4560      4570      4580      4590      4600
```

```
HUMAN*  -FLKASLHLHVPSV--QSDELLHSKHS-----------------------
CHICK   -FLKASLHLHVPSV--QSDELLHSKHS-----------------------
XENTR   -FLKASLHLHVPSV--QSDELLHSKHS-----------------------
DANRE   -FLKASLHLNVSSV--QSDELLHSKHS-----------------------
CIOIN   -ISGRNRHIRL---------------------------------------
ANOGA   -FLRTALHLHSPTILQNTDDPLQQNQSSTE--------------------
TETNG   -FLKASLHLHVSSV--QSDELLHSKHS-----------------------
DROME   -FLKTALHLHVPSI-QSADDILNSTNAHQSGND-----------------
BRUMA   -HLKSSLHLNTPNV--QQGDDISSFGKGTEACH-----------------
CAEEL   -HLKSSLHIHISEV--KNDDIAMESTKEKEKDKEKDEKDA----------
YEAST*  -KFAVNL-------------------------------------------
SCHPO   -LLSIHL-------------------------------------------
CANAL   LVYEINL-------------------------------------------
CANGA   -KFEVNL-------------------------------------------
ASPNI   -TMNVNLLF-----------------------------------------
RHIO9   --------------------------------------------------
CRYNE   --------------------------------------------------
USTMA   HVLAVDMLQVWRLEAAQQTSV-----------------------------
ARATH*  -VLSVSLIDYYGGY-DNAHDKILQGIV-------------KQGGG--TKE
VITVI   --------------------------------------------------
SOYBN   -VLSVSLIDYYGGT-NIPQEKIVRGIN-------------KQGGRSLSWE
SOLLC   -VLSVSLVDYYGGS-NVIHEKFLKGVG-------------KVGGRGTGSE
ORYSJ*  -VLSVSIIDHYGGSIAAVQEKMSRGSG--------------KQTRNFTQE
HORVU   -VLSVSIVDYYGGGTATVQEKMSRGVG---------GSTTSKQARNISHE
SORBI   -VLLVSIIDHYGGSIATVQEKMSRGGG---------GSNMSKQARNFTQE
SELML   -TLQIGLVAHFGSSKARQETQGMRLGGNGLCDNAGSSSNTGGSSAGTGKQ
PHYPA   -TMSVGLVAHYGTS---------SRLGTPVQDSAGVAMSKSFKSTVVE--
                 4610      4620      4630      4640      4650
```

MED13

```
HUMAN*  --HPLDSNQTSDVLRF-----VLEQYNALSWLT-------------CDPA
CHICK   --HPLDSNQTSDVLRF-----VLEQYNALSWLT-------------CDPA
XENTR   --HPLDSNQTSDVLRF-----VLEQYNALSWLT-------------CDPA
DANRE   --HPLDSSHTSDVLRF-----VLEQYNALSWLT-------------CDPA
CIOIN   --HSLSSLEGGDGEAI-----KIEESKGCNAVT-------------SGPF
ANOGA   --HPLDSNITADVLRY-----VLEGYNLLSWLA-------------MDSN
TETNG   --HPLDSNHTSDVLRF-----VLEQYNALSWLT-------------CDPA
DROME   --HPLDSNLTADVLRF-----VLEGYNALSWLA-------------LDSN
BRUMA   --HQLDSQATADVLRY-----VLEMYNALSWLN-------------MDLV
CAEEL   --HPLESRQTEEVLRH-----VLESYNALSWLN-------------LNRQ
YEAST*  --LNCPHSDSTQLLET-----ILEEFRNLAALN-----------------
SCHPO   --LISRNHDPYLMLNL-----ILKHYLSMIYLQ-----------------
CANAL   --LSCSNYWNLDVLMK----LIMLQYKQMIALNDILCMNPIVGELDILGT
CANGA   --LNCPHSDSFKLLET-----ILEEFRNLAALN-----------------
ASPNI   --TQRPSTHYETTLRE-----IIGMYRDLATLA-----------------
RHIO9   --FNQTSHSAYSTLRD-----IIQKFHALSYVN-------------IMSS
CRYNE   -------QEDGGIVAE-----IAEEYHRLGCLA-----------------
USTMA   --QNKEAKEAEEALRSGALDGIMRSLHRLRLIS-----------------
ARATH*  --TRDFEVESHLILES-----IAAELHALSWMT-------------VSPA
VITVI   --------------------------------------------------
SOYBN   --AKDFEIETHLVLES-----LAAELHALSWMT-------------VSPT
SOLLC   --TRDVEIAAHLILEN-----IAAELHALSWMT-------------VSPA
ORYSJ*  AAGRDHETEMHNVLEA-----VAAELHSLSWMT-------------VSPV
HORVU   TSARDYEMDMHNVLEA-----VAAELHALSWMT-------------VSPV
SORBI   TTTRDCEVEMHNVLEA-----VATELHSLSWLT-------------VSPV
SELML   GSSSGIKQQIRYVLEQ-----LGAEFYGLSWLT-------------ISPM
PHYPA   --SKEQALEVHRVLQA-----VASDLHALSWLN-------------VGLT
                          4660      4670      4680      4690      4700
```

```
HUMAN*  TQDR------RSCLPIHFV-------------------------------
CHICK   TQDR------RSCLPIHFV-------------------------------
XENTR   TQDR------RSCLPVHFV-------------------------------
DANRE   TQDR------RSCLPVHFV-------------------------------
CIOIN   RQGS------TTCLCVAIKKHVFVYEMNNTKLRHKRIRDIMLPSPAQWLC
ANOGA   THDR------LSCLPIHVQ-------------------------------
TETNG   TQDR------RSCLPVYFV-------------------------------
DROME   THDR------LSCLPINVQ-------------------------------
BRUMA   SGER------RSCLPIHIQ-------------------------------
CAEEL   TGDR------YSCLPIHIQ-------------------------------
YEAST*  -VWYGLSDGEDGHIPWHIL-------------------------------
SCHPO   ----FRTYVSFSSLPLHIS-------------------------------
CANAL   HSRN------NAIVPWHIN-------------------------------
CANGA   -VWFGITNGENGHIPWHVL-------------------------------
ASPNI   -RVRGTRIVQTGTLPWHIA-------------------------------
RHIO9   NSNR---------LPIHLA-------------------------------
CRYNE   -RERWEGQGWLDSVRWGRA-------------------------------
USTMA   -QERHQLPWPYNAQPWCVA-------------------------------
ARATH*  YLDR------RTALPFHCD-------------------------------
VITVI   --------------------------------------------------
SOYBN   YLER------RTALPFHCD-------------------------------
SOLLC   YLER------RSALPLHCD-------------------------------
ORYSJ*  YMER------RSALPSHCD-------------------------------
HORVU   YTER------RSALPFHCD-------------------------------
SORBI   YTER------RTALPFHCD-------------------------------
SELML   YLHR------RRPLPYHCD-------------------------------
PHYPA   YVKR------RSPLPFHCE-------------------------------
                          4710      4720      4730      4740      4750
```

```
HUMAN*  --------------------------------------------------
CHICK   --------------------------------------------------
XENTR   --------------------------------------------------
DANRE   --------------------------------------------------
CIOIN   VMNERLCAGYVSGFALFSIQGNGKPIPLMYYDDGSLAFLYQPPVDALCAV
ANOGA   --------------------------------------------------
TETNG   --------------------------------------------------
DROME   --------------------------------------------------
BRUMA   --------------------------------------------------
CAEEL   --------------------------------------------------
YEAST*  --------------------------------------------------
SCHPO   --------------------------------------------------
CANAL   --------------------------------------------------
CANGA   --------------------------------------------------
ASPNI   --------------------------------------------------
RHIO9   --------------------------------------------------
CRYNE   --------------------------------------------------
USTMA   --------------------------------------------------
ARATH*  --------------------------------------------------
VITVI   --------------------------------------------------
SOYBN   --------------------------------------------------
SOLLC   --------------------------------------------------
ORYSJ*  --------------------------------------------------
HORVU   --------------------------------------------------
SORBI   --------------------------------------------------
SELML   --------------------------------------------------
PHYPA   --------------------------------------------------
                          4760      4770      4780      4790      4800
```

MED13

```
HUMAN*   --------------------------------------------------
CHICK    --------------------------------------------------
XENTR    --------------------------------------------------
DANRE    --------------------------------------------------
CIOIN    DVSTKECLLCFATCGVYVTWDGRRSRQQELMWPAPPTAIVYNRPYLVVYS
ANOGA    --------------------------------------------------
TETNG    --------------------------------------------------
DROME    --------------------------------------------------
BRUMA    --------------------------------------------------
CAEEL    --------------------------------------------------
YEAST*   --------------------------------------------------
SCHPO    --------------------------------------------------
CANAL    --------------------------------------------------
CANGA    --------------------------------------------------
ASPNI    --------------------------------------------------
RHIO9    --------------------------------------------------
CRYNE    --------------------------------------------------
USTMA    --------------------------------------------------
ARATH*   --------------------------------------------------
VITVI    --------------------------------------------------
SOYBN    --------------------------------------------------
SOLLC    --------------------------------------------------
ORYSJ*   --------------------------------------------------
HORVU    --------------------------------------------------
SORBI    --------------------------------------------------
SELML    --------------------------------------------------
PHYPA    --------------------------------------------------
                   4810      4820      4830      4840      4850
```

```
HUMAN*   --------------------------------------------------
CHICK    --------------------------------------------------
XENTR    --------------------------------------------------
DANRE    --------------------------------------------------
CIOIN    ENAIDIFDPASMEWLQTIPLKKVRPLSTDGSLNLACSMEPPKLVHLKNKY
ANOGA    --------------------------------------------------
TETNG    --------------------------------------------------
DROME    --------------------------------------------------
BRUMA    --------------------------------------------------
CAEEL    --------------------------------------------------
YEAST*   --------------------------------------------------
SCHPO    --------------------------------------------------
CANAL    --------------------------------------------------
CANGA    --------------------------------------------------
ASPNI    --------------------------------------------------
RHIO9    --------------------------------------------------
CRYNE    --------------------------------------------------
USTMA    --------------------------------------------------
ARATH*   --------------------------------------------------
VITVI    --------------------------------------------------
SOYBN    --------------------------------------------------
SOLLC    --------------------------------------------------
ORYSJ*   --------------------------------------------------
HORVU    --------------------------------------------------
SORBI    --------------------------------------------------
SELML    --------------------------------------------------
PHYPA    --------------------------------------------------
                   4860      4870      4880      4890      4900
```

```
HUMAN*   --------------------VLNQLYNFIMNML-----------------
CHICK    --------------------VLNQLYNFIMNML-----------------
XENTR    --------------------VLNQLYNFIMNML-----------------
DANRE    --------------------VLNQMYNFIMNML-----------------
CIOIN    DDFVWFAVAATIVDMNKSALLLLLVIGLLVSIETTNADTAGASALALTRR
ANOGA    --------------------VLMQLYHMTAALA-----------------
TETNG    --------------------VLTQMYNFIRNTL-----------------
DROME    --------------------TLMDLYYLTAAIA-----------------
BRUMA    --------------------ALMWLCNAVNRFIN----------------
CAEEL    --------------------HLLRLYHSVARLLA----------------
YEAST*   --------------------AVKKMMNTLVHTRVKIANTSAATVHTATSS
SCHPO    --------------------TVLYQKQLLQFMASDITHPVTS--------
CANAL    --------------------AVGKLLDYLVHIYVDKD-------------
CANGA    --------------------AVKKMMKTLVHTRVKVAQ------------
ASPNI    --------------------TAMRAQEILSYVL-----------------
RHIO9    --------------------LVERLSRILLVVNT----------------
CRYNE    --------------------LLEKR-------------------------
USTMA    --------------------SVNTLAACLDGIVLRD--------------
ARATH*   --------------------MVLRLRRLLHFADK----------------
VITVI    --------------------------------------------------
SOYBN    --------------------MVLRLRRLLHFADK----------------
SOLLC    --------------------MVLRLRRLLHFADK----------------
ORYSJ*   --------------------MVLRLRRLLHYADR----------------
HORVU    --------------------MVLRLRRLLHYADR----------------
SORBI    --------------------MVLRMRRLLHYADK----------------
SELML    --------------------VAQRLEKLLGYMEAAAELSPTATTATAAAT
PHYPA    --------------------VAQRSQRLLDFLDAEHRHGIGFILRKTFQR
                   4910      4920      4930      4940      4950
```

MED13

```
HUMAN*  --------------------------------------------------
CHICK   --------------------------------------------------
XENTR   --------------------------------------------------
DANRE   --------------------------------------------------
CIOIN   RSTPTPWTGRWNQRQNVEEMMEELNDDPEYFMDN----------------
ANOGA   --------------------------------------------------
TETNG   --------------------------------------------------
DROME   --------------------------------------------------
BRUMA   --------------------------------------------------
CAEEL   --------------------------------------------------
YEAST*  SIILSDK-------------------------------------------
SCHPO   --------------------------------------------------
CANAL   --------------------------------------------------
CANGA   --------------------------------------------------
ASPNI   --------------------------------------------------
RHIO9   --------------------------------------------------
CRYNE   --------------------------------------------------
USTMA   --------------------------------------------------
ARATH*  -------------------EVSRIPDKTGV--------------------
VITVI   --------------------------------------------------
SOYBN   -------------------ELSKQSEKS----------------------
SOLLC   -------------------EVSRQPVKSQV--------------------
ORYSJ*  -------------------HLSQSTEKGDVA-------------------
HORVU   -------------------HLSQLTVKGEM--------------------
SORBI   -------------------YLSQPAEKGETT-------------------
SELML   AS------------------------------------------------
PHYPA   IWRVATLIFEHVLIARYQIPKDVIPVIVSTLPKYKGSGNMTKRIFWGGEV
                 4960      4970      4980      4990      5000
```

```
HUMAN*  --------------------------------------------------
CHICK   --------------------------------------------------
XENTR   --------------------------------------------------
DANRE   --------------------------------------------------
CIOIN   --------------------------------------------------
ANOGA   --------------------------------------------------
TETNG   --------------------------------------------------
DROME   --------------------------------------------------
BRUMA   --------------------------------------------------
CAEEL   --------------------------------------------------
YEAST*  --------------------------------------------------
SCHPO   --------------------------------------------------
CANAL   --------------------------------------------------
CANGA   --------------------------------------------------
ASPNI   --------------------------------------------------
RHIO9   --------------------------------------------------
CRYNE   --------------------------------------------------
USTMA   --------------------------------------------------
ARATH*  --------------------------------------------------
VITVI   --------------------------------------------------
SOYBN   --------------------------------------------------
SOLLC   --------------------------------------------------
ORYSJ*  --------------------------------------------------
HORVU   --------------------------------------------------
SORBI   --------------------------------------------------
SELML   --------------------------------------------------
PHYPA   GRRVRRDRRSLSIHDQSNVVLSGCKGGHQGRKRDKSSGIVDRKKMRIESK
                 5010      5020      5030      5040      5050
```

```
HUMAN*  -
CHICK   -
XENTR   -
DANRE   -
CIOIN   -
ANOGA   -
TETNG   -
DROME   -
BRUMA   -
CAEEL   -
YEAST*  -
SCHPO   -
CANAL   -
CANGA   -
ASPNI   -
RHIO9   -
CRYNE   -
USTMA   -
ARATH*  -
VITVI   -
SOYBN   -
SOLLC   -
ORYSJ*  -
HORVU   -
SORBI   -
SELML   -
PHYPA   M
```

MED14

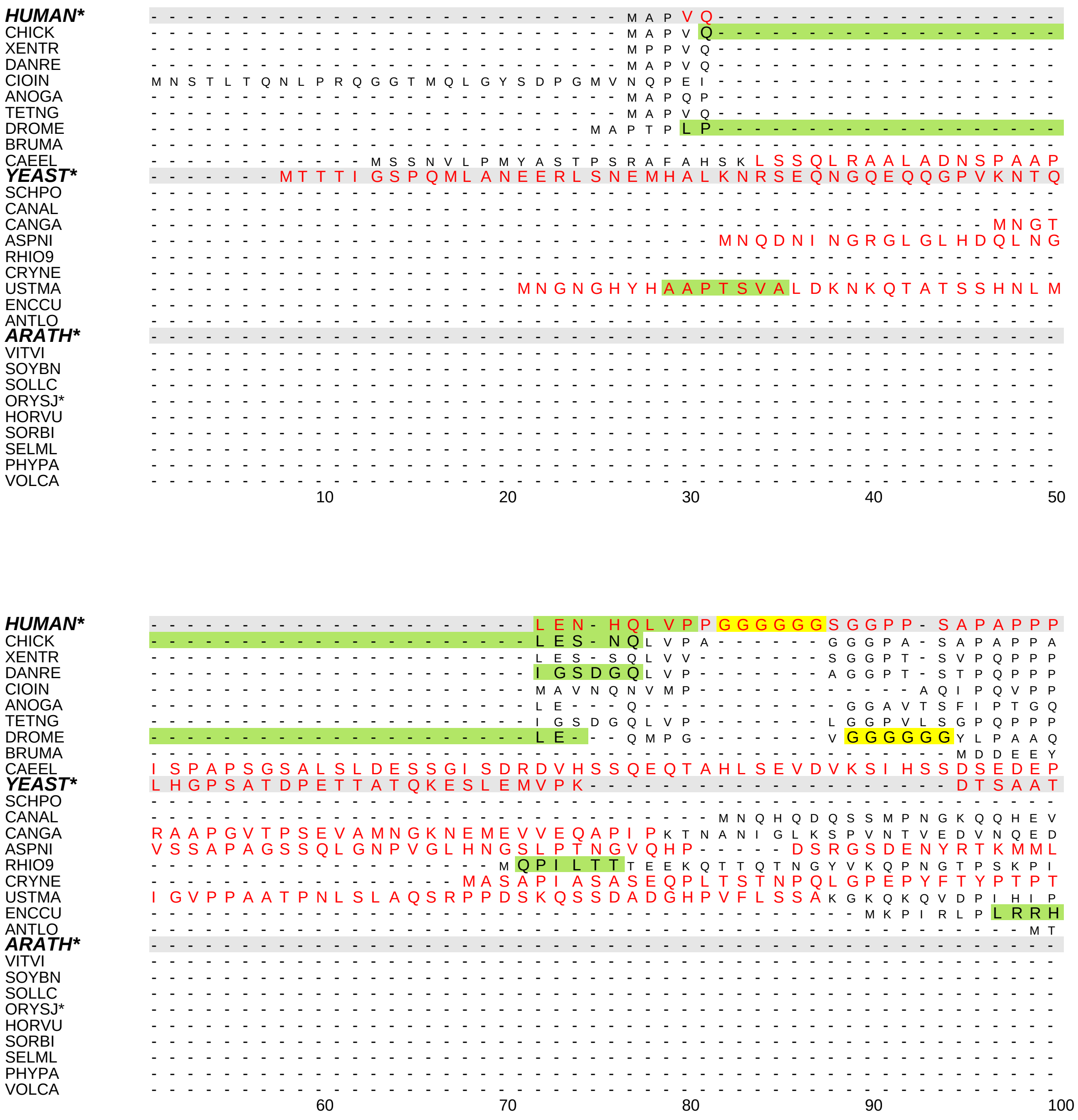
HUMAN*
CHICK
XENTR
DANRE
CIOIN
ANOGA
TETNG
DROME
BRUMA
CAEEL
YEAST*
SCHPO
CANAL
CANGA
ASPNI
RHIO9
CRYNE
USTMA
ENCCU
ANTLO
ARATH*
VITVI
SOYBN
SOLLC
ORYSJ*
HORVU
SORBI
SELML
PHYPA
VOLCA
10
20
30
40
50
60
70
80
90
100

MED14

```
HUMAN*   PGAAVAAAAAAAASPGYRLSTLIEFLLHRAYSELMV-LTDLLP-------
CHICK    PG-----AVTAAASPGYRLSTLIDFLLHRTYAELTV-LADLLP-------
XENTR    PGTVV---ASTAASHGYRLSTLIEFLLQRTYQELNV-LADLLP-------
DANRE    -G--------APATQGVRLSLLIEFLLQRTYHEITL-LAELLP-------
CIOIN    GQ--------MQGGGTIPLSLLVDFVVHKTYHDITV-MADLLP-------
ANOGA    EM--------AQRQNLIPLGRLIDFIIQRTYHELTV-LAELLP-------
TETNG    TG--------GGATQGVRLSLLIEFLLQRTYHEITL-LAELLP-------
DROME    EG--------GPRINTMSMSVLIDFIIQRTYHELTV-LAELLP-------
BRUMA    MKPMLPTVPEKCGPPVIPLGHLIEFAVQQIFHELTV-LSELLP-------
CAEEL    AQNPLPEVPANCGPPTIPLNVLLDFAIQHVYHEITV-LAELMQ-------
YEAST*   MTSAPPPALPHVEINQVSLALVIRNLTVFTMKELAQYMKTNVH-------
SCHPO    ---MEPPAIPHITEGFYPLPEIVETFSHHVLQELVS-LAEVLP-------
CANAL    TESSSPPEIPHITANMIPLSNIIKYYTQEAYKQLTT-AIENLS-------
CANGA    STNSPPP-IPHVEINQMPLSMIIRNLTVYTVKEISQYMKTTVH-------
ASPNI    SASGDPPELQHIVQGFFPLAKLLNRSAQQCWNDLADLVAELAEIQVPSHD
RHIO9    MKTEEKIVLPVEMASMVSLKSLIGKLVHKSYSDLMT-LTDTLP-------
CRYNE    PDQLEEELPPYFENENVSLGLVLDRFARKGNHDLRVLLGEVLP-------
USTMA    LAQLEQE-LPLEEADLISLAALVERLANFGYEALQN-LAETLP-------
ENCCU    RIEGGRECREDNGRETVDLKRFVSMFVNQTLKEMKE-ALGKFY-------
ANTLO    VVKLQTRLFHREDNETIDCGRYVRNVVNKVYEELDE-CLESLK-------
ARATH*   --------MAELGQQTVDFSALVGRAAEESFLSFKE-LVDKSK-------
VITVI    --------MAELGHQTVEFSTLVSRAAEESFLSLKD-LMEISK-------
SOYBN    -------MTAELGQQTVELSTLVTRAANDSYASLKE-LVDKCK-------
SOLLC    --------MAELGQQTVDFSALVSRAAEESYVTLKE-LVEKCK-------
ORYSJ*   ------MAEGELGQQTVELGAVVRRAAEESYLSLRE-LVEKSQ-------
HORVU    --------------------------------------------------
SORBI    -------MAGELGQQTVELGAVVRRAAEESYLALRE-LVEKSQ-------
SELML    ---------MALGQETVPLSLLVKRAAEAAYTGLKE-VCEAAP-------
PHYPA    -------MAEAAPLGSVDFSVLVRKAAEASVSGLKE-LTETAP-------
VOLCA    ---MEAPGPLRGAQPSVELQKLFADIFDNVLSDFIK-LSQPDS-------
                  110       120       130       140       150
```

```
HUMAN*   ----------------RKSDVERKIEIVQFASRTRQLFVRLLALVKWANN
CHICK    ----------------RKTDMERKIEIVQFASRTRQLFVRLLALVKWANN
XENTR    ----------------RKSDMERKIEIVQFASRTRQLFVRLLALVKWANN
DANRE    ----------------RKTDMERKIEIVQFASRTRQLFVRLLALVKWASN
CIOIN    ----------------SKSDLERKISVAQFASRYRQLFIRLFSLVKWASS
ANOGA    ----------------RKTDMDRKIEIYNFSASTRQLFIRLLALVKWANS
TETNG    ----------------RKTDMERKIEIVQFASRTRQLFVRLLALVKWASN
DROME    ----------------RKTDMERKVEIYNYAARTRHLFTRLNALVKWGNS
BRUMA    ----------------KKLDSDRKISIVQFAHSTRVLFIKLLAVVKWVKS
CAEEL    ------------RKTNDQGEQERKMSLVHFAHATRSQFLKLVALVKWIRI
YEAST*   -------------TQANEPNSAKKIRFLQLIIFLRTQFLKLYVLVKWTRT
SCHPO    ----------------SMSNVEKKKKILDWLLRSRAFTMRLLVLARWVHL
CANAL    ------------MNVNEESDIKRKKYFLNVIINLRQDFIKVYTLIKWASI
CANGA    -------------MNTADTSTARKMRFLNLLIFIRNQFLKLYVLIKWCRT
ASPNI    SNFSPISPNTKVLGNQSPENVRKKLRALEFAQKKRGEFIKLLVLSQWSRQ
RHIO9    ----------------SMSDVEKKRQILNYTTFVRKQFLKLLALVKWADC
CRYNE    ----------------QLSIRERPKPIIEYAKTTRQALLKYLAVLRW-KT
USTMA    ----------------SLPSSSKRAKIFNTALNVRKQFIKLLVLARWSKD
ENCCU    ----------------SISETVCRRELSVFFRQKAADFECLFLLISGVEY
ANTLO    -------------------YTGNREELARCHLSHSNLFKKISLLFKVLKH
ARATH*   ST--------------ELSDTEKKVSLLKYVAKTQQRMLRLNALAKWCKQ
VITVI    SS--------------DQSDSEKKISLLKFIVKTQQRMLRLNVLAKWCQQ
SOYBN    SS--------------ELSDTDKKISILKFLSKTQQRMIRLNVLSKWCQQ
SOLLC    SS--------------NLSDSEKKIGILKYVVKTQQRMLRLNVLSKWCQQ
ORYSJ*   DEGEGKG---GAYGARQRSDSEKKIDLLKFIARTRQRMLRLHVLAKWCQQ
HORVU    --------------------------------------------------
SORBI    AEAEGKGLGAGANGGWQRSDTEKKIDLLKFITRTRQRMLRLHVLAKWCQQ
SELML    ----------------EQSDAERKISLLKFILKTRQRLLRLLVICKWCRQ
PHYPA    ----------------ELSDTEKKIGLLKYIVKTRQRLLRIAVLTKWCRQ
VOLCA    -----------VGQADRESADQRKYRLLQHLNGTRQQLLRAVALLNW---
                  160       170       180       190       200
```

MED14

```
HUMAN*   A G K - - V E K C A M I S S F L D Q Q A I L F V D T A D R L A S L A R D A L V H A R - - - - - - - -
CHICK    A G K - - V E K C A M I S S F L D Q Q A I L F V D T A D R L A S L A R D A L V H A R - - - - - - - -
XENTR    A G K - - V E K C A M I S N F L D Q Q T I L F V D T A D R L A S L A R D A L V H A R - - - - - - - -
DANRE    A G K - - V E K C A M I S S F L D Q Q A Y L F V D T A D R L A S L A R D A L V H A R - - - - - - - -
CIOIN    V D K - - V V K C G D I S S F L D H Q A M L F V D T A D K L Y Q L S N Q L V I Q A R - - - - - - - -
ANOGA    A S K - - V D K S A K I M G F L D K Q S M L F I D T A D M L S R V A R E T L V H A R - - - - - - - -
TETNG    A G K - - V E K C A M I S S F L D Q Q T I L F V D T A D R L A S L A R D A L V H A R - - - - - - - -
DROME    V S K - - V D K S S Q I M S F L D K Q N M L F V E T A D M L A R M S R E T L V R A R - - - - - - - -
BRUMA    S K K - - F E S C A S I C Y F L D Q Q S Q Y F V D T A D R L V Q L A R E E L V F A R - - - - - - - -
CAEEL    S K R - - M D V C Y S I D Y L L D L Q S Q Y F I D T A D R L V A M T R G D L E L A R - - - - - - - -
YEAST*   I K Q N N F H V L I D L L N W F R T T N M N V N N C I W A L K S S - L N S M T N A K - - - - - - - -
SCHPO    S P S - - V H R C I D V V A F L Q G Q K F C F Q N L V H V L Q D I - R Y Q L S F A R - - - - - - - -
CANAL    S K D - - V S K F I D L L N W F R I Q E F H F E N L I F Q L N A L - - T G Y N A A K - - - - - - - -
CANGA    I K E N N F N I M I D L L N W F R G T N M T V N N C I W A L K S T - L E S T T N A K - - - - - - - -
ASPNI    A A D - - V S R L I D I Q N F I R T Q H Q A Y A G A L Q C M G D M - K R D L V R A Q - - - - - - - -
RHIO9    A D D - - I Q T C Q N I M A Y L A N Q N K T F Q D T V D Y L H K I - H V E L P A A R - - - - - - - -
CRYNE    S G K - - V T D A K R I A H F M E H Q N Q Q H E V A I E H V K H V - T K V V E G L R - - - - - - - -
USTMA    I S D - - L Q K S R N I I A L L S E Q Q W Q H E D V F A G L T D I - R K I L P N A R - - - - - - - -
ENCCU    L K D - - A G S N R H R I R K I K E V D G E Y V S A A D R L A Y L - F E S F K S M I - - - - - - - -
ANTLO    A S L - - L R R S T E K L K D A I V G N Q N F I S A A D R I A Y L - F E D M K L G L - - - - - - - -
ARATH*   V P L - - I N Y F Q D L G S T L S A H D I C F T Q A A D S L F F M - H E G L Q Q A R - - - - - - - -
VITVI    V P L - - I Q Y C Q Q L A S T L S S H D T C F T Q A A D S L F F M - H E G L Q Q A R - - - - - - - -
SOYBN    V P L - - I H H C Q Q L A S T V S N H D M C F T Q A A D S L F F M - H E G L Q Q A R - - - - - - - -
SOLLC    V P L - - I Q Y S Q Q L A S T L S S H D T C F T Q A A D S L F F M - H E G L Q Q A R - - - - - - - -
ORYSJ*   V P L - - V N Y C Q Q L G S T L S S H E T C F T Q T A D S L Y F M - H E G L Q Q A R - - - - - - - -
HORVU    - - - - - - - - - - - - - - - - - - - - - - - - - - - - - - - - M - H E G L Q H A R - - - - - - - -
SORBI    V P L - - V H Y C Q Q L G S T L H S H E T C F T Q T A D S L F Y M - H E C L Q Q A R - - - - - - - -
SELML    I P L - - V E R C Q Q L M G T L S N H D M S F T Q A A D S M F Y L - H E S L Q Q S R - - - - - - - -
PHYPA    I P L - - V E R C Q Q L S G T L S N H N M S F T Q A A D F L F Y L - H E T L Q Q A R - - - - - - - -
VOLCA    - - - - - - - K P W K V L N E L V D H D R V L D V A A S H V R T M - T E V V Q Q M R Q Q A A G R I M
                             210                 220                 230                 240                 250
```

```
HUMAN*   - - - - L P S F A I P Y A I D V L T T G S Y P R L P T C - - - - - - I R D K - - I I P P D P - I T K
CHICK    - - - - L P S F A I P Y A I D V L T T G S Y P R L P T C - - - - - - I R D K - - I I P P D P - I T K
XENTR    - - - - L P S F A I P Y A I D V L T T G S Y P R L P T C - - - - - - I R D K - - I I P P D P - I T K
DANRE    - - - - L P S F A I P F A I D V L T T G S Y P R L P T C - - - - - - I R D K - - I I P P D P - I T K
CIOIN    - - - - L P N F S I P T A V D V L T N G S Y T R L P T C - - - - - - I R D S - - V I P P K A - I L P
ANOGA    - - - - L P N F H I P A A V E I L T T G S Y S R L P S V - - - - - - I R D R - - I V P P D P - I T P
TETNG    - - - - L P S F A I P F A I D V L T T G S Y P R L P T C - - - - - - I R D K - - I I P P D P - I T K
DROME    - - - - L P N F H I P A A V E V L T T G T Y N R L P T C - - - - - - I R E R - - I V P A D A - I T P
BRUMA    - - - - L P I F Q V A L A V D V L T V G T F P R L P L C - - - - - - I K S R - - F I P D P P - I T S
CAEEL    - - - - L P E Y H I A P A I D V L V L G T Y N R M P S K - - - - - - I K E A - - F I P P A K - I T P
YEAST*   - - - - L P N V D L V T A L E V L S L G R - P N L P T H N F K L S G V S N S M D M V D G M A K V P I
SCHPO    - - - - L R N S D L V T A L D I L S T G T S L R L A N A - - - - - - P T S K L Y M L S E S P - L S T
CANAL    - - - - L P N S D I I T A L E V L Y H G R - P K L P S Y - - - - - - - - - - - N Y I K S D N - L S P
CANGA    - - - - L P N V D L V T A L E V L T L G R - P N L P T H D F K L S G E N N E Y E T V N G Q L R I P T
ASPNI    - - - - V A N P D L N T A L E V L L R G E V V S M P D L - - - - - - G - - - - - Y R P P K P - L T P
RHIO9    - - - - V R N F D I P T A V D I L T T G T Y Q R M P T K - - - - - - L K D - - - M I P P T P - L A D
CRYNE    - - - - E R N P D L L T A L S L L A T G T Y N R L P A S - - - - - - L V D P - - Y L P K P V - L T N
USTMA    - - - - M R N A D L P T A I D V L H T G T Y R R L P A S - - - - - - I K Q M - - A V A P K P - F T D
ENCCU    - - - - V P K S D V L T S L Q T A Y N P G Y - L L P K L - - - - - - G R D G - - - - - - - - - - - G
ANTLO    - - - - V P E I N I N V M L R - - - - - - - - - - - - - - - - - - - L Y K D Y R L L Y P K I - F V S
ARATH*   - - - - A P V Y D V P S A V E I L L T G S Y Q R L P K C - - - - - - L D D - - - V G M Q S S - L D E
VITVI    - - - - A P I Y D V P S A V E V L L T G T Y E R L P K C - - - - - - V E D - - - V G V Q G T - L T G
SOYBN    - - - - A P V Y D V P S A I D I L L T G S Y Q R L P K C - - - - - - I E D - - - V G T Q Y A - L T E
SOLLC    - - - - A P I Y D V P S A V E V L L T G S Y D R L P K C - - - - - - I E D - - - V G L Q S T - L N D
ORYSJ*   - - - - A P M F D I P S A L E V M L T G N Y Q R L P L C - - - - - - I E D - - - I G S Q N K - L S P
HORVU    - - - - A P I F D V S S A I E V I H T G S Y R R L P K S - - - - - - V E E - - - I G T Q N T - L F Q
SORBI    - - - - A P M F D V P S A I E V M L T G G Y Q R L P R C - - - - - - I E E - - - I G S Q N K - L S P
SELML    - - - - A P M Y D V A S A M D V L F T G G F P R M P K C - - - - - - I E D - - - L N V Q P V - L E G
PHYPA    - - - - A P V Y D I P T A T E I L V A G T Y N R L P K C - - - - - - I E D - - - L G M Q P V - L I G
VOLCA    A G S Y L N M Y D V N T A L E V L T T G T Y Q E L P S I - - - - - - I L Q R P P L F Q P P P - A S P
                             260                 270                 280                 290                 300
```

MED14

```
HUMAN*   I E K Q A T L H Q L N Q I L R H R L - - V T T D L P P Q L A N L T V A N G R V K F R V E G E F E A T
CHICK    S E K Q T T L H Q L N Q I L R H R L - - V T T D L P P Q L A N L T V A N G R V K F R V E G E F E A T
XENTR    N E K Q T T L H Q L N Q I L R H R L - - V T T D L P P Q L A N L T V A N G R V K F R V E G E F E A T
DANRE    A E K Q T T L N Q L N Q I L R H R L - - V T T D L P P Q L A N L T V A N G R V K F R V E G E F E A T
CIOIN    S E R K A T L K R I Q Q I V L F R L - - V T E V I P P Q I K D L A V E G G I V H L T V P G K F K A S
ANOGA    A E K R Q T L Q R L N Q V I Q H R L - - V T G S L L P Q L R K F R I E N G R V T F K V D H E F E V S
TETNG    S E K Q T T L N Q L N Q I L R H R L - - V T T D L P P Q L A N L T V A N G R V K F R V E G E F E A T
DROME    A E K R Q T L L R L N Q V I Q H R L - - V T G K L L P Q M R E F R I R N G R V T F E V K H E F S V S
BRUMA    R E E A C V L A R L N Q V I Q Y R L S S Y G T K L S P R I S K T V I R N G M V T M T V N G E F E V S
CAEEL    R E Q K L V T S R L N Q L I E S R L S R L S S G I P P N I K E I H I N N G L A T L L V P G E F E I K
YEAST*   G L I L Q R L K D L N L T V S I K I - - A L M N I P K P L N S Y H I K N G R I Y F T V P N E F E I Q
SCHPO    K Q I L Q T L H A L N M L I R I R L S - L Y E I I P T P F Q H F T I A N G R C T F T V P N E F S V S
CANAL    Q K I L E T L N D L N L V L M T R F A - L M D N I P K R F - D Y E I K D G R A Y I R V S N E F E V S
CANGA    K L I M Q R L K D L N I I V M I K I - - S S I D I P K Q F N N Y E I R D G R V Y I T V P G E F E I Q
ASPNI    K S T L K K M R K I N R V I S T R L A - L H D D I P L A F Q K Y R V H D G R V T F V V P G E F E L D
RHIO9    E E V L D V F R K M K E T I R V R M L - T E E V L P S P M Q K Y R I E N G R I Y F S I D N E F E I A
CRYNE    S A I L K V L R R L N R Q I R Y R L R - C L D Y L P P E L I V E D I K D G R M Y A R G D G - W R A E
USTMA    Q Q A L L I V S R L E D A L R T R M A - C R E L V P A P L S N Y T I H D G K V H F H V P G L F Q A Q
ENCCU    G R R V T S F E E I D R I S R I Y L - - D K E D L - S V Y S S V V I E E G I V I L S T R - F F S F S
ANTLO    E R Q I T S Y S E I H M L T K I Y L - - L N E D T G E - - F N V H I E K G A V V L D S R - Y F V F K
ARATH*   H Q Q K P A L R K L E V L V R S K L - - L E I T L P K E I T E V K I S K G T V T L S V D G E F K V L
VITVI    D Q Q K A A L K K L D T L V R S K L - - L E V S L P K E I S E V K V S D G T A L L C V D G E F K V L
SOYBN    E Q Q K P A L K K L D T L V R S K L - - L Q V S I P K E F S N I M V S D G T A M L R L D G E F K V L
SOLLC    D Q Q K P A L K K L D T L V R S K L - - L E V S L P K D I T E V K V S D G T V L L R V E G E F K V L
ORYSJ*   D E E K R A L Q K L N A S V R Y K V - - L V T P R P K E V S N V S V A D G I A V F R V D G E F K V L
HORVU    D E R M P T L K K L S T L V R A K L - - L E T L V P K E I S E V L V A D G I A N V Q V D G E F K V L
SORBI    D E E K C A L R K L D A S V R Y K V - - L V T P R P K E V S N V S V T D G I A V F R V D G E F K V L
SELML    W K K D N V L Q K L D T L L R S K L - - L D I A V P K E F T S V S V R D G R L T L R V D G E F E V E
PHYPA    E E R G G A V K K L N S V L R T R L - - L D I E L P K Q I T D I K V R G G R V I F K V A G E F E A H
VOLCA    A R Q R S R L A H V T H L I R A R L - - V Q V A L P P G L K V A S V S H G T A V L Q A E G L Y E A K
                                   310                 320                 330                 340                 350
```

```
HUMAN*   L T V M G - - - - - - - - D D P - - - - - - - - - - - - - - - - - - - - - - - - - - - - - - - - - -
CHICK    L T V M G - - - - - - - - D D P - - - - - - - - - - - - - - - - - - - - - - - - - - - - - - - - - -
XENTR    L T V M G - - - - - - - - D D P - - - - - - - - - - - - - - - - - - - - - - - - - - - - - - - - - -
DANRE    L T V M G - - - - - - - - D D P - - - - - - - - - - - - - - - - - - - - - - - - - - - - - - - - - -
CIOIN    L T L M G - - - - - - - - D S D - - - - - - - - - - - - - - - - - - - - - - - - - - - - - - - - - -
ANOGA    L T V M G - - - - - - - - D A P - - - - - - - - - - - - - - - - - - - - - - - - - - - - - - - - - -
TETNG    L T V M G - - - - - - - - D D P - - - - - - - - - - - - - - - - - - - - - - - - - - - - - - - - - -
DROME    L T V M G - - - - - - - - D N P - - - - - - - - - - - - - - - - - - - - - - - - - - - - - - - - - -
BRUMA    L T L L G - - - - - - - - E R P - - - - - - - - - - - - - - - - - - - - - - - - - - - - - - - - - -
CAEEL    I T L L G - - - - - - - - E T E - - - - - - - - - - - - - - - - - - - - - - - - - - - - - - - - - -
YEAST*   L S T V N - - - - - - - - R Q S - - - - - - - - - - - - - - - - - - - - - - - - - - - - - - - - - -
SCHPO    L T T N S - - - - - - - - Q D P - - - - - - - - - - - - - - - - - - - - - - - - - - - - - - - - - -
CANAL    I T V G N D L I I D N P E E Y Y - - - - - - - - - - - - - - - - - - - - - - - - - - - - - - - - - -
CANGA    L S T I D - - - - - - - - R H S - - - - - - - - - - - - - - - - - - - - - - - - - - - - - - - - - -
ASPNI    L S I G E - - - - - - - - E D V - - - - - - - - - - - - - - - - - - - - - - - - - - - - - - - - - -
RHIO9    L T L M G - - - - - - - - Q S H - - - - - - - - - - - - - - - - - - - - - - - - - - - - - - - - - -
CRYNE    L T V V G - - - - - - - - F E D - - - - - - - - - - - - - - - - - - - - - - - - - - - - - - - - - -
USTMA    L T A S G S A Q H E L T P S S S - - - - - - - - - - - - - - - - - - - - - - - - - - - - - - - - - -
ENCCU    L A L C G - - - - - - - - E I S - - - - - - - - - - - - - - - - - - - - - - - - - - - - - - - - - -
ANTLO    L I L C G - - - - - - - - E L K - - - - - - - - - - - - - - - - - - - - - - - - - - - - - - - - - -
ARATH*   V T L G Y - - - - - - - - R G H - - - - - - - - - - - - - - - - - - - - - - - - - - - - - - - - - -
VITVI    V T L G Y - - - - - - - - R G H - - - - - - - - - - - - - - - - - - - - - - - - - - - - - - - - - -
SOYBN    I T L G Y - - - - - - - - R G H - - - - - - - - - - - - - - - - - - - - - - - - - - - - - - - - - -
SOLLC    V T L G Y - - - - - - - - R G H - - - - - - - - - - - - - - - - - - - - - - - - - - - - - - - - - -
ORYSJ*   L T L G Y - - - - - - - - R G H - - - - - - - - - - - - - - - - - - - - - - - - - - - - - - - - - -
HORVU    L T L G Y - - - - - - - - R G H - - - - - - - - - - - - - - - - - - - - - - - - - - - - - - - - - -
SORBI    L T L G Y - - - - - - - - R G N - - - - - - - - - - - - - - - - - - - - - - - - - - - - - - - - - -
SELML    L T L G Y - - - - - - - - R G N - - - - - - - - - - - - - - - - - - - - - - - - - - - - - - - - - -
PHYPA    L T L G Y - - - - - - - - R G H - - - - - - - - - - - - - - - - - - - - - - - - - - - - - - - - - -
VOLCA    L T L V P - - - - - - - - S E P L L G V D A A P D A A A G G G G E A G G S G G G A G Y D W T A A G A
                                   360                 370                 380                 390                 400
```

MED14

HUMAN*	----------DV-----PWRLLKL--EILV--------------------
CHICK	----------DI-----PWRLLKL--EILV--------------------
XENTR	----------DI-----PWRLLKL--EILV--------------------
DANRE	----------DI-----PWRLLKL--EILV--------------------
CIOIN	----------NL-----PWRLIKL--EFLV--------------------
ANOGA	----------TV-----PWRLLDI--DFLV--------------------
TETNG	----------DI-----PWRLLKL--EILV--------------------
DROME	----------TV-----PWRLLDI--DVLV--------------------
BRUMA	----------AT-----KWTLLNI--KILV--------------------
CAEEL	----------MT-----KWTLLNI--KILV--------------------
YEAST*	-----------------PLFFVDL--KLLFNTEAEQTVSAVTEATSTNGD
SCHPO	----------KSTGISFQWIVVDF--QFHL--------------------
CANAL	----------KS-----PFYFIDF--KFLF--------------------
CANGA	-----------------PFFFVDF--KLLF--------------------
ASPNI	----------TS-----QFFFVDI--RFLF--------------------
RHIO9	----------TR-----RWWIVSL--DILV--------------------
CRYNE	----------TS-----RWWLTGV--EWGWKAKEKGVDDPGGRVTKKFTG
USTMA	----------DD-----RWWLLDLTFDVIA--------------------
ENCCU	----------KP-----EWKLINV--K-----------------------
ANTLO	----------SP-----KWKLISV--------------------------
ARATH*	----------LS-----MWRILHL--DLLV--------------------
VITVI	----------LS-----MWRILHL--ELLV--------------------
SOYBN	----------LS-----LWRILHL--ELLV--------------------
SOLLC	----------LS-----MWRILHM--ELLV--------------------
ORYSJ*	----------LD-----LWRILHL--EVLV--------------------
HORVU	----------FS-----LWRILHI--ELLV--------------------
SORBI	----------VD-----LWRILHM--ELLV--------------------
SELML	----------AS-----LWRVLHL--KLLV--------------------
PHYPA	----------LS-----YWRVLKI--DILV--------------------
VOLCA	AAAAADPLAAAAASQRWKWRLIHF--VLAI--------------------

410 420 430 440 450

HUMAN*	----EDKETGDG----RALV---H-SMQI-------SFIHQLVQSR----
CHICK	----EDKETGDG----RALV---H-SMQI-------NFIHQLVQSR----
XENTR	----EDKETGDG----RALV---H-SMQI-------NFIHQLVQSR----
DANRE	----EDKETGDG----RALV---H-SMQV-------NYIHELVQSR----
CIOIN	----HDKEVGGG----RTLV---H-DLQV-------NYIHELVQSR----
ANOGA	----EDKETGDG----KALV---H-PLQV-------NYIHQLIQGR----
TETNG	----EDKETGDG----RALV---H-SLQV-------NFIHELVQAR----
DROME	----EDKETGDG----KSLV---H-PLQV-------NYIHQLIQAR----
BRUMA	----EDYEIGFG----TKLV---H-PLQV-------NMLHNVLQLR----
CAEEL	----EDYELGMG----LPLV---H-PLQL-------NQLHGVLQSR----
YEAST*	SENNEENSSSNGNNL----------PLNK-------PRLEKLINEI----
SCHPO	----PDFSST------PAKY---R-VFIE-------LHLNEEIAAA----
CANAL	GANPESGLITFNDDKISTKL---P-TSSH-------KKLEKLVNQT----
CANGA	----------AGTDS----------LFNK-------PRLEKIINDV----
ASPNI	----SPSPSIPA----GRML---S-ELDI-------KIN-----------
RHIO9	----QASTCGGAATDIDISL---N-DAQK-------QHLRMNAQKQ----
CRYNE	EERQGILDIANGEVLPPRELPENEMEVDTVITEDPQVKKSEVVEVV----
USTMA	----SGSCAASCNRAFPRKP---KRAYRERLRVWGDQQLSPVIQSDGVDE
ENCCU	----GDNEVFNK----HLLF---AMPHRI-------DKISRFLR------
ANTLO	------NRINKA----LWLV---HLPSDV-------TKLNFFIR------
ARATH*	----GERS---G----PIKL---E-VTRR-------HILGDDLERR----
VITVI	----GERG---G----LVKL---E-ELRR-------HALGDDLERR----
SOYBN	----GEKD---K----PVKL---E-ATRR-------HLLGDDLERR----
SOLLC	----GERS---G----PMKL---D-DFRR-------HALGDDLERR----
ORYSJ*	----GDKG---G----PIKL---E-ERRR-------FALGDDIERR----
HORVU	----GEKT---G----PIKL---E-ETRR-------YVLGDDIERR----
SORBI	----GEKK---G----PIKL---D-QSRR-------FALGDDIERR----
SELML	----GESS---R----PAKF---S-DMQR-------FALSDDLERR----
PHYPA	----GEPS---G----PTQF---T-DPQA-------YFLGDELERR----
VOLCA	-----------G----VPFY---D-PRQP-------GQILNTCNFHMVLA

460 470 480 490 500

MED14

```
HUMAN*  ------------------------------------------LFADE---
CHICK   ------------------------------------------LFADE---
XENTR   ------------------------------------------LFADD---
DANRE   ------------------------------------------LFGDD---
CIOIN   ------------------------------------------LISNK---
ANOGA   ------------------------------------------IVDCT---
TETNG   ------------------------------------------LCADE---
DROME   ------------------------------------------LVENP---
BRUMA   ------------------------------------------MDKSL---
CAEEL   ------------------------------------------MNVSL---
YEAST*  ------------------------------------------LLKSN---
SCHPO   ------------------------------------------FVLQK---
CANAL   ------------------------------------------LLT-----
CANGA   ------------------------------------------LLRNK---
ASPNI   ------------------------------------------DTLRN---
RHIO9   ------------------------------------------LIPPTTPE
CRYNE   ------------------------------------------KKTVD---
USTMA   SNARPLDIAADKTEQGTDVQIAPEKPTD--------------APTRD---
ENCCU   ------------------------------------------IYETH---
ANTLO   ------------------------------------------LYRSL---
ARATH*  ------------------------------------------MSVAE---
VITVI   ------------------------------------------MAAAE---
SOYBN   ------------------------------------------MAAAE---
SOLLC   ------------------------------------------MAAAD---
ORYSJ*  ------------------------------------------MAVSE---
HORVU   ------------------------------------------MAVAD---
SORBI   ------------------------------------------MAASE---
SELML   ------------------------------------------MSALE---
PHYPA   ------------------------------------------MAASD---
VOLCA   ADNAAYLRRQRQGAGGVAAAGGGPTASTMSESTATPAASGLQLPGAAAAG
                  510       520       530       540       550
```

```
HUMAN*  -----------------KPLQDMYNCLHSFCL----------SLQLEV--
CHICK   -----------------KPLQDMYNCLHSFCL----------ALQLEV--
XENTR   -----------------KPLQDMYNCLHSFCL----------SLQLEV--
DANRE   -----------------KPLQDMYTCLHSFCL----------SLQLEV--
CIOIN   -----------------TPFVDLFDILHSFSL----------SLQLEV--
ANOGA   -----------------DALAEVYTCLHYFCQ----------SLQLEV--
TETNG   -----------------KPLQDMYNCLHSFCL----------SLQLEV--
DROME   -----------------NALSEVYNCLHYFCQ----------SLQLEV--
BRUMA   -----------------EPLEEVYNLLHSFAQ----------MLQLDV--
CAEEL   -----------------NPIKEAFSFLHSFCV----------SLQLDV--
YEAST*  -----------------DPLLSLYNFLHKYVL----------TLQLYM--
SCHPO   -----------------PILPLIYNILHKFCL----------YQRLNL--
CANAL   -----------------RGLQGLYELLHKYSN----------SFKIYL--
CANGA   -----------------KPLYSLYSLLHRYVL----------TLQLYM--
ASPNI   -----------------GGLSECFNWLHNLIL----------TNKINI--
RHIO9   GGDDKPTGLF-------FPLVNLYDYLHLCCL----------NMQLEM--
CRYNE   -----------------APLVRVYNLLQHLSL----------SYRLEV--
USTMA   -----------------APLSRLFAFLQERAL----------HYQMDI--
ENCCU   -----------------TKAREMFLMVKRSSE----------HIEMSIKG
ANTLO   -----------------EEAKKVYYTFVNDCFFRNLVKGTPREFSLNY--
ARATH*  -----------------NPFTILYAVLHELCV----------AIVMDT--
VITVI   -----------------NPFMMLYSVLHELCV----------ALIMDT--
SOYBN   -----------------NPFSVLYSVLHELCV----------ALVMDT--
SOLLC   -----------------HPFMTLYSILHELCV----------ALVMDT--
ORYSJ*  -----------------NPFMVLYAILHELCI----------SLAMDT--
HORVU   -----------------NPFTILYTILHELCI----------SFVMDT--
SORBI   -----------------NPFAVLYAILHEFCI----------SLAMDT--
SELML   -----------------NPFGIIYTVLHEFCA----------ALVMDT--
PHYPA   -----------------DPLGLMYSILHQFCT----------ALVMDT--
VOLCA   GGTLAAAVRRSEDDEVGAPLAVMHAILTDVAG----------RLLVDE--
                  560       570       580       590       600
```

MED14

```
HUMAN*   ----------LHSQTLMLIR-E-RWGD-LVQVERYHAG------------
CHICK    ----------LHSQTLMLIR-E-RWGD-LVQVERYHAG------------
XENTR    ----------LHSQTLMLIR-E-RWGD-LVQVDRYLAG------------
DANRE    ----------LHSQTLMLIR-E-RWGD-LVQVERYIPA------------
CIOIN    ----------LQAQAER-SR-S-RWNK-FVNVEEYSPG------------
ANOGA    ----------LYTQTLRLIR-D-RLDD-HIHVDEYVVG------------
TETNG    ----------LHSQTLMLIR-E-RWGD-LVQEERYVPA------------
DROME    ----------LYTQTLRLNY-E-RLDDNNITVEEYVPG------------
BRUMA    ----------LFCQATQVFS-G-DMCQ-YAAIERYDRN------------
CAEEL    ----------LFCQTSRLAA-G-RLRD-NITIEKYDPK------------
YEAST*   ----------VHREFLKLANGG-KFSKSNLIHNYDSKK------------
SCHPO    ----------LSQQTFQLSR-E-SWLG-HLRGVYDEKP------------
CANAL    ----------LAKQFQTLLINS-RWRG-NFQINYQTNK------------
CANGA    ----------IHLELLNLENDG-KFSGGNLVHSYNAKK------------
ASPNI    ----------LARQASELSR-S-LWSN-VLRSELLHRT------------
RHIO9    ----------VHIQFLMLSK-T-RWLD-QLKVQMDQER------------
CRYNE    ----------LFTQAIALSQ-G-KWRG-QLIPEMDRED------------
USTMA    ----------LQHQAYELCR-L-NWGS-NLRIETAERP------------
ENCCU    HYRRFEVSFHVFKMAVHVER-C------DIRGKLFVGE------------
ANTLO    ----------IVRLKLQVKK-G------RLACAVSHGD------------
ARATH*   ----------VIRQVRALLQ-G-RWKD-AIRFDLISDT----------GT
VITVI    ----------VIRQVKALRQ-G-RWKD-AIRFELISDG--NIAQGGSAGS
SOYBN    ----------VIRQVQVLRQ-G-RWKD-AIRFELISEG------HGASSS
SOLLC    ----------VIRQVQTLRQ-G-RWKD-AIRFELITDV--STGQAGSAGS
ORYSJ*   ----------IIRKTNVLRQ-G-RWKD-AIRSELVLDS--TTGQTGSAPL
HORVU    ----------VIRQTNVLRQ-G-RWKE-AIKSELVSDSHRSAGQGGNNAP
SORBI    ----------IIRQANVLRQ-G-RWKD-AIRSELISDS--ATGQTGNTPL
SELML    ----------LWRQVRALRH-G-RWKD-AIRLDLSFET-----AASGNPA
PHYPA    ----------LFRQIKALLV-G-RWRD-AIKYEKLSET--GGGSGPAAGQ
VOLCA    ----------LAAAARALAAPGSRWHG-HISVK-----------------
                  610       620       630       640       650
```

```
HUMAN*   ---------------KC-LSLSVWNQQVLGRK-----TG-----------
CHICK    ---------------KC-LSLSVWNQQVLGRK-----TG-----------
XENTR    ---------------KF-LTLIVWNQQVLGRK-----TG-----------
DANRE    ---------------KC-LTLSVWNQQVLGRK-----TG-----------
CIOIN    ---------------QK-LLMSYW---TLNAN-----KP-----------
ANOGA    ---------------SR-LTVSYW-RELTNKD-----PK-----------
TETNG    ---------------KY-LTLSVWNQQVLGRK-----TG-----------
DROME    ---------------VK-LTVSYW-RDL----------K-----------
BRUMA    ---------------MGILTLAYWLKRTHNRY------------------
CAEEL    ---------------ERVLVVGYWVKRSKSRRLTVGQVK-----------
YEAST*   ----------------STITVRYWLNGKMDSK------------------
SCHPO    ----------------PRLRLYYWPQLNVKKE----GKP-----------
CANAL    ----------------SLIVINYWSQHYLSRN------------------
CANGA    ----------------SLIVMKYWRNGKLGNK------------------
ASPNI    ------------------LVLQYWASKPNTKS------------------
RHIO9    ----------------TKLAITYWAGGSSAAH------------WARPQM
CRYNE    ----------------KTLRLKYWLRERPMQV------------------
USTMA    ----------------RNLTVHYWTQAQGAAGATQRVES-----------
ENCCU    --------------------------------------------------
ANTLO    --------------------------------------------------
ARATH*   TPANQEGEADSVSLRTPGMKLFYWSDSDKNSG------------------
VITVI    MQMNQDGEADSAGLRTPGLKIVYWLDLDKNSG----TSD-----------
SOYBN    SALNPDGESDSSAMRTPGLKIVYWLDFDKNAG----ASE-----------
SOLLC    TQTSQDGESDSASLRTPGLKILYWLDLDKNSG----TSE-----------
ORYSJ*   MQLGQDGEYDLSGSRIPGLKINYWLD-EKAGG----SAE-----------
HORVU    TQLGQDGELDSSGLKIPGLKVNYWLD-ERNSG----SAE-----------
SORBI    MQLVQDGEFDASGFKIPGLKVNYWLD-EKSTG----TTE-----------
SELML    TQAPADAEADPSKSRVHGVKITYWLDMAKGGE------------------
PHYPA    ATKVRG---------SPGLKIMYWLETQR---------------------
VOLCA    ----------PSQLLHPGVRIEYWTQAPQLVS-------NRALPPACPGE
                  660       670       680       690       700
```

MED14

```
HUMAN*  -----TASVHKVTIKI----------------------------------
CHICK   -----TASVHKVTIKI----------------------------------
XENTR   -----TASVHKVTVKI----------------------------------
DANRE   -----TASVHKVTIKI----------------------------------
CIOIN   -----GKTRYSVTVQQ----------------------------------
ANOGA   -----SELGYRLTIQT----------------------------------
TETNG   -----AASVYKVTIKI----------------------------------
DROME   -----SELGYRLTVQS----------------------------------
BRUMA   ------TSQYKIKIFI----------------------------------
CAEEL   -----CDAQYRVQIYE----------------------------------
YEAST*  -------GKITIGIQR----------------------------------
SCHPO   -----GKIGHYIHIFV----------------------------------
CANAL   -----WKSFIELGIDSHSNLNYRWFKNGQYCFGDQGNNELDKIFHLQRRN
CANGA   -------TKIIIGVDR----------------------------------
ASPNI   --------WLEIGIRR----------------------------------
RHIO9   EKEASKSTVIEISVSD-------------EHEKELAKKNMAIVVRDETKG
CRYNE   ------QRQDSTSIKR----------------------------------
USTMA   -----AAAGGSVQITIVDLPDTPGATETLAALFDGDDDQ-----------
ENCCU   --------------------------------------------------
ANTLO   --------------------------------------------------
ARATH*  ---------PFIKIEP----------------------------------
VITVI   -----SGSCPFIKVEP----------------------------------
SOYBN   -----SGTCPFLKIEP----------------------------------
SOLLC   -----IGTCPFIKIEP----------------------------------
ORYSJ*  -----SDSSPFIKIEA----------------------------------
HORVU   -----SDSSPFIKVEA----------------------------------
SORBI   -----PDSSPFIKIEA----------------------------------
SELML   ------SLLPSLRIEP----------------------------------
PHYPA   -----GEVPPSLRIEH----------------------------------
VOLCA   DWRSAKLGPPYLELGM----------------------------------
                 710       720       730       740       750
```

```
HUMAN*  -------DEN---------DVSKPLQIFHDPPL-----------------
CHICK   -------DET---------DVSKPLQISHEPPL-----------------
XENTR   -------DET---------DCSKTLQISHEPPL-----------------
DANRE   -------DES---------DGSKPLQISHEPPL-----------------
CIOIN   -------CPT---------DSTKPLQVIHTPKL-----------------
ANOGA   -------DPN---------DAAKQLAILHVPSI-----------------
TETNG   -------DES---------DGSKPLQISHEPPL-----------------
DROME   -------DPS---------EIGRPLAVVHVPSL-----------------
BRUMA   -------DKE---------NQHNGLRVRHFPVG-----------------
CAEEL   -------DPN---------DKLGGLKVRHFPHA-----------------
YEAST*  -------TTE---SLILKWDNQSASRAKNMPVI-----------------
SCHPO   -------NTQ-------------PISAFERTLS-----------------
CANAL   SNNGVTTSSNTISTSAMIAGIRRSTELSNETAI-----------------
CANGA   -------KSE---DLVLKWDNENAKQSMHMPLV-----------------
ASPNI   -------GPR-----------ESTIGGMRPPSL-----------------
RHIO9   LIQKAGLGASVRLSKVDPLEKPKIVSLLKYPKN-----------------
CRYNE   ------------------PQAPATLPASRQTMVGGIIHITLAEKTTPRSE
USTMA   -------SSTTHDTASLTTVKRRGLQVTWDADR-----------------
ENCCU   --------------------------------------------------
ANTLO   --------------------------------------------------
ARATH*  -------GSD------------LQIKCSHSTFV-----------------
VITVI   -------GPD------------LQIKCLHSTFV-----------------
SOYBN   -------GSD------------LQIKCLHSSFV-----------------
SOLLC   -------GPD------------LRIKCLHSTFV-----------------
ORYSJ*  -------RQD------------MQIKCQHSSFI-----------------
HORVU   -------LQD------------MQIKCQHSSFV-----------------
SORBI   -------GLD------------MQIKCQHSSFI-----------------
SELML   -------GSD------------QRISCQHQPPL-----------------
PHYPA   -------GAD------------QQIRCSHSPAV-----------------
VOLCA   -------GEG------------GCVEVLNSPSS-----------------
                 760       770       780       790       800
```

MED14

```
HUMAN*   - - - - - - - - - - - - - - - P A S - - - - - - - - - - - - - - - - - - - - - - - - D - S K L V E R
CHICK    - - - - - - - - - - - - - - - P S C - - - - - - - - - - - - - - - - - - - - - - - - D - S K L M E R
XENTR    - - - - - - - - - - - - - - - P A C - - - - - - - - - - - - - - - - - - - - - - - - D - S K L M E R
DANRE    - - - - - - - - - - - - - - - P A C - - - - - - - - - - - - - - - - - - - - - - - - D - S K L M E R
CIOIN    - - - - - - - - - - - - - - - P L D - - - - - - - - - - - - - - - - - - - - - - - - D - I K L V E A
ANOGA    - - - - - - - - - - - - - - - G N K - - - - - - - - - - - - - - - - - - - - - - - - E - A D I A D R
TETNG    - - - - - - - - - - - - - - - P S C - - - - - - - - - - - - - - - - - - - - - - - - D - S K L I E R
DROME    - - - - - - - - - - - - - - - G A K - - - - - - - - - - - - - - - - - - - - - - - - E S A E V A D R
BRUMA    - - - - - - - - - - - - - - - - - - - - - - - - - - - - - - - - - - - - - - - - - - - - K G L P Y I
CAEEL    - - - - - - - - - - - - - - - - - - - - - - - - - - - - - - - - - - - - - - - - - - - - P Q L G R L
YEAST*   - - - - - - - - - - - - - - - Y N N I V S - - - - - - - - - - - - - - - - - - - - - - - - - - - - -
SCHPO    - - - - - - - - - - - - - - - S K R - - - - S S C E Y D H F L L L V E W - - - - - - H H D G I V E H
CANAL    - - - - - - - - - - - - - - - V D D N D N D - - - - - - - - - - - - - - - - - - - - N N D T N N T G
CANGA    - - - - - - - - - - - - - - - Y T H V V G - - - - - - - - - - - - - - - - - - - - - - - - - - - - -
ASPNI    - - - - - - - - - - - - - - - G L R - - - - - - - - - - - - - - - - - - - W I R D G Q E V N S D D V
RHIO9    - - - - - - - - - - - - - - - C L N - - - - - - - - - - - - - - - - - - V L W G D S K D L H T N A N
CRYNE    L D N L L S D I A C G G L V P S E R V L R L E L N V K W V V D E A G V G G G L K S G D V M D N S M L
USTMA    - - - - - - - - - - - - - - - S I L - - - - - - - - - - - - - - - - - - - - - - - - A D A Q S N N L
ENCCU    - - - - - - - - - - - - - - - - - - - - - - - - - - - - - - - - - - - - - - - - - - - - - - - - - -
ANTLO    - - - - - - - - - - - - - - - - - - - - - - - - - - - - - - - - - - - - - - - - - - - - - - - - - -
ARATH*   - - - - - - - - - - - - - - - I D P - - - - - - - - - - - - - - - - - - - - - - - - L T G K E A E F
VITVI    - - - - - - - - - - - - - - - I D P - - - - - - - - - - - - - - - - - - - - - - - - L T G K E A E F
SOYBN    - - - - - - - - - - - - - - - I D P - - - - - - - - - - - - - - - - - - - - - - - - L M G K E A E F
SOLLC    - - - - - - - - - - - - - - - I D P - - - - - - - - - - - - - - - - - - - - - - - - L T G K E A E F
ORYSJ*   - - - - - - - - - - - - - - - L D P - - - - - - - - - - - - - - - - - - - - - - - - L T D K E A N L
HORVU    - - - - - - - - - - - - - - - L D P - - - - - - - - - - - - - - - - - - - - - - - - L T D K E A D L
SORBI    - - - - - - - - - - - - - - - L D P - - - - - - - - - - - - - - - - - - - - - - - - F T D K E A N L
SELML    - - - - - - - - - - - - - - - T D P - - - - - - - - - - - - - - - - - - - - - - - - G T G N D A E F
PHYPA    - - - - - - - - - - - - - - - M D S - - - - - - - - - - - - - - - - - - - - - - - - A T G M E A E F
VOLCA    - - - - - - - - - - - - - - - L H K - - - - - - - - - - - - - - - - - - - - - - - - P M S L S K L R
                             810                 820                 830                 840                 850
```

```
HUMAN*   A M K I D H L S I E K L - - - L I D S V H A R A H Q K L Q E L K A I L R G - - - - F N A N E N S S I
CHICK    A M K I D H L S I E K L - - - L I D S V H A R S H Q K L Q E L K A I L K S - - - - Y N V N D N S F I
XENTR    A M R I D H L S I E K L - - - L I D S V H A R S H Q K L Q E L K T I L K S - - - - F N P S D N S F V
DANRE    A M K I D H L S V E K L - - - L I D S V H A R S H Q K L Q E L K A I L K S - - - - S N P N D N S F I
CIOIN    A S H I N H L S L E Q L - - - T H Q V M Y Y V R S L H F L L Q V K D M L H K - - - - H R K K D Q S Y I
ANOGA    A V R S D L L S M E R L - - - L V H T V Y Y V R S L A R L N D V K T E L Q L - - - - F L K D V E Y N I
TETNG    A M K I D H L S V E K L - - - L I D S V H A R S H Q K L Q E L K A I L K A - - - - N N P S D N A F I
DROME    A V R S E H L S M E R L - - - I V H T V Y I R S V S R L S D L K L E F Q A - - - - F L K D V D F N L
BRUMA    D D R S G R L S I N R L - - - L S E T V G V R C R E R L L R I R E R L G L - - - - L K P P V I V S L
CAEEL    D S G A G M L S I D R L - - - L S E T Y V V R C K E R L M R L R R I L E A - - - - A E P R L E V K M
YEAST*   - - - - - - - N I E G I - - - L D E I M F N H A R I I R S E L L A R D I F Q E D E E N S - - D V L L
SCHPO    V P L D D H M D A Q H L - - - L L L I T Q K H A Q L I L E Q I R K E L H P N I F S E H V - - - - - -
CANAL    E T E N E D L N V D L I - - - L N V V V N Q H A K S I M S E I Y S Q L L T R F S E T D V - - - - - -
CANGA    - - - - - - - N V E A I - - - L D E I M F N H S R L I R A D L L S R G V F Q E D E E D P - - S V L L
ASPNI    E F D T D S L S V D R L - - - L R S A I A L H I S H I L S S A F R R I S E K L L Y S N G - - - - - -
RHIO9    L L N A S D L N A E E L - - - L L H V T K Y H S K C I K D R M R Q L L E S Q K D F L K E - - - - - -
CRYNE    H L D P D A L S V E D L - - - L H V C T R T H A S H L T R S H G T S L L T S P R F L N A - - - - - -
USTMA    A I S P H S L D I E A L - - - L S S V I K Q H T L A L L R N L Q R R I L V S D H A L S R - - - - - -
ENCCU    - - - - - - - D V Y P S G P D V I K S F E D S V S E Y L C R D G R N V S F - - - - - - - - - - - - -
ANTLO    - - - - - - - N I E Y L C R N I K E R Y E Q E T E K V L K N I D Q R A R F - - - - - - - - - - - - -
ARATH*   S L D Q S C I D V E K L - - - L L K A I C C N R Y T R L L E I Q K E L L R N T R I C R T P S D V I L
VITVI    S L D Q N C I D V E K L - - - L L R A I C C S R Y T R L L E I Q K E L A K N S Q I C R T M G D V L L
SOYBN    V L D Q S C I D V E R L - - - L L R A I C C N K Y T R L L E I K R E L V K N V Q V C R T A D D V V L
SOLLC    S L D Q S C I D I E K L - - - L L R V I C C N R Y T R L L E I L K E L E K N S Q I C R V P S D I Q L
ORYSJ*   S L D L C C I D V E Q L - - - I L R A I A S N R H T R L L D I Q R Q L S K N V Q I S Q S P K D V I L
HORVU    S L D L S C I D V E A L - - - I L K A I A C N I H T R L L E I Q R E L K K N T Q I S R S P S D V V L
SORBI    S L D L S C I D V E Q L - - - I L R A I A C N R H T R L L N I Q R Q L C K N V Q I S Q S P K D V V L
SELML    A L D Q S R L D V E K L - - - I L R V I N C N I H S R L V E V Q K S L K A N T Y I H R V E D D V F L
PHYPA    A V D S S C I N V E K L - - - L L R A I S C S V H T R L L E V Q R G L K G G V H L W Q S D N D V V L
VOLCA    R L D T F R V N V E E V - - - L M R A A N M T S Q F Q L M Q L T P H F L A Y D F P I - - P A R V S L
                             860                 870                 880                 890                 900
```

MED14

```
HUMAN*   --------------------------------------------------
CHICK    --------------------------------------------------
XENTR    --------------------------------------------------
DANRE    --------------------------------------------------
CIOIN    V-------------------------------------------------
ANOGA    --------------------------------------------------
TETNG    --------------------------------------------------
DROME    --------------------------------------------------
BRUMA    --------------------------------------------------
CAEEL    --------------------------------------------------
YEAST*   F-------------------------------------------------
SCHPO    --------------------------------------------------
CANAL    --------------------------------------------------
CANGA    F-------------------------------------------------
ASPNI    --------------------------------------------------
RHIO9    --------------------------------------------------
CRYNE    --------------------------------------------------
USTMA    --------------------------------------------------
ENCCU    --------------------------------------------------
ANTLO    --------------------------------------------------
ARATH*   Q-------------------------------------------------
VITVI    H-------------------------------------------------
SOYBN    Q-------------------------------------------------
SOLLC    Q-------------------------------------------------
ORYSJ*   K-------------------------------------------------
HORVU    K-------------------------------------------------
SORBI    T-------------------------------------------------
SELML    K-------------------------------------------------
PHYPA    R-------------------------------------------------
VOLCA    LPFTFLPTHAGTTPSASGVDPQIRHELKVILMEAKRAPTLRLSSIARQLW
                  910       920       930       940       950
```

```
HUMAN*   --------------------------------------------------
CHICK    --------------------------------------------------
XENTR    --------------------------------------------------
DANRE    --------------------------------------------------
CIOIN    --------------------------------------------------
ANOGA    --------------------------------------------------
TETNG    --------------------------------------------------
DROME    --------------------------------------------------
BRUMA    --------------------------------------------------
CAEEL    --------------------------------------------------
YEAST*   --------------------------------------------------
SCHPO    --------------------------------------------------
CANAL    --------------------------------------------------
CANGA    --------------------------------------------------
ASPNI    --------------------------------------------------
RHIO9    --------------------------------------------------
CRYNE    --------------------------------------------------
USTMA    --------------------------------------------------
ENCCU    --------------------------------------------------
ANTLO    --------------------------------------------------
ARATH*   --------------------------------------------------
VITVI    --------------------------------------------------
SOYBN    --------------------------------------------------
SOLLC    --------------------------------------------------
ORYSJ*   --------------------------------------------------
HORVU    --------------------------------------------------
SORBI    --------------------------------------------------
SELML    --------------------------------------------------
PHYPA    --------------------------------------------------
VOLCA    CRDGHVPHQQSGGTQEDEPQADGQPQSQTRDGGEAAGALSTAGEVAGSAV
                  960       970       980       990      1000
```

MED14

```
HUMAN*  --------------------------------------------------
CHICK   --------------------------------------------------
XENTR   --------------------------------------------------
DANRE   --------------------------------------------------
CIOIN   --------------------------------------------------
ANOGA   --------------------------------------------------
TETNG   --------------------------------------------------
DROME   --------------------------------------------------
BRUMA   --------------------------------------------------
CAEEL   --------------------------------------------------
YEAST*  --------------------------------------------------
SCHPO   --------------------------------------------------
CANAL   --------------------------------------------------
CANGA   --------------------------------------------------
ASPNI   --------------------------------------------------
RHIO9   --------------------------------------------------
CRYNE   ---------------------------------------------SSVQP
USTMA   -----------------------------------LLRPEDCTLCLEHIQ
ENCCU   --------------------------------------------------
ANTLO   --------------------------------------------------
ARATH*  ----------------------------------------ALLDEPGIEG
VITVI   ----------------------------------------CHADESEVDN
SOYBN   ----------------------------------------SQMGELDIEY
SOLLC   ----------------------------------------CHVEEMLGDS
ORYSJ*  ----------------------------------------RDVEIAKDPV
HORVU   ----------------------------------------QKEVHMDVLQ
SORBI   ----------------------------------------RDVAAAKDPT
SELML   ----------------------------------------SSAQD-----
PHYPA   ----------------------------------------RSIPDELES-
VOLCA   PMDLDPAPDTSGAAAVGGGDSGGATAPPPPPRILPPTAPPPEVSGLLARA
                         1010      1020      1030      1040      1050
```

```
HUMAN*  ------ETALPALVVPILEPCGNS-E----------------------CL
CHICK   ------ETALPTLVIPILEPCGRS-E----------------------CL
XENTR   ------ETALPTLVIPILEPCGRS-E----------------------CL
DANRE   ------ETALPTLVIPILEPCGRS-E----------------------CL
CIOIN   ---RNSVDQPVILVVPLLNNSARN-S-----------------------L
ANOGA   ------QGTPAMLTVPVLNPCLRA-E----------------------HI
TETNG   ------ETALPTLIIPILEPCGRS-E----------------------CL
DROME   ------QGTPAILTVPVLSPCLRA-E----------------------QI
BRUMA   -----TGRAAPTLTYPLLGAQSHADE----------------------ML
CAEEL   -----TGISAPSLSLALLPDTSSKDE----------------------MM
YEAST*  ----------------QLPTTCVSMA----------------------PI
SCHPO   --------------GGGLKIHVFDNE-----------------------I
CANAL   --------SMVSPHQLLLQISPKKST------------------------
CANGA   ----------------QLPTTCLSTA----------------------PI
ASPNI   SLSLHAYLTRTEPGDCQLDVQLTASR----------------------RL
RHIO9   -NGLHLIEDNGNNDESPVIVQYRHDK----------------------YI
CRYNE   SLQESTDPSARPLTLRVPLPSRYAVS----------------------HL
USTMA   RQGDEFSTATETTTFNFLQIDLHASQRPRTAKSKPNPKPEQRSASCLAPL
ENCCU   ------------------------------------------------TF
ANTLO   ------------------------------------------------SI
ARATH*  ---DNMVDSKERVEPEVLRVRAYGSS----------------------FF
VITVI   KK---VVSSRECEGQEVLRVRAYGSS----------------------FF
SOYBN   KQ-DDKCCSKDSEGHEVLCVRAYGSS----------------------FF
SOLLC   RKKDNKFDSREYQGQEVLRVRAFGSS----------------------FF
ORYSJ*  KK-TEQKDFADCCGNEVLQVRAYGQA----------------------YI
HORVU   KR-VDRRGFENCCTNEVLQVRAYGKS----------------------YI
SORBI   KN-AEKKGFSDCFGNEVLQVRAYGQA----------------------YV
SELML   ---------TSDIGEDSLHVRAYGDL----------------------YI
PHYPA   ---SGEEENMEEAGEEVLCVRAFGVS----------------------HM
VOLCA   APEEEVAGALVVLSAPVLEYLVSGTT----------------------EL
                         1060      1070      1080      1090      1100
```

MED14

```
HUMAN*   HIFVDLHSGMFQL--------------------ML---YG-LDQAT---L
CHICK    HVFVDLHSGMFQL--------------------ML---YG-VDQVT---L
XENTR    HVFVDLHSGMFQL--------------------ML---YG-FDQTT---L
DANRE    HIFVDLHSGMFQP--------------------ML---YG-VDQSM---L
CIOIN    RIYVDLQTGMLVP--------------------SL---YG-VDVSQ---L
ANOGA    YITVDTHTGMLRC--------------------HV--PKH-LDCPI---M
TETNG    HIFVDLHSGMFQP--------------------ML---YG-LDQSM---L
DROME    HITIDTHTGMFRC--------------------HV--PKH-LDCPI---M
BRUMA    VVSVNDFSGNVIA--------------------VV---RALGSCTE---L
CAEEL    TVSVNSFCGKVLC------------------------NVHILSAEHEDVL
YEAST*   QLKIDLLSGQFYF--------------------RN--PTPL--------L
SCHPO    IVKVNSVTGRLVL--------------------SS--SASPLSPPRH--L
CANAL    VFAINPLTGFFYF--------------------ID--PTPI--------Q
CANGA    QLKIDLVSGVFYF--------------------KN--PSPL--------L
ASPNI    RVAIEPLSGAIVL--------------------AA--TPNTLE------R
RHIO9    NIEVDSRTGKVRAHETKRSTNEGNGKYA-----II--KKTCTQFSFLVKL
CRYNE    LIGVSSFSGLLEV--------------------ED--EGAKGNDARAERA
USTMA    RLSVDPVTGRLLLDSEPAQSTTDLSSEQTSQQAASFTASILTTRPNYARL
ENCCU    EKGFTVRDGK--------------------------------DGCAFRSF
ANTLO    ERKINMGNVFFCS------------------------------------V
ARATH*   TLGINIRTGRFLL--------------------QS--SKSILTSSI---L
VITVI    TLGINIRNGRFLL--------------------QS--SRNILTPST---L
SOYBN    TLGINIRNGRFLL--------------------QS--SQNIVVSSA---L
SOLLC    TLAINIRNGRFIL--------------------HS--SKNVISSSV---V
ORYSJ*   GLGINIRSGRFLL--------------------QS--PENILPPSA---L
HORVU    HLGINIRSGGFLL--------------------QS--PKNILPPSA---V
SORBI    SLGINIRSGRFLL--------------------QS--PENILPRAA---I
SELML    CLSISIRNGRFVL--------------------RA--PSTLLSLSL---V
PHYPA    TLGISIRNGRFLL--------------------RC--PSTVLAAPM---V
VOLCA    KIGKHLFSGRLTLRPGQDE--------------RD--EGSLDHAAT---I
                  1110      1120      1130      1140      1150
```

```
HUMAN*   DDMEKSVND----------------------DMKRIIPWIQQL----KFW
CHICK    DDIEKCVND----------------------DMKRIIPWLQQL----KFW
XENTR    DDMEKSIND----------------------DMKRIIPWLQQL----KFW
DANRE    DDIEKSIND----------------------DMKRIVTWLLQL----KFW
CIOIN    ENMEKEINE----------------------NQKNLLTWISTL----HYQ
ANOGA    PEMQHALNN----------------------DWSKLQHLISEL----RYW
TETNG    DDIEKSIND----------------------DMKRIISWLQQL----KFW
DROME    EEMQDCLNG----------------------DRSKLPALLSEL----RFW
BRUMA    RELEQLIDD--------------------RVPLQTITDKMERL----RIL
CAEEL    AFGKALYSS--------------------QCSAHTIQMYLRKL----RVA
YEAST*   SNYASKINR--------------------AEGPEELARILQQL----KLD
SCHPO    RAAEKNIAL----------------------NTQPPAQILNRL----YFF
CANAL    TYITKKINS---PPPTLSQVSIKQSFIPESDMISYVIDQIIQL----RLE
CANGA    LEYVSQINR--------------------AETPEDLTSVLQRL----KLD
ASPNI    VDTDRNMDR---------------------STIDDIVSRVGRL----RCA
RHIO9    SGLEERLNN----------------------DPENICKHLLWL----RSD
CRYNE    AMSMKSVNE----------------------GKTKLVDDITRL----TTA
USTMA    AEASDRINE----------------------SIDALLDVLYRL----DVF
ENCCU    VDLDVFLER--------------------ERENSVFYSFFEE-----RFP
ANTLO    AELQEHLYR--------------------EKEDAAFYSMLKHC----KFI
ARATH*   EEFEDALNQ----------------------GSISAVDAFINL----R--
VITVI    SDCEEALNQ----------------------GSMTAAEVFISL----R--
SOYBN    LECEEALNQ----------------------GSMTAAEVFISL----R--
SOLLC    VECEEALNQ----------------------GSMSAAEAFISL----R--
ORYSJ*   LDCEEALNK----------------------GSITATDVFASL----R--
HORVU    LDSEEALNK----------------------RSITPPEVFVSL----K--
SORBI    MDCEEALNK----------------------GSTSATEVFSSL----R--
SELML    QEMEEAINQ----------------------GT-SVVDVFINL----R--
PHYPA    KEMEDALNL----------------------GSVQALDIFMKL----R--
VOLCA    AMNEDYVNQAAMAAYNSASADSCPAWRALKEACVALCHVLQRTHTSTDAT
                  1160      1170      1180      1190      1200
```

MED14

```
HUMAN*  LGQQRCKQSIKH--------LPTISSE--TLQL--------SNYSTHPIG
CHICK   LGQQRCKQSIKH--------LPTVSSE--TLQL--------ANHASHPVG
XENTR   LGQQRCRHSVKH--------LPTVCSE--TLHL--------TNYTTHPIS
DANRE   LGEQRCRQSVKH--------LPTLCTD--TLHL--------SNLTTHPVG
CIOIN   LLRQLCKNAVQH--------LPVVYLD--MVPV--------LEKS--PAV
ANOGA   ITQRRCEKTLQH--------LPAATQD--RLPL--------IYSHTHPIA
TETNG   LGEQRSRQSVKH--------LPTVCTN--VLHL--------SNSASHPVG
DROME   ITHRRCDKTLQH--------LPATATE--TLPF--------LVQPDQEIL
BRUMA   LMLERYRKAVAP--------LPVRIVP-----------ENVFCARLSSLP
CAEEL   LVIERYRRSVKA--------LPVREVQ----------EAELLPFAKECLG
YEAST*  KIIHVLTTMFEN--------TGWSCSR--IIKIDKPIRTQVNTGGESVVK
SCHPO   CIQTQLLEV-------------AQCAE--LHAVQGYYSFPYLTFSKGKWR
CANAL   VFNKEVNTKLAT--------TAWINNG--IIKLSDHELSKLTQFLIQNEE
CANGA   KIVHIIRNMFDK--------TGWVCSK--VIKLDNPIATSVNS-------
ASPNI   AAIEEVESQVKM--------LGFISVSPRNVRI-----------------
RHIO9   MVIREIISLAK------------------LLNLQPYHSSQMNLRPEDFTK
CRYNE   IVVENIEAQMRQ--------LGWKPTRQIALRLKDLIKADLHP-------
USTMA   ARVQEWERMASY--------LGLRSVR--KLALRPQDLAKLGASTLASAE
ENCCU   CYRNHRSSMFGD--------------------------------------
ANTLO   KNYKRKDFGMRN--------------------------------------
ARATH*  ------SKSILH--------FFAAIGK--FLGLEVYEHGFGINKVPKSLL
VITVI   ------SKSILH--------LFASIGS--FLGLEVYEHGFAAVKLPKHIL
SOYBN   ------SKSLLH--------LFASIGR--VLGLEVYEHEFNTVKIPKNVS
SOLLC   ------SKSILH--------LFACIGR--FLGLEVFEHGSAAVKVPKSIS
ORYSJ*  ------TRSILH--------LFAATGS--FFGLKVYEKSQGTLKIPKDIL
HORVU   ------TRSILH--------LLAATGR--FLGLK----------------
SORBI   ------TRSILH--------LFAASGS--FFGLKVYQQSQGTLKIPKTIL
SELML   ------NQSIIH--------HYTSLGG--SMNLKVFEKGAITLKFPEDGP
PHYPA   ------TKCLVH--------YFGLIGK--SLNLKLYERGASSVKVPAEGP
VOLCA   HFIQAARRAALHISRLPHGMLEAFTATTPGLAIEPRPREKVATWALQCVP
                 1210      1220      1230      1240      1250
```

```
HUMAN*  NLS-------------------KNKLFIKL---TR-LPQYYIVVEMLEVP
CHICK   NLS-------------------KHKLFIKL---TR-LPQYYIVVEMFDVP
XENTR   NLS-------------------RHSLYIKL---TR-LPQYYIVVEMFEVP
DANRE   NLS-------------------KHRLFIKL---TR-LPQYYIVVEMFDVP
CIOIN   KDD-------------------PGRIYIRL---NH-HPSYYLIVE-FHIA
ANOGA   RMG-------------------PHKVFIQL---YR-HANVILIVELKEKK
TETNG   SLS-------------------KHKLFVKL---TR-LPQYYIVVEMFEIP
DROME   QPG-------------------RHKIYVKL---HR-HPNIVLVVQLKEKT
BRUMA   ELP-------------------PDRICLQF---IK-EEMYYLIVSF--TP
CAEEL   DAP-------------------AQRIILQF---LR-SEDYYLLVTF--SP
YEAST*  KEDNKYAIAGNSTTNSDVSLLLQRDLFIRL---PHWPLNWYLILSIISSK
SCHPO   KDG-------------------DSLWVLAY---NVESNSWSVRL------
CANAL   QEGDDNEEDSSTNTRLDSV---LQSFKVQFYRRKNWPSSWFLINMI--SG
CANGA   -EEIK-------------TNLLQNDMFIRL---ASWPVNWYLILTIVSSN
ASPNI   ---------------DARSLFPANVLRFSFFWHHHWERSWLLAATS----
RHIO9   LFG-------DFVPSNKTERFPAHCVFLQF---SQFENWYFVLATI----
CRYNE   ----------------------VTTVFVPL---ASSPNHYFVTKAV----
USTMA   A---------------------APQLFLPL---RQSFGGYFLALQPSELA
ENCCU   ----------------------RNVFIMRN--------GLFVCVRLVRLV
ANTLO   -------------------------VFMRV-------GLNFVCIKL----
ARATH*  D-G-------------------SSILTLGF---PDCESSHLLLMEL----
VITVI   N-G-------------------SNLLLMGF---PDCGSSYFLLMQL----
SOYBN   N-G-------------------SAMLLMGF---PDCGSSYFLLMQL----
SOLLC   S-G-------------------TNLLLMGF---PECGSSYFLLMEL----
ORYSJ*  H-G-------------------SDLMVMGF---PQCANAYYLLMQL----
HORVU   --------------------------------------------------
SORBI   H-G-------------------SDLMVMGF---PHCANAYYLLMQL----
SELML   KLG-------------------SDVLVLGF---PNTGDLYFLAVHL----
PHYPA   KLG-------------------GDVLIMGF---PDCDESFFLCVQL----
VOLCA   FPPNFPSLEPVA----------PDTLPLRM---PQCL-TFFLQSRL----
                 1260      1270      1280      1290      1300
```

MED14

```
HUMAN*   NKPTQLSYKYYFMSVNAADREDS---------------------------
CHICK    GNPTELEYKYYFLSVSYAEGDDN---------------------------
XENTR    GSPTQLEYAYHFLSVSHTEGEDN---------------------------
DANRE    SNLTELQYKYYFLSVSQMESEDG---------------------------
CIOIN    G---ETNNKYKLMKTSSPTGFQK---------------------------
ANOGA    TCPNEMTYTFYLVLVKPSSVEEGQSPDVLSG-------------------
TETNG    NSPTALQYKYSFLSVSQLEGEDG---------------------------
DROME    TMPNEMEYTFHLGFV----AYQKDELDVIDD-------------------
BRUMA    NGQIGVNIDMYLLSNVER--------------------------------
CAEEL    DEKAVVKTHIQLLEVVGD--------------------------------
YEAST*   TSCVVEKRIGKIVSQRGKWNLKYLDNSNVMT-------------------
SCHPO    -----LNAAGQTLYTQDVHTTKGTLSIES---------------------
CANAL    VTTKSFWWVARIKSISGDWKIQWAQIIKF---------------------
CANGA    SSCIIEKRIGKIISTSGKWKVQYLDTTNVLS-------------------
ASPNI    --SMDGDKWWVVQTPSADSVTINRSLDAAVH-------------------
RHIO9    --KNEFKSWLCCLNKIYDQNSIYQAIVDFIYIDCDELRK-----------
CRYNE    ----QNGIVFELLKVAKVPNENGLGMKYMVG-------------------
USTMA    GVSIALVYVVQVMDPTGTPALSVQSIEWLD--------------------
ENCCU    EGSMEVRIFVGNCE------------------------------------
ANTLO    --------------------------------------------------
ARATH*   --EKDFTPLFKLLETQMDGSGKPQSFNDPSN--ILRAKKIDIGQIRILED
VITVI    --DKDFKPLFKLLETQPDPSGKSSSFGDMNH--VIRIKKIDIGQMQMFED
SOYBN    --DKDFKPLFKLLETQPNPSVKDNLSGELNQ--VLRIKEIDIGQMQVHED
SOLLC    --DKDFKPVFKLLESRSDSPAKAQSLADLSN--VVRVETIDVGRMQICED
ORYSJ*   --DKDFRPVFHLLETQSDANDKTNTNACTKE--ALRFNKIDIGQIQISKS
HORVU    --------------------------------------------------
SORBI    --DKDFRPVFHLLETQCDANDKTNANGDAKE--AIRFNKINIGQMQILKN
SELML    --DPAFMPLFVLLEAQKLAPLPNRTTVAAKHLVIRRYMNIDISKFSLAGN
PHYPA    --DANFTPLFTLLEAESQSSSGHASLASSAF-NISRWMRIDIEAMPILRD
VOLCA    ----SLTSPHVLVVCSCNKQLMPIKVWQANA-------------------
                      1310      1320      1330      1340      1350
```

```
HUMAN*   --------------------------------------------------
CHICK    --------------------------------------------------
XENTR    --------------------------------------------------
DANRE    --------------------------------------------------
CIOIN    --------------------------------------------------
ANOGA    --------------------------------------------------
TETNG    --------------------------------------------------
DROME    --------------------------------------------------
BRUMA    --------------------------------------------------
CAEEL    --------------------------------------------------
YEAST*   --------------------------------------------------
SCHPO    --------------------------------------------------
CANAL    --------------------------------------------------
CANGA    --------------------------------------------------
ASPNI    --------------------------------------------------
RHIO9    --------------------------------------------------
CRYNE    --------------------------------------------------
USTMA    --------------------------------------------------
ENCCU    --------------------------------------------------
ANTLO    --------------------------------------------------
ARATH*   DLNLITSDVVKFVSSFSDAEGINQASGHR--QPGLVDEALTEMSGSQLSF
VITVI    ELNLSLVDWGKLLSFLPNAGVPNQTSEHGLLSEFSLESSMHNPGCPPTSF
SOYBN    EMNLSLVDWGKLRSVLPNAVCPNQTSGHEFFSDIRLENSIQIARGHPSGF
SOLLC    ELNLSLLNSKKLLSVLRSDGGSHQTSENSLLADFSLEGSIV-ASGVQSTF
ORYSJ*   ESNTNMFDAK--LHALQNIGNCDDVMENRLPIQSGIEPLPLLPACSPS-F
HORVU    --------------------------------------------------
SORBI    ETNANPFDMK--LQALQSLVDPADMMESDLPVQNGIETLPLLPACSPS-F
SELML    DENLSLLSDN---------------------VKSGGRSLVPKTAEVRNGF
PHYPA    DTGFSLLEQ-------------------SGSEGREGYGSMVVKRVEEEGY
VOLCA    --------------------------------------------------
                      1360      1370      1380      1390      1400
```

MED14

```
HUMAN*  --------------------------------------------------
CHICK   --------------------------------------------------
XENTR   --------------------------------------------------
DANRE   --------------------------------------------------
CIOIN   --------------------------------------------------
ANOGA   --------------------------------------------------
TETNG   --------------------------------------------------
DROME   --------------------------------------------------
BRUMA   --------------------------------------------------
CAEEL   --------------------------------------------------
YEAST*  --------------------------------------------------
SCHPO   --------------------------------------------------
CANAL   --------------------------------------------------
CANGA   --------------------------------------------------
ASPNI   --------------------------------------------------
RHIO9   --------------------------------------------------
CRYNE   --------------------------------------------------
USTMA   --------------------------------------------------
ENCCU   --------------------------------------------------
ANTLO   --------------------------------------------------
ARATH*  SSVVDGVFGLQKVTS--------------------------ALMSIDGHG
VITVI   SSIVDEVFELEKGASLPPFSVPNLSSSY-SSPGSHFGAGPMNLPGMKAGA
SOYBN   SSLVDEVFGLEKGSSTPPFSVKNLSSSVNTSLPSQYGSVPMTLHSLKAGS
SOLLC   LSIVDEVFELEKGSSVPSFSGQIPPSTFGASPASHFGTGVANYQSLKVGT
ORYSJ*  SSVVDEVFEYEHGALAVPNHSLPQTT---LQSTSHPGSLSVGFQGVGTRA
HORVU   --------------------------------------------------
SORBI   SSIVDEVFEYERGSTAAQNH-----------------SLSVDIQGINARV
SELML   DRIDNQVGKLEKAEQQPNFAMGQLQRYHAS-------------PSVSLGS
PHYPA   SKL------------------------------GIPPVPKGPIRGGGLGL
VOLCA   --------------------------------------------------
                 1410      1420      1430      1440      1450
```

```
HUMAN*  --------------------------------------------------
CHICK   --------------------------------------------------
XENTR   --------------------------------------------------
DANRE   --------------------------------------------------
CIOIN   --------------------------------------------------
ANOGA   --------------------------------------------------
TETNG   --------------------------------------------------
DROME   --------------------------------------------------
BRUMA   --------------------------------------------------
CAEEL   --------------------------------------------------
YEAST*  --------------------------------------------------
SCHPO   --------------------------------------------------
CANAL   --------------------------------------------------
CANGA   --------------------------------------------------
ASPNI   --------------------------------------------------
RHIO9   --------------------------------------------------
CRYNE   --------------------------------------------------
USTMA   --------------------------------------------------
ENCCU   --------------------------------------------------
ANTLO   --------------------------------------------------
ARATH*  LVPK-----NLSAVTGHGKAPMLTSYHSDSLYNR---QGPLQSSSYNMLS
VITVI   SSP------------------NVAPHYGGSLYSSGNMKGSMQSSSVSLQS
SOYBN   PSPKWEVGMQMPLVSNVTKASSATNHYSGSLFSSGSVKGPVQSSSVGSIP
SOLLC   LSPKWDRG---------------VGNYSNSMY-----KGVIQSGSVGSLA
ORYSJ*  NASIEG------ASSAYSG----SKFSPGVGLNSYLPSNLRHVQSTNAFS
HORVU   --------------------------------------------------
SORBI   VSPMHD------GSLSHTQANNIAKIHPSGSLNSYFPSNFRYLQGVN---
SELML   PSSRASFGNHNRKSSSSNNLLALVSQ---------------------AMG
PHYPA   DGGQSGLDQYYGGGGASHKGNMLAVHSPSNLQG----------------G
VOLCA   --------------------------------------SPPHQRTRVGFA
                 1460      1470      1480      1490      1500
```

MED14

```
HUMAN*  --------------------------------------------------
CHICK   --------------------------------------------------
XENTR   --------------------------------------------------
DANRE   --------------------------------------------------
CIOIN   --------------------------------------------------
ANOGA   --------------------------------------------------
TETNG   --------------------------------------------------
DROME   --------------------------------------------------
BRUMA   --------------------------------------------------
CAEEL   --------------------------------------------------
YEAST*  --------------------------------------------------
SCHPO   --------------------------------------------------
CANAL   --------------------------------------------------
CANGA   --------------------------------------------------
ASPNI   --------------------------------------------------
RHIO9   --------------------------------------------------
CRYNE   --------------------------------------------------
USTMA   --------------------------------------------------
ENCCU   --------------------------------------------------
ANTLO   --------------------------------------------------
ARATH*  SPPGKGSAMKKIAISNSDQELSLILSPSL---------------------
VITVI   SAPVRSAAGKKLSASKSDQDLASLRSPHS-LEIGSGTTMDEDHLRLLSDS
SOYBN   TGQGRNSAGTKLSASKSEQDLASLKSLHS-VDSSSSAAMDEEQLRVFSDN
SOLLC   A----TQTGKKLTASKSEQDLTSLRSPHS-AGVGSYTSLDEDQLTV----
ORYSJ*  SSTVTKSSSIKLPSSNSNHELSSLSSPT----------------------
HORVU   --------------------------------------------------
SORBI   -----KSLHLVPSSNNNSSQIPAQSSHS----------------------
SELML   SPARNSFSRMSPSTKLPELEYGRLKSPLLHVDNPASPDLDDDHLSKLIES
PHYPA   SPMQHAGSPLQHG----NVGYGVNAGPIM---------------------
VOLCA   SPRHSTALHDRAKKYITWHDMAAYGTVLSCIPVPASTYRLPDHVVAAYMA
                            1510      1520      1530      1540      1550
```

```
HUMAN*  --------------------------------------------------
CHICK   --------------------------------------------------
XENTR   --------------------------------------------------
DANRE   --------------------------------------------------
CIOIN   --------------------------------------------------
ANOGA   --------------------------------------------------
TETNG   --------------------------------------------------
DROME   --------------------------------------------------
BRUMA   --------------------------------------------------
CAEEL   --------------------------------------------------
YEAST*  --------------------------------------------------
SCHPO   --------------------------------------------------
CANAL   --------------------------------------------------
CANGA   --------------------------------------------------
ASPNI   --------------------------------------------------
RHIO9   --------------------------------------------------
CRYNE   --------------------------------------------------
USTMA   --------------------------------------------------
ENCCU   --------------------------------------------------
ANTLO   --------------------------------------------------
ARATH*  ----------------------------STGNGVSESGS----------R
VITVI   SKEA----------------------------------------------
SOYBN   SNDA--LAGSRSSRLLSPP--------RPTGSRMSIPNSRPNGPQVESFK
SOLLC   -------STNRSARLLSPPHRVSASSGKASGSRNSAVGTLPGG-----FR
ORYSJ*  ----------------------------------------------EHVI
HORVU   --------------------------------------------------
SORBI   ----------------------------------------------GNL-
SELML   ISTGGPATDNMASTGLRQVHSSPSSRTVKSASKNS---------------
PHYPA   -------VSPSMASGGYHQGKSLGGSPAFGDGSHR---------------
VOLCA   ASTAAHGGGSGGSGGGHGTVNDNGLHSPHSGG-----NSPRAAANGHEVG
                            1560      1570      1580      1590      1600
```

MED14

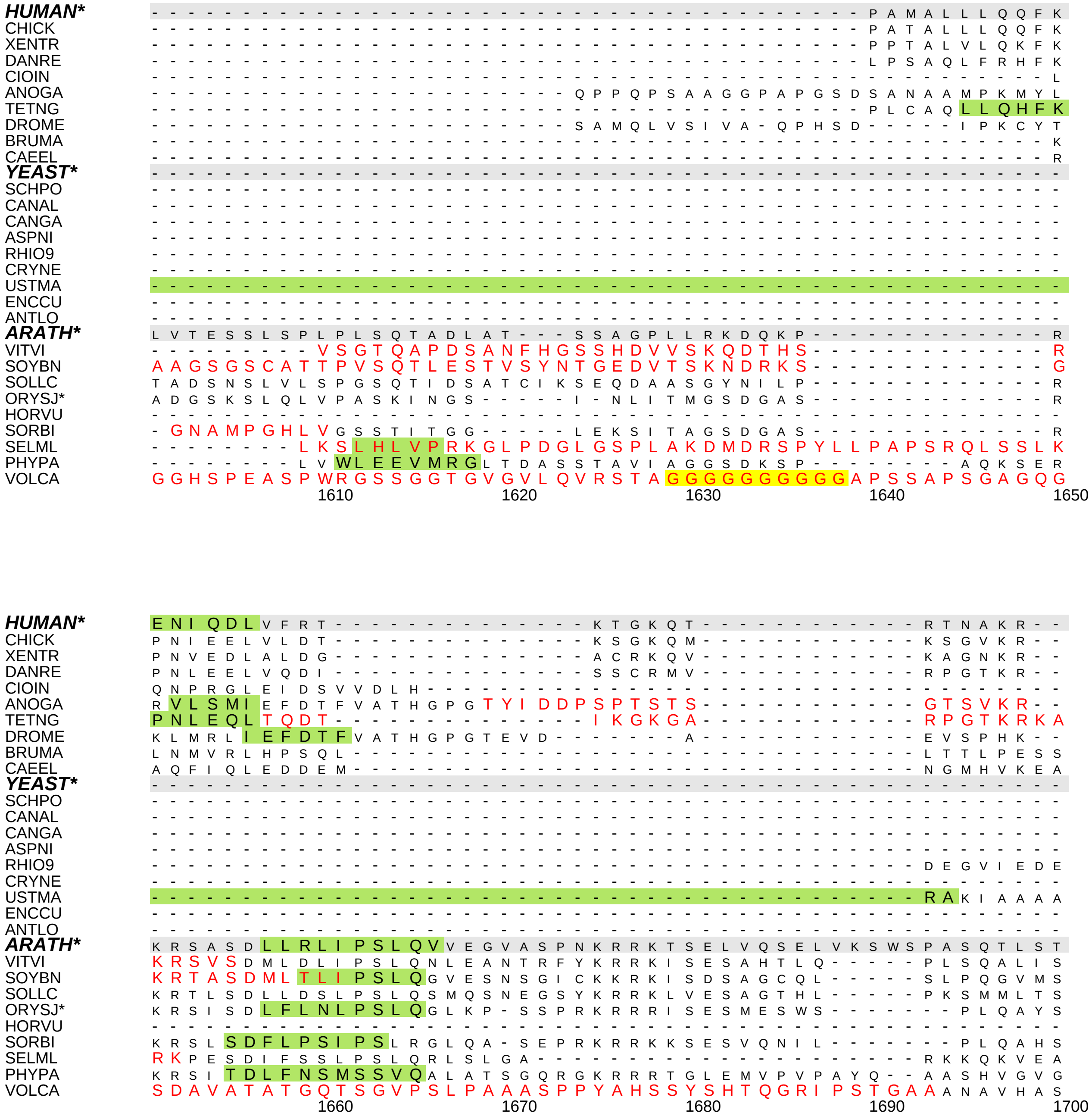
HUMAN*
CHICK
XENTR
DANRE
CIOIN
ANOGA
TETNG
DROME
BRUMA
CAEEL
YEAST*
SCHPO
CANAL
CANGA
ASPNI
RHIO9
CRYNE
USTMA
ENCCU
ANTLO
ARATH*
VITVI
SOYBN
SOLLC
ORYSJ*
HORVU
SORBI
SELML
PHYPA
VOLCA
1610
1620
1630
1640
1650
1660
1670
1680
1690
1700

MED14

```
HUMAN*   ---KLSDDPCPVE----SKKTKRAGEMCAFNKV----------------L
CHICK    ---KLSGDPCSIE----PKKPKRTGEMCAFNKV----------------L
XENTR    ---KLSGEQGSPE----SKKPKRTGEICAFNKV----------------L
DANRE    ---KLSGEQSAVE----PKKPKRSGEMCAFNKE----------------L
CIOIN    ---YLFDQTGL-----------EKNEDCV--KE----------------L
ANOGA    ---KVSPLDNALSAIGPPLKQQKTIYPAYFIPE----------------L
TETNG    ATAKMSGEHGDPE----PKKPKRSGEMCAFNKE----------------L
DROME    ---RKSNGD----LLAPPAKQQKTIFPAYFIPE----------------L
BRUMA    FLSGKSDDD--------LEPPTKRRRWLGDKRL----------------L
CAEEL    INQGTMRFS--------PSHKTSLQEECSREQR----------------L
YEAST*   ---------------------------------------VKLESITYQKI
SCHPO    -------------------------------------------------F
CANAL    ------------HGDNAKQELSPNESKVFDKPKRTVDCSIESEQLNFEFF
CANGA    ---------------------------------------LKLESITYKKI
ASPNI    -----------------------HASSAVRSAQVICNLLLPAEQPDYSSL
RHIO9    CNKKRRSEELKEEDCLKKRKTSNEACDMIHLKQ---SDKADNLFMDFQYL
CRYNE    -----------------DRTMMDLGKLKARRKSGDDKLQRSAFEVQNKDL
USTMA    SKVGSSGEDVGQEFVATEAGTKRKAPPGVQGETNDVDANFGSSELTIEEL
ENCCU    -------------------------------------------------L
ANTLO    -------------GRVENEGVMHHIEADVFVGK----------------L
ARATH*   AVSTSTKTIGCSYGNLIAEANKGNAPSSVFVYA----------------L
VITVI    S-EIACKTEGYSYGNLIAEANKGNAPSSVYVSA----------------L
SOYBN    A-EIIPRTEGYSYGSLIAEANKGNVPSSIYVAA----------------L
SOLLC    S-DISGKTEEYSYGSLIAEANKGNAPSSIYVSS----------------L
ORYSJ*   S-DSQSRT-SLTYGNILAERNN-CVPATTYASV----------------L
HORVU    --------------------------------------------------
SORBI    S-NLQSRV-SLTYGDVLAERNN-CVPATIYASV----------------L
SELML    PSNALEVFSGQPYAAVVAAANQGKAPPVTHMAV----------------L
PHYPA    STAVSQSVLGQSYGLVVRAANQGKASAGAYRAV----------------F
VOLCA    CGGGVRAAVGSKPGGASGRPTGQQVAVMIAQQQ----------------L
                  1710      1720      1730      1740      1750
```

```
HUMAN*   AHFVAMCDTNM---PFVGLRLELSNLE-IPH-------------------
CHICK    AHIVAMCDTNM---PFIGLRLELSNMD-IPH-------------------
XENTR    AHFVAMCDSNM---PFIGLRMELSTME-IPH-------------------
DANRE    AHLVAMCDTNM---PLIGLRSELSNME-IPH-------------------
CIOIN    IKMISICESRI---PFISLLQGIAGEEGIIH-------------------
ANOGA    AHVVAMCDEKL---PFVTLAKEFSMRK-IPH-------------------
TETNG    AHVVAMCDTNM---PFIGLRTELSHME-IPN-------------------
DROME    AHVVAMCDEKI---PFMNLAQTLSKHN-IPH-------------------
BRUMA    TSAVATLDDRL---AFMRICEELERRN-VKY-------------------
CAEEL    AFAVATVEDRI---TYMYLAAELMKKG-IGV-------------------
YEAST*   MILQRTILNRI---INHMLIDSLNQLE-IRNKICSSEMINEQKLPQYIIQ
SCHPO    SRLSYLLEVQI---LLFNVQTACQARG-MPFEYLPIPPKALI--------
CANAL    KTLSTLSSNLI---LDHMILEELQVRS-IKFIKLDWETIDDNKIFSKFKQ
CANGA    ISLQKSTLNRI---INHTLIDSLNHLK-TRNKVCSPDLI-VNSLPKHLLE
ASPNI    ADLGHCLSGFLAIYANARFLQDLQFIKIWPH-------------------
RHIO9    AKLNSFCRGYI---TNRKIELQLQIYKEALH-------------------
CRYNE    KDLFILSNALV---AQTIVEQQLKDRS-IPYIQQFPPTAGPASP------
USTMA    ADVHSYCIALV---SYFRVEQQLRMRG-LPYLHVGSNTMRSSPPCKRQRR
ENCCU    VDFLSYTEVKLEVGSNGEVVGILGRRE-GPG-------------------
ANTLO    IENNHLYYVKLLSRYNGDILLTNSCRHAIDW-------------------
ARATH*   LHVVRHSSLSI---KHAKLTSQMEALD-IQY-------------------
VITVI    LHVVRHCSLCI---KHARLTSQMEALD-IPY-------------------
SOYBN    LHVVRHCSLCI---KHARLTSQMDALD-ISY-------------------
SOLLC    LHVVRHCSLCI---KHARLTSQMEALD-IPY-------------------
ORYSJ*   LHVIRHCSLSI---KHAQITAQMDSLA-IPY-------------------
HORVU    --------------------------------------------------
SORBI    LHVIRHCSLCI---KHAQLTAQMDSLA-IPY-------------------
SELML    LHVVKRSWLVI---KHARLTNQMDSLG-ISY-------------------
PHYPA    QQVEERCRLCI---KHARLTRQMDAQG-IPY-------------------
VOLCA    SSVVRWCRRRM---VWEVLLVQLKAVG-ATFVELCNPPC-----------
                  1760      1770      1780      1790      1800
```

MED14

HUMAN*	-------------------------------QGVQVEGDG-FSHAIRLLK
CHICK	-------------------------------QGVQVEGDG-FSHAIRLLK
XENTR	-------------------------------QGVQIEGDG-FSHAIRLLR
DANRE	-------------------------------QGVQVEGDG-CSHAIRILK
CIOIN	-------------------------------QGVYEEEN--LALVCKLKT
ANOGA	-------------------------------GGLQVEANA-TSLVLKLLT
TETNG	-------------------------------QGVQVEGDG-SSHAICLLK
DROME	-------------------------------SGLQVEANA-TSLVLKILA
BRUMA	-------------------------------KPLTVEPTV-GGLILHITD
CAEEL	-------------------------------DVRKDSAHVPGGLALHITD
YEAST*	GS---------------------------------NTNDNISIITLELES
SCHPO	-------------------------------EDDFTTYVQTGCLCIMMPS
CANAL	NHDISLKRKNSHNINID--------------VNVDVADNTSTTTNTKYIR
CANGA	DEVDDKV------------------------PHSIENYHHISLITIELES
ASPNI	------------------------LEHLRIGPGLQVPDLNIEYEATKLPE
RHIO9	---------------YRKRPLLKTISPTETQVKNHPISHKMETICISRQD
CRYNE	-------------------------------KSTSPLAGMVPTICVDVRD
USTMA	QQTADARDDGLFGEDASGGTASSDDEAFAGPAGVAGVAALVPSLCLRSID
ENCCU	--
ANTLO	--
ARATH*	-------------------------------VEEMGLRDAFSDIWFRLPF
VITVI	-------------------------------VEEVGLRNASSNLWFRLPF
SOYBN	-------------------------------VEEVGLRSGSSNIWFRLPL
SOLLC	-------------------------------VEEVGLRSASSNLWFRVPF
ORYSJ*	-------------------------------VEEVGLRSPSSNLWLRLPF
HORVU	--
SORBI	-------------------------------VEEVGLRTPSSNLWLRLPF
SELML	-------------------------------VEE-GRLSSSAGLIFRLAT
PHYPA	-------------------------------VNEVGLREPSTVLRFRLPG
VOLCA	-------------------------------QRVRVTAVAGTALPCHMVR
	1810 1820 1830 1840 1850

HUMAN*	IPPCKG--
CHICK	IPPCKG--
XENTR	VPPCKG--
DANRE	VPPCMA--
CIOIN	LPKVSG--
ANOGA	LPQPKPPQAPPTPQQQQQQQQQQQQPGTSDAKSSGAGASANEPK-TVHVP
TETNG	IPPCKS--
DROME	LPQPGKTNF------ATQQQQQQGAP---------AVAGENKPSGTSGLP
BRUMA	FSEAIP--
CAEEL	VKNVVP--
YEAST*	FL--
SCHPO	--
CANAL	APKLKYESMFL---------------------------------------
CANGA	FL--
ASPNI	ALRVAL--
RHIO9	LLRTCAYYHH--
CRYNE	LLRA--
USTMA	VLGPSKSHLAKPNISLR---------------------------------
ENCCU	--
ANTLO	--
ARATH*	A--------------QNDSWQHICLQ------------------------
VITVI	S--------------SGDSWQHICLR------------------------
SOYBN	A--------------RGDSWQHICLR------------------------
SOLLC	A--------------RDDTWQHICLR------------------------
ORYSJ*	A--------------RDDSWKHICLR------------------------
HORVU	--
SORBI	A--------------QDDSWKHICLR------------------------
SELML	S------RMRGDMGKYCNAWQRISLS------------------------
PHYPA	TVPSGDPRTRPSRSDGSYGWQQMGLC------------------------
VOLCA	RRGLTPPRVGPTCLELELGPDPLAGQVTARLYGRFLCSPGCAPLDRNTAA
	1860 1870 1880 1890 1900

MED14

```
HUMAN*   -ITEETQKALDRSLLDCTFRLQ-----GRNN---RTWVAELVFANCPL--
CHICK    -VNEETQKALDRSLLDCTFRLQ-----GRNN---RTWVAELVFANCPL--
XENTR    -TSEETQKALEQSLLDCTFRLQ-----GRNN---RTWVAELVFANCPL--
DANRE    -VGEETRKALERSLLDCTFRLQ-----GRNN---RTWVAELIFSSCPL--
CIOIN    -TNELTTEILHDSILECSFRIQ-----ERIT---QMWVAEIL-----I--
ANOGA    PIDKQVWNALLKRLLSVSVRAQ-----VNKSNQTRLWTMELVFYGSPL--
TETNG    -VGEETRRALERSLLDCTFRLQ-----GRNN---RTWVAELVLANCPL--
DROME    KIDSHVWDDLMRRVLSISIRSQ-----TNKNSQVRIWVVEFVFYSTPL--
BRUMA    ----AGSDDFFSSVINCCLRLD-----TRTR---VIWPFECTLINTPLVA
CAEEL    ----FEASEFFECCIRCCLRLD-----NRNR---YTFQFEMCFENIPLVR
YEAST*   -EGSKALNSILESSM--FLRID-----YSNSQIRLYAKF-----------
SCHPO    -SNEDMLPVVFVRAHDGQLIFD-----SRI--------------------
CANAL    -IYNDKLLPIYNSATILFLKIE-----LVESNKMYLKLFGNLR-------
CANGA    -EGSKALSGILESTM--FLRMD-----YKKFEIRLYGKF-----------
ASPNI    -PAGFKRKTFVKKTIRLAFHGI-----DRCRKVAIMVAYGNLSTSFPALG
RHIO9    -QRDGKKKEKRIETVPWIEKLL-----SRMKNEILIRSFGWWDCDRGECY
CRYNE    -GRGGGSAAMEVAMPKVWLQIEGWWEGGKCEVVTIVVLRHQPSTVAPGTV
USTMA    -VRHWDDDEKLCVEMRIKLRMK-----SRQFRTLHEVVA-----------
ENCCU    --------------------------------------------------
ANTLO    --------------------------------------------------
ARATH*   -LGRPGSMCWDVKINDQHFR--------------DLWELQKGSKTTPW--
VITVI    -LGRPGSMYWDVKIIDQHFR--------------DLWELQKGSSNTTW--
SOYBN    -LGRPGCMYWDVKINDQHFR--------------DLWELQKGINNTPW--
SOLLC    -LGRPGSMYWDVKINDQHFQ--------------DLWELQKGSNSTPW--
ORYSJ*   -LGKAGSMSWDVRINDPHFR--------------ELWELSTGSTTTSW--
HORVU    --------------------------------------------------
SORBI    -LGKAGSMSWDVRINDPHFR--------------ELWELNGGSTTTQW--
SELML    -LGQHGSDGWEVKVRDSHYK--------------SLWSLQK-QKVADW--
PHYPA    -LGKPGSEGWEVKVMDAYFS--------------GLWKLQKQSEGVG---
VOLCA    AAGGPGARAFASATAGFVLDEQ-----GALA-------------------
                  1910      1920      1930      1940      1950
```

```
HUMAN*   ---------NGTSTREQGPSRHVYLTYEN-------------LLSEPVGG
CHICK    ---------NSTSSREQGPTRHVYLTYEN-------------QLSEPVGG
XENTR    ---------NSIVSREQGPTRHVNLTYEN-------------PLSEPVGG
DANRE    ---------NSTSIKEQASTRHVYLTYEN-------------PLSEPVGG
CIOIN    ---------KSLFPSNDVGVDRVMLTYDA----------FGGQANNPKP-
ANOGA    ---------PSLHHKEQGMRRAVYLQYEM-------------QPVESVS-
TETNG    ---------NSTHSKEQASTRHIYLTYEN-------------PLSEPVGG
DROME    ---------QSSHPKEQGSRRTVYLTYE--------------QANYDFS-
BRUMA    DFYEPIMHVDYESAKTKTTVLEIH---------------GSGGVSSPNTS
CAEEL    DVPHGLPHRRDGEPKDRTSKDATWLQELN---------------HINQSS
YEAST*   ----------------KRNTMMIQCQIDKLYIHFVQEEPLAFYLEESFTN
SCHPO    ---------KGKLPYQSETETEKNCYIDW----------RTGRITIRVQN
CANAL    ---------NLQIKNTSEDIQKLHLNIDE-ANQYFEIDNTVDLSTVINEP
CANGA    ----------------KRSTMAFHCQCDDLLINFVEREPLAFYLAETFTN
ASPNI    DLV-------------AEDDHSLVLQKTG----------TGFALRLLASP
RHIO9    VVFQDTLDCDNITL--QSNDIGDHISLDK----------LNSTVSFTYAN
CRYNE    SQE------GEDEKDRGESAKAEGISFDP----------KSSIVRFRAKD
USTMA    ---------LAASSDQQSTCALTWIDFDR----------EASLLTLTTRD
ENCCU    ----------------------------------------------PNIG
ANTLO    -----------------------------------------NPECKTTVP
ARATH*   ---------GSGVHIANSSDVDSHIRYDP----------EGVVLSYQSVE
VITVI    ---------GSGVRIANTSDIDSHIRYDP----------EGVVLSYQSVE
SOYBN    ---------GSGVRIANTSDIDSHIHYDP----------DGVVLSYQSVE
SOLLC    ---------DSGIRIANTSDADSHIRYDC----------EGVVLSYYSVD
ORYSJ*   ---------GVGVRVANTSEMDSHISFDA----------EGVILTYSNVE
HORVU    --------------------------------------------------
SORBI    ---------GVGIRIANSSEMDSHISFDS----------NGVVLTYNTVE
SELML    ---------GAGVQLAALSEADAHIKCSQ----------EGLILSYSTVE
PHYPA    ---------GTGEQFASPPQDDSHMQCTQ----------EGLVLKYSTVQ
VOLCA    ---------GGATHNGSSASGDPGLVCLR---------------RQYCLE
                  1960      1970      1980      1990      2000
```

MED14

Species	Sequence
HUMAN*	-RKVVEMFLNDWNSI---ARLYECVLEFA---------------------
CHICK	-RKVVEMFLNDWNSI---ARLYECVLEFA---------------------
XENTR	-RKVVEMFLNDWNSI---ARLYDCVLEFS---------------------
DANRE	-RKVVEMFLNDWNSI---SKLYECVLEFS---------------------
CIOIN	--NVIKSFVEDWSCI---VRLFGLAFAYK---------------------
ANOGA	--KVVDQLLSDWSKI---VYLYTLVHEFR---------------------
TETNG	-RKVVEMFLNDWTAI---SQLYQCVLNFS---------------------
DROME	--KTVEDLLNDWSKI---VYLYTLVYDFA---------------------
BRUMA	-ESVSTHMIDRLIVF---AHMFDPVKEFA---------------------
CAEEL	PEKLVELIIHRLMRY---LYMYKVVHQFS---------------------
YEAST*	LGIIVQYLTKFRQKL---MQLVVLTDVVERLHKNFESEN-----------
SCHPO	----FSSFEKTWIGL---LKLVALSKTSA---------------------
CANAL	KTLLLNLIFNTLNKLNSLIKILDQLNKSN---------------------
CANGA	LNEIVQYLTLFRQKL---MQLVVLTDVVERLHKNFASDH-----------
ASPNI	GYPVVVALLESLQRLECVLSVYEILRRKK---------------------
RHIO9	IDTCVDQFLNDWERI---FMMINLSRQVPSVWFAKYKDQ-----------
CRYNE	ISRCVPAFLEQWERL---SKVIVVAGEVSRLNKTESFKD-----------
USTMA	LDNCMSIFHTHWERV---MRMVQLTREVLNASRAWQQRA-----------
ENCCU	IEDLHEYFEASMDLLFFSYRFLPV--------------------------
ANTLO	ANILISFIDKKLDMLLGMADML----------------------------
ARATH*	-ADSIKKLVADIQRL---SNARMFSLGMRKLLGIKPDEKTEECSANSTMK
VITVI	-ADSIKKLVADIQRL---SNARMFALGMRKLLGVRMDEKPEEISANCDGK
SOYBN	-VDSIKKLVADIQRL---ANARTFALGMRKLLGVRAEEKSEELVTSSDTK
SOLLC	-ADSIKKLVADIQRL---SNARTFALGMRKLLGARADEKFEENNANSESK
ORYSJ*	-PDSVQKLVSDLRRL---ANARSFARGMRRLIGVKLNDKLDDDQTSTDIK
HORVU	--
SORBI	-ADSVQKLVSDLRRL---SNARSFARGMRRLIGVKLDDKLDDNQLSMEVK
SELML	-DDSIKKLLADLERL---RSARAFAHCMKKLLEDRMDEGDQGA-------
PHYPA	-DDSVTKLVKDLERI---WRARAFAVGIRKLLGSREEQKKRGSEQGGKSQ
VOLCA	RGESALTLVQEVSAI-----------------------------------

2010 2020 2030 2040 2050

Species	Sequence
HUMAN*	----------RSLPDIPAHLNIFSEVRVYNYRKLIL----CYGT---T--
CHICK	----------RSLPDIPNHLNIFSEVRIYNYRKLIL----CYGT---T--
XENTR	----------RSLQDIPAHLNMFCEVRVYNYRKLIL----CYGP---T--
DANRE	----------RSLTEMPSYLSLFSEIRLYNYRKLVL----CYGS---T--
CIOIN	----------QAQKQHTS----LPEVHSYNYKSITL----KYGA---D--
ANOGA	----------EQYNNEKYNLPSMVAIKSYSYTNLLL----AYGP---N--
TETNG	----------RALPDMPSYLSLFSEVRLYNYRKLVL----CYGA---T--
DROME	----------EQLQNKRLSLCDMLVVKSYSYMNLLL----GYGP---K--
BRUMA	-------------AAYYSHYNKYCNVVAFTYHKLVL----AYGS---N--
CAEEL	-------------LAYEKHFKNYCNIEAYTFHKLVV----SYGD---N--
YEAST*	-----------------------FKIIALQPNEISF----KYLSNNDE--
SCHPO	-----------------------FNVDCITLKHVDF----TYLD---D--
CANAL	-----------------------VTVLDNSMDNITINIKDKYND---N--
CANGA	-----------------------FKIISLKPNEVTF----KYLKSS-T--
ASPNI	-----------------------MDARFLSLSRLGF----AYGP---N--
RHIO9	-----------------------LIFDPTDLQELSF----TYAE------
CRYNE	-----------------------VKILSFDLRTATL----QYAP------
USTMA	----------LRLRRSCKKPGERVELCRFELDSVVF----SYGSIKVDGL
ENCCU	-----------------------IDGNITVGRKILL----SHRA------
ANTLO	----------------------------VSYRNIVF----CMRN------
ARATH*	GSTGGKG-SGEPVDRW-----RAFKIEAVGLTSLWF----SFGS---G--
VITVI	APVGVKG-V-EVSDKLSEQMRRAFRIEAVGLMSLWF----SFGS---G--
SOYBN	TPSTKVA-L-DTADKLTEQMRRAFRIEAVGLMSLWF----SFGS---G--
SOLLC	APAALKG-TTDATDRISEQMRKQFRIEAVGLMSLWF----SFGS---G--
ORYSJ*	SQPVNKG-NSDAADRLSEQMRKTFRIEAVGLMSLWF----SYGT---M--
HORVU	--
SORBI	SQSVNKG-NSDAADKLSEQMRKTFRIEAVGLMSLWF----SYGT---M--
SELML	----------SRTDTKWEAITRAFKVEALGLTSLWF----SYTGSMPG--
PHYPA	GRGGSVGDTGEAEEKKWEIMRRAFRVEAVGLTSVSF----TYVGTTPG--
VOLCA	--

2060 2070 2080 2090 2100

MED14

```
HUMAN*  -KGSSISIQWNSIHQK--------------FHISLGTVGPNSGCS-----
CHICK   -KGSSISIQWNSILQK--------------FHISLGTVGPNSGCS-----
XENTR   -KGSSISIQWNSVHRK--------------FHISLGTVGPNSGCS-----
DANRE   -KGSSVTIQWNSVTHR--------------YHLALGTVGPNSGCS-----
CIOIN   -SQFFVTVSWSSQEKK--------------FKLSFGCCGHNSTS------
ANOGA   -KDVSVNICWDTEAKE--------------FRLVF--TGGNSAI------
TETNG   -KGSSVTIQWNSSSQR--------------FHLALGTVGPNSGCS-----
DROME   -KEVSCNIYWSVQSHG--------------FRLTF--VGGMSAV------
BRUMA   -RNMLMMLSWRTQKAP---------NNKQYFYLTFG-IGPSRATTTADLS
CAEEL   -RDMLMILAFNVKSQAPG-------SSEDFFFLNFGQSMPHRQFNSTEID
YEAST*  -DDKDCTIKISTNDDSIK-------N-----------------------L
SCHPO   -EKFRATIHDDNT-----------------FTLHF---------------
CANAL   -NDKLIIIKLPEQAT----------------------------------D
CANGA   -DDKDCTINIITNEQTIK-------N-----------------------L
ASPNI   -KELLAQLDIGETQPQLP-------TEMDPLKLAF-----RTGHLFHYRL
RHIO9   --HYTCTINWSSSAKG----------RSRRYDIEFGIVDKNKST------
CRYNE   --GHNASITYSPTDDS--------------YRITFFLSSPSSDT------
USTMA   EAKLLVRVRWQDAKLEMQAFSPIGVTQSGGYILEFGSMRSVQVEALDQDA
ENCCU   -KGDQIDIRIERRDDGFV-------VETSRFGTRTAGVEGIYGCVAFALL
ANTLO   ------TLDIWLGDERFE-------VTRDRDSFLINGVYTMHNP-DNICK
ARATH*  -VLARFVVEWESGKDGCT------------MHVSPDQLWPHTKF------
VITVI   -VLARFVVEWESGKEGCT------------MHVSPDQLWPHTKF------
SOYBN   -VLARFVVEWESGKEGCT------------MHVSPDQLWPHTKF------
SOLLC   -VLARFVVEWESGKEGCT------------MHVSPDQLWPHTKF------
ORYSJ*  -PMVHFVVEWESAKGGCT------------MHVSPDQLWPHTKF------
HORVU   --------------------------------------------------
SORBI   -PMVHIVVEWEIDKGGCT------------MHVSPDQLWPHTKF------
SELML   -ILARFVVEWASDQEGCI------------VHVSPEQLWPHTKY------
PHYPA   -IMARFVVEWGTSRRGCT------------MH-SPEQLWPHTKF------
VOLCA   --------------------------------------------------
                  2110      2120      2130      2140      2150
```

```
HUMAN*  ------------------NCHNTILHQL----------------------
CHICK   ------------------NCHNTILHQL----------------------
XENTR   ------------------NCHNIILHQL----------------------
DANRE   ------------------NCHNIILHQL----------------------
CIOIN   ------------------NGHCITQHYI----------------------
ANOGA   ------------------NAHSMMRDQL----------------------
TETNG   ------------------NCHNIILHQL----------------------
DROME   ------------------NGHSMMRDQL----------------------
BRUMA   NSEI------------LWNPHILLASRL----------------------
CAEEL   WHQKP-----------RWNPHSMMSQLM----------------------
YEAST*  TVQLS-----------PSNPQHIIQPFL----------------------
SCHPO   ---FN-----------RHSPFHLISQFL----------------------
CANAL   SIQLL-----------MKNGSTTTTDEIDLRNNNILEFELILKFLNQYLR
CANGA   TVKLS-----------DKNPQHIIQPFI----------------------
ASPNI   SISFD-----------HSNPHRRIQGPL----------------------
RHIO9   --LLV-----------SSNPHWKITTFL----------------------
CRYNE   ----------------DRNPHELVAPLL----------------------
USTMA   QLREDGVSRWFDETHCLANPHKVMALEL----------------------
ENCCU   LMDVF-----------TASRDSEVLSII----------------------
ANTLO   FFEFN-----------LINRQLKAVQDM----------------------
ARATH*  --------------------------------------------------
VITVI   --------------------------------------------------
SOYBN   --------------------------------------------------
SOLLC   --------------------------------------------------
ORYSJ*  --------------------------------------------------
HORVU   --------------------------------------------------
SORBI   --------------------------------------------------
SELML   --------------------------------------------------
PHYPA   --------------------------------------------------
VOLCA   --------------------------------------------------
                  2160      2170      2180      2190      2200
```

MED14

```
HUMAN*   -QEMFNK---TPNVVQLLQ-------------VLFDTQAPLNAIN-----
CHICK    -QEMFNK---TPNVVLLLQ-------------VLFDTQAPLNAIN-----
XENTR    -QEMFNK---VPNVVQLLQ-------------VLYDTQAPLTSIN-----
DANRE    -QEMFNK---TPNVVQLLQ-------------VLSDTQAPLNAIN-----
CIOIN    -SEGFNS---DPDLAKLLY-------------VLKDTSSPLQSLT-----
ANOGA    -QAHLNH---NYSLAQVVH-------------MLHETYQPLSSIA-----
TETNG    -QELFNK---SPNVMQLLQ-------------VLYDTQAPLNAIN-----
DROME    -AQHLNQ---QHSITQIAQ-------------ILHETYNPLSSIA-----
BRUMA    -QERFNK---QRSLIGLVQ-------------YLIDSVAPLSALH-----
CAEEL    -RDDLKE---TNDLVFTMH-------------FLCETIRPLVAIG-----
YEAST*   -DNS------KMDYHFIFS-------------YLQFTSSLFKALKVILNE
SCHPO    -QDTFSD---GPSAIQPLR-------------VIMDRTRGVLVAQ-----
CANAL    ESQSNNHNRQQISIIKIIQ-------------YLTEINPILQSTKAINQQ
CANGA    -DNQ------QFEYQFIFS-------------YLQFTSPLFCCLKEILDE
ASPNI    -ASNLNRPTAEAGLDTLTE-------------ILSFTLPLMQALD-----
RHIO9    -RDILNE---KRDLIYFVQ-------------VKYIYTPLFYFILC----
CRYNE    -SARLNE---LTSTGIAGENKPKGQVGREFMGLLKNTLPVLKIGK-----
USTMA    -RRTVNVAARSAAMHAMLQPQAERIVWRGFLELLQHTLPLVREMA-----
ENCCU    -SIDLSKGV-SLRLSGFFI-------------TLQLNEEDALEAS-----
ANTLO    -EFSVEGGCHHYRLKDIRF-------------SLVFADKIVLETE-----
ARATH*   -LEDFIN---GAEVESLLD-------------CIRLTAGPLHALAAATRP
VITVI    -LEDFIN---GAEVASLLD-------------CIRLTAGPLHALAAATRP
SOYBN    -LEDFIN---GGEVSPLLD-------------CIRLTAGPLHALAAATRP
SOLLC    -LEDFIN---GAEVASLLD-------------CIRLTAGPLHALAAATRP
ORYSJ*   -LEDFVN---GGEVASFLD-------------CIRLTAGPLLALGGAIRP
HORVU    --------------------------------------------------
SORBI    -LEDFVN---GGEVASFLD-------------CIRLTAGPLLALGGAIRP
SELML    -LEDLIN---GGDVELLLD-------------AIRITAGPLHALAGAIRP
PHYPA    -LEDYIN---GGEVELLLD-------------AIQVTAGPLHALAAAIRP
VOLCA    -----------IRMQTLLA-------------RLEMTTRPVATTSAAGRG
                  2210      2220      2230      2240      2250
```

```
HUMAN*   --------------------------------------------------
CHICK    --------------------------------------------------
XENTR    --------------------------------------------------
DANRE    --------------------------------------------------
CIOIN    --------------------------------------------------
ANOGA    --------------------------------------------------
TETNG    --------------------------------------------------
DROME    --------------------------------------------------
BRUMA    --------------------------------------------------
CAEEL    --------------------------------------------------
YEAST*   -------RGGKFH-------------------------------------
SCHPO    --------------------------------------------------
CANAL    LAELKRINSTNNNNNNAKRRSILKLSNGL---------------------
CANGA    SNKEERLENAKEE-------------------------------------
ASPNI    --------------------------------------------------
RHIO9    --------------------------------------------------
CRYNE    --------------------------------------------------
USTMA    --------------------------------------------------
ENCCU    --------------------------------------------------
ANTLO    --------------------------------------------------
ARATH*   ARASTATGMPVVPATASSRQSNQIQQTQG-IIAPSTLAAPNATGQ--SAS
VITVI    ARAGPAAGVPGVTAANSS-----IPKQSG-YIPSQGLLPSSSTTN--VSQ
SOYBN    ARAGP---VPGVAAALSS-----IPKQNGSYISSHGLLLSNSTTN--VGL
SOLLC    ARAAPVSGVPGVTAPISS-----VAKQTG-YVPS---LPSNVNSS--INQ
ORYSJ*   ARMPV-----TVSSGYNS-----MPKQMN-NIPTQGPLANGSSST--TMH
HORVU    --------------------------------------------------
SORBI    ARMPV-----TVSSGYSS-----MPKQP--NILTQGPLANGSSSS--TIH
SELML    ARMTAPVSSIVASAISPP--------------------------------
PHYPA    ARMAAPASGSSHRPSSPSPGTGMAARAGA--SPAGSGMSSYGSGGAGQGQ
VOLCA    --------------------------------------------------
                  2260      2270      2280      2290      2300
```

MED14

```
HUMAN*  ---------------------KLPTVPMLGLTQRTNTAYQC---------
CHICK   ---------------------KLPTVPMLGLTQRTNTAYQC---------
XENTR   ---------------------KLPTVPMLGLTQRINTAYQC---------
DANRE   ---------------------KLPTVPMLGLTQRTNTAYQC---------
CIOIN   ---------------------RLPTTPLLGVNQKITIGEQM---------
ANOGA   ---------------------KLPIIPHLAILQSPKIPVLS---------
TETNG   ---------------------KLPTVPMLGLTQRTNTAYQC---------
DROME   ---------------------KLPVLPFLGI-PRPQVPVLS---------
BRUMA   ---------------------SFTRIRL----QSLRAFCQMMCYDAALAL
CAEEL   ---------------------NFSRIRF----QSQKSLSQLIGPDVHFPF
YEAST*  --------------------------------------------ESGSQY
SCHPO   --------------------------------------------------
CANAL   -------------------------------------------------Y
CANGA   --------------------------------------------SDSTRT
ASPNI   ---------------------RFMANP-------------------SRNE
RHIO9   --------------------------------------------------
CRYNE   ----------------------------------------------ILET
USTMA   -----------------------------------------PLVDKCLTH
ENCCU   ---------------------PLAVRHALGRCPLLGVMDLLCCFRAFFPS
ANTLO   ------------------------NSVFSRMVPKGSFVNAIFGFYYIYIY
ARATH*  ATSGNTVASSAPSPLGG----GFHGVAML----AAAGRS-GPGIVPSSLL
VITVI   ATSGPGVTPPASAASGPLGNHSLHGAAML----AAAGRG-GPGIVPSSLL
SOYBN   PTSGPGANTVMPTASGL----TSQTLSML----AASGRG-GPGIVPSSLL
SOLLC   PAPGAGVNP-VSASVGTLGAHSHPSAAMLAAAAAAAGRG-GPGIVPSSLL
ORYSJ*  HAPSP-----ANVAATHLGSHNLHTAAML----SAAGRG-GPGLVPSSLL
HORVU   --------------------------------------------------
SORBI   HAPVP-----QNAAAAQLGSHNLHAAAML----SAAGRG-GPGLVPSSLL
SELML   --GQVSPPNSSATPQSRATGLSIQIPTIT----SGPGRGPGPGIVPSSLL
PHYPA   GGQGAGANGSVNYGMNSNGGPGTPQGGMP----SNSGRNPGPGLVPSSLL
VOLCA   ---------------------GVRVQRLAVLTPPTAATDPAAVDADGQGR
                2310      2320      2330      2340      2350
```

```
HUMAN*  ---FSILPQSSTHIRLA--FR-------N-------MYCIDIYCRS----
CHICK   ---FSILPQSPTHIRLA--FR-------N-------MYCIDIYCRS----
XENTR   ---FSILPQSPTHIRLA--FR-------N-------MYCIDIYCRS----
DANRE   ---FSILPQSPTHIRLA--FR-------N-------MYCIDIYCRS----
CIOIN   ---FTLTAQTATVVRVT--FF-------S-------SYSINIYLHS----
ANOGA   ---FCIIPQSPTLLRIS--FQ-------G-------VYCLEVRFRG----
TETNG   ---FSILPQSATHIRLA--FR-------N-------MYCIDIYCRS----
DROME   ---FCVLAQSPCLIRLT--YQ-------A-------VYCLELRFRA----
BRUMA   SLQVTLSIVDEYRIRLI--YG---------------HVHLEFFLLG----
CAEEL   RLKYHLYALDQTTLRLM-----------------QGNVILEIKLLE----
YEAST*  STMVNIGLHNLNEYQIV--YY-------NPQAGTKITICIELKTVLHNGR
SCHPO   ELGYVVLARSLRQYRII--LS-------K-------NHGIQVLLNR----
CANAL   KLYFNLNIISLTHLQLV--FFMNSNTGSNLKKIQRDKIMINLSLIKFDRF
CANGA   STSITLGLHNLSEYQLV--YF-------NPELGYKITLILELKEVSINGK
ASPNI   SSIVHVTVRNATSFQIHYPYE-------K----------CRLRLVAHQHQ
RHIO9   -------------------LNFLNSYAEKKQKKNKICYSMHIFKLC----
CRYNE   KMGWGVVVLGVAKYRLIKDHN-------------GKRFSVDVTLLP----
USTMA   AHVPEVEIKSATWFRFR--FQ-------D-------RYAIDVRLAT----
ENCCU   GLVPTTSTPTSLIFA----FKHLFKGSVKVRLIDRTRYRLSLRGGR----
ANTLO   SIHPKFICQDYLIFD----FRMTLGDHVVLKTENDTTFLVKKTYFA----
ARATH*  PIDVSVVLRGPYWIRII--YR-------K-------RFAVDMRCFA----
VITVI   PIDVSVVLRGPYWIRII--YR-------K-------YFAVDMRCFA----
SOYBN   PIDVSVVLRGPYWIRIM--YR-------K-------QFAVDMRCFA----
SOLLC   PIDVSVVLRGPYWIRII--YR-------K-------KFAVDMRCFA----
ORYSJ*  PFDVSVVLRGPYWIRII--YR-------K-------KFSVDMRCFA----
HORVU   --------------------------------------------------
SORBI   PFDVSVVLRGPYWIRII--YR-------K-------KFSVDMRCFA----
SELML   PTDISVLMRSPYWIRIV--YR-------K-------QFSVDMRCFE----
PHYPA   PTDVSVLLRSAYWIRIV--YR-------R-------DFAIDMRCFA----
VOLCA   PLQPKTPLHSQQLQQPI---------------------------------
                2360      2370      2380      2390      2400
```

MED14

```
HUMAN*  -------R----GVVAIRDGAYSLFDNSKLVEGFYPAPGLKTFLNMFVDS
CHICK   -------R----GVVAIRDGAYSLFDNSKIVEGFYPAPGLKTFLNMFVDS
XENTR   -------R----GVVAIRDGAYSLFDNSKIVEGFYPAPGLKTFLNMFVDS
DANRE   -------R----GVVAIRDGAYSLFDNTKIVEGFYPAPGLKTFLNMFVDS
CIOIN   -------H----GKVSIQDGAFSQFDYSKVKNGSIPIPGLCSFLSRFQEQ
ANOGA   -------G----GLCTIRDGAYSRFDRSHVVEEFTPTQGLKGFLSKYVDE
TETNG   -------R----GVVAIRDGAYSLFDNTKIVEGFYPAPGLKTFLNMFVDS
DROME   -------N----RLVSIRDGASSRFER-NVVEEFTPIQGLKAFLSKYVDE
BRUMA   -------N----SRIGLRD----------CSLGSACAFRLRQFCSNFLSP
CAEEL   -------G----CKIAVRD----------VSRYRPRCAGLFQLFSNIDSE
YEAST*  DKIQFHIHFADVAHITTKSPAYPMMH------------------------
SCHPO   -------H----GCI-LQDLSYLSADSRYLEGTQTLTSQWEP--------
CANAL   SKPNGNFQDSTNGHLIKISFKDSLIN------ENLKFKNLFEIIFKNINE
CANGA   KKIQFYVHFSQDEHITTKSSAYPLIH------------------------
ASPNI   NQPVWVLR----DVLSFQDGSCEPEF------------------------
RHIO9   --------------------------------------------------
CRYNE   ----------DLEHYLIQDGATPRGPDHVVDVYTGPLTSL----------
USTMA   -------R----SRLVVSDASRALFA----------------------SE
ENCCU   --------------------TRDIFGGFGTEILSSDTVFPRKLAGFYFKE
ANTLO   ----------------TSSLQLPCFNYFYSECSATNVDFFVHLRSTYLKE
ARATH*  -------G----DQVWLQP-ATPPKG-GASIGGSLPCPQFRPFIMEHVAQ
VITVI   -------G----DQVWLQP-ATPPKG-GPSVGGSLPCPQFRPFIMEHVAQ
SOYBN   -------G----DQVWLQP-ATPPKE-GRLSGGSLPCPQFRPFIMEHVAQ
SOLLC   -------G----DQVWLQP-ATPPKG-GPEVGGSLPCPQFRPFIMEHVAQ
ORYSJ*  -------G----DQVWLQP-ATPPKG-GPLVGGSLPCPQFRPFIMEHVAQ
HORVU   --------------------------------------------------
SORBI   -------G----DQVWLQP-ATPPKG-GPSVGGSLPCPQFRPFIMEHVAQ
SELML   -------G----DQVWLQP-APPPQGAGPDAGGSLPCPQFRPFVMENVLN
PHYPA   -------G----DQVWLQP-APPPRSGGKGGGGSLPCPQFRTFVVEHVTL
VOLCA   -------G----GTCSFRPGSVDAAGPGEYGAGAAASSSSYHPLQPAAKR
                 2410      2420      2430      2440      2450
```

```
HUMAN*  NQDARRRSVNEDDNPPSPIGGDMMD-----------------------SL
CHICK   NQDARRRSVNEDDNPPSPIGGDMMD-----------------------SL
XENTR   NQDARRRSVNEDDNPPSPIGGDMMD-----------------------SL
DANRE   NQDARRRSVNEDDNPPSPI--SMED-----------------------TF
CIOIN   NEV-RYKSDTEKDNPPSPD-------------------------------
ANOGA   TAVFRRRSQSEDDNPPSPV--TLEDPSAGAGNNGGGGGGGGGAGGGANTF
TETNG   NQDARRRSVNEDDNPPSPV--DVID-----------------------SL
DROME   SAVYRGRASHEDDNPLSPM--GMED------NFGGPSSVAGVSAGGSSPF
BRUMA   ----ELRSANEEKSPSAFA-------------------------------
CAEEL   TTAIMNDEIAIPQS------------------------------------
YEAST*  --------------------------------------------------
SCHPO   --------------------------------------------------
CANAL   LLITKSKQIKSLNQ------------------------------------
CANGA   --------------------------------------------------
ASPNI   --------------------------------------------------
RHIO9   --------------------------------------------------
CRYNE   --------------------------------------------------
USTMA   VRTSESESESETTD------------------------------------
ENCCU   RIYLIADAARKSYD------------------------------------
ANTLO   RFLVLGRSLKKE--------------------------------------
ARATH*  ELNGLEPNLTGSQ---GATNPNSGN-------------------------
VITVI   ELNGLEPNFAGGQQTIGLANSNNPN-------------------------
SOYBN   ELNGLDPSFTG-QQAGGMANSNNPN-------------------------
SOLLC   ELNGIDSNFTGSQQAVGVPNSNSLN-------------------------
ORYSJ*  GLNALEPSFMNATQAGAHLNSS----------------------------
HORVU   --------------------------------------------------
SORBI   GLNALEPNFMNATQASGHLNNN----------------------------
SELML   SSETVVTGVQQNGPASANPRSN----------------------------
PHYPA   GMSSSVDSVGGGAIGSSSPS------------------------------
VOLCA   IKTEPGAAAPAPGADLAYGDGRWSS-------------------------
                 2460      2470      2480      2490      2500
```

MED14

Species	Sequence (2501–2550)
HUMAN*	ISQLQP--PPQQ--QPFPKQPGTSGAY-PLTSPPTSY-HSTVNQSPSMMH
CHICK	ISQLQPQQPPQQPQQPFAKQAGASGAY-PLTSPPTSY-HNTVTPSPSMMH
XENTR	LSQLQP--QGQQT-QQFTKQMGASGAY-PLTSPPSSY-HNTVTPSPSMMH
DANRE	MPHTQT-----QP----PGR----GVYPPLTSPPNPY-H--VPPSPSMIT
CIOIN	--------------------------------------QAALNSSQNM--
ANOGA	LSGGTGMRGPQSP-RD----PGLRFAA-PLTPPTSSNPHTPASPHPIGGG
TETNG	MNQLQA---PQQP-QPQPMRAAAGGVYPPLTSPPPNY-HANVTPSPSMMP
DROME	L--GTGMRGPQSP-RD----SGLRFPA-PHTPPSSSNPHTPASPHPSAGA
BRUMA	--
CAEEL	--
YEAST*	--
SCHPO	--
CANAL	--
CANGA	--
ASPNI	--
RHIO9	--
CRYNE	--
USTMA	--
ENCCU	--
ANTLO	--
ARATH*	---PTV--
VITVI	--------------------------------------PSSGSQLSAA--
SOYBN	--------------------------------------PGSGSQMMAA--
SOLLC	--AGSQLPAA--
ORYSJ*	--AGTLQPAP--
HORVU	--
SORBI	--AGAPQTAP--
SELML	--
PHYPA	--
VOLCA	----------------------------WQQQQPQPQKQLQQQQQPGLAG
	2510 2520 2530 2540 2550

Species	Sequence (2551–2600)
HUMAN*	TQSPG-------------------NLHAASSPSGALRAPSP-APFVPT--
CHICK	TQSPG-------------------NLHAASSPSGALRAPSP-ASFGPT--
XENTR	SQSPG-------------------NLQ--SSPSGVLRAPSP-ASFGPT--
DANRE	TQSPG-------------------TL------------------------
CIOIN	--YGLVDVTP
ANOGA	GGAGGAGGQGGQGGQGGQQQSHMNNFNMTSPPASHMPHPSPGGGLMPSSP
TETNG	TQSPG-------------------NIHTSGSPSGVLRAPSP---FGPT--
DROME	GGGSGPQGHG--------------NFNLTSPPAPHMPHPSP-SGLMPSSP
BRUMA	--
CAEEL	--
YEAST*	--
SCHPO	--
CANAL	--
CANGA	--
ASPNI	--
RHIO9	--
CRYNE	--
USTMA	--
ENCCU	--
ANTLO	--
ARATH*	--
VITVI	--
SOYBN	--
SOLLC	--
ORYSJ*	--
HORVU	--
SORBI	--
SELML	--
PHYPA	--
VOLCA	YVPPGAGLLPANGVMDGHAMGVGQVPGRPGSCAPGAASSGGANGGGGSAS
	2560 2570 2580 2590 2600

MED14

```
HUMAN*  --PPPSSHGI-SIGPGAS-FASPHGT-LDPSSPYTMVSPSGRAGNWPGSP
CHICK   --PSPSSLGI-TMGQTAN-FASPHGT-IDPSSPYTMMSPSQRAGNWPGSP
XENTR   --PSPSSLGI-SMVPTSN-FASPHGA-LDPSSPYTMMSPSQRAGNWPGSP
DANRE   ----------------------------DPSSPYTLVSASPRVGPWPGSP
CIOIN   --------------------------------------------------
ANOGA   LNAQPSPMAAHSPGPSSLSYMQSHTD----GSPFAALSPAA--SNWPGSP
TETNG   --PSPSSLGI-AMGQTS--FASPHGTGVDPSSPYAMVSPSHR-GQWPGSP
DROME   LNPQPSPHMVHSPGPNTL-YMQSHQD-----SPFTAMSPAN--NNWPGSP
BRUMA   -----------------------------------WTATSSGPGSLPSHQ
CAEEL   -----------------------------------DNPQTAGPTMWTPEQ
YEAST*  --------------------------------------------------
SCHPO   --------------------------------------------------
CANAL   --------------------------------------------------
CANGA   --------------------------------------------------
ASPNI   --------------------------------------------------
RHIO9   --------------------------------------------------
CRYNE   --------------------------------------------------
USTMA   --------------------------------------------------
ENCCU   --------------------------------------------------
ANTLO   --------------------------------------------------
ARATH*  --------------------------------------NGVNRVNFSPSS
VITVI   --------------------------------------NG-NRVGLPNSA
SOYBN   --------------------------------------NG-NRINLPISA
SOLLC   --------------------------------------NT-NRTNLSNST
ORYSJ*  --------------------------------------NA-NRVNATQGI
HORVU   --------------------------------------------------
SORBI   --------------------------------------SA-NRLSATPGV
SELML   --------------------------------------------------
PHYPA   --------------------------------------TGANSGARQAGG
VOLCA   GVTGTASSGLTWHWPLVGTLTLEDYSCSAATLRVTPCTTGPRQGSAPAAA
                          2610      2620      2630      2640      2650
```

```
HUMAN*  QVSGPSPAARMPGMSPANPSLHSPVPDASHSPRAGTSSQTMPTNMPPPRK
CHICK   QVSGPSPAARMPGMSPANPSLHSPIPDASHSPRAGTSSQAMPTSMPPPRK
XENTR   QVSGPSPAARMPGMSPANPSLHSPVPDASHSPRAGTSSQSMPTNMPPPRK
DANRE   QVSGPSPGARIPGMSPGNPSLHSPIPDPSHSPRAGTSSQ----AMPPPRK
CIOIN   --------------------------------------------------
ANOGA   GMPRPSP---RPGQSPE------------HK--------VQTSHHYTSRV
TETNG   QFSGPSPGARIHSMSPGNPSLHSPIPDP-HSPRAGTSSQIMPTSMPPPRK
DROME   SMPRPSP---RPGQSPD------------HKSTGGGAGVAGGTDRGGSRG
BRUMA   MVRSPASRIVSSPMSHM------------------------------SLS
CAEEL   FMDSLDER---------------------------------------PEE
YEAST*  --------------------------------------------------
SCHPO   --------------------------------------------------
CANAL   --------------------------------------------------
CANGA   --------------------------------------------------
ASPNI   --------------------------------------------------
RHIO9   --------------------------------------------------
CRYNE   --------------------------------------------------
USTMA   --------------------------------------------------
ENCCU   --------------------------------------------------
ANTLO   --------------------------------------------------
ARATH*  AR------------AAM------------NRVASVASGS---LVVSSGLP
VITVI   GISRPGNQA-----TGM------------NRVGSALSASQNLAMVNSGLP
SOYBN   AMPRTGNQV-----ASL------------NRVGNALAGSSNLALMTSAVS
SOLLC   GLARPANAV-----TGF------------NRTANGLPAASNLAGVNAGMP
ORYSJ*  GMSRPASGVANHVAANL------------SRAGNAMLASSGLASGIGGAS
HORVU   --------------------------------------------------
SORBI   SMSRPTSGVANHVAASL------------SRAGNAMLASSALASGISGAS
SELML   -----------------------------------------------ASA
PHYPA   GVGRAGNSVGSTIAAMQ----------------------GGSGLGGGSRS
VOLCA   DAGHNAVMPPPPLLLHMTWAPRDVRDPYGGTGGAGGGTAAAAGGGATPGT
                          2660      2670      2680      2690      2700
```

MED14

```
HUMAN*   LPQRSWAASI-----------PTILTHSALNILLLPSPTPG---LVPGLA
CHICK    LPQRSWAASI-----------PTILTHSALNVLLLPSPTPG---LVPGLA
XENTR    LPQRSWAASI-----------PTILTHSALHVLLLPSPTPG---LAPALA
DANRE    LPQRSWAASI-----------PTILTHSKLHVLLLPSPMPC---PVPGLA
CIOIN    ---ASWSGSQ-----------PTVLSHAGLHSLCLKVPN----------V
ANOGA    LPARSWAGAI-----------PTTLTYEALDTLCRATPHPQKEVPGPEL-
TETNG    LPQRPWAASI-----------PTILTHNALHVLLLPSPTPC---LVPGLA
DROME    TLNRPWAGAV-----------PTLLTHEALETLCRPSPYPNKDINVPDM-
BRUMA    VPPLDSFGSSLRYSSDCRSRTPILLDHDSLHCMTSVDESGS---------
CAEEL    IDPRMAITSQ-----------PILMSHDTIIKACDFKDTEG---------
YEAST*   --------------------------------------------------
SCHPO    --------------------------------------------------
CANAL    --------------------------------------------------
CANGA    --------------------------------------------------
ASPNI    --------------------------------------------------
RHIO9    --------------------------------------------------
CRYNE    --------------------------------------------------
USTMA    --------------------------------------------------
ENCCU    --------------------------------------------------
ANTLO    --------------------------------------------------
ARATH*   VRRTPGTAVP-----------AHVRGELNTAIIGLGDDGGYGGGWVPLVA
VITVI    LRRSPGAGVP-----------AHVRGELNTAIIGLGDDGGYGGGWVPLVA
SOYBN    LRRPPGTVVP-----------AHVRGELNTAIIGLGDDGGYGGGWVPLVA
SOLLC    LRRAPGTGVP-----------AHVRGELNTAIIGLGDDGGYGGGWVPLVA
ORYSJ*   VRLTSGANLP-----------VHVKGELNTAFIGLGDDGGYGGGWVPLAA
HORVU    --------------------------------------------------
SORBI    VRLTAGAGLP-----------VHMKGELNTAFIGLGDDGGYGGGWVPLAA
SELML    TRNTPSSAASGRNLVIGGPGYPSYRGELNSAFCGVSDDGGYSGAWVPLPA
PHYPA    LATGSGVGGA-----------ISIRAELNPALVGMGDDGGYGGAWVPLAA
VOLCA    STPAPTPGGIIALPPSQPSPSPAILGGVAASPLTAATP--------PQPY
                  2710      2720      2730      2740      2750
```

```
HUMAN*   GSYLCSPLERFLGSVIMRRHLQRIIQQE----------------TLQLIN
CHICK    GSYLCSPLERFLGSVIMRRHLQRIIQQE----------------TLQLIN
XENTR    GSYLCSPLERFLGSVNMRRHLQRIIQQE----------------TLQLIN
DANRE    GSYLCSPLERFLGSVIMRRHLQRIIQLEP---------------NLTIVN
CIOIN    HHTSATQLEMFFAAAIVKRHLVKCVRAIEVG----------PGVQAMIEG
ANOGA    -----SPLERFLGSVFMRRQLQRIIHQEE---------------SLMAIT
TETNG    GSYLCSPLERFLGSVIMRRHLQRIIQQEA---------------NLSIVN
DROME    -----SPLERFLGCVYMRRQLHRNIQSEE---------------TLTALN
BRUMA    -----CALDQYLFALAYLSKLGPALEGYRRE---HRGSGAHPVIQFRQLI
CAEEL    --RITCPLDEYLCSISYLQRALLTLERMSPRATLNKNSSSNLSCGFVTII
YEAST*   --------------------------------------------------
SCHPO    --------CSWLNTVW----------------------------------
CANAL    --------------------------------------------------
CANGA    --------------------------------------------------
ASPNI    --------------------------------------------------
RHIO9    --------------------------------------------------
CRYNE    --------------------------------------------------
USTMA    --------------------------------------------------
ENCCU    --------------------------------------------------
ANTLO    --------------------------------------------------
ARATH*   LKKVLRGILKYLGVLWLFAQLPDLLREILGSILKDNEG------ALLNLD
VITVI    LKKVLRGILKYLGVLWLFAQLPDLLKEILGSILKDNEG------ALLNLD
SOYBN    LKKVLRGILKYLGVLWLFAQLPELLKEILGSILKENEG------ALLNLD
SOLLC    LKKVLRGILKYLGVLWLFAQLPDLLKEILGSILKDNEG------ALLNLD
ORYSJ*   LKKVLRGILKYLGVLWLFAQLPELLKEILGSILKENEG------ALLNLD
HORVU    --------------------------------------------------
SORBI    LKKVLRGILKYLGVLWLFAQLPELLKEILGSILKDNEG------ALLNLD
SELML    LKKVLRSTLKYLGVLWLFAQFPNIIREVLGSVMNGRDG------TLLHLD
PHYPA    LKKVLRGTLRYLGGLWLFAQFPGILREVLASTLNQNEG------ALLNHD
VOLCA    PSGLLPPVDA----------------------------------GVRTTR
                  2760      2770      2780      2790      2800
```

MED14

HUMAN*	SNEPGVIMFKTDA-----------LKCRVALSPK-TNQTLQLKVTPENAG
CHICK	SNEPGVIMFKTEA-----------LKCRVALNPK-TNQTLQLKVTPENTG
XENTR	SNEPGVIMFKTDV-----------LKCRVALNPT-TNQTLQLKVTPENAG
DANRE	SNEPGVIMFKTEV-----------LKCRVALNPK-SYQTLQLKVTPENTG
CIOIN	ADDSLSAVFIARS-----------LQFTCQPHPQ-HLR-LVLSVQRAQQE
ANOGA	SNEPGVVVFKADC-----------LQYQVFLNPN-HMQSLHLKVDQLPMG
TETNG	SNEPGVIMFKTDM-----------LKCRVALNPK-NYQTLQLKVTPENTG
DROME	STEPGVVLFKVDG-----------LQCQVVLNQM-HMQTLHLKVSQLP--
BRUMA	ATAESVRLSVLGAAPPNLKHEPFLVNMNIYLDE--ETMSLKMKLDFGKKF
CAEEL	DAKPDFIRFRASQMNGDGVNATSMVHYKIYLCPVAMTLKIRIEFEEGTNS
YEAST*	--
SCHPO	--
CANAL	------------------------------------TENQQQQQPLKQED
CANGA	--
ASPNI	--------------------------------------KHKLQERLYNSK
RHIO9	--
CRYNE	-----------------------------------EMDKVVHAVFDAEKS
USTMA	-AVPLDAVFGGSQLLNDLLDATTLARKCTQFGPIPNIEHVLRSVHAKLDA
ENCCU	------------------------VTVSENEVGI-TTPCGHFKVWIENNR
ANTLO	---------------------------------V-CIEKKKFTIHISTKK
ARATH*	QEQPALRFFVGGY-----VFAVSVHRVQLLLQVL-SVRRFHHQA-----Q
VITVI	QEQPALRFFVGGY-----VFAVSVHRVQLLLQVL-SVKRFHHQQQP---Q
SOYBN	PEQPALRFFVGGY-----VFAVSVHRVQLLLQVL-SVKRFHQQQQ----Q
SOLLC	QEQPALRFFVGGY-----VFAVSVHRVQLLLQVI-SVKRFHQSQQ----Q
ORYSJ*	QEQPALRFYVGGY-----VFAVSVHRVQLLLQVL-SVKRFHHQQ-----Q
HORVU	--
SORBI	QEQPALRFYVGGY-----VFAVSVHRVQLLLQVL-SVKRFHHQQ-----Q
SELML	PEQPALRFNIGNC-----MFAVNMHRHQLYLQGL-NVKKFQQPP-----P
PHYPA	PEQPALRFFIRGC-----VFAVSMHRQQLFLQAI-NVNRYKQQQAAAAHQ
VOLCA	LGPPRSPGEPFGSQFCDLVLCTISCSSGHSLPPE-YLQVLEELAEAGEEG

2810 2820 2830 2840 2850

HUMAN*	-----------QWKPDELQVLEKFFETRVA--GPPFKANTLIAFTKLLGA
CHICK	-----------QWKSEELQVLEKFFETRVA--GPPFKANTLIAFTKLLGA
XENTR	-----------QWKPEELQVLERFFETRVA--GPPFKANTLIAFTKLLGA
DANRE	-----------PWSQEELQVLEKFFETRVA--GPPFKYNTLNAFTKLLGA
CIOIN	-----------NWSQDDLQTLEKYFDARIA--GSPYRYNVVASYVNMIFA
ANOGA	PMMDG-K-PPYQWAAQDLQILEQFFDHRVA--APPYRPAVMTSFTRMLNL
TETNG	-----------PWSQEELQVLEKFFETRVA--GPPFKYNTLNAFTKLLGA
DROME	PMPDK-HPPPFQLSQDDLLVIEQYFDTRVA--APPYRPNSLHSICRLLNL
BRUMA	RIFKFLDGEDIPA-DNDIRTAELYFVRFVARLGNEIAVVAFANACRL--A
CAEEL	-----------AATADNLKTLTTYFEKVVFPCGDEYALQSYICLTRL--T
YEAST*	------------------QVRNQVFMLD-TKRLGTPESVKPANASHAIRL
SCHPO	--------------EGDLGDDELNGQIEAAPEMHLIKMNKTADLTAILKR
CANAL	NSDSAIIKKESQSIEDDLLDFGEYDNDIKPQQQEPQQNEKENSKTT----
CANGA	------------------QVRNEIFMLEMKKRDGTPSLIKLDNTRNVIRL
ASPNI	GTGWRGLGNGVIAEPDHIGDLLDELDRCLASTKANTTPKALDSRTS----
RHIO9	---------------------LSMFMRIMMSKPPSFTIYPLPHLKSSCVT
CRYNE	VSLAETGDEKGQGKKRHV--------------PPVMKLDQGKSLVCEVAR
USTMA	ITWIDARDHRSSAARLRVYELGRALLISLPSASHPNAQAAYHAIAHLVPL
ENCCU	CMFRVCPEDTPEVSDEFAQTIGKYFGEAMATERNIHDSMKLLNSTDELVK
ANTLO	SIVLCLENDQLHIHID----------------GKPLYSNLMTRFVRIFCE
ARATH*	QNGSS-AAAQEELTQSEIGEICDYFSRRVA--SEPYDASRVASFITLLTL
VITVI	QQPNS-ATAQEELTQSEIGEICDYFSRRVA--SEPYDASRVASFITLLTL
SOYBN	QQQNS-NPAPEELSQSEISEICDYFSRRVA--SEPYDASRVASFITMLTL
SOLLC	QQQNP-GSAQEELTQSEIGEICDYFSRRVA--SEPYDASRVASFITLLTL
ORYSJ*	QQQAQ-NSAQEELAPPEINEICDYFSRRVA--SEPYDASRVASFITLLTL
HORVU	--
SORBI	QQQAQ-SNAQEELAAAEINEICDYFSRRVA--SEPYDASRVASFITLLTL
SELML	ELQTDPPGADADLTNGEMLEITEFFAQRVA--SEPYDASRLASFVTILTL
PHYPA	QVAPV-MNMAVELSLGEMQDIGDFFARRVA--SEPYDASRLASFVTMLTL
VOLCA	KLLD----------------------------AVAVAAWPVAGFCASLRP

2860 2870 2880 2890 2900

MED14

HUMAN*	PTHILRDCVHIMKLELFPDQA---T----QLKWNVQFCLTIP--------
CHICK	PTHILRDCVHIMKLELFPDQA---S----QLKWNVQFCLTIP--------
XENTR	PAHILRDCVHIMKLELFPDQT---T----QLKWNVQFCLTIP--------
DANRE	PTNILRDCVRIMKLELFPDQA---A----QLNWNVQFCLTIP--------
CIOIN	PPHILRDFIHLMRLELTGRNV---TAMQPNIKWNMSLCLTSP--------
ANOGA	PAKVLKDFIQIMRLDLMPELV---QG---N-KWNVQFVLRVP--------
TETNG	PTNILRDCVHIMKLELFPDQA---A----QLKWNVQFCLTIP--------
DROME	PAQVLKDFVQIMRLDLKPELG---GD---QLKWTVQICLRMP--------
BRUMA	APGAFSAITRIMSVQMNWTED-------TPWRPSIQLVMWSQ--------
CAEEL	SFEATQSIANLMNVQMEHPPT---------SKCCVQLSLTYN--------
YEAST*	GNGVACDPSEIEPILMEIHNILKVDSNSSSS-------------------
SCHPO	ILAISRKK--
CANAL	--
CANGA	GNGISCDSDSIVEVLMSINKILLGDSKSAPQITTVPPTNKTI--------
ASPNI	----------------HDAPAANNQSPAIRPEKSVEAADMHGGFVNRNLQ
RHIO9	KLRHYKNVLIFTFSFIFYNK------------------------------
CRYNE	FERVLRKVMEEASKVMGLGNNMEMDS------------------------
USTMA	LID----------------------------RVEAQLARSTP--------
ENCCU	LFKDIKSKEKIKYYNDQIH-------------------------------
ANTLO	EYCFIGPLVEMLGML-----------------------------------
ARATH*	PISVLREFLKLIAWKKGLSQSQQAGEIAPAQRPRIELCLENH--SGTDLD
VITVI	PISVLREFLKLIAWKKGLAQA-QGGDTAPAQKPRIELCLENH--AGLKMD
SOYBN	PVAVLREFLKLIAWKKGLSQA-QVGDVVSAQKPRIELCLENH--SGLNMD
SOLLC	PISVLREFLKLIAWKKGLSQV-QGGDMVPTQKSRIELCLENH--AGYSID
ORYSJ*	PISVLREFLKLIAWKKGFSQA--HGDMATAQRARIEICLENH--SGSVSD
HORVU	--
SORBI	PISVLREFLKLITWKKGLSPV--NGDIATAQRARIELCLENH--SGSAAA
SELML	PIAVLREFLGLISWMKEAQKN---QAVEATPRPRIELCLENRLVINTTMK
PHYPA	PVPVLREFLILIQWKANGGPGGDASGQGASPRPQVELSLESQ----GGDE
VOLCA	PVLSRYGLLPDMDVRLTMQQP--------------------PFCLRIVCR
	2910 · 2920 · 2930 · 2940 · 2950

HUMAN*	PSAPPIAPPGTPAVV-L-KSKMLFFLQLTQKTSVPP--------QEPVSI
CHICK	PSAPPIAPPGTPAVV-L-KSKMLFFLQLTQKTAV-S--------QEAVSI
XENTR	PSAPPIAPSGTPAVV-L-KSKMLFFLQLTQKTSA-P--------TDAFSI
DANRE	PSAPPIAPPGTIAVV-L-KTKMLFFLQLTQRLPV-P--------QEPISI
CIOIN	PRVPLLL--GQPSIL-I-KDKVLMFVQLTQAIQQ-T--------NAPGTI
ANOGA	PSATPIVPVGTTTIL-SHRQKILFFIQITRVPYL-PNM----EWKDAVTM
TETNG	PSAPPIAPPGTIAVV-L-KSKMLFFLQLTQRLPV-P--------QDPVSI
DROME	PSAVPIVPSGNACVV-MGRMKILFFLQITRIPYG-AVIGVGKDWKDSPSL
BRUMA	EGSVPRARISPGIVIDQTNISVLILLCLRPARHNPS-----EKNADASKH
CAEEL	--NTSTKKLAPATKVDQPLQNIIFNVIVSQSR------------TSESFS
YEAST*	--
SCHPO	--
CANAL	--SKENET
CANGA	--
ASPNI	QKAGPASQKTDVITID----------------------------------
RHIO9	--
CRYNE	--
USTMA	--
ENCCU	--
ANTLO	--
ARATH*	----NNCAAKSNIHYDRPHNTVDFALTVVLDPVHIP-----HINAAGGAA
VITVI	ESSENSSTSKSNIHYDRSHNSVDFGLTVVLDPAHIP-----HINAAGGAA
SOYBN	ENSESSSAFRSNIHYDRLHNSVDFALTVVLDSAHIP-----HVNAAGGAA
SOLLC	GSSENTSASKSNIHYDRAHNSVDFALTVVLDHAHIP-----HINAAGGAA
ORYSJ*	DITE-STLAKSNVKYDRAHSSLEFALTFVLDHALIP-----HMNVAGGAA
HORVU	--
SORBI	DNTENSTLAKSNIHHDRAHSSVEFALTFVLDHALIP-----HMNVAGGAA
SELML	EEAGSSTSVKSSILHDREREIVEFALTVFLDLGPHV-----NVNVAGGAG
PHYPA	SGVGGGSQGRSSITHNHHRNTVEFTLTFTFDASSLP-----QLNVAGGAG
VOLCA	KRGSGGGSSNNVLIIDLICLALGRSFLRQQQQQQQQQQQQGLGALEAAAG
	2960 · 2970 · 2980 · 2990 · 3000

MED14

```
HUMAN*  IVPIIYDMASGTTQQADIPRQQ------------NSSVAAPMM-VSNILK
CHICK   IVPIIYDMASGTTQQADIPRQQ------------NSSVAAPMM-VSNILK
XENTR   IVPIIYDMASGTTQQAEIPRQQ------------NSSVTAPLT-VSNILK
DANRE   IVPIVYDMATGLTQQADIPRQH------------SSSGAAALM-VSSILR
CIOIN   VVPFLHERQNNLVQVLPHAMSA------------QTQQTRNLQHVSSFLK
ANOGA   LLPMVYDMNMNHTTLAERREPM------------PPQLTSA---VSAHLR
TETNG   IVPIVYDMATGLTQQADIPRQH------------SSSGAAALM-VSNILK
DROME   VLPIVYDIQTNVTQLAERTGQV------------ISPTMTA---ASTLLR
BRUMA   LIPLIYNTQTNTVARRSSGADD----DASNATTVLSNISEQYSLTNEP--
CAEEL   VLRFIYRIKENFVVVPSANEKNKQMADEVNAETKTSGGNPIWNLVRLVMD
YEAST*  --------------------------------------------------
SCHPO   --------------------------------------------------
CANAL   VRPDDILIKLNYDYLLSMNNLQLMINEITKSCFQYLQQQQE---------
CANGA   --------------------------------------------------
ASPNI   --------------------------------------------------
RHIO9   --------------------------------------------------
CRYNE   --------------------------------------------------
USTMA   --------------------------------------------------
ENCCU   --------------------------------------------------
ANTLO   --------------------------------------------------
ARATH*  WLPYCVSVRLRYTFGEN-PSVTFLGMEGSHGGRACWQRVDDWEKCKQRVS
VITVI   WLPYCVSVRLRYSFGEN-STVSFLGMEGSHGGRACWLRIDDWEKCKHRVV
SOYBN   WLPYCVSVRLRYSFGES-PNVSFIGMNGSHGGRACWLRVDDWEKCKQRVA
SOLLC   WLPYCVSVRLRYAFGEN-PNVLFLGMEGSHGGRACWLRVDDWERCKQRVA
ORYSJ*  WLPYCVSVRLKYSFGES-NHIAYLAMDGSHGGRACWLQYEDWERCKQKVA
HORVU   --------------------------------------------------
SORBI   WLPYCVSVKLRYSFGDN-NHIAFLAMNGSHGGRACWLQFEEWERCKQKVS
SELML   WLPQCVSVRMRYYYNEGGRRVLLVGLEGSHGGRACWSRGDEWERLKERTK
PHYPA   WLPQSVALRLAYAYGEGVNRISLSSMDGSHGGRSCWARAEEWERCKEGVM
VOLCA   GAPERQSMTGGPSVKAESGATSGGAAAADAASTSQAILASVQQQVRERL-
                           3010      3020      3030      3040      3050
```

```
HUMAN*  RFAEMNPPR--QGEC---TIFAAVRDLMANLTLP----------------
CHICK   RFAELNSPR--PGEC---TIFAAVRDLMLNLTLP----------------
XENTR   RFAEMNPPR--PGEC---TIFAAVRDLMANLTLP----------------
DANRE   RFNEHHPPR--PGEC---TIFAAVHELMANLTLN----------------
CIOIN   RLEDIRNQQTINDPN---YLTVTVRYLMENLTFP----------------
ANOGA   RFSECSVLL--PDEC---SLFPAVHDLLLTLTLPNEPPAPGQMQMQLGGV
TETNG   RFNELHPAR--QGEC---TIFTSVHELMANLTLP----------------
DROME   RFAEFNA-Q--QNQC---TLFPAITDLLTNLQLAAEMPQPPPNQ------
BRUMA   ------------------ALWPAIYSLAHNFTVQR---------------
CAEEL   RFNSGDWNPGNRDEPIISSVAPPTYVNPGSVAGPSSVAAPGSVSIQQPGS
YEAST*  --------------------------------------------------
SCHPO   --------------------------------------------------
CANAL   --------------------------------------------------
CANGA   --------------------------------------------------
ASPNI   --------------------------------------------------
RHIO9   --------------------------------------------------
CRYNE   --------------------------------------------------
USTMA   --------------------------------------------------
ENCCU   --------------------------------------------------
ANTLO   --------------------------------------------------
ARATH*  RTVE-VNGS-AAGDLTQGKLKLVADSVQRTLHLCLQGLREGGNNNNNTHQ
VITVI   RTVE-MSGC-SPGDMSQGRLKIVADNVQRALHVNLQGLRDGSGVASNSGA
SOYBN   RTVE-VNGN-SAADVSQGRLKLIADSVQRNLHMCIQGLRDGSGVTTSSGA
SOLLC   RTVE-VNGN-SAGDANQGRLRVVADSVQRTLHAYLQGLRDGGGVAAGIGS
ORYSJ*  RAVETVNGSVAVGESGQGRLRMVAEMIQKQLQLCLQQLRDGPLSAGSTAS
HORVU   --------------------------------------------------
SORBI   RAVETVNGSGVAGEVGQGRLRMVAEMIQKQLQLCLQQLRDGPLSAGSTAS
SELML   -----VLEAAAAGQESSGRLRAVAESVQLTLQSALQQLRAV---------
PHYPA   RAVQ-------VAPGEGRGRLRALAEQVQMALQTALTQIGRGAGQ-----
VOLCA   ------------GDRVHDCVQQAVEIVYGALG------------------
                           3060      3070      3080      3090      3100
```

MED14

HUMAN*	--
CHICK	--
XENTR	--
DANRE	--
CIOIN	--
ANOGA	MQPGGGPGVPGGPGGPMGGQIGGPTPQVVPQVGSSPSPMMHSPMQQMGGG
TETNG	--
DROME	-----------SIGPPVGVGVG---------VGSSPNPMM--PMQQL---
BRUMA	--
CAEEL	VLQPGSMMGPQSVNALQYGMHRQPMGGPQSMQMNPSSVGQPGSVGGPGSH
YEAST*	--
SCHPO	--
CANAL	--
CANGA	--
ASPNI	--
RHIO9	--
CRYNE	--
USTMA	--
ENCCU	--
ANTLO	--
ARATH*	KEFTI---
VITVI	T---
SOYBN	A---
SOLLC	--
ORYSJ*	--
HORVU	--
SORBI	--
SELML	--
PHYPA	--
VOLCA	--

3110 3120 3130 3140 3150

HUMAN*	-------------PGGRP--------------------------------
CHICK	-------------PGGRP--------------------------------
XENTR	-------------PGGRP--------------------------------
DANRE	-------------SGGRP--------------------------------
CIOIN	--
ANOGA	GPQPGAYGGMVGGPGGGPQSGGPVGGG--------------------PGG
TETNG	-------------PCTRQ--------------------------------
DROME	-PQQVGPQGPV-GPGGYPQ----MGPN--------------------PGG
BRUMA	--
CAEEL	QQHMMNPGSVGPGSVGGPGSVNPGSVGYPQWNPPSVGQSYHHPLHHQQYP
YEAST*	--
SCHPO	--
CANAL	--
CANGA	--
ASPNI	--
RHIO9	--
CRYNE	--
USTMA	--
ENCCU	--
ANTLO	--
ARATH*	--
VITVI	--
SOYBN	--
SOLLC	--
ORYSJ*	--
HORVU	--
SORBI	--
SELML	--
PHYPA	--
VOLCA	--

3160 3170 3180 3190 3200

HUMAN*	-	-
CHICK	-	-
XENTR	-	-
DANRE	-	-
CIOIN	-	-
ANOGA	P	N
TETNG	-	-
DROME	P	Q
BRUMA	-	-
CAEEL	P	Q
YEAST*	-	-
SCHPO	-	-
CANAL	-	-
CANGA	-	-
ASPNI	-	-
RHIO9	-	-
CRYNE	-	-
USTMA	-	-
ENCCU	-	-
ANTLO	-	-
ARATH*	-	-
VITVI	-	-
SOYBN	-	-
SOLLC	-	-
ORYSJ*	-	-
HORVU	-	-
SORBI	-	-
SELML	-	-
PHYPA	-	-
VOLCA	-	-

MED15

Species	10 20 30 40 50
HUMAN*	--
CHICK	--
XENTR	--
DANRE	--
CIOIN	--
ANOGA	--
TETNG	--
DROME	--
BRUMA	--
CAEEL	--
YEAST*	--
SCHPO	--
CANAL	--
CANGA	--
ASPNI	--
RHIO9	--
CRYNE	--
USTMA	--
ENCCU	--
ANTLO	--
ARATH*	MDNNN---
VITVI	MGFGPKWIGWMWSCIFIAKFSVLVNGVPAGFFSSSKGLRQGDPLSPYLFV
SOYBN	MDSNNWRPN---
SOLLC	MDGNNWRAA---
ORYSJ*	--
HORVU	--
SORBI	--
SELML	--
PHYPA	-----MGCGKIPGGCQYRELSY----------------------------
VOLCA	--

Species	60 70 80 90 100
HUMAN*	--
CHICK	--
XENTR	--
DANRE	--
CIOIN	--
ANOGA	--
TETNG	--
DROME	--
BRUMA	--
CAEEL	--
YEAST*	--
SCHPO	--
CANAL	--
CANGA	--
ASPNI	--
RHIO9	--
CRYNE	--
USTMA	--
ENCCU	--
ANTLO	--
ARATH*	------------------------WR------------------------
VITVI	MGMEVLSVLITRAVEGGFIHGCRIWRGRGQGVKISHLLFADDTIVFCEAK
SOYBN	--
SOLLC	--
ORYSJ*	--
HORVU	--
SORBI	--
SELML	--
PHYPA	-----------------------TRGSLASVSKVIRVEWSMFRSACGGGG
VOLCA	--

MED15

```
HUMAN*  --------------------------------------------------
CHICK   --------------------------------------------------
XENTR   --------------------------------------------------
DANRE   --------------------------------------------------
CIOIN   --------------------------------------------------
ANOGA   --------------------------------------------------
TETNG   --------------------------------------------RRKMRW
DROME   --------------------------------------------------
BRUMA   --------------------------------------------------
CAEEL   --------------------------------------------------
YEAST*  --------------------------------------------------
SCHPO   --------------------------------------------------
CANAL   ------------------------------------------------MN
CANGA   --------------------------------------------------
ASPNI   --------------------MNPANFPNVNGGMSAGVKPPGQMQGVPKAD
RHIO9   ------------------------------------MNQQNPNIPNAVQQ
CRYNE   --------------------------------------------------
USTMA   --------------------------------------------------
ENCCU   --------------------------------------------------
ANTLO   --------------------------------------------------
ARATH*  --------------------------------------------------
VITVI   KESLLYLSWILFWFEAALGLKINLDKSMVIPVGEVEGVLELAAEVGCRVG
SOYBN   --------------------------------------------------
SOLLC   ------------------------------------------------QA
ORYSJ*  --------------------------------------------------
HORVU   --------------------------------------------------
SORBI   --------------------------------------------------
SELML   --------------------------------------------------
PHYPA   DSKGRILGTSSRGSGTGFDGSSSCAPTDDYIRNHPNHCLEMCPIVKEQLF
VOLCA   ----------MAMGYLGQGSLLHGVLPTPPVEAPASGQPALGHDHQSLAQ
                 110       120       130       140       150
```

```
HUMAN*  -----------MDVSGQETDW----------RSTAFRQ------------
CHICK   -----------MDVTGPETDW----------RSTNFRQ------------
XENTR   -----------MDA---DSDW----------RSAPFRQ------------
DANRE   -----------MDVPGPDSDW----------RSPAFRQ------------
CIOIN   ---------MDPNQMRPNDSW----------KTTQLRN------------
ANOGA   --------------MAEDNSW----------KTSNFRQ------------
TETNG   GTEALRPSAPAMEVPGPDSDW----------KSPQFRQ------------
DROME   ----------------MTEDW----------QSQKFRQ------------
BRUMA   ---------------MGDDDW----------PSQRFRD------------
CAEEL   ---------------MSEEDW----------PSPKFRE------------
YEAST*  --------------------MSAAPVQDKDTLSNAERA------------
SCHPO   ------------MNRNVDKGW-------QAQIRPNERQ------------
CANAL   IPPNQNSLQQMGGGSNPNASW-------RAMYSGEERQ------------
CANGA   -------------------------MSSKETIPMHQRS------------
ASPNI   MQHILNNVAQMLQSQGPWSGW-------RAEVSVRDRT------------
RHIO9   NQQQQQGLNLNLQNDVPNANW-------RDELNIQDRT------------
CRYNE   -------MSNNNQEQNPFAGF-----------------NM----------
USTMA   --------------------------------------------------
ENCCU   ------------------------------MLSQEERK------------
ANTLO   ----------------MPLNETYFSLNPMINISIEKRR------------
ARATH*  ------PSLPNGEPAMDTGDW-------RTQLPPDSRQ------------
VITVI   QLPTVYLGLPLGAPNRASSVWDGVEEKVRRRLALWKRQYLSKGGRITLIK
SOYBN   ---------QGTNPTMDTSDW-------RAQLPTDSRQ------------
SOLLC   QAPGGGEGGGAAAGAMESGDW-------RTQLMPDSRQ------------
ORYSJ*  --MERATAAHPASSSVSAAHW-------MATEDLGLRR------------
HORVU   --------------------------------------------------
SORBI   --------------------------------------------------
SELML   --------------------------------------------------
PHYPA   IPSVTREQIAVNKANMDNSSW-------RSSLAHDSRQ------------
VOLCA   PLPFQQFGMPIQAQGSLPADF--------GAGTTAPRS------------
                 160       170       180       190       200
```

MED15

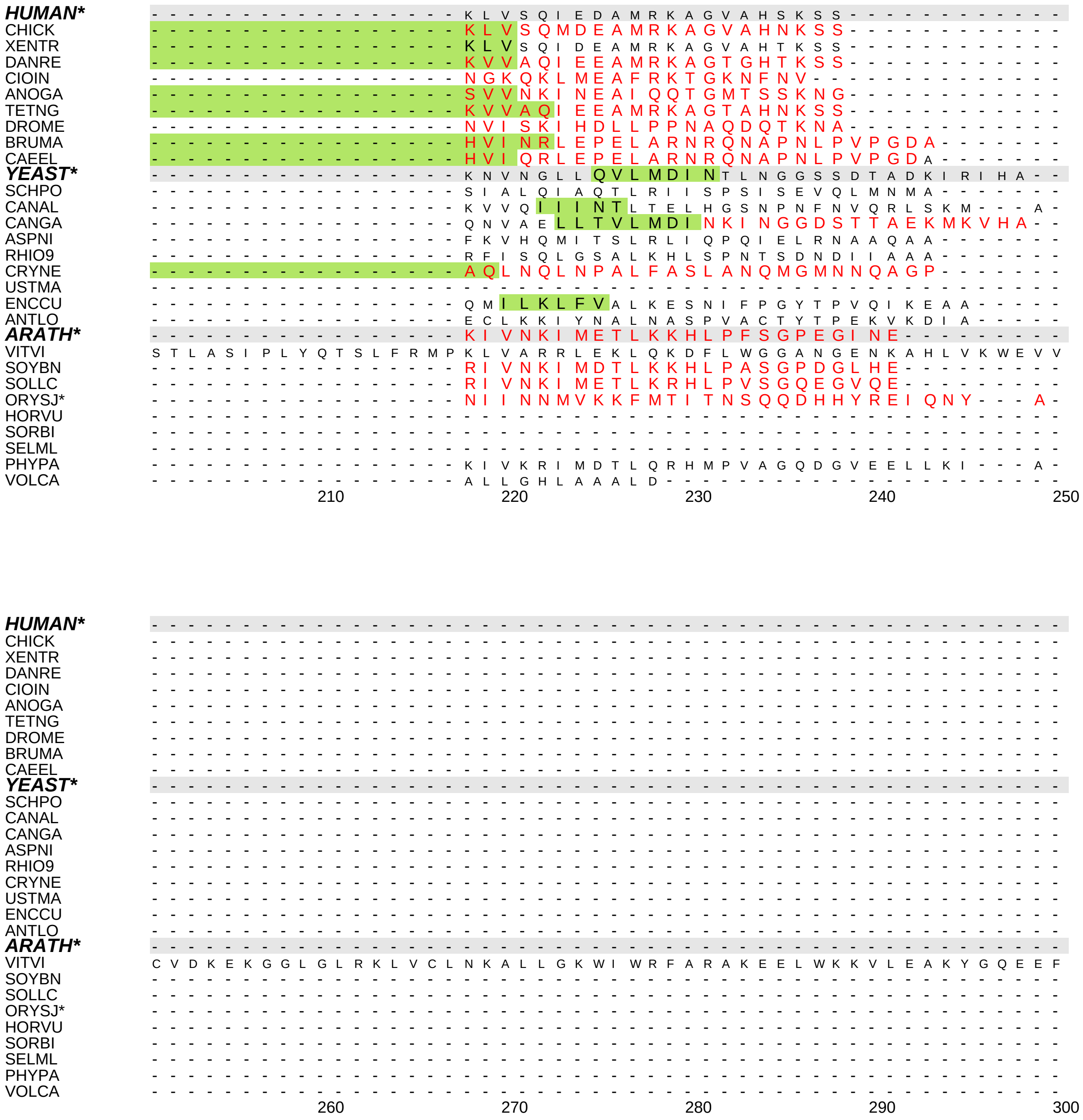
HUMAN*
CHICK
XENTR
DANRE
CIOIN
ANOGA
TETNG
DROME
BRUMA
CAEEL
YEAST*
SCHPO
CANAL
CANGA
ASPNI
RHIO9
CRYNE
USTMA
ENCCU
ANTLO
ARATH*
VITVI
SOYBN
SOLLC
ORYSJ*
HORVU
SORBI
SELML
PHYPA
VOLCA
210
220
230
240
250
260
270
280
290
300

MED15

```
HUMAN*  ------------------------KDMESHVFLKAKTR------------
CHICK   ------------------------KDMESHVFMKAKTR------------
XENTR   ------------------------KEMESHVFLKAKTR------------
DANRE   ------------------------TEMESHVFTKAKTR------------
CIOIN   ------------------------TTIEGNICTRAKSM------------
ANOGA   ------------------------IEMENHVFHKARNK------------
TETNG   ------------------------TDMENHVFIKAKSR------------
DROME   ------------------------GVMENHIFRKSRTK------------
BRUMA   ------------------------RQVEEYVFNKCVSK------------
CAEEL   ------------------------RQVEEYVFAKCMSK------------
YEAST*  ------------------------KNFEAALFAKSSSK------------
SCHPO   ------------------------LSFERQAFDGASSK------------
CANAL   ------------------------QDFEKLVYERSASK------------
CANGA   ------------------------KSFEAALFEKSSSK------------
ASPNI   ------------------------MSFEVKAFKEAATK------------
RHIO9   ------------------------KNFELNLYNKSPSK------------
CRYNE   ------------------------SQPQNQAQQPAMGN------------
USTMA   ----------------------------MSGFDQSHNG------------
ENCCU   ------------------------IRAEQRIYEESGSK------------
ANTLO   ------------------------ITSEYECCENSKSE------------
ARATH*  -------------------LRRIAARFEEKIFSGALNQ------------
VITVI   GWRTRKANGVFGVGVWKEILKELAWCWENMVFKVGKGN------------
SOYBN   -------------------LRKIAQRFEDKIYTAATSQ------------
SOLLC   -------------------LKKIAVRFEEKIYSAATSQ------------
ORYSJ*  ------------------------IRCEQDALNKTTNK------------
HORVU   --------------------------------------------------
SORBI   --------------------------------------------------
SELML   --------------------------------------------------
PHYPA   ------------------------WRFEEKIFQAATDQ------------
VOLCA   ------------------------QRFDHRRLSEADSWHAEILRTEASYL
                 310       320       330       340       350
```

```
HUMAN*  ----------------------------------DEYLSLVARLIIHFRD
CHICK   ----------------------------------EEYLSLVARLIIHFRD
XENTR   ----------------------------------EEYLSLVARLIIHFRD
DANRE   ----------------------------------EEYLSMVARLIIHFRD
CIOIN   ----------------------------------EEYLRFIDKIVQKFNN
ANOGA   ----------------------------------DEYLGFVARLILHVRE
TETNG   ----------------------------------DEYLSLVARLIIHFRD
DROME   ----------------------------------DEYLGLVAKLFMHYKD
BRUMA   ----------------------------------DEYMRTIAKVINAINC
CAEEL   ----------------------------------DEYMRTIAKVINAINC
YEAST*  ----------------------------------KEYMDSMNEKVAVMRN
SCHPO   ----------------------------------NEYLTTCGKKTAQLRD
CANAL   ----------------------------------EDYLRAIKMKVHQLRV
CANGA   ----------------------------------EEYQKTMKSKIDAMRS
ASPNI   ----------------------------------ADYDREFNDKLVHIRD
RHIO9   ----------------------------------NQYILAYAKKFHQIKF
CRYNE   -------------------------------------LRNIPPQLLQ---
USTMA   --------------------------------AFFNFNNLTPQQLNMLRQ
ENCCU   ----------------------------------EEYLRGMDERFQRIER
ANTLO   ----------------------------------SEYHSGIVQIIQRILS
ARATH*  ----------------------------------TDYLRKISMKMLTMET
VITVI   ---------------KIRFWTDPWCGNNMLSQGFPDLFSMAAQRNATVED
SOYBN   ----------------------------------PDYLRKISLKMLTMET
SOLLC   ----------------------------------QDYLRKISLKMLTMET
ORYSJ*  ----------------------------------EDYLRCIAQRIMNMEM
HORVU   --------------------------------------------------
SORBI   --------------------------------------------------
SELML   --------------------------------------------------
PHYPA   ----------------------------------QDYLRKISLKMLSLET
VOLCA   SQRNFDMAKVNNGDVAQEPKPCVSRESCTDSSADVEYLLAGGLSLPAMRR
                 360       370       380       390       400
```

MED15

Species	401–450
HUMAN*	IHN---
CHICK	IHN---
XENTR	IHN---
DANRE	IH--
CIOIN	EAR---
ANOGA	MTA---
TETNG	I---
DROME	MSR---
BRUMA	NSKPSAVPSLL---------------------------------------
CAEEL	NSKSAAVPSVL---------------------------------------
YEAST*	TYNTRKNAVTAA--------------------------------------
SCHPO	QIRDTL--
CANAL	QKQQIAANQGGQI-------------------------------------
CANGA	TRDKRKRESVGSA-------------------------------------
ASPNI	TRARHAA---
RHIO9	QLQNQNGSAPPMM-------------------------------------
CRYNE	--
USTMA	QSQMQQPVQMPQ--------------------------------------
ENCCU	VVSNRSRADG--
ANTLO	TGW---
ARATH*	KSQNAAG------------SSAAIPAAN----------------------
VITVI	YWDHNLGQGGWNLRLLRDFNDWELGLVGNLIVVLRDYRVTLEEDSVFWKE
SOYBN	KSQNTLA------------NNMPPNQVGP---------------------
SOLLC	KSQPPMT------------NSVQPNPASS---------------------
ORYSJ*	KVR---
HORVU	--
SORBI	--
SELML	--
PHYPA	KANGNPLQRPPPGAQGATGAPGAPGTGG----------------------
VOLCA	TSS---

410 420 430 440 450

Species	451–500
HUMAN*	--------------------------------KKSQASVSDPMNALQSLT
CHICK	--------------------------------KKSQASV-DPMNALQNLT
XENTR	--------------------------------KKTQPAVNDPMNALQNLT
DANRE	--------------------------------KKAQGGP-DPINALQNLP
CIOIN	--------------------------------VMQPNQEQHIQQ------
ANOGA	--------------------------------AAQQAQQDGGGNSSQQGG
TETNG	--------------------------------RKYSGTLADPMNALTNLT
DROME	--------------------------------KSQQQQQQQQQQG-----
BRUMA	--------------------------------QPSRIQS-----------
CAEEL	--------------------------------QPSQFHSPPCTTAALLGN
YEAST*	--------------------------------AANNNIKPVEQHHINNLK
SCHPO	--------------------------------QATQMKQMPSVYNNAGNV
CANAL	--------------------------------NPQQRQQQQQQQI-----
CANGA	--------------------------------SMMANLGQDGTNNNNNNN
ASPNI	--------------------------------AMQGGMMQQGPPTGMIGV
RHIO9	--------------------------------NDNQLQINLNALNGQQMS
CRYNE	--------------------------------QLQQQQQQRQQSGQPPSQ
USTMA	--------------------------------QMPQQQQQQQSQPPPGTQ
ENCCU	--
ANTLO	---ETAGVTS
ARATH*	----------NGTSIDSIPTNQGQLLPGSLSTNQSQAPQPLLSQTMQNNT
VITVI	GEGGLLKSRKPSHSMQPQVHNQGQQLPVPLAPNQSQTRQQLLAQNIQTNI
SOYBN	----SNKPPDQGLVLQSQVHNLGQQHSIPLS-SQLQPHQQLLSQNVQNNV
SOLLC	---GQNALGPGSHSMQSQVNSQAQQLPVPMVANQTQTRQPLLQQNLQNNM
ORYSJ*	--------------------------------------RSQSLQAGTTPS
HORVU	--
SORBI	--
SELML	--
PHYPA	-------LQMRSVAALGNGVQLGGQNQQQQQLQQRQQQQQLLTQSLQGNG
VOLCA	---------------------------------------ATTSHVLAAGG

460 470 480 490 500

MED15

HUMAN*	GGPAAGAAG-IGMPPRGPGQSLGGMGSLGAM----GQPMSLSGQP-PPGT
CHICK	GGPPAGAPG-MTIASRAQGAPMSGMGGLGPM----GQQMSLPGQQQPPGS
XENTR	GTAVVGGQG-LGLGVRPQGAQMGGMGGMAQM----AQQMNLPGQP-QPGA
DANRE	GGVPG------VIGPRPPGAQMGGMGQMS---------------------
CIOIN	--
ANOGA	G---------------------GGGGGSGMPDPINALQNLASQGTRPQMM
TETNG	G--VGGGPGAMGVGPRPTGAPVGGMGAMGPMQIGQHAMAGVAGNPQPLGP
DROME	---------------GPPPNAEMGGGQNMMQDPLNALQNLASQGNRNPQM
BRUMA	----ASQNYRSAGLGIPPDPQPTHQQHQQKVMDRYHTSLCQTPPMMQSQQ
CAEEL	TPAGGTPGYRAP---VPPDPQPTSAQARNPPVTVATTQASTTPS----AP
YEAST*	NSGNSANNMNVNMNLN----------------------------------
SCHPO	GALPTAGPNRLANNPRVMPRLQNQNVPMQAGMQQFARNMKLTPQ------
CANAL	---------------SNSNSMNPVNAQNVQFLRQQAQARSQSQAQIQARQ
CANGA	NNNNNNLNMAASFMGGDMFGRNQSPAQNSNANTNLNTNVGPGVN------
ASPNI	GQSPFSQQLSRS---MQPSPMPGQQQMQMSMNN-----------------
RHIO9	PLLQQQQPQKQA----ASPQVQPVVQQQMQDMTPLNQQQATINNQNQIRA
CRYNE	ASINDTLQNIML---PTFNRLQSQNSQQMQQERLQTSNNPAI--------
USTMA	PQPQMQQRNAMMPQLGLPAGFNQQQLQTLMAQQQSQLANGIMSNNQNPLM
ENCCU	--
ANTLO	KQRPTVIGNSQVFFGSSMGAS-----------------------------
ARATH*	AS-GMTGSTALP---SSMPPVSSITNNNTTSVVNQNAN---MQNVAGMLQ
VITVI	AS-GVPSSASLP---STLSSVTSLNQTPMQNVVGQNSG---MQNISGIPQ
SOYBN	ASQ------------PNLPPVSSLAQTTSQNIV-QNSN---MQNITG--P
SOLLC	ASTGLQNSASLA---PSLPSVSNLTQGTMPNVVGQNSNLQTMQNMPNVGQ
ORYSJ*	TQRPSSQQQNVCTTPQNPGQVPDQHRASAPNSQIEASQEQTVMVAAPDCY
HORVU	--
SORBI	--
SELML	--
PHYPA	AGGIQTPATGLT---SNLSAMTGVPQNPMAAMAGQVMN------------
VOLCA	LLPAPLRPGSIVATGSGSLPPPPLSSGPATHLPSLNSGIGPAAGAHHHIT
	510 520 530 540 550

HUMAN*	SGMAPHSMAVVSTATPQTQLQ--LQQV-ALQQQQQ-QQQFQ---------
CHICK	TGMAPHGCYCIAGSF--TILA--FLAF-QMQQQQQ-QQQ-----------
XENTR	SGMAPHGITGVAAGNQATQLQ--LQQI-AQHQQQQFQQQQQ---------
DANRE	--MGPHAMQGVAGGQQGAGAAGPMQQM-IQQQQQQ-QQQQQ---------
CIOIN	-------------QQHMQNMQQMQQQN-IQPQQNI-QQQNI---------
ANOGA	GQMGVGPGGPMGGQMGGAGQASNLLHS-LRPQMQM---------------
TETNG	APPSPSPPSAVGGPGQMPVQQ--MVQA-AAEAQNQ-QQQTQ---------
DROME	MPMGAGGGAPVPG---GPGTASNLLQS-LNQQRPG-QQQMQ---------
BRUMA	SPSGALSGGNMQLSQSSLQQHSPVTHP-VVPQVPY-NMLSP---------
CAEEL	NPPGGLPAPSASATAAVAAAVASFPSP-DTSIRPG-GQITP---------
YEAST*	-------------PQMFLNQQAQARQQ-VAQQLRN-QQQQQ---------
SCHPO	-------------QRQFLLQQSQIQQQ-RQQQQQQ-SQQPQ---------
CANAL	QQ-----------LRNMVNQQSQQQQQ-PQPQQTV-QPQSQ---------
CANGA	---GPNGNDGTANPQMFMNQQAQARQQAAARQLKN-RQMGG---------
ASPNI	-------------PGQQAAVQQRQQQP-QQPQAML-QQQRPQQRLGGAAA
RHIO9	SMFPQTMLQGRSPQPQQAQQQ----QQ-PQPQPPQ-QQQQQ---------
CRYNE	-------------QAQVAAQQAKARQA-AQAVQAA-QAQAH---------
USTMA	QMM----------RPQPMVQPQATQQQ-MQPQQHA-QMQGQ---------
ENCCU	-------------RGKSTQEQMGSYEH-ARPQFQG-FRNGS---------
ANTLO	-------------NAFVTMPGTRMHKI-PQSQINR-QEINA---------
ARATH*	DSSGQ---HGLS-SNMFSGPQRQMLGR--PHAMSS-QQQQQ-----PYLY
VITVI	NSVGNSMGQGVP-SNMFANSQRQSQGR--PQVVPQ-QQQQQSQSSQQYIY
SOYBN	NSVGSTISQNSNLQNMFPGSQRQMPGR--QQIVPP-QNSPQYLYQQQRLK
SOLLC	NLVGNAIGQGMP-PNMVANSQRQMQGR-QQQVVSQ-QQQQQSQTTQQYMY
ORYSJ*	LNFN---------TTAISPVAMCVHPS-QQPQSQQ-HQQQA---------
HORVU	--
SORBI	--
SELML	---------------MMQARQTQVQPQ-QQQQQQQ-QQQQQ---------
PHYPA	-------------PTMMNNRLQQMQQQ-QRQAVQQ-QQVSQ---------
VOLCA	QLRHPAALQMPPHPGLNAPQLQRPELP-QSQQHLQ-QQISS---------
	560 570 580 590 600

MED15

Species	601–650
HUMAN*	--
CHICK	--
XENTR	--
DANRE	--
CIOIN	--
ANOGA	--
TETNG	--
DROME	--
BRUMA	--
CAEEL	--
YEAST*	--
SCHPO	--
CANAL	--EHQQDQQNTS
CANGA	--
ASPNI	LNDDLNSLTPQEYENVCRIATQILQKTSPEDMNKIKMNLQNMSPDQKVYL
RHIO9	--
CRYNE	--
USTMA	--
ENCCU	--
ANTLO	--
ARATH*	QQQL-QQQLLKQNFQSGNVPNPNSLLPS-------------HIQQQQ---
VITVI	QQQL-QHHLLKQKY---------------------------QQQQQQ---
SOYBN	QKQL-----------------------------------QSQMQQQQ---
SOLLC	QQQLHQHQMMKQKFQPGS--TSQSLMQSHMQQQQQPQEQQQQLPQQQ---
ORYSJ*	--
HORVU	--
SORBI	--
SELML	--QQQ---
PHYPA	--QQQ---
VOLCA	--

610 620 630 640 650

Species	651–700
HUMAN*	--
CHICK	--
XENTR	--
DANRE	--
CIOIN	--
ANOGA	--
TETNG	--
DROME	--
BRUMA	-------------IPSVPGSQNTSPNHQMYSRVKTEQKSSGNDGGAAEIP
CAEEL	------------------GSQAPGGGPTPAPNVPFPNGSSQMNGGPAMGQ
YEAST*	------------------------QQQQQQQRRQLTPQQQQLVNQMKVAP
SCHPO	--------------------------QTQQPQASSPTAPNTEANQQRSGS
CANAL	S-----------QSTQQNVASGAGSGGRGNASSTQGQLPPQLVNLMRTSP
CANGA	---------------------------SSAQQQQLTQQQQQLLNQMRVAP
ASPNI	SKKGMDPITYFFRCQAMNHIRRVKRSRLEMSRNNQNNGGDSANNLMGDPM
RHIO9	------------------AAPGNQQQQVNAQMLALQRTAAQAMAAAAAAA
CRYNE	------------AQAQAQAQAQAQTQQ-----------------------
USTMA	------------MPHQQPLGSQPAHIQQQPQQTNNLGAPPQMTGLGAPTP
ENCCU	--
ANTLO	---------------------------------TGVYTSHALDSNAKACS
ARATH*	------------QNVLQPNQLHSSQQPGVPTSAT-QPSTVNSAPLQGLHT
VITVI	------------QTLLQSNQFQSSQQSVMQTS-----SGMQSAPLSGLQQ
SOYBN	------------QNLLQPNQLQSSQQSIMQTSSVMQPSMMQTSSLSSIPQ
SOLLC	------------QNLLQPTQTQPSQQAMM------QPSSIQSTSLSNLQQ
ORYSJ*	-----------------------KQLHPTNVVGYNPTSLNQIQGQSVSG-
HORVU	--
SORBI	--
SELML	------------QPQQQPQHQQQQQQPQ----------------------
PHYPA	------------QNNQQQQILYQQQQRLQQHQAMMKQKLQQ---------
VOLCA	-----------------HAGSVPSAQQSGVLGLREPTRSETLGSGIGTAG

660 670 680 690 700

MED15

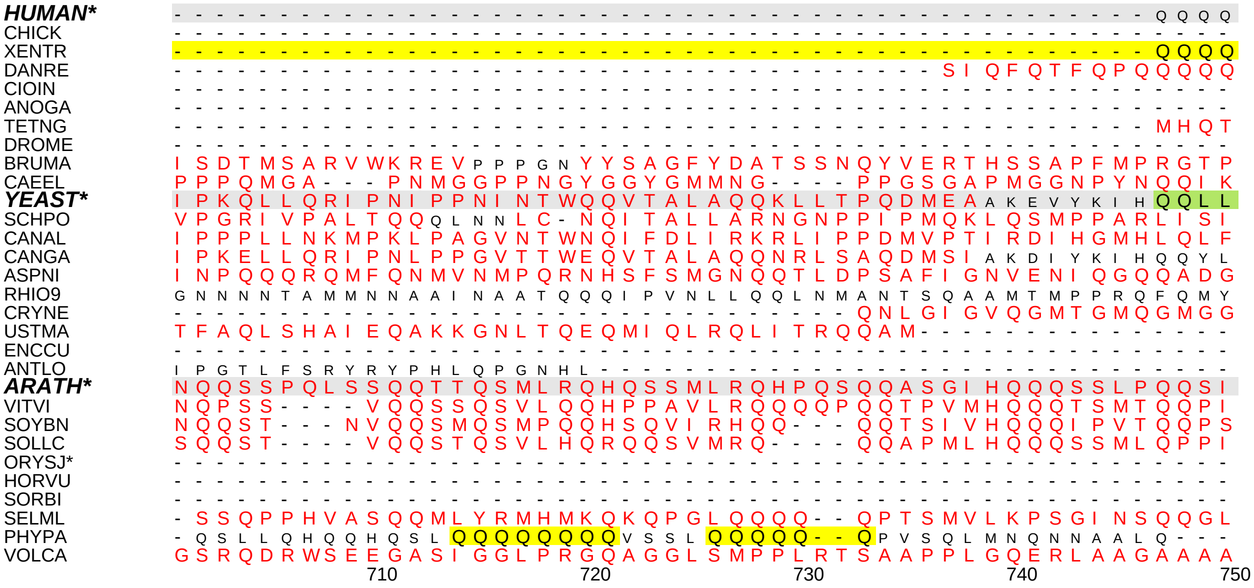
HUMAN*
CHICK
XENTR
DANRE
CIOIN
ANOGA
TETNG
DROME
BRUMA
CAEEL
YEAST*
SCHPO
CANAL
CANGA
ASPNI
RHIO9
CRYNE
USTMA
ENCCU
ANTLO
ARATH*
VITVI
SOYBN
SOLLC
ORYSJ*
HORVU
SORBI
SELML
PHYPA
VOLCA
710
720
730
740
750

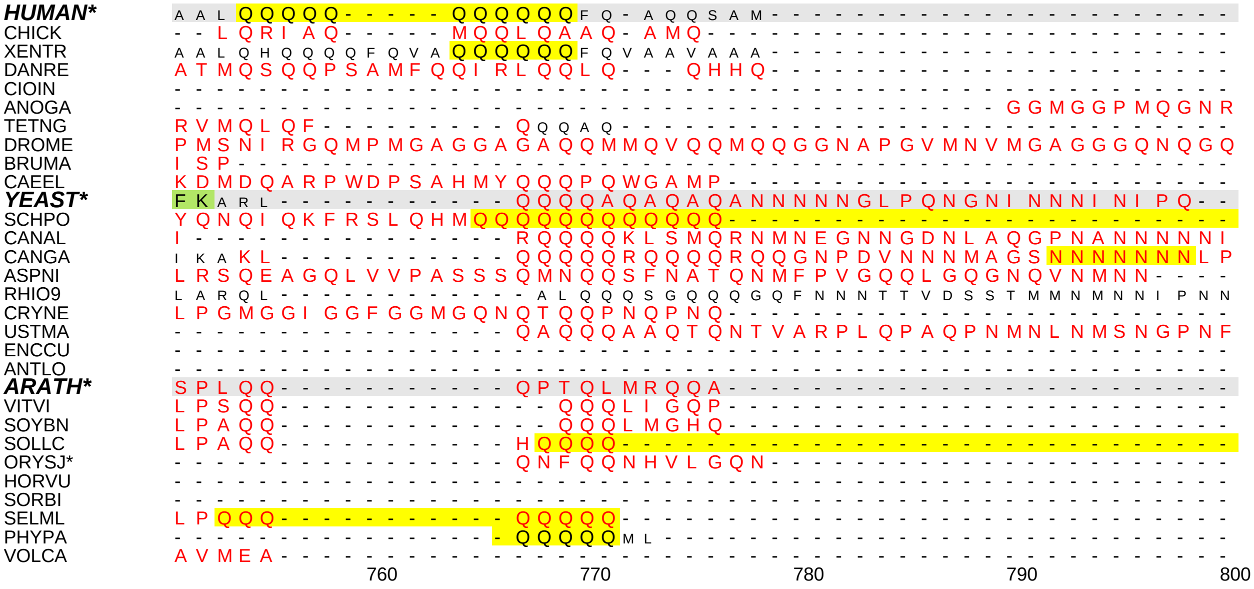
HUMAN*
CHICK
XENTR
DANRE
CIOIN
ANOGA
TETNG
DROME
BRUMA
CAEEL
YEAST*
SCHPO
CANAL
CANGA
ASPNI
RHIO9
CRYNE
USTMA
ENCCU
ANTLO
ARATH*
VITVI
SOYBN
SOLLC
ORYSJ*
HORVU
SORBI
SELML
PHYPA
VOLCA
760
770
780
790
800

MED15

```
HUMAN*  --------------------------------------------------
CHICK   --------------------------------------------------
XENTR   --------------------------------------------------
DANRE   --------------------------------------------------
CIOIN   --------------------------------------------------
ANOGA   VGMGPGNQMGGMMGGPNQMQGPGAGMVGGMPGQMGVGMGPGGMSGGKIQM
TETNG   --------------------------------------------------
DROME   IVGNPGQQMGVGVGMPNQMVGPGPNSGPAVGGAGGPNAAPGAGGPGPNQM
BRUMA   --------------------------------------------------
CAEEL   --------------------------------PQQPHGYPGRPMNGQGAT
YEAST*  --------------------------------------------------
SCHPO   --------------------------------------------------
CANAL   NTSTHNNSNPTPNNFNNLTPQQRESLLRQTRMQQQLEQQKKGQQP-----
CANGA   MA------------------------------------------------
ASPNI   -----------------------AGISPQFLTQQHLPNAQPGPQDRPQ--
RHIO9   VAQNFRPPVSNVNTNNNNTVMQTNNFQNQNQQAQNMMGQQQHLNPEIL--
CRYNE   --------------------------------VQRNQNQFSVHSHQPD--
USTMA   --------------------ANAMQRAPQMPTQAQMMAGLQQVRPGQSTP
ENCCU   --------------------------------------------------
ANTLO   --------------------------------------------------
ARATH*  --------------------ANSSGIQQKQMMGQHV----VGDMQQQH--
VITVI   --------------------PNATTMQSNQLIGQQNSFSDLQQQQQQQ--
SOYBN   --------------------PNTTNMQHAQMLGQQNNVGDIQQ-------
SOLLC   --------------------------QQQQLIAQQTNVANLQ--------
ORYSJ*  --------------------------------------------------
HORVU   --------------------------------------------------
SORBI   --------------------------------------------------
SELML   --------------------------------------------PQQQPP
PHYPA   --------------------------------------------------
VOLCA   ------------------------TVDQSLPCPEGPPAVMACRSPDMD--
                  810       820       830       840       850
```

```
HUMAN*  -------QQQFQAVVQQQQQLQQQQQ-----QQQHLIKL-----HHQNQQ
CHICK   --------------VQQQQQQQQQQQ-----QQ---------------IP
XENTR   -------QQQQQQQQQQQQQVQQQQQLQAAAQQQHILKLQL--QQQQNQQ
DANRE   -------NQQMQHQNQQQQQAQNQQQ-----QNQLHQTRI---QQQLQLQ
CIOIN   -------QQQNIQQQNLQQQNIQQQ-------------------------
ANOGA   GVGPGGPNQMNPMVMGQIQAQLQNQNAMGGQQMGTVGGTMNAGNQMGQMV
TETNG   -------AQSIQHMVQQQVQAQ----------------------------
DROME   QGGPM--NVNAMQQMPPMQQIQQNQLGMGMNPMMRMGQGNGMGG------
BRUMA   -------SQQLTASPHHHHQHHQQH-------------------------
CAEEL   PTGPSSVLESLINQPQQYPGHHNQM-------------------------
YEAST*  -------QQQMQPPNSSANNNPLQQ-------------QSSQNTVPNVLN
SCHPO   -------QQQQQQQQQQQQQQQQQQQQQKQAPQNAFFPNPQGNVGAQSLQ
CANAL   -------MQQATSSSQQQQQKQQQQ----QQPV------------SQSGQ
CANGA   -------QQQMQQRQQQQQQSQQQQ-------------NRNPNQRHNVLS
ASPNI   -------QATQFQSQPQTTQAQRVQ----AAQ------------------
RHIO9   -------MQHLQQQQQQVQQAQQQQ----------------AQQVQTPAQ
CRYNE   -------LGLNQSSQPQSSNTNIQQGNIGQGQSGQFNLGMNMGMIGDDSS
USTMA   TMGSQGFLQPAAPSQQQQQQQQQQQ----------------QTQVSNQIQ
ENCCU   -------------EEPQDSTKQGYH-------------------------
ANTLO   -------VGQDDFISQHVPGIRGYQARDCTVP------------------
ARATH*  -------QQRLLNQQNNVMNIQQQQSQQQPLQQPQQQQKQQPPAQQQLMS
VITVI   -------QQRLLSQQNNLPNLQQQQ----------------QQQQHQLMA
SOYBN   -------PQRMLSQQSNLTNLQQRQ---------------------QLIN
SOLLC   -------QNQLMSQQNAMPDVQQRL----------------VGQQNNYMH
ORYSJ*  -------ASGSGTQQRQLVETPEQH-------------------------
HORVU   --------------------------------------------------
SORBI   --------------------------------------------------
SELML   VTLLQHHQQQQQQQQQQLPPLQQQQ-------------------------
PHYPA   -------QQQMMQSKQQQAQLQQQQ-------------------------
VOLCA   -------TDLLTRTPAGSPQDTEWQ-----SPLGPGGLLSDAGGGNVGCV
                  860       870       880       890       900
```

MED15

```
HUMAN*  Q--IQQQQQQLQRIAQLQL-------------------------------
CHICK   Q--QQLQQQPPQQVMQQQM-------------------------------
XENTR   QQLQQQQQQQLQRIAHLQQ-------------------------------
DANRE   Q--LQQQQQQQQQLHQQQV-------------------------------
CIOIN   --------------------------------------------------
ANOGA   GANAGMNPQAMGMASNQ---------------------------------
TETNG   --------------------------------------------------
DROME   --------------------------------------------------
BRUMA   --------------------------------------------------
CAEEL   --------------------------------------------------
YEAST*  QINQIFSPEEQRSLLQEAIETCKNFEKTQLGSTMTE--------------
SCHPO   SMSPQDQPSTQQQQPQRTAAPPNNPNVNATNNNRINIEMLNIPVPKQLFD
CANAL   TPQLAQQPNNQQQQQQQPQITITQEDYAKYSNDALNL-LAKLQQQKQMQG
CANGA   QINQMFTADEQRALLQEAMEACKNFQKTHFGGQMSD--------------
ASPNI   ---KAQMAMSQANMQQPITQSPAMPMLNRPIAAPGQMSPAQAAAQVHPSS
RHIO9   ALAQAQQQAQQQAQQQAQQQAQQQAQQQAQQQ------------------
CRYNE   TQNQNQNQPPPPQPAPSNPTPQISPEVHEFMRTRSDIASAVFKKHGNPQA
USTMA   SSPQVMQGQVQMARAPTPQQIQGMPQNAQQSQQILGQTPAQPAQQRPPVG
ENCCU   --------------------------------------------------
ANTLO   --------------------------------------------------
ARATH*  QQNSLQATHQNPLGTQSNVAGLQQPQQQMLNSQVGN--------------
VITVI   QQSNLSNIHQQQLGHQSNVSALQQ-QQQMLGTQSGN--------------
SOYBN   QQNNPANIHQQ---LGNNGPGLQ--QQHLLGHESGN--------------
SOLLC   QQNNFQNMHQQQLGSQSSIAGVQQ-QQLSGSQQPGN--------------
ORYSJ*  --------------------------------------------------
HORVU   --------------------------------------------------
SORBI   --------------------------------------------------
SELML   ------------QQQQPQLAGLSQPQ------------------------
PHYPA   --------------------------------------------------
VOLCA   GTVAAASAEVDDRHISASAFLEFAGEAGLFTSSGGGGGGLNCGRGGGGGG
                 910       920       930       940       950
```

```
HUMAN*  --------------------------------------------------
CHICK   --------------------------------------------------
XENTR   --------------------------------------------------
DANRE   --------------------------------------------------
CIOIN   --------------------------------------------------
ANOGA   --------------------------------------------------
TETNG   --------------------------------------------------
DROME   --------------------------------------------------
BRUMA   --------------------------------------------------
CAEEL   --------------------------------------------------
YEAST*  -----PVKQSFIRKYINQKALRKIQALRDVKNNNNANNNGSNLQRAQNVP
SCHPO   QIPNLPPNVKIWRDVLELGQSQRLPPEQLKLIGMLYRKHLQIILQHRQQQ
CANAL   QINGQMKEAFIKKFIIHQKTLQYKQRLSQQATGNANVNIGPSSTNQQQL-
CANGA   -----ANKQAFIKKFINSKALKKLEAMRMAQGGNNNANLNKG-------Q
ASPNI   RQPSTKQLPANVQPMGTQQGIQNRPPMPANFPPHIQEQLARMTPEQRNAF
RHIO9
CRYNE   ALE----------------------------------------DLQRIVG
USTMA   RPDPTPGTSNQQNPLNFLRLLHQRVTDIESKLNSDLSTEERQVVQQQHAE
ENCCU   --------------------------------------------------
ANTLO   --------------------------------------------------
ARATH*  --------------------------------------------------
VITVI   --------------------------------------------------
SOYBN   --------------------------------------------------
SOLLC   --------------------------------------------------
ORYSJ*  --------------------------------------------------
HORVU   --------------------------------------------------
SORBI   --------------------------------------------------
SELML   --------------------------------------------------
PHYPA   --------------------------------------------------
VOLCA   DSPRSPSHLSVLAAMQ----------------------------------
                 960       970       980       990      1000
```

MED15

```
HUMAN*   ---------------------------------------QQQQQQQQQQQ
CHICK    ---------------------------------------QQMQQQQQQQQ
XENTR    ---------------------------------------MQQMQQQQQQQ
DANRE    ---------------------------------------HAQAQAQAQAQ
CIOIN    ---------------------------------------NLQQQNIQQQN
ANOGA    ---------------------------------------VVRQQQLQQQG
TETNG    --------------------------------------------------
DROME    ---------------------------------------PQGMPGQGMQG
BRUMA    ------------------------QPSVLENLINSSHYGSPMQSSQHVNG
CAEEL    --------------------------GPPGDRNVAAQRAAAQQQQQQQQQ
YEAST*   MNIIQQQQQQNTNNNDTIATSATPNAAAFSQQQNASSKLYQMQQQQQAQA
SCHPO    LNKIQNARMNSQNAPNTNKLGNPQPDNTGNPQAF-----SQQAFAQQQQQ
CANAL    ---------------------------------------SQIPNQQQQQQ
CANGA    ADMLQRQQAN-----------------------------MQMNQQQQRAA
ASPNI    FLNQQRRMMASNP--------------------------ALARQNAVQPN
RHIO9    ---------------------------------------AQQQAQQQAQQ
CRYNE    MLKGQNQAQNQPQAQTGNNMGAAGVPVGINGQMFNQRIASGQTQSAQNQG
USTMA    AKRLQSALLQRLNVQNAQQFA------------------ALMQQGQQTPG
ENCCU    -------------------------------YGNPRLERGERRLSEQLFT
ANTLO    ---------------------------------------PLLRHHEAAPN
ARATH*   ---------------------------------------SSLQNNQHSVH
VITVI    ---------------------------------------PSMQTNQHPVH
SOYBN    ---------------------------------------ADMQTSHHSAH
SOLLC    ---------------------------------------SGLTSNQHPIH
ORYSJ*   ---------------------------------------QLLRMKQQHMR
HORVU    --------------------------------------------------
SORBI    --------------------------------------------------
SELML    ---------------------------------------SNMSQHQPSLM
PHYPA    ---------------------------------------QQQQQQQQQQQ
VOLCA    ---------------------------------------LSRQQQQQQQQ
                   1010      1020      1030      1040      1050
```

```
HUMAN*   QQQALQ--------------------------------------------
CHICK    VAQAQQ--------------------------------------------
XENTR    QQ------------------------------------------------
DANRE    AQAQAQ--------------------------------------------
CIOIN    IQQQNL--------------------------------------------
ANOGA    MGQVQM--------------------------------------------
TETNG    --------------------------------------------------
DROME    MPQGPH--------------------------------------------
BRUMA    SGNQSV--------------------------------------------
CAEEL    QQQQRP--------------------------------------------
YEAST*   QAQAQA---------------------------QAQAQAQAQAQAAQAAQ
SCHPO    QQQQLH---------------------------RTSNPTSASVTSQNGQQ
CANAL    QQQQQQ---------------------------VPQSQPHASQQSPVLQQ
CANGA    QNQRRG---------------------------PVMNDAVSQGYNNQMNS
ASPNI    MAMQQG---------------------------IPQPGQSQHMINGQMVN
RHIO9    QAQQQA---------------------------QQQAQQQAQTKAQALAQ
CRYNE    QSQQAGHQRIPSSGNFDLPGSQFANTLSQLEALQQVKERQQRGGLKTPQM
USTMA    QNQQQP---------------------------PPQQPNQASQT------
ENCCU    ENKQVF--------------------------------------------
ANTLO    VTQAFR--------------------------------------------
ARATH*   MLSQPT---------------------------VGLQRTH-QAGH-----
VITVI    ILQQSK---------------------------VPVQQQTQQGVS-----
SOYBN    MLQQPK---------------------------VPMQQQSQQSTS-----
SOLLC    LMQQPK---------------------------VPVQQQMLQSTT-----
ORYSJ*   GNQQQN----------------------FTQRNQILPAQQAHLGKMQIGH
HORVU    --------------------------------------------------
SORBI    --------------------------------------------------
SELML    QQQQQP--------------------------------------------
PHYPA    QSQQQQ----------------------VATPTQSQQSQQSQPIGQQPGQ
VOLCA    QQQQQS---------------------------LLLQQQQQ---------
                   1060      1070      1080      1090      1100
```

MED15

```
HUMAN*  -------------------------AQPPIQQPPMQQPQP----------
CHICK   -------------------------SQLPPQSQPQPQPMV----------
XENTR   --------------------------QPPPQQ-VMQLQQM----------
DANRE   -------------------------AQAQAQAQAQAQAQAQAQ-------
CIOIN   --------------------------------------------------
ANOGA   --------------------------------------------------
TETNG   --------------------------------------------------
DROME   --------------------------------------------------
BRUMA   -------------------------SDLPFGNAMQQNDERMYT-------
CAEEL   -------------------------GMVPNQGMMSSEDQTVYS-------
YEAST*  -------------------------AQAQAQAQAQAQAQAQAQ-------
SCHPO   PINTKLSANAAKTNYQSYLTNKARNATQPTQPPVSQVDYSNNLPP-----
CANAL   -------------------------SQPPQQAPVQNVPQG---SP-----
CANGA   -------------------------AADSTMNNSNQPMNIGNN-------
ASPNI   PQNMRAS------------------MDMQQQFASLGGAQQPNQMI-----
RHIO9   -------------------------AQIQAQQQVQQAQQMKSQ-------
CRYNE   PQSNTLPMTSPQMGNNANNLNPPFNLTLPNQPNNAQNPVQNRQ-------
USTMA   -------------------------SSQPQQQQQQQQQQQQQQGLSMMGS
ENCCU   --------------------------------------------------
ANTLO   --------------------------------------------------
ARATH*  -------------------------GLYSSQGQQSQNQPSQQQMM-----
VITVI   -------------------------NLLATQGQQSQQQPSQQQLM-----
SOYBN   -------------------------NLLLPHSQQSQPLGSQQQLM-----
SOLLC   -------------------------TLLPNQGQQSQSQPAQQQMM-----
ORYSJ*  P------------------------AVQNNQQNVGMSCQHMTP-------
HORVU   --------------------------------------------------
SORBI   ---------------------------MQPQSQQNQLQQPQQHMM-----
SELML   -------------------------YMQQQQQQPSAQALHQQQLL-----
PHYPA   -------------------------QGQLGQQQSGSNQQPQQR-I-----
VOLCA   -------------------------QIRQLSRGGLSMQQTMQP-------
                          1110      1120      1130      1140      1150

HUMAN*  --------------------------PPSQALPQQLQQMHHTQHHQPPPQ
CHICK   --------------------------SQAQAI------------------
XENTR   --------------------------QQQQVAQSQQQQLLSTQPQAASLV
DANRE   --------------------------AQAQAQAQSIQQMQQQQQLQVQAQ
CIOIN   --------------------------------------------------
ANOGA   --------------------------------------------------
TETNG   --------------------------------------------------
DROME   --------------------------------------------------
BRUMA   --------------------------------------------------
CAEEL   --------------------------------------------------
YEAST*  ---------------------------AQAQAQAQAQAQAQAH-AQHQPS
SCHPO   --------------------------NLDTSSTFRSSASPPSAFTKAGNE
CANAL   --------------------------GLGNMATPQMKQGITSNPTNLSSP
CANGA   ---------------------------GVNMIPNQSQQQQQTNRPKEQTP
ASPNI   --------------------------PGQQMTVQQRQQQLQQQQQLHQFQ
RHIO9   ---------------------------QQQSQTTQMNQQQQVASNTMQPT
CRYNE   -------------------------------TPRMSNNIPTLPSGPAAP
USTMA   NIQASPVVRPPNKPGSARPPQGQFSSPMLNQSVPQAQSGQQAPPQQQTPQ
ENCCU   --------------------------------------------------
ANTLO   --------------------------------------------------
ARATH*  --------------------------PQLQSHHQQ----LGLQQQPNLLQ
VITVI   --------------------------SQFQSQSTQLQ------PQPNSLQ
SOYBN   --------------------------PQIHTQSAQLQQQLGLPQQLNPSQ
SOLLC   --------------------------SQSQSQPGQLQPPLGLQQQANQLQ
ORYSJ*  --------------------------PQCQVATAQQSSLGCDSPQTLEPI
HORVU   --------------------------------------------------
SORBI   --------------------------SQFQSQPNQVQQQLG---------
SELML   --------------------------SNGQQMLQQ------------QML
PHYPA   --------------------------GQSGGLQQQQQQQQQQQQQTNMLL
VOLCA   --------------------------------------------------
                          1160      1170      1180      1190      1200
```

MED15

HUMAN*	PQQPPVAQ--
CHICK	-SGQIPGQ--
XENTR	AQGQIPSQ--
DANRE	GQPQVQGQ--
CIOIN	--
ANOGA	--
TETNG	--
DROME	--
BRUMA	--
CAEEL	--
YEAST*	QQPQQAQQQPNP-----------------------------------LHG
SCHPO	ALSVPLSGARNTAASRPTNLAAGNSSASIVQQLLECGGTMGQQKMTS---
CANAL	LVPQQVPVSNNNSASQNNNINRGRPVTAPANITMSSILPPLTDEVKL---
CANGA	QQPQQRIQSNRS-----------------------------------VPM
ASPNI	LLRQQAGSNM--
RHIO9	QMNATQAVHP---------------------------------------S
CRYNE	RPPQGTNPVQSGARPLP---------------------------------
USTMA	VRPSPQMGAINVAGQPSMTGLQGVPSLRPEQFMKALVDLMKKRGTPI---
ENCCU	--
ANTLO	--
ARATH*	QDVQQRLQASGQ--------VTGSLLPPQNVVDQQRQLYQSQRTLPE---
VITVI	RDMQQRLQTS------------GALLQTQNVIDQQKQLFQSQRALPE---
SOYBN	RDMQQRIQAS------------GSLLQQQNVLDQQKQLYQTQRTLPE---
SOLLC	REMQQRLQPS------------APLLQQQNVMEQQKQLYQSQRVAPE---
ORYSJ*	--
HORVU	--
SORBI	--MQQRLQTS------------AGMLLQQNNIDQQKQYVQAQRGLQE---
SELML	QPKQQQALPQQN----------------------QPLGMMQQRAVSE---
PHYPA	QAQQQRAIPDTS--------TGFFCFKHMYGMLVQRIFLESFLLLGT---
VOLCA	--
	1210 1220 1230 1240 1250

HUMAN*	--
CHICK	--
XENTR	--
DANRE	--
CIOIN	--
ANOGA	--
TETNG	--
DROME	--
BRUMA	--
CAEEL	--
YEAST*	LTPTAKDVEVIKQLSLDASKTNLRLTDV-TNSLSNEEKEKIKMKLKQGQK
SCHPO	---------RIRELTERVMHSLMRPV---PLDLPHDQKVMIASLIKSAYP
CANAL	---------RLRQLIEEVSRNNVVLRDV-TSLLSNQDKTTVRDSMNRIQE
CANGA	LNPTPEDVEVVRRISAEAAKTQLRLTDL-TNSLTPQERDEIKKRLQKNQQ
ASPNI	--------------------------------------EMTPEEISRMDN
RHIO9	LQQRTQAAEYVRQMDESVRNSRVRNNP--IEGISEQEKAAIRDSVALIKP
CRYNE	--
USTMA	-----------------------------TSLPKVGGRELDLYRFYQIVQ
ENCCU	--
ANTLO	--
ARATH*	-------------MPSSSLDSTAQT----ESANGGDWQEEVYQKIKSMKE
VITVI	-------------ASSTSLDSTAQT----GTINVGDWQEEVYQKIKRMKE
SOYBN	-------------TSATSLDSTTQT----AQLSGADWQEEVYQKLQTMKE
SOLLC	-------------ASSTSLDSTAQT----GNANAADWQEEVYQKIKSMKE
ORYSJ*	-----------------------------VIAGEVDWREEIFQKIKSFKD
HORVU	---MIKNLKD
SORBI	-------------APSTSVDSTAQT----GH-PGGDLQEELYQMIKSLKD
SELML	-------------ASSASVDSGQT-----AQSATIDQQETVWNKLQNLKD
PHYPA	-------------NGGASVESSAQSGPTGAAAGNVDQRELVWNKLQLLKD
VOLCA	--
	1260 1270 1280 1290 1300

MED15

```
HUMAN*  -----------------NQPSQLPPQS-----------------------
CHICK   -----------------VMPVTLTP-------------------------
XENTR   -----------------VMPVTLTPQQ-----------------------
DANRE   -----------------GGAVQMPPHSQQQQVLVPQMVQGQHSQMS----
CIOIN   --------------------------------------------------
ANOGA   --------------------------------------------------
TETNG   -----------------AQVGQMPPHMVLQS-LAGQMVSSQHVPLSCFLC
DROME   --------------------------------------------------
BRUMA   -----------------EKLRSLRP----------------YVESLRARA
CAEEL   -----------------AKLRNMRG----------------SCDSLRTRA
YEAST*  LF---------------VQVSNFAP----------------QVYIITKNE
SCHPO   MF---------------SRTNQLIC----------------LFYCLTGNE
CANAL   QY---------------GNVDSIIS----------------YFYLLTKNG
CANGA   LF---------------AQVSSYAP----------------QVYLFTKSE
ASPNI   MPFP-------------PAIFNNNP----------------NAASIPKNI
RHIO9   MY---------------DKIDSLLP----------------VFFALTSNV
CRYNE   -----------------NQVIATWP----------------LDKLIGASS
USTMA   AHGGSEGAHQKGAWPAIAMSLELAPLGSPQTQALGQALATEYRNYLAPFE
ENCCU   -----------------GMEDGFSP----------------SKIMGMNRK
ANTLO   -----------------------------------------KWDMHDRDI
ARATH*  TYLPDLNEIYQRVAAKLQQ-DS-MP----------------Q----QQRS
VITVI   MYLPDLNEMHQKIAAKLQQHDS-LP----------------Q----QPKT
SOYBN   SYLPEMNEMYQKIANKLHQHDS-LP----------------Q----QPKL
SOLLC   SYLAELNDLYHKIASKVQQHDS-LP----------------Q----RPQN
ORYSJ*  AYLSEVLEYDQIVHVPKLTEEQLRS----------------LP---VENA
HORVU   QYFPELSELFNKISVKLQHVDNMIP----------------P----QKPS
SORBI   QYFAELNDLYNKISMKIQHIDNHMP----------------A----QKSA
SELML   KYLPDMNDLRNMLVARSQQS---MP------------------------A
PHYPA   KYLQDMTELFRMLSTRSTQP---MP------------------------A
VOLCA   -----------------GPLYGGTS----------------VPQLMRPSA
                 1310      1320      1330      1340      1350
```

```
HUMAN*  --------------------------------------------------
CHICK   --------------------------------------------------
XENTR   --------------------------------------------------
DANRE   -ALSQ---------------------------------------------
CIOIN   --------------------------------------------------
ANOGA   --------------------------------------------------
TETNG   CALKQARA------------------------------------------
DROME   --------------------------------------------------
BRUMA   QQCRLEGN----------------------------EIAASKFDTMCNVL
CAEEL   RQCRHEGN----------------------------HEAAHKLEVMLSVL
YEAST*  NFLKEVFQ------------------------------LRIFVKEILEKC
SCHPO   EATIQLIQ------------------------------MRHIFKLQLEGL
CANAL   DGTKRLIQ------------------------------MKYMTKNILDNL
CANGA   SFLKEVLQ------------------------------LRIFIKEILEKC
ASPNI   KTWGQLKQ------------------------------LAAASPQLLGGL
RHIO9   EATKRLML------------------------------MKYMFEDQLNAL
CRYNE   NLSKKIIESEAAHGNIIQPGKPSTMGLGVPGRTAAEQAAKFQLLVMIAEI
USTMA   ETWQRAMQ------------------------------HQYQMSMASVGL
ENCCU   HNLEEMGD------------------------------------------
ANTLO   NRLHNIRT------------------------------SASMRNSVLHNF
ARATH*  DQLEKLRQ------------------------------FKTMLERMIQFL
VITVI   EQLEKLKI------------------------------FKAMLERMITVL
SOYBN   DQIDKLRA------------------------------YKSMLERMMALL
SOLLC   EQIEKLKM------------------------------FKITLERIVLFL
ORYSJ*  EKLRRIRH------------------------------VKKIIAIMLDLL
HORVU   EQYDRMKN------------------------------FKTMLGRILHML
SORBI   EQYEKMKG------------------------------FKLMLERTLHFL
SELML   DQLQKLNH------------------------------YKDVLSRMIPYL
PHYPA   EQLQKLKH------------------------------YKDVLHRMIPYL
VOLCA   AMFTAA--------------------------------AAAGLQEGLPRL
                 1360      1370      1380      1390      1400
```

MED15

```
HUMAN*   --------------------------------------------------
CHICK    --------------------------------------------------
XENTR    --------------------------------------------------
DANRE    --------------------------------------------------
CIOIN    --------------------------------------------------
ANOGA    --------------------------------------------------
TETNG    --------------------------------------------------
DROME    --------------------------------------------------
BRUMA    DGKSRVSF-------------------------EYLLQIEAWIYKK----
CAEEL    EGKRVVSL-------------------------EYLNHLEMWIARK----
YEAST*   AEGIFVVK-------------LDTVDRLIIKYQKYWESMRIQILRRQ---
SCHPO    QQGVFTCA-------------PQTLAKIKEKTSRYFAFVKAQLLRLH---
CANAL    HRGIYLAG-------------PDLLEKVRSQYAKYFEYVK----------
CANGA    SKGIYVVK-------------LDTVDKLVIKYQKYWESMKIQLLRRQ---
ASPNI    DH------------------------QKLMTYQKFHLA------------
RHIO9    QQNKYVIS-------------LDHLTKLREQFSGYFSWVRNAV-------
CRYNE    KRRAVPVPDDALNVAASLLPVANAKEQILQMEHSQLSEVARATMSQLSR-
USTMA    SQAQGAAQNNPGAPSFRPPSTPQMQS------------------------
ENCCU    --------------------------------------------------
ANTLO    EGSKV---------------------------------------------
ARATH*   SVSKSNIM--------------PALKDKVAYYEKQI--IG-FLNMHRPR-
VITVI    QLSKNSIT--------------PNFKEKLIGYEKQI--VS-FINTHRPR-
SOYBN    QIPKNNIL--------------PNFKEKLGSYEKQI--INLLINSNRPR-
SOLLC    RLNKQDIQ--------------LSHKEKLASVEKHI---SFFLSSNRPRS
ORYSJ*   NTQKSNVR--------------KGMQNIFPIFQQYLGQLRLSISKSKARK
HORVU    QISKSSIQ--------------PAMRDKVPQYEKQI--IS-ILSSQRTK-
SORBI    QVNKNSIQ--------------PAFREKIPVYERQI--LS-ILSSQRRK-
SELML    SASRTNLP-------------KEFRLDKLEAFEKQIVS---IMETFKKRK
PHYPA    RVPKDRVP-------------KEFNQDKVDAFEKQIVS---IMETFKKRR
VOLCA    PGAVAGAG------------------------------------------
                   1410      1420      1430      1440      1450
```

```
HUMAN*   --------------------------------------------------
CHICK    --------------------------------------------------
XENTR    --------------------------------------------------
DANRE    --------------------------------------------------
CIOIN    --------------------------------------------------
ANOGA    --------------------------------------------------
TETNG    --------------------------------------------------
DROME    --------------------------------------------------
BRUMA    --------------------------------------------------
CAEEL    --------------------------------------------------
YEAST*   --------------------------------------------------
SCHPO    --------------------------------------------------
CANAL    --------------------------------------------------
CANGA    --------------------------------------------------
ASPNI    --------------------------------------------------
RHIO9    --------------------------------------------------
CRYNE    --------------------------------------------------
USTMA    --------------------------------------------------
ENCCU    --------------------------------------------------
ANTLO    --------------------------------------------------
ARATH*   KPV---QQ------------------------------------------
VITVI    KPVPPLQH------------------------------------------
SOYBN    KGMNSVQA------------------------------------------
SOLLC    KPSSSPLQ------------------------------------------
ORYSJ*   TVAKIGCQ------------------------------------------
HORVU    PVQPQIQQ------------------------------------------
SORBI    PVQAPGQQ------------------------------------------
SELML    LPQQQQQQ------------------------------------------
PHYPA    QPQAPLPAGPTVSGPPPTQQTPAQAVPAQHPDQVSMAQASQQQPSQMLQN
VOLCA    --------------------------------------------------
                   1460      1470      1480      1490      1500
```

MED15

```
HUMAN*   --------------------------------------------------
CHICK    --------------------------------------------------
XENTR    --------------------------------------------------
DANRE    --------------------------------------------------
CIOIN    --------------------------------------------------
ANOGA    --------------------------------------------------
TETNG    --------------------------------------------------
DROME    --------------------------------------------------
BRUMA    --------------------------------------------------
CAEEL    --------------------------------------------------
YEAST*   --------------------------------------------------
SCHPO    --------------------------------------------------
CANAL    --------------------------------------------------
CANGA    --------------------------------------------------
ASPNI    --------------------------------------------------
RHIO9    --------------------------------------------------
CRYNE    ------------------------------QAQAQAQAQAAQAQG-----
USTMA    --------------------------------------------------
ENCCU    --------------------------------------------------
ANTLO    --------------------------------------------------
ARATH*   -------------GQLPQSQMQPMQQP---QSQTVQDQSHDNQTNPQMQS
VITVI    -------------GQSSVSHMHSMQQPQQSQSQLSQGQPHENQMNPQLQP
SOYBN    -------------GHHPPTHMSSMQQP---QSQVTQVHSHENQMNSQLQS
SOLLC    -------------GQLPQS-----------SMQLQQPPSLDGQSNPSMQP
ORYSJ*   --------------------------------------------------
HORVU    -------QFQPPAGQAPDSSILQQQQT---SQNLQQHDSHTNP-------
SORBI    -------IFQQSGGQAPSSNISQQHQT---SQGLQQHDSHTNQMP-----
SELML    ------------------------------QVQLQQQQHQQQGM------
PHYPA    GQPLQQIPSNSQGIQLVQQQQQQQQQQHQQQHQQLQQQQHQQQLQHQQQL
VOLCA    --------------------------------------------------
                                  1510      1520      1530      1540      1550
```

```
HUMAN*   --------------------------------------------------
CHICK    --------------------------------------------------
XENTR    --------------------------------------------------
DANRE    --------------------------------------------------
CIOIN    --------------------------------------------------
ANOGA    --------------------------------------------------
TETNG    --------------------------------------------------
DROME    --------------------------------------------------
BRUMA    --------------------------------------------------
CAEEL    --------------------------------------------------
YEAST*   --------------------------------------------------
SCHPO    --------------------------------------------------
CANAL    --------------------------------------------------
CANGA    --------------------------------------------------
ASPNI    --------------------------------------------------
RHIO9    --------------------------------------------------
CRYNE    --------------------------------------------------
USTMA    --------------------------------------------------
ENCCU    --------------------------------------------------
ANTLO    --------------------------------------------------
ARATH*   MSMQGAGPRA------------------QQSSMTNMQSNVLSSRP--GVS
VITVI    MNLQGSVATM------------------QQNNVPSLQPSSMPSLS--GLP
SOYBN    TNLQGSVPTM------------------QQNNIASMQHNSLS-----GVS
SOLLC    S--QGSMAAM------------------PQNNLTNLQHNTLSGVP--TIS
ORYSJ*   --------------------------------------------------
HORVU    ---QASLSSKSTGL--------------QSSGVTGIQHVPLPPTTNFSAT
SORBI    ---QASIPSMNTGV--------------QTSGAPGIQHVPAPQATNFGVP
SELML    ----------------------------LGGQISSVQHPPVTI-------
PHYPA    LSQMQQEKQVQHQLAGIGSQAAGTMIQPTNNALTSLQQNPGTLQQ--TLA
VOLCA    --------------------------------------------------
                                  1560      1570      1580      1590      1600
```

MED15

```
HUMAN*  --------------------------------------------------
CHICK   --------------------------------------------------
XENTR   --------------------------------------------------
DANRE   --------------------------------------------------
CIOIN   --------------------------------------------------
ANOGA   --------------------------------------------------
TETNG   --------------------------------------------------
DROME   --------------------------------------------------
BRUMA   --------------------------------------------------
CAEEL   --------------------------------------------------
YEAST*  --------------------------------------------------
SCHPO   --------------------------------------------------
CANAL   --------------------------------------------------
CANGA   --------------------------------------------------
ASPNI   --------------------------------------------------
RHIO9   --------------------------------------------------
CRYNE   --------------------------------------------------
USTMA   --------------------------------------------------
ENCCU   --------------------------------------------------
ANTLO   --------------------------------------------------
ARATH*  APQQNIPSSI-PASSLESGQGNTLNNGQQVAM-GSMQQN----------T
VITVI   NAQQNIMNSLQSSANLDSGQGNALTSLQQAAV-GALQQNP-------VSA
SOYBN   TGQQNMMNSMQPGTNLDSVHGNSVNSLQQIPM-NSLQQNP-------VST
SOLLC   NSQQHMINTVQPGSTVDLGQGNSLNSLQQVAT-GSLQQNP-------VNS
ORYSJ*  --------------------------------------------------
HORVU   TQHQAV-------SNLEAAQRGNFNSLQHGLVSGALQQGT-------MNT
SORBI   TTQQNVTNAPQAGSNLENAQGNSFNPVQHGSMGTALQQGSTGPMQGALNA
SELML   --------------------------------------------------
PHYPA   GAPLQTTVVPTSQSVGAAMPTSSTGSMGQSAL-TSLSANPNVPGNASQSG
VOLCA   --------------------------------------------------
                  1610      1620      1630      1640      1650
```

```
HUMAN*  --------------------------------------------------
CHICK   --------------------------------------------------
XENTR   --------------------------------------------------
DANRE   --------------------------------------------------
CIOIN   --------------------------------------------------
ANOGA   ------------------------------------------------GL
TETNG   -------------------------------------------------V
DROME   ------------------------------------------------NV
BRUMA   --------------------------------------------------
CAEEL   --------------------------------------------------
YEAST*  ------------------------------------------------AI
SCHPO   ------------------------------------------------HE
CANAL   --------------------------------------------------
CANGA   ------------------------------------------------QL
ASPNI   ------------------------------------------------QI
RHIO9   --------------------------------------------------
CRYNE   --------------------------------------------------
USTMA   --------------------------------------------------
ENCCU   -----------------------------------------------IPR
ANTLO   --------------------------------------------------
ARATH*  SQLVNNSSASAQSGLSTLQSNVNQPQLSS-------------------SL
VITVI   PQQANINNLSSQNGVNVLQQNINQLQPNS-------------------NM
SOYBN   AQQTNINSLPSQGGANVIQPN--ALQSGS-------------------SA
SOLLC   PQQVNMSSLSSQSGTNPVQANLGSLQQNS-------------------NA
ORYSJ*  --------------------------------------------------
HORVU   QLQTS-GSMLSHNPISTMQASGNSMQPNA-------------------SS
SORBI   QQQSS-SNMISNNAMSTMQTNTNAMQANA-------------------NS
SELML   ------------PGQNTAQPNMMHSL----------------------NP
PHYPA   MNLPQSDGVSSQSGVNTYQQSLNNTLQQSVVGGLQQNGSSVTSLPNQNNA
VOLCA   AHGAGSGLQAPGGGGGSSGGYASPPVLVAHGGGAVSDGGRSPVTASPRHT
                  1660      1670      1680      1690      1700
```

MED15

```
HUMAN*   -QTQPLV-----------SQAQALPGQMLYTQPPLKF-VRAPMV------
CHICK    --QQLKAMQWRVAYANPAKSSETTVRENDHRTYGPRWDANKTTV------
XENTR    -QQQLKILQQVRAQHQAQQQAQQQAQQQAQQQAQQQAQQQAQQV------
DANRE    -QQQLKLQQAMQARMQQQQQQQQQQQQQQQQQQQQQQQQQQQQ-------
CIOIN    -QQQNIQQQQNMQQDSMYINRAGNQQTIYQ--------------------
ANOGA    GPGQMGQLSAGVQGGVGHGGPA----------------------------
TETNG    LQQQAAQQAAAQAQLNAAAAAAATAVPGQLGYHLDPKQTKPP--------
DROME    VGGPAGQQQVGGAGLPPNA-------------------------------
BRUMA    -QQFLQVSLNTGTLQPQPLVDAVNAVLLINSEAQVGYGDRV---------
CAEEL    -QDFLNIAPMSQNQNHMGMNDP----MMNGEHAMLGNGQVPNPYGGHPGY
YEAST*   LRQQQQMANNNGNPGTTSTGNNNNIATQQNMQQSLQQMQHLQQLKMQ---
SCHPO    VNNNNMSIQNALAHISSLRTASINQQQQPQ--------------------
CANAL    -EQINLRRQQVLQQGGANMMSSQQQQQQQQQNMTRSQPQ-----------
CANGA    LQQQQQQQQQGMDPNR----------------------------------
ASPNI    LKETSNRNPEQKGQPSWASPNFQGQPQPFMNAQQFQPGQQQAQFP----M
RHIO9    ------------------GAHSQQQTQAQQQQQQLQQQQV----------
CRYNE    -QNQSQLGQAPGQQGSQGTLNAQQMARFLQQQQQQSQQQQQNQQQALRGS
USTMA    -QQLNQQPTQVPGQQTSVMASNQSQQQQQQQQQQQQQAQMQTQNAAGAPQ
ENCCU    FNSRSAEGNTRGRSNSCNIQNSLHESVSHTMNRESFADNSRNPMPFRRFQ
ANTLO    --------------------------------------------------
ARATH*   LQHQHLKQQQDQQM----QLK--QQFQ----QRQMQQQLQ----------
VITVI    LQHQ-HLKQQQEQMMQTQQLK--QQMQQRQMQQQLMQKQQLMQQQQQQQQ
SOYBN    LQHQ-LKHQQEQQMLQSQQLK--QQYQ----RQQLMQRQLL---------
SOLLC    LQQS-LPKQHEQQMLQNQQLR--QQYHHRHMQQQLFQRQQI---------
ORYSJ*   -SQNCSENSHIVNLGSNTAPFTCDARQQKQQEQVISAKTSRMEQAIMTRT
HORVU    LQQQ----QQDHHLMQSEQMK--RQMYQYQQKQQMLQQQLP---------
SORBI    LQQL-KQQHQEHQMMQSQQMKQRQQMMHQIQQKQMLQQQLP---------
SELML    LQQQ---QQQQQHVFKQDLLQQQQMKRIQLA-------------------
PHYPA    LQQQILKQQQEQH--QQQLLQAQHQRMIQQDLQQRNLQQQQQQMLQQQQQ
VOLCA    LQQQ------------------------SPYNAHISPHLQQNSLL-----
                  1710      1720      1730      1740      1750
```

```
HUMAN*   ------------------------VQQPP---------------------
CHICK    ------------------------PAYHR---------------------
XENTR    ------------------------AQQQA---------------------
DANRE    -------------------------QQQQ---------------------
CIOIN    ------------------------TNQIQ---------------------
ANOGA    ------------------------MQQQQ---------------------
TETNG    ------------------------KPGRE---------------------
DROME    ------------------------VQQGG---------------------
BRUMA    ------------------------QTNTG---------------------
CAEEL    GHQQYMGPP---------------PPHMQ---------------------
YEAST*   ------------------------QQQQQ---------------------
SCHPO    ------------------------PQSQQ---------------------
CANAL    ------------------------SQQQQ---------------------
CANGA    ------------------------AQNSQ---------------------
ASPNI    PHMR--------------------QITPQ---------------------
RHIO9    ------------------------QQQVQ---------------------
CRYNE    QANPIELVTPTMTNTQLPHFPTPSQTSAT---------------------
USTMA    QPSLAGFNPTPPPILSAENLAKAGMSQEQ---------------------
ENCCU    SEVNSYGAVNGKDFRNATDQRHFYMQREE---------------------
ANTLO    ------------------------KEKPN---------------------
ARATH*   ------------------------ARQQQ---------------------
VITVI    QQQ---------------------QQQQQ---------------------
SOYBN    ------------------------QQQQQ---------------------
SOLLC    ------------------------IQQQQ---------------------
ORYSJ*   PT----------------------PQQESHGCHLLGVPSSCFSPKALQPS
HORVU    ------------------------VQQQQ---------------------
SORBI    ------------------------IQQLQ---------------------
SELML    ------------------------QQQQQ---------------------
PHYPA    QRQI--------------------QQNHQ---------------------
VOLCA    ------------------------AQSQQ---------------------
                  1760      1770      1780      1790      1800
```

MED15

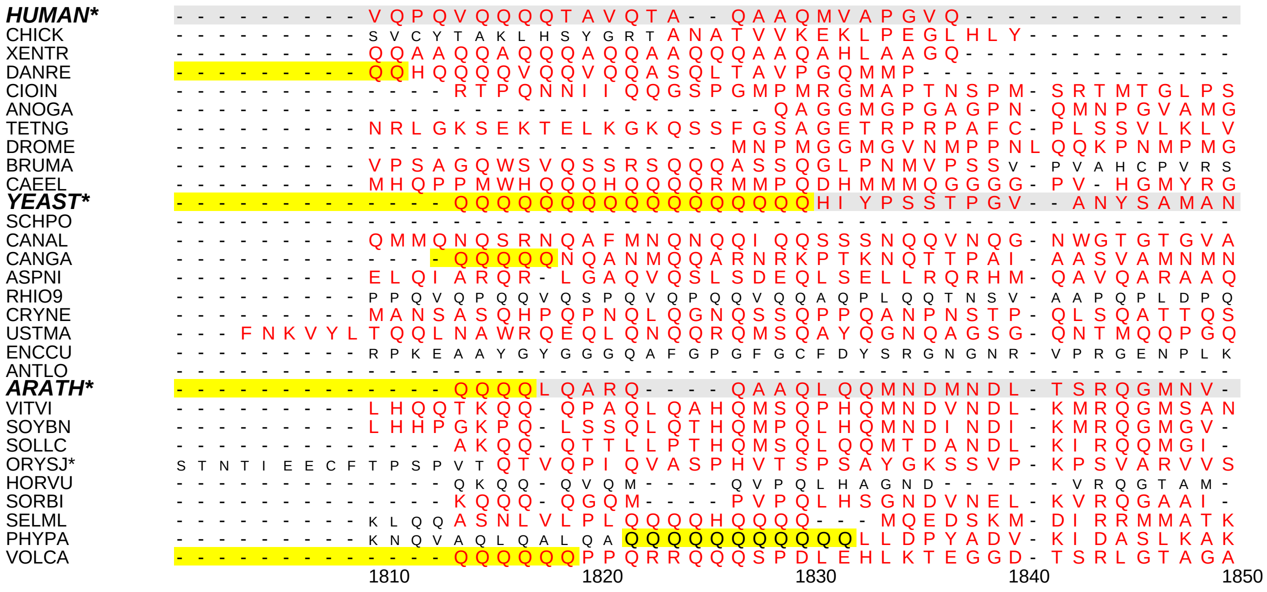
HUMAN*
CHICK
XENTR
DANRE
CIOIN
ANOGA
TETNG
DROME
BRUMA
CAEEL
YEAST*
SCHPO
CANAL
CANGA
ASPNI
RHIO9
CRYNE
USTMA
ENCCU
ANTLO
ARATH*
VITVI
SOYBN
SOLLC
ORYSJ*
HORVU
SORBI
SELML
PHYPA
VOLCA
1810
1820
1830
1840
1850

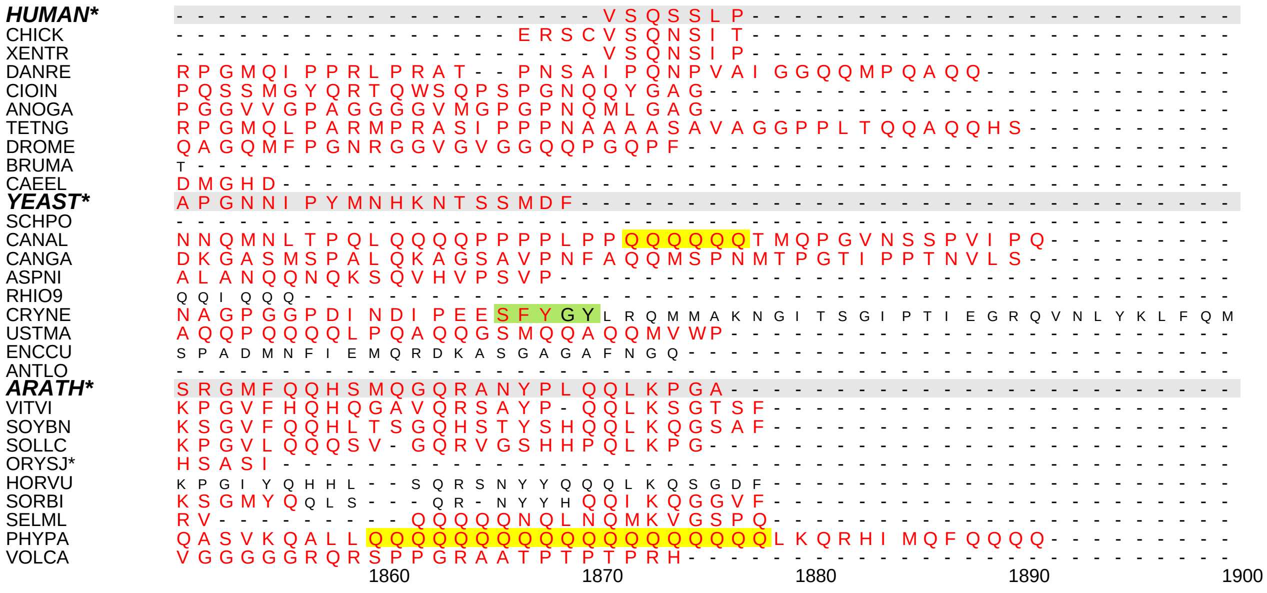
HUMAN*
CHICK
XENTR
DANRE
CIOIN
ANOGA
TETNG
DROME
BRUMA
CAEEL
YEAST*
SCHPO
CANAL
CANGA
ASPNI
RHIO9
CRYNE
USTMA
ENCCU
ANTLO
ARATH*
VITVI
SOYBN
SOLLC
ORYSJ*
HORVU
SORBI
SELML
PHYPA
VOLCA
1860
1870
1880
1890
1900

MED15

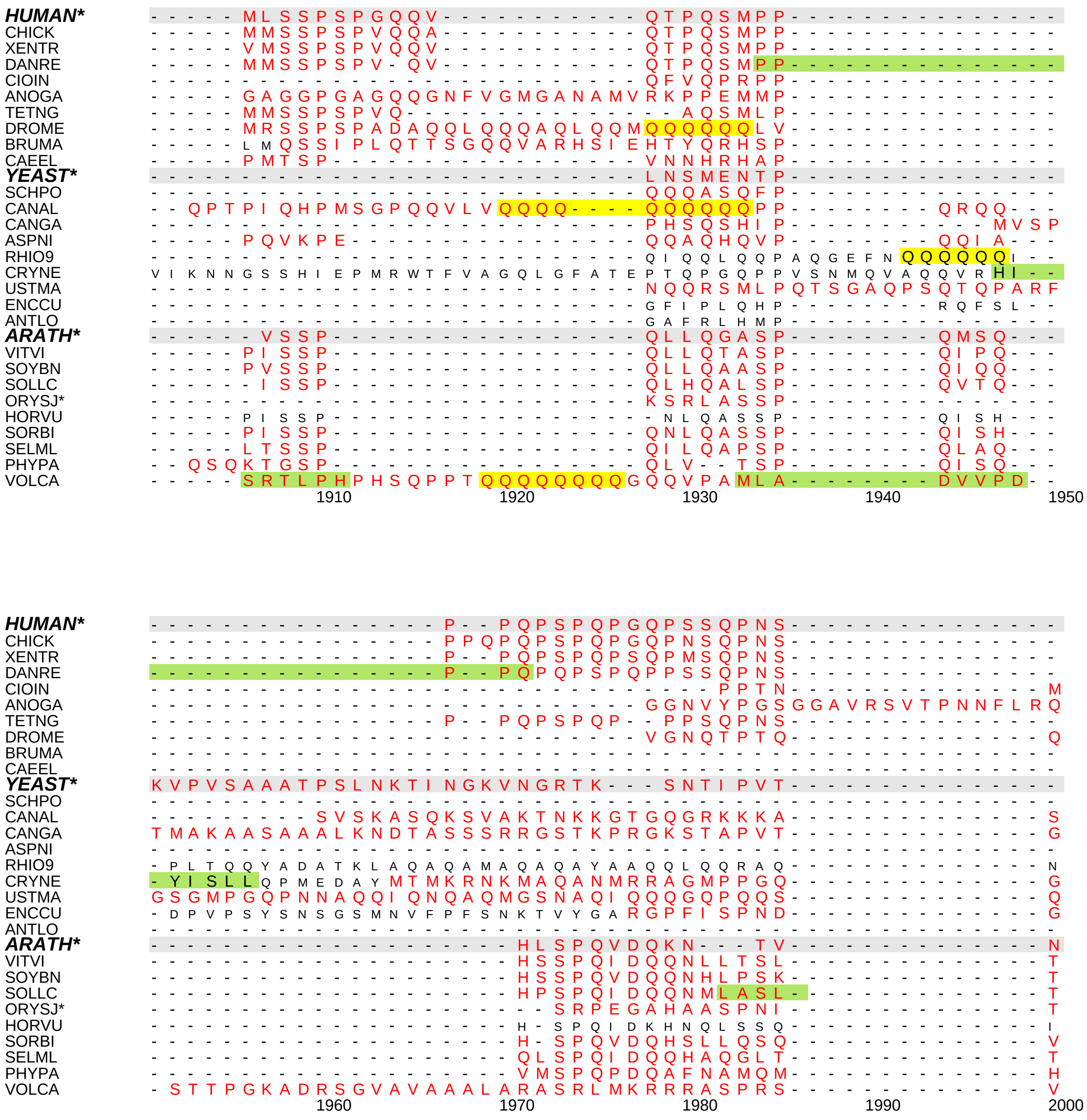
HUMAN*
CHICK
XENTR
DANRE
CIOIN
ANOGA
TETNG
DROME
BRUMA
CAEEL
YEAST*
SCHPO
CANAL
CANGA
ASPNI
RHIO9
CRYNE
USTMA
ENCCU
ANTLO
ARATH*
VITVI
SOYBN
SOLLC
ORYSJ*
HORVU
SORBI
SELML
PHYPA
VOLCA
1910
1920
1930
1940
1950
1960
1970
1980
1990
2000

MED15

```
HUMAN*   -----------NVSSGPAPSPSSFLPSP-----SPQPSQ--------SPV
CHICK    -----------NVSSGPAPSPSSFLPSP-----SPQPSQ--------SPA
XENTR    -----------NVSSGPAPSPSSFMPSP-----SPQPSQ--------SPA
DANRE    ------------VSSGPTPSPGGFQPSP-----SPQPSQ--------SPA
CIOIN    QPILKMPDSTGQPHSNTIPSLSPNHPSPQVHNTSPGLRT-----------
ANOGA    SPSPSVPSPVGPGAHGPPSHPGQMIPSP-----ALIPSP--------NPH
TETNG    ------------ASSGPTPSPGGFQPSP-----SPQPSQ--------SPS
DROME    PPTPQMPTP-------------NMIPSP-----ALVPQS--------SPQ
BRUMA    ---------------------------------YPNPNA-----------
CAEEL    ---------------------------------YPNPAA-----------
YEAST*   ----------------SIPSTNKKLSISNAASQQP------------TPR
SCHPO    ----------------QAPSVSSNVRPINGSIPNAQPSV---------PG
CANAL    ISAGTAPTPGALPATTPGTLANAIKTPHN----IPTPQI--------PPQ
CANGA    KKTSNAPTPQVVPATVPSTTNLSAAGTPNIRNKSA------------TPL
ASPNI    ---------QNQAIKTQAVSPSKAAKGP-----APKQTP--------PTA
RHIO9    PSATSTPNMGAIGQIGIKRGSIDLKLPPNKKQKAAGSTP--------QQT
CRYNE    STPGQAPGQVRPPMNVAGPSVTPTLASQNQNQGQPLSEQQRRFLEAAKNA
USTMA    AQSSQMPSSSAQPQQMSAQAISATSASGAPQGQPGQPGQQGQQGQIAGRP
ENCCU    RPGHIYPPRMPNVQNFPMPSSSDAIGSF-----SPNPHDPVTNMFSMGPQ
ANTLO    ------------------PSEDSIHTHRDVLQFSPFQCT-----------
ARATH*   -KMGTPLQPANSPFVVPSPSSTPLAPSP-----MQVDSE--------KPG
VITVI    -KAGTPLQSANSPFVVPSPST-PLAPSP-----MPGESE--------KLN
SOYBN    -KVTTPLQSSNSPFVGPTPSP-PLAPSP-----MPGESE--------KSI
SOLLC    -KAGTPLQSASSPFVVPSPST-PLAPSP-----MPGDSE--------KVG
ORYSJ*   SVESTLPTPIAKPGTVRAASPCTPVKST-----SQSQLSKPAVTEVDSCR
HORVU    -KTGTPLHSANSPFV-PSPSP-SIAPSP-----IPVDSD--------KPH
SORBI    -KTGTPLHSANSPFV-PSPSP-PVAPSP-----MPVDSD--------KPL
SELML    VKVGTPLQAATPPFMTPSPLL-PVASPPI----EDAEHK--------AAT
PHYPA    -KTGTPPQS----FRIPSPSALPTTPTQ-----DDADHK--------GLT
VOLCA    VK--------------HAPGVSLAEPDAEAGQ-GPGPGP--------GPS
                   2010      2020      2030      2040      2050
```

```
HUMAN*   TA--------------RTPQNF--SVPSPGPLNTPVNPSSVM-SPA----
CHICK    AA--------------RTPQNF--SVPSPGPLNTPGNPNSVM-SPA----
XENTR    SA--------------RTPQNF--SVPSPGPLNTPGNPNSVM-SPA----
DANRE    SS--------------RTPQSYPLQVPSPGPLNTPGNPSSVM-SPA----
CIOIN    -------------------PGSVQAAPSPGTLAINTIPSP---TPL----
ANOGA    MG--------------GVAQRSTIGQSPGGSLNTPGQPGGAVPSPL----
TETNG    NA--------------RPVSNY--SVPSPGPLNTPGNPSSVM-SPA----
DROME    MM------------QMQNSQRNIRQQSPSASINTPGQVTGN--SPF----
BRUMA    -----------GLRSGTQSVVL----HHGLSRVQPAATVAAL-PPF----
CAEEL    ------MRNNMRMPNGPGPIGRDRNSMSGSSMSGPSSGAPSM-NPM----
YEAST*   SASNTAKSTPNTNPSPLKTQTKNGT-------PNPNNMKTVQ-SPM----
SCHPO    QAQPAAKANSVGNTSFPVDSKLA---FQSLDVSQPDLQAKQKIASQ----
CANAL    TQ---------SNKNTPSAQSPAYPVKATPSSTTPGTANHSH-NPS----
CANGA    TAGLSPKSTIRSNSNTALASAKTPS-PMTVSIPQPGNSSV----------
ASPNI    KRKLQNEEMAIGQASPIQNSNQPAI-SQTLPVTAPSRPAM----PF----
RHIO9    TIDLTQTPPPPQHAEPASTETMANS-NTSIANANPGGMTASQ-RQT----
CRYNE    GAGNVGTGVGLGEAWQGQAQQNAGQGSQSQSIQPPTQPQT-Q-QPA----
USTMA    SNMSQARFIAVSPEQLEQARAAMQKIDQELATQRPR-------LPI----
ENCCU    SK------------AGAGGAWMGDPPSFDRHYPNPGQGAMKS-SPLFFNQ
ANTLO    --------------------------AEGAKRSSSASTSVFRRNPK----
ARATH*   S----SSLS-MGNIARQQATGMQGV-VQSLAIGTPGISAS----PL----
VITVI    SG--ISLITNAGNIGHHQTTGALAP-PPSLAIGTPGISAS----PL----
SOYBN    PC--VSSISNAANIGLQQTGGAVAP-AQSLAIGTPGISAS----PL----
SOLLC    AG--LGSHTTAGNIMHQQATVASAP-AQSLAIGTPGISAS----PL----
ORYSJ*   ACVTSKLKSPVGKPETAGAASPCASVKSTVSLDVDSVTEFLQ-HRV----
HORVU    SI--ISSLSNTGQTGHQQT--SLAPQTKSMSLNTPGISAS----PL----
SORBI    SN--LSSLTSAGQ-AHQQT--SLAPQTQSIAVNTPGISAS----PL----
SELML    SA--VSSITSSQPIGYNQS-------TATPVIGTPGISAS----PF----
PHYPA    PA---PSSGTPGTVQAFQTAIRNTP-GMSGSINTPGMSVS----PL----
VOLCA    SGSQRSHQPSGGGLSRTRSAAMASP-AGSLQRRTTSLSGIGG-GPV----
                   2060      2070      2080      2090      2100
```

MED15

```
HUMAN*  -----------GSSQAEEQQYLDKLK--------------QLSKYIEPLR
CHICK   -----------SSSQSEEQQYLEKLK--------------QLSKYIEPLR
XENTR   -----------STNQSEEQQYLDKLK--------------QLSKYIEPLR
DANRE   -----------GASQSEDQLYMDKLR--------------QLSKYIEPLR
CIOIN   ----------NNSQVEDDKEYMEKLK--------------QLQKYIEPLS
ANOGA   -------------NPQDEQLYREKYR--------------ALTKYIEPLK
TETNG   -----------GPTSLEDQQYMEKLK--------------QLSKYIEPLR
DROME   -------------NPQEEALYREKYK--------------QLTKYIEPLK
BRUMA   --------------------------------------------------
CAEEL   ---------------------------------------------GTPNQ
YEAST*  -----------GAQPSYNSAII----------------------------
SCHPO   ----------------------------------------VMKHGLKPED
CANAL   --------------------------------------------YVYPTP
CANGA   --------------------------------------------------
ASPNI   ----------TR----------------------------EQLAAMTPQQ
RHIO9   ---QSQMIYQAAISSGIPPQIVKLLPPRALQCSWLLQQAAQNRINVTPQQ
CRYNE   ----------GHQTATPTAQRLELSAL-------------KILEFIRMQE
USTMA   ------IQNIADDERDQILDQVQKLAPIQATVTALLPAFYAMNGNLEPAK
ENCCU   QLQHQPYFHHPRNSGQQNSMVDRKKPLSAPENPSRWMFNHSGEDFRVPYD
ANTLO   ----------------------EDMLKTSYSYACGKTELQSTACSSTPVT
ARATH*  ----------LQEFTSPDGNILNSSTITSG----------KPSATELPIE
VITVI   ----------LAEFTSLDGNHGNASTMVSG----------KSSVTEQPLE
SOYBN   ----------LAEFSCPDGAHGNALAATSG----------KSTVTEQPLE
SOLLC   ----------LAEFTPLDGTHANVSAAVPG----------RS-SVEQPLD
ORYSJ*  --VAPTVANGGSSNQAIHTLVSAAPPKAAHQADDQVQNGAEEMEAKKPFS
HORVU   ----------LAEFTSVDGSQANMPTQAPT----------KSNAAERPMD
SORBI   ----------LAEFTSADGSQVNLPTQVPT----------KS-AAERPLD
SELML   -----------LEFSPSPAQVAGSQDPAPGTP--------FGQKGLDPLE
PHYPA   ----------MENTLSPSPAQLLMVHDAAQAFSPGTPLMDKTNSTEPPLE
VOLCA   ----------RPDISAATANATVTATATANATATTTSARLFSESEGEEVQ
                 2110      2120      2130      2140      2150
```

```
HUMAN*  RM------------------------------------INKIDKNEDRKK
CHICK   RM------------------------------------INKIDKNEDRKK
XENTR   RM------------------------------------INKIDKNEERKK
DANRE   RM------------------------------------INKIDKNEDRKK
CIOIN   RM------------------------------------ISKFNEDEEKYN
ANOGA   RM------------------------------------IAKMENDDIDKI
TETNG   RM------------------------------------INKIDKNEDRKK
DROME   RM------------------------------------LAKISNDGTNVE
BRUMA   --------------------------------------------------
CAEEL   KM------------------------------------------------
YEAST*  --------------------------------------ENAFRKEELLLK
SCHPO   --------------------------------------LKLPPSKKKKIE
CANAL   EID-----------------------------------IFAMSSNDSKSA
CANGA   -----------------------------------------FKKEEEYLS
ASPNI   RMQIEAHLRRQQ--------------------------GQARGTISRAAA
RHIO9   QQQ-----------------------------------IRNMLNERVEAA
CRYNE   SLI-----------------------------------RNGLEKQPNTDV
USTMA   RLKIMAFMYKDQFDLLPKKQCILRLADLDKLKMQMSRCISFVRSSNPELA
ENCCU   ELPHRYESTKKS--------------------------MGSFDSSKKDER
ANTLO   KYTEEMFYRPQC--------------------------ANMLTKPAAGAR
ARATH*  RL------------------------------------IRAVKSISPQAL
VITVI   RL------------------------------------IKVVKLMSPKAL
SOYBN   RL------------------------------------INAVKSISRKAL
SOLLC   RL------------------------------------MRAVKNMSDKAL
ORYSJ*  RL------------------------------------IETLLSSSPEAL
HORVU   RL------------------------------------LKALRTTQRESL
SORBI   RL------------------------------------LKALRTTQRESL
SELML   RL------------------------------------LKIVSTMSPKAL
PHYPA   RL------------------------------------KRKVDSMPREIL
VOLCA   RRR-----------------------------------RDGEDGEEGHEV
                 2160      2170      2180      2190      2200
```

MED15

```
HUMAN*   DL----SKM-KSLLDILTDPSKRC---PLKT-------------------
CHICK    DL----SKM-KSLLDILTDPSKRC---PLKT-------------------
XENTR    DL----SKM-KSLLDILTDPNKRC---PLKT-------------------
DANRE    DL----SKM-KSLLNILTDPNTRC---PLKT-------------------
CIOIN    NDL---CKM-KTLRDTLMNPNKRL---LMST-------------------
ANOGA    ------AKM-KRLLEILSNPSVRI---PLET-------------------
TETNG    DL----SKM-KSLLNILTDPTTRC---PLKT-------------------
DROME    KM----TKM-SKLLEILCNPTQRV---PLET-------------------
BRUMA    ------------------SEANRA---PATTDCSRVEDLYV---------
CAEEL    ----------------------GT---PGSMGGMSGLDDLN---------
YEAST*   DLEIRKLEI-SSRFKHRQEIFKDS---PMDLFMSTLGDCLG---------
SCHPO    NLSTVQKPK-DSVVNTPDVIMSSVDEIPSSVSPGTIAKEEG---------
CANAL    KR--------RELINID----------PEKFFYASLANLLD---------
CANGA    KLQLRKEEI-RFRQKQRLDILSSS---PVDLFLTTTVADCLG---------
ASPNI    EKAW--SNLPESIRQLYNELGKNA---PEDKPVAVTPEQRA--A------
RHIO9    KQQLA-----RQAMEAQQQQQSQA---PPPVSTATMVTPSP---------
CRYNE    NPDIYRNEL-RTILPIAKEAESRL---PIYLLMISDGGSVEPQSVLTIIN
USTMA    QRIVTSLGE-QSRAAQQNGAAPSA---TGSNNSASQNVNQG---------
ENCCU    NFSTNSNEY-KGCINTSSWHSGGF---RSFFPESNPPDSID---------
ANTLO    NVSSIGSE--TSGFDLSEKLADVA---IGESEKTVVRNKLA---------
ARATH*   SSAV--SDI-GSVVSMVDRIAGSA---PGNGSRASVGEDLV--A------
VITVI    SASV--SDI-GSVVSMIDRIAGSA---PGNGSRAAVGEDLV--A------
SOYBN    SAAV--MDI-GSVVSMNDRIAGSA---PGNGSRAAVGEDLV--S------
SOLLC    QSSV--QDI-YSVVSMNDRIAGSA---PGNGSRAAVGEDLV--A------
ORYSJ*   LHSS--NSM-RLAIWEADRIPAPS---PLPYRPRNG--------------
HORVU    NAAV--SDI-RSVVSMMDRIAGSA---PGNGSRAAVGEDLV--A------
SORBI    SAAV--SDI-GSVVSMIDRIAGSA---PGNGSRAAVGEDLV--A------
SELML    SAGV--REM-RSVVNLSDKLAASA---PGPGCRAAVGEDLA--A------
PHYPA    KKAV--NEM-NDMVSCGDKLGASA---PGTGSRAAIGEDLS--A------
VOLCA    GREVAIAAGDSDDEDGSSDGADSS---PDTATVAPSGGDAD---------
                  2210      2220      2230      2240      2250
```

```
HUMAN*   ----------------------------------LQKCEIALEKLK-NDM
CHICK    ----------------------------------LQKCEIALEKLK-NDM
XENTR    ----------------------------------LQKCEIALEKLK-NDM
DANRE    ----------------------------------LQKCEIALEKLK-NDM
CIOIN    ----------------------------------LDKCEKVLQNWL---D
ANOGA    ----------------------------------LHKCEAALTSQLGSIR
TETNG    ----------------------------------LQKCEIALEKLK-NDM
DROME    ----------------------------------LLKCEKALEKMD----
BRUMA    -----------------------------------------------MDD
CAEEL    -----------------------------------------------YDD
YEAST*   ----------------------------------IKDEEMLTSCTI----
SCHPO    ----------------------------------MAKAREEAIANP----
CANAL    --------------------------------------------------
CANGA    ----------------------------------INDEEIELINKI----
ASPNI    ----------------------------------MNQQLRDCTDYL-G--
RHIO9    ---------------------------------PMVKAQTKTPDAV-LQQ
CRYNE    MCTTPSYAALLAERNRFIFNLADFPRLRAGLSQFLSKAQATYRTMA-EKP
USTMA    ----------------------------------TAANAMQAGSSAPLPA
ENCCU    -----------------------------------SQKRIDSSLWF-RDK
ANTLO    --------------------------------------------------
ARATH*   ----------------------------------MTKCRLQARNFM-TQE
VITVI    ----------------------------------MTKCRLQARNFI-TLD
SOYBN    ----------------------------------MTNCRLQARNFI-AQD
SOLLC    ----------------------------------MTKCRLQARNYF-TQD
ORYSJ*   --------------------------------------------------
HORVU    ----------------------------------MTKCRLQARNFI-TND
SORBI    ----------------------------------MTKCRLQARNFI-THD
SELML    ----------------------------------LTKSRQQSRALASTQE
PHYPA    ----------------------------------KTRSTMQARALS-HDN
VOLCA    ------------------------------EAYGRVGSSRRSRTAI-SGG
                  2260      2270      2280      2290      2300
```

MED15

```
HUMAN*  AVPTPPPPPVPPTKQQY-------LCQPLLDAVLA---------------
CHICK   AVPTPPPPPVPPTKQQY-------LCQPLLDAVLA---------------
XENTR   AVPTPPPPTVPSTKQQY-------LCQPLLDAVLA---------------
DANRE   AVPTPPPPPVPCTKKQY-------LCQPILDAVLA---------------
CIOIN   QKPSQPPLLQPRPDQH--------MCQPILNVIAS---------------
ANOGA   ETP---------------------TNNPLVEAVSS---------------
TETNG   AVPTPPP---VATKQQY-------LCQPLLDAVMT---------------
DROME   --------LISYSGQQFGK-----SSNPLLEVINT---------------
BRUMA   FLPTP------------------------IEAVSN---------------
CAEEL   FLPNPTP----------------------IDALQP---------------
YEAST*  -------------------------PKAVVDHING---------------
SCHPO   -------------------------LKYAIDAFVAVDH------------
CANAL   --------------------------------------------------
CANGA   -------------------------PETTADNINN---------------
ASPNI   ------------------------RMDALVQFIAKVPGQEKNLRSLLGMR
RHIO9   QPPVVKA-------------------------------------------
CRYNE   VGQA--------------------KLKSMVSAVQM---------------
USTMA   APTAAAAAEVATLPAQGAEAGAGVNADGTFDAVAAMHGIKRGLRPENLKL
ENCCU   FRYGRAP-----------------EAEDPMNTIEGNHDVYKKEKSPDHHR
ANTLO   --------------------------------------------------
ARATH*  GMMATK------------------KMKRHTTAMPL---------------
VITVI   GAAGTR------------------KMRRYTSAMPL---------------
SOYBN   GSNGIK------------------RMKRYTSAIPL---------------
SOLLC   GPTGTK------------------KMKRYTTS------------------
ORYSJ*  ------------------------KMKRDFDHVTS---------------
HORVU   GSGASK------------------KMKRDTSAMPL---------------
SORBI   GSGASK------------------KMKRDTSAMPL---------------
SELML   GPAAAK------------------KVKRQLDSIAL---------------
PHYPA   CNVS--------------------KKRRRVDSIAL---------------
VOLCA   VGAAAAARGGPDAPDAPVAVGASADTQMIIDPITG---------------
                  2310      2320      2330      2340      2350
```

```
HUMAN*  -------------NIRSP--VFNHSLYRTFVPAMTAIHGPPITAPVVCTR
CHICK   -------------NIRSP--VFNHSLYRTFMPAMTAIHGPPITAPVISSR
XENTR   -------------NIRSP--VFNHSLYRTFMPAMTAIHGQPITSQLVVPR
DANRE   -------------NIRSP--VFNHSLYRTFAPAMTAIHGPQITGPSIPSR
CIOIN   -------------ATKSP--LLNHTLKRTFGPAMKTLCGNYTIPEFVPNK
ANOGA   -------------SLQAA--TGNHTLQRTFRPCLEALFGPDIKNLPPPAK
TETNG   -------------NIRSP--VFNHSLYRTFAPAMSAIHGPPISCPSVCGR
DROME   -------------TLQSP--VANHTLYRTFRPTLELLFGTDITA-PVPAK
BRUMA   --------------------------------------------------
CAEEL   --------------------------------------------------
YEAST*  --------------------------------------------------
SCHPO   --------------------------------------------------
CANAL   --------------------------------------------------
CANGA   --------------------------------------------------
ASPNI   IQLMRQ-------FKPSPDWALNDQFTITPEYLIGTTNYIKKLFHHMIAR
RHIO9   --------------------------------------------------
CRYNE   --------------------------------------------------
USTMA   PPNKRAKNVTGPGKAMSPSSGANESPRVAPSPAQPEDTPTPAGSTPAGKG
ENCCU   SDFVAQQQTGLRETSQNECGVSNPETFLNFPSEVNAFL------------
ANTLO   --------------------------------------------------
ARATH*  -------------SVASLGGSVGDNYKQFAG-----------------SE
VITVI   -------------NVVSSAGSVNDSFKQLAG-----------------SE
SOYBN   -------------NVVSSPGSMNDSIKQLTA-----------------SE
SOLLC   -------------NVVSSSSSLNDSFWQVNY-----------------SE
ORYSJ*  ------------RPISSPLRSMDESCMTYECVAFE---------------
HORVU   -------------N-VSSTGSVDDSFKQTFS-----------------AD
SORBI   -------------N-VSSAGSVNDSLKQSYT-----------------VG
SELML   -------------TLISSDGSVVNSLHK--------------------GD
PHYPA   -------------NMISSDGTISNTLHRS-------------------CY
VOLCA   -------------LPRRRAMTQEERMVRNREAAAKSR-----------AR
                  2360      2370      2380      2390      2400
```

MED15

HUMAN*	KRRLEDDERQSI----------PSVLQGEVARLDPKF-------------
CHICK	KRKYEEDDRQTI----------PNVLQGEVARLNPKF-------------
XENTR	KRKFEEDERQSI----------PNVLQGEVAKLHSKF-------------
DANRE	KRKLEDDERQTI----------PNILQGEVARLNSKF-------------
CIOIN	RQKLQEAVLELRANKAKGKL--SDNVKREIANLDSKF-------------
ANOGA	QSRLALDDTGAAVGTGGGEI--PHILQGEIARLDQKF-------------
TETNG	KRKQEEEEERQSV---------PNILQGEVARLDLKF-------------
DROME	RPRVEEKSTSFEQEV-------PHVLQGEIARLDTKF-------------
BRUMA	----------NTSSQMEGLL--PEAVRQELLAFGDRI-------------
CAEEL	TLHVGQNSMNAGPPVQRSNL--NETARKELQILDARF-------------
YEAST*	SGKRKPTKAAQRARDQDSI---DISIKDNKLVMKSKF-------------
SCHPO	--------------------------EEEVSAIKSSQ-------------
CANAL	----LDDSIVNNHDIMKTSPRLSNGKPAATSPLSPKTGGEWTSEVKALAI
CANGA	TGKKKLTKAAQKLRDKEILNVSIQVGEKDKLIMSSKA-------------
ASPNI	VNQQQNQGPGQRVGMSQGPN--PQQTNQNMPPLNASN---LQQLQQQEEA
RHIO9	----EPDYKNNESLQQAAQM--MHLLQQQQQQQQQRQQPRQPTVAAPQPS
CRYNE	-----AKGLIAQKDREKQAQTTPNAAPANVAAAGSGLGLNLGSTQGATGI
USTMA	SKKIEKTGKGSKATAKKGKGGAAGTPTSSAAEPDKKFKTSADIMAEVKRE
ENCCU	----------------------KDHEMEKIYLCNQEL---LDLKTRLKEG
ANTLO	------------------------VFQKELLAVEEDM-------------
ARATH*	TSDLESTATSDG-KKARTET--EHALLEEIKEINQRL---IDTVVEISDD
VITVI	TSDLESTATSSA-KRPRIEV--NHALLEEIREINQRL---IDTVVDISHE
SOYBN	TSDLESTATSSV-KKPKIEV--NHALLEEIREINHRL---IDTVVDISNE
SOLLC	TPELESTATSNA-KRPKIEV--NIALVEEIQKINRQL---IDTVVEISDE
ORYSJ*	----DESSGEYNAKRQKTQVNANDALVDEIKTINNKL---VDTVMNF---
HORVU	TPDVQSTATSRANKRQKVEV--NHALLEEIQAINQEL---IDTELNVCEE
SORBI	TPELQSTATSRV-KWQRAEV--NHALMEEIQEINQQL---IDTELHVSED
SELML	EADLESSVTSGW-KSQKLEI--SYVLEQEIRDTNDHL---IDTVVDISED
PHYPA	NTDAENHMVSHI-NSPLITA----SLEEEIRDINSRL---IDTVVEVSPE
VOLCA	RHQYQQNLEQHIRALQQHSAELRKLLEAHAVPLPPHL---LEQQVRLGDG
	2410 2420 2430 2440 2450

HUMAN*	LVNLDP------SHCSNNGTV-----------------------------
CHICK	LVNLDP------SHCSNNGTV-----------------------------
XENTR	LVNLDP------SQCSNNGTV-----------------------------
DANRE	LVNLDP------SFCSNNGMV-----------------------------
CIOIN	NIVVDE------VGNDMGGNV-----------------------------
ANOGA	KVSLDQ------CAISGTRTI-----------------------------
TETNG	LVNLDP------SQCSTNGTV-----------------------------
DROME	KVKLDT------TSQINNKAI-----------------------------
BRUMA	--LADS---NIEQMTDSHSVIVKFSLTSHNV-------------------
CAEEL	--EIDP----NHQRHDANHII-----------------------------
YEAST*	---NKSNRSYSIALSNVAAIF-----------------------------
SCHPO	--
CANAL	TTSFKQ-------------------------------------V------
CANGA	---PDKVMDYSISAMSLAAVF-----------------------------
ASPNI	LQRARR----ASSQTAPSGAIPAAPFGAPSPQGVPH--------------
RHIO9	PVVQRP--
CRYNE	GLDVQQ-----MVQQAHNHAA----------------------S------
USTMA	LAEAERKRREEGSAGDASGAAAKVDMANVKAEQEAEERRKKDQELAGRDP
ENCCU	ISVINK--
ANTLO	--
ARATH*	EDAADP-SEVAISSIGCEGTTVRFSFIAVSLS-----------P------
VITVI	D--VDPAAAAAAAAEGGEGTIVKCSFSAVALS-----------P------
SOYBN	D--VDP-TAAVAAAEGAEGIIVKCSFIAVALS-----------P------
SOLLC	G--VDP-SALAAATEGGEGTTVKCSFTAVALS-----------P------
ORYSJ*	---ADENGTDEIIYQNGGGMLIKLSYISMSLS-----------P------
HORVU	D--ADS----FAAA--SEGTVIKCTYTPVAVS-----------P------
SORBI	D--AES----FTASEGGEGTVIRCTFTAVAVS-----------P------
SELML	G--TEE-----AASEGKEGLVLTFSYNGLEIP-----------E------
PHYPA	A--TEE-----NAAQGGNGIVLSCCYKGNALS-----------P------
VOLCA	LPPPPPRTSPPRSRKGIGGRPRKYPLNPDGSKPSRKRRRTSTG-------
	2460 2470 2480 2490 2500

MED15

```
HUMAN*  -----------------------------------------------HLI
CHICK   -----------------------------------------------HLI
XENTR   -----------------------------------------------YLI
DANRE   -----------------------------------------------HLI
CIOIN   -----------------------------------------------QLF
ANOGA   -----------------------------------------------KLI
TETNG   -----------------------------------------------HLI
DROME   -----------------------------------------------RLI
BRUMA   --------------------------------------------------
CAEEL   ------------------------------------------------VV
YEAST*  --------------------------------------------------
SCHPO   --------------------------------------------------
CANAL   -----------------------------------------------DLI
CANGA   --------------------------------------------------
ASPNI   --------------------------------------------------
RHIO9   --------------------------------------------------
CRYNE   -----------------------------------------------QLQ
USTMA   AGLVESTWHELLATGKVSNVPGGANASVGAQASGPSASNGSDPKALQSLM
ENCCU   --------------------------------------------------
ANTLO   -------------------------------------------ARHAKLG
ARATH*  -----------------------------------------------ALK
VITVI   -----------------------------------------------NLK
SOYBN   -----------------------------------------------SLK
SOLLC   -----------------------------------------------NLK
ORYSJ*  -----------------------------------------------SLK
HORVU   -----------------------------------------------SLK
SORBI   -----------------------------------------------SLK
SELML   -----------------------------------------------TI-
PHYPA   -----------------------------------------------NIS
VOLCA   --------------------------------------------------
                  2510      2520      2530      2540      2550
```

```
HUMAN*  CKLDD-KDLPSV-PPL-ELSVPA---------------------------
CHICK   CKLDD-KNLPNV-PPL-QLSVPA---------------------------
XENTR   CKLDD-KNLPSV-PPL-QLSVPA---------------------------
DANRE   CKLDD-KNLPSV-PPL-QLSIPA---------------------------
CIOIN   CYLND-PKLPGI-PPI-QISVPQ---------------------------
ANOGA   CWLDD-KNLPCV-PPV-AVTIPE---------------------------
TETNG   CKLDD-KNLPSV-PPL-QLRIPA---------------------------
DROME   CCLDD-KRLPSV-PPV-SVSVPE---------------------------
BRUMA   -------------PPL-QLIIPK---------------------------
CAEEL   CKIRN-QQF----PPL-RLVVPT---------------------------
YEAST*  ------KGIGGNFKDL-STLVHS---------------------------
SCHPO   ------TPSSILKTPQ-SFFIPP---------------------------
CANAL   RELANDDFISS--SPM--LAIQE---------------------------
CANGA   ------KNLSST-GSLNNIALSG---------------------------
ASPNI   ------AYGPGSIPPQ-ELKLPPPKRR-----------------------
RHIO9   ---QPPQPPPAQILPL-KTTVPS---------------------------
CRYNE   SSQNQQQQQPPA-QPQ-TLLPPP---------------------------
USTMA   SMLGQDSMLSGL--PINGLNVPSKGLMPDESSGGAAVPSFMEGALDSLPS
ENCCU   -----------------SLRIYN--AF-----------------------
ANTLO   CSLDLNKKLETTCRII-HTQIEY---------------------------
ARATH*  AHLSS-TQMSPI-QPL-RLLVPC---------------------------
VITVI   SQYTS-TQMSPI-QPL-RLLVPT---------------------------
SOYBN   SQYAS-SQMSPI-QPL-HLLVPA---------------------------
SOLLC   ALYAS-AQMSPI-QPL-RLLVPV---------------------------
ORYSJ*  SLFAA-SEMTIV-MPV-KLLVPA---------------------------
HORVU   SMLAS-AQTSLI-MPL-RLLVPA---------------------------
SORBI   SMFAS-AQMSPI-LPL-RLLVPA---------------------------
SELML   ------HHMAVL-SPL-RVLVPP---------------------------
PHYPA   FLQSKEYALVSR-ASL-YLTVPP---------------------------
VOLCA   ------TALTTAATPV-----PA---------------------------
                  2560      2570      2580      2590      2600
```

MED15

HUMAN*	---DYP
CHICK	---DYP
XENTR	---DYP
DANRE	---DYP
CIOIN	---SYP
ANOGA	---DYP
TETNG	---DYP
DROME	---EYP
BRUMA	---AYP
CAEEL	---TYP
YEAST*	---SSP
SCHPO	---STP
CANAL	--VEEP
CANGA	---SNA
ASPNI	---KQSHP
RHIO9	---PKP
CRYNE	---ALP
USTMA	ADADTGLDIDLFDFIEASAAGPDAFNDTDVDLSGVFGSSALNASTQESEP
ENCCU	---KDAFP
ANTLO	--
ARATH*	---SYP
VITVI	---NYP
SOYBN	---NYP
SOLLC	---NYP
ORYSJ*	---DYP
HORVU	---GYP
SORBI	---SYP
SELML	---TYP
PHYPA	---TYP
VOLCA	---ARG

2610 2620 2630 2640 2650

HUMAN*	AQ-SPLWID-----------------RQWQYDANPFLQSVHRCMTSRLL-
CHICK	DQ-SPLWIK-----------------NPRQYAANPFLQSVYQYMTSKLL-
XENTR	DQ-SPLWMD-----------------NPRQYEANPFLQSVYRYMTSKLL-
DANRE	DQ-SPHWED-----------------DGQQYEANPFLQTVHKNMTSKLL-
CIOIN	TQ-PPTWVQGFNYEPRENDIQISDP-EQTNLLGSDFLLKLRPMIRSRLT-
ANOGA	ST-APSCSL-----------------IEQEYNATPFLILVQKSLMARIC-
TETNG	EQ-SPCWDD-----------------HGDQYGSNSFLQKVHRTMTSKLL-
DROME	WQ-APDCSL-----------------AEQEYSATPFLQTVQQALIARIS-
BRUMA	NG---------------EVMVGRAALDLDSFFFDDLQNVIHERLA-----
CAEEL	AG---------------NVTVDRAVIDLDAYLYDDLQNSVYERLS-----
YEAST*	STSSNMDVG-----------------NPRKRKASVLEISPQDSIASVL--
SCHPO	DL-SFTDNKNSLSPSNILSLDGKFSFNDDSELWADLGNEINSEIGFLK--
CANAL	QS------------------------VKREHDADDDLDSLFTNKKANLDG
CANGA	TT-------------SKDIGNIYSHTGGVKRKFDEVEISPNSNG------
ASPNI	ST-TPVSGTSGAK-------------AQANKQAAAEARPINGAFKCSVPE
RHIO9	TPGAALKLA-----------------VQSLDSKTSKLIDIQKEVERLFS-
CRYNE	KD-SPSNLQEAIRHKGLRVEDLKPPPSKRQKSKGSPATPANAQVPTPES-
USTMA	PVETPELVASARLPGGS---------SNASTISNSTSNSMRGKMPAALG-
ENCCU	SS----------------------ELLQKYETIKNLLEKQEEYIKYGAYF
ANTLO	------------------------ILKDKYFLREVFVDVAIERIRKYAA-
ARATH*	NG-SPSLLDKLP--------------VETSKENEDLSSKAMARFNILLR-
VITVI	NS-SPILLDKFP--------------VEISKEYEDLSVKAKSRFSISLR-
SOYBN	NC-SPILLDKFP--------------VESSKENEDLSVKARSKFSTSLR-
SOLLC	NC-CPILLDKFP--------------VEVSKEYEDLSTKAKSRFSVSLR-
ORYSJ*	KS-SPILVDN----------------DDEQRRLSDISYAVAVAFGCAVD-
HORVU	KCASPVILDKFP--------------DEQ-RNSDDLSSKARSKFGVSLR-
SORBI	KC-SPVLLDKFP--------------DEQCRNSDDLSTKAKSKFSVLLR-
SELML	KT-PPLLMSD----------------NFDPNSNHLLSVVARDSFSRAVR-
PHYPA	VH-FPDIWFD----------------QSNPQANHHCFKGAREEFMRNLR-
VOLCA	VSAAAVETTG----------------GASDEDVKPRRVTAGRRTSTAAGG

2660 2670 2680 2690 2700

MED15

```
HUMAN*  ------------------------------QLP-----------------
CHICK   ------------------------------QLP-----------------
XENTR   ------------------------------QLP-----------------
DANRE   ------------------------------QLP-----------------
CIOIN   ------------------------------MVN-----------------
ANOGA   ------------------------------KLP-----------------
TETNG   ------------------------------QLP-----------------
DROME   ------------------------------KLP-----------------
BRUMA   ----------------------------RPGLR-----------------
CAEEL   ----------------------------RPGLS-----------------
YEAST*  --------------------------------------------------
SCHPO   --------------------------------------------------
CANAL   LDFDRYLY------------------------------------------
CANGA   --------------------------------------------------
ASPNI   CQYHYQGFGSQGDLDKHVEESHKAEEPIENALQFALQCFSTLVKDEQKPD
RHIO9   --------------------------------------------------
CRYNE   ----------------------------------------AKTPANTAVI
USTMA   ----------------------------ASGLDFGSAGPSSTSPAEMSSC
ENCCU   LKAR----------------------------------------------
ANTLO   --------------------------------------------------
ARATH*  ------------------------------SLS-----------------
VITVI   ------------------------------SLS-----------------
SOYBN   ------------------------------SLS-----------------
SOLLC   ------------------------------SLS-----------------
ORYSJ*  ------------------------------ELP-----------------
HORVU   ------------------------------GLA-----------------
SORBI   ------------------------------GLA-----------------
SELML   ----------------------------LGNYT-----------------
PHYPA   ------------------------------CLP-----------------
VOLCA   GNDGGSGEGGDGGGGEEGVRGAGGGNRGGRGRRGRGGGGSRARGRGIIGG
                 2710      2720      2730      2740      2750
```

```
HUMAN*  -------------------------------DKH-SVTALLNT-------
CHICK   -------------------------------DKH-SVTALLNT-------
XENTR   -------------------------------DKH-SLTALLNT-------
DANRE   -------------------------------DKH-SVTALLNT-------
CIOIN   -------------------------------NTHCTISSLLQT-------
ANOGA   -------------------------------GLF-TLSHLLDT-------
TETNG   -------------------------------DKH-SLTELLNT-------
DROME   -------------------------------KNY-SLSHLLDT-------
BRUMA   -----------------------------------TVTDFLET-------
CAEEL   -----------------------------------SITDYLNA-------
YEAST*  -------------------------------SPDSNIMSDSKK-------
SCHPO   -------------------------------EPD-TMNLALDADKDKTKM
CANAL   -------------------------------EPV-SFDE-----------
CANGA   -------------------------------SPSASIMSESKK-------
ASPNI   SQGLTKGFGFATALPAKQEVKAEGATPVSGATPMGRVSSQVAA-------
RHIO9   -----------------RPDQPKTMKNEACLQPEGPLNTTIGK-------
CRYNE   GTQGTLGESPKEKKATKRKRQSSTATAAGTEKPVKQTKAEAAKAKKAAVA
USTMA   DEIDLDKSPFVHRMSLDRMGLTMAPAVAGTEAPK-AINTVGGQSTLKAQE
ENCCU   -----------------------------------SIDSFIDQ-------
ANTLO   -----------------------------------LIRSELDSAQKETEV
ARATH*  -------------------------------QPM-SLKDIAKT-------
VITVI   -------------------------------QPM-SLGEIART-------
SOYBN   -------------------------------QPM-SLGEIART-------
SOLLC   -------------------------------QPM-SLKDIVKT-------
ORYSJ*  -------------------------------EPR-SIEAMAMA-------
HORVU   -------------------------------EPM-SLREIARA-------
SORBI   -------------------------------EPM-SLREIART-------
SELML   -------------------------------HPL-TIKAMARA-------
PHYPA   -------------------------------PPV-SLASIATS-------
VOLCA   GGRTGRKKLQSDDVEEDDAGSGPGAAGGGEEQVRDSGEERLEQQVVAGAS
                 2760      2770      2780      2790      2800
```

MED15

HUMAN*	------------------------WAQSV--HQACLSA----A-------
CHICK	------------------------WAQSI--RQACLSA----A-------
XENTR	------------------------WAQSI--RQACLSA----A-------
DANRE	------------------------WAQSV--RQACLSA------------
CIOIN	------------------------WASSA--ASASTLV----H-------
ANOGA	------------------------WEMSV--RQACSPN----PTIVAPTG
TETNG	------------------------WAQSV--QQACRWA----A-------
DROME	------------------------WEMAV--RQACSPQ----SKPRAVCE
BRUMA	------------------------WNEFWFFKTFCFQS------------
CAEEL	------------------------WEEQV--NQYQNQT----SGGLDVAF
YEAST*	------------------------IKVDSPDDPFMTKSGATTSEKQEVTN
SCHPO	QNKLTQINFDESCFLDPAIDDKDPWNEMLHEKKVLLKQ----LQVGNEDE
CANAL	------------------------W------KDFVVSS----IQ------
CANGA	------------------------IKIDSPEDMFVTHS----SEAAKGTN
ASPNI	-------------------------------KPASPAS----SQQLTPVR
RHIO9	------------------------VDEKDVQEKKVPTE----EE------
CRYNE	AAAAVAIPATVPEDPVNALGIQLAADEAATKAEIAQHK----AFFDDQRA
USTMA	ETAINQVELGPGSQGITAGSSDDWWDLDHFGSVTNAGF----GSDANADQ
ENCCU	-IKSLTVDMSYDLKLNTMDASDINYGECL--MNAVKAF----AERKKKED
ANTLO	YDF----------------------------AKVCEKV----KKAFRKMK
ARATH*	------------------------WDACA--RAVICEY----AQ----QF
VITVI	------------------------WDVCA--RAVISEY----AQ----QS
SOYBN	------------------------WDVCA--RSVISEH----AQ----QS
SOLLC	------------------------WDVCA--RAVICEY----AQ----QS
ORYSJ*	------------------------WDGCV--RRAVTEV----AH----RH
HORVU	------------------------WDASA--RGAIVEC----AQ----KT
SORBI	------------------------WDACA--RKVIAEY----AQ----QT
SELML	------------------------WDECA--RKVVSEA----IYGSSGSS
PHYPA	------------------------WDECA--RKSLTEY----AH----QQ
VOLCA	PRSAGATAAASSGGGGAGGAGGLDISRTVNKAALATEE----ALTPPRPR
	2810 2820 2830 2840 2850

HUMAN*	--
CHICK	--
XENTR	--
DANRE	--
CIOIN	--
ANOGA	TSVLLGM---
TETNG	--
DROME	LSTLLGV---
BRUMA	--
CAEEL	NVGNDFFYDNLNL-------------------------------------
YEAST*	EAPFLTS---
SCHPO	DNIAFPT---
CANAL	--
CANGA	NSSLMDS--------------------------------GKEGSCKSMAG
ASPNI	GATKGSG---
RHIO9	--
CRYNE	LASAAAP---
USTMA	PWAVLS--
ENCCU	TFSLNVI---
ANTLO	DANPNFVFCIDPQHV-----------------------------------
ARATH*	GGGTFSS---
VITVI	GGGSFSS---
SOYBN	GGGSFSS---
SOLLC	GGGTFSS---
ORYSJ*	GGGTFSS---
HORVU	GGGSFSS---
SORBI	GGGSFSS---
SELML	NGGPFLS---
PHYPA	GGGSFTT---
VOLCA	RSTTTSAVGRLATEAAVDAVDGDTAVVVVGQSPLRQQPFEMAPFPQYRPG
	2860 2870 2880 2890 2900

MED15

```
HUMAN*   --------------------------------------------------
CHICK    --------------------------------------------------
XENTR    --------------------------------------------------
DANRE    --------------------------------------------------
CIOIN    --------------------------------------------------
ANOGA    --------------------------------------------------
TETNG    --------------------------------------------------
DROME    --------------------------------------------------
BRUMA    -------SRIFMYGCFAKCERELLHVVLLNFCPLYLFVLFYLIIFIHIVK
CAEEL    --------------------------------------------------
YEAST*   -GTSSEQFNVWDWNNWTSAT------------------------------
SCHPO    -----------STNIWQVVI-----------------------------
CANAL    --------------------------------------------------
CANGA    SATEVNDTSIWDWNFWTSIE------------------------------
ASPNI    -----------SMSVKPTLSEDGKRDSRKTAEQDPASMAKDLWADSAISF
RHIO9    -----------VLNSWYEVGLETGVPEIYEIMPGFSFDDSPIVST-----
CRYNE    -----------GDGEKKVGGEDAMAVFTKIFEAHQAGIQADMVRTDPVAQ
USTMA    --------------------------------------------------
ENCCU    -----------GKNTYE---------------------------------
ANTLO    --------------------------------------------------
ARATH*   -----------KYGTWEKYVAAS---------------------------
VITVI    -----------RYGAWENCLSAA---------------------------
SOYBN    -----------KYGTWENCLTTN---------------------------
SOLLC    -----------KYGSWENCSTAA---------------------------
ORYSJ*   -----------RHNQWRAGYLHGPMMSL----------------------
HORVU    -----------SYGCWEACVGA----------------------------
SORBI    -----------SYGCWESCVGAS---------------------------
SELML    -----------SIGVWENCVAT----------------------------
PHYPA    -----------AYGTWETTAHQIAC-------------------------
VOLCA    GEVAADVTRLDTFGPAEADASAAATAADPAQPPFTDPWQLPQWPPPPPPP
                  2910      2920      2930      2940      2950
```

```
HUMAN*   --------------------------------------------------
CHICK    --------------------------------------------------
XENTR    --------------------------------------------------
DANRE    --------------------------------------------------
CIOIN    --------------------------------------------------
ANOGA    --------------------------------------------------
TETNG    --------------------------------------------------
DROME    --------------------------------------------------
BRUMA    LG------------------------------------------------
CAEEL    --------------------------------------------------
YEAST*   --------------------------------------------------
SCHPO    --------------------------------------------------
CANAL    --------------------------------------------------
CANGA    --------------------------------------------------
ASPNI    DAIRDAFGNLSDDHDLDLGVMDEFLNAEMFNGTQDTPDSVETGVVTQTPK
RHIO9    --------------------------------------------------
CRYNE    SASTAAGHMPPVTSAGDPNDQDLFDTYFDVTLFSLAADLPTPDLVIEATP
USTMA    --------------------------------------------------
ENCCU    --------------------------------------------------
ANTLO    --------------------------------------------------
ARATH*   --------------------------------------------------
VITVI    --------------------------------------------------
SOYBN    --------------------------------------------------
SOLLC    --------------------------------------------------
ORYSJ*   --------------------------------------------------
HORVU    --------------------------------------------------
SORBI    --------------------------------------------------
SELML    --------------------------------------------------
PHYPA    --------------------------------------------------
VOLCA    PPLIPGPPRPSLESPAGPSARPARAPCATDPRVAAATALLPPLQPQPPPH
                  2960      2970      2980      2990      3000
```

MED15

```
HUMAN*  --------------------------------------------------
CHICK   --------------------------------------------------
XENTR   --------------------------------------------------
DANRE   --------------------------------------------------
CIOIN   --------------------------------------------------
ANOGA   --------------------------------------------------
TETNG   --------------------------------------------------
DROME   --------------------------------------------------
BRUMA   --------------------------------------------------
CAEEL   --------------------------------------------------
YEAST*  --------------------------------------------------
SCHPO   --------------------------------------------------
CANAL   --------------------------------------------------
CANGA   --------------------------------------------------
ASPNI   DGEVLKNDEGVNKDSDATDGSWIPADWCRLPGRFEEPLLKDEVCKEIDWD
RHIO9   --------------------------------------------------
CRYNE   QEVDGESPESVRTVGSTAGHTVPGKEVKSGSKEEKGKEEGTKIILGSPGS
USTMA   --------------------------------------------------
ENCCU   --------------------------------------------------
ANTLO   --------------------------------------------------
ARATH*  --------------------------------------------------
VITVI   --------------------------------------------------
SOYBN   --------------------------------------------------
SOLLC   --------------------------------------------------
ORYSJ*  --------------------------------------------------
HORVU   --------------------------------------------------
SORBI   --------------------------------------------------
SELML   --------------------------------------------------
PHYPA   --------------------------------------------------
VOLCA   GESGPEALGLDDVASGGAQPAMVLSPGGVGGGDGDGGVLGSLASPGGDIA
                 3010      3020      3030      3040      3050
```

```
HUMAN*  --------------------------------------
CHICK   --------------------------------------
XENTR   --------------------------------------
DANRE   --------------------------------------
CIOIN   --------------------------------------
ANOGA   --------------------------------------
TETNG   --------------------------------------
DROME   --------------------------------------
BRUMA   --------------------------------------
CAEEL   --------------------------------------
YEAST*  --------------------------------------
SCHPO   --------------------------------------
CANAL   --------------------------------------
CANGA   --------------------------------------
ASPNI   MIDLKGGTLSADDNGIAIYAM-----------------
RHIO9   --------------------------------------
CRYNE   MAYNGGIEW-----------------------------
USTMA   --------------------------------------
ENCCU   --------------------------------------
ANTLO   --------------------------------------
ARATH*  --------------------------------------
VITVI   --------------------------------------
SOYBN   --------------------------------------
SOLLC   --------------------------------------
ORYSJ*  --------------------------------------
HORVU   --------------------------------------
SORBI   --------------------------------------
SELML   --------------------------------------
PHYPA   --------------------------------------
VOLCA   GSSADGGSAGPSGGDMGPFDMLGLGFGDLLGAGFLGQQ
                 3060      3070      3080
```

MED16

```
HUMAN*  --------------------------------------------------
CHICK   --------------------------------------------------
DANRE   --------------------------------------------------
CIOIN   --------------------------------------------------
TETNG   --------------------------------------------------
DROME   --------------------------------------------------
YEAST*  --------------------------------------------------
CANAL   --------------------------------------------------
CANGA   --------------------------------------------------
ASPNI   --------------------------------------------------
RHIO9   --------------------------------------------------
CRYNE   --------------------------------------------------
USTMA   --------------------------------------------------
ARATH*  MNQQNPEEEVSLVNNSGGGGIIEAPAIVEEKEEEGL----QQKQEETIES
VITVI   ------------MSPGSG---------AKDSEDEPV----PQALEVAKGL
SOYBN   ------------MNQIGG---------TKDPEAEPVSPSLPQPQDTPKSS
SOLLC   ----------------------------MNNQQPPV----AKDTEVEKEE
ORYSJ*  --------------------------------------------------
HORVU   --------------------------------------------------
SORBI   MTSSAPPNPSPVPVPVPVPTPIPTANGTASPPKEQPPPPPQQQQQQGGGQ
PHYPA   --------------------------------------------------
                 10        20        30        40        50
```

```
HUMAN*  --------------------------------------------------
CHICK   --------------------------------------------------
DANRE   --------------------------------------------------
CIOIN   --------------------------------------------------
TETNG   --------------------------------------------------
DROME   --------------------------------------------------
YEAST*  --------------------------------------------------
CANAL   --------------------------------------------------
CANGA   --------------------------------------------------
ASPNI   --------------------------------------------------
RHIO9   --------------------------------------------------
CRYNE   --------------------------------------------------
USTMA   --------------------------------------------------
ARATH*  TDPI---LVVVEEKLLEKSVD----------------------GEKEDDN
VITVI   EKSE---VVSVDEEEGEQEEE----------------------GEQVVEK
SOYBN   DKTDSATAVVVVAAASEEQEV----------------------GEEEKPP
SOLLC   ESSS---VITNSLEPSTNKVHEKPDIIIEDDDDDHPAAIVAMVTEKEVSS
ORYSJ*  --------------------------MEVDGGAGGGGGGVGDVEGGGGGG
HORVU   --------------------------------------------------
SORBI   EEAAAADGGGAEAAEAGVAAGVAPEAMEVDGGSGAGDAEVGGSVGGGGGG
PHYPA   ------------------------------------MEGVEGDQQWGSFN
                 60        70        80        90       100
```

```
HUMAN*  ------MCDLRRPAAGGMMDLAYVCEWEKWSKSTH----------CPSVP
CHICK   ------------PFPGAAMDLAYVCEWEKRPKSDH----------CPPIP
DANRE   ------------------MDIAYVCEWEKRPKSNH----------CPSIP
CIOIN   ------------------MDPVYEIDWQADVNQIT----------LGHGD
TETNG   ------------------MELAYVCEWDKRSKSTH----------CPSIP
DROME   ------------------MTILYKVEGKEFSKDSV----------FPHKK
YEAST*  -----------------------------MMLGEH---------------
CANAL   ----------------------MATQTDKQTIGRH----------LEQVP
CANGA   ----------------------------MCELNNK---------------
ASPNI   MPLIMEDGINVDDLFGESTSLELGLPTSTPTASTK-----GLAQRLDEMR
RHIO9   ------------------MVQLVTSKRQKLSYTDHKIPRYSSSFNFSSLT
CRYNE   --------------------------------------------------
USTMA   --MANHGSRIRHEKIVNHIYPSHESHRPQFSFSKHSHHRESLVTLLHSFI
ARATH*  SS----SSNMEIDPVSPATVFCVKLKQPNSNLLHK----------MSVPE
VITVI   TA----DDPMEEDSVNPATVFCIRLKQPRSNLQHK----------MSVPE
SOYBN   PP----PVPMEEDPVNPATVFSIRLKQPRSNLLHK----------MSVPE
SOLLC   VD----DDPMDEDTVNPAVVFCIRLKQPRSNLLHK----------MSVPE
ORYSJ*  GAGGGGGGGGGGQQASPATVFRIRLKQPPSSLRHK----------MRVPE
HORVU   --------------------------------------------------
SORBI   GA----GAGGGAQQASPATVFRIRLKQSPASLRHK----------MRVPE
PHYPA   NQATQKSEELEDEEDDEKTILQVALRSPPFSTALK----------MNFPE
                110       120       130       140       150
```

MED16

```
         151                                              200
HUMAN*   L-------------------------------------------------
CHICK    L-------------------------------------------------
DANRE    L-------------------------------------------------
CIOIN    IV------------------------------------------------
TETNG    L-------------------------------------------------
DROME    V-------------------------------------------------
YEAST*   --------------------------------------------------
CANAL    KASN----------------------------------------------
CANGA    --------------------------------------------------
ASPNI    LVGCCQ--------------------------------------------
RHIO9    FKQSPQ--------------------------------------------
CRYNE    --------------------------------------------------
USTMA    IARTNETTSADIKRAAYSSHAIGAIDIERLLQRLPRIATFSPRHNTSKPI
ARATH*   LCRNFS--------------------------------------------
VITVI    LCRNFS--------------------------------------------
SOYBN    LCRNFS--------------------------------------------
SOLLC    LCRNFS--------------------------------------------
ORYSJ*   LCRNFS--------------------------------------------
HORVU    --------------------------------------------------
SORBI    LCRNFS--------------------------------------------
PHYPA    LCRKFS--------------------------------------------
                  160       170       180       190       200
```

```
HUMAN*   --------------------------------------------------
CHICK    --------------------------------------------------
DANRE    --------------------------------------------------
CIOIN    --------------------------------------------------
TETNG    --------------------------------------------------
DROME    --------------------------------------------------
YEAST*   --------------------------------------------------
CANAL    --------------------------------------------------
CANGA    --------------------------------------------------
ASPNI    --------------------------------------------------
RHIO9    --------------------------------------------------
CRYNE    --------------------------------------------------
USTMA    PASTWLECLQRSDRPHLLKKCSFFISMSMETPAKRQCTDNTSANSSSRSI
ARATH*   --------------------------------------------------
VITVI    --------------------------------------------------
SOYBN    --------------------------------------------------
SOLLC    --------------------------------------------------
ORYSJ*   --------------------------------------------------
HORVU    --------------------------------------------------
SORBI    --------------------------------------------------
PHYPA    --------------------------------------------------
                  210       220       230       240       250
```

```
HUMAN*   ---------------------------------------ACAW-SCRNLI
CHICK    ---------------------------------------VCAW-SCRNLI
DANRE    ---------------------------------------VCAW-SCRNLI
CIOIN    ---------------------------------------ECAW-SASNFF
TETNG    ---------------------------------------VCSW-SCRNLV
DROME    ---------------------------------------LCSV-SARNIV
YEAST*   ---------------------------------------LMSW-SKTGII
CANAL    ---------------------------------------LISW-SRNGFI
CANGA    ---------------------------------------RVSW-SKTGLI
ASPNI    ---------------------------------------KIAW-SRLGCI
RHIO9    ---------------------------------------ALSI-SKQNIL
CRYNE    ------------------------------------------YPPRTHLP
USTMA    RKTLSVEQQRSIRHAQCLENGLGAIQRLERLRSGKADLAKIAW-SPQNVL
ARATH*   ---------------------------------------AVAWCGKLNAI
VITVI    ---------------------------------------AVAWCGKLNAI
SOYBN    ---------------------------------------AVSWCGKLNAI
SOLLC    ---------------------------------------AVAWCGKLNAI
ORYSJ*   ---------------------------------------AVAWCGKLNAI
HORVU    --------------------------------------------------
SORBI    ---------------------------------------AVAWCGKLNAI
PHYPA    ---------------------------------------AVAWCSKTNLI
                  260       270       280       290       300
```

MED16

```
HUMAN*   AF------------------------------------------------
CHICK    AF------------------------------------------------
DANRE    AF------------------------------------------------
CIOIN    AF------------------------------------------------
TETNG    AF------------------------------------------------
DROME    AF------------------------------------------------
YEAST*   AY------------------------------------------------
CANAL    AY------------------------------------------------
CANGA    AY------------------------------------------------
ASPNI    AS------------------------------------------------
RHIO9    IS------------------------------------------------
CRYNE    HA------------------------------------------------
USTMA    AIASAHRSSSASPNGHVVVQYPLASSSKPPLKSSYLYPDQDGFSSSSTAA
ARATH*   AC------------------------------------------------
VITVI    AC------------------------------------------------
SOYBN    AC------------------------------------------------
SOLLC    AC------------------------------------------------
ORYSJ*   AC------------------------------------------------
HORVU    -C------------------------------------------------
SORBI    AC------------------------------------------------
PHYPA    CC------------------------------------------------
                  310       320       330       340       350
```

```
HUMAN*   --------------TMDLRS--DDQDL------------------TRMIH
CHICK    --------------TTDLRNE-EERDL------------------THMVH
DANRE    --------------TTDLKNEEDDKDL------------------SHMIH
CIOIN    --------------TTEKNNQ---------------------GSVTQYLH
TETNG    --------------TTDLKNNNEDKDI------------------SHMIH
DROME    --------------SALQSAIFPASHGGDAGDGSLPGAGLAVGASNSHVY
YEAST*   -------------------SDSQSSNANICLTFLESINGINWRFHTPQKY
CANAL    ----IPPIITTTTTTPTTTTTTNNNKSNLLLT-----------------Y
CANGA    -------------------ADEQSTYGNLCISFLETINGVNCRFHPPQRY
ASPNI    --------------ISQDSTRVNVRHLQ----------------------
RHIO9    --------------APIQSI-------------------------SDQIH
CRYNE    --------------------------------------------------
USTMA    FSHQRSTVTTATTAAAASTTSSQSTRFDDPTNISFSPCGYYLCAFFPVPH
ARATH*   --------------ASETCARIPSSKANTPFWIPI--------------H
VITVI    --------------ASETCARIPSSNANPPFWIPI--------------H
SOYBN    --------------AAETCARIPSSTANPPFWIPI--------------H
SOLLC    --------------ASETCARIPSSNANPPFWIPI--------------H
ORYSJ*   --------------ASETCARIPSSNSSPPFWIPI--------------H
HORVU    ----------------------------TTLYISV---------------
SORBI    --------------ASETCARIPSSNSSPPFWIPI--------------H
PHYPA    --------------AVETCSRDQGSTQEPAFWIPM--------------H
                  360       370       380       390       400
```

```
HUMAN*   ILDTE---------HPWDLH-------SIPSEHHEA--------------
CHICK    IIDTE---------HPWDVY-------SVNSGHVEA--------------
DANRE    IIDTD---------HPWDVY-------SINSGHNEI--------------
CIOIN    ILNPN---------QPWGVF--------STKYNDGA--------------
TETNG    IIDTE---------HPWDVY-------CISSGHTEV--------------
DROME    VCDIV---------TPWEYY--------KVCSSKSL--------------
YEAST*   VLHPQ------LHEVQYQESSSTLSTHSTTTSVNGSTTAGVGSTPNFGGN
CANAL    IKNSDGKKWQLASPEPINIKLENNFLPQ----------------------
CANGA    VIHPQ------LHEVPLKTETGNGINGASGSSTAQSNIHGTTSVP---GS
ASPNI    -CQQSDGKWLLSDETPLNQ--------VSEVHAGHA--------------
RHIO9    LIAGDLYAHNRIHIQKTNVEI------IQNYHKQHT--------------
CRYNE    --------------------------------------------------
USTMA    DAATTNAKNNAFDPTPSNVSLMTNGLATRTLPTSSA--------------
ARATH*   ILIPE---------RPTECAVFN----VVADSPRDS--------------
VITVI    IVIPE---------RPTECSVFS----VIADSPRDS--------------
SOYBN    IVIPE---------RPTECAVFN----VIADSPRDS--------------
SOLLC    IVIPE---------RPTECTVFN----VIADSPRDS--------------
ORYSJ*   ILNPE---------RPTECSVFN----VKADSPRDF--------------
HORVU    -------------------SVFV---------------------------
SORBI    ILNPE---------RPTECSVFN----VKADSPRDF--------------
PHYPA    LVDPE---------RPTEHSVFN----VPADSPGDS--------------
                  410       420       430       440       450
```

MED16

```
HUMAN*   -----------ITCLEWDQ----SGSRLLSADA--------------DGQ
CHICK    -----------ITCLEWDQ----SGSRLLSADA--------------DGH
DANRE    -----------ISCLEWDQ----SGSRLLSADG--------------DGQ
CIOIN    -----------ITCLQWDH----TGTRLVSGDN--------------HGL
TETNG    -----------ISCLEWDQ----SGSRLLSADG--------------DGQ
DROME    -----------ISVLQWSP----NGEQLLLGYV--------------GGR
YEAST*   SNKSPPQFFYNISSIHWNNWFSLPGDMLAVCDE--------------LGN
CANAL    -----------LSLVSWGS----LNTDLAVSDI--------------YGN
CANGA    AGKHQHQFFYDISSIHWNNWFSLPGDMLAVCDE--------------LGN
ASPNI    -----------LVHLCWND----SGTELAVADS--------------SGR
RHIO9    -----------LTHIEWNQ----KGNAFASIDE--------------TGQ
CRYNE    --------------------------------------------------
USTMA    -----------PTITQLSANLPALDQSLPASHHIAATDVPPAPSVGPRAR
ARATH*   -----------VQFIEWSP--TSCPRALLIANF--------------HGR
VITVI    -----------VQFIEWSP--ASCPRALLIANF--------------HGR
SOYBN    -----------VQFIEWSP--SSCPRALLIANF--------------HGR
SOLLC    -----------VQFIEWSP--TSCPRALLIANF--------------HGR
ORYSJ*   -----------VQFIEWSP--RSCPRALLVANF--------------HGR
HORVU    ----------------------------FFCN------------------
SORBI    -----------VQFIEWSP--RSCPRALLVANF--------------HGR
PHYPA    -----------IQHLEWSP--NSCPRALLIVNA--------------SGR
                  460       470       480       490       500

HUMAN*   IKCW-------------SMADHLA--------------------------
CHICK    IKCW-------------SMTDHLA--------------------------
DANRE    IKCW-------------GMTEHLV--------------------------
CIOIN    CCLW-------------TMSNSLC--------------------------
TETNG    IKCW-------------SMSEHLV--------------------------
DROME    VEIW-------------QPRNQSI--------------------------
YEAST*   MTMLITGQRPDRATTYEKLTMVFQ--------------------------
CANAL    FYILLAGVGLLDPNHIPSTITTTSTIKTEGNTEKNKDTKQIGNGSGTNGH
CANGA    MTMLIAGQSPDGPTTLDKLTMLFQ--------------------------
ASPNI    VSIY--------------SIPIAL--------------------------
RHIO9    LALW--------------QLEGPI--------------------------
CRYNE    --------------------------------------------------
USTMA    LCIW------------SRAETGAL--------------------------
ARATH*   ITIW--T----QPTQGSANLVHDA--------------------------
VITVI    ITIW--T----QPSQGPANLVRDA--------------------------
SOYBN    VTIW--T----QPPQGPANLVHDT--------------------------
SOLLC    ITIW--T----QPSQGSANLVRDA--------------------------
ORYSJ*   ITIW--T----QPTKGPTNLVRDA--------------------------
HORVU    ---------------------CSA--------------------------
SORBI    ITIW--T----QPTKGPVNLVRDA--------------------------
PHYPA    ASIW--S----QPTQGGANVARTC--------------------------
                  510       520       530       540       550

HUMAN*   ---------------NSW--ESSVGSLV----------------------
CHICK    ---------------NSW--ENTMGSMV----------------------
DANRE    ---------------NSW--ECAQSSSV----------------------
CIOIN    ---------------NVWTHQNKHNIFL----------------------
TETNG    ---------------NSW--ENILLSSV----------------------
DROME    ---------------NLW--VLQYYATV----------------------
YEAST*   ---------------DNVYKIYNHVMPLKPVDKLKPMNIERKQTRKE---
CANAL    GDSPINTPSFELTSYNHM--EMIYRDIINPDINSVVN-------------
CANGA    ---------------DNVYKIHNHVMQLEPERENTDSKLERKKTKKE---
ASPNI    ---------------NSV--NVTRQAAFDPDD------------------
RHIO9    ---------------ENW--KLKYRVNL----------------------
CRYNE    --------------------------------------------------
USTMA    ---------------NDW--KLVQTIICEPACSQSHAISSTKTENVLKVN
ARATH*   ---------------TSW--QCEHE-------------------------
VITVI    ---------------SCW--EREYE-------------------------
SOYBN    ---------------SCW--QREHE-------------------------
SOLLC    ---------------SCW--QRDYE-------------------------
ORYSJ*   ---------------SSW--QCEHE-------------------------
HORVU    ---------------SVF--DYLFI-------------------------
SORBI    ---------------SSW--QCEHE-------------------------
PHYPA    ---------------NGW--SCEYE-------------------------
                  560       570       580       590       600
```

MED16

```
HUMAN*  --------------------------EGDPIVALSWLHNGVKLALHVE--
CHICK   --------------------------EGDPVVALSWLHNGVKLALHVE--
DANRE   --------------------------DGDPIVALSWLHNGVKLALHVE--
CIOIN   --------------------------EGEEILSLGWLHNGARFLFHID--
TETNG   --------------------------DGDPIVALTWLHNGVKLALHVE--
DROME   --------------------------PSEDIIEAQFFQNGKGVIFNAL--
YEAST*  --------------------------YNTSILEFRWLTSSKSVIVS----
CANAL   --------------------------PGASIVAFKWLNIEKPQIVNKAAT
CANGA   --------------------------YNTTILDFHWLSSSKPVIVS----
ASPNI   --------------------------DGAQIVGMMWLSQYRSVHSFYQ--
RHIO9   ---------------------------KQPLAAFFWLNA-----------
CRYNE   --------------------------------------------------
USTMA   EKGQSIPTQGLRASPSDADSGNGTGTLLGGVKHVVWLNRHRRIVLNDQ--
ARATH*  ---W----------------------RQDIAVVTKWLTGASPYRWLSS--
VITVI   ---W----------------------HQDIAVVTKWLSGVSPYRWLSS--
SOYBN   ---W----------------------RQDIAVVTKWLSGVSPYRWLSS--
SOLLC   ---W----------------------HQDIAVVTKWLSGVSPYRWLST--
ORYSJ*  ---W----------------------RQDLSVVTKWLSGISPYRWLPA--
HORVU   ---P----------------------AQLLAAAKLLCALVLQYRWLPA--
SORBI   ---W----------------------RQDLSVVTKWLSGISPYRWLPT--
PHYPA   ---W----------------------RQEQSITTKWLESPPPYRWTTS--
                 610       620       630       640       650

HUMAN*  ------KSG-----ASSFGEKFSRV-KFSPSLTLF----GGKPMEGWIAV
CHICK   ------KSG-----ASNFGEKFSRV-KFSPSLTLF----GGKPMEGWIAV
DANRE   ------KSG-----STNFGEKFSRV-KFSPSLTLF----GGKPMEGWLAV
CIOIN   ------KME-----SSNLGEKFDRA-KFWPTVMQH----GGKAMEGFIVV
TETNG   ------QSG-----STNFGEKFSRV-KFSPSLTLF----GGKPMEGWLAV
DROME   ------KKD-----HTYYAEKFERVEQQRPTLSGF----GGVASEGCVLL
YEAST*  ------QFCAFDSSSNTYRSRAQQV-P--PYGVYH----PPFIKYACLAI
CANAL   RLAENPTTTSSNSSSSIYGYGINQY-Q--PYGVCH----PIPTKQACVAL
CANGA   ------QFCAFDSSINMYRNKAQQL-S--PHGIFH----PPFMKYAGLAI
ASPNI   ----------AAKVQGRWAYSPFRR-R--PIGTFH----PA-HKGALLCI
RHIO9   --------------EREYTDNLIRE----PEIHLR----NPYGHLGFLTV
CRYNE   --------------------RAPPA-G--PRPPLA----PAPHGPTFAVL
USTMA   ------STSPSHYGASSFSRLASRGPSFIPTSTAYTDNSGPDQDLALVVF
ARATH*  ------KPSSGTNAKSTFEEKFLSQ-S--SESSAR----WP-NFLCVCSV
VITVI   ------KTSSSTSLKSPFEEKFLSQ-Q--PQTSDR----WP-NFLCVCSV
SOYBN   ------KSSAPANSKLTFEEKYLSQ-Q--SQTSAR----WP-NFLCVCSV
SOLLC   ------RTGGSA--KSTFEEKFLSQ-Q--PQSPAG----WP-NFLCVCSV
ORYSJ*  ------NSSTSSNLK-TFEEKFLTQ-Q--PQS----------------SV
HORVU   ------NSST-SNLK-AFEEKFLTQ-H--PQNS-G----WP-NILCVCSV
SORBI   ------N-STSSNLK-TFEEKFLTQ-Q--PQSSVG----WP-SILCVCSV
PHYPA   ----------STSTKGSFEEKYLPY-Q--PRSTS------------INLV
                 660       670       680       690       700

HUMAN*  TVSGLVTV-----------------------SLLKPSG------QVLTST
CHICK   TISGLVTV-----------------------SLLKPNG------QVLTST
DANRE   TVSGLVTV-----------------------SLLKPNG------TLLTAS
CIOIN   TGSGLAAV-----------------------RLLLVDG------ESVESQ
TETNG   TVSGLVTV-----------------------SLLKPGG------ALLTAS
DROME   TSSGLLAAFS--------------LPAIQKTSAAGADGPAHEVVELTPAL
YEAST*  RKNGQIDFWYQ-------------FSNS--KDHKKITL------QLLDTS
CANAL   RKNGQFILFYQ---------------GEHKVEYHKICC------------
CANGA   RRNGQLDFWYQ-------------FSNS--KDHKKITL------QLFNPH
ASPNI   TRSGVMKLLYQ-------------NPDG---RWAEITT------ELKNTS
RHIO9   TVHGEITVHYQRCGSLFSSFSTY-IPNTTQRDIKRSDS------SCFSMS
CRYNE   TASTLVLVHPQ-----------------------PTPQ------SAMHAV
USTMA   GHHGQVTLLSS---CKADRSAAVNLSTSSPVDAASPTNVCQAFFSVMLQS
ARATH*  FSSGSVQIHWSQWPSNQGSTAPKWFSTK--KGLLGAGP------SGIMAA
VITVI   FSSGSIQLHWSQWPP-QNGATSKWFSTA--KGLLGAGP------SGIMAA
SOYBN   FSSGSVQLHWSQWPPTQNGTTPKWFCTS--KGLLGCGP------SGIMAG
SOLLC   FSSGSVQLHWSQWPPNQNGAPSKWFCTS--KGLLGAGP------SGIMAA
ORYSJ*  FSSGSVQLHWSQWPS-QNSAQPRWFSTS--KGLLGAGP------SGIMAA
HORVU   FSSGSVQLHWSQWPP-QNSAQPRWFSTS--KGLLGAGP------SGIMAA
SORBI   FSSGSVQLHWSQWPS-QNSAHPRWFSTS--KGLLGAGP------SGIMAA
PHYPA   FWAGTVQLHWKQWP----SSSPKWFATK--KGVLGAGA------SGVFTG
                 710       720       730       740       750
```

MED16

```
HUMAN*  E-----------------------------------SLCRLRARVALADI
CHICK   E-----------------------------------SLCRLRCRVALADV
DANRE   E-----------------------------------SLCRLRGRVALADI
CIOIN   F------------------------------------RLNVMCHQQIADI
TETNG   E-----------------------------------SLCRLRGRVALADI
DROME   H-----------------------------------SIGISRSFIEHCSM
YEAST*  N-----------------------------------QRFKDLQWLEFARI
CANAL   ------------------------------------NLTDNISIIEKASI
CANGA   N-----------------------------------ERSKGLDFLQYAKI
ASPNI   Y---------------------------------------SDRLLTHAAL
RHIO9   L-----------------------------------SGLDDWQRISHAAI
CRYNE   Q-----------------------------------CPLHTRCHATDAAP
USTMA   SLYTPSVGEKPAEIVAPYLSPFSATRAVDDYTSDYAQERESGNFLTHLAV
ARATH*  D-----------------------------------AIITDSGAMHVAGV
VITVI   D-----------------------------------AIITDTGAMHVAGV
SOYBN   D-----------------------------------AIITDSGAMHVAGV
SOLLC   D-----------------------------------AIITDSGAMHVAGV
ORYSJ*  D-----------------------------------AIITETGALHVAGV
HORVU   D-----------------------------------AIITESGALHVAGV
SORBI   D-----------------------------------AIVTESGALHVAGV
PHYPA   D-----------------------------------AIINDSGSLLVAAA
                 760       770       780       790       800
```

```
HUMAN*  AFTGGGNIVVATADGSSASP--VQFY-KVCVSV-----------------
CHICK   AFTGGGNIVVATSDGSSASP--VQFY-KVCVSV-----------------
DANRE   AFTGGGNIVVAATDGSSSSP--VQFY-KVCVSV-----------------
CIOIN   AFTSQGKVVVMTTDSEIQHF--IQFH-EIQLTY-----------------
TETNG   AFTGGGNIVVAASDGSSSSP--VQFY-KVVVSV-----------------
DROME   TPSPSGALNIAFSCGWQQQL--VQCF-KVSLSLEGELG------------
YEAST*  TPMNDDQCMLITTYSKLSKN--ISFY-KLHVNWNLNATKPN---------
CANAL   GFNNDKQIIVTAWDSLSND---INVY-SIDINWGFLVESA----------
CANGA   TSVNNDNSILITTYSRLTGK--LSFY-KLFVDWNVNTAKPV---------
ASPNI   VTTQAG--ILVATHSACQR---ISLY-RVSIQWT--------PSQWDPT-
RHIO9   TLVKDKIYLATHCASFQPKA--IQIY-TIDIKFPT---------------
CRYNE   APDAPGDA-------------------QIDRGW----------------M
USTMA   GMPANDSVLLVASKRRSSSAGLIHIT-EITVDL-----------------
ARATH*  PIVNPSTIVVWEVTPGPGNG--LQATPKISTGSR------VPPSLSSSSW
VITVI   PIVNPSTVVVWEVTPGPGNG--FQVIPKSSATNG------VPPSLNPPSW
SOYBN   PIVNPSTIVVWEVAPGPGNG--FQVTPKTSTTCG------VPP-LSPPNW
SOLLC   PIVNPSTVVVWEVAPGPGNG--FQATPKTSVSNG------VPASLNPPSW
ORYSJ*  PLVNPSTVVVWEVMPGLGNG--IQATAKINATSS------LPPSLNPPLW
HORVU   PLVNPSTVVVWEVMPGLGNG--IQATAKINATSP------LPPSLNPPSW
SORBI   PLVNPSTVVVWEVMPGHGNG--IQATAKINATSS------VPPSLNPPSW
PHYPA   PVGNPSTVVVWEVTAWDLNT--PSNTQQVSAKI-------LIGNIQSPSW
                 810       820       830       840       850
```

```
HUMAN*  ----------------------------VSEK------CRIDTEILPSLF
CHICK   ----------------------------VNEK------CKIDTEILPSLF
DANRE   ----------------------------VNEK------CRIDTELLPSLF
CIOIN   ----------------------------NTQD----HLISHEVSALPSLQ
TETNG   ----------------------------VNEK------CRIDTELLPSLF
DROME   ----------------------------LEQH------LAIKSESQTSIF
YEAST*  ----------------------------VLND--------------PSLK
CANAL   ----------------------------KRQKLDQHYHTPKEAQKPPRLT
CANGA   ----------------------------VLND--------------PSLK
ASPNI   ----------------------------QQKQ-------GSQQFPTPSFR
RHIO9   ----------------------------VDKK------GSIQRKLVASTC
CRYNE   DIVPGVQGVWVGWQRGATTGVLRAEVGVDKAGNHCASCFALQTNADPHAV
USTMA   ----------------------------HAES------ITMITRPLPPVS
ARATH*  TGFAPLAAYLFSWQEYLISEIKQGKK--PSDQ---------DSSDAISLS
VITVI   AGFSPLAAYLFSWQEFLASEMKQGRK--QTDQ---------DFNDAVLLH
SOYBN   DGFAALAAYLFSWQDYLLSEAKQGRK--QTDQ---------NLVDAVPLY
SOLLC   DGYAPLAAYLLSSQEYLLQEAKQGKK--LTEQ---------HYSDMVTLH
ORYSJ*  AGFAPLASYLFSLQDYLVSEGAQTKKQAQVDN---------ETTEVASIH
HORVU   SGFAPLAAYLFSLQDYLVSEAAQTRK--QIDN---------EITEAASIH
SORBI   PGFAPLAAYLFSLQDYLVSEGAQTKK--LTDN---------ETTEAASIH
PHYPA   PGFSPLAAYLFTWQEQSTPET-------LLEG---------EDCPGPLLH
                 860       870       880       890       900
```

MED16

```
HUMAN*   MRCTTDL-------------------------------------NRKDKF
CHICK    MRCTTDP-------------------------------------ARKDKY
DANRE    MRCTTDP-------------------------------------LRRDKY
CIOIN    LRCASEE---------------------------------------HSPY
TETNG    LRCTTDP-------------------------------------LRRDKY
DROME    LGPLDGR-------------------------------------------
YEAST*   IQFILST-----------------------------------TLDPTDDE
CANAL    LKKLHQMKPIQCFKEEDGESQSQSVVPEMGKLKVEELSSIDIISSNPDPK
CANGA    IKHILDA-----------------------------------TVDQTDDE
ASPNI    LVHMKVE------------------------VPYNIPSSNRDAGENPDEQ
RHIO9    LDKDDFH-------------------------------------------
CRYNE    IQTVPLA------------------------LPVMTPPAFDGVGKRTRIH
USTMA    LSPADYAPMSHCAEDGTAFGDNSISRIDLDDISILTWAEIDSLGSNQDED
ARATH*   CSPVSNF------------------------SAYVSPEAAAQSAATTTWG
VITVI    CSPVSNF------------------------SAYVSPETAAQSAATTTWG
SOYBN    CSPVSNF------------------------SAYVSPEAAAQSAATTTWG
SOLLC    CSPVSNF------------------------SAYVSPEAAAQSTATTTWG
ORYSJ*   CCPVSNF------------------------SAYVSPEAAAQSATTTTWG
HORVU    CCPVSNF------------------------SAYVSPEAAAQSATTTTWG
SORBI    CCPVSNF------------------------SAYVSPEAAAQSATTTTWG
PHYPA    CSVVSNF------------------------SAYVSPDAA----ATTSWG
                  910       920       930       940       950

HUMAN*   PAITHLKFLA-------RDMSEQVLLCASSQ---------------TSSI
CHICK    PAITHLKFLA-------RDMSEQVLLCASNQ---------------NNSI
DANRE    PAVTHLKFLT-------RENSEQVLLCASSQ---------------MGSI
CIOIN    TYISHAHFVS-------RENQDHVLICVSGP---------------TGSK
TETNG    PAVTHLKFLT-------RENSEQVLLCASNQ---------------NGSI
DROME    -RISHLKWT--------RIANEDVIFIAYAC------------PDGAGSQ
YEAST*   GHVLKLENLHVVSKSSIEKDPSPEILVLYNV-------------------
CANAL    SGLS--------------------ILITYGS-------------------
CANGA    GRILDFTHVHVLAKVVAEKDAAPEVLLAYDV-------------------
ASPNI    LPFTSSLYCLT---------SLHIILPASDN--------------SAGST
RHIO9    --VTQMVFKR-------RADNLQLYLGLGEL-------------------
CRYNE    STLQSMSFISTPAKDEAAAESVAAVLVYEDH-------VFTPPESIRRTR
USTMA    AGKSGCLRLVACTSHFEASGSDDSADHASTA-------------SKMHST
ARATH*   SGVTAVAFDPT------RGGSVIAVVIVEGQ--YMSPYDPDEGPSITGWR
VITVI    SGVTAVAFDPT------RGGSVIAVAIVEGQ--YMSPYDPDEGPSITGWR
SOYBN    SGVTAVAFDPT------RAGSVIAVVIVEGQ--YMSPYDPDEGPSITGWR
SOLLC    SGVTAVAFDPT------RGGSVIAVVIVEGQ--YMSPYDPDEGPSITGWR
ORYSJ*   SGVTSVAFDPT------RGGSVITVVIVEGQ--YMSPYDPDEGPSITGWR
HORVU    SGVTSVAFDPT------RGGGVITVVIVEGQ--YMSPYDPDEGPSITGWR
SORBI    SGVTSVAFDPT------RGGSVITVVIVEGQ--YMSPYDPDEGPSITGWR
PHYPA    SGVTAVAFDPS------RGGSALVTTVIEGKRYYISPGNPDAGPALTGWR
                  960       970       980       990      1000

HUMAN*   VECWSLRKEGLPVNNIFQQISPVVGDKQPTILKWRILSA-----------
CHICK    VECWSLRKEGLPVNNIFQQISPVVGDKQPMILKWRILSA-----------
DANRE    VECWSLRKEGLPVNNIFQHRSPVVGEKQPMTLKWRILST-----------
CIOIN    FESWLIRKESVQLHRIFQQNTNPVSTTPDVSKCWHYVTN-----------
TETNG    VECWSLRKEGLPVNNIFQHRSPVVGEKQPTILKWRILTT-----------
DROME    LEQWTLTRRHQSVHALLQGGGGANNKPNEFVQ------------------
YEAST*   -----------------------CDTSKSLVKRYRLAPTQLSA-------
CANAL    ----------------------------SIIYRYALEKSDSSNTTSGGET
CANGA    -----------------------VGTPESLIKRFKLGQVRLPL-------
ASPNI    PNPWIVAVFSIPTHAILPHPQPQI--PASVIVRWQLETSLMTLHPRFDDV
RHIO9    -------------------------GWNGLVQQWGLKQVEQSFTTDLGSV
CRYNE    LETATFQRRLIQLAPGFSEIS---GGDTELNSMWEWYAAPQSTNTVLSPV
USTMA    IAVWDLIKAENELSDAFGSLECRKTGHHPKWLEWQLRFT-----------
ARATH*   VQRWESSVQPVVLHQIFGNPTSNFGGQVPTQTVWVSRVD------MSIPP
VITVI    VQRWESSLQPVVLHQIFGNPTSSFGGQAPVQTVWQTKVN------KSISP
SOYBN    VQRWESSLQHVVLHPIFGNPTSSMGGQPPMQTVWQSKVD------LSIPP
SOLLC    VQRWESSVEDVVLHQIFGNPTSSFGGQAPKQTVWVSKVI------KCIPA
ORYSJ*   VQCWESSVQPVVLHPIFGSP-ANFGGQPPTQTVWSTRVN------KSIPP
HORVU    VQCWESSLQPVVLHPIFGSP-SSFGGQPPMQTVWSTRVN------KSIAP
SORBI    VQCWESSRQPVVLHPIFGSP-TSLGGQPPMQTVWSTRVN------KSIPP
PHYPA    IHRWESGTKPVNIHPLL----ESTNGPPPTMTTWTSVAK------QNITR
                 1010      1020      1030      1040      1050
```

MED16

```
HUMAN*   -------------------------------TNDLDRVSAVALPKLPISL
CHICK    -------------------------------TNDLDRVSAVALPKLPISL
DANRE    -------------------------------TNDLDRVSAVALPKLPISI
CIOIN    -------------------------------STHLPQVLNFSIPTLPIDA
TETNG    -------------------------------TSDLERVSAVALPKLPISI
DROME    -------------------------------SESWEQVGKVQLNASLADI
YEAST*   ------EYLVILKPDLNIDRNNSTNQIFQSRRYNLRRHSDIVLDKKVTLI
CANAL    SKNPISQAFYNLGVEKKIDWKGDDS------SVRLVFKDKLMRRGQIESI
CANGA    ------DYLGILKPELNTSNENH-NQALRSNRSNLRFLGVLNLHHKVASI
ASPNI    PSKKGNGQVKP--------------------KTVLRRLDDITSGRHVISV
RHIO9    SRN----------------------------RTTLHCQLKIQVDGKYITC
CRYNE    NTT----------------------------IQALQPLHSIPPHSLALAV
USTMA    -------------------------------------HFKVLTDRLVTSI
ARATH*   TKDFKNHQVAAAGPSV-DAPKEPDSGDEKANKVVFDPFDLPSDIRTLARI
VITVI    TSDFKSQQTPATGPTS--ERNTSDSSMDMGKRVSFDPFDLPSDVRTLAQI
SOYBN    TNDFKNHQSPAIGMSS-DVQKVSESVSDKSKRVNFDPFDLPSDVRALARV
SOLLC    GNDFKRPQAVGAGPVP-FGKNMADSGVEMGKRVSFDPFDLPSDVRTLARI
ORYSJ*   SEDLKNPQSYVPMPTTSDERSSSECSVDRANRLSFDPYDLPNDVRQLAQI
HORVU    TEDLKNPQTYVPMPTTSDERSSSECSVDRANRLSFDPYDLPNDVRQLAQI
SORBI    TEDLKNPQTYVPMPTTSDERSSSECSVDRANRLSFDPYDLPNDVRQLAQI
PHYPA    GRFSKNSHFQQKHCER----------------------ERPVNPRAIARI
                  1060      1070      1080      1090      1100
```

```
HUMAN*   TNTDLKVASDTQFYPGLGLALAFHDGS-------VHIVHRLSLQTM----
CHICK    TNTDLKVANDTKFFPGLGLALAFHDGS-------VHIVHRLSLQMM----
DANRE    SNTDLKVASDTKFFPGLGLALAFHDGS-------IQILHRLSLQTV----
CIOIN    SSSENKVSQESKFFCGFCFAVALADNT-------IQILHRLFLQVL----
TETNG    SNTDLKVASDTKFCPGLGLALAFNDGS-------IQILHRLSLHTM----
DROME    SVTRLSVSTPD----SCQVYAILQDNS-------VQVLEPNSMKQL----
YEAST*   TSEMF----------DAFVSFYFEDGT-------IESYNQNDWKLE----
CANAL    INGFL----------DLSFLIIYKDGT-------VDVVDTTSWQIV----
CANGA    SSEVL----------DGFVSFYYRNGE-------IEIYNQNDWKLE----
ASPNI    DQTEY----------GNVLAVSYDDSS-------IAFYDPKTMAAF----
RHIO9    LKCTK----------DGHLAMGLSDGS-------IHLELSKQGLLK----
CRYNE    ISSP-----------DGTRSRVHLDLA-------AKHWNPTGHHPIKGIR
USTMA    VADASGLAVADL---ALLTALTRVDGGSGLLSEQVGFFDLKTVDLV----
ARATH*   VYSAH----------GGEIAIAFLRGG-------VHIFSGPTFSPV----
VITVI    SYSAH----------GGEIAVAFLRGG-------VHIFSGPNFTPV----
SOYBN    VYSAH----------GGEIAIAFLRGG-------VHVFSGPNFTPV----
SOLLC    VYSAH----------GGEIAVAFLRGG-------VHVFSGPSFTPV----
ORYSJ*   VYSAH----------GGEVAVAFLRGG-------VHIFSGPNFEQV----
HORVU    VYSAH----------GGEVAVAFLRGG-------VHIFSGPNFDQV----
SORBI    VYSAH----------GGEVAVAFLRGG-------VHIFSGPNFDQV----
PHYPA    VFSAH----------GGELAVALFSGE-------VHVFSGSTMTPV----
                  1110      1120      1130      1140      1150
```

```
HUMAN*   ---AVFYSSA----------------APRPVDEPAMKRPRTAGPAVHLKA
CHICK    ---AVFHGSS----------------SQRPVDEQTIKRQRTTGPLVHFKA
DANRE    ---GMFYGSSGS--------------SQRVGEEPAIKRQRAGGPMLHFKA
CIOIN    ---VTQHLPVTTN-------------SQETTPGKAMKMTQQFG----VRH
TETNG    ---GVFYGSSTAS------------NAQRPGEESAIKRQRTGSPTVHFKA
DROME    -------------------------------NHTQFDRLSDASGAVKFVS
YEAST*   ---TERLISQSQL---------GKFKNIIASPLSAGFNYGKLPLPPSVEW
CANAL    ---NNSKNNNSVTGDEMELDNNDYPPKLISNLFDQGFQFPKISHKNRLV-
CANGA    ---TERLLNQGPQ---------GKFSNIITSILSAGFKYPTIKNLGTVEW
ASPNI    --------------------DGIDDANTVTSLAQAGFHYPLEPS---ALH
RHIO9    ---NNETISYAPNFWCVVQPKDSDD----------------------VVD
CRYNE    GDFPSLVVSQGAERGQLGLCAVVDQYRSHLGPVNKLDEQPLLGELPQSAS
USTMA    ----------------------QGDLSAVPAQALERCSNPVISSNGTLFA
ARATH*   ---ENYQINVGSA----------------------------------IAA
VITVI    ---DNYQINVGSA----------------------------------IAT
SOYBN    ---DNYQINVGSA----------------------------------IAA
SOLLC    ---DNYHIDVGSA----------------------------------IAS
ORYSJ*   ---DSYHVNVGSA----------------------------------IAP
HORVU    ---DSYHVNVGSS----------------------------------IAP
SORBI    ---DSYHVNVGSA----------------------------------IAP
PHYPA    ---DLFHVKVDPH----------------------------------LPT
                  1160      1170      1180      1190      1200
```

MED16

```
HUMAN*   MQLSWTSLALVGIDSHG-----KLSVLRLSPSMGHPLEVGL--ALRHLLF
CHICK    MQLSWTSLALAGIDSHG-----KLSMLRISPSMGHVLDMNT--SLRHLLF
DANRE    LQFSWTSLALVGIDNHG-----KLHMFRVSPSMGQMLDMNT--LLRHLLF
CIOIN    VSFSTLSCAMIAADKHG-----RLLLYRIPPWLTTPNRTNTMQSLKQLLQ
TETNG    LQFSWTSLALVGIDNHG-----KLYMLRVSPSMGQVLEMNT--TLRHLLF
DROME    GDLTPSSQILLIFDSHA-----QLYAMQAPLPKQGGSGLL----------
YEAST*   MKVSPSMCGVIVKQYNK-------KWPQFYAAVQKNYADPEKDSINATAL
CANAL    LAISPTMSSIVYTEIYGETVNLQLKPLERIDNFGTNSHDLYYTSVAF---
CANGA    IRVSPAMAGILYKYRHD-------DLPQFQPMNIDDVSDKSKDEINAATM
ASPNI    MSFSPSACLIVGLDNDG---QTHLRIMEHSYGAENGHYDENKYSAAIASL
RHIO9    IIFSPNETHLLYIHASR-----KIGIARATHNIFTEEHAKILSI------
CRYNE    DTEKYAACA-----------------------------------------
USTMA    -----TLASIHGGSSDKGVVMTKLPLLPCRPSAQHGNRTAEQVKTAEEES
ARATH*   PAFSPTSCCSASVWHDAAKDCAMLKIIRVLPPALPRNQSKVDQSTWERAI
VITVI    PAFSSTSCCSASVWHDTNKDRTMLKIIRVLPPAVPSSQVKANSSNWERAI
SOYBN    PAFSSTSCCSASVWHDSSKNCTILRIIRVLPPSIPISQAKANSSTWERAI
SOLLC    PAFSSTSCCSASVWHDTTRDCTILKIIRVLPPAVPSNQVKANSANWERAI
ORYSJ*   PAFSSSGCCLASVWHDTLKDRTILKIIRVLPPAILNAQTKVSSAVWERAI
HORVU    PAFSSSSCCLASVWHDTLKDRTILKIIRVLPPAILNVQTKVSSAVWERAI
SORBI    PAFSSSSCCLASVWHDTLKDRTILKIIRVLPPAILSTQTKISSAAWERAI
PHYPA    PAFSPTSCCLASVWHDKKTDVCTLRIAQIPPLLFTHTITFLTLSCNHQLL
                      1210      1220      1230      1240      1250
```

```
HUMAN*   ---LLEYC----MVTGYD--WWDILLHV---------------------Q
CHICK    ---LLEYC----MVTGYD--WWDILLHV---------------------Q
DANRE    ---LLEYC----MVTGYD--WWDVLLHA---------------------Q
CIOIN    ---LMLYC----LVSGKE--WWEILLHV---------------------H
TETNG    ---LLEYC----MVTGYD--WWDVLLHV---------------------Q
DROME    ---LLEYC----IVTGCD--ASDVLLL----------------------N
YEAST*   ---AFGYV----KSLHKQISAEDLTIAAKTHILRI-------SFLDRKRA
CANAL    ---AHRYA----FALYTSTCSDDLLLLIQSEINRIKDAVDGDTITRNKLG
CANGA    ---AFGYV----TSAHRQLSGEDIALACKQHILKI-------AKIDEKRA
ASPNI    ---TLAFC----RGCGGEVNTDDILLILIRQL-----------------S
RHIO9    ---KIQQC----LFNNMD--ASDLTSELVRLSKQP-------GFEDK--P
CRYNE    ---ATSII----LAERQDTNWSDVIHALEAIL-----------------P
USTMA    ---HLQMSQLLALSCLRKTDASDIGRAFAASA-----------------D
ARATH*   AE-RFWWS----LLVGVD--WWDAVGCTQSAAED---------------G
VITVI    AE-RFWWS----LLVGVD--WWDAVGCTQSAAED---------------G
SOYBN    AE-RFWWS----LLVSVD--WWDAVGCTQSAAED---------------G
SOLLC    AE-RFWWS----LLVGVD--WWDAVGCTQSAAED---------------G
ORYSJ*   ADSRFWWS----LLAGVD--WWDAVGCTQSAAED---------------G
HORVU    AD-RFWWS----LMAGVD--WWDAVGCTQSAAED---------------G
SORBI    AD-RFWWS----LLAGVD--WWDAVGCTQSAAED---------------G
PHYPA    DF-RFWWS----LVSEVD--WWDVVAATQAAAED---------------G
                      1260      1270      1280      1290      1300
```

```
HUMAN*   PSMVQSLVEKLHEEYTR-QTA-ALQ-QV-------LSTRILAMKASLCKL
CHICK    PNMVQNLVEKLHEEYTR-QSA-ALQ-QV-------LATRIVAMKASLCKL
DANRE    PGMVHNLLEKLHEEYMR-QNQ-ALQ-QV-------LSTRIVAVKASLCKL
CIOIN    PNLVEPLVKQLSVECGN-QTS-AVQ-QV-------LELRLQALKISIYHI
TETNG    PTMVHNLVEKLHEEYMR-QNL-ALQ-QV-------LATRILAVKASLCKL
DROME    LGNLEALVEKLTDNFTR-QPS-FVR-HF-------YYANFLALKSNICRM
YEAST*   KEFITTLLKSLYSFFNI-SPDAPKEIMDKIITS-RPLQKIMLLQLELG--
CANAL    KQLCDSVIIECHKAINFHLDTITKESLDKLLSNNASLQKLLSLQL-----
CANGA    KDFTTTLMFNLYNFFNF-SPGAPKELMDKIISS-RPLQKVMLLQLELG--
ASPNI    PDAQTAFLNEVYRALSI-NCNFTTE-QDKLMNH-PYIPRALSIQAALG--
RHIO9    QEIIDKVLSTYESHYQD-EEEWNVG-QLQKAYG-LALTVFTR--------
CRYNE    ASSRGEFIPLVLQRIYD-LAAKEIH-ID-------QLHLVSRVQIAL---
USTMA    RENAGKLLVQVGDLLKI-TES--KR-QD-------VLVKIEGAT------
ARATH*   IVSLNSVIAVMDADFHS-LPSTQHR-QQ-------YGPNLDRIKCRLLEG
VITVI    IVSLNSVIAVLDADFHS-LPSTQHR-QQ-------YGPSLDRIKCRLLEG
SOYBN    IVSLNSVIAVLDADFHS-LPSVQHR-QQ-------YGPSLDRIKCRLLEG
SOLLC    IVALNSVIAVLDADFHS-LPSTQHR-QQ-------YGPSLDRIKCRLLEG
ORYSJ*   IVSLNSVIALLDADFHC-LPTIQQR-QQ-------HCPNLDRIKCRLLEG
HORVU    IVSLNSVIALLDTDFHC-LPTMQQR-QQ-------HCPNLDRIKCRLLEG
SORBI    IVSLNSVIAFLDADFHS-LPTMQQR-QQ-------HCPNLDRIKCRLLEG
PHYPA    HVTVAKVMSVLDADFHS-LQP-QQR-HH-------YGPALDRIKCRVLEG
                      1310      1320      1330      1340      1350
```

MED16

```
HUMAN*   SPCTVTRV--CDYHTKLFLIAISSTLKSLLRPH-FLNT----PDKSPGDR
CHICK    SSSTIARV--CDYHAKLFLIAISCTLKSLLRPH-FLNT----PDKSPGDR
DANRE    SSATAARA--CDFHAKLLLIAISSTLKSLLRPH-VLNT----PDKSPGDR
CIOIN    TASPVA----TDLHLKMLFQAISLVFRSLVRPHQQVGG----EEIDPATK
TETNG    STATAARA--CDFYAKLLLIAISSTLKSLLRPH-VLNT----PDKSPGDR
DROME    QQ--------QEFDNLIILHAISTTFKSLLRPS-DLGF----QDKGPADN
YEAST*   --------------------------------------------------
CANAL    ----------------------------------ILGEFQQSSHFPNNYV
CANGA    --------------------------------------------------
ASPNI    ----------------------------------FKGK------------
RHIO9    ----------------------------------LPGKQVQSTNLARGLQ
CRYNE    ------------------------------------------FSAFKDAR
USTMA    ----------------------------------QKGPSHDSNSIPFHQA
ARATH*   TNAQEVRAMVLDMQARLLLD--------------MLGKGIESALVNPSAL
VITVI    INAQEVRAMVLDMQARLLLD--------------MLGKGIESALMKPSAL
SOYBN    SNAQEVRAMVLDMQARLLLD--------------MLGKGIESALINSSAL
SOLLC    TNAQEVRAMVLDMQARLLLD--------------MLGKGIESALTNPSAL
ORYSJ*   TNAQDVRALVLDMQARLLLD--------------MLGKGIESALINPSTL
HORVU    TNAQDVRALVLDMQARLLLD--------------MLGKGIESALINPSTL
SORBI    TNAQDVRALVLDMQARLLLD--------------MLGKGIESALTNPSTL
PHYPA    VQAADVRVLVLDMQGRLMLD--------------MLGRGIEAALMNPSTL
               1360      1370      1380      1390      1400
```

```
HUMAN*   LTEICT---KITDVDIDKVMINLKTEEF----------------------
CHICK    LTEVCS---KITDIDIDKVMINLKTEEF----------------------
DANRE    LSEICA---KNTDTDIDKVMINLKTEEF----------------------
CIOIN    LQNISR---TSTETNIDQLVQNVDTDP-----------------------
TETNG    LTEICA---KNTDTEIDKVMINLKTEEF----------------------
DROME    LAIKLA----ESIPDVDTVMLNLDAKDF----------------------
YEAST*   -----SCFSQENIEEMARVILYLKNVLFAFNGVARNFHFAIEQISNNSNQ
CANAL    VP------------DIAWIVLNLRSASL----------------------
CANGA    -----SIFTDENTCEMARVILYLKNVSFAFNGVARNLQFAIEQMTNSTNS
ASPNI    ------FRARNIASNVPWAIIQLRQAAMIYPSFFQYNKGVQGP-------
RHIO9    LPVILECFISSCSSNYKDVIRMFNKEDVHL--------------------
CRYNE    LALATDIFRLNEASELVDRCATFQDDGS----------------------
USTMA    LRLLRIRMVMDNSSLIPKPAARIERLVLELGLCYQILRLARVKTEFTMAP
ARATH*   VFEPWR--------VDGETITGINPEAM----------------------
VITVI    VPEPWQ--------ATGETLSGIDPEEM----------------------
SOYBN    VPELWQ--------ASGETLSSIDPESM----------------------
SOLLC    VPEPWQ--------ASSETLFGIDPEAM----------------------
ORYSJ*   LPEPWQ--------ASSDMLSSIGPDKM----------------------
HORVU    LPEPWQ--------ASSELLSNIEPDKM----------------------
SORBI    LPEPWQ--------ASSDMLSSIEPDKM----------------------
PHYPA    IAEPWQ--------ASSDTLSGLGPDAM----------------------
               1410      1420      1430      1440      1450
```

```
HUMAN*   --------------------------------VLDMTHCRRCSSSCSGWA
CHICK    --------------------------------VLEM-HCSLCSSSSSGWG
DANRE    --------------------------------VLDG-PPLQSLQQLIQWV
CIOIN    -------------------------------------QTLQSMQQLIQWV
TETNG    --------------------------------VLDG-PPLQSLQQLIQWV
DROME    --------------------------------TVEP-VMLQSLQQLIQWV
YEAST*   QQNP----KLFQT-------------------IFSKQDLIHSLIPVAKWF
CANAL    -------GIMFTLSSIYRQVSKKKPSEDTLQDSITRGECIMSIIGNFKWL
CANGA    SNPPLSGDKFFQT-------------------AFSKQDLVHSLIPVTKWF
ASPNI    --------------------------------EPHDPDVLRLVLGNTKWA
RHIO9    --------------------------------EFEP-NSLWSLVLLSTWI
CRYNE    --------------------------------ITFDLDSIWPLITVFDWA
USTMA    SPPAPEGGKKKGGSAVKAAAAAAAAAGETRDRVYYRLESVWPLLAQLQWF
ARATH*   --------------------------------AVDP-ALVSSIQAYVDAV
VITVI    --------------------------------AVDP-ALVFSIQAYVDSV
SOYBN    --------------------------------AVEP-ALIPSIQAYVDSV
SOLLC    --------------------------------AVEP-SLVPSIQAYVDAI
ORYSJ*   --------------------------------TVDP-ALLLSIQGYVDAV
HORVU    --------------------------------TVDP-ALLPSIQGYVDAV
SORBI    --------------------------------TVEP-ALLPSIQGYVDAV
PHYPA    --------------------------------AVDG-ALVPTIQAYVDAV
               1460      1470      1480      1490      1500
```

MED16

```
HUMAN*  TSCCTCWPAYPTSPAPPRSPAPPRSPPPPRSPPPPRSPPLHEASAGSLLR
CHICK   ILCSIYWPACLTS-----SSRLCLSPDPKMS-----------SLQGSPVR
DANRE   GDFVLYLLANLPN-------------------------------QGSIVR
CIOIN   ADVALYLLASVPL-------------------------------YKQANR
TETNG   GDFVLYLLANLPN-------------------------------QGSMVR
DROME   TDLALNMLHRLPE--------------------------EVMKTKLSGKR
YEAST*  VKFITYLTQEILI------------------------------LINDPTN
CANAL   IDLLVYLNQELLQ-----------------LIYVKNNFLNNGNTTTSKLT
CANGA   VKFITYLIQQILI------------------------------LTNDPEN
ASPNI   LDFSLYILNELFD-----------------LADDFESVSSDQEAFNQKLK
RHIO9   FDYVKWVLHEWNM-------------------------------------
CRYNE   IGVIARAMREAIL-----------------VGASAEWQGSDDSLMIDPCS
USTMA   LALLDGISKLALA---------------TTAGNNTSKTSSDANAVHAKTS
ARATH*  LDLASHFITRLRR-------------------------------------
VITVI   LDLASHFITRLRR-------------------------------------
SOYBN   LDLASHFITRLRR-------------------------------------
SOLLC   LDLASHFITRLRR-------------------------------------
ORYSJ*  LDLASHFITRLRR-------------------------------------
HORVU   LDLASHFITRLRR-------------------------------------
SORBI   LDLASHFITRLRR-------------------------------------
PHYPA   LDLASHFLTRLRR-------------------------------------
                 1510      1520      1530      1540      1550
```

```
HUMAN*  PGH-------SFLRDGTSLGMLRELMVVIR---------IWGLL-----K
CHICK   PGH-------SFLRDGASLGMFRELMVVIR---------IWGLL-----K
DANRE   PGF-------GFLRDGASLGMLREMLVMIR---------IWGLL-----K
CIOIN   PGY-------SLYWDRDWLSLLREMLVLIR---------VWGLL-----K
TETNG   PGF-------GFMRDGASLGMLREMLVMIR---------IWGLL-----K
DROME   PSY-------DISRDIVAISSLRELLVMIR---------IWGLL-----N
YEAST*  KEY-------TLVHGIFGAKMSRTLILSILNEIKKVTQ-IVAKF-----P
CANAL   LSN-------SIVLPLILNKVSRLFLMYAISAMGRTHE-ILKKLHKDLTE
CANGA   PDN-------RLVLGVFGAKIPRTLILTILKEIKNVTA-IITKF-----P
ASPNI   TTT-------SLPLIILLSSMSRAFLRFICRGLRGIHA-----------G
RHIO9   ----------LFHSKKSQLSESREYLIKILMLIQHF--------------
CRYNE   P------------LLLLLHPILRSLVLRLLSQFHQLSI-FLSTLERPILQ
USTMA   STTLDENSLEADFVRMLNKPTLRRLVLQIVAHFCDFEQWIKSTTKKSNLP
ARATH*  -----------------YASFCRTLASHAA--SAGTGS-NRNNVTSP-TQ
VITVI   -----------------YASFCRTLASHAV--TAGTGS-NRSAVASP-TQ
SOYBN   -----------------YASFCRTLASHAV--TAGSGS-NRNMVASP-TQ
SOLLC   -----------------YASFCRTLASHAV--TAGTGG-SRSMVTSP-TQ
ORYSJ*  -----------------YASFCRTLASHAVGASSGSGN-SRNMVTSP-TN
HORVU   -----------------YASFCRTLASHAVGASSSSGN-SRNMVTSP-TN
SORBI   -----------------YASFCRTLASH-VGPSSTTGA-SRNMVTSP-TN
PHYPA   -----------------YASFCRTLTAHAASG------------------
                 1560      1570      1580      1590      1600
```

```
HUMAN*  PSC--LP-----------VYTATSDTQDSM--------------------
CHICK   PSC--LP-----------VYTATSDTQDSM--------------------
DANRE   PGC--LP-----------IYTATSDNQDSM--------------------
CIOIN   SAC--SP-----------VFNVLDSRMDVL--------------------
TETNG   PGC--LP-----------TFTAMSDNQDSL--------------------
DROME   TQC--LP-----------VYTKTMDNIDVL--------------------
YEAST*  ETS--YP-----------ILNESSTFLKLV--------------LSESPV
CANAL   ANKLFAP------------MKESLNRYFSI--------------SNNSPI
CANGA   ETN--YP-----------ILNESSTYLKML--------------LIESPI
ASPNI   YAN--AP-----------LTGDSRVYYTEI-----------CQTLDASPV
RHIO9   -----IR-----------YTNEADYQLDHL--------------------
CRYNE   PESKTLP-----------ASNTRDPMATVV--------------------
USTMA   PATGAVPGDETGGGFSASPEASTEARVLAVSEQMALARDALHSVVGDASI
ARATH*  NAS--SP-----------ATPQVFPDKSLYLAVGQPTTTTTTTA-TTNSS
VITVI   SSA--SP-----------ATSQ-----------------GGQSG-TTSST
SOYBN   SSA--TP-----------ATSQ-----------------GGQNG-TSSSM
SOLLC   SAS--SP-----------ATSQ-----------------GAQGG-TASSA
ORYSJ*  SSP--SP-----------STNQ----------------------------
HORVU   NSP--SP-----------SNNQ-----------------GNQGG-VASAT
SORBI   SSP--SP-----------SNNQ-----------------GNQGGSTTSAT
PHYPA   ---------------------------------------GAPAG-AASSG
                 1610      1620      1630      1640      1650
```

MED16

```
HUMAN*   ------SLLFRLLTKLWICC--------------------------RDEG
CHICK    ------SLLFRLLTKLWLCC--------------------------REES
DANRE    ------FLLFRLLTKLWLCS--------------------------RDES
CIOIN    ------SHVFKLVTQLWMAS--------------------------TDDH
TETNG    ------QLLFRLLTKLWLCA--------------------------RDDG
DROME    ------VILFRLLTRLAQN-------------------------------
YEAST*   DFEKFETFLVDVNNKFIALC------------------------------
CANAL    TVNLFESYL-----------------------------------------
CANGA    NFEKFETFLMDVNNKLSAFS------------------------------
ASPNI    RTDVYEKLLAGVDSAVKHAY------------------------------
RHIO9    ------SESQHILQQYANTL------------------------------
CRYNE    --------------------------------------------------
USTMA    ELAEFAQLLVKIEADWNVGG------------------------------
ARATH*   GSSHVQAWMQGAIAKISSSNDG-----SNSTASPISGSPTFMPISINTGT
VITVI    GSTQMQAWVQGAIAKISSTTDGV----PNSTPNPINGPPSFIPISINTGT
SOYBN    GSAQLQTWVQGAIAKISNTTDGG----SNPTPNPISGPSTFMPISINTGT
SOLLC    GSTQMQAWVQGAIAKISSTADSV----PSSAPNPITGPSTFMPISINTGT
ORYSJ*   --------------------------------------------------
HORVU    GSSQMQEWVQGAIAKISNNADGA----ANAAPNPVSGRSSFIPISINTGT
SORBI    GNSQMQEWVQGAIAKISNNSDGA----VTATQNQMSTRSSFMPISINTGT
PHYPA    GAAQVQAWVQGAIAKINSSSTNTDATTSAVSSTTVAGPATNLPLSVSTAT
                  1660      1670      1680      1690      1700
```

```
HUMAN*   PASEPDEALVDECCLL----------------------------------
CHICK    HITEPDDTLIDECCLL----------------------------------
DANRE    HPQDPDETLIDECCLL----------------------------------
CIOIN    RMTDLPTSLINECSAL----------------------------------
TETNG    PPQDPDESLIDECCLL----------------------------------
DROME    -PAEPDEMLLDECSLL----------------------------------
YEAST*   ---EQQPSQEREFSLL----------------------------------
CANAL    ----------RECDAL----------------------------------
CANGA    ---EQQPSIMREPTLL----------------------------------
ASPNI    --------------------------------------------------
RHIO9    -KTDPPIAIKEVINLL----------------------------------
CRYNE    -----AREQIRDIPLR----------------------------------
USTMA    --------------------------------------------------
ARATH*   FPGTPAVRLIGDCHFLHRLCQLLLFCFLQRSSRFP------QRNADVSSQ
VITVI    FPGTPAVRLIGDCHFLHRLCQLLLFCFFFRRTQLPRVIGVAQRNADTNMH
SOYBN    FPGTPAVRLIGDCHFLHRLCQLLLFCFFFRRTQLPRYI---NRTSDTNIQ
SOLLC    FPGTPAVRLIGDCHFLHRLCQLLHFCFFFRGTQLPRFMGAAQRNADSSMQ
ORYSJ*   --------------------------------------------------
HORVU    FPGTPAVRLIGDCHFLHRLCQLLLFCLLFRRRQSPRLLANAQRNPDSAMQ
SORBI    FPGTPAVRLIGDCHFLHRLCQLLLFCLLFRRRQSPRLLANLQKNQDSAIQ
PHYPA    FPGTPAVRLISDCHFLHRLCQLLLFCLIFRKRQLPRHVQISAGRPAASVD
                  1710      1720      1730      1740      1750
```

```
HUMAN*   --------------------------------------------------
CHICK    --------------------------------------------------
DANRE    --------------------------------------------------
CIOIN    --------------------------------------------------
TETNG    --------------------------------------------------
DROME    --------------------------------------------------
YEAST*   --------------------------------------------------
CANAL    --------------------------------------------------
CANGA    --------------------------------------------------
ASPNI    --------------------------------------------------
RHIO9    --------------------------------------------------
CRYNE    ----------------------------------------------QGVD
USTMA    --------------------------------------------------
ARATH*   KLQTG--ATSKLEEVN----SAKPTPALNRIEDAQGFRGAQLGTGVKGID
VITVI    KPQAT--GTSKVEENST--VSNKLASAMARSEEGQVARANHAINGAKGAE
SOYBN    KPQSNTPAPGKVEEIA------KPVSAVVKSDDGQTGR-----TGAKGAE
SOLLC    KPQSG--IPGKTEDSN---------SGAKPTLGGQVG------TGAKGSE
ORYSJ*   --------------------------------------------------
HORVU    KIQHL--MNSKIEDSSSAISAVRSGLGAAKVEDGAATRG-QLVLGAKGLE
SORBI    KIQHM--MNAKTEDNS---TTVRSGLGAAKVEDGQSTRGGQFVLGTKGPE
PHYPA    GTNKL--GNIKEE------PLIGQRTGLVKVEEGGHTVTRTTSTVSKGAE
                  1760      1770      1780      1790      1800
```

MED16

```
HUMAN*   -------------------------PSQLLIPSLD------WLPASDGLV
CHICK    -------------------------PSQLLIPNID------WLPINDGII
DANRE    -------------------------PSQLLVPSMD------WLPINDGVI
CIOIN    -------------------------PFHIIIPPLE------PTPPPNSVL
TETNG    -------------------------PSQLLVPSMD------WLPVNDGII
DROME    -------------------------SKQLLIPQ--------PTKFNPTTL
YEAST*   -------------------------VKAEIPPEYAKVGDFLLQYANNAVI
CANAL    ---------------------------------LNKEVPQKILTANNSIN
CANGA    -------------------------VRSSVPNELNKITEFLLQYSSNTVI
ASPNI    -------TGAGFGDNERPGPEKELLVSSRIPPVLVPAVSTILRQTVPAMR
RHIO9    -----NDLNSIESNTKSKYYFIHIIQSNFIIIVNNRWTVLLKSTLEGYSI
CRYNE    VEQWGRALESLTTTSEQKDIDKSLIELSLTPLQPQIPTLINILHTSSNLF
USTMA    --------------------------------------------------
ARATH*   ENSARTT-KMGSGNAGQGYTYEEVRVLFHILMDLCKRTSGLAHPLPGSQV
VITVI    EGPTGRS-RLGSGNAGQGYTFEEVKVLFLILMDLCRRTSALQHPLPISQV
SOYBN    EVPSGRS-RLGSGNAGQGYTFEEVKVLFMMLMDLCRRTAGLQHPLPVSQV
SOLLC    EGPSKRS-RIGSGNAGQGYTYEEVKVLFLILMDLCRRTAGLVHPLPVSQV
ORYSJ*   --------------------------------------------------
HORVU    ENPMGKSVRIGSGNAGQGYTSDEVKVLFLILVDLCRRTSGLQHPLPVSQV
SORBI    ENPIGKSVRIGSGNAGQGYTSDEVKVLFLILVDLCRRTSTLPHPLPASQV
PHYPA    DIATSRAQRVGLGNCGQGYTPDEVKYLFLVLVDLCKRTAPLPHPLPKSQL
                  1810      1820      1830      1840      1850

HUMAN*   S----RLQPKQP-------LRLQFG-----------RAPTLPGSAATLQ-
CHICK    S----KLQNKQL-------VRLQFG-----------KAPGLIGHTVSSQ-
DANRE    T----KIQGKHQ-------LRLQFG-----------KPYTLPGVNPNAQ-
CIOIN    S----QMRNFSGT------RRFTFG-----------ETPQDSYIMLSRQH
TETNG    V----KLQGKHP-------LRLQFG-----------KASSLSGGAGGATS
DROME    L----SAQGFAAVKS----GQLQFTSMEEPTCLQDMETEEVVF-------
YEAST*   S----HANA----------AAVYFA--------------DTSGLKISNSE
CANAL    G----NVNGNSGTSTNKPYSALKFE-----------QKLLIKGIDVNDDN
CANGA    S----HADA----------SAIYFS--------------DTSGLRISCDE
ASPNI    G----EMDR----------LAIFMG-----------D-------------
RHIO9    P----QLRN----------ISVKYK-----------DKCTKPCIYIKKEI
CRYNE    TSEYFQLDASAGSSTSLAYDAIDWSVLQEHGHRDDDDGGDDDGEAGDKDK
USTMA    -----TGGAASGANQSRETDRFWWA-TLDGSKVDQLDGASAVKLEQDPNT
ARATH*   G----SGNIQ---------VRLHYI-----------DGNYTVLPEVVEAA
VITVI    G----SNNIQ---------VRLHYI-----------DGSYTVLPEVVEAS
SOYBN    G----SNNIQ---------VRLHYI-----------DGNYSVQPEVVEAS
SOLLC    G----SNNIQ---------VRLHYI-----------DGNYTVLPEVVEAS
ORYSJ*   --------------------------------------------------
HORVU    G----TSNII---------IRLHFI-----------DGTYTVLPEVVEAS
SORBI    G----SSSII---------IRLHYI-----------DGNYTVLPEVVEAS
PHYPA    Q----SANPI---------LRLHFI-----------DGQFTVAPEVVEAS
                  1860      1870      1880      1890      1900

HUMAN*   -LDGLARAP-----------------------------------------
CHICK    -FDAFVRAP-----------------------------------------
DANRE    -VEIF-RSP-----------------------------------------
CIOIN    MMDHVAVAG-----------------------------------------
TETNG    PLELFNRSP-----------------------------------------
DROME    --------------------------------------------------
YEAST*   FFNPEIFHLLQPLEEGLIIDTDKLPIKNRTSKSFSKLLYDDVTC------
CANAL    DSSTKISNTIIEELSNMILDRYSISISRET--KLSELMFYDTDWLNIGIN
CANGA    FFEPGVHRLLQPIEDGIVIDEKNMPATFRDSKTFTKLTYDGITY------
ASPNI    --------------------------------------YGWLGFGNDARS
RHIO9    IGKY----------------------------------------------
CRYNE    --------------------------------------------------
USTMA    GLQAKLLSSLVDPQSGALVDVLTLFISPSDISDEHNVLYDKGALDDGVGD
ARATH*   LGPHMQNMPRPRGADAAGLLLRELELHPPS--------------------
VITVI    LGPHMQNMPRPRGADAAGLLLRELELHPPA--------------------
SOYBN    LGPHMQNMPRPRGADAAGLLLRELELHPPA--------------------
SOLLC    LGPHMQNMPRPRGADAAGLLLRELELHPPA--------------------
ORYSJ*   --------------------------------------------------
HORVU    --------------------------------------------------
SORBI    LGPHMQNMPRPRGADAAGLLLRELELQPPA--------------------
PHYPA    LGPHMQNLPRPRGADAAGLLMRELELHPPA--------------------
                  1910      1920      1930      1940      1950
```

MED16

```
HUMAN*   ----GQPKIDHLRRLHLGACPT----------------------------
CHICK    ----GQPKIDHLRRLHLGAYPT----------------------------
DANRE    ----ASQRVDHLRCLHLGISPT----------------------------
CIOIN    ----SSGKVDSLRRIFLGKSPN----------------------------
TETNG    ----GSQKMDNLRCVHMGVCPT----------------------------
DROME    ----ASSVKDGVSNLQLGARP-----------------------------
YEAST*   ---------DKLSVSEISD-------------------------------
CANAL    KKEVPSYTIDALRKLIISDSSTSTSLAKG---------------------
CANGA    ---------DRFSKEELSD-------------------------------
ASPNI    ELYRRERDVDIIKKTPIASRS-----------------------------
RHIO9    ---------DPIRKRRLTED------------------------------
CRYNE    --------------------------------------------------
USTMA    QVGTRSTLIDALSSLSAKRSDVDERRDVVRKVLLQHAVTQASLVNDAATL
ARATH*   ---------EEWHRRNLFGGPGSEPEDMILTDDVSKL--SNSLDLPDTNF
VITVI    ---------EEWHRRNMFGGPWSDPEDMGSVDDTPKL--STYADPLESNS
SOYBN    ---------EEWHRRNMFGAPWSDPEDVDCANDTPKL--VNS-DPLDFSS
SOLLC    ---------EEWHRRNMFGGPWSDPED---VDDNSKL--SPSRDLTQYSS
ORYSJ*   --------------------------------------------------
HORVU    --------------------------------------------------
SORBI    ---------EEWHRRNMFGGPWSEPDDFGPLDNMPRLKISGSINSHLSDM
PHYPA    ---------EDWNRRMLAGGPV--------------------FAFLENEY
                  1960      1970      1980      1990      2000
```

```
HUMAN*   --------------------------------------------------
CHICK    --------------------------------------------------
DANRE    --------------------------------------------------
CIOIN    --------------------------------------------------
TETNG    --------------------------------------------------
DROME    --------------------------------------------------
YEAST*   --------------------------------------------------
CANAL    --------------------------------------------------
CANGA    --------------------------------------------------
ASPNI    --------------GKGSGLGSVEAVMGLGARNGNGS-------------
RHIO9    --------------------------------------------------
CRYNE    --------------------------------------------------
USTMA    LKTRPTTVVGASAAQRSQSALPSSAAANEPAQAESNLIRLSA--------
ARATH*   SGICDGYNRVHSLWPRKRRMSERDAAFGSNTSVGLGAYLGIMGSRRDVVT
VITVI    FENNDTYYGARGLWPRKRRLSERDAAFGLNTSVGLGAYLGIMGSRRDVVT
SOYBN    LEHCDVYYGTHRLWPRKRRMSERDAAFGLNTSVGLGGYLGIMGSRRDVVT
SOLLC    LENCDVYYGAHGLWPRKRRMSERDAAFGLNTSVGLGAYLGIMGSRRDVVT
ORYSJ*   --------------------------------------------------
HORVU    --------------------------------------------------
SORBI    EEDAGSSVGIHSLWPRKRRLSERDAAFGLKTSVGLGGYLGVMGSRRDVVT
PHYPA    EETENLDPSPIDFWPRKRRRVEHDAAFGLRTAVGLGSFTTLLGSRRDVIT
                  2010      2020      2030      2040      2050
```

```
HUMAN*   --------EECKACTRCGCVTMLKSPNRTTA-------------------
CHICK    --------EECKSCTRCGCVTMLKSPNKVTA-------------------
DANRE    --------EDSKACTRCGCVTMLRSPNKTNA-------------------
CIOIN    --------QELKECVRCGVTTTCNNLLRSTL-------------------
TETNG    --------EDSKACTRCGCVTMLRSPNKTNA-------------------
DROME    --------SHIRRCARCGFVF------VNNASKVAKTSA-----------
YEAST*   --------GKLKRCSRCGSVTRAGNIISSDKTIVPTSIQ-----------
CANAL    --------EKLRVCTRCRAVSLVGDV------------------------
CANGA    --------NKLKRCNRCGAVTKAGYPIPTNKTIVPTSIS-----------
ASPNI    --------QLSRRCVRCCEVSEAMYP----------PRS-----------
RHIO9    --------VKTSLCTRCHQRI----LPVHTESDDLDPCS-----------
CRYNE    --------MTVVVCDRCGWRTEALTMSVPAASGIKTHET-----------
USTMA    --------MRAKRCVRCHALSQDPIIATYSLAPPPTSSTNPAAIMVDPNL
ARATH*   ATWKTGLEGVWYKCIRCLRQTSAFASPGA-TKQPNPNER-----------
VITVI    AVWKTGLEGVWYKCIRCLRQTSAFAADAT-N-QPNQNER-----------
SOYBN    ATWKTGLEGVWYKCVRCQRQTSAFTSPDA-TASPSQNDR-----------
SOLLC    AVWKTGLEGVWYKCIRCLRQTSAFASPGG-NPSANQHEK-----------
ORYSJ*   --------------------------------------------------
HORVU    --------------------------------------------------
SORBI    AVWRTGLDGEWYKCIRCLRQTCAFAHPGA-PNQ--TNER-----------
PHYPA    SQWKPPSHGVWHKCLRCGRQTSALTVPSKNTAQTSPPFA-----------
                  2060      2070      2080      2090      2100
```

MED16

```
HUMAN*   - - - - - - - - - - - - - - - - - - - - - - - - - - - - - - - - - - - - - - - - - - - - - - - - - -
CHICK    - - - - - - - - - - - - - - - - - - - - - - - - - - - - - - - - - - - - - - - - - - - - - - - - - -
DANRE    - - - - - - - - - - - - - - - - - - - - - - - - - - - - - - - - - - - - - - - - - - - - - - - - - -
CIOIN    - - - - - - - - - - - - - - - - - - - - - - - - - - - - - - - - - - - - - - - - - - - - - - - - - -
TETNG    - - - - - - - - - - - - - - - - - - - - - - - - - - - - - - - - - - - - - - - - - - - - - - - - - -
DROME    - - - - - - - - - - - - - - - - - - - - - - - - - - - - - - - - - - - - - - - - - - - - - - - - - -
YEAST*   - - - - - - - - - - - - - - - - - - - - - - - - - - - - - - - - - - - - - - - - - - - - - - - - - -
CANAL    - - - - - - - - - - - - - - - - - - - - - - - - - - - - - - - - - - - - - - - - - - - - - - - - - -
CANGA    - - - - - - - - - - - - - - - - - - - - - - - - - - - - - - - - - - - - - - - - - - - - - - - - - -
ASPNI    - - - - - - - - - - - - - - - - - - - - - - - - - - - - - - - - - - - - - - - - - - - - - - - - - -
RHIO9    - - - - - - - - - - - - - - - - - - - - - - - - - - - - - - - - - - - - - - - - - - - - - - - - - -
CRYNE    - - - - - - - - - - - - - - - - - - - - - - - - - - - - - - - - - - - - - - - - - - - - - - - - - -
USTMA    A P Q V Q M Q L Q M Q M Q M H M M H M Q L Q M Q H Q Q Q P S Q P G I V L P P M Q P I P T F L P L I Q
ARATH*   - - - - - - - - - - - - - - - - - - - - - - - - - - - - - - - - - - - - - - - - - - - - - - - - - -
VITVI    - - - - - - - - - - - - - - - - - - - - - - - - - - - - - - - - - - - - - - - - - - - - - - - - - -
SOYBN    - - - - - - - - - - - - - - - - - - - - - - - - - - - - - - - - - - - - - - - - - - - - - - - - - -
SOLLC    - - - - - - - - - - - - - - - - - - - - - - - - - - - - - - - - - - - - - - - - - - - - - - - - - -
ORYSJ*   - - - - - - - - - - - - - - - - - - - - - - - - - - - - - - - - - - - - - - - - - - - - - - - - - -
HORVU    - - - - - - - - - - - - - - - - - - - - - - - - - - - - - - - - - - - - - - - - - - - - - - - - - -
SORBI    - - - - - - - - - - - - - - - - - - - - - - - - - - - - - - - - - - - - - - - - - - - - - - - - - -
PHYPA    - - - - - - - - - - - - - - - - - - - - - - - - - - - - - - - - - - - - - - - - - - - - - - - - - -
                             2110                2120                2130                2140                2150
```

```
HUMAN*   - - - - - - - - - V - - - - K Q W E Q R W I K N C - L C G G L W W R V P L S Y P - - -
CHICK    - - - - - - - - - V - - - - K Q W E Q R W V K N C - L C G G L W R R M P L S Y - - - -
DANRE    - - - - - - - - - T - - - - R Q W E Q R W I K N C - L C G G L W R R I P S T L S - - -
CIOIN    - - - - - - - - - - - - - - K V W D Q R F V S S C - V C G G H W K K L N S H P V P L H
TETNG    - - - - - - - - - M - - - - K Q W E Q R W I K N C - L C G G L W R R I P - - - - - - -
DROME    - - - - - - - - - L - - - - K A W F S K W L - H C - H C G G F W K Q V H - - - - - - -
YEAST*   - - - - - - - - - T - - - - K R W P T M Y T R L C - I C S G M L F E M D G - - - - - -
CANAL    - - - - - - - - - T - - - - G L W T M V F Q R T C - M C G N A W V N V - - - - - - - -
CANGA    - - - - - - - - - T - - - - R R W P T M Y T R M C - I C L G M L Y E L - - - - - - - -
ASPNI    - - - - - - - - - V L S F K M T L K L G H L R S C - I C G G M W N L E G G L P G - - -
RHIO9    - - - - - - - - - S - - - - A Q W Y Q S L R K R C - V C G G I Y I S I - - - - - - - -
CRYNE    - - - - - - - - - T I S P W M E W K K Q S E A N C - I C G G T W V R K Q V E I E - - Y
USTMA    P P P K L V S I D M P A R A S S L M D R F R F T C - L C G G S W W V L - - - - - - - -
ARATH*   - - - - - - - - - E - - - - T W W T S R W V Y C C P M C G G T W V R V V - - - - - - -
VITVI    - - - - - - - - - E - - - - M W W I S R W A Y G C P M C G G T W V R V V - - - - - - -
SOYBN    - - - - - - - - - E - - - - M W W I S R W V H S C P M C G G T W A R V V - - - - - - -
SOLLC    - - - - - - - - - E - - - - V W W I S R W A C G C P M C G G T W V R V V - - - - - - -
ORYSJ*   - - - - - - - - - - - - - - - - - - - - - - - - - - - - - - - - - - - - - - - - - - -
HORVU    - - - - - - - - - - - - - - - - - - - - - - - - - - - - - - - - - - - - - - - - - - -
SORBI    - - - - - - - - - E - - - - A W W I S R W S H A C P M C G G S W V K V V - - - - - - -
PHYPA    - - - - - - - - - T - - - - E M W N G R W P H G C P M C G G R W S R I V - - - - - - -
                             2160                2170                2180                2190
```

MED17

```
HUMAN*  -----------------------------MSGVRAVRISIES--------
CHICK   -----------------------------MAGVPAVRISIES--------
XENTR   -----------------------------MSGVPAVNISIES--------
DANRE   -----------------------------MSGGPAVRVSIES--------
CIOIN   -----------------------------MSNPLNVKISLQP--------
ANOGA   -------------------------------MSLSANISVEA--------
TETNG   -----------------------------MSGGPAVRISIES--------
DROME   -------------------------------MSNSVNISVET--------
BRUMA   -------------------MFGTGQPTSSTSQPAGVNIAIEA--------
CAEEL   ---------MEDGDEVVEPIEPESEDIVNLTKRLGVDLVLES--------
YEAST*  -------------M------TTEDPDSNHLSSETGIKLALDPNLITLALS
SCHPO   ----------------------MAEEANKDADISSLSLSLDP--------
CANAL   ------------------------------MVEKQFNIDLEL--------
CANGA   ----------------------MTQLTENTDELHSIRLAIDPNLITLPIG
ASPNI   -------------------------------MSDSFTVSLRP--------
RHIO9   --------------------------MESEPTAKRVKLSLEP--------
CRYNE   ------------------MNGISEQRSSPKTVFEDLRLSIDPTTIRLA--
USTMA   ----------MSDA------SSASAGLSNRVVLDEVPLAVERVHVYTDSD
ENCCU   MEPKALVLNPRSLMDFTGNEEERFRKMLKSGKKEELLMELSK--------
ANTLO   ----------MLCF------SDKDREVLRTSTPQALAEVLEQ--------
ARATH*  --------------------------M---DS--DMEISLDR--------
VITVI   --------------------------M---DG--KMEISLDK--------
SOYBN   --------------------------M---DDGMQLQISLDK--------
SOLLC   --------------------------M---NG--NLEISVDK--------
ORYSJ*  --------------------------M---EE--AVRVDLDK--------
HORVU   --------------------------MAAMGD--DLPLDFDK--------
SORBI   --------------------------M--EKD--DVRVDLDK--------
SELML   ----------------------------------MAKVTLDS--------
PHYPA   -----------------------------------MEVSLDP--------
VOLCA   ---------------------------------------MEP--------
                 10        20        30        40        50
```

```
HUMAN*  ------------------ACEKQVHEVGLDGTETYLPPLSMSQN------
CHICK   ------------------ACEKQVQEVGLDGSETYLQPLSMSQN------
XENTR   ------------------SAEKQVQEVGLDGSETYVLPLSMSQN------
DANRE   ------------------SCERQVQEVSLDGMETYVPPLSMSQN------
CIOIN   ------------------LSETKIQEIAYNGRETFIPPLSMSES------
ANOGA   ------------------PIENQIQEIAYDGTEIYQLPPTLSEH------
TETNG   ------------------VCEKQVQEVALDGTETYVPPLSMSQN------
DROME   ------------------TCENQIREIGYDGTELYQPPPTLSES------
BRUMA   ------------------CSEWEIQEIGFDGVEKYMRPLDYSDH------
CAEEL   ------------------NDDWKIQEIGFDGVERYLQPETFTDH------
YEAST*  SNPNSSLHSPTSDEPVPESAGKADTSIRLEGDELENKTKKDNDKNLKFLK
SCHPO   ------------------EIIGGQNNFLENN-------------------
CANAL   ------------------NDTGHIDPFLQDEDKLK---------------
CANGA   SKSTSPHSNS--------TSDGNPESHNTENEEVDNKAGSLPPE--QFSN
ASPNI   ------------------IREKR----------------DRPDS------
RHIO9   ------------------FIDNKILDITNSGHEIPKTEIPLAEK------
CRYNE   ------------------LGEKPVKSIDPDGTIAYDETKTPDAK------
USTMA   AAASATDSHTILDLF---DREPSLADITAEGKNIFVTPKPLSVQ------
ENCCU   ------------------EIEKKEKEMMENISQDYMGIINRCSG------
ANTLO   ----------------------RRKALLEAVSERYLSIIS----------
ARATH*  ------------------LPIKRLESIEENGAERFPSDVDYDDK------
VITVI   ------------------LPIKRLDAIEENGAERFPTDVGYDDK------
SOYBN   ------------------LPIKRLDSIEENGIERFPPDVDYDEK------
SOLLC   ------------------LPIKRLEYIEEQGAERFPSDIGYDEK------
ORYSJ*  ------------------LPIKRLHAIDEAGNEHYPPDTSSEEQ------
HORVU   ------------------LPIKRLEAIDEAGNEHYPP----EEQ------
SORBI   ------------------LPIKRLEAIDEIGNEHYPPDTSNEEQ------
SELML   ------------------LPLKRVLALEESGIELYPPESSQDEK------
PHYPA   ------------------LPLKRVLAMEESGTELLPPEPSQEEK------
VOLCA   -----------------------------NGKEVREGWDPMDMS------
                 60        70        80        90       100
```

MED17

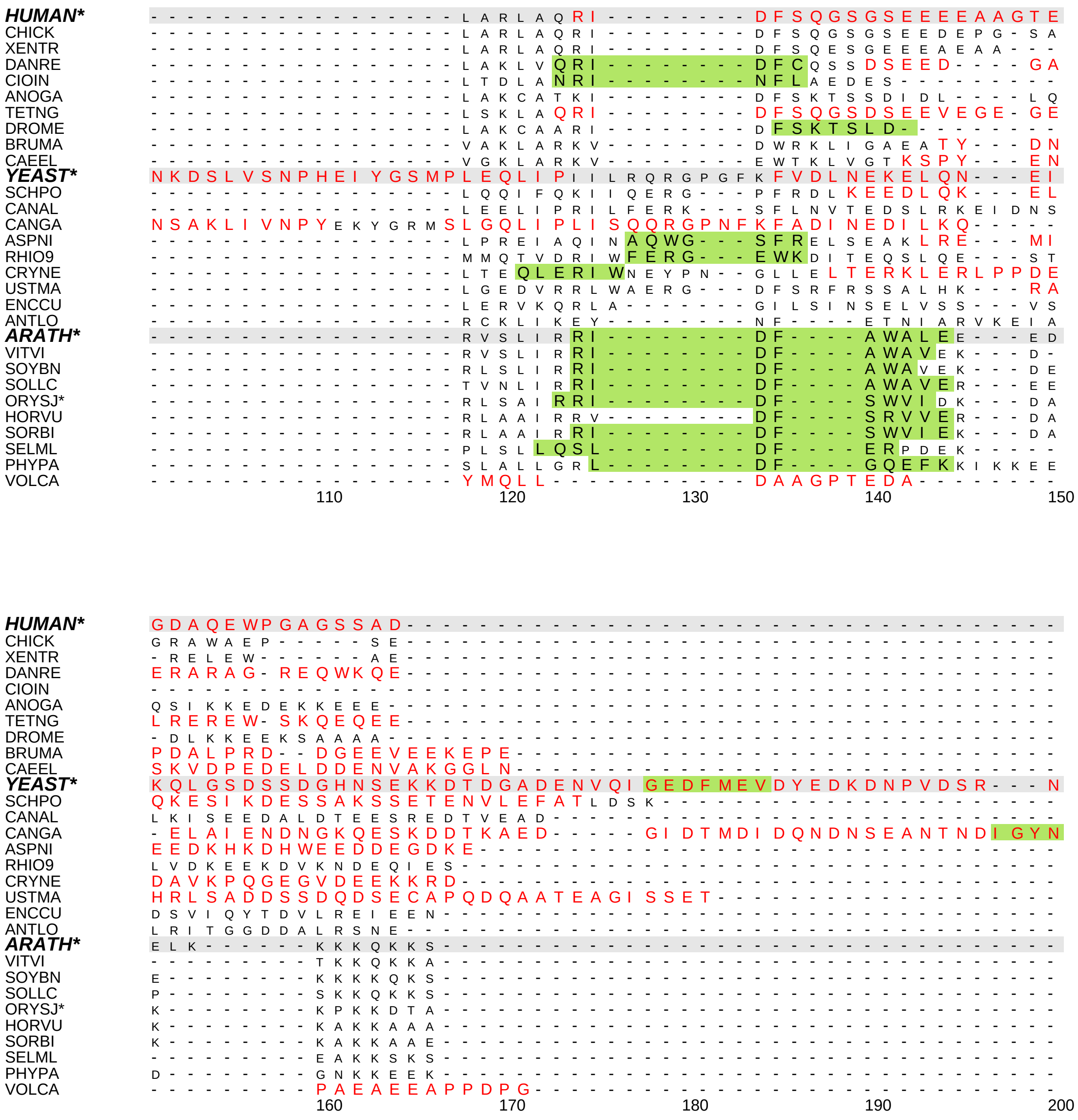
HUMAN*
CHICK
XENTR
DANRE
CIOIN
ANOGA
TETNG
DROME
BRUMA
CAEEL
YEAST*
SCHPO
CANAL
CANGA
ASPNI
RHIO9
CRYNE
USTMA
ENCCU
ANTLO
ARATH*
VITVI
SOYBN
SOLLC
ORYSJ*
HORVU
SORBI
SELML
PHYPA
VOLCA
110
120
130
140
150
160
170
180
190
200

MED17

```
HUMAN*   ---------------QDD------EEGVVKFQP-SLWPWDSVRNNLRSAL
CHICK    ---------------AED------EEGLVKFQP-SLWPWDSVRNNLRSAL
XENTR    ---------------QEE------EEGLVKFQP-SLWPWDSVRNNLRSTL
DANRE    ---------------PEE------DEGQLKFQP-SLWPWDSVRNNLRSAL
CIOIN    ---------------DVD------QDVERKLTPGKQWPWENIRSHLQSAL
ANOGA    ---------------SKD------PKE--QFQS-SLWPWDSVRNKLKEAL
TETNG    ---------------EDD------SKGTVKFQP-SLWPWDSVRNNLRSAL
DROME    ---------------ADE------DKDATQFQE-SLWPWDAVRNKLKDAL
BRUMA    ---------------EVI------IGMEDRKTP-EAGPWHIVAKRLHEAL
CAEEL    ---------------------------EELVTP-EAGPWSSVAKYLHESL
YEAST*   ETDHKTNENGETDDN---------IETVMTQEQ-FVKRRRDMLEHINLAM
SCHPO    ---------------RNV------NDTEVESMD-SQAYKKELIEQIMIAQ
CANAL    -----------------------------QQEV-FNKHKFELSKNINNAL
CANGA    EWSNEPKEDTGILDNTQDTNINGEMESQLTQEE-FNKIRKVMLEHINMAM
ASPNI    ---------------STD------LETSEQLDQ-LYKRRAEIIQYALQAH
RHIO9    ---------------QEQ------QQVVPPGFD-VVKLRESVINKLLHAK
CRYNE    ------------------------PFSMMTYEE-MEKLRSEIFLQLNDAR
USTMA    ---------------SEK------IGGTIAESQ-FVELRNQVLANLDVAH
ENCCU    ---------------------------------------SLVESRLSLVV
ANTLO    ----------------------------------------QMLSKAHEVV
ARATH*   ---------------SKD------SVE--------QWKWKGMVENLQLAH
VITVI    ---------------SKE------EAT--------PWPWQSLVENLRLAH
SOYBN    ---------------SKE------TST--------PWQWQGMVENLQLAH
SOLLC    ---------------SSA------TSSKETSSS-QPWQWQSLVENLRLAH
ORYSJ*   ---------------QQQ------QQQ--------AWPWQGMMESLQQAQ
HORVU    ---------------EDA------AHK--------AWQWQGLHESLQLAH
SORBI    ---------------ADT------TQQ--------VWPWQGLMESLQQAH
SELML    ---------------GKD------GAS--------QWPWQGLIEHFHQAR
PHYPA    ---------------EKE------IVQ--------VWPWQGLVDHLQQAH
VOLCA    ----------------------------------------EIIKIFEDAK
                  210       220       230       240       250
```

```
HUMAN*   TEMCVLYDVLSIVRDKK---------------------------------
CHICK    TEMCVLYDVLSIVKDKK---------------------------------
XENTR    TEMCVLYDVLNIVKEKR---------------------------------
DANRE    TEMCVLHDVLSVLKERK---------------------------------
CIOIN    IEMNVLVDVMAIAQNKR---------------------------------
ANOGA    TEVCVLSDVLNIAKEKR---------------------------------
TETNG    TEMCVLYDVLSVVKEKK---------------------------------
DROME    TEICVLSDVISIAKDKR---------------------------------
BRUMA    QQVNVLVDTISVLKTP----------------------------------
CAEEL    NQLNSLLDVISVTKSTD---------------------------------
YEAST*   NESSLALEFVSLLLSSV--------------------KESTGMSSMSPFL
SCHPO    TECSLALDMTSLLLSKF---------------------KENSIETISPFL
CANAL    NETQLSLDFVSLLISSV--------------------KPSLAKSTISPHL
CANGA    NESSLAMEFVSLLLSPV--------------------RESTAVSSMSPFL
ASPNI    MEASFALDFVSLLLSKH--------------------QPRQAETSMSPFL
RHIO9    SEIDVALDVINILAASH------------------------------HAS
CRYNE    NELWFVLELAKTLSSSSSFTTQPPPPPAALHQKKGKAKPAPPAPTTLPSI
USTMA    FNSIHAHQLLGMLIKQHRSTA---------------VSSNNAGVAMQPVA
ENCCU    SELKEILSFTGIASEYE---------------------------------
ANTLO    QQVSLLLEIVRALAESS---------------------------------
ARATH*   QELTVIIDLIDTVQAND---------------------------------
VITVI    QELSVIIDLISTVEAND---------------------------------
SOYBN    QELSVIIDLINTVEAND---------------------------------
SOLLC    QELQVIIDLIHTVEAND---------------------------------
ORYSJ*   QELSVVIDLISTVEAND---------------------------------
HORVU    QELTVVLDLISTVEAND---------------------------------
SORBI    HELSVVIDLIGTVEAND---------------------------------
SELML    EELMTLLDFIAHVESNE---------------------------------
PHYPA    HELAIILDMINHVEAGE---------------------------------
VOLCA    GNLDILVDVVTNLERSF---------------------------------
                  260       270       280       290       300
```

MED17

```
HUMAN*   ----------FM--------------------------------------
CHICK    ----------FM--------------------------------------
XENTR    ----------YM--------------------------------------
DANRE    ----------YM--------------------------------------
CIOIN    ----------YL--------------------------------------
ANOGA    ----------YM--------------------------------------
TETNG    ----------YM--------------------------------------
DROME    ----------YL--------------------------------------
BRUMA    ----------YMEALT----------------------------------
CAEEL    ----------YMKALT----------------------------------
YEAST*   RK---VVKPSSL--------------------------------------
SCHPO    KS---TVPPSSL--------------------------------------
CANAL    SK---FVKPTSL--------------------------------------
CANGA    KK---TVNPGSL--------------------------------------
ASPNI    KS---AAPLGSL--------------------------------------
RHIO9    TAKDLVLPAGSL--------------------------------------
CRYNE    PGEPPILPPGTF--------------------------------------
USTMA    RMASPAPSVGALSNRSASITAAQTKAPLGIFSHLNHASTREEEFVLDPLA
ENCCU    --------------------------------------------------
ANTLO    --------------------------------------------------
ARATH*   ----------AV--------------------------------------
VITVI    ----------AV--------------------------------------
SOYBN    ----------AV--------------------------------------
SOLLC    ----------AV--------------------------------------
ORYSJ*   ----------AV--------------------------------------
HORVU    ----------TV--------------------------------------
SORBI    ----------AV--------------------------------------
SELML    ----------AL--------------------------------------
PHYPA    ----------AV--------------------------------------
VOLCA    --------IEAK--------------------------------------
                  310       320       330       340       350
```

```
HUMAN*   ----------------------------------TLDPVSQDALP-----
CHICK    ----------------------------------TLDPVVQDPPP-----
XENTR    ----------------------------------HLDPVSQEALP-----
DANRE    ----------------------------------TLDPVSQDPAM-----
CIOIN    ----------------------------------MFSSVAKEAET-----
ANOGA    ----------------------------------VLDPIPQEPPE-----
TETNG    ----------------------------------ALDPVSQDQTG-----
DROME    ----------------------------------VLDPLLEEADD-----
BRUMA    ----------------------------------VADAFEAQHNM-----
CAEEL    ----------------------------------VLDPITVQEPT-----
YEAST*   ----------------------------------NSDKIPYVAPT-----
SCHPO    ----------------------------------QFSRSQPPESK-----
CANAL    ----------------------------------NSDRLGQDSND-----
CANGA    ----------------------------------NSEKVKMPAVS-----
ASPNI    ----------------------------------NSEVVNPPPRP-----
RHIO9    ----------------------------------SATYVTKPKST-----
CRYNE    ----------------------------------STTPSDFPIKP-----
USTMA    IALSRTALNTSTAVRHSNDSDQHEQEQDEFADPTSAEYALKQAQKEIEST
ENCCU    ----------------------------------DADKEVREDPL--YYY
ANTLO    ----------------------------------------------GDLY
ARATH*   ----------------------------------TVAGMTRPKPM-----
VITVI    ----------------------------------TVASMTRPKPL-----
SOYBN    ----------------------------------TVASMTRPKLL-----
SOLLC    ----------------------------------TVAGMTRPKQL-----
ORYSJ*   ----------------------------------AVAGMLKPKSL-----
HORVU    ----------------------------------TVATFSKPKKR-----
SORBI    ----------------------------------AVASTTKPKSQ-----
SELML    ----------------------------------TVTHMMKPKPL-----
PHYPA    ----------------------------------AVARMERPKLQ-----
VOLCA    ----------------------------------PFSAGRRPGVKR----
                  360       370       380       390       400
```

MED17

```
HUMAN*  -----PKQNPQTLQLISKKKSLAGAAQILLKGAERLTKSVTENQ-ENK--
CHICK   -----PKQNPQFLQLISKKKSLAGAAQILLKGAERLSKSVAENQ-ENK--
XENTR   -----PKPNQHGLLLVSKKKSLAAAAQILLKGAERLSKSVAENQ-ENK--
DANRE   ------AKTPQVFQLISKKKSLGTAAQLLLKGAEKLSKSVSENQ-EQR--
CIOIN   --------PKPMLQMITKKRCLGAAGKILLDGAERLNKARN----EVS--
ANOGA   --------VKQMVLVYARKKALASAANILQSGVERLKAVQSDQGVSRS--
TETNG   -----RAQTPQVFQLLSKKKSLATAGQLLLKGAEKLSKSVAENL-ENQ--
DROME   --------TKPIVQVYSRKKAISQAAQVLLSGAERLRNAHSEQ--RNR--
BRUMA   ---QDMIQQSKQFQWVTRRKALGEAIGVLEQAQKFRSKLLTE---TDP--
CAEEL   ---PETISTNRGTQWIWKRRALQEAVQVLDMAQKQRQRASSNLGLSADYM
YEAST*  ---KKEYIELDILNKGWKLQSLNESKDLLRASFNKLSS-------ILQ--
SCHPO   -------ESDATLAKCWKEKSLTSSCKFLFEAKERLTS-------VVE--
CANAL   ---NQESKATDSFGQGWKLESLGKITDLFREASTNLND-------QVI--
CANGA   ---RRDKLSLSILSRGWKLRALNEARAILKKNFTEISS-------SLK--
ASPNI   ---DSTLKDIKSVARGWRLQNFNSAADKLLNAGSRLET-------EVN--
RHIO9   ---MKAELEAVQLNLGLKRKQQKQASDYLKKSASALNS-------IIE--
CRYNE   ---PHAQIYELERLLQAKRIALNECQGLIDGAVGELRL-------MAH--
USTMA   PEFKENELREFRIVLETKRKALEDAAQLLSSAATELRS-------SQA--
ENCCU   DMTSRVLSMEKKLCTLEKYTFFVNANQICIRSRRTLVD-------LMM--
ANTLO   EMMGKLMFVKERRLQFRQNKFYNVVDRVYKSRLVLLSK-------RVE--
ARATH*  ---PNEILSDLAVSTATKLQGYRNLGNYFKQSAKALEQ-------KIN--
VITVI   ---PNEVLSDLGVSAATKLQCFRHLSKYFKQSAKALEQ-------QIA--
SOYBN   ---PNEALSDLAVSAATKLQCYRHVGKYFKQSAKAFEQ-------QVA--
SOLLC   ---PNEHLSDLAVSMATKLQCFRHLGKYFKQSAKALEQ-------QVA--
ORYSJ*  ---PTETLVDTAVSAATKLQRVRHLSRYFKQSAKTMEQ-------QFQ--
HORVU   ---LDKVLVNMAVSAATKLQRLRHLGRYFKQSAKTMEQ-------QFQ--
SORBI   ---PNEILVDMAVSAATKLQRLRHLSRYFKQSARTMEQ-------QFQ--
SELML   ---PNEAGADLALRTASKSRSYKEIASYLKRNAKSLER-------QVE--
PHYPA   ---PQEICSDVALRASSKLHHFRNVGKYLKRSAQSLEQ-------QVE--
VOLCA   ---EDAVANSLQSRVAATSGSLLRVSRRLAGGAELLKA-------HVE--
                 410       420       430       440       450
```

```
HUMAN*  ---LQRDFNSEL---LRLRQ------HWKL--------------------
CHICK   ---RQRDFNSEL---LRLRQ------HWKL--------------------
XENTR   ---RQRDFNSEL---LRLRQ------HWKL--------------------
DANRE   ---RQRDFNSEL---LRLRS------QWKL--------------------
CIOIN   ---TQQNFHAQL---LKLRQ------RWRV--------------------
ANOGA   ---NSTDFHIEL---LRLRR------NWRL--------------------
TETNG   ---RQRDFNSEL---LRLRS------QWKL--------------------
DROME   ---NVSDFHIEL---LRLRQ------NWRL--------------------
BRUMA   ---DKAMFFREL---EKMRE------LWRV--------------------
CAEEL   AHLQRTKFFEEL---REMRE------IWRV--------------------
YEAST*  ---NEHDYWNKIMQSISNKD-----VIFKI---------RD---------
SCHPO   ---TEHEYYTEL---VKVKE-----ASWPL----------F---------
CANAL   ---KERRYWNMINLVLANDE-----VLFRM---------RD---------
CANGA   ---QEHHYWSSIAYNISNKD-----VLFKI---------RD---------
ASPNI   ---SETKYWNEV---LAVKE-----KGWKI---------CR---------
RHIO9   ---KEHEFWDEA---LNLRR-----HNWIM--------------------
CRYNE   ---AGDRFWKDI---RNLKEGEGGRGQWAVVPKPDFGRVGG---------
USTMA   ---GNRERWRSL---IGLHG-----RGWGMTPGRPLLDVERFGVGESEGE
ENCCU   ---KDIDLWISGAC-NNVRQVGIEVSAMLI--------------------
ANTLO   ---SEIEAWQGV---IRATS---RSIFCKI--------MER---------
ARATH*  ---REARFYGAL---IRLQR------NWKV--------------------
VITVI   ---REARFYGAL---IRLQQ------NWKV--------------------
SOYBN   ---REARFYGAL---IRLQQ------NWKV--------------------
SOLLC   ---REARFYGAL---IRLQQ------NWKV--------------------
ORYSJ*  ---KESRFYGSL---IRLQQ------NWKV--------------------
HORVU   ---KEARFYGSL---IRLQQ------NWKV--------------------
SORBI   ---KETRFYSSL---IRLQQ------NWKV--------------------
SELML   ---RESVFYGAL---MRLQR------NWKI--------------------
PHYPA   ---REAVFYGAL---MRLQR------NWKV--------------------
VOLCA   ---REDKFYQQL---RQLQM------FWK---------------------
                 460       470       480       490       500
```

MED17

```
HUMAN*  --------------------------------------------------
CHICK   --------------------------------------------------
XENTR   --------------------------------------------------
DANRE   --------------------------------------------------
CIOIN   --------------------------------------------------
ANOGA   --------------------------------------------------
TETNG   --------------------------------------------------
DROME   --------------------------------------------------
BRUMA   --------------------------------------------------
CAEEL   --------------------------------------------------
YEAST*  --------------------------------------------------
SCHPO   --------------------------------------------------
CANAL   --------------------------------------------------
CANGA   --------------------------------------------------
ASPNI   --------------------------------------------------
RHIO9   --------------------------------------------------
CRYNE   --------------------------------------------------
USTMA   GEEEEDEEEVLNSDATADDGEKEAKNQQSSIKPGKKKSKDEKQAKAAGLQ
ENCCU   --------------------------------------------------
ANTLO   --------------------------------------------------
ARATH*  --------------------------------------------------
VITVI   --------------------------------------------------
SOYBN   --------------------------------------------------
SOLLC   --------------------------------------------------
ORYSJ*  --------------------------------------------------
HORVU   --------------------------------------------------
SORBI   --------------------------------------------------
SELML   --------------------------------------------------
PHYPA   --------------------------------------------------
VOLCA   --------------------------------------------------
                 510       520       530       540       550
```

```
HUMAN*  --------------RKVGDKILGDLSYR-----SAGSLFPHHGTFE----
CHICK   --------------RKVGDKILGDLSYR-----SAGSLFLHHGTFE----
XENTR   --------------RKLGDKILGDLSYR-----SAGSLFPHHGTFE----
DANRE   --------------RKVGDKILGDLSYR-----SAGSLFPHHGTFE----
CIOIN   --------------RRHGDKILGDLSYR-----TAGSDFLHHGSFE----
ANOGA   --------------KKVSNTIIGDLSYR-----TAGSKFMHPGMFE----
TETNG   --------------RKVGDKILGDLSYR-----SAGSLFPHHGTFE----
DROME   --------------KKVSNAIIGDLSYR-----TAGSKFGMSGTFE----
BRUMA   --------------RKTGNVTYGDLGYK-----MFGPKYNPKELFD----
CAEEL   --------------RKVGDYICGDLSYH-----IFGWKYDTPAIFD----
YEAST*  -------------RTSGQKLLAIKYGYE-----DSGSTYKHDRGIA----
SCHPO   -------------NSQGSNHLSVQYSCLGGISLGLG--------------
CANAL   -------------PQNNARAIGVKYGYG-----DSGSNF-HDQGLA----
CANGA   -------------KQTTKRSLGLKYGYE-----DSGSTFRNDRGTA----
ASPNI   -------------LPRESQALGVQYGFL-----EATPIF-RDRGLA----
RHIO9   ----------YANTTHPNNSFLIQYGFA-----EVGSDF-NEASLG----
CRYNE   -------------EGEKAKDVVIPYAID-----EAPSGT-RARCLAGFDL
USTMA   GFGTPIITSDGKVKEEGARDAWIGFGLP-----EAPIEL-RRRSLAYWAD
ENCCU   -------------EGRKKSHVFDPLDSL------------HHYLISKGFL
ANTLO   -------------LDKKRAQVSVIFNAM----------------------
ARATH*  --------------KRQRMLASNA-SNE-----GFTIDLSDSSLYDPTS-
VITVI   --------------KRQRVAASAP-GNE-----GFSIDLFSNSLHDPVA-
SOYBN   --------------KRQRQAAIVP-GNE-----GFTFDLFDNS-YDQAA-
SOLLC   --------------KWHRMVAAAS-GNE-----GFYIDLFDTSLYDPAV-
ORYSJ*  --------------KRQRFGGSGP-GSE-----GFMFDLIDTSQLDTAA-
HORVU   --------------NRQ--CGNAP-GSN-----SFMFDVVDTSHLDTAV-
SORBI   --------------KRQRAVGS-P-GSE-----GFMFDLVDSSQLDTTT-
SELML   --------------KRQRGVSAGPGGKA-----GFLMDLSFPLSVDPSLI
PHYPA   --------------KRQRGVAAGPGGSA-----GFSIDVGTS--------
VOLCA   --------------HDVIDIFCLSFSLHFAPLVSSGQVHLNPRVVDCPFM
                 560       570       580       590       600
```

MED17

Species	601–650 (610, 620, 630, 640, 650)
HUMAN*	-VIK-----NTDLDLDKKIPED---------------YCPLDV------Q
CHICK	-VIK-----NTDIDLDKKIPED---------------YCPLDV------Q
XENTR	-VIK-----NTDIDLDKKMPED---------------YCPLDV------H
DANRE	-VIK-----NTDIDLDKKIPDD---------------YCPLNV------Q
CIOIN	-VVK-----VSADDMDHDVGNE---------------NLPLRV------V
ANOGA	-VTK----AED--EESGSPPASPSGSGAAGTVACPKINSALRV------N
TETNG	-VIK-----NTDIDLDKKVPED---------------YCPLDV------Q
DROME	-VTK----AEETGDEDTASSSNSSSSVSGNNGMQLKASSALRV------I
BRUMA	-ITR----RTGTSGGDGGNLSALS-------------ESCLQV------Q
CAEEL	-ISR----RSLSNNMEN--------------------LSIIEV------S
YEAST*	-NIR---NNIESQNLDLIPHSSSV---FKGTDFVHSVKKFLRV------R
SCHPO	-LIR---MKPESKSFEVQSSLLYS-------------QAALKI------S
CANAL	-LLR---KDNQTGEISFHPISS-----INNAKIVEKVSRFIRV------K
CANGA	-ILR---GTDEANGLELIPLTLGR---TSTVGSVYKGGKFLRV------R
ASPNI	-ALR---RTDDGSLFLDKGLIPLK-------------SQGVRV------R
RHIO9	-ELK---RADDGVQLSVAH------------------DQPRRV------T
CRYNE	DPTK---------------------------------KDGLTFGDRHHLR
USTMA	VPSS-------------VGGATTTEEVRKKLVFPDRMHRRLRVRFVLWPR
ENCCU	CILH--------------------------------ESKRLAVDLAVVER
ANTLO	----------------------------------YDIRPMLCL-------
ARATH*	-GFR--PSTLSTIRVDHDS------------------AGMLAI------N
VITVI	-VFR--PSSLSTVRVDHDS------------------AGMLAV------H
SOYBN	-IIR--SLSMSTVRVNHDA------------------AGNLAI------N
SOLLC	-AFR--PSSASVVRIEHDP------------------AGMLAV------N
ORYSJ*	-MPR--LSSLPLIPIDQDS------------------SGTLSV------Q
HORVU	-MPR--SSSLSLVPIDQDS------------------SGTLSV------H
SORBI	-MPR--LSPLSLIPIDQDS------------------SGTLSV------Q
SELML	SSSR--TAAWTLIKVNQDA------------------NGLLSV------Q
PHYPA	------ESAPCLITLEQNS------------------VGLLTA------H
VOLCA	VDILAAGQALTGNTYELGSGSLLVPLMKGD-------GGALRV------Q

Species	651–700 (660, 670, 680, 690, 700)
HUMAN*	IPSDLEGSAYIKVSIQKQAPDIG-----------------------DLGT
CHICK	IPSDLEGSAYIKVSIQKQAPDIG-----------------------DLGT
XENTR	IPSDLEGSAYLKVSIQKQAPDIG-----------------------DLGT
DANRE	IPSDLEGSAYIKVSIQKQSPDIG-----------------------DLGT
CIOIN	MSPDLKGRSTISVMIIDTSGTDYIT---------------------DMAS
ANOGA	VPTELQGVAFIKVITQKDQEDL------------------------CTAT
TETNG	IPSDLEGSAYIKVSIQKQAHDIG-----------------------DLGA
DROME	VPAELQGVAYIKVITQKDQEDL------------------------CTAQ
BRUMA	VPCDLIRRSTIAVSIEIDN--------D------------------DPKT
CAEEL	VPKDLARRSMLAVSIVQDDIQSGN----------------------GLFR
YEAST*	IFTKIESEDDYILSGE------------------------------SVMD
SCHPO	ILNKDRDEIGSSTWSWPSQNCN----------------------------
CANAL	ILSQI--DGDYMLTGQ------------------------------SIFN
CANGA	IFTKIESEGDYILSGE------------------------------SSLD
ASPNI	VRRGDRIVGCSKVCRPPQDA------------------------------
RHIO9	VHTRHANSLL------------------------------------GMED
CRYNE	LRATLRDDSGAVISSTPVKVEDQ---------------------------
USTMA	VSSMQHDEGRIEWTSD------------------------------PTAR
ENCCU	VNEKRKEFAERTLSGD----------------------------------
ANTLO	--
ARATH*	VPQDSWYSLRFGYVGL-------NPIGNSNESDEHIDSTTG-----HDIP
VITVI	LPPNSCRALHFGFLGG-------HLGNIPKEPSKTKTYGPD-----ELPS
SOYBN	VSPDLCHSLQFGFVGA-------QSDDTRRKSNQNKSHFSG-----ELNL
SOLLC	LPPNSCRTLQFEFLGA-------STPAGVIKHSRLRPKVSF-----EDSS
ORYSJ*	VPQKSCRFLSLNFRGD-------SANGVENYGHKLKDGIS------SITS
HORVU	VPQKSCRFLSLQFCGD-------STSGTESYACNTK-GVS------STTS
SORBI	IPQKSFRSLTLQFRGD-------IANNAERSAIKKKEGTL------TNTT
SELML	IPAGKTITTLQVYLSR------------ELEEYTSKQ---------QLEQ
PHYPA	LPAALTLSSLHVTLLS-TPAPFKFGKDTERLSATSKEGTSDATVLPEAPK
VOLCA	VGAVPGGTGGTMAASWWGCGGVLGFTEALGLELPLERPNVNDPSRVGETR

MED17

```
HUMAN*   VNLFKRP------LPKSKPGS----PHWQTKLEAAQNVLLCKEIFAQLSR
CHICK    VNLFKRP------MPKSKPGT----PHWQTKLETAQNVLLCKEIFAQLSR
XENTR    VNLFRRP------LPKAKPGT----PHWQTRLEAAQNVLLCKEVFAQLSR
DANRE    VNLFRR-------QAKAKPAS----QMWHSRLEAAQNVLLCKEIFAQLSR
CIOIN    LNYNDEVG-----FNTSSMHD----PSWHKKLCQAQNVLYCKELFAHLTK
ANOGA    VNMMG--------STQLVPQA----GAWQQTLEYAQNVLFCKELFNQLAR
TETNG    FNLFRRPGHLSSPRSATVAGT----QAWHMKLEAAQNVLFCKEIFAQLSR
DROME    LNLMGH-------GPNITAQV----GVWQKTLEFAQNVLFCKELFAQLAR
BRUMA    LFATAENDLDYMKVDREQAMK----VHWSKALQWAQESLLCRDIFKQLCK
CAEEL    DPKDKKYTYSYRESDSEKVKL----LHWKDSLKWAQNTLLLRDVFKTICT
YEAST*   R----D-------SESEEAET----KDIRKQIQLLKKIIFEKELMYQIKK
SCHPO    -------------------------SVLLKDIYKLQEILFEMDIWNSLLQ
CANAL    FDFEKS-------K-----------QSIINDIEKARFFLFEEDLFHQLIR
CANGA    KLFKNH-------SENSDSKN----DDVRLQISKLKFFIFEQELMHQLKK
ASPNI    -------------------------ESIESRILQARDTVFEEELFYEVMR
RHIO9    QVKDDE-------KAYYEPEA----KEIQNQLMQATSTVFDAELFSNILA
CRYNE    -------------------------SDVRAMMEAAQMEAFDEDLFNEIRF
USTMA    WQENES-------SDACGAPAVAPGQILDQELQQASREATDELVFGDVVA
ENCCU    ----EP-------VLVSDVAGFILWSHYLTTL-DMRFKMYDRLVFGFLSR
ANTLO    -------------------------SRVLVPLYVSREFNFGASPFPERFD
ARATH*   GTSEKL-------SASDDKYV----KETHSLLREVHKSIFAEQLFDMLNR
VITVI    KEI-KK-------PLSDNECV----KETHSVLREGHQAIFDEQVFDLVNR
SOYBN    GKTGEE-------SLSDEECV----KKTHSLLREVHEAIFNEQVFDLVNR
SOLLC    GET-KR-------EKSDDEHV----RETHSTLREVHRAIFDEQVFDLVNR
ORYSJ*   SETD---------NDDVNKSI----KHAHSILRNIHKSIFEEQVFDMVIR
HORVU    SAVEDD----VPENDDVNKSV----KQAHSILRNIHRSIFEEQVFDMVTC
SORBI    TEAEKD----VLENGDVNKSI----KHAHSILRDIHKSIFEEQVFDMVIR
SELML    PPRRRD-------NRDPDEGT----SSANHRLRNIQLSIFDELIFECLCR
PHYPA    PGERGV-------GPGVDRGA----TRTQAMLRQIQTANFDAQVFEWVGR
VOLCA    LNGKGA-------RTQLVVGS----RRVHALLLQLQRRQLWRRVAAGLER
                   710       720       730       740       750
```

```
HUMAN*   EAVQIKS---QVPHIVVKNQIISQPFPSLQLSISLCHSSNDKKSQKFATE
CHICK    EAVQIKS---QIPHIVVKNQIISQPFPGLQLSISLCHSSNDKKSQKSASE
XENTR    EAVQIKS---QIPHIVVKNQIISQPFPGLQLSISLCHSSNDKKSQKSNSD
DANRE    EAVQIKS---QIPHIVVKNQIISQPFPGLQLSISLCHSTGEKKSQRSSPD
CIOIN    EAVQYKNVGAIAPIVVIKDTISTEIFPSIRLVVKLIHSMDTDNQQSQTEN
ANOGA    EAVQLQA---PIPHVVVGNQIRATLLPGIQLIISLCHSTSSDSNNSSE--
TETNG    EAVQIKS---QIPHIVVKNQIISQLFPGLQLSISLCHSANEKKHQRVSPE
DROME    EAIQLQA---PIPHVVIGNQIRATLLPNIQLIISLCHSTTFDSSQPA---
BRUMA    DAVILKD----RITVIRDGVLIASLFDNILLRVELAFHPFEDGP------
CAEEL    DAIKLRN----RLSIIRDNVLLIHLFDDYLLRFELQWFPFQTGE------
YEAST*   ECALLIS-YGVSIE--NENKVIIELPNE-KFEIELLSLDDDSIVNHEQDL
SCHPO    EAQSCGN-QGVNFT---GDEILVPISDDHVVRITLETSSKNTESGFTEDK
CANAL    EAKLLVN-YNVSII---SNKIIIEINN-IIIEIESIVYDELNEEELENYY
CANGA    ECAYLIS-YGVTVE--NEHKIVIELPNE-KFEIEYLSLDDDSVVNHEQDA
ASPNI    EARILGT-QGVKTR---QNLVQVPVSDEQEILLDLVDWDQDRDPDAADST
RHIO9    EAQTLN--SNVRIG---NDEIEINIDGDINLSITKLPVTSTIES------
CRYNE    AAARIPK-------NETEPQRVSFPVADKI-LSFELYDTRSLPSS------
USTMA    QARLLPPMFGVRLT---SSSVRLVLTKRLDCVVELVRISDQNDANVSDSG
ENCCU    SKMLLKS-------------------------------------------
ANTLO    TTEEMLS-FALSLAFIKEKGIVVRGPDYRAMNVSQVRLVRRLFDKMGTCY
ARATH*   EAFNEG--VGFNISGLRENFMEMSIGQGASLFVSLHPSGKNPS------I
VITVI    EAFNSS--SGVNVTGIRENYLQLNIGQGASVFMSLVPSGQDEKTADGMGM
SOYBN    EAFNTV--AGVSVTGIRENYLQLSLGQETSVYLTLVSNSQDHSTVEGELT
SOLLC    EAFNPA--LGADVTGIQENLLRLSIGQRASLSISLVPSADDGQTANAVGD
ORYSJ*   ETFVQS--QGINVTGMREDFLQLAIGQECSLCLSLAHSGDGSDSEMVDHE
HORVU    ETFVQT--KGVNVTGMWEDFLQIAIDQEILLCLSLVNSGQDSDSEMAGHE
SORBI    ETFVQS--QGINVTGMCEDFLQLAIGQECSLCLSLELSRQNSISETVGQE
SELML    DVLQPS--SATSLHEIGESSLDIGAGPSIASFFRLVEDDAANDAALK---
PHYPA    QALSLS--PYINVTGLSDTCLQLSLGNAASLNVHLTPMAPIADESQGSDK
VOLCA    DAASQG----------------------------LVQGGGAGAAAAGGAG
                   760       770       780       790       800
```

MED17

```
HUMAN*   KQ---------------------------CPEDHLYVLEHNLHLLIREFH
CHICK    KQ---------------------------NPEDHLYVLEHNLHQLIRECH
XENTR    KM---------------------------GAEDHLYVLEHNLHQLIREFH
DANRE    KS---------------------------KPDDHLYVLEHNLHQLIREFH
CIOIN    P----------------------------SALETVFSLKFTLLQLLQEVH
ANOGA    -----------------------------PIKDHDHVLEHSLHQLLREFH
TETNG    KP---------------------------KADDCLYVLEHNLHQMMREFH
DROME    -----------------------------PINDHDHVLEHSLHQLLREVH
BRUMA    -----------------------------LPSIGDDYLNRALRQLFLSDL
CAEEL    -----------------------------IKEEGDIYLNRVLREMIIGFE
YEAST*   PK---------------------------INDKRANLMLVMLRLLLVVIF
SCHPO    KS---------------------------NEDTSTNFVTIKQEKELLKCL
CANAL    QNVNEYST---------------------LHNKKCQLILNYLKLMLCCYY
CANGA    PK---------------------------ANDRRANLMLVTLRMLLIVIY
ASPNI    -----------------------------EQDVFADAVAHSIRILLTYAH
RHIO9    -----------------------------YQDNKICMTNLAFRLLLLQRY
CRYNE    -----------------------------PTSSICDIIVSSTRLSLLNIH
USTMA    QQSKDDTEEKMQLGGEKQQ----------RYSPHASLLLAFLRMGPLRKY
ENCCU    -----------------------------SNFSRIREALVSLRRLTVHLN
ANTLO    HE---------------------------IEDAERSMCMEILR-------
ARATH*   KKSES---ATLLIESSGRVEPAEGGDY--RLKKLGFPNRTSYEIYLQQIF
VITVI    QNLES---AILPMDTFDGVKLSD-GKHDNDKKKSGFPNRISSEIYLKQLF
SOYBN    DNAEN---AILPLESSDGMKREA-KQN--TSNKGQFSNSICYEIYIQQIF
SOLLC    EHPET---AIVPFESVDASKQDE-GKL--DLKKLGFPNRISFEIYLQQLF
ORYSJ*   DHANSEDASNLVLVTMNGKLDPL------RKDVTGFPNPRSLEIYLLQLF
HORVU    EHNNSE--ANLVLATTNGKQEPL------KSDASGFLNPKSLEIYLLHMF
SORBI    DHMDTDYTENLAVATVNGKESS-------NKDVRGFPNPKCLEIYLLHMF
SELML    -----------------------------EVDVKRLPLAGSLRIYLQQGF
PHYPA    VLAPRTPD---------------------HQNPRLLPNEGSLSVCLQQAY
VOLCA    TSGATAAAAPAGGGGGSGG----------AHPVHTSLARLAAQNVLRTSD
                       810       820       830       840       850
```

```
HUMAN*   ----------------------------KQTLSSIMMPHPASAPF---GH
CHICK    ----------------------------KQTLSSVVMPHPASAPF---GH
XENTR    ----------------------------KQTLSNIVMPHPASAPF---GH
DANRE    ----------------------------KQTLSSVVMPHPASAPF---GH
CIOIN    ----------------------------RRKL-EISPPRPTSAVLGLTPH
ANOGA    ----------------------------HKN-THHPFPHPASGPLG--PS
TETNG    ----------------------------KQQLSSVVMPHPASAPF---GH
DROME    ----------------------------YKN-SHHPFPHPASAPLG--PT
BRUMA    ---------------------------CARNIRHQTFVAMPLSAL---PN
CAEEL    ---------------------------CTKFIRPQFFCSMPVTHL---PE
YEAST*   ----------------------------KKTLRSRISSPHGLINL---N-
SCHPO    ---------------CDTLNAIAHILFLKHCRKSDRRSQQPELYM---AI
CANAL    ----------------------------KYNLKLKQKVPTALTKW---K-
CANGA    ----------------------------KKNLRQKMVSNTRKHIA---S-
ASPNI    ----------------------------RQNLRRRTQPPPPLAPK---RR
RHIO9    ----------DFNMWKNKARILSTNHIIQNSLAKKSDTGTSSTNT---ST
CRYNE    ----------------------------RQRKINLVAPSQNFASP-----
USTMA    --HAFVNATEQARRADAAARAAAIKAIATAKPKKSTSAANTATAT---AT
ENCCU    ---------VDYEDVDRVISSVAINYFESQGPKNADLSSCDMEQL---K-
ANTLO    --------------------------------RNEHLLETSVAEY---VD
ARATH*   ----------------------HEHAFGKAKDQLKS-KSIRASNQ---T-
VITVI    ----------------------HEHVFVRAKDKHISAGRTQPSSQ---P-
SOYBN    ----------------------HEYIFGKGGDKPSSSGN-RLSGI---Q-
SOLLC    ----------------------HEHVFIKAKNRLSSLGKPEISSQ---P-
ORYSJ*   ----------------------HENILRKVREKSLNIGRYQSPAQ---V-
HORVU    ----------------------HDNILRKVREKYRNIVRYQSPGQ---TA
SORBI    ----------------------HENILRKLREKSRHMVRYQSSAQ---A-
SELML    ----------------------YSMLLASIVQR--SAPGPRAGTG---K-
PHYPA    ----------------------QRHLHGRDD----NALSKETGKH---KD
VOLCA    PSLAALRPLMLEHLVACRLPPPYKPPPPRPAVGTRGLHHPYGGAA---Y-
                       860       870       880       890       900
```

MED17

```
HUMAN*   KRMRLSGPQAFDKNEINSLQSSEG--------LLEKIIKQAKHIF-----
CHICK    KRMRLAGPQAFDKNEISSLQTNEG--------LLEKIIKQAKHIF-----
XENTR    KRMRLAGPQAFDKNEMGFMQQNEG--------LLEKIIKQAKHIF-----
DANRE    KRLRLAGPLAYDKAEISSLQQTEG--------LLEKIIKQAKHIF-----
CIOIN    MRRAAVQGNTLKQLTLASDKNNIS--------FIESLLKMAKHHV-----
ANOGA    KKRMLAGPSAYDRHELLEMTKSQT--------LLEQIIAQAQHIF-----
TETNG    KRLRLAGPLAYDKTEISNLQHSEG--------LLEKIIKQAKHIF-----
DROME    KKRMLAGPMAADRETLLDMTKSQT--------ILEQIIAQAQHIF-----
BRUMA    TLDLRGPYAMTDEEIESRLRQKRT--------LLERLLLLSSHFV-----
CAEEL    ALDLRGCGGFNTAQIEERAVRSRS--------ILQRMLDVASHRA-----
YEAST*   --VDDD----------------IL--------IIRPILGKVRFAN-----
SCHPO    DANA------------------PL--------ILRPLI------------
CANAL    --QSNSH---------------PL--------ILRPLVGNMRHEL-----
CANGA    --TEKD----------------IL--------LIRPLLGKMRHSN-----
ASPNI    PVPE------------------YH--------ILRPIMAYLQHKS-----
RHIO9    SNHHSAVISRARNRVELPKT--VA--------ILSPVLSISRFWA-----
CRYNE    ---------------------IPS--------ILLPIIDTLRFRQ-----
USTMA    GNGAASTIEPASDKGGIAAGGRSTSAALSKLDSIGPIVVTLHYWS-----
ENCCU    ----------------------SS--------MIAFIDECDSFVS-----
ANTLO    ACREAGYIEEQAETIDTFFCRKLA--------ALEQNVGSVEDRLENLNL
ARATH*   --EKDSN---------------SG--------LLDHFCLSLTHRI-----
VITVI    --AKDG----------------FG--------LLGHFCMSLAHRI-----
SOYBN    --AKDG----------------SS--------LLGHFFKSLAHRI-----
SOLLC    --VKDG----------------PN--------LLGHFCVSLAHRI-----
ORYSJ*   --AGDD----------------YG--------LLGHFCLTVAHRI-----
HORVU    EPAGDE----------------CG--------LLGHFCMTVAHKI-----
SORBI    --APDD----------------CG--------LLSHFCMTVSHRI-----
SELML    --EEP-----------------AN--------LLKNFSVLARHRS-----
PHYPA    GYAES-----------------AG--------LVKHVSAIMKHRV-----
VOLCA    --GG---VLSS-----------VP--------LLPDLAALLNHIV-----
                  910       920       930       940       950
```

```
HUMAN*   ---------------LRSRAAATIDSLASRIEDPQIQA------------
CHICK    ---------------LRRRTARTIDSLASRIEDPQIQA------------
XENTR    ---------------LRRRTARTIDSLASRIEDPQIQA------------
DANRE    ---------------LRSRTARTIDSLASRIEDPQIQA------------
CIOIN    ---------------VRKRVVSLIQTVTKSFQDPDVQA------------
ANOGA    ---------------TRRRTQYVLDTVARDVKDPMITS------------
TETNG    ---------------LRSRTARTIDSLASRIEDPQIQA------------
DROME    ---------------MRKRTQYVLDTLARDVKDPQIVS------------
BRUMA    ---------------LTNRVTTSLKRYLLRVTDPQVMW------------
CAEEL    ---------------LVTMVSDVAERVSRISLDPTVQY------------
YEAST*   ---------------YKLLLKKIIKDYVL--------------DIVPGSS
SCHPO    ---------------FYYNLNQESLEFQRWLKQRDISFKFMPNY------
CANAL    ---------------NLLNMKSVLDRLMH-------------------AH
CANGA    ---------------HKKLIRKILKECVL--------------EVVPDTE
ASPNI    ---------------HIQWLESLINDLRGVLQSAGIPC---------DFK
RHIO9    ---------------QFDRIRQVVHSILYPFRRLGFSV-----H------
CRYNE    ---------------LCSLVSSTLNGFDRTLRDAGLDS------------
USTMA    ---------------FVHRLGSVLKDFQETALQKGIKVVVQLVPITLPTS
ENCCU    ---------------NISQFSNELDELLAKKIDQHLCSLVERNKGDMDLF
ANTLO    GRVEDVVLDKQAVPDVVERIRQDIEQLKS--RDPLFES-----H-RWNCE
ARATH*   ---------------FSNRVLVHLESVVCKVPYLHLIS-----HPTWNSR
VITVI    ---------------FSSKVLMELENLVSRVPYLHLLS-----HPTWHSR
SOYBN    ---------------FSTKVLAELENVVCKVPYIQLIS-----NPTWHSR
SOLLC    ---------------FSNKVLAELESLVSRVPYVQLIS-----HPTWHSR
ORYSJ*   ---------------FSNKVLVELESVVSRVPYLHLRS-----LPTWHSR
HORVU    ---------------FSNKVQLELESVLSRVPYLHLQS-----LPTWHSR
SORBI    ---------------FSNKVHLELESVVSRVPYLHLCS-----LPTWHSR
SELML    ---------------AGDKIVSLLDTLVLQVPQLWFRS-----HPTWRPF
PHYPA    ---------------ASDKIISVLEKQVHGVPELRFIS-----HPTWHAQ
VOLCA    ---------------FRDHQIYHLDLEAHALPYITLRW-----R------
                  960       970       980       990      1000
```

MED17

```
HUMAN*  --HWSNINDV--YESSVKVL-----------ITSQGYEQI--CKSIQLQL
CHICK   --HWSNINDV--YESSVKVL-----------ITSQGYEQI--CKSIQLQL
XENTR   --HWSSINDV--YESSVKVL-----------ITSQGYEQI--CKSIQLQL
DANRE   --HWSNINDV--YESSVKVL-----------ITSQGYEQI--CKSIQLQL
CIOIN   --HWSTIGNM--FESSVRIL-----------IASSGFES---CYRTVVQL
ANOGA   --HWNAMNSP--TMSCVKIN-----------ITSHGYDA---NLRTSLVI
TETNG   --HWSNINDV--YESSVKVL-----------ITSQGYEQI--CKSIQLQL
DROME   --HWNAMNSP--TMSCVKIN-----------IVTHGYDA---IGRTSLVI
BRUMA   --KWLRATPV---QSSIIVT----------ASNRNYDY---AGKTTFYI
CAEEL   --RWLNCGRT---TSRMMFN-----------MTSKDFEMYIGTVRSVFFA
YEAST*  ITETEVEREQ--PQENKNID-------DENITKLNKEIR---AFDKLLNI
SCHPO   --PWEKAKDFLELENSLSIN------------------------------
CANAL   ESELSYSKLD--VEKFINLA-TRSKKQNPFQKSIEKPIS---KFHLVLCN
CANGA   LQERSIQSLD--KEDFETFD-----LQDAHIVKLTKDIN---AFRNVLDV
ASPNI   ATQFSSIGTL--QPSN-----QVPKVEAVAGVFLAPFLS---TFSGNLVT
RHIO9   ---YKILTDN--NSNNLHPS------------------------------
CRYNE   RMEKRMLKDG--QDEIKEMR------------------------------
USTMA   TRTGSSNLAQ--LTSLLSQM-----------------------MDATPSL
ENCCU   IKAQSVVGDV--LGHAIERNNFYRGLEFRCSAEVDRGNR---KFEGEVVE
ANTLO   SAAEKFVKRA--EQNVFQAK------------------------------
ARATH*  TSSWTVFMTV--PPSIIPQGRSETQSPDG---KRN-LKT---QFRTKVVV
VITVI   TSSWTLLMKV--PESSFHPG-CQTRTSDIHNVKNI-IKT---HFRTKVVI
SOYBN   ASSWTLYMEV--PQSILR-G-SQTKTSDYYE-KNA-VKR---QFWTKVVV
SOLLC   TSSWTLSMDV--PESILHAG-SLSHSSDYVKN----VKF---HFRTKVMV
ORYSJ*  TSSWSLCLKV--PQPILAAD-RIAKPSDNHELKYK-SRS---QFNTKVIV
HORVU   TSSWSLCLRI--PPPILAAD----KPSDNGEPKYKSSRT---QFNTKIVL
SORBI   TSSWSLCLKV--PQPILATD-RVRKPSDHHKLKYK-SRS---QFSTKVIL
SELML   VSAWDVYFDI--PEAIMNGG--QMKHLEWNALVRE-TSA---TFQCTLVL
PHYPA   VSTWDLWLDV--PDSALLGR-----------------------WQASVVL
VOLCA   ----SCSTSL--LTSAMEIR------------------VGP---SHRVLVLV
                 1010      1020      1030      1040      1050
```

```
HUMAN*  NIGVEQ---IRVVHRDGRVI---------------TLSYQEQELQDFLLS
CHICK   NIGVEQ---IRVVHRDGRVI---------------TLSHQEQELQDFLLS
XENTR   NIGVDQ---IRVVHRDGRVI---------------TLSHQEQELQDFLLS
DANRE   NICVEQ---IRVVHRDGRVI---------------TLSHQEQELQDFLLS
CIOIN   TLYSDH---IKALQREGRVL---------------TLSTEYSDLYLYFIT
ANOGA   HVKERS---LKCICRDGRIM---------------HMSYEPQELRDLILC
TETNG   NIGVEQ---IRVVHRDGRVV---------------TLSHQEQELQDFLLS
DROME   HVKERS---LKCICRDGRVM---------------RLSYEPQELRDLILC
BRUMA   RIGAEN---FYIATKEGQNI---------------ECRRDDEMLIYTIDM
CAEEL   NISSDG---VEVETKDGIKM---------------KCQRDPARVMYACQY
YEAST*  P-RRELKINLPLTEHKSPNL---------------SLMLESPNYCNALIH
SCHPO   --RLSISWRIMVSNFEPAIFIQHTPTLHGTDKSVWRCKDQYSSNQFSSLK
CANAL   KTSNMLDVNIQLTTNE--------------------------LFVNLIIN
CANGA   G-KTEFTIDMKQSGKL-------------------SLILESPNYCNAQVS
ASPNI   PQSSFR---IQIRTNLAVPPFGTFY----------ELSVNLPQYP-----
RHIO9   --CGKMSLYFNIGLNKGSHL---------------QFALNQQSGTISVIL
CRYNE   ---------AVLLGKRGAEVLTGEYSLGIDDSYIITINISAPYNTIVYLS
USTMA   PYRNSNAPDRNAGSNGTAGTLHSIYPTHANGSTLN---------------
ENCCU   QKKKEIDELFRMVTKND------------------DFGVDLLKLFSRVRK
ANTLO   --KAAI---VKICKRDGDVG------------------------------
ARATH*  --KDDC---ISVEAECTPNVVGLLKSSSSCNLFSINKYECDVADLPVMLQ
VITVI   --NDDC---INVEGDGAPNVVGLFKGSSEDVCSMNRYDCDLADIPVILLQ
SOYBN   --NDDC---INVKAEGSPNVAGLFKGKIEETHSINKYNCNLADLPVIILQ
SOLLC   --RDDC---ISLEGEGAPNIVGLFKGKPDSICPMNRYDCDLSDLPMVLLQ
ORYSJ*  --KDSQ---ISLMGEGSPSIAGSLTGKPSDGYLVNSYNCDLEDLPTMLLQ
HORVU   --KDVQ---ISLFGEGSPSIAGSLTRKPSDGYLINNYNCDLEDLPTMVLQ
SORBI   --KDGQ---ISLMGEGSPSIAGSLTGKPSDGRLINSYNCDLEDLPMMLLQ
SELML   --RQEI---LSIEGLDGCMPLG---ASGQESTGMKSCVCSVSELPFFLLT
PHYPA   --REDM---LTIAGLPS-----------GNGNRLTSTACTLSELSPFLLF
VOLCA   --RDGR---TDVEGVVQATGLPSAGSGAGGSRWVRDTHM-----------
                 1060      1070      1080      1090      1100
```

MED17

```
HUMAN*  ---------QMSQHQVH------------------------------AVQ
CHICK   ---------QMSQHQVH------------------------------AVQ
XENTR   ---------QMSQHQVL------------------------------AVQ
DANRE   ---------QMSQHQVH------------------------------AVQ
CIOIN   ---------LISSHQLQ------------------------------TVQ
ANOGA   ---------QISQHQIV------------------------------CLQ
TETNG   ---------QMSQHQVH------------------------------AVQ
DROME   ---------QINSHQIS------------------------------CLI
BRUMA   ---------LICNYMLN------------------------------TVA
CAEEL   ---------AMCCYSVT------------------------------MVS
YEAST*  IKFSAG--------------------------------------------
SCHPO   ---------NVCQYIEH------------------------------HIN
CANAL   MTIIRFETEDDFKNNVN------------------------------GIN
CANGA   IKYD----------------------------------------------
ASPNI   ---------DVQPPNHV------------------------------GLQ
RHIO9   ---------PQATVVLQ------------------------------HVS
CRYNE   SISFPLANPDELSQVVSDDLSVQLLRLASRHLHGKLADEHKKGLYFDSLE
USTMA   ---------ALQGHAKV------------------------------WIH
ENCCU   LRFPESINAEIKRTLVS------------------------------HIK
ANTLO   ---------QALVRELH------------------------------GVE
ARATH*  ---------QVASQIVC------------------------------WLL
VITVI   ---------QVASQVIR------------------------------WLH
SOYBN   ---------QVASQIIN------------------------------WLY
SOLLC   ---------QVASQVIR------------------------------WLH
ORYSJ*  ---------QVASQVIH------------------------------WLH
HORVU   ---------QVASQVIN------------------------------WLH
SORBI   ---------QVASQVIH------------------------------WLH
SELML   ---------QIAGKVIG------------------------------WLE
PHYPA   QRLIADVCWQMAAHLVT------------------------------WLH
VOLCA   ---------GFSRHDVP------------------------------CVL
                 1110      1120      1130      1140      1150
```

```
HUMAN*  QLAK----VMGWQVLSFSNHVGLGPIESIGN-ASAITVASPSGDYAISVR
CHICK   QLAK----VMGWHVLSFSNHVGLGPVESIGN-ASAITVASPNGDYAISVR
XENTR   QLAK----VMGWHVLSFTNHVGLGQVESVGN-ASSITVTSPNGDYAISVR
DANRE   QLAK----VMGWHVLSFSNHVGLGSIESIGN-ASSITVASPSGEYAISVR
CIOIN   ALSK----ILNWSLLHMAPHAGVCINNSVIN-RGTMLLASPSNDSKLSLS
ANOGA   NLAK----CMAWQILSSSSHLGIGSVEPLGN-ASSCVLASPNSDRLIAVQ
TETNG   QLAK----VMGWHVLSFSNHVGLGPVESIGN-ASAITVASPNGENAISVR
DROME   SLAR----CMSWTVLSNSNHLGIGKVEPLGN-ASSCLLASPNSDRMIAVQ
BRUMA   MIAS---KLWQWQVL----HANINATDDRAEPSPTVYMCNQSATRAIFMQ
CAEEL   TMSR----NNNWVTPFQTMCANVFALDERGNPAPNIVLCNQAATRSILFV
YEAST*  --------------------------------------------------
SCHPO   SLSR----------------------------------------------
CANAL   VLQL----------------------------------------------
CANGA   --------------------------------------------------
ASPNI   DEVS----------------------------------------------
RHIO9   ELRA-----------FLSREINIMCLGFICDVANDIIQIHPHSAQKMLLW
CRYNE   EVIR----IGETGLLRLSIPASFHTIYGSVESDWISVQAYDSRQSDITVF
USTMA   TQAG----------------------------------------------
ENCCU   DRFT----------------------------------------------
ANTLO   PLVA----------------------------------------------
ARATH*  EEAR---------------TVGTKASREFLS----LSLEIVEGER-VSLV
VITVI   EEAL---------------KVGIKANRDFLC----LSFEMDQGEM-LSLV
SOYBN   QEAM---------------MVGIKANRDFLC----LSLELEQGET-LGLV
SOLLC   EEAL---------------MVGIKANRDFLS----LSFELEQGET-LSLV
ORYSJ*  EEAL---------------VLGMNVTRDFLC----LYFDLEQGET-LGLV
HORVU   EETQ---------------VLGMSVTRDFLG----LYFDLDHGDTMLGLV
SORBI   DEAM---------------VLGMNVTRDFLC----LYFDLDQGET-LGLV
SELML   EEAV---------------LLGPRVKRDFLS----IVFNIQNNGL-VSLV
PHYPA   AEAS---------------GMGVEARLDSLS----ITFELDNFED-VSLV
VOLCA   RAVLQTLGVPLDRLLGVSALSAQNLAQAQQQLPLALAAQNHGGGSGVPVL
                 1160      1170      1180      1190      1200
```

MED17

HUMAN*	NGP---
CHICK	NGP---
XENTR	NGP---
DANRE	NGP---
CIOIN	ITHNTDH---
ANOGA	VRC---
TETNG	NGP---
DROME	IRC---
BRUMA	FHLNEPP---
CAEEL	FHVGMEP---
YEAST*	--
SCHPO	--
CANAL	--
CANGA	--
ASPNI	--
RHIO9	KLDRIDEVIHSSLC------------------------------------
CRYNE	AW--
USTMA	--
ENCCU	--
ANTLO	--
ARATH*	A-HVNPE---
VITVI	A-HVNPD---
SOYBN	A-SVDPE---
SOLLC	A-HVDPE---
ORYSJ*	A-NVDPD---
HORVU	A-HVDPD---
SORBI	A-HVDPD---
SELML	AA--
PHYPA	ASPA--
VOLCA	HGQMPPPPPVLAAPPSAPLLGRSGGGGGGGLSLAGHGAHLRHQPPLPTPL
	1210 1220 1230 1240 1250

HUMAN*	---ESG
CHICK	---ESG
XENTR	---ESG
DANRE	---ESG
CIOIN	---SIS
ANOGA	---DSQ
TETNG	---ESG
DROME	---DPQ
BRUMA	--
CAEEL	--
YEAST*	---TEA
SCHPO	--
CANAL	--
CANGA	---NQT
ASPNI	--
RHIO9	---TKE
CRYNE	--
USTMA	--
ENCCU	--
ANTLO	---EML
ARATH*	---DAK
VITVI	---DPQ
SOYBN	---DSE
SOLLC	---DIQ
ORYSJ*	---DTC
HORVU	---DAY
SORBI	---DTY
SELML	---PSG
PHYPA	---VKS
VOLCA	PPPQQQLQQLPDDLQSQDQQTQYQLQQQQQQLQYGGQTQPHQDTHSYSQG
	1260 1270 1280 1290 1300

MED17

Species	1301–1350
HUMAN*	SKIMVQFPRNQCKDLP-------KSDVLQDNKWSHLRGPFKEVQWNKMEG
CHICK	SKVMVQFPRSQCKDLP-------KGDVLQDNKWNHLRGPFKEVQWNKMEG
XENTR	TKVMVQFPRSQCKDLP-------KGDVLQENKWQYLRGPYKEVQWNKMEG
DANRE	CKVLVQFPRSQAKDAG-------RSDAVQDGKWTQLRGAHREVHWDRMEG
CIOIN	HNVRIQFSKSLDLKLNNQVFTETQDNLTLSSKYLGLGGEWKIVNWQNLHG
ANOGA	IDVKVFIAQSPAKDFF-------PGSLVQGRHWEHLGGHFKEVRFDKMEG
TETNG	CRVLIQFPRSQSKELP-------KNDVVQDAKWSHLRGPSKEVHWSRMEG
DROME	IDVKVYIARSPRQDFF-------PSPLVPEKLWENLGGTFKEVRFDKIEG
BRUMA	-------------------TIRVRKCSPNKPASAEQPGEFLVLNYDRLVG
CAEEL	-------------------EVFVRRFIVNEETMKPEEHEWKKLCYSRLHG
YEAST*	NAVSFDTTFSDFKEVEDFLHFIVAEYIQQKKV------------------
SCHPO	-----------------------RSKKTE---------------------
CANAL	-------GFSDFNEIEECLDWSIQNFV-----------------------
CANGA	SNTHFNTVSTEFKEVEEFLHFLISTYVNPE--------------------
ASPNI	AILTHVIMLDIAAAIPLQCRDTADKEAKERVSWDVDYPHHGELHFLSRNG
RHIO9	DQWKHINVQLFHDATSFHVQFIVTNETKEEKVFIKDTQEYSAMTFKEKVS
CRYNE	--
USTMA	------------ERLVCSLTFGQPSLVSVQFAGARAGAIHRKTRGVALTN
ENCCU	----------------------GAMSGKDQAPQEKKVVRGHLCSFYGYLR
ANTLO	CEVIRDGLRQ-------------RMTGDEKTRRRAVGDALVVVKFYRDSG
ARATH*	GCISWWLVME-------------NGCTEER-------EG--VSESRKLLG
VITVI	GCISWWLVMD-------------DGFSEDLKFHTDAPDG--GSEYRKFLG
SOYBN	GCISWWLVME-------------DSFAEEQKLHMSITDG--ASEYRKFLG
SOLLC	GCISWWLVMG-------------DGFSEENKLQMDVNSG--ESETRKFLG
ORYSJ*	GCISWYLTI--------------DHPTEDGKMSADSQ----EFEKRRFLG
HORVU	GCVSWYLTV--------------DHPAEEDGMTPAANGPWAEEEKCRFLG
SORBI	GCISWYLTV--------------DHPMEEGKMSADSP----ELEKRRFLG
SELML	YCINWWLRFY-------------SFGSDDTAAASGASAP----ASERFLG
PHYPA	HTINWSLRLS-------------GSRTEDSDATS------------RFLG
VOLCA	QSLQPNHMQQQQQPLSPPAPPPPPHQQQQQQQQQVAAATYQVHMHGQYLP

1310 1320 1330 1340 1350

Species	1351–1400
HUMAN*	RNFVYKMELLMSALSPCLL-------------------------------
CHICK	RNFVYKMELLMAALTPC---------------------------------
XENTR	RNFVYKMELLMAALTPC---------------------------------
DANRE	KNFVYKMELLMAALTPCP--------------------------------
CIOIN	RHFVQKMETLLTSLVSN---------------------------------
ANOGA	KNFHNKMEFLMASLTSQS--------------------------------
TETNG	RNFVYKMELLMAAMTPCP--------------------------------
DROME	KSFLNKMEFLMASLTSNTA-------------------------------
BRUMA	SSLCRKIDNLCSMLKS----------------------------------
CAEEL	ATLCRKIDALLVFLRDH---------------------------------
YEAST*	--
SCHPO	--
CANAL	--
CANGA	--
ASPNI	QSRKMKISLSRQELTIETYQLNRMRGVTPGAATSPTLWQTWRADSPETRP
RHIO9	K-------IIQEMMGDD---------------------------------
CRYNE	------LDTVTHAIESSLSSNGSTTQVR----------------------
USTMA	KPLAVDLDALAQLMAQQLGQYL----------------------------
ENCCU	NNEPSLQDLLGPTVEHEKS-------------------------------
ANTLO	IPAGVMEELLQ---------------------------------------
ARATH*	H---LSLDVLYSVLMDLI--------------------------------
VITVI	H---LSLEVLYSTLMDLV--------------------------------
SOYBN	H---LSLDLLYATLIDLV--------------------------------
SOLLC	Y---LSLEVLYSTLMDMV--------------------------------
ORYSJ*	Y---VSLEVLYSTLMDLI--------------------------------
HORVU	Y---LSLEVLYSTLMDLI--------------------------------
SORBI	Y---LSLEVLYSTLMDLI--------------------------------
SELML	P---LPLETLRAVVQRVIFVSPAKLKIHCSTQLAGTPTWRFSLGAYVSVT
PHYPA	P---LPLETLRAIVIDLMNEKLDVKPAGDASLRGEGFFGA----------
VOLCA	QTIQQQQQQLQQQTPLNQ--------------------------------

1360 1370 1380 1390 1400

MED17

```
HUMAN*   --------------------------------------------------
CHICK    --------------------------------------------------
XENTR    --------------------------------------------------
DANRE    --------------------------------------------------
CIOIN    --------------------------------------------------
ANOGA    --------------------------------------------------
TETNG    --------------------------------------------------
DROME    --------------------------------------------------
BRUMA    --------------------------------------------------
CAEEL    --------------------------------------------------
YEAST*   --------------------------------------------------
SCHPO    --------------------------------------------------
CANAL    --------------------------------------------------
CANGA    --------------------------------------------------
ASPNI    TLSEFVAQASQP--------------------------------------
RHIO9    --------------------------------------------------
CRYNE    --------------------------------------------------
USTMA    --------------------------------------------------
ENCCU    --------------------------------------------------
ANTLO    --------------------------------------------------
ARATH*   ---------------NLCGTGRNALERL----------------------
VITVI    ---------------SLCNGGGRH--------------------------
SOYBN    ---------------SLCSGGGSQ--------------------------
SOLLC    ---------------SVSSTAGH---------------------------
ORYSJ*   ---------------NLCNAGAHH--------------------------
HORVU    ---------------NLCGTGHRPTR------------------------
SORBI    ---------------KLCSTGVQH--------------------------
SELML    WINRTDARWTPAYEQNAVGGGKVVGEEANANAATGHFGPRRRRNRKQGDI
PHYPA    --------------------------------------------------
VOLCA    ---------------HQQQQQQQQQQQPYHQHHAQHLGPPQLQQQQQPPA
                  1410      1420      1430      1440      1450
```

```
HUMAN*   ----------------------------------------------
CHICK    ----------------------------------------------
XENTR    ----------------------------------------------
DANRE    ----------------------------------------------
CIOIN    ----------------------------------------------
ANOGA    ----------------------------------------------
TETNG    ----------------------------------------------
DROME    ----------------------------------------------
BRUMA    ----------------------------------------------
CAEEL    ----------------------------------------------
YEAST*   ----------------------------------------------
SCHPO    ----------------------------------------------
CANAL    ----------------------------------------------
CANGA    ----------------------------------------------
ASPNI    ----------------------------------------------
RHIO9    ----------------------------------------------
CRYNE    ----------------------------------------------
USTMA    ----------------------------------------------
ENCCU    ----------------------------------------------
ANTLO    ----------------------------------------------
ARATH*   ----------------------------------------------
VITVI    ----------------------------------------------
SOYBN    ----------------------------------------------
SOLLC    ----------------------------------------------
ORYSJ*   ----------------------------------------------
HORVU    ----------------------------------------------
SORBI    ----------------------------------------------
SELML    NISIssKHNATDFN--------------------------------
PHYPA    ----------------------------------------------
VOLCA    PYPQQQQQLQHGMQQQPQQPYGGVAGDTSRGLGTLGGRSGAWGPQG
                  1460      1470      1480      1490
```

MED18

```
HUMAN*   ------------------------MEAPPVTMMPVTGG---TINMMEYLL
CHICK    ------------------------MEAPPVTMMPVTGG---TINMMEYLL
XENTR    ------------------------MEAPPVTTMPVSGG---TINMMEYLL
DANRE    ------------------------MEAPPVTVMPVTGG---TINMMEYLL
CIOIN    --------------KISLCRLFSGIMVDAADYQPLSGDAPVIANMQEFFL
ANOGA    ------------------RTMGAQVNAAELLQQALSSN---IIPNQEFLL
TETNG    ------------------------MEAPPVTVMPVTGG---TVNMMEYLL
DROME    --------------------MAIVSSARESLSHAMNNR---FLPNLEYLL
BRUMA    ------------MERFPERSREIIASVPLITSTSPSSS---TYQCKECIL
CAEEL    --------------------MDNLVDIDEDGTQEKLSVNSTPYQTQECVL
YEAST*   -------------------------------------------MVQQLSL
SCHPO    --------------------------------------------MQELYL
CANAL    -------------------------------------------MVHQLSL
CANGA    -------------------------------------------MVQRLSL
ASPNI    --------------------------------------------MHELLL
RHIO9    ------------------------------------------MSNYECSL
CRYNE    -----------------------------------MASTSSQTTPLEPTL
USTMA    MVLTAASRAQANAAGSTAAGSSSATTSATASSATTSSSSSAWAQQSQVSL
ENCCU    ------------------------------------MDGSISPRIKRLVD
ANTLO    ----------------------------------------MEEKLNELLK
ARATH*   -------------------------------------------MSMECVV
VITVI    ---------------------------------------------MECVV
SOYBN    ---------------------------------------------MECVV
SOLLC    ---------------------------------------------MECVV
ORYSJ*   ---------------------------------------------MECVV
HORVU    ---------------------------------------------MECVV
SORBI    ---------------------------------------------MECVV
SELML    ---------------------------------------------MECVV
PHYPA    ---------------------------------------------MECVV
VOLCA    --------MSAAAAAAGPGAAAAAGGAAAASAAAANAAPSGPASLTECRA
                  10        20        30        40        50
```

```
HUMAN*   QGSVL-DHSLESL--IHRLRGLCDNME--PETFLDHEMVFLLKGQQA---
CHICK    QGSVL-DQSLESL--LHRLRGLCDNME--PETFLDHEMVFLLKGQQA---
XENTR    QGSIL-DQGLESL--LHRLRGLCDNME--PETFADHESVYLLKGQQA---
DANRE    QGSVL-DQSLESL--LHRLRGLCDNME--PESFADHELVYLLKGQQG---
CIOIN    QGSVL-DTCVDKI--HERLQGLCDNVS--PEAFTDHERTYVMRNTTSN--
ANOGA    QGSIL-DSAAENL--LHRLRGLCDNVDASPETFSDIEMCFSLKLPTEK--
TETNG    QGNM----------------------------------------------
DROME    QGSIL-DSAVEHL--MHRLKGLCDNVDTSPEPFHDLEVCMSLRQPNAN--
BRUMA    YGSVL-KEHEEKL--LQRLRGLCDPG---QHSFNEHEMVFSLKTG--Q--
CAEEL    YGSIF-AKNVPDL--ERRLAGLCDPG---CEEFFEHEMSFSLRSSGIM--
YEAST*   FGSIG-DDGYDLL--ISTLTTISGNP---PLLYNSLCTVWKPNPSYDVEN
SCHPO    LGVVP-SRRFEAV--VNSLSKTLDG----PKTILEFWVVYRPKDVPPNL-
CANAL    VSSIPHNKYLQTISTLQALTGLIQ-----PESISTYTLLAKPSYAFKPKF
CANGA    MATIA-DDDFPLF--KTSINILTGSP---AVVYANLMVVWKPNPNYEVDN
ASPNI    FASVP-SHQHHEL--LQQLTGLTAMQ---PRHCLERRLIFKATR------
RHIO9    QGLIIGHQQKDKL--IERLEGICGNNS--MVDLFEHEIIFTPTVGP----
CRYNE    YSLLP-PSLHTSI--LSHLSLLAIH----AEPLHLIERIYTTT-------
USTMA    LGTLP-MHVVPAL--LGRIESQLVTLP--ARVLCEKETVLSRHD------
ENCCU    SGIFK-DPEIDRLGYGTFQKQQGAE----PNQSVRRARDLRARVGAVL--
ANTLO    KGVFN-DVRLSRLGYGTFRKIQAPVVYTKSEELSKEAAVLKKRALSLM--
ARATH*   QGIIE-TQHVEAL--EILLQGLCGVQ---RERLRVHELCLRSGPNLGV--
VITVI    QGIIE-TQHVEAL--EILLQGLCGVH---RERLRIHELCLKSGPNLGL--
SOYBN    QGIIE-TQHVEAL--EILLQGLCGVQ---RERLRIHEICLKSGPHLGT--
SOLLC    QGIIE-TQHVEAL--EILLQGLCGVH---KQSLRIHELCLKSVPNLGL--
ORYSJ*   QGIIE-TQHVDAL--EVLLQGLSGVP---KERVRVHELCLKSGPNLGV--
HORVU    QGIIE-TQHVEAL--EVLLQGLSGVP---KERVRVHELCLKSVPMLGA--
SORBI    QGIIE-TQHVEAL--EVLLQGLSGVP---KERVRVHELCLKSGPNLGV--
SELML    QGIIE-AQHVEAL--DVLLQGLCEGQ---REAVKTHEICLKKSLETGT--
PHYPA    QGILE-AKHVDAL--EVLLQGLCGGL---RETIKVHEICLKTGPNLGA--
VOLCA    VGILDRPEHIGGL--ANLLRSLCSSYCTHAVHMVVLRMPLPPQPGAPPGG
                  60        70        80        90       100
```

MED18

```
HUMAN*   ------------------------------------------SPFV-LRA
CHICK    ------------------------------------------SPFV-LRA
XENTR    ------------------------------------------SPFV-LRA
DANRE    ------------------------------------------NPFI-LRA
CIOIN    ------------------------------------------MPIT-VKA
ANOGA    ------------------------------------------TPVMTVRV
TETNG    --------------------------------------------------
DROME    ------------------------------------------QPLL-LRV
BRUMA    ------------------------------------------DPDVTVRL
CAEEL    ------------------------------------------DIKTDIKL
YEAST*   VNSRNQLV----------------------------------EPNR-IKL
SCHPO    ---------------PRQ------------------------PDSW-LRL
CANAL    EPGKVNQIEQYYMRCITTWNSDKQGDDEKVEKGFDISEPFINKESN-IVV
CANGA    LNSKNELV----------------------------------EPTR-MTT
ASPNI    -----KPDLANMRSGTGQ------------------------GPDI-VRL
RHIO9    ---------------SRN------------------------DDVV-LRV
CRYNE    -----NPV-------LPG------------------------QTRN-LRF
USTMA    -----DHASAVRETGDNA------------------------WASV-VKA
ENCCU    ------------------------------------------KESR-REG
ANTLO    ------------------------------------------ESAD-IRS
ARATH*   ------------------------------------------VSSE-VRL
VITVI    ------------------------------------------VASE-VRV
SOYBN    ------------------------------------------VASE-VRL
SOLLC    ------------------------------------------VASE-IRL
ORYSJ*   ------------------------------------------VPSE-VRL
HORVU    ------------------------------------------VPSE-VRL
SORBI    ------------------------------------------VPSE-VRL
SELML    ------------------------------------------VASE-LHL
PHYPA    ------------------------------------------VASE-VHL
VOLCA    ------------------------------------------TSAL-LRL
                  110       120       130       140       150
```

```
HUMAN*   RRSM----------------------------------------------
CHICK    RRSM----------------------------------------------
XENTR    RRPL----------------------------------------------
DANRE    RRSL----------------------------------------------
CIOIN    CQSLV---------------------------------------------
ANOGA    RRAQ----------------------------------------------
TETNG    --------------------------------------------------
DROME    RRAL----------------------------------------------
BRUMA    RRKFGGP-------------------------------------------
CAEEL    RRRFRTE-------------------------------------------
YEAST*   SKEVPFSYLIDETMMDKPLNFRILKSFTNDKIPLNYAMTRNILHNTVPQV
SCHPO    CSNIESH-------------------------------------------
CANAL    RKLFTEE-------------------------------------------
CANGA    ATSIPLELILNDPVSKN---------------------------------
ASPNI    NKMLNGQMFY----------------------------------------
RHIO9    QSRITTE-------------------------------------------
CRYNE    RSIR----------------------------------------------
USTMA    RKRIKLRVRE----------------------------------------
ENCCU    AKMLMEI-------------------------------------------
ANTLO    AKQCQID-------------------------------------------
ARATH*   LCDL----------------------------------------------
VITVI    LCDL----------------------------------------------
SOYBN    LCDL----------------------------------------------
SOLLC    LCDL----------------------------------------------
ORYSJ*   LCDL----------------------------------------------
HORVU    LCDL----------------------------------------------
SORBI    LCDL----------------------------------------------
SELML    LCDL----------------------------------------------
PHYPA    CCSL----------------------------------------------
VOLCA    SRRLPGSGLLTPQGGAA-------------------RGPQHPFL------
                  160       170       180       190       200
```

MED18

```
HUMAN*  -----------------------------------------DRAGAPW--
CHICK   -----------------------------------------DKSGMPW--
XENTR   -----------------------------------------DRPGAPW--
DANRE   -----------------------------------------LDPSVPW--
CIOIN   -----------------------------------------DKSAVPW--
ANOGA   -----------------------------------------DV-EAPL--
TETNG   --------------------------------------------------
DROME   -----------------------------------------GR-DAPF--
BRUMA   -----------------------------------------DANSFQW--
CAEEL   -----------------------------------------HRIQNYW--
YEAST*  TNFNSTNEDQNNSKHTEDTVNESRNSDDIIDVDMDASPAPSNESCSPW--
SCHPO   -----------------------------------DETDTEWSKNTQW--
CANAL   -----------------------------------------DSVERVW--
CANGA   -NGVDSDEILNTMKEQEFPIKQEQLFDMINNP---------SNNTINW--
ASPNI   ----TQVVGPLSEADFDGKPSSSDSQDVSMSGTHEAAGSSYDYDSQPW--
RHIO9   --------------------------------------KEKSFRFRQW--
CRYNE   -----------------------------IAGTVRDKGKGKEKDDGDWVH
USTMA   ----AIKRYNTSSMHRSD---------------------CHTVDSVEC--
ENCCU   --------------------------------------------------
ANTLO   --------------------------------------------------
ARATH*  -----------------------------------------DQPEPTW--
VITVI   -----------------------------------------EQPEPTW--
SOYBN   -----------------------------------------EQAEPSW--
SOLLC   -----------------------------------------EQPEPAW--
ORYSJ*  -----------------------------------------AQSTPSW--
HORVU   -----------------------------------------AQPTPSW--
SORBI   -----------------------------------------AQPTPSW--
SELML   -----------------------------------------SKRPQIW--
PHYPA   -----------------------------------------SQPEPVW--
VOLCA   -----------------------------------------EDPPERW--
                 210       220       230       240       250
```

```
HUMAN*  HLRYLGQPEMG---------------------------------------
CHICK   HLRYLGQPEIG---------------------------------------
XENTR   HLRYLGQPEAG---------------------------------------
DANRE   HLRYLGQPEVG---------------------------------------
CIOIN   QIKYMGNTDVG---------------------------------------
ANOGA   QLRYIGQPELG---------------------------------------
TETNG   --------------------------------------------------
DROME   QMRYLGNPEV----------------------------------------
BRUMA   HFRYMGAAEPD---------------------------------------
CAEEL   QLKYIGVPEPD---------------------------------------
YEAST*  SLQISDIPAAG---------------------------------------
SCHPO   SMYLEGNS--E---------------------------------------
CANAL   TLQVSDIPVAGKNQGCCQQQIYESTLVHTHTAIEIKVGNGDPIDIDTNND
CANGA   TLTTADIPAAG---------------------------------------
ASPNI   RLEFRDTPEAG---------------------------------------
RHIO9   YLCMQGNP--E---------------------------------------
CRYNE   TLAYVSSRLRS---------------------------------------
USTMA   TLCLALPS--L---------------------------------------
ENCCU   VLMYIKGYMEE---------------------------------------
ANTLO   AIFCLLQAILA---------------------------------------
ARATH*  TVKHVGGAMRG---------------------------------------
VITVI   IVRHIGGAMRG---------------------------------------
SOYBN   TVKHVGGAMRG---------------------------------------
SOLLC   TVRHVGGPMRG---------------------------------------
ORYSJ*  TIRHVGGAMRG---------------------------------------
HORVU   TIRHVGGAMRG---------------------------------------
SORBI   TIRHVGGAMRG---------------------------------------
SELML   TVKHIGGAMRG---------------------------------------
PHYPA   SVRHVGGAMRG---------------------------------------
VOLCA   EVRHEGPPLQG---------------------------------------
                 260       270       280       290       300
```

MED18

HUMAN*	--
CHICK	--
XENTR	--
DANRE	--
CIOIN	--
ANOGA	--
TETNG	--
DROME	--
BRUMA	--
CAEEL	--
YEAST*	--
SCHPO	--
CANAL	KQGDNNTDKPKQEHDGKLPEAIDEDIIKNGDEKKTTHDDNDSDIMEIDEP
CANGA	--
ASPNI	--
RHIO9	--
CRYNE	--
USTMA	--
ENCCU	--
ANTLO	--
ARATH*	--
VITVI	--
SOYBN	--
SOLLC	--
ORYSJ*	--
HORVU	--
SORBI	--
SELML	--
PHYPA	--
VOLCA	--
	310 320 330 340 350

HUMAN*	---DKNRHALVRN--CVD-----IATS-----------------------
CHICK	---DKNRHALVRN--CVD-----IATS-----------------------
XENTR	---DRSRHTLVRN--CVD-----IATS-----------------------
DANRE	---DKSRHALVRN--CVD-----VAAS-----------------------
CIOIN	---DRTKSAMIRS--SVS-----VAVS-----------------------
ANOGA	---DRTRPTLVRS--SLD-----IACT-----------------------
TETNG	--
DROME	---DLRRPTLVRS--CMD-----CACT-----------------------
BRUMA	----VHCPTIIRK--SIDS----LIYS-----------------------
CAEEL	----QKCPTIVRK--EISS----LVHS-----------------------
YEAST*	-NNRSVSMQTIAETIILSS----AGKN-----------------------
SCHPO	-P-KREDKCGIRP--VNRA----KLTN-----------------------
CANAL	NPETQTLPQSLSNGVSQR-----TTRK-----------------------
CANGA	-SNRKVSMQAIQEATIVHQ----AGNN-----------------------
ASPNI	----FRSTITARL--AASA----ALPK-----------------------
RHIO9	-P-QRARTVTVRP--IAR-----VQLS-----------------------
CRYNE	-A--EYSEAAVRA--VMSVEVQDMSSS-----------------------
USTMA	-PERQYPKASVRP--CYNV----ELLSDTPIGPHHDMAKHPIHPLTDTHP
ENCCU	-SARCRVVDMIRRWKGLA--------------------------------
ANTLO	-MSQCDISKIVSEYRALA--------------------------------
ARATH*	-AGADQISVLVRN--MIE-----SKVS-----------------------
VITVI	-SGAEQISVLVRT--MVE-----SKVS-----------------------
SOYBN	-AGAEQISVLVRS--MVE-----SKTS-----------------------
SOLLC	-AGAEQISVLVRP--MQE-----SKIS-----------------------
ORYSJ*	-AGAEQISVLVRS--IVE-----SKAS-----------------------
HORVU	-AGAEQISILVRT--IVE-----SKAS-----------------------
SORBI	-AGAEQISVLVRT--IVE-----SKAS-----------------------
SELML	-TGAEQLPALVRN--IVE-----SKMS-----------------------
PHYPA	-AGADQLPALVRP--VLE-----AGIS-----------------------
VOLCA	-PALNTLPAAMRE--VLDT----ACFG-----------------------
	360 370 380 390 400

MED18

HUMAN*	---------------ENLTDFLMEMGFRMDHEFVAKGHLFR-------KG
CHICK	---------------DNLTDFLVEMGFRMDHEFVAKGHVFR-------KG
XENTR	---------------DVLPEFLQEMGFRMDHEFVARGHLFR-------KG
DANRE	---------------HSLPDFLNEMGFRMDHEFVAKGQVFR-------KG
CIOIN	---------------KDPSEFLEDLGFVLQFEVILKGYFYR-------KG
ANOGA	---------------PHVIDFLTEMGFRLDFEYSTKGYMFR-------KG
TETNG	---------------------------XXXFRMDHEFVTSGHIFR-------KS
DROME	---------------NGILEFLTEMGFRLEFEYIAKGYMFR-------KG
BRUMA	---------------SNMMEFVKTLGLRMDYEYLTKGYLFT-------KG
CAEEL	---------------QDMMTYAKSLGLRMDYEYMTQGKLWT-------KG
YEAST*	---------------SSVSSLMNGLGYVFEFQYLTIGVKFFM------KH
SCHPO	---------------GSVTEFVEKMGYEFSHEYIIQGLEYFFF-----DT
CANAL	---------------DSFLIFLSDLGYEVINQYWQKGVRFF-------YG
CANGA	---------------PTLANVMAELGYSFDYMYVTVGVRFVH------KR
ASPNI	---------------GDVVPSMNAWGYSFVTEYVVEGDMFI-------YN
RHIO9	---------------GDMFRFMKALGYSYSFEVVKKGHLIAY------DN
CRYNE	---------------RDIEDFVKALGFRHSHTFTLTGHLLHLPIPIPTSP
USTMA	LLGFNPDSYLDTDLVNGWKSFVSSIGWRPHFKFLRYGLTFPLNDFLSRHS
ENCCU	---------------KYIAEAMEELGEEEAGTFLR---------------
ANTLO	---------------NYINEVLAFYNTKDHAKFVA---------------
ARATH*	---------------KNALRMFYALGYKLDHELLKVGFAFHFQR----TA
VITVI	---------------KNVLRLFYALGYRLDHELLRVGFAFHFQR----GA
SOYBN	---------------KNALRMFYTLGYKLDHELLRVGFSFNFHR----GA
SOLLC	---------------KNALRLFYSLGYKLDHEQLRVGFAFHFQR----GA
ORYSJ*	---------------NNVLRYFYGIGYKLDHEVLKGGFAFRFHR----GA
HORVU	---------------SNVLRYFYGIGYKLDHEILKVGFAFRFQR----GA
SORBI	---------------KNVLHYFYTLGYKLDHELLKIGFAFRFHR----GA
SELML	---------------NNALRFFYAVGYKMDYELLRDGFAYRFQR----AF
PHYPA	---------------NNSLRFMYSLGYKLDYEMLRTGFSFQLHKTVPIAV
VOLCA	---------------SGTLEFWKALGGKLEYEMYREGNEYSIIFH---GH
	410 420 430 440 450

HUMAN*	IMKI--------------MVYKIFRILVPGNTDST--------------E
CHICK	IMKI--------------VVYKIFRILMPGNTDSI--------------E
XENTR	VMKV--------------AVYKVFRVLVAGAAEGA--------------E
DANRE	VMKV--------------VVSKLSRVLVPGNTDNT--------------E
CIOIN	RLKI--------------TVAKLSGSIDPNKH-----------------E
ANOGA	RMKI--------------TVSKILK---------NMT------------E
TETNG	AMKI--------------VVSRLLRIVVPGNTENT--------------E
DROME	RMKI--------------TVSKLIKIV-PGKQQDMAN------------E
BRUMA	YIKI--------------IINNITRTEKIGTYDPNVL------------K
CAEEL	NIKI--------------LHSILTSTSRAGTYDSNSI------------K
YEAST*	GLIL--------------ELQKIWQIEEAGNSQITSG-------------
SCHPO	TVRIYQT-----------LIPSQQRSIKPPFHPMNEE-------------
CANAL	DIII--------------EIYKIFIRDESSQADSNEGIKLKLLDD-----
CANGA	DVII--------------HCHKVWDIAEDNIIQVTKG-------------
ASPNI	DIVI--------------FLHRVLQYPATGQEPHEPRRQLPSFRDLAPLE
RHIO9	VLKI--------------TVSRLHKLRNKVDVSSAVP------------F
CRYNE	PLTL--------------HLSITRLTPSASTTDEQSA-------------
USTMA	STQIPALTNPAHGTMHTLNVFRIFTPDPSGYTD-----------EWTPLD
ENCCU	---------------------TVLFNVKFHYLHLESSLIAKQGKKSEGRE
ANTLO	------------------SLKYALFNIKFHYLHLEGILMQRSIESAQHFN
ARATH*	HISV--------------SVSSVNKMPKVHAIDEAVP-------------
VITVI	SITV--------------TVSSVNKMLKLHATDEAVP-------------
SOYBN	QITV--------------TVSSINKMLKLHATDEAVP-------------
SOLLC	QITV--------------TVSSINKMLKPHAIDDAVP-------------
ORYSJ*	QITV--------------TVTSVSKMTKLHATNEAVP-------------
HORVU	QFTV--------------TVTSANKMPKLHATDEAVQ-------------
SORBI	QITV--------------TVTSTNKMPRLHATDEAVP-------------
SELML	PITV--------------TVTSVCRLPKQHSIDEATP-------------
PHYPA	TITI--------------SVTAVHRLPKIHAVDDPQP-------------
VOLCA	EIKV--------------VLASVYALAEPHVLKDASS-----LKRVAPGS
	460 470 480 490 500

MED18

```
HUMAN*  ALSLSYLVE------LSVVAPAGQD-------------------------
CHICK   PLSLSYLVE------LNVVAPAGQD-------------------------
XENTR   PLSLSYLVE------LSAVAPAGQD-------------------------
DANRE   PLSLSYLVE------LSVLAPAGQD-------------------------
CIOIN   AGNMSHLIE------VSALASPLDE-------------------------
ANOGA   PISQSYLVE------LSVLAPKGQD-------------------------
TETNG   RVSSSYLVE------LSVLAPAGQD-------------------------
DROME   PISQSYIVE------LSVVAPTGQE-------------------------
BRUMA   PLSDSLLIE------MSAALPDTKE-------------------------
CAEEL   SVSDSALVE------ISISLPESAE-------------------------
YEAST*  ----GFLLK------AYINVSRGTDI------------------------
SCHPO   ---QPWILH------VYTHVADASNQ------------------------
CANAL   --SNQFQIK------AYININKSTEI------------------------
CANGA   ----GYVVH------TFVTVNRATDI------------------------
ASPNI   -KTGSYILQ------AAITVQDGSNQ------------------------
RHIO9   GQEDSWIVE------VTTLPVPQEQ-------------------------
CRYNE   -PDEPYLVQLQPSRPVYAIAPPGDI-------------------------
USTMA   PDGATVVVE------LIATVGAQLDQSHFNIYCSTTDAGAAQTGGGLDPT
ENCCU   SILVYFLNE----------------------------------------Y
ANTLO   AKHFLYFLK---------------------------------------EY
ARATH*  VTPGMQIVD--------VTAPATSE-------------------------
VITVI   VTPGIQLVE--------VTAPASSE-------------------------
SOYBN   VTPGIQMVE--------VTAPATAE-------------------------
SOLLC   VTPGIQLVE--------VTAPASSE-------------------------
ORYSJ*  ITPAIQLVE--------ITAPAAAD-------------------------
HORVU   VTPGIQLVE--------ITAPAAAN-------------------------
SORBI   VTPGIQLVE--------ITAPAAAD-------------------------
SELML   VTPNIQLVE--------VTAPATAD-------------------------
PHYPA   VTPGLQMVQ--------VSAPASPD-------------------------
VOLCA   SGSGNLVVE------VSLLVAEGRHY------------------------
                 510       520       530       540       550
```

```
HUMAN*  ----MVSDDMKNFAEQLKPLVHLEKIDPKRLM------------------
CHICK   ----IVSDDMRNFAEQLKPLVHLEKIDPKRLM------------------
XENTR   ----NIADEVRGFAEQLRPLVQLEKIDPKRLM------------------
DANRE   ----TVSEDMRSFAEQLKPLVHLEKIDPKRLM------------------
CIOIN   ----SAGDEVRIFSQFLRPIVFLDKVDVRRLQQKT---------------
ANOGA   ----AIAEDMRIFAEQLKPLVQLEKIDYKRFAQMP---------------
TETNG   ----TVSEDMRSFAEQLKPLVHLEKIDPSKQRH-----------------
DROME   ----NVGEEMRVFAEQLKPLVQLEKIDYKRLGGMP---------------
BRUMA   --YMTTAKALRSFADQLSPICDMQK-NYKRQDPNFPEKISTFYLSIKPVS
CAEEL   --YMPAAKSLRDFADQLMPLVNMEKVDYWKKMFPAQAAPRR---------
YEAST*  DRINYTETALMNLKKELQGYIELSVPDRQSMDSRVAHGNILI--------
SCHPO   VAMAKAEANLTKVKTLLSAFCDLKNVRL----------------------
CANAL   DSINSGVKELIKLQEFLKNLFVLEIPDRMFMDSRIKQ-------------
CANGA   ERLNLAEANLLSLQRDLSGYLDFKVPDRKSMDSRTNLTQKDITL------
ASPNI   ELMRTASQHLFGLREQLKSAVRLEHADRLSLDTRAR--------------
RHIO9   --VNQMAEHLNKFKTLLTAIVDLEYVDTRALQNKVHYTS-----------
CRYNE   -GLQDMIGVMQSTAGRLDGL------EWSTGQ------------------
USTMA   STIENAIEFADSVAKTLRGLVDLKREAYD---------------------
ENCCU   NDLYSIFASSKAKGFSVLQLCDLEDMIREKINSM----------------
ANTLO   RSLSEIFNESPLVDFILARVDDLEKTMKTKMRIQKKP-------------
ARATH*  -NYSEVAAAVSSFCEFLAPLVHLSKPSISTGVVPTAAAAAASLMSD---G
VITVI   -NYSEVAAAVSSFCEYLAPLLHLSKPGTSTGVVPTAAAAAASLMSD---A
SOYBN   -TYAEVASAVSSFCEYLAPLLHLSKPGISTGVVPTAAAAAASLMSD---G
SOLLC   -NYNEVVASVTSFCEYLAPLLHLSKPGVSTGVVPTAAAAAASLMSD---G
ORYSJ*  -NYNDVVSAVTSFCEYLAPLLHLSKPGNSTGIVPTAGAAAASLMSSGGGG
HORVU   -NYNDVVSAVTSFCEYLAPLLHLSKPGNSTGIVPTAGAAAASLMSS---G
SORBI   -NYNDVVSAVSAFCEYLAPLLHLSKPGHSTGIVATAGAAAASLMSS---G
SELML   -NFSDVVPAISSFAEHLSPLVNLSKPGSFAGIVSTASTAAASLLSR---S
PHYPA   -NYSEVVGAISGLAEYLAPLVHLSKPGVPAGIVPTASTAAAALVSR---G
VOLCA   ----EGANLLGAFGAQLAPYVRLIRAEVPPAAAAAAAAAAAASRRK----
                 560       570       580       590       600
```

MED18

HUMAN*	--
CHICK	--
XENTR	--
DANRE	--
CIOIN	--
ANOGA	--
TETNG	--
DROME	--
BRUMA	LNIKSVGFYNESNIVSLLMELNCIMKLIKPDNFYKSIQKKYDMD
CAEEL	--
YEAST*	--
SCHPO	--
CANAL	--
CANGA	--
ASPNI	--
RHIO9	--
CRYNE	--
USTMA	--
ENCCU	--
ANTLO	--
ARATH*	GGTTL---------------------------------------
VITVI	GVTT--
SOYBN	GGTNL---------------------------------------
SOLLC	GGTK--
ORYSJ*	GGKTL---------------------------------------
HORVU	GAKTL---------------------------------------
SORBI	GGKTL---------------------------------------
SELML	DRLEHRV-------------------------------------
PHYPA	GITKVL--------------------------------------
VOLCA	--

610 620 630 640

MED19

```
HUMAN*   --------------------------------------------------
CHICK    --------------------------------------------------
XENTR    --------------------------------------------------
DANRE    --------------------------------------------------
CIOIN    --------------------------------------------------
ANOGA    --------------------------------------------------
TETNG    --------------------------------------------------
DROME    --------------------------------------------------
BRUMA    --------------------------------------------------
CAEEL    --------------------------------------------------
YEAST*   --------------------------------------------------
SCHPO    --------------------------------------------------
CANAL    --------------------------------------------------
CANGA    --------------------------------------------------
ASPNI    MSDRISSAPFRTGPPSPSPSSPAPFTSLKENTYSTIPPEQIPQTPTSPPL
RHIO9    --------------------------------------------------
CRYNE    --------------------------------------------------
USTMA    --------------------------------------------------
ARATH*   MEP-----ERLKFG------------------------------------
VITVI    M-------------------------------------------------
SOYBN    MDP-----EGKKFGG-----------------------------------
SOLLC    MDP-----DNKRFGR-----------------------------------
ORYSJ*   MDS-----DDKKFGK-----------------------------------
HORVU    MDS-----DDKKFGK-----------------------------------
SORBI    MSSSNQMCSDGKFGR-----------------------------------
PHYPA    MAGGPQSKRRESNSKHDSSAKQPHKRGRGITKATTTNPSVRCVGAVGRGT
                  10        20        30        40        50
```

```
HUMAN*   -------------MENFTALFGA-------QADPPP--PPTALGFGPGKP
CHICK    -------------MENFSALFGG--------AEPPPATAAAALGFGPAK-
XENTR    -------------MEGFSGLFAASEPSSIPSAPGPPSVVPTCTGAFVSGK
DANRE    ------------MTEIFSSLYGQ------PDSQGPA--GPSALGFGSGKP
CIOIN    --------------------------------------------MMEFPN
ANOGA    ----------------MFNNYGNTMASDPFRKVEQYSPKSSPRAGGAGGR
TETNG    ------------MTEMFSTLFGQ------NEAQGPP--GSSSLGFGPSKP
DROME    ----------------MMSNYGNMMD----------SPKSSPHG---GGR
BRUMA    -------------------------------------------------M
CAEEL    --------------------------------------------------
YEAST*   --------------------------------------------------
SCHPO    --------------------------------------------------
CANAL    --------------------------------------------------
CANGA    --------------------------------------------------
ASPNI    MSVSATNDASNLANLQASSQATSQTASLSSPPSTAPMTTQNSQQPTAGAT
RHIO9    --------------------------------------------------
CRYNE    -------------------------------------------------M
USTMA    --------------------------------------------------
ARATH*   --------------------------------------------------
VITVI    --------------------------------------------------
SOYBN    --------------------------------------------------
SOLLC    --------------------------------------------------
ORYSJ*   --------------------------------------------------
HORVU    --------------------------------------------------
SORBI    --------------------------------------------------
PHYPA    WMWGDGCEGFMQVFASASGRNAGLHLALVLELHLGMQGRLPDLREPYSIW
                  60        70        80        90       100
```

```
HUMAN*   PPPPPPPAGGGPGTAPPPTAATAPPGADKS-G--AG--------------
CHICK    --------AAGTGAAPPPAAAVPPPGEDAARK--AA--------------
XENTR    TQTSTPTVPGPPLPIPPPLSSSSS-GEDSSRR--PA--------------
DANRE    QVP----QNMGPMCFPHQMMEEGA----PVRKPAAM--------------
CIOIN    SNFHFDQTQTLLDSNTANFGTNHD----IVKS--TH--------------
ANOGA    SPVVARQDSSGTLKTTIQLG-KNP------SI--LH--------------
TETNG    PPPLPQSQVALAAQMPPQHGDEGP----ALRKPGAM--------------
DROME    SPVVARQDSSGTLKTTISLG-KTP------TI--IH--------------
BRUMA    DSIGSGVASSGSLRTKISLKGRYS------QL--SL--------------
CAEEL    --MNEPSACSGP-----PVK---S------KS--SK--------------
YEAST*   ------------------MASRVD------ET--TV--------------
SCHPO    -------------------------------M--AQ--------------
CANAL    -------------------MTTLP------NN--TQ--------------
CANGA    ---------------------MEM------EE--FA--------------
ASPNI    NSFPTPASSVGPDHMNKSFGTDFS------ET--GASNTTGASAVPTQQS
RHIO9    --------------------------------------------------
CRYNE    DIIQNGESSKSPHAQGINGDLTKA----DGDV--EMMDGENGVGVELGRP
USTMA    --MSTDMAASALHSHGHDALASTS------HS--SH--------------
ARATH*   --------------------------------------------------
VITVI    --------------------------------------------------
SOYBN    --------------------------------------------------
SOLLC    --------------------------------------------------
ORYSJ*   --------------------------------------------------
HORVU    --------------------------------------------------
SORBI    --------------------------------------------------
PHYPA    FSSFVSQSAAGMASNG----------------------------------
                 110       120       130       140       150
```

MED19

```
HUMAN*  --------------------------------------------------
CHICK   --------------------------------------------------
XENTR   --------------------------------------------------
DANRE   --------------------------------------------------
CIOIN   --------------------------------------------------
ANOGA   --------------------------------------------------
TETNG   --------------------------------------------------
DROME   --------------------------------------------------
BRUMA   --------------------------------------------------
CAEEL   --------------------------------------------------
YEAST*  --------------------------------------------------
SCHPO   --------------------------------------------------
CANAL   --------------------------------------------------
CANGA   --------------------------------------------------
ASPNI   EHRRTDHNRDSKSARARQAVKDSQQLGESGHAPHGSAMDLDTERPAQTNA
RHIO9   --------------------------------------------------
CRYNE   EMKNGDERKRGTARLIMPHWDVA---------------------------
USTMA   --------------------------------------------------
ARATH*  --------------------------------------------------
VITVI   --------------------------------------------------
SOYBN   --------------------------------------------------
SOLLC   --------------------------------------------------
ORYSJ*  --------------------------------------------------
HORVU   --------------------------------------------------
SORBI   --------------------------------------------------
PHYPA   --------------------------------------------------
                 160       170       180       190       200
```

```
HUMAN*  ------------CGPFYLMREL--P---GSTELTGSTNLITHYNLEQAYN
CHICK   ------------AGPFYLLREL--P---GSTELTGSTNLITHYNLEHAYN
XENTR   ------------AGPFYILREL--P---GLSDLTGSVNLILHYNLEHSFS
DANRE   ------------NEPFYLLREL--P---MENELTGHTNLITHYNLEHAYN
CIOIN   ------------SSSLYLLKPL--P---PPAQVTGSTNLLAHFGLESTYN
ANOGA   ------------SGPFYLMKEP--P---GEGELTGATNLMAHYGLEHSYS
TETNG   ------------NEPFYLLREL--P---VGNELTGNTNLITHYNLEHAYN
DROME   ------------TGPFYSMKEP--P---AKAELTGDKDLMTEYGLHHTLT
BRUMA   ------------VCPFHLMKPEL-P---QQSVLLGSNDLLAEYDMGSSYQ
CAEEL   ------------NEPFYTLKALL-P---PYSEIQGNHDLLMSYELGPVEG
YEAST*  ------------PSYYYYVDPETTY---TYQQPNPLQDLISVYGLDDISR
SCHPO   ------------APEYHYVGSVD-Y---QPTRPSAHQNLIELYGLTELAK
CANAL   ------------SNDYYLINPENIY---QSPKPTPKDNLIKLYGLIPVTK
CANGA   ------------PSYYYYVEPEMGY---QPQQPNPMEDLITVYHLDDISR
ASPNI   NWLSLDSLQKDFSSAFHLCKSA--H---IATGPDPSVDLISLYGLGPVAK
RHIO9   ---------MHFFFSFYSSMTAE-Q---KRNKLSGSQDLLTYFNLLPIYN
CRYNE   ----------------------------PVKPIHSSQDLISLLHLDTLYN
USTMA   ------------SLPPYLASTTSKPADLPRAYLNGTQDMLSLFGLMPIYD
ARATH*  ----------------------------GPRELCGAADLISQFKLVQHHE
VITVI   ------------------------------------------LQLVAHHE
SOYBN   ----------------------------GPRELTGAVDLISHFKLLPHYE
SOLLC   ----------------------------GPRELTGAVDLISHFKLLPHQE
ORYSJ*  ----------------------------GPRELTGAVDLISHYKLLAHHD
HORVU   ----------------------------GPRELTGAVDLISQYKLQPHHD
SORBI   ----------------------------GPRELSGAVDLISRYKLLNHHS
PHYPA   ------------DCFFRMLAIAP-----RPRELTGAVDLVSHYQLKEQFE
                 210       220       230       240       250
```

```
HUMAN*  KFCGK-------------------------KVKEKLSN--FLPDL--PGM
CHICK   KFCGK-------------------------KVKEKLSN--FLPDL--PGM
XENTR   KFCGK-------------------------KMKEKLSN--FLPDL--PGM
DANRE   KFCGK-------------------------KVKEKLSN--FLPEL--PGM
CIOIN   KFCGK-------------------------KLKETLSS--FLPHL--PGV
ANOGA   KFSGK-------------------------KVKEQLSS--FLPNL--PGV
TETNG   KFCGK-------------------------KVKEKLSN--FLPEL--PGM
DROME   KFKEK-------------------------KFKESLAS--FLQNI--PGI
BRUMA   RFCGS------------------------KRLREDLGS--FLPHL--VGS
CAEEL   GFSGS--R----------------------RVKEKISS--FLPHI--IGE
YEAST*  QVART--NLDGTKAV---------------KLRKSYKN--QIADL--SGK
SCHPO   KVGRV--DEFGNKR----------------KMRRSYKA--YIQDL--PGY
CANAL   SLART--NPDGSKGV---------------KLRKSYKN--HIQDL--PGK
CANGA   QVART--NEDGTKAV---------------KLRKSYKN--QITDL--SGK
ASPNI   SVARN--DPVTGEKI--------------NRLRKSYEG--KLKGLGLAGR
RHIO9   KVVKP--YPPTDRAS---------------GLNPTLFP--YISDL--PDG
CRYNE   TYVRPFADLPGDESQPGAGAGADGNDTLQRGKKKGASM---------PGR
USTMA   RAVRP--YNPDSIKAEDEAASTSVNVAAGKKVASAIAA---------EGN
ARATH*  FFCKK-------------------------SLPVSLSDSHYLHNV--VGD
VITVI   FFCKR-------------------------SLPLSISDTHYLHNV--VGD
SOYBN   FFCKR-------------------------SLPVSIADTHYLHNV--VGD
SOLLC   FFCKR-------------------------SLPLSISDTHYLHNV--VGD
ORYSJ*  FFCKK-------------------------PLPLAISDTHYLDNV--VGD
HORVU   FFCKR-------------------------PLPLAISDTHYLHNV--VGD
SORBI   FFCKK-------------------------PLPLAISDTNYLNNV--VGD
PHYPA   AFCKK-------------------------PSPTSVSDSMYLGNV--VGD
                 260       270       280       290       300
```

MED19

```
HUMAN*  IDLPGS-HDNSS----LRSLIEK--------------------PPILS-S
CHICK   IDLPGS-HDNSS----LRSLIEK--------------------PPICG-S
XENTR   IDTPGA-QDNSS----LRSLIEK--------------------PPICG-N
DANRE   IDSPGI-QDNSS----LRSLIEK--------------------PPVCN-N
CIOIN   TDGPAA-SNGSS----LRSVIDK--------------------PPIVGSK
ANOGA   IDGPGH-LDNSS----LRSVIEK--------------------PPIVG-K
TETNG   IDCPGT-QDGSS----LRSLIDK--------------------PPVCG-N
DROME   NDLITHPVENST----LRSVIEK--------------------PPIGG-K
BRUMA   FNFENA-LEFSS----LRMLVEK--------------------PPITG-K
CAEEL   FHLDAT-KEASS----LRALIEK--------------------PPI-H-K
YEAST*  FSTIPT-RENGKGGQIAHILFQNNPDMMIQPPQQGQNMSEQQWREQLRNR
SCHPO   NEILRD----NT----IKQWLTN-----------------PIREEV----
CANAL   HQISPA-KPIPPGL--LDPLIEQ------------------------HPD
CANGA   FSSIPN-RENRKGGEIAHILFQNNPDMMNQ-VHRSPDMTDEQWREALMNV
ASPNI   NKAVKH-DPATPGG--LRQMTMW--------------PEEEWQNQKVFGK
RHIO9   Y---------------MLGLIRD-------------------PQAVENGP
CRYNE   KKMEKG----------YQHLIEDCIDPTPLGYKNDNPSLLPLLNDVLYPS
USTMA   SAAGQAANTGSA--------------------------------------
ARATH*  TEIRKG--EGMQ----LDQLIES------------------ISQSRETNI
VITVI   TEIRKG--DGMQ----LDQLIQN------------------TSYSRDTNV
SOYBN   TEIRKG--DGMQ----LEQLIQN------------------TSSFRDTNA
SOLLC   TEIRKG--EGMQ----LDQLIQD------------------TSLSRETSS
ORYSJ*  TEIRKG--EGME----LDQLVQN-------------------AYLRDKPA
HORVU   TEIRKG--EGME----LDQLVQN-------------------AYLRDKPA
SORBI   TEIRKG--EGME----LDQLFQN-------------------SYSSEKTA
PHYPA   SEFRRG--EGME----LCQLVQG----------------SAGSWMPSDRV
                 310       320       330       340       350
```

```
HUMAN*  SFNPITG------TMLAGF-RLH---TGPLPE---------QCRLMHIQ-
CHICK   SFTPLTG------TMLTGF-RLH---AGPLPE---------QCRLMHIQ-
XENTR   SFTPLTG------ALLTGF-RLH---TGPLPE---------QCRLMHIQ-
DANRE   SFSPLTG------AMLTGF-RLH---TGPLPE---------QYRLMHIQ-
CIOIN   EIQPLSSG-----VLMSAF-RLH---PGPLPE---------QYRPLHQQ-
ANOGA   ELLPLTS------VQLAGF-RLH---PGPLPE---------QYKHLKTA-
TETNG   SFSPLTG------ALLTGF-RLH---TGPLPE---------QYRLMHIQ-
DROME   ELLPLTP------VQLAGF-RLH---PGPLPE---------QYRTTYVT-
BRUMA   EITSLSA------GAMAGF-RLT---PGAVPE---------PYRYFDNKV
CAEEL   EISNLTS------SSMQGF-KLS---AGPVDE---------RYRHLFERR
YEAST*  DIALFQPPNFDWDLCSSVLSQFERSYPSEFANQNQGGAQAPFDIDDLAFD
SCHPO   ---PIDIE-----FLHHVF-SVE---PGIIPG---------FNPKVFGLE
CANAL   IIKPLDPE-----LLNKAL-KFDRTPINGIPG---------FNSSDLAIN
CANGA   DASLFQAPNMDWEVCQSVLSQFDRSYPTEFQNDPN------FHVDDLAFD
ASPNI   EIKVADMDSALYNLQMKAM-KME---PGTVPN---------NDYWEDVLG
RHIO9   DIKPLDSE-----TLRDAF-SLR---EGPIPG---------FDASVLGTD
CRYNE   APPPTLSQEPIEALPKEAF-AIAKLEPGTVQD---------GYAGGQKAG
USTMA   -------------TIGATA-TAGSATQGATPS---------TSQPDLAQA
ARATH*  RIQPFDID-----ELQESF-QLNDMTPVELPP---------AEKGAPTIP
VITVI   RIQPFDLD-----VLREAF-QLRETAPIDLPP---------AEKGIPTIA
SOYBN   RIQPFDLD-----VLKEAF-QLRETAPIDLPA---------AEKGIPTIA
SOLLC   CIQPFDLD-----VLGEAF-RLREAAPVDLPP---------SEKGIPTVA
ORYSJ*  YIQPFDME-----TLGQAF-QLRETAPVDLPS---------AEKGIPTIS
HORVU   YIQPFDME-----TLGQAF-QLRETAPVDLPS---------AEKGIPTIS
SORBI   YIQPFDME-----TLGQAF-QLRETAPVDLPS---------AEKGTPTIS
PHYPA   PFQHFDIE-----LLRRAF-ALKETGPIFLPE---------SERGLPTIR
                 360       370       380       390       400
```

```
HUMAN*  ----PPKKKNKHKHKQSRTQDPVPPETPS--------DSD----------
CHICK   ----PPKKKNKHKHKQSRTQDPVPPETPS--------DSD----------
XENTR   ----PPKKKNK-KHKQSRTQEPAPPETPS--------DSD----------
DANRE   ----PPKKKNKHKHKHHRPQDPLPPETPS--------DSD----------
CIOIN   -----NESRRKPKHKKNRPDPVSLDQRGP--------ELE----------
ANOGA   -----PTRKHKNKHKKHKHKDGVAPPEQSA-LEAAGLDTH----------
TETNG   ----PPKKKSKHKHKHHRPQDPLPQETPS--------DTD----------
DROME   -----PARKHKNKHKKQKHKDGVTTGQESTLLDSAGLETY----------
BRUMA   DDLSSDTMNYDENGEETKHKRKYKWSLDD----LDDADLD----------
CAEEL   KE--EPNLAYSEKLNLIRVRQQYDAYGFD---DDETEKGF----------
YEAST*  LDGTGKSQSGSNSGN------------------------N----------
SCHPO   DDVVMGNVSRDSS--------------------------Q----------
CANAL   DQQTLMRGDDMGE------------------------DEH----------
CANGA   LDGTGKS--------------------------------N----------
ASPNI   HDKPTKNANAGEGAKKTHPSASAPRAVSQPNGTPVPAEPE----------
RHIO9   DATTTEGNYEGTQGEGEHDG-------------------E----------
CRYNE   VREAEEKRRRKRAARTTTADIISPINATFPGHSQGRSSTPGTPLLPVPPP
USTMA   NATFRPSAASGAA--------------------------G----------
ARATH*  SKSKSESKDRDRKHKKHKDRDKDKDR--E---------------------
VITVI   GKSKSESKDKERKHKKHKDRDKEKDK--E---------------------
SOYBN   GKSKGENKDKEKKHKKHKDKDKDKDRE---------------------
SOLLC   GKSKSESKDKEKKHKKHKDKDKEKDK--E---------------------
ORYSJ*  GKLKSESKDKEKKHKKHKDKDRDKDK--E---------------------
HORVU   GKPKSESKDKEKKHKKHKDK--DKDR--E---------------------
SORBI   GKSKVKSKDKVKKHKKHKEKDRDKEK--E---------------------
PHYPA   VKSKDQDEDKKKRKHKHRSKDREKAKD-----------------------
                 410       420       430       440       450
```

MED19

```
HUMAN*  --------------------------------------------------
CHICK   --------------------------------------------------
XENTR   --------------------------------------------------
DANRE   --------------------------------------------------
CIOIN   --------------------------------------------------
ANOGA   --------------------------------------------------
TETNG   --------------------------------------------------
DROME   --------------------------------------------------
BRUMA   --------------------------------------------------
CAEEL   --------------------------------------------------
YEAST*  --------------------------------------------------
SCHPO   --------------------------------------------------
CANAL   --------------------------------------------------
CANGA   --------------------------------------------------
ASPNI   --------------------------------------------------
RHIO9   --------------------------------------------------
CRYNE   TRGFPPGANTARRGTAPPGQGGSGGGSGIQGVHPFARHGPATGAGPGGRP
USTMA   --------------------------------------------------
ARATH*  --------------------------------------------------
VITVI   --------------------------------------------------
SOYBN   --------------------------------------------------
SOLLC   --------------------------------------------------
ORYSJ*  --------------------------------------------------
HORVU   --------------------------------------------------
SORBI   --------------------------------------------------
PHYPA   --------------------------------------------------
                 460       470       480       490       500
```

```
HUMAN*  -HKKKKKK-K----------------------------------------
CHICK   -HKKKKKK-K----------------------------------------
XENTR   -HRKKKKKQR----------------------------------------
DANRE   -HKKKKKK-K----------------------------------------
CIOIN   -SKKHKKRK-----------------------------------------
ANOGA   -EKKHKKQKRH---------------------------------------
TETNG   -PKKKKKK-R----------------------------------------
DROME   -EKKHKKQKRH---------------------------------------
BRUMA   -RKYRKHR------------------------------------------
CAEEL   -PKKHKKK------------------------------------------
YEAST*  -SKKRKNKSSGS--------------------------------------
SCHPO   -PRSPSRRKK----------------------------------------
CANAL   -GKKIKRKKKIQ--------------------------------------
CANGA   -AKKRKNRSNGS--------------------------------------
ASPNI   -RSRPSRGRKRHYDDNSFVGYGE---------------------------
RHIO9   -RRHKKKKKKRH--------------------------------------
CRYNE   FTQSSKQRPASMEVQGSGGGRKGLPNRSASPMPLSAVASPGGPGGGGGKG
USTMA   -AATTGLASHPSHGASSTGGRISTPALQLNGGPPMFPLHTDSSAILAASP
ARATH*  -HKKHKHKHKDR--------------------------------------
VITVI   -HKKHKHRH-NR--------------------------------------
SOYBN   -HKKHKHRHKDR--------------------------------------
SOLLC   -HKKHKHRHKDR--------------------------------------
ORYSJ*  -HKKHKHRHKDR--------------------------------------
HORVU   -HKKHKHRHKDR--------------------------------------
SORBI   -QKKHKHRHKDR--------------------------------------
PHYPA   -KEKKKDRDKDK--------------------------------------
                 510       520       530       540       550
```

```
HUMAN*  -------EEDPERKRKK-------------------KEKKKKKNRHS-PD
CHICK   -------EEDPERKRKK-------------------KEKKKKKNRHS-PE
XENTR   -------EDDPERRRKK-------------------KDKKKKKSRHS-PE
DANRE   -------DDDPDRKKKK-------------------KDKKKKKNRHS-PD
CIOIN   -------HEDGDKKKRK-------------------KEKKRKREREKERM
ANOGA   -------EDDKERKKRK-------------------KEKKRKKQRHS-PE
TETNG   -------DDDPDRKKKK-------------------KDKKKKKVRPAIKT
DROME   -------EDDKERKKRK-------------------KEKKRKKKNQS-PE
BRUMA   -------SEDKDRKKEK-------------------KKKKDKKRKRDSKE
CAEEL   -------KKDKKRKKEK-------------------EELGDAEKRKRAAD
YEAST*  -------SMATPTHSDS--------------------HEDMKRRRLE---
SCHPO   --------------------------------------------------
CANAL   -------NGADPKRQHI---------------------------------
CANGA   -------SMATP-NSEM--------------------QDDVKRRRLE---
ASPNI   ----GYADDDDDAAIYS-------------NGEGGGKKKRKKDHVPRIPA
RHIO9   -------SHDEEGHIHS-------------------EHKKKKKKKREDFV
CRYNE   GGTLPSASVSASKSGQRQFPPNRNKRPGSADVQSAQMKKSKGGSRSASPM
USTMA   GQLAAAASAAPPARKPSKPPGTYAHYVDDLAGRCTCGQRGQRNHVGLDPV
ARATH*  -------SKDKDKDKDRDR--KKDKNGHHDSG-DHSKKHHDKKRKHDGDE
VITVI   -------SKEKDKE-------KKDRSGQHDSAADHSKKHHEKKRKHDGEE
SOYBN   -------TKDKDKDRDK----KKDKSGHRDSSADHSKKHHEKKRKHDGDD
SOLLC   -------SKDKDKDK------KRDKSVHHDSGADLSKKHHEKKRKHDGEE
ORYSJ*  -------SKDKDKDKDKDK--KKDKSGHHDSGGDHSKKHHEKKRKHEGME
HORVU   -------SKEKDKDKDKKK-----------------DKHHEKKRKHEGTE
SORBI   -------SKDKDKDKDKDKKEKKKDKSGNHDSGGDHSKK-HEKKRKQEVTG
PHYPA   -------KRDKDKDKDR------------SKGENGEKKHKKKKRRHDGEE
                 560       570       580       590       600
```

MED19

```
HUMAN*   HPGMGSSQASSSSSLR---------------------------------
CHICK    HPGVGSSQASSSSSLR---------------------------------
XENTR    HPGAGSSQSGLR--------------------------------------
DANRE    HPGMTGAQPSTSS-LR----------------------------------
CIOIN    --------------------------------------------------
ANOGA    HPAGGGGGTASVPPQTQVY-------------------------------
TETNG    KPVMFLKA------------------------------------------
DROME    PGVGLLPGGASLGGAAGVMGATSLGSLGGGPGGPGLSSLSASMGPGVVGN
BRUMA    EDQNNKVRKV----------------------------------------
CAEEL    EPMEF---------------------------------------------
YEAST*   --------------------------------------------------
SCHPO    --------------------------------------------------
CANAL    --------------------------------------------------
CANGA    --------------------------------------------------
ASPNI    PSDRGGSYGVGMFGIGAR--------------------------------
RHIO9    DI------------------------------------------------
CRYNE    PGAGVGQGQLRR--------------------------------------
USTMA    QA------------------------------------------------
ARATH*   DLNDVQRHKKN-KHKSSKLDEV----------------------------
VITVI    DVNDIQRHKKS-KHKSSKIDEL----------------------------
SOYBN    DVNDVHKHKKS-KHKSSKIDEL----------------------------
SOLLC    EFNDVHKHKRS-KHKSSKIDDI----------------------------
ORYSJ*   DSADVHKHKKS-KHKSSRTDDT----------------------------
HORVU    DSADVHKHKKS-KHKSSKTDEM----------------------------
SORBI    SSANVQNHKKTQKHKNQ---------------------------------
PHYPA    GDDGH-------KHKSKKVFSL----------------------------
                  610       620       630       640       650
```

```
HUMAN*   --------------------------------------------------
CHICK    --------------------------------------------------
XENTR    --------------------------------------------------
DANRE    --------------------------------------------------
CIOIN    --------------------------------------------------
ANOGA    --------------------------------------------------
TETNG    --------------------------------------------------
DROME    MNSFSSSMQMQQQQQMPMQQQQMSSGGLLGSVLGTSGGPGGGGGGGGGGG
BRUMA    --------------------------------------------------
CAEEL    --------------------------------------------------
YEAST*   --------------------------------------------------
SCHPO    --------------------------------------------------
CANAL    --------------------------------------------------
CANGA    --------------------------------------------------
ASPNI    --------------------------------------------------
RHIO9    --------------------------------------------------
CRYNE    --------------------------------------------------
USTMA    --------------------------------------------------
ARATH*   ------------------------------------------------GA
VITVI    ------------------------------------------------GA
SOYBN    ------------------------------------------------GA
SOLLC    ------------------------------------------------GA
ORYSJ*   ------------------------------------------------GN
HORVU    ------------------------------------------------GN
SORBI    --------------------------------------------------
PHYPA    ------------------------------------------------GF
                  660       670       680       690       700
```

```
HUMAN*   ------------
CHICK    ------------
XENTR    ------------
DANRE    ------------
CIOIN    ------------
ANOGA    ------------
TETNG    ------------
DROME    GGVGGSLLMSQF
BRUMA    ------------
CAEEL    ------------
YEAST*   ------------
SCHPO    ------------
CANAL    ------------
CANGA    ------------
ASPNI    ------------
RHIO9    ------------
CRYNE    ------------
USTMA    ------------
ARATH*   IRVAG-------
VITVI    IKVAG-------
SOYBN    IKVAG-------
SOLLC    IKLL--------
ORYSJ*   GLS---------
HORVU    GLS---------
SORBI    ------------
PHYPA    AESCTELTRGSS
                  710
```

MED20

```
HUMAN*   MG-------------------VTCVSQMPVAEGKSV-QQTVELL-TRKLE
CHICK    MG-------------------VTCVTQVPVLEGKSV-QQTVELL-SKKLE
XENTR    ------------------------SQVPLAEGKSV-QQTVEIL-TCKLE
DANRE    MG-------------------VTCVCQVPVAEGKSV-QQTVDLL-HRKLE
CIOIN    MG-------------------VAYVSPWKVPAGQNV-QQNIEEL-HNSIE
ANOGA    MG-------------------VTVLQPYPLE--KSG-AQTIDFL-LKRVV
TETNG    MG-------------------VTCVCQVPVAEGKSV-QQTVDAL-HRRLE
DROME    MG-------------------VTILQPYPLPEGKSG-AHIIDQL-SKRLL
BRUMA    MG-------------------VAWVFET---------DKSAVSI-ERAIE
CAEEL    MG-------------------VTWVFEA---------EQTAKSV-ERLLE
YEAST*   MG-------------------KSAVIFVERATPATL-TELKDAL-SNSIL
SCHPO    MP-------------------VHGVIYYSSPSMATFLSPAQDNL----VR
CANAL    M--------------------VTAVLLVQKANLETI-TQFHDQI-SNELP
CANGA    MS--------------------SAVIFVESATPGTL-TEFKDLL-AKQLL
ASPNI    MS-------------------LTGVFFIPSNPNAATALATVTER-LTALF
RHIO9    MG-------------------ITCLVRWKNATGMRDFTFIAEHV----TK
CRYNE    MG-------------------FTLLLQWSCTPPAGRAVSLADVVCARVLE
USTMA    MG-------------------VSSTARLPSLGAQSI---------PPLFS
ENCCU    ME----------------------------------------GV-LHKIN
ANTLO    ----------------------------------------MDEV-IVNLN
ARATH*   MP-------------------VKWVLHWQPNQGSTVSSQILNEA-TQCVE
VITVI    MSFSHSQSTQLLSRLAQLXCRLSWVFHWQPNAGTTVNSQMINEV-SQCVE
SOYBN    MP-------------------IRCILHWQPNQGSVVNSQVLNEI-SQCVE
SOLLC    MS-------------------IKWVLCWQPDAGTTINNQILIEV-SKCVE
ORYSJ*   MP------------------AVKWLMHWHPNQGATLNSQILAEA-CACAE
HORVU    MA------------------PAKWLMHWHPNPGVTLNTQILSEA-CGCAE
SORBI    MP------------------PVKWLMHWHPSPGATINSQILSEA-CGCAE
SELML    MG-------------SQSVCLDSRVLHWQPSAGAQVTSQTLVDL-FRSIE
PHYPA    MT-------------------VKWLYYWQPSIGVTMSSQTLSIA-IKRIE
VOLCA    MG-------------------VKLLIRPVPPKGVTG-PTSYDLL-KRLID
                  10        20        30        40        50
```

```
HUMAN*   ML----GAEKQGTFCVDCETYHTAAS------------------------
CHICK    LL----GAEKHGAFGVDCETYHTAAA------------------------
XENTR    QL----GAEKQGTFYVDCETYHTAGA------------------------
DANRE    QL----GAVKQGNFWVDCETYHATGN------------------------
CIOIN    QL----GAIKTGLFTVECESYHAVNV------------------------
ANOGA    AL----GAVPAGQFLVDCETYSSNPQ------------------------
TETNG    QL----GAVKQGSFCVDSETYAATAS------------------------
DROME    AL----GATHAGQFLVDCETFISTPQ------------------------
BRUMA    GE----GGVKGGIFTVDSTPFKPSDN------------------------
CAEEL    SI----GGDLHGTFIVDVTPFNPPTP------------------------
YEAST*   SV--------RDPWSIDFRTYRCSIK------------------------
SCHPO    TY----FAQHLKKWVVQYKLYRNAVT------------------------
CANAL    TA--------KGKWNFNFKIFRNNQY-SIP--------------------
CANGA    EV--------RETWSLEFRTYRTLVK------------------------
ASPNI    AD----EATPVSRWALEHKLMRDTPS------------------------
RHIO9    SL----QGKNTGPWSLSFKVFRDINN------------------------
CRYNE    SY----GHNPPSRLGLQVRTYRAQFPLSPT--------------------
USTMA    HLQHTSNAKVVARWSITIRSFRAIPVPTNAWPPANEFDSSFSPIPSQAAS
ENCCU    GL------------------------------------------------
ANTLO    AI------------------------------------------------
ARATH*   SI----NGVKEGRWKATLNYYKPMLK-DQA--------------------
VITVI    SI----NGVKEGRWKATLTFYKPMFRADQA--------------------
SOYBN    SL----NGVKEGRCKASLTFYRPNLR-DQS--------------------
SOLLC    K---------------------------QA--------------------
ORYSJ*   SL----GGSKDGRWKTSIIFYRAMAR-DGA--------------------
HORVU    SL----GGTKDGRWKTSIIFYRPMTR-DGA--------------------
SORBI    SL----GGSKDGKWKTSIFFYRAMTR-DGS--------------------
SELML    SM----NGVRTGRWQITAAQHRPFTR-DQA--------------------
PHYPA    AL----HGVKTSRWQITASQFRPNQR-EPV--------------------
VOLCA    ELCVSQPSGRSEAWSVTCQLMKQ----GHG--------------------
                  60        70        80        90       100
```

MED20

```
HUMAN*  --------------------------------TLGSQGQTGKLMYVMHNS
CHICK   ---------------------------------ISSQGQTGKLMYVMHNS
XENTR   --------------------------------SMGTPGQAGKLLYVMHNS
DANRE   -----------------------------------ATGQPSKLLYVMHNS
CIOIN   --------------------------------------ANSKTLHLIHST
ANOGA   -------------------------------------LGPPKTVHILHNS
TETNG   -----------------------------------VGGQPSKLLYVMHNT
DROME   ----------------------------------PHNGAPGRAVHVLHNS
BRUMA   ------------------------------------VQGAINHVFILHHS
CAEEL   ------------------------------------SSDYPSNV-VMHHS
YEAST*  ----------------------------------NLPADVSKLMYSITFH
SCHPO   ----------------------------------------PKTLEFLKQN
CANAL   ----------------------------QELVDTHEQAPESKFLFTLSPS
CANGA   -----------------------------------DFPSREKLLYSLTFP
ASPNI   ----------------------------CLPNSGTQRPGAPKYMQFLSLT
RHIO9   --------------------------------NKAVSLKDSKLLYQVSLG
CRYNE   ----------------------------TDDNNQDNNNQVSRYLTIISNL
USTMA   VTAGQNTTSSSGSSAAAQYSGPTSSGGAGSSASGAPPLTKPRTMWQVQLS
ENCCU   -----------------------------------------------LKN
ANTLO   -----------------------------------------------LVN
ARATH*  -----------------------------------NQLEFPRDFLGISLA
VITVI   -----------------------------------VPAEYPRDFLGISLP
SOYBN   -----------------------------------VTIDFPRDFLGISML
SOLLC   -----------------------------------NASEFPQHFLGASLQ
ORYSJ*  -----------------------SAAAAGGGGPPQQHPDVPRELLGVALH
HORVU   -----------------------------GGAQQNQLADVPRELLGVALH
SORBI   -----------------------------GGPAGQQHPDLPRELLGVALH
SELML   -----------------------------------MAIDCAKELLGVAFS
PHYPA   -----------------------------------PLVECARELLGVVFS
VOLCA   -----------------------LVGTVGGPTAGAGGGGGLRDTWAVTFR
                 110       120       130       140       150
```

```
HUMAN*  EYP---------------LSCFALFENGP---------------------
CHICK   EYP---------------LSCFALFENGP---------------------
XENTR   EYP---------------LSTFALFENGP---------------------
DANRE   EAP---------------LSCMALFEGGP---------------------
CIOIN   EYP---------------ASRFACFEDGP---------------------
ANOGA   EQP---------------ASVFSILDTGT---------------------
TETNG   ETP---------------LSCFALFEGGP---------------------
DROME   EYP---------------ASTFSIIDNGT---------------------
BRUMA   RFP---------------QSTFTIAPNEKQ--------------------
CAEEL   KCP---------------QSTFSICPKDTF--------------------
YEAST*  HHG---------------RQTVLIKDNSAM--------------VTTAAA
SCHPO   INP----------------SMLACVDEAT---------------------
CANAL   YLR---------------DSTITLINGKSAGVFTNSIEE-ELSELGHPSD
CANGA   HHD---------------KKTVLIRNSLAW----------------VLGT
ASPNI   YHP---------------NHGFIYTSQQADKDGPGAVAAPTPSSSQSSQA
RHIO9   QQP---------------GNVYCMVDGSV---------------------
CRYNE   MPPAQPGEAKTNAPIIKDDTAYLFLDDRSS------------------TA
USTMA   DHP---------------GVLFVIVEDSGRSSRAKVWRDWELSTKQWKKQ
ENCCU   PRL---------------EDILAVFEIDGY--------------------
ANTLO   PTS---------------ESILSLFEPEAH--------------------
ARATH*  DQP---------------NKYYFIIRTQRI--------------------
VITVI   EQP---------------NRYYFIIRSQKI--------------------
SOYBN   EQP---------------NKYYFIIRGQKI--------------------
SOLLC   EQP---------------DKFYMALSKKRL--------------------
ORYSJ*  ERP---------------GLYFSILRAHRL--------------------
HORVU   ERP---------------GLYFSILRNQRL--------------------
SORBI   ERP---------------GLYFSIVRAQRL--------------------
SELML   EFA---------------SKHYFVLRAERM--------------------
PHYPA   EVP---------------DKYYFALRQEHM--------------------
VOLCA   DAP---------------DVVFLLQRPECK--------------------
                 160       170       180       190       200
```

MED20

```
HUMAN*   -------------CLIADTNFDVLMVKLKGFFQSAKASKIET-RGTR---
CHICK    -------------CLIADANFDTLMVKLKGFFQNAKANKIES-RGTR---
XENTR    -------------CLVADLNLDVLMVKLKGFFQNAKANKMET-RGTR---
DANRE    -------------GLSADANFDVLMIKLKSHFQNAKGHKVES-RGTR---
CIOIN    -------------SIVSDVMLPSFLSKLRSVWQPRKGGRIES-KGHR---
ANOGA    ----------KQIPLVTDGLFDLLMTRIAPAYTSKKQTKIES-KGQR---
TETNG    -------------CLKADTNFDILMVKLKSHFQNAKGHKVEC-RGSR---
DROME    ---------GKQVAIVADNIFDLLMLKMTNTFTSKKQTKIES-RGAR---
BRUMA    ---------KHCPRAISDRGFDCILAKLSSGLVQDTAGKFEV-SGHE---
CAEEL    ---------KKSPKAVCDRGFDLILSKLSSGLIADNAGKIEI-IGNE---
YEAST*   ADIPPALVFNGSSTGVPESIDTILSSKLSNIW--MQRQLIKG-DAGETLI
SCHPO    -------------MIDAEPELEDIIVRTK-LW--NFRQSFTI-EGSI---
CANAL    LSIPNDHLRRGATTGLNDKFDNFIGAKLQSLW--TQRQLIKG-DGGQIYE
CANGA    AEIPDNL--QTCSTGLSESIDQLLASKLSNMW--AQRQVIRG-DAGQTLL
ASPNI    TSVTTPMVMTTVPLPSTGALYQHFSYACQPFW--CHRHTVTVPNGVV---
RHIO9    -------------VVEAGKEMEIILSRLKNLW--QLRQNVTI-EGTS---
CRYNE    TGGESEHVNRATETSTAAHSSDPIPLQMDE-------------EGKR---
USTMA    KRRENAARREKEQKAVRDKAQLELNAAVHSTTAAADLSEIKSNEQTEAQD
ENCCU    --------IRLNRVYISPFSLRQLLIKVAGLD--LVSWQMTP-SGLF---
ANTLO    --------IRFNNIFISPITLIDFKAKLDSFG--LVSWEVIE-DGIL---
ARATH*   -------------VLEADSSIQLIMEKLQS-Y--KSKVALYF-DGFQ---
VITVI    -------------VLEADSSIQMIMEKLQS-Y--KSRVSLTF-EGFQ---
SOYBN    -------------VLEADSSILLIMEKLQS-Y--KSKVALHF-EGVL---
SOLLC    -------------IVEAESSMQTIMENLQS-Y--RIKFALNC-EGFQ---
ORYSJ*   -------------VLQADSAFPQVMEKLQS-Y--KARVTLNF-EGFQ---
HORVU    -------------VLQADAAFSQVMEKLQS-F--KARVNLNF-EGFQ---
SORBI    -------------VLQADAAFPQVMEKLQS-Y--RIRVALNF-EGFQ---
SELML    -------------VVEADSSIQAIMEKLQV-Y--RNRLSMVF-EGFQ---
PHYPA    -------------VVEADATMQAIMEKLQV-Y--RNRLTILF-EGFQ---
VOLCA    -------------VLECESAVMRLLEKKLE-F--AKQMAVRV-EGHT---
                  210       220       230       240       250
```

```
HUMAN*   YQY-----------------------------------------------
CHICK    YQY-----------------------------------------------
XENTR    YQY-----------------------------------------------
DANRE    YRY-----------------------------------------------
CIOIN    YEL-----------------------------------------------
ANOGA    FEF-----------------------------------------------
TETNG    YRY-----------------------------------------------
DROME    FEY-----------------------------------------------
BRUMA    YTL-----------------------------------------------
CAEEL    YSLY----------------------------------------------
YEAST*   L-------------------------------------------------
SCHPO    YEV-----------------------------------------------
CANAL    LEN-----------------------------------------------
CANGA    IT------------------------------------------------
ASPNI    YEI-----------------------------------------------
RHIO9    YEI-----------------------------------------------
CRYNE    FRCVAVRPSTAVIPMLQSLL------------------------------
USTMA    HKHVAVKESAEPMDEDAPTSLITDGDAKTDSEFTFSQNTAGSQQVEAGIP
ENCCU    FQF-----------------------------------------------
ANTLO    FQF-----------------------------------------------
ARATH*   YQL-----------------------------------------------
VITVI    YQL-----------------------------------------------
SOYBN    YKL-----------------------------------------------
SOLLC    YRL-----------------------------------------------
ORYSJ*   YQL-----------------------------------------------
HORVU    YQL-----------------------------------------------
SORBI    YQL-----------------------------------------------
SELML    YQL-----------------------------------------------
PHYPA    YEL-----------------------------------------------
VOLCA    YTK-----------------------------------------------
                  260       270       280       290       300
```

MED20

```
HUMAN*   --------------------------------------------------
CHICK    --------------------------------------------------
XENTR    --------------------------------------------------
DANRE    --------------------------------------------------
CIOIN    --------------------------------------------------
ANOGA    --------------------------------------------------
TETNG    --------------------------------------------------
DROME    --------------------------------------------------
BRUMA    --------------------------------------------------
CAEEL    --------------------------------------------------
YEAST*   --------------------------------------------------
SCHPO    --------------------------------------------------
CANAL    --------------------------------------------------
CANGA    --------------------------------------------------
ASPNI    --------------------------------------------------
RHIO9    --------------------------------------------------
CRYNE    --------------------------------------------------
USTMA    ALDTTVSMEQAITEPAGPRPKLQLPSHTRYTASAITSSMSAMLSSLNLPP
ENCCU    --------------------------------------------------
ANTLO    --------------------------------------------------
ARATH*   --------------------------------------------------
VITVI    --------------------------------------------------
SOYBN    --------------------------------------------------
SOLLC    --------------------------------------------------
ORYSJ*   --------------------------------------------------
HORVU    --------------------------------------------------
SORBI    --------------------------------------------------
SELML    --------------------------------------------------
PHYPA    --------------------------------------------------
VOLCA    --------------------------------------------------
                  310       320       330       340       350
```

```
HUMAN*   --------------------------------------------------
CHICK    --------------------------------------------------
XENTR    --------------------------------------------------
DANRE    --------------------------------------------------
CIOIN    --------------------------------------------------
ANOGA    --------------------------------------------------
TETNG    --------------------------------------------------
DROME    --------------------------------------------------
BRUMA    --------------------------------------------------
CAEEL    --------------------------------------------------
YEAST*   --------------------------------------------------
SCHPO    --------------------------------------------------
CANAL    --------------------------------------------------
CANGA    --------------------------------------------------
ASPNI    --------------------------------------------------
RHIO9    --------------------------------------------------
CRYNE    --------------------------------------------------
USTMA    PHGAPVGTAGPGAWVPRGAAVSIEGLVLEINSQSLNALPGISPSLASLST
ENCCU    --------------------------------------------------
ANTLO    --------------------------------------------------
ARATH*   --------------------------------------------------
VITVI    --------------------------------------------------
SOYBN    --------------------------------------------------
SOLLC    --------------------------------------------------
ORYSJ*   --------------------------------------------------
HORVU    --------------------------------------------------
SORBI    --------------------------------------------------
SELML    --------------------------------------------------
PHYPA    --------------------------------------------------
VOLCA    --------------------------------------------------
                  360       370       380       390       400
```

MED20

```
HUMAN*  ----------------------CDFLVKVGTVT----M------------
CHICK   ----------------------CDFLVKVGTVT----M------------
XENTR   ----------------------CDFLVKIGTVT----V------------
DANRE   ----------------------CDFLVKIGTVA----M------------
CIOIN   ----------------------GDFVLKTGIIS----M------------
ANOGA   ----------------------GDFVIKLGSVT----M------------
TETNG   ----------------------CDFLIKVGTVT----M------------
DROME   ----------------------GDFVIKLGSVT----M------------
BRUMA   ----------------------QDFIIRTGNAS----M------------
CAEEL   ----------------------KDWMIRVGTAT----Q------------
YEAST*  ----------------------DGLTVRLVNLF----S------------
SCHPO   ----------------------GSFKVAIANVL----Q------------
CANAL   ----------------------GNLCIRTSNVF----L------------
CANGA   ----------------------GDVTVRIINLF----A------------
ASPNI   ----------------------GDFRVRAGDVRQTQ-P------------
RHIO9   ----------------------GDFTLRVANIL----L------------
CRYNE   ----------------------SPFVMGLTKAARTTASQTSSTPVPTSLP
USTMA   TEDAALITSSTEAGAGGAGRGGADWRVRVGSVVGG---------------
ENCCU   ----------------------RTFCLKFYGSVN---I------------
ANTLO   ----------------------ERTFIHFQGFYE---I------------
ARATH*  ----------------------GDFRLRVGKVVPT--H------------
VITVI   ----------------------GDFQLRVGKVAPS--A------------
SOYBN   ----------------------GDFQMRVIKVVPS--Q------------
SOLLC   ----------------------GDFRVRVGKVVPM--N------------
ORYSJ*  ----------------------GDFCLRIGKCVPN--N------------
HORVU   ----------------------GDFCLRIGKCVPN--N------------
SORBI   ----------------------GDFCLRIGKCVPN--N------------
SELML   ----------------------GDFQLKAGRAVLS--Q------------
PHYPA   ----------------------GDFHIKAGRAVLA--H------------
VOLCA   ----------------------GDFVLRLCTATQAIHS------------
                 410       420       430       440       450
```

```
HUMAN*  -----------------------------GPSARGISVEVEYGPC----V
CHICK   -----------------------------GPSARGISVEVEYCPC----V
XENTR   -----------------------------GQSGRGITVEVDYCPC----A
DANRE   -----------------------------SSSARGISVEVEYCAC----V
CIOIN   -----------------------------GPSTKGISLEVEYTPC----V
ANOGA   -----------------------------SQNFKGVLVEVEYRPC----L
TETNG   -----------------------------NSSARGISVEVEYCPC----V
DROME   -----------------------------MEHFKGILIEIEYKSC----V
BRUMA   -----------------------------GVISKGVIVEVEYAPS----C
CAEEL   -----------------------------GTTVKGVVVEIEYDPS----I
YEAST*  -----------------------------STGFKGLLIELQADEAGEFET
SCHPO   -----------------------------KSVWKGILFHVTYDGT----E
CANAL   -----------------------------HGNFRGLLIQIEMSNS----L
CANGA   -----------------------------ATGFKGLLIELDNLQSA---T
ASPNI   -----------------------------AVRMRGTVVEIEWQGP----S
RHIO9   -----------------------------GSTYKGLLLEVDYHPC----S
CRYNE   GSTLLMTVLTFNALLSPQPPVILRLYILPNQSASSIFLEGEYAGS----V
USTMA   -----------------------------GGRSAGAIIEAEFLPA----S
ENCCU   -----------------------------VVKQKVFCIEIDFDPS----I
ANTLO   -----------------------------FNRMKLFSFELNFEPV----T
ARATH*  -----------------------------SENVRGIVMEVEYLPI----S
VITVI   -----------------------------SENLRGIVFEVEYLPL----S
SOYBN   -----------------------------AESLRGIMIEIEYLPI----S
SOLLC   -----------------------------SENLRGIVMEMEYLPI----Y
ORYSJ*  -----------------------------SETLRGIMMEVEYYPL----S
HORVU   -----------------------------TETLRGIMMEVEYYPL----S
SORBI   -----------------------------SEALRGIMMEVEYYPL----S
SELML   -----------------------------AENLRGIVLEVEYSPV----S
PHYPA   -----------------------------TENLRGIVLEVDYKPL----S
VOLCA   -----------------------------TQALLGYCLEVEYLPV----S
                 460       470       480       490       500
```

MED20

```
HUMAN*   VASDCWSLLLEF-LQSFLG-------------------------------
CHICK    IANDCWNLLMEF-MQSFMG-------------------------------
XENTR    VPGDCWNLMVEF-MQSFMG-------------------------------
DANRE    IPGDCWNLMKEF-MQSFLG-------------------------------
CIOIN    YPCDCWGIITEL-LKGFVG-------------------------------
ANOGA    VPGSCWELMREF-LQGFLG-------------------------------
TETNG    VPGDCWNLMKEF-MQSFLS-------------------------------
DROME    ILAYCWEMIREM-LQGFLG-------------------------------
BRUMA    VASQCGNFLQEF-VAVFFP-------------------------------
CAEEL    IVIQCKDMMIEF-VKSVFN-------------------------------
YEAST*   KIAGIEGHLAEIRAKEYKT-------------------------------
SCHPO    SVDLARPIIQEFFLKCFLQ-------------------------------
CANAL    CDTNNHNSFKEH-FNKIVE-------------------------------
CANGA    SLANITDLLNEMKVKVFKV-------------------------------
ASPNI    LATTLSGLYRSR-RQKRRV-------------------------------
RHIO9    TPNNASELFKEF-VQSIVP-------------------------------
CRYNE    DGKSEEEIEGEV-KKYLES-------------------------------
USTMA    TLLPTSKFMHDF-LFSLFPPGFIPVPPPAASVPAAGIPNVASLHGSAAAS
ENCCU    DGGTGVPVLKEL-ERYLKG-------------------------------
ANTLO    STEEGRLLMNEI-EIFLRK-------------------------------
ARATH*   SMEKAQKVMEEF-LEIWNE-------------------------------
VITVI    SMEKSRQILDGF-VEIWQE-------------------------------
SOYBN    SVEKSKQILEEF-IDIWKE-------------------------------
SOLLC    SCKTSHLIMSEF-FEILKE-------------------------------
ORYSJ*   SIEKSRAVMEDF-FDIWQE-------------------------------
HORVU    SIEKSRAIMVDF-FDIWQE-------------------------------
SORBI    SIEKSKAVMEDF-FDIWQE-------------------------------
SELML    SIEKTRQMMQEF-VDLWQE-------------------------------
PHYPA    SVEQSRRVVQDF-MEVWQK-------------------------------
VOLCA    SMSSAEGMLGEL-LDLMKQ-------------------------------
                  510       520       530       540       550
```

```
HUMAN*   --------------------------SHTPGAPAVFG-------------
CHICK    --------------------------SHTPGIPSVFG-------------
XENTR    --------------------------SHAPSVPPVFS-------------
DANRE    --------------------------SNTPELPTVFT-------------
CIOIN    --------------------EN-----MATNMPTLIL-------------
ANOGA    --------------------SN-----VSNSMPAYFT-------------
TETNG    --------------------------SSVPELPSVFA-------------
DROME    --------------------IA-----VNKDFPSYFA-------------
BRUMA    --------------------------DHVADKPAVLQ-------------
CAEEL    --------------------------KYHETLPEIFK-------------
YEAST*   --------------------------SSDSLGPDTSN-------------
SCHPO    ---------------------------NNKSVTPVYE-------------
CANAL    ----------------------------KYGFPEGNLCCDVLDKKNLDKY
CANGA    --------------------------ASAPGLKSEDV-------------
ASPNI    --------------------ASNTYMWDNEDGEDHDSGIDMAFPDGVEEA
RHIO9    ---------------------------PTAQLSCEYE-------------
CRYNE    --------------------------------------------------
USTMA    QTGSAVGTPRINNATLGTIPTSNGNALSTSAVTSGSGFSYTIGGSAGASA
ENCCU    --------------------CDALFFMTYFADIDKEI-------------
ANTLO    --------------------CDALFFLCYTQNIDAVI-------------
ARATH*   --------------------AL-----AKRSLPGKFV-------------
VITVI    --------------------AM-----SKRSLPGHFM-------------
SOYBN    --------------------VV-----SKKSLAGQFM-------------
SOLLC    --------------------TL-----GKKSLPGHFV-------------
ORYSJ*   --------------------TV-----AKKSLPGHFI-------------
HORVU    --------------------TL-----AKKSLPGRFI-------------
SORBI    --------------------TI-----GKKSLPGQFI-------------
SELML    --------------------AI-----SAQSMSGRIS-------------
PHYPA    -----------------------------GETTGQFV-------------
VOLCA    --------------------LMNTIVAAAAAANTTTT-------------
                  560       570       580       590       600
```

MED20

```
HUMAN*  --------------------------------------------------
CHICK   --------------------------------------------------
XENTR   --------------------------------------------------
DANRE   --------------------------------------------------
CIOIN   --------------------------------------------------
ANOGA   -------------------------------------------------Q
TETNG   --------------------------------------------------
DROME   -------------------------------------------------P
BRUMA   --------------------------------------------------
CAEEL   --------------------------------------------------
YEAST*  --------------------------------------------------
SCHPO   --------------------------------------------------
CANAL   G-------------------------------------------------
CANGA   --------------------------------------------------
ASPNI   DIDAEYAATAALIKEFWGKLEIEGAREAILVPDVGKEVKERLRKLKQPDK
RHIO9   --------------------------------------------------
CRYNE   --------------------------------------------------
USTMA   APNRHLNIPVVSDQLWEEVVPQSGEGWRRRVVKRSRQMTLTARAQRRQRK
ENCCU   --------------------------------------------------
ANTLO   --------------------------------------------------
ARATH*  --------------------------------------------------
VITVI   --------------------------------------------------
SOYBN   --------------------------------------------------
SOLLC   --------------------------------------------------
ORYSJ*  --------------------------------------------------
HORVU   --------------------------------------------------
SORBI   --------------------------------------------------
SELML   --------------------------------------------------
PHYPA   --------------------------------------------------
VOLCA   -------------------------------------------------Q
                           610                 620                 630                 640                 650
```

```
HUMAN*  -------------NRHDAVYGPA---DT----------------------
CHICK   -------------TKHDSIYSPA---DT----------------------
XENTR   -------------AKHDPLYTPA---DT----------------------
DANRE   -------------NKAEGLYVPVDCMDT----------------------
CIOIN   -------------EKSKEIYTPD---IT----------------------
ANOGA   RSSINPN-----HSKANEIYQPI---DT----------------------
TETNG   -------------TRPEGLFAPADAIDT----------------------
DROME   QTIMTAMGQQQLHAKHNDIFEPM---DT----------------------
BRUMA   -------------KAQPEPYSAL---DT----------------------
CAEEL   ------------ITEKPESYTAL---DT----------------------
YEAST*  ----EICDL-----------------------------------------
SCHPO   ----------SFFNQPRHSLDSKLLLQL----------------------
CANAL   --------------------------DL----------------------
CANGA   ----EVVDSSSTMESGNEALFTL---------------------------
ASPNI   QGVSHVVNEDGSGLQVDEDPDPDAGVDV----------------------
RHIO9   -------YNYEAVGLSNHEFTTA---HT----------------------
CRYNE   --------------------------------------------------
USTMA   EGAKPSTAHNGRVAQDNSGSTDVFGWHTFGSDDEEDASTTAHMNGDWQDQ
ENCCU   ------------------LHNPL---SE----------------------
ANTLO   ------------------LHNPL---SN----------------------
ARATH*  ----NIDLNFGEFGLGDI-YTPQ---HT----------------------
VITVI   ----HIEPNFAEFGLSDH-YTSQ---HT----------------------
SOYBN   ----HTEPNYAEYGLSDN-YTSQ---HT----------------------
SOLLC   ----HAEQNFSEFGLSDQ-YTSW---HT----------------------
ORYSJ*  ----HVESNFSEYGLSDH-YSFQ---HT----------------------
HORVU   ----HVESNFSEYGLSDH-YSFQ---HT----------------------
SORBI   ----HVDSNFSDYGLSDQ-YSFQ---HT----------------------
SELML   ----LLEPNFAEYALSDT-YTWQ---HT----------------------
PHYPA   ----PLDPNFSEFNLPDL-YSWQ---HT----------------------
VOLCA   EGQGPAGGGGGAGALSQTLHNNN---NS----------------------
                           660                 670                 680                 690                 700
```

MED20

```
HUMAN*  - - - - - - - - - - - - - - - - - - - - - - - - - - - - - - - - - - - - - - - - - - - - - - - - - -
CHICK   - - - - - - - - - - - - - - - - - - - - - - - - - - - - - - - - - - - - - - - - - - - - - - - - - -
XENTR   - - - - - - - - - - - - - - - - - - - - - - - - - - - - - - - - - - - - - - - - - - - - - - - - - -
DANRE   - - - - - - - - - - - - - - - - - - - - - - - - - - - - - - - - - - - - - - - - - - - - - - - - - -
CIOIN   - - - - - - - - - - - - - - - - - - - - - - - - - - - - - - - - - - - - - - - - - - - - - - - - - -
ANOGA   - - - - - - - - - - - - - - - - - - - - - - - - - - - - - - - - - - - - - - - - - - - - - - - - - -
TETNG   - - - - - - - - - - - - - - - - - - - - - - - - - - - - - - - - - - - - - - - - - - - - - - - - - -
DROME   - - - - - - - - - - - - - - - - - - - - - - - - - - - - - - - - - - - - - - - - - - - - - - - - - -
BRUMA   - - - - - - - - - - - - - - - - - - - - - - - - - - - - - - - - - - - - - - - - - - - - - - - - - -
CAEEL   - - - - - - - - - - - - - - - - - - - - - - - - - - - - - - - - - - - - - - - - - - - - - - - - - -
YEAST*  - - - - - - - - - - - - - - - - - - - - - - - - - - - - - - - - - - - - - - - - - - - - - - - - - -
SCHPO   - - - - - - - - - - - - - - - - - - - - - - - - - - - - - - - - - - - - - - - - - - - - - - - - - -
CANAL   - - - - - - - - - - - - - - - - - - - - - - - - - - - - - - - - - - - - - - - - - - - - - - - - - -
CANGA   - - - - - - - - - - - - - - - - - - - - - - - - - - - - - - - - - - - - - - - - - - - - - - - - - -
ASPNI   - - - - - - - - - - - - - - - - - - - - - - - - - - - - - - - - - - - - - - - - - - - - - - - - - -
RHIO9   - - - - - - - - - - - - - - - - - - - - - - - - - - - - - - - - - - - - - - - - - - - - - - - - - -
CRYNE   - - - - - - - - - - - - - - - - - - - - - - - - - - - - - - - - - - - - - - - - - - - - - - - - - -
USTMA   D D S Q V S S S D S E T E P T L D S S S G A V T G A G G L T G T A V T M Q Q V D E A D D D D D D D R
ENCCU   - - - - - - - - - - - - - - - - - - - - - - - - - - - - - - - - - - - - - - - - - - - - - - - - - -
ANTLO   - - - - - - - - - - - - - - - - - - - - - - - - - - - - - - - - - - - - - - - - - - - - - - - - - -
ARATH*  - - - - - - - - - - - - - - - - - - - - - - - - - - - - - - - - - - - - - - - - - - - - - - - - - -
VITVI   - - - - - - - - - - - - - - - - - - - - - - - - - - - - - - - - - - - - - - - - - - - - - - - - - -
SOYBN   - - - - - - - - - - - - - - - - - - - - - - - - - - - - - - - - - - - - - - - - - - - - - - - - - -
SOLLC   - - - - - - - - - - - - - - - - - - - - - - - - - - - - - - - - - - - - - - - - - - - - - - - - - -
ORYSJ*  - - - - - - - - - - - - - - - - - - - - - - - - - - - - - - - - - - - - - - - - - - - - - - - - - -
HORVU   - - - - - - - - - - - - - - - - - - - - - - - - - - - - - - - - - - - - - - - - - - - - - - - - - -
SORBI   - - - - - - - - - - - - - - - - - - - - - - - - - - - - - - - - - - - - - - - - - - - - - - - - - -
SELML   - - - - - - - - - - - - - - - - - - - - - - - - - - - - - - - - - - - - - - - - - - - - - - - - - -
PHYPA   - - - - - - - - - - - - - - - - - - - - - - - - - - - - - - - - - - - - - - - - - - - - - - - - - -
VOLCA   - - - - - - - - - - - - - - - - - - - - - - - - - - - - - - - - - - - - - - - - - - - - - - - - - -
                            710                 720                 730                 740                 750
```

```
HUMAN*  - - - - - - - - - - - - - - - - - - - - - - - - - - - - - - - - - - - - - - - - - - - - - - - - - -
CHICK   - - - - - - - - - - - - - - - - - - - - - - - - - - - - - - - - - - - - - - - - - - - - - - - - - -
XENTR   - - - - - - - - - - - - - - - - - - - - - - - - - - - - - - - - - - - - - - - - - - - - - - - - - -
DANRE   - - - - - - - - - - - - - - - - - - - - - - - - - - - - - - - - - - - - - - - - - - - - - - - - - -
CIOIN   - - - - - - - - - - - - - - - - - - - - - - - - - - - - - - - - - - - - - - - - - - - - - - - - - -
ANOGA   - - - - - - - - - - - - - - - - - - - - - - - - - - - - - - - - - - - - - - - - - - - - - - - - - -
TETNG   - - - - - - - - - - - - - - - - - - - - - - - - - - - - - - - - - - - - - - - - - - - - - - - - - -
DROME   - - - - - - - - - - - - - - - - - - - - - - - - - - - - - - - - - - - - - - - - - - - - - - - - - -
BRUMA   - - - - - - - - - - - - - - - - - - - - - - - - - - - - - - - - - - - - - - - - - - - - - - - - - -
CAEEL   - - - - - - - - - - - - - - - - - - - - - - - - - - - - - - - - - - - - - - - - - - - - - - - - - -
YEAST*  - - - - - - - - - - - - - - - - - - - - - - - - - - - - - - - - - - - - - - - - - - - - - - - - - -
SCHPO   - - - - - - - - - - - - - - - - - - - - - - - - - - - - - - - - - - - - - - - - - - - - - - - - - -
CANAL   - - - - - - - - - - - - - - - - - - - - - - - - - - - - - - - - - - - - - - - - - - - - - - - - - -
CANGA   - - - - - - - - - - - - - - - - - - - - - - - - - - - - - - - - - - - - - - - - - - - - - - - - - -
ASPNI   - - - - - - - - - - - - - - - - - - - - - - - - - - - - - - - - - - - - - - - - - - - - - - - - - -
RHIO9   - - - - - - - - - - - - - - - - - - - - - - - - - - - - - - - - - - - - - - - - - - - - - - - - - -
CRYNE   - - - - - - - - - - - - - - - - - - - - - - - - - - - - - - - - - C L I T D L L G E R R W I D C S R
USTMA   P L G A P I W S N A N R T P A T S T T N P T A A P A T P P G A T K K E D T A Q L I F Q G L R P D E A
ENCCU   - - - - - - - - - - - - - - - - - - - - - - - - - - - - - - - - - - - - - - - - - - - - - - - - - -
ANTLO   - - - - - - - - - - - - - - - - - - - - - - - - - - - - - - - - - - - - - - - - - - - - - - - - - -
ARATH*  - - - - - - - - - - - - - - - - - - - - - - - - - - - - - - - - - - - - - - - - - - - - - - - - - -
VITVI   - - - - - - - - - - - - - - - - - - - - - - - - - - - - - - - - - - - - - - - - - - - - - - - - - -
SOYBN   - - - - - - - - - - - - - - - - - - - - - - - - - - - - - - - - - - - - - - - - - - - - - - - - - -
SOLLC   - - - - - - - - - - - - - - - - - - - - - - - - - - - - - - - - - - - - - - - - - - - - - - - - - -
ORYSJ*  - - - - - - - - - - - - - - - - - - - - - - - - - - - - - - - - - - - - - - - - - - - - - - - - - -
HORVU   - - - - - - - - - - - - - - - - - - - - - - - - - - - - - - - - - - - - - - - - - - - - - - - - - -
SORBI   - - - - - - - - - - - - - - - - - - - - - - - - - - - - - - - - - - - - - - - - - - - - - - - - - -
SELML   - - - - - - - - - - - - - - - - - - - - - - - - - - - - - - - - - - - - - - - - - - - - - - - - - -
PHYPA   - - - - - - - - - - - - - - - - - - - - - - - - - - - - - - - - - - - - - - - - - - - - - - - - - -
VOLCA   - - - - - - - - - - - - - - - - - - - - - - - - - - - - - - - - - - - - - - - - - - - - - - - - - -
                            760                 770                 780                 790                 800
```

MED20

```
HUMAN*  -----------------MVQYMELFNK--IRKQQQVPVAGIR--------
CHICK   -----------------MVQYMELFNK--IRKQQQVPVAGIR--------
XENTR   -----------------MVQYMELLNK--IRKQQ-VTVAGMR--------
DANRE   -----------------MSQYLELFSK--VRKQQVLPGSGVR--------
CIOIN   -----------------MQQYVELFSN--MRKSNLVR---------------
ANOGA   -----------------INQYLEHFTN--YRKQTMAPVGVRP--------
TETNG   -----------------MTQYLEVFNK--LRKLQ-IPGSNVR--------
DROME   -----------------VKQYLEQFTN--YRKHVTLMGGMGSGPGSQQVG
BRUMA   -----------------MHQYLDIFQN--MRKKT----------------
CAEEL   -----------------MWQYLGIATK--LRKKT----------------
YEAST*  -----------------AYQYVRALEL-----------------------
SCHPO   -----------------FKQRIDTVSQR----------------------
CANAL   -----------------CLQYSKILNF-----------------------
CANGA   -----------------AKQYIEVLE------------------------
ASPNI   -----------------ARQFMEIFRFNR---------------------
RHIO9   -----------------SYQYIMLFKNDGLF-------------------
CRYNE   GQENWRAWDDAERNKCAMLTLAKTLRQGNFI-------------------
USTMA   DSTAEDGWSGIERGRRIAFQYVQMLRAEGII-------------------
ENCCU   -----------------SLEFLYRKNS--VRK------------------
ANTLO   -----------------CVPFLAKRTKEDLNNV-----------------
ARATH*  -----------------AVRYALVMAH--MIATVQAVRG-----------
VITVI   -----------------AVQYATVMAQ--LISAVQTAQGVRN--------
SOYBN   -----------------AVQYAAALAQ--LIQSAQLRN------------
SOLLC   -----------------VVQYASILAQ--MSTTMVLKKEDGKIPFASISP
ORYSJ*  -----------------AVQYATCLQQ--LMAAVRPQ-------------
HORVU   -----------------AVQYATCLQQ--LMAAVRA--------------
SORBI   -----------------AVQYATCLQQ--LMAAVTVRA------------
SELML   -----------------ALQYIFLFAY--FLSQSQRA-------------
PHYPA   -----------------ALQYVTLMAF--VFSQQRT--------------
VOLCA   -----------------SNSNNSLNRHRSNRKRY----------------
                 810       820       830       840       850
```

```
HUMAN*  --------------------------------------------------
CHICK   --------------------------------------------------
XENTR   --------------------------------------------------
DANRE   --------------------------------------------------
CIOIN   --------------------------------------------------
ANOGA   --------------------------------------------------
TETNG   --------------------------------------------------
DROME   PNVHMSPAVAGLHRS-----------------------------------
BRUMA   --------------------------------------------------
CAEEL   --------------------------------------------------
YEAST*  --------------------------------------------------
SCHPO   --------------------------------------------------
CANAL   --------------------------------------------------
CANGA   --------------------------------------------------
ASPNI   --------------------------------------------------
RHIO9   --------------------------------------------------
CRYNE   --------------------------------------------------
USTMA   --------------------------------------------------
ENCCU   --------------------------------------------------
ANTLO   --------------------------------------------------
ARATH*  --------------------------------------------------
VITVI   --------------------------------------------------
SOYBN   --------------------------------------------------
SOLLC   CSKMHKNFPQHFLGASLQEQPDKFYMALSRKRRIVEAESSMQTIMANLQS
ORYSJ*  --------------------------------------------------
HORVU   --------------------------------------------------
SORBI   --------------------------------------------------
SELML   --------------------------------------------------
PHYPA   --------------------------------------------------
VOLCA   --------------------------------------------------
                 860       870       880       890       900
```

MED20

```
HUMAN*  --------------------------------------------------
CHICK   --------------------------------------------------
XENTR   --------------------------------------------------
DANRE   --------------------------------------------------
CIOIN   --------------------------------------------------
ANOGA   --------------------------------------------------
TETNG   --------------------------------------------------
DROME   --------------------------------------------------
BRUMA   --------------------------------------------------
CAEEL   --------------------------------------------------
YEAST*  --------------------------------------------------
SCHPO   --------------------------------------------------
CANAL   --------------------------------------------------
CANGA   --------------------------------------------------
ASPNI   --------------------------------------------------
RHIO9   --------------------------------------------------
CRYNE   --------------------------------------------------
USTMA   --------------------------------------------------
ENCCU   --------------------------------------------------
ANTLO   --------------------------------------------------
ARATH*  --------------------------------------------------
VITVI   --------------------------------------------------
SOYBN   --------------------------------------------------
SOLLC   YRIKFALNCENLRGIVMEMEYLPISSWKTSHLIMSEFFKILKETLGKKSL
ORYSJ*  --------------------------------------------------
HORVU   --------------------------------------------------
SORBI   --------------------------------------------------
SELML   --------------------------------------------------
PHYPA   --------------------------------------------------
VOLCA   --------------------------------------------------
                 910       920       930       940       950
```

```
HUMAN*  -----------------------------------------
CHICK   -----------------------------------------
XENTR   -----------------------------------------
DANRE   -----------------------------------------
CIOIN   -----------------------------------------
ANOGA   -----------------------------------------
TETNG   -----------------------------------------
DROME   -----------------------------------------
BRUMA   -----------------------------------------
CAEEL   -----------------------------------------
YEAST*  -----------------------------------------
SCHPO   -----------------------------------------
CANAL   -----------------------------------------
CANGA   -----------------------------------------
ASPNI   -----------------------------------------
RHIO9   -----------------------------------------
CRYNE   -----------------------------------------
USTMA   -----------------------------------------
ENCCU   -----------------------------------------
ANTLO   -----------------------------------------
ARATH*  -----------------------------------------
VITVI   -----------------------------------------
SOYBN   -----------------------------------------
SOLLC   PGHFVHAEQNFSEFGLFDQYTSRHTVVQYSSILAQMSTTAQ
ORYSJ*  -----------------------------------------
HORVU   -----------------------------------------
SORBI   -----------------------------------------
SELML   -----------------------------------------
PHYPA   -----------------------------------------
VOLCA   -----------------------------------------
                 960       970       980       990
```

MED21

```
HUMAN*  --------------------------------------------------
CHICK   --------------------------------------------------
XENTR   --------------------------------------------------
DANRE   --------------------------------------------------
CIOIN   --------------------------------------------------
ANOGA   --------------------------------------------------
TETNG   --------------------------------------------------
DROME   --------------------------------------------------
BRUMA   --------------------------------------------------
CAEEL   --------------------------------------------------
YEAST*  --------------------------------------------------
SCHPO   --------------------------------------------------
CANAL   --------------------------------------------------
CANGA   --------------------------------------------------
ASPNI   --------------------------------------------------
RHIO9   --------------------------------------------------
CRYNE   --------------------------------------------------
USTMA   --------------------------------------------------
ENCCU   --------------------------------------------------
ANTLO   --------------------------------------------------
ARATH*  --------------------------------------------------
SOYBN   --------------------------------------------------
SOLLC   --------------------------------------------------
ORYSJ*  MGPRASQRVACALSMCWAHQFSLMGLDVIGPWWIERRRREKEREKRRRRR
HORVU   --------------------------------------------------
SORBI   --------------------------------------------------
SELML   --------------------------------------------------
PHYPA   --------------------------------------------------
VOLCA   --------------------------------------------------
                 10        20        30        40        50
```

```
HUMAN*  -----------MADRLTQLQDAVNSLADQFCNAIGVLQQCGPPAS-----
CHICK   -----------MADRLTQLQDAVNSLADQFCNAIGVLQQCGPPAS-----
XENTR   -----------MADRLTQLQDALNSLADQFCNAIGVLQQCAPPAS-----
DANRE   -----------MADRLTQLQDAVNSLADQFCNAIGVLQQCAPPAS-----
CIOIN   -----------MSDRLTQLQDAVNQLAEHFCNGVGILQQSAVPGS-----
ANOGA   -----------MADRLTQLQDTVNQQAEHFCNSIGILQQCSVPSK-----
TETNG   -----------MADRLTQLQDAVNSLADQFCNAIGVLQQCAPPAS-----
DROME   -----------MADRLTQLQDTVNQQAEHFCNAIGVIQQTSLPSK-----
BRUMA   -----------MADRLTQLQDLVNELANLMCNSIGVLRLTAPSCD-----
CAEEL   -----------MADRMTQLQDMINEMAGLMTNAIGVLQATAPPCE-----
YEAST*  -----------MTDRLTQLQICLDQMTEQFCATLNYIDKNHGFER-----
SCHPO   -----------MACRCTQLQDTIDEVATQFYSSIHYLSSHHDFVP-----
CANAL   -----------MADRLTQLQICLDQLIQQFNSTINYVNTSAEPSL-----
CANGA   -----------MTDRLTQLQRCLDQVMEQFCATLNFIDKRHDFEP-----
ASPNI   -----------MADILTQLQTCLDQLATQFYATIGYLTTYHDNSPAIPPD
RHIO9   ------------MDRLTQLQDAIDAMARMFTNSIYYVHEKSSMAE-----
CRYNE   --------------MITQLQDAILDLLTITSTSIEYITKRTQFEQ-----
USTMA   ------------MDLLTQLDNDIDLLLKIMSSSIAYISRKAKHAP-----
ENCCU   -------------MKITDLQNEINHLLFLYFTSIGVIQRDAGSSD-----
ANTLO   --------------MFMDLRNETNHLLYLYFNSIGIMQRDYGNED-----
ARATH*  ------------MDIISQLQEQVNTIAAITFNAFGTLQRDAPPVQ-----
SOYBN   ------------MDIISQLQEQVNLIAHLAFNTVGTLQRDAPPNR-----
SOLLC   ------------MDIISQLQEQVNTIAALAFNTFGTLQRDAPPVR-----
ORYSJ*  SPAARRGTRSPEMDIISQLQEQLNEMAMVAVNTFGTLQRDAPPVR-----
HORVU   ------------MDIISQLQEQLNEIAMVSVNTFGTLQRDAPPVR-----
SORBI   ------------MDIITQLQDQLDEMAVLAVNTFGTLQRDAPPDR-----
SELML   ------------MDIITQLQDQVNKIAFLAFNSVGSLQRDAAPVR-----
PHYPA   ------------MDIIAQLQEHASAIAYIAFNAAGTIQRDAPPSQ-----
VOLCA   ----------MSSDLLTELQRQLRDVNDQFAIVIEQLIHIAPPAP-----
                 60        70        80        90       100
```

```
HUMAN*  ----------FNNIQTAINKDQP---------------------------
CHICK   ----------FSNIQTAINKDQP---------------------------
XENTR   ----------FSNIQTGINKDQP---------------------------
DANRE   ----------FSNIQTAINKDQP---------------------------
CIOIN   ----------FAGLEKSGSKEPA---------------------------
ANOGA   ----------FAGFERTGSQTP----------------------------
TETNG   ----------FSNGKTAINKEQP---------------------------
DROME   ----------FVNFERIGPQTPI---------------------------
BRUMA   ----------FNGTSKALEDEE----------------------------
CAEEL   ----------FGAISQELEDEP----------------------------
YEAST*  ----------LTVNEPQMSDKHA---------------------------
SCHPO   ----------LPGQEKVSDSKV----------------------------
CANAL   ----------LDDDDVNSYSNMAANAPLPQSQQQRQQQKKQQEPQQEIEQ
CANGA   ----------FNDKEPKMTDKHA---------------------------
ASPNI   NPTSAPALAKIQKNSTNPPIPAGAAAILNASQGSPSGAATGAVTPGPPAP
RHIO9   ----------LNKEIP----------------------------------
CRYNE   ----------TSISIPTTLSTP----------------------------
USTMA   ----------LPDSTIPLTVLGKT--------------------------
ENCCU   ----------VHD-------------------------------------
ANTLO   ----------ILE-------------------------------------
ARATH*  ----------LSPNYPEP--------------------------------
SOYBN   ----------LSPNYPEP--------------------------------
SOLLC   ----------LSPNYPEP--------------------------------
ORYSJ*  ----------LSNNYPDPLNPAAAAAANPNPDDPA-QPQPGAAAAAAAGA
HORVU   ----------LSNSYPDPLNP------NPNPDGPASQPQ-------APPA
SORBI   ----------LSNSYPDPLNP------NPKPEDVSTKPQ-------VQGQ
SELML   ----------LSQNYPDPQALAPGAPPLPGQQQQQQQQAQQSQ-------
PHYPA   ----------LAQDYPKPTG------------------------------
VOLCA   ----------LEGDSGQIVAEEP---------------------------
                110       120       130       140       150
```

MED21

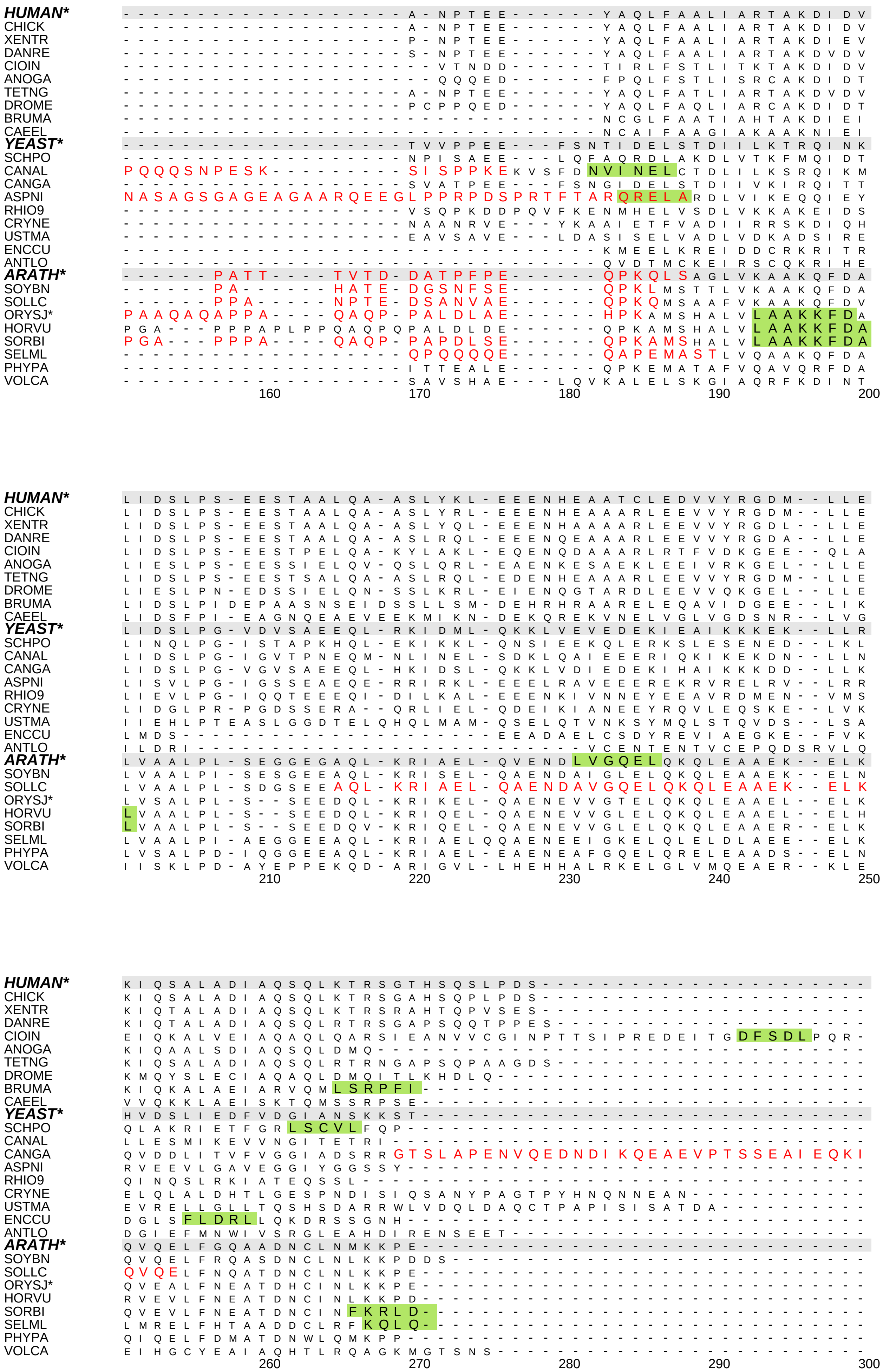

MED21

```
HUMAN*  -------------------------------------------------
CHICK   -------------------------------------------------
XENTR   -------------------------------------------------
DANRE   -------------------------------------------------
CIOIN   -------------------------------------------------
ANOGA   -------------------------------------------------
TETNG   -------------------------------------------------
DROME   -------------------------------------------------
BRUMA   -------------------------------------------------
CAEEL   -------------------------------------------------
YEAST*  -------------------------------------------------
SCHPO   -------------------------------------------------
CANAL   -------------------------------------------------
CANGA   ANEKIESKIEGEYNDNINEDSDSKSADSELFMDKDDADNISESISPGKI
ASPNI   -------------------------------------------------
RHIO9   -------------------------------------------------
CRYNE   -------------------------------------------------
USTMA   -------------------------------------------------
ENCCU   -------------------------------------------------
ANTLO   -------------------------------------------------
ARATH*  -------------------------------------------------
SOYBN   -------------------------------------------------
SOLLC   -------------------------------------------------
ORYSJ*  -------------------------------------------------
HORVU   -------------------------------------------------
SORBI   -------------------------------------------------
SELML   -------------------------------------------------
PHYPA   -------------------------------------------------
VOLCA   -------------------------------------------------
                 310       320       330       340
```

```
HUMAN*
CHICK
XENTR
DANRE
CIOIN
ANOGA
TETNG
DROME
BRUMA
CAEEL
YEAST*
SCHPO
CANAL
CANGA
ASPNI
RHIO9
CRYNE
USTMA
ENCCU
ANTLO
ARATH*
SOYBN
SOLLC
ORYSJ*
HORVU
SORBI
SELML
PHYPA
VOLCA
```

MED22

Species	Positions 1–50
HUMAN*	--
CHICK	--
XENTR	--
DANRE	--
CIOIN	--
ANOGA	--
TETNG	--
DROME	--
BRUMA	--
CAEEL	--
YEAST*	--
SCHPO	--
CANAL	--
CANGA	--
ASPNI	--
RHIO9	--
CRYNE	----------------------MSQPFKTDSLRPSSLPSATLGRRRPQLP
USTMA	MESDAPTLAHCSYAGGSPDTTWSSASSSTFRSDVALPTALNLGKIGTSAT
ENCCU	--
ANTLO	--
ARATH*	---MMNKG
VITVI	--MNK-
SOYBN	--MNKG
SOLLC	--MNKG
ORYSJ*	--MSK-
HORVU	--MSKP
SORBI	--MSKA
SELML	--MNKP
PHYPA	--
VOLCA	--

10 20 30 40 50

Species	Positions 51–100
HUMAN*	--------------MAQQRALPQSKETLLQSYNKRL--KDDIKSIMDNFT
CHICK	--------------MAQQRVLPQSKETLLQSYNKRL--KDDVKSIMDNFT
XENTR	--------------MSQQRILPQSKETLLQSYNKRL--KDDIKSIMDNFT
DANRE	--------------MSTPRVLPQSKETLLQNYNKRL--KDDIRSILDNFT
CIOIN	--------------MAQRQALSHTIQNQLNSYNKRL--KDDIRSMLDNFC
ANOGA	----------------MQRNLTQSKEALLKSYNSRL--KEDIRSMRENFE
TETNG	--------------MATQRVLPQSKETLLQSYNKRL--KDDIKSILENFT
DROME	-----------MASGSRTTILPQSKEALLKSYNARL--KDDVRSMLENFE
BRUMA	------MAQVSAATVRKSGRNIATKALIVQEYKRRL--KDNIKSLNDNFI
CAEEL	-----MSGVAGGASKKSSSRTMATKKLIIDEFKRRL--RDNIKSLNDNFF
YEAST*	----------------------MSNQALYE----KL--EQTRTILSVKLA
SCHPO	-MSSDSFQRQLVQR----------TNTLNS----SI--DNATLTILSRFQ
CANAL	--------------------MQPKSISLLQ----KI--DSIIETIIVKFT
CANGA	----------------------MSNQALLE----TL--NQTTELLSVKLA
ASPNI	------------------MDLQPTSKNMHN----RI--NTDISQLLQRFE
RHIO9	--MTTIPSFHSSNKSAPRPFLVQVDDQYNK----RI--DDDISKLVDCYT
CRYNE	TTAALEDGENLTPGERAQRVLEKEHERWNE----RI--DRDVKACVEGLK
USTMA	TASTAAASAAPTRSGDSAAYLDSLEEGVNK----TI--DAEVDTLLSSYK
ENCCU	----------------------MERIELVK----GI--EANAERILEDFT
ANTLO	--------------------MQSAYIEFDM----KIMGQKTSFRIFETRT
ARATH*	G----GSGGGSGPTAAAAAAALQKQKALLQ----RV--DTDITSVVDNFN
VITVI	-----GGGAGGGPTAAAAAAAAQKQKTLLQ----RV--ETDIANVVDHFT
SOYBN	AAGGLGGGAGSGPTAAAAAAAAQKQKTLLQ----RV--EGDIANIVDNFN
SOLLC	ANV-----GGGGPTAAAAAAAAQKQKSLLQ----RV--DADIGNIVDNYG
ORYSJ*	-----SGGAAAGPTAAAAAAAVQKQKTLLQ----KA--DADVSSLVDNFA
HORVU	GGS--AGGAMAGPTAAAAAAAAQKQRALLQ----KA--DADVGSLVDNFS
SORBI	GG---GPGSAPGPTAAAAAAAVQKQKSLLQ----KA--DADVSSLVDNFS
SELML	GQ---GGGGAAATTAAAAAATIHRQRALLQ----RA--DADIANLIDNFA
PHYPA	-------GGVAGPTAAAAAQAAQRQRSLLQ----RA--DADIGSLLDNFS
VOLCA	---------------------MSLEGNSWS----EL--VEQLNMMVTNFN

60 70 80 90 100

MED22

```
HUMAN*   E I I K T A K I E D E T - - - - - - - - - Q V S R A T Q G E Q D N Y - - - - E M H V R A A N I V R A
CHICK    E I I K T A K I E D E T - - - - - - - - - Q V S R A T Q S E Q D N Y - - - - E M H V R A A N I V R A
XENTR    E I I K T A K I D E E H - - - - - - - - - Q V S R S T Q G E Q D N Y - - - - E M H V R S A N I V R A
DANRE    E I I K T A K V E D E T - - - - - - - - - Q V S R A T Q A E Q D H F - - - - E M H V R A A N I V R A
CIOIN    E I I K S G K V D E N Q - - - - - - - - - Q L D R A T Q N Y Y D V Y - - - - Q M R V R S A N I V R A
ANOGA    E I I R L A K G E N D T - - - - - - - - - Q L S K I T Q C E Q D T Y - - - - E T Q V R A A N I V R A
TETNG    E I I K T A K I E D E T - - - - - - - - - Q V S R P T Q A E Q D H Y - - - - E M H V R A A N I V R A
DROME    E I L K L A R R E S H S - - - - - - - - - Q I S K T T Q C E Q D A L - - - - E M Q V R A A N M V R A
BRUMA    N I I S V A K I N I D D - - - - - - - - A A H K N P V G R M S D Y Y T M R N E T A A R A A L M I R A
CAEEL    H I I Q A A K V N P D D - - - - - - - - N A Y K N Q T G K M T E F Y T T K N E M A V R A Q L M V R A
YEAST*   E L I N M T T I A D R N D D D E G S F A Q E N S E L A V A T T S V M - - - - M V N N Q T M Q L I K N
SCHPO    D I L D I A I N E G K D - - - - - - - - - - - - - K Y T V A P E V Y - - - - Q I E C H T V S M V R A
CANAL    N I F E N L Q D A N K T - - - - - - - - - - - - - T E I L S M E S L - - - - A M E N N C I Q I I R L
CANGA    E L A K L A S M E T N E D - - - - - - - T E D C Q L S V V T N G L M - - - - M V N N Q T L Q L V R G
ASPNI    N I M A T A T V E S T S - - - - - - - - - - - - - H T T T A V E T Y - - - - Q L D V E S T A L I R A
RHIO9    D I V R I G E D K - - - - - - - - - - - - - - - - F K V A Q E G Y - - - - Q I E S Q A A Q I V R S
CRYNE    E L V Q L A D I S - - - - - - - - - - - T V Q P N S L T S T T L P L - - - - H L P V R T S S L I R S
USTMA    E L V S L A T I A D K D - - - - - - - - - - - - - K Y R V A Q E A F - - - - Q S E A R A D I M V R S
ENCCU    E I L Q Y L R S G G S D - - - - - - - - - - - - - K C D E V Q N L L - - - - F A S R R T V D L I D S
ANTLO    H L F L Y I S Q D - - - - - - - - - - - A P D A K Y F N K M L E K S V S K K S I A S R K K H L V F C
ARATH*   Q I V N V A R V S D P - - - - - - - - - - - - - - P M K N S Q E A Y - - - - M M E M R A S R L V Q A
VITVI    F L V N V A R V N D P - - - - - - - - - - - - - - P V R N S Q E A F - - - - M M E M R A A R M V Q A
SOYBN    H L V N V A R V N D P - - - - - - - - - - - - - - P V R N S Q E A F - - - - M M E M R A A R M V Q A
SOLLC    F I V N A A R V N D P - - - - - - - - - - - - - - P V R N S Q E A F - - - - M M E M R A S R M V H A
ORYSJ*   A L I N I A R V N D P - - - - - - - - - - - - - - P V R N T Q E A F - - - - Q M D M R G S R M V H S
HORVU    A L I N I A R V N D P - - - - - - - - - - - - - - P V R N S Q E A F - - - - Q M D M R A S R M V H S
SORBI    S L I N I A R V N D P - - - - - - - - - - - - - - P V R N S Q E V F - - - - Q M E M R A A R M V H A
SELML    H M L K A A R V N D - - - - - - - - - - - - - - - P V R N A Q E N F - - - - Q M D I H A S R V V Q A
PHYPA    H L L K A A R I N D - - - - - - - - - - - - - - - P V R N G Q E A F - - - - Q M E V H A A R V V Q A
VOLCA    D L V K A S R I P D E S - - - - - - - - - - - V D E L S A S R E R K V P G D L Q E V L A E K L L F S
                             110                 120                 130                 140                 150
```

```
HUMAN*   G E S L M K L V S D L K Q F L I L N D F P - - S V N E A I D Q R N Q Q L R T L Q E E C D R K L I T -
CHICK    G E S L M K L V S D L K Q F L I L N D F P - - S V N E A I N Q R N Q Q L R S L Q E E C D K K L I A -
XENTR    G E S L M K L V S D L K Q F L I L N D F P - - S V N E S I N Q R N Q Q L R A L Q D E C D K K L I A -
DANRE    G E S L M K L V S D L K Q F L I L N D F P - - S V N E A I S L R N Q Q L R T L Q E E C D K K L I S -
CIOIN    G E S L L R L I A E L K T F L I L N D F P - - S I N R H I E A Q N N H L N L V S G K W N E Q L V R -
ANOGA    G E S L M K L V S D I K Q Y L I L N D F H - - S V N E A I C S N S T L Y R T T Q I D R D N K L M A -
TETNG    G E S L M K L V S D L K Q F L I L N D F P - - S V N D A I S L Q N Q Q L R S L Q E E C D K K L I S -
DROME    G E S L M K L V A D L K Q Y L I L N D F H - - S V N E A I T N N S Q L F R N T Q S E C D K K L M K -
BRUMA    A D E L L K L T H D L K E F L I L H D F N - - F L S Y A I Q N A E D R A E K K M D D C I L R Y D A -
CAEEL    S D E L L K L T A D L K E F L I L H D F H - - F L T H N I K Q A E A Q C E E T L R Q Q S H Q H N C -
YEAST*   V Q D L L I L T R S I K E K W L L N Q I P - - V T E H S K - V T R F D E K Q I E E L L D N C I E T -
SCHPO    V E Q L L D V S R Q I K S Y W L T N S L S - - T S F P T V D Y S E P D L E K V K R T L T K L Q N H -
CANAL    C Q D L I S I S R N L K E I W V L N S I K - - V T Q E K F E W K Q E E I D T M F T Q F N L - - - - -
CANGA    V Q D L I L L T R N I R E K W L L T Q I P - - E Q L T P K E Q D S I N H E E L T E L L E S C V S E -
ASPNI    A E D I L S L T R T M K E T W L F G K L D T L G E D E S E T K R R E G L E Q D A A V I Q K I I E D A
RHIO9    C E S L L S L T N E L K Q F L L L N D A E - - T L A Q L R K T Q S E K L V E S T T T I K S K V N T -
CRYNE    A L N I R D M A H E L K L L L L L S D E V - - G V V Q A R D R E R R L V G E E I Q R G R E N V L R E
USTMA    T Q T L G L L S E A L K L S L L L S K T A D P A L N T E A M H L I E S T E A E K L Q C A H L L S H -
ENCCU    A L E M Y H Y F V R L K - - Y L T A D V E K P E A M D K R D G G K M L A N - - - - - - - - - - - - -
ANTLO    R L K G Q E Y L K T V S H I I L - - D V L Q G K A V D K I T G G R - - - - - - - - - - - - - - - - -
ARATH*   A D S L L K L V S E L K Q T A I F S G F A - - S L N D H V E Q R I A E F D Q E A E K T N R L L A R -
VITVI    A D S L L K L V S E L K Q T A I F S G F A - - S L N D H V D Q R E A E F N Q Q A E K T D H L L A R -
SOYBN    A D S L L K L V S E L K Q T A I F S G F A - - S L N D H V E E R R I E F N Q L A E K T D R T L S R -
SOLLC    A D S L L K L V S E L K Q T A M F S G F A - - S L N D H V E Q R T E E F T E Q A E K T E C M L S R -
ORYSJ*   A D S L L K L V S E L K R T A I F S G L A - - S L T E N V D R R I E I F S Q Q V E G T E R M L E R -
HORVU    A D S L L K L V S E L K R T A I F S G L A - - S L S E N V D R R I E V L S Q Q A E E T E R M L E R -
SORBI    A D S L L K L V S E L K R T A I F S G L A - - S L N E N V D R R I E V L G Q Q A E G T E R M L E R -
SELML    A D S L L K L V S E L K Q N A V F S D F I - - S L N E C I A E R A A R F E E Q N S D T E R L L L S -
PHYPA    A D S L L K L V S E L K Q S A V F S D F L - - S L N R S I N Q R I V K F G E Q S Q D S D K L L F N -
VOLCA    A Y M C L H H I S E L K K A A A L S D H T - - T Q I G N A R A V K S Q M Q R T E D V I A S K I G L -
                             160                 170                 180                 190                 200
```

MED22

```
HUMAN*   ----LRDEISIDLYELEEEYYSSRYK------------------------
CHICK    ----LRDEISIDLYELEEEYYSSRYK------------------------
XENTR    ----LRDDIAIDLYELEEEYYSSSVC--DPSDLPLCEVYWNRESTASSPE
DANRE    ----LRDEIAIDLYELEEEYYSSRYK------------------------
CIOIN    ----LRDEASVVLHDLESELYSSIRPKQ----------------------
ANOGA    ----VRDDMAADLYDLEEEYYTSIYK------------------------
TETNG    ----LRDEIAIDLFELEEEYYSSRYNHWDRTDLPLCESYHQGDSWASPGS
DROME    ----LRDEMAMDLYDLEEEYYTSIFK------------------------
BRUMA    ----MRLDISSMVTDIDRELMDHFSIRH----------------------
CAEEL    ----LDSEVSNILFDLEREIAENFYLRHT---------------------
YEAST*   ----FVAEKTT---------------------------------------
SCHPO    ----L---LEVSLIEPEASETTEAPTVSDT--------------------
CANAL    ----LTDKIAEFETDMNKE-------------------------------
CANGA    ----IISDV-----------------------------------------
ASPNI    GILKAPKK------------------------------------------
RHIO9    ----MRSELSKAVFDLETVYYRSLIDE-----------------------
CRYNE    IYKGLGRELEGDAEEIAMDKTTGAESSVKDEKYQGAKDGEDVMEIASEER
USTMA    ----IMQLDVRNAQHLVSHLDRALVDPTTRPEAH----------------
ENCCU    ----MFGELNANLGV-----------------------------------
ANTLO    --------------------------------------------------
ARATH*   ----IADDASASLKELESHYYSSAQRSTLD--------------------
VITVI    ----IGEEAAASLKELESHYYSSAQRTSEPPQ------P-----------
SOYBN    ----VGEEAAASLKGLESHYSSSVQKIIQNPQ------P-----------
SOLLC    ----IGEEAAASLKELESHYYSSAERTSSLPSYSQETMP-----------
ORYSJ*   ----IGQEAAGSLKELEAHYYSSVVRTPPDE-------------------
HORVU    ----IGQEAAASLKELEAHYYSSVVRTPLYD-------------------
SORBI    ----IGQEAAASLKELEAHYYSSVVRSPSYD-------------------
SELML    ----VGEELGASLVELETHYYKSLQRLSPAEHQAQE--------------
PHYPA    ----LGDEIAASLQELETHYYASPFRS-----------------------
VOLCA    ----IKVEAQELLGQLEESYYSSKFKGRPTESGV----------------
                  210       220       230       240       250
```

```
HUMAN*   --------------------------------------------------
CHICK    --------------------------------------------------
XENTR    DPSTPLAPIMAEASAAPSQIHTPPHLNGHGAGMAEHT-------------
DANRE    --------------------------------------------------
CIOIN    --------------------------------------------------
ANOGA    --------------------------------------------------
TETNG    SCSSTQGDQNEVEGLSSQETDLQHHVNGHGTSSLEKI-------------
DROME    --------------------------------------------------
BRUMA    --------------------------------------------------
CAEEL    --------------------------------------------------
YEAST*   --------------------------------------------------
SCHPO    --------------------------------------------------
CANAL    --------------------------------------------------
CANGA    --------------------------------------------------
ASPNI    --------------------------------------------------
RHIO9    --------------------------------------------------
CRYNE    PQPSSPASPPDAVPQLTRGALEEEQEVKKEEVMEVDDDENEDDDGFDIVE
USTMA    -------------------------------------DQSQLAPNQHIPD
ENCCU    --------------------------------------------------
ANTLO    --------------------------------------------------
ARATH*   --------------------------------------------------
VITVI    --------------------------------------------------
SOYBN    --------------------------------------------------
SOLLC    --------------------------------------------------
ORYSJ*   --------------------------------------------------
HORVU    --------------------------------------------------
SORBI    --------------------------------------------------
SELML    --------------------------------------------------
PHYPA    --------------------------------------------------
VOLCA    -------------------------------------SEQLRELCALALE
                  260       270       280       290       300
```

MED22

HUMAN*	--
CHICK	--
XENTR	--
DANRE	--
CIOIN	--
ANOGA	--
TETNG	--
DROME	--
BRUMA	--
CAEEL	--
YEAST*	--
SCHPO	--
CANAL	--
CANGA	--
ASPNI	--
RHIO9	--
CRYNE	VHNNSNLASSAPASVSASAPEVPTIIKDDSEEDEDFEEIF
USTMA	DDMQPVSTG-------------------------------
ENCCU	--
ANTLO	--
ARATH*	--
VITVI	--
SOYBN	--
SOLLC	--
ORYSJ*	--
HORVU	--
SORBI	--
SELML	--
PHYPA	--
VOLCA	THTGHRK---------------------------------
	310 320 330 340

MED23

```
HUMAN*  --------------------------------------------------
CHICK   --------------------------------------------------
XENTR   --------------------------------------------------
DANRE   --------------------------------------------------
CIOIN   --------------------------------------------------
ANOGA   --------------------------------------------------
TETNG   --------------------------------------------------
DROME   --------------------------------------------------
BRUMA   MGTTTEAIATSATSLTAAVGNDAEKLQWIVLNQVEGVQMREMFWDLSKDV
CAEEL   --------------------------------------------------
RHIO9   MTDNKYDHELIQLPLSSNLIDYDALTNDFIKTCQMGNNVAEQLKVIMIKY
ARATH*  --------------------------------------------------
VITVI   --------------------------------------------------
SOYBN   --------------------------------------------------
SOLLC   --------------------------------------------------
ORYSJ*  --------------------------------------------------
HORVU   --------------------------------------------------
SORBI   --------------------------------------------------
SELML   --------------------------------------------------
PHYPA   --------------------------------------------------
                 10        20        30        40        50
```

```
HUMAN*  --------------------------------------------------
CHICK   --------------------------------------------------
XENTR   -----------------------------------------------E--
DANRE   --------------------------------------------------
CIOIN   --------------------------------------------------
ANOGA   --------------------------------------------------
TETNG   --------------------------------------------------
DROME   --------------------------------------------------
BRUMA   DVDVLACSEAVKMLRTMTEEEKTQCCKASLSLLSNKDDPRYIHYERILSS
CAEEL   ------------------------------------MTDKPGSAEDTI--
RHIO9   REPPLTPEKTYRLITFLHHTLKEIPITANAGGFPLGSVIEDLSLTLSL--
ARATH*  ------------------------------------------MDQSQR--
VITVI   --------------------------------------------------
SOYBN   ----------------------------------------MDQSQNQR--
SOLLC   --------------------------------------------------
ORYSJ*  ----------------------------MDGAHGQRQPMSPAISASAV--
HORVU   --------------------------------------------------
SORBI   ----------------------------MDGGHGTRQPMSPAISASAV--
SELML   --------------------------------------------------
PHYPA   --------------------------------------------------
                 60        70        80        90       100
```

```
HUMAN*  --------------------------------------------------
CHICK   --------------------------------------------------
XENTR   --------------------------------------------------
DANRE   --------------------------------------------------
CIOIN   --------------------------------------------------
ANOGA   --------------------------------------------------
TETNG   --------------------------------------------------
DROME   --------------------------------------------------
BRUMA   IFMIACNEGVLPLSDCCELLILCTNFSLTTPMDSRKFEYMQKNLHLIDYK
CAEEL   --------------------------------------------------
RHIO9   --------------------------------------------------
ARATH*  --------------------------------------------------
VITVI   --------------------------------------------------
SOYBN   --------------------------------------------------
SOLLC   --------------------------------------------------
ORYSJ*  --------------------------------------------------
HORVU   --------------------------------------------------
SORBI   --------------------------------------------------
SELML   --------------------------------------------------
PHYPA   --------------------------------------------------
                110       120       130       140       150
```

```
HUMAN*  --------------------------------------------------
CHICK   --------------------------------------------------
XENTR   --------------------------------------------------
DANRE   --------------------------------------------------
CIOIN   --------------------------------------------------
ANOGA   --------------------------------------------------
TETNG   --------------------------------------------------
DROME   --------------------------------------------------
BRUMA   GLRNILKLLVVERMQEVPSTITHHHRHMLLPVENMLLTLIDRQLNLLPCI
CAEEL   --------------------------------------------------
RHIO9   --------------------------------------------------
ARATH*  --------------------------------------------------
VITVI   --------------------------------------------------
SOYBN   --------------------------------------------------
SOLLC   --------------------------------------------------
ORYSJ*  --------------------------------------------------
HORVU   --------------------------------------------------
SORBI   --------------------------------------------------
SELML   --------------------------------------------------
PHYPA   --------------------------------------------------
                160       170       180       190       200
```

MED23

```
HUMAN*  --------------------------------------------------
CHICK   --------------------------------------------------
XENTR   --------------------------------------------------
DANRE   --------------------------------------------------
CIOIN   --------------------------------------------------
ANOGA   --------------------------------------------------
TETNG   --------------------------------------------------
DROME   --------------------------------------------------
BRUMA   FTITELHRVSNNSRAFLLPRVAKKFNDMFISFRPLTEMVTIIGRSWLYPI
CAEEL   --------------------------------------------------
RHIO9   --------------------------------------------------
ARATH*  --------------------------------------------------
VITVI   --------------------------------------------------
SOYBN   --------------------------------------------------
SOLLC   --------------------------------------------------
ORYSJ*  --------------------------------------------------
HORVU   --------------------------------------------------
SORBI   --------------------------------------------------
SELML   --------------------------------------------------
PHYPA   --------------------------------------------------
                           210       220       230       240       250

HUMAN*  --------------------------------------------------
CHICK   --------------------------------------------------
XENTR   --------------------------------------------------
DANRE   --------------------------------------------------
CIOIN   --------------------------------------------------
ANOGA   --------------------------------------------------
TETNG   --------------------------------------------------
DROME   --------------------------------------------------
BRUMA   AAHISFPVSTPSWKLEVTTTRLHQRAHLPYKSELFAPQSFLLYTLLRQPR
CAEEL   --------------------------------------------------
RHIO9   --------------------------------------------------
ARATH*  --------------------------------------------------
VITVI   --------------------------------------------------
SOYBN   --------------------------------------------------
SOLLC   --------------------------------------------------
ORYSJ*  --------------------------------------------------
HORVU   --------------------------------------------------
SORBI   --------------------------------------------------
SELML   --------------------------------------------------
PHYPA   --------------------------------------------------
                           260       270       280       290       300

HUMAN*  --------------------------------------------------
CHICK   --------------------------------------------------
XENTR   --------------------------------------------------
DANRE   --------------------------------------------------
CIOIN   --------------------------------------------------
ANOGA   --------------------------------------------------
TETNG   --------------------------------------------------
DROME   --------------------------------------------------
BRUMA   GKDTISYVMRQNTSLTPQRLQCDELLHMIILEAMSEMEKTDTRLDDPANQ
CAEEL   --------------------------------------------------
RHIO9   --------------------------------------------------
ARATH*  --------------------------------------------------
VITVI   --------------------------------------------------
SOYBN   --------------------------------------------------
SOLLC   --------------------------------------------------
ORYSJ*  --------------------------------------------------
HORVU   --------------------------------------------------
SORBI   --------------------------------------------------
SELML   --------------------------------------------------
PHYPA   --------------------------------------------------
                           310       320       330       340       350

HUMAN*  --------------------------------------------------
CHICK   --------------------------------------------------
XENTR   --------------------------------------------------
DANRE   --------------------------------------------------
CIOIN   --------------------------------------------------
ANOGA   --------------------------------------------------
TETNG   --------------------------------------------------
DROME   --------------------------------------------------
BRUMA   YQWMNITQTVTFSLLHGNASFARLLKILYESLSETVYRKGRDELMWVILQ
CAEEL   --------------------------------------------------
RHIO9   --------------------------------------------------
ARATH*  --------------------------------------------------
VITVI   --------------------------------------------------
SOYBN   --------------------------------------------------
SOLLC   --------------------------------------------------
ORYSJ*  --------------------------------------------------
HORVU   --------------------------------------------------
SORBI   --------------------------------------------------
SELML   --------------------------------------------------
PHYPA   --------------------------------------------------
                           360       370       380       390       400
```

MED23

```
HUMAN*  --------------------------------------------------
CHICK   --------------------------------------------------
XENTR   --------------------------------------------------
DANRE   --------------------------------------------------
CIOIN   --------------------------------------------------
ANOGA   --------------------------------------------------
TETNG   --------------------------------------------------
DROME   --------------------------------------------------
BRUMA   YVAVYIDRVSNEEMVRVAEIYNLLYSDEQTWSGADTDPLLFVRFLVPAAI
CAEEL   --------------------------------------------------
RHIO9   --------------------------------------------------
ARATH*  --------------------------------------------------
VITVI   --------------------------------------------------
SOYBN   --------------------------------------------------
SOLLC   --------------------------------------------------
ORYSJ*  --------------------------------------------------
HORVU   --------------------------------------------------
SORBI   --------------------------------------------------
SELML   --------------------------------------------------
PHYPA   --------------------------------------------------
                  410       420       430       440       450
```

```
HUMAN*  --------------------------------------------------
CHICK   --------------------------------------------------
XENTR   --------------------------------------------------
DANRE   --------------------------------------------------
CIOIN   --------------------------------------------------
ANOGA   --------------------------------------------------
TETNG   --------------------------------------------------
DROME   --------------------------------------------------
BRUMA   WIHFYKKLGNSHTEILPKPSESLWRQIQFLQERTADSDPNIQNVADHNAV
CAEEL   --------------------------------------------------
RHIO9   --------------------------------------------------
ARATH*  --------------------------------------------------
VITVI   --------------------------------------------------
SOYBN   --------------------------------------------------
SOLLC   --------------------------------------------------
ORYSJ*  --------------------------------------------------
HORVU   --------------------------------------------------
SORBI   --------------------------------------------------
SELML   --------------------------------------------------
PHYPA   --------------------------------------------------
                  460       470       480       490       500
```

```
HUMAN*  --------------------------------------------------
CHICK   --------------------------------------------------
XENTR   --------------------------------------------------
DANRE   --------------------------------------------------
CIOIN   --------------------------------------------------
ANOGA   --------------------------------------------------
TETNG   --------------------------------------------------
DROME   --------------------------------------------------
BRUMA   LAAVANAYEKAIKFLVSFLDSSDMPNFQKLVLTAVDVFLDGSPEEMNTVW
CAEEL   --------------------------------------------------
RHIO9   --------------------------------------------------
ARATH*  --------------------------------------------------
VITVI   --------------------------------------------------
SOYBN   --------------------------------------------------
SOLLC   --------------------------------------------------
ORYSJ*  --------------------------------------------------
HORVU   --------------------------------------------------
SORBI   --------------------------------------------------
SELML   --------------------------------------------------
PHYPA   --------------------------------------------------
                  510       520       530       540       550
```

```
HUMAN*  --------------------------------------------------
CHICK   --------------------------------------------------
XENTR   --------------------------------------------------
DANRE   --------------------------------------------------
CIOIN   --------------------------------------------------
ANOGA   --------------------------------------------------
TETNG   --------------------------------------------------
DROME   --------------------------------------------------
BRUMA   HLPYGIISYSKKTPLPLSLIDSLTFHARNHLFQLCLLKLTAMLSIQQAQK
CAEEL   --------------------------------------------------
RHIO9   --------------------------------------------------
ARATH*  --------------------------------------------------
VITVI   --------------------------------------------------
SOYBN   --------------------------------------------------
SOLLC   --------------------------------------------------
ORYSJ*  --------------------------------------------------
HORVU   --------------------------------------------------
SORBI   --------------------------------------------------
SELML   --------------------------------------------------
PHYPA   --------------------------------------------------
                  560       570       580       590       600
```

MED23

HUMAN*	--
CHICK	--
XENTR	--
DANRE	--
CIOIN	--
ANOGA	--
TETNG	--
DROME	--
BRUMA	VPSPATIDTLVRLAVTTEFEYGVKQVLALLSSTLASVNKSTNLGPAQQDR
CAEEL	--
RHIO9	--
ARATH*	--
VITVI	--
SOYBN	--
SOLLC	--
ORYSJ*	--
HORVU	--
SORBI	--
SELML	--
PHYPA	--
	610 620 630 640 650

HUMAN*	--
CHICK	--
XENTR	--
DANRE	--
CIOIN	--
ANOGA	--
TETNG	--
DROME	--
BRUMA	SRDLLFVLCDILSYRFISYPFPINLMLWCYTALGNSQVQMNIVLCSALEQ
CAEEL	--
RHIO9	--
ARATH*	--
VITVI	--
SOYBN	--
SOLLC	--
ORYSJ*	--
HORVU	--
SORBI	--
SELML	--
PHYPA	--
	660 670 680 690 700

HUMAN*	--
CHICK	--
XENTR	--
DANRE	--
CIOIN	--
ANOGA	--
TETNG	--
DROME	--
BRUMA	VMMRYWMWNSPQEMFYLSNAFLGKQGKLAVIFNTANPAFCDPQHGNVSVE
CAEEL	--
RHIO9	--
ARATH*	--
VITVI	--
SOYBN	--
SOLLC	--
ORYSJ*	--
HORVU	--
SORBI	--
SELML	--
PHYPA	--
	710 720 730 740 750

HUMAN*	--
CHICK	--
XENTR	--
DANRE	--
CIOIN	--
ANOGA	--
TETNG	--
DROME	--
BRUMA	QQYTNSHISLELLRCLLLSMFRSLKMTGMEMTAEMMQRCNVNFCWPLSIN
CAEEL	--
RHIO9	--
ARATH*	--
VITVI	--
SOYBN	--
SOLLC	--
ORYSJ*	--
HORVU	--
SORBI	--
SELML	--
PHYPA	--
	760 770 780 790 800

MED23

Species	801–850
HUMAN*	--
CHICK	--
XENTR	--
DANRE	--
CIOIN	--
ANOGA	--
TETNG	--
DROME	--
BRUMA	RTYSSQLIGCNVDDGAADTVIYDELMHRVIQEVHQVQAAEEQLLKFFSGE
CAEEL	--
RHIO9	--
ARATH*	--
VITVI	--
SOYBN	--
SOLLC	--
ORYSJ*	--
HORVU	--
SORBI	--
SELML	--
PHYPA	--

810 820 830 840 850

Species	851–900
HUMAN*	--
CHICK	--
XENTR	--
DANRE	--
CIOIN	--
ANOGA	--
TETNG	--
DROME	--
BRUMA	RRQTIFCVVYNMLFETKKIHPVIYSVLSSMNNKELTATINKFTDYFIFIF
CAEEL	--
RHIO9	--
ARATH*	--
VITVI	--
SOYBN	--
SOLLC	--
ORYSJ*	--
HORVU	--
SORBI	--
SELML	--
PHYPA	--

860 870 880 890 900

Species	901–950
HUMAN*	--
CHICK	--
XENTR	--
DANRE	--
CIOIN	--
ANOGA	--
TETNG	--
DROME	--
BRUMA	KKNLPSDDQQFTAMIGILNDMAFNLHLIPLDRLLISLVLHPTDDGATEIA
CAEEL	--
RHIO9	--
ARATH*	--
VITVI	--
SOYBN	--
SOLLC	--
ORYSJ*	--
HORVU	--
SORBI	--
SELML	--
PHYPA	--

910 920 930 940 950

Species	951–1000
HUMAN*	--
CHICK	--
XENTR	---S
DANRE	--
CIOIN	--
ANOGA	--
TETNG	--
DROME	--
BRUMA	MLIIHSLVGSYPDLHNRITALVHIIPSNKIGNSGAAFNKMSEYYSQFPPE
CAEEL	---E
RHIO9	---K
ARATH*	---T
VITVI	--
SOYBN	---P
SOLLC	--
ORYSJ*	---L
HORVU	--
SORBI	---L
SELML	--
PHYPA	--

960 970 980 990 1000

MED23

```
HUMAN*  ---------------METQLQSIFEEVVKTEVIEE---------------
CHICK   ------------MVPMETQLQSIFEEVVKTEVIEE---------------
XENTR   TLASPTSVRNYTLPEMESQLLSIFEEMVKTEIIEE---------------
DANRE   --------------------------------------------------
CIOIN   -------------MSVESQLEAVFGKALNTDIIVE---------------
ANOGA   -------------MSADLRIVKLFEEVVEDKNIVP---------------
TETNG   ---------------MEAQLQSIFEDVVKTEIIEE---------------
DROME   -------------MDMEAQVIDTVNEFLKVDSLDE---------------
BRUMA   LSYREMEAKMRREMQIELGMRPIEQSTVNPELHMPIYYGNIMERILPIVD
CAEEL   MDDTCSPQFVQEVNRIKSQIKSLVEEN-----------------------
RHIO9   PNTQSYSIWIITIQFMLSCLKSTPQNHITTPVILSVI-------------
ARATH*  VAATPSSSRSYQFHPARAAIIDLFNLY--LGRGSR---------------
VITVI   --------------------------------------------------
SOYBN   ATSTTTPSRSFQFHPLRVPIFDLFNLYLGLGRNSR---------------
SOLLC   --------------------------------------------------
ORYSJ*  PQQRQMQLHHHHHHPARSSIADLFTLY--LGMNSK---------------
HORVU   --------------------------------------------------
SORBI   PQQRQMQL---HHHPARPAIADLFTLY--LGMNSK---------------
SELML   --------------------------------------------------
PHYPA   --------------------------------------------------
                  1010      1020      1030      1040      1050
```

```
HUMAN*  --------------------------------------------------
CHICK   --------------------------------------------------
XENTR   --------------------------------------------------
DANRE   --------------------------------------------------
CIOIN   --------------------------------------------------
ANOGA   --------------------------------------------------
TETNG   --------------------------------------------------
DROME   --------------------------------------------------
BRUMA   IILLRAIETVVADQLFTTLLMCFKPCYRYHPQPAAYMYSVLYCLDKTISH
CAEEL   --------------------------------------------------
RHIO9   --------------------------------------------------
ARATH*  --------------------------------------------------
VITVI   --------------------------------------------------
SOYBN   --------------------------------------------------
SOLLC   --------------------------------------------------
ORYSJ*  --------------------------------------------------
HORVU   --------------------------------------------------
SORBI   --------------------------------------------------
SELML   --------------------------------------------------
PHYPA   --------------------------------------------------
                  1060      1070      1080      1090      1100
```

```
HUMAN*  --------------AFPGMFMDTPEDEKTKLISCLGAFRQFW---GGLSQ
CHICK   --------------AFPGMFMDTPEDERTKLISCLGAFRQFW---SSLSQ
XENTR   --------------AFAGMFMDTPEDERTKLISCLGAFRQYW---SVLPQ
DANRE   ------------------MFMDSPEDEKTKLTSCLGAFRQFW---STLPQ
CIOIN   --------------VFTGFVSLANLDHTKRLKTCIKDFRTLY---NSQST
ANOGA   --------------------NFDEEEQRKKTEEGYKKIGTLF---GAMAP
TETNG   --------------AFAGMFMDTPEDERTKLITCLGAFRQYW---VTLPL
DROME   --------------AFVSVIVFKPNTEQERATRFANDLVTAF---GNVAQ
BRUMA   TVRARDFFLRDGKYALLTPSFISDNHQLSLPSQFCQALVDRILQASNYAH
CAEEL   --------------HTRKFFRPLTSNIGDETAILRIQFNNMM---SKMEE
RHIO9   KQ------------VLLEMIRLPEKEMSPIEQKCFSVGKELL---DLFFI
ARATH*  --QKPDESLRDPPNKSQKRVHAPNRDLPPRNEQFLLDFELLQ---SQFND
VITVI   --------------------------------------------------
SOYBN   --QKPDDSLRDPPNKTQKRVHALNRELPPPNEQFILDFEQLQ---SQCAD
SOLLC   --------------------------------------------------
ORYSJ*  --QRVEDPTRETSNKLQKRVTAMNRDLPPRDEQFISDFEQLH---MQFPD
HORVU   --------------------------------------------------
SORBI   --QRAEDPLRESPNKLQKRVTALNRDLPPRDEQFISDYEQLR---MPFPD
SELML   --------------RTKQRKKASTKPHPP-NEQFQSDYLLLQ---NQFMD
PHYPA   ----------------RKRVAAVSRPQAPKEDQFSSDFLMLQ---SQFTV
                  1110      1120      1130      1140      1150
```

```
HUMAN*  ESHEQCIQWIVK---FIHGQHSPKRI-------SFLYDCLAMAVETGLLP
CHICK   ESHEQCVQWIVR---FIHSQHSPKRI-------SFLYDCLAMAVETGLLP
XENTR   ESHEQCVQWIVK---FIHGQHSPKRI-------SFLYDCLAMAVETGLLP
DANRE   ESHEQCVQWIVR---FIHGQHSPKRI-------AFLYDCLAMAVETSLLP
CIOIN   EEHVTSITLLIK---FIHKQQHPRRI-------SFMFDFLETCIQDGFLL
ANOGA   ESKEAAIRNYLG---YVASVNHQNRV-------HNYLQVMNRMVALNIIP
TETNG   ESHEQCVQWIVR---FIHSQHSPKRI-------SFLYDCLAMAVETSLLP
DROME   ENREQVLRLYL----LRAAGASGYHI-------KVLMAALVKLVDAHVIT
BRUMA   QPPAFVYKDWR----FAEMPPAGQAL-------TGACIELLASPHAPVIT
CAEEL   KEKQSLVKELIKMVHHVAEKNSPDRVFVGTNYERVVDQLLRYAHQKGAIS
RHIO9   RKEDGLVQSVGYGIISYY--------DVIYSFPELYGHFLTTVQSSTTTT
ARATH*  PEQLRTITESVLISLVVQCSNHAPRA-------EFLLFALRTLCRISYIN
VITVI   --------------------------------------------------
SOYBN   QDQLRSVTEAILISLVVQCSGHGPRA-------DFLLFVLRSLCGIGCIN
SOLLC   --------------------------------------------------
ORYSJ*  QEQLQAVTESVLISFVLQCSSHAPQS-------EFLLFATRCLCARGHLR
HORVU   --------------------------------------------------
SORBI   AEQLQAVTESVLISFVLQCSSHAPQS-------EFLLFATRCLCARGHLR
SELML   HNQLQAITESLVAMLFIQCGSHAPQA-------DFLLFALRTLARAGLLR
PHYPA   S--------------------------------DFLLSCLQILVKMGFVR
                  1160      1170      1180      1190      1200
```

MED23

**HUMAN***	PRLVCESLINSDTLEWERTQLWALTFKLV---------------------
CHICK	PRMVCESLINSDTLEWERTQLWALTFKLI---------------------
XENTR	PRMVCESLINSDSLEWERTQLWILTFKLI---------------------
DANRE	PRMVCQALISSDSLEWERTQLWALTFKLI---------------------
CIOIN	PRPVLDQLQGAE-LVHTNRLHWCRTYEVI---------------------
ANOGA	AKLLCDQLMCSEKLVYKNPCFWIEAFRLI---------------------
TETNG	PRMVCAALISSDSLEWERTQLWALTFKLI---------------------
DROME	ARMLCDKVLMCEKLDFEHRTFWIESFRLI---------------------
BRUMA	AHALIDLVF----IRPLHQPFVF---------------------------
CAEEL	TNLCAEGLIMTSDFRLCSR-ICQEKWKFI---------------------
RHIO9	ASAASTSTTNQPSVTNSNIWLMTLTYMTISILQD----------------
**ARATH***	WDTFLPSLLSSVSAAEASLSQGVQAAAATAGSSATSSQSVVPVSV-----
VITVI	--
SOYBN	WDSLLPSLLSSVSSAELPVGQLSQAVPTVSSSSLSQTGMLPPPSTIANSS
SOLLC	--
ORYSJ*	WDSLLPSLLNVVSSVEVPMGQGV---SVTTGGPATSSSSAI---AVPNAP
HORVU	--
SORBI	WDSLLPSLLNTVSSIEAPMVQGV---SVTGGGPAT-PSSAI---TMPNAP
SELML	SDMFLLAFFRAVAMVESN-----------------------PVAVTQAQS
PHYPA	WDSFLHILLRAAEKAE-NIGQATGSHQGMNVGVTGPQLTGPLAMMMQGTT
	1210 1220 1230 1240 1250

**HUMAN***	--
CHICK	--
XENTR	--
DANRE	--
CIOIN	--
ANOGA	--
TETNG	--
DROME	--
BRUMA	--
CAEEL	--
RHIO9	--
**ARATH***	-----NPTSLLPSAHGIGSPSASEVKSVE-------NGQQIARAGQIVRE
VITVI	--
SOYBN	NFQSSNPASPLTSVHTIGSPAQSTMEPLSCAAMSPVKSSDISSAGQ---Q
SOLLC	--
ORYSJ*	SFHPSNPTSPLSAMNTIGSPTQSGIDQPIGANVSPIKGAEFSSPGQL--G
HORVU	--
SORBI	NFHPSNPASPLSVMNTIGSPTQSGIDQPVGANVSPIKAAEFSSSAQL--G
SELML	SPLVST--SGEPSTNILVTPSSAKRAS-----------------------
PHYPA	ATPASTPASPSPSSNLLTSSAHGNVAA---------SPAHSGVEYSSVNP
	1260 1270 1280 1290 1300

**HUMAN***	-------------------RKIIGGVDYKGVRDLLKVILE-KILTIPN--
CHICK	-------------------RKIIGGVDYKGVRDLLKVILE-KILTIPN--
XENTR	-------------------RKIIGGVDYKGVRDLLKVILE-KIQTVPD--
DANRE	-------------------RKIIGGVDYKGVRDLLKAVLD-KIQTIPN--
CIOIN	-------------------RKFVHMVDYKGCRNLLHSMVL-KMTQIPQ--
ANOGA	-------------------RKVIGGVDYKGVREIMKCCKE-KALSFPA--
TETNG	-------------------RKIIGGVDYKGVRDLLKAVLD-KIQTIPT--
DROME	-------------------KRVIVQVDYKGVREIMKVCRD-KAQWFPL--
BRUMA	--------------------LIIYATINAVALILTA---------LPV--
CAEEL	-------------------NDCIPKIDYKGIRNILRYILESQLRRLPY--
RHIO9	-------------------HKLLERIPTLMAINIIKHVLK-EINTLPP--
**ARATH***	NAMRNSQRIRAAAV--NSLRQLSCKIILIGVESSLKPVTHAEIFQYMMNW
VITVI	--
SOYBN	SKLRGSPSVRTNDISNSSLRQLCCKIILTGLEFSLKPVTYAEIFNYMLNW
SOLLC	--MFKLGW
ORYSJ*	LTARGDQSRRGAEI--SYLHHLSCRIILAGLESDLKPATHAVIFQHMVNW
HORVU	--
SORBI	TAARGDQSRRGAEA--SYLHHLSCRIILAGLEFNLKPATHAVIFQHMVNW
SELML	----------------VWLRPLVCNVMGAALEAELRPVASFDMLYLTVQW
PHYPA	SSNGGTGASGTSII--NSLRPLVCRVMLAALDNELKPVTCLDILLHVAQW
	1310 1320 1330 1340 1350

**HUMAN***	----------------------TVSSAVVQQLLAAREVIAYIL----ERN
CHICK	----------------------TVSSAVVQQLLAAREVVAYIL----ERN
XENTR	----------------------TVSSAVVQQLLAAREVVAYIL----ERN
DANRE	----------------------FVSSAVVQQLLAAREVVEYIL----DRN
CIOIN	----------------------SVWIEEKQQVDAICSTIQLLL----DPE
ANOGA	----------------------SVSVNILPQLLELVELIEHIF----NRN
TETNG	----------------------TVSSSIVQQLLAAREVVEYIL----DRN
DROME	----------------------NVNVTYMPQLLAVEDILRFIF----DRN
BRUMA	----------------------SFQQVFYDHIVSVLDSEALHG----DPS
CAEEL	----------------------SLSPEQVNEIRIVENVILHIV----DRD
RHIO9	----------------------NLTTTQVDCINKGKELINLLLLGSSGDG
**ARATH***	LVNWDRRDLGTEDS-V--GKSWRSEKTLAEWLRSCLDVIWLLV----EEG
VITVI	--
SOYBN	LVNWDQRQQGIDESDV--IKSWRPDKAVIAWLHSCLDVIWLLV----DEG
SOLLC	--
ORYSJ*	LVNWDQRPHGVDQADALQLQTLRLERPLHEWMHLCLDVIWILV----NED
HORVU	--
SORBI	LVNWDQRPHGMDEADA--MQTCRLEKPLHEWMHLCLDVIWILV----NED
SELML	INDVASGSEG------------SLKD--VTWLDNCLEVLRMLI----DER
PHYPA	IQTWDAEVDGKEDESKSRLNSWKLKS--KMWIHSCVEVIWALI----DEN
	1360 1370 1380 1390 1400

MED23

```
HUMAN*   ACLLPAYFAVTEIRKLY--------PEGKL-PHWLLGNLVSDFVDTFRPT
CHICK    ACLLPAYFAVTEIRKLY--------PEGKL-PHWLLGNLVSDFVDTFRPT
XENTR    ACLLPAYLAVTEVLKLY--------PEGKL-PHWLLGDLVSDFVDSFRPT
DANRE    ACLLPAYFAVTEIRKLC--------PEGAL-SHWLLGSLISDFVDSFRPT
CIOIN    ACLLPGYLAVNEIRQNM--------QDDLLDAPWPLLGKLTEFIDRFRAT
ANOGA    ACLLPAYFIINEIKKTD--------YQN---MHWRVSNLTANFIEEFVSV
TETNG    ACLLPAYFAVTEIRKLY--------PEGKL-SHWLLGSLISDFVDSFRPT
DROME    NCLLPAYFIANEIMRPF-------------PYHWKLNRLMTDFVEEFRTT
BRUMA    VCFGS---LESECL------------------------LLTE--------
CAEEL    SNLMPPLITLSEIMRGM--------PKQAL-MLPRLTEKLASLSVHFRPI
RHIO9    AGVVSHYDIILALPDVYGHFLGGTFPQS---IHWTMEKFINELGKRRRHL
ARATH*   ESRIPFYELLRSGLQF----------IENIPDDEALFTLIMEIHRRRDAM
VITVI    --------------------------------------------------
SOYBN    KCRVPFYELLRSDLQF----------IENIPDDEALFTLILEIHRRRDMM
SOLLC    --------LERGILQF----------I-----------------------
ORYSJ*   KCRVPFYELVRSNLQF----------LENIPDDEALVSIIMEIHRRRDMV
HORVU    --------------------------------------------------
SORBI    KCRIPFYELVRCNLQF----------LENIPDDEALVSIIMEIHRRRDMV
SELML    KCRVPFYALLHDQTLLQ---------ADYWPENEALLGLFLDLHRRRDRI
PHYPA    KCRVPFYALLHDKTQLQ---------VERWPDDEALFALFLEVHRRRDKI
                  1410      1420      1430      1440      1450
```

```
HUMAN*   ARINSIC---GRCSLLPVVNNSG---AICNSWK---LDPATLRF------
CHICK    ARINSIC---GRCSLLPVVNNSG---AMCNSWK---LDPTTLRF------
XENTR    ARISSICAFAGRCSLLPVVNNSG---AICNSWK---LDPLTLRF------
DANRE    ARINSIC---GRCSLLPVVNNSG---AICNSWK---LDPTTLRF------
CIOIN    ATMVSTT---ARWMLLPVVDHAFY--QLPSTWK---LEADTLRF------
ANOGA    AQMLSII---GHSAMLPIVEHSSYADNLIIPWK---LDPDTLKL------
TETNG    ARINSIC---GRCSLLPVVNNSG---AICNSWK---LDPSTLHF------
DROME    AQMVSII---GHASMLPIVEHFGYADHMMNSWR---LDHNTLKF------
BRUMA    -------------------------NQLLTNLA---LGHAYLQH------
CAEEL    ADLSHVC---GRGFVYPIPLHPSFY-PLTSCWEEHGLNASNTIV------
RHIO9    AFLMMPPD--ASWPLISQMSRRHEYNDLTTRTSYHMFEGDLIEF------
ARATH*   AMHMLMLDQHLHCPSFGTHRIVSQITANVPPEAVPHLRHSPITYPSVLGE
VITVI    --------------------------------------------------
SOYBN    AMHMQMLDQHLHCPTFGTHRILNQTIPNVSGEAVAHLRLSPITYLSVLGE
SOLLC    --------------------------------------------------
ORYSJ*   CMHMQMLDQHLHCPTFATHRFLSQSYPSIAGESVANLRYSPITYPSVLGE
HORVU    --------------------------------------------------
SORBI    CMHMQMLDQHLHCPTFGTHRFLSQSYPSIAGESVTNLRYSPITYPSVLGE
SELML    ALHMLMFDHHLQCPTFST------------------LRSTALTYAGAMGE
PHYPA    ALHMLPLDQHLHCPTFAT------------------LRVSSLTYPGTMGE
                  1460      1470      1480      1490      1500
```

```
HUMAN*   -----PLKGLLPYDKDLFEPQ-TALLRYVLEQPYSRDMVCNMLGLNK---
CHICK    -----PLKGLLPYDKDLFEPQ-TALLRYVLEQPYSRDMVCNMLGLNKQAF
XENTR    -----PLKGLLPYDKDLFEPQ-TALLRYVLEQPYSRDMVCNMLGLNK---
DANRE    -----PLRGMLPYDKDLFDPQ-TGLLRYVLEQPYSRDMVCNMLGLNK---
CIOIN    -----QLKKQLPYRPEMYQRQ-HRLLNYILDQTYSRDMVLQVLGMQK---
ANOGA    -----SLRGNLPYDEELLQPQ-ARLLRFVLQQPYSRDMVCSMLNFQK---
TETNG    -----PLKGMLPFDKELFEPQ-TGLLRYVLEQPYSREMVCNMLGLNK---
DROME    -----NFKGSLPYEPELLEEQ-KPLLRYVLEQPYSREMVSQMLNLQK---
BRUMA    -----CNTSSLAALPEFVRDQLAPKLVTEAQLIFVLRLVVPILQRFY---
CAEEL    -----QPHHTLPYRPEHTSTY-LYTLYMILRQPLGKD------SLHP---
RHIO9    ---------VKEGNSTNTETH----FRDMLYQRASMDEFRQLAMSLT---
ARATH*   PLYGEDLAMSIPKGSLDWERA-VRCIRHAIRTTPSPDWWKRVLVVAP---
VITVI    ---MEDLANSIQRGSLDWERA-LRCIRHALRTTPSPDWWKRVLLVAP---
SOYBN    PLHGEDIASSIQKGSLDWERA-VRCIRHALRTTPSPDWWRRVLVLAP---
SOLLC    --------------STEYYFA-----------SPSPSLLRFLMI------
ORYSJ*   PLHGEDLANSIPKGGLDWERA-LRCLRHALRTTPSPDWWRRVLLVAP---
HORVU    --------------------------------------------------
SORBI    PLHGEDLANSIPKGGLDWERA-LRCLRHALRTTPSPDWWRRVLLVAP---
SELML    ALYGEEVAASVQRDSLDYEKA-LRCLKHGLRTNPSADIWRRVLVCAP---
PHYPA    LLHGEDVATAIPKGSVEWEKA-MRCLRQALRACVSTDWWKKVLVLAP---
                  1510      1520      1530      1540      1550
```

```
HUMAN*   ---------QHKQRCPV----------------------LEDQLVD-LVV
CHICK    GILYLISALQHKQRCPV----------------------LEDQLVD-LVV
XENTR    ---------QHKQRCPV----------------------LEDQLVD-LVV
DANRE    ---------QHKQRCPV----------------------LEDQLVD-LVV
CIOIN    ---------HQKQRCEA----------------------LEMGLVD-LMF
ANOGA    ---------QQKQKCAA----------------------LEEQLVW-LVL
TETNG    ---------QQKQRCPV----------------------LEEQLVD-LVV
DROME    ---------HQKQRYNA----------------------LEEQLVN-LIV
BRUMA    ---------DAKER--------------------------SKQIQD-LAV
CAEEL    ---------PSKTKTKT----------------------NWEMLISVMIC
RHIO9    ---------QTGLDTTQ----------------------IRKAMVS-KIR
ARATH*   ---------CYRPSTQA----------GPIPGAVFTSDMICEAIID-RIV
VITVI    ---------CYRSHPQ-----------GPSAGAVFTSEMICEATID-RIV
SOYBN    ---------CYRNSSQ-----------GPTAGAVFSSEMICEATID-RIV
SOLLC    ----------------------------SIVGFIF---------------
ORYSJ*   ---------CYRQHPQQ----------SSTPGAVFSPDMIGEAVAD-RTI
HORVU    --------------------------------------------------
SORBI    ---------CYRSQSQQ----------SSTPGAVFSPDMIGEAVAD-RTI
SELML    ---------RHRQQQPVPPRPGPNTSWTQPGLVFNSEMTCEAVVE-RIM
PHYPA    ---------RFKKAPQSMGPRTG-SLFVGQPSLEFSADMTCDAVVE-RIM
                  1560      1570      1580      1590      1600
```

MED23

HUMAN*	YAMERSETEEKF---------------DD--GGTS--------------Q
CHICK	YAMERSETEEKF---------------DD--GGTS--------------Q
XENTR	YAMERSETEEKF---------------DD--GGTS--------------Q
DANRE	YAMERSETEEQF---------------DD--GGTS--------------Q
CIOIN	STVKKCSTFEDK-------------------SNIQ--------------H
ANOGA	NAMECSEKEAAQGGQGAAGGTGMNEHEGD--QSHT--------------Q
TETNG	YAMERSESEEHF---------------DADVGGTS--------------Q
DROME	QAMEMTEANDAT------AGSGFNSSDEQ--ITPY--------------E
BRUMA	DVYKMTVKVNER------VG-----------LLKY--------------E
CAEEL	ESMAEAESLPET-------------------EPIP--------------R
RHIO9	DVIRCIQQHSKKGKREDRDEKAFEPFNPSLTTTIRGEDIKMMIPDNIHLW
ARATH*	ELLKLTNSGNDCFGIDLV--SVTFPPLYA--DANC--------------W
VITVI	ELLKLTNS-----------------------DINC--------------W
SOYBN	ELLKMTNS-----------------------EINC--------------W
SOLLC	--
ORYSJ*	ELLRLTNS-----------------------ETQC--------------W
HORVU	--
SORBI	ELLRLTNS-----------------------ETQC--------------W
SELML	DLMQPISTGRGFYHIFSF--PAFSATSTT--ESGR--------------W
PHYPA	ELMQPLPTGYAF--------------------TCR--------------W
	1610 1620 1630 1640 1650

HUMAN*	LLWQHLSSQLIFFVLFQFAS---------FPHMVLSLHQKLAGRGLIK--
CHICK	LLWQHLSSQLIFFVLFQFAS---------FPHMVLSLHQKLAGRGLIK--
XENTR	LLWQHLSSQLIFFVLFQFAS---------FPHMVLSLHQKLAGRGLIK--
DANRE	LLWQHLSSQLIFFVLFQFAS---------FPHMVLSLHQKLAGRGLIK--
CIOIN	LMWQHVACQVIYFVLYQLAS---------FPHIVEDLHTKLAASEELRDQ
ANOGA	WIWLHLSSQLIYFVLFQFAT---------FQNIVNTLHEKLAVRNLRR--
TETNG	LLWQHLSSQLIFFVLFQFAS---------FPHMVLSLHQKLAGRGLNK--
DROME	WMWLHLSSQLIYFVLFQFVS---------FMHIVLALHEKLSKLELRK--
BRUMA	DSICDFLYHMKYMYVGDFVK-----------------------NEAEQ--
CAEEL	YQWDNIVNIVIYGITQHLLV---------PKTFFNVLKNLIKRCKYTR--
RHIO9	ELMTETADQLYSFVSGECSSTCLLFLLPNYVKDIEKVSTNVIQKDFEQ--
ARATH*	QEWLVFSDIFFFLIKSGCTD---------FVDFIDKLVLRLNGVDNHIL-
VITVI	QEWLVFSDIFFFLMKNGCID---------FVDFVDKLILRLIEGDNHIL-
SOYBN	QDWLVFSDIFYFLIKSGCID---------FVDFVDKLVSRLTEGDHHIL-
SOLLC	-----------------CI-------------------------------
ORYSJ*	QDWLLFADIFFFLMKSGCID---------FLDFVDKLASRVTNSDQQIL-
HORVU	--
SORBI	QDWLLFADIFFFLMKSGCID---------FLDFVDKLASRVTNSDQQIL-
SELML	QEWLNFADLFYYFMRRGYLD---------FLEFIEKLAGAFASGEQSIQ-
PHYPA	QEWLMFADLFYFFMSTSCLD---------FLELIDKLSLRFANG--TVM-
	1660 1670 1680 1690 1700

HUMAN*	----GRDHL---MWVLLQFISGSIQKNALA------DFLPVMKLFDLLYP
CHICK	----GRDHL---MWVLLQFISGSIQKNALA------DFLPVMKLFDLLYP
XENTR	----GRDHL---MWVLLQFISGSIQKNALA------DFLPVMKLFDLLYP
DANRE	----GRDHL---MWVLLQFISGSIQKNALG------DFLPVMKLFDLLYP
CIOIN	CIQGGRDYI---MWVLLQFISGSIQKNPLS------DFLSVIKLVNLLYP
ANOGA	----GRDHL---MWVFLQYISGSIQKHSIL------NFLPILKLYDILYP
TETNG	----GRDHL---MWVLLQFISGSIQKNALA------DFLPVMKLFDLLYP
DROME	----GRDQL---MWILLQFISGSIQKNPIT------NFLPVFRLFDLLYP
BRUMA	----AIQRLSPSMRNKLKYISHTQMNTAI----------------DLPHK
CAEEL	----ARDEV---MWIVFQVVGSLSNLTRLD-----DAVTEIVELYNELF-
RHIO9	---------------------------------DERLFDKLVKLYNDEQT
ARATH*	----RTNHV---TWLLAQIIRVELVMTALN--SDAKKVETTRKILSFHRE
VITVI	----RTNHV---TWLLAQIIRVELVMNALT--SDPRKMETTRKILSFHKE
SOYBN	----KTNHV---TWLLAQIIRIELVMNALN--SDPRKVETTRKILSFHRE
SOLLC	--
ORYSJ*	----RSNHV---TWLLAQIIRIEIVMNTLS--SDPRKVETTRKIISFHKE
HORVU	--
SORBI	----RSNHV---TWLLAQIIRIEIVMNTLS--SDPRKVETTRKIISFHKE
SELML	----RSNHV---TWLLAQVFRLETVTQALSEDEDSRKVETAQKILSFHMA
PHYPA	----ASNHV---TWLLAQVFRLEIVTSALS--SEILQVETARKILSFNMA
	1710 1720 1730 1740 1750

HUMAN*	EKEYIPVPDINKPQSTHAF-AMTCIWIHLNRKAQ-------NDNSKLQIP
CHICK	EKECIPVPDINKPQSTHAF-AMTCIWIHLNRKAH-------SDNSKLQIP
XENTR	EKECIPVPDITKPQSTHSF-AMTCIWIHLNRKAQ-------NDNSKLQIP
DANRE	EKECIPVPDINKPQSTHAF-AMTCIWIHLNRKAQ-------NDNSKLQIP
CIOIN	EKQPLELPEMKSHSCTISF-AAVCNLVHLNRKAQAKPPSDGNQDRNQQWN
ANOGA	EKEPLPVPDNNNPNCTHQM-APTCIWIHLMKRAQ-------TENYNINRP
TETNG	EKECIPVPDITKPQSTHSF-AMTCIWIHLNRKAQ-------NDNSKLQIP
DROME	ELEPLKLPDINKSSMVRHM-APICVWIHLMKKAR-------VENMNITRP
BRUMA	LAPGPSSLFINKSSMVRHM-APICVWIHLMKKAR-------VENMNITRP
CAEEL	DGDVVWMGASDHPALFARFLAAAGTWMILE-----------KDFADKMPP
RHIO9	ESK--------DAFTLRDL-SLQCA----------------INHQKAHIK
ARATH*	DRNSDPNN---PQSVLLDF-VSSCQNLRIWSLSTTTRA---YLNN----E
VITVI	DRSSDPNN---PQSILLDF-ISSCQNLRIWSLNTSTRE---YLNN----E
SOYBN	DRSSDPNN---PQSILLDF-VSSCQNLRIWSLNSSTRE---YLNN----E
SOLLC	--
ORYSJ*	DKSLDPNN-ISPQSILLDF-ISSSQTLRIWSFNTSIRE---HLNS----D
HORVU	--
SORBI	DKSLDANN-IGPQSILLDF-ISSSQTLRIWSFNTSIRE---HLNS----D
SELML	DRSVDHSNAVSPQAKLLDY-VGSSQILRLWALNRTVME---QLVGNRMPE
PHYPA	ERSVEQTSNTTAQALLLDF-VGSSQTLRLWSINRNMME---NLHGNRVQE
	1760 1770 1780 1790 1800

MED23

```
HUMAN*   IPHSLRLHHEFLQQSLR--------------------------NKSLQM-
CHICK    IPHSLKHHHEFLQQSLR--------------------------NKSLQM-
XENTR    IPHSLKLHHEFLQQSLR--------------------------NKSLQM-
DANRE    IPHSLKLHHEFLQQSLR--------------------------NKSLPM-
CIOIN    IPVALHNQHECIKQCLK--------------------------TTSINL-
ANOGA    VPIALRLHFEYLQHLAMPSN-----------------------NPTLFMG
TETNG    IPHSLKLHHEFLQQSLR--------------------------NKTLGM-
DROME    LPIALKNHYDFLQHLVTANT-----------------------MMNMTLG
BRUMA    LPIALKNHYDFLQHLVTANT-----------------------MMNMTLG
CAEEL    ANETIKSHIKFIQDGVD----------------------------NFDSS
RHIO9    DVTNIKQRHPLIAASLQYAGLCYQIQAYFNNHYHSNVPTNSTLFSNLDLQ
ARATH*   QLLKGKQIDEWWR--SK--G------------------ERMMDYMNMDDR
VITVI    QLQKGKQIDEWWRLANK--G------------------ERMMDYVTLDDR
SOYBN    QLQKGKQIDEWWRQASK--G------------------ERMMDYMNMDER
SOLLC    ---------------KS--G------------------ERMMDYMNLDDR
ORYSJ*   QLQKGKQIDEWWRQMTKASG------------------ERMIDFTSLDER
HORVU    --------------------------------------------------
SORBI    QLQKGKQIDEWWKQMTKASG------------------ERMIDFTNLDER
SELML    HIQKGKTIDEWWKKVLK--G------------------ERFLDYANLDDN
PHYPA    HMQKGKVIDEWWKQVMK--G------------------EWVLDYAQLDEK
                   1810      1820      1830      1840      1850
```

```
HUMAN*   NDYKIALLCNAYSTNSEC----FTLPMGALVETIYG-NGIMRIPLP----
CHICK    NDYKIALLCNAYSTNSEC----FTLPMGVLVETIYG-NGNMRISLP----
XENTR    TDYKIALLCNAYSTNSEC----FTLPMGVLVETIYG-NGNVNIPLP----
DANRE    TDYKIALLCNAYSTNSEC----FTLPMGVLVETIYG-NGSMRITLP----
CIOIN    KDSQVALISNACSSNTEM----FNMAVSVLVESVFG-DTSSTSPMP----
ANOGA    SEYRFALLCNAYSTQTDY----FSRPMQALIDTILG-NSKN----P----
TETNG    SDYKIALLCNAYSTNSEC----FPLPMGALVETIYG-NGTMRINLP----
DROME    NDFRIILICNAYSTNQEY----FGRPMSLLLDAL---NGTSKSPNG----
BRUMA    NDFRIILICNAYSTNQEY----FGRPMGLLLDAL---NGTSKSPNG----
CAEEL    NTAMLAVLSNIYRTDTKM----GKLIVPTLQQMLESIDDTKSLFEL----
RHIO9    EMTKIAMESQLRQDVVPYTLYVYLVPTKADIDTL----------------
ARATH*   SIGMFWVVSYTMAQ-----------PACETVINWLSSAGMAEL-PG--LQ
VITVI    SIGMFWVMSYTMAQ-----------PACDTVMNWFSSAGAAELIPGSHLQ
SOYBN    SIGMFWVVTYTMAQ-----------PACETVMNWLNSAGVADLLPGANLQ
SOLLC    SIGMFWVVSYTMAQ-----------PACETVMNWLTSAGVTEHLPGPNLQ
ORYSJ*   AMGMFWVLSFTMAQ-----------PACEAVMNWFTSVGVADLIQGPNLQ
HORVU    --------------------------------------------------
SORBI    ATGMFWVLSFTMAQ-----------PACEAVMNWFTSAGMADLIQGPNMQ
SELML    SMGMVWVLSHTMTQ-----------PVADAVMNWMRSNGVGEVLVG----
PHYPA    AMGMVWVLSNTMTQ-----------PISEALMIWFRSSGLSEIILQ----
                   1860      1870      1880      1890      1900
```

```
HUMAN*   -GTNCMASGS---ITPLPMNLLDSLTVHAKMSLIHSIATRVI------KL
CHICK    -GTNCMASGS---ITPLPMNLLDSLTVHAKMSLIHSIATRVI------KL
XENTR    -GSNCMASGS---ITPLPMNLLDSLTVHAKMSLIHSIATRVI------KM
DANRE    -GTNCMASGS---VTPLPMNLLDSLTVHAKMSLIHSIATRVI------KL
CIOIN    -GNNCVAFNK---TVPCSMDLLDTLTVHTKMSMLHSISTKIQ------KF
ANOGA    -QANMVGGGQPLPTAPLSMRVLDSLTIHSKMSLIHSIVMHMI------KQ
TETNG    -GTNCMASAA---VTPLPMNLLDSLTVHAKMSLIHSIATRVI------KL
DROME    -GQ--------IPAVTFSVTVLDSLTVHSKMSLIHSFVTQML------KQ
BRUMA    -GQ--------IPAVTFSVTVLDSLTVHSKMSLIHSFVTQML------KQ
CAEEL    -SYKRMAVNC---FSAFPVEFMEALTFRSKKTLLIQCFQPLR--------
RHIO9    -GHSSTTYIK---GGTLDYRLLDLLCVNAKHRLLQVIYKMMLDSELGPRY
ARATH*   PNDRVMMTQE---VTPLPMSLLSGFSMNLCLKLALQMEEALF--------
VITVI    SNERVMVMKE---ISPLPMSLLSGFSLHLCMKLAFQMEDSLF--------
SOYBN    QAERLMATRE---VSPLPMSLLSGFSINLCVKLSYQMEDSLF--------
SOLLC    SNERLMVMRE---VCPLPISLLSGLSINLCLKVAFQLEESMF--------
ORYSJ*   PNERMTMMRE---TYPLSMSLLSGLSINLCLKLAFQLEETIF--------
HORVU    --------------------------------------------------
SORBI    PSERIMMMRE---TYPLSMSLLSGLSINLCLKLAFQLEETIF--------
SELML    -NERIAVVHE---TSPLPIAMLSGLSLHLCMRLIQQIEEQIF--------
PHYPA    -GERLSVIHE---TRPLPMTLLSGLSLHLCLRLLTQIEELIF--------
                   1910      1920      1930      1940      1950
```

```
HUMAN*   AHAKSSVA----LAPALVETYSRLLVYMEIE-SLGIKGF-----------
CHICK    AHAKSSVA----LAPALVETYSRLLVYMEIE-SLGIKGF-----------
XENTR    AQAKSTFA----LAPALVETYSRLLVYMEIE-SLGIKGF-----------
DANRE    AHAKSSLA----LAPALVETYSRLLVYMEIE-SLGIKGF-----------
CIOIN    AQAKSSVG----LAPALVETYSRLLIYYEIE-TLGIKIL-----------
ANOGA    AQNKSSIPNGNNMAPALVETYSRLLVYTEIE-SLGIKGF-----------
TETNG    AHAKSSIA----LAPALVETYSRLLVYMEIE-SLGIKGF-----------
DROME    AQSKGQVP-----AAALLETYARLLVYTEIE-SLGIKGF-----------
BRUMA    AQTKGQVP-----AAALLETYARLLVYTEIE-SLGIKGF-----------
CAEEL    SFSTVRLP-----SPAVFETVAKICESEDY--EMAVKDM-----------
RHIO9    QSPASPVA---CVPPNVLDVVYKLLYSAPCSAELMVKEIFDKLRRCDKMI
ARATH*   --VSQVVP-----SIAMVETYTRLLLISPH--SMFRSHF-----------
VITVI    --SGQVVP-----SIALVETYTRLLLIAPH--SLFRSHF-----------
SOYBN    --SGQVIP-----SIAMVETYTRLLLLAPH--SLFRSHF-----------
SOLLC    --SGQAVP-----SIAMVETYCRLMLISPH--SLFRSLL-----------
ORYSJ*   --LGQNVP-----SIAIVETYVRLLLITPH--SLFRPHF-----------
HORVU    --------------------------------------------------
SORBI    --LGQAVP-----SIAMVETYVRLLLIAPH--SLFRPHF-----------
SELML    --AGQMVP-----SIAMLETYVRLLLVAPQ--TLFQHHL-----------
PHYPA    --AGQFIP-----SIAMVETYVRLLLAAPQ--NLFRHHL-----------
                   1960      1970      1980      1990      2000
```

MED23

```
HUMAN*  ------ISQLLPTVFKS-----------HAWGILHTLLEMFSYRMHHIQP
CHICK   ------ISQLLPTVFKS-----------HAWGILHTLLEMFSYRMHHIQP
XENTR   ------ISQLLPTVFKS-----------HAWGILHTLLEMFSYRMHHIQP
DANRE   ------ISQLLPNVFKS-----------HAWGILHTLLEMFSYRMHHIQP
CIOIN   ------VSQVVPKVYEY-----------RAWGILNTLLELMSYRMHHIQP
ANOGA   ------LGQLLPQVFKS-----------QAWGILYTLLEMFSYRMHHHHS
TETNG   ------ISQLLPNVFKC-----------HAWGILHTLLEMFSYRMHHIQP
DROME   ------LSQLMPTVFKN-----------HAWAMLHTLMEMFSYRLHHVPT
BRUMA   ------LSQLMPTVFKN-----------HAWAMLHTLMEMFSYRLHHVPT
CAEEL   ----EHLAQRSLHVATAADRSSGEHQNVQAKDQCYFLFDFLVYRLPHLHA
RHIO9   KMKRTGESEPSPTQLPD-----------QTMRWLQTILQLMNYRFIRFLK
ARATH*  ----SQLAQRNASLLSK-----------PGVTLL--VLEILNYRLLPLYR
VITVI   ----SHLSQRYPAILSK-----------PGATLL--VLEILNYRLLPLYR
SOYBN   ----NHLVQRNPSLLSK-----------PGVTLL--VLEILNYRLLPLYR
SOLLC   ----THLTSRNPTTLTK-----------PGNTIL--VFEILNYRFLSLYR
ORYSJ*  ----TTLTQRSPSILSK-----------SGVSLL--LLEILNYRLLPLYR
HORVU   --------------------------------------------------
SORBI   ----TTLTQRSPSILSK-----------SGVSLL--LLEILNYRLLPLYR
SELML   ----NGMMQKYQSGAAK-----------AGISLI--LLELLNYRLMPLYR
PHYPA   ----NGMAHRFQSGMTK-----------PAVSLL--LLELFNYRLLPLYR
                 2010      2020      2030      2040      2050
```

```
HUMAN*  HYRVQLLSHLHTLAAVAQT-----NQNQLHLCVESTALR---------LI
CHICK   HYRVQLLSHLHSLAAVPQR-----NQNQLHLCVESTALR---------LI
XENTR   HYRVQLLSHLHSLAGVPQT-----NQNQLHLCVESTALR---------LI
DANRE   HYRVQLLSHLHSLAAVPQT-----NQNQLHLCVESTALR---------LI
CIOIN   NYRIQLLTHLHTLANSPHA-----NQRQLHLRLESTALK---------LI
ANOGA   HYRVQLLTHLHSLASVPHT-----NQMQLHSCVESTALR---------LI
TETNG   HYRVQLLSHLHSLAAVPQT-----NQNQLHLCVESTALR---------LI
DROME   HYRVQLLSLLHSLSSVPQT-----NKMQLNLCFESTALR---------LI
BRUMA   HYRVQLLSLLHSLSSVPQT-----NKMQLNLCFESTALR---------LI
CAEEL   -YS-KYSSTVNALSYFTQHIPNNPQNHQIYRLMEQFLMRRWCWAGFHGCI
RHIO9   -YSPLSSGLLHYIRYSISFL----DNRQCYRSLESFTVN---------VI
ARATH*  -YQGKSKTLMYDVTKIISALKGKRGDHRIFRLAENLCMN---------LI
VITVI   -YQGKGKTLMYDVTKIVSALKGKRGDHRAFRLAENLCMN---------LI
SOYBN   -YQGKSKALMYDVTKIISAIKGKRGDHRVFRLAENLCLN---------LI
SOLLC   -YQGKSKTLMYDVTKMISTLKGKRGDHRIFRLAENLCMN---------LI
ORYSJ*  -YHGKSKALMYDVTKIISMIKVKRGEHRLFRLAENLCMN---------LI
HORVU   --------------------------------------------------
SORBI   -YHGKSKALMYDVTKIISMIKGKRGEHRLFRLAENLCMN---------LI
SELML   -FHGKLKQLVFDMAKIIIPMKAKRGDHRLFRLAENLGIN---------LV
PHYPA   -FHNKLKQLIFDIAKIIITVKVKRGEHRLFRLAENLGIN---------LI
                 2060      2070      2080      2090      2100
```

```
HUMAN*  TA----LGSSEVQPQFTRFLSDPK------TVLSAESEELNRALILTLAR
CHICK   TA----LGSSEVQPQFTRFLSDPK------TVLSAESEELNRALILTLAR
XENTR   TA----LGSSEVQPQFTRFLSDPK------TVLSAESEELNRALILTIAR
DANRE   TA----LGSSEVQPQFTRFLNDPK------TVLSAESEELNRALILTLAR
CIOIN   MG----FDNFGIKPQLVRMFNEQKQ-----SIVSQKSEELNRVLVLTLAR
ANOGA   IG----LGSVEVQAQLSRYVSEPKP---PGNIVSGESEELNRALILTLAR
TETNG   TA----LGSSEVQPQFTRFLSDPK------TVLSAESEELNRALILTLAR
DROME   TS----IGSAEFQPQFSRYFNDKS----PGAVASNESEELNRVLILTLAR
BRUMA   TS----IGSAEFQPQFSRYFNDKS----PGAVASNESEELNRVLILTLAR
CAEEL   TAHTQMFGTSYKDNTMMRHMAYPKTFSVPDQYPFAINPEIFKMAIYSFLR
RHIO9   NM--------QMDVKLLRSLDDPLR---EKAIWFGESEMLARLTVGTISR
ARATH*  LS-------------LRDFFSVKRE---GKGP-TEFTETLNRITIMTLAI
VITVI   LS-------------LRDPFQVKKE---GKGP-TEFTETLNRITIITLAI
SOYBN   FS-------------LRDFFLVKRE---GKGP-TEFTETLNRVTVITLAI
SOLLC   LS-------------LRDFFFVKRE---GKGP-TEFTETLNRITIVTLAI
ORYSJ*  LS-------------LRDFFLVKKE---LKGP-TEFTETLNRITIISLAI
HORVU   --------------------------------------------------
SORBI   LS-------------LKDFFFVKKE---LKGP-TEFTETLNRITIISLAI
SELML   LS-------------MKEVLLIKKE----KGATTEFTETLNRIMVVSLAI
PHYPA   LN-------------MKDVLLVKKE---LKGATTEFTETLNRIMVLNLAF
                 2110      2120      2130      2140      2150
```

```
HUMAN*  ATHVTDFFTGSDSIQGTWCKDILQTIMSFTPHNWASHTLSCFPGPLQAFF
CHICK   ATHVTDFFTGSDSIQGTWCKDILQTIMSFTPHNWASHTLSCFPAPLQVFF
XENTR   ATHVTDFFTGSESIQGTWCKDILQTIISFTPHNWALHTLSCFPAPLQAFF
DANRE   ATHVTDFFTGSDSIQGTWCKDILQTIMNFTPHNWASHTLSCFPAPLQAFF
CIOIN   SIHITG----AESISGSWCTDILKAVMSATPHRWPQQTLACFPQSIQDFF
ANOGA   SMHITG--TGNDPQSSAWCKDLLQNIMVNTPHAWPQHTLYCFPPVLNEFF
TETNG   ATHVTDFFTGSDSIHGTWCKDILQTIMNFTPHNWASHTLSCFPAPLQAFF
DROME   SMHVHG---GGDEMQG-WCKDFLSNIIQHTPHSWPMHSLACFPPALNEYF
BRUMA   SMHVHG---GGDEMQG-WCKDFLSNIIQHTPHSWPMHSLACFPPALNEYF
CAEEL   AVKITA-------QDIAIEKTMFPTIIN--GFGWPEKSTSYFPKWALDAI
RHIO9   LIKTRG---QAD-IKTEQIHRVLSSLYDF-PLEWSPAALAHFPPVVRSFY
ARATH*  TIKTRG---IADPDHMVYLQTMLEQILATSQHTWSEKTMRHFPSLLRETL
VITVI   IIKTRG---IAEADHLPYLQTMLEQIMATSQHTWSEKTLRYFPSLLREAM
SOYBN   LIKTRG---IADAEHLLYLQNMLEQIMATSHHTWSEKTLHHFPSVLREAL
SOLLC   IIKTRG---IGEFEQLLYLQTMLEQILATSQHTWSEKTLRYFPSILRDAL
ORYSJ*  TMKTRG---IAEVEHIIHLQPLLEQIMATSQHTWSEKTLRYFPPLIRDFL
HORVU   --------------------------------------------------
SORBI   TIKTRG---IAEVEHMIYLQPLLEQIMATSQHTWSEKTLRYFPPLIRDFL
SELML   TIKTRG---IAEFEQLLVLQTALEQILANSKHTWSEKTMRHFPQTLREVL
PHYPA   TMKTRG---IADFEQMIILKPALEQILASINHTWSEKTMRHFPPLLREAL
                 2160      2170      2180      2190      2200
```

MED23

```
HUMAN*   K----QNNVPQESRFNLKKNVEEEYRKWK---------------SMSN--
CHICK    K----QNNVPQESRFNLKKNVEEEYRKWK---------------SMTN--
XENTR    K----QNNVPQESRFNLKKNVEEEYRKWK---------------SMTN--
DANRE    K----QNNVPQESRFNLKKNVEEEYRKWK---------------SMTN--
CIOIN    N----HSNVEPLNKKQLKTNVEQEYIKWAPLLSADGMDSSSSSGSSLT--
ANOGA    M----QHNIPKESKQLLKKTVDEEYRNWA---------------SMTN--
TETNG    K----QNNVPQESRFNLKKNVEEEYRKWK---------------SMAN--
DROME    T----QNNQPPENKQQLKKAVEEEYRTWT---------------SMTN--
BRUMA    T----QNNQPPENKQQLKKAVEEEYRTWT---------------SMTN--
CAEEL    KA---SDTSNAVNTEEILSDVRNTARMHT---------------SLT---
RHIO9    E----NVNAEPRAGVTAAK-VQQTMSTHK---------------SLTTYL
ARATH*   KGRVDKRGLSIQAWQQAETTVINQCTQLL---------------SPSA--
VITVI    IGRIDKKNLAIQAWQQAETTVIAQCTNLL---------------LSSG--
SOYBN    SGQTDKRSLAIQTWQQAETTVIHQCTQLL---------------SPSA--
SOLLC    SGRMDKRGLAIQAWQQAETTVINQCTQLL---------------SPSA--
ORYSJ*   MGRMDKRGQAIQAWQQAETTVINQCNQLL---------------SPSA--
HORVU    --------------------------------------------------
SORBI    MGRMDKRGQAIQAWQQAETTVINQCNQLL---------------SPSA--
SELML    ALRSDGRLLTLQTWQQVETTVLHQCQQLL-----------------AG--
PHYPA    ANRIDTRAHVIQSWQQAEHTVMSHCRQLL---------------SANS--
                  2210      2220      2230      2240      2250
```

```
HUMAN*   ------ENDIITHFSMQGSPP--LFLCLLWKM-------LLETDHIN---
CHICK    ------ENEIITRFSVQGSPP--LFLCLLWKM-------LLETDHIN---
XENTR    ------ENDIITHFSLQGSPP--LFLCLLWKM-------LLETDQIN---
DANRE    ------ENDIITHFSMQGSPP--LFLCLLWKM-------LLETDHIN---
CIOIN    ------EADALNHFSQPGTPP--YYLCLLWKV-------LFEEKKLS---
ANOGA    ------ENDIIAHFGMTVNPP--LFLCLVFKM-------IIETDGIS---
TETNG    ------ENDIITHFSMQGSPP--LFLCLLWKM-------LLETDHIN---
DROME    ------ENDIIAHFLRPTTNP--LFLCLLFKI-------IWETENIS---
BRUMA    ------ENDIIAHFLRPTTNP--LFLCLLFKI-------IWETENIS---
CAEEL    ------PNQFVIRYGEDRDPA--TSHCMLAVLFHFAYNSVDSTYNIT---
RHIO9    LQGGSAESEALSFLATQENHS--CLFCILWVI-------AVSRNTVDCFH
ARATH*   ------EPAYVSTYLSHSFPQHRQYLCAGACL-------LMQG-HAENIN
VITVI    ------DPSYVMTYISHSFPQHRRYLCAAACM-------LMHG-HPDNIN
SOYBN    ------DPSYVMTYISHSFPQHRQYLCAGALI-------LMHG-HAENIN
SOLLC    ------DPSYVVTYINHSFPQHRQYLCAGAWI-------LMHG-HPENIN
ORYSJ*   ------EPTYVMTYLSHSFPQHRQYLCAGAWM-------LMNG-HLE-IN
HORVU    --------------------------------------------------
SORBI    ------EPNYVMTYLSHSFPQHRQYLCAGAWM-------LMNG-HLE-IN
SELML    ------DPAFAMSFVNHSFPPHRPFLCAAAWV-------LMDC-RPENFN
PHYPA    ------DPSYVSQYIQHSLPAHRQFLCAGAWM-------SMDFERPDSIS
                  2260      2270      2280      2290      2300
```

```
HUMAN*   -QIGYRVLERIGARALVAHVRTFADFLVY----EFSTSAGGQ-QLNKCIE
CHICK    -QIGYRVLERIGARALVAHVRTFADFLVY----EFSTSAGGQ-QLNKCIE
XENTR    -QIGYRVLERIGARALVAHVRTFADFLVY----EFSTSAGGQ-QLNKCIE
DANRE    -QIGFRVLERIGARALVAHVRTFADFLVY----EFSTSAGGQ-QLNKCIE
CIOIN    -STSYKVLERIGASQLSQHVRKFSDFLVY----EFSVLEGGS-DVKICAN
ANOGA    -PVAYKILERIGARALSTHLRKMCDYLLF----EVANSGGGA-HVNKCVD
TETNG    -QIGFRVLERIGARALVAHVRTFADFLVY----EFSTSAGGQ-QLNKCIE
DROME    -PVAYKILEGISARALSTHLRKFCDYLVA----EVASSSDGRDFIHKCVD
BRUMA    -PVAYKILEGISARALSTHLRKFCDYLVA----EVASSSDGRDFIHKCVD
CAEEL    -SEFYEVMEKKTPKEIVVMGNYLVDYIIA----DAKTQDCNEKTFKNIAK
RHIO9    MPSVRKMLLMIPPSKMATHVIDLVDFIL-----SVEYPANSQALL---FT
ARATH*   STNLARVLREVSPEEVTANIYTLVDVLLHHVHVDLQQGQSLEAVLDKAGA
VITVI    VANLARVLREFSPEEVTSNIYTMVDVLLHHIHMELQHGHSLQDLLSKACA
SOYBN    SGNLGRVLREFSPEEVTSNIYTMVDVLLHHMQIELQQGHSSQDLMLKACA
SOLLC    CTNLGRVLREFSPEEVTANIYTMVDVLLHHIHLELQRGHPLQDLMLKACG
ORYSJ*   SANLARVLREFSPEEVTANIYTMVDVLLHHIQLELQRGHQIQDLLSKAIT
HORVU    --------------------------------------------------
SORBI    SANLARVLREFSPEEVTANIYTMVDVLLHHIQFEVQRQHLAQDLLSKAIT
SELML    CGNLGKALKELSPEDVTSNIYMLVDILLHHMHLQLQHGHSQQDLLLRASA
PHYPA    IANLGRTLKEFSPEEVTTNVYMMVDVLLHHIHVQLQHGHSLQNLLLATSA
                  2310      2320      2330      2340      2350
```

```
HUMAN*   ILNDMVWKYNIVTLDRLILCLAMRSHEGNEAQVCYFIIQLLLLKPNDFRN
CHICK    ILNDMVWKYNIVTLDRLILCLAMRSHEGNEAQVCYFIIQLLLLKPNDFRN
XENTR    MLNDMVWKYNIVTLDRLILCLAMRSHEGNEAQVCYFIIQLLLLKPNDFRN
DANRE    ILNDMVWKYNIVTLDRLILCLAMRSHEGNEAQVCYFIIQLLLLKPNDFRN
CIOIN    VLQDMVWKYNIVFLDRLILCLALRSSEANDVQVAFFIIQYLLIKPDDFLN
ANOGA    TINDMIWKYNIITIDRLVLCLILRTLDGNEAQVSFYIIQLLLLKTTEFRN
TETNG    ILNDMVWKYNIVTLDRLILCLAMRSHENNEAQVCYFIIQLLLLKPNDFRN
DROME    TINNMIWKFNVVTIDRVVLCLALRTHEGNEAQVCFLIIQLLLLKASELRN
BRUMA    TINNMIWKFNVVTIDRVVLCLALRTHEGNEAQVCFLIIQLLLLKASELRN
CAEEL    AAALLVFQFQVLRADRLLLSLIMHPATDEDALICIQIANEFIL-TPEFQE
RHIO9    LLDGFIWKHQWINFNHVLFALTKGSGTPERTKKAMILLQYLLLESREIAQ
ARATH*   NLAFFFWTHEMLPLDIFLLALIDRDDDPHALIIA-----MSLLKTPDLLL
VITVI    NLAFFIWTNELLPLDILLLALIDRDDDSHALRIV-----ISLLDKQELQQ
SOYBN    SIAFFVWTNELLPLDILLLALIDRDDDPHALRMV-----ISLLDRPELQQ
SOLLC    NLSVFIWNHELLPPDILLLALIDRDDDPHALRIV-----INLLDSKELQQ
ORYSJ*   NLAFFIWTHELLPLDILLLALIDRDDDPYALRLV-----INLLERPELQQ
HORVU    --------------------------------------------------
SORBI    NLSFFIWTHELLPLDILLLALIDRDDDPYALRLV-----ISLLEKPELQQ
SELML    TLTYLVWSQEILPFDILLLALLDRDDDIHALRLV-----SLLLDRKEFQQ
PHYPA    GLAHFMWNQELLPFDIVLLALTDRDDDAHALRLV-----GLLLDRPEFQQ
                  2360      2370      2380      2390      2400
```

MED23

```
HUMAN*   - R V S D F V K E - N S P E H W L Q N D W H T K - - - - - - - - - H M N Y H K K Y P E K L Y F E G L
CHICK    - R V S D F V K E - N S P E H W L Q N D W H T K - - - - - - - - - H M S Y H K K Y P E K L Y F E G L
XENTR    - R V S D F V K E - N S P E H W L Q N D W H T K - - - - - - - - - H M N Y H K K Y P E K L Y F E G L
DANRE    - R V S D F V K E - N A P E H W L Q S D W H T K - - - - - - - - - H M T Y H K K Y P E K L Y F E G L
CIOIN    - R V R S F D H D V E N L D H W M Q D D W H A K - - - - - - - - - H M A Y H Q K F P E H Y F F E G L
ANOGA    - R V Q E F I T I - N S P E H W K Q N N W H E R - - - - - - - - - H L A F H Q K F P E K F A P D E S
TETNG    - R V N D F V K E - N A P E H W L Q S D W H N K - - - - - - - - - H M N Y H K K Y P E K L Y F E G L
DROME    - R V Q E F C K D - N N P D H W K Q S N W Q D K - - - - - - - - - H L S F H Q K Y P E K F A L D E S
BRUMA    - R V Q E F C K D - N N P D H W K Q S N W Q D K - - - - - - - - - H L S F H Q K Y P E K F A L D E S
CAEEL    - R I R W Y H Q N V P K K D H F - P T E Y I K A - - - - - - - - - I V K Y H D A F P E F E A C E L V
RHIO9    - R V H H W E S L G I S C R P W T E A D Y Q E K - - - - - - - - L M R Y L Q K F P E Y D E F E - -
ARATH*   - R I K N Y C Q N R G S P E H W L V T Q V F K R N E L Q K A L G N H L S W K D R Y - - - - - - - - -
VITVI    - R V K L F C N N R G S P E H W L C S G M F K R A D L Q K A L G N H L S W K E R Y - - - - - - - - -
SOYBN    - R V K H F C M T R G H P E H W L Y S G I F K R V E L Q K A L G N H L A W K D R Y - - - - - - - - -
SOLLC    - R V K V Y L L N R G P P E H W L S P G P F K R V E L Q K A L G N Y L S W K E R Y - - - - - - - - -
ORYSJ*   - R I K A F C T S R - S P E H W L K N Q P P K R V E L Q K A L G N H L S G K E R Y - - - - - - - - -
HORVU    - - - - - - - - - - - - - - - - - - - - - - - - - - - - - - - - - - L C C E C S Y - - - - - - - - -
SORBI    - R V K N F C N T R - S P E H W L K N Q H P K R A E L Q K A L G S H L S W K D R Y - - - - - - - - -
SELML    L R V Q N Y C R Q H G Q Q E H W L H P G P F Q R V E P P Q A L G N H L A G K E R Y - - - - - - - - -
PHYPA    - R V Q T Y Y V H R G Q T E H W M Q L G P F Q R P E Y P Q A L G S H L A G K D R F - - - - - - - - -
                              2410                2420                2430                2440                2450
```

```
HUMAN*   A E Q V D P P V Q - - - - - - - I Q S P Y L P I Y F G N V C L R F L P V F D I V I H R F L E L - - L
CHICK    A E Q V N P P V Q - - - - - - - I Q P Q Y L P I Y F G N V C L R F L P V F D I V I H R F L E L - - L
XENTR    A E Q V N P P V Q - - - - - - - I Q P Q Y L P I Y F G N V C L R F L P V F D I V I H R F L E L - - L
DANRE    A E Q V N P P I Q - - - - - - - L Q S Q Y L P I Y F G N V C L R F L P V F D I V I H R F L E L - - L
CIOIN    V A H C S S P E Q P N A T G E K P Q P T Y L P T Y F C N I T L R F L P V L D I V I H R C L E L - - P
ANOGA    V S H P S - - - - - - - - - - - - - - - - L P V Y F G N V C L R F L P V L D I I V H R Y L E V P - T
TETNG    A E Q V S P P M Q - - - - - - - L Q S Q Y L P I Y F G N V C L R F L P V F D I V I H R F L E L - - L
DROME    A S Q I P - - - - - - - - - - - - - - - - L P V Y F S N V C L R F L P V L D V V V H R F I E L T I T
BRUMA    A S Q I P - - - - - - - - - - - - - - - - L P V Y F S N V C L R F L P V L D V V V H R F I E L T I T
CAEEL    R S Y D S - - - - - - - - - - - S S N V H M P T Y Y G C L I E R L L P I M D Q Y L H V A L E - - - Q
RHIO9    - - - - - - - - - - - - - - - - - - - - - - - - - - - - - - - - - - - - - - - - - - - - - - - - A
ARATH*   - - - - - - - - - - - - - - - - - - - - - - P T F F D D I A A R L L P V I P L V L Y R L I E - - - N
VITVI    - - - - - - - - - - - - - - - - - - - - - - P V F F D D A A A R L L P V I P L V V Y R L I E - - - N
SOYBN    - - - - - - - - - - - - - - - - - - - - - - P V F F D D I A A R L L P V I P L I I Y R L I E - - - N
SOLLC    - - - - - - - - - - - - - - - - - - - - - - P T F F D D I A A R L L P V I P L I I Y R L I E - - - N
ORYSJ*   - - - - - - - - - - - - - - - - - - - - - - P P F F D D I A A R L L L V I P L I I Y R L I E - - - N
HORVU    - - - - - - - - - - - - - - - - - - - - - - P T F F D D I A A R L L P V I P L I I Y R L I E - - - N
SORBI    - - - - - - - - - - - - - - - - - - - - - - P P F F D D I A A R L L P V I P L I I Y R L I E - - - N
SELML    - - - - - - - - - - - - - - - - - - - - - - P V F F D D M A L R A L P V I H L V V Y R L I E - - - N
PHYPA    - - - - - - - - - - - - - - - - - - - - - - P L F F D D M C L R A L P V I P L I V Y R L I E - - - N
                              2460                2470                2480                2490                2500
```

```
HUMAN*   P V S K S L E T L L D H L G G L Y K F H - D R P V T Y L Y N T L H Y Y E M H L R D R A F L K R K L V
CHICK    P V S K S L E T L L D H L G G L Y K F H - D R P V T Y L Y N T L H Y Y E R H L R E R T N L K R K L V
XENTR    P V S K S L E T L L D H L G G L Y K F H V D R P V T Y L Y N T L H Y Y E M Q L R D R T N L K R K L V
DANRE    P V S K S L E T L L D H L G G L Y K F H - D R P V T Y L Y N T L H Y Y E R H L R D R T N L K R K L V
CIOIN    P S S K S L E T L L E H L G L L Y K F H - D Q P V T Y L F N T L H Y Y E S K L R G R H L L K K K L V
ANOGA    Q L S K T L D V I L D H L G S L Y K F H - D R P I T Y L Y N T L H Y Y E R K L R D R P Q L K K R L V
TETNG    P V S K S L E T L L D H L G S L Y K F H - D R P V T Y L Y N T L H Y Y E R Q L R D R T N L K R K L V
DROME    N V H Q I L G F I L D H L S I L Y K F H - D R P I T Y L Y N T L H Y Y E R I L R D R P A L K K K L V
BRUMA    N V H Q I L G F I L D H L S I L Y K F H - D R P I T Y L Y N T L H Y Y E R I L R D R P A L K K K L V
CAEEL    Q G Y K L N P Q I L Q S V S M L Y K F H - A M P I H F M Y S V L F T S H G L M S G P D A - - K S F V
RHIO9    F A M Q G F E T T I E L Q P P L Q I - - - K L P I Y F A N F F L Y Y H A S P T L T N P S V R K R I L
ARATH*   N A M E Q A D N L L L A H S H F L A Y H - P L R F T F V R D I L A Y F Y G H L P G - - - - - - K L V
VITVI    D A T D Q A D R V L A M Y S Q L L A Y H - P L R F T F V R D I L A Y F Y G H L P G - - - - - - K L T
SOYBN    D A M D T A E R L L A M Y S P L L A Y Y - P L R F T F V R D I L A Y F Y G H L P G - - - - - - K L I
SOLLC    D A M D A A D R I L Q V Y S P F L H Y Y - P L N F T F V R D I L S Y F Y G H L P G - - - - - - K L I
ORYSJ*   D A T D I A D R V L A V Y S T F L A F H - P L R F T F V R D I L A Y F Y G H L P S - - - - - - K L I
HORVU    D A T D I A D R V L A F Y S T F L A F H - P L R F T F V R D I L A Y F Y G H L P S - - - - - - K L I
SORBI    D A T D I A D R V L A F Y S S L L A F H - P L R F T F V R D I L A Y F Y G H L P I - - - - - - K L I
SELML    D A T D T A E H V L T A Y S P L L R Y H - P T R F T F V R D T L A Y F Y G H L P N - - - - - - K L I
PHYPA    D A T E T A E R L L H V Y T P L I F Y H - P T R F S F V R D T L A Y F Y G H L P T - - - - - - K L V
                              2510                2520                2530                2540                2550
```

```
HUMAN*   H A I I G S L K D N - R P Q G W C L S D T Y L K C A M N A R E E N - P W V P D D T Y Y C R L I G R L
CHICK    H A I I G S L K D N - R P L G W C L S D T Y L K C A M N A R E D N - P W V P D D T Y Y C K L I G R L
XENTR    H A I I G S L K D N - R P Q G W C L S D T Y L K F G M N P R D D N - P W I P D D T Y Y C K L I G R L
DANRE    H A I M S S L K D N - R T P G W C L S E T Y L K C G M N A R E D N - V W I P D D T Y Y C K L I G R L
CIOIN    S T I I G A I T K N - K P P G W I L S E T F M D Y L Q Q P N D Q - - Q W Q P D S V Y Y C Q L I Q R L
ANOGA    G T V V G S L K D V - R P E N W A L T E A Y Q A Y M M T K E S D N V N W V P E L S Y Y I N L V R R M
TETNG    H A I M S S L K D N - R S P G W C L S E T Y L K F G I N P R E D N - V W I P D D T Y Y C K L I G R L
DROME    G A I T S A F S E I - R P P N W S V S E P Y K V Y L Q S Q D S - - - L W T P E L S Y Y M S L I R R L
BRUMA    G A I T S A F S E I - R P A N W S V S E P Y K V Y L Q G Q D S - - - L W T P E L S Y Y M S L I R R L
CAEEL    L A F A T Q I E E C - - - - - - H L T E A F E K F N H Q K S S R E - - - - - - - Q L I M E L I D R M
RHIO9    R L I - - - - - - - - D F D Q Y T I A P E A V N Y A K N E Q D D - - G S A F D A G Y F E R I I Y K L
ARATH*   L R M L K V L D L S - K I P - - - F S E S F P Q Y I S P T G A - - - P V C P P L D Y F A S L L L N L
VITVI    V R I L N I L D L G - K I P - - - F S E S F L K H M S S S N P - - - V I C P P L D Y F A T L L L G L
SOYBN    V R I L N V L D I S - K I P - - - F S E S F P Q Q I S L T N P - - - V M C P P L D Y F T T L L L G I
SOLLC    L R I L N I L D I K - K I P - - - F S E S F P Q H I N S S N A - - - A M C P P L D Y F A T L L L G L
ORYSJ*   V R I L N V L G V S T K T P - - - F S E S F A Q Y L A S S N S - - - S I C P P P E Y F A N L L F G L
HORVU    V R I L N V L G V S T K T P - - - F S E S F A Q Y L G S S N S - - - S I C P P P E Y F A N L L L G L
SORBI    G R I L N L L G V S T K T P - - - F S E S F A K Y L V S S N S - - - S V C P P P E Y F A N L L L N L
SELML    L R L L S S L D I A - K M P - - - V S E A F I R I L N T G T - - - - - - - P P Y D Y Y V T M L L N L
PHYPA    R R L L G S L D L P - K I P - - - F S E T F L Q Q I K A F N T - - - G S S P S Y E Y F H N L L V G L
                              2560                2570                2580                2590                2600
```

MED23

```
HUMAN*   VDT----MAGKSPG-----------------------------------P
CHICK    VDNI---LLSKSPG-----------------------------------P
XENTR    VDNILYSMAGKSPG-----------------------------------P
DANRE    VDT----MAGKSPG-----------------------------------P
CIOIN    ANT----VGGKMTS-----------------------------------S
ANOGA    QDT----MDGKN-------------------------------------I
TETNG    VDN----ILHYSPS-------------------------LNTLTHTHRCP
DROME    ADT----ISGKN-------------------------------------V
BRUMA    ADT----ISGKN-------------------------------------V
CAEEL    SASLDFILTPP--------------------------------------P
RHIO9    AKS----LNPQTCA--------------------------------PRSK
ARATH*   VNN----VIPPLSSSSNCSSRSGSMADILNSSARPPHGKTPGTSQPGPAN
VITVI    VNN----VIPPINT----NSKSGSMGDTSNNTLRAPHNKTPAASQSGPTN
SOYBN    VNN----VIPPLHN----NSKSGSMGDASSNTLRTAQSKPPAVSQSGSAN
SOLLC    VNH----VIPALNN----SSKCAVMGDFANNSTRAPHGKIPATSQSGTTN
ORYSJ*   VNN----VIPPLSC----KSKSNP-SDAAGSTARTTYNKPYTSS-AGGIS
HORVU    VNN----VIPPLSS----KSKSNP-ADASG--GRTNFSKPHASTQAGGNS
SORBI    VNN----VIPPLSS----KSKSNP-AD----TTRSTFNKHHASSQPGGVG
SELML    VNN----VIPSFTN----KPRTSSPTMESFCTTRST---TPGKSPASSTN
PHYPA    VNN----VIPPLHG----KPGTSATGGDGLLSSRFVLNRNQAAA-STANN
                  2610      2620      2630      2640      2650

HUMAN*   FPNCDWRFNEFPNPAAH---ALHVTCVELMALAVSGKEVGNALLNVVLKS
CHICK    FPNCDWRFNEFPNPAAH---ALHVTCVELMALAVPGTDVGNALLNVVLKS
XENTR    FPNCDWRFNEFPNPAAH---ALHVTCVELMALAVPGKEVGNALLNVVLKS
DANRE    FPNCDWRFNEFPNPAAH---ALHVTCVELMALAVPGKDVGNALLNVVLKS
CIOIN    FSSRDWRFNEFPNASSH---ALHVTCVEALALPLPGEQVGQALMDVVLKT
ANOGA    FAGTDWRFNEFPNPPAH---ALYVTCVELLGLPVGPSVVANSIIDVLVKG
TETNG    FPNCDWRFNEFPNPAAH---ALHVTCVELMALAVPGKDVGNALLNVVLKS
DROME    FYSTDWRFNEFPNAPTH---ALYVTCVELLGLPVAPPLVASNLIDVIVSG
BRUMA    FYSTDWRFNEFPNAPTH---ALYVTCVELLGLPVAPPLVASNLIDVIVSG
CAEEL    FVAKDWKIAEL-SPGAQ---TLYLACIELMASPHSPETLVAAMINVM---
RHIO9    SSLPERHFREIGSPAVE---GIIVATLEIMLTTTPPATIVKYLLDLALLR
ARATH*   ASEGQKAFYQIQDPGTYTQLVLETAVIEILSLPVSAAQIVSSLVQIIVNI
VITVI    ASEGQKSFYQTQDPGTFTQLVLETAVIEILSLPVPATQIVSSLVQIIVHI
SOYBN    ASEGQKAFYQIQDPGTYTQLVLETAVIEILSLPISASQIVQSLVQIVVNI
SOLLC    SFDGQKPYYQMQDPGISTQLTLETAVIELLSLPVSPSQIVSSLVQIVVHI
ORYSJ*   NSDGQRAFYQNQDPGSYTQLVLETAAIEILSLCVPASQIVSSLVQIIAHV
HORVU    NTDAQRAFYQNQDPGSYTQLVLETAAIEILSLSVPASQIVSSLVQLIAHV
SORBI    NTDGQRAFYQNQDPGSYTQLVLETAAIEILSLPVPAAQIVSSLVQIIAHV
SELML    SADNHKAFYHHQDPGSYCQLVLETCVIELLSLPTPPAQAVAMLVQIAVRV
PHYPA    TTEVNKAFYLHQDPGSYNQLLLETAVVELLSLPPPPNQIVAMLIHIAVKL
                  2660      2670      2680      2690      2700

HUMAN*   QPLVPRENIT----------------------------------------
CHICK    QPLVPRENIT----------------------------------------
XENTR    QPLVPRENIT----------------------------------------
DANRE    QPLVPRENIT----------------------------------------
CIOIN    QSFVEREQSL----------------------------------------
ANOGA    YPVVPNGVIH----------------------------------------
TETNG    QPLVPRENIT----------------------------------------
DROME    YAVIPQKDIH----------------------------------------
BRUMA    YAVIPQKDIH----------------------------------------
CAEEL    -QMKPHARPF----------------------------------------
RHIO9    ESHHVGVSA-----------------------------------------
ARATH*   QSTLIQSGNGFHGAANGVGQGSVLPTSPSGGSTDSMSASRSTCLIPGINT
VITVI    QSTLIQSSNGLHGPPNGVGQGSVLPTSPSGGSTDSLSASRSSASVSGINA
SOYBN    QPTLIQSSNALHGGSNSVGQGSVLPTSPSGGSTDSLGASRSTPSVSGINT
SOLLC    QPTLVQSSNGLHGAPGSSGQGSILPTSPSGGSTDSLGATRTTPSLSGLNT
ORYSJ*   QAMLIQSNSG-HGMSGGLGQNSGVPTSSGGG-VEPVGANRPNTTASGINA
HORVU    QAMLIQSNTG-QGMSAGLGQNSGLPTSPSGAGAESAGASRANTSASGISA
SORBI    QAMLIQSNSG-QGMSGGLGQSSGLPTSPSGA-AESSGPNQANSAASGINA
SELML    PTPPSQAYAS--ASQSAAPRSPLLPTSPPSACTAESSTPSSTSVSTGQTI
PHYPA    PTSFPQ------------GGPPKTPQSPSSG-----GEALSGQSSSGLNN
                  2710      2720      2730      2740      2750

HUMAN*   ----------------AWMNAIGLIITALPE-PYWIVLHDRIVSVI----
CHICK    ----------------AWMNAIGLIITALPE-PYWIVLHDHIVSVI----
XENTR    ----------------AWMNAIGLIITALPE-PYWIVLHDRIVSIL----
DANRE    ----------------AWMNAIGLVITALPE-PYWIVLHDRIVSVI----
CIOIN    ----------------PWLNAVALVLTALPE-IYWKPLHQNILKML----
ANOGA    ----------------NWANTIGLIMAALPE-AYWSVIYERMQEAL----
TETNG    ----------------AWMNAIGLVITALPE-PYWIVLHDRIVSVI----
DROME    ----------------SYINAVGIVLAALPE-PYWSGIYDRLQDML----
BRUMA    ----------------SYINAVGIVLAALPE-PYWSGIYDRLQDML----
CAEEL    ----------------NIVNLTALLLTALPA-AYSNALHDEFVAVF----
RHIO9    ----------------LTMHAIGLLIPLLPQQEFIHPIFKELNHLIMTNP
ARATH*   ASFVSRSGYTCQQLSCLLIQACGLLLAQLPP-DFHVQLYLEAARVT-RET
VITVI    SNFVSRSGYTCQQLSCLLIQACGLLLAQLPP-DFHKQLYIEASCLI-KES
SOYBN    SNFASRSGYTCQQLSCLLIQACGLLLAQLPS-DFHSQLYLETTRII-KEN
SOLLC    SNFVSRSGYTCQQLSCLLIQACGLLLAQLPP-EFHVQLYVEAARII-KES
ORYSJ*   SNFVSRSGYSCQQLSVLMIQACGLLLAQLPP-EFHTLLYAEAARII-KDC
HORVU    -NFVSRSGYSCQQLSVLMIQACGFLLAQLPP-EFHMQLYSEAARII-KDC
SORBI    TNFVSRSGYSCQQLSVLMIQACGLLLAQLPP-EFHMQLYSEAARVI-KDC
SELML    SPFI----------SPQVIQACGLLLAQLPR-QFHDVFYKEVARIL-KDC
PHYPA    SSPQFTH-------SPLMIQACGLLLAQLPV-AFHGAIYSETARIIKKDC
                  2760      2770      2780      2790      2800
```

MED23

```
HUMAN*  --------------------------------------SSPSLTSETE-W
CHICK   --------------------------------------NSPSLTSETE-W
XENTR   --------------------------------------NSPSLTSETE-W
DANRE   --------------------------------------SSAVLSSGSD-W
CIOIN   --------------------------------------QCPTLTADIEPG
ANOGA   --------------------------------------GSKAMKEWT--Y
TETNG   --------------------------------------SSPALTSETE-W
DROME   --------------------------------------NTPNMLNWT--Y
BRUMA   --------------------------------------NTPNMLNWN--Y
CAEEL   ------------------------------------------VNGETANL
RHIO9   YLSAVSEPCRLIRCGIPKSVNGKYTMNQSLPDLTTTPILKDSMNARVGKA
ARATH*  WW------------------------------------LKDGKRSQGELD
VITVI   WW------------------------------------LTDGKRSLGELD
SOYBN   WW------------------------------------LKDGTRSLGEID
SOLLC   WW------------------------------------LTDAKRSVGELE
ORYSJ*  WW------------------------------------LADSSRPVKELD
HORVU   RW------------------------------------LSDSSRPVKELN
SORBI   WW------------------------------------LADSSRPVKELD
SELML   WW------------------------------------LKDPKKTPKEMD
PHYPA   WW------------------------------------LTDPSKQSRDLD
                         2810      2820      2830      2840      2850
```

```
HUMAN*  VGYPFRLFDFTACHQSYSEMSCSYTLALAHAVWHHSSIGQLSLIPKFLTE
CHICK   VGYPFQLFDFTACHQSYSEMSCSYTLALAHAVWHHSSIGQLSLIPKFLTE
XENTR   VGYPFQLFDFTACHKAYSEMSCSYTLALAHAVWHHSSIGQLSLIPKFLPE
DANRE   LGYPFQLLDFTACHQSYSEMHCSYVLALAHAVWHHSSIGQLSLIPKFLSE
CIOIN   SSYPYHILSYSNQQLSFTDMPCTRLLALTHVVWHHMSIGQLSLVSQFLKE
ANOGA   RQSPFDMFNFKIVKEAMLDRSYVTVLAIVHSIFHHFGIGQLATITDSIKE
TETNG   AGYPFALLDFTACHQSYSEMNCSYVLALAHAVWHHSSIGQLSLIPRFLSE
DROME   RFNAFELFNFKTVREAMLEKTYAVVLAVAHSVFHHMGAFKLAAMTRYLKE
BRUMA   RFNAFELFNFKTVREAMLEKTYAVVLAVAHSVFHHMGAFKLAAMTRYLKE
CAEEL   KFEEIVFDNYEESLLLNLPNRARTINVISQQYWLHCSLSLLNFFSHEYVP
RHIO9   MIFPYIFNDYTFNLHNYSTNAPNSFLTLFHSIVHYSSLDIFGIFLEYFQT
ARATH*  SAVGYALMDPTWAAQDNTSTAIGNIVALLHAFFSNLPQEWLDG-TNAIIT
VITVI   SAVGYALLDPTWAAQDNTSTAIGNIVALLHAFFSNLPQEWLEG-THLIIK
SOYBN   SAVGYALLDPTWAAQDNTSTAIGNVVALLHSFFSNLPQEWLEG-TNVIIK
SOLLC   SAVSYALLDPTWAAQDNTSTAIGNIVALLHAFFCNLPQEWLEG-THLIIK
ORYSJ*  SAVGYALLDPTWASQDNTSTAIGNIVALLHSFFSNLPHEWLES-THTVIK
HORVU   SAVGYALLDPTWASQDSTSTAIGNIVALLHSFFSNLPQEWLES-SHTVIK
SORBI   SAVGYALLDPTWASQDNTSTAIGNIVALLHSFFSNLPQEWLES-THTVIK
SELML   AAFGYSVWDPSWAVQDGTSSVIGNAAALFHAFTANISFEWLEN-THDVVN
PHYPA   AIFGYSTWDPSWGARDDTATVIANTVTLLHTLQSNLPFEWLEG-MHSVIT
                         2860      2870      2880      2890      2900
```

```
HUMAN*  -VLLPIVKTEFQLLYVYHLVGPFLQRF-----------QQERTRCMIEIG
CHICK   -VLIPIVKTEFQLLYVYHLVGPFLQRF-----------QQERTRCMIEIG
XENTR   -VLIPIVKTEYQLLYVYHLVGPFLQRF-----------QQERTRCMIEIG
DANRE   -VLKPIVKTEFQLLYVYHLVGPFLQRF-----------QQERTRCMLEIG
CIOIN   -EAKPLIETELQLLYIFHLIGPYFPRF-----------QLERTRCMMDVA
ANOGA   -KLKPLVHTEYQLIYLCHVVGPFLHRL-----------SIERARAVADIT
TETNG   -TLKPIVQTEFQLLYVYHLVGPFLQRF-----------QQERTRCMLEIG
DROME   -KLKPCVRTEQQLLYLCHVFGPFLQRI-----------ELEKPNAVAGIA
BRUMA   -KLKPCVRTEQQLLYLCHVFGPFLQRI-----------ELEKPNAVAGIA
CAEEL   -RILEHVKTEKDLWYTLRLVMPYLRRYYENWDTAKQMRSQRENFGPLHIV
RHIO9   LRHSGKLLTDVQLLYVCFLIGPALHRI-----------EKLDSSEAEFLI
ARATH*  -NLRP-VTSVAMLRVVFRIMGPLLPRL-----------ASTHTLFNKTLM
VITVI   -HLRP-VTSVAMLRIAFRIMGPLLPRL-----------SNAHSLFNKTLS
SOYBN   -QLRP-VTSVAMLRIAFRVMGPLLPKL-----------ANAHALFNKTLS
SOLLC   -HLRP-VTSVAVLRISFRIMGPLLPRL-----------VNAHTLFSKTVS
ORYSJ*  -HLRP-VNSVAMLRIAFRILGPLLPRL-----------AFARPLFMKTLA
HORVU   -HLRP-VTSVAMLRIAFRILGPLLPRL-----------AFARPLFMKTLA
SORBI   -HLRP-VNSVAMLRIAFRILGPLLPRL-----------AFARPLFMKTLA
SELML   -LQRP-MTTVAQLRLAFRMLGPLVPRF-----------VISRPLFAKTLA
PHYPA   -QQRP-FVSVVHLRLAYRIMGPLLPRL------------LSRPLFAKTLA
                         2910      2920      2930      2940      2950
```

```
HUMAN*  VAFYDMLLNVDQC--ST-----HLNYMDPICDFLYHMKYMF-------TG
CHICK   VAFYEMLLNADQY--SS-----HLNYMDPICDFLYHMKYMF-------TG
XENTR   VAFYEMLLNVDQC--SA-----HLNFMDPICDFLYHMKYMF-------TG
DANRE   VAFYEMLQAVDQH--SN-----HLAYMDPICDFLYHIKYMF-------TG
CIOIN   QLLYNILLRVSKH--SS-----EIHFADEIADFLYHVKYMH-------IG
ANOGA   LMLYELLEQVDKAQPAQ-----PLRYMDPICDLLYHIKYMF-------VG
TETNG   VAFYEMLQAVDQH--CQ-----HLSFMDPICDFLYHIKYMY-------TG
DROME   VLLYEILEIVDKHHGPK-----PLQYMDQICDFLYHIKYIH-------VG
BRUMA   VLLYEILEIVDKQHGPK-----PLQYMDQICDFLYHIKYIH-------VG
CAEEL   KLVFQKLGSMAEE-------GVEIVYEQHLCDLFYNCKYFF-------AG
RHIO9   ELMHIVKQVTSLMDMKD---GWTTQALEQSFDFLYHIRTRF------VKT
ARATH*  LLLSALVDVFGKTAQTT--APVEASQIADLIDFLHHIIHYEGQGGAVQTS
VITVI   LLLNTMVDVFGRNSQPA--TPVEASEIADLIDFLHHAVHYEGQGGPVQAS
SOYBN   SLLTILVDVFGKNSQTS--IAVDASDIADIIDFLHHVVHYEGQGGPVQAS
SOLLC   LLLNILVDVFGKNSQLS--APIEATEISDLIDFLHHVIHYE------VAS
ORYSJ*  LLFNVLGDVFGKNSQAS--PPVEASEIADIIDFLHHAVMYEGQGGPVQST
HORVU   LLFNVLGDVFGKNSQAS--PHVPASEIGDIIDFLHHAVMYEGQGGPVQST
SORBI   LLFNVLGDVFGKNPPVSNPNPVEASEIADIIDFLHHAVMYEGQGGPVQST
SELML   LLFTMLAEVFGRNAQVH-----DVTEITDLVDFMHHVVMLEAQ----QAG
PHYPA   LLFTILADVFGRNSQVP--SATEATDIVDLVDFLHHAVMMEVQATG-QAG
                         2960      2970      2980      2990      3000
```

MED23

```
HUMAN*  DSVKEQV----EKIICNLKPALKLRLRFITH----ISKMEPAAVP-----
CHICK   DSVKDQV----EKIICNLRPALKLRLRFITH----ISKMEPAGVP-----
XENTR   DSIKDQV----EKIICNLRPALKLRLRHITH----ISTESAAPPP-----
DANRE   DSVKDQV----ERIICSLRPAMRLRLRFITH----ISKMEPAAVP-----
CIOIN   VAINDRV----EEVVVQLQPKLRKRLQFITQ----APASSAARQQ-----
ANOGA   DTMKTEL----EAIIRRLRPALQMRLRFITR----LNVEEIGVDQ-----
TETNG   DSVKEQV----GRIIMTLRPAMKLRLRISSRTVSMVETSSSAAAA-----
DROME   NIIKNES----EAIIKRLRPLLQLRLRFITH----LNLEDIHTEK-----
BRUMA   NIIKNES----EAIIKRLRPLLQMRLRFITH----LNLEDIHSEK-----
CAEEL   DFLRNTA----ITEFAKLPEKMRDRLKFYVS------QSEPTAEQETPPE
RHIO9   NELAAQI----REIIRSMNPPISQRLLRLVM-------------------
ARATH*  SKPRPDILALIGRAAETLRPDVQHLLAHLKT----NPNSSIYAAA-----
VITVI   SKPRPEVLALCGRASESLRPDIQHLLSHLKT----DINSSIYAAT-----
SOYBN   SKPRPEVLALIGRASESLRPDIQHLLSHLNP----DVNSSVYAAF-----
SOLLC   SKPRSEILALFGRAAENLRPDVQHLLSHLNT----DVNTSVYA-------
ORYSJ*  SKPKLEILTLCGKVMEILRPDVQHLLSHLKT----DPNSSVYAAT-----
HORVU   SKPKVEILTLCGKVVDMLRPDVQHLLSHLKT----DPTSSIYAAT-----
SORBI   SKPKLEILTLCGKVIEILRPDVQHLLSHLKI----DPASSIYAAT-----
SELML   GKPRPDTLGLCNKAVERLHPDVQHLFQHLTV----DPQRSIYAAT-----
PHYPA   GRPRPETLVLCSKAVERLRPDVQLLFRHLSP----DGASSIYAAT-----
                  3010      3020      3030      3040      3050

HUMAN*  -----------------------PQAMNSGSPAPQSNQVPVS--LPV---
CHICK   -----------------------QQPLSNGSPAQQASQVPVNVALPV---
XENTR   ------------------------PPMNSGSPAPQPSQVPVSVPLTV---
DANRE   ----------------VSNSSSVQQTSSASSPTAQ-STAGAAIPLSV---
CIOIN   ----------------VHSNTTTEKM------------------------
ANOGA   ----------------NAAAAAGGGAGGAATATGQGVAGAGPGVGPP---
TETNG   ----------------VASASSAPSSTPQPPTSSL-STAPGAASPSS---
DROME   ----------------INDNTSNNAITSQTQSPMQTQHQQQP-QQPHQQQ
BRUMA   ----------------INDNASSNAITNQTQSPLQTQHQQQP-QQPH---
CAEEL   KEKSPEKEKEQEQEQHVKAHQPLESTPSVSSLPQMQHHLQQAPLLPSHQM
RHIO9   --------------------------------------------------
ARATH*  --------------------------------------------------
VITVI   --------------------------------------------------
SOYBN   --------------------------------------------------
SOLLC   --------------------------------------------------
ORYSJ*  --------------------------------------------------
HORVU   --------------------------------------------------
SORBI   --------------------------------------------------
SELML   --------------------------------------------------
PHYPA   --------------------------------------------------
                  3060      3070      3080      3090      3100

HUMAN*  --------------------------------------------------
CHICK   --------------------------------------------------
XENTR   --------------------------------------------------
DANRE   --------------------------------------------------
CIOIN   --------------------------------------------------
ANOGA   -------------PGGVAAGAPGATQGVVASGQAAPGTAAGPQQPQQVVG
TETNG   --------------------------------------------------
DROME   QQQQQQQQQQQQQQQQQQQQMQQQQINAVQTTSVPLGSGGNLQQQQQI--
BRUMA   ---------QQPHQPQQQQQMQQQQISAVQTTSVPLGSGGSLQQHQQI--
CAEEL   MPPPQQHSSSLQHHLQHHTSTHQMMDTSQHQTIQQQSNHPTQQQLQHQIP
RHIO9   --------------------------------------------------
ARATH*  --------------------------------------------------
VITVI   --------------------------------------------------
SOYBN   --------------------------------------------------
SOLLC   --------------------------------------------------
ORYSJ*  --------------------------------------------------
HORVU   --------------------------------------------------
SORBI   --------------------------------------------------
SELML   --------------------------------------------------
PHYPA   --------------------------------------------------
                  3110      3120      3130      3140      3150

HUMAN*  --------------------------------------------------
CHICK   --------------------------------------------------
XENTR   --------------------------------------------------
DANRE   --------------------------------------------------
CIOIN   --------------------------------------------------
ANOGA   VVGNQNVALQQ---------------------------------------
TETNG   --------------------------------------------------
DROME   --------------------------------------------------
BRUMA   --------------------------------------------------
CAEEL   NMSMHQQMGPQYPGAVFHHPSGPVGHVPMQYGMGHHMQQHPHLPHHQQMP
RHIO9   --------------------------------------------------
ARATH*  -----------HQ-------------------------------------
VITVI   -----------HP-------------------------------------
SOYBN   -----------HP-------------------------------------
SOLLC   --------------------------------------------------
ORYSJ*  -----------HP-------------------------------------
HORVU   -----------HP-------------------------------------
SORBI   -----------HP-------------------------------------
SELML   -----------HP-------------------------------------
PHYPA   -----------HP-------------------------------------
                  3160      3170      3180      3190      3200
```

MED23

```
HUMAN*  ------------------------------------------TQ------
CHICK   ------------------------------------------TQ------
XENTR   --------------------------------------------------
DANRE   ------------------------------------------TQ------
CIOIN   --------------------------------------------------
ANOGA   -----------------------------------QLQQQQMGQGIQSHH
TETNG   ------------------------------------------SQHSHTAM
DROME   ---------------------------NQQQQMYMQHMQQHQHMQNMRHN
BRUMA   ---------------------------NQQQQMYMQHMQQQQHMQNMRHN
CAEEL   APMHTMNPMMQNMTPQQQYLYMQQLQQHQQHQQYMQQQQQHHHQHQQQPH
RHIO9   -------------------------------------------------L
ARATH*  -----------------------------------QNTAKTNTS------
VITVI   -----------------------------------KLVQNPS--------
SOYBN   -----------------------------------KLAQNPT--------
SOLLC   -----------------------------------KIIHNA---------
ORYSJ*  -----------------------------------KLVQNPS--------
HORVU   -----------------------------------KLVQQHPS-------
SORBI   -----------------------------------KLVQSSS--------
SELML   -----------------------------------KIAQRPLSCFATTT-
PHYPA   -----------------------------------KLQRPGSSSLMGSVM
                          3210      3220      3230      3240      3250
```

```
HUMAN*
CHICK
XENTR
DANRE
CIOIN
ANOGA
TETNG
DROME
BRUMA
CAEEL
RHIO9
ARATH*
VITVI
SOYBN
SOLLC
ORYSJ*
HORVU
SORBI
SELML
PHYPA
```

MED25

HUMAN*	--
CHICK	--
XENTR	--
DANRE	--
CIOIN	--
TENG	--
DROME	--
RHIO9	--
CRYNE	--
USTMA	--
ARATH*	--
VITVI	--
SOYBN	--
SOLLC	--
ORYSJ*	--
HORVU	--
SORBI	--
SELML	--
PHYPA	--
VOLCA	MNQQTYLQQQQQYQQYQQYQQHASSQQFHQQATPPQQHYASPQQHYTPPH
	10 20 30 40 50

HUMAN*	--
CHICK	--
XENTR	--
DANRE	--
CIOIN	--
TENG	--
DROME	--
RHIO9	------------------------------------MMPSSNLVATPGSQ
CRYNE	--
USTMA	----------------------------MTTPTHAGSSAAAMGIATPSPL
ARATH*	--
VITVI	--
SOYBN	--
SOLLC	--
ORYSJ*	--
HORVU	--
SORBI	--
SELML	---MAT
PHYPA	--
VOLCA	AHLTPPQQPTPMLNPGQAQQQLFQQHQQHQQHATYIQQGVLPGTGNALVS
	60 70 80 90 100

HUMAN*	--------MVPGSEGPARAGSVVADVVFVIEGTANLGPYFEGLRKH----
CHICK	----------MVVSGP---SAMVADVVFVIEGSANLGPYFETLRKH----
XENTR	--------MDAATPGN---GGIISDVVFVIEGTANLGPYFESLRKH----
DANRE	--------MDPTSKSG---LNQVSDVVFVIEGTANLGPYFESLRNN----
CIOIN	----------MVKEEN---APSSIEVVIVVEATSNMVSHWDLLKKQ----
TENG	---------------------------------GPIKP------------
DROME	------------MEVD---QIPLADVVFVIEGSAINGAYINELKTN----
RHIO9	SSPAPIQSPQILVSHH---QRKDVAIVFIVDGSARMKPHLNLLYEA----
CRYNE	--------------------MSACTVTFVFDSALPLQQYTKLVQPDGIIY
USTMA	SNATPRSSSIALSSLQ---ALPETQLVLVLFDHPIVRRKWRTVVVP----
ARATH*	---------------M---SSEVKQLIVVAEGTAALGPYWQTIVSD----
VITVI	--------------------MAEKQLVVAVEGTAAMGPYWQAVVSD----
SOYBN	--------------------MAEKQLIVAVESTAAMGPYWNTILMD----
SOLLC	---------------------MVDKLIVAVEGTAVLGPYWKIIVSD----
ORYSJ*	-------------MAA---AAAERQLVVAVEGTAALGPYWPVTVAD----
HORVU	------------MVEG---MASERQLVVVVEGTAALGPYWTAIATE----
SORBI	---------------M---AAADRQLVVAVEGTAALGPYWSTIVAE----
SELML	SSSAGPPSSGSGVTAQ---QPQAQYLVLAVEATAALGPSWPLLRSE----
PHYPA	-------------------MMSQRQLVVAVEATGALGPFWSVLLTE----
VOLCA	ASAASATMAVATAAPS---SPSYSRVVFVIEATASMLQFWHNLRTL----
	110 120 130 140 150

HUMAN*	--------------------YLLPAIEYFNGGPPAETDFGGD----YGGT
CHICK	--------------------YLLPAIEYFNGGPPAETDFGGD----YGGT
XENTR	--------------------YLLPAIEYFNGGPPAETDFGGD----YGGT
DANRE	--------------------YILPTIEYFNGGPPAETDFGGD----YGGT
CIOIN	--------------------YILPAVNHFHQQTNTSDELFTS----ANGN
TENG	--
DROME	--------------------YILPTLEHFTTGSIDEREYLIAE---RFAT
RHIO9	--------------------YVEPILKQLRSPIITEADERQSKTKITPTL
CRYNE	KLIQRLEEKHGGNHYLHVSVLITPPTSSNRSIKKSMSKAFRSEYGSASTF
USTMA	--------------------LVESISKKLNTAYS------------GSRL
ARATH*	--------------------YLEKIIRSFCGSELNGERNP------VSTV
VITVI	--------------------YLDKIIRYFCGNELAGQKPS------SSNF
SOYBN	--------------------YLDKMIRCFGGNDSTGQKFS------ASNV
SOLLC	--------------------YLDKIIRCFFGVDSTSQKSS------AADV
ORYSJ*	--------------------YVEKIVRSFCAHEMAGQKLA------GTPP
HORVU	--------------------YVEKIVRSFCSTELSGQKLA------GVPP
SORBI	--------------------YVEKIVRSFCASELPGQKLA------GPPP
SELML	--------------------YLDKIARAFYGQDGNVQKDN------GATL
PHYPA	--------------------YVDKIVRAFFD-ESNNQKNG------SSPG
VOLCA	--------------------YVELLLRYIDKGV-------------FGRV
	160 170 180 190 200

MED25

```
HUMAN*  Q Y S L - - - - V V F N T V D C A P E S Y - - - - - - - - - - - - - - - - - - - - - - - - - - - - -
CHICK   Q Y S L - - - - V V F N T V D C A P E S Y - - - - - - - - - - - - - - - - - - - - - - - - - - - - -
XENTR   Q Y S L - - - - V V F N T V D C A P E S Y - - - - - - - - - - - - - - - - - - - - - - - - - - - - -
DANRE   Q Y G L - - - - V V F N T V D C A P E S Y - - - - - - - - - - - - - - - - - - - - - - - - - - - - -
CIOIN   R Y G L - - - - V L F H A A D V V P Q S L - - - - - - - - - - - - - - - - - - - - - - - - - - - - -
TENG    - - - - - - - - V V - - - - - - - - - - - - - - - - - - - - - - - - - - - - - - - - - - - - - - - -
DROME   L Y G I - - - - V V Y R T A A N L L E P V - - - - - - - - - - - - - - - - - - - - - - - - - - - - -
RHIO9   K Y G L - - - - V V F G D Y E P L S E I T - - - - - - - - - - - - - - - - - - - - - - - - - - - - -
CRYNE   L A A L P K L S V L F P P R S L T P H H W - - - - - - - - - - - - - - - - - - - - - - - - - - - - -
USTMA   T I G S - - - - V V Y R S T A D K S L L R P S W T L S R T K F Q P G S K L I A S N M Q T A E F W L G
ARATH*  E L S L - - - - V I F N S H G S Y C A C L - - - - - - - - - - - - - - - - - - - - - - - - - - - - -
VITVI   E L S L - - - - V M F N A H G S Y C S C L - - - - - - - - - - - - - - - - - - - - - - - - - - - - -
SOYBN   E F A L - - - - V T Y N T H G C Y S S C L - - - - - - - - - - - - - - - - - - - - - - - - - - - - -
SOLLC   E V S M - - - - V M F N T H G P Y S A C L - - - - - - - - - - - - - - - - - - - - - - - - - - - - -
ORYSJ*  E L A L - - - - V V F H T H G P Y S A F C - - - - - - - - - - - - - - - - - - - - - - - - - - - - -
HORVU   E L A L - - - - V V F H T H G P Y S A F V - - - - - - - - - - - - - - - - - - - - - - - - - - - - -
SORBI   E L A L - - - - V V F H T H G P Y S A F D - - - - - - - - - - - - - - - - - - - - - - - - - - - - -
SELML   E M A L - - - - V V F R G H D S Y S G C L - - - - - - - - - - - - - - - - - - - - - - - - - - - - -
PHYPA   E V A L - - - - V V F N T H G S H S D F L - - - - - - - - - - - - - - - - - - - - - - - - - - - - -
VOLCA   E L A V - - - - V L V G V A D G T S D Q P - - - - - - - - - - - - - - - - - - - - - - - - - - - - -
                            210                 220                 230                 240                 250
```

```
HUMAN*  V Q C H A P T S S A Y E F V T W L D G I K F M G G G G E S C S L I A E G L S T A L Q L F D D F K K -
CHICK   V Q C Q A P T S S A H E F L T W L D G I Q F V G G G G E S C S L I A E G L S T A L Q L F D D F K K -
XENTR   V Q C H A P T S S A Y E F V Q W L D S I R F M G G G G E S C S L I A E G L S T A L Q L F D D F K K -
DANRE   V Q C H A P T S S A F E F V S W I D S I Q F M G G G A E S C S L I A E G L S V A L Q L F D D F K K -
CIOIN   S Q C F P P T R N V H T F I K T L D A I K F G G G G G E Q Y S H I N E G L A T S L Q L F N E F S G -
TENG    - - - - - - - - - - - - - - - - - - - - - - - - - - - - - - - - - - - - - - - - - - - - - - - - - -
DROME   C S T Y G P F L Q P Q K V M E T I E R L P L V G G G M E S C A H M A E G F A A A H G C F D D I S E -
RHIO9   V D R K Y F T S D P I L F Q D I F K S I E C K Y G G P L K N A - V E E G M V A A L E L F D G Y K Q -
CRYNE   - - - - - P Q F D P R I L P S E T R A K D V K R K R S E D V R - I L D G V V G G L E L L E R P Q P I
USTMA   T E R S S V S H D A D P S A S I Y S L I D G R S T T K P T A H - V L E A F V A A L E L L Q P S D N -
ARATH*  V Q R S G W T R D V D I F L H W L S S I Q F G G G G F N E V A - T A E G L A E A L M M F S P P - - -
VITVI   V Q R S G W T R D V D L F L Q W L S A L P F A G G G F N D A A - I A E G L A E A L M M F S V A A N -
SOYBN   V Q R S G W T R D P D V F F S W L S S I P F N G G G F N D A A - I A E G L S E A L M M L W N S Q S -
SOLLC   V Q R S G W T K D M D T F L Q W L S A I P F S G G G F N D A A - V A E G L A E A L V M F S V - P N -
ORYSJ*  V Q R S G W T K D M N V F L S W L S G I S F S G G G F S E A A - I S E G L A E A L M I L Q G S S S -
HORVU   V Q R S G W T K D M D A F L S W L S G I S F S G G G F S G A A - I C E G L A E A L M I L Q G S P S -
SORBI   V Q R S G W T K D T D A F L S W L S G I S F S G G G F S E A S - T C E G L A E A L K I L Q G S P N -
SELML   L Q R S G W T P S L E L F Q L W L S S I D F S G G G F G E V A - V A E G L A E A L V M C C P T T Q -
PHYPA   L R Q S G W T S S M D L F F K W L S A L T F E G G G F S E A A - V A E A L A E A L M E W T I S Y D -
VOLCA   V D G I T W T T S V S E V R S T L D A I Q F A G G G F G P I A - L A Q A L A H V I Y L Q T L P S S M
                            260                 270                 280                 290                 300
```

```
HUMAN*  - - - - - - - - - - - - - - - - - - - - - - - - - - - - M R E Q I G Q T H R V C L L I C - - - - - -
CHICK   - - - - - - - - - - - - - - - - - - - - - - - - - - - - M R E Q I G Q T H K V C V L V C - - - - - -
XENTR   - - - - - - - - - - - - - - - - - - - - - - - - - - - - M R E Q I G Q T H K V C I L I C - - - - - -
DANRE   - - - - - - - - - - - - - - - - - - - - - - - - - - - - M R E Q I G Q T H K V C V L L C - - - - - -
CIOIN   - - - - - - - - - - - - - - - - - - - - - - - - - - - - - T Q S D P Q V T K L C F I V S - - - - - -
TENG    - - - - - - - - - - - - - - - - - - - - - - - - - - - - - - - - - - - - - - - - - - - - - - - - - -
DROME   - - - - - - - - - - - - - - - - - - - - - - - - - - R R Q L L D Q T S V Q R H C I L I C - - - - - -
RHIO9   - - - - - - - - - - - - - - - - - - - - - - - - - - - - - E V E S R E L V Q H C I L L S - - - - - -
CRYNE   H G P S P S Q A Y M S L T P N T S A P L L E Y I C T P S N A M Q D S P L Q R Y L V M V S - - - - - -
USTMA   - - - - - - - - - - - - - - - - G - - - - - - V H N P L A P K S P A V L A R H L L H V S L D S S D V
ARATH*  - - - - - - - - - - - - - - - - - - - - - - - - - - S G Q A Q P S N D L K R H C I L I T - - - - - -
VITVI   - - - - - - - - - - - - - - - - - - - - - - - - - - G S Q T Q Q N V D G Q R H C I L V A - - - - - -
SOYBN   - - - - - - - - - - - - - - - - - - - - - - - - - - G A P N Q Q S V D M H K H C I L V A - - - - - -
SOLLC   - - - - - - - - - - - - - - - - - - - - - - - - - - G N Q T Q Q K M E G K K H C I L I S - - - - - -
ORYSJ*  - - - - - - - - - - - - - - - - - - - - - - - - - - N S Q N H Q S H E V Q K H C I L V A - - - - - -
HORVU   - - - - - - - - - - - - - - - - - - - - - - - - - - N S Q N H Q N R E L Q K H C L L V A - - - - - -
SORBI   - - - - - - - - - - - - - - - - - - - - - - - - - - A T Q S H Q N H E A Q K H C I L V A - - - - - -
SELML   - - - - - - - - - - - - - - - - - - - - - - - - - - - - - P P S N Q A C Q R H C I L V A - - - - - -
PHYPA   - - - - - - - - - - - - - - - - L Y E L N L Q M C C P G P K P P T V P H Q K H C L L I A - - - - - -
VOLCA   P V S A P - - - - - - L P G G G - - - - - - - - - - S A P P P A D P V V P C Y C M L L V - - - - - -
                            310                 320                 330                 340                 350
```

```
HUMAN*  - - - - N S P P Y L L P A - - - - - - - - - - - - - - - - - V E S T T Y S G C T T E N L V Q Q I G E
CHICK   - - - - N S P P Y L L P A - - - - - - - - - - - - - - - - - V E S T T Y S G C S T E S L V Q R I G E
XENTR   - - - - N S P P Y L L P A - - - - - - - - - - - - - - - - - V E S T T Y S G Y T T E N L V Q K I G E
DANRE   - - - - N S P P Y L L P A - - - - - - - - - - - - - - - - - V E S V S Y T G C T A D S L V Q I I R D
CIOIN   - - - - N T P P Y Q L P C - - - - - - - - - - - - - - - - - V E S F E Y A G L T L N Q I T D K L S E
TENG    - - - - - - P P Q P L P S - - - - - - - - - - - - - - - - - - - - - - - - - - - - - - - - - - - - Q
DROME   - - - - N S P P Y Q M P T - - - - - - - - - - - - - - - - - T E S W K Y P G K S C E Q L A A L F N E
RHIO9   - - - - N S L P N K L S T R C - - - - - - - - - - - - - - - N M D A K Y D G F K M D K I I E E M K K
CRYNE   - - - - - - - - - - M A T S A P S P S Y D G S V D V F A H Y N W D S I W D G R G W A A L E Q E C K K
USTMA   S L D L S S P P F F - - - - - - - - - - - - - - - - - - - - N A D A A N D R A T A P T I G S K L A R
ARATH*  - - - - A S N P H I L P T P V Y R P R L Q N V E R N E N G D A Q A E S - R L S D A E T V A S Y F A K
VITVI   - - - - A N N P Y P L P T P V Y Q P Q M Q N M E Q N E S I E S Q T E S - R L S D A E A V A K S F A Q
SOYBN   - - - - A S N P Y P L Q T P V Y V P R P Q N L E Q S E T I D S D S G S - H L Y D A E A V A K A F P Q
SOLLC   - - - - G S N P Y P L P T P V Y R P Q M Q K L E Q N E N I E A Q T D S - R L A D A E T V A K T F P Q
ORYSJ*  - - - - A S N P Y P L P T P V Y R P L V Q S S D H K E N N D G A K E S - C L A D A E T V A K S F A Q
HORVU   - - - - A S N P Y P L P T P V Y R P S V Q S S D H K K S N E A T K E S - C L A D A E A V A V S F A Q
SORBI   - - - - A S N P Y P L P T P V Y C L P T Q S T D H K E N I E T S K E P - S I A D A E T V A K S F A Q
SELML   - - - - A S N P H R L Q T P V P H P P L S T G A D - - - - - A K P D H W W L A D A E A V A R A F S Q
PHYPA   - - - - A S N P Y R L P T P V M R P P V I L L S T G Q A E - L Q S E Q W W L A D A E T V A K A F P -
VOLCA   - - - - Y S P P D Q L Q V Y V P G L N N P A A V A A A A G G G G A G P A Q A A S A A A Q Q S G M A H
                            360                 370                 380                 390                 400
```

MED25

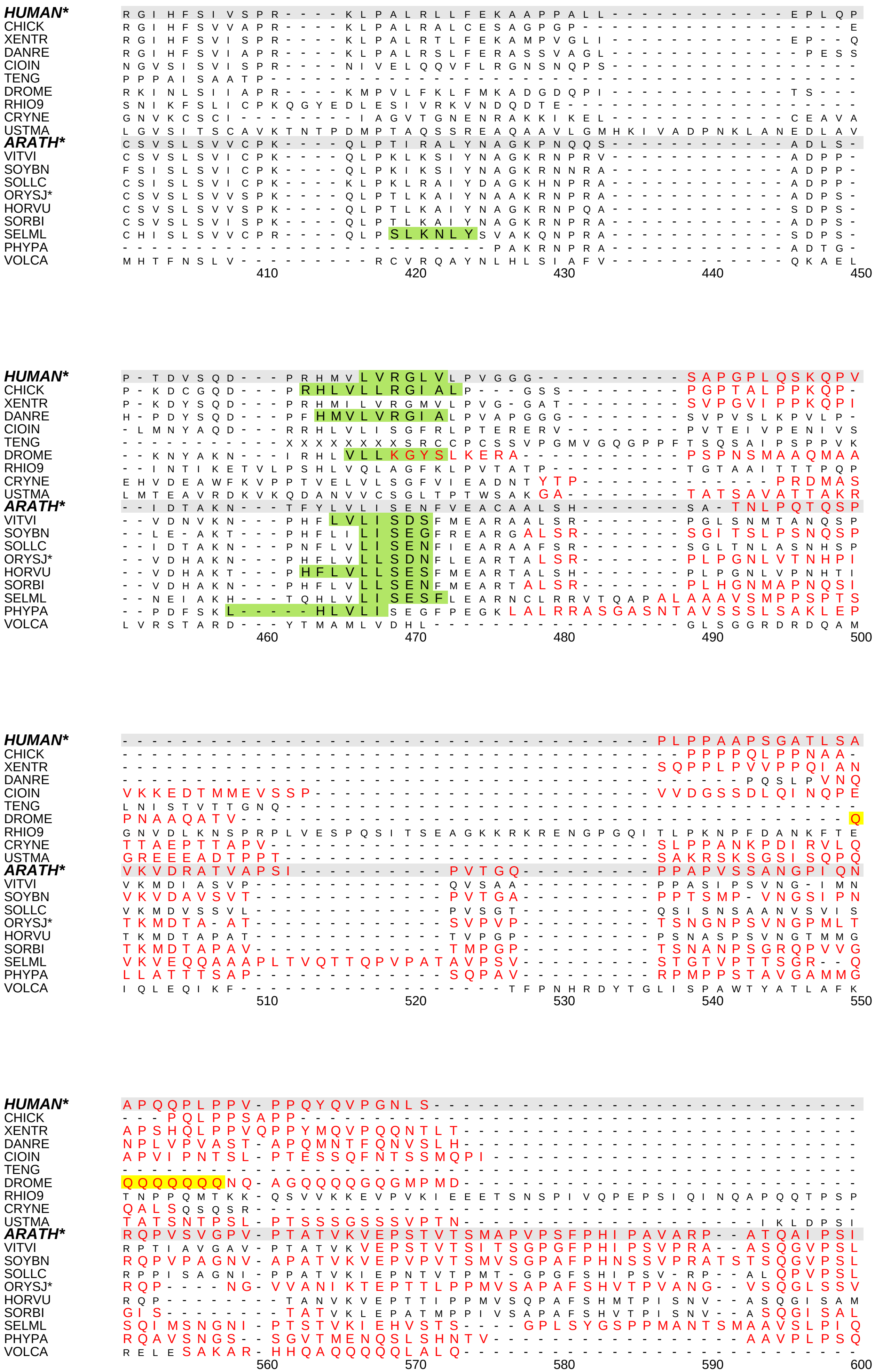
HUMAN*
CHICK
XENTR
DANRE
CIOIN
TENG
DROME
RHIO9
CRYNE
USTMA
ARATH*
VITVI
SOYBN
SOLLC
ORYSJ*
HORVU
SORBI
SELML
PHYPA
VOLCA
410
420
430
440
450
460
470
480
490
500
510
520
530
540
550
560
570
580
590
600

MED25

```
HUMAN*  --------------------------------------------------
CHICK   --------------------------------------------------
XENTR   --------------------------------------------------
DANRE   --------------------------------------------------
CIOIN   --------------------------------------------------
TENG    --------------------------------------------------
DROME   --------TTPAQQQQQQQQQQQQQQQQGNPQQQVMNMNTMQQQQPGPNP
RHIO9   QTQQPFQQTQPQPQPQPQPQPQQLQQNQPSPQQPISQIQQQPVQQQGARP
CRYNE   --------------------------------------------------
USTMA   STKVLFLRS----------QQETMVKNWAAAYSAVVKAESVGETLTSKAP
ARATH*  QTSSASPVSQDMV------SNAENAPDIKPVVVSGMTPPLRTGPPGGANV
VITVI   QTSSPSSTSQEMI------SNGDNVQDLKP-IVSGISQTLRPVVPAAANV
SOYBN   QTSSPSSVSQDII------TNNETAQDTKP-TVSML--PLRPVNPVQANV
SOLLC   QASSPLSVSQEMV------SHTENVQEMKP-IVSGMTQSLRPVAAAAANV
ORYSJ*  QSPSPSLISQETN------LANDSVQEHKP-LINPIQQSIRPGGP--ANV
HORVU   QTSSPLIISQETN------VANEILQEHKP-LVNPIQQQVRPGGP--ANG
SORBI   QTSSPSLISQEAN------MANDNVQEHKP-IINPVQQPVRPGG----HG
SELML   SSGATSAMSQEVLRPPLGSDSSTAVQDFKS-INTNTLQSHRPVPA-----
PHYPA   HPSSSGVSGTDTM------QTSVQTSD----TSASSQAKVGPVGGNPSTL
VOLCA   --------RQAQAQAQAQAQAQAQAQAQAQAQAQAQAQAQAHAKAGGPGVQA
               610       620       630       640       650

HUMAN*  ---------------AAQVAAQNAVEAAKNQKAGLGPRFSPITPLQQAAP
CHICK   --------------------------------------------------
XENTR   ---------------TAHAAAQSAVEAAKNQKNNLPNRF-PLPNLNQMPP
DANRE   ---------------AAHDAAQKALEEAANQQQK--NRFGQIS-------
CIOIN   --------------------------------------------------
TENG    --------------------------------------------------
DROME   PA--GLLNPQQQQQLLQQQQQNQFVSNQMQNQNFQQNVGPGQNRWMYPNQ
RHIO9   VGAPPVPQPLAGRMQNPLQLQQYLLQQRNNLLALGPNLSPVQQSQLQTIQ
CRYNE   --------------------------------------------------
USTMA   GMSRAYLEQLKAQLLTQQHALKMLVGRIVSGAETAPNFNVSLQSLINIDK
ARATH*  NLLNNLSQVRQVMSSAALAGAAS----SVGQSAVAMHMSNMISTGMATSL
VITVI   SILNNLSQARQVMHSAALSGGTSIGLQSMGGTSMAMHMSNMISSGMASSV
SOYBN   NILNNLSQARQVMNSAALSGGTSMGLPSMGQTPVAMHMSNMISSGMTSSV
SOLLC   KILNGVAQAHQV-----LGGGTSIGLQSMGGTPM---LSSMISSGMASSV
ORYSJ*  SILNNLSQHR--------------------------SVATIISGGMPGI-
HORVU   SILNNLSQHRHSLT-----AATSMG-PNMGATPIQVHMSNMLSSGMTST-
SORBI   SLLNNLSQVRLMNSTSLGGGATSMGLPNMGATPIQVHMSNMISSGMTST-
SELML   --ASVLNQIRQGAGSAAVSSILSAGSLAVG------------QAGMGITQ
PHYPA   GHVGTLTQPHKMPGGAVANGGPAVP----SGASSNPTAPSSLTSGPATII
VOLCA   GAVSPFANAGEAVAAAAAAAAAAAPGGAANGL-VAVHAAARQHGAVAAAA
               660       670       680       690       700

HUMAN*  --GVGPPFSQAPAPQLPPG-------PPGAPKPP----PASQPSLVSTVA
CHICK   -RANPQPFLVPP--------------------------------------
XENTR   TSAVGTPFNQPPPPAMPQNTSVPKMVTSTASLMT----PASQPSLVTTVT
DANRE   ----TPPFSQSPVLPPGGKPSLSTVTTVSPPVLTQQQVPPQQPQQQTQVP
CIOIN   -------------------------------------------TVSDTMT
TENG    --------------------------------------------------
DROME   PGQARPPFMQGAGNVGGVGQGGGMQQNPNSALISRINAPPPNQTVTSLQQ
RHIO9   TALVKLSEQIRRSTAARAAAGNPMSQMN-------SNAAVATTTTSSPFN
CRYNE   -------------------------------------------------S
USTMA   EAREMGVNLGGPSGGGAA-------------------------ALVRPAR
ARATH*  PPSQ-TVFSTGQQGITSMAGSGALMGSAQTGQ---SPGPNNAFSPQTTSN
VITVI   PATQ-TVFSSGQSAVSSITGSGTLAGTAQVAQ---NSALGSFTSATSNMS
SOYBN   PAAQ-NVFSSGQSGITSMTSSGPLTVPAQVGQ---NSGLGSLTSNTSNLS
SOLLC   PASQ-AVLSSGQSGVTTMTGAVPLAGSAQNTQ---NSAPSSFTSTAPSMS
ORYSJ*  -----------P-----MSGTGQSIGSQQVVQ---NTA---FGSNTP-IT
HORVU   -----------PAVISSISGTVQPIGAQQLVQ---NTALGSFGSNTSTVS
SORBI   -----------PSVISSMSGPGQPIGTQQMVQ---STALGSFGSNTSTVS
SELML   PVSQPASLAHGLSGDAGSASLGPVQGNANTSSILPSNAPAGMMQPASMPS
PHYPA   P---------GNGNVLTTIGSATTVGMNTGIG---HSSIGTLSANANTIA
VOLCA   AAAANMMGQYGQGPAAAAGTPYNVAGSTPGQTAAAAAAAAAAAAAAAPNA
               710       720       730       740       750

HUMAN*  PGSGLAPT--------AQPGAPSMAG------------------------
CHICK   --------------------------------------------------
XENTR   TGPGPAPVQLQQQGAQQQPVPPSMPI------------------------
DANRE   QPGLPAVNQQPPQASQQQP-------------------------------
CIOIN   KTSAVATQNIQPQQISQTPSSMMQPM------LTTASTSSVTNPIRNIAS
TENG    --------------------------------------------------
DROME   QQQQQAQQQQQQQQQAQQQQQQRMQM------LSQQQMLNHQQLQQQQQL
RHIO9   SNAPTATTDNLMQHQNQINMASINPSQIPANLTPQQRMLLATQLAQR---
CRYNE   SLEAGLGSSFNPATLSKMDPQLIASMLTAIKAQNEQQGVGDQRRSQVQQL
USTMA   STSVTAASQPPVASLQQVPSSVAKPAVTALSSASGDAATTANAS------
ARATH*  VASNLGVSQPM-------------QG------MNQGS--HSGAMMQG-GI
VITVI   VNSNLGISQPLSNLQGGVSMGQTVPG------MSQGN-LPGGQMVQS-GI
SOYBN   SSSNIGISQPLGNLQGVVSIGQQVSG------MSQGN-LSGAQMVQG-GV
SOLLC   --------------------GQTVPA------MSQGN-IPGTQMMPS-GT
ORYSJ*  GNSNIAVSSSLGGIQSNIGI--SGPP------VTQGGSMGSTQLGQG-GI
HORVU   GNSNIAVSSSLANIQSNMAMGQSVPS------MAQGGLMAGSQSGQG-GI
SORBI   GNSNVAVSSSLTNNQSSMGMGQSVQP------VAQGGLVAGSQLGQG-GI
SELML   GLPPVAST---------VGMAQSLQA--------ANPLVVGGQSVQGAGL
PHYPA   SSNLVGPTGSLVPNQLGPSTVSPLQQ-------------GVGQLPVS-GA
VOLCA   ATSPTPYYHPSPQYRAAAAAAAAAVA------------------------
               760       770       780       790       800
```

MED25

```
HUMAN*  --------------------------TVAPGGVSGPSPA-----------
CHICK   --------------------------------------------------
XENTR   --------------------------TGAAGGVQAPQPS-----------
DANRE   ------------------------------NNQQTPPPT-----------
CIOIN   GDSLSQISSQPSFQSVVPQPSINTFPSTSGNTDPNPSSTADSPY------
TENG    --------------------------------------------------
DROME   AQQQQQQQQGQQQQQVNPNAGNNMMPASNAGNMSNPQQQQQQVG------
RHIO9   -------------------------NVAAGKPS-----------------
CRYNE   LEMQQKVLAAKAAQSRGKNGQGETVMAAI---------------------
USTMA   -------------------------ATTSGAGGVQPPP------------
ARATH*  SMNQN-----------MMSGLGQG-NVSSGTGGMMPTPGVGQQAQSGIQQ
VITVI   GMNQN-----------MMSGLGPS-GISSGTGTMIPTPGMSQQVQPGMPS
SOYBN   SMNQN-----------VMSGLGQS-VVSSGTGTMIPTPGMSQPVQSVMQP
SOLLC   GMNQN-----------MLTGLGAT-GLPSGTGTMMPTPGMSQQGQPGMQP
ORYSJ*  NTNQN-----------MISSLGTT-TVSS-APAMMPTPGMAQQA--GVNS
HORVU   GTNQN-----------MINNLGTT-AISS-TPTMMPTPGMVQQT--GVNA
SORBI   GANQN-----------VMSSLGST-AISS-APAMMPTPGMVPQT--GVNS
SELML   GMPHS-----------SIAAPSNSVGSAPNAGVLLPMSQPGQNPATGNAG
PHYPA   GQPQAKPLGAIAGMGVTTTALGTA-GVNAGVNSMPPSSGFVQPVQP-VQP
VOLCA   --------------------------AAAGGRPVGPVQQPQQQPPSAGVL
                  810       820       830       840       850
```

```
HUMAN*  ------QLG-----APALGGQQS----------------VSNKLLAWSGV
CHICK   --------------------------------------------------
XENTR   ------QPQIGT--AQLPCTQTP---------------VNGNKLLAWSGV
DANRE   ------QPGMAAVPAPPPGAQQG----------------VANKVVAWSGV
CIOIN   ------AAAKKEAQRVVQIAQKG-----------------SAPKIIWNGA
TENG    --------------------------------------------------
DROME   ------QQSNPQQQGNPQQQQQGNSQQE----------QASLREKIWTGV
RHIO9   ------NINELQAQLLQQQQQQQGGN------------DAVSTQPLWTGQ
CRYNE   -------------------QQQRAQQAQASGQPTQVHSVQPRSQTIWSGA
USTMA   --------------------------------------SPTRPKPFWRGA
ARATH*  LG----GSNSSAPNMQLS-QPSSGAMQT----------SQSKYVKVWEGN
VITVI   LG----VNNNAAANMPLP-QQTSGAMQT----------AQSKYVKVWEGN
SOYBN   L------VNNAAANMPLS-QQTSGGMQS----------AQSKYVKVWEGS
SOLLC   VG-----VNSTSANMPLSQQQTSGALPS----------AQSKYVKVWEGN
ORYSJ*  LG----VTNSSAMNMPIV-QHPNAQQQQQQQQQQQQQQPPPKYVKIWEGT
HORVU   LS----VNNSSAMNMPMP-QHPNGQQ-------------PSKYVKIWEGT
SORBI   LG----VNNNPAMNMPIP-QHANAQQ------------PPPKYVKIWEGT
SELML   VS----HLVSNGVATTPP-QQP----------------ANTKYTKLWQGT
PHYPA   LAPGIHGVTQTAQSATPP-QQPSGSM---------------KYTKLWEGI
VOLCA   HQFVQAQAAAAAAAAMAAAKMQGQGMPQNLTKATPQPQPRPAAAGAGAGR
                  860       870       880       890       900
```

```
HUMAN*  LEWQE---KPKPASVDANTKLTRSLPCQVYVNHGEN-------LKTEQWP
CHICK   --------------------------------------------------
XENTR   LEWQE---KPRSVSVDNNAKLTRSLPCQVYVNPGEN-------LKTDQWP
DANRE   LEWQE---KPKASSMDSNTKLTRSLPCQVQVSQGEN-------LNTDQWP
CIOIN   LEWKQTRRQPGTAQGQAVPRIVHTVRCQIIQQQSNP----TPEIDTNGWP
TENG    --------------------------------------------------
DROME   LEWSE---KPKSDQ----QKIPHTLQCTVCTNIKDG----EPEIKAENWP
RHIO9   IVWH--------IKTPEGQLQEFNCLCGAFAIPTKGSITNIEEYRPEVWP
CRYNE   IVWG----------APGGSGSNASGSVGVEAQLFSGI---PDSVMVSHWP
USTMA   LTWS--------VISDPATKQKRDVATLVSATSNSS----QLERLLMPWP
ARATH*  LSGQ--------------RQGQPVLITRLEGYRSAS----ASDSLAANWP
VITVI   LSGQ--------------RQGQPVFITRLEGYRSAS----ASESLAANWP
SOYBN   LSGQ--------------RQGQPVFITKLEGYRNSS----ASETLAANWP
SOLLC   LSGQ--------------RQGQPVFITRLEGYRSAS----ASESLAANWP
ORYSJ*  LSGQ--------------RQGQPVFICKLEGYRSGT----ASETLAADWP
HORVU   LSGQ--------------RQGQPVFICKLEGYRSGT----ASDALASDWP
SORBI   LSGQ--------------RQGQPVFICKLEGYRSGT----ASETLAADWP
SELML   LAGQ--------------RHGKPVPICSLEGYRQIS----SPETLAADWP
PHYPA   LAGQ--------------RQQKPVPICKLEGYRQTS----SSEKLAADWP
VOLCA   LGFT-------------QQEGYQYPIFQVQLSREGSISLHPPSSGQHAHP
                  910       920       930       940       950
```

```
HUMAN*  QKL---------------IM--------QLIPQQLLTTLGPLF-------
CHICK   --------------------------------------------------
XENTR   QKL---------------IM--------QLIPQQLLTTLGPLF-------
DANRE   QKL---------------IM--------QLIPQQLLTTLGPLF-------
CIOIN   QKL---------------MV--------QFIPQSFLSHQAVHMQLQP---
TENG    ----------------------------PIMPQPPVP-------------
DROME   PKL---------------LM--------QLMPKHLVGNIGGQFL------
RHIO9   SRI--------------QIAGRSPLSRGNVLHQQALE-------------
CRYNE   KEL---------------LL--------RNLAIIHLPS-------LTEYA
USTMA   DKL--------------QIT--------AITQLSPRN--------LQVYA
ARATH*  PTM--------------QIV--------RLISQDHMN--------NKQYV
VITVI   ATM--------------QIV--------RLISQDHMN--------NKQYV
SOYBN   PVM--------------QIV--------RLISQDHMN--------NKQYV
SOLLC   PTM--------------QIV--------RLISQDHMN--------NKQYV
ORYSJ*  ETM--------------QIV--------RLIAQEHMN--------NKQYV
HORVU   ETM--------------QIV--------RLIAQEHMN--------NKQYV
SORBI   ETM--------------QIV--------RLIAQEHMN--------NKQYV
SELML   LTM--------------QIV--------RLIPQDYMS--------NREYQ
PHYPA   PTM--------------QIV--------RLIAQEYMN--------SKEYQ
VOLCA   GAMLDALFDGFTKCTYASMVRTVPNEEMPAVRQAIVADVRLEVLMVTEYA
                  960       970       980       990      1000
```

MED25

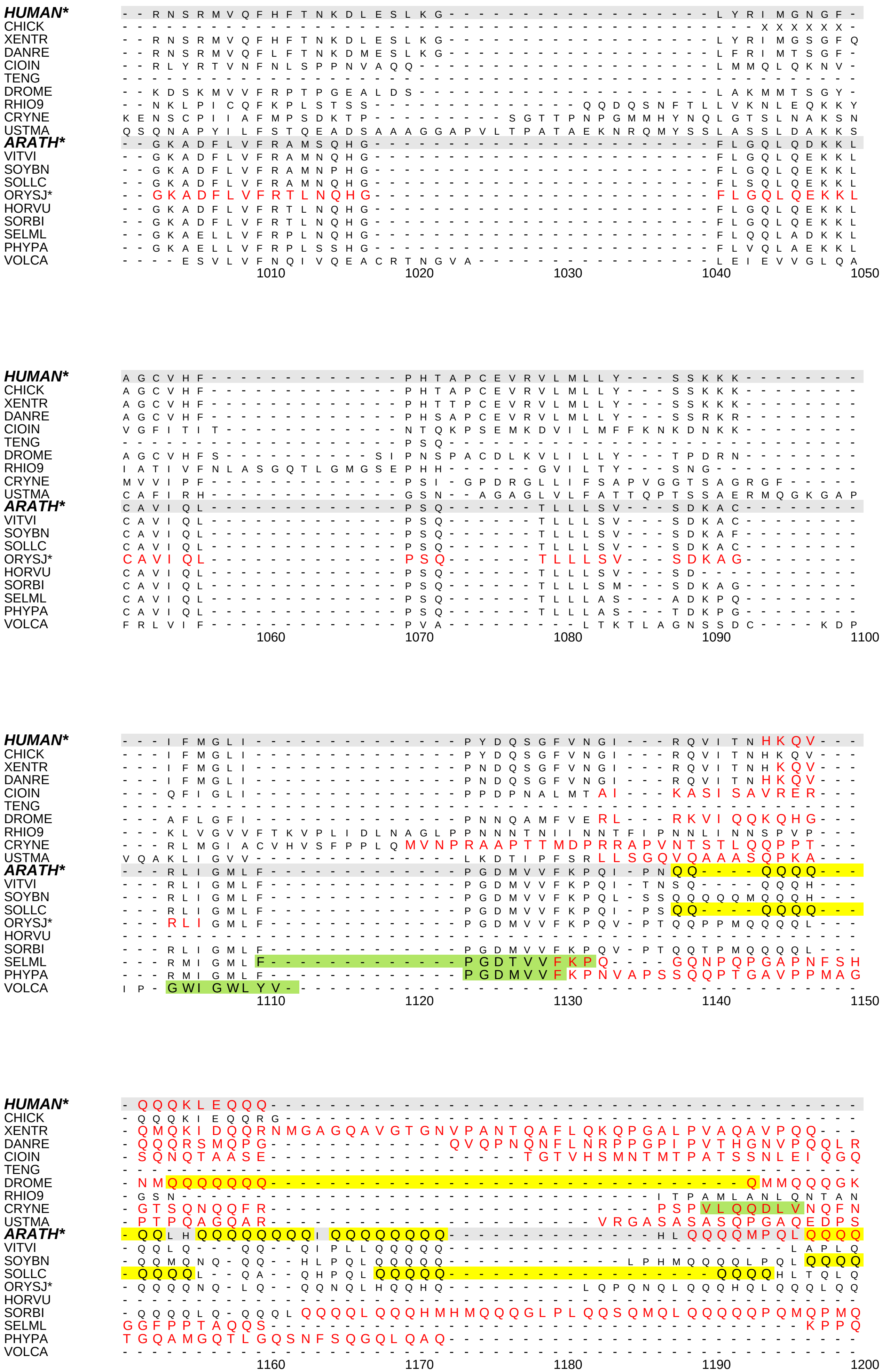

HUMAN*
CHICK
XENTR
DANRE
CIOIN
TENG
DROME
RHIO9
CRYNE
USTMA
ARATH*
VITVI
SOYBN
SOLLC
ORYSJ*
HORVU
SORBI
SELML
PHYPA
VOLCA
1010 1020 1030 1040 1050
1060 1070 1080 1090 1100
1110 1120 1130 1140 1150
1160 1170 1180 1190 1200

MED25

```
HUMAN*  RGMGGQQAPPGLGPI----LEDQARPSQNLLQLRPPQ-------------
CHICK   --------------------------------------------------
XENTR   --MQGQQVAPGMPSISQVTMMDEQQRTQNLLHIRVQQ-------------
DANRE   AAATGNQQAP----------------------------------------
CIOIN   PNALPGTTNQQLSMQQNFNRVQPQQQKPNTGSIQFQQ-------------
TENG    -------QPPGPPPPQPVPSQPPPPQ------------------------
DROME   SPMELQQQQQQQQQQQQMQQDNSQQQHYNQFQLNMQMGGGGPGGGPGPGP
RHIO9   MGLLNTTTTPTANNSNVQHIQNMRQQQQMMNTTGI---------------
CRYNE   QGQSQGQATPAAQFAALMQQQAQAQSQTQSRVLQSQPPQLTPQGQNPPIN
USTMA   ALPAPPSTQPQPPPQQPQPQPQPQPQ------------------------
ARATH*  --Q---QHQQQQQQQHQLSQLQHHQQQ-----------------------
VITVI   --QQ--QQLPQLQQQQQLPQLQQQQQ---------LQ-------------
SOYBN   --Q---QLPQLQQQQQQLPQLQQQQQQLPQLQ---QQ-------------
SOLLC   --QQPLQQLQQQQQQQPLMQLQQQQQIP-------LQ-------------
ORYSJ*  --Q---QLQQHMQLQTQGLPLQQQQSQGHPLQ--QQQ-------------
HORVU   --------------------------------------------------
SORBI   --QQPTQMQHQQQQPSQMQPMQHQQPQPQQMQSMQHQ-------------
SELML   MMPGGSQMPMAGSGGTQIPGQNVHASVMVAITNSIS--------------
PHYPA   ------MHPQQLNAAQQMTAQQLNAQQMGPQQLNSQQ-------------
VOLCA   --------------------------------------------------
                 1210      1220      1230      1240      1250
```

```
HUMAN*  ---PQPQGTVGASGATGQPQPQGTAQPPPGAP------------------
CHICK   --------------------------------------------------
XENTR   ---PQ------QAASQAPPQATQTTVQAPGQP------------------
DANRE   -------------VTGAPPNQVQGQAQAPG--------------------
CIOIN   ---PRPQYHVQQNISQGMQM------------------------------
TENG    ---QQPPVPTQPPVPPAQPNMXXXXXXSLSLS------------------
DROME   GGMPMQQNQMQMNMMQQQRM------------------------------
RHIO9   ---VNPQQQANAPVTASINGLNPNSLLLSNLN------------------
CRYNE   SSLPHPQNTTRGITFQQYQQILSHAQ------------------------
USTMA   ---PQPQPQPQPQPQPQSTGMGLGSSMPFNLAALNAAGNVPISGLGAPGT
ARATH*  ---QQQQQQQQ---------------------------------------
VITVI   ---QQQHPQLQ---------------------------------------
SOYBN   ---QQQLPQLQQ--------------------------------------
SOLLC   ---QSQVPQM----------------------------------------
ORYSJ*  ---MQQMQQQQ---------------------------------------
HORVU   --------------------------------------------------
SORBI   ---QQMQHQQQ---------------------------------------
SELML   ---IAAIPLLTSAGRIPKRAIYLAMI------------------------
PHYPA   ---LNAAQQMNTQQQMNAQQMGVQQMNPQQLN------------------
VOLCA   --------------------------------------------------
                 1260      1270      1280      1290      1300
```

```
HUMAN*  --------------------------------------------------
CHICK   --------------------------------------------------
XENTR   --------------------------------------------------
DANRE   --------------------------------------------------
CIOIN   --------------------------------------------------
TENG    --------------------------------------------------
DROME   --------------------------------------------------
RHIO9   --------------------------------------------------
CRYNE   --------------------------------------------------
USTMA   MGGAMNLNSFLPNLATSQSTHSQPQPVGATNIPDLTNLAHLGNMANLNLG
ARATH*  --------------------------------------------------
VITVI   --------------------------------------------------
SOYBN   --------------------------------------------------
SOLLC   --------------------------------------------------
ORYSJ*  --------------------------------------------------
HORVU   --------------------------------------------------
SORBI   --------------------------------------------------
SELML   --------------------------------------------------
PHYPA   --------------------------------------------------
VOLCA   --------------------------------------------------
                 1310      1320      1330      1340      1350
```

```
HUMAN*  ----------------QGPPGAASGPPPPGPILRPQNPGANPQLRSLLLN
CHICK   --------------------------------------------------
XENTR   ----------------QN--------PQPGAMLRPQNPGANPQLRNLLLS
DANRE   -----------------------------GGMLRLQNPGANPQLRSLLLS
CIOIN   -----------------------SMANPVSRLNQPIQQGHRMSGMTISPT
TENG    -------------------------------------------------L
DROME   ----------------PLGVGVGVGVGPGGVPNPNLQQQLQQVAPNVAAM
RHIO9   --------------------LTNTSGNNNNNNNTNLLNINNINMANLANL
CRYNE   ----------------KLGIVLPAFDYTSIPQDKLQTLINGLKMAEMKQR
USTMA   QFGIPNQQLQNAFTAAAAGAGAGAAPATQQPNLAALAQLLGFGTQSMTQQ
ARATH*  --------------------------------------------------
VITVI   --------------------------------------------------
SOYBN   --------------------------------------------------
SOLLC   --------------------------------------------------
ORYSJ*  --------------------------------------------------
HORVU   --------------------------------------------------
SORBI   --------------------------------------------------
SELML   -----------IVVLTTSLSLCLEAKLTADVGVSRDFGCVDWSLASREAI
PHYPA   ----------------AAQQMNTQQQLNAQQMGVQQMNPQQLNAAQQMNT
VOLCA   --------------------------------------------------
                 1360      1370      1380      1390      1400
```

MED25

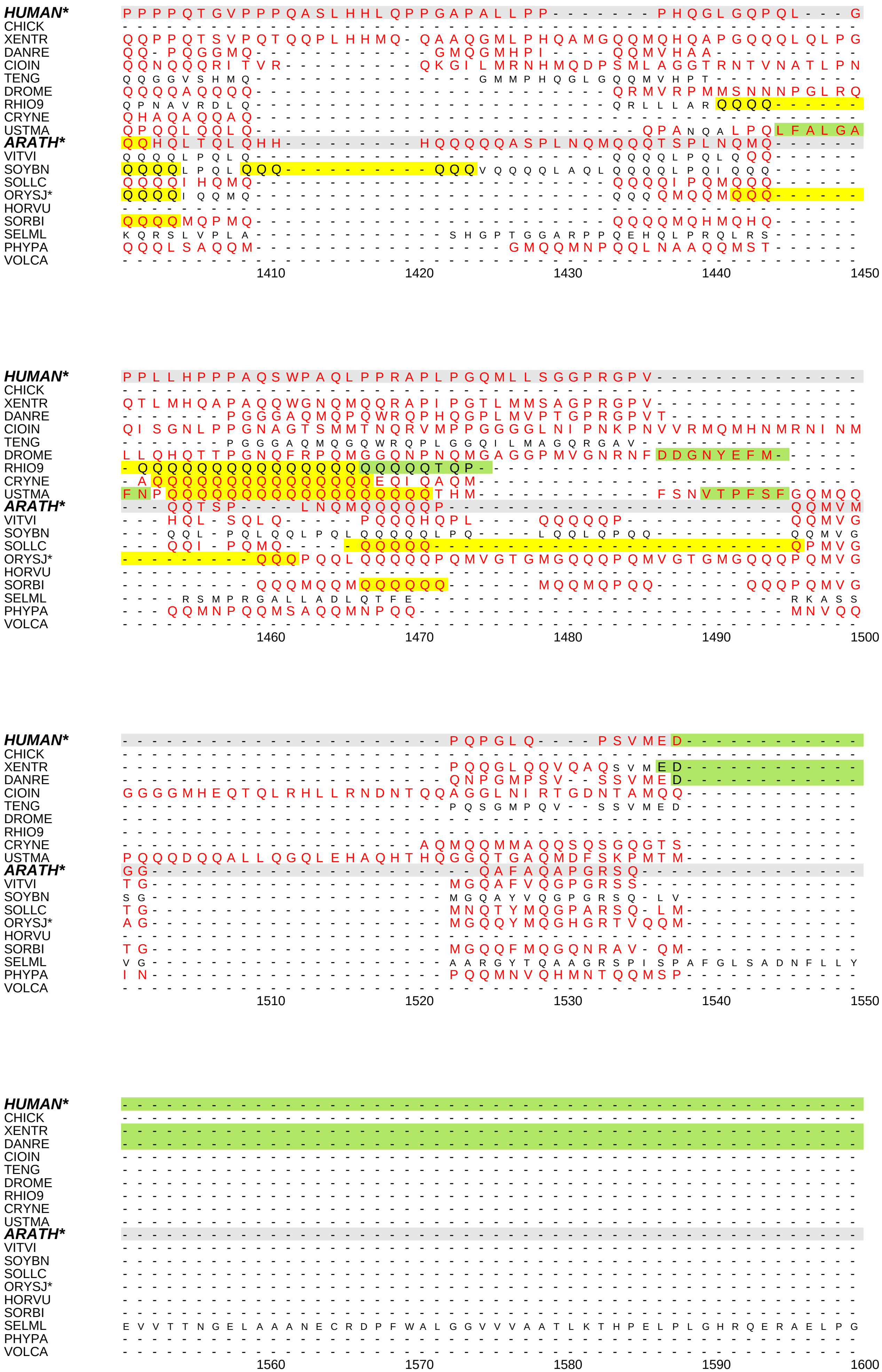

MED25

```
HUMAN*  --------------------------------------------------
CHICK   --------------------------------------------------
XENTR   --------------------------------------------------
DANRE   --------------------------------------------------
CIOIN   --------------------------------------------------
TENG    --------------------------------------------------
DROME   --------------------------------------------------
RHIO9   --------------------------------------------------
CRYNE   --------------------------------------------------
USTMA   --------------------------------------------------
ARATH*  --------------------------------------------------
VITVI   --------------------------------------------------
SOYBN   --------------------------------------------------
SOLLC   --------------------------------------------------
ORYSJ*  --------------------------------------------------
HORVU   --------------------------------------------------
SORBI   --------------------------------------------------
SELML   WATSSDLSRYSTFQRSLEPLRRGLTWSTLVDHFFFLRGELRRHLRWEPFS
PHYPA   --------------------------------------------------
VOLCA   --------------------------------------------------
                 1610      1620      1630      1640      1650
```

```
HUMAN*  ------------------------------------DILMDLI-------
CHICK   --------------------------------------------------
XENTR   ------------------------------------DILMDLI-------
DANRE   ------------------------------------EILMDLI-------
CIOIN   ------------------------------------PAQSNLPVQQQQTY
TENG    ------------------------------------EILMDLI-------
DROME   --------------------------------------------------
RHIO9   --------------------------------------------------
CRYNE   ------------------------------------GAGIGLRNFQPGP-
USTMA   ------------------------------------DQLRALGFLS----
ARATH*  ---------------------------------------QGGG-------
VITVI   --------------------------------------------------
SOYBN   ------------------------------------SQGQVSS-------
SOLLC   ------------------------------------SQSQGSS-------
ORYSJ*  ------------------------------------MQGKMAP-------
HORVU   --------------------------------------------------
SORBI   ------------------------------------MQGKIMP-------
SELML   GFQADPARDPLELLLSPLDELGRGCYRVSGMKLREISQVQTSC-------
PHYPA   ------------------------------------QQARPQI-------
VOLCA   --------------------------------------------------
                 1660      1670      1680      1690      1700
```

```
HUMAN*  ----------------------------------------
CHICK   ----------------------------------------
XENTR   ----------------------------------------
DANRE   ----------------------------------------
CIOIN   MQRPNLMPGMQNNQNNQRRY--------------------
TENG    ----------------------------------------
DROME   ----------------------------------------
RHIO9   ----------------------------------------
CRYNE   ----------------------------------------
USTMA   ----------------------------------------
ARATH*  ---------------------GGQPNMPGAGFMG------
VITVI   ---------------------QGPPNMPGGAFLG------
SOYBN   ---------------------QGATNIGGGGFMS------
SOLLC   ---------------------QGLPITPGGGFMN------
ORYSJ*  ---------------------QGPGSMPGAGSMPGGGYLS
HORVU   ----------------------------------------
SORBI   ---------------------QGPGSMPGGGFLP------
SELML   ----------------------------------------
PHYPA   --------------------MPGPASLQGSGYLP------
VOLCA   ----------------------------------------
                 1710      1720      1730      1740
```

MED26

```
HUMAN*  --------------------------------------------------
CHICK   --------------------------------------------------
XENTR   --------------------------------------------------
DANRE   --------------------------------------------------
CIOIN   --------------------------------------------------
TENG    --------------------------------------------------
DROME   --------------------------------------------------
BRUMA   --------------------------------------------------
CAEEL   --------------------------------------------------
ARATH*  MAKMRPSVSLDTWR------------------------------------
VITVI   -------MDRDEFR------------------------------------
SOYBN   -------MDLDDFR------------------------------------
SOLLC   --MAKSSGTLDKWR------------------------------------
ORYSJ*  MAAAVR--------------------------------------------
HORVU   ----MADDGLDRWR------------------------------------
SORBI   ---------MDRGQR-----------------------------------
SELML   ---------MDKWK------------------------------------
PHYPA   MCSMPSKIYFQEASSGLHANLDRKKWKPTFYQQSKRLKPNGLRTQQYKEK
VOLCA   --------------------------------------------------
                 10        20        30        40        50

HUMAN*  --------------------------------------------------
CHICK   --------------------------------------------------
XENTR   --------------------------------------------------
DANRE   --------------------------------------------------
CIOIN   --------------------------------------------------
TENG    --------------------------------------------------
DROME   --------------------------------------------------
BRUMA   --------------------------------------------------
CAEEL   --------------------------------------------------
ARATH*  --------------------------------------------------
VITVI   --------------------------------------------------
SOYBN   --------------------------------------------------
SOLLC   --------------------------------------------------
ORYSJ*  --------------------------------------------------
HORVU   --------------------------------------------------
SORBI   --------------------------------------------------
SELML   --------------------------------------------------
PHYPA   EEDVGEDKDEREGKERKERKKERGTGRGTGGWERNGHLHHMGGVGVVVVR
VOLCA   --------------------------------------------------
                 60        70        80        90       100

HUMAN*  --------------------------------------------------
CHICK   --------------------------------------------------
XENTR   --------------------------------------------------
DANRE   --------------------------------------------------
CIOIN   --------------------------------------------------
TENG    --------------------------------------------------
DROME   --------------------------------------------------
BRUMA   --------------------------------------------------
CAEEL   --------------------------------------------------
ARATH*  --------------------------------------------------
VITVI   --------------------------------------------------
SOYBN   --------------------------------------------------
SOLLC   --------------------------------------------------
ORYSJ*  --------------------------------------------------
HORVU   --------------------------------------------------
SORBI   --------------------------------------------------
SELML   --------------------------------------------------
PHYPA   VWGPSMWSRTREGKGRKRKGEERKDREHRWPARSTSRAQSTRPVLAVADP
VOLCA   --------------------------------------------------
                110       120       130       140       150

HUMAN*  --------------------------------------------------
CHICK   --------------------------------------------------
XENTR   --------------------------------------------------
DANRE   --------------------------------------------------
CIOIN   --------------------------------------------------
TENG    --------------------------------------------------
DROME   --------------------------------------------------
BRUMA   --------------------------------------------------
CAEEL   --------------------------------------------------
ARATH*  --------------------------------------------------
VITVI   --------------------------------------------------
SOYBN   --------------------------------------------------
SOLLC   --------------------------------------------------
ORYSJ*  --------------------------------------------------
HORVU   --------------------------------------------------
SORBI   --------------------------------------------------
SELML   --------------------------------------------------
PHYPA   VPRNSHAGHGHEQRGHRHGMTHGHGWVCYPGLRELLVVLGESRRGGAALG
VOLCA   --------------------------------------------------
                160       170       180       190       200
```

MED26

```
HUMAN*  --------------------------------------------------
CHICK   --------------------------------------------------
XENTR   --------------------------------------------------
DANRE   --------------------------------------------------
CIOIN   --------------------------------------------------
TENG    --------------------------------------------------
DROME   --------------------------------------------------
BRUMA   --------------------------------------------------
CAEEL   --------------------------------------------------
ARATH*  --------------------------------------------------
VITVI   --------------------------------------------------
SOYBN   --------------------------------------------------
SOLLC   --------------------------------------------------
ORYSJ*  --------------------------------------------------
HORVU   --------------------------------------------------
SORBI   --------------------------------------------------
SELML   --------------------------------------------------
PHYPA   ACDAGLCCWRWAGGVEVCLVPQGVEFRARKCGWRSRGVVFALGHGCKGRV
VOLCA   --------------------------------------------------
                       210       220       230       240       250
```

```
HUMAN*  --------------------------------------------------
CHICK   --------------------------------------------------
XENTR   --------------------------------------------------
DANRE   --------------------------------------------------
CIOIN   --------------------------------------------------
TENG    --------------------------------------------------
DROME   --------------------------------------------------
BRUMA   --------------------------------------------------
CAEEL   --------------------------------------------------
ARATH*  --------------------------------------------------
VITVI   --------------------------------------------------
SOYBN   --------------------------------------------------
SOLLC   --------------------------------------------------
ORYSJ*  --------------------------------------------------
HORVU   --------------------------------------------------
SORBI   --------------------------------------------------
SELML   --------------------------------------------------
PHYPA   ESRRMCEFVLFLCFLAHDRVVGFSDGVCCSLHAVMWHEAGIVVNICKIIR
VOLCA   --------------------------------------------------
                       260       270       280       290       300
```

```
HUMAN*  --------------------------------------------------
CHICK   --------------------------------------------------
XENTR   --------------------------------------------------
DANRE   --------------------------------------------------
CIOIN   --------------------------------------------------
TENG    --------------------------------------------------
DROME   --------------------------------------------------
BRUMA   --------------------------------------------------
CAEEL   --------------------------------------------------
ARATH*  --------------------------------------------------
VITVI   --------------------------------------------------
SOYBN   --------------------------------------------------
SOLLC   --------------------------------------------------
ORYSJ*  --------------------------------------------------
HORVU   --------------------------------------------------
SORBI   --------------------------------------------------
SELML   --------------------------------------------------
PHYPA   NRKVRAVEAMTLIVTRRRNGSKVCFLCVVSDLSLKKTLNIVLRNLTESFI
VOLCA   --------------------------------------------------
                       310       320       330       340       350
```

```
HUMAN*  --------------------------------------------------
CHICK   --------------------------------------------------
XENTR   --------------------------------------------------
DANRE   --------------------------------------------------
CIOIN   --------------------------------------------------
TENG    --------------------------------------------------
DROME   --------------------------------------------------
BRUMA   --------------------------------------------------
CAEEL   --------------------------------------------------
ARATH*  ----------------------------EYFRRGDSDIFGIIDHAIMVAA
VITVI   ----------------------------LILRNSGVDVWEFINTAISVAY
SOYBN   ----------------------------SILDTAGVDVWMFIDAAIAVAS
SOLLC   ----------------------------DYFRTANSDIFDIIEYAVMVAA
ORYSJ*  ----------------------------EMVRSMGAEQ---LDEAIAFAT
HORVU   ----------------------------GFFRGAGVGICEVIEKAILVAA
SORBI   --------------------------LGRALDAFGGDMWALVDAALDAAA
SELML   ----------------------------SFFQSSGSDIWTVIDRAITIAA
PHYPA   LGMVKEVWGRVEVLAVAPCVLRSPVVRVEFLNGAGADIWTVIDNVLDIAA
VOLCA   --------------------------------------------------
                       360       370       380       390       400
```

MED26

```
HUMAN*  --------------------------------------------------
CHICK   --------------------------------------------------
XENTR   --------------------------------------------------
DANRE   --------------------------------------------------
CIOIN   --------------------------------------------------
TENG    --------------------------------------------------
DROME   --------------------------------------------------
BRUMA   --------------------------------------------------
CAEEL   --------------------------------------------------
ARATH*  ADWPKEFKSRSDRIAELLFSCKVSR--CIGCDHLELSIAGDEAAVEIVGV
VITVI   SDYGDELRHRRDGIVERMYMSS-------RCGSCNLRYQHSG--------
SOYBN   SDSAGDLKRRRDGIVERLYAATAAPPWCRNCDEGGHRLNGHR--------
SOLLC   VDCPKEFKMRRDRIAEMLFTCKVTM--CFGCEKVDLAVPIAG--------
ORYSJ*  MELAGR--------------------------------------------
HORVU   ADEPQEFLRRRDRIAERLFNALLARPSCHGCTASTGSVPPATPAVAEDKG
SORBI   RDRPDDLRARRDGIVERLYAAATA-----GCSNCAGRPPPLAA-------
SELML   VEQPGELRARRDKFTEKMFCPTAHLTGLCECDDGEGEEEENGGGAMSRSD
PHYPA   TDYPHELRIRRDGFVEKLFAHPALLSKSNGC-------------------
VOLCA   --------------------------------------------------
               410       420       430       440       450
```

```
HUMAN*  --------------------------------------------------
CHICK   --------------------------------------------------
XENTR   --------------------------------------------------
DANRE   --------------------------------------------------
CIOIN   --------------------------------------------------
TENG    --------------------------------------------------
DROME   --------------------------------------------------
BRUMA   --------------------------------------------------
CAEEL   --------------------------------------------------
ARATH*  GG------------------------------------------------
VITVI   --------------------------------------------------
SOYBN   --------------------------------------------------
SOLLC   -----------------------------------------------NDD
ORYSJ*  --------------------------------------------------
HORVU   SVRRVPDKECKVDSSSLGAPGRGGLGGGGGISEDDDDSDSDSEDDERLRR
SORBI   --------------------------------------------------
SELML   GGDGEEDEDEDEEDDVAENKLVVSGGGALREDEEDLEEEEEVEVVDEEED
PHYPA   -----------------------------------LPVVACHDVEEAHDD
VOLCA   --------------------------------------------------
               460       470       480       490       500
```

```
HUMAN*  --------------------------------------------------
CHICK   --------------------------------------------------
XENTR   --------------------------------------------------
DANRE   --------------------------------------------------
CIOIN   --------------------------------------------------
TENG    --------------------------------------------------
DROME   --------------------------------------------------
BRUMA   ----------------------------------------------MQQQ
CAEEL   --------------------------------------------------
ARATH*  -----------------------------------GGDRGDSGVATGEGE
VITVI   ---------LDKGCDEEKEDEK-------------DDEKDKSPLTPQSIN
SOYBN   ---------IKKQSRHSPSPERQPH----------RSTATASPATPQSLD
SOLLC   EGKIKSKDEFGGSKESKVNSRIDHHDVEMNVNQVSNYSYGEAEALTEEME
ORYSJ*  -----------------------------------DIPFEDVFRLCDEQE
HORVU   AAASNYGHNYDDDNDDDEEQDAAAPAEEDHHAEDDDPEAEELEALTNEID
SORBI   ---------LAAAGLDEEDGEEAAP----------ASSAVALEAEADAQA
SELML   QEEEHDDQDDNADQEQSRNRRKSKA----------DCSYMDVEALTDEME
PHYPA   KGVDPPDYDYDHVRTELSDDEDRLVNS--------TEEYDEAEALNDAME
VOLCA   --------------------------------------------------
               510       520       530       540       550
```

```
HUMAN*  -----------------MTAAPASPQQIRDRLLQ--AIDPQSNIRNMVAV
CHICK   -------------------------------------LSSLLQIHNMVAV
XENTR   -----------------MTAAPVTPREIRERLLQ--AIDTQSNIHNMVAV
DANRE   -----------------MTTASATPQQMRDRLLQ--AIDSHSNICNMVAV
CIOIN   --------------------MPPTVVQLTHRLLQ--IINKDGTISDQDVA
TENG    -----------------MTTVSATPQQMKDRLLQ--AVDSQSNICNMVVV
DROME   -------------------MNQIEIQELTTHLSQ--ALDQNYDVVNMDAV
BRUMA   HPAAASNSGLSVAVPTVEPTTVTVVDRLKHQLLQ------AYDNGDTKQA
CAEEL   -----------------MAASAQNVEEIKKRLQI------AAENDDQTLC
ARATH*  ----EAS------------VSVDEVMRIRDILSN-------KDDEKDSVL
VITVI   RSGSEDR------CGGLPDEEEARILDIKEHLEA--------PDQPDDSI
SOYBN   NGEDEDNDEDLDPYGGLLDDEQKKILEIKEQLEE--------PDQSEDSL
SOLLC   ----EET------------QTFGEVMRIKDVIDN--------NQAESAEL
ORYSJ*  LRRAKKS------------SMAEEVERIKGKLVGGEDGGRPSSDSSEETV
HORVU   ----EES------------QILGEVLRIKELLLH-------KQDHSDTTL
SORBI   GGAEERAEELGAGAPGL----ESKIVAIRDFLDD--------PNQPEDEL
SELML   ----EES------------RRIKEINRIKNQISH--------FADEEDYL
PHYPA   ----EEA------------RQTLEVLRVKDILAD--------SSQDESAI
VOLCA   ----------------MGTLSEADLNTCLKTLESCGRGAADGDRADESRM
               560       570       580       590       600
```

MED26

```
HUMAN*  LEVISSLEKYPITKEALEETRLGKLINDV-RKKTKNE--ELAKRAKKLLR
CHICK   LEVISSLEKYPITKEALEETRLGRLINEV-RKKTSNE--ELAKRAKKLLR
XENTR   LEVISSLEKYPITKEALEETRLGKLINDV-RKKTSNE--ELAKRAKKLLR
DANRE   LDVITNLEKYPITKEALEETRLGKLINDV-RKKTKDE--DLAKRAKKLLR
CIOIN   LEIIAMLETTVISCEELEETRLGKYINDV-RRAATDT--SIQRRAKKLIK
TENG    LEVISCLEKYPITKEALEETRLGKLINDV-RKKTKDE--DLAKRAKKLLR
DROME   LCVICALEGTTITKEQLEATRLAKYINQL-RRRTKNE--HLARRAKSLLK
BRUMA   TDVIIKLEKSNLTKELLEMTRVGAVVNDI-RKKVAQSAPELSKRCRTLIK
CAEEL   ESLLNEVRPLVTSREILEETRIGFVVNQL-RRQYFKKWPALLRISREVIK
ARATH*  LESLRKLESMSMSVDILKDTEIGKAVNGL-RRHSS-D--KISKLAKTLFA
VITVI   VDLLQTLADMDITFKALKETDIGRHVNRL-RKHPS-N--DVRRLVKHLVR
SOYBN   VELLQSLADMDITFQGLKETDIGRHVNRL-RKHPS-N--DLRRLVKLLVR
SOLLC   FECLRRLQLMALSVETLKATEIGKSVNSL-RKHNS-K--DVRHLSRTLIE
ORYSJ*  VELLRALRSTPMTFETLEASRIGKTISGLRRKHSS-E--KVRGLAAALYK
HORVU   FDSLRRLQLMQLSVSTLKATEIGRAVNGL-RKHSS-Q--QIRHLVQTLID
SORBI   VRLLQSLADMDVTYSALQETDIGRQVNGL-RKHSS-A--EVRRLVKQLIR
SELML   CEALERLESMHISFEALKATEIGRPVNNL-RKHPS-L--RVRSIVKRLVS
PHYPA   LTALRTLEDMHISVEALKQTEIGKEVNAL-RKHPS-K--RVRSLAKDLVR
VOLCA   LDVLKQLQKGLVTAELLKRTNAGKRLNKF-CKHSC-V--SVAQAAKAAVE
                 610       620       630       640       650
```

```
HUMAN*  SWQKLIEPAHQHEAALRGLAGATGS---------------AN-GGAHNCR
CHICK   NWQKLIEPVTPNEAVPRGLQNPPGS---------------AN-GGAHNCK
XENTR   NWQKLIEPVTQNEQLVRGISNLPGS---------------ANGGGSHNCK
DANRE   NWQKLIEPG-QGDTPVRGPPNVPGS---------------AN-GGAHPCR
CIOIN   TWQQMIQVKKRPKTVPEVVAAKTKP-------------------VGKPKQ
TENG    NWQKLIEP---------GPAAAPGS---------------TN-GSSHPCQ
DROME   KWREMVGIQQTATENLAHPSQISSSQPALDLVKSPITSFITE-PIAPSQQ
BRUMA   CWQKLAEPKPTSSGSSSANGTPSYA---------------------SPAV
CAEEL   SWSEIVCDEVKRPQSGASSRNTTPN---------------VLSPSVARLM
ARATH*  EWKRLVDQWMNTPEEMAGTEGTPES---------------LNLSVID---
VITVI   KWKDLVDEWVKSNPAGEFTSSALMD-------------------------
SOYBN   KWKEIVDEWVKLNPR--GGSNTLMA-------------------------
SOLLC   DWKVLVDEWVNATAAFAGNESTPES---------------MKASVVD--Q
ORYSJ*  NWKAIVDEHLTRSSSKPPAPAPTKT-------------------------
HORVU   DWKILVDEWVSTTN-VALADNSPGT---------------SNPSVVDDDE
SORBI   KWKEIVDEWVRLHNSGSDGSATVMT-------------------------
SELML   GWKTLAEEWTKSAEHFAGGTNATGH-----------------------FD
PHYPA   SWKDLVDEWVKSAGDVAAAAAADSA---------------------GVDN
VOLCA   AWKQCVKQEQQQNDG--GEAETPDA---------------VAQREAPAST
                 660       670       680       690       700
```

```
HUMAN*  PE--VG-AAGPPRS----------------IHDLKSR-------------
CHICK   PEAPLP-AVAGSKP----------------ISELKSR-------------
XENTR   AEPTPT-TLLGGKP----------------IQGLKDR-------------
DANRE   TDTPPA-VPPPSKV----------------APELKTR-------------
CIOIN   AQRTTKQTIVNGEK----------------PNKQRNN-------------
TENG    GEASPSDISVLGKG----------------VLDVKIR-------------
DROME   IVSDLHSNIDSAEPPLSGEHTLLHPNFSNLVNSIKDSDRHENIAITTLHT
BRUMA   KKGLTPGTPARGPRVISGGSPRLTPAEKSRFTALPSS-------------
CAEEL   QRGITPNTPAGRR-----------------VTSTGLP-------------
ARATH*  EEEAFPSPPHDLD-IYAPE-----------PNGFELS-------------
VITVI   --------------------------------------------------
SOYBN   --DGDSSPVQKTTQNGHHQQ----------IPDFAYS-------------
SOLLC   DEEGLPSPPLDDLAFFSAQ-----------TTSIELS-------------
ORYSJ*  --------------------------------------------------
HORVU   EEEGLPSPPLDEGAFFAPE-----------TTAIQLS-------------
SORBI   --ADGDSPDKIQGRSHQSPR----------VSGFQYS-------------
SELML   NNYGLPSPPVDEGELLAAG-----------TAPLEVN-------------
PHYPA   GEEGLPSPPLDPGALLASTR----------ANSIEMS-------------
VOLCA   SQPGLRAPLALTGK----------------ASAMTSG-------------
                 710       720       730       740       750
```

```
HUMAN*  ---------------------------NDLQRLPGQRLDRLGSRKRRGDQ
CHICK   ---------------------------NDIQKLNSPKPEKLGNRKRKGEH
XENTR   ---------------------------NDIQRAHSPKADKTTNRKRKGEH
DANRE   ---------------------------NDIHNTYSPKAEKSSSRKRRAEQ
CIOIN   ---------------------------SPAKPNEKNKIISKNAKPKVPEI
TENG    ---------------------------NDVHNTYSPKAEKSSSRKRRMEN
DROME   HKDRRHSHSIRSCHVASPQPIVIDHSVNSVINLTDDSTVKINEASVVIDI
BRUMA   --------------------------------VPTPSESSGSWNTDASPT
CAEEL   ---------------------------GSQQRLVSPANGSYAPRSQVSPG
ARATH*  ---------------------------QILDCLDCDGNPRHSVES-----
VITVI   --------------------------------------GSSGSDK-----
SOYBN   ---------------------------------PNPHNGSSGSDR-----
SOLLC   ---------------------------QFFDGMDDDGNPRNSGG------
ORYSJ*  --------------------------------------------------
HORVU   ---------------------------AFFDEMDEDGNLRHNNDGRLGN-
SORBI   ---------------------------------PSPQRQNGSASERAING
SELML   ---------------------------KLFDFIEDDMSAGSGDMNEKPRR
PHYPA   ---------------------------HFFDDFLDDLSAGSGPSPPSSNG
VOLCA   --------------------------------------VGAGQRPTCASF
                 760       770       780       790       800
```

MED26

```
HUMAN*  RDLGHPGPPPKVSKASHDPLVPNSSPLPTNGISGS-PESFA--SSLDGSG
CHICK   RDGHQGPPPPKVSKVSHE-ILQNSSPPPTNGIGGS-PESFP--SPVDVNL
XENTR   RDGGL-STPGHVSKPNHE-LFQNSSPPPTNGIGGSPPENLP--SPLDG--
DANRE   RDSP--HLPAKMSKTSLY-EPVYSSSPPSNGIRGT-PEPL-----LEKDD
CIOIN   RDSEN--------------NQSNNFTCMTNGLKVT-FSNLPTKSPSNNIS
TENG    RDSGV-HLPEKLFKMSSY-DDFASPPLPTNGIAGS-PEDP-----AEQQP
DROME   ASDSD--------------ENDNNGSLNPKKLNSIVPAPLPIPSTPSSRQ
BRUMA   FSNGR-TVTPYQRSEQKMSANIVANTWKSHAVSADLTIKAVENSLLSDDR
CAEEL   ESTPV------------AASVVVNGMHKSASVGGDLLKHATASLSTKPIT
ARATH*  -------------------------------------KHERKSQSSAGRR
VITVI   ----------------------------NNAEPEPKVKAIPRKEVPTRPA
SOYBN   ----------------------------NNSEAERKPKVVPRSEPRPKHA
SOLLC   -----------------FNVNRGNGRKPSLG-----------NQNIPLQK
ORYSJ*  -----------------ASASDHAKKTDMAAAHKPAPAPSPRKTASNKHE
HORVU   -------------------KRENNGRRPPNYSTVSKPELTRPVGTVERDQ
SORBI   FDS----------------------TMDAKRRASPVPAHHNSKQMNNNHN
SELML   GSSGDWVRGIYEDAHSPEETKPAAAVSRKTGESEADRKNKHQVSVSRKDS
PHYPA   ACD---------DIVAPLSKRQQQSRGPTASPESQAPKNKGRINTSNSHH
VOLCA   IVDPPRCGNDTRDKVRVMLAEALAVGFNSGGAVIEDALHELLAGSGSSVS
                 810       820       830       840       850
```

```
HUMAN*  HAGPEGSRLERDEND---KHSGKIPVNAVRPHTSSPGLGKPPGPCLQPKA
CHICK   HAGPESSRTELSEND---KHN-KIPVNAVKPHTSSPGLVKPSSTSSLLKT
XENTR   --GLQSNRLEPAEND---KHG-KIPVNAVRPHTNSPGLVKHPSTSSLLKA
DANRE   EVPSDRIRLEHLDND---RHN-KIPVNAVKPHPSSPGLTKLPSTSSLLKA
CIOIN   MKHPTKRKRTDENQDFVAKSE-KIPAKN-NTNISSISLSNVTKVNDVTKT
TENG    LRSPDRSRLDPLDND---KVS-RIPVNAVKPRP-SPGAAKLPSTSSLIKA
DROME   RK-LKKEKRSKEREGQVATNT-RFGAKA-SDGFQQAALTTDSEIFSLSNS
BRUMA   VIRNGKRKGGDIATSDIGATN----------------GLSATKRLHYTSA
CAEEL   PKENGKRKAVDEAAAPSIKRT------------KTAAAALSVSITPGTPT
ARATH*  PKGTNDANVVGRYCNDQQTRREEADVRPMKHSATDV--------------
VITVI   Q-------------------------------------------------
SOYBN   P-------------------------------------------------
SOLLC   KQSADCFNASPKENKGEQQKKQEAVIK------------KQTTVAKPTKP
ORYSJ*  --------------------------------------------------
HORVU   FRRPELTRQEPPMRHTNQQRPQGSNLQAKPHVMLNKQSRPPSSD------
SORBI   SA------------------------------------------------
SELML   SKNSDSKRSGERESSRAKVPNSQQQNGFRGSQAGGAAAAAANGKHSSGSS
PHYPA   EIRNGDGRSIQSNGRKAFDTPNGIDRSAHGISRGN---------------
VOLCA   GEYKAKARSLCFN-------------------------LKDAKNPDLRER
                 860       870       880       890       900
```

```
HUMAN*  SVLQ----------------------------------------------
CHICK   AVLQ----------------------------------------------
XENTR   VVLQ----------------------------------------------
DANRE   SVL-----------------------------------------------
CIOIN   SLET----------------------------------------------
TENG    AVMQ----------------------------------------------
DROME   SMSSIVSGDATSSYSQNKSRLNSNELTFTGRFKAVDHLFRSTESGKHKID
BRUMA   SVSP----------------------------------------------
CAEEL   TMAP----------------------------------------------
ARATH*  --------------------------------------------------
VITVI   --------------------------------------------------
SOYBN   --------------------------------------------------
SOLLC   SGGE----------------------------------------------
ORYSJ*  --------------------------------------------------
HORVU   --------------------------------------------------
SORBI   --------------------------------------------------
SELML   SVAA----------------------------------------------
PHYPA   --------------------------------------------------
VOLCA   VLSG----------------------------------------------
                 910       920       930       940       950
```

```
HUMAN*  --------------QLDRVDETPG-PP-----------------------
CHICK   --------------QHDKLEETTG-SH-----------------------
XENTR   --------------QHSGGLEDVL-SH-----------------------
DANRE   --------------QQARVDSGG--QH-----------------------
CIOIN   --------------------------------------------------
TENG    --------------QQAHLDEGGGAYY-----------------------
DROME   EYSAYDSNASCSRLSPSTVDEVKKAEHLFDAQLTNTTANQIPMSAVSIGY
BRUMA   --------------------------------------------------
CAEEL   --------------------------------------------------
ARATH*  --------------------------------------------------
VITVI   --------------------------------------------------
SOYBN   --------------------------------------------------
SOLLC   --------------------------------------------------
ORYSJ*  --------------------------------------------------
HORVU   --------------------------------------------------
SORBI   --------------------------------------------------
SELML   --------------------------------------------------
PHYPA   --------------------------------------------------
VOLCA   --------------------------------------------------
                 960       970       980       990      1000
```

MED26

```
HUMAN*  ---------HPKGPP-----------------------------------
CHICK   ---------QPKSPR-----------------------------------
XENTR   ---------QPRSPR-----------------------------------
DANRE   ---------QPKSPR-----------------------------------
CIOIN   --------------------------------------------------
TENG    ---------QAKSPR-----------------------------------
DROME   ESNTRHEYLESDSPSQIPKRRGRKKGSKGVDALIAKESSSLSQQIFFGGS
BRUMA   --------------------------------------------------
CAEEL   --------------------------------------------------
ARATH*  --------------------------------------------------
VITVI   --------------------------------------------------
SOYBN   --------------------------------------------------
SOLLC   --------------------------------------------------
ORYSJ*  --------------------------------------------------
HORVU   --------------------------------------------------
SORBI   --------------------------------------------------
SELML   --------------------------------------------------
PHYPA   --------------------------------------------------
VOLCA   --------------------------------------------------
                 1010      1020      1030      1040      1050
```

```
HUMAN*  -------------------------------RCSFSPRNSRHEGSFARQQ
CHICK   -------------------------------CSSFSPRNVRHD-TFARQH
XENTR   -------------------------------CSSFSPRGTRAE-LATRQH
DANRE   --------------------------------YSSSPRSATHE-TMAKRS
CIOIN   --------------------------------------------------
TENG    -------------------------------GLSTSPRSLKAD-VVAKRS
DROME   TVKKVKTTKELFNEIQSRKLSVSMQSSASNLSNSSTNRDLPSHTTFPRQT
BRUMA   --------------------------------------------------
CAEEL   --------------------------------------------------
ARATH*  --------------------------------------------------
VITVI   --------------------------------------------------
SOYBN   --------------------------------------------------
SOLLC   --------------------------------------------------
ORYSJ*  --------------------------------------------------
HORVU   --------------------------------------------------
SORBI   --------------------------------------------------
SELML   --------------------------------------------------
PHYPA   --------------------------------------------------
VOLCA   --------------------------------------------------
                 1060      1070      1080      1090      1100
```

```
HUMAN*  S-------------------------------------------------
CHICK   T-------------------------------------------------
XENTR   T-------------------------------------------------
DANRE   T-------------------------------------------------
CIOIN   --------------------------------------------------
TENG    A-------------------------------------------------
DROME   SSCSDTSMNSPHILETLSGSAIFASKIDDLGNTDSDTVTSDPSHDSNKSQ
BRUMA   --------------------------------------------------
CAEEL   --------------------------------------------------
ARATH*  --------------------------------------------------
VITVI   --------------------------------------------------
SOYBN   --------------------------------------------------
SOLLC   --------------------------------------------------
ORYSJ*  --------------------------------------------------
HORVU   --------------------------------------------------
SORBI   --------------------------------------------------
SELML   --------------------------------------------------
PHYPA   --------------------------------------------------
VOLCA   --------------------------------------------------
                 1110      1120      1130      1140      1150
```

```
HUMAN*  --------------------------------------------------
CHICK   --------------------------------------------------
XENTR   --------------------------------------------------
DANRE   --------------------------------------------------
CIOIN   --------------------------------------------------
TENG    --------------------------------------------------
DROME   EIKECTSLDSNSNSIQSLPLANNRENLIPTNVNDITTQLMHLIHSLNSPL
BRUMA   --------------------------------------------------
CAEEL   --------------------------------------------------
ARATH*  --------------------------------------------------
VITVI   --------------------------------------------------
SOYBN   --------------------------------------------------
SOLLC   --------------------------------------------------
ORYSJ*  --------------------------------------------------
HORVU   --------------------------------------------------
SORBI   --------------------------------------------------
SELML   --------------------------------------------------
PHYPA   --------------------------------------------------
VOLCA   --------------------------------------------------
                 1160      1170      1180      1190      1200
```

MED26

```
HUMAN*   -------------------------------------LYAPK------
CHICK    -------------------------------------TYSPK------
XENTR    -------------------------------------TYAPK------
DANRE    -------------------------------------TYAPK------
CIOIN    --------------------------------------------------
TENG     ---------------------------------------AYTPK------
DROME    SEIEIERVYQEKIIPCTCIVIEGVQGTLGESNNSTLDEQSYNAKISLNSK
BRUMA    --------------------------------------------------
CAEEL    --------------------------------------------------
ARATH*   --------------------------------------------------
VITVI    --------------------------------------------------
SOYBN    --------------------------------------------------
SOLLC    --------------------------------------------------
ORYSJ*   --------------------------------------------------
HORVU    --------------------------------------------------
SORBI    --------------------------------------------------
SELML    --------------------------------------------------
PHYPA    --------------------------------------------------
VOLCA    --------------------------------------------------
                  1210      1220      1230      1240      1250
```

```
HUMAN*   -----------------------GSVPSPSPRPQALDATQVPSPLP-LAQ
CHICK    -----------------------DSMPSPSQRSQFLDTAQVPSPPPSLMQ
XENTR    -----------------------GSAPSPSQRLPGVDSAH-QSPL----H
DANRE    -----------------------GTLSSPS-----LNSAQVPSPLPTL-Q
CIOIN    --------------------------------------------------
TENG     -----------------------ET---PV-----PSPSSRDSPLSSS-Q
DROME    DNLSCHNQDNYSTSQRSVNGEVIDTQPKLVKSIFDLDFDDNDDPLYFIMD
BRUMA    -----------------------------------------TAPHQSLLA
CAEEL    -----------------------------------------QTP-TMSVS
ARATH*   -----------------------------------------VEP-KRQTK
VITVI    -----------------------------------------SAPVSSSFP
SOYBN    -----------------------------------------SPSVSTPAS
SOLLC    -----------------------------------------FGP-GRPTK
ORYSJ*   -----------------------------------------AAP------
HORVU    -----------------------------------------SGSIMRPMK
SORBI    -----------------------------------------TTTSSAPAK
SELML    -------------KQQRPDDQRHRAATSNTSRSLKPGVASVSKPKSSSTS
PHYPA    -----------------------------------------SGP-GRPVD
VOLCA    -----------------------------------------SIPPESLVR
                  1260      1270      1280      1290      1300
```

```
HUMAN*   PSTPP--------------------V--RRLELLPSAESPVC--WLEQ--
CHICK    PSTPP--------------------MPAKRLEFSQQSVSEVSQHWQEQQV
XENTR    PSTPP--------------------ATAKRLESPRQD-RISSPHKPVEQL
DANRE    PLTSP--------------------AQVCISDGPSSVGLEGSLHLHRS--
CIOIN    --------------------------------------------------
TENG     PVASP--------------------AQASNADRPPHSSYRSSTHWAGS--
DROME    EIQKPIARADELKNNKSDTKNINFGASAQNASLNIIHFNDDAQQDNSNQE
BRUMA    ARRADVKSTSELVAQLTENLPGYLTI------------------------
CAEEL    AARKAVQSTSELFAQMSQTLPGHIDI------------------------
ARATH*   QSREQMVSA----IQRKPTAVTEQKRKLAGP-------------------
VITVI    PP------------------------------------------------
SOYBN    ATQ-----------------------------------------------
SOLLC    PALEQKLKVNEMNFQQKSDKGTIQKRPVAS--------------------
ORYSJ*   --------------------------------------------------
HORVU    AATQQK-PIGEMKYKQTQEHFGVERKPAMG--------------------
SORBI    VT------------------------------------------------
SELML    LPS-----------------------------------------------
PHYPA    RSGPPK-AVGNGTVRKFETKP-----------------------------
VOLCA    MSAE----------------------------------------------
                  1310      1320      1330      1340      1350
```

```
HUMAN*   --PESHQRLAG-------PGCKA---------------------------
CHICK    PSDSQHRHTAGTLPQHTSPSCKT---------------------------
XENTR    PSTDCHQVLPRTSQQHTPRSSLV---------------------------
DANRE    --SDRVSQ--P-------PHSTA---------------------------
CIOIN    --------------------------------------------------
TENG     --FDLSSHCSP-------PDMYS---------------------------
DROME    RGKNETQNAIPAFTVHEDPDCLARQRFYIQTNKVTSFHINALHNYYIPNI
BRUMA    --------------------------------------------------
CAEEL    --------------------------------------------------
ARATH*   --------------------------------------------------
VITVI    --------------------------------------------------
SOYBN    --------------------------------------------------
SOLLC    --------------------------------------------------
ORYSJ*   --------------------------------------------------
HORVU    --------------------------------------------------
SORBI    --------------------------------------------------
SELML    --------------------------------------------------
PHYPA    --------------------------------------------------
VOLCA    --------------------------------------------------
                  1360      1370      1380      1390      1400
```

MED26

```
HUMAN*   --------------------------------------------------
CHICK    --------------------------------------------------
XENTR    --------------------------------------------------
DANRE    --------------------------------------------------
CIOIN    --------------------------------------------------
TENG     --------------------------------------------------
DROME    NGNWDSVESFTSFQSEHTIMDTMESYTVTNGADVVPKYGLLTSDRIRKDL
BRUMA    --------------------------------------------------
CAEEL    --------------------------------------------------
ARATH*   --------------------------------------------------
VITVI    --------------------------------------------------
SOYBN    --------------------------------------------------
SOLLC    --------------------------------------------------
ORYSJ*   --------------------------------------------------
HORVU    --------------------------------------------------
SORBI    --------------------------------------------------
SELML    --------------------------------------------------
PHYPA    --------------------------------------------------
VOLCA    --------------------------------------------------
                   1410      1420      1430      1440      1450
```

```
HUMAN*   --------------------------------------------------
CHICK    --------------------------------------------------
XENTR    --------------------------------------------------
DANRE    --------------------------------------------------
CIOIN    --------------------------------------------------
TENG     --------------------------------------------------
DROME    SSLKSIKPYRVKNFTSLISPFLGVAKCLPTCRRARRRFKNFVNSSTANDK
BRUMA    --------------------------------------------------
CAEEL    --------------------------------------------------
ARATH*   --------------------------------------------------
VITVI    --------------------------------------------------
SOYBN    --------------------------------------------------
SOLLC    --------------------------------------------------
ORYSJ*   --------------------------------------------------
HORVU    --------------------------------------------------
SORBI    --------------------------------------------------
SELML    --------------------------------------------------
PHYPA    --------------------------------------------------
VOLCA    --------------------------------------------------
                   1460      1470      1480      1490      1500
```

```
HUMAN*   --------------------------------------------------
CHICK    --------------------------------------------------
XENTR    --------------------------------------------------
DANRE    --------------------------------------------------
CIOIN    --------------------------------------------------
TENG     --------------------------------------------------
DROME    ISNNFESLENMKTSFKNCNPLNVKIDGTTSAIYSHNENHVPMQVNTNVEQ
BRUMA    --------------------------------------------------
CAEEL    --------------------------------------------------
ARATH*   --------------------------------------------------
VITVI    --------------------------------------------------
SOYBN    --------------------------------------------------
SOLLC    --------------------------------------------------
ORYSJ*   --------------------------------------------------
HORVU    --------------------------------------------------
SORBI    --------------------------------------------------
SELML    --------------------------------------------------
PHYPA    --------------------------------------------------
VOLCA    --------------------------------------------------
                   1510      1520      1530      1540      1550
```

```
HUMAN*   --------------GLSP-AEPLLSRAGFSPDSSKADSDAASSGGS----
CHICK    --------------SSHP-GESLMSHIGFSQDASKMDSDDAAS-GS----
XENTR    --------------DSQ------TPRTGFSPESSKVDSDDATS-GS----
DANRE    --------------TLEPLSGALLATHGLEGLETKAEREGASS-NS----
CIOIN    --------------------------------------------------
TENG     --------------TPEA-TGTSSASEG--SASVSEDVDGAVG-PG----
DROME    LPPSQSFSSNAISYNLLKLADDKVCHDGISDKSSNTGCQGNSPYSSSSSS
BRUMA    --------------------------------------------------
CAEEL    --------------------------------------------------
ARATH*   --------------------------------------------------
VITVI    --------------------------------------------------
SOYBN    --------------------------------------------------
SOLLC    --------------------------------------------------
ORYSJ*   --------------------------------------------------
HORVU    --------------------------------------------------
SORBI    --------------------------------------------------
SELML    --------------------------------------------------
PHYPA    --------------------------------------------------
VOLCA    --------------------------------------------------
                   1560      1570      1580      1590      1600
```

MED26

```
HUMAN*  ----------------------------------------DSKKKKRYRP
CHICK   ----------------------------------------DSKKKKRYRP
XENTR   ----------------------------------------DNRKRKKSRL
DANRE   ----------------------------------------DGKKRKKYRP
CIOIN   --------------------------------------------------
TENG    ----------------------------------------SEHKRRKYRS
DROME   SYSGRKEDQHIITKNLQNKNIQLNSRKSDMKRRKRIYLENDSKKKKHYRK
BRUMA   ---NISQNHRLTGSEHGDNEGQANMDTGHISRTPILFIQTEASKKKEKRG
CAEEL   -GTDIREHEERVKRELKDEELAHSLQSSASYGNFGSATTTERKKRKYERK
ARATH*  --------------------------------------------------
VITVI   --------------------------------------------------
SOYBN   --------------------------------------------------
SOLLC   --------------------------------------------------
ORYSJ*  --------------------------------------------------
HORVU   --------------------------------------------------
SORBI   --------------------------------------------------
SELML   --------------------------------------------------
PHYPA   --------------------------------------------------
VOLCA   --------------------------------------------------
                 1610      1620      1630      1640      1650

HUMAN*  RDYTV----------NLDGQVAEAGVK-----------------------
CHICK   RDYTV----------NLDGHVTEGGVK-----------------------
XENTR   R---T----------EFEGHSADGTGK-----------------------
DANRE   RDYTV----------NLQGQSSEDRTK-----------------------
CIOIN   --------------------------------------------------
TENG    RDYSV----------NLDGQRVEEAAK-----------------------
DROME   RIKTAVNRTFSNLHRNLYSIDSENGMKNDYKNEYGNSNNEEYAIVQRPAG
BRUMA   RSK-----------------------------------------------
CAEEL   IQKGTAPGT-----------------------------------------
ARATH*  --------------------------------------------------
VITVI   --------------------------------------------------
SOYBN   --------------------------------------------------
SOLLC   --------------------------------------------------
ORYSJ*  --------------------------------------------------
HORVU   --------------------------------------------------
SORBI   --------------------------------------------------
SELML   --------------------------------------------------
PHYPA   --------------------------------------------------
VOLCA   --------------------------------------------------
                 1660      1670      1680      1690      1700

HUMAN*  ----------------PVRL-K--ERKLTFDP--------MTRQIKPLTQ
CHICK   ----------------PVRL-K--ERKLTFDP--------MTGQIKPLTP
XENTR   ----------------PARVKK--ERKLTFDV--------MTGQIKPLTL
DANRE   ----------------P-RL-K--ERRLTFDP--------VTGQIKPLTP
CIOIN   --------------------------------------------------
TENG    ----------------PVRL-K--ERRLTFDP--------VTGQIKPLVH
DROME   DGENCSNHIVLTIKKTPSKINSPANSMTAFSPPSTSDGANLQKSILDLRL
BRUMA   --------------------------------------------------
CAEEL   ----------------PAAVASPLLQTTTMVTPPSSNNIPEERLPSLVIR
ARATH*  --------------------------------------------------
VITVI   --------------------------------------------------
SOYBN   --------------------------------------------------
SOLLC   --------------------------------------------------
ORYSJ*  --------------------------------------------------
HORVU   --------------------------------------------------
SORBI   --------------------------------------------------
SELML   --------------------------------------------------
PHYPA   --------------------------------------------------
VOLCA   --------------------------------------------------
                 1710      1720      1730      1740      1750

HUMAN*  KEPVRADS----PVHMEQQSRTELDKQEAKAS------------------
CHICK   KDPLQVEI----PA-LTEQHRTETEKQEQKPN------------------
XENTR   KDPVQAES----TA-PSEQHRTETDKQDPKLC------------------
DANRE   KESHQEEECQGQPI-PEPSVRTDIPQQKPPTS------------------
CIOIN   --------------------------------------------------
TENG    KEPSPAEE-GSTPS-PGPYLHPGEFMQRTESP------------------
DROME   EDTNISQSITEIPQ-KKDLSKTDRIVHQSRSSAYRRIFRKPDKSQTKFID
BRUMA   --KNKEKEGRIKDQFVANEKVIAVDNEKASTSA-----------------
CAEEL   LGKNGLTSAASTPAFVSSEESAKRKYEKSIRSI-----------------
ARATH*  --------------------------QQDKLK------------------
VITVI   --------------------------NKQRDT------------------
SOYBN   --------------------------NRQRES------------------
SOLLC   --------------------------QQNKLR------------------
ORYSJ*  --------------------------------------------------
HORVU   --------------------------HVDKSR------------------
SORBI   --------------------------REQRQS------------------
SELML   --------------------------------------------------
PHYPA   --------------------------------------------------
VOLCA   --------------------------------------------------
                 1760      1770      1780      1790      1800
```

MED26

```
HUMAN*  ----------LQSPFEQTNWK---------ELSRNE---IIQSYLSRQSS
CHICK   ----------LQSPFEQTNWK---------ELSRNE---IIQSYLNRQSS
XENTR   ----------LPSPFQLTNWK---------ELSRNE---IIQSYLNRQSS
DANRE   ----------VPNPFQQTNWK---------ELSRNE---IIQSYLNLQSN
CIOIN   --------------------------------------------------
TENG    ----------THQPYHQTNWK---------ELSRNE---IIQSYLNLQSS
DROME   LDLKHLFHLKANSPCISANAKLHNKLFFPHELSRDNTSGIRERLINYSS-
BRUMA   ------------------------------MGNADSVPVFNVANSVPSTD
CAEEL   ------------------------------PRSTTPPPTARMQEKEREKE
ARATH*  ------------------------------ALDPDS--------------
VITVI   ------------------------------AIDPD---------------
SOYBN   ------------------------------SFDAE---------------
SOLLC   ------------------------------HSDEDAVQV-----------
ORYSJ*  ------------------------------ARADDA--------------
HORVU   ------------------------------LRAQPSAGVRLESAKPKTQD
SORBI   ------------------------------LLDLD---------------
SELML   ------------------------------SASKRSVDERIQSCKMSSSS
PHYPA   ------------------------------PVPKDNKPKPVQKNSQEPEE
VOLCA   ------------------------------ELASD---------------
                  1810      1820      1830      1840      1850
```

```
HUMAN*  LLSSSGA-----------QTPGAHHFMSEYLKQEES--------------
CHICK   LLSSSGV-----------QTPGAHYFMSEYLKQEES--------------
XENTR   LLSSSGV-----------QTQSAHYYMSEYLKQEEC--------------
DANRE   VLTSSGA-----------QTHGAHFFMTEYLKREEH--------------
CIOIN   ------------------REVTATKTENVNHKPSSP--------------
TENG    LLTSSGV-----------QAPGAHFFMSEYLKREGK--------------
DROME   --SSSSS-----------YDDDSEVENTSFLKKANV--------------
BRUMA   KMVVPTRNGKYDWYAMLPSLETLRNREHFRSKPSSN--------------
CAEEL   EMKREKE-----------RQEAIRRREQEEEEEEEIVDDKPKPVLSKLKD
ARATH*  ------------------KFEFAKRKLQESYHQHEN--------------
VITVI   ------------------KFASARKRLHENYQEAQNGLILSSK----LWI
SOYBN   ------------------RLASARRRLQENYKEAEIGGIFISIKFSFLMI
SOLLC   ------------------KLEATKRKLQERYQEAEK--------------
ORYSJ*  ------------------KLAAARRKLQDGYKEAAS--------------
HORVU   GLESNG------------RLEAAKRRLQERYQEAEN--------------
SORBI   ------------------KLDSARKRLQENYQEAQN--------------
SELML   KAGGSTD-----------KLAAAKRKLHEGYKQAEH--------------
PHYPA   NVSVSA------------RLEVAKRRMHERYEQENN--------------
VOLCA   ------------------EQKRKNREMKEWLAKEAT--------------
                  1860      1870      1880      1890      1900
```

```
HUMAN*  ------------------TRQGARQLHVLVPQSP----------------
CHICK   ------------------TRKEARKTHVLAPNSK----------------
XENTR   ------------------TKREARKTHVLVPIVL----------------
DANRE   ------------------DVKEPRKMHVLAPGSS----------------
CIOIN   ------------------------NIEAKTLSDAGEPGSL----------
TENG    ------------------ESAN-SQVHALQTDSS----------------
DROME   ------------------NKFSASNNRNLKTETETCAATAISEFKFESDE
BRUMA   -------------------QRKSYIVNVLGREVTFFLPKVS---------
CAEEL   WKSLIPSMEELRIRAQLAEKETKKVLEVSAVKTKKD--------------
ARATH*  -------------------AKRQRTIQVLETI------------------
VITVI   HNVLLLVLELMVCVGLFVEAKKQRTIQVMDIHDI----------------
SOYBN   HLSLSLYIYITDGLFVVFV----RCIYSQKAKNDSGDGH-----------
SOLLC   -------------------AKRQRTIQVMELRDI----------------
ORYSJ*  -------------------AKKQRVIQVIDT-------------------
HORVU   -------------------AKKQRTIQVMELGDI----------------
SORBI   -------------------AKKQRTIQVMDINDI----------------
SELML   -------------------AKKQRTVQVMELTDL----------------
PHYPA   -------------------AKRQRVVQVMELTEL----------------
VOLCA   -----------------------RGVNNAATTDMFQCGRCKQRKCTYYPM
                  1910      1920      1930      1940      1950
```

```
HUMAN*  --------------------------------------------------
CHICK   --------------------------------------------------
XENTR   --------------------------------------------------
DANRE   --------------------------------------------------
CIOIN   --------------------------------------------------
TENG    --------------------------------------------------
DROME   DSILTSLGDSDIDIQDEIQVINDKVDYCNDFQNNKMLESFNSIFVDSLRT
BRUMA   --------------------------------------------------
CAEEL   --------------------------------------------------
ARATH*  --------------------------------------------------
VITVI   --------------------------------------------------
SOYBN   --------------------------------------------------
SOLLC   --------------------------------------------------
ORYSJ*  --------------------------------------------------
HORVU   --------------------------------------------------
SORBI   --------------------------------------------------
SELML   --------------------------------------------------
PHYPA   --------------------------------------------------
VOLCA   TTFVTCTNCGQRWKFC----------------------------------
                  1960      1970      1980      1990      2000
```

MED26

```
HUMAN*  ----------------------PTDLPGLTREVTQDDLDRIQASQWP---
CHICK   ----------------------PTDLPGVTREVTSDDLNRICEHNWP---
XENTR   ----------------------PSDLPGRTREITSSDTDRIHNQHWP---
DANRE   ----------------------TTELPGVSRDVTNEDLLRIHNEHWP---
CIOIN   ----------------------PRGIDTKDSGPGTSDERP----------
TENG    ----------------------PEDVPGVTREVSAEDLERIHTQHWP---
DROME   NTKNASDNMGSSAHITSLESLKPENADAITRYCNNNNLGVNLSNELSNIS
BRUMA   ----------------------PSHVLLSKPRIASSDRYPLQVLALPYID
CAEEL   ----------------------PTKVQLVK-------NFRRPILILPYLE
ARATH*  ----------------------PKQNKVQKPQLKRPATRR----------
VITVI   ----------------------PKPKNTFFAKPKVGSQGRHR--------
SOYBN   ----------------------P---------------------------
SOLLC   ----------------------PKKGPNQGVGLKNAHMRPGNNNR-----
ORYSJ*  ----------------------PKKVNRRPVAVVERRRIMPGVATVAPLR
HORVU   ----------------------PKPKSHTKQPMAKSRNNIRSR-------
SORBI   ----------------------PKPKNRNAFIRKSASSGLPARNR-----
SELML   ----------------------PKGKGGPKAAKASGSSKLHHAQHNNRYN
PHYPA   ----------------------PKGGPQRAKVGQGHGHGPAKPSIANRFR
VOLCA   --------------------------------------------------
                  2010      2020      2030      2040      2050
```

```
HUMAN*  ----------------GVNGCQDTQGN------------------WYDWT
CHICK   ----------------GVNGCYDTQGN------------------WYDWT
XENTR   ----------------GVNGCHDTQGN------------------WYDWT
DANRE   ----------------GVNGCYDTKGA------------------WFDWT
CIOIN   ----------------GIDGCRTNNGL------------------WFSWQ
TENG    ----------------GVNGCYDSKGR------------------WYDWT
DROME   AKPLKSFDSTMKLPSLGIEHCTGIKTSSDMASTNIAEKKLTRIQQFKEWH
BRUMA   V-GLPDFLEYQYPKPERFYAEENFMYGASRPN------------------
CAEEL   NPTGPDFLLHKYPNSLQYYSEDNFRFGEKRPTAFFLFSLSLCPYWSY---
ARATH*  ------------------------------------------YIYWYFWS
VITVI   --------------------------------------------------
SOYBN   --------------------------------------------------
SOLLC   --------------------------------------------------
ORYSJ*  MCRAV---------------------------------------------
HORVU   --------------------------------------------------
SORBI   --------------------------------------------------
SELML   SIHGR-----------RLDQVFGGLGNGSAPSLWSVSGTQLERQQWRPAA
PHYPA   YQQIRR--------------------------------------------
VOLCA   --------------------------------------------------
                  2060      2070      2080      2090      2100
```

```
HUMAN*  QCIS------LDPHGDDGRLNILPYVCLD---------------------
CHICK   QCIS------LDPHGDDGRLNILPYVCLD---------------------
XENTR   QCIS------LDPHGDDGRLNVLPYVCLD---------------------
DANRE   DCIS------LDPHGDESKLNILPYVCLD---------------------
CIOIN   DGISVQRADLFETNDQDDILTILPYIELD---------------------
TENG    ECIS------LDPHGDENKLNILPYVCLD---------------------
DROME   QVLQ------LRSYNNEP-LIVLPYVLLE---------------------
BRUMA   --------------------------------------------------
CAEEL   --------------------------------------------------
ARATH*  SEFPGYELPSLILNLANPRDSSSQFSRHDELMIDIPVYFGLVLVQIDKAE
VITVI   --------------------------------------------------
SOYBN   --------------------------------------------------
SOLLC   --------------------------------------------------
ORYSJ*  --------------------------------------------------
HORVU   --------------------------------------------------
SORBI   --------------------------------------------------
SELML   NSDDEDEGPTCSSSFRSEEEQATKRVRQDENGDENVDEEEEMIRVQAMVG
PHYPA   --------------------------------------------------
VOLCA   --------------------------------------------------
                  2110      2120      2130      2140      2150
```

```
HUMAN*  --------------------------------------------------
CHICK   --------------------------------------------------
XENTR   --------------------------------------------------
DANRE   --------------------------------------------------
CIOIN   --------------------------------------------------
TENG    --------------------------------------------------
DROME   --------------------------------------------------
BRUMA   --------------------------------------------------
CAEEL   --------------------------------------------------
ARATH*  MVDEMMIFKKMSVLLTEKDFVDAIVSVSLKDLTRSLFVRFEAKACPKFDL
VITVI   --------------------------------------------------
SOYBN   --------------------------------------------------
SOLLC   --------------------------------------------------
ORYSJ*  --------------------------------------------------
HORVU   --------------------------------------------------
SORBI   --------------------------------------------------
SELML   MDTTLDYEDEEDEYDKVAVGREGAGDRLYTSDVKASFDSLSVLSGASFMA
PHYPA   --------------------------------------------------
VOLCA   --------------------------------------------------
                  2160      2170      2180      2190      2200
```

MED26

```
HUMAN*   --------------------------------------------------
CHICK    --------------------------------------------------
XENTR    --------------------------------------------------
DANRE    --------------------------------------------------
CIOIN    --------------------------------------------------
TENG     --------------------------------------------------
DROME    --------------------------------------------------
BRUMA    --------------------------------------------------
CAEEL    --------------------------------------------------
ARATH*   IHKTSVETVRCVGKTFMHMLSKVSREFPPRNRT-----------------
VITVI    --------------------------------------------------
SOYBN    --------------------------------------------------
SOLLC    --------------------------------------------------
ORYSJ*   --------------------------------------------------
HORVU    --------------------------------------------------
SORBI    --------------------------------------------------
SELML    RDDLASRKAAAADRLAEAREAKINGTTPDLHRTGDLYRRTSSSRPKSSGD
PHYPA    --------------------------------------------------
VOLCA    --------------------------------------------------
                  2210      2220      2230      2240      2250
```

```
HUMAN*   --------------------------------------------------
CHICK    --------------------------------------------------
XENTR    --------------------------------------------------
DANRE    --------------------------------------------------
CIOIN    --------------------------------------------------
TENG     --------------------------------------------------
DROME    --------------------------------------------------
BRUMA    --------------------------------------------------
CAEEL    --------------------------------------------------
ARATH*   --------------------------------------------------
VITVI    --------------------------------------------------
SOYBN    --------------------------------------------------
SOLLC    --------------------------------------------------
ORYSJ*   --------------------------------------------------
HORVU    --------------------------------------------------
SORBI    --------------------------------------------------
SELML    GTRTSDSSDKRKNESRDDRPQKKVRFAVEEENQKTRRTPRDDGGHRQRAG
PHYPA    --------------------------------------------------
VOLCA    --------------------------------------------------
                  2260      2270      2280      2290      2300
```

```
HUMAN*   --------------------------------------------------
CHICK    --------------------------------------------------
XENTR    --------------------------------------------------
DANRE    --------------------------------------------------
CIOIN    --------------------------------------------------
TENG     --------------------------------------------------
DROME    --------------------------------------------------
BRUMA    --------------------------------------------------
CAEEL    --------------------------------------------------
ARATH*   --------------------------------------------------
VITVI    --------------------------------------------------
SOYBN    --------------------------------------------------
SOLLC    --------------------------------------------------
ORYSJ*   --------------------------------------------------
HORVU    --------------------------------------------------
SORBI    --------------------------------------------------
SELML    IDRYRNVPHHVRHPEKYTHYTLDWGEEGNSNNVQAWQSTMAAINSTKNEG
PHYPA    --------------------------------------------------
VOLCA    --------------------------------------------------
                  2310      2320      2330      2340      2350
```

```
HUMAN*   --------------------------------------------------
CHICK    --------------------------------------------------
XENTR    --------------------------------------------------
DANRE    --------------------------------------------------
CIOIN    --------------------------------------------------
TENG     --------------------------------------------------
DROME    --------------------------------------------------
BRUMA    --------------------------------------------------
CAEEL    --------------------------------------------------
ARATH*   -------------------------------------HPGGLLYQRFDPT
VITVI    --------------------------------------------------
SOYBN    --------------------------------------------------
SOLLC    --------------------------------------------------
ORYSJ*   --------------------------------------------------
HORVU    --------------------------------------------------
SORBI    --------------------------------------------------
SELML    EGEQPFELPQHVPFVPRANKERAGSSKPGGSTKDSTTPSGSATSVSFAVR
PHYPA    --------------------------------------------------
VOLCA    --------------------------------------------------
                  2360      2370      2380      2390      2400
```

MED26

HUMAN*	--------------------------------
CHICK	--------------------------------
XENTR	--------------------------------
DANRE	--------------------------------
CIOIN	--------------------------------
TENG	--------------------------------
DROME	--------------------------------
BRUMA	--------------------------------
CAEEL	--------------------------------
ARATH*	HLSLARQQVAICKETDSQYPHEIVIHWTSASA
VITVI	--------------------------------
SOYBN	--------------------------------
SOLLC	-------------------------HWTNGRR
ORYSJ*	--------------------------------
HORVU	---------------------------VLGRR
SORBI	--------------------------------
SELML	GLEDEGVAMEENAQSSSKNQKSRSYRSRITEE
PHYPA	--------------------------------
VOLCA	--------------------------------

2410 2420 2430

MED28

HUMAN*	--
CHICK	--
XENTR	--
DANRE	--
CIOIN	--
ANOGA	--
TENG	--
DROME	--
BRUMA	M---
CAEEL	MTDVEQPVSVEDQQAQAQSYYDQVLGNAYVQTAINAYTKTKEFHPLLNST
ARATH*	--
VITVI	--
SOYBN	--
SOLLC	--
ORYSJ*	--
HORVU	--
SORBI	--
SELML	--
PHYPA	--
VOLCA	--
	10 20 30 40 50

HUMAN*	--
CHICK	--
XENTR	--
DANRE	--
CIOIN	--
ANOGA	--
TENG	--
DROME	--
BRUMA	--
CAEEL	LNSAEEKVSTVGNYAAQKAYDGYNSYYVKPKNTAYEAVSYGTERAKTAVE
ARATH*	--
VITVI	--
SOYBN	--
SOLLC	--
ORYSJ*	--
HORVU	--
SORBI	--
SELML	--
PHYPA	--
VOLCA	--
	60 70 80 90 100

HUMAN*	--MAAPLG
CHICK	--MAGALG
XENTR	--
DANRE	--MASSMG
CIOIN	--
ANOGA	--
TENG	--MASSMS
DROME	--
BRUMA	----------------------------------LFNAILNGKEALEHAS
CAEEL	SGKQAAIVGGTFGIGAAVVLTQFSLALSAGGAALVLEQVDSAKKLGSSAI
ARATH*	---MDYQQ
VITVI	--
SOYBN	--
SOLLC	--
ORYSJ*	--
HORVU	--
SORBI	--
SELML	--
PHYPA	--
VOLCA	--MI
	110 120 130 140 150

HUMAN*	GMFSG-QPPGP-PQAPPGLPGQASLLQAAPGAPRPSSSTLVDELES-SFE
CHICK	GMFAN-QPPGPPPPGPPGGPGPAGLI-PPPTGPRNPNNTLVDELEA-SFE
XENTR	-MFSA-QQPGPPPPNQPG--APAGLM-STPPGAKNPSSTLVDDLES-SFE
DANRE	GMFPGQQPPGSHPPPGPGGPGQPGLL-TGTPGNRGANNTLVDELEA-SFE
CIOIN	------------------------------MERSGGSGSVIDDLES-AFR
ANOGA	----------------------------MASSSNVGSGNLVDELEE-AFQ
TENG	GMFSGQQPTGAHPVGGPGGQGQPGFP-GAVPRAQG-GNTLVDELEA-SFE
DROME	-----------------------------MASNESGGGNLMDEFEE-AFQ
BRUMA	KTTSNAQATARQMVTDLSEKVQQISR-ASTEKAEEQANRILDGANRVAHL
CAEEL	STIKEAELAVEHRIFSALHQAQRIAM-VPVEKITENTNSLLDILDG-AVQ
ARATH*	KPPQSSDPSPSPPDRPPGIRSPETPS-NNQNNDIEDIMACVTALEA-ALL
VITVI	MAEQSPQVDAQAAAGPPL---------PP---PRQDMIACVMALEA-ALL
SOYBN	MGDRQ-VVDQQHMGDPQLP-----SS-PP---SKDDMVSCVMALEA-ALL
SOLLC	MAERHSVDQQQ-------TPESQMQS-PA---SRDDMIACVMALEA-ALL
ORYSJ*	MAE---PPSQSPAQTPPQAAQQQQQG-PGES-ARDDMMACVAALEA-ALL
HORVU	MAEPPPPPSQSPAQTPPPT---QPQT-PAAT-ARDDMMACVAALEA-ALL
SORBI	MAD--PTPSQSPAQTPPPPPAQQAAA-AG---GREDMLACVAALEA-ALL
SELML	MS------------------------------SLNEVQSWVASLDA-TLL
PHYPA	MAATPGGGGGGNGGGGGGGGGGNGGG-GGGG-GGPDLLSLVASLDA-SLL
VOLCA	QLTTALNRVFPFPALLSYFQVERGLA-MANPLPQRDVLGLLDKMEQ-AVA
	160 170 180 190 200

MED28

```
HUMAN*   A C - - - - - - - - - - - - - - - - - - - F A S L V S Q D Y V N G - T D Q E E I R T G V D Q C I Q K
CHICK    A C - - - - - - - - - - - - - - - - - - - F A S L V S Q D Y V N G - T D Q K E I R T G V D Q C I Q K
XENTR    A C - - - - - - - - - - - - - - - - - - - F A S L V S Q D Y V N G - T D Q E E I R T G V E Q C I Q K
DANRE    A C - - - - - - - - - - - - - - - - - - - F A S L V S Q D Y V N G - T D Q E E I R T G V D Q C I Q K
CIOIN    S C - - - - - - - - - - - - - - - - - - - I S Q F V S D E P S V G V T H Q D E Q R P T V E F A V N E
ANOGA    S C - - - - - - - - - - - - - - - - - - - I H A L T K E E S A T G - I D K D E I K V E V D Q T T L K
TENG     A C - - - - - - - - - - - - - - - - - - - F A S L V S Q D Y V N G - T D Q E E I R T G V D Q C I Q K
DROME    S C - - - - - - - - - - - - - - - - - - - L L T L T K Q E P N S G - T N K E E I D L E V Q K T T N R
BRUMA    T M G I S D E S D D I E K T P W S R F Q D L S H R I L E N S K S - - R V E G L Y W T Q F D - - A Q S
CAEEL    K G L N I E V P P S V N L T I G Q R V K N L A S L I V Q G V S N - - K A H D H V I D P I N E R A R N
ARATH*   P C - - - - - - - - - - - - - - - - - - - L P A R E L Q A I D R - - S P H P S H Q I D V E R H A R D
VITVI    P C - - - - - - - - - - - - - - - - - - - L P A R E L Q A I D R - - S P H P S H Q I D V E R H A R D
SOYBN    P C - - - - - - - - - - - - - - - - - - - L P A R E L Q A I D R - - S P H P S H Q I D V D R Y A R D
SOLLC    P C - - - - - - - - - - - - - - - - - - - L P A R E L Q A I D R - - S A H P Y H Q I D V E R H A R D
ORYSJ*   P C - - - - - - - - - - - - - - - - - - - L P A R E L Q A V D R - - S L Q S S H Q I D V E R H A R D
HORVU    P C - - - - - - - - - - - - - - - - - - - L P A R E L Q A V D R - - S L Q S S H Q I D V E R H A R D
SORBI    P C - - - - - - - - - - - - - - - - - - - L P A R E L Q A V D R - - S L Q S S H Q I D V E R H A R D
SELML    P C - - - - - - - - - - - - - - - - - - - L P A R E L Q A A D R - - S T H P S H H V D V E R H A R E
PHYPA    P C - - - - - - - - - - - - - - - - - - - L P A R E L Q A V D R - - S T H P S H Q V D V E R H A A D
VOLCA    D M - - - - - - - - - - - - - - - - - - - L P R - - - - - - - - - - P N D P A G S A R A S E A V R N
                             210                 220                 230                 240                 250
```

```
HUMAN*   F L - - - - - - - - - - D I A R Q T E C F F L Q K R - L Q L S V Q - - - - - - - - - - - - - - - - -
CHICK    F L - - - - - - - - - - D V A R Q T E C F F L Q K R - L Q L S V Q - - - - - - - - - - - - - - - - -
XENTR    F L - - - - - - - - - - D V A R Q T E C F F L Q K R - L Q L S V Q - - - - - - - - - - - - - - - - -
DANRE    F L - - - - - - - - - - D V A R Q T E C F F L Q K R - L Q L S V Q - - - - - - - - - - - - - - - - -
CIOIN    F L - - - - - - - - - - K L A R Q T E A Y F L K E R - A S L A M K - - - - - - - - - - - - - - - - -
ANOGA    F I - - - - - - - - - - D L A R Q M E A F F L Q K R - F L L S A L - - - - - - - - - - - - - - - - -
TENG     F L - - - - - - - - - - D V A R Q I E C F F L Q K R - F Q L S V Q - - - - - - - - - - - - - - - - -
DROME    F I - - - - - - - - - - D V A R Q M E A F F L Q K R - F L V S T L - - - - - - - - - - - - - - - - -
BRUMA    I V E C L R N S I S L A D M V R K N K S W V A G K V - G E L W T S L D E L K V S L E M E G E R L K K
CAEEL    Y L E Q L S Q S F V L L D I V R E K K T W V I E K S - N E L S T S V F D F K K T L E E E A Q K Y K V
ARATH*   F M - - - - - - - - - - E A A K K L Q L Y F M G L K - R E D R A P - - - - - - - - - - - - - - - - -
VITVI    F M - - - - - - - - - - E A A K K L Q V Y F I S L Q - R E D Q - P - - - - - - - - - - - - - - - - -
SOYBN    F M - - - - - - - - - - E A A K K L Q L Y F I S L Q - R E D K - P - - - - - - - - - - - - - - - - -
SOLLC    F M - - - - - - - - - - E A A K K L Q L Y L I G L Q - R E D L - P - - - - - - - - - - - - - - - - -
ORYSJ*   F M - - - - - - - - - - E A A K K L Q S Y F I S L Q - R E D R - P - - - - - - - - - - - - - - - - -
HORVU    F M - - - - - - - - - - E A A K K L Q S Y F I S L Q - R E D Q - P - - - - - - - - - - - - - - - - -
SORBI    F M - - - - - - - - - - E A A K K L Q S Y F I S L Q - R E D Q - P - - - - - - - - - - - - - - - - -
SELML    F M - - - - - - - - - - E A A K Q L Q V F F I R V Q - H E H Q - P - - - - - - - - - - - - - - - - -
PHYPA    F M - - - - - - - - - - E A A K T L Q L Y F I R A Q - H R H Q - P - - - - - - - - - - - - - - - - -
VOLCA    F A - - - - - - - - - - G A V G E L E K F F R Q L Q E L K V I P Q - - - - - - - - - - - - - - - - -
                             260                 270                 280                 290                 300
```

```
HUMAN*   K P E Q V I K E D V S E L R N E L - - - - - - - - - - - - - - - - - - - - - - - - - - - - - - - - -
CHICK    K P E Q V I K E D V S E L R N E L - - - - - - - - - - - - - - - - - - - - - - - - - - - - - - - - -
XENTR    K P E Q V I K E D V S E L R N E L - - - - - - - - - - - - - - - - - - - - - - - - - - - - - - - - -
DANRE    K P E Q V E K E D A S E L K N E L - - - - - - - - - - - - - - - - - - - - - - - - - - - - - - - - -
CIOIN    Q P E L V L Q E D I E E L E T E L - - - - - - - - - - - - - - - - - - - - - - - - - - - - - - - - -
ANOGA    K P D L L L K E E N F D L K Q E I - - - - - - - - - - - - - - - - - - - - - - - - - - - - - - - - -
TENG     K P E Q V V K E D I S E L R N E L - - - - - - - - - - - - - - - - - - - - - - - - - - - - - - - - -
DROME    K P Y M L I K D E N Q D L S I E I - - - - - - - - - - - - - - - - - - - - - - - - - - - - - - - - -
BRUMA    N P E E M L M R Y L Q R T S S I L P E R L R I L E E T T G K V L X E Q A T S K L V A L I S Y I E K L
CAEEL    A P E E M L M K H I Q S T S E Q L S T Q L Q S L R E K G Q N V F G D - - G T K I D S T I D Y L E N L
ARATH*   S R A E S L K K D I A V M E E E L - - - - - - - - - - - - - - - - - - - - - - - - - - - - - - - - -
VITVI    T K A E T L R K E I A V M E E E L - - - - - - - - - - - - - - - - - - - - - - - - - - - - - - - - -
SOYBN    T K V E M L R K E I A L M E E E L - - - - - - - - - - - - - - - - - - - - - - - - - - - - - - - - -
SOLLC    S K P D M L R K E I A K M E E E L - - - - - - - - - - - - - - - - - - - - - - - - - - - - - - - - -
ORYSJ*   S T E E M L R K D I A I M E E E L - - - - - - - - - - - - - - - - - - - - - - - - - - - - - - - - -
HORVU    T N E E L L R K E I T T M E E E L - - - - - - - - - - - - - - - - - - - - - - - - - - - - - - - - -
SORBI    T A E E M L R K E I T T M E E E L - - - - - - - - - - - - - - - - - - - - - - - - - - - - - - - - -
SELML    P K E E L L K K E I A G L E S E L - - - - - - - - - - - - - - - - - - - - - - - - - - - - - - - - -
PHYPA    S R E E A L K K E I A A L E A E L - - - - - - - - - - - - - - - - - - - - - - - - - - - - - - - - -
VOLCA    G P A A A Q A L E V E E L Q A E I - - - - - - - - - - - - - - - - - - - - - - - - - - - - - - - - -
                             310                 320                 330                 340                 350
```

```
HUMAN*   - - - - - - - - - - - Q R K D A L V Q K H L T K L R H W Q Q V L E D I N V Q H K K P A D I - P Q G -
CHICK    - - - - - - - - - - - Q R K E A L I Q K H L S K L R H W Q Q V L E D I S V Q H K K P A E M - P Q G -
XENTR    - - - - - - - - - - - Q R K E A L I Q K H L T K L R S W Q Q V L E E I N G Q H K K T S E M - P Q G -
DANRE    - - - - - - - - - - - Q R K E M L I Q K H L A K I H H W Q Q V L E D I N V Q H K K P T E L - P Q G -
CIOIN    - - - - - - - - - - - Q R K D D A I R N H G E K L Q Q W K N A L H Q I G Q G G I D A G F V - K P G -
ANOGA    - - - - - - - - - - - G R K D E L I R K H Y E K I E S W K Q L L S D Q Q N F N K P I Q S M - P P D -
TENG     - - - - - - - - - - - Q R K E M L V Q K H L S K L H H W Q Q V L E E V S L Q H R K P S D L P P P G -
DROME    - - - - - - - - - - - Q R K E A L L Q K H Y N R L E E W K A C L S D I Q Q G V H S R P T P - P I G S
BRUMA    N E K L M E A D N V H E L K D E V L K E A H D R L V D L L Q W L T D H I D F S D S T H T E - - - - -
CAEEL    K K N F T D A E D V Y K V R D E V L N E G R Q R I A E L S T W T T S L L I I S A E W Q F E - P - - -
ARATH*   - - - - - - - - - - - K T K D E L I K K H M R L F Q E S Q K L V K E Q I E K H R D E L E K - V - - -
VITVI    - - - - - - - - - - - R L K T E L I N K Q E R L I Q G W R K E L K D Q L D K H N T E L E R - V - - -
SOYBN    - - - - - - - - - - - N I K N E L I K K Q E N L I Q E W K K E L K D Q L D K H K I E L D R - V - - -
SOLLC    - - - - - - - - - - - K T K T E L L S K Q E R L I Q E W R K E L K D Q L D K H N M E L E R - V - - -
ORYSJ*   - - - - - - - - - - - K T K S E L I D K H K K L I E G W Q K E L K D Q L G K H V T E L E R - V - - -
HORVU    - - - - - - - - - - - K T K S E L I A K H K S L I E G W Q K E L K D Q L G K H N T E L E R - V - - -
SORBI    - - - - - - - - - - - K T K S E L I A K H K K L I E G W R K E L K E Q L G K H I T E L E R - V - - -
SELML    - - - - - - - - - - - R A K D E L I K R Q K R L L Q G W R D I L K A Q K L K H I H E L E R - V - - -
PHYPA    - - - - - - - - - - - K V K D A L M E K Q S K L L R H W Q S L L E A Q K N T H I E E L E R - V - - -
VOLCA    - - - - - - - - - - - E E K D R L I A H A R A Q V A R W Q D I L Q Q Q E Q L Q E N T L F A - G M G -
                             360                 370                 380                 390                 400
```

MED28

```
HUMAN*  --------------------------SLAYLEQASANI-PAPLKPT----
CHICK   --------------------------PLAYLEQASANI-PAPMKQS----
XENTR   --------------------------PLAYLEQASANI-PAPMKPT----
DANRE   --------------------------PLAFLEQASANL-PAPMKP-----
CIOIN   --------------------------SIVLMSPGAGSQ-QMKK-------
ANOGA   -------------MRGNLAGGAPGGGPAGMMASGGMNL-PMQMQSQH-QQ
TENG    --------------------------PLTFLEQASANLPPAPLKPN----
DROME   GMLQGPGGGMPPMGGTPPRPGMMPGMPPGAMQPGG----PMQPSPHMLQA
BRUMA   --------------------------NLXSVNDGSTN-------------
CAEEL   --------------------------EDLLIEELYFDA-PPPVRTRNLYR
ARATH*  --------------------------------------------------
VITVI   --------------------------------------------------
SOYBN   --------------------------------------------------
SOLLC   --------------------------------------------------
ORYSJ*  --------------------------------------------------
HORVU   --------------------------------------------------
SORBI   --------------------------------------------------
SELML   --------------------------------------------------
PHYPA   --------------------------------------------------
VOLCA   -------------G------------------------------------
                 410       420       430       440       450

HUMAN*  --------------------------------------------------
CHICK   --------------------------------------------------
XENTR   --------------------------------------------------
DANRE   --------------------------------------------------
CIOIN   --------------------------------------------------
ANOGA   QQMQQMQVQQQQMQQQMQQSMPMGASNAQMFQQGGIPRGVGQAGGGPGFP
TENG    --------------------------------------------------
DROME   QQMQQLRMISRQMPPK----------------------------------
BRUMA   --------------------------------------------------
CAEEL   NRA-----------------------------------------------
ARATH*  --------------------------------------------------
VITVI   --------------------------------------------------
SOYBN   --------------------------------------------------
SOLLC   --------------------------------------------------
ORYSJ*  --------------------------------------------------
HORVU   --------------------------------------------------
SORBI   --------------------------------------------------
SELML   --------------------------------------------------
PHYPA   --------------------------------------------------
VOLCA   --------------------------------------------------
                 460       470       480       490       500

HUMAN*  -------------------------------
CHICK   -------------------------------
XENTR   -------------------------------
DANRE   -------------------------------
CIOIN   -------------------------------
ANOGA   PGAGPNLQGPLAYLEKTASNIDLVGLGDGRR
TENG    -------------------------------
DROME   -------------------------------
BRUMA   -------------------------------
CAEEL   -------------------------------
ARATH*  -------------------------------
VITVI   -------------------------------
SOYBN   -------------------------------
SOLLC   -------------------------------
ORYSJ*  -------------------------------
HORVU   -------------------------------
SORBI   -------------------------------
SELML   -------------------------------
PHYPA   -------------------------------
VOLCA   -------------------------------
                 510       520       530
```

MED30

```
HUMAN*  --------------------------------------------------
CHICK   --------------------------------------------------
XENTR   --------------------------------------------------
DANRE   --------------------------------------------------
CIOIN   --------------------------------------------------
ANOGA   ---------------MAGQFPGGYQSPSGHRGN-----------------
TENG    --------------------------------------------------
DROME   MWKYGQNQGNQGPSSGGGGGGGPNMMPMGGFGM-----------------
BRUMA   ----------------MQNSGVGPNQPVYYQQT-----------------
CAEEL   MNPNQPPNYPPGNPNQPPPFPPTPYYPMYQAGMPMPYIPQSPQYPQPGPP
ARATH*  --------------------------------------------------
VITVI   --------------------------------------------------
SOYBN   --------------------------------------------------
SOLLC   --------------------------------------------------
ORYSJ*  --------------------------------------------------
HORVU   --------------------------------------------------
SORBI   --------------------------------------------------
SELML   --------------------------------------------------
                 10        20        30        40        50
```

```
HUMAN*  --------------------------------------------------
CHICK   --------------------------------------------------
XENTR   --------------------------------------------------
DANRE   --------------------------------------------------
CIOIN   --------------------------------------------------
ANOGA   -----------------------------------------YNSPIIQQH
TENG    --------------------------------------------------
DROME   -----------------------------------------QHGNMQQMH
BRUMA   -----------------------------------------QGGAMMMGY
CAEEL   GGPPPFMGIQIKQEPMWSQENRYGMQAGPSGMPGMPPSSHPYGYPPMHGM
ARATH*  --------------------------------------------------
VITVI   --------------------------------------------------
SOYBN   --------------------------------------------------
SOLLC   --------------------------------------------------
ORYSJ*  --------------------------------------------------
HORVU   --------------------------------------------------
SORBI   --------------------------------------------------
SELML   --------------------------------------------------
                 60        70        80        90       100
```

```
HUMAN*  --------------------------------------------------
CHICK   --------------------------------------------------
XENTR   --------------------------------------------------
DANRE   --------------------------------------------------
CIOIN   --------------------------------------------------
ANOGA   LN--QMNVPNQMGMMGFNQNNMMNSPQMQGVPGQGNQDMGLNPGMMQQNQ
TENG    --------------------------------------------------
DROME   MSPQHQQQQQQMGMMGGPGSMQMN-PQGPGGPG------GLMPGMSPQHQ
BRUMA   PPSTIDQRQQQQVMNMQSISAAQIYSGTGANI------------------
CAEEL   PPGMPQGMPPMMDPNMPDFSSPPMHHPTTSLLAQHLSSAPLPPAPPQQQS
ARATH*  --------------------------------------------------
VITVI   --------------------------------------------------
SOYBN   --------------------------------------------------
SOLLC   --------------------------------------------------
ORYSJ*  --------------------------------------------------
HORVU   --------------------------------------------------
SORBI   --------------------------------------------------
SELML   --------------------------------------------------
                110       120       130       140       150
```

```
HUMAN*  --------------------------------------------------
CHICK   --------------------------------------------------
XENTR   --------------------------------------------------
DANRE   --------------------------------------------------
CIOIN   --------------------------------------------------
ANOGA   QQQQPGQGMQPNPQQVATNQHQPGANQMVGQNPTHQAAQPSPQMGMQQQQ
TENG    --------------------------------------------------
DROME   MQQQQQQQMM--QQQMMVPQQGVGVGVGMGGGVGMGGGGVVPQ---QQQQ
BRUMA   ------------QSMPGRQYSAGTVQESSPGIRPSSHSQSNYQLGVQSQT
CAEEL   HLSQLLQQPTTYSTATNPHAAPTQTIARRPDNEARNAQLQEQELAARQQQ
ARATH*  --------------------------------------------------
VITVI   --------------------------------------------------
SOYBN   --------------------------------------------------
SOLLC   --------------------------------------------------
ORYSJ*  --------------------------------------------------
HORVU   --------------------------------------------------
SORBI   --------------------------------------------------
SELML   --------------------------------------------------
                160       170       180       190       200
```

MED30

```
HUMAN*  -------------------------------------MSTPPLAASGM--
CHICK   -------------------------------------MSTPPLAGAGM--
XENTR   -------------------------------------MSTPPLSGPGMGA
DANRE   -------------------------------------MTTPPLA------
CIOIN   --------------------------------------------------
ANOGA   QQQQQQQQQQQQQQQQSSNVGPGNP----MNNPQSQNQSVPPNQP-----
TENG    -------------SQQHPHLPPGGA-------PAPGQQPMPP--------
DROME   QPQQNMPQQNIPQQQQQLNPVAG----------------IPPGGA-----
BRUMA   PNEQQQQQQQQQFQNRQSYQP----------------QQQPQQCTQRISD
CAEEL   QQEYMLQQHQQQQQNRAGQLQAEQAFANPPTVSIPPQTGHPPQQTSASSQ
ARATH*  --------------------------------------------------
VITVI   --------------------------------------------------
SOYBN   --------------------------------------------------
SOLLC   --------------------------------------------------
ORYSJ*  --------------------------------------------------
HORVU   --------------------------------------------------
SORBI   --------------------------------------------------
SELML   --------------------------------------------------
                 210       220       230       240       250
```

```
HUMAN*  --A------------PGPFAG--PQAQQAAREVNTASLCRIGQETVQDIV
CHICK   --P------------PGAFSG--TQ-AQAAREVNTASLCRIGQETVQDIV
XENTR   AAG------------PGGFPG--AQAATAAREVNTASLCRIGQETVQDIV
DANRE   -----------------QFSGQQQQQTQAARDVNTASLCRIGQETVQDIV
CIOIN   ----------------MAHMQEEPRRSQSAVEFNPVSLYQAGQDIVQDIA
ANOGA   ---------------GAGTAV----QQQQKAEFNLLSLCRIGQETVQDIV
TENG    --------------------------QGALREISPVFLCRIGQETVQDIV
DROME   ---------------GGSNNMLAISQQNPHKEINIVQLSRLGQETVQDIA
BRUMA   QVGEKSKKDETKAARPMNPPPYSPEILSDLSKYSVASLACIGRELVQELL
CAEEL   QRAQETMAQQTE---PQSHIRESPQKETNLEKYSTGELCLYGRELVNDLN
ARATH*  ---------------------MLQKQVSTTTTMTTQELAMEGEKQLEETI
VITVI   ----------------------MEDK----VMKATQELALEGQKHLEETI
SOYBN   ----------------------MEEQ----QSKTTQELAMEGQKYLEETI
SOLLC   ----------------------MEDKGTITSPKTIQELAVEGQKHLEDTI
ORYSJ*  ---------------------MASSA----AARRRQEMAAEGQRHLEETI
HORVU   ----------------------MASL----AARRRQELAAEGQRHLEETI
SORBI   ----------------------MASE----AARRRQEMALEGQRHLEETI
SELML   -----------------------------MEETKLEVMAIEGQKQLEIAV
                 260       270       280       290       300
```

```
HUMAN*  YRTMEIFQLLRNMQLPNGVTYHTGTYQDRL--------------------
CHICK   FRTMEIFQLLRNMQLPNGVTYHTGTYQDRL--------------------
XENTR   FRTMEIFQLLRNMQLPNGVTYHTVTYQDRL--------------------
DANRE   LRTMEIFQLLRNMQLPNGVTYHPNTHQDRL--------------------
CIOIN   QKAQEIFQALKLSQLPNGIATGQRNAQERI--------------------
ANOGA   SRFQEVFGILRSIQPPNGTNQGQLSSNDKK--------------------
TENG    TRTMEVFQIARATQLPNGVTQSQAMYQDRF--------------------
DROME   SRFQEVFASLKGIQPTSH------RENSSE--------------------
BRUMA   LRTYTLMTSLTKSVDRWHQQQGVSDPEQLL--------------------
CAEEL   VKTSYLSTILKKVMERKPLNQGE-NPNDLA--------------------
ARATH*  EAAFQIISAMNDELCNPSLWSTSATPSSAATTTGS------------NGS
VITVI   ESAFQILSSMNDELCNPTLWSTPPPPATAASS---------------HSS
SOYBN   ENAFQILSSMNDELCNPVLWSTSPSASSSP--------------------
SOLLC   EAAHQILSAMNDELCNPSLWSTLNTAAASSAVAVAGGGVGASGVLSNGQQ
ORYSJ*  AAAFQILSSMNDELCNPTLWSSSSAAAAAAGGAGGLQHHGNNHHHHHQHH
HORVU   ASAFQILSSMNDELCNPALWSSSATATATAASAAS------------QHP
SORBI   AAAFQILVSMNDELCNAGLWSSSSVSAAAAGS-----------------Q
SELML   QSAHQILNSLNEVLSNPALWEG----------------------------
                 310       320       330       340       350
```

```
HUMAN*  -----------------------------------TKLQDNLRQLSVLFR
CHICK   -----------------------------------GKLQEHLRQLSILFR
XENTR   -----------------------------------GKLQEHLRQLSILFR
DANRE   -----------------------------------GKLQEHLRTLSVLFR
CIOIN   -----------------------------------NKLKDLLNKVEVLFK
ANOGA   -----------------------------------AKVQEQFRTIRLLFK
TENG    -----------------------------------GKLQEHLRQLTLLFR
DROME   -----------------------------------KKVQEYFRTIRLLFK
BRUMA   -----------------------------------SYCEYILSKI----T
CAEEL   -----------------------------------DQCKNALQRM----S
ARATH*  ALVSADAAAI---DGTSHH-SESAGGGGG-GGSGNSVLDEASLRYKNSVT
VITVI   ASLNGDASSD---FSHHME-IA------G-----GGALDDARLRYKSSVA
SOYBN   ---NADATSE---NSNHHA-DATTAAAGG-----GGALEQARSRYKKAVA
SOLLC   HHTNGDISSD---TSSSSS-ASAQHLDIG-----GGALDESRLRYKSSIA
ORYSJ*  HHHGGLPPPPPPLHSADSDAADAAGGGPGGAPGSGGSLDEARHRYKVAMA
HORVU   HHQNAAPPPP---HSADSD-ADAMGGAAG---GSGGSLDEARHRYKIAVA
SORBI   HHHSATTPPPP--HSADSDAADA-----GGGPGPGGSLDEARHRYKSAVA
SELML   ---------------------------GGKEEAGWAAVDDARQKYKAATT
                 360       370       380       390       400
```

MED30

```
HUMAN*   KLRLVYDKCNENC-GGMDPIPVE--QLIPYVEE---DGSKNDDR--AGPP
CHICK    KLRLVYDKCNENC-AGLDPIPIE--QLIPYVEE---DGSKHDDRGAASQL
XENTR    KLRLVYDKCNENC-AGLDPVPIE--QLVPYVEE---EYSKHDDRGIASQL
DANRE    KLRLVYDKCNENC-AGLEPIPSE--QLIPYVED---DSSKLEDR-MANQL
CIOIN    QLRVIYNECQKRV-GDVEP-NVN--TLVPILKEGEIPNTEHEQA-LKSHK
ANOGA    RLRLLYDKCNDNCQQGMEYTHVE--SLIPLKGE----LERTEPV-HTEEY
TENG     KLRLLYERCVEMT-SDLQEGPAESLQLVPYVGE---EMVSVKTE---SCS
DROME    RVRIIYEKCND---AGMDYMSAE--SLIPYRDE---PEPRIEPS-LCDEY
BRUMA    EIRLRIDYV-----PRVANISED--EFITLMSD---PSPPQKPPELTAKE
CAEEL    DIRQIIEKRRE---PTWKRMTGE--DYIELMLD---DSELKKPMDEERQK
ARATH*   SLRAVLAAIPNSQKAKASEMQNG-------------------------LG
VITVI    SLRAVLNAIQSSSQKAKAFEASSA---------------------ASGSA
SOYBN    ALRTILTAIPNSQKANAFNASSA--------------------------A
SOLLC    SLRSVLTAISNSQKAKALEAASA-----------------------SGSL
ORYSJ*   ALRTSIAAVSSC--------AQE----------------------MGSTE
HORVU    ALRASIAAVSPS--------TQE----------------------PGPTE
SORBI    ALRASISALSSC--------AQD----------------------TGSTE
SELML    ALRGIVAAIFNHPQMTSLDVDST-------------------------VE
                  410       420       430       440       450

HUMAN*   RFASEERR-------------------EIAE--VN---KKLKQKNQQLKQ
CHICK    RFASEERR-------------------EIME--VN---KKLKLKNQQLKQ
XENTR    RFASEEKR-------------------EILE--VN---KKLKQKNQQLKQ
DANRE    RAASEERR-------------------EVLE--VN---KKLKQKNQQLKM
CIOIN    ELVLQSRK-------------------KDLE-------MKLCMKNKQLET
ANOGA    KKALQENR-------------------ELVA--M------VQLKNKQLRE
TENG     PAVSQERT-------------------EVLE--INSVFQKVRQKNQEMKV
DROME    RKVLQENH-------------------ELIE--T------VKLKNRQLRE
BRUMA    ETFEVQRR-------------------KLVK-------LNTALKNLDWMC
CAEEL    RRAELEQRFAIVISKTPPVEGHIWYQGKWISEELHKKYMQFEANKEIVKN
ARATH*   SPESEDEI-------------------EKLEEQALSLRMEIAKKNVHVKE
VITVI    SQADQAEI-------------------EKLEERASNLRKELADKNKYLKL
SOYBN    SPADEAEI-------------------DKLEERASSLRRELANKNLHLKT
SOLLC    SAADQAEI-------------------EQLEDRASSLKKELVDKNKHLKL
ORYSJ*   HKADQAEI-------------------ERLEEHASALRKEIESKNKHVKL
HORVU    SKGDQAEI-------------------ERLEEHASSLRKEIGSKNKQLKL
SORBI    SEADHAEI-------------------ERLEEHASALRKEIESKNKQVKL
SELML    KAVDQAEI-------------------DRLQKQTVELREEIVRKNDVLKQ
                  460       470       480       490       500

HUMAN*   IMDQLRNLIWDINAM----------------LA-MRN-
CHICK    IMDQLRNLIWDINAM----------------LA-MRN-
XENTR    IMDQLRNLIWDINSM----------------LA-MRN-
DANRE    IMDQLRNLIWEINSM----------------LA-VRS-
CIOIN    LVAQMTDFVWDINAA----------------LASPYGS
ANOGA    IIDKIRLTIWEINTM----------------LS-MRRC
TENG     LMDQMRNLLWDVNAM----------------LT-LRK-
DROME    IIDRTRIIIWEINTM----------------LA-MRRS
BRUMA    LVSDPRFL-----------------------KK-ADKN
CAEEL    LAAELKALAWKIDVTSPGHLKKVDQTKISKKKT-DDQV
ARATH*   LIDKLRELIADISTW----------------QS-PCSV
VITVI    LIDQLRDLITDISTW----------------QS-PCSV
SOYBN    LIDQLRDLITDISTW----------------HS-PFST
SOLLC    LIDQLRDLLADLCTW----------------QS-PCST
ORYSJ*   LIDQLHDLISDISMW----------------QS-PCSV
HORVU    LIDQLRDLISDISMW----------------QS-PCSV
SORBI    LMDQLRDLITDVAMW----------------QS-PCSV
SELML    LITQMRELVRDISMW----------------QT-PPPS
                  510       520       530
```

MED31

```
HUMAN*   ----------------------------MAAAVAMETDDA---GNRLRFQ
CHICK    ----------------------------------MDADDT---GNRLRFQ
XENTR    ------------------------------MAAAMET-DE---QQRIRFQ
DANRE    ------------------------------MAGVMET-DE---QARQRFQ
CIOIN    -----------------------MAQQLPAMLGQKSINLN---DDSVRFQ
ANOGA    ---------------------------MNPYGIPVES-ED---AQKLRFQ
TENG     ----------------------------------MET-EE---QAKNRFQ
DROME    -----------------------MAKMYGKGKTAIES-EE---LQKRRWQ
BRUMA    MIKLWKFIVSKNSNAILPYKNYSIEGSGLNSVSGPET-SE---EQKKRFE
CAEEL    ----------------------------GYNTMQMES-VE---SEKTRFE
YEAST*   -----------------------MSSTNGNAPATPSSDQN---PLPTRFE
SCHPO    ----------------------------METKWLLSK-VP---DDKSRFE
CANAL    ---------------MSAQTDQPITEQQKKEQEQYTNLIN---SLPTRWE
CANGA    ---------------------MSDNKEDSTVDNSNSTEEV---PLPTRFE
ASPNI    ----------------------MAQPQNDQAQPPPAT------LTNPRFT
RHIO9    -----------------------------------MD-SE---EEKKRFQ
CRYNE    --------------MQALPTILPPPPLPDDSEAPARTPEKH--ANLVRFQ
USTMA    ---------------MTLGAATANDPIDSTLASPSRD-EQRRQANQAAFS
ENCCU    -------------------------------------------MSGSRFE
ANTLO    -------------------------------------------MAKSRFE
ARATH*   --------MASPEEMGDDASEIPSPP-----KNTYKD-PD---GGRQRFL
VITVI    --------MDSGKE-SDSAADTPTSP-----KNVYKD-PD---DGRQRFL
SOYBN    --------MASKIESENSTDTSPSSP-----KNIYKD-PD---DGQQRFL
SOLLC    --------MA-----STSNPETHESPQ----KSVYKD-PD---DGRQRFL
ORYSJ*   ---------------MEEAEARPAPP----------D-PN---DARQRFL
HORVU    --------MDAMEEDTPPAPPLPPPPTTGAKQPPYKD-PD---DGRQRFL
SORBI    ------------------MADAESNPGAGGKPPPYKD-PD---NGSQRFL
SELML    ------LAMESG-----------------------EE-VD---GGRQRFL
PHYPA    -----------------------SPPW----HQKFQD-PD---DGRQRFL
VOLCA    -----------------------------------TD-QEVGIGGHERFA
                  10        20        30        40        50
```

```
HUMAN*   LELEFVQCLANPNYLNFL--AQR--------------------GYFKDKA
CHICK    LELEFVQCLANPNYLNFL--AQR--------------------GYFKDKA
XENTR    LELEFVQCLANPNYLNFL--AQR--------------------GYFKDKS
DANRE    LELEFVQCLANPNYLNFL--AQR--------------------GYLREKP
CIOIN    VELEFVQCLSNPNYLNFI--AQR--------------------GYFKEST
ANOGA    VELEFVQCLANPNYLHFL--AQR--------------------GYFKDAA
TENG     AELEFVQCLANPNYLNFL--AQR--------------------GVFREKT
DROME    IELEFVQCLSNPNYLNFL--AQR--------------------GFFKDQS
BRUMA    IECEFVQALANPHYLNFL--AQR--------------------GYFKEPY
CAEEL    VECEFVQALANPNYLNFL--AQR--------------------GYFKEEY
YEAST*   VELEFIQSLANIQYVTYLL-TQQ--------------------QIWKSPN
SCHPO    IELEFVQMLSNPWYLNFL--AQH--------------------KYFEDEA
CANAL    IELEFVQSLSNIPYVNYL--AQN--------------------NYFNDEN
CANGA    VELEFVQSLANIPYVTYLLTQQQ--------------------LLWKDPK
ASPNI    LELEFVSSLANPYYLSHL--AVNYPSLLGISKSGNENEVNDDNSDPDAEA
RHIO9    IELEFIQCLSNPWYLNNL--AQQ--------------------QYFRDPA
CRYNE    SELEFIQCLAHPQYLHEL--HIQ--------------------GYLGKPA
USTMA    RDLEFLSSLANPYYLNHL--ALS--------------------GALSSPS
ENCCU    RELEFVQLLCNPDYLRWL--TRE--------------------GHFESEE
ANTLO    MELEFIQSLCNPGYLMFL--YKN--------------------RYFEDPI
ARATH*   LELEFIQCLANPTYIHYL--AQN--------------------RYFEDEA
VITVI    LELEFVQCLANPTYIHYL--AQN--------------------RYFEDEA
SOYBN    LELEFVQCLANPTYIHYL--AQN--------------------RYFEDEA
SOLLC    LELEFVQCLANPTYIHYL--AQN--------------------RYFEDEA
ORYSJ*   LELEFIQCLANPTYIHYL--AQN--------------------RYFEDEA
HORVU    LELEFIQCLANPIYINYL--AQN--------------------RYFEDEA
SORBI    LELEFVQCLANPTYIHYL--AQN--------------------RYFEDEA
SELML    LELEFVQCLANPTYIHYL--AQN--------------------RYFDDEA
PHYPA    LELEFVQLLANPTYIHHL--AQN--------------------RYFDDEA
VOLCA    LELEFVQCLANPLYINWL--ATK--------------------QYFENPS
                  60        70        80        90       100
```

MED31

HUMAN*	F V N Y L K Y L - L Y W K D P E Y A K Y L K Y P - Q C L H M L E L L Q Y - E H F R K E L V N A Q C A
CHICK	F V N Y L K Y L - L Y W K E P E Y A K Y L K Y P - Q C L H M L E L L Q Y - E H F R K E L V N A Q C A
XENTR	F V N Y L K Y L - L Y W K D P E Y A K Y L K Y P - Q C L H M L E L L Q Y - E H F R K E L V N A Q C A
DANRE	F V N Y L K Y L - L Y W K E P E Y A K F L K Y P - H C L H M L E L L Q Y - E H F R K E L V N A Q C A
CIOIN	F V N Y L E Y L - L Y W K E P E Y V K Y L R Y P - Q C L H M L E L L Q H - Q E F R N E L V N A P C A
ANOGA	F V N Y L K Y L - L Y W K E P E Y A K Y L K F P - M C L Y F L D L L Q Y - E H F R R E I V S A Q C C
TENG	F I N Y L K Y L - L Y W K E P E Y A K Y L K Y P - H C L H M L E L L Q Y - E H F R K E L V N A Q C A
DROME	F I N Y L K Y L - Q Y W K E P D Y A K Y L M Y P - M C L Y F L D L L Q Y - E H F R R E I V N S Q C C
BRUMA	F I N Y L K Y L - L Y W K R P E Y A R A L K Y P - Q C L H F L E A V Q S - S A F R E A I T C T A N A
CAEEL	F V N Y L K Y L - L Y W K D P Q Y A R C L K F P - Q C L H M L E A L Q S - Q Q F R D S M A Y G P S A
YEAST*	F K N Y L K Y L - E Y W C N P P Y S Q C I V Y P - N C L F I L K L L N G - F M E S A I V N E D G L L
SCHPO	F L Q Y L E Y M - E Y W R E P E Y V K F I I Y P - T C L H M L T L L K N - P Q F R N D I S R A D L S
CANAL	F I N Y L N Y L - Q Y W T Q P E Y S K F L V Y P - N C L H I L K L L Q D - E N F R K N I I N Q D F M
CANGA	F K N Y L K Y L - E Y W C E P P Y A Q C I V Y P - N A L F I L K L L N G - F M E K A T V N E D G L L
ASPNI	F A A Y L A Y L Y S Y W K T P E Y S Q F L T H P G A T L R A L R L L Q E - E K F R R D I I R P Q V I
RHIO9	F L N Y L K Y L - Q Y W K Q P E Y A K F V V Y P - H A L Y F L D L L Q H - A E F R D Y I S T S E N T
CRYNE	F L N Y L K Y L - E Y W R E P Q Y V R F I I Y P - T C L V Y L T L L Q T - E L F R S R L G D M G F I
USTMA	F K R Y L K Y L - D Y F R H P K Y V K Y L H Y P - Q A L H F L D L L Q N E E E F R L A C R D P A F A
ENCCU	F R S Y L R Y L - E Y W R S P E Y S R F L T Y P - Q C L A V L E H L N S - E N I N D M L S D E N F F
ANTLO	F V D F L I Y L - R Y F K R Q P F R N Y L T Y P - I C L S I L D S L N D R E N Q I F E W L R K G D L
ARATH*	F I G Y L K Y L - Q Y W Q R P E Y I K F I M Y P - H C L Y F L E L L Q N - P N F R T A M A H P A N K
VITVI	F I G Y L K Y L - Q Y W Q Q P E Y I K F I M Y P - H C L F F L E L L Q N - A N F R N A M A H P G S K
SOYBN	F I G Y L K Y L - Q Y W Q R P E Y I K F I M Y P - H C L Y F L E L L Q N - A N F R N A M A H P T N K
SOLLC	F I G Y L K Y L - Q Y W Q R P E Y I K F I M Y P - H C L F F L E L L Q N - P T F R N A M A H P A N K
ORYSJ*	F I G Y L K Y L - K Y W Q R P E Y I K Y I M Y P - H C L F F L E L L Q N - A N F R N A M A H P A S K
HORVU	F I G Y L K Y L - K Y W Q R P E Y I K Y I M Y P - H C L F F L E L L Q N - A N F R N A M A H P A S K
SORBI	F I G Y L K Y L - K Y W Q R P E Y I K H I M Y P - H C L F F L E L L Q N - A N F R N A M A H P T N K
SELML	F V N Y L K Y L - Q Y W R R P E Y A K F I M Y P - H C L F F L E L L Q S - S H F R A A M V H - - - -
PHYPA	F V G Y L K Y L - L Y W K Q P E Y A K Y I M Y P - H A F F F L D M L Q S - A N F R A A M A H P A N K
VOLCA	F I N Y L K Y L - Q Y W K Q P A Y A I H I T -
	110 120 130 140 150

HUMAN*	K F I D E Q - - - - - - - Q I L H W - - - - - - - Q H Y S R K R M R L Q Q A L A E Q - - - - - - - - - -
CHICK	K F I D E Q - - - - - - - Q I L H W - - - - - - - Q H Y S R K R M R L Q Q A L A E Q - - - - - - - - - -
XENTR	K F I D E Q - - - - - - - Q I L H W - - - - - - - Q H Y S R K R M R M Q Q A L A E Q - - - - - - - - - -
DANRE	K F I D E Q - - - - - - - Q I L H W - - - - - - - Q H Y S R K R T R L Q Q A L A E Q - - - - - - - - - -
CIOIN	K F I E D Q - - - - - - - M L L H W - - - - - - - Q F Y T R K R I R V Q Q S M V E Q - - - - - - - - - -
ANOGA	K F I D D Q - - - - - - - A I L L W - - - - - - - Q H Y T R R R T R L T A L G T T S L T G L A V G G Q P
TENG	K F I D E Q - - - - - - - Q L L H W - - - - - - - Q H Y S R K R M R L Q Q A L A E Q - - - - - - - - - -
DROME	K F I D D Q - - - - - - - A I L Q W - - - - - - - Q H Y T R K R I K L I E N V T A A - - - - - - - - - -
BRUMA	K F I E E Q - - - - - - - Q L L Q W - - - - - - - Q Y Y T R K R Q R L H V Y T T E H - - - - - - - - - -
CAEEL	K F V E D Q - - - - - - - V V L Q W - - - - - - - Q F Y L R K R H R L C M M P D E G Q E L E E S E D E A
YEAST*	E G L D E L P K I I Q L Q G P Q W - - - - - - - M N E M V E R W A N - - - - - - - - - - - - - - - - - -
SCHPO	K Q V N D E - - - - - - - I Y Y E W L G K G L Q Q Y G S A D D A T L S Q P Q Q E E - - - - - - - - - -
CANAL	N S L M N D - - - - - - - M V K R W - - - - - - - Q S N A N D Q D E N K E K E E N K - - - - - - - - - -
CANGA	D G L E D L P K V I Q L Q G N Q W - - - - - - - M N E M V E R W A N - - - - - - - - - - - - - - - - - -
ASPNI	E R L A G V - - - - - - - D L N G E - - - - - - - Q S A A E A G E Q N T E Q N - - - - - - - - - - - - -
RHIO9	Q E V H R M - - - - - - - Q Y Y H W - - - - - - - Q Y L R N P P S T A T V E E P Q - - - - - - - - - - -
CRYNE	T E L M R V - - - G S R H H A T W - - - - - - - R V G K P A E D K Q S E E K P A V S M A P L D D D E -
USTMA	G E V M A K - - - - - - - Q I G H W - - - - - - - A T W R D P E N A A G Q E Q D E - - - - - - - - - - -
ENCCU	L A L G E Q - - - - - - - Q Y F I W - - - - - - - L N K H K E G W N K - - - - - - - - - - - - - - - - -
ANTLO	S I L N E Q - - - - - - - L W R L W - - - - - - - K N N N R -
ARATH*	E L A H R Q - - - - - - - Q F Y Y W - - - - - - - K N Y R N N R L K H I L P R P L P E P V P P Q P P - -
VITVI	E L A H R Q - - - - - - - Q F F Y W - - - - - - - K N Y R N N R L K H I L P K P L P E P L A - - - - - -
SOYBN	E L A H R Q - - - - - - - Q F Y F W - - - - - - - K N Y R N N R L K H I L P R S L P E P A T P A A P - -
SOLLC	E V A H R Q - - - - - - - Q F Y F W - - - - - - - K N Y R N N R L K H I L P R P L P E P A T A P S S - -
ORYSJ*	E V A H R Q - - - - - - - Q Y F F W - - - - - - - K N Y R N N R L K H I L P R P P P E P T P T P A P - -
HORVU	E V A H R Q - - - - - - - Q Y F F W - - - - - - - K N Y R N N R L K H I L P R P P P E P T P A P A P - -
SORBI	E L A H R Q - - - - - - - Q Y F F W - - - - - - - K N Y R N N R L K H I L P R P P P E P T P A P A P S Q
SELML	- -
PHYPA	E I A H R Q - - - - - - - Q F Y F W - - - - - - - K H Y R N N R L K Q I L P R P L P E - - - - - - - - -
VOLCA	- -
	160 170 180 190 200

MED31

```
HUMAN*   ---------QQQNNTSGK--------------------------------
CHICK    ---------QQQNTSVK---------------------------------
XENTR    ---------QQQNNTSGK--------------------------------
DANRE    --------QQQQQPQAPSHANTTSK------------------------
CIOIN    ---------ARQTGQNNENRGVLNQPQQKT--------------------
ANOGA    VGGGVQGTLLSNDPAIMPNSSSNNGSNNGSSNSSNNGAGGGGTGGVGGVG
TENG     ---------QQPQQQPLP-HGNATTK------------------------
DROME    ---------QQQQQQLQQQQQQANGMEAATGGESAAPTP-----NVNGSA
BRUMA    ---------DDDEQEWKQQHPQRQKTSTTSS-------------------
CAEEL    DIRQKDTEDEDDEETMKKPDADTAEKNSTTSTVSKKEK------------
YEAST*   --------------------------------------------------
SCHPO    ---------DEKKVDVKKENE-----------------------------
CANAL    ---------EVPEVRINGTN------------------------------
CANGA    --------------------------------------------------
ASPNI    ---------KGDRGNVENQHGKT---------------------------
RHIO9    --------------------------------------------------
CRYNE    ---------EEDEPERGQETKGKRKKKKSRSGNVGAGQAL----------
USTMA    ---------AGEQGEARETTGGQTA-------------------------
ENCCU    --------------------------------------------------
ANTLO    --------------------------------------------------
ARATH*   ---------VAPSTSLPPAP-------SATAALSPALSPMQY----NNML
VITVI    ---------GTPAPGPPPQSAPPTTI-AVTSAGSPALSPMQYGIPAGSSL
SOYBN    --APASTPSQAPVSALPPVPATSV---AVTAAPSQAPSPMPYVMPPGSGL
SOLLC    -----APPASLPSSVAPPTAPPPAPT-PVTAA---ALSPMQYAIPPGSGL
ORYSJ*   APAAVPPSASVPSTVVPPVAAPPSALLPMSAAGASAMSPMQFAGTPGTNI
HORVU    ----------APVPTPPPVPAAPSSLPTMSAVGASAMPPMQFIGTPGTNN
SORBI    APATLPLPASVPTPVAPPVPGPTSSMPPVVAGGASAMSPMQFVGTPGTNM
SELML    --------------------------------------------------
PHYPA    ----------APAPPPPPPPAPTPVVVAATA-------------------
VOLCA    --------------------------------------------------
                  210       220       230       240       250
```

```
HUMAN*   --------------------------------------------------
CHICK    --------------------------------------------------
XENTR    --------------------------------------------------
DANRE    --------------------------------------------------
CIOIN    --------------------------------------------------
ANOGA    GISNANNGSSNNGPNSGSVNVPSSVGQQQQQQQNGAGITQHNGVGGGGGV
TENG     --------------------------------------------------
DROME    STADSQQTSSALQPVQAQPGNPQQQQQINGVASGANIKLELN--------
BRUMA    --------------------------------------------------
CAEEL    --------------------------------------------------
YEAST*   --------------------------------------------------
SCHPO    --------------------------------------------------
CANAL    --------------------------------------------------
CANGA    --------------------------------------------------
ASPNI    --------------------------------------------------
RHIO9    --------------------------------------------------
CRYNE    --------------------------------------------------
USTMA    --------------------------------------------------
ENCCU    --------------------------------------------------
ANTLO    --------------------------------------------------
ARATH*   SKNDTRNMGATGIDRRKRKKGI----------------------------
VITVI    SKNDMRN---SAIDRRKRKKEG----------------------------
SOYBN    AKNDMRN---PTVDNRRKRK------------------------------
SOLLC    AKTDPRN---ASVDRRKRKKDG----------------------------
ORYSJ*   PKNDMRNVM-GGQGGRKRKKDG----------------------------
HORVU    PKNDMRNVM----GGRKRKMG-----------------------------
SORBI    PKNDMRNAM----GNRKRKMG-----------------------------
SELML    --------------------------------------------------
PHYPA    --------------------------------------------------
VOLCA    --------------------------------------------------
                  260       270       280       290       300
```

MED31

HUMAN*	--------------------------------
CHICK	--------------------------------
XENTR	--------------------------------
DANRE	--------------------------------
CIOIN	--------------------------------
ANOGA	VGGGGMLGNPMGALGGGGSSLPGGGGINQKVP
TENG	--------------------------------
DROME	--------------------------------
BRUMA	--------------------------------
CAEEL	--------------------------------
YEAST*	--------------------------------
SCHPO	--------------------------------
CANAL	--------------------------------
CANGA	--------------------------------
ASPNI	--------------------------------
RHIO9	--------------------------------
CRYNE	--------------------------------
USTMA	--------------------------------
ENCCU	--------------------------------
ANTLO	--------------------------------
ARATH*	--------------------------------
VITVI	--------------------------------
SOYBN	--------------------------------
SOLLC	--------------------------------
ORYSJ*	--------------------------------
HORVU	--------------------------------
SORBI	--------------------------------
SELML	--------------------------------
PHYPA	--------------------------------
VOLCA	--------------------------------
	310 320 330

MED34

```
ARATH*  - - - - - - - - - - - - - - - - - - - - - - - - - - - - - - - - - - - - - - - - - - - - - - - - - -
VITVI   - - - - - - - - - - - - - - - - - - - - - - - - - - - - - - - - - - - - - - - - - - - - - - - - - -
SOYBN   - - - - - - - - - - - - - - - - - - - - - - - - - - - - - - - - - - - - - - - - - - - - - - - - - -
SOLLC   - - - - - - - - - - - - - - - - - - - - - - - - - - - - - - - - - - - - - - - - - - - - - - - - - -
ORYSJ*  M I K P R V N W S D H A N A V Q S S C I K D E F L S S S F L F S L P T Q R P N Q E A D C T G M L P L
HORVU   - - - - - - - - - - - - - - - - - - - - - - - - - - - - - - - - - - - - - - - - - - - - - - - - - -
SORBI   - - - - - - - - - - - - - - - - - - - - - - - - - - - - - - - - - - - - - - - - - - - - - - - - - -
SELML   - - - - - - - - - - - - - - - - - - - - - - - - - - - - - - - - - - - - - - - - - - - - - - - - - -
PHYPA   - - - - - - - - - - - - - - - - - - - - - - - - - - - - - - - - - - - - - - - - - - - - - - - - - -
VOLCA   - - - - - - - - - - - - - - - - - - - - - - - - - - - - - - - - - - - - - - - - - - - - - - - - - -
                          10                  20                  30                  40                  50
```

```
ARATH*  - - - - - - - - - - - - - - - - - - - - - - - - - - - - - - - - - - - - - - - - - - - - - - - - - -
VITVI   - - - - - - - - - - - - - - - - - - - - - - - - - - - - - - - - - - - - - - - - - - - - - - - - - -
SOYBN   - - - - - - - - - - - - - - - - - - - - - - - - - - - - - - - - - - - - - - - - - - - - - - - - - -
SOLLC   - - - - - - - - - - - - - - - - - - - - - - - - - - - - - - - - - - - - - - - - - - - - - - - - - -
ORYSJ*  R S A A C R I Q G L E R L Q A P S I E K A W R S L R N T Q V A R K N Y L R P G L S G K V K D C D S D
HORVU   - - - - - - - - - - - - - - - - - - - - - - - - - - - - - - - - - - - - - - - - - - - - - - - - - -
SORBI   - - - - - - - - - - - - - - - - - - - - - - - - - - - - - - - - - - - - - - - - - - - - - - - - - -
SELML   - - - - - - - - - - - - - - - - - - - - - - - - - - - - - - - - - - - - - - - - - - - - - - - - - -
PHYPA   - - - - - - - - - - - - - - - - - - - - - - - - - - - - - - - - - - - - - - - - - - - - - - - - - -
VOLCA   - - - - - - - - - - - - - - - - - - - - - - - - - - - - - - - - - - - - - - - - - - - - - - - - - -
                          60                  70                  80                  90                 100
```

```
ARATH*  - - - - - - - - - - - - - - - - - - - - - - - - - - - - - - - - - - - - - - - - - - - - - - - - - -
VITVI   - - - - - - - - - - - - - - - - - - - - - - - - - - - - - - - - - - - - - - - - - - - - - - - - - -
SOYBN   - - - - - - - - - - - - - - - - - - - - - - - - - - - - - - - - - - - - - - - - - - - - - - - - - -
SOLLC   - - - - - - - - - - - - - - - - - - - - - - - - - - - - - - - - - - - - - - - - - - - - - - - - - -
ORYSJ*  H A H T Y G T S S S Y N V N K M D S V S R N R N P T Q E S M H Q T T E S G T M E K N S S H L P A G T
HORVU   - - - - - - - - - - - - - - - - - - - - - - - - - - - - - - - - - - - - - - - - - - - - - - - - - -
SORBI   - - - - - - - - - - - - - - - - - - - - - - - - - - - - - - - - - - - - - - - - - - - - - - - - - -
SELML   - - - - - - - - - - - - - - - - - - - - - - - - - - - - - - - - - - - - - - - - - - - - - - - - - -
PHYPA   - - - - - - - - - - - - - - - - - - - - - - - - - - - - - - - - - - - - - - - - - - - - - - - - - -
VOLCA   - - - - - - - - - - - - - - - - - - - - - - - - - - - - - - - - - - - - - - - - - - - - - - - - - -
                         110                 120                 130                 140                 150
```

```
ARATH*  - - - - - - - - - - - - - - - - - - - - - - - - - - - - - - - - - - - - - - - - - - - - - - - - - -
VITVI   - - - - - - - - - - - - - - - - - - - - - - - - - - - - - - - - - - - - - - - - - - - - - - - - - -
SOYBN   - - - - - - - - - - - - - - - - - - - - - - - - - - - - - - - - - - - - - - - - - - - - - - - - - -
SOLLC   - - - - - - - - - - - - - - - - - - - - - - - - - - - - - - - - - - - - - - - - - - - - - - - - - -
ORYSJ*  K S C T R T Y L N N H V V Q A D T I T T T N Q S L A R T G P E L F K T A P F I D N M C D D A K L D A
HORVU   - - - - M A Y Q S N H V V G - - - - T T Y Q S S F A R T D A M S C Q T V P V A D N M C A D D K L D A
SORBI   - - - - - - - - - - - - - - - - - - - - - - - - - - - - - - - - - - - - - - - - - - - - - - - - - -
SELML   - - - - - - - - - - - - - - - - - - - - - - - - - - - - - - - - - - - - - - - - - - - - - - - - - -
PHYPA   - - - - - - - - - - - - - - - - - - - - - - - - - - - - - - - - - - - - - - - - - - - - - - - - - -
VOLCA   - - - - - - - - - - - - - - - - - - - - - - - - - - - - - - - - - - - - - - - - - - - - - - - - - -
                         160                 170                 180                 190                 200
```

```
ARATH*  - - - - - - - - - - - - - - - - - - - - - - - - - - - - - - - - - - - - - - - - - - - - - - - - - -
VITVI   - - - - - - - - - - - - - - - - - - - - - - - - - - - - - - - - - - - - - - - - - - - - - - - - - -
SOYBN   - - - - - - - - - - - - - - - - - - - - - - - - - - - - - - - - - - - - - - - - - - - - - - - - - -
SOLLC   - - - - - - - - - - - - - - - - - - - - - - - - - - - - - - - - - - - - - - - - - - - - - - - - - -
ORYSJ*  M D E D E L L A S I D V D R I V M E H Y Q A T N T P R G S S K S P L - - - - - E K C N F N G F D E N
HORVU   M D D D E I L A S I D V D R I V M E H Y E A T N T P R G L A S R Q M S T P S G N K F N F T G L D E N
SORBI   - - - - - - - - - - - - - - - - - - - - - - - - - - - - - - - - - - - - - - - - - - - - - - - - - -
SELML   - - - - - - - - - - - - - - - - - - - - - - - - - - - - - - - - - - - - - - - - - - - - - - - - - -
PHYPA   - - - - - - - - - - - - - - - - - - - - - - - - - - - - - - - - - - - - - - - - - - - - - - - - - -
VOLCA   - - - - - - - - - - - - - - - - - - - - - - - - - - - - - - - - - - - - - - - - - - - - - - - - - -
                         210                 220                 230                 240                 250
```

```
ARATH*  - - - - - - - - - - - - - - - - - - - - - - - M E S E A I Q E D L Q N L D V E L K D V Q G Q I S A L
VITVI   - - - - - - - - - - - - - - - - - - - - - - M D S N Y D L L E E L F N V E L E L Q D V Q D Q I K N L
SOYBN   - - - - - - - - - - - - - - - - - - - - - - - M E N N E I L E E L L N I E V E I Q D V Q E Q I R A L
SOLLC   - - - - - - - - - - - - - - - - - - - - - - M G R T E E L L E E L I N V E V E L L D V Q D Q I K Y L
ORYSJ*  N L P Q E L S I M C D H G S K L A F C P E A K S H L L E M K D N L L A I S H E L I D - - G Q L S P -
HORVU   S L P Q E L S E I C S H G Y K L A F C P E A N Y H L Q G M K D Q L I S V S N K L L D G S G E L S P -
SORBI   - - - - - - - - - - - - - - - - - - - - - - M E D E E N F E E E L L L V E S E L D D I Q G Q I K R L
SELML   - - - - - - - - - - - - - - - - - - - - - - - - - - - - - - - E N V L Q E R D L F A F T A Q I A V L
PHYPA   - - - - - - - - - - - - - - - - - - - - - - - - M E S D L K Q Q L R L V D Q E L H Q V Q D Q I Q S L
VOLCA   - - - - - - - - - - - - - - - - - - - - - - - - - - - - - - - - - - - - - - - - - - - - - - - - - -
                         260                 270                 280                 290                 300
```

MED34

```
ARATH*   IEHQDRLYERKSELKTLLKALAASGSPVASS-------------------
VITVI    LDRQEKLHERQSELKTLLEACKGSGSP-VKD-------------------
SOYBN    IERQESLYERKSELSAILEACKESGNE-ANN-------------------
SOLLC    LDRQEKLHERQFELKALVESCESSSGR-ASD-------------------
ORYSJ*   -QQSDDLHQKRALLKKQIELLGEYTARLTQDEERQQSHSMASTTAHQGHH
HORVU    -QHSEELRQQRAHLNKQIQILGDYMARPTQDDERKRSHSMASTTAAEGHR
SORBI    LDRQEELYERQSQLKALLEVSKVSRNK-SNC-------------------
SELML    CDRQARLLEEQAELRQLIDCCRASE----ED-------------------
PHYPA    LQHQESLLEQQAELQQLIASCRYFACP-----------------------
VOLCA    ----------------MYACVSLPSPPPPPQHIPTRPHAHAHTRPHR---
                  310       320       330       340       350

ARATH*   --------------------------------------------------
VITVI    --------------------------------------------------
SOYBN    --------------------------------------------------
SOLLC    --------------------------------------------------
ORYSJ*   PTSILSSSFVKDTNIFQSPIYTRNEPGESGLCFSSAPYSYMDGLSMPLPS
HORVU    P-PMTPSTFV-DNNRSQSQFYDTNGPWDGGSCYTPAPCPYMDS-NIPLTS
SORBI    --------------------------------------------------
SELML    --------------------------------------------------
PHYPA    --------------------------------------------------
VOLCA    --------------------------------------------------
                  360       370       380       390       400

ARATH*   --------------GGSSAIENWSET-FEWDSRADDVRFNVFGISKYRAN
VITVI    --------------GASVAVENWSGP-FDWDAQADDVRFNIFGISTYRAN
SOYBN    --------------AASSAAENWSGE-FEWDSEADDVRLNVFGISSYRAN
SOLLC    --------------AAAVPVDDWSRP-FEWDSQADDIRFNVFGISKYRAN
ORYSJ*   VQRDYTPRAIDISYTEGSGDKQWSSTHFAWTKELEANNKRVFGNRSFRPN
HORVU    VQRDYTRRNIDISYTDGSGDKKWSSTDFPWTKELEVHNKRVFGNRSFRPN
SORBI    --------------KPSVTLEDWSGS-FPWDSQADDTRLNIFGITSYRSN
SELML    --------------DIQIIEQDWSGN-FEWDNEASNLLLNVFGISTYRRN
PHYPA    --------------TSKSGGVNWSGS-FEWDNRAADIRLNLFGIKSYRQN
VOLCA    --------------------------------------------SCGDTS
                  410       420       430       440       450

ARATH*   QKEIINAIMTGRDVLVIMAAGGGKSLCYQLPAM----LR-GGTTLVVSPL
VITVI    QREIINAVMSGRDVLVIMAAGGGKSLCYQLPAI----LR-DGVALVVSPL
SOYBN    QREIINAIMSGRDVLVIMAAGGGKSLCYQLPAV----LR-DGIALVVSPL
SOLLC    QREIINTIMSGRDVLVIMAAGGGKSLCYQLPAV----LR-DGVALVVSPL
ORYSJ*   QREIINATMSGNDVFVLMPTGGGKSLTYQSQNVSIFEVG-TGIKRV----
HORVU    QREIINATMNGSDVFVLMPTGGGKSLTYQLPAL----IE-EGITLVVCPL
SORBI    QREIINAVMSGRDVLVIMAAGGGKSLCYQLPAV----LR-DGIALVVSPL
SELML    QREIVNALMSNKNVVVVMAAGGGKSLCYQLPAL----LR-PGIALVVSPL
PHYPA    QHEIINAVMSGRDVIVIMAAGGGKSLCYQLPAM----LR-TGTALVISPL
VOLCA    QEAIINATLDGRDVFVLMPTGGGKSLCYQLPAL----MSDSGVTVVVSPL
                  460       470       480       490       500

ARATH*   LSLIQDQVMGLAALGISAYMLTSTSGKENEKFVYKALEKG--EDDLKILY
VITVI    LSLIQDQVMGLAALGIPAYMLTSTTSKEDEKFIYKALEKG--DGDLKILY
SOYBN    LSLIQDQVMGLTALGIPAYMLTS-TNKGDEKFIYKTLEKG--EGELKILY
SOLLC    LSLIQDQVMGLAALGIPAFMLTSTTTKENEKFIYKALEKG--GDELKILY
ORYSJ*   ------GRNDWRKANISAAYLSASMEWSEQQEILRELMSP--TCTYKLLY
HORVU    VSLIQDQIMHLSQANIPATYLSANLEWTEQQRILRDLMSPTSTCNYKLLY
SORBI    LSLIQDQVMGLTALGIPAYMLTSTTNKEVEKFIYRALDKG--EGELKILY
SELML    LSLIQDQVMGLAALGVSAAMLTSTTSKEEEKEIYRVLEKG--DGNLRILY
PHYPA    LSLIQDQVMGLTALGISASMLTSTTTKDEEKSIYKSLEKG--EGDLKILY
VOLCA    VSLIQDQIHHLREAGIGTAHLGGSQEWSEAAGVMESIRSG--ASDVRLLF
                  510       520       530       540       550

ARATH*   VTPEKVSKSKRFMSKLEKCHNAGRLSLISIDEAHCCSQWGHDFRPDY---
VITVI    VTPEKISKSKRFVSKLEKCHHSGCLSLISIDEAHCCSQWGHDFRPDY---
SOYBN    VTPEKISKSKRFMSKLEKCHHAGRLSLISIDEAHCCSQWGHDFRPDY---
SOLLC    VTPEKISKSKRFMSKLEKCHHAGRLSLISVDEAHCCSQWGHDFRPDY---
ORYSJ*   VTPEKIAKSDALLRQLENLYSRGHLSRIVIDEAHCVSQWGHDFRPDYQFF
HORVU    VTPEKIAKSDVLLRQLENLYSRGYLSRIVIDEAHCVSQWGHDFRPDY---
SORBI    VTPEKISKSKRFMSKVEKCHHAGRLSLVAVDEAHCCSQWGHDFRPDY---
SELML    VTPEKIAKSKRFMSKLEKCNRAGRLSLVAIDEAHCCSQWGHDFRPDY---
PHYPA    VTPEKVAKSKRFVSKLEKCNHGGRLSLIAIDEAHCCSQWGHDFRPDY---
VOLCA    VTPEKVARSDALVRLLDGLVAAGRLDRCVVDEAHCVSQWGHDFRPDY---
                  560       570       580       590       600
```

MED34

```
ARATH*   - - K N L S I L K T Q F P K V P M V A L T A T A T Q K V Q N D L I E M L H I P K C V K F V S S V N R
VITVI    - - K N L G I L K T Q F P D V P V V A L T A T A T K K V Q N D L M E M L H I P K C I K F V S T V N R
SOYBN    - - K S L S I L K T Q F P R V P I V A L T A T A T Q R V Q N D L I E M L H I P R C V K F V S T V N R
SOLLC    - - K N L G I L K T Q F P N V P M V A L T A T A T K K V Q D D L M E M L H I P R C V K F V S S V N R
ORYSJ*   I H Q H L G I L K Q K F P Q T P V L A L T A T A T A S V K E D V V Q V L G L A N C I I F R Q S F N R
HORVU    - - Q H L G V L K Q K F P E T P V L A L T A T A T A S V K E D V V Q A L G L A N C V V F K Q S F N R
SORBI    - - K N L S I L K I Q F P T V P M I A L T A T A T S K V Q T D L I E M L H I P R C V K F V S T V N R
SELML    - - K N L G I L K K Q F P K V P M I A L T A T A T G R V Q R D L Q E M L Q I L P C E R F T S S V N R
PHYPA    - - K Q L G I L K K Q F P R V P M I A L T A T A T E R V Q T D L R E M L Q I T R C E K F V S T V N R
VOLCA    - - R E L A V F K S K W P R V P L L A L T A T A T P R V Q A D V R L Q L R I P D C V V F K S S F N R
                             610                 620                 630                 640                 650

ARATH*   P N L F Y S V R E K S A V G K L V V D E I A E F I R E S Y S N N - - - - - E S G I V Y C F S R K E C
VITVI    P N L F Y M V R E K S S V G R V V I D E I A E Y I Q E S Y P N N - - - - - E S G I V Y C F S R K E C
SOYBN    P N L F Y M V K E K S S V G K V V I D E I A E F I Q E S Y P N N - - - - - E S G I V Y C F S R K E C
SOLLC    P N L Y Y M V R E K S S I A K T V V D E I A E Y I Q S S Y P N N - - - - - E S G I V Y C F S R K E C
ORYSJ*   P N L R Y F V W P K T - - - K K C L E D I H N F I H A N H - N K - - - - - E C G I I Y C L S R M D C
HORVU    P N L R Y F V M P K T - - - K K C L E D I D R F I R E N H - H K - - - - - E C G I I Y C L S R M D C
SORBI    P N L F Y K V Y Q K S P V G K V V I D E I T N F I T E S Y P N N - - - - - E S G I V Y C F S R K E C
SELML    P N L F Y E V R D K K Q V A A D V I E D I A L F I K E T Y P S N - - - - - E S G I V Y C F S R K E C
PHYPA    P N L F Y E V R E K K A N G S A A I D D I A S F I L D K Y S K K - - - - - E S G I V Y C F S R K E C
VOLCA    P N L R Y E V V R K S - - - K A I V E D L K T L L L E R F V D R V K K R V Q C G I V Y C Q S R G E C
                             660                 670                 680                 690                 700

ARATH*   E Q I A G D L R E - - - - - - - R G I S A D Y Y H A D M D A N M R E K V H M R W S K N K L Q V I V G T
VITVI    E Q V A K E L R E - - - - - - - R G I S A D Y Y H A D M D V N A R E R V H L R W S N S K L Q V I V G T
SOYBN    E Q V A K E L R E - - - - - - - R G I S A D Y Y H A D M D V N A R E K V H M R W S N N K L Q V I V G T
SOLLC    E Q V A Q E L R E - - - - - - - R G I S A D H Y H A D M D V N A R E R V H L R W S N G K L Q V I V G T
ORYSJ*   E K V A A K L R E - - - - - - - Y G H T A S H Y H G S M D P E D R A N I Q K Q W S K D R I N I I C A T
HORVU    E K V A E K L R E - - - - - - - Y G H Q A S H Y H G N M E P F D R T Q V Q R L W S M D K I N I I C A T
SORBI    E Q V A K E L R D - - - - - - - R G V L A E H Y H A D M D I V A R E K I H M R W S K G Q S Q V I V G T
SELML    E Q V A D A L R K - - - - - - - R Q I S A A H Y H A D M D S G L R T N V H R R W S S N Q L Q V I V G T
PHYPA    E Q V A A E L R K - - - - - - - R G I S A A H Y H A D M K P E T R S S V H M R W S T N K L Q V I V G T
VOLCA    E R V A E E L R K V R Q P N G R M L N A A H Y H A A L S H D E R E Q V Q T R W S N D E V Q V I V A T
                             710                 720                 730                 740                 750

ARATH*   V A F G M G I N K P D V R F V I H H S L S K S M E T Y Y Q E S G R A G R D G L P S E C I L F F R S A
VITVI    V A F G M G I N K P D V R F V I H H S L S K S M E T Y Y Q E S G R A G R D G L P S E C L L Y F R P G
SOYBN    V A F G M G I N K P D V R F V I H H S L S K S M E T Y Y Q E S G R A G R D G L P S E C L L Y F R P G
SOLLC    V A F G M G I N K P D V R F V V H H S L S K S M E T Y Y Q E S G R A G R D G L P S E C V L F F R P A
ORYSJ*   V A F G M G I N K P D V R F V I H H S L P K S I E G Y H Q E C G R A G R D S Q L S T C V L F Y N Y S
HORVU    V A F G M G I N K P D V R F V I H H S L P K S I E G Y H Q E C G R A G R D G Q R S S C V L Y Y N Y S
SORBI    V A F G M G I N K P D V R F V I H H S L S K S M E T Y Y - - - - - - - - - - - - - - - - - - - - - -
SELML    V A F G M G I N K P D V R F V I H H T L S K S L E T Y Y Q E S G R A G R D G L P S R C V L F F R P A
PHYPA    V A F G M G I N K P D V R F V I H H S L S K S L E T Y Y Q E S G R A G R D G L P A H C L L Y F R P A
VOLCA    I A F G M G I N K P D V R F V I H F S L P K S L E G Y H Q E T G R G G R D G A E A I C L L Y Y N Y G
                             760                 770                 780                 790                 800

ARATH*   D V P R Q S S M V F Y E - - - - - - - - - - - - - - - - - - - - - - - - - Y S G L Q N L Y D I V R Y
VITVI    D V P R Q S S M V F Y E - - - - - - - - - - - - - - - - - - - - - - - - - N S G L Q N L Y D I V Q Y
SOYBN    D A P R Q S S M V F Y E - - - - - - - - - - - - - - - - - - - - - - - - - N S G L Q N L Y D I V R Y
SOLLC    D A P R Q S S M V F Y E - - - - - - - - - - - - - - - - - - - - - - - - - N S G L K N L Y D I V R Y
ORYSJ*   D Y I R L K H M V T Q G F A E Q G T S A P R G G - - - - - - S S Q E Q A L E T H K E N L L R M V S Y
HORVU    D Y I R V K H M I T Q G S A E Q V R S S - - S S - - - - - - S S H G Q A L A T H K E N L L C M V S Y
SORBI    - - - - Q S S M V F Y E - - - - - - - - - - - - - - - - - - - - - - - - - N C G L Q N L Y D I V R Y
SELML    D V P R Q S C M V F S E - - - - - - - - - - - - - - - - - - - - - - - - - N T G L Q N L Y A M A R Y
PHYPA    D L P R Q S S M V F A E - - - - - - - - - - - - - - - - - - - - - - - - - M A G L H N L Y A I C R F
VOLCA    D A Q K A R H M L T T S - A Q C G S C T T P G G V F W T R V C N L D H F L L S A V P F K A R I I M F
                             810                 820                 830                 840                 850

ARATH*   C Q S K T K C R R S A F F R H F G E P S - - Q D C N G M C D N C A L S S E V K E V D V S D L S K L V
VITVI    C Q S K R E C R R N A F F R H F A E P L - - Q D C N G M C D N C A F S T E V K E V D V S C L A K A M
SOYBN    C L S K R Q C R R S A F F H H F A E P L - - Q E C N G M C D I C A F S S E V K E V D V S G H A K L M
SOLLC    C Q S K R E C R R S A F F R H F A E P L - - Q E C N G M C D N C A F S N E V E E L D V S S H A K L I
ORYSJ*   C E N D V D C R R L L Q L I H F G E M F N P S C C A K T C D N C L K E L R W V E K D V T N I A R Q L
HORVU    C E N D V D C R R L L Q L I H F G E T F D P S H C A K T C D N C K K G L R W I E K D V T N I A K Q L
SORBI    C - - - - - - - - - - - - - - - - - - - - - Q E C - - - - - - - - - - - - - - - - - - - - H T K I L
SELML    C Q S K Q R C R R A A F F Q Y F A E Q V - - Q E C N G M C D T C A F E N D V V E K D V T E Y G K S L
PHYPA    C Q S K Q A C R R A A F F R H F G E K I - - Q K C N G M C D N C A F T K E V V D T D V T G V S L S L
VOLCA    A E E Q V E C R R V L L M Q H F G E A F D P S Q C R G T C D N C K H R Q - - - S H D L T S M A V Q L
                             860                 870                 880                 890                 900
```

MED34

```
ARATH*  VSMVQETQAKDQRVTMLQLGDKLRNKHKDLIA-----------------E
VITVI   ISLLQDMQDNDQRLTMLQMVDKMKVKNKDLGS-----------------D
SOYBN   VSLLQDMQANDQRSTMLQLVDKMKIKQKELGS-----------------E
SOLLC   VSMLKDIQENDQRVTMLQLVEKLKVKQKNLGP-----------------D
ORYSJ*  VDLVMMTK---QTYSTTHILEVYRGSVNQNVK---KHRHDTLSLHGAGKH
HORVU   VELVLATG---QPCSSSHILEVYRGSLNQNVK---KNRHDMLPLHGAGKH
SORBI   VSLLHDLQLNDQRATLLQLVDKFKVKWKGLGRSNQAV------------D
SELML   VQSVMDVAESDKKTTLLQLVDLWRSQLRQAGNHISGVYGHILQSQTGDKE
PHYPA   VSMVKESNDVEQKLTILQLVEQWRNTDAQLAK-----------------D
VOLCA   VDLIRSAG---QKWSASHVVDVFRGSQAKEIK---AAGHTELPLHGAGKS
                  910       920       930       940       950
```

```
ARATH*  LKRDEVEHLVIKLIVDSVL-KEEFQHTPY--STNAYVTMGPLANQLLQGR
VITVI   LRKEEIEHLVIQLIRDRVL-KEEFQHTAY--ATNAYVTLGLLWKQVSHGK
SOYBN   LKREEIEQLILHLLLARFL-KEEFQHTPY--ATNAYIAVGSLAKQILQGK
SOLLC   LKKDELEQLVIKLLRASVLHKEEFQHTAY--ATNAYVTVGPLAKQVLQGN
ORYSJ*  LAKGEAARILRHLVIEEIL-IEDVKKSENYGSVSSVLK--------FKTY
HORVU   LAKGEAARVLRNLVTEGIL-AEDVKKSDTYGSVSSVLKVNQVKVGGLRSG
SORBI   LKREEIEQLIVQLIVDRVL-KEEFQHTAY--STNAYVTLGPLWKPALEGN
SELML   MSKDDAEQIILQLILDGTL-AKDFQHTAY--ATNAYVKAGSAARLILQGE
PHYPA   LSKDDVERVIVQLILDGIL-KEEFAHTAY--ATNAYVALGTVKLEIVKGA
VOLCA   LQKVRKGSKYMYMYMYDIV-----------------------------GR
                  960       970       980       990      1000
```

```
ARATH*  KTIKMETS---SRQT------------KK------LKRSITFSGLELKLD
VITVI   RIVKLEIS---TKVK------------TKTGNMKSSKRSLTSSGLELKLD
SOYBN   KSVKLEIY---TEQR------------TKDG-VRSAKQCLGSSGLELKLD
SOLLC   KIIKVELS---TRQT------------RTSSSMKSSKR-ERSTGLNDKLD
ORYSJ*  EKIYQRFPTPEKAPKMGVLDESSVPRINKTNQ-QSQVDGSLAAELYEALQ
HORVU   NQIVLKFPTPDKAPKMGKLDESSISQVNKPVQRQSEMDENFSSLLYETLR
SORBI   RPVKLEIAVLGSQAR------------AGGGDISKGTKRSRMSDLEAKLD
SELML   LP-SCKRKVILEQCE------------KRGPVKTTLDDCAMQSGMARMLD
PHYPA   QSAKKK---------------------SKANTAPISEEGMTFSGLGSALD
VOLCA   SPTRIRTP------------------------------------------
                 1010      1020      1030      1040      1050
```

```
ARATH*  ELRKEISAADGS---ILPHTVLSTQQIGSISSQKPVSLQELESI--IGKL
VITVI   ELRKELSSTHGG---IFPHSVLSTQQISILSAQKPTSILEAKTM--IGKL
SOYBN   ELRKELSSAHGG---ILPHSVLSTEQIIQLASQKPNSLEQLEKL--IGKL
SOLLC   ELRKELASIHGG---IFPHSVLSTQQISTLSAQKPKSIEELEKI--IGKL
ORYSJ*  CLRTQIMDENPQ---LLAYHIFKNETLKEISNRMPRTKEELVEINGIGKN
HORVU   ILRSQIA-EAAG---CGVHHIFNNETLKEISTRVPRTKEELLEINGIGKV
SORBI   ELRRELSSSSTGAISIFPHAVLSTQQISLLSCQKPTTEAELEKV--IGKV
SELML   TLRQTLAAADGG---IFPYAVLSSQHIKRLSSHVPTSITELDEI--IGKT
PHYPA   DLRQNLAASHGG---IFPHSVLTSQHISLLASQKPVTMEELEEV--IGKR
VOLCA   ----------------------------------PS--------------
                 1060      1070      1080      1090      1100
```

```
ARATH*  KTEKYGDRILEEVMRHEAVSEQLV--------------------------
VITVI   KTEKYGSKILEEIAKYTETEQPENVPPDDSTLGEGQGSEKKPTKRLKTDE
SOYBN   RTEKYGNRILKQIEKYYDFEPTDK--------------------------
SOLLC   KTEKYGGRILEEIASYESDPQKNN--------------------------
ORYSJ*  KLNKYGDRVLATIEDFLARYPNAT-RKTSSGGSNEHSEAVKKRRGFSVTN
HORVU   KLNKYGDRVLATLEEFLNQFSSGSKRSSSSGGSNEQNEAVKKRRGFTAID
SORBI   KTEKYGNRIIELMRSHIADPGAGN--------------------------
SELML   KSSKYGEQIMTTLNEYTTKNTDGP--------------------------
PHYPA   IAEQYGNEILVSI-------------------------------------
VOLCA   --------------------------------------------------
                 1110      1120      1130      1140      1150
```

```
ARATH*  -------------EDPTKEETCKSRLRKRAKTQKDVVLVESSGEEEA---
VITVI   AQGSEKKPTKRLKTDEGQG--SENRAPKRLKSNKAVVLIESSGDET----
SOYBN   -----------------QE--IDARAAKRLKSKKNLVIIE----------
SOLLC   -------------ADGSKE--TKGNTVKKLKTKKTLVVLESSEDET----
ORYSJ*  TSTNCDDFEERTVQSKKRA--AKTR-TRQEISDAASIVQDVRYIDLELDG
HORVU   TSGNGNDFEERTVQSKKHA--GKTQNSKQGIADAASVIQDIRYIDVDLDG
SORBI   ----------------------GSVAKRRPKKDKDVVCVESSDDE-----
SELML   -------------------DTKEASSSKRVATTGGQARQTKRSRNA----
PHYPA   --------------------------------------------------
VOLCA   --------------------------------------------------
                 1160      1170      1180      1190      1200
```

MED34

```
ARATH*   - - - - - - - - - - - - - - - - - - - - - - - - - - - - - - - - - - - - - - - - - - - -
VITVI    - - - - - - - - - - - - - - - - - - - - - - - - - - - - - - - - - - - - - - - - - - - -
SOYBN    - - - - - - - - - - - - - - - - - - - - - - - - - - - - - - - - - - - - - - - - - - - -
SOLLC    - - - - - - - - - - - - - - - - - - - - - - - - - - - - - - - - - - - - - - - - - - - -
ORYSJ*   C E Q V N - E V P Y S V Q K P V A S G R V L P A W Q S A R I A - - - - - - - - - - - - - -
HORVU    C E D V D E E L C S S V Q Q P V A S G R V L P K W S A G G N A P P P N I F E E F K Y T N
SORBI    - - - - - - - - - - - - - - - - - - - - - - - - - - - - - - - - - - - - - - - - - - - -
SELML    - - - - - - - - - - - - - - - - - - - - - - - - - - - - - - - - - - - - - - - - - - - -
PHYPA    - - - - - - - - - - - - - - - - - - - - - - - - - - - - - - - - - - - - - - - - - - - -
VOLCA    - - - - - - - - - - - - - - - - - - - - - - - - - - - - - - - - - - - - - - - - - - - -
                             1210                1220                1230                1240
```

MED35

```
ARATH*   --------------------------------------------------
VITVI    --------------------------------------------------
SOYBN    --------------------------------------------------
SOLLC    --------------------------------------------------
ORYSJ*   --------------------------------------------------
HORVU    --------------------------------------------------
SORBI    --------------------------------------------------
SELML    --------------------------------------------------
PHYPA    MAQQQQQAGPPGAGRGRGMTLPAWMTGFRVCPWGWHGRRRDPSARSGFLS
VOLCA    M-----------AGRGRAATLPAWMTTRTFRAWRPALRWAPKLPTAPAPL
         10 20 30 40 50

ARATH*   --------------------------------------------------
VITVI    --------------------------------------------------
SOYBN    --------------------------------------------------
SOLLC    --------------------------------------------------
ORYSJ*   --------------------------------------------------
HORVU    --------------------------------------------------
SORBI    --------------------------------------------------
SELML    --------------------------------------------------
PHYPA    IQFLELPGSLFEEGVEECVSIRPSFSARYSFFISLIVVFEHKRKTHDCCP
VOLCA    PRGSTDPATHRCHAARLLLRGRHPGTLQEALGVGRITSRRKGRELRGLRP
         60 70 80 90 100

ARATH*   -----M--------------------------------------ANN---
VITVI    -----M--------------------------------------------
SOYBN    -----M--------------------------------------ANNSQP
SOLLC    -----M--------------------------------------ASNPPP
ORYSJ*   -----M--------------------------------------ASNMQP
HORVU    -----M--------------------------------------ASNMQT
SORBI    -----M--------------------------------------ASNMQA
SELML    -----M--------------------------------------SNVPPP
PHYPA    CNVWSM--VSWESGGPPSPGMPPGGGPPQGHL------------QTNPGA
VOLCA    RGLGHHREQALRRQGIHRQDNSRLPGRAHRHLCLLRRQDSRRRWASNLGL
         110 120 130 140 150

ARATH*   ---------------------------------------PPQSSG-----
VITVI    --------------------------------------------------
SOYBN    SS------------------------------------------------
SOLLC    SGPQ----PL--------------------WPPSVGST-PPQGFG-----
ORYSJ*   SGPPQQSRPPMMGSSVPPQNLGPPMPMQQSRPPMMGSSVPPQNLG----P
HORVU    PGPPQ---PP--------------------RPPMMGSTA-PQNMG----Q
SORBI    PGPPQ---PP--------------------RPPMMGSSAQPQNLG----P
SELML    MSSQ----A---------------------------------------AY
PHYPA    VGAM----PP--------------------RMMGMGPSAPTSTFQ----S
VOLCA    HGAP----P----------------------PGMMGPRPPPGPFPGMGPP
         160 170 180 190 200

ARATH*   ---TQFRPMVPGQQGQHFVPA--ASQPFHPYGHVPPN----------VQS
VITVI    --------------------------------------------------
SOYBN    ---MQFRPVTQAQQGQPFVPM--NSQQFGPAGHAIPS--SNAGMPV-IQG
SOLLC    SFPMQFRPALSTQQGQHFAPPISASPQYRPVGQT-----PNAGMPP-GQG
ORYSJ*   PMPMQFRPVIHQQQPPQFMQP---GQQFRPVGQAMPG--ANIGMP----G
HORVU    PMPMQWPP-GPPQQPPQFMQP--APQQYRPVGQAMPG--VNMGMP----G
SORBI    PMPMQFRPVVPSQQPPQFMPP--AAQQFRPVGQPMPG--ANIGMP----G
SELML    PPPMQMRP---NQASGPFAQP-----------------------------
PHYPA    APQMPYRPGGPPQQGQQFMGP--GGQQYHPSVGMGPP--QQLGMPPNMQG
VOLCA    PPGMPYPP------PGAHMPP--GPGMMPPPYGMQPGGPPPMGMPP----
         210 220 230 240 250

ARATH*   QPPQYSQPIQQQQLFPVRPG---QPVHITSSSQAVSVPYIQTNKILTSGS
VITVI    ----------------------------------------------VSSA
SOYBN    QQLQYSQPMQQ---LTQRPM---QPGHPAPSSQAIPMQYIQTNRPLTSIP
SOLLC    QIPQFSQTMQQ---FPPRPG---QPGHGTPSSQAIQMSYNQ------SSI
ORYSJ*   QMPHFQQPTQH---LP-------HSGQVPPASQAVPMAY-QPARPMSSGP
HORVU    QMQHFQQPGPH---MP-------HSGHVPPASQAVPMPY-QAVRPMSSAP
SORBI    QMPHFPQPGQH---LP-------HSNQGPPASQGVPMVY-QPARPMSSAP
SELML    ----------------QRPP------QGPPGMYGSAVPF-----------
PHYPA    MNPQQPQQFST-----RPPA---QQSQGQPSSQSSGMPFGAQPRPISSTP
VOLCA    --PAGWHPGMPPPGYPPRPMPGFPPGMPPPGMPPPGMPGMPMGPPPMAGG
         260 270 280 290 300
```

MED35

```
ARATH*  TQP---------------QPNAPP----MTG----FATS-GPPFSSPYTF
VITVI   NYS---------------KDTLVL---SFPT----MKHSVNPT---SSTL
SOYBN   PHS---------------QQNVPPLSNHMPG----LAVSVAAPHSSYFTL
SOLLC   SQP---------------QQVNPPLNSHMPG----VSGA-GNPFSSSYTV
ORYSJ*  LQP-----------------PATFSGGHMPT----MGGPIPPP---SYT-
HORVU   MQP---------------QQQAVFPGGHMPT----MGTPMPPP---SYTY
SORBI   MQP---------------QQQTAYPGGHLPT----MGAPMQPP---TYTY
SELML   --P---------------QQGNAYPGS-----------------------
PHYPA   LPP--QAQQLQQQPSSQQQQQQPVPPPSGPGNHALQPASSYPQFSSTSSF
VOLCA   MQPLPGAPPGHPAPQQQAPQQQPPPQAAAPG-------------------
                  310       320       330       340       350

ARATH*  VPSS-YPQQQPTSLVQPNSQMHVAGVPPAANTWPVPVNQSTSLVSPVQQT
VITVI   IIL-----------------------------------------------
SOYBN   SYGQQQDNANALAQYQHPPQMF---APPSGQPWPSSASQSAVAVTSVQPA
SOLLC   QSSS---------------QMHGPTFPAGGQPWLSSGSQTTPVGDPTPPS
ORYSJ*  ----------------------------AMQSWGTAPGQNVPL----VQP
HORVU   QPTT---------------------VPPGAQPWGTVPGQGAPLVSPMVQP
SORBI   QPTS---------------------IPPVVQAWGTGPGQNVPHVPHLVQS
SELML   --------------------YQVGNPGQLLQSYPVNAGQTNNGMAQGLHS
PHYPA   SQPA-V-SYGLAPPYQVPTQMQIQQPQPGSQPWSGVGGQTSSHSASL--
VOLCA   ------------QQAQGQQQAQQQHSAAAQQAAAASAAAT----------
                  360       370       380       390       400

ARATH*  G-QQTPVAVSTDP-GNLTPQSASDWQEHTSADGRKYYYNKRTKQSNWEKP
VITVI   --LRTSATKAGNV-PNPTHQSSSDWQEHTSADGRRYYYNKKTRLSSWEKP
SOYBN   GVQSSGATSTDAVINATNQQSLSDWQEHTSADGRRYYYNKRTRQSSWEKP
SOLLC   S-HQLLAVAPAVPASTASQQTASDWQEYEAADGRRYYYNKNTKQSSWEKP
ORYSJ*  G-HQPMSSSATMPSINSSETSSSDWQEHTSADGKKYYYNKKTRQSSWEKP
HORVU   G-HQ--SLSASVPPVSSTEPSSSADWQEHSSGDGKKYYYNKRTKQSSWEKP
SORBI   G-HQPVSAPTTLPSVNLSEPSSSDWQEHTAAEGKKYYYNKKTRQSSWEKP
SELML   G-------------VPPPGSRASEWQEHFSPDGRRYYYNKRTKQSSWEKP
PHYPA   --QSVQPPPSSGLSIAVPSPGAADWQEHVAPDGRRYYYNRRTKQSSWEKP
VOLCA   --------AAQGAAAAASSGPPKEWTEHTAPDGRKYYYNAKTKQSSWDKP
                  410       420       430       440       450

ARATH*  LELMTPLERADASTVWKEFTTPEGKKYYYNKVTKESKWTI----PEDLKL
VITVI   LELMTPIERADASTVWKEFTTPEGRKYYYNKVTKQSKWTI----PEELKL
SOYBN   LELMSPIERADASTVWKEFTSSEGRKYYYNKVTQQSTWSI----PEELKL
SOLLC   LELMTPLERADASTVWKEFTTADGRKYYYNKETKQSKWTM----PDELKL
ORYSJ*  AELMTPLERADASTEWKEFTTQEGRKYYYNKVTKQSKWTI----PDELKI
HORVU   AELMTPLERADASTEWKEFTTAEGRKYYYNKVTKQSKWSI----PDELRI
SORBI   VELMTPLERADASTEWKEFTTPEGRKYYFNKVTKQSKWTI----PDELKV
SELML   DELMTPTERADASTVWKEFVTADSRKYYYNKLTRQSTWTM----PEEMRV
PHYPA   TELMTPIERADASTPWKEFTTADGRKYYYNKEKTRRDHLVMCLSAGYVQL
VOLCA   EEFMTPQEKAEASG-WKEYTAPDGRKYYHNRVTKESKWTM----PDELKA
                  460       470       480       490       500

ARATH*  AREQAQL--ASEKTSLSEAGSTPLSHH-AASSSDLAVS-TVTSVVPSTSS
VITVI   AREQAEK--SVSQETQSEMGTTSNEPAVVAVSLAETPSTASVSVSSTTSS
SOYBN   AREQAQN--AANQGMQSETSDTCNAVVSS--TETPTPT-AANAASLNTSL
SOLLC   ARELAEN--VASQVVQTGTSTNSGVQVSEAVTSTEQPS-AVTPVSSTPSS
ORYSJ*  ARELAEK--ASNPRPDQETETTAGAPS-GPTSNSVEPS--SVPANQSSTT
HORVU   ARELAEK--TSNQQPVREIESTTVAPV-GSTSVSVEPS--VLPAKQSSSL
SORBI   ARELAEK--ASNQQPDRESGIAASALV-G--SAASEPS--TIPANQSSSA
SELML   AREQADR--GYMGVAKMETAPAPV--------AQTLTPA-----------
PHYPA   AREQVEKSVGPVSQAAVLTPPTSV------------KQPA--SAALPLPQTT
VOLCA   ALAGGGGGAAAGTGGAIASAPAPA----AGGAAAAKPA-AVQVVSLAAAD
                  510       520       530       540       550

ARATH*  ALTGHSSSPIQAGLAVPVTRPPSVA-------------------------
VITVI   TISGMTSSPVPVTPVVAVVNPPPVVVSGTSAIP-IAQSAVTTSAVGVQPS
SOYBN   TSNGLASSPSSVTPIAA--TDSQRLVSGLSGTS-VSHSMATPSTTGVEPS
SOLLC   TVSGVPSSPVPVTPAVSDVNTPPLVVSGSSAIP-TVSFAVTSSAGISSP-
ORYSJ*  I-MGAPSTLDAAANSVPPGAGPSHNMENTSSSSNTAMQNGGPST-VVTPV
HORVU   VGTVASSAHDATANLPPPGAAPSYNGDIPSSGS---MQNGGTSAAVVVPV
SORBI   VGIIAPSSHDGSSNSAPPGAAPSHNVENTSSSI-VGMQNGAPSTAVVPVA
SELML   -ATGIPS-------------------------------------------
PHYPA   ISNVAANS------------------------------------------
VOLCA   GAASRGGEAAAATPA-----------------------------------
                  560       570       580       590       600
```

MED35

```
ARATH*  ---PVTPTSGAIS----------DTEATTIKG--DNLSSRGADDSNDGAT
VITVI   ---MGTPLPAAVSGSTGVAAAFINPNATSMTS-FENLSA----DATNGAS
SOYBN   TVVTTSAAPTIVAGSSGLAEN--SPQQPKMPPVVENQASQDF-ASANGSS
SOLLC   ----------AVSGNTR-SAALANAYQTQMSG-IENLSPQVA-SSLSGAS
ORYSJ*  ---ISTEIP-SVASDAGISRA--NNEYPSLASTA---------DTQNGAS
HORVU   ---TSTGVP-QLASDAGTNRN--TYGSSSLPSTT---------DTKVGAS
SORBI   ---TSTEVP-LVATDAGASRN--NNENSSLTTGV---------DTQDGTS
SELML   --------PAPLAGS----------GADAAESTEETQQ-----------T
PHYPA   ---LTSVLPGATTANAKLQVA----GDAKKDLKEE---------VMNGTS
VOLCA   ----------AAGTGGGAASSLASPSATEGPS------------------
                  610       620       630       640       650
```

```
ARATH*  AQNNEAENKEMSVNGKANLSP-AGDKAN--VEEPMVYATKQEAKAAFKSL
VITVI   MQDIEEAKKGVAVAGKINVTP-LEEKTL--DDEPLVYSTKLEAKNAFKAL
SOYBN   LQDIEEAKRPLPVVGKNNVTP-PEEKTN--DDETLVYANKLEAKNAFKAL
SOLLC   SQDIEEAKKGMAVAGKINVVP-AEEKSA--DEEPFLYATKQEAKHAFKSL
ORYSJ*  AEELEEAKKTMAVPGKINVTP-VEDKTS--EEEPVTYANKLEAKNAFKAL
HORVU   AEDLEEAKKTMPTAGKINVTP-LEDKTI--EEEPVVYATKLEAKNAFKSL
SORBI   VEDLEEAKKTMPVAGKINVTP-LEEKTN--EEEPVVYASKMEAKNAFKSL
SELML   QQDLE-----------VGISE-GKSFST--EEPLISYATKNEAKSAFKEL
PHYPA   AQDFEEAKKVMLVTSKINISPVLEEKPSLVSEEPQTYASKTEAKNAFKEL
VOLCA   ----------------------QSAASA--DPKSYTFATKEEAKDAFKEL
                  660       670       680       690       700
```

```
ARATH*  LESVNVHSDWTWEQTLKEIVHDKRYGALRTLGERKQAFNE----------
VITVI   LESANVESDWTWDQAMKAIINDKRYGALKTLGERKQAFNE----------
SOYBN   LESVSVQSDWTWEQAMREIINDKRYNALKTLGERKQAFNE----------
SOLLC   LESATVESDWTWEQTMRVIINDKRYGALKTLGERKQAFNE----------
ORYSJ*  LESSNVESDWTWDQAMRVIINDKRYGALKTLGERKQAFNE----------
HORVU   LESANVQSDWSWDQAMRVIISDKRYGALKTLGERKQAFNE----------
SORBI   LESVNVESDWTWDQTMRVIINDKRYGALKTLGERKQAFNE----------
SELML   LEAMHVQSDWTWDQAMRVIINDKRYGALKSLGERKQAFNE----------
PHYPA   LESVHVEADWTWEQAMRVIINDKRYGALKTLGERKQAFNEASVEPSIIAA
VOLCA   LASVGCRSDWTWEQAMRLTVNDPRYSALKTLGERKQTFNE----------
                  710       720       730       740       750
```

```
ARATH*  --------YLGQRKKVEAEERRRRQKKAREEFVKMLEECEELSSSLKWSK
VITVI   --------YLGQRKKIEAEERRMRQKKAREEFTTMLEECKELTSSIKWSK
SOYBN   --------YLGQRKKLEAEERRMKQKRAREEFTKMLEECKELTSSMRWSK
SOLLC   --------YLMQRKKQEAEERRLRQRKAKEEFTKMLEESKELTSSTRWSK
ORYSJ*  --------YLNQRKKLEAEERRIKQRKARDDFLAMLEESKELTSSTRWSK
HORVU   --------YLNQRKKIEVEERRVKQRKARDDFLTMLEECKDLTSSMRWSK
SORBI   --------YLNQRKKFEAEEKRVKQRKARDDFLAMLEECKELTSLTRWSK
SELML   --------YLAQRKKLDLEEKRLKQKKAREDFIKMLEESKELTSAMRWSK
PHYPA   NCTFYSRHYLAHRKKQESEEKRAKQKIAREQFRAMLEESKDLTSSMRWSK
VOLCA   --------YVQARRNEEKEEERRRQRQAREDFLAMLASSEELKVTHPFRR
                  760       770       780       790       800
```

```
ARATH*  AMSLFENDQRFKAVDRPRDREDLFDNYIVELERKEREKAAEEHRQYMADY
VITVI   AVDMFQDDERFKAVERSRDREDLFENFIMELQKKERTKALEEQKRNRMEY
SOYBN   AISMFENDERFNAVERPRDREDLFESYMVELERKEKENAAEEHRQNIAEY
SOLLC   AVTMFEDDERFKGVEREADREDLFRNYLVDLQKKERSKAQEEYRRNRLEY
ORYSJ*  AITMFEDDERFSAVERPREREDLFESYLMELQKKERARAAEEHKRHIAEY
HORVU   AITMFGHDERFNAVERPKEREDLFENYLVELQKKEKAKAAEEHKRRIAEY
SORBI   AILMFEDDERFKAVERPREREDLFENYLVELHKKEKAKAAEEHKRYVAEY
SELML   VVSLFESDPRFHAVDKEREREDLFDDYLLDLERKERDKAREEKKKSRADF
PHYPA   AFSIFEDDPRFLAVERDREREELYEDYMVDLERKEREKAREERKKYIAEY
VOLCA   AKELFEDDPRWKAVP-EREREELFHDAQRERDKREKEERRQERKRRCNAF
                  810       820       830       840       850
```

```
ARATH*  RKFLETCDYIKAGTQWRKIQDRLEDDDRCSCLEKIDRLIGFEEYILDLEK
VITVI   RQFLESCDFIKVNSQWRKVQDRLEDDERCSRLEKIDRLEIFQEYIRDLER
SOYBN   RKFLESCDYVKVNSPWRKIQDRLEDDDRYLRLEKIDRLLVFQDYIRDLEK
SOLLC   KQFLETCGFIKVDTQWRKVQDLLEDDERCSRLEKLDRLDIFQEYIRDLEK
ORYSJ*  RAFLESCEFIKTSTQWRKVQERLEDDERCSRLEKIDRLEIFQEYIRDLEK
HORVU   REFLESCDFIKANTQWRKVQDRLEDDERYTRLEKIDRLDVFQDYIRHLEK
SORBI   RAFLESCDFIKATTQWRKVQERLEDDERYSRLEKIDRLDIFQEYIRHLEK
SELML   RSYLESCDFIKVNSHWRKIQDKLDDDERWSRLDKMDRLEVFQEYIRDLEK
PHYPA   RSYLENCGFIKATTQWRKVQDRLEDDERCSRLDKLDRLEVFQEYIRDLEK
VOLCA   RELLESSG-LKPGAEWRKVSKRLEGEEEYEALDKVERLEVFQEFVKDLER
                  860       870       880       890       900
```

MED35

```
ARATH*   EEEELKRVEKEHVRRAERKNRDAFRTLLEEHVAAGILTAKTYWLDYCIEL
VITVI    EEEEQRKIQKEQLRRAERKNRDEFRKLMEEHVAAGTLTAKTHWRDYCMKV
SOYBN    EEEEQKRIQKDRIRRGERKNRDAFRKLLGEHVSAGILTAKTQWREYCLKV
SOLLC    EDEEQRKLQKEQLRRAERKNRDAFRKMIEEHIAAGMLTAKTYWRDYWQMV
ORYSJ*   EEEEHKRIHKEQVRRQERKNRDEFRKMLEEHVAEGMLTAKTRWRDYCAQV
HORVU    EEEEQKRIQKEQLRRQERKNRDDFRKMMEEHVADCTLNAKTYWRDYCSQV
SORBI    EEEEQKRIQKDQVRRQERKNRDGFRKMLEEHVADGTLNARTRWRDYCAQI
SELML    EEEEEKKMQKEQIRRKERKNRDEFRSLLEFHKAAGILVARCPWRDYLAKI
PHYPA    EEDEAKRLQKDQLRRKERKHRDEFRKLMDEHKAAGILTAKTVWRDYLTKV
VOLCA    REKEEKEREKEERKRQERRARDAFKDLLRRHRDEGLISVRSRWKEYSEQV
                   910       920       930       940       950
```

```
ARATH*   KDLPQYQAVASNTSGSTPKDLFEDVTEELEKQYHEDKSYVKDAMKSRKIS
VITVI    KDSSPYLAVASNTSGSTPKDLFEDVAEELEKQYHEDKARIKDAMKLSKVT
SOYBN    RDLPQYQAVASNTSGSTPKDLFEDVAEDLEKQYHEDKTLIKDTVKSGKIT
SOLLC    KESVAYQAVASNTSGSTPKDLFEDVTEELEKQYHEDKIHVKDVVKSEKIT
ORYSJ*   KDSSVYLAVASNISGSMPKDLFEDVMEELEKQYQDDKARIKEAVKSGKIP
HORVU    KDSRAYLAVASNLSGSMPKELFDDVMEELDKQYQDDRALIKDEVKSGKIP
SORBI    KDSESYLAVASNTSGSTPKELFDDVIEELDKQYQEDKTQIKEVVKSGKIP
SELML    KEHPAYQAICTNLSGSTPKELFMDVLEELDKLYLEDKAKIKDIMKVGKIT
PHYPA    KDNSAYQAVASNTSGTTPKELFEDVVEELVKQYHDDKARVKDVMKAGKIS
VOLCA    VSEEAYRAVEANTSGSRPKELFEDLIEEMEQEYEKAKDTLKVLLKDGGFT
                   960       970       980       990      1000
```

```
ARATH*   MVSSWLFEDFKSAISEDLSTQQISDINL----------------------
VITVI    IASTWTFGDFKAAILDDVGSPNISDVNL----------------------
SOYBN    VVTTSVFEEFKVAVLEGAACQTISEINL----------------------
SOLLC    ISPTCTFEDFKVAILEGISSPSIQDVNL----------------------
ORYSJ*   MTTSWTLEDFETAVTEDDTLKGITNINM----------------------
HORVU    MMSSWTLEDFQAAVTEHEKCKGVSKINI----------------------
SORBI    MTTSWTLEEFQTAILEDDALKGISTINV----------------------
SELML    VVPTTTYDDFKAALAEAGDLTAISELHI----------------------
PHYPA    VGGTWTFDKFKAAYAEAGDLAAIAVPNLKKITAGAVLCGVLATSILSADA
VOLCA    VTPTTSYEQFEDALAAAAAGSPDADTTGKLAALLPT--------------
                  1010      1020      1030      1040      1050
```

```
ARATH*   ------KLIYDDLVGRVKEKEEKEARKLQRLAEEFTNLLHTFK-EITVAS
VITVI    ------KLVFEELLDRIKEKEEKEAKKRQRLADDFNDLLRSKK-EITASS
SOYBN    ------KLIFEELLERAKEKEEKEAKKRQRLADDFTNLLYTFK-DITTSS
SOLLC    ----------QDLVERAKEKEEKEAKKRQRLAKDFTDKLSSIK-EITDSS
ORYSJ*   ------KLIFDDQVERLREKEVKEAKKRQRLGENFSDLLYSIK-EISASS
HORVU    ------KLIYEDLIERLKEKDLKEAKKRQRLGDNFLDLLYSIK-EITAAS
SORBI    ------KLIYDDQLERLKEKEQKEAKKRQRLGENFSDLLYSIK-EISASS
SELML    ------KLAFEDALERLKEKEEKEAKKRRRLAEEFSTLLRSNK-TITATS
PHYPA    DQFWLQKLVFEDYVERAKEKEEKEAKKRRRMADDFTNLLRSTK-AVTLSS
VOLCA    ----YKRTYYEETVAKAKVREAEEAERRRRARDKLSSYMRHCRPAVREDS
                  1060      1070      1080      1090      1100
```

```
ARATH*   NWEDSKQLVEESQEYRSIGDESVSQGLFEEYITSLQEKAKEKERKRD-EE
VITVI    NWEDCKPLFEESQEYRSIGEESFGREIFEEYIAHLQEKAKEKERKRE-EE
SOYBN    KWEDCKSLFEETQEYRSIGDESYSREIFEEYITYLKEKAKEKDRKRE-EE
SOLLC    SWEESKELVEDSSEFRAIGEETISRAVFEEYVAWLQEKAKEKERKRE-EE
ORYSJ*   TWDDSKALFEDSQEYKALDSETYSRELFEECVVHLKERLKEKERLRE-EE
HORVU    TWDDSKSLFDDTQEYRDLGGETYAKELFEEYIVRLKERLKEKERMRE-EE
SORBI    TWDDSKQLFEDSEEFRALDSETYARELFEECVVHLKERLKEKERLRE-EE
SELML    NWEESKPLLQETVEFRAIDDEVVLRKLFDEHVAHLQQKLKEKDRKRE-KE
PHYPA    KWEDVRPLIEDSQEYRALPDDGQRKKLFEDYVIHLVRKAKEKDKKREKEE
VOLCA    AWEDWLREHEREPEVKALPLEEV-HAMFDEH-----------DRSDE-DE
                  1110      1120      1130      1140      1150
```

```
ARATH*   KVRKEKERDEKEKRKDKDKER-REKER----------EREKEKGKERSKR
VITVI    KAKKEKEREEKEKRKEKER---KEKDR----------DREREKGKERSRK
SOYBN    KAKKEKEREEKRKEKEK-----KEKDR----------EREKDKSKERNKK
SOLLC    KVKKEKEKDEKEKRKDKERRE-KEKER----------EKEKERGKERSRK
ORYSJ*   KARKEKEREEKERRKEKEK---KEKERKEKERD-KEKEREKDKGKDRSRK
HORVU    KAKKEKDREEREKKKEKEKEK-KEKDRKEKERD-REKEREKEKGKDRSRR
SORBI    KAKREKEREEKEKKKEKEKER-KDKERKEKERDHREKEREKEKGKDRSRR
SELML    E-KKDKDKDSKD-------------DK----------DKERKKDKSSKKD
PHYPA    KVEKEKEKE-KEKEKEKD----KDKDK----------DREKRKEKEKEKE
VOLCA    DHKSKKHKKDKDRERDRDRERERERER----------EREREGGRAGDS
                  1160      1170      1180      1190      1200
```

MED35

```
ARATH*   EESDGETAMD----------------VSEGHKDEKRKGKDR------DRK
VITVI    DETESENV-D--------------VTGSYGYKEDKKREKDK------DRK
SOYBN    DETDSDNQ-D--------------MADSHGYKEEKKKEKDK------ERK
SOLLC    DEPDSVIM-D--------------VTESFEHKEERKREKDR------ERK
ORYSJ*   DEMDIDAV-D---------------ADSHGSK-DKKREKDK------EKK
HORVU    DEMDVDD--D---------------VEIHGSK-DKKREKDK------EKK
SORBI    DEMDLDGA-D---------------LDTHGSK-DKKR--DK------EKK
SELML    RNEDGEPALS---------------------KEEKKKDKDRDRDK--EKR
PHYPA    KEKEKEKEKSSHEKISLHVDEMEGVTNHSSHKDERRKDKDRDKDKEREKK
VOLCA    DQDSGEI-------------------------------------------
                             1210                1220                1230                1240                1250
```

```
ARATH*   HRRRHHNNSDE----------------------------D--VSSDRDDR
VITVI    HRKRHQSAVD-----------------------------D--ASSDKEEK
SOYBN    HRKRHQSSID-----------------------------D--VDSEKEEK
SOLLC    HRKRHHSSNDELTSDKDEKEESERDKERKHRKRHQSSNDE--VASDRDER
ORYSJ*   HKRHHHDTAD-----------------------------D--VSSERDEK
HORVU    HKRRHHDTTD----------------------------------SEKDEK
SORBI    HKRRHHDT-D-----------------------------D--VSSERDDK
SELML    HRRRHRSASS-----------------------------D--DGSDRDDR
PHYPA    HRKRHRSVSSE----------------------------EIATDKDRDEK
VOLCA    ----------------------------------------------RD--
                             1260                1270                1280                1290                1300
```

```
ARATH*   DESKKSSRKHGNDRKK-SRKVGTP--------------------------
VITVI    EESKK-SRRHGSDRKK-SRKHAYTPESDTESRHKRHKREHWDGSRRNGGY
SOYBN    EESKK-SRRHGSERKK-SRKHANSPESDNENRHRRHKRDHWDGSRKTGGH
SOLLC    EDLRK-SHRHSSDRKK-SKKHAHSPESDGESRHKRYRRDR-DGSRRNGGH
ORYSJ*   DDSKK-SRRHSSDRKK-SRKHTHASDSDSENRHKRHKKDR-DSSRRNGTR
HORVU    DDSRK-SRRHSSDRKK-SRKHTHASDSDSENRHRRHKRDR-DSSRKNGAH
SORBI    DDSKK-SRRHSSDRKK-SRKHAHGSDSDSENRHKRHKKDR-DSSRRNGGH
SELML    DEARK-SRKHGHEHRRSSRRHGHDSDSDTEAKPKRQKRER-------RST
PHYPA    DSSRK-SRRHGSDHKKSSRKH--ESESEGDTKQKRHRR---DGGRRNGGP
VOLCA    --------------------------------------------------
                             1310                1320                1330                1340                1350
```

```
ARATH*   -----------------------------
VITVI    EELEDGEL--------------GEDGEIR
SOYBN    EELEDGEL--------------GDDAEN-
SOLLC    EELEDGEL--------------GEDGES-
ORYSJ*   DDLEDGEL--------------GEDGEIH
HORVU    EELEDGEL--------------GEDGEIH
SORBI    -ELEDGEL--------------GEDGEVN
SELML    EELEDGEILGLHSWSCLFVDRLRGSGFWY
PHYPA    DELEDGEL--------------GEDGEIQ
VOLCA    -----------------------------
                             1360                1370
```

MED36

```
ARATH*   -MRPP--LTGSG-GGFSGGRGRGGYSGGRGD-GGFS--GGRGG-GG-RGG
VITVI    -MRPPAGRGRGG-GGFGGGGFRGRGDGGRG--------RGRGGFGG-RGG
SOYBN    -MAPP--RGRGGFGGGGGGGFRGGGRGD----RGRG--RGGGGRGG-DRG
SOLLC    MVAPT--RGRGG-GGFRGGRGDGGGRGGRGGRGGFS--GGRGGFGS---G
ORYSJ*   -MRPP--RGRGF-GRGGGGRGDGGGRGGGGG-RGFGRVGDSGGRGG----
HORVU    --------------------------------------------------
SORBI    -MRPP---ARGG-RGGRGGRFDGGGRGGGGG-RGFG--GGRGGGGG----
SELML    -M-----RGRG--GGGRGGR-DGGGRG-----------RGRGRGGGRDGG
PHYPA    -MRPP--LTRGG-GGFRGGRGDGGFGGGRGGGRGFG--GGRGGRGG-DRG
VOLCA    -MGFGDRGGRG--GGFGRGGGRGGGRG-----------GGRGGAPGGGRG
                  10        20        30        40        50

ARATH*   GRGFSDRGGRGR--GRGPPRGGARGG--RGPAGRGGMKGGSKVIVEPHRH
VITVI    SDMNSRGGGRG---DRG-GRGGRGGGRGRGGRGGGGMKGGSRVVVEPHRH
SOYBN    TPFKARGGGRG---GGG-RGGGRGGG--RGGG-RGGMKGGSKVVVQPHRH
SOLLC    GSGMKRGGGRG---GRG-DRGGRGGG--RG---RGGMKGGSKVVVEPHRH
ORYSJ*   ------RGGRG---GRT-PR-GRGGG-RGGGG-RGGMKGGSKVVVVPHKH
HORVU    ------------------------------------MKGGAKVVVVPHKH
SORBI    ------RGGRG---GRT-PRGGRGGG-GRGGR-GGGMKGGSKAVVVPHKH
SELML    GRSGGRG-------------FGRGGG--RGGR-GGGMKGGAKVSLEPHRH
PHYPA    GRGGGRGGGRGA--PRG-GRGGRGGG---GGR-GGGMKGGAKVIIEPHRH
VOLCA    GRGGGRGGGFGARGGVA-KRGGRGGG--RGGG-RGGMKGGSKAVIEAHRH
                  60        70        80        90       100

ARATH*   AGVFIAKGKEDALVTKNLVPGEAVYNEKRISVQNEDGTKTEYRVWNPFRS
VITVI    EGVFIAKGKEDALVTKNMVPGEAVYNEKRISVQNEDGSKIEYRVWNPFRS
SOYBN    DGIFIAKGKEDALVTKNLVPGEAVYNEKRITVQNEDGTKEEYRVWNPFRS
SOLLC    GGVFIAKGKEDALCTKNLVPGEAVYNEKRISVQNEDGTKVEYRVWNPFRS
ORYSJ*   DGVFIAKAKEDALCTKNMVPGESVYGEKRISVQNEDGTKVEYRVWNPFRS
HORVU    DGVFIAKAKEDALCTKNMVAGESVYGEKRVSVQNEDSSKVEYRVWNPFRS
SORBI    AGVFISKSKEDALCTKNMVPGESVYAEKRVSVQNEDGTKVEYRVWNPFRS
SELML    EGIFIAKAKDDALVTRNLVPGESVYGEKRITVE-ADGEKVEYRVWNPFRS
PHYPA    EGVFVAKAKEDALVTKNLVPGESVYGEKRISVDNEDGTKTEYRVWNPFRS
VOLCA    DGVFIARGKEDALVTKNLVPGVSVYGEKRIAVEDE-GDKIEYRVWNPFRS
                 110       120       130       140       150

ARATH*   KLAAAILGGVDNIWIKPGAKVLYLGAASGTTVSHVSDLVGPEGCVYAVEF
VITVI    KLAAAILGGVDEIWIKPGARVLYLGAASGTTVSHVSDVVGPTGVVYAVEF
SOYBN    KLAAAILGGVDNIWIKPGARVLYLGAASGTTVSHVSDVVGPTGVVYAVEF
SOLLC    KLAAAILGGVDDIWIKPGARVLYLGAASGTTVSHVSDLVGPEGVVYAVEF
ORYSJ*   KLAAAVLGGVDNIWIAPGTRVLYLGGASGTTVSHVSDIVGPTGLVYAVEF
HORVU    KLAAAVLGGVDNIWIAPGTRVLYLGAASGTTVSHVSDIVGPTGLVYAVEF
SORBI    KLAAAVLGGVDNIWIAPGTRVLYLGAASGTTVSHVSDIVGPDGLVYAVEF
SELML    KLAAAVLGGVDNIWIKPGSRVLYLGAASGTTVSHVSDVIGPTGIVYAVEF
PHYPA    KLAAAILGGVDNIWIKPGAHVLYLGAASGTTVSHVSDVVGPSGIVYAVEF
VOLCA    KLAASILAGVDNIYVKPGSKLLYLGAASGTSVSHCSDLVGPEGAVYAVEF
                 160       170       180       190       200

ARATH*   SHRSGRDLVNMAKKRTNVIPIIEDARHPAKYRMLVGMVDVIFSDVAQPDQ
VITVI    SHRSGRDLVNMAKKRTNVIPIIEDARHPAKYRMLVGMVDVIFSDVAQPDQ
SOYBN    SHRSGRDLVNMAKKRTNVIPIIEDARHPAKYRMLVGMVDVIFSDVAQPDQ
SOLLC    SHRSGRDLVNMAKKRTNVIPIIEDARHPAKYRMLVGMVDVIFSDVAQPDQ
ORYSJ*   SHRSGRDLVNMAKKRTNVIPIIEDARHPARYRMLVGMVDVIFSDVAQPDQ
HORVU    SHRSGRDLVNMAKKRTNVIPIIEDARHPAKYRMLVGMVDVIFSDVAQPDQ
SORBI    SHRSGRDLVNMAKKRTNVIPIIEDARHPARYRMLVGMVDVIFSDVAQPDQ
SELML    SHRCGRDLVNMAKKRTNVIPIIEDARHPARYRMLVGMVDVIFADVAQPDQ
PHYPA    SHRSGRDLVNMAKKRTNVIPIIEDARHPMKYRMLVGMVDVIFADVAQPDQ
VOLCA    SHRSGRDLVNMAKQRPNVIPIIEDARHPQKYRMLVPMVDVIFADVAQPDQ
                 210       220       230       240       250

ARATH*   ARILALNASYFLKSGGHFVISIKANCIDSTVPAEAVFQTEVKKLQQEQFK
VITVI    ARILALNASYFLKAGGHFVISIKANCIDSTVPAEAVFAQEVKKLQADMFK
SOYBN    ARILGLNASYYLKTGGHFVISIKANCIDSTVPAEAVFESEVNKLKADQFK
SOLLC    ARILALNASYFLKAGGHFVISIKANCIDSTVPAEAVFAQEVKKLQAEQFK
ORYSJ*   ARILALNASYFLKNGGHFVISIKANCIDSTMPAEAVFASEVEKLKADQFK
HORVU    ARILALNASYFLKNGGHFVISIKANCIDSTQAAEAVFASEVEKLKADQFK
SORBI    ARILALNASYFLKNGGHFVISIKANCIDSTLPAEAVFAAEVEKLKADQFK
SELML    ARIIALNAHSFLKADGKFVMSIKANCIDSTAPPEAVFAQEVAKLQQEKFK
PHYPA    ARIVAINAHNFLKNGGHFVISIKANCIDSTVPAEAVFAAEVKKLQKEQFK
VOLCA    ARIVGHNAQYFLKNGGNFVISIKASCIDSTAPPEAVFAREVKKLQEMQLK
                 260       270       280       290       300
```

MED36

```
ARATH*   P A E Q V T L E P F E R D H A C V V G G Y R M P K K P K A A T A A
VITVI    P S E Q V T L E P F E R D H A C V V G G Y R M P K K Q K P T A - -
SOYBN    P F E Q V T L E P F E R D H A C V V G G Y R M P K K K K D A A E -
SOLLC    P V E Q V T L E P F E R D H A C V V G A Y R V P K K Q K A A A - -
ORYSJ*   P S E Q V T L E P F E R D H A C V V G G Y R M P K K Q K G T S - -
HORVU    P S E Q V T L E P F E R D H A C V V G G Y R M P K K Q K - - - - -
SORBI    P A E Q V T L E P Y E R D H A C V V G G Y R M P K K Q K A T S - -
SELML    P Y E Q V T L E P F E R D H A C V V G A Y R A S K K P K A P S A A
PHYPA    P A E Q V T L E P F E R D H A C V V G T Y R A P K K A K P A A G S
VOLCA    P K E Q V T L E P Y E R D H A M V V G V Y R P P A K K K E - - - -
                           310                 320                 330
```

MED37

```
ARATH*   MIFIKENTAKMTRNKAIACLVFLTVL--DFLMNIGAALMSSLAIEGEEQK
VITVI    M---------ESSWRRRGSLIVAAIVCFGFLAAISIA-------KEEATK
SOYBN    M---------ACSFSRGSLLPLAIIVSLGCLFAISIA-------KEEATK
SOLLC    M--------------------------------------------A-GKG
ORYSJ*   M--------------------------------------------APTKG
HORVU    M------------DRARGSALLLGVLLAGSLFALCAA-------KEEAKK
SORBI    M---------RTTKPTILALVLLSLLCSGYLTGLADGAEARKGKKASGPG
SELML    M---------GAIKKVLPCLFITAWL---FLITIN---------------
PHYPA    M--------------------------------------------A-GKG
VOLCA    M-----------------------------------------------GR
                  10        20        30        40        50

ARATH*   LGTVIGIDLGTTYSCVGVYHNKHVEIIANDQGNRITPSWVAFTDTERLIG
VITVI    LGTVIGIDLGTTYSCVGVYKNGHVEIIANDQGNRITPSWVAFTDSERLIG
SOYBN    LGTVIGIDLGTTYSCVGVYKNGHVEIIANDQGNRITPSWVAFTDSERLIG
SOLLC    EGPAIGIDLGTTYSCVGVWQHDRVEIIANDQGNRTTPSYVAFTDTERLIG
ORYSJ*   EGPAIGIDLGTTYSCVGVWQHDRVEIIANDQGNRTTPSYVGFTDTERLIG
HORVU    LGTVIGIDLGTTYSCVGVYKNGHVEIIANDQGNRITPSWVGFTDGERLIG
SORBI    PGPVIGIDLGTTYSCVGVYRNGHVDIVANDQGNRITPSWVAFTDEERLVG
SELML    -ATSIGIDLGTTYSCVGVYKNGKVEIIANDQGNRITPSWVAFTENERLIG
PHYPA    EGPAIGIDLGTTYSCVGVWQHDRVEIIANDQGNRTTPSYVAFTDTERLIG
VOLCA    EAPAIGIDLGTTYSCVGVWQNDRVEIIANDQGNRTTPSYVAFTDSERLIG
                  60        70        80        90       100

ARATH*   EAAKNQAAKNPERTIFDPKRLIGRKFDDPDVQRDIKFLPYKVVNKDG-KP
VITVI    EAAKNQAAVNAERTIFDVKRLIGRKFEDKEVQKDMKLVPYNIVNKDG-KP
SOYBN    EAAKNLAAVNPERVIFDVKRLIGRKFEDKEVQRDMKLVPYKIVNKDG-KP
SOLLC    DAAKNQVAMNPTNTVFDAKRLIGRRFSDPSVQSDMKLWPFKVIPGPADKP
ORYSJ*   DAAKNQVAMNPINTVFDAKRLIGRRFSDASVQSDIKMWPYKVIPGPGDKP
HORVU    EAAKNQAAVNPERTVFDVKRLIGRKFEDKEVQRDMRLVPYKIVNKEG-KP
SORBI    EAAKNQAPLNPQRTIFDIKRLIGRRFDDEEVQRDVRYLPYKVVNKGG-KP
SELML    EAAKNQATSNPTRTIHDVKRLIGMKYDDKEVQRDMKHLGFKIVNKDG-KP
PHYPA    DAAKNQVAMNPTNTVFDAKRLIGRRFSDASVQSDMKLWPFKITPGPGEKP
VOLCA    DAAKNQVAMNPRNTVFDAKRLIGRKFSDPIVQADIKLWPFGVRAGAGDVP
                 110       120       130       140       150

ARATH*   YIQVKVK-GEEKLFSPEEISAMILTKMKETAEAFLGK--KIKDAVITVPA
VITVI    YIQVKIKDGETKVFSPEEISAMILTKMKETAEAFLGK--KIKDAVVTVPA
SOYBN    YIQVKIKDGETKVFSPEEISAMILTKMKETAEAFLGK--KINDAVVTVPA
SOLLC    MIVVNYK-GEEKQFSAEEISSMVLIKMKEIAEAFLGT--TIKNAVVTVPA
ORYSJ*   MIVVQYK-GEEKQFSAEEISSMVLIKMREIAEAYLGS--TVKNAVVTVPA
HORVU    YIQVKIKDGETKVFSPEEVSAMILGKMKETAEAYLGK--KINDAVVTVPA
SORBI    YVEVPMKGGERKTFSPEEISAMILSKMRETAESYLGH--RVADAVVTVPA
SELML    YIEVEVS-GKTKIYSPEEISAMVLTKMKETAEAYLGE--KVTDAVITVPA
PHYPA    MISVMYK-GEEKTFAAEEVSSMVLIKMKEIAEAYLGS--TVKNAVVTVPA
VOLCA    EIVVTYK-GEEKVFKAEEISSMVLIKMKETAQAYLGADREVKKAVITVPA
                 160       170       180       190       200

ARATH*   YFNDAQRQATKDAGAIAGLNVVRIINEPTGAAIAYGLDKKG---GE-SNI
VITVI    YFNDAQRQATKDAGVIAGLNVARIINEPTAAAIAYGLDKKG---GE-KNI
SOYBN    YFNDAQRQATKDAGVIAGLNVARIINEPTAAAIAYGLDKKG---GE-KNI
SOLLC    YFNDSQRQATKDAGTISGLNVMRIINEPTAAAIAYGLDKKISSTGE-KTV
ORYSJ*   YFNDSQRQATKDAGVIAGLNVMRIINEPTAAAIAYGLDKKATSVGE-KNV
HORVU    YFNDAQRQATKDAGVIAGLNVARIINEPTAAAIAYGLDKRG---GE-KNI
SORBI    YFNDAQRQATKDAGTIAGLNVPRIINEPTAAAIAYGLDKKG---AEMMNV
SELML    YFNDAQRGATSDAGRIAGLNVMRIINEPTAAALAYGLDNERN--GE-KKI
PHYPA    YFNDSQRQATKDAGVIAGLNVLRIINEPTAAAIAYGLDKKATSVGE-KNV
VOLCA    YFNDSQRQATKDAGMIAGLEVLRIINEPTAAAIAYGLDKKDSGLGE-RNV
                 210       220       230       240       250

ARATH*   LVYDLGGGTFDVSILTIDNGVFEVLSTSGDTHLGGEDFDHRVMDYFIKLV
VITVI    LVFDLGGGTFDVSILTIDNGVFEVLATNGDTHLGGEDFDQRIMEYFIKLI
SOYBN    LVFDLGGGTFDVSILTIDNGVFEVLATNGDTHLGGEDFDQRIMEYFIKLI
SOLLC    LIFDLGGGTFDVSLLTIEEGIFEVKATAGDTHLGGEDFDNRMVNHFVQEF
ORYSJ*   LIFDLGGGTFDVSLLTIEEGIFEVKATAGDTHLGGEDFDNRLVNHFVQEF
HORVU    LVFDLGGGTFDVSILTIDNGVFEVLATNGDTHLGGEDFDHRIMDYFIKLI
SORBI    LVYDLGGGTFDVSVLSLDHGVFEVLATSGDTHLGGEDFDQRVMDHFIRLV
SELML    LVFDLGGGTFDVSILAVDAGVFEVLTTNGDTHLGGEDFDQRLIDYFVKLV
PHYPA    LIFDLGGGTFDVSILTIEEGIFEVKATAGDTHLGGEDFDNRMVNHFVQEF
VOLCA    LIFDLGGGTFDVSLLTIEEGIFEVKATAGDTHLGGEDFDERLVNHFANEF
                 260       270       280       290       300
```

MED37

```
ARATH*  KKKYNKDISKDHKALGKLRRECELAKRSLSNQHQVRVEIESLFDGVDFSE
VITVI   KKKHGKDISKDNRALGKLRREAERAKRALSSQHQVRVEIESLYDGLDFSE
SOYBN   NKKHKKDISKDSRALSKLRREAERAKRALSSQHQVRVEIESLFDGVDFSE
SOLLC   KRKHKKDISGNPRALRRLRTACERAKRTLSSTAQTTIEIDSLYEGIDFYT
ORYSJ*  KRKHKKDISGNPRALRRLRTSCERAKRTLSSTAQTTIEIDSLFEGVDFYS
HORVU   KKKHGKDISKDNRALGKLRREAERAKRALSNQHQVRVEIESLFDGTDFSE
SORBI   KRKHGKDISKDGRAMGKLRRECERAKRALSSQHQVRVEIESLFDGVDFSE
SELML   KKKYQRDISQEPRALAKLRREVERSKRALSSQHQVRVEIESLIDGIDLSE
PHYPA   KRKYKKDISSNPRALRRLRTAAERAKRTLSSTAQTTIEIDSLYEGVDFYS
VOLCA   QRKYKKDMKTSPRALRRLRTACERAKRTLSSAAQTTIELDSLFEGIDFAT
                       310       320       330       340       350
```

```
ARATH*  PLTRARFEELNMDLFKKTMEPVKKALKDAGLKKSDIDEIVLVGGSTRIPK
VITVI   PLTRARFEELNNDLFRKTMGPVKKAMEDAGLEKRQIDEIVLVGGSTRIPK
SOYBN   PLTRARFEELNNDLFRKTMGPVKKAMEDAGLQKNQIDEIVLVGGSTRIPK
SOLLC   TITRARFEELNMDLFRKCMEPVEKCLRDAKIDKSGVHDIVLVGGSTRIPK
ORYSJ*  TITRARFEELNMDLFRKCMEPVEKCLRDAKMDKSTVHDVVLVGGSTRIPR
HORVU   PLTRARFEELNNDLFRKTMGPVKKAMDDAGLEKTQIHEIVLVGGSTRIPK
SORBI   QLTRAKFEELNMDLFKKTLGPVKKAIADAKLKKSDIHEIVLVGGSTRIPK
SELML   TLTRARFEELNADLFKKTMAPVKKALEDAKLKKSDIDEIVLVGGSTRIPK
PHYPA   TITRARFEELNMDMFRKCMEPVEKSLRDAKMDKSSIHDVVLVGGSTRIPK
VOLCA   SITRARFEELCMDLFRKCMDPVEKCLRDAKMDKSTVHDVVLVGGSTRIPK
                       360       370       380       390       400
```

```
ARATH*  VQQMLKDFFDGKEPSKGTNPDEAVAYGAAVQGGVLSGEGGEETQNILLLD
VITVI   VQQLLKDYFDGKEPNKGVNPDEAVAFGAAVQGSILSGEGGDETKDILLLD
SOYBN   VQQLLKDYFDGKEPNKGVNPDEAVAYGAAVQGSILSGEGGEETKDILLLD
SOLLC   VQQLLQDFFNGRELCKSINPDEAVAYGAAVQAAILSGEGNEKVQDLLLLD
ORYSJ*  VQQLLQDFFNGKELCKSINPDEAVAYGAAVQAAILSGEGNEKVQDLLLLD
HORVU   VQQLLRDYFDGKEPNKGVNPDEAVAFGAAVQGSILSGEGGDETKDILLLD
SORBI   VQELLTEMFDGKEPTKGINPDEAVAYGAAIQASIISGEGGAETKDILLLD
SELML   IQQMLKDFFDGKEPSKGVNPDEAVAYGAAVQTGILN--KATTLKDVLVVD
PHYPA   VQQLLQDFFNGKELCKSINPDEAVAYGAAVQAAILSGEGNEKVQDLLLLD
VOLCA   VQQLLQDFFNGKELNKSINPDEAVAYGAAVQAAILTGEGGEKVQDLLLLD
                       410       420       430       440       450
```

```
ARATH*  VAPLSLGIETVGGVMTNIIPRNTVIPTKKSQVFTTYQDQQTTVTINVYEG
VITVI   VAPLTLGIETVGGVMTKLIPRNTVIPTKKSQVFTTYQDQQTTVSIQVFEG
SOYBN   VAPLTLGIETVGGVMTKLIPRNTVIPTKKSQVFTTYQDQQSTVSIQVFEG
SOLLC   VTPLSLGLETAGGVMTTLIPRNTTIPTKKEQVFSTYSDNQPGVLIQVYEG
ORYSJ*  VTPLSLGLETAGGVMTVLIPRNTTIPTKKEQVFSTYSDNQPGVLIQVYEG
HORVU   VAPLTLGIETVGGVMTKLIPRNTVIPTKKSQVFTTYQDQQTTVSIQVFEG
SORBI   VTPLTLGIETAGGVMTKLIPRNTRIPVKKSQVFTTYEDHQTTVSIKVFEG
SELML   ATALTLGIETVGGVMTPVIPKDTNVPVKKSQTFTTYQDNQPAVNIRVFEG
PHYPA   VTPLSLGLETAGGVMTVLIARNTTIPTKKEQVFSTYSDNQPGVLIQVYEG
VOLCA   VTPLSLGLETAGGVMTVLISRNTTIPTKKEQVFSTYSDNQPGVLIQVYEG
                       460       470       480       490       500
```

```
ARATH*  ERSMTKDNRELGKFDLTGILPAPRGVPQIEVTFEVDANGILQVKAEDKVA
VITVI   ERSLTKDCRQLGKFDLNGIPPAPRGTPQIEVTFEVDANGILNVKAEDKGT
SOYBN   ERSLTKDCRLLGKFELSGIPPAPRGTPQIEVTFEVDANGILNVKAEDKGT
SOLLC   ERARTRDNNLLGKFELTGIPPAPRGVPQITVCFDIDANGILNVSAEDKTT
ORYSJ*  ERTRTRDNNLLGKFELSGIPPAPRGVPQITVCFDIDANGILNVSAEDKTT
HORVU   ERSMTKDCRLLGKFDLSGIPAAPRGTPQIEVTFEVDANGILNVKAEDKGT
SORBI   ERSLTKDCRELGRFDLTGIPPAPRGVPQIEVTFEVDENGILHVTAADKAG
SELML   ERTMTKNNHELGNFELSGIPPAPRGVPQIEVTFEVDTNHMLQVTAKDIGR
PHYPA   ERARTRDNNLLGKFELSGIPPAPRGVPQITVCFDIDANGILNVSAEDKTT
VOLCA   ERARTKDNNLLGKFELTGIPPAPRGVPQINVIFDIDANGILNVSAEDKTT
                       510       520       530       540       550
```

```
ARATH*  KTSQSITITNDKGRLTEEEIEEMIREAEEFAEEDKIMKEKIDARNKLETY
VITVI   GKSEKITITNDKGRLSQEEIDRMVREAEEFAEEDKKVKEKIDARNSLETY
SOYBN   GKSEKITITNEKGRLSQEEIERMVREAEEFAEEDKKVKERIDARNSLETY
SOLLC   GQKNKITITNDKGRLSKDEIEKMVQEAEKYKAEDEEHKKKVEAKNALENY
ORYSJ*  GQKNKITITNDKGRLSKEEIEKMVQDAEKYKSEDEEHKKKVDAKNSLENY
HORVU   GKSEKITITNEKGRLSQEEIDRMVKEAEEFAEEDKKVKERIDARNQLETY
SORBI   GRSKSITITNDKGRLSQEEIDRMVREAEEFAEEDRRVRERVDARNRLENY
SELML   GKEETITITNDKNRLTEAEIKRMIEEAEENAREDQELREKIDARNKLEAY
PHYPA   GQKNKITITNDKGRLSKDEIEKMVQEAEKYKSEDEDHKKKIEAKNGLENY
VOLCA   GNKNKITITNDKGRLSKEEIERMVQEAEKYKADDEQLKKKVEAKNSLENY
                       560       570       580       590       600
```

MED37

```
ARATH*   VYNMKSTVADKEKLAKKISDEDKEKMEGVLKEALEWLEEN-----VNAEK
VITVI    VYNMKNQINDKDKLADKLESDEKEKIETAVKEALEWLDDN-----QSAEK
SOYBN    VYNMKNQVSDKDKLADKLESDEKEKVETAVKEALEWLDDN-----QSVEK
SOLLC    AYNMRNTIKD-DKIASKLSPDEKKKIEDSVEQAIQWLDGN-----QLAEA
ORYSJ*   AYNMRNTIQD-EKIASKLPADDKKKIEDAVEQAIQWLDNN-----QLAEV
HORVU    VYNMKNTVGDKDKLADKLESEEKEKMEGS------WPNNN----------
SORBI    VYRVRATVRDAGGMARKIGDEDRERMEAALAEALEWLEEQDGAAGRTAEK
SELML    LYNMKHTVSDQDKLRDKIDSDDKEKIESAIKEAHEWLDDNN----ETADK
PHYPA    AYNMRNTIKD-EKIASNLDAADKKKIEEAVEAAIQWLDQN-----QLAES
VOLCA    AYNMRNTIRE-DKVASQLSASDKETMEKALTGAMDWLERN-----QMAEV
                   610       620       630       640       650

ARATH*   EDYDEKLKEVELVCDPVIKSVYEKTEG------ENEDDDGD--DHDEL--
VITVI    EDYDEKLKEVEAVCNPIITAVYQRSGGAPGAGSDGGEDD-D--SHDEL--
SOYBN    EEYEEKLKEVEAVCNPIISAVYQRSGGAPGGGASGEDDDED--SHDEL--
SOLLC    DEFEDKMKELESICNPIIAKMYQGRAGPDMAG--GMDEDGPSAGASSA--
ORYSJ*   EEFEDKMKELEGLCNPIIAKMYQG-AGADMGG--GMDDDAPAAGGS----
HORVU    --------------------------------------------------
SORBI    EEYEEKLREVEEVCGPIIKQVYEKSAGSDAAA-----DEED--DVNEL--
SELML    EAFEEQLRELEDVCNPIISKVYQGSRSTQS---EDDDDDADESDHSEL--
PHYPA    DEFDDKMKELEGICNPIIARMYQGGAGGEGA--------PPSYGGSDSPP
VOLCA    EEFEHQLKELENVCNPIITRLYQGGAGG-MPG--GPAPGAGASSGGS---
                   660       670       680       690       700

ARATH*   -----------
VITVI    -----------
SOYBN    -----------
SOLLC    -GAGPKIEEVD
ORYSJ*   -GAGPKIEEVD
HORVU    -----------
SORBI    -----------
SELML    -----------
PHYPA    SGAGPKIEEVD
VOLCA    -GAGPKIEEVD
                   710
```
