# Supplementary material for: Evolution of disorder in Mediator complex and its functional relevance
Source: Nucleic Acids Res. 2015 Nov 20;44(4):1591–612. doi: 10.1093/nar/gkv1135 (PMC4770211; doi:10.1093/nar/gkv1135)
Supplement: SUPPLEMENTARY DATA [file supp_gkv1135_nar-01763-n-2015-File011.zip › Supplementary_legends.doc]

**Supplementary Figure S1.** IDRs in Mediator complex subunits Med1 to Med10 of metazoans.

Format: pdf

**Supplementary Figure S2.** IDRs in Mediator complex subunits Med11 to Med17 of metazoans.

Format: pdf

**Supplementary Figure S3.** IDRs in Mediator complex subunits Med18 to Med23, Med25, Med26, Med28, Med30, Med31, Cdk8, CycC of metazoans.
Format: pdf

**Supplementary Figure S4.** IDRs in Mediator complex subunits of plants.

Format: pdf

**Supplementary Figure S5.** IDRs in Mediator complex subunits of fungi.

Format: pdf

**Supplementary Figure S6.** Comparison of IDR in Med8 of all the three kingdoms.

Format: pdf

**Supplementary Figure S7.** Number of phosphorylation and acetylation sites in Mediator complex subunits of eight model organisms.

Format: pdf

**Supplementary Figure S8.** Yeast two-hybrid assay of AtMed4 and AtMed9 with randomly selected subunits.

Format: pdf

**Supplementary Figure S9.** MoRFs in Mediator complex subunits of 30 model organisms.

Format: pdf

**Supplementary Figure S10.** Quality of homology models.

Format: pdf

**Supplementary Figure S11.** Structure of CBP KIX domain stabilized by diverse ligands (TADs). Different structures were obtained from PDB database and aligned to see the differences. Please note that the unstable carboxyl end of third helix is stabilized in ligand-bound complex. Partially stabilized carboxyl end of third helix after binding to one TAD at Myb site (green structure) is further stabilized by binding to second TAD at MLL site (grey structure) highlighting the importance of carboxyl end in protein-protein interaction.

Format: pdf

**Supplementary Figure S12.** Homology models of double mutated human Med15 KIX domain.

Format: pdf

**Supplementary Figure S13.** Interacting partners of ‘hubs’ in human and yeast Mediator complex subunits.

Format: pdf

**Supplementary Table ST1.** Scientific names and number of IDRs found in each subunit of the metazoans, plants and fungi used in the current study

Format: xls

**Supplementary Table ST2.** Primers used in Yeast two-hybrid assays

Format: xls

**Supplementary Table ST3.** Disorder propensity of predicted Post Translational Modification sites (PTMs) in model organisms.

Format: xls

**Supplementary Table ST4.** Number of experimentally observed PTM sites in disordered regions of Mediator subunits of yeast and human.

Format: pdf

**Supplementary Table ST5**: Statistics of the homology modelling of AtMed7, AtMed21 and AtMed31.

Format: pdf

**Supplementary Table ST6**: Statistics of the homology modelling of KIX variants with double mutation.

Format: pdf
